# Phylodynamics and Molecular Mutations of the Hemagglutinin Affecting Global Transmission and Host Adaptation of H5Nx Viruses

**DOI:** 10.1155/2023/8855164

**Published:** 2023-04-14

**Authors:** Atanas V. Demirev, Heedo Park, Kyuyoung Lee, Sejik Park, Joon-Yong Bae, Man-Seong Park, Jin Il Kim

**Affiliations:** ^1^Department of Microbiology, Institute for Viral Diseases, College of Medicine, Korea University, Seoul, Republic of Korea; ^2^Biosafety Center, College of Medicine, Korea University, Seoul, Republic of Korea; ^3^Vaccine Innovation Center, College of Medicine, Korea University, Seoul, Republic of Korea

## Abstract

Highly pathogenic avian influenza (HPAI) H5 viruses have circulated globally causing incidental human infection with a substantial pandemic threat. The present study investigated the molecular evolution and phylodynamics of hemagglutinin (HA) in avian and human-isolated H5Nx viruses globally circulating since 2000. We investigated the dynamics of amino acid substitution in the HA sequences of avian and human H5Nx viruses and performed a phylogenetic analysis. Our study found that the H5Nx lineages dominantly expanded since 2000 and diverged into multiple sublineages with unique genetic mutations. P185S mutation in HA became a molecular characteristic of dominant H5Nx viruses throughout clades 2.3.4.1 to 2.3.4.4 (2.3.4.1–4). The key mutations, ΔE130 and I155T, and potential N-linked glycosylation at residues 128, 144, and 159 in the HA gene of human-isolated viruses possibly contributed to both the individual and population levels of the H5 evolution and the host adaptation. Our analysis detected heterogeneity in amino acid sites under positive selection in the HA gene of clades 2.3.4.1–4. Accumulated mutations in the HA protein may potentially affect not only the genetic and antigenic diversity of HPAI H5Nx viruses but also increase the functional compatibility with NA subtypes. Given the global spread and incessantly occurring HA mutations of H5Nx viruses, our results emphasize the importance of early identification of HA mutations as well as the need for a comprehensive assessment of H5Nx variants in terms of pandemic preparedness.

## 1. Introduction

Highly pathogenic avian influenza (HPAI) viruses of the H5 subtype have caused continuous outbreaks in poultry, highlighting the continued risk of transmission in humans [1, 2]. Since the first report of fatal human infection of HPAI H5N1 in 1997 [3], the A/goose/Guangdong/1/96 (Gs/Gd) lineage has diverged into 10 distinct clades (0–9) [4, 5]. Interestingly, the Gs/Gd lineage generated descendant strains which after 2007 showed increasing preference to pair with a variety of neuraminidase (NA) subtypes except for N7 [6–8]. Initially, the HPAI of subtypes H5N2, H5N5, H5N6, and H5N8 were globally observed and classified in phylogenetic clade 2.3.4.4, one of the major descendent lineages from the Gs/Gd lineage [9]. The clade 2.3.4.4 H5Nx viruses were extensively spread, and in particular, the HPAI H5N8 subtype has caused global outbreaks in poultry [10–13].

Although the molecular determinants for H5Nx viruses to cross interspecies barriers are still not completely understood, the impact of mutation in hemagglutinin (HA) as a key determinant of host adaptation was observed in previous studies [14]. Evolutionary dynamics of the HA protein regarding HA receptor binding specificity play a central role in the influenza viruses (AIVs) switch from avian to mammalian hosts [15–18]. Specifically, nonsynonymous mutations in the proximity of the HA receptor binding site (RBS) appear to enhance host adaptation and aerosol transmission of H5N1 viruses in mammals [19–21]. Naturally occurring mutations within the 130- and 220-loops of H5N1 HAs (such as S137A and K222Q) could increase the affinity for human-like* α*2,6-linked sialic acid (SA) receptors [22, 23]. Residues increasing *α*2,6 SA binding of the H5N1 HA and lysine at PB2 residue 627 (K627) presented a way to achieve airborne transmission between mammals [16]. It was also reported that human-isolated H5N1 and H5N6 viruses might recognize both avian-like* α*2,3 and *α*2,6 SA receptors but could not sustain respiratory-droplet transmission in ferrets [24]. In addition, the increasing antigenic diversity in HA and NA can also potentially broaden the host ranges of AIVs because immune pressure in different animal hosts can partially drive the selection and preservation of genetic mutations that arise in both surface glycoproteins [25, 26]. Identifying specific residues within HA and NA by reverse genetics has made it possible to study the effect of certain mutations on the interspecies transmission of H5Nx viruses [16, 17, 24, 27].

Hence, amino acid mutations in HA, especially around the RBS region, of H5Nx variants should be carefully monitored in terms of global public health. This study investigated the molecular and evolutionary dynamics of H5Nx HAs using the complete genomic sequences of the HA gene in recently expanded H5 (sub)clades. The phylodynamics were used to investigate the genealogical relationship of H5Nx HAs, and the selection pressure profile. The genetic diversity in the key antigenic sites and molecular changes in the HA crystal structure were assessed to investigate the evolutionary dynamics of H5Nx HAs on the molecular level and its potential effects on the viral characteristics of H5Nx viruses.

## 2. Materials and Methods

### 2.1. Data Collection and Phylogenetic Analyses

A total of 4,069 complete HA sequences of avian and human H5Nx viruses were collected from the database of the National Center for Biotechnology Information (Bethesda, MD, USA) (*n* = 2,690) and the Global Initiative on Sharing Avian Influenza Data (GISAID) (*n* = 1,379) from 1996 to July 2022. We manually inspected HA sequence data and initially selected 3,890 HA sequences of avian and human H5Nx viruses (H5N1 subtype, *n* = 1,733; H5N2, *n* = 659; H5N3, *n* = 90; H5N4, *n* = 5; H5N5, *n* = 53; H5N6, *n* = 379; H5N8 *n* = 947; and H5N9, *n* = 24) (Supplemental Table 1 and Supplemental Data S1). The random subsampling was performed only from HAs containing complete genomic information based on the subtype clades from 0 to 9 based on H5 subclades nomenclature [28], year of isolation (1996–2022), isolation region, and host using an in-house command-line script. Finally, our study used 739 HA sequences including 655 avian and human H5Nx HAs and 84 references HAs (Supplemental Table 2 and Supplemental Data S2).

Multiple alignment using fast Fourier transform (MAFFT) (v7.419) was used to align our HA sequences [29]. The phylogeny of 739 HA sequences was reconstructed by the time-framed Bayesian inference method implemented on Bayesian evolutionary analysis by sampling trees (BEAST) (v1.10.4) [30]. The general time-reversible + I + G nucleotide substitution [31] and lognormal uncorrelated relaxed clock [32] models were selected for the phylogenetic estimation. The Markov chain Monte Carlo (MCMC) chain length was 200 million with sampling every 200 thousand steps and the MCMC chains were assessed on Tracer (v1.7.1). The maximum clade credibility (MCC) tree was prepared using TreeAnnotator (v1.10.4) and visualized using FigTree (v1.4.4) (https://tree.bio.ed.ac.uk).

### 2.2. Genetic Diversity Analysis

We built three subsets based on the phylogenetic classification in the MCC tree of 739 HA sequences: the clade 2.3.2.1, the clade 2.3.4.4, and the combined clades 2.3.4.1 to 2.3.4.4 (2.3.4.1–4). The Bayesian Skygrid plots of four datasets (the complete Gs/Gd lineage, the clade 2.3.2.1, the clade 2.3.4.4, and the clades 2.3.4.1–4) were estimated to evaluate temporal dynamics of genetic diversity in the HA gene of H5Nx viruses over time. The coalescent-based Bayesian Skygrid model was used to estimate the lineage through time (LTT) plots reconstructed based on the inferred coalescent trees for the four datasets on Tracer [33]. The lineage-specific evolutionary rate and divergence times of nucleotide substitutions of the HA genes in four datasets were also estimated and summarized based on the posterior probability of sampling phylogenies.

### 2.3. Selection Pressure Profiles Analysis

Natural selection pressure at individual codon sites was estimated from the ratio of nonsynonymous (d*N*) vs. synonymous (d*S*) nucleotide substitutions per site (d*N/*d*S*, also known as *ω*) using online Datamonkey site [34]. The estimated *ω* (d*N/*d*S*) lower than 1 indicated an overall negative or purifying selective pressure and the *ω* higher than 1 signified that the amino acid position is under positive selection. The individual sites under either positive or negative selection pressure were detected using single-likelihood ancestor counting (SLAC) [35], fast unconstrained Bayesian approximation (FUBAR) [36], and mixed-effects model evolution (MEME) [37] models with the cutoffs of *p* value <0.1 (SLAC and MEME) and posterior probability >0.9 (FUBAR).

### 2.4. Frequency Analysis of Amino Acid Mutations

We used 3,890 HA sequences for the frequency analysis of amino acid mutations in H5Nx HAs. The amino acid substitution was investigated at the 13 key antigenic residues around the RBS in the HA gene (118, 130, 131, 133(a), 137, 155, 160, 185, 187, 189, 193, 196, and 222; H3 numbering). Amino acid mutations of H5Nx HAs were then analyzed based on the HA sequences of the A/Anhui/2/2005 strain located at the tip of clade 2.3.4.1 as a reference to all other HAs. We manually calculated the proportion of HA sequences with each key amino acid substitution in the residue among the total 3,890 sequences by 6 time periods (before 2004, *n* = 147; between 2005 and 2008, *n* = 533; between 2009 and 2012, *n* = 425; between 2013 and 2016, *n* = 1,082; between 2017 and 2020, *n* = 842; and after 2021, *n* = 860). We additionally evaluated the amino acid substitution at the 13 key antigenic residues including two key amino acid substitutions, I155T and T160A, only in the human-isolated H5Nx sequences (before 2004, *n* = 61; between 2005 and 2008, *n* = 249; between 2009 and 2012, *n* = 148; between 2013 and 2016, *n* = 67; between 2017 and 2020, *n* = 15; and after 2021, *n* = 9) (Supplemental Table 3 and Supplemental Data S3).

### 2.5. Structural Analysis of the HA RBS Region

Our study reconstructed the globular head regions of the HA protein based on H5N1 (A/Vietnam/1194/2004, VN1194, clade 1.1; PDB ID: 4BGX) [18, 38] and H5N6 (A/Sichuan/26221/2014, SC26221, clade 2.3.4.4a; PDB ID: 5HU8) [39] viruses using PyMOL Molecular Graphics System v2.1.0 (Schrödinger, Inc., New York, NY, USA). The conserved amino acids at Y95, W153, H183, and Y195 lining the base of each HA RBS were exposed [40], and molecular interactions within 3.0 Å of specific amino acid residues around the HA RBS were investigated.

## 3. Results

### 3.1. Phylogenetic Relationships of H5Nx HAs

**Figure 1:**
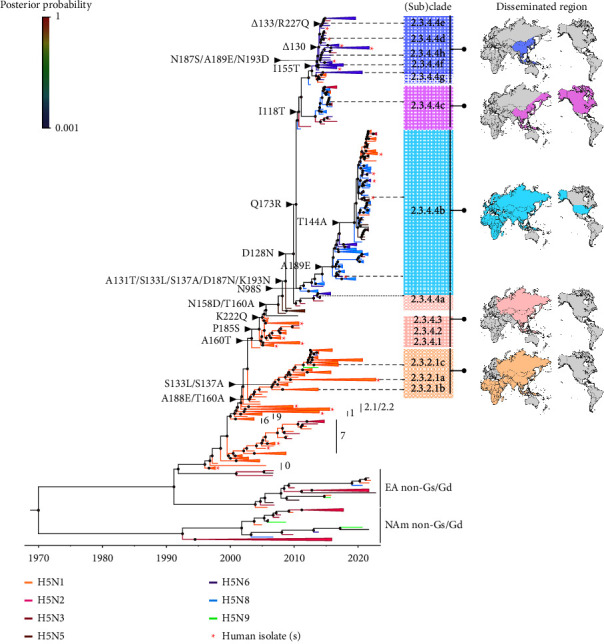
Phylogenetic relationships of the H5Nx HAs between 1996 and 2022. The MCC tree of the H5Nx HAs is reconstructed using the Bayesian evolutionary interference method. The phylogenetic branches with specific subtypes are color-labeled in orange (H5N1), strawberry (H5N2), wine red (H5N3), brown (H5N5), purple (H5N6), light blue (H5N8), and green (H5N9). The genetic clusters containing the same H5 subtype are collapsed to simplify the tree representation. The HAs of human-isolated H5Nx viruses are shown with red asterisks. The geographic regions that have confirmed isolated H5 viruses from clades 2.3.2.1, 2.3.4.1-3, and 2.3.4.4 are presented on the right.

Our MCC phylogeny of the HA gene showed that global H5Nx viruses formed a polyphyletic topology with continuous generation and extinction of sublineages. Our study observed independent diversification of two major clades 2.3.2.1 and 2.3.4.4 following the recent H5 nomenclature [28], and each subclade heterogeneously involved multiple genotypes and human infection strains (Figure 1). The clade 2.3.4.4 diverged into multiple subclades from 2.3.4.4a to 2.3.4.4h. The subclade 2.3.4.4b involved the most recent prevalent strains with multiple H5Nx subtypes, globally spread, and formed the largest monophyletic subclade. We noted that subclades 2.3.4.4d, e, f, and h mostly involved H5N2 and H5N6 viruses that have evolved gradually in a single phylogenetic cluster. The subclade 2.3.4.4g involved the H5N1 and H5N6 viruses, although they appeared to share the same common ancestor with the subclades 2.3.4.4d, e, f, and h (tMRCA–2012.47; 10.47-11.49 HPD) (Table 1). Subclade 2.3.4.4a also involved H5N2 and H5N6 viruses, and subclade 2.3.4.4c held H5N1, H5N2, and H5N8 viruses. The H5N6 in the subclades 2.3.4.4d–h were limitedly identified in Southeast Asia, but the H5N8 viruses were globally detected from East Asia to Russia, the Middle East, Africa, and Europe (2.3.4.4b) as well as in North America (2.3.4.4c). The H5N1 subtype mostly belonged to clades 2.3.2.1 and 2.3.4.1–3, which were detected in East Asia, India, the Middle East, Russia, and Central Africa (Figure 1). Some H5N6 and H5N9 HAs were classified in clade 2.3.2.1 and H5N2 HAs in clade 2.3.4.2, but most H5Nx HAs other than the H5N1 HAs were included in clade 2.3.4.4 (Table 1).

### 3.2. Temporal Dynamics of the Genetic Diversity in HAs

**Figure 2:**
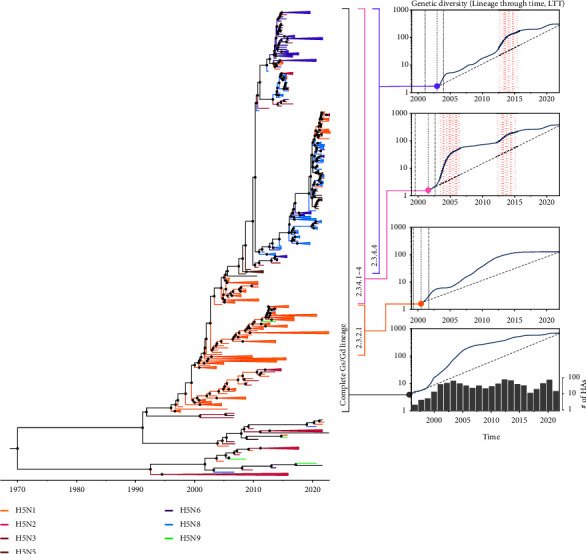
Bayesian coalescent LTT plots of H5Nx HAs by clade over time. The LTT plots display the temporal patterns of lineage diversification through time based on the phylogeny inferred by the Bayesian coalescent approach. The solid lines and blue shading indicate the mean and the 95% confidence interval of the effective population size in the log scale at the designated time interval (years). The dashed line under each curve represents the temporal change of the effective population size with an estimated constant rate of diversification without extinction within the lineages. The vertical, bold-dashed lines represent the divergence time of each clade estimated by the molecular clock model. The pink shade explains the period with a dramatic increase in the effective population size. The bar plot under the curve in the complete Gs/Gd lineage explains the number of the HAs used for the phylogenetic analysis.

Here, we generated lineage through time (LTT) plots and examined the gradual lineage diversification rate following a coalescent-based Bayesian model separately for clades 2.3.2.1, 2.3.4.4, and 2.3.4.1–4, and for the complete Gs/Gd lineage (Figure 2). The LTT plots of all four datasets generally described the expansion of genetic diversity in the HA gene of H5Nx viruses over time. The LTT plots of each clade showed a difference in the period of the increase in clade genetic diversity. The LTT plot for clade 2.3.2.1 showed two subsequent increases in lineage diversity from 2000 to 2003 and from 2006 to 2013. However, the LTT plot for clades 2.3.4.1–4 exhibited two drastic exponential increases from 2000 to 2006 and from 2012 to 2015 marked by the expansion of subclade 2.3.4.4b and the appearance of subclades 2.3.4.4c, 2.3.4.4d–h (Figure 2). The LTT plots for clade 2.3.4.4 specifically showed a single marked increase from 2012 to 2015. The estimated nucleotide substitution rates were similar around 4.66 to 4.80 × 10^−3^ substitution/site/year in all four datasets. The mean nucleotide substitution rate of the HA gene in the Gs/Gd lineage was higher than the substitution rates in clades 2.3.2.1, 2.3.4.1–4, and clade 2.3.4.4 (Table 1).

### 3.3. Key Amino Acid Mutations in H5Nx HAs

Comparing to the HA of A/Anhui/2/2005 as a reference strain, we observed amino acid mutations I118T, D128N, A131T, S133L, S137A, T160A, D187N, A189E, K193N, K222Q, and S227R around the RBS of clade 2.3.4.4 HAs (Table 2). Of these, only T160A, D187N, K193R, K222Q, and S227R were observed in H5N5 HA at the base of clade 2.3.4.4, whereas the mutations S133L, S137A, T160A, and K193R were also identified in clade 2.3.2.1 HAs. The A189E mutation was preserved in the HAs of subclades 2.3.4.4b, 2.3.4.4d, and 2.3.4.4h after 2016 (Table 2, Supplemental Table 3), albeit the 2.3.4.4b evolved independently (Figure 1). Mutations distant from the RBS pocket, such as N98S(T) and Q173R, were found specifically in subclade 2.3.4.4b (and including T199D in 2.3.4.4c). The T160A mutation was persistent in both clades 2.3.2.1 and 2.3.4.4 (Table 2). I155T was detected in subclades 2.3.4.4d–h (except 2.3.4.4g) mostly in H5N6 subtype viruses in East Asia (Figure 1, Table 2).

**Figure 3:**
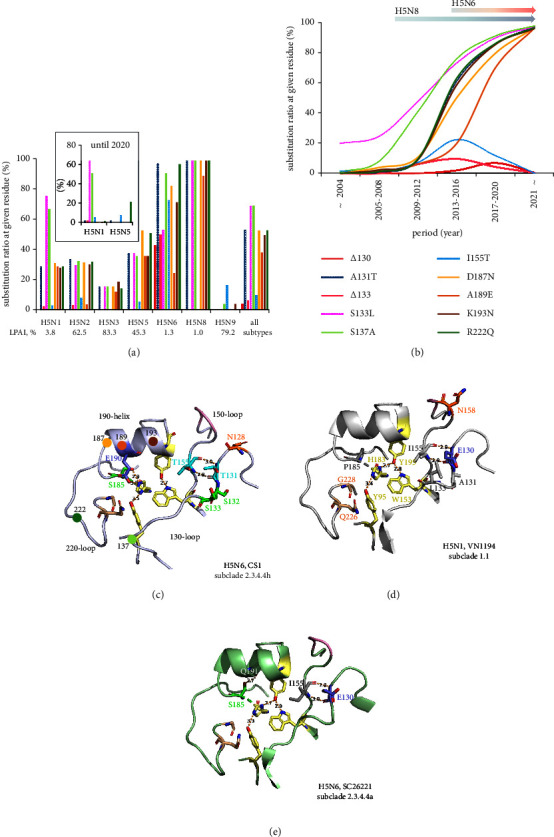
The temporal dynamics and molecular interactions of amino acid substitutions around the HA globular head region of H5Nx viruses. The proportion of amino acid substitutions around the HA globular head region of H5Nx viruses is calculated by (a) the number of H5Nx subtypes or by (b) the 6 time periods: (a) ∼2004 (*n* = 147), (b) 2005–2008 (*n* = 533), (c) 2009–2012 (*n* = 425), (d) 2013–2016 (*n* = 1082), (e) 2017–2020 (*n* = 842), and (f) 2021∼ (*n* = 860). Using the HA structure of VN1194, molecular interactions in the HA globular head region of (c) human-isolated H5N6, A/Changsha/1/2014 (CS1, subclade 2.3.4.4h), (d) human H5N1 HA (VN1194, subclade 1.1), and (e) human H5N6 HA of A/Sichuan/26221/2014 (SC26221, subclade 2.3.4.4a) are analyzed. The amino acid residues of Y95, W153, H183, and Y195 (pale yellow) constitute the conserved floor in the H5 HA RBS. The hydrophobic residues of I155, A131, and L133 are colored gray, the oxygen atoms are red, and the nitrogen atoms are blue. The potential NLG sites at N128 and N158 are colored orange and residues T131 and T155 are colored cyan. S132, S133, and S185 side chains are colored green.

Among the mutations around the RBS of various H5 subtypes, the substitution ratios of six mutations (A131T, S133L, S137A, D187N, K193N, and R222Q) were with higher proportions in the HAs of the H5N6 and H5N8 subtypes (Figure 3(a), Supplemental Figure 1(a)) and appeared to continuously increase over the past decade (Figure 3(b), Supplemental Figure 1(b)). However, most of these mutations were not detected in the HAs of H5N2, H5N3, H5N5, and H5N9 subtypes before 2020 (Figure 3(a)). Specifically, RBS mutations, such as Δ130, Δ133, and I155T mutations, were not found in HAs of the H5N8 (Figure 3(a)) but were detected in 50–80% of the H5N6 and frequently detected in the HAs of human-isolated H5N6 of subclade 2.3.4.4d and 2.3.4.4h (Supplemental Table 3). We noted that the proportion of the A189E mutation increased after 2014 (Figure 3(b)) and was retained in the HAs of two independent subclades 2.3.4.4b and 2.3.4.4h after 2016 (Supplemental Table 3). Importantly, the subtle molecular changes appear to be dominant in the H5N6 and H5N8 HAs (Figure 3(a)). Deletions at residues 130 (Δ130) and 133 (Δ133) were detected mostly in the H5N6 subtype HAs (Figure 3(a), Table 2), where Δ130 was in increased proportions between 2013 and 2020, while Δ133 increased between 2017 and 2019 and then decreased (Figure 3(b)). Mostly observed in the H5N6 HAs, the I155T mutation also showed an increased proportion between 2013 and 2016. We further noted that the proportions of I155T and T160A mutations in human-isolated H5Nx HAs exhibited a similar pattern from 2006 to 2016 (Supplemental Figure 2). However, the number of human H5Nx HAs with the T160A mutation increased after 2017 (Supplemental Figure 2), while I155T proportions were dramatically reduced, as this mutation was not observed in subclade 2.3.4.4b (Supplemental Table 3).

### 3.4. Genetic Polymorphism and Natural Selection Pressure in H5Nx HAs

Our study found that estimates of selection pressures in all four datasets ranged from 0.172 to 0.207, which were lower than 1 (Table 3, Supplemental Table 4). The Gs/Gd lineage showed the highest estimate of HA genes selective pressure (*ω* = 0.207) and the clades 2.3.4.1-4 showed a slightly higher ratio (*ω* = 0.19) than clades 2.3.2.1 (*ω* = 0.18) and 2.3.4.4 (*ω* = 0.17). Among the individual sites in the global Gs/Gd lineage analysis (Table 3), we detected four residues around the RBS under positive selection, 133, 159, 160, and 193 (SLAC) that belong to H5 HA antigenic regions A and B [41, 42]. In addition to these residues, the MEME model predicted episodic diversifying pressure at three additional residues (130, 132, and 142), but residue 160 was not involved in the episodic diversification of the global Gs/Gd lineage. FUBAR, on the other hand, detected two residues 144 and 159 within antigenic regions A and B, respectively. Variations of these two residues are specific in clades 2.3.2.1 and 2.3.4.4 (Supplemental Table 3). The FUBAR model further showed that residues 158, 159, and 193 were consistently selected across clade 2.3.2.1, while residues 133, 193, and 199 have continuously been selected in the clade 2.3.4.4 HAs. Among the amino acid residues under episodic selection (MEME), residues 155 and 222 were specifically detected in clade 2.3.4.4 but not in clade 2.3.2.1 (Table 3).

### 3.5. Molecular Structure and Interaction of Human-Isolated H5Nx HAs

Our study observed structural differences of the HA globular head region in the human A/Changsha/1/2014 (CS1) H5N6 strain in subclade 2.3.4.4h (Figure 3(c)), compared to two other reference HAs in clade 1.1 (human H5N1, VN1194) (Figure 3(d)) [18] and clade 2.3.4.4a (human H5N6, SC26221) (Figure 3(e)) [39]. The simulation of CS1 HA revealed a cluster of polar amino acid residues T131-S(T)132-S133 and T155 were mainly detected in the H5N6 subtype of subclades 2.3.4.4d, 2.3.4.4e, and 2.3.4.4h (Figure 3(c), Supplemental Table 3). The combination of such residues was not observed in the HAs of human H5N6 in subclades 2.3.4.4a (Figure 3(e)) or 2.3.4.4b (Supplemental Table 3), where more hydrophobic side chains were prevalent (A131/S132/L133 and I155) (Figures 3(d) and 3(e)). We found that the hydrogen bonds (H-bonds) between I155 and E130 in the VN1194 and SC26221 HAs shifted to an analogous interaction between amino acid residues T155 and T131 in CS1 HA. Interestingly, putative physical interaction between residues 155 and 131 was also detected for other simulated HAs of human H5Nx viruses within both clades 2.3.2.1 and 2.3.4.4 isolated after 2017 (Supplemental Figure 3). Furthermore, we found that the P185S mutation might alternatively interact with residues Y95, H183, and E190 residues within the RBS pocket of the simulated CS1 HA (Figure 3(c)). Within the RBS of SC26221 HA, however, in the presence of L133 and I155, the S185 amino acid physically interacted with the Q191 sidechain (Figure 3(e)). CS1 HA simulation (Figure 3(a)) also showed that Δ130 in CS1 generates a potential N-linked glycosylation (NLG) site at N128 (Table 2, Figure 3(c)), which is in proximity to the previously well-known NLG site at N158 in the HA of VN1194 (Figure 3(d)).

## 4. Discussion

Our study explored the phylodynamics of influenza H5Nx HAs, detected key determinants on the molecular evolutionary level, and tried to unravel the impact of reassortment with the NA genes with the potential of the emergence of novel zoonotic strains. While fitness difference among AIVs continuously affects the dominance of variant selection through the rapid extinction of variants with low fitness, a variant with beneficial antigenic mutations effectively becomes a dominant strain and generates new sublineages. The HA has been considered a key gene segment that contributes to the fitness of variants due to the impact of its mutation on hosts' partial immunity and transmissibility [43]. Our approach based on the HA gene sequences found that the H5Nx viruses from the Gs/Gd lineage dominantly expanded and diverged into multiple sublineages with unique adaptive genetic mutations in the HAs through their global spread.

Since the HA protein is a constant target for neutralizing antibodies, here, we demonstrated that the HA RBS mutations of H5N6 and H5N8 subtypes would play a key role in forming the antigenicity of clade 2.3.4.4. As previous research reported, clades 2.3.2.1 and 2.3.4.4 were distinctly dominant among H5Nx viruses [10, 12, 13, 44]. Our phylogenetic analysis of the HA gene found that clade 2.3.4.4 would genetically diverge into five major groups; a, b, c, g, and others (d, e, f, and h) [28] (Figure 1, Table 1). However, the genetic classification may not follow the antigenicity change of H5Nx viruses [41]. We observed that the amino acid residues in the HA RBS of subclades 2.3.4.4b (H5N8, and more recently variety of H5N1, H5N2, H5N3, H5N5, and H5N6 subtypes) and 2.3.4.4c (H5N2 and H5N8) did not show the significant antigenic difference (Table 2). Despite the low variations, only H5N8 viruses of subclade 2.3.4.4b spread over 51 countries throughout Asia, Europe, the Middle East, Africa, [11] and recently North America by 2019 [45] (Figure 1). We observed the accumulation of mutations in key antigenic sites around the HA RBS in the H5N8 viruses (Figure 3(a)). Prominent molecular substitutions were A131T, S133L, S137A, D187N, A189E, K193N, and R222Q, and especially most H5Nx viruses in the clade 2.3.4.4 identified after 2020 held these mutations (Figure 3(a), Table 2).

The P185S mutation was first detected in clade 2.3.4.1 and became molecular characteristics of the currently dominant HAs of H5Nx viruses throughout clades 2.3.4.1–4 (Figure 1). Yet with largely unknown function in the presence of L133 and I155, the S185 amino acid residue might physically interact with Q191, thus generating a pull toward the 190-helix (Figure 3(e)). The HA RBS structure in subclade 2.3.4.4h H5N6 (CS1), however, revealed that the amino group of H183 also likely form H-bonds with the -OH groups of S185 and E190, causing a conformational shift and averting its amino acid residues from the usual physical connection between Y95, H183, and Y195 side chains (the base of the RBS dictate the receptor-binding mode [40, 46] (Figures 3(d) and 3(e)). This plasticity and physical interactions among Y95, H183, S185, and E190 residues might generate a pull toward the 220-loop in the HA of the H5N6 subtype (Figure 3(c)), therefore, are readily accessible for the SA receptors. An increased polarity in amino acid residues T131, S132, S133, T155, and S185 and a shorter 130-loop (ΔE130) would also be a reason for a more flexible RBS pocket in the human H5N6 HA (Figure 3(c)). Thus, the P185S mutation is a unique and significant determinant in the evolution of H5Nx HAs forming novel RBS plasticity.

H5Nx viruses with deletions in the 130-loop, such as ΔE130 or ΔL/S133, in combination with I155T, might have a potential for zoonotic infections because these were detected in the HAs of H5N1 viruses instigating sporadic human infections between 2007 and 2014 [27, 47]. High proportions of deletion in 130-loop and I155T are rarely observed in subtypes other than H5N6 in the past decade (Figure 3(b)), and ΔE130 and I155T in the HAs were notable in the avian and human-isolated H5N6 viruses of subclades 2.3.4.4d and 2.3.4.4h (Table 2, Supplemental Table 3). The proportions of strains with these two substitutions increased since 2013 and then declined after 2017, which coincided with the period of subclades 2.3.4.4d–h expansion. Another deletion ΔL133 was also reported to enhance the *α*2,6 SA receptor recognition [21, 47] and perhaps mimics the shorter 130-loop in H2/H3 HAs [48]. Therefore, the dynamics of H5Nx avian influenza viruses in wild migratory birds with these molecular features should be carefully monitored to prevent the risk of the emergence of zoonotic influenza [49].

Introduction of potential NLG at N128 and/or N158 was detected in the HAs of many human-isolated H5N1 and H5N6 viruses (Supplemental Table 3), whereas NLG at N144 was a characteristic of H5N1 HAs in clade 2.3.2.1 (Table 2). NLG is an important molecular modification due to its impact on the modulation of glycoprotein stability and virus pathogenicity [50, 51]. Since amino acid residues 128 and 158 are structurally close to each other (Figures 3(c) and 3(d)), potential NLGs at N128 and N158 might be mutually exclusive. Sun et al. demonstrated that, although NLG at N128 is positioned away from the RBS, it may improve viral fitness by stabilizing the HA trimeric structure [51]. The impact of the lack of NLG at position N158 around the HA globular head on the pathogenicity of H5 AIVs in mammals remains controversial. Yen et al. [52] and Zhao et al. [53] found that NLG at N158 can increase virulence in mice but does not affect receptor binding preferences. However, Zang et al. [54] and Yin et al. [55] reported that a lack of NLG at N158 enhances virulence in mice. Recently, Gao et al. also showed that NLG at N158 increases H5N6 pathogenicity by inducing higher levels of inflammatory factors in mice [50]. These observations suggest that a functional NLG site with a shorter 130-loop might be an important mechanism for adaptation and increased pathogenicity in mammalian hosts.

Natural selection pressure formed the evolutionary characteristics of H5N1 HAs in clade 2.3.2.1 and H5Nx HAs in clade 2.3.4.4. Both clades shared common residues under selection pressure. Specifically, residues 130, 173, and 193 were consistently diversified in both clades. However, each clade showed its unique positive selection profiles at a molecular level. Amino acid residues 158, 159, and 193 within the proximal antigenic site B [41, 42] were under consistent positive selection in the H5N1 HAs of clade 2.3.2.1. In contrast, the clade 2.3.4.4 HAs uniquely showed positive selection in residues 133, 193, and 199, and an additional genetic variation at both individual host and population levels, including residues 155 and 222 (Table 3). Furthermore, the changes in the selective pressure also did not follow key changes in antigenic properties. The A189E mutation was observed dominantly in subclades 2.3.4.4b as well as 2.3.4.4d and 2.3.4.4h since 2017 (Table 2). Advantageous mutations, such as S133L and S137A, were simultaneously preserved in the clades 2.3.2.1 and 2.3.4.4 HAs. These residues were not recognized in the HA selection profile analysis (Table 3).

The lineage diversity plot for clades 2.3.4.1–4 did not follow the logistic-like growth curve (observed for clades 2.3.2.1 and 2.3.4.4), indicating that genetic shift has led to the continuous replacement of H5N1 with more adapted H5Nx subtypes (Figure 2). Notably, the increased diversity between 2000 and 2006 coincides with the transition from the H5N1 (in clades 2.3.4.1–3) to the H5Nx subtype (clade 2.3.4.4). The second stepwise increase after 2012 marks the global spread and dominance of H5Nx HAs in the LTT plot of clade 2.3.4.4 HAs and the expansion and increasing diversity in subclades 2.3.4.4b, 2.3.4.4c, and 2.3.4.4d–h (Figure 2). The stepwise lineage diversity we observed for clades 2.3.4.1–4 often ensues adaptations that enable a breach in the ecological barrier [56] and provides evidence that the expansion of one genetic group is gained at the expense of another [57]. We also noted a small increase in the genetic diversity after 2019 was characteristic for clades 2.3.4.1–4 and 2.3.4.4 alone, marked by the current expansion and diversity in subclade 2.3.4.4b, which might result in the emergence of novel subclades. Altogether, the lineage diversity in clade 2.3.4.1–4 affected the phylodynamics of the H5Nx HAs in two consequent exponential phases ensuing viral adaptations, one between 2004 and 2006 and another between 2012 and 2014.

Our investigation of the lineage-accumulated genetic changes in H5Nx HAs over time showed modulations in the H5 HA lineage diversity with NA subtype preferences. The present study found the H5N6 HAs belonged to subclades 2.3.4.4a, 2.3.4.4b, and 2.3.4.4d–h, and gene reassortment and consequent antigenic drift by each subclade possibly formed the high heterogeneity in the HAs [10, 13, 58]. Particularly, the I118T and I155T mutations were not observed in the H5N6 HAs of subclades 2.3.4.4a and 2.3.4.4b but persisted in the HAs of subclades 2.3.4.4d, 2.3.4.4e, 2.3.4.4f, and 2.3.4.4h. Furthermore, the persistence and stepwise genetic diversity increase of H5N1 HAs in subclade 2.3.2.1c in the past decade is likely related to the genetic reassortment within their genetic group (H5N6 and H5N9 detected) (Figure 1). It is still unclear how the maintained balance between HA and NA in the H5N1 subtype suddenly shifted to other stable combinations with various NA subtypes, resulting in the emergence and global spread of H5Nx [59, 60]. An NLG camouflage in major HA epitope regions and a shorter 130-loop might contribute to the adaptation and evasion of H5Nx viruses from the host immune response [51]. The shorter 130-loop might also shift the sialic acid's sitting position and strengthen the epistatic interactions between the host receptor and HA RBS [48]. As shown previously, a combination of shortened 130-loop and polar amino acid residues T131, S132, and T155 (Figure 3(c)) in the H5N1 or H5N6 HAs could enhance *α*2,6 SA receptor recognition [47]. Interestingly, these were not observed in the recently reported HAs of human-isolated H5N1 and H5N6 viruses in subclade 2.3.4.4b (Supplemental Table 3), which also suggests an improved genetic and functional balance with the NAs of N1 and N6 subtypes.

The human mortality rate with H5Nx viruses is higher than that with other influenza subtypes, including seasonal influenza viruses [61]. The evolving H5Nx viruses are becoming increasingly virulent in mammalian models [1, 62, 63]. Specifically, the HAs of the H5N1 and H5N6 subtypes isolated after 2020 in the subclades 2.3.4.4b and 2.3.4.4h showed an affinity for both *α*2,3 and *α*2,6 SA receptors and high replication capacity in mice [1, 64]. Although human-to-human transmission of the H5Nx viruses was very limitedly reported, these H5Nx viruses would have a potential for host adaptation in humans through the accumulation of genetic mutations in the HA key antigenic sites and its functional interaction with the NA as H5N1 and H5N6 viruses showed. Due to the low herd immunity of humans against such novel H5Nx variants, global public health authorities should carefully monitor molecular changes in novel H5Nx viruses. Though our study solely focused on the genetic characteristics of the H5Nx HAs, our findings would be still meaningful because the molecular characteristics of H5Nx HAs in the present study will provide valuable insight for better surveillance to precisely monitor key genetic mutations and their potential for zoonotic influenza H5Nx emergence through both high transmissibility and host adaptation.

## 5. Conclusions

The present study highlighted unique evolutionary characteristics of the HAs of currently circulating influenza H5Nx viruses. The global spread of the H5Nx viruses promoted the expansion of their genetic diversity, and diverse viral variants continuously gained adaptive advantages by harboring accumulated mutations in HAs and by the genetic shift. Our study suggests key molecular mutations and evolutionary dynamics of the HAs of HPAI H5Nx viruses. We believe this study will facilitate step-changes in our understanding of molecular virology as well as the evolution of H5Nx viruses for the better molecular monitoring of novel influenza variants.

## Figures and Tables

**Table 1:** Evolutionary rates and time of most recent common ancestor (tMRCA) estimates of H5Nx HAs by (sub)clade.

(Sub)clade	Main subtypes	Evolutionary rate (10^−3^substitution/site/year)	tMRCA in year
Gs/Gd lineage	H5Nx	4.86 (4.49–5.19)^a^	1995.83 (1994.84–1996.60)
2.3.2.1	H5N1	4.70 (4.08–5.23)	2003.26 (1999.45–2002.99)
2.3.4.1-4	H5Nx	4.67 (4.27–5.18)	2002.58 (1998.25–2004.32)
2.3.4.4	H5Nx	4.66 (4.19–5.18)	2005.02 (2004.10–2005.98)
2.3.4.4a	H5N2 and H5N6	—	2009.77 (2009.49–2010.65)
2.3.4.4b	H5N1, H5N2, H5N3, H5N5, H5N6, and H5N8	—	2010.46 (2010.09–2011.43)
2.3.4.4c	H5N1, H5N2, and H5N8	—	2013.31 (2012.41–2013.57)
2.3.4.4 d	H5N2 and H5N6	—	2014.15 (2013.53–2014.24)
2.3.4.4e	H5N2 and H5N6	—	2014.18 (2013.58–2014.32)
2.3.4.4f	H5N6	—	2013.78 (2013.19–2013.86)
2.3.4.4g	H5N1 and H5N6	—	2013.10 (2012.71–2013.70)
2.3.4.4h	H5N6	—	2014.07 (2013.47–2014.20)

^a^The lower and upper limits of 95% highest posterior density (HPD) are presented in parenthesis.

**Table 2:** Amino acid mutations in the HA globular head region of H5Nx viruses by (sub)clade.

Representative strains	Subtypes	(Sub)clade	*Amino acid signature at a given residue (H3 numbering)*																
			*HA head*			*130-loop*					*HA head*			*190-helix*				*220-loop*	
			98	118	128	130	131	133	137	144	155	160	173	187	189	193	199	222	227
A/Anhui/2/2005	H5N1	2.3.4.1	N	I	D	E	A	S	S	T	I	T	Q	D	A	K	T	K	S

A/Nepal/19FL1997/2019	H5N1	2.3.2.1a						L	A	N^a^		A				R			
A/duck/Bangladesh/17D1821/2022	H5N1	2.3.2.1a			N			L	A	N^a^		A				R			
A/duck/Laos/NL-2072062/2020	H5N1	2.3.2.1c						L	A	N^a^		A	R			R			

A/goose/Guangdong/k0103/2010	H5N5	2.3.4.4										A		N		R		Q	R

A/duck/Zheijiang/6DK19/2013	H5N2	2.3.4.4a					T	L	A			A		N		N		Q	R
A/duck/Vietnam/NCDV15A57/2015	H5N6	2.3.4.4a					T	L	A			A		N		D		Q	R

A/duck/Shandong/Q1/2013	H5N8	2.3.4.4b	S				T	L	A			A	R	N		N		Q	R
A/common teal/Korea/W547/2016	H5N8	2.3.4.4b	S		N		T	L	A			A	R	N	E	N		Q	R
A/Turkey/Norway/FU496/2020	H5N8	2.3.4.4b	S		N		T	L	A	A		A	R	N	E	N		Q	R
A/Hunan/10117/2021	H5N6	2.3.4.4b	S		N		T	L	A	A		A	R	N	E	N		Q	R
A/England/215201407/2021	H5N1	2.3.4.4b	S		N		T	L	A	A		A	R	N	E	N		Q	R

A/mallard/Korea/W452/2014	H5N8	2.3.4.4c	T	T	N		T	L	A	A		A	R	N		N	D	Q	R
A/chicken/Iowa/04-20/2015	H5N2	2.3.4.4c	T	T	N		T	L	A	A		A	R	N		N	D	Q	

A/chicken/Guangdong/GD1602/2016	H5N6	2.3.4.4d		T	N^a^	Δ^b^	T		A	M	T	A	R	N	E	N		Q	
A/Hubei/29578/2016	H5N6	2.3.4.4d		T	N^a^	Δ^b^	T		A	M	T	A	R	N	E	N		Q	

A/chicken/Hokkaido/002/2017	H5N6	2.3.4.4e		T	N		T	Δ	A	V	T	A	G	N		N		Q	Q
A/wild duck/South Korea/1920/2019	H5N6	2.3.4.4e		T	N		T	Δ		V	T	A	G	N		N		Q	Q

A/duck/Vietnam/LBM638/2014	H5N1	2.3.4.4g		T			T	L	A	M		T	R	N		D		Q	R
A/duck/Vietnam/HU13-65/2019	H5N6	2.3.4.4g		T	N		T	L	A	M		A	S	N		N		Q	R

A/chicken/Vietnam/BD1113/2017	H5N6	2.3.4.4f		T	N		T		A	V	T	A	G	N		N		Q	Q

A/Guangdong/18SF020/2018	H5N6	2.3.4.4h		T	N^a^	Δ^b^	T		A	V	T	A	K	S	E	D		Q	R
A/Chongging/00013/2021	H5N6	2.3.4.4h		T	N^a^	Δ^b^	T		A	V	T	A	K	S	E	D		Q	R

^a^Potential *N*-linked glycosylation site. ^b^Δ, deletion of amino acid.

**Table 3:** Selection pressure profiles in H5Nx HAs by clade.

Clades	The number of sequences	d*N*/d*S*	*Amino acid residue under positive selection*		
			SLAC	FUBAR	MEME
Gs/Gd lineage	697	0.207	40, 133, 159, 160, 173, 192, 193, 199, 481	144, 159	40, 114, 124, 130, 132, 133, 142, 159, 193, 296, 297, 324, 481
2.3.2.1	129	0.182	158, 193	158, 159, 193	130, 132, 140, 145, 158, 159, 173, 193
2.3.4.1-4	358	0.19	119, 133, 173, 192, 199, 326	119, 192, 193, 199, 326, 537	40, 119, 127, 130, 133, 142, 199, 222, 324, 326, 537
2.3.4.4	306	0.172	173, 199	133, 193, 199	40, 130, 133, 142, 155, 173, 199, 222, 273

## Data Availability

All the data in this study are included within the article and supplementary information files. The HA genomic data of H5Nx viruses are obtained from the database of Influenza Virus Resource (https://www.ncbi.nlm.nih.gov/genomes/FLU/Database/nph-select.cgi?go=database) and the EpiFlu database of Global Initiative on Sharing Avian Influenza Data (GISAID; https://gisaid.org).

## Supplementary Materials

Supplementary Materials Supplemental Table 1: the number of the H5Nx HA sequences by subtype collected. Supplemental Table 2: the number of the H5Nx HA sequences by subtype and isolation year used for the phylogenetic analysis. Supplemental Table 3: amino acid mutations in the HA globular head region of reference human-isolated H5Nx viruses by clade. Supplemental Table 4: summary of natural selection pressure profiles of the H5Nx HAs by clade. Supplemental Data 1: initial sequence set of H5Nx HAs. Supplemental Data 2: selected sequence set of H5Nx HAs. Supplemental Data 3: sequence set of human-isolated H5Nx HAs. Supplemental Figure 1: the proportion of amino acid mutations around the HA globular head region of clade 2.3.4.4 H5Nx viruses by subtype and collection year. (a) The proportion of amino acid mutations around the HA globular head region of clade 2.3.4.4 H5Nx viruses is presented by each subtype (Supplemental Table 1). (b) The H5Nx HA sequences are divided into six periods; (a) 1997–2004 (*n* = 147), (b) 2005–2008 (*n* = 533), (c) 2009–2012 (*n* = 425), (d) 2013–2016 (*n* = 1082), (e) 2017–2020 (*n* = 842) and 2021-2022 (*n* = 860). Supplemental Figure 2: the proportion of the I155T and T160A mutations and molecular interactions of the HA globular head region of H5Nx viruses. (a) The proportion of the I155T (blue) and T160A (magenta) mutations in avian (dashed lines) and human-isolated H5Nx viruses (solid lines) is presented by each period (years). (b) Using the HA structure of VN1194, the receptor-binding pocket in H5 HA contains a conserved floor of residues Y95, W153, H183, Y195, and E190 (pale yellow), and Q226 and G228 (orange) that interact with *α*2,3 SA receptors. Residues 155 and 160 are colored blue and magenta. Supplemental Figure 3: structural analysis of the HA globular head region of human-isolated H5Nx viruses. Using the HA structure of VN1194, molecular interactions of the HA globular head region residues are estimated; (a) A/Nepal/19FL1997/2019 (H5N1) (NP19FL1197) in subclade 2.3.2.1; (b) A/England/215201407/2021 (H5N1) (END215201407) in subclade 2.3.4.4b; (c) A/Changsha/1/2014 (H5N6) (CS1) in subclades 2.3.4.4e; and (d) A/Guangdong/18SF020/2018 (H5N6) in subclade 2.3.4.4e.

## References

[1] Cui P., Shi J., Wang C. (2022). Global dissemination of H5N1 influenza viruses bearing the clade 2.3.4.4b HA gene and biologic analysis of the ones detected in China. *Emerging Microbes and Infections*.

[2] King J., Schulze C., Engelhardt A. (2020). Novel HPAIV H5N8 reassortant (clade 2.3.4.4b) detected in Germany. *Viruses*.

[3] de Jong J. C., Claas E. C., Osterhaus A. D., Webster R. G., Lim W. L. (1997). A pandemic warning?. *Nature*.

[4] Boltz D. A., Seiler P., Govorkova E. A., Mondry R. (2010). Emergence of H5N1 avian influenza viruses with reduced sensitivity to neuraminidase inhibitors and novel reassortants in Lao People’s Democratic Republic. *Journal of General Virology*.

[5] Who/Oie/Fao H5N1 Evolution Working Group (2008). Toward a unified nomenclature system for highly pathogenic avian influenza virus (H5N1). *Emerging Infectious Diseases*.

[6] Lee Y. J., Kang H. M., Lee E. K. (2014). Novel reassortant influenza A(H5N8) viruses, South Korea, 2014. *Emerging Infectious Diseases*.

[7] Liu C. G., Liu M., Liu F. (2013). Emerging multiple reassortant H5N5 avian influenza viruses in ducks, China, 2008. *Veterinary Microbiology*.

[8] Zhao K., Gu M., Zhong L. (2013). Characterization of three H5N5 and one H5N8 highly pathogenic avian influenza viruses in China. *Veterinary Microbiology*.

[9] de Vries E., Guo H., Dai M., Rottier P. J., van Kuppeveld F. J., de Haan C. A. (2015). Rapid emergence of highly pathogenic avian influenza subtypes from a subtype H5N1 hemagglutinin variant. *Emerging Infectious Diseases*.

[10] Lee D. H., Bahl J., Torchetti M. K. (2016). Highly pathogenic avian influenza viruses and generation of novel reassortants, United States, 2014-2015. *Emerging Infectious Diseases*.

[11] Lee D. H., Bertran K., Kwon J. H., Swayne D. E. (2017). Evolution, global spread, and pathogenicity of highly pathogenic avian influenza H5Nx clade 2.3.4.4. *Journal of Veterinary Science*.

[12] Sims L., Brown I., Gaidet N. (2017). *Highly pathogenic H5 avian influenza in 2016 and 2017 – observations and future perspectives FOCUS ON*.

[13] Yang L., Zhu W., Li X. (2017). Genesis and dissemination of highly pathogenic H5N6 avian influenza viruses. *Journal of Virology*.

[14] Subbarao K. (2019). The critical interspecies transmission barrier at the Animal(-)Human interface. *Tropical Medicine and Infectious Disease*.

[15] Chen L. M., Blixt O., Stevens J. (2012). In vitro evolution of H5N1 avian influenza virus toward human-type receptor specificity. *Virology*.

[16] Herfst S., Schrauwen E. J., Linster M. (2012). Airborne transmission of influenza A/H5N1 virus between ferrets. *Science*.

[17] Imai M., Watanabe T., Hatta M. (2012). Experimental adaptation of an influenza H5 HA confers respiratory droplet transmission to a reassortant H5 HA/H1N1 virus in ferrets. *Nature*.

[18] Xiong X., Coombs P. J., Martin S. R. (2013). Receptor binding by a ferret-transmissible H5 avian influenza virus. *Nature*.

[19] Gu M., Li Q., Gao R. (2017). The T160A hemagglutinin substitution affects not only receptor binding property but also transmissibility of H5N1 clade 2.3.4 avian influenza virus in Guinea pigs. *Veterinary Research*.

[20] Wang W., Lu B., Zhou H. (2010). Glycosylation at 158N of the hemagglutinin protein and receptor binding specificity synergistically affect the antigenicity and immunogenicity of a live attenuated H5N1 A/Vietnam/1203/2004 vaccine virus in ferrets. *Journal of Virology*.

[21] Yamada S., Suzuki Y., Suzuki T. (2006). Haemagglutinin mutations responsible for the binding of H5N1 influenza A viruses to human-type receptors. *Nature*.

[22] Guo H., de Vries E., McBride R. (2017). Highly pathogenic influenza A(H5Nx) viruses with altered H5 receptor-binding specificity. *Emerging Infectious Diseases*.

[23] Yang Z. Y., Wei C. J., Kong W. P. (2007). Immunization by avian H5 influenza hemagglutinin mutants with altered receptor binding specificity. *Science*.

[24] Herfst S., Mok C. K. P., van den Brand J. M. A. (2018). Human clade 2.3.4.4 A/H5N6 influenza virus lacks mammalian adaptation markers and does not transmit via the airborne route between ferrets. *mSphere*.

[25] Chen R., Holmes E. C. (2006). Avian influenza virus exhibits rapid evolutionary dynamics. *Molecular Biology and Evolution*.

[26] Webster R. G., Bean W. J., Gorman O. T., Chambers T. M., Kawaoka Y. (1992). Evolution and ecology of influenza A viruses. *Microbiological Reviews*.

[27] Xiong X., Xiao H., Martin S. R. (2014). Enhanced human receptor binding by H5 haemagglutinins. *Virology*.

[28] Who (2022). Zoonotic influenza: candidate vaccine viruses and potency testing reagents - development of candidate vaccine viruses for pandemic preparedness. https://www.who.int/teams/global-influenza-programme/vaccines/who-recommendations%20-%20last.

[29] Katoh K., Standley D. M. (2013). MAFFT multiple sequence alignment software version 7: improvements in performance and usability. *Molecular Biology and Evolution*.

[30] Suchard M. A., Lemey P., Baele G., Ayres D. L., Drummond A. J., Rambaut A. (2018). Bayesian phylogenetic and phylodynamic data integration using BEAST 1.10. *Virus Evolution*.

[31] Posada D., Crandall K. A. (2001). Selecting the best-fit model of nucleotide substitution. *Systematic Biology*.

[32] Drummond A. J., Ho S. Y., Phillips M. J., Rambaut A. (2006). Relaxed phylogenetics and dating with confidence. *PLoS Biology*.

[33] Hill V., Baele G. (2019). Bayesian estimation of past population dynamics in BEAST 1.10 using the Skygrid coalescent model. *Molecular Biology and Evolution*.

[34] Delport W., Poon A. F., Frost S. D., Kosakovsky Pond S. L. (2010). Datamonkey 2010: a suite of phylogenetic analysis tools for evolutionary biology. *Bioinformatics*.

[35] Pond S. L., Frost S. D. (2005). Datamonkey: rapid detection of selective pressure on individual sites of codon alignments. *Bioinformatics*.

[36] Murrell B., Moola S., Mabona A. (2013). FUBAR: a fast, unconstrained bayesian approximation for inferring selection. *Molecular Biology and Evolution*.

[37] Murrell B., Wertheim J. O., Moola S., Weighill T., Scheffler K., Kosakovsky Pond S. L. (2012). Detecting individual sites subject to episodic diversifying selection. *PLoS Genetics*.

[38] Bergervoet S. A., Ho C. K. Y., Heutink R., Bossers A., Beerens N. (2019). Spread of highly pathogenic avian influenza (HPAI) H5N5 viruses in Europe in 2016-2017 appears related to the timing of reassortment events. *Viruses*.

[39] Yang H., Carney P. J., Mishin V. P. (2016). Molecular characterizations of surface proteins hemagglutinin and neuraminidase from recent H5Nx avian influenza viruses. *Journal of Virology*.

[40] Ni F., Kondrashkina E., Wang Q. (2015). Structural and functional studies of influenza virus A/H6 hemagglutinin. *PLoS One*.

[41] Kong H., Burke D. F., da Silva Lopes T. J. (2021). Plasticity of the influenza virus H5 HA protein. *mBio*.

[42] Li J., Gu M., Liu K. (2020). Amino acid substitutions in antigenic region B of hemagglutinin play a critical role in the antigenic drift of subclade 2.3.4.4 highly pathogenic H5NX influenza viruses. *Transbound Emerg Dis*.

[43] Russell C. J., Webster R. G. (2005). The genesis of a pandemic influenza virus. *Cell*.

[44] Smith G. J., Donis R. O. (2015). Nomenclature updates resulting from the evolution of avian influenza A(H5) virus clades 2.1.3.2a, 2.2.1, and 2.3.4 during 2013-2014. *Influenza and Other Respiratory Viruses*.

[45] Fao (2019). H5N8 HPAI Global Situation Update. https://www.fao.org/ag/againfo/programmes/en/empres/H5N8/situation_update.html.

[46] Lazniewski M., Dawson W. K., Szczepinska T., Plewczynski D. (2018). The structural variability of the influenza A hemagglutinin receptor-binding site. *Brief Funct Genomics*.

[47] Watanabe Y., Ibrahim M. S., Ellakany H. F. (2011). Acquisition of human-type receptor binding specificity by new H5N1 influenza virus sublineages during their emergence in birds in Egypt. *PLoS Pathogens*.

[48] Ni F., Kondrashkina E., Wang Q. (2018). Determinant of receptor-preference switch in influenza hemagglutinin. *Virology*.

[49] Shin J., Kang S., Byeon H. (2020). Highly pathogenic H5N6 avian influenza virus subtype clade 2.3.4.4 indigenous in South Korea. *Scientific Reports*.

[50] Gao R., Gu M., Shi L. (2021). N-linked glycosylation at site 158 of the HA protein of H5N6 highly pathogenic avian influenza virus is important for viral biological properties and host immune responses. *Veterinary Research*.

[51] Sun H., Deng G., Sun H. (2022). N-linked glycosylation enhances hemagglutinin stability in avian H5N6 influenza virus to promote adaptation in mammals. *PNAS Nexus*.

[52] Yen H. L., Aldridge J. R., Boon A. C. (2009). Changes in H5N1 influenza virus hemagglutinin receptor binding domain affect systemic spread. *Proceedings of the National Academy of Sciences of the United States of America*.

[53] Zhao D., Liang L., Wang S. (2017). Glycosylation of the hemagglutinin protein of H5N1 influenza virus increases its virulence in mice by exacerbating the host immune response. *Journal of Virology*.

[54] Zhang X., Chen S., Jiang Y. (2015). Hemagglutinin glycosylation modulates the pathogenicity and antigenicity of the H5N1 avian influenza virus. *Veterinary Microbiology*.

[55] Yin Y., Yu S., Sun Y. (2020). Glycosylation deletion of hemagglutinin head in the H5 subtype avian influenza virus enhances its virulence in mammals by inducing endoplasmic reticulum stress. *Transbound Emerg Dis*.

[56] Simpson G. G. (1953). *The Major Features of Evolution*.

[57] Reznick D. N., Ricklefs R. E. (2009). Darwin’s bridge between microevolution and macroevolution. *Nature*.

[58] Poen M. J., Venkatesh D., Bestebroer T. M. (2019). Co-circulation of genetically distinct highly pathogenic avian influenza A clade 2.3.4.4 (H5N6) viruses in wild waterfowl and poultry in Europe and East Asia, 2017-18. *Virus Evolution*.

[59] Antigua K. J. C., Baek Y. H., Choi W.-S. (2022). Multiple HA substitutions in highly pathogenic avian influenza H5Nx viruses contributed to the change in the NA subtype preference. *Virulence*.

[60] Li Y.-T., Su Y. C. F., Smith G. J. D. (2021). H5Nx viruses emerged during the suppression of H5N1 virus populations in poultry. *Microbiology Spectrum*.

[61] Wu S., Wei Z., Greene C. M. (2018). Mortality burden from seasonal influenza and 2009 H1N1 pandemic influenza in Beijing, China, 2007-2013. *Influenza and Other Respiratory Viruses*.

[62] Ding L., Li J., Li X., Qu B. (2022). Evolutionary and mutational characterization of the first H5N8 subtype influenza A virus in humans. *Pathogens*.

[63] Duong B. T., Than D. D., Ankhanbaatar U. (2022). Assessing potential pathogenicity of novel highly pathogenic avian influenza (H5N6) viruses isolated from Mongolian wild duck feces using a mouse model. *Emerging Microbes & Infections*.

[64] Zhang C., Cui H., Chen L. (2022). Pathogenicity and transmissibility of goose-origin H5N6 avian influenza virus clade 2.3.4.4h in mammals. *Viruses*.

