# Supplementary material for: Phylodynamics and Molecular Mutations of the Hemagglutinin Affecting Global Transmission and Host Adaptation of H5Nx Viruses
Source: Transbound Emerg Dis. 2023 Apr 14;2023:8855164. doi: 10.1155/2023/8855164 (PMC12017097; doi:10.1155/2023/8855164)
Supplement: Supplementary Materials — Supplemental Table 1: the number of the H5Nx HA sequences by subtype collected. Supplemental Table 2: the number of the H5Nx HA sequences by subtype and isolation year used for the phylogenetic analysis. Supplemental Table 3: amino acid mutations in the HA globular head region of reference human-isolated H5Nx viruses by clade. Supplemental Table 4: summary of natural selection pressure profiles of the H5Nx HAs by clade. Supplemental Data 1: initial sequence set of H5Nx HAs. Supplemental Data 2: selected sequence set of H5Nx HAs. Supplemental Data 3: sequence set of human-isolated H5Nx HAs. Supplemental Figure 1: the proportion of amino acid mutations around the HA globular head region of clade 2.3.4.4 H5Nx viruses by subtype and collection year. (a) The proportion of amino acid mutations around the HA globular head region of clade 2.3.4.4 H5Nx viruses is presented by each subtype (Supplemental Table 1). (b) The H5Nx HA sequences are divided into six periods; (a) 1997–2004 (n = 147), (b) 2005–2008 (n = 533), (c) 2009–2012 (n = 425), (d) 2013–2016 (n = 1082), (e) 2017–2020 (n = 842) and 2021-2022 (n = 860). Supplemental Figure 2: the proportion of the I155T and T160A mutations and molecular interactions of the HA globular head region of H5Nx viruses. (a) The proportion of the I155T (blue) and T160A (magenta) mutations in avian (dashed lines) and human-isolated H5Nx viruses (solid lines) is presented by each period (years). (b) Using the HA structure of VN1194, the receptor-binding pocket in H5 HA contains a conserved floor of residues Y95, W153, H183, Y195, and E190 (pale yellow), and Q226 and G228 (orange) that interact with α2,3 SA receptors. Residues 155 and 160 are colored blue and magenta. Supplemental Figure 3: structural analysis of the HA globular head region of human-isolated H5Nx viruses. Using the HA structure of VN1194, molecular interactions of the HA globular head region residues are estimated; (a) A/Nepal/19FL1997/2019 (H5N1) (NP19FL1197) in subclade 2. [file 8855164.f1.zip › dataS1_revision.docx]

>H5N1_A/American green-winged teal/Washington/195750/2014

cttgttaaaagtgatcagatttgcattggttaccatgcaaacaactcaacaaagcaggttgacacgataatggagaaaaacgtcactgttacacatgcccaagacatactggaaaagacacacaacgggaagctctgcgatcttaatggagtgaagcccctgattctaaaggattgtagcgtagctgggtggctccttggaaatccaatgtgcgacgagtttatcagggtaccggaatggtcttacatcgtggagagggctaacccagccaacgacctctgttacccagggactctcaatgactatgaggaactgaaacacctattgagcagaataaatcattttgagaaaactctgatcatccccaggagttcttggcccaatcacgaaacatcattaggggtgagcgcagcatgtccataccagggagcatcctcatttttcagaaatgtggtatggctcatcaaaaagaacgatgcatacccgacaataaagataagctacaataataccaatcgggaagatcttttgatactgtgggggattcatcattccaacaatgcagcagagcagacaaatctttataaaaacccagacacttatgtttccgttgggacatcaacattaaaccagagattggtgccaaaaatagctactagatcccaagtaaacgggcagagtggaagaatggatttcttctggacaattttaaaaccgaatgatgcaatccactttgagagtaatggaaatttcattgctccagaatatgcatacaaaattgtcaagaaaggggactcaacaattatgaaaagtgaaatggagtatggccactgcaacaccaaatgtcaaactccaataggggctataaactctagcatgccattccacaatatacaccctctcaccatcggggaatgccccaaatacgtgaagtcaaacaaattagtccttgcgactgggctcagaaatagtcctctaagagaaagaagaagaaaaGGGagaggactatttggagctatagcagggtttatagagggaggatggcagggaatggtagacggttggtatgggtatcatcatagcaatgagcaggggagtgggtacgctgcagacaaagaatccacccaaaaggcaatagatggagttaccaataaggtcaactcaatcattgacaaaatgaacactcaatttgaggccgttggaagggaatttaataacttagaaaggagaatagagaatttaaacaagaaaatggaagacggattcctagatgtctggacttataatgctgaacttttagttctcatggaaaatgagagaactctagatttccatgactcaaatgtcaagaacctttacgacaaagtccgactacagcttagggataatgcaaaggagctgggtaatggttgtttcgagttctatcacaaatgtgataacgaatgtatggagagcgtaagaaatgggacgtatgactaccctaagtattcagaagaagcaatattaaaaagagaaaaaataagcggagtgaaattagaatcaataggaacttaccagatactgtcaatttattcaacagtggcgagttccctagcactggcaatcatagtggctggtttatctttatggatgtgctctaatgggtcgttacaatgcagaatttgcatctga

>H5N1_A/American wigeon/Washington/196336/2015

cttgttaaaagtgatcagatttgcattggttaccatgcaaacaactcaacaaagcaggttgacacgataatggagaaaaacgtcactgttacacatgcccaagacatactggaaaagacacacaacgggaagctctgcgatcttaatggagtgaagcccctgattctaaaggattgtagcgtagctgggtggctccttggaaatccaatgtgcgacgagtttatcagggtaccggaatggtcttacatcgtggagagggctaacccagccaacgacctctgttacccagggactctcaatgactatgaggaactgaaacacctattgagcagaataaatcattttgagaaaactctgatcatccccaggagttcttggcccaatcacgaaacatcattaggggtgagcgcagcatgtccataccagggagcatcctcatttttcagaaatgtggtatggctcatcaaaaagaacgatgcatacccgacaataaagataagctacaataataccaatcgggaagatcttttgatactgtgggggattcatcattccaacaatgcagcagagcagacaaatctttataaaaacccagacacttatgtttccgttgggacatcaacattaaaccagagattggtgccaaaaatagctactagatcccaagtaaacgggcagagtggaagaatggatttcttctggacaattttaaaaccgaatgatgcaatccactttgagagtaatggaaatttcattgctccagaatatgcatacaaaattgtcaagaaaggggactcaacaattatgaaaagtgaaatggagtatggccactgcaacaccaaatgtcaaactccaataggggctataaactctagcatgccattccacaatatacaccctctcaccatcggggaatgccccaaatacgtgaagtcaaacaaattagtccttgcgactgggctcagaaatagtcctctaagagaaagaagaagaaaaGGGagaggactatttggagctatagcagggtttatagagggaggatggcagggaatggtagacggttggtatgggtatcatcatagcaatgagcaggggagtgggtacgctgcagacaaagaatccacccaaaaggcaatagatggagttaccaataaggtcaactcaatcattgacaaaatgaacactcaatttgaggccgttggaagggaatttaataacttagaaaggagaatagagaatttaaacaagaaaatggaagacggattcctagatgtctggacttataatgctgaacttttagttctcatggaaaatgagagaactctagatttccatgactcaaatgtcaagaacctttacgacaaagtccgactacagcttagggataatgcaaaggagctgggtaatggttgtttcgagttctatcacaaatgtgataacgaatgtatggagagcgtaagaaatgggacgtatgactaccctaagtattcagaagaagcaatattaaaaagagaagaaataagcggagtgaaattagaatcaataggaacttaccagatactgtcaatttattcaacagtggcgagttccctagcactggcaatcatagtggctggtttatctttatggatgtgctctaatgggtcgttacaatgcagaatttgcatctga

>H5N1_A/American wigeon/Washington/196340/2015

cttgttaaaagtgatcagatttgcattggttaccatgcaaacaactcaacaaagcaggttgacacgataatggagaaaaacgtcactgttacacatgcccaagacatactggaaaagacacacaacgggaagctctgcgatcttaatggagtgaagcccctgattctaaaggattgtagcgtagctgggtggctccttggaaatccaatgtgcgacgagtttatcagggtaccggaatggtcttacatcgtggagagggctaacccagccaacgacctctgttacccagggactctcaatgactatgaggaactgaaacacctattgagcagaataaatcattttgagaaaactctgatcatccccaggagttcttggcccaatcacgaaacatcattaggggtgagcgcagcatgtccataccagggagcatcctcatttttcagaaatgtggtatggctcatcaaaaagaacgatgcatacccgacaataaagataagctacaataataccaatcgggaagatcttttgatactgtgggggattcatcattccaacaatgcagcagagcagacaaatctttataaaaacccagacacttatgtttccgttgggacatcaacattaaaccagagattggtgccaaaaatagctactagatcccaagtaaacgggcagagtggaagaatggatttcttctggacaattttaaaaccgaatgatgcaatccactttgagagtaatggaaatttcattgctccagaatatgcatacaaaattgtcaagaaaggggactcaacaattatgaaaagtgaaatggagtatggccactgcaacaccaaatgtcaaactccaataggggctataaactctagcatgccattccacaatatacaccctctcaccatcggggaatgccccaaatacgtgaagtcaaacaaattagtccttgcgactgggctcagaaatagtcctctaagagaaagaagaagaaaaGGGagaggactatttggagctatagcagggtttatagagggaggatggcagggaatggtagacggttggtatgggtatcatcatagcaatgagcaggggagtgggtacgctgcagacaaagaatccacccaaaaggcaatagatggagttaccaataaggtcaactcaatcattgacaaaatgaacactcaatttgaggccgttggaagggaatttaataacttagaaaggagaatagagaatttaaacaagaaaatggaagacggattcctagatgtctggacttataatgctgaacttttagttctcatggaaaatgagagaactctagatttccatgactcaaatgtcaagaacctttacgacaaagtccgactacagcttagggataatgcaaaggagctgggtaatggttgtttcgagttctatcacaaatgtgataacgaatgtatggagagcgtaagaaatgggacgtatgattaccctaagtattcagaagaagcaatattaaaaagagaagaaataagcggagtgaaattagaatcaataggaacttaccagatactgtcaatttattcaacagtggcgagttccctagcactggcaatcatagtggctggtttatctttatggatgtgctctaatgggtcgttacaatgcagaatttgcatctga

>H5N1_A/American_blue-winged_teal/South_Carolina/AH0195150/2021

cttgttaaaagtgatcagatttgcattggttaccatgcaaacaattcgacagagcaagttgacacgataatggaaaagaacgtcactgttacacatgcccaagacatactggaaaaaacacacaacgggaagctctgtgatctaaatggggtgaagcctctgattttaaaggattgtagtgtagctggatggctcctcggaaacccaatgtgcgacgaattcatcagagtgccggaatggtcctacatagtggagcgggctaacccagctaatgacctctgttacccagggagcctcaatgactatgaagaactgaaacacatgttgagcagaataaatcattttgagaagattctgatcatccccaagagttcctggccaaatcatgaaacatcactaggggtgagcgcagcttgtccataccagggagcgccctcctttttcagaaatgtggtgtggcttatcaaaaagaacgatgcatacccaacaataaagataagctacaataataccaatcgggaagatctcttgatactgtgggggattcatcattccaacaatgcagaagagcagacaaatctctacaaaaacccaaccacctacatttcagttggaacatcaactttaaaccagaggctggcaccaaaaatagctactagatcccaagtaaacgggcaacgtggaagaatggacttcttctggacaatcttaaaaccagatgatgcaatccatttcgagagtaatggaaatttcattgctccagaatatgcatacaaaattgtcaagaaaggggactcaacaattatgaaaagtggagtggaatatggccactgcaacaccaaatgtcaaaccccagtaggtgcgataaattctagtatgccattccacaacatacatcctctcaccattggggaatgccccaaatacgtgaagtcaaacaagttggtccttgcgactgggctcagaaataatcctctaagagaaaagagaagaaaaGGGagaggcctgtttggggcgatagcagggtttatagagggaggatggcagggaatggttgatggttggtatgggtaccatcatagcaatgagcagggaagtgggtacgctgcggacaaagaatccacccaaaaggcaatagatggagttaccaataaggtcaactcaatcattgacaaaatgaacactcaatttgaggcagttggaagggagtttaataacttagaaaggaggatagagaatttgaacaagaaaatggaagacggattcctagatgtctggacctataatgctgaacttctagttctcatggagaacgagaggactctagatttccatgattcaaatgtcaagaacctttacgacaaagtcagattacagcttagggataatgcaaaggagctgggtaacggctgtttcgaattctatcacaaatgtgataatgaatgtatggaaagtgtgagaaatgggacgtatgactaccctcagtattcagaagaagcaagattaaaaagagaagaaataagcggagtgaaattagaatcagtagggacttaccagatactgtcaatttattcaacagcggcaagttccctagcactggcaatcatgatggctggtctatctttatggatgtgctccaatgggtcgttacagtgcagaatttgcatttag

>H5N1_A/Anser cygnoides/China/P126/2015

cttgttaaaagtgatcagatttgcattggttaccatgcaaataactcgacagagcaggttgacacgataatggaaaaaaacgtcactgttacacatgcccaagacatactggaaaagacacacaacgggaggctctgcgatctgaatggagtgaaacctctgattttaaaggattgtagtgtagctggatggctccttggaaacccaatgtgcgacgagttcatcagagtgccggaatggtcttacatagtggagagggctaacccatccaatgacctctgttacccagggaacctcaatgactatgaagaactgaaacacctattgagcagaataaatcattttgagaagactctgatcatccccaagagttcttggcccgatcatgaaacatcattaggggtgagcgcagcatgtccataccagggaatgccctcctttttcagaaatgtggtatggcttatcaagaagaacgatacatacccaacaataaagatgagctacaataataccaatagggaagatcttttgatactgtgggggattcatcattccaacaacgcagcagagcagacaaatctttataaaaacccaaccacctatgtttccgttgggacatcaacattaaaccagagattggtgcccaaaatagctactagatcccaagtaaacgggcaacgtggaagaatggatttcttctggacaattttaaaaccgaatgatgcaatccacttcgagagtaatggaaattttattgctccagagtatgcatacaaaattgtcaagaaaggggactcaacaatcatgaaaagtgaaatggaatatggccactgcaacaccaaatgtcaaactccaataggggctataaactctagtatgccattccacaatatacaccctctcaccattggggaatgccccaaatacgtgaaatcaaacaaattagtccttgcgactgggctcagaaatagtcctctaagagagaggagaagaaaaGGGagagggctatttggagctatagcagggtttatagagggaggatggcaaggaatggtagatggttggtatgggtaccaccatagcaatgaacaggggagtgggtacgctgcagacaaagaatccacccaaaaggcaatagatggagttaccaataaggtcaactcgatcattgacaagatggacactcaatttgaggccgttggaagggaatttaataacttagaacggagaatagagaatttaaataagaaaatggaagacggattcctagatgtctggacttataatgctgaacttctagttctcatggaaaatgagagaacactagatttccatgactcaaatgtcaagaacctttacgacaaagtccgactacaacttagggataatgcaaaggagctgggtaatggttgtttcgagttctatcataaatgtgataatgaatgtatggaaagtgtcagaaatgggacgtatgactaccctcagtattcagaagaagcaagattaaaaagagaagaaataagcggagtgaaattggaatcaataggaacttaccaaatactgtcaatttattcaacagtggcgagttccctagcactggcaatcattgtggctggtctatctttatggatgtgctccaatgggtcgttacaatgcagaatttgcatttga

>H5N1_A/Anser_albifrons/Belgium/15465_0010/2021

cttgttaaaagtgatcagatttgcattggttaccatgcaaacaattcgacagagcaggttgacacgataatggaaaagaacgtcactgttacacatgcccaagacatactggaaaaaacacacaacgggaagctctgtgatttaaatggggtgaagcctctgattttaaaagattgtagtgtagctggatggctcctcggaaacccaatgtgcgacgaattcatcagagtgccggaatggtcctacatagtggagcgggctaatccagccaatgacctctgttacccagggagcctcaatgactatgaagaactgaaacacctgttgagcagaataaatcattttgagaagattctgatcatccccaagagttcctggccaaatcatgaaacatcactaggggtgagcgcagcttgtccataccagggagcgccctcctttttcagaaatgtggtgtggcttatcaaaaagaacgatgcatacccaacaataaagataagctacaataataccaatcgggaagatctcttgatactgtgggggattcatcattccaacaatgcagaagagcagacaaatctctataaaaacccaaccacctacatttcagttggaacatcaactttaaaccagaggttagtaccaaaaatagctactagatcccaagtaaacgggcaacgtggaagaatggacttcttctggacaattttaaaaccagatgatgcaatccatttcgagagtaatggaaatttcattgcaccagaatatgcatataaaattgtcaagaaaggggactcaacaattatgaaaagtggagtggaatatggccactgcaacaccaaatgtcaaaccccagtaggagcgataaattctagtatgccattccacaacatacatcctctcaccattggggaatgccccaaatacgtgaagtcaaacaagttggtccttgcgactgggctcagaaatagtcttctaagagaaaagagaagaaaaGGGagaggcctgtttggggcgatagcagggtttatagagggaggatggcagggaatggttgatggttggtatgggtaccatcatagcaatgagcaggggagtgggtacgctgcagacaaagaatccacccaaaaggcaatagatggagttaccaataaggtcaactcaatcattgacaaaatgaacactcaatttgaggcagttggaagggagtttaataacttagaaaggaggatagagaatttgaacaagaaaatggaagacggattcctagatgtctggacctataatgctgaacttctagttctcatggaaaacgagaggactctagatttccatgattcaaatgtcaagaacctttacgacaaagtcagactacagcttagggataatgcaaaggagctgggtaatggctgtttcgaattctatcacaaatgcgataatgaatgtatggaaagtgtgagaaatgggacgtatgactaccctcagtattcagaagaagcaagattaaaaagagaagaaataagcggagtgaaattagaatcaataggaacttaccagatactgtcaatttattcaacagcggcgagttccctagcactggcaatcatgatagctggtctatctttatggatgtgctccaatgggtcgttacagtgcagaatttgcatttag

>H5N1_A/Avian/Viet Nam/Egg/2014

cttgtcaaaagcgatcatatttgcattggttatcatgcaaataactcgacagagcaggtggacacaataatggaaaagaacgttactgttacacatgcccaagacatactggaaaagacacacaacgggaagctctgcgatctaaatggagtgaagcctctgattttaaaagattgtagtgtagcaggatggctcctcggaaatccattgtgtgacgaattcaccaatgtgccagagtggtcttacatagtagagaaggccaatccagccaatgacctctgttatccagggaatttcaacgattatgaagaattgaaacacctattgagcaggataaaccattttgagaaaatacagatcatccccaaagattcttggtcagatcatgaagcctcattaggggtgagcgcagcatgttcataccagggaaatccctccttcttcagaaatgtggtgtggcttatcaaaaaggacaatgcatacccaacaatagagaaaggctacaataataccaaccgagaagatctcttgatactgtgggggatccaccatcctaatgatgaggcagagcagacaaggctataccaaaacccaactacctatatttccattgggacttcaacactaaaccagagattggtaccaaaaatagccactagatccaaaataaacgggcaaaggggcaggatagatttcttctggacaattttaaaaccgaatgacgcaatccatttcaagagtaatggaaatttcattgttccagaatatgcatacaaaattgtcaagaagggagactccacaatcatgagaagtgaagtggaatatggtgactgcaacaccaggtgtcagactccaataggagcgataaactctagtatgccattccacaacatacaccctctcaccatcggagaatgtcccaaatatgtgaaatcaaacaaattagtccttgcaactgggctcagaaatagtcctcaaagagagagaagaagaaaaGGGagagggctgtttggagctatagcaggtttcatagagggaggatggcagggaatgatagatggttggtatgggtaccaccacagcaatgaacaagggagtggttacgctgcagacaaagaatctactcaaaaggcgatagacggagtcaccaataaggtcaattcgatcattgacaaaatgaacactcagtttgaggctgtaggaagggaatttaataacttagagaggagaatagagaatttaaacaagaagatggaagacggattcctagatgtctggacttataatgctgaacttctggtcctcatggagaatgagagaactctagacttccatgactcaaatgtcaagaacctttacgataaggtccgactacaacttaaggataatgcaaaagagctgggaaacggttgtttcgagttctatcacaaatgtgataatgaatgtatggaaagtgtaagaaacgggacgtatgactacccgcagtattcagaagaagcaagattaaaaagagaagaaataagtggagtaaaactggaatcaataggaatctaccaaatactgtcaatttattcaacagtggcgagttccctagtgctggcaatcatgatggctggtctatctttatggatgtgttccaacgggtcgttacagtgcagaatttgcatttga

>H5N1_A/Avian/Viet Nam/Egg/2017

cttgtcaaaagcgatcatatttgcattggttatcatgcaaataactcgacagagcaggttgacacaataatggaaaagaacgttactgttacacatgcccaagacatactggaaaagacacacaacgggaagctctgcgatctaaatggagtgaagcctctgattttaaaagattgtagtgtagcaggatggctcctcggaaatccattgtgtgacgaattcaccaatgtgccagaatggtcttacatagtagagaaggccaatccagccaatgacctctgttacccagggaatttcaacgattatgaagaattgaaacacctattgagcaggataaaccactttgagaaaatacagatcatccccaaagattcttggtcagatcatgaagcctcattgggggtgagcgcagcatgttcataccagggaaattcctccttcttcagaaatgtggtgtggcttatcaaaaaggacaatgcatacccaacaataaaaaaaggctacaataataccaaccgagaagatctcttgatactgtgggggatccaccatcctaatgatgaggcagagcagacaaggctctaccaaaacccaactacctatatttccattgggacttcaacactaaaccagagattggtaccaaaaatagccactagatccaaaataaacgggcaaagtggcaggatagatttcttctggacaattttaaaaccgaatgacgcaatccacttcgagagtaatggaaatttcattgctccagaatatgcatacaaaattgtcaagaagggagactccacaatcatgagaagtgaagtggaatatggtaactgcaacaccaggtgtcagactccaataggggcgataaactctagtatgccattccacaacatacaccctctcaccatcggagaatgtcccaaatatgtgaaatcaaacaaattagtccttgcaactgggctcagaaatagtcctcaaagagagagaagaagaaaaGGGagaggactgtttggagctatagcaggttttatagagggaggatggcagggaatggtagatggttggtatgggtaccaccacagcaatgaacaggggagtggttatgctgcagacaaagaatctactcaaaaggcgatagacggagtcaccaataaggtcaattcgatcattgacaaaatgaacactcagtttgaggctgtaggaagggaatttaataacttagagaggagaatagaaaatttaaacaagaagatggaagacggattcctagatgtctggacttataatgctgaacttctggttctcatggagaatgagagaactctagacttccatgactcaaatgtcaagaacctttacgatagggtccgactacagcttaaggataatgcaaaagagctgggaaacggttgtttcgagttctatcacaaatgtaataatgaatgtatggaaagtgtgagaaatgggacgtatgactacccgcagtattcagaagaagcaagattaaaaagagaggaaataagtggagtaaaactggaatcaataggaatctaccaaatactgtcaatttattcaacagtggcgagttccctagtgctggcaatcatgatggctggtctatctttatggatgtgttccaacgggtcgttacagtgcagaatttgcatttga

>H5N1_A/Barnacle_Goose/Netherlands/10/2022

cttgttaaaagtgatcagatttgcattggttaccatgcaaacaattcgacagagcaggttgacacgataatggaaaagaacgtcactgttacacatgcccaagacatactggaaaaaacacacaacgggaagctctgtgatttaaatggggtgaagcctctgattttaaaggattgtagtgtggctggatggctcctcggaaacccaatgtgcgacgaattcatcagagtgccggaatggtcctacatagtggagcgggctaatccagctaatgacctctgttacccagggagcctcaatgactatgaagaactgaaacacctgttgagcagaataaaccattttgagaagattcttatcatccccaagagttcctggccaaatcatgaaacatcactaggggtgagcgcagcttgtccataccagggagcgccctcctttttcagaaatgtggtgtggcttatcaaaaagaacgatgcatacccaacaataaagataagctacaataataccaatcgggaagatctcttgatactgtgggggattcatcattccaacaatgcagaagaacagacaaatctctataaaaacccaaccacctacatttcagttggaacatcaactttaaaccagaggttggtaccaaaaatagctactagatcccaagtaaacgggcaacgtggaagaatggacttcttctggacaattttaaaaccagatgatgcaatccatttcgagagtaatggaaatttcattgctccagaatatgcatataaaattgtcaagaaaggggactcaacaattatgaaaagtggagtggaatatggccactgcaacaccaaatgtcaaaccccagtaggagcgataaattctagtatgccattccacaacatacatcctctcaccattggggaatgccccaaatacgtgaagtcaaacaagttggtccttgcgactgggctcagaaatagtcctctaagagaaaagagaagaaaaGGGagaggcctgtttggggcgatagcagggtttatagagggaggatggcagggaatggttgatggttggtatgggtaccatcatagcaatgagcaggggagtgggtacgctgcagacaaagaatccacccaaaaggcaatagatggagttaccaataaggtcaactcaatcattgacaaaatgaacactcaatttgaggcagttggaagggagtttaataacttagaaaggaggatagagaatttgaacaagaaaatggaagacggattcctagatgtctggacctataatgctgaacttctagttctcatggaaaacgagaggactctagatttccatgattcaaatgtcaagaacctttacgacaaagtcagactacagcttagggataatgcaaaggagctgggtaatggctgtttcgaattctatcacaaatgcgataatgaatgtatggaaagtgtgagaaatgggacgtatgactaccctcagtattcagatgaagcaagattaaaaagagaagaaataagcggagtgaaattagaatcaataggaacttaccagatactgtcaatttattcaacagcggcgagttccctagcactggcaatcatgatagctggtctatctttatggatgtgctccaatgggtcgttacagtgcagaatttgcatttag

>H5N1_A/Barnacle_Goose/Netherlands/11/2022

cttgttaaaagtgatcagatttgcattggttaccatgcaaataattcgacagagcaggttgacacgataatggaaaagaacgtcactgttacacatgcccaagacatactggaaaaaacacacaacgggaagctctgtgatttaaatggggtgaagcctctgattttaaaggattgtagtgtagctggatggctcctcggaaacccaatgtgcgacgaattcatcagagtgccggaatggtcctacatagtggagcgggctaatccagctaatgacctctgttacccagggagcctcaatgactatgaagaactgaaacacctgttgagcagaataaatcattttgagaagattcttatcatccccaagagttcctggccaaatcatgaaacatcactaggggtgagcgcagcttgtccataccagggagcgccctcctttttcagaaatgtggtgtggcttatcaaaaagaacgatgcatacccaacaataaagataagctacaataataccaatcgggaagatctcttgatactgtgggggattcatcattccaacaatgcagaagaacagacaaatctctataaaaacccaaccacctacatttcagttggaacatcaactttaaaccagaggttggtaccaaaaatagctactagatcccaagtaaacgggcaacgtggaagaatggacttcttctggacaattttaaaaccagatgatgcaatccatttcgagagtaatggaaatttcattgctccagaatatgcatataaaattgtcaagaaaggggactcaacaattatgaaaagtggagtggaatatggccactgcaacaccaaatgtcaaaccccagtaggagcgataaattctagtatgccattccacaacatacatcctctcaccattggggaatgccccaaatacgtgaagtcaaacaagttggtccttgcgactgggctcagaaatagtcctctaagagaaaagagaagaaaaGGGagaggcctgtttggggcgatagcagggtttatagagggaggatggcagggaatggttgatggttggtatgggtaccatcatagcaatgagcaggggagtgggtacgctgcagacaaagaatccacccaaaaggcaatagatggagttaccaataaggtcaactcaatcattgacaaaatgaacactcaatttgaggcagttggaagggagtttaataacttagaaaggaggatagagaatttgaacaagaaaatggaagacggattcctagatgtctggacctataatgctgaacttctagttctcatggaaaacgagaggactctagatttccatgattcaaatgtcaagaacctttacgacaaagtaagactacagcttagggataatgcaaaggagcttggtaatggctgtttcgaattctatcacaaatgcgataatgaatgtatggaaagtgtgagaaatgggacgtatgactaccctcagtattcagaagaagcaagattaaaaagagaagaaataagcggagtgaaattagaatcaataggaacttaccagatactgtcaatttattcaacagcggcgagttccctagcactggcaatcatgatagctggtctatctttatggatgtgctccaatgggtcgttacagtgcagaatttgcatttag

>H5N1_A/Barnacle_Goose/Netherlands/12/2022

ctagttaaaagtgatcagatttgcattggttaccatgcaaacaattcgacagagcaggttgacacgataatggaaaagaacgtcactgttacacatgcccaagacatactggaaaaaacacacaacgggaagctctgtgatttaaatggggtgaagcctctgattttaaaggattgtagtgtagctggatggctcctcggaaacccaatgtgcgacgaattcatcagagtgccggaatggtcctacatagtggagcgggctaatccagctaatgacctctgttacccagggagcctcaatgactatgaagaactgaaacacctgttgagcagaataaatcattttgagaagattcttatcatccccaagagttcctggccaaatcatgaaacatcactaggggtgagcgcagcttgtccataccagggagcgccctcctttttcagaaatgtggtgtggcttatcaaaaagaacgatgcatacccaacaataaagataagctacaataataccaatcgggaagatctcttgatactgtgggggattcatcattccaacaatgcagaagaacagacaaatctctataaaaacccaaccacctacatttcagttggaacatcaactttaaaccagaggttggtaccaaaaatagctactagatcccaagtaaacgggcaacgtggaagaatggacttcttctggacaattttaaaaccagatgatgcaatccatttcgagagtaatggaaacttcattgctccagaatatgcatataaaattgtcaagaaaggggactcaacaattatgaaaagtggagtggaatatggccactgcaacaccaaatgtcaaaccccagtaggagcgataaattctagtatgccattccacaacatacatcctctcaccattggggaatgccccaaatacgtgaagtcaaacaagttggtccttgcgactgggctcagaaatagtcctctaagagaaaagagaagaaaaGGGagaggcctgtttggggcgatagcagggtttatagagggaggatggcagggaatggttgatggttggtatgggtaccatcatagcaatgagcaggggagtgggtacgctgcagacaaagaatccacccaaaaggcaatagatggagttaccaataaggtcaactcaatcattgacaaaatgaacactcaatttgaggcagttggaagggagtttaataacttagaaaggaggatagagaatttgaacaagaaaatggaagacggattcctagatgtctggacctataatgctgaacttctagttctcatggaaaacgagaggactctagatttccatgattcaaatgtcaagaacctttacgacaaagtcagactacagcttagggataatgcaaaggagctgggtaatggctgtttcgaattctatcacaaatgcgataatgaatgtatggaaagtgtgagaaatgggacgtatgactaccctcagtattcagaagaagcaagattaaaaagagaagaaataagcggagtgaaattagaatcaataggaacttaccagatactgtcaatttattcaacagcggcgagttccctagcactggcaatcatgatagctggtctatctttatggatgtgctccaatgggtcgttacagtgcagaatttgcatttag

>H5N1_A/Barnacle_Goose/Netherlands/13/2022

cttgttaaaagtgatcagatttgcattggttaccatgcaaacaattcgacagagaaggttgacacgataatggaaaagaacgtcactgttacacatgcccaagacatactggaaaaaacacacaacgggaagctctgtgatttaaatggggtgaagcctctgattttaaaggattgtagtgtagctggatggctcctcggaaacccaatgtgcgacgaattcatcagagtgccggaatggtcctacatagtggagcgggataatccagctaatgacctctgttacccagggagcctcaatgactatgaagaactgaaacacctgttgagcagaataaaccattttgagaagattctgatcatccccaagagttcctggccaaatcatgaaacatcacttggagtgagcgcagcttgtccataccagggagcgccctcctttttcagaaatgtggtgtggcttatcaaaaagaacaatgcatacccaacaataaagataagctacaataataccaatcgggaagatctcttgatactgtgggggattcatcattccaacaatgcagaagaacagacaaatctctataaaaacccaaccacctacatttcagttggaacatcaactttaaaccagaggttggtaccaaaaatagctactagatcccaagtaaacgggcaacgtggaagaatggacttcttctggacaattttaaaaccagatgatgcaatccatttcgagagtaatggaaatttcattgctccagaatatgcatataaaattgtcaagaaaggggactcaacaattatgaaaagtggagtggaatatggccactgcaacaccaaatgtcaaaccccagtaggagcgataaattctagtatgccattccacaacatacatcctctcaccattggggaatgccccaaatacgtgaagtcaaacaagttggtccttgcgactgggctcagaaatagtcctctaaaagaaaagagaagaaaaGGGagaggcctgtttggggcgatagcagggtttatagagggaggatggcagggaatggttgatggttggtatgggtaccatcatagcaatgagcaggggagtgggtacgctgcagacaaagaatccacccaaaaggcaatagatggagttaccaataaggtcaactcaatcattgacaaaatgaacactcaatttgaggcagttggaagggagtttaataacttagaaaggaggatagagaatttgaacaagaaaatggaagacggattcctagatgtctggacctataatgctgaacttctagttctcatggaaaacgagaggactctagatttccatgattcaaatgtcaagaacctttacgacaaagtcagactacagcttagggataatgcaaaggagctgggtaatggctgtttcgaattctatcacaaatgcgataatgaatgtatggaaagtgtgagaaatgggacgtatgactaccctcagtattcagaagaagcaagattaaaaagagaagaaataagcggagtgaaattagaatcaataggaacttaccagatactgtcaatttattcaacagcggcgagttccctagcactggcaatcatgatagctggtctatctttatggatgtgctccaatgggtcgttacagtgcagaatttgcatttag

>H5N1_A/Barnacle_Goose/Netherlands/15/2022

cttgttaaaagtgatcagatttgcattggttaccatgcaaataattcgacagagcaggttgacacgataatggaaaagaacgtcactgttacacatgcccaagacatactggaaaaaacacacaacgggaagctctgtgatttaaatggggtgaagcctctgattttaaaggattgtagtgtagctggatggctcctcggaaacccaatgtgcgacgaattcatcagagtgccggaatggtcctacatagtggagcgggctaatccagctaatgacctctgttacccagggagcctcaatgactatgaagaactgaaacacctgttgagcagaataaatcattttgagaagattcttatcatccccaagagttcctggccaaatcatgaaacatcactaggggtgagcgcagcttgtccataccagggagcgccctcctttttcagaaatgtggtgtggcttatcaaaaagaacgatgcatacccaacaataaagataagctacaataataccaatcgggaagatctcttgatactgtgggggattcatcattccaacaatgcagaagaacagacaaatctctataaaaacccaaccacctacatttcagttggaacatcaactttaaaccagaggttggtaccaaaaatagctactagatcccaagtaaacgggcaacgtggaagaatggacttcttctggacaattttaaaaccagatgatgcaatccatttcgagagtaatggaaatttcattgctccagaatatgcatataaaattgtcaagaaaggggactcaacaattatgaaaagtggagtggaatatggccactgcaacaccaaatgtcaaaccccagtaggagcgataaattctagtatgccattccacaacatacatcctctcaccattggggaatgccccaaatacgtgaagtcaaacaagttggtccttgcgactgggctcagaaatagtcctctaagagaaaagagaagaaaaGGGagaggcctgtttggggcgatagcagggtttatagagggaggatggcagggaatggttgatggttggtatgggtaccatcatagcaatgagcaggggagtgggtacgctgcagacaaagaatccacccaaaaggcaatagatggagttaccaataaggtcaactcaatcattgacaaaatgaacactcaatttgaggcagttggaagggagtttaataacttagaaaggaggatagagaatttgaacaagaaaatggaagacggattcctagatgtctggacctataatgctgaacttctagttctcatggaaaacgagaggactctagatttccatgattcaaatgtcaagaacctttacgacaaagtaagactacagcttagggataatgcaaaggagcttggtaatggctgtttcgaattctatcacaaatgcgataatgaatgtatggaaagtgtgagaaatgggacgtatgactaccctcagtattcagaagaagcaagattaaaaagagaagaaataagcggagtgaaattagaatcaataggaacttaccagatactgtcaatttattcaacagcggcgagttccctagcactggcaatcatgatagctggtctatctttatggatgtgctccaatgggtcgttacagtgcagaatttgcatttag

>H5N1_A/Barnacle_Goose/Netherlands/16/2022

ctagttaaaagtgatcagatttgcattggttaccatgcaaacaattcgacagagcaggttgacacgataatggaaaagaacgtcactgttacacatgcccaagacatactggaaaaaacacacaacgggaagctctgtgatttaaatggggtgaagcctctgattttaaaggattgtagtgtagctggatggctcctcggaaacccaatgtgcgacgaattcatcagagtgccggaatggtcctacatagtggagcgggctaatccagctaatgacctctgttacccagggagcctcaatgactatgaagaactgaaacacctgttgagcagaataaatcattttgagaagattcttatcatccccaagagttcctggccaaatcatgaaacatcactaggggtgagcgcagcttgtccataccagggagcgccctcctttttcagaaatgtggtgtggcttatcaaaaagaacgatgcatacccaacaataaagataagctacaataataccaatcgggaagatctcttgatactgtgggggattcatcattccaacaatgcagaagaacagacaaatctctataaaaacccaaccacctacatttcagttggaacatcaactttaaaccagaggttggtaccaaaaatagctactagatcccaagtaaacgggcaacgtggaagaatggacttcttttggacaattttaaaaccagatgatgcaatccatttcgagagtaatggaaatttcattgctccagaatatgcatataaaattgtcaagaaaggggactcaacaattatgaaaagtggagtggaatatggccactgcaacaccaaatgtcaaaccccagtaggagcgataaattctagtatgccattccacaacatacatcctctcaccattggggaatgccccaaatacgtgaagtcaaacaagttggtccttgcgactgggctcagaaatagtcctctaagagaaaagagaagaaaaGGGagaggcctgtttggggcgatagcagggtttatagagggaggatggcagggaatggttgatggttggtatgggtaccatcatagcaatgaacaggggagtgggtacgctgcagacaaagaatccacccaaaaggcaatagatggagttaccaataaggtcaactcaatcattgacaaaatgaacactcaatttgaggcagttggaagggagtttaataacttagaaaggaggatagagaatttgaacaagaaaatggaagacggattcctagatgtctggacctataatgctgaacttctagttctcatggaaaacgagaggactctagatttccatgattcaaatgtcaagaacctttacgacaaagtcagactacagcttagggataatgcaaaggagctgggtaatggctgtttcgaattctatcacaaatgcgataatgaatgtatggaaagtgtgagaaatgggacgtatgactaccctcagtattcagaagaagcaagattaaaaagggaagaaataagcggagtgaaattagaatcaataggaacttaccagatactgtcaatttattcaacagcggcgagttccctagcactggcaatcatgatagctggtctatctttatggatgtgctccaatgggtcgttacagtgcagaatttgcatttag

>H5N1_A/Barnacle_Goose/Netherlands/17/2022

ctagttaaaagtgatcagatttgcattggttaccatgcaaacaattcgacagagcaggttgacacgataatggaaaagaacgtcactgttacacatgcccaagacatactggaaaaaacacacaacgggaagctatgtgatttaaatggggtgaagcctctgattttaaaggattgtagtgtagctggatggctcctcggaaacccaatgtgcgacgaattcatcagagtgccggaatggtcctacatagtggagcgggctaatccagctaatgacctctgttacccagggagcctcaatgactatgaagaactgaaacacctgttgagcagaataaatcattttgagaagattcttatcatccccaagagttcctggccaaatcatgaaacatcactaggggtgagcgcagcttgtccataccagggagcgccctcctttttcagaaatgtggtgtggcttatcaaaaagaacgatgcatacccaacaataaagataagctacaataataccaatcgggaagatctcttgatactgtgggggattcatcattccaacaatgcagaagaacagacaaatctctataaaaacccaaccacctacatttcagttggaacatcaactttaaaccagaggttggtaccaaaaatagctactagatcccaagtaaacgggcaacgtggaagaatggacttcttctggacaattttaaaaccagatgatgcaatccatttcgagagtaatggaaacttcattgctccagaatatgcatataaaattgtcaagaaaggggactcaacaattatgaaaagtggagtggaatatggccactgcaacaccaaatgtcaaaccccagtaggagcgataaattctagtatgccattccacaacatacatcctctcaccattggggaatgccccaaatacgtgaagtcaaacaagttggtccttgcgactgggctcagaaatagtcctctaagagaaaagagaagaaaaGGGagaggcctgtttggggcgatagcagggtttatagagggaggatggcagggaatggttgatggttggtatgggtaccatcatagcaatgagcaggggagtgggtacgctgcagacaaagaatccacccaaaaggcaatagatggagttaccaataaggtcaactcaatcattgacaaaatgaacactcaatttgaggcagttggaagggagtttaataacttagaaaggaggatagagaatttgaacaagaaaatggaagacggattcctagatgtctggacctataatgctgaacttctagttctcatggaaaacgagaggactctagatttccatgattcaaatgtcaagaacctttacgacaaagtcagactacagcttagggataatgcaaaggagctgggtaatggctgtttcgaattctatcacaaatgcgataatgaatgtatggaaagtgtgagaaatgggacgtatgactaccctcagtattcagaagaagcaagattaaaaagagaagaaataagcggagtgaaattagaatcaataggaacttaccagatactgtcaatttattcaacagcggcgagttccctagcactggcaatcatgatagctggtctatctttatggatgtgctccaatgggtcgttacagtgcagaatttgcatttag

>H5N1_A/Barnacle_Goose/Netherlands/18/2022

cttgttaaaagtgatcagatttgcattggttaccatgcaaacaattcgacagagcaggttgacacgataatggaaaagaacgtcactgttacacatgcccaagacatactggaaaaaacacacaacgggaagctctgtgatttaaatggggtgaagcctctgattttaaaggattgtagtgtagctggatggctcctcggaaacccaatgtgcgacgaattcatcagagtgccggaatggtcctacatagtggagcgggctaatccagctaatgacctctgttacccagggagcctcaatgactatgaagaactgaaacacctgttgagcagaataaatcattttgagaagattcttatcatccccaagagttcctggccaaatcatgaaacatcactaggggtgagcgcagcttgtccataccagggagcgccctcctttttcagaaatgtggtgtggcttatcaaaaagaacgatgcatacccaacaataaagataagctacaataataccaatcgggaagatctcttgatactgtgggggattcatcattccaacaatgcagaagaacagacaaatctctataaaaacccaaccacctacatttcagttggaacatcaactttaaaccagaggttggtaccaaaaatagctactagatcccaagtaaacgggcaacgtggaagaatggacttcttctggacaattttaaaaccagatgatgcaatccatttcgagagtaatggaaatttcattgctccagaatatgcatataaaattgtcaagaaaggggactcaacaattatgaaaagtggagtggaatatggccactgcaacaccaaatgtcaaaccccagtaggagcgataaattctagtatgccattccacaacatacatcctctcaccattggggaatgccccaaatacgtgaagtcaaacaagttggtccttgcgactgggctcagaaatagtcctctaagagaaaagagaagaaaaGGGagaggcctgtttggggcgatagcagggtttatagagggaggatggcagggaatggttgatggttggtatgggtaccatcatagcaatgagcaggggagtgggtatgctgcagacaaagaatccacccaaaaggcaatagatggagttaccaataaggtcaactcaatcattgacaaaatgaacactcaatttgaggcagttggaagggagtttaataacttagaaaggaggatagagaatttgaacaagaaaatggaagacggattcctagatgtctggacctataatgctgaacttctagttctcatggaaaacgagaggactctagatttccatgattcaaatgtcaagaacctttacgacaaagtcagactacagcttagggataatgcaaaggagctgggtaatggctgtttcgaattctatcacaaatgcgataatgaatgtatggaaagtgtgagaaatgggacgtatgactaccctcagtattcagaagaagcaagattaaaaagagaagaaataagcggagtgaaattagaatcaataggaacttaccagatactgtcaatttattcaacagcggcgagttccctagcactggcaatcatgatagctggtctatctttatggatgtgctccaatgggtcgttacagtgcagaatttgcatttag

>H5N1_A/Barnacle_Goose/Netherlands/6/2021

cttgttaaaagtgatcagatttgcattggttaccatgcaaacaattcgacagagaaggttgacacgataatggaaaagaacgtcactgttacacatgcccaagacatactggaaaaaacacacaacgggaagctctgtgatttaaatggggtgaagcctctgattttaaaggattgtagtgtagctggatggctcctcggaaacccaatgtgcgacgaattcatcagagtgccggaatggtcctacatagtggagcgggctaatccagctaatgacctctgttacccagggagcctcaatgactatgaagaactgaaacacctgttgagcagaataaaccattttgagaagattctgatcatccccaagagttcctggccaaatcatgaaacatcactaggggtgagcgcagcttgtccataccagggagcgccctcctttttcagaaatgtggtgtggcttatcaaaaagaacgatgcatacccaacaataaagataagctacaataataccaatcgggaagatctcttgatactgtgggggattcatcattccaacaatgcagaagaacagacaaatctctataaaaacccaaccacctacatttcagttggaacatcaactttaaaccagaggttggtaccaaaaatagctactagatcccaagtaaacgggcaacgtggaagaatggacttcttctggacaattttaaaaccagatgatgcaatccatttcgagagtaatggaaatttcattgctccagaatatgcatataaaattgtcaagaaaggggactcaacaattatgaaaagtggagtggaatatggccactgcaacaccaaatgtcaaaccccagtaggagcgataaattctagtatgccattccacaacatacatcctctcaccattggggaatgccccaaatacgtgaagtcaaacaagttggtccttgcgactgggctcagaaatagtcctctaagagaaaagagaagaaaaGGGagaggcctgtttggggcgatagcagggtttatagagggaggatggcagggaatggttgatggttggtatgggtaccatcatagcaatgagcaggggagtgggtacgctgcagacaaagaatccacccaaaaggcaatagatggagttaccaataaggtcaactcaatcattgacaaaatgaacactcaatttgaggcagttggaagggagtttaataacttagaaaggaggatagagaatttgaacaagaaaatggaagacggattcctagatgtctggacctataatgctgaacttctagttctcatggaaaacgagaggactctagatttccatgattcaaatgtcaagaacctttacgacaaagtcagactacagcttagggataatgcaaaggagctgggtaatggctgtttcgaattctatcacaaatgcgataatgaatgtatggaaagtgtgagaaatgggacgtatgactaccctcagtattcagaagaagcaagattaaaaagagaagaaataagcggagtgaaattagaatcaataggaacttaccagatactgtcaatttattcaacagcggcgagttccctagcactggcaatcatgatagctggtctatctttatggatgtgctccaatgggtcgttacagtgcagaatttgcatttag

>H5N1_A/Barnacle_Goose/Netherlands/7/2021

cttgttaaaagtgatcagatttgcattggttaccatgcaaacaattcgacagagaaggttgacacgataatggaaaagaacgtcactgttacacatgcccaagacatactggaaaaaacacacaacgggaagctctgtgatttaaatggggtgaagcctctgattttaaaggattgtagtgtagctggatggctcctcggaaacccaatgtgcgacgaattcatcagagtgccggaatggtcctacatagtggagcgggctaatccagctaatgacctctgttacccagggagcctcaatgactatgaagaactgaaacacctgttgagcagaataaaccattttgagaagattctgatcatccccaagagttcctggccaaatcatgaaacatcactaggggtgagcgcagcttgtccataccagggagcgccctcctttttcagaaatgtggtgtggcttatcaaaaagaacgatgcatacccaacaataaagataagctacaataataccaatcgggaagatctcttgatactgtgggggattcatcattccaacaatgcagaagaacagacaaatctctataaaaacccaaccacctacatttcagttggaacatcaactttaaaccagaggttggtaccaaaaatagctactagatcccaagtaaacgggcaacgtggaagaatggacttcttctggacaattttaaaaccagatgatgcaatccatttcgagagtaatggaaatttcattgctccagaatatgcatataaaattgtcaagaaaggggactcaacaattatgaaaagtggagtggaatatggccactgcaacaccaaatgtcaaaccccagtaggagcgataaattctagtatgccattccacaacatacatcctctcaccattggggaatgccccaaatacgtgaagtcaaacaagttggtccttgcgactgggctcagaaatagtcctctaagagaaaagagaagaaaaGGGagaggcctgtttggggcgatagcagggtttatagagggaggatggcagggaatggttgatggttggtatgggtaccatcatagcaatgagcaggggagtgggtacgctgcagacaaagaatccacccaaaaggcaatagatggagttaccaataaggtcaactcaatcattgacaaaatgaacactcaatttgaggcagttggaagggagtttaataacttagaaaggaggatagagaatttgaacaagaaaatggaagacggattcctagatgtctggacctataatgctgaacttctagttctcatggaaaacgagaggactctagatttccatgattcaaatgtcaagaacctttacgacaaagtcagactacagcttagggataatgcaaaggagctgggtaatggctgtttcgaattctatcacaaatgcgataatgaatgtatggaaagtgtgagaaatgggacgtatgactaccctcagtattcagaagaagcaagattaaaaagagaagaaataagcggagtgaaattagaatcaataggaacttaccagatactgtcaatttattcaacagcggcgagttccctagcactggcaatcatgatagctggtctatctttatggatgtgctccaatgggtcgttacagtgcagaatttgcatttag

>H5N1_A/Barnacle_Goose/Netherlands/8/2022

cttgttaaaagtgatcagatttgcattggttaccatgcaaacaattcgacagagaaggttgacacgataatggaaaagaacgtcactgttacacatgcccaagacatactggaaaaaacacacaacgggaagctctgtgatttaaatggggtgaagcctctgattttaaaggattgtagtgtagctggatggctcctcggaaacccaatgtgcgacgaattcatcagagtgccggaatggtcctacatagtggagcgggataatccagctaatgacctctgttacccagggagcctcaatgactatgaagaactgaaacacctgttgagcagaataaaccattttgagaagattctgatcatccccaagagttcctggccaaaccatgaaacatcactaggggtgagcgcagcttgtccataccagggagcgccctcctttttcagaaatgtggtgtggcttatcaaaaagaacgatgcatacccaacaataaagataagctacaataataccaatcgggaagatctcttgatactgtgggggattcatcattccaacaatgcagaagaacagacaaatctctataaaaacccaaccacctacatttcagttggaacatcaactttaaaccagaggttggtaccaaaaatagctactagatcccaagtaaacgggcaacgtggaagaatggacttcttctggacaattttaaaaccagatgatgcaatccatttcgagagtaatggaaatttcattgctccagaatatgcatataaaattgtcaagaaaggggactcaacaattatgaaaagtggagtggaatatggccactgcaacaccaaatgtcaaaccccagtaggagcgataaattctagtatgccattccacaacatacatcctctcaccattggggaatgccccaaatacgtgaagtcaaacaagttggtccttgcgactgggctcagaaatagtcctctaagagaaaagagaagaaaaGGGcgaggcctgtttggggcgatagcagggtttatagagggaggatggcagggaatggttgatggttggtatgggtaccatcatagcaatgagcaggggagtgggtacgctgcagacaaagaatccacccaaaaggcaatagatggagttaccaataaggtcaactcaatcattgacaaaatgaacactcaatttgaggcagttggaagggagtttaataacttagaaaggaggatagagaatttgaacaagaaaatggaagacggattcctagatgtctggacctataatgctgaacttctagttctcatggaaaacgagaggactctagatttccatgattcaaatgtcaagaacctttacgacaaagtcagactacagcttagggataatgcaaaggagctgggtaatggctgtttcgaattctatcacaaatgcgataatgaatgtatggaaagtgtgagaaatgggacgtatgactaccctcagtattcagaagaagcaagattaaaaagagaagaaataagcggagtgaaattagaatcaataggaacttaccagatactgtcaatttattcaacagcggcgagttccctagcactggcaatcatgatagctggtctatctttatggatgtgctccaatgggtcgttacagtgcagaatttgcatttag

>H5N1_A/Barnacle_Goose/Netherlands/9/2022

cttgttaaaagtgatcagatttgcattggttaccatgcaaacaattcgacagagcaggttgacacgataatggaaaagaacgtcactgttacacatgcccaagacatactggaaaaaacacacaacgggaagctctgtgatttaaatggggtgaagcctctgattttaaaggattgtagtgtagctggatggctcctcggaaacccaatgtgcgacgaattcatcagagtgccggaatggtcctacatagtggagcgggctaatccagctaatgacctctgttacccagggagcctcaatgactatgaagaactgaaacacctgttgagcagaataaatcattttgagaagattcttatcatccccaagagttcctggccaaatcatgaaacatcactaggggtgagcgcagcttgtccataccagggagcgccctcctttttcagaaatgtggtgtggcttatcaaaaagaacgatgcatatccaacaataaagataagctacaataataccaatcgggaagatctcttgatactgtgggggattcatcattccaacaatgcagaagaacagacaaatctctataaaaacccaaccacctacatttcagttggaacatcaactttaaaccagaggttggtaccaaaaatagctactagatcccaagtaaacgggcaacgtggaagaatggacttcttctggacaattttaaaaccagatgatgcaatccatttcgagagtaatggaaatttcattgctccagaatatgcatataaaattgtcaagaaaggggactcaacaattatgaaaagtggagtggaatatggccactgcaacaccaaatgtcaaaccccagtaggagcgataaattctagtatgccattccacaacatacatcctctcaccattggggaatgccccaaatacgtgaagtcaaacaagttggtccttgcgactgggctcagaaatagtcctctaagagaaaagagaagaaaaGGGagaggcctgtttggggcgatagcagggtttatagagggaggatggcagggaatggttgatggttggtatgggtaccatcatagcaatgagcaggggagtgggtacgctgcagacaaagaatccacccaaaaggcaatagatggagttaccaataaggtcaactcaatcattgacaaaatgaacactcaatttgaggcagttggaagggagtttaataacttagaaaggaggatagagaatttgaacaagaaaatggaagacggattcctagatgtctggacctataatgctgaacttctagttctcatggaaaacgagaggactctagatttccatgattcaaatgttaagaacctttacgacaaagtcagactacagcttagggataatgcaaaggagctgggtaatggctgtttcgaattctatcacaaatgcgataatgaatgtatggaaagtgtgagaaatgggacgtatgactaccctcagtattcagaagaagcaagattaaaaagagaagaaataagcggagtgaaattagaatcaataggaacttaccagatactgtcaatttattcaacagcggcgagttccctagcactggcaatcatgatagctggtctatctttatggatgtgctccaatgggtcgttacagtgcagaatttgcatttag

>H5N1_A/Barnacle_goose/Netherlands/1/2022

cttgttaaaagtgatcagatttgcattggttaccatgcaaacaattcgacagagcaggttgacacgataatggaaaagaacgtcactgttacacatgcccaagacatactggaaaaaacacacaacgggaagctctgtgatttaaatggggtgaagcctctgattttaaaggattgtagtgtagctggatggctcctcggaaacccaatgtgcgacgaattcatcagagtgccggaatggtcctacatagtggagcgggctaatccagctaatgacctctgttacccagggagcctcaatgactatgaagaactgaaacacctgttaagcagaataaatcattttgagaagattcttatcatccccaagagttcctggccaaatcatgaaacatcactgggggtgagcgcagcttgtccataccagggagcgccctcctttttcagaaatgtggtgtggcttatcaaaaagaacgatgcatacccaacaataaagataagctacaataataccaatcgggaagatctcttgatactgtgggggattcatcattccaacaatgcagaagaacagacaaatctctataaaaacccaaccacctacatttcagttggaacatcaactttaaaccagaggttggtaccaaaaatagctactagatcccaagtaaacgggcaacgtggaagaatggacttcttctggacaattttaaaaccagatgatgcaatccatttcgagagtaatggaaatttcattgctccagaatatgcatataaaattgtcaagaaaggggactcaacaattatgaaaagtggagtggaatatggccactgcaacaccaaatgtcaaaccccagtaggagcgataaattctagtatgccattccacaacatacatcctctcaccattggggaatgccccaaatacgtgaagtcaaacaagttggtccttgcgactgggctcagaaatagtcctctaagagaaaagagaagaaaaGGGagaggcctgtttggggcgatagcagggtttatagagggaggatggcagggaatggttgatggttggtatgggtaccatcatagcaatgagcaggggagtgggtacgctgcagacaaagaatccacccaaaaggcaatagatggagttaccaataaggtcaactcaatcattgacaaaatgaacactcaatttgaggcagttggaagggagtttaataacttagaaaggaggatagagaatttgaacaagaaaatggaagacggattcctagatgtctggacctataatgctgaacttctagttctcatggaaaacgagaggactctagatttccatgattcaaatgtcaagaacctttacgacaaagtcagactacagcttagggataatgcaaaggagctaggtaatggctgtttcgaattctatcacaaatgcgataatgaatgtatggaaagtgtgagaaatgggacgtatgactaccctcagtattcagaagaagcaagattaaaaagagaagaaataagcggagtgaaattagaatcaataggaacttaccagatactgtcaatttattcaacagcggcgagttccctagcactggcaatcatgatagctggtctatctttatggatgtgctccaatgggtcgttacagtgcagaatttgcatttag

>H5N1_A/Barnacle_goose/Netherlands/2/2022

ctagttaaaagtgatcagatttgcattggttaccatgcaaacaattcgacagagcaggttgacacgataatggaaaagaacgtcactgttacacatgcccaagacatactggaaaaaacacacaacgggaagctctgtgatttaaatggggtgaagcctctgattttaaaggattgtagtgtagctggatggctcctcggaaacccaatgtgcgacgaattcatcagagtgccggaatggtcctacatagtggagcgggctaatccagctaatgacctctgttacccagggagcctcaatgactatgaagaactgaaacacctgttgagcagaataaatcattttgagaagattcttatcatccccaagagttcctggccaaatcatgaaacatcactaggggtgagcgcagcttgtccataccagggagcgccctcctttttcagaaatgtggtgtggcttatcaaaaagaacgatgcatacccaacaataaagataagctacaataataccaatcgggaagatctcttgatactgtgggggattcatcattccaacaatgcagaagaacagacaaatctctataaaaacccaaccacctacatttcagttggaacatcaactttaaaccagaggttggtaccaaaaatagctactagatcccaagtaaacgggcaacgtggaagaatggacttcttctggacaattttaaaaccagatgatgcaatccatttcgagagtaatggaaatttcattgctccagaatatgcatataaaattgtcaagaaaggggactcaacaattatgaaaagtggagtggaatatggccactgcaacaccaaatgtcaaaccccagtaggagcgataaattctagtatgccattccacaacatacatcctctcaccattggggaatgccccaaatacgtgaagtcaaacaagttggtccttgcgactgggctcagaaatagtcctctaagagaaaagagaagaaaaGGGagaggcctgtttggggcgatagcagggtttatagagggaggatggcagggaatggttgatggttggtatgggtaccatcatagcaatgagcaggggagtgggtacgctgcagacaaagaatccacccaaaaggcaatagatggagttaccaataaggtcaactcaatcattgacaaaatgaacactcaatttgaggcagttggaagggagtttaataacttagaaaggaggatagagaatttgaacaagaaaatggaagacggattcctagatgtctggacctataatgctgaacttctagttctcatggaaaacgagaggactctagatttccatgattcaaatgtcaagaacctttacgacaaagtcagactacagcttagggataatgcaaaggagctgggtaatggctgtttcgaattctatcacaaatgcgataatgaatgtatggaaagtgtgagaaatgggacgtatgactaccctcagtattcagaagaagcaagattaaaaagagaagaaataagcggagtgaaattagaatcaataggaacttaccagatactgtcaatttattcaacagcggcgagttccctagcactggcaatcatgatagctggtctatctttatggatgtgctccaatgggtcgttacagtgcagaatttgcatttag

>H5N1_A/Barnacle_goose/Netherlands/3/2022

ctagttaaaagtgatcagatttgcattggttaccatgcaaacaattcgacagagcaggttgacacgataatggaaaagaacgtcactgttacacatgcccaagacatactggaaaaaacacacaacgggaagctctgtgatttaaatggggtgaagcctctgattttaaaggattgtagtgtagctggatggctcctcggaaacccaatgtgcgacgaattcatcagagtgccggaatggtcctacatagtggagcgggctaatccagctaatgacctctgttacccagggagcctcaatgactatgaagaactgaaacacctgttgagcagaataaatcattttgagaagattcttatcatccccaagagttcctggccaaatcatgaaacatcactaggggtgagcgcagcttgtccataccagggagcgccctcctttttcagaaatgtggtgtggcttatcaaaaagaacgatgcatacccaacaataaagataagctacaataataccaatcgggaagatctcttgatactgtgggggattcatcattccaacaatgcagaagaacagacaaatctctataaaaacccaaccacctacatttcagttggaacatcaactttaaaccagaggttggtaccaaaaatagctactagatcccaagtaaacgggcaacgtggaagaatggacttcttctggacaattttaaaaccagatgatgcaatccatttcgagagtaatggaaatttcattgctccagaatatgcatataaaattgtcaagaaaggggactcaacaattatgaaaagtggagtggaatatggccactgcaacaccaaatgtcaaaccccagtaggagcgataaattctagtatgccattccacaacatacatcctctcaccattggggaatgccccaaatacgtgaagtcaaacaagttggtccttgcgactgggctcagaaatagtcctctaagagaaaagagaagaaaaGGGagaggcctgtttggggcgatagcagggtttatagagggaggatggcagggaatggttgatggttggtatgggtaccatcatagcaatgagcaggggagtgggtacgctgcagacaaagaatccacccaaaaggcaatagatggagttaccaataaggtcaactcaatcattgacaaaatgaacactcaatttgaggcagttggaagggagtttaataacttagaaaggaggatagagaatttgaacaagaaaatggaagacggattcctagatgtctggacctataatgctgaacttctagttctcatggaaaacgagaggactctagatttccatgattcaaatgtcaagaacctttacgacaaagtcagactacagcttagggataatgcaaaggagctgggtaatggctgtttcgaattctatcacaaatgcgataatgaatgtatggaaagtgtgagaaatgggacgtatgactaccctcagtattcagaagaagcaagattaaaaagagaagaaataagcggagtgaaattagaatcaataggaacttaccagatactgtcaatttattcaacagcggcgagttccctagcactggcaatcatgatagctggtctatctttatggatgtgctccaatgggtcgttacagtgcagaatttgcatttag

>H5N1_A/Barnacle_goose/Netherlands/4/2022

ctagttaaaagtgatcagatttgcattggttaccatgcaaacaattcgacagagcaggttgacacgataatggaaaagaacgtcactgttacacatgcccaagacatactggaaaaaacacacaacgggaagctctgtgatttaaatggggtgaagcctctgattttaaaggattgtagtgtagctggatggctcctcggaaacccaatgtgcgacgaattcatcagagtgccggaatggtcctacatagtggagcgggctaatccagctaatgacctctgttacccagggagcctcaatgactatgaagaactgaaacacctgttgagcagaataaatcattttgagaagattcttatcatccccaagagttcctggccaaatcatgaaacatcactaggggtgagcgcagcttgtccataccagggagcgccctcctttttcagaaatgtggtgtggcttatcaaaaagaacgatgcatacccaacaataaagataagctacaataataccaatcgggaagatctcttgatactgtgggggattcatcattccaacaatgcagaagaacagacaaatctctataaaaacccaaccacctacatttcagttggaacatcaactttaaaccagaggttggtaccaaaaatagctactagatcccaagtaaacgggcaacgtggaagaatggacttcttctggacaattttaaaaccagatgatgcaatccatttcgagagtaatggaaatttcattgctccagaatatgcatataaaattgtcaagaaaggggactcaacaattatgaaaagtggagtggaatatggccactgcaacaccaaatgtcaaaccccagtaggagcgataaattctagtatgccattccacaacatacatcctctcaccattggggaatgccccaaatacgtgaagtcaaacaagttggtccttgcgactgggctcagaaatagtcctctaagagaaaagagaagaaaaGGGagaggcctgtttggggcgatagcagggtttatagagggaggatggcagggaatggttgatggttggtatgggtaccatcatagcaatgagcaggggagtgggtacgctgcagacaaagaatccacccaaaaggcaatagatggagttaccaataaggtcaactcaatcattgacaaaatgaacactcaatttgaggcagttggaagggagtttaataacttagaaaggaggatagagaatttgaacaagaaaatggaagacggattcctagatgtctggacctataatgctgaacttctagttctcatggaaaacgagaggactctagatttccatgattcaaatgtcaagaacctttacgacaaagtcagactacagcttagggataatgcaaaggagctgggtaatggctgtttcgaattctatcacaaatgcgataatgaatgtatggaaagtgtgagaaatgggacgtatgactaccctcagtattcagaagaagcaagattaaaaagagaagaaataagcggagtgaaattagaatcaataggaacttaccagatactgtcaatttattcaacagcggcgagttccctagcactggcaatcatgatagctggtctatctttatggatgtgctccaatgggtcgttacagtgcagaatttgcatttag

>H5N1_A/Barnacle_goose/Netherlands/5/2022

ctagttaaaagtgatcagatttgcattggttaccatgcaaacaattcgacagagcaggttgacacgataatggaaaagaacgtcactgttacacatgcccaagacatactggaaaaaacacacaacgggaagctctgtgatttaaatggggtaaagcctctgattttaaaggattgtagtgtagctggatggctcctcggaaacccaatgtgcgacgaattcatcagagtgccggaatggtcctacatagtggagcgggctaatccagctaatgacctctgttacccagggagcctcaatgactatgaagaactgaaacacctgttgagcagaataaatcattttgagaagattcttatcatccccaagagttcctggccaaatcatgaaacatcactaggggtgagcgcagcttgtccataccagggagcgccctcctttttcagaaatgtggtgtggcttatcaaaaagaacgatgcatacccaacaataaagataagctacaataataccaatcgggaagatctcttgatactgtgggggattcatcattccaacaatgcagaagaacagacaaatctctataaaaacccaaccacctacatttcagttggaacatcaactttaaaccagaggttggtaccaaaaatagctactagatcccaagtaaacgggcaacgtggaagaatggacttcttctggacaattttaaaaccagatgatgcaatccatttcgagagtaatggaaatttcattgctccagaatatgcatataaaattgtcaagaaaggggactcaacaattatgaaaagtggagtggaatatggccactgcaacaccaaatgtcaaaccccagtaggagcgataaattctagtatgccattccacaacatacatcctctcaccattggggaatgccccaaatacgtgaagtcaaacaagttggtccttgcgactgggctcagaaatagtcctctaagagaaaagagaagaaaaGGGagaggcctgtttggggcgatagcagggtttatagagggaggatggcagggaatggttgatggttggtatgggtaccatcatagcaatgagcaggggagtgggtacgctgcagacaaagaatccacccaaaaggcaatagatggagttaccaataaggtcaactcaatcattgacaaaatgaacactcaatttgaggcagttggaagggagtttaataacttagaaaggaggatagagaatttgaacaagaaaatggaagacggattcctagatgtctggacctataatgctgaacttctagttctcatggaaaacgagaggactctagatttccatgattcaaatgtcaagaacctttacgacaaagtcagactacagcttagggataatgcaaaggagctgggtaatggctgtttcgaattctatcacaaatgcgataatgaatgtatggaaagtgtgagaaatgggacgtatgactaccctcagtattcagaagaagcaagattaaaaagagaagaaataagcggagtgaaattagaatcaataggaacttaccagatactgtcaatttattcaacagcggcgagttccctagcactggcaatcatgatagctggtctatctttatggatgtgctccaatgggtcgttacagtgcagaatttgcatttag

>H5N1_A/Barnacle_goose/Netherlands/6/2022

cttgttaaaagtgatcagatttgcattggttaccatgcaaacaattcgacagagcaggttgacacgataatggaaaagaacgtcactgttacacatgcccaagacatactggaaaaaacacacaacgggaagctctgtgatttaaatggggtgaagcctctgattttaaaagattgtagtgtagctggatggctcctcggaaacccaatgtgcgacgaattcatcagagtgccggaatggtcctacatagtggagcgggctaatccagccaatgacctctgttacccagggagcctcaatgactatgaagaactgaaacacctgttgagcagaataaatcattttgagaagattctgatcatccccaagagttcctggccaaatcatgaaacatcactaggggtgagcgcagcttgtccataccagggagcgccctcctttttcagaaatgtggtgtggcttatcaaaaagaacgatgcatacccaacaataaagataagttacaataataccaatcgggaagatctcttgatactgtgggggattcatcattccaacaatgcagaagagcagacaaatctctataaaaacccaaccacctacatttcagttggaacatcaactttaaaccagaggttagtaccaaaaatagctactagatcccaagtaaacgggcaacgtggaagaatggacttcttctggacaattttaaaaccagatgatgcaatccatttcgagagtaatggaaatttcattgcaccagaatatgcatataaaattgtcaagaaaggggactcaacaattatgaaaagtggagtggaatatggccactgcaacaccaaatgtcaaaccccagtaggagcgataaattctagtatgccattccacaacatacatcctctcaccattggggaatgccccaaatacgtgaagtcaaacaagttggtccttgcgactgggctcagaaatagtcctctaagagaaaagagaagaaaaGGGagaggcctgtttggggcgatagcagggtttatagagggaggatggcagggaatggttgatggttggtatgggtaccatcatagcaatgagcaggggagtgggtacgctgcagacaaagaatccacccaaaaggcaatagatggagttaccaataaggtcaactcaatcattgacaaaatgaacactcaatttgaggcagttggaagggagtttaataacttagaaaggaggatagagaatttgaacaagaaaatggaagacggattcctagatgtctggacctataatgctgaacttctagttctcatggaaaacgagaggactctagatttccatgattcaaatgtcaagaacctttacgacaaagtcagactacagcttagggataatgcaaaggagctgggtaatggctgtttcgaattctatcacaaatgcgataatgaatgtatggaaagtgtgagaaatgggacgtatgactaccctcagtattcagaagaagcaagattaaaaagagaagaaataagcggagtgaaattagagtcaataggaacttaccagatactgtcaatttattcaacagcggcgagttccctagcactggcaatcatgatagctggtctatctttatggatgtgctccaatgggtcgttacagtgcagaatttgcatttag

>H5N1_A/Black-headed_gull/Netherlands/1/2022

cttgttaaaagtgatcagatttgcattggttaccatgcaaacaattcgacagagcaggttgacacgataatggaaaagaacgtcactgttacacatgcccaagacatactggaaaaaacacacaacgggaagctctgtgatttaaatggggtgaagcctctgattttaaaggattgtagtgtagctggatggctcctcggaaacccaatgtgcgacgaattcatcagagtgccggaatggtcctacatagtggagcgggctaatccagctaatgacctctgttacccagggagcctcaatgactatgaagaactgaaacacctgttgagcagaataaatcattttgagaagattctgatcatccccaagagttcctggccaaatcatgaaacatcactaggggtgagcgcagcttgtccataccagggagcgccctcctttttcagaaatgtggtgtggcttatcaaaaagaacgatgcatacccaacaataaagataagctacaataataccaatcgggaagatctcttgatactgtgggggattcatcattccaacaatgcagaagagcagacaaatctctataaaaacccaaccacctacatttcagttggaacatcaactttaaaccagaggttggtaccaaaaatagctactagatcccaagtaaacgggcaacgtggaagaatggacttcttctggacaattttaaaaccagatgatgcaatccatttcgagagtaatggaaatttcattgctccagaatatgcatataaaattgtcaagaaaggggactcaacaattatgaaaagtggagtggaatatggccactgcaacaccaaatgtcaaaccccagtaggagcgataaattctagtatgccattccacaacatacatcctctcaccattggggaatgccccaaatacgtgaagtcaaacaagttggtccttgcgactgggctcagaaatagtcctctaagagaaaagagaagaaaaGGGagaggcctgtttggggcgatagcagggtttatagagggaggatggcagggaatggttgatggttggtatgggtaccatcatagcaatgaacaggggagtgggtacgctgcagacaaagaatccacccaaaaggcaatagatggagttaccaataaggtcaactcaatcattgacaaaatgaacactcaatttgaggcagttggaagggagtttaataacttagaaaggaggatagaaaatttgaacaagaaaatggaagacggattcctagatgtctggacctataatgctgaacttctagttctcatggaaaacgagaggactctagatttccatgattcgaatgtcaagaacctttacgacaaagtcagactacagcttagggataatgcaaaggagctgggtaatggctgtttcgaattctatcacaaatgcgataatgaatgtatggaaagtgtgagaaatgggacgtatgactaccctcagtattcagaagaagcaagattaaaaagagaagaaataagcggagtgaaattagaatcaataggaacttaccagatactgtcaatttattcaacagcggcgagttccctagcactggcaatcatgatagctggtctatctttatggatgtgctccaatgggtcgttacagtgcagaatttgcatttag

>H5N1_A/Branta_leucopsis/Belgium/14735_0001/2021

cttgttaaaagtgatcagatttgcattggttaccatgcaaacaattcgacagagaaggttgacacgataatggaaaagaacgtcactgttacacatgcccaagacatactggaaaaaacacacaacgggaagctctgtgatttaaatggggtgaagcctctgattttaaaggattgtagtgtagctggatggctcctcggaaacccaatgtgcgacgaattcatcagagtgccggaatggtcctacatagtggagcgggctaatccagctaatgacctctgttacccagggagcctcaatgactatgaagaactgaaacacctgttgagcagaataaaccattttgagaagattctgatcatccccaagagttcctggccaaatcatgaaacatcactaggggtgagcgcagcttgtccataccagggagcgccctcctttttcagaaatgtggtgtggcttatcaaaaagaacgatgcatacccaacaataaagataagctacaataataccaatcgggaagatctcttgatactgtgggggattcatcattccaacaatgcagaagaacagacaaatctctataaaaacccaaccacctacatttcagttggaacatcaactttaaaccagaggttggtaccaaaaatagctactagatcccaagtaaacgggcaacgtggaagaatggacttcttctggacaattttaaaaccagatgatgcaatccatttcgagagtaatggaaatttcattgctccagaatatgcatataaaattgtcaagaaaggggactcaacaattatgaaaagtggagtggaatatggccactgcaacaccaaatgtcaaaccccagtaggagcgataaattctagtatgccattccacaacatacatcctctcaccattggggaatgccccaaatacgtgaagtcaaacaagttggtccttgcgactgggctcagaaatagtcctctaagagaaaagagaagaaaaGGGagaggcctgtttggggcgatagcagggtttatagagggaggatggcagggaatggttgatggttggtatgggtaccatcatagcaatgagcaggggagtgggtacgctgcagacaaagaatccacccaaaaggcaatagatggagttaccaataaggtcaactcaatcattgacaaaatgaacactcaatttgaggcagttggaagggagtttaataacttagaaaggaggatagagaatttgaacaagaaaatggaagacggattcctagatgtctggacctataatgctgaacttctagttctcatggaaaacgagaggactctagatttccatgattcaaatgtcaagaacctttacgacaaagtcagactacagcttagggataatgcaaaggagctgggtaatggctgtttcgaattctatcacaaatgcgataatgaatgtatggaaagtgtgagaaatgggacgtatgactaccctcagtattcagaagaagcaagattaaaaagagaagaaataagcggagtgaaattagaatcaataggaacttaccagatactgtcaatttattcaacagcggcgagttccctagcactggcaatcatgatagctggtctatctttatggatgtgctccaatgggtcgttacagtgcagaatttgcatttag

>H5N1_A/Brent_goose/Netherlands/1/2022

cttgttaaaagtgatcagatttgcattggttaccatgcaaataattcgacagagcaggttgacacgataatggaaaagaacgtcactgttacacatgcccaagacatactggaaaaaacacacaacgggaagctctgtgatttaaatggggtgaagcctctgattttaaaggattgtagtgtagctggatggctcctcggaaacccaatgtgcgacgaattcatcagagtgccggaatggtcctacatagtggagcgggctaatccagctaatgacctatgttacccagggagcctcaatgactatgaagaactgaaacacctgttgagcagaataaatcattttgagaagattcttatcatccccaagagttcctggccaaatcatgaaacatcactaggggtgagcgcagcttgtccataccagggagcgccctcctttttcagaaatgtggtgtggcttatcaaaaagaacgatgcatacccaacaataaagataagctacaataataccaatcgggaagatctcttgatactgtgggggattcatcattccaacaatgcagaagaacagacaaatctctataaaaacccaaccacctacatttcagttggaacatcaactttaaaccagaggttggtaccaaaaatagctactagatcccaagtaaacgggcaacgtggaagaatggacttcttctggacaattttaaaaccagatgatgcaatccatttcgagagtaatggaaatttcattgctccagaatatgcatataaaattgtcaagaaaggggactcaacaattatgaaaagtggagtggaatatggccactgcaacaccaaatgtcaaaccccagtaggagcgataaattctagtatgccattccacaacatacatcctctcaccattggggaatgccccaaatacgtgaagtcaaacaagttggtccttgcgactgggctcagaaatagtcctctaagagaaaagagaagaaaaGGGagaggcctgtttggggcgatagcagggtttatagagggaggatggcagggaatggttgatggttggtatgggtaccatcatagcaatgagcaggggagtgggtacgctgcagacaaagaatccacccaaaaggcaatagatggagttaccaataaggtcaactcaatcattgacaaaatgaacactcaatttgaggcagttggaagggagtttaataacttagaaaggaggatagagaatttgaacaagaaaatggaagacggattcctagatgtctggacctataatgctgaacttctagttctcatggaaaacgagaggactctagatttccatgattcaaatgtcaagaacctttacgacaaagtaagactacagcttagggataatgcaaaggagcttggtaatggctgtttcgaattctatcacaaatgcgataatgaatgtatggaaagtgtgagaaatgggacgtatgactaccctcagtattcagaagaagcaagattaaaaagagaagaaataagcggagtgaaattagaatcaataggaacttaccagatactgtcaatttattcaacagcggcgagttccctagcactggcaatcatgatagctggtctatctttatggatgtgctccaatgggtcgttacagtgcagaatttgcatttag

>H5N1_A/Canada goose/Delaware Bay/601/2016

attgtaaaaggcgaccaaatttgcattggttaccatgcaaacaattcaacagagcaggttgatacaatcatggaaaagaatgtgacggtcacacatgctcaggacatactggagaaagaacacaatgggaaactttgcagtcttaaaggagtgaggcccctcattctgaaggactgcagcgtagctggatggcttcttggaaacccaatgtgtgatgaattcctgaatgtaccagaatggtcatacattgtggaaaaagataatccagtcaatggcctgtgctatccaggagacttcagcgactatgaagaactgaagcatttaatgagcagcacaaaccagtttgagaaaattcggataattcctaggagttcttggtccaatcatgatgcctcatcaggagtaagttcggcatgcccatacaatggtagaccttcctttttcaggaatgtagtttggttgatcaagaagaataatgcgtacccaacaatcaagaggacctataacaacaccaatgtagaagaccttttagtaatatggggaatacaccaccctaatgatgcagctgaacaaacacaactctaccagaactcgaacacttatgtgtctgtaggaacatcaacactgaatcaaagatcaatcccagaaatagccactagacccaaagtgaacggacaaagtgggagaatggaatttttctggacaatactgaagtcgaacgatgcaatcagctttgaaagtaacgggaattttatagctcctgaatatgcgtacaaaattgtcaagaaaggagattcagcaatcatgagaagtgaattagagtatggtaactgtgacaccaaatgtcagactccattaggtgctataaattccagtatgcccttccacaatgttcatcctcttaccattggggagtgccccaagtatgtaaaatcggataaactggtccttgcaacgggactaagaaacataccccaaagagaaacaGGGGGGGGGGGGagaggcctatttggtgcaatagcaggattcatagagggaggatggcaaggaatggttgacgggtggtacgggtaccatcacagcaatgagcagggaagtgggtatgctgcagacaaagaatctacccagaaagcaattgatggaatcaccaacaaagtaaactcaatcattgacaaaatgaacactcaattcgaagccgctgggaaagaattcaacaacctggaaaggagaatagaaaacttgaataagaaaatggaggatgggtttttagatgtatggacttacaatgcagaacttcttgtactcatggaaaatgaaagaactctggatttccatgattcaaatgtcaagaacctatatgataaggtccgactccagctgagagacaatgcaaaagaattgggcaacggatgctttgaattctaccacaagtgtgacaatgaatgcatggaaagtgtgagaaatggaacgtatgactatccgcaatattcagaggaatcaagactgaatagagaggaaatagacggagtcaaattggaatcaatgggcacctatcagatattatcaatctacgcaacagtggcgagttccctagcactggcaatcatgatagctggtctatctttttggatgtgttccaatgggtcattgcagtgcagaatttgcatctga

>H5N1_A/Canada_goose/England/385250/2021

cttgttaaaagtgatcagatttgcattggttaccatgcaaacaattcgacagagcaagttgacacgataatggaaaagaacgtcactgttacacatgcccaagacatactggaaaaaacacacaacgggaagctctgtgatctaaatggggtgaagcctctgattttaaaggattgtagtgtagctggatggctcctcggaaacccaatgtgcgacgaattcatcagagtgccggaatggtcctacatagtggagcgggctaatccagctaatgacctctgttacccagggagcctcaatgactatgaagaactgaaacacctgttgagcagaataaatcattttgagaagattctgatcatccccaagagttcctggccaaatcatgaaacatcactaggggtgagcgcagcttgtccataccagggaacgccctcctttttcagaaatgtggtgtggcttatcaaaaagaacgatgcatacccaacaataaagataagctacaataataccaatcgggaagatctcttgatactgtgggggattcatcattccaacaatgcagaagagcagacaaatctctacaaaaacccaaccacctacatttcagttggaacatcaactttaaaccagaggttggtaccaaaaatagctactagatcccaagtaaacgggcaacgtggaagaatggacttcttctggacaattttaaaaccagatgatgcaatccatttcgagagtaatggaaatttcattgctccagaatatgcatacaaaattgtcaagaaaggggactcaacaattatgaaaagtggagtggaatatggccactgcaacaccaaatgtcaaaccccagtaggagcgataaattctagtatgccattccacaacatacatcctctcaccattggggaatgccccaaatacgtgaagtcaaacaagttggtccttgcgactgggcttagaaatagtcctctaagagaaaagagaagaaaaGGGagaggcctgtttggggcgatagcagggtttatagagggaggatggcagggaatggttgatggttggtatgggtaccatcatagcaatgagcaggggagtgggtacgctgcagacaaagaatccacccaaaaggcaatagatggagttaccaataaggtcaactcaatcattgacaaaatgaacactcaatttgaggcagttggaagggagtttaataacttagaaaggaggatagagaatttgaacaagaaaatggaagacggattcctagatgtttggacctataatgctgaacttctagttctcatggaaaacgagaggactctagatttccatgattcaaatgtcaagaacctttacgacaaagtcagactacagcttagggataatgcaaaggagctgggtaatggctgtttcgaattctatcacaaatgcgataatgaatgtatggaaagtgtgagaaatgggacgtatgactaccctcagtattcagaagaagcaagattaaaaagagaagaaataagcggagtgaaattagaatcaataggaacttaccagatactgtcaatttattcaacagcggcaagttccctagcactggcaatcatgatggctggtctatctttatggatgtgctccaatgggtcgttacagtgcagaatttgcatttag

>H5N1_A/Caspian_Gull/Netherlands/2/2022

ctagttaaaagtgatcagatttgcattggttaccatgcaaacaattcgacagagcaggttgacacgataatggaaaagaacgtcactgttacacatgcccaagacatactggaaaaaacacacaacgggaagctctgtgatttaaatggggtgaagcctctgattttaaaggattgtagtgtagctggatggctcctcggaaacccaatgtgcgacgaattcatcagagtgccggaatggtcctacatagtggagcgggctaatccagctaatgacctctgttacccagggagcctcaatgactatgaagaactgaaacacctgttgagcagaataaatcattttgagaagattcttatcatccccaagagttcctggccaaatcatgaaacatcactaggggtgagcgcagcttgtccataccagggagcgccctcctttttcagaaatgtggtgtggcttatcaaaaagaacgatgcatacccaacaataaagataagctacaataataccaatcgggaagatctcttgatactgtgggggattcatcattccaacaatgcagaagaacagacaaatctctataaaaacccaaccacctacatttcagttggaacatcaactttaaaccagaggttggtaccaaaaatagctactagatcccaagtaaacgggcaacgtggaagaatggacttcttctggacaattttaaaaccagatgatgcaatccatttcgagagtaatggaaatttcattgctccagaatatgcatataaaattgtcaagaaaggggactcaacaattatgaaaagtggagtggaatatggccactgcaacaccaaatgtcaaaccccagtaggagcgataaattctagtatgccattccacaacatacatcctctcaccattggggaatgccccaaatacgtgaagtcaaacaagttggtccttgcgactgggctcagaaatagtcctctaagagaaaagagaagaaaaGGGagaggcctgtttggggcgatagcagggtttatagagggaggatggcagggaatggttgatggttggtatgggtaccatcatagcaatgagcaggggagtgggtacgctgcagacaaagaatccacccaaaaggcaatagatggagttaccaataaggtcaactcaatcattgacaaaatgaacactcaatttgaggcagttggaagggagtttaataacttagaaaggagaatagagaatttgaacaagaaaatggaagacggattcctagatgtctggacctataatgctgaacttctagttctcatggaaaacgagaggactctagatttccatgattcaaatgtcaagaacctttacgacaaagtcagactacagcttagggataatgcaaaggagctgggtaatggctgtttcgaattctatcacaaatgcgataatgaatgtatggaaagtgtgagaaatgggacgtatgactaccctcagtattcagaagaagcaagattaaaaagagaagaaataagcggagtgaaattagaatcaataggaacttaccagatactgtcaatttattcaacagcggcgagttccctagcactggcaatcatgatagctggtctatctttatggatgtgctccaatgggtcgttacagtgcagaatttgcatttag

>H5N1_A/Caspian_gull/Netherlands/1/2022

cttgttaaaagtgatcagatttgcattggttaccatgcaaacaattcgacagagcaggttgacacgataatggaaaagaacgtcactgttacacatgcccaagacatactggaaaaaacacacaacgggaagctctgtgatttaaatggggtgaagcctctgattttaaaggattgtagtgtagctggatggctcctcggaaacccaatgtgcgacgaattcatcagagtgccggaatggtcctacatagtggagcgggctaatccagccaatgacctctgttacccagggagcctcaatgactatgaagaactgaaacacctgttgagcagaataaatcattttgagaagattctgatcatccccaagagttcctggccaaatcatgaaacatcactaggggtgagcgcagcttgtccataccagggagcgccctcctttttcagaaatgtgttgtggcttatcaaaaagaacgatgcatacccaacaataaagataagctacaataataccaatcgggaagatctcttgatactgtgggggattcatcattccaacaatgcagaagagcagacaaatctctataaaaacccaaccacctacatttcagttggaacatcaactttaaaccagaggttggtaccaaaaatagctactagatcccaagtaaacgggcagcgtggaagaatggacttcttctggacaattttaaaaccagatgatgcaatccatttcgagagtaatggaaatttcattgcaccagaatatgcatataaaattgtcaagaaaggggactcaacaattatgaaaagtggagtggaatatggccactgcaacaccaaatgtcaaaccccagtaggagcgataaattctagtatgccattccacaacatacatcctctcaccattggggaatgccccaaatacgtgaagtcaaacaagttggtccttgcgactgggctcagaaatagtcctctaagagaaaagagaagaaaaGGGagaggcctgtttggggcgatagcagggtttatagagggaggatggcagggaatggttgatggttggtatgggtaccatcatagcaatgagcaggggagtgggtacgctgcagacaaagaatccacccaaaaggcaatagatggagttaccaataaggtcaactcaatcattgacaaaatgaacactcaatttgaggcagttgggagggagtttaataacttagaaaggaggatagagaatttgaacaagaaaatggaagacggattcctagatgtctggacctataatgctgaacttctagttctcatggaaaacgagaggactctagatttccatgattcaaatgtcaagaacctttatgacaaagtcagactacagcttagggacaatgcaaaggagctgggtaatggctgtttcgaattctatcacaaatgcgataatgaatgtatggaaagtgtgagaaatgggacgtatgactaccctcagtattcagaagaagcaagattaaaaagagaagaaataagcggagtgaaattagaatcaataggaacttaccagatactgtcaatttattcaacagcggcgagttccctagcactggcaatcatgatagctggtctatctttatggatgtgctccaatgggtcgttacagtgcagaatttgcatttag

>H5N1_A/Chicken/Laos/Xaythiani-26/2006

cttgttaaaagtgatcagatttgcattggttaccatgcaaacaactcaacagagcaggttgacacaataatggaaaagaacgttactgttacacatgcccaagatatactggaaaagacacacaacgggaagctctgcgatctagatggagtgaagcctctgattttaagagattgtagtgtagctggatggctcctcggaaacccaatgtgtgacgaattcatcaatgtgccggaatggtcttacatagtggagaaggccaatccagccaatgacctctgttacccagggaatttcaacgactatgaagaactgaaacacctattgagcagaataaaccattttgagaaaattcagatcatccccaaaagttcttggtccgatcatgaagcctcatcaggggtgagctcagcatgtccataccagggaacgccctcctttttcagaaatgtggtatggctcatcaaaaagaacaatacatacccaacaataaagagaagctacaataataccaaccaggaagatcttttggtactgtgggggattcatcattctaatgatgcggcagagcagacaaagctctatcaaaacccaaccacctatatttccgttgggacatcaacactaaaccagagattggtaccaaaaatagctactagatccaaagtaaacgggcaaagtggaaggatggatttcttctggacaatgttaaaaccgaatgatgcaatcaacttcgagagtaatggaaatttcattgctccagaatatgcatacaaaattgtcaagaaaggggactcagcaattatgaaaagtgaagtggaatatggtaactgcaacaccaagtgtcaaactccaataggggcgataaactctagtatgccattccacaacatacaccctctcaccatcggggaatgccccaaatatgtgaaatcaaacaaattagtccttgcgactgggctcagaaatagtcctctaagagaaagaagaagaaaaGGGagaggactatttggagctatagcaggttttatagagggaggatggcagggaatggtagatggttggtatgggtaccaccatagcaatgagcaggggagtgggtacgctgcagacaaagaatccactcaaaaggcaatagatggagtcaccaataaggtcaactcgatcattgacaaaatgaacactcagtttgaggccgttggaagggaatttaataacttagaaaggagaatagagaatttaaacaagaaaatggaagacggattcctagatgtctggacttataatgctgaactcctggttctcatggaaaatgagagaactctagacttccatgactcaaatgtcaagaacctttacgacaaggtccgactacagcttagggataatgcaaaggagctgggtaacggttgttttgagttctatcacaaatgtgataatgaatgcatggaaagtgtaagaaacggaacgtatgactacccgcagtattcagaagaagcaagattaaaaagagaggaaataagtggagtaaagttggaatcaataggaacttaccaaatactgtcaatttattcaacagctgcgagttctctagcactggcaatcatggtggctggtctatctttatggatgtgctccaatgggtcgttacaatgcagaatttgcatttga

>H5N1_A/Chicken/Sweden/SVA211130SZ0427/FB290424-IP-1/M-2021

cttgttaaaagtgatcagatttgcattggttaccatgcaaacaattcgacagagcaagttgacacgataatggaaaagaacgtaactgttacacatgcccaagacatactggaaaaaacacacaacgggaagctctgtgatctaaatggggtgaagcctctgattttaaaggattgtagtgtagctggatggctcctcggaaacccaatgtgcgacgaattcatcagagtgccggaatggtcctacatagtggagcgggctaatccagctaatgacctctgttacccagggagcctcaatgactatgaagaactgaaacacctgttgagcagaataaatcattttgagaagattctgatcatccccaagagttcctggccaaatcatgaaacatcactaggggtgagcgcagcttgtccataccagggagcgccctcctttttcagaaatgtggtgtggcttatcaaaaagaacgatgcatacccaacaataaagataagctacaataataccaatcgggaagatctcttgatactgtgggggattcatcattccaacaatgcagaagagcagacaaatctctacaaaaacccaaccacctacatttcagttggaacatcaactttaaaccagaggttggtaccaaaaatagctactagatcccaagtaaacgggcaacgtggacgaatggacttcttctggacaattttaaaaccagatgatgcaatccatttcgagagtaatggaaatttcattgctccagaatatgcatacaaaattgtcaagaaaggggactcaacaattatgaaaagtggagtggaatatggccactgcaacaccaaatgtcaaaccccagtaggagcgataaattctagtatgccattccacaacatacatcctctcaccattggggaatgccccaaatacgtgaagtcaaacaagttggtccttgcgactgggcttagaaatagtcccctaagagaaaagagaagaaaaGGGagaggcctgtttggggcgatagcagggtttatagagggaggatggcagggaatggttgatggttggtatgggtaccatcatagcaatgagcaggggagtgggtacgctgcagacaaagaatccacccaaaaggcaatagatggagttaccaataaggtcaactcaatcattgacaaaatgaacactcaatttgaggcagttggaagggagtttaataacttagaaaggaggatagagaatttgaacaagaaaatggaagacggattcctagatgtctggacctataatgctgaacttctagttctcatggaaaacgagaggactctagatttccatgattcaaatgtcaagaacctttacgacaaagtcagactacagcttagggataatgcaaaggagctgggtaacggctgtttcgaattctatcacaaatgcgatgatgaatgtatggaaagtgtgagaaatgggacgtatgactaccctcagtattcagaagaagcaagattaaaaagagaagaaataagcggagtgaaattagaatcaataggaacttaccagatactgtcaatttattcaacagcggcaagttccctagcactggcaatcatgatggctggtctatctttatggatgtgctccaatgggtcgttacagtgcagaatttgcatttag

>H5N1_A/Common_Gull/Netherlands/1/2022

cttgttaaaagtgatcagatttgcattggttaccatgcaaacaattcgacagagcaggttgacacgataatggaaaagaacgtcactgttacacatgcccaagacatactggaaaaaacacacaacgggaagctctgtgatttaaatggggtgaagcctctgattttaaaggattgtagtgtagctggatggctcctcggaaacccaatgtgcgacgaattcatcagagtgccggaatggtcctacatagtggagcgggctaatccagctaatgacctctgttacccagggagcctcaatgactatgaagaactgaaacacctgttgagcagaataaatcattttgagaagattcttatcatccccaagagttcctggccaaatcatgaaacatcactaggggtgagcgcagcttgtccataccagggagcgccctcctttttcagaaatgtggtgtggcttatcaaaaagaacgatgcatacccaacaataaagataagctacaataataccaatcgggaagatctcttgatactgtgggggattcatcattccaacaatgcagaagaacagacaaatctctataaaaacccaaccacctacatttcagttggaacatcaactttaaaccagaggttggtaccaaaaatagctactagatcccaagtaaacgggcaacgtggaagaatggacttcttctggacaattttaaaaccagatgatgcaatccatttcgagagtaatggaaatttcattgctccagaatatgcatataaaattgtcaagaaaggggactcaacaattatgaaaagtggagtggaatatggccactgcaacaccaaatgtcaaaccccagtaggagcgataaattctagtatgccattccacaacatacatcctctcaccattggggaatgccccaaatacgtgaagtcaaacaagttggtccttgcgactgggctcagaaatagtcctctaagagaaaagagaagaaaaGGGagaggcctgtttggggcgatagcagggtttatagagggaggatggcagggaatggttgatggttggtatgggtaccatcatagcaatgagcaggggagtgggtacgctgcagacaaagaatccacccaaaaggcaatagatggagttaccaataaggtcaactcaatcattgacaaaatgaacactcaatttgaggcagttggaagggagtttaataacttagaaaggaggatagagaatttgaacaagaaaatggaagacggattcctagatgtctggacctataatgctgaacttctagttctcatggaaaacgagaggactctagatttccatgattcaaatgtcaagaacctttacgacaaagtcagactacagcttagggataatgcaaaggagctgggtaatggctgtttcgaattctatcacaaatgcgataatgaatgtatggaaagtgtgagaaatgggacgtatgactaccctcagtattcagatgaagcaagattaaaaagagaagaaataagcggagtgaaattagaatcaataggaacttaccagatactgtcaatttattcaacagcggcgagttccctagcactggcaatcatgatagctggtctatctttatggatgtgctccaatgggtcgttacagtgcagaatttgcatttag

>H5N1_A/Cygnus cygnus/Iran/754/2006

cttgttaaaagtgatcagatttgcattggttaccatgcaaacaactcgacagagcaggttgacacaataatggaaaagaacgtcactgttacacacgcccaagatatactggaaaaggcacacaacgggaagctctgcgatctagacggagtgaagcctctaattttaagagattgtagtgtagctggatggctcctcgggaacccaatgtgtgacgaattcctcaatgtgccggaatggtcttacatagtggagaagatcaatccagccaatgacctctgttacccagggaatttcaacgactatgaagaactgaaacacctattgagcagaataaaccattttgagaaaattcagatcatccccaaaagttcttggtcagatcatgaagcctcatcaggggtgagctcagcatgtccataccagggaaggtcctccttttttagaaatgtggtatggcttatcaaaaagaacgatgcatacccaacaataaagagaagttacaataataccaaccaagaagatcttttggtactgtgggggattcaccatccaaatgatgcggcagagcagacaaggctctatcaaaacccaaccacctatatttccgttgggacatcaacactaaaccagagattggtaccaaaaatagctactagatccaaggtaaacgggcaaagtggaaggatggagttcttttggacaattttaaaaccgaatgatgcaataaactttgagagtaatggaaatttcattgctccagaaaatgcatacaaaattgtcaagaaaggggactcaacaatcatgaaaagtgaattggaatatggtaactgcaacaccaagtgtcaaactccaataggggcgataaactctagtatgccattccacaacatccaccctctcaccatcggggaatgccccaaatatgtgaaatcaaacagattagtccttgcgactgggctcagaaatagccctcaaggagagagaagaagaaaaaagagaggactatttggagctatagcaggttttatagagggaggatggcagggaatggtagatggttggtatgggtaccaccatagcaacgagcaggggagtgggtacgctgcagacaaagaatccactcaaaaggcaatagatggagtcaccaataaggtcaactcgatcattgacaaaatgaacactcagtttgaggccgttggaagggaatttaataacttagaaaggagaatagaaaatttaaacaagaagatggaagacggattcctagatgtctggacttataatgctgaacttctggttctcatggaaaatgagagaactctagactttcatgactcaaatgtcaagaatctttacgacaaggtccgactacagcttagggataatgcaaaggagcttggtaacggttgtttcgagttctatcacagatgtgataatgaatgtatggaaagtgtaagaaacggaacgtatgactacccgcagtattcagaagaagcaagattaaaaagagaggaaataagtggagtaaaattggaatcaataggaacttaccaaatactgtcaatttattcaacagtggcgagctccctagcactggcaatcatggtggctggtctatctttatggatgtgctccaatggatcgttacaatgcagaatttgcatttga

>H5N1_A/Cygnus olor/Astrakhan/Ast05-2-1/2005

cttgttaaaagtgatcagatttgcattggttaccatgcaaacaactcgacagagcaggttgacacaataatggaaaagaacgtcactgttacacacgcccaagacatactggaaaagacacacaacgggaagctctgcgatctagatggagtgaagcctctaattttaagagattgtagtgtagctggatggctcctcgggaacccaatgtgtgacgaattcctcaatgtgccggaatggtcttacatagtggagaagatcaatccagccaatgacctctgttacccagggaatttcaacgactatgaagaactgaaacacctattgagcagaataaaccattttgagaaaattcagatcatccccaaaagttcttggtcagatcatgaagcctcatcaggggtgagctcagcatgtccataccagggaaggtcctccttttttagaaatgtggtatggcttatcaaaaaggacaatgcatacccaacaataaagagaagttacaataataccaaccaagaagatcttttggtactgtgggggattcaccatccaaatgatgcggcagagcagacaaggctctatcaaaacccaaccacctatatttccgttgggacatcaacrctaaaccagagattggtaccaaaaatagctactagatccaaggtaaacgggcaaagtggaaggatggagttcttttggacaattttaaaaccgaatgatgcaataaactttgagagtaatggaaatttcattgctccagaaaatgcatacaaaattgtcaagaaaggggactcaacaattatgaaaagtgaattggaatatggtaactgcaacaccaagtgtcaaactccaataggggcgataaactctagtatgccattccacaacatccaccctctcaccatcggggaatgccccaaatatgtgaaatcaaacagattagtccttgcgactgggctcagaaatagccctcaaggagagagaagaagaaaaaagagaggactatttggagctatagcaggttttatagarggaggatggcagggaatggtagatggttggtatgggtaccatcatagcaacgarcaggggagtgggtacgctgcagacaaagaatccactcaaaaggcaatagatggagtcaccaataaggtcaactcgatcattgacaaaatgaacactcagtttgaggccgttggaagggaatttaataacttagaaaggagaatagaaaatttaaacaagaagatggaagacggattcctagatgtctggacttataatgctgaacttctggttctcatggaaaatgagagaactctagactttcatgactcaaatgtcaagaacctttacgacaaggtccgactacagcttagggataatgcaaaggaacttggtaacggttgtttcgagttctatcacagatgtgataatgaatgtatggaaagtgtaagaaacggtacgtatgactacccgcagtattcagaagaagcaagattaaaaagagaggaaataagtggagtaaaattggaatcaataggaacctaccaaatactgtcaatttattcaacagtggcgagctccctagcactggcaatcatggtggctggtctatctttatggatgtgctccaatggatcgttgcaatgcagaatttgcatttga

>H5N1_A/Cygnus olor/Astrakhan/Ast05-2-2/2005

cttgttaaaagtgatcagatttgcattggttaccatgcaaacaactcgacagagcaggttgacacaataatggaaaagaacgtcactgttacacacgcccaagacatactggaaaagacacacaacgggaagctctgcgatctagatggagtgaagcctctaattttaagagattgtagtgtagctggatggctcctcgggaacccaatgtgtgacgaattcctcaatgtgccggaatggtcttacatagtggagaagatcaatccagccaatgacctctgttacccagggaatttcaacgactatgaagaactgaaacacctattgagcagaataaaccattttgagaaaattcagatcatccccaaaagttcttggtcagatcatgaagcctcatcaggggtgagctcagcatgtccataccagggaaggtcctccttttttagaaatgtggtatggcttatcaaaaaggacaatgcatacccaacaataaagagaagttacaataataccaaccaagaagatcttttggtactgtgggggattcaccatccaaatgatgcggcagagcagacaaggctctatcaaaacccaaccacctatatttccgttgggacatcaacactaaaccagagattggtaccaaaaatagctactagatccaaggtaaacgggcaaagtggaaggatggagttcttttggacaattttaaaaccgaatgatgcaataaactttgagagtaatggaaatttcattgctccagaaaatgcatacaaaattgtcaagaaaggggactcaacaattatgaaaagtgaattggaatatggtaactgcaacaccaagtgtcaaactccaataggggcgataaactctagtatgccattccacaacatccaccctctcaccatcggggaatgccccaaatatgtgaaatcaaacagattagtccttgcgactgggctcagaaatagccctcaaggagagagaagaagaaaaaagagaggactatttggagctatagcaggttttatagaaggaggatggcagggaatggtagatggttggtatgggtaccatcatagcaacgagcaggggagtgggtacgctgcagacaaagaatccactcaaaaggcaatagatggagtcaccaataaggtcaactcgatcattgacaaaatgaacactcagtttgaggccgttggaagggaatttaataacttagaaaggagaatagaaaatttaaacaagaagatggaagacggattcctagatgtctggacttataatgctgaacttctggttctcatggaaaatgagagaactctagactttcatgactcaaatgtcaagaacctttacgacaaggtccgactacagcttagggataatgcaaaggaacttggtaacggttgtttcgagttctatcacagatgtgataatgaatgtatggaaagtgtaagaaacggtacgtatgactacccgcagtattcagaagaagcaagattaaaaagagaggaaataagtggagtaaaattggaatcaataggaacctaccaaatactgtcaatttattcaacagtggcgagctccctagcactggcaatcatggtggctggtctatctttatggatgtgctccaatggatcgttgcaatgcagaatttgcatttga

>H5N1_A/Cygnus olor/Astrakhan/Ast05-2-3/2005

cttgttaaaagtgatcagatttgcattggttaccatgcaaacaactcgacagagcaggttgacacaataatggaaaagaacgtcactgttacacacgcccaagacatactggaaaagacacacaacgggaagctctgcgatctagatggagtgaagcctctaattttaagagattgtagtgtagctggatggctcctcgggaacccaatgtgtgacgaattcctcaatgtgccggaatggtcttacatagtggagaagatcaatccagccaatgacctctgttacccagggaatttcaacgactatgaagaactgaaacacctattgagcagaataaaccattttgagaaaattcagatcatccccaaaagttcttggtcagatcatgaagcctcatcaggggtgagttcagcatgtccataccagggaaggtcctccttttttagaaatgtggtatggcttatcaaaaaggacaatgcatacccaacaataaagagaagttacaataataccaaccaagaagatcttttggtactgtgggggattcaccatccaaatgatgcggcagagcagacaaggctctatcaaaacccaaccacctatatttccgttgggacatcaacgctaaaccagagattggtaccaaaaatagctactagatccaaggtaaacgggcaaagtggaaggatggagttcttttggacaattttaaaaccgaatgatgcaataaactttgagagtaatggaaatttcattgctccagaaaatgcatacaaaattgtcaagaaaggggactcaacaattatgaaaagtgaattggaatatggtaactgcaacaccaagtgtcaaactccaataggggcgataaactctagtatgccattccacaacatccaccctctcaccatcggggaatgccccaaatatgtgaaatcaaacagattagtccttgcgactgggctcagaaatagccctcaaggagagagaagaagaaaaaagagaggactatttggagctatagcaggttttatagagggaggatggcagggaatggtagatggttggtatgggtaccaccatagcaacgaacaggggagtgggtacgctgcagacaaagaatccactcaaaaggcaatagatggagtcaccaataaggtcaactcgatcattgacaaaatgaacactcagtttgaggccgttggaagggaatttaataacttagaaaggagaatagaaaatttaaacaagaagatggaagacggattcctagatgtctggacttataatgctgaacttctggttctcatggaaaatgagagaactctagactttcatgactcaaatgtcaagaacctttacgacaaggtccgactacagcttagggataatgcaaaggagcttggtaacggctgtttcgagttctatcacagatgtgataatgaatgtatggaaagtgtaagaaacggaacgtatgactacccgcagtattcagaagaagcaagatttaaaagagaggaaataagtggagtaaaattggaatcaataggaacctaccaaatactgtcaatttattcaacagtggcgagctccctagcactggcaatcatggtggctggtctatctttatggatgtgctccaatggatcgttacaatgcagaatttgcatttga

>H5N1_A/Cygnus olor/Astrakhan/Ast05-2-7/2005

cttgttaaaagtgatcagatttgcattggttaccatgcaaacaactcgacagagcaggttgacacaataatggaaaagaacgtcactgttacacacgcccaagacatactggaaaagacacacaacgggaagctctgcgatctagatggagtgaagcctctaattttaagagattgtagtgtagctggatggctcctcgggaacccaatgtgtgacgaattcctcaatgtgccggaatggtcttacatagtggagaagatcaatccagccaatgacctctgttacccagggaatttcaacgactatgaagaactgaaacacctattgagcagaataaaccattttgagaaaattcagatcatccccaaaagttcttggtcagatcatgaagcctcatcaggggtgagctcagcatgtccataccagggaaggtcctccttttttagaaatgtggtatggcttatcaaaaaggacaatgcatacccaacaataaagagaagttacaataataccaaccaagaagatcttttggtactgtgggggattcaccatccaaatgatgcggcagagcagacaaggctctatcaaaacccaaccacctatatttccgttgggacatcaacactaaaccagagattggtaccaaaaatagctactagatccaaggtaaacgggcaaagtggaaggatggagttcttttggacaattttaaaaccgaatgatgcaataaactttgagagtaatggaaatttcattgctccagaaaatgcatacaaaattgtcaagaaaggggactcaacaattatgaaaagtgaattggaatatggtaactgcaacaccaagtgtcaaactccaataggggcgataaactctagtatgccattccacaacatccaccctctcaccatcggggaatgccccaaatatgtgaaatcaaacagattagtctttgcgactgggctcagaaatagccctcaaggagagagaagaagaaaaaagagaggactatttggagctatagcaggttttatagaaggaggatggcagggaatggtagatggttggtatgggtaccatcatagcaacgagcaggggagtgggtacgctgcagacaaagaatccactcaaaaggcaatagatggagtcaccaataaagtcaactcgatcattgacaaaatgaacactcagtttgaggccgttggaagggaatttaataacttagaaaggagaatagaaaatttaaacaagaagatggaagacggattcctagatgtctggacttataatgctgaacttctggttctcatggaaaatgagagaactctagactttcatgactcaaatgtcaagaacctttacgacaaggtccgactacagcttagggataatgcaaaggaacttggtaacggttgtttcgagttctatcacagatgtgataatgaatgtatggaaagtgtaagaaacggtacgtatgactacccgcagtattcagaagaagcaagattaaaaagagaggaaataagtggagtaaaattggaatcaataggaacctaccaaatactgtcaatttattcaacagtggcgagctccctagcactggcaatcatggtggctggtctatctttatggatgtgctccaatggatcgttgcaatgcagaatttgcatttga

>H5N1_A/Cygnus olor/Astrakhan/Ast05-2-8/2005

cttgttaaaagtgatcagatttgcattggttaccatgcaaacaactcgacagagcaggttgacacaataatggaaaagaacgtcactgttacacacgcccaagacatactggaaaagacacacaacgggaagctctgcgatctagatggagtgaagcctctaattttaagagattgtagtgtagctggatggctcctcgggaacccaatgtgtgacgaattcctcaatgtgccggaatggtcttacatagtggagaagatcaatccagccaatgacctctgttacccagggaatttcaacgactatgaagaactgaaacacctattgagcagaataaaccattttgagaaaattcagatcatccccaaaagttcttggtcagatcatgaagcctcatcaggggtgagctcagcatgtccataccagggaaggtcctccttttttagaaatgtggtatggcttatcaaaaaggacaatgcatacccaacaataaagagaagttacaataataccaaccaagaagatcttttggtactgtgggggattcaccatccaaatgatgcggcagagcagacaaggctctatcaaaacccaaccacctatatttccgttgggacatcaacactaaaccagagattggtaccaaaaatagctactagatccaaggtaaacgggcaaagtggaaggatggagttcttttggacaatcttaaaaccgaatgatgcaataaactttgagagtaatggaaatttcattgctccagaaaatgcatacaaaattgtcaagaaaggggactcaacaattatgaaaagtgaattggaatatggtaactgcaacaccaagtgtcaaactccaataggggcgataaactctagtatgccattccacaacatccaccctctcaccatcggggaatgccccaaatatgtgaaatcaaacagattagtccttgcgactgggctcagaaatagccctcaaggagagagaagaagaaaaaagagaggactatttggagctatagcaggttttatagaaggaggatggcagggaatggtagatggttggtatgggtaccatcatagcaacgagcaggggagtgggtacgctgcagacaaagaatccactcaaaaggcaatagatggagtcaccaataaggtcaactcgatcattgacaaaatgaacactcagtttgaggccgttggaagggaatttaataacttagaaaggagaatagaaaatttaaacaagaagatggaagacggattcctagatgtctggacttataatgctgaacttctggttctcatggaaaatgagagaactctagactttcatgactcaaatgtcaagaacctttacgacaaggtccgactacagcttagggataatgcaaaggaacttggtaacggttgtttcgagttctatcacagatgtgataatgaatgtatggaaagtgtaagaaacggtacgtatgactacccgcagtattcagaagaagcaagattaaaaagagaggaaataagtggagtaaaattggaatcaataggaacctaccaaatactgtcaatttattcaacagtggcgagctccctagcactggcaatcatggtggctggtctatctttatggatgtgctccaatggatcgttgcaatgcagaatttgcatttga

>H5N1_A/Cygnus olor/Caspian Sea/2006

cttgttaaaagtgatcagatttgcattggttaccatgcaaacaactcgacagagcaggttgacacaataatggaaaagaacgtcactgttacacacgcccaagacatactggaaaagacacacaacgggaagctctgcgatctagatggagtgaagcctctaattttaagagattgtagtgtagctggatggctcctcgggaacccaatgtgtgacgaattcctcaatgtgccggaatggtcttacatagtggagaagatcaatccagccaatgacctctgttacccagggaatttcaacgactatgaagaactgaaacacctattgagcagaataaaccattttgagaaaattcagatcatccccaaaagttcttggtcagatcatgaagcctcatcaggggtgagctcagcatgtccataccagggaaggtcctccttttttagaaatgtggtatggcttatcaaaaaggacaatgcatacccaacaataaagagaagttacaataataccaaccaagaagatcttttggtactgtgggggattcaccatccaaatgatgcggcagagcagacaaggctctatcaaaactcaaccacctatatttccgttgggacatcaacactaaaccagagattggtaccaaaaatagctactagatccaaggtaaacgggcaaagtggaaggatggagttcttttggacaattttaaaaccgaatgatgcaataaactttgagagtaatggaaatttcattgctccagaaaatgcatacaaaattgtcaagaaaggggactcaacaattatgaaaagtgaattggaatatggtaactgcaacaccaagtgtcaaactccaataggggcgataaactctagtatgccattccacaacatccaccctctcaccatcggggaatgccccaaatatgtgaaatcaaacagattagtccttgcgactgggctcagaaatagccctcaaggagagagaagaagaaaaaagagaggactatttggagctatagcaggttttatagaaggaggatggcagggaatggtagatggttggtatgggtaccatcatagcaacgagcaggggagtgggtacgctgcagacaaagaatccactcaaaaggcaatagatggagtcaccaataaggtcaactcgatcattgacaaaatgaacactcagtttgaggccgttggaagggaatttaataacttagaaaggagaatagaaaatttaaacaagaagatggaagacggattcctagatgtctggacttataatgctgaacttctggttctcatggaaaatgagagaactctagactttcatgactcaaatgtcaagaacctttacgacaaggtccgactacagcttagggataatgcaaaggaacttggtaacggttgtttcgagttctatcacagatgtgataatgaatgtatggaaagtgtaagaaacggtacgtatgactacccgcagtattcagaagaagcaagattaaaaagagaggaaataagtggagtaaaattggaatcaataggaacctaccaaatactgtcaatttattcaacagtggcgagctccctagcactggcaatcatggtggctggtctatctttatggatgtgctccaatggatcgttgcaatgcagaatttgcatttga

>H5N1_A/Cygnus_olor/Romania/16381_21VIR10306/2021

cttgttaaaagtgatcagatttgcattggttaccatgcaaacaattcgacagagcaggttgacacgataatggaaaagaacgtcactgttacacatgcccaagacatactggaaaaaacacacaacgggaagctctgtgatttaaatggggtgaagcctctgattttaaaggattgtagtgtagctggatggctcctcggaaacccaatgtgcgacgaattcatcagggtgccggaatggtcctacatagtggagcgggctaatccagccaatgacctctgttacccagggagcctcaatgactatgaagaactgaaacacctgttgagcagaataaatcattttgagaagattctgatcatccccaagagttcctggccaaatcatgaaacatcactaggggtgagcgcagcttgtccataccagggagcgccctcctttttcagaaatgtggtgtggcttgtcaaaaagaacgatgcatacccaacaataaagataagctacaataatacaaatcgggaagatctcttgatactgtgggggattcatcattccaacaatgcagaagagcagacaaatctctataaaaacccaaccacctacatttcagttggaacatcaactttaaaccagaggttggtaccaaaaatagctactagatcccaagtaaacgggcaacgtggaagaatggacttcttctggacaattttaaaaccagatgatgcaatccatttcgagagtaatggaaatttcattgcaccagaatatgcatataaaattgtcaagaaaggggactcaacaattatgaaaagtggagtggaatatggccactgcaacaccaaatgtcaaaccccagtaggagcgataaattctagtatgccattccacaacatacatcctctcaccattggggaatgccccaaatacgtgaagtcaaacaagttggtccttgcgactgggctcagaaatagtcctctaagagaaaagagaagaaaaGGGagaggcctgtttggggcgatagcagggtttatagagggaggatggcagggaatggttgatggttggtatgggtaccatcatagcaatgagcaggggagtgggtacgctgcagacaaagaatccacccaaaaggcaatagatggagttaccaataaggtcaactcaatcattgacaaaatgaacactcaatttgaggcagttggaagggagtttaataacttagaaaggaggatagagaatttgaacaagaaaatggaagacggattcctagatgtctggacctataatgctgaacttctagttctcatggaaaacgagaggactctagatttccatgattcaaatgtcaagaacctttacgacaaagtcagactacagcttagggataatgcaaaggagctgggtaatggctgtttcgaattctatcacaaatgcgataatgaatgtatggaaagtgtgagaaatgggacgtatgactaccctcagtattcagaagaagcaagattaaaaagagaagaaataagcggagtgaaattagaatcaataggaacttaccagatactgtcaatttattcaacagcggcgagttccctagcactggcaatcatgatagctggtctatctttatggatgtgctccaatgggtcgttacagtgcagaatttgcatttag

>H5N1_A/Domestic duck/Viet Nam/A_duck_Vietnam_HU12-1542_2019/2019

cttgtcaaaagcgatcatatttgcattggttaccatgcaaataactcgacagagcaggtcgacacaataatggaaaaaaacgttactgttacacaagcccaagacatactggaaaagacacacaacgggaagctctgcgatctaaatggagtgaagcctctgattttaaaagattgtagtgtagcaggatggctcctcggaaatccattgtgtgacgaattcaccaatgtgccagaatggtcctacatagtagagaaggccaacccagccaatgacctctgttacccagggaatttcaatgattatgaagaattgaaacacctattgagcaggataaaccattttgagaaaatacagatcatccccaaaaactcttggtcagatcatgaagcctcactgggggtaagcgccgcgtgttcataccagggaaattcctccttcttcagaaatgtggtgtggcttatcaaaaaggacaatgcatacccaacaataaagaaaggctacaataacaccaatcgggaagatctcttgatactgtggggaatccaccatcctaatgatgaagcagagcagacgaggctctaccaaaacccaactacctatatttccattgggacttcaacattaaaccagaggttggtgccaaaaatagccactagacccaaaataaacgggcaaagtggcaggatagatttcttctggacaattttaaaaccgaatgacgcaatccacttcgagagtaatggaaatttcattgctccagaatatgcatacaaaattgtcaagaagggagactccacaatcatgagaagtgaagtacaatatggcaactgcaacaccaggtgtcagactccaataggagcgataaactctagtatgccattccacaacatacaccctctcactatcggagaatgccccaaatatgtgaaatcaagcaaattagtccttgcaaccgggctcagaaatagtcctcaaagagagagaagaagaaaaGGGagaggactgtttggagctatagcaggctttatagagggaggttggcaaggaatggtagatggttggtatgggtaccaccacagtaatgaacaggggagtggttacgctgcagacaaagaatctactcaaaaggcgatagacggggtcaccaataaggtcaattcgatcattgacaaaatgaacactcagtttgaggctgtaggaagggaatttaataacttagagaggagaatagaaaatctaaacaagaagatggaagatggattcctagatgtctggacttataatgctgaacttctggttctcatggagaatgagagaactctagacttccatgactcaaatgtcaagaacctttatgataaggtccgactacagcttaaggataatgcaaaagaactgggaaatggttgtttcgagttctatcacaaatgtaataatgaatgcatggaaagtgtaagaaacgggacgtatgactacccgcagtattcagaagaagcaagattaaaaagagaggaaataagtggagtaaaattggaatcaataggaatctaccaaatactgtcaatttatgcaacagtggcgagttccctagtgctggcaatcatgatggctggtctatctttatggatgtgttccaacgggtcgttacagtgcagaatttgcatttga

>H5N1_A/Domestic duck/Viet Nam/A_duck_Vietnam_HU12-1587_2019/2019

cttgtcaaaagcgatcatatttgcattggttaccatgcaaataactcgacagagcaggtcgacacaataatggaaaaaaacgttactgttacacaagcccaagacatactggaaaagacacacaacgggaagctctgcgatctaaatggagtgaagcctctgattttaaaagattgtagtgtagcaggatggctcctcggaaatccattgtgtgacgaattcaccaatgtgccagaatggtcctacatagtagagaaggccaacccagccaatgacctctgttacccagggaatttcaatgattatgaagaattgaaacacctattgagcaggataaaccattttgagaaaatacagatcatccccaaaaactcttggtcagatcatgaagcctcactgggggtaagcgccgcgtgttcataccagggaaattcctccttcttcagaaatgtggtgtggcttatcaaaaaggacaatgcatacccaacaataaagaaaggctacaataacaccaatcgggaagatctcttgatactgtggggaatccaccatcctaatgatgaagcagagcagacgaggctctaccaaaacccaactacctatatttccattgggacttcaacattaaaccagaggttggtgccaaaaatagccactagacccaaaataaacgggcaaagtggcaggatagatttcttctggacaattttaaaaccgaatgacgcaatccacttcgagagtaatggaaatttcattgctccagaatatgcatacaaaattgtcaagaagggagactccacaatcatgagaagtgaagtacaatatggcaactgcaacaccaggtgtcagactccaataggagcgataaactctagtatgccattccacaacatacaccctctcactatcggagaatgccccaaatatgtgaaatcaagcaaattagtccttgcaaccgggctcagaaatagtcctcaaagagagagaagaagaaaaGGGagaggactgtttggagctatagcaggctttatagagggaggttggcaaggaatggtagatggttggtatgggtaccaccacagtaatgaacaggggagtggttacgctgcagacaaagaatctactcaaaaggcgatagacggggtcaccaataaggtcaattcgatcattgacaaaatgaacactcagtttgaggctgtaggaagggaatttaataacttagagaggagaatagaaaatctaaacaagaagatggaagatggattcctagatgtctggacttataatgctgaacttctggttctcatggagaatgagagaactctagacttccatgactcaaatgtcaagaacctttatgataaggtccgactacagcttaaggataatgcaaaagaactgggaaatggttgtttcgagttctatcacaaatgtaataatgaatgcatggaaagtgtaagaaacgggacgtatgactacccgcagtattcagaagaagcaagattaaaaagagaggaaataagtggagtaaaattggaatcaataggaatctaccaaatactgtcaatttatgcaacagtggcgagttccctagtgctggcaatcatgatggctggtctatctttatggatgtgttccaacgggtcgttacagtgcagaatttgcatttga

>H5N1_A/Duck/Hong Kong/380.5/2001

cttgttaaaagtgatcagatttgcattggttaccatgcaaacaactcgacagaactggttgacacaataatggaaaagaacgttactgttacacatgcccaagacatactggaaaagacacacaacgggaagctctgcgatctagatggagtgaagcctctaattttgagagattgtagtgtggctggatggctcctcggaaacccaatgtgtgacgaattcatcaatgtgccggaatggtcttacatagtggagaaggccaatccagccaatgacctctgttacccaggggatttcaacgactatgaagaactgaaacacctattgagcagaataaaccattttgagaaaattcagatcatccccaaaagttcttggtccaatcatgaagcctcatcaggggtgagctcagcatgtccataccaggggaagtcctcctttttcagaaatgtggtatggcttatcaaaaagaacagtgcatacccaacaataaagaggagctacaataataccaaccaagaagatcttttggtactgtgggggattcaccatcctaatgatgcggcagagcagacaaagctctatcaaaacccaaccacctatatttccgttggaacatcaacactaaaccagagattggtaccaaaaatagctactagatccaaagtaaacggacaaagtggaagaatggagttcttctggacaattttaaagccgaatgatgctatcaatttcgagagtaatggaaatttcattgctccagaatatgcatacaaaattgtcaagaaaggggactcagcaattatgaaaagtgaattggaatatggtaactgcaacaccaagtgtcaaactccactgggggcgataaactctagtatgccattccacaacatacaccctctcaccatcggggaatgccccaaatatgtgaagtcaaacagattagtccttgcgactggactcagaaatacccctcaaagagagagaagaagaaaaaagagaggactatttggagccatagcaggttttatagagggaggatggcagggaatggtagatggttggtatgggtaccaccatagcaatgagcaggggagtggatacgctgcagacaaagaatccactcaaaaggcaatagatggagtcaccaataaggtcaactcgatcattgacaaaatgaacactcaatttgaagccgttggaagggaatttaataacttagaaaggagaatagaaaatttaaacaagaagatggaagacggattcctagatgtttggacttataatgctgaacttctggttctcatggaaaatgagagaactctagactttcatgactcaaatgtcaagaacctttacgacaaggtccgactacagcttagggataatgcaaaggggctgggtaacggttgtttcgagttctatcacaaatgtgataatgaatgtatggaaagtgtaaaaaacggaacgtatgactacccgcagtattcagaagaagcaagactaaacagagaggaaataagtggagtaaaattggaatcaatgggaacttaccaaatactgtcaatttattcaacagtggcgagttccctagcactggcaatcatggtagctggtctatctttatggatgtgctccaatggatcgttacaatgcagaatttgcatttga

>H5N1_A/Eurasian eagle owl/Korea/23/2010

cttgttaaaagcgatcatatttgcattggttatcatgcaaataactcgacagagcaggttgacacaataatggaaaagaacgttactgttacacatgcccaagacatactggaaaagacacacaacgggaagctctgcgatctaaatggagtgaagcctctgattttaaaagattgtagtgtagcgggatggctcctcggaaacccattgtgtgacgaattcatcaatgtgccagaatggtcttacatagtagagaaggccaagccagccaatgacctctgttacccagggaatttcaacgattatgaagaattgaaacacctattgagcaggataaaccattttgagaaaatacagatcatccccaaatactcttggtcagatcatgaagcctcattgggggtgagcgcagcatgttcataccagggaaattcctccttcttcagaaatgtggtatggcttatcaaaaaggacaatgcatacccaacaataaagaaaggctacaataataccaaccaagaagatctcttggtactgtgggggattcaccatcctaatgatgaggcagagcagacaaggctctatcaaaacccaaccacctatatttccattgggacatcaacactaaaccagagattggtaccaaaaatagccactagatccaaaataaacgggcaaagtggcaggatagatttcttctggacaattttaaaaccgaatgatgcaatccacttcgagagtaatggaaatttcattgctccagaatatgcatacaaaattgtcaagaaaggagactccacaattatgaaaagtgaagtggaatatggtaactgcaacaccaggtgtcagactccgataggggcgataaactctagtatgccattccacaacatacaccctctcaccatcggagaatgtcccaaatatgtgaaatcaaacaaattagtccttgcgactgggctcagaaatagtcctcaaagagagagaagaagaaaaGGGagaggactgtttggagctatagcaggttttatagagggaggatggcagggaatggtagatggttggtatgggtaccaccacagcaatgagcaggggagtgggtacgctgcagacaaagaatctactcaaaaggcaatagacggagtcaccaataaggtcaactcgatcattgacaaaatgaacactcagtttgaggccgtgggaagggaatttaataacttagagaggagaatagagaatttaaacaagaagatggaagacggattcctagatgtttggacttataatgctgaacttctggttctcatggaaaatgagagaactctagatttccatgactcaaatgtcaagaacctttacgataaggtcagactacagcttaaggataatgcaaaagagttgggtaacggttgtttcgagttctatcacaaatgtaataatgaatgtatggaaagtgtaagaaacggaacgtatgactacccgcagtattcaaaagaagcaagactaaaaagagaggaaataagtggagtaaaattggaatcaataggaatctaccaaatactgtcaatttattcaacagtggcgagttccctagtgctggcaatcatgatggctggtctgtctttatggatgtgttccaacggatcgttacagtgcagaatttgcatttga

>H5N1_A/Eurasian_Curlew/Netherlands/1/2022

ctagttaaaagtgatcagatttgcattggttaccatgcaaacaattcgacagagcaggttgacacgataatggaaaagaacgtcactgttacacatgcccaagacatactggaaaaaacacacaacgggaagctctgtgatttaaatggggtgaagcctctgattttaaaggattgtagtgtagctggatggctcctcggaaacccaatgtgcgacgaattcatcagagtgccggaatggtcctacatagtggagcgggctaatccagctaatgacctctgttacccagggagcctcaatgactatgaagaactgaaacacctgttgagcagaataaatcattttgagaagattcttatcatccccaagagttcctggccaaatcatgaaacatcactaggggtgagcgcagcttgtccataccagggagcgccctcctttttcagaaatgtggtgtggcttatcaaaaagaacgatgcatacccaacaataaagataagctacaataataccaatcgggaagatctcttgatactgtgggggattcatcattccaacaatgcagaagaacagacaaatctctataaaaacccaaccacctacatttcagttggaacatcaactttaaaccagaggttggtaccaaaaatagctactagatcccaagtaaacgggcaacgtggaagaatggacttcttctggacaattttaaaaccagatgatgcaatccatttcgagagtaatggaaacttcattgctccagaatatgcatataaaattgtcaagaaaggggactcaacaattatgaaaagtggagtggaatatggccactgcaacaccaaatgtcaaaccccagtaggagcgataaattctagtatgccattccacaacatacatcctctcaccattggggaatgccccaaatacgtgaagtcaaacaagttggtccttgcgactgggctcagaaatagtcctctaagagaaaagagaagaaaaGGGagaggcctgtttggggcgatagcagggtttatagagggaggatggcagggaatggttgatggttggtatgggtaccatcatagcaatgagcaggggagtgggtacgctgcagacaaagaatccacccaaaaggcaatagatggagttaccaataaggtcaactcaatcattgacaaaatgaacactcaatttgaggcagttggaagggagtttaataacttagaaaggaggatagagaatttgaacaagaaaatggaagacggattcctagatgtctggacctataatgctgaacttctagttctcatggaaaacgagaggactctagatttccatgattcaaatgtcaagaacctttacgacaaagtcagactacagcttagggataatgcaaaggagctgggtaatggctgtttcgaattctatcacaaatgcgataatgaatgtatggaaagtgtgagaaatgggacgtatgactaccctcagtattcagaagaagcaagattaaaaagagaagaaataagcggagtgaaattagaatcaataggaacttaccagatactgtcaatttattcaacagcggcgagttccctagcactggcaatcatgatagctggtctatctttatggatgtgctccaatgggtcgttacagtgcagaatttgcatttag

>H5N1_A/Eurasian_eagle-owl/Finland/10617_21VIR7689-15/2021

cttgttaaaagtgatcagatttgcattggttaccatgcaaacaattcgacagagcaagttgacacgataatggaaaagaacgtcactgttacacatgcccaagacatactggaaaaaacacacaacgggaagctctgtgatctaaatggggtgaagcctctgattttaaaggattgtagtgtagctggatggctcctcggaaacccaatgtgcgacgaattcatcagagtgccggaatggtcctacatagtggagcgggctaatccagctaatgacctctgttacccagggagcctcaatgactatgaagaactgaaacacctgttgagcagaataaatcattttgagaagattctgatcatccccaagagttcctggccaaatcatgaaacatcactaggggtgagcgcagcttgtccataccagggagcgccctcctttttcagaaatgtggtgtggcttatcaraaagaacgatgcatacccaacaataaagataagctacaataataccaatcgggaagatctcttgatactgtgggggattcatcattccaacaatgcagaagagcagacaaatctctacaaaaacccaaccacctacatttcagttggaacatcaactttaaaccagaggttggtaccaaaaatagctactagatcccaagtaaacgggcaacgtggaagaatggacttcttctggacaattttaaaaccagatgatgcaatccatttcgagagtaatggaaatttcattgctccagaatatgcatacaaaattgtcaagaaaggggactcaacaattatgaaaagtggagtggaatatggccactgcaacaccaaatgtcaaaccccagtaggagcgataaattctagtatgccattccacaacatacatcctctcaccattggggaatgccccaaatacgtgaagtcaaacaagttggtccttgcgactgggcttagaaatagtcctctaagagaaaagagaagaaaaGGGagaggcctgtttggggcgatagcagggtttatagagggaggatggcagggaatggttgatggttggtatgggtaccatcatagcaatgagcaggggagtgggtacgctgcagacaaagaatccacccaaaaggcaatagatggagttaccaataaggtcaactcaatcattgacaaaatgaacactcaatttgaggcagttggaagggagtttaataacttagaaaggaggatagagaatttgaacaagaaaatggaagacggattcctagatgtctggacctataatgctgaacttctagttctcatggaaaacgagaggactctagatttccatgattcaaatgtcaagaacctttacgacaaagtcagactacagcttagggataatgcaaaggagctgggtaacggctgtttcgaattctatcacaaatgcgataatgaatgtatggaaagtgtgagaaatgggacgtatgactaccctcagtattcagaagaagcaagattaaaaagagaagaaataagcggagtgaaattagaatcaataggaacttaccagatactgtcaatttattcaacagcggcaagttccctagcactggcaatcatgatggctggtctatctttatggatgtgctccaatgggtcgttacagtgcagaatttgcatttag

>H5N1_A/Eurasian_teal/Denmark/24115-2/2021-10-16

cttgttaaaagtgatcagatttgcattggttaccatgcaaacaattcgacagagaaggttgacacgataatggaaaagaacgtcactgttacacatgcccaagacatactggaaaaaacacacaacgggaagctctgtgatttaaatggggtgaagcctctgattttaaaggattgtagtgtagctggatggctcctcggaaacccaatgtgcgacgaattcatcagagtgccggaatggtcctacatagtggagcgggctaatccagctaatgacctctgttacccagggagcctcaatgactatgaagaactgaaacacctgttgagcagaataaaccattttgagaagattctgatcatccccaagagttcctggccaaatcatgaaacatcactaggggtgagcgcagcttgtccataccagggagcgccctcctttttcagaaatgtggtgtggcttatcaaaaagaacgatgcatacccaacaataaagataagctacaataataccaatcgggaagatctcttgatactgtgggggattcatcattccaacaatgcagaagaacagacaaatctctataaaaacccaaccacctacatttcagttggaacatcaactttaaaccagaggttggtaccaaaaatagctactagatcccaagtaaacgggcaacgtggaagaatggacttcttctggacaattttaaaaccagatgatgcaatccatttcgagagtaatggaaatttcattgctccagaatatgcatataaaattgtcaagaaaggggactcaacaattatgaaaagtggagtggaatatggccactgcaacaccaaatgtcaaaccccagtaggagcgataaattctagtatgccattccacaacatacatcctctcaccattggggaatgccccaaatacgtgaagtcaaacaagttggtccttgcgactgggctcagaaatagtcctctaagagaaaagagaagaaaaGGGagaggcctgtttggggcgatagcagggtttatagagggaggatggcagggaatggttgatggttggtatgggtaccatcatagcaatgagcaggggagtgggtacgctgcagacaaagaatccacccaaaaggcaatagatggagttaccaataaggtcaactcaatcattgacaaaatgaacactcaatttgaggcagttggaagggagtttaataacttagaaaggaggatagagaatttgaacaagaaaatggaagacggattcctagatgtctggacctataatgctgaacttctagttctcatggaaaacgagaggactctagatttccatgattcaaatgtcaagaacctttacgacaaagtcagactacagcttagggataatgcaaaggagctgggtaatggctgtttcgaattctatcacaaatgcgataatgaatgtatggaaagtgtgagaaatgggacgtatgactaccctcagtattcagaagaagcaagattaaaaagagaagaaataagcggagtgaaattagaatcaataggaacttaccagatactgtcaatttattcaacagcggcgagttccctagcactggcaatcatgatagctggtctatctttatggatgtgctccaatgggtcgttacagtgcagaatttgcatttag

>H5N1_A/Eurasian_wigeon/Denmark/24279-1/2021-10-24

cttgttaaaagtgatcagatttgcattggttaccatgcaaacaattcgacagagaaggttgacacgataatggaaaagaacgtcactgttacacatgcccaagacatactggaaaaaacacacaacgggaagctctgtgatttaaatggggtgaagcctctgattttaaaggattgtagtgtagctggatggctcctcggaaacccaatgtgcgacgaattcatcagagtgccggaatggtcctacatagtggagcgggctaatccagctaatgacctctgttacccagggagcctcaatgactatgaagaactgaaacacctgttgagcagaataaaccattttgagaagattctgatcatccccaagagttcctggccaaatcatgaaacatcactaggggtgagcgcagcttgtccataccagggagcgccctcctttttcagaaatgtggtgtggcttatcaaaaagaacgatgcatacccaacaataaagataagctacaataataccaatcgggaagatctcttgatactgtgggggattcatcattccaacaatgcagaagaacagacaaatctctataaaaacccaaccacctacatttcagttggaacatcaactttaaaccagaggttggtaccaaaaatatctactagatcccaagtaaacgggcaacgtggaagaatggacttcttctggacaattttaaaaccagatgatgcaatccatttcgagagtaatggaaatttcattgctccagaatatgcatataaaattgtcaagaaaggggactcaacaattatgaaaagtggagtggaatatggccactgcaacaccaaatgtcaaaccccagtaggagcgataaattctagtatgccattccacaacatacatcctctcaccattggggaatgccccaaatacgtgaagtcaaacaagttggtccttgcgactgggctcagaaatagtcctctaagagaaaagagaagaaaaGGGagaggcctgtttggggcgatagcagggtttatagagggaggatggcagggaatggttgatggttggtatgggtaccatcatagcaatgagcaggggagtgggtacgctgcagacaaagaatccacccaaaaggcaatagatggagttaccaataaggtcaactcaatcattgacaaaatgaacactcaatttgaggcagttggaagggagtttaataacttagaaaggaggatagagaatttgaacaagaaaatggaagacggattcctagatgtctggacctataatgctgaacttctagttctcatggaaaacgagaggactctagatttccatgattcaaatgtcaagaacctttacgacaaagtcagactacagcttagggataatgcaaaggagctgggtaatggctgtttcgaattctatcacaaatgcgataatgaatgtatggaaagtgtgagaaatgggacgtatgactaccctcagtattcagaagaagcaagattaaaaagagaagaaataagcggagtgaaattagaatcaataggaacttaccagatactgtcaatttattcaacagcggcgagttccctagcactggcaatcatgatagctggtctatctttatggatgtgctccaatgggtcgttacagtgcagaatttgcatttag

>H5N1_A/Eurasian_wigeon/Germany-SH/AI05948/2021

cttgttaaaagtgatcagatttgcattggttaccatgcaaacaattcgacagagcaagttgacacgataatggaaaagaacgtaactgttacacatgcccaagacatactggaaaaaacacacaacgggaagctctgtgatctaaatggggtgaagcctctgattttaaaggattgtagtgtagctggatggctcctcggaaacccaatgtgcgacgaattcatcagagtgccggaatggtcctacatagtggagcgggctaatccagctaatgacctctgttacccagggagcctcaatgactatgaagaactgaaacacctgttgagcagaataaatcattttgagaagattctgatcatccccaagagttcctggccaaatcatgaaacatcactaggggtgagcgcagcttgtccataccagggagcgccctcctttttcagaaatgtggtgtggcttatcaaaaagaacgatgcatacccaacaataaagataagctacaataataccaatcgggaagatctcttgatactgtgggggattcatcattccaacaatgcagaagagcagacaaatctctacaaaaacccaaccacctacatttcagttggaacatcaactttaaaccagaggttggtaccaaaaatagctactagatcccaagtaaacgggcaacgtggacgaatggacttcttctggacaattttaaaaccagatgatgcaatccatttcgagagtaatggaaatttcattgctccagaatatgcatacaaaattatcaagaaaggggactcaacaattatgaaaagtggagtggaatatggccactgcaacaccaaatgtcaaaccccagtaggagcgataaattctagtatgccattccacaacatacatcctctcaccattggggaatgccccaaatacgtgaagtcaaacaagttggtccttgcgactgggcttagaaatagtcctctaagagaaaagagaagaaaaGGGagaggcctgtttggggcgatagcagggtttatagagggaggatggcagggaatggttgatggttggtatgggtaccatcatagcaatgagcaggggagtgggtacgctgcagacaaagaatccacccaaaaggcaatagatggagttaccaataaggtcaactcaatcattgacaaaatgaacactcaatttgaggcagttggaagggagtttaataacttagaaaggaggatagagaatttgaacaagaaaatggaagacggattcctagatgtctggacctataatgctgaacttctagttctcatggaaaacgagaggactctagatttccatgattcaaatgtcaagaacctttacgacaaagtcagactacagcttagggataatgcaaaggagctgggtaacggctgtttcgaattctatcacaaatgcgataatgaatgtatggaaagtgtgagaaatgggacgtatgactaccctcagtattcagaagaagcaagattaaaaagagaagaaataagcggagtgaaattagaatcaataggaacttaccagatactgtcaatttattcaacagcggcaagttccctagcactggcaatcatgatggctggtctatctttatggatgtgctccaatgggtcgttacagtgcagaatttgcatttag

>H5N1_A/Eurasian_wigeon/Italy/20VIR7301-206/2020

cttgttaaaagtgatcagatttgcattggttaccatgcaaacaattcgacagagcaagttgacacgataatggaaaagaacgtcactgttacacatgcccaagacatactggaaaaaacacacaacgggaagctctgtgatctaaatggggtgaagcctctgattttaaaggattgtagtgtagctggatggctcctcggaaacccaatgtgcgacgaattcatcagagtgccggaatggtcctacatagtggagcgggctaatccagctaatgacctctgttacccagggagcctcaatgactatgaagaactgaaacacctgttgagcagaataaatcattttgagaagattctgatcatccccaagagttcctggccaaatcatgaaacatcactaggggtgagcgcagcttgtccataccagggagcgccctcctttttcagaaatgtggtgtggcttatcaaaaagaacgatgcatacccaacaataaagataagctacaataataccaatcgggaagatctcttgatactgtgggggattcatcattccaacaatgcagaagagcagacaaatctctataaaaacccaaccacctacatttcagttggaacatcaactttaaaccagaggttggtaccaaaaatagctactagatcccaagtaaacgggcaacgtggaagaatggacttcttctggacaattttaaaaccagatgatgcaatccatttcgagagtaatggaaatttcattgctccagaatatgcatacaaaattgtcaagaaaggggactcaacaattatgaaaagtggagtggaatatggccactgcaacaccaaatgtcaaaccccagtaggagcgataaattctagtatgccattccacaacatacatcctctcaccattggggaatgccccaaatacgtgaagtcaaacaagttggtccttgcgactgggctcagaaatagtcctctaagagaaaagagaagaaaaGGGagaggcctgtttggggcgatagcagggtttatagagggaggatggcagggaatggttgatggttggtatgggtaccatcatagcaatgagcaggggagtgggtacgctgcagacaaagaatccacccaaaaggcaatagatggagttaccaataaggtcaactcaatcattgacaaaatgaacactcaatttgaggcagttggaagggagtttaataacttagaaaggaggatagagaatttgaacaagaaaatggaagacggattcctagatgtctggacctataatgctgaacttctagttctcatggaaaacgagaggactctagatttccatgattcaaatgtcaagaacctttacgacaaagtcagactacagcttagggataatgcaaaggagctgggtaacggctgtttcgaattctatcacaaatgcgataatgaatgtatggaaagtgtgagaaatgggacgtatgactaccctcagtattcagaagaagcaagattaaaaagagaagaaataagcggagtgaaattagaatcaataggaacttaccagatactgtcaatttattcaacagcggcgagttccctagcactggcaatcatgatggctggtctatctttatggatgtgctccaatgggtcgttacagtgcagaatttgcatttag

>H5N1_A/Eurasian_wigeon/Italy/21VIR8919-3/2021

cttgttaaaagtgatcagatttgcattggttaccatgcaaacaattcgacagagcaggttgacacgataatggaaaagaacgtcaccgttacacatgcccaagacatactggaaaaaacacacaacgggaagctctgtgatctaaatggggtgaagcctctgattttaaaggattgtagtgtagctggatggctcctcggaaacccaatgtgcgacgaattcatcagagtgccggaatggtcctacatagtggagcgggctaatccagctaatgacctctgttacccagggagcctcaatgactacgaagaactgaaacacctgttgagcagaataaaccactttgagaagattctgatcatcccaaagagttcctggccaaatcatgaaacatcactaggggtgagcgcagcttgtccataccagggagcgccctcctttttcagaaatgtggtgtggcttatcaaaaagaacgatgcatacccaacaataaagataagctacaataataccaatcaggaagatctcttgatactgtgggggattcatcattccaacaatgcagaagagcagacaaatctctataaaaacccaaccacctacatttcagttggaacatcaactttaaaccagaggttggtaccaaaaatagctactagatcccaagtaaacgggcagcgtggaagaatggacttcttctggacaattttaaaaccagatgatgccatccatttcgagagtaatggaaatttcattgctccagaatatgcatacaaaattgtcaagaaaggggactcaacaattatgaaaagtggagtggaatatggccactgcaacaccaaatgtcaaaccccagtaggagcgataaattctagtatgccattccacaacatacatcctctcaccattggggaatgccccaaatacgtgaaatcaaacaagttggtccttgcgactgggctcagaaatagtcctctaagagaaaagagaagaaaaGGGagaggcctgtttggggcgatagcagggtttatagagggaggatggcagggaatggttgatggttggtatgggtaccatcatagcaatgagcaggggagtgggtacgctgcagacaaagaatccacccaaaaggcaatagatggagttaccaataaggtcaactcaatcattgacaaaatgaacactcaatttgaggcagttggaagggagtttaataacttagaaaggaggatagagaatttgaacaagaaaatggaagacggattcctagatgtctggacctataatgctgaacttctagttctcatggaaaacgagaggactctagatttccatgattcaaatgtcaagaacctttacgacaaagtcagactacagcttagggataatgcaaaggagctgggcaatggctgtttcgaattctatcacaaatgcgataatgaatgtatggaaagtgtgagaaatgggacgtatgactacccccagtattcagaagaagcaagattaaaaagagaagaaataagcggagtgaaattagaatcaataggaacttaccagatactgtcaatttattcaacagcggcgagttccctagcactggcaatcatgatggctggtctatctttatggatgtgctccaatgggtcgttacagtgcagaatttgcatttag

>H5N1_A/European bee-eater/Chokpak/7a/2007

cttgttaaaagtgatcagatttgcattggttaccatgcaaacaactcgacagagcaggttgacacaataatggaaaagaacgtcactgttacacacgcccaagacatactggaaaagacacacaacgggaagctctgcgatctagatggagtgaagcctctaattttaagagattgtagtgtagctggatggctcctcgggaacccaatgtgtgacgaattcctcaatgtgccggaatggtcttacatagtggagaagatcaatccagccaatgacctctgttacccagggaatttcaacgactatgaagaactgaaacacctattgagcagaataaaccattttgagaaaattcagatcatccccaagagttcttggtcagatcatgaagcctcatcaggggtgagctcagcatgtccataccagggaaggtcctccttttttagaaatgtggtatggcttattaaaaagaacaatacatacccaacaataaagagaagttacaataataccaaccaagaagatcttttggtactgtgggggattcaccatccaaatgatgcggcagagcagacaaggctctatcaaaacccaaccacctatatttccgttgggacatcaacactaaaccagagattggtaccaaaaatagctactagatccaaagtaaacgggcaaagtggaaggatggagttcttttggacaattttaaaaccgaatgatgcaataaactttgagagtaatggaaatttcattgctccagaaaatgcatacaaaattgtcaagaaaggggactcaacaattatgaaaagtgaattggaatatggtaactgcaacaccaagtgtcaaactccaataggggcgataaactctagtatgccattccacaacatccaccctctcaccatcggggaatgccccaaatatgttaaatcaaacagattagtccttgcgactgggctcagaaatagccctcaaggagagagaagaagaaaaaagagaggactatttggagctatagcaggttttatagagggaggatggcagggaatggtagatggttggtatgggtaccaccatagcaacgagcaggggagtgggtacgctgcagacaaagaatccactcaaaaggcaatggatggagtcaccaataaggtcaactcgatcattgacaaaatgaacactcagtttgaggccgttggaagggaatttaataacttagaaaggagaatagaaaatttaaacaagaagatggaagacggattcctagatgtctggacttataatgctgaacttctggttctcatggaaaatgagagaactctagactttcatgactcaaatgtcaagaacctttacgacaaggtccgactacagcttagggataatgcaaaggagcttggtaacggttgtttcgagttctatcacagatgtgataatgaatgtatggaaagtgtaagaaacggaacgtatgactacccgcagtattcagaagaagcaagattaaaaagagaggaaataagtggagtaaaattggaatcaataggaacttaccaaatactgtcaatttattcaacagtggcgagctccctagcactggcaatcatggtggctggtctatctttatggatgtgctccaatggatcgttacaatgcagaatttgcatctga

>H5N1_A/European_Herring_Gull/Netherlands/2/2022

ctagttaaaagtgatcagatttgcattggttaccatgcaaacaattcgacagagcaggttgacacgataatggaaaagaacgtcactgttacacatgcccaagacatactggaaaaaacacacaacgggaagctctgtgatttaaatggggtgaagcctctgattttaaaggattgtagtgtagctggatggctcctcggaaacccaatgtgcgacgaattcatcagagtgccggaatggtcctacatagtggagcgggctaatccagctaatgacctctgttacccagggagcctcaatgactatgaagaactgaaacacctgttgagcagaataaatcattttgagaagattcttatcatccccaagagttcctggccaaatcatgaaacatcactaggggtgagcgcagcttgtccataccagggagcgccctcctttttcagaaatgtggtgtggcttatcaaaaagaacgatgcatacccaacaataaagataagctacaataataccaatcgggaagatctcttgatactgtgggggattcatcattccaacaatgcagaagaacagacaaatctctataaaaacccaaccacctacatttcagttggaacatcaactttaaaccagaggttggtaccaaaaatagctactagatcccaagtaaacgggcaacgtggaagaatggacttcttctggacaattttaaaaccagatgatgcaatccatttcgagagtaatggaaacttcattgctccagaatatgcatataaaattgtcaagaaaggggactcaacaattatgaaaagtggagtggaatatggccactgcaacaccaaatgtcaaaccccagtaggagcgataaattctagtatgccattccacaacatacatcctctcaccattggggaatgccccaaatacgtgaagtcaaacaagttggtccttgcgactgggctcagaaatagtcctctaagagaaaagagaagaaaaGGGagaggcctgtttggggcgatagcagggtttatagagggaggatggcagggaatggttgatggttggtatgggtaccatcatagcaatgagcaggggagtgggtacgctgcagacaaagaatccacccaaaaggcaatagatggagttaccaataaggtcaactcaatcattgacaaaatgaacactcaatttgaggcagttggaagggagtttaataacttagaaaggaggatagagaatttgaacaagaaaatggaagacggattcctagatgtctggacctataatgctgaacttctagttctcatggaaaacgagaggactctagatttccatgattcaaatgtcaagaacctttacgacaaagtcagactacagcttagggataatgcaaaggagctgggtaatggctgtttcgaattctatcacaaatgcgataatgaatgtatggaaagtgtgagaaatgggacgtatgactaccctcagtattcagaagaagcaagattaaaaagagaagaaataagcggagtgaaattagaatcaataggaacttaccagatactgtcaatttattcaacagcggcgagttccctagcactggcaatcatgatagctggtctatctttatggatgtgctccaatgggtcgttacagtgcagaatttgcatttag

>H5N1_A/European_herring_gull/Denmark/19968-1.02/2021-05-14

cttgttaaaagtgatcagatttgcattggttaccatgcaaacaattcgacagagcaagttgacacgataatggaaaagaacgtcactgttacacatgcccaagacatactggaaaaaacacacaacgggaagctctgtgatctaaatggggtgaagcctctgattttaaaggattgtagtgtagctggatggctcctcggaaacccaatgtgcgacgaattcatcagagtgccggaatggtcctacatagtggagcgggctaatccagctaatgacctctgttacccaggaagcctcaatgactatgaagaactgaaacacctgttgagcagaataaatcattttgagaagattctgatcatccccaagagttcctggccaaatcatgaaacatcactaggggtgagcgcagcttgtccataccagggagcgccctcctttttcagaaatgtggtgtggcttatcaaaaagaacgatgcatacccaacaataaagataagctacaataataccaatcgggaagatctcttgatactgtgggggattcatcattccaacaatgcagaagagcagacaaatctctacaaaaacccaaccacctacatttcagttggaacatcaactttaaaccagaggttggtaccaaaaatagctactagatcccaagtaaacgggcaacgtggaagaatggacttcttctggacaattttaaaaccagatgatgcaatccatttcgagagtaatggaaatttcattgctccagaatatgcatacaaaattgtcaagaaaggggactcaacaattatgaaaagtggagtggaatatggccactgcaacaccaaatgtcaaaccccagtaggagcgataaattctagtatgccattccacaacatacatcctctcaccattggggaatgccccaaatacgtgaagtcaaacaagttggtccttgcgactgggctcagaaatagtcctctaagagaaaagagaagaaaaGGGagaggcctgtttggggcgatagcagggtttatagagggaggatggcagggaatggttgatggttggtatgggtaccatcatagcaatgagcaggggagtgggtacgctgcagacaaagaatccacccaaaaggcaatagatggagttaccaataaggtcaactcaatcattgacaaaatgaacactcaatttgaggcagttggaagggagtttaataacttagaaaggaggatagagaatttgaacaagaaaatggaagacggattcctagatgtctggacatataatgctgaacttctagttctcatggaaaacgagaggactctagatttccatgattcaaatgtcaagaacctttacgacaaagtcagactacagcttagggataatgcaaaggagctgggtaacggctgtttcgaattctatcacaaatgcgataatgaatgtatggaaagtgtgagaaatgggacgtatgactaccctcagtattcagaagaagcaagattaaaaagagaagaaataagcggagtgaaattagaatcaataggaacttaccagatactgtcaatttattcaacagcggcaagttccctagcactggcaatcatgatggctggtctatctttatggatgtgctccaatgggtcgttacagtgcagaatttgcatttag

>H5N1_A/European_herring_gull/Finland/9722_21VIR7689-13/2021

cttgttaaaagtgatcagatttgcattggttaccatgcaaacaattcgacagagcaagttgacacgataatggaaaagaacgtcactgttacacatgcccaagacatactggaaaaaacacacaacgggaagctctgtgatctaaatggggtgaagcctctgattttaaaggattgtagtgtagctggatggctcctcggaaacccaatgtgcgacgaattcatcagagtgccggaatggtcctacatagtggagcgggctaatccagctaatgacctctgttacccagggagcctcaatgactatgaagaactgaaacacctgttgagcagaataaatcattttgagaagattctgatcatccccaagagttcctggccaaatcatgaaacatcactaggggtgagcgcagcttgtccataccagggagcgccctcctttttcagaaatgtggtgtggcttatcaaaaagaacgatgcatacccaacaataaagataagctacaataataccaatcgggaagatctcttgatactgtgggggattcatcattccaacaatgcagaagagcagacaaatctctacaaaaacccaaccacctacatttcagttggaacatcaactttaaaccagaggttggtaccaaaaatagctactagatcccaagtaaacgggcaacgtggaagaatggacttcttctggacaattttaaaaccagatgatgcaatccatttcgagagtaatggaaatttcattgctccagaatatgcatacaaaattgtcaagaaaggggactcaacaattatgaaaagtggagtggaatatggccactgcaacaccaaatgtcaaaccccagtaggagcgataaattctagtatgccattccacaacatacatcctctcaccattggggaatgccccaaatacgtgaagtcaaacaagttggtccttgcgactgggcttagaaatagtcctctaagagaaaagagaagaaaaGGGagaggcctgtttggggcgatagcagggtttatagagggaggatggcagggaatggttgatggttggtatgggtaccatcatagcaatgagcaggggagtgggtacgctgcagacaaagaatccacccaaaaggcaatagatggagttaccaataaggtcaactcaatcattgacaaaatgaacactcaatttgaggcagttggaagggagtttaataacttagaaaggaggatagagaatttgaacaagaaaatggaagacggattcctagatgtctggacctataatgctgaacttctagttctcatggaaaacgagaggactctagatttccatgattcaaatgtcaagaacctttacgacaaagtcagactacagcttagggataatgcaaaggagctgggtaacggctgtttcgaattctatcacaaatgcgataatgaatgtatggaaagtgtgagaaatgggacgtatgactaccctcagtattcagaagaagcaagattaaaaagagaagaaataagcggagtgaaattagaatcaataggaacttaccagatactgtcaatttattcaacagcggcaagttccctagcactggcaatcatgatggctggtctatctttatggatgtgctccaatgggtcgttacagtgcagaatttgcatttag

>H5N1_A/European_herring_gull/Sweden/SVA211116SZ0432/FB004518/M-2021

cttgttaaaagtgatcagatttgcattggttaccatgcaaacaattcgacagagaaggttgacacgataatggaaaagaacgtcactgttacacatgcccaagacatactggaaaaaacacacaacgggaagctctgtgatttaaatggggtgaagcctctgattttaaaggattgtagtgtagctggatggctcctcggaaacccaatgtgcgacgaattcatcagagtgccggaatggtcctacatagtggagcgggctaatccagctaatgacctctgttacccagggagcctcaatgactatgaagaactgaaacacctgttgagcagaataaaccattttgagaagattctgatcatccccaagagttcctggccaaatcatgaaacatcactaggggtgagcgcagcttgtccataccagggagcgccctcctttttcagaaatgtggtgtggcttatcaaaaagaacgatgcatacccaacaataaagataagctacaataataccaatcgggaagatctcttgatactgtgggggattcatcattccaacaatgcagaagaacagacaaatctctataaaaacccaaccacctacatttcagttggaacatcaactttaaaccagaggttggtaccaaaaatagctactagatcccaagtaaacgggcaacgtggaagaatggacttcttctggacaattttaaaaccagatgatgcaatccatttcgagagtaatggaaatttcattgctccagaatatgcatataaaattgtcaagaaaggggactcaacaattatgaaaagtggagtggaatatggccactgcaacaccaaatgtcaaaccccagtaggagcgataaattctagtatgccattccacaacatacatcctctcaccattggggaatgccccaaatacgtgaagtcaaacaagttggtccttgcgactgggctcagaaatagtcctctaagagaaaagagaagaaaaGGGagaggcctgtttggggcgatagcagggtttatagagggaggatggcagggaatggttgatggttggtatgggtaccatcatagcaatgagcaggggagtgggtacgctgcagacaaagaatccacccaaaaggcaatagatggagttaccaataaggtcaactcaatcattgacaaaatgaacactcaatttgaggcagttggaagggagtttaataacttagaaaggaggatagagaatttgaacaagaaaatggaagacggattcctagatgtctggacctataatgctgaacttctagttctcatggaaaacgagaggaccctagatttccatgattcaaatgtcaagaacctttacgacaaagtcagactacagcttagggataatgcaaaggagctgggtaatggctgtttcgaattctatcacaaatgcgataatgaatgtatggaaagtgtgagaaatgggacgtatgactaccctcagtattcagaagaagcaagattaaaaagagaagaaataagcggagtgaaattagaatcaataggaacttaccagatactgtcaatttattcaacagcggcgagttccctagcactggcaatcatgatagctggtctatctttatggatgtgctccaatgggtcgttacagtgcagaatttgcatttag

>H5N1_A/Gallus_gallus/Belgium/3194_0001/2022

cttgttaaaagtgatcagatttgcattggttaccatgcaaacaattcgacagagcaggttgacacgataatggaaaagaacgtcactgttacacatgcccaagacatactggaaaaaacacacaacgggaagctctgtgatttaaatggggtgaagcctctgattttaaaggattgtagtgtagctggatggctcctcggaaacccaatgtgcgacgaattcatcagagtgccggaatggtcctacatagtggagcgggctaatccagctaatgacctctgttacccagggagcctcaatgactatgaagaactgaaacacctgttgagcagaataaatcattttgagaagattcttatcatccccaagagttcctggccaaatcatgaaacatcactaggggtgagcgcagcttgtccataccagggagcgccctcctttttcagaaatgtggtgtggcttatcaaaaagaacgatgcatatccaacaataaagataagctacaataataccaatcgggaagatctcttgatactgtgggggattcatcattccaacaatgcagaagaacagacaaatctctataaaaatccaaccacctacatttcagttggaacatcaactttaaaccagaggttggtaccaaaaatagctactagatcccaagtaaacgggcaacgtggaagaatggacttcttctggacaattttaaaaccagatgatgcaatccatttcgagagtaatggaaatttcattgctccagaatatgcatataaaattgtcaagaaaggggactcaacaattatgaaaagtggagtggaatatggccactgcaacaccaaatgtcaaaccccagtaggagcgataaattctagtatgccattccacaacatacatcctctcaccattggggaatgccccaaatacgtgaagtcaaacaagttggtccttgcgactgggctcagaaatagtcctctaagagaaaagagaagaaaaGGGagaggcctgtttggggcgatagcagggtttatagagggaggatggcagggaatggttgatggttggtatgggtaccatcatagcaatgagcaggggagtgggtacgctgcagacaaagaatccacccaaaaggcaatagatggagttaccaataaggtcaactcaatcattgacaaaatgaacactcaatttgaggcagttggaagggagtttaataacttagaaaggaggatagagaatttgaacaagaaaatggaagacggattcctagatgtctggacctataatgctgaacttctagttctcatggaaaacgagaggactctagatttccatgattcaaatgttaagaacctttacgacaaagtcagaatacagcttagggataatgcaaaggagctgggtaatggctgtttcgaattctatcacaaatgcgataatgaatgtatggaaagtgtgagaaatgggacgtatgactaccctcagtattcagaagaagcaagattaaagagagaagaaataagcggagtgaaattagaatcaataggaacttaccagatactgtcaatttattcaacagcggcgagttccctagcactggcaatcatgatagctggtctatctttatggatgtgctccaatgggtcgttacagtgcagaatttgcatttag

>H5N1_A/Graylag_goose/Netherlands/2/2022

cttgttaaaagtgatcagatttgcattggttaccatgcaaacaattcgacagagcaggttgacacgataatggaaaagaacgtcactgttacacatgcccaagacatactggaaaaaacacacaacgggaagctctgtgatttaaatggggtgaagcctctgattttaaaggattgtagtgtagctggatggctcctcggaaacccaatgtgcgacgaattcatcagagtgccggaatggtcctacatagtggagcgggctaatccagctaatgacctctgttacccagggagcctcaatgactatgaagaactgaaacacctgttgagcagaataaatcattttgagaagattcttatcatccccaagagttcctggccaaatcatgaaacatcactaggggtgagcgcagcttgtccataccagggagcgccctcctttttcagaaatgtggtgtggcttatcaaaaagaacgatgcatacccaacaataaagataagctacaataataccaatcgggaagatctcttgatactgtgggggattcatcattccaacaatgcagaagaacagacaaatctctataaaaacccaaccacctacatttcagttggaacatcaactttaaaccagaggttggtaccaaaaatagctactagatcccaagtaaacgggcaacgtggaagaatggacttcttctggacaattttaaaaccagatgatgcaatccatttcgagagtaatggaaatttcattgctccagaatatgcatataaaattgtcaagaaaggggactcaacaattatgaaaagtggagtggaatatggccactgcaacaccaaatgtcaaaccccagtaggagcgataaattctagtatgccattccacaacatacatcctctcaccattggggaatgccccaaatacgtgaagtcaaacaagttggtccttgcgactgggctcagaaatagtcctctaagagaaaagagaagaaaaGGGagaggcctgtttggggcgatagcagggtttatagagggaggatggcagggaatggttgatggttggtatgggtaccatcatagcaatgagcaggggagtgggtacgctgcagacaaagaatccacccaaaaggcaatagatggagttaccaataaggtcaactcaatcattgacaaaatgaacactcaatttgaggcagttggaagggagtttaataacttagaaaggaggatagagaatttgaacaagaaaatggaagacggattcctagatgtctggacctataatgctgaacttctagttctcatggaaaacgagaggactctagatttccatgattcaaatgtcaagaacctttacgacaaagtcagactacagcttagggataatgcaaaggagctgggtaatggctgtttcgaattctatcacaaatgcgataatgaatgtatggaaagtgtgagaaatgggacgtatgactaccctcagtattcagaagaagcaagattaaaaagagaagaaataagcggagtgaaattagaatcaataggaacttaccagatactgtcaatttattcaacagcggcaagttccctagcactggcaatcatgatagctggtctatctttatggatgtgctccaatgggtcgttacagtgcagaatttgcatttag

>H5N1_A/Great_black-backed_Gull/Netherlands/2/2022

ctagttaaaagtgatcagatttgcattggttaccatgcaaacaattcgacagagcaggttgacacgataatggaaaagaacgtcactgttacacatgcccaagacatactggaaaaaacacacaacgggaagctctgtgatttaaatggggtgaagcctctgattttaaaggattgtagtgtagctggatggctcctcggaaacccaatgtgcgacgaattcatcagagtgccggaatggtcctacatagtggagcgggctaatccagctaatgacctctgttacccagggagcctcaatgactatgaagaactgaaacacctgttgagcagaataaatcattttgagaagattcttatcatccccaagagttcctggccaaatcatgaaacatcactaggggtgagcgcagcttgtccataccagggagcgccctcctttttcagaaatgtggtgtggcttatcaaaaagaacgatgcatacccaacaataaagataagctacaataataccaatcgggaagatctcttgatactgtgggggattcatcattccaacaatgcagaagaacagacaaatctctataaaaacccaaccacctacatttcagttggaacatcaactttaaaccagaggttggtaccaaaaatagctactagatcccaagtaaacgggcaacgtggaagaatggacttcttctggacaattttaaaaccagatgatgcaatccatttcgagagtaatggaaacttcattgctccagaatatgcatataaaattgtcaagaaaggggactcaacaattatgaaaagtggagtggaatatggccactgcaacaccaaatgtcaaaccccagtaggagcgataaattctagtatgccattccacaacatacatcctctcaccattggggaatgccccaaatacgtgaagtcaaacaagttggtccttgcgactgggctcagaaatagtcctctaagagaaaagagaagaaaaGGGagaggcctgtttggggcgatagcagggtttatagagggaggatggcagggaatggttgatggttggtatgggtaccatcatagcaatgagcaggggagtgggtacgctgcagacaaagaatccacccaaaaggcaatagatggagttaccaataaggtcaactcaatcattgacaaaatgaacactcaatttgaggcagttggaagggagtttaataacttagaaaggaggatagagaatttgaacaagaaaatggaagacggattcctagatgtctggacctataatgctgaacttctagttctcatggaaaacgagaggactctagatttccatgattcaaatgtcaagaacctttacgacaaagtcagactacagcttagggataatgcaaaggagctgggtaatggctgtttcgaattctatcacaaatgcgataatgaatgtatggaaagtgtgagaaatgggacgtatgactaccctcagtattcagaagaagcaagattaaaaagagaagaaataagcggagtgaaattagaatcaataggaacttaccagatactgtcaatttattcaacagcggcgagttccctagcactggcaatcatgatagctggtctatctttatggatgtgctccaatgggtcgttacagtgcagaatttgcatttag

>H5N1_A/Great_black-backed_gull/1/2022

cttgttaaaagtgatcagatttgcattggttaccatgcaaacaattcgacagagcaggttgacacgataatggaaaagaacgtcactgttacacatgcccaagacatactggaaaaaacacacaacgggaagctctgtgatttaaatggggtgaagcctctgattttaaaggattgtagtgtagctggatggctcctcggaaacccaatgtgcgacgaattcatcagagtgccggaatggtcctacatagtggagcgggctaatccagccaatgacctctgttacccagggagcctcaatgactatgaagaactgaaacacctgttgagcagaataaatcattttgagaagattctgatcatccccaatagttcctggccaaatcatgaaacatcactaggggtgagcgcagcttgtccataccagggagcgccctcctttttcagaaatgtgttgtggcttatcaaaaagaacgatgcatacccaacaataaagataagctacaataataccaatcgggaagatctcttgatactgtgggggattcatcattccaacaatgcagaagagcagacaaatctctataaaaacccaaccacctacatttcagttggaacatcaactttaaaccagaggttggtaccaaaaatagctactagatcccaagtaaacgggcaacgtggaagaatggacttcttctggacaattttaaaaccagatgatgcaatccatttcgagagtaatggaaatttcattgcaccagaatatgcatataaaattgtcaagaaaggggactcaacaattatgaaaagtggagtggaatatggccactgcaacaccaaatgtcaaaccccagtaggagcgataaattctagtatgccattccacaacatacatcctctcaccattggggaatgccccaaatacgtgaagtcaaacaagttggtccttgcgactgggctcagaaatagtcctctaagagaaaaaagaagaaaaGGGagaggcctgtttggggcgatagcagggtttatagagggaggatggcagggaatggttgatggttggtatggataccatcatagcaatgagcaggggagtgggtacgctgcagacaaagaatccacccaaaaggcaatagatggagttaccaataaggtcaactcaatcattgacaaaatgaacactcaatttgaggcagttggaagggagtttaataacttagaaaggaggatagagaatttgaacaagaaaatggaagacggattcctagatgtctggacctataatgctgaacttctagttctcatggaaaacgagaggactctagatttccatgattcaaatgtcaagaacctttacgacaaagtcagactacagcttagggacaatgcaaaggagctgggtaatggatgtttcgaattctatcacaaatgcgataatgaatgtatggaaagtgtgagaaatgggacgtatgactaccctcagtattcagaagaagcaagattaaaaagagaagaaataagcggagtgaaattagaatcaataggaacttaccagatactgtcaatttattcaacagcggcgagttccctagcactggcaatcatgatagctggtctatctttatggatgtgttccaatgggtcgttacagtgcagaatttgcatttag

>H5N1_A/Grey_heron/Netherlands/1/2022

cttgttaaaagtgatcagatttgcattggttaccatgcaaacaattcgacagagcaggttgacacgataatggaaaagaacgtcactgttacacatgcccaagacatactggaaaaaacacacaacgggaagctctgtgatttaaatggggtgaagcctctgattttaaaagattgtagtgtagctggatggctcctcggaaacccaatgtgcgacgaattcatcagagtgccggaatggtcctacatagtggagcgggctaatccagccaatgacctctgttacccagggagcctcaatgactatgaagaactgaaacacctgttgagcagaataaatcattttgagaagattctgatcatccccaagagttcctggccaaatcatgaaacatcactaggggtgagcgcagcttgtccataccagggagcgccctcctttttcagaaatgtggtgtggcttatcaaaaagaacgatgcatacccaacaataaagataagctacaataataccaatcgggaagatctcttgatactgtgggggattcatcattccaacaatgcagaagagcagacaaatctctataaaaacccaaccacctacatttcagttggaacatcaactttaaaccagaggttagtaccaaaaatagctactagatcccaagtaaacgggcaacgtggaagaatggacttcttctggacaattttaaaaccagatgatgcaatccacttcgaaagtaatggaaatttcattgcaccagaatatgcatataaaattgtcaagaaaggggactcaacaattatgaaaagtggagtggaatatggccactgcaacaccaaatgtcaaaccccagtaggagcgataaattctagtatgccattccacaacatacatcctctcaccattggggaatgccccaaatacgtgaagtcaaacaagttggtccttgcgactgggctcagaaatagtcctctaagagaaaagagaagaaaaGGGagaggcctgtttggggcgatagcagggtttatagagggaggatggcagggaatggttgatggttggtatgggtaccatcatagcaatgagcaggggagtgggtacgctgcagacaaagaatccacccaaaaggcaatagatggagttaccaataaggtcaactcaatcattgacaaaatgaacactcaatttgaggcagttggaagggagtttaataacttagaaaggaggatagagaatttgaacaagaaaatggaagacggattcctagatgtctggacctataatgctgaacttctagttctcatggaaaacgagaggactctagatttccatgattcaaatgtcaagaatctttacgacaaagtcagactacagcttagggataatgcaaaggagctgggtaatggctgtttcgaattctatcacaaatgcgataatgaatgtatggaaagtgtgagaaatggaacgtatgactaccctcagtattcagaagaagcaagattaaaaagagaagaaataagcggagtgaaattagagtcaataggaacttaccagatactgtcaatttattcaacagcggcgagttccctagcactggcaatcatgatagctggtctatctttatggatgtgctccaatgggtcgttacagtgcagaatttgcatttag

>H5N1_A/Greylag_goose/England/054503/2021

cttgttaaaagtgatcagatttgcattggttaccatgcaaacaattcgacagagcaggttgacacgataatggaaaagaacgtcactgttacacatgcccaagacatactggaaaaaacacacaacgggaagctctgtgatttaaatggggtgaagcctctgattttaaaggattgtagtgtagctggatggctcctcggaaacccaatgtgcgacgaattcatcagagtgccggaatggtcctacatagtggagcgggctaatccagccaatgacctctgttacccagggagcctcaatgactatgaagaactgaaacacctgttgagcagaataaatcattttgagaagattctgatcatccccaagagttcctggccaaatcatgaaacatcactaggggtgagcgcagcttgtccataccagggagcgccctcctttttcagaaatgtgttgtggcttatcaaaaagaacgatgcatacccaacaataaagataagctacaataataccaatcgggaagatctcttgatattgtgggggattcatcattccaacaatgcagaagagcagacaaatctctataaaaacccaatcacctacatttcagttggaacatcaactttaaaccagaggttggtaccaaaaatagctactagatcccaagtaaacgggcaacgtggaagaatggacttcttctggacaattttaaaaccagatgatgcaatccatttcgagagtaatggaaatttcattgcaccagaatatgcatataaaattgtcaagaaaggggactcaacaattatgaaaagtggagtggaatatggccactgcaacaccaaatgtcaaaccccagtaggagcgataaattctagtatgccattccacaacatacatcctctcaccattggggaatgccccaaatacgtgaagtcaaacaagttggtccttgcgactgggctcagaaatagtcctctaagagaaaagagaagaaaaGGGagaggcctgtttggggcgatagcagggtttatagagggaggatggcagggaatggttgatggttggtatgggtaccatcatagcaatgagcaggggagtgggtacgctgcagacaaagaatccacccaaaaggcaatagatggagttaccaataaggtcaactcaatcattgacaaaatgaacactcaatttgaggcagttggaagggagtttaataacttagagaggaggatagagaatttgaacaagaaaatggaagacggattcctagatgtctggacctataatgctgaacttctagttctcatggaaaacgagaggactctagatttccatgattcaaatgtcaagaacctttacgacaaagtcagactacagcttagggacaatgcaaaggagctgggtaatggctgtttcgaattctatcacaaatgcgataatgaatgtatggaaagtgtgagaaatgggacgtatgactaccctcagtattcagaagaagcaagattaaaaagagaagaaataagcggagtgaaattagaatcaataggaacttaccagatactgtcaatttattcaacagcggcgagttccctagcactggcaatcatgatagctggtctatctttatggatgtgctccaatgggtcgttacagtgcagaatttgcatttag

>H5N1_A/Herring_Gull/Netherlands/1/2021

cttgttaaaagtgatcagatttgcattggttaccatgcaaacaattcgacagagcaggttgacacgataatggaaaagaacgtcactgttacacatgcccaagacatactggaaaaaacacacaacgggaagctctgtgatttaaatggggtgaagcctctgattttaaaggattgtagtgtagctggatggctcctcggaaacccaatgtgcgacgaattcatcagagtgccggaatggtcctacatagtggagcgggctaatccagctaatgacctctgttacccagggagcctcaatgactatgaagaactgaaacacctgttaagcagaataaatcattttgagaagattcttatcatccccaagagttcctggccaaatcatgaaacatcactaggggtgagcgcagcttgtccataccagggagcgccctcctttttcagaaatgtggtgtggcttatcaaaaagaacgatgcatacccaacaataaagataagctacaataataccaatcgggaagatctcttgatactgtgggggattcatcattccaacaatgcagaagaacagacaaatctctataaaaacccaaccacctacatttcagttggaacatcaactttaaaccagaggttggtaccaaaaatagctactagatcccaagtaaacgggcaacgtggaagaatggacttcttctggacaattttaaaaccagatgatgcaatccatttcgagagtaatggaaatttcattgctccagaatatgcatataaaattgtcaagaaaggggactcaacaattatgaaaagtggagtggaatatggccactgcaacaccaaatgtcaaaccccagtaggagcgataaattctagtatgccattccacaacatacatcctctcaccattggggaatgccccaaatacgtgaagtcaaacaagttggtccttgcgactgggctcagaaatagtcctctaagagaaaagagaagaaaaGGGagaggcctgtttggggcgatagcagggtttatagagggaggatggcagggaatggttgatggttggtatgggtaccatcatagcaatgagcaggggagtgggtacgctgcagacaaagaatccacccaaaaggcaatagatggagttaccaataaggtcaactcaatcattgacaaaatgaacactcaatttgaggcagttggaagggagtttaataacttagaaaggaggatagagaatttgaacaagaaaatggaagacggattcctagatgtctggacctataatgctgaacttctagttctcatggaaaacgagaggactctagatttccatgattcaaatgtcaagaacctttacgacaaagtcagactacagcttagggataatgcaaaggagctgggtaatggctgtttcgaattctatcacaaatgcgataatgaatgtatggaaagtgtgagaaatgggacgtatgactaccctcagtattcagaagaagcaagattaaaaagagaagaaataagcggagtgaaattagaatcaataggaacttaccagatactgtcaatttattcaacagcggcgagttccctagcactggcaatcatgatagctggtctatctttatggatgtgctccaatgggtcgttacagtgcagaatttgcatttag

>H5N1_A/Japanese white-eye/Taoyuan/Q454/2012

cttgtcaaaagcgatcatatttgcataggttatcatgcaaataactcgacagagcaggttgacacaataatggaaaagaacgttactgttacacatgcccaagacatactggaaaagacacacaacgggaagctctgcgatctaaatggagtgaagcctctgattctaaaagattgtagtgtagcaggatggctcctcggaaatccattgtgtgacgaattcaccaatgtgccagaatggtcttacatagtagagaaggccaatccagccaatgacctctgttacccagggaatttcaacgattatgaagaattgaaacacctattgagcaggataaaccattttgagaaaatacagatcatccccaaagattcttggtcagatcatgaagcctcattgggggtgagcgcagcatgttcataccagggaaatccctccttcttcagaaatgtggtgtggcttatcaaaaaggacaatgcatacccaacaataaagaaaggctacaataataccaaccgagaagatctcttgatactgtgggggatccaccatcctaatgatgaggcagagcagacaaggctctatcaaaacccaactacctatatttccattgggacttcaacactaaaccagagattggtaccaaaaatagccactagatccaaaataaacgggcaaagtggcaggatagatttcttctggacaattttaaaaccgaatgacgcagtccacttcgagagtaatggaaatttcattgctccagaatatgcatacaaaattgtcaagaaaggagactccacaatcatgagaagtgaagtggaatatggtaattgcaacaccaggtgtcagactccaataggggcgataaactctagtatgccattccacaacatacaccctctcaccatcggagaatgtcccaaatatgtgaaatcaaacaaattagtccttgcaactgggctcagaaatagtcctcaaagagagagaagaagaaaaGGGagaggactgtttggagctatagcaggttttatagagggaggatggcagggaatggtagatggttggtatgggtaccaccacagcaatgaacaggggagtggttacgctgcagacaaagaatctactcaaaaggcgatagacggagtcaccaataaggtcaattcgatcattgacaaaatgaacactcagtttgaggctgtaggaagggaatttaataacttagagaggagaatagaaaatttaaacaagaagatggaagacggattcctagatgtctggacttataatgctgaacttctggttctcatggagaatgagagaactctagacttccatgactcaaatgtcaagaacctttacgataaggtccgactacagcttaaggataatgcaaaagagctgggaaacggttgttttgagttctatcacaaatgtaataatgaatgtatggaaagtgtaagaaacgggacgtatgactacccgcagtattcagaagaagcaagattaaaaagagaggaaataagtggagtaaaactggaatcaataggaatctaccaaatactgtcaatttattcaacagtggcgagttccctagtgctggcaatcatgatggctggtctatctttatggatgtgttccaacgggtcgttacagtgcagaatttgcatttga

>H5N1_A/Larus argentatus/Kostanay/7/2007

cttgttaaaagtgaccagatttgcattggttaccatgcaaacaactcgacagaacaggttgacacaataatggaaaagaatgttactgtcacacatgcccaagacatactagaaaaggcacacaacgggaagctctgcagcctaaatggagtgaagcctctcattctgagggattgtagtgtagctggatggcttctaggaaaccccatgtgtgacgaattcctcaatgtgccagaatggtcttacatagtggagaaggacaacccagtcaatggcctctgctacccaggggacttcaacgactatgaagaactaaaacacctattgagtagcacaaaccattttgagaaaattcaaatcatccccagaagttcttggtccaatcatgatgcatcatcaggggtgagctctgcatgcccatataatgggaggtcctccttttttcgaaatgtggtgtggcttatcaaaaagaacaatgcatacccaacaataaagaggagttacaataataccaatcaagaagatcttttggtgctgtgggggattcatcaccctaatgatgaagcagagcagacgaagctctatcaaaacccaaccacttacgtttcagttggaacatcaatactgaaccagagatcggtaccagaaatagctactagacccaaagtaaacgggcaaagtggaagaatggagttcttctggacaattttaaagccgaatgatgccatctattttgagagcaatgggaattttattgctccagaatatgcatacaaaattgtcaagaagggggattcagcaatcatgaaaagtggcttggaatacggtaactgcaacaccaagtgtcaaactccaatgggtgcgataaactctagcatgccattccacaacatacaccctctcaccattggggaatgccccaaatacgtgaaatcagatagattagtccttgcgactggactcaggaatgtccctcaaagagagacaGGGGGGGGGGGGagaggactatttggggctatagcaggctttatagaaggagggtggcaaggcatggtagatggttggtatggataccaccatagcaatgagcaggggagtgggtacgctgcagacaaggagtccactcagaaagcaatagatggaatcactaataaggtcaactcaatcattgacaaaatgaacactcagtttgaggccgttggaaaggaattcaacaacctggaaaggaggatagagaatctaaacaagaagatggaagacggatttctagatgtatggacttataatgttgaacttctggttctcatggaaaatgagagaactctagactttcatgattcaaatgtcaagaacctttatgacaaggttcgactacagcttagagataatgcaaaggaactgggtaatggttgtttcgagttctatcacaaatgtgataatgaatgtatggaaagtgtaagaaacggaacatacgattacccgcagtattcagaagaggcaagactgaatagagaggaaataagtggagtaaaattggaatcaatgggaacttaccaaatactgtcaatttattcaacagtggcgagttccctagcactggcaatcatgatagctggtctatctttctggatgtgctccaatgggtcattgcagtgcagaatttgcatctga

>H5N1_A/Mallard duck/New Zealand/08.272.73/2008

attgtcaaggccgaccaaatctgcattggttaccatgcgaacaattccacagaacaagttgatacgataatggagaagaatgtgacagtcacacatgctcagaacatacttgaaaaggaacacaatgggaaactctgcagtctcaaaggagtgaaacccctcattctaaaggattgcagtgtggctgggtggcttcttggaaatccgatgtgtgatgaattcctgagtgtaccggaatggtcatacatcgtggaaaaagataatccagcccatggcttgtgctatccaggagacttcaatgattatgaggaactgaaacacttaatgagcagcacaaaccattttgagaaaattcagataattcctagaagctcttggtccaaccatgatgcttcatcaggagtgagctcagcgtgcccatacaatgggagatcctcctttttcaggaatgtggtgtggttgatcaagaaaaataatgcgtatccaacaataaaaaggacctacaacaacactaatatagaagacctccttatattatggggaattcatcaccctaatgatgcagcagaacaaacacaactctaccagaactcaaatacttatgtatctgtgggaacatcaacactgaatcagaggttaattccagaaatagccaccaggcccaaagtaaacggacaaagtggaagaatggaatttttttggacaatactaaaaccgaatgatgcaattagctttgagagcaacgggaactttatagctcctgaatatgcatacaagattgtcaaaaggggagattcagcaatcatgaaaagtgaactggaatatggtgattgtgacaccaaatgtcagactccagtgggtgctataaattccagcatgcctttccacaatgttaatccccttactatcggggagtgccccaaatatgtcaagtcggacaggctaattcttgcaacagggctaagaaacgtacctcagagagaagcgGGGGGGGGGGGGaggggtctatttggagcaatagcaggattcatagaaggagggtggcaaggaatggtggatggatggtatggataccatcatagcaatgaacaagggagtggatatgctgcagacagagaatctacccagaaagcaatagatggaatcaccaataaggtgaactcaatcattgacaaaatgaacactcaatttgaggctgttgggaaagaattcaataacctagaaagaagaatagaaaatttaaataagaaaatggaggacggatttttagatgtatggacttacaacgcagaactcctggtactaatggaaaaagaaagaaccctggatttccatgattcaaatgtcaagaacctatatgataaagtcagactccagctgagagacaatgcaaaagaattaggcaatgggtgttttgagttttatcacaagtgtgacaatgagtgcatggaaagtgtgagaaatggaacatatgactacccaaaatactcagaagaatcaagactgaacagggaagagataggcggggtcaaattggaatcattgggtacttatcaaatactatcaatctattcaacagtggcgagttcactagcactggcaatcatgatagctggtctatttttttggatgtgctcaaatggatcattgcagtgcagaatttgcatctga

>H5N1_A/Mallard/Netherlands/1/2022

cttgttaaaagtgatcagatttgcattggttaccatgcaaacaattcgacagagcaggttgacacgataatggaaaagaacgtcactgttacacatgcccaagacatactggaaaaaacacacaacgggaagctctgtgatttaaatggggtgaagcctctgattttaaaagattgtagtgtagctggatggctcctcggaaacccaatgtgcgacgaattcatcagagtaccggaatggtcctacatagtggagcgggctaatccagccaatgacctctgttacccagggagcctcaatgactatgaggaactgaaacacctgttgagcagaataaatcattttgagaagattctgatcatccccaagagttcctggccaaatcatgaaacatcactaggggtgagcgcagcttgtccataccagggagcgccctcctttttcagaaatgtggtgtggcttatcaaaaagaacgatgcatacccaacaataaagataagctacaataataccaatcgggaagacctcttgatactgtgggggattcatcattccaacaatgcagaagagcagacaaatctctataaaaacccaaccacctacatttcagttggaacatcaactttaaaccagaggttagtaccaaaaatagctactagatcccaagtaaacgggcaacgtggaagaatggacttcttctggacaattttaaaaccagatgatgcaatccatttcgagagtaatggaaatttcattgcaccagaatatgcatataaaattgtcaagaaaggggactcaacaattatgaaaagtggagtggaatatggccactgcaacaccaaatgtcaaaccccagtaggagcgataaattctagtatgccattccacaacatacatcctctcaccattggggaatgccccaaatacgtgaagtcaaacaagttggtccttgcgactgggctcagaaatagtcctctaagagaaaagagaagaaaaGGGagaggcctgtttggggcgatagcagggtttatagagggaggatggcagggaatggttgatggttggtatgggtaccatcatagcaatgagcaggggagtgggtacgctgcagacaaagaatccacccaaaaggcaatagatggagttaccaataaggtcaactcaatcattgacaaaatgaacactcaatttgaggcagttggaagggagtttaataacttagaaaggaggatagagaatttgaacaagaaaatggaagacggattcctagatgtctggacctataatgctgaacttctagttctcatggaaaacgagaggactctagatttccatgattcaaatgtcaagaacctttacgacaaagtcagactacagcttagggataatgcaaaggagctgggtaatggctgtttcgaattctatcacaaatgcgataatgaatgtatggaaagtgtgagaaatgggacgtatgactaccctcagtattcagaagaagcaagattaaaaagagaagaaataagcggagtgaaattagaatcaataggaacttaccagatactgtcaatttattcaacagcggcgagttccctagcactggcaatcatgatagctggtctatctttatggatgtgctccaatgggtcgttacagtgcagaatttgcatttag

>H5N1_A/Mallard/Netherlands/13/2021

cttgttaaaagtgatcagatttgcattggttaccatgcaaacaattcgacagagcaggttgacacgataatggaaaagaacgtcactgttacacatgcccaagacatactggaaaaaacacacaacgggaagctctgtgatttaaatggggtgaagcctctgattttaaaggattgtagtgtagctggatggctcctcggaaacccaatgtgcgacgaattcatcagagtgccggaatggtcctacatagtggagcgggctaatccagccaatgacctctgttacccagggagcctcaatgactatgaagaactgaaacacctgttgagcagaataaatcattttgagaagattctgatcatccccaagagttcctggccaaatcatgaaacatcactaggggtgagcgcagcttgtccataccagggagcgccctcctttttcagaaatgtggtgtggcttatcaaaaagaacgatgcatacccaacaataaagataagctacaataataccaatcgggaagatctcttgatactgtgggggattcatcattccaacaatgcagaagagcagacaaatctctacaaaaacccaaccacctacatttcagttggaacatcaactttaaaccagaggttggtaccaaaaatagctactagatcccaagtaaacgggcaacgtggaagaatggacttcttctggacaattttaaaaccagatgatgcaatccatttcgagagtaatggaaatttcattgcaccagaatatgcatataaaattgtcaagaaaggggactcaacaattatgaaaagtggagtggaatatggccactgcaacaccaaatgtcaaaccccagtaggagcgataaattctagtatgccattccacaacatacatcctctcaccattggggaatgccccaaatacgtgaagtcaaacaagttagtccttgcgactgggctcagaaatagtcctctaagagaaaagagaagaaaaGGGagaggcctgtttggggcgatagcagggtttatagagggaggatggcagggaatggttgatggttggtatgggtaccatcatagcaatgagcaggggagtgggtacgctgcagacaaagaatccacccaaaaggcaatagatggagttaccaataaggtcaactcaatcattgacaaaatgaacactcaatttgaggcagttggaagggagtttaataacttagaaaggaggatagagaatttgaacaagaagatggaagacggattcctagatgtctggacctataatgctgaacttctagttctcatggaaaacgagaggactctagatttccatgattcaaatgtcaagaacctttacgacaaagtcagactacagcttagggataatgcaaaagagttgggtaatggctgtttcgaattctatcacaaatgcgataatgaatgtatggaaagtgtgagaaatgggacgtatgactaccctcagtattcagaagaagcaagattaaaaagagaagaaataagcggagtgaaattagaatcaataggaacttaccagatactgtcaatttattcaacagcggcgagttccctagcactggcaatcatgatagctggtctatctttatggatgtgctccaatgggtcgttacagtgcagaatttgcatttag

>H5N1_A/Mallard/Netherlands/14/2021

cttgttaaaagtgatcagatttgcattggttaccatgcaaacaattcgacagagcaggttgacacgataatggaaaagaacgtcactgttacacatgcccaagacatactggaaaaaacacacaacgggaagctctgtgatttaaatggggtgaagcctctgattttaaaggattgtagtgtagctggatggctcctcggaaacccaatgtgcgacgaattcatcagagtgccggaatggtcctacatagtggagcgggctaatccagccaatgacctctgttacccagggagcctcaatgactatgaagaactgaaacacctgttgagcagaataaatcattttgagaagattctgatcatccccaagagttcctggccaaatcatgaaacatcactaggggtgagcgcagcttgtccataccagggagcgccctcctttttcagaaatgtggtgtggcttatcaaaaagaacgatgcatacccaacaataaagataagctacaataataccaatcgggaagatctcttgatactgtgggggattcatcattccaacaatgcagaagagcagacaaatctctacaaaaacccaaccacctacatttcagttggaacatcaactttaaaccagaggttggtaccaaaaatagctactagatcccaagtaaacgggcaacgtggaagaatggacttcttctggacaattttaaaaccagatgatgcaatccatttcgagagtaatggaaatttcattgcaccagaatatgcatataaaattgtcaagaaaggggactcaacaattatgaaaagtggagtggaatatggccactgcaacaccaaatgtcaaaccccagtaggagcgataaattctagtatgccattccacaacatacatcctctcaccattggggaatgccccaaatacgtgaagtcaaacaagttagtccttgcgactgggctcagaaatagtcctctaagagaaaagagaagaaaaGGGagaggcctgtttggggcgatagcagggtttatagagggaggatggcagggaatggttgatggttggtatgggtaccatcatagcaatgagcaggggagtgggtacgctgcagacaaagaatccacccaaaaggcaatagatggagttaccaataaggtcaactcaatcattgacaaaatgaacactcaatttgaggcagttggaagggagtttaataacttagaaaggaggatagagaatttgaacaagaagatggaagacggattcctagatgtctggacctataatgctgaacttctagttctcatggaaaacgagaggactctagatttccatgattcaaatgtcaagaacctttacgacaaagtcagactacagcttagggataatgcaaaagagttgggtaatggctgtttcgaattctatcacaaatgcgataatgaatgtatggaaagtgtgagaaatgggacgtatgactaccctcagtattcagaagaagcaagattaaaaagagaagaaataagcggagtgaaattagaatcaataggaacttaccagatactgtcaatttattcaacagcggcgagttccctagcactggcaatcatgatagctggtctatctttatggatgtgctccaatgggtcgttacagtgcagaatttgcatttag

>H5N1_A/Mallard/Netherlands/15/2021

cttgttaaaagtgatcagatttgcattggttaccatgcaaacaattcgacagagcaggttgacacgataatggaaaagaacgtcactgttacacatgcccaagacatactggaaaaaacacacaacgggaagctctgtgatttaaatggggtgaagcctctgattttaaaggattgtagtgtagctggatggctcctcggaaacccaatgtgcgacgaattcatcagagtgccggaatggtcctacatagtggagcgggctaatccagccaatgacctctgttacccagggagcctcaatgactatgaagaactgaaacacctgttgagcagaataaatcattttgagaagattctgatcatccccaagagttcctggccaaatcatgaaacatcactaggggtgagcgcagcttgtccataccagggagcgccctcctttttcagaaatgtggtgtggcttatcaaaaagaacgatgcatacccaacaataaagataagctacaataataccaatcgggaagatctcttgatactgtgggggattcatcattccaacaatgcagaagagcagacaaatctctacaaaaacccaaccacctacatttcagttggaacatcaactttaaaccagaggttggtaccaaaaatagctactagatcccaagtaaacgggcaacgtggaagaatggacttcttctggacaattttaaaaccagatgatgcaatccatttcgagagtaatggaaatttcattgcaccagaatatgcatataaaattgtcaagaaaggggactcaacaattatgaaaagtggagtggaatatggccactgcaacaccaaatgtcaaaccccagtaggagcgataaattctagtatgccattccacaacatacatcctctcaccattggggaatgccccaaatacgtgaagtcaaacaagttagtccttgcgactgggctcagaaatagtcctctaagagaaaagagaagaaaaGGGagaggcctgtttggggcgatagcagggtttatagagggaggatggcagggaatggttgatggttggtatgggtaccatcatagcaatgagcaggggagtgggtacgctgcagacaaagaatccacccaaaaggcaatagatggagttaccaataaggtcaactcaatcattgacaaaatgaacactcaatttgaggcagttggaagggagtttaataacttagaaaggaggatagagaatttgaacaagaagatggaagacggattcctagatgtctggacctataatgctgaacttctagttctcatggaaaacgagaggactctagatttccatgattcaaatgtcaagaacctttacgacaaagtcagactacagcttagggataatgcaaaagagttgggtaatggctgtttcgaattctatcacaaatgcgataatgaatgtatggaaagtgtgagaaatgggacgtatgactaccctcagtattcagaagaagcaagattaaaaagagaagaaataagcggagtgaaattagaatcaataggaacttaccagatactgtcaatttattcaacagcggcgagttccctagcactggcaatcatgatagctggtctatctttatggatgtgctccaatgggtcgttacagtgcagaatttgcatttag

>H5N1_A/Mallard/Netherlands/2/2022

cttgttaaaagtgatcagatttgcattggttaccatgcaaacaattcgacagagcaggttgacacgataatggaaaagaacgtcactgttacacatgcccaagacatactggaaaaaacacacaacgggaagctctgtgatttaaatggggtgaagcctctgattttaaaagattgtagtgtagctggatggctcctcggaaacccaatgtgcgacgaattcatcagagtaccggaatggtcctacatagtggagcgggctaatccagccaatgacctctgttacccagggagcctcaatgactatgaggaactgaaacacctgttgagcagaataaatcattttgagaagattctgatcatccccaagagttcctggccaaatcatgaaacatcactaggggtgagcgcagcttgtccataccagggagcgccctcctttttcagaaatgtggtgtggcttatcaaaaagaacgatgcatacccaacaataaagataagctacaataataccaatcgggaagacctcttgatactgtgggggattcatcattccaacaatgcagaagagcagacaaatctctataaaaacccaaccacctacatttcagttggaacatcaactttaaaccagaggttagtaccaaaaatagctactagatcccaagtaaacgggcaacgtggaagaatggacttcttctggacaattttaaaaccagatgatgcaatccatttcgagagtaatggaaatttcattgcaccagaatatgcatataaaattgtcaagaaaggggactcaacaattatgaaaagtggagtggaatatggccactgcaacaccaaatgtcaaaccccagtaggagcgataaattctagtatgccattccacaacatacatcctctcaccattggggaatgccccaaatacgtgaagtcaaacaagttggtccttgcgactgggctcagaaatagtcctctaagagaaaagagaagaaaaGGGagaggcctgtttggggcgatagcagggtttatagagggaggatggcagggaatggttgatggttggtatgggtaccatcatagcaatgagcaggggagtgggtacgctgcagacaaagaatccacccaaaaggcaatagatggagttaccaataaggtcaactcaatcattgacaaaatgaacactcaatttgaggcagttggaagggagtttaataacttagaaaggaggatagagaatttgaacaagaaaatggaagacggattcctagatgtctggacctataatgctgaacttctagttctcatggaaaacgagaggactctagatttccatgattcaaatgtcaagaacctttacgacaaagtcagactacagcttagggataatgcaaaggagctgggtaatggctgtttcgaattctatcacaaatgcgataatgaatgtatggaaagtgtgagaaatgggacgtatgactaccctcagtattcagaagaagcaagattaaaaagagaagaaataagcggagtgaaattagaatcaataggaacttaccagatactgtcaatttattcaacagcggcgagttccctagcactggcaatcatgatagctggtctatctttatggatgtgctccaatgggtcgttacagtgcagaatttgcatttag

>H5N1_A/Mallard/Netherlands/4/2021

cttgttaaaagtgatcagatttgcattggttaccatgcaaacaattcaacagagaaggttgacacgataatggaaaagaacgtcactgttacacatgcccaagacatactggaaaaaacacacaacgggaagctctgtgatttaaatggggtgaagcctctgattttaaaggattgtagtgtagctggatggctcctcggaaacccaatgtgcgacgaattcatcagagtgccggaatggtcctacatagtggagcgggctaatccagctaatgacctctgttacccagggagcctcaatgactatgaagaactgaaacacctgttgagcagaataaaccattttgagaagattctgatcatccccaagagttcctggccaaatcatgaaacatcactaggggtgagcgcagcttgtccataccagggagcgccctccttcttcagaaatgtggtgtggcttatcaaaaagaacgatgcatacccaacaataaagataagctacaataataccaatcgggaagatctcttgatactgtgggggattcatcattccaacaatgcagaagaacagacaaatctctataaaaacccaaccacctacatttcagttggaacatcaactttaaaccagaggttggtaccaaaaatagctactagatcccaagtaaacgggcaacgtggaagaatggacttcttctggacaattttaaaaccagatgatgcaatccatttcgagagtaatggaaatttcattgctccagaatatgcatataaaattgtcaagaaaggggactcaacaattatgaaaagtggagtggaatatggccactgcaacaccaaatgtcaaaccccagtaggagcgataaattctagtatgccattccacaacatacatcctctcaccattggggaatgccccaaatacgtgaagtcaaacaagttggtccttgcgactgggctcagaaatagtcctctaagagaaaagagaagaaaaGGGagaggcctgtttggggcgatagcagggtttatagagggaggatggcagggaatggttgatggttggtatgggtatcatcatagcaatgagcaggggagtgggtacgctgcagacaaagaatccacccaaaaggcaatagatggagttaccaataaggtcaactcaatcattgacaaaatgaacactcaatttgaggcagttggaagggagtttaataacttagaaaggaggatagagaatttgaacaagaaaatggaagacggattcctggatgtctggacctataatgctgaacttctagttctcatggaaaacgagaggactctagatttccatgattcaaatgtcaagaacctttacgacaaagtcagactacagcttagggataatgcaaaggagctgggtaatggctgtttcgaattctatcacaaatgcgataatgaatgtatggaaagtgtgagaaatgggacgtatgactaccctcagtattcagaagaagcaagattaaaaagagaagaaataagcggagtgaaattagaatcaataggaacttaccagatactgtcaatttattcaacagcggcgagttccctagcactggcaatcatgatagctggtctatctttatggatgtgctccaatgggtcgttacagtgcagaatttgcatttag

>H5N1_A/Mallard/Netherlands/5/2021

cttgttaaaagtgatcagatttgcattggttaccatgcaaacaattcgacagagaaggttgacacgataatggaaaagaacgtcactgttacacatgcccaagacatactggaaaaaacacacaacgggaagctctgtgatttaaatggggtgaagcctctgattttaaaggattgtagtgtagctggatggctcctcggaaacccaatgtgcgacgaattcatcagagtgccggaatggtcctacatagtggagcgggctaatccagctaatgacctctgttacccagggagcctcaatgactatgaagaactgaaacacctgttgagcagaataaaccattttgagaagattctgatcatccccaagagttcctggccaaatcatgaaacatcactaggggtgagcgcagcttgtccataccagggagcgccctccttcttcagaaatgtggtgtggcttatcaaaaagaacgatgcatacccaacaataaagataagctacaataataccaatcgggaagatctcttgatactgtgggggattcatcattccaacaatgcagaagaacagacaaatctctataaaaacccaaccacctacatttcagttggaacatcaactttaaaccagaggttggtaccaaaaatagctactagatcccaagtaaacgggcaacgtggaagaatggacttcttctggacaattttaaaaccagatgatgcaatccatttcgagagtaatggaaatttcattgctccagaatatgcatataaaattgtcaagaaaggggactcaacaattatgaaaagtggagtggaatatggccactgcaacaccaaatgtcaaaccccagtaggagcgataaattctagtatgccattccacaacatacatcctctcaccattggggaatgccccaaatacgtgaagtcaaacaagttggtccttgcgactgggctcagaaatagtcctctaagagaaaagagaagaaaaGGGagaggcctgtttggggcgatagcagggtttatagagggaggatggcagggaatggttgatggttggtatgggtatcatcatagcaatgagcaggggagtgggtacgctgcagacaaagaatccacccaaaaggcaatagatggagttaccaataaggtcaactcaatcattgacaaaatgaacactcaatttgaggcagttggaagggagtttaataacttagaaaggaggatagagaatttgaacaagaaaatggaagacggattcctggatgtctggacctataatgctgaacttctagttctcatggaaaacgagaggactctagatttccatgattcaaatgtcaagaacctttacgacaaagtcagactacagcttagggataatgcaaaggagctgggtaatggctgtttcgaattctatcacaaatgcgataatgaatgtatggaaagtgtgagaaatgggacgtatgactaccctcagtattcagaagaagcaagattaaaaagagaagaaataagcggagtgaaattagaatcaataggaacttaccagatactgtcaatttattcaacagcggcgagttccctagcactggcaatcatgatagctggtctatctttatggatgtgctccaatgggtcgttacagtgcagaatttgcatttag

>H5N1_A/Mallard/Netherlands/6/2021

cttgttaaaagtgatcagatttgcattggttaccatgcaaacaattcgacagagaaggttgacacgataatggaaaagaacgtcactgttacacatgcccaagacatactggaaaaaacacacaacgggaagctctgtgatttaaatggggtgaagcctctgattttaaaggattgtagtgtagctggatggctcctcggaaacccaatgtgcgacgaattcatcagagtgccggaatggtcctacatagtggagcgggctaatccagctaatgacctctgttacccagggagcctcaatgactatgaagaactgaaacacctgttgagcagaataaaccattttgagaagattctgatcatccccaagagttcctggccaaatcatgaaacatcactaggggtgagcgcagcttgtccataccagggagcgccctccttcttcagaaatgtggtgtggcttatcaaaaagaacgatgcatacccaacaataaagataagctacaataataccaatcgggaagatctcttgatactgtgggggattcatcattccaacaatgcagaagaacagacaaatctctataaaaacccaaccacctacatttcagttggaacatcaactttaaaccagaggttggtaccaaaaatagctactagatcccaagtaaacgggcaacgtggaagaatggacttcttctggacaattttaaaaccagatgatgcaatccatttcgagagtaatggaaatttcattgctccagaatatgcatataaaattgtcaagaaaggggactcaacaattatgaaaagtggagtggaatatggccactgcaacaccaaatgtcaaaccccagtaggagcgataaattctagtatgccattccacaacatacatcctctcaccattggggaatgccccaaatacgtgaagtcaaacaagttggtccttgcgactgggctcagaaatagtcctctaagagaaaagagaagaaaaGGGagaggcctgtttggggcgatagcagggtttatagagggaggatggcagggaatggttgatggttggtatgggtatcatcatagcaatgagcaggggagtgggtacgctgcagacaaagaatccacccaaaaggcaatagatggagttaccaataaggtcaactcaatcattgacaaaatgaacactcaatttgaggcagttggaagggagtttaataacttagaaaggaggatagagaatttgaacaagaaaatggaagacggattcctggatgtctggacctataatgctgaacttctagttctcatggaaaacgagaggactctagatttccatgattcaaatgtcaagaacctttacgacaaagtcagactacagcttagggataatgcaaaggagctgggtaatggctgtttcgaattctatcacaaatgcgataatgaatgtatggaaagtgtgagaaatgggacgtatgactaccctcagtattcagaagaagcaagattaaaaagagaagaaataagcggagtgaaattagaatcaataggaacttaccagatactgtcaatttattcaacagcggcgagttccctagcactggcaatcatgatagctggtctatctttatggatgtgctccaatgggtcgttacagtgcagaatttgcatttag

>H5N1_A/Mallard/Netherlands/7/2021

cttgttaaaagtgatcagatttgcattggttaccatgcaaacaattcaacagagaaggttgacacgataatggaaaagaacgtcactgttacacatgcccaagacatactggaaaaaacacacaacgggaagctctgtgatttaaatggggtgaagcctctgattttaaaggattgtagtgtagctggatggctcctcggaaacccaatgtgcgacgaattcatcagagtgccggaatggtcctacatagtggagcgggctaatccagctaatgacctctgttacccagggagcctcaatgactatgaagaactgaaacacctgttgagcagaataaaccattttgagaagattctgatcatccccaagagttcctggccaaatcatgaaacatcactaggggtgagcgcagcttgtccataccagggagcgccctccttcttcagaaatgtggtgtggcttatcaaaaagaacgatgcatacccaacaataaagataagctacaataataccaatcgggaagatctcttgatactgtgggggattcatcattccaacaatgcagaagaacagacaaatctctataaaaacccaaccacctacatttcagttggaacatcaactttaaaccagaggttggtaccaaaaatagctactagatcccaagtaaacgggcaacgtggaagaatggacttcttctggacaattttaaaaccagatgatgcaatccatttcgagagtaatggaaatttcattgctccagaatatgcatataaaattgtcaagaaaggggactcaacaattatgaaaagtggagtggaatatggccactgcaacaccaaatgtcaaaccccagtaggagcgataaattctagtatgccattccacaacatacatcctctcaccattggggaatgccccaaatacgtgaagtcaaacaagttggtccttgcgactgggctcagaaatagtcctctaagagaaaagagaagaaaaGGGagaggcctgtttggggcgatagcagggtttatagagggaggatggcagggaatggttgatggttggtatgggtatcatcatagcaatgagcaggggagtgggtacgctgcagacaaagaatccacccaaaaggcaatagatggagttaccaataaggtcaactcaatcattgacaaaatgaacactcaatttgaggcagttggaagggagtttaataacttagaaaggaggatagagaatttgaacaagaaaatggaagacggattcctggatgtctggacctataatgctgaacttctagttctcatggaaaacgagaggactctagatttccatgattcaaatgtcaagaacctttacgacaaagtcagactacagcttagggataatgcaaaggagctgggtaatggctgtttcgaattctatcacaaatgcgataatgaatgtatggaaagtgtgagaaatgggacgtatgactaccctcagtattcagaagaagcaagattaaaaagagaagaaataagcggagtgaaattagaatcaataggaacttaccagatactgtcaatttattcaacagcggcgagttccctagcactggcaatcatgatagctggtctatctttatggatgtgctccaatgggtcgttacagtgcagaatttgcatttag

>H5N1_A/Mallard/Netherlands/8/2021

cttgttaaaagtgatcagatttgcattggttaccatgcaaacaattcgacagagaaggttgacacgataatggaaaagaacgtcactgttacacatgcccaagacatactggaaaaaacacacaacgggaagctctgtgatttaaatggggtgaagcctctgattttaaaggattgtagtgtagctggatggctcctcggaaacccaatgtgcgacgaattcatcagagtgccggaatggtcctacatagtggagcgggctaatccagctaatgacctctgttacccagggagcctcaatgactatgaagaactgaaacacctgttgagcagaataaaccattttgagaagattctgatcatccccaagagttcctggccaaatcatgaaacatcactaggggtgagcgcagcttgtccataccagggagcgccctccttcttcagaaatgtggtgtggcttatcaaaaagaacgatgcatacccaacaataaagataagctacaataataccaatcgggaagatctcttgatactgtgggggattcatcattccaacaatgcagaagaacagacaaatctctataaaaacccaaccacctacatttcagttggaacatcaactttaaaccagaggttggtaccaaaaatagctactagatcccaagtaaacgggcaacgtggaagaatggacttcttctggacaattttaaaaccagatgatgcaatccatttcgagagtaatggaaatttcattgctccagaatatgcatataaaattgtcaagaaaggggactcaacaattatgaaaagtggagtggaatatggccactgcaacaccaaatgtcaaaccccagtaggagcgataaattctagtatgccattccacaacatacatcctctcaccattggggaatgccccaaatacgtgaagtcaaacaagttggtccttgcgactgggctcagaaatagtcctctaagagaaaagagaagaaaaGGGagaggcctgtttggggcgatagcagggtttatagagggaggatggcagggaatggttgatggttggtatgggtatcatcatagcaatgagcaggggagtgggtacgctgcagacaaagaatccacccaaaaggcaatagatggagttaccaataaggtcaactcaatcattgacaaaatgaacactcaatttgaggcagttggaagggagtttaataacttagaaaggaggatagagaatttgaacaagaaaatggaagacggattcctggatgtctggacctataatgctgaacttctagttctcatggaaaacgagaggactctagatttccatgattcaaatgtcaagaacctttacgacaaagtcagactacagcttagggataatgcaaaggagctgggtaatggctgtttcgaattctatcacaaatgcgataatgaatgtatggaaagtgtgagaaatgggacgtatgactaccctcagtattcagaagaagcaagattaaaaagagaagaaataagcggagtgaaattagaatcaataggaacttaccagatactgtcaatttattcaacagcggcgagttccctagcactggcaatcatgatagctggtctatctttatggatgtgctccaatgggtcgttacagtgcagaatttgcatttag

>H5N1_A/Muscovy duck/Ca Mau/07-04/2007

cttgttaaaagtgatcagatttgcattggttaccatgcaaacaactcgacagagcaggttgacacaataatggaaaagaacgttactgttacacatgcccaagacatactggaaaagacacataacgggaagctctgtgatctagatggagtgaggcctctaattttgagagattgtagtgtagctggatggcttctcggaaacccaatgtgtgacgaattcatcaatgtgccggaatggtcttacatagtggagaaggccaatccagtcaatgacctctgttacccaggagttttcaatgactatgaagaattgaaacacctattgagcagaataaaccattttgagaaaattcagatcatccccaaaagttcttggcccagtcatgaagcctcattgggggtgagcgcagcatgtccataccagggaaagtcctcttttttcagaaatgtggtatggcttatcaaaaagaacagtacatacccaacaataaagaggagttacaataataccaaccaagaagatcttttggtaatgtgggggatccaccatcctaatgatgcggcagagcagacaaagctctatcaaaatccaaccacctatatctccgttgggacatcaacactaaaccagagattgacaccaagaatagctactagatccaaagtaaacgggcaaagtgggaggatggagttcttctggacaattttaagaccgaatgatgcaatcaacttcgagagtaatggaaatttcattgctccagaatatgcatacaaaattgtcaagaaaggggactcaacaattatgaaaagtgaattggaatatggtaactgcaacaccaagtgtcaaactccaatgggggcgataaactctagtatgccattccacaatatacatcctctcactattggggaatgccccaaatatgtgaaatcaaacagattagtccttgcgactgggctcagaaatagccctcaaagagagggaagaagaaaaaagagaggattatttggagctatagcaggttttatagagggaggatggcagggaatggtagatggttggtatgggtaccaccatagcaatgagcaggggagtgggtacgctgcagacaaagaatccactcaaaaggctatagatggagtcaccaataaggtcaactcgatcattgacaaaatgaacactcagtttgaggccgttggaagggaatttaacaacttagaaagaagaatagagaatttaaacaagaagatggaagacgggttcctagatgtctggacttataatgctgaacttctggttctcatggaaaatgagagaactctagacttccatgactcaaatgtaaagaacctttacgacaaggtccgattacagcttagggataatgcaaaggagctgggtaacggttgtttcgagttctatcacaaatgtgataatgaatgtatggaaagtgtgagaaacgggacgtatgactacccgcagtattcagaagaagcaagattaaaaagagaggaaataagtggagtgaaattggaatcgataggaatttaccaaatactgtcaatttattctacagtggcgagttccctagcactggcaatcatggtagctggtctatccttatggatgtgctccaatgggtcgttacaatgcagaatttgcatttga

>H5N1_A/Muscovy duck/Ca Mau/1159/2006

cttgttaaaagtgatcagatttgcattggttaccatgcaaacaactcgacagagcaggttgacacaataatggaaaagaacgttactgttacacatgcccaagacatactggaaaagacacataacgggaagctctgtgatctagatggagtgaggcctctaattttgagagattgtagtgtagctggatggcttctcggaaacccaatgtgtgacgaattcatcaatgtgccggaatggtcttacatagtggagaaggccaatccagtcaatgacctctgttacccaggagttttcaatgactatgaagaattgaaacacctattgagcagaataaaccattttgagaaaattcagatcatccccaaaagttcttggcccagtcatgaagcctcattgggggtgagcgcagcatgtccataccagggaaagtcctcttttttcagaaatgtggtatggcttatcaaaaagaacagtacatacccaacaataaagaggagttacaataataccgaccaagaagatcttttggtaatgtgggggatccaccatcctaatgatgcggcagagcagacaaagctctatcaaaatccaaccacctatatctccgttgggacatcaacactaaaccagagattgacaccaagaatagctactagatccaaagtaaacgggcaaagcgggaggatggagttcttctggacaattttaaaaccgaatgatgcaatcaacttcgagagtaatggaaatttcattgctccagaatatgcatacaaaattgtcaagaaaggggactcaacaattatgaaaagtgaattggaatatggtaactgcaacaccaagtgtcaaactccaatgggggcgataaactctagtatgccattccacaatatacatcctctcactattggggaatgccccaaatatgtgaaatcaaacagattagtccttgcgactgggctcagaaatagccctcaaagagagggaagaagaaaaaagagaggattatttggagctatagcaggttttatagagggaggatggcaaggaatggtagatggttggtatgggtaccaccatagcaatgagcaggggagtgggtacgctgcagacaaagaatccactcaaaaggctatagatggagtcaccaataaggtcaactcgatcattgacaaaatgaacactcagtttgaggccgttggaagggaatttaacaacttagaaagaagaatagagaatttaaacaagaagatggaagacgggttcctagatgtctggacttataatgctgaacttctggttctcatggaaaatgagagaactctagacttccatgactcaaatgtaaagaacctttatgacaaggtccgattacagcttagggataatgcaaaggagctgggtaacggttgtttcgagttctatcacaaatgtgataatgaatgtatggaaagtgtgagaaacgggacgtatgactacccgcagtattcagaagaagcaagattaaaaagagagggaataagtggagtgaaattggaatcgataggaatttaccaaatactgtcaatttattctacagtggcgagttccctagcactggcaatcatggtagctggtctatccttatggatgtgctccaaggggtcgttacaatgcagaatttgcatttga

>H5N1_A/Muscovy duck/Ca Mau/1181/2006

cttgttaaaagtgatcagatttgcattggttaccatgcaaacaactcgacagagcaggttgacacaataatggaaaagaacgttactgttacacatgcccaagacatactggaaaagacacataacgggaagctctgtgatctagatggagtgaagcctctaattttgagagattgtagtgtagctggatggcttctcggaaacccaatgtgtgacgaattcatcaatgtgccggaatggtcttacatagtggagaaggccgatccagtcaatgacctctgttacccaggagttttcaatgactatgaagaattgaaacacctattgagcagaataaaccattttgagaaaattcagatcatccccaaaagttcttggcccagtcatgaagcctcattgggggtgagcgcagcatgtccataccagggaaagtcctcttttttcagaaatgtggtatggcttatcaaaaagaacagtacatacccaacaataaagaggagctacaataataccaaccaagaagatcttttggtaatgtgggggatccaccatcctaatgatgcggcagagcagacaaagctctatcaaaatccaaccacctatatctccgttgggacatcaacactaaaccagagattgacaccaagaatagctactagatccaaagtaaacgggcaaagtgggaggatggagttcttctggacaattttaaaaccgaatgatgcaatcaacttcgagagtaatggaaatttcattgctccagaatatgcatacaaaattgtcaagaaaggggactcaacaattatgaaaagtgaattggaatatggtaactgcaacaccaagtgtcaaactccaatgggggcgataaactctagtatgccattccacaatatacatcctctcactattggggaatgccccaaatatgtgaaatcaaacagattagtccttgcgactgggctcagaaatagccctcaaagagagggaagaagaaaaaagagaggattatttggagctatagcaggttttatagagggaggatggcagggaatggtagatggttggtatgggtaccaccatagcaatgagcaggggagtgggtacgctgcagacaaagaatccactcaaaaggctatagatggagtcaccaataaggtcaactcgatcattgacaaaatgaacactcagtttgaggccgttggaagggaatttaacaacttagaaagaagaatagagaatttaaacaagaagatggaagacgggttcctagatgtctggacttataatgctgaacttctggttctcatggaaaatgagagaactctagacttccatgactcaaatgtaaagaacctttacgacaaggtccgattacagcttagggataatgcaaaggagctgggtaacggttgtttcgagttctatcacaaatgtgataatgaatgtatggaaagtgtgagaaacgggacgtatgactacccgcagtattcagaagaagcaagattaaaaagagaggaaataagtggagtgaaattggaatcgataggaatttaccaaatactgtcaatttattctacagtggcgagttccctagcactggcaatcatggtagctggtctatccttatggatgtgctccaatgggtcgttacaatgcagaatttgcatttga

>H5N1_A/Muscovy duck/Ca Mau/1185/2006

cttgttaaaagtgatcagatttgcattggttaccatgcaaacaactcgacagagcaggttgacacaataatggaaaagaacgttactgttacacatgcccaagacatactggaaaagacacataacgggaagctctgtgatctggatggagtgaagcctttaattttgagagattgtagtgtagctggatggcttctcggaaacccaatgtgtgacgaattcatcaatgtgccggaatggtcttacatagtggagaaggccaatccagtcaatgacctctgttacccaggagttttcaatgactatgaagaattgaaacacctattgagcagaataaaccattttgagaaaattcagatcatccccaaaagttcttggcccagtcatgaagcctcattgggggtgagcgcagcatgtccataccagggaaagtcctcttttttcagaaatgtggtatggcttatcaaaaagaacagtacatacccaacaataaagaggagttacaataataccaaccaagaagatcttttggtaatatgggggatccaccatcctaatgatgcggcagagcagacaaagctctatcaaaatccaaccacctatatctccgttgggacatcaacactaaaccagagattgacaccaagaatagctactagatccaaagtaaacgggcaaagtgggaggatggagttcttctggacaattttaaaaccgaatgatgcaatcaacttcgagagtaatggaaatttcattgctccagaatatgcatacaaaattgtcaagaaaggggactcaacaattatgaaaagtgaattggaatatggtaactgcaacaccaagtgtcaaactccaatgggggcgataaactctagtatgccattccacaatatacatcctctcactattggggaatgccccaaatatgtgaaatcaaacagattagtccttgcgactgggctcagaaatagccctcaaagagaggggagaagaaaaaagagaggattatttggagctatagcaggttttatagagggaggatggcagggaatggtagatggttggtatgggtaccaccatagcaatgagcaggggagtgggtacgctgcagacaaagaatccactcaaaaggctatagatggagtcaccaataaggtcaactcgatcattgacaaaatgaacactcagtttgaggccgttggaagggaatttaacaacttagaaagaagaatagagaatttaaacaagaagatggaagacgggttcctagatgtctggacttataatgctgaacttctggttctcatggaaaatgagagaactctagacttccatgactcaaatgtaaagaacctttacgacaaggtccgattacagcttagggataatgcaaaggagctgggtaacggttgtttcgagttctatcacaaatgtgataatgaatgtatggaaagtgtgaggaacgggacgtatgactacccgcagtattcagaagaagcaagattaaaaagagaggaaataagtggagtgaaattggaatcgataggaatttaccaaatactgtcaatttattctacagtggcgagttccctagcactggcaatcatggtagctggtctatccttatggatgtgctccaatgggtcgttacaatgcagaatttgcatttga

>H5N1_A/Muscovy duck/Ca Mau/22/2007

cttgttaaaagtgatcagatttgcattggttaccatgcaaacaactcgacagagcaggttgacacaataatggaaaagaacgttactgttacacatgcccaagatatactggaaaagacacacaacgggaagctctgcgatctagatggagtgaagcctctaattttaagagattgtagtgtagctggatggctcctcggaaacccaatgtgtgacgaattcatcaatgtgccggaatggtcttacatagtggagaaggccaacccagccaatgacctctgttacccagggaatttcaacgactatgaagaactgaaacacctattgagcagaataaaccattttgagaaaattcagatcatccccaaaagttcttggtccgatcatgaagcctcattaggggtgagctcagcatgtccataccagggaacgccctcctttttcagaaatgtggtatggcttatcaaaaagaacaatacatacccaacaataaagaaaagctacaataataccaaccaggaagatcttttgatactgtgggggattcatcattctaatgatgcggcagagcagacaaagctctatcaaaacccaaccacttatatttccgttgggacatcaacactaaaccagagattggtaccaaaaatagccactagatccaaagtaaacgggcaaagtggaaggatggatttcttctggacaattttaaaaccgaatgatgcaatcaacttcgagagtaatggaaatttcattgctccagaatatgcatacaaaattgtcaagaaaggggactcagcaattatgaaaagtgaagtggaatatggtaactgcaacaccaagtgtcaaactccaataggggcgataaactctagtatgccattccacaacatacaccctctcaccatcggggaatgccccaaatacgtgaaatcaaacaaattagtccttgcgactgggctcagaaatagtcctctaagagaaagaagaagaaaaGGGagaggactatttggagctatagcaggttttatagagggaggatggcagggaatggtagatggttggtatgggtaccaccatagcaatgagcaggggagtgggtacgctgcagacaaagaatccactcaaaaggcaatagatggagtcaccaataaggtcaactcgatcattgacaaaatgaacactcagtttgaggccgttggaagggaatttaataacttagaaaggagaatagagaatttaaacaagaaaatggaagacggattcctagatgtctggacttataatgctgaacttctggttctcatggaaaacgagagaactctagacttccatgactcaaatgtcaagaacctttacgacaaggtccgactacagcttagggataatgcaaaggagctgggtaacggttgtttcgagttctatcacaaatgtaataatgaatgcatagaaagtgtaaaaaacggaacgtatgactacccacagtattcagaagaagcaagattaaaaagagaggaaataagtggagtaaaattggaatcaataggaacttaccaaatactgtcaatttattcaacagtggcgagttccctagcactggcaatcatggtggctggtctatctttatggatgtgctccaatgggtcgttacaatgcagaatttgcatttga

>H5N1_A/Muscovy duck/Hai Duong/46/2007

cttgttaaaagtgatcagatttgcattggttaccatgcaaacaactcgacagagcaggttgacacaataatggaaaagaacgttactgttacacatgcccaagatatactggaaaagacacacaacgggaagctctgcgatctagatggagtgaagcctctgattttaagagattgtagtgtagctggatggctcctcggaaacccaatgtgtgacgaattcatcaatgtgccggaatggtcttacatagtggagaaggccaacccagccaatgacctctgttacccagggaatttcaacgactatgaagaactgaaacacctattgagcagaataaaccattttgagaaaattcagatcatccccaaaagttcttggtccgatcatgaagcctcattaggggtgagctcagcatgtccataccatgggaggccctcctttttcagaaatgtggtatggcttatcaaaaagaacaatacatacccaacaataaagagaagctacaataataccaaccaagaagatcttttgatactgtgggggattcatcattctaatgatgcggcagagcagacaaagctctatcaaaacccaaccacctatatttccgttgggacatcaacactaaatcagagattgataccaaaaatagctactagatccaaagtaaacgggcaaagtggaaggatggatttcttctggacaattttaaaacagaatgatgcaatcaacttcgagagtaatggaaatttcattgctccagaatatgcatacaaaattgtcaagaaaggggactcagcaattatgaaaagtgaagtggaatatggtaactgcaacaccaggtgtcaaactccaataggggcgataaactctagtatgccattccacaacatacaccctctcaccattggggaatgccccaaatatgtgaaatcaaacaaattagtccttgcgactgggctcagaaatagtcctctaagagaaagaagaagaaaaGGGagaggattatttggagctatagcaggttttatagagggaggatggcagggaatggtagatggttggtatgggtaccaccatagcaatgagcaggggagtgggtacgctgcagacaaagaatccactcaaaaggcaatagatggagtcaccaataaggtcaactcgatcattgacaaaatgaacactcagtttgaggccgttggaagggaatttaataacttagaaaggagaatagagaatttaaacaagaaaatggaagacggattcctagatgtctggacttataatgctgaacttctggttctcatggaaaatgagagaactctagacttccatgactcaaatgtcaagaacctttacgacaaggtccgactacagcttagggataatgcaaaggagctggggaacggttgtttcgagttctatcacaaatgtgataatgaatgcatggaaagtgtaagaaacggaacgtatgactacccgcagtattcagaagaagcaagattaaaaagagaggaaataagtggagtaaaattggaatcaataggaacttaccaaatactgtcaatttattcaacagttgcaagttctctagcactggcaatcatggtggctggtctatctttatggatgtgctccaatgggtcgttacaatgcagaatttgcatttga

>H5N1_A/Muscovy duck/Vietnam/213/2005

cttgttaaaagtgatcagatttgcattggttaccatgcaaacaactcgacagagcaggttgacacaataatggaaaagaacgttactgttacacatgcccaagatatactggaaaagacacacaacgggaagctctgcgatctagatggagtgaagcctctgattttaagagattgtagtgtagctggatggctcctcggaaacccaatgtgtgacgaattcatcaatgtgccggaatggtcttacatagtggagaaggccaacccagccaatgacctctgttacccagggaatttcaacgactatgaagaactgaaacacctattgagcagaataaaccattttgagaaaattcagatcatccccaaaagttcttggtccgatcatgaagcctcatcaggggtgagctcagcatgtccataccagggaacgccctcctttttcagaaatgtggtatggcttatcaaaaagaacaatacatacccaacaataaagagaagctacaataataccaaccaggaagatcttttgatactgtgggggattcatcattctaatgatgcggcagagcagataaagctctatcaaaacccaaccacctatatttccgttgggacatcaacactaaaccagagattggtaccaaaaatagctactagatccaaagtaaacgggcaaagtggaaggatggatttcttctggacaattttaaaaccgaatgatgcaatcaacttcgagagtaatggaaatttcattgctccagaatatgcatacaaaattgtcaagaaaggggactcagcaattatgaaaagtgaagtggaatatggtaactgcaacaccaagtgtcaaactccaataggggcgataaactctagtatgccattccacaacatacaccctctcaccatcggggaatgccccaaatatgtgaaatcaaacaaattagtccttgcgactgggctcagaaatagtcctctaagagaaagaagaagaaaaGGGagaggactatttggagctatagcaggttttatagagggaggatggcagggaatggtagatggttggtatgggtaccaccatagcaatgagcaggggagtgggtacgctgcagacaaagaatccactcaaaaggcaatagatggagtcaccaataaggtcaactcgatcattgacaaaatgaacactcagtttgaggccgttggaagggaatttaataacttagaaaggagaatagagaatttaaacaagaaaatggaagacggattcctagatgtctggacttataatgctgaacttctggttctcatggaaaatgagagaactctagacttccatgactcaaatgtcaagaacctttacgacaaggtccgactacagcttagggataatgcaaaggagctgggtaacggttgtttcgagttctatcacaaatgtgataatgaatgcatggaaagtgtaagaaacggaacgtatgactacccgcagtattcagaagaagtaagattaaaaagagaggaaataagtggagtaaaattggaatcaataggaacttaccaaatactgtcaatttattcaacagttgcgagttctctagcactggcaatcatggtggctggtctatctttatggatgtgctccaatgggtcgttacaatgcagaatttgcatttga

>H5N1_A/Muscovy duck/Vietnam/217/2005

cttgttaaaagtgatcagatttgcattggttaccatgcaaacaactcgacagagcaggttgacacaataatggaaaagaacgttactgttacacatgcccaagatatactggaaaagacacacaacgggaagctctgcgatctagatggagtgaagcctctgattttaagagattgtagtgtagctggatggctcctcggaaacccaatgtgtgacgaattcatcaatgtgccggaatggtcttacatagtggagaaggccaacccagccaatgacctctgttacccagggaatttcaacgactatgaagaactgaaacacctattgagcagaataaaccattttgagaaaattcagatcatccccaaaagttcttggtccgatcatgaagcctcatcaggggtgagctcagcatgtccataccagggaacgccctcctttttcagaaatgtggtatggcttatcaaaaagaacaatacatacccaacaataaagagaagctacaataataccaaccaggaagatcttttgatactgtgggggattcatcattctaatgatgcggcagagcagataaagctctatcaaaacccaaccacctatatttccgttgggacatcaacactaaaccagagattggtaccaaaaatagctactagatccaaagtaaacgggcaaagtggaaggatggatttcttctggacaattttaaaaccgaatgatgcaatcaacttcgagagtaatggaaatttcattgctccagaatatgcatacaaaattgtcaagaaaggggactcagcaattatgaaaagtgaagtggaatatggtaactgcaacaccaagtgtcaaactccaataggggcgataaactctagtatgccattccacaacatacaccctctcaccatcggggaatgccccaaatatgtgaaatcaaacaaattagtccttgcgactgggctcagaaatagtcctctaagagaaagaagaagaaaaGGGagaggactatttggagctatagcaggttttatagagggaggatggcagggaatggtagatggttggtatgggtaccaccatagcaatgagcaggggagtgggtacgctgcagacaaagaatccactcaaaaggcaatagatggagtcaccaataaggtcaactcgatcattgacaaaatgaacactcagtttgaggccgttggaagggaatttaataacttagaaaggagaatagagaatttaaacaagaaaatggaagacggattcctagatgtctggacttataatgctgaacttctggttctcatggaaaatgagagaactctagacttccatgactcaaatgtcaagaacctttacgacaaggtccgactacagcttagggataatgcaaaggagctgggtaacggttgtttcgagttctatcacaaatgtgataatgaatgcatggaaagtgtaagaaacggaacgtatgactacccgcagtattcagaagaagcaagattaaaaagagaggaaataagtggagtaaaattggaatcaataggaacttaccaaatactgtcaatttattcaacagttgcgagttctctagcactggcaatcatggtggctggtctatctttatggatgtgctccaatgggtcgttacaatgcagaatttgcatttga

>H5N1_A/Muscovy duck/Vietnam/BG80-1/2013

cttgtcaaaagcgatcatatttgcattggttatcatgcaaataactcgacagagcaggttgacacaataatggaaaaaaacgttaccgttacacatgcccaagacatactggaaaggacacacaacgggaagctctgcgatctaaatggagtgaagcctctgattttaaaagattgtagtgtagcaggatggctcctcggaaatccattgtgtgacgaattcaccaatgtgccagaatggtcttacatagtagagaaggccaatccagccaatgacctctgttacccagggaatttcaacgattatgaagaattgaaacacctattgagcaggataaaccattttgagaaaatacagatcatccccaaagattcttggtcagatcatgaagcctcattgggggtgagcgcagcatgttcataccagggaaattcctccttcttcagaaatgtggtgtggcttatcaaaaaggacaatgcatacccaacaataaagaaaggctacaataataccaatcgagaagatctcttgatactgtgggggatccaccatcctaatgatgaggcagagcagacaaggctctaccaaaacccaactacctatatttccattgggacttcaacactaaaccagagattggtaccaaaaatagccactagatccaaaataaacgggcaaagtggcaggatagatttcttctggacaattttaaaaccgaatgacgcaatccacttcgagagtaatggaaatttcattgctccagaatatgcatacaaaattgtcaagaagggagactccacaatcatgagaagtgaagtggaatatggtaactgcaacaccaggtgccagactccaataggggcgataaactctagtatgccattccacaacatacaccctctcaccatcggagaatgtcccaaatatgtgaaatcaaacaaattagtccttgcaactgggctcagaaatagtcctcaaagagagagaagaagaaaaGGGagaggactgtttggagctatagcaggttttatagagggaggatggcagggaatggtagatggttggtatgggtaccaccacagcaatgaacaggggagtggttacgctgcagacaaagaatctactcaaaaggcgatagacggagtcaccaataaggtcaattcgatcattgacaaaatgaacactcagtttgaggctgtaggaagggaattcaataacttggagaggagaatagaaaatttaaacaagaagatggaagacggattcctagatgtctggacttataatgctgaacttctggttctcatggagaatgagagaactctagacttccatgactcaaatgtcaagaacctttacgataaggtccgactacagcttaaggataatgcaaaagagctgggaaacggttgtttcgagttctatcacaaatgtaataatgaatgtatggaaagtgtaagaaacgggacgtatgactacccacagtactcagaagaagcaagattaaaaagagaggaaataagtggagtaaaactggaatcaataggaatctaccaaatactgtcaatttattcaacagtggcgagttccctagtgctggcaatcatgatggctggtctatctttatggatgtgttccaacgggtcgttacagtgcagaatttgcatttga

>H5N1_A/Muscovy duck/Vietnam/BG80-2/2013

cttgtcaaaagcgatcatatttgcattggttatcatgcaaataactcgacagagcaggttgacacaataatggaaaaaaacgttaccgttacacatgcccaagacatactggaaaggacacacaacgggaagctctgcgatctaaatggagtgaagcctctgattttaaaagattgtagtgtagcaggatggctcctcggaaatccattgtgtgacgaattcaccaatgtgccagaatggtcttacatagtagagaaggccaatccagccaatgacctctgttacccagggaatttcaacgattatgaagaattgaaacacctattgagcaggataaaccattttgagaaaatacagatcatccccaaagattcttggtcagatcatgaagcctcattgggggtgagcgcagcatgttcataccagggaaattcctccttcttcagaaatgtggtgtggcttatcaaaaaggacaatgcatacccaacaataaagaaaggctacaataataccaatcgagaagatctcttgatactgtgggggatccaccatcctaatgatgaggcagagcagacaaggctctaccaaaacccaactacctatatttccattgggacttcaacactaaaccagagattggtaccaaaaatagccactagatccaaaataaacgggcaaagtggcaggatagatttcttctggacaattttaaaaccgaatgacgcaatccacttcgagagtaatggaaatttcattgctccagaatatgcatacaaaattgtcaagaagggagactccacaatcatgagaagtgaagtggaatatggtaactgcaacaccaggtgccagactccaataggggcgataaactctagtatgccattccacaacatacaccctctcaccatcggagaatgtcccaaatatgtgaaatcaaacaaattagtccttgcaactgggctcagaaatagccctcaaagagagagaagaagaaaaGGGagaggactgtttggagctatagcaggttttatagagggaggatggcagggaatggtagatggttggtatgggtaccaccacagcaatgaacaggggagtggttacgctgcagacaaagaatctactcaaaaggcgatagacggagtcaccaataaggtcaattcgatcattgacaaaatgaacactcagtttgaggctgtaggaagggaattcaataacttggagaggagaatagaaaatttaaacaagaagatggaagacggattcctagatgtctggacttataatgctgaacttctggttctcatggagaatgagagaactctagacttccatgactcaaatgtcaagaacctttacgataaggtccgactacagcttaaggataatgcaaaagagctgggaaacggttgtttcgagttctatcacaaatgtaataatgaatgtatggaaagtgtaagaaacgggacgtatgactacccacagtactcagaagaagcaagattaaaaagagaggaaataagtggagtaaaactggaatcaataggaatctaccaaatactgtcaatttattcaacagtggcgagttccctagtgctggcaatcatgatggctggtctatctttatggatgtgttccaacgggtcgttacagtgcagaatttgcatttga

>H5N1_A/Muscovy duck/Vietnam/HU10-1139/2018

cttgtcaaaagcgatcatatttgcattggttaccatgcaaataactcgacagagcaggttgacacaataatggaaaaaaacgttactgttacacaagcccaagacatactggaaaagacacacaacgggaagctctgcgatctaaatggagtgaagcctctgattttaaaagattgtagtgtagcaggatggctcctcggaaatccattgtgtgacgaattcaccaatgtgccagaatggtcttacatagtagagaaggccaatccagccaatgacctctgttacccagggaatttcaatgattatgaagaattgaaacacctattgagcaggataaaccattttgagaaaatacagatcatccccaaaaactcttggtcagatcatgaagcctcattgggggtaagcgccgcgtgttcataccagggaaattcctccttcttcagaaatgtggtgtggcttatcaaaaagaacaatgcatacccaacaataaagaaagactacaataacaccaatcgagaagatctcttgatactgtggggaatccaccatcctaatgatgaggcagagcagacgaggctctaccaaaacccaactacctatatttccattgggacttcaacattaaaccagaggttggtgccaaaaatagccactagacccaaaataaacgggcaaagtggcaggatagatttcttctggacaattttaaaaccgaatgacgcaatccacttcgagagtaatggaaatttcattgctccagaatttgcatacaaaattgtcaagaagggagactccacaatcatgagaagtgaagtacaatatggcaactgcaacaccaggtgtcagactccaataggagcgataaactctagtatgccattccacaacatacaccctctcactatcggagaatgtcccaaatatgtgaaatcaagcaaattagtccttgcaaccgggctcagaaatagtcctcaaagagagagaagaagaaaaGGGagaggactgtttggagctatagcaggctttatagagggaggttggcaaggaatggtagatggttggtatgggtaccatcacagcaatgaacaggggagtggttacgctgcagacaaagaatctactcaaaaggcgatagacggggtcaccaataaggtcaattcgatcattgacaaaatgaacactcagtttgaggctgtaggaagggaatttaataacttagagaggagaatagaaaatctaaacaagaagatggaagatggattcctagatgtctggacttataatgctgaacttctggttctcatggagaatgagagaactctagacttccatgactcaaatgtcaagaacctttatgataaggtccgactacagcttaaggataatgcaaaagaactgggaaatggttgtttcgagttctatcacaaatgtaataatgaatgtatggaaagtgtaagaaacgggacgtatgactacccgcagtattcagaagaagcaagattaaaaagagaggaaataagtggagtaaaattggaatcaataggaatctaccaaatactgtcaatttattcaacagtggcgagttccctagtgctggcaatcatgatggctggtctatctttatggatgtgttccaacgggtcgttacagtgcagaatttgcatttga

>H5N1_A/Muscovy duck/Vietnam/HU10-1703/2018

cttgtcaaaagcgatcatatttgcattggttaccatgcaaataactcgacagagcaggttgacacaataatggaaaaaaacgttactgttacacaagcccaagacatactggaaaagacacacaacgggaagctctgcgatctaaatggagtgaagcctctgattttaaaagattgtagtgtagcaggatggctcctcggaaatccattgtgtgacgaattcaccaatgtgccagaatggtcttacatagtagagaaggccaatccagccaatgacctctgttacccagggaatttcaatgattatgaagaattgaaacacctattgagcaggataaaccattttgagaaaatacagatcatccccaaaaactcttggtcagatcatgaagcctcattgggggtaagcgccgcgtgttcataccagggaaattcctccttcttcagaaatgtggtgtggcttatcaaaaagaacaatgcatacccaacaataaagaaagactacaataacaccaatcgagaagatctcttgatactgtggggaatccaccatcctaatgatgaagcagagcagacgaggctctaccaaaacccaactacctatatttccattgggacttcaacattaaaccagaggttggtgccaaaaatagccactagacccaaaataaaagggcaaagtggcaggatagatttcttctggacaattttaaaaccgaatgacgcaatccacttcgagagtaatggaaatttcattgctccagaatatgcatacaaaattgtcaagaagggagactccacaatcatgagaagtgaagtacaatatggcaactgcaacaccaggtgtcagactccaataggagcgataaactctagtatgccattccacaacatacaccctctcactatcggagaatgtcccaaatatgtgaaatcaagcaaattagtccttgcaaccgggctcagaaatagtcctcaaagagagagaagaagaaaaGGGagaggactgtttggagctatagcaggctttatagagggaggttggcagggaatggtagatggttggtatgggtaccaccacagtaatgaacaggggagtggttacgctgcagacaaagaatctactcaaaaggcgatagacggggtcaccaataaggtcaattcgatcattgacaaaatgaacactcagtttgaggctgtaggaagggaatttaataacttagagaggagaatagaaaatctaaacaagaagatggaagatggattcctagatgtctggacttataatgctgaacttctggttctcatggagaatgagagaactctagacttccatgactcaaatgtcaagaacctttatgataaggtccgactacagcttaaggataatgcaaaagaactgggaaatggttgtttcgagttctatcacaaatgtaataatgaatgtatggaaagtgtaagaaacgggacgtatgactacccgcagtattcagaagaagcaagattaaaaagagaggaaataagtggagtaaaattggaatcaataggaatctaccaaatactgtcaatttattcaacagtggcgagttccctagtgctggcaatcatgatggctggtctatctttatggatgtgttccaacgggtcgttacagtgcagaatttgcatttga

>H5N1_A/Muscovy duck/Vietnam/HU3-1079/2015

cttgtcaaaagcgaccatatttgcattggttatcatgcaaataactcgacagagcaggttgacacaataatggaaaagaacgttactgttacacatgcccaagacatactggaaaagacacacaacgggaagctctgcgatctaaatggagtgaagcctctgattttaaaagattgtagtgtagcaggatggctcctcggaaatccattgtgtgacgaattcaccaatgtaccagaatggtcttacatagtagagaaggccaatccagccaatgacctctgttacccagggaatttcaacgattatgaagaattgaaacacctattgagcaggataaaccattttgagaaaatacagatcatccccaaagattcttggtcagatcatgaagcctcattgggggtgagcgccgcatgttcataccagggaaattcctccttcttcagaaatgtggtgtggcttatcaaaaaggacaatgcatacccaacaataaagaaaggctacaataacaccaatcgagaagatctcttgatactgtgggggatccaccatcctaatgatgaggcagagcagacaaggctctaccaaaacccaactacctatatttccattgggacttcaacactaaaccagagattggtaccaaaaatagccactagatccaaaataaacgggcaaagtggcaggatagatttcttctggacaattttaaaaccgaatgacacaatccacttcgagagtaatggaaatttcattgctccagaatatgcatacaaaattgtcaagaagggagactccacaatcatgagaagtgaagtgaaatatggtaactgcaacaccaggtgtcagaccccaataggggcaataaactctagtatgccattccacaacatacaccctctcactatcggagaatgtcccaaatatgtgaaatcaaacaaattagtccttgcaactgggctcagaaatagtcctcaaagagagagaagaagaaaaGGGagaggactgtttggagctatagcaggttttatagagggaggatggcaaggaatggtagatggttggtatgggtaccaccacagcaatgaacaggggagtggttacgctgcagacagagaatctactcaaaaggcgatagacggggtcaccaataaggtcaattcgatcattgacaaaatgaacactcagtttgaggctgtaggaagggaatttaataacttagagaggagaatagaaaatttaaacaagaagatggaagacggattcctagatgtctggacttataatgctgaacttctggttctcatggagaatgagagaactctagacttccatgactcaaatgtcaagaacctttacgataaggtccgactacagcttaaggataatgcaaaagagctgggaaacggttgtttcgagttctatcacaaatgtaataatgaatgtatggaaagtgtaagaaacgggacgtataactacccgcagtattcagaagaagcaagattaaaaagagaggaaataagtggagtaaaactggaatcaataggaatctaccaaatactgtcaatttattcaacagtggcgagttccctagtgctggcaatcatgatggctggtctatctttatggatgtgttccaacgggtcgttacagtgcagaatttgcatttga

>H5N1_A/Muscovy duck/Vietnam/HU3-1360/2015

cttgtcaaaagcgaccatatttgcattggttatcatgcaaataactcgacagagcaggttgacacaataatggaaaagaacgttactgttacacatgcccaagacatactggaaaagacacacaacgggaagctctgcgatctaaatggagtgaagcctctgattttaaaagattgtagtgtagcaggatggctcctcggaaatccattgtgtgacgaattcaccaatgtaccagaatggtcttacatagtagagaaggccaatccagccaatgacctctgttacccagggaatttcaacgattatgaagaattgaaacacctattgagcaggataaaccattttgagaaaatacagatcatccccaaagattcttggtcagatcatgaagcctcattgggggtgagcgccgcatgttcataccagggaaattcctccttcttcagaaatgtggtgtggcttatcaaaaaggacaatgcatacccaacaataaagaaaggctacaataacaccaatcgagaagatctcttgatactgtgggggatccaccatcctaatgatgaggcagagcagacaaggctctaccaaaacccaactacctatatttccattgggacttcaacactaaaccagagattggtaccaaaaatagccactagatccaaaataaacgggcaaagtggcaggatagatttcttctggacaattttaaaaccgaatgacacaatccacttcgagagtaatggaaatttcattgctccagaatatgcatacaaaattgtcaagaagggagactccacaatcatgagaagtgaagtgaaatatggtaactgcaacaccaggtgtcagaccccaataggggcaataaactctagtatgccattccacaacatacaccctctcactatcggagaatgtcccaaatatgtgaaatcaaacaaattagtccttgcaactgggctcagaaatagtcctcaaagagagagaagaagaaaaGGGagaggactgtttggagctatagcaggttttatagagggaggatggcaaggaatggtagatggttggtatgggtaccaccacagcaatgaacaggggagtggttacgctgcagacagagaatctactcaaaaggcgatagacggggtcaccaataaggtcaattcgatcattgacaaaatgaacactcagtttgaggctgtaggaagggaatttaataacttagagaggagaatagaaaatttaaacaagaagatggaagacggattcctagatgtctggacttataatgctgaacttctggttctcatggagaatgagagaactctagacttccatgactcaaatgtcaagaacctttacgataaggtccgactacagcttaaggataatgcaaaagagctgggaaacggttgtttcgagttctatcacaaatgtaataatgaatgtatggaaagtgtaagaaacgggacgtataactacccgcagtattcagaagaagcaagattaaaaagagaggaaataagtggagtaaaactggaatcaataggaatctaccaaatactgtcaatttattcaacagtggcgagttccctagtgctggcaatcatgatggctggtctatctttatggatgtgttccaacgggtcgttacagtgcagaatttgcatttga

>H5N1_A/Muscovy duck/Vietnam/HU3-1403/2015

cttgtcaaaagcgatcatatttgcattggttatcatgcaaataactcgacagagcaggttgacacaataatggaaaagaacgttactgttacacatgcccaagacatactggaaaagacacacaacgggaagctctgcgatctaaatggagtgaagcctctgattttaaaagattgtagtgtagcaggatggctcctcggaaatccattgtgtgacgaattcaccaatgtgccagaatggtcttacatagtagagaaggccaatccagccaatgacctctgttacccagggaatttcaacgattatgaagaattgaaacacctattgagcaggataaaccattttgagaaaatacagatcatccccaaagattcttggtcaaatcatgaagcctcattgggggtgagcgccgcatgttcataccagggaaattcctccttcttcagaaatgtggtgtggcttatcaaaaagaacaatgcatacccaacaataaagaaaggctacaataacaccaatcgagaagatctcttgatactgtgggggatccaccatcctaatgatgaggcagagcagacaaggctctaccaaaacccaactacctatatttccattgggacttcaacactaaaccagagattggtaccaaaaatagccactagatccaaaataaacgggcaaagtggcaggatagatttcttctggacaattttaaaaccgaatgacacaatccacttcgagagtaatggaaatttcattgctccagaatatgcatacaaaattgtcaagaagggagactccacaatcatgagaagtgaagtgaaatatggcaactgcaacaccaggtgtcagaccccaataggggcgataaactctagtatgccattccacaacatacaccctctcactatcggggaatgtcctaaatatgtgaaatcaaacaaattagtccttgcaactgggctcagaaatagtcctcaaaaagagagaagaagaaaaGGGagaggactgtttggagctatagcaggttttatagaaggaggatggcaaggaatggtagatggttggtatgggtaccaccacagcaatgaacaggggagtggttacgctgcagacaaagaatctactcaaaaggcgatagacggggtcaccaataaggtcaattcgatcattgacaaaatgaacactcagtttgaggctgtaggaagggaatttaataacttagagaggagaatagaaaatttaaacaagaagatggaagacggattcctagatgtctggacttataatgctgaacttctggttctcatggagaatgagagaactctagacttccatgactcaaatgtcaagaacctttacgataaggtccgactacagcttaaggataatgcaaaagagctgggaaacggttgtttcgagttctatcacaaatgtaataatgaatgtatggaaagtgtaagaaacgggacgtatgactacccgcagtattcagaagaagcaagattaaaaagagaggaaataagtggagtaaaactggaatcaataggaatctaccaaatactgtcaatttattcaacagtggcgagttccctagtgctggcaatcatgatggctggtctatctttatggatgtgttccaacgggtcgttacagtgcagaatttgcatttga

>H5N1_A/Muscovy duck/Vietnam/HU3-1408/2015

cttgtcaaaagcgatcatatttgcattggttatcatgcaaataactcgacagagcaggttgacacaataatggaaaagaacgttactgttacacatgcccaagacatactggaaaagacacacaacgggaagctctgcgatctaaatggagtgaagcctctgattttaaaagattgtagtgtagcaggatggctcctcggaaatccattgtgtgacgaattcaccaatgtgccagaatggtcttacatagtagagaaggccaatccagccaatgacctctgttacccagggaatttcaacgattatgaagaattgaaacacctattgagcaggataaaccattttgagaaaatacagatcatccccaaagattcttggtcaaatcatgaagcctcattgggggtgagcgccgcatgttcataccagggaaattcctccttcttcagaaatgtggtgtggcttatcaaaaagaacaatgcatacccaacaataaagaaaggctacaataacaccaatcgagaagatctcttgatactgtgggggatccaccatcctaatgatgaggcagagcagacaaggctctaccaaaacccaactacctatatttccattgggacttcaacactaaaccagagattggtaccaaaaatagccactagatccaaaataaacgggcaaagtggcaggatagatttcttctggacaattttaaaaccgaatgacacaatccacttcgagagtaatggaaatttcattgctccagaatatgcatacaaaattgtcaagaagggagactccacaatcatgagaagtgaagtgaaatatggcaactgcaacaccaggtgtcagaccccaataggggcgataaactctagtatgccattccacaacatacaccctctcactatcggggaatgtcctaaatatgtgaaatcaaacaaattagtccttgcaactgggctcagaaatagtcctcaaaaagagagaagaagaaaaGGGagaggactgtttggagctatagcaggttttatagaaggaggatggcaaggaatggtagatggttggtatgggtaccaccacagcaatgaacaggggagtggttacgctgcagacaaagaatctactcaaaaggcgatagacggggtcaccaataaggtcaattcgatcattgacaaaatgaacactcagtttgaggctgtaggaagggaatttaataacttagagaggagaatagaaaatttaaacaagaagatggaagacggattcctagatgtctggacttataatgctgaacttctggttctcatggagaatgagagaactctagacttccatgactcaaatgtcaagaacctttacgataaggtccgactacagcttaaggataatgcaaaagagctgggaaacggttgtttcgagttctatcacaaatgtaataatgaatgtatggaaagtgtaagaaacgggacgtatgactacccgcagtattcagaagaagcaagattaaaaagagaggaaataagtggagtaaaactggaatcaataggaatctaccaaatactgtcaatttattcaacagtggcgagttccctagtgctggcaatcatgatggctggtctatctttatggatgtgttccaacgggtcgttacagtgcagaatttgcatttga

>H5N1_A/Muscovy duck/Vietnam/HU3-575/2015

cttgtcaaaagcgatcatatttgcattggttatcatgcaaataactcgacagagcaggttgacacaataatggaaaagaacgttactgttacacatgcccaagacatactggaaaagacacacaacgggaaactctgcgatctaaatggagtgaagcctctgattttaaaagattgtagtgtagcaggatggctcctcggaaacccattgtgtgacgaattcacaaatgtgccagaatggtcttacatagtagagaaggccaatccagccaatgacctctgttacccagggaatttcaatgattatgaagaattgaaacacctattgagcaggataaaccattttgagaaaatacagatcatccccaaagattcttggtcaaatcatgaagcctcattgggggtgagcgcagcatgttcataccaaggaaattcctccttcttcagaaatgtggtgtggcttatcaaaaagaacaatgcatacccaacaataaagaaaggctacaataacgccaatcgagaagatctcttgatcctgtgggggatccaccatcctaatgatgaggcagagcagacaaggctctaccaaaacccagctacctatatttccattgggacttcaacactgaaccagagattggtaccaaaaatagccactagatccaaaataaacgggcaaagtggcaggatagatttcttctggacaattttaaaaccgaatgacacaatccatttcgaaagtaatggaaatttcattgctccagaatatgcatacaaaattgtcaagaagggagactccacaatcatgagaagtgaagtggaatatggtaattgcaacaccaggtgtcagactccaataggggcgataaactctagtatgccattccacaacatacaccctctcactatcggagaatgtcccaaatatgtgaaatcaaacaaattagtccttgcaactgggctcagaaatagtcctcaaagagagagaagaagaaaaGGGagaggactgtttggagctatagcaggttttatagagggaggatggcagggaatggtagatggttggtatgggtaccaccacagtaatgaacaggggagtggttatgctgcagacaaagaatctactcaaaaggcgatagacggagtcaccaataaggtcaattcgatcattgacaaaatgaacactcagtttgaggctgtaggaagggaatttaataacttagagaggagaatagaaaatttaaacaagaagatggaagacggattcctagatgtctggacttataatgctgaacttctggttctcatggagaatgagagaactctagacttccatgactcaaatgtcaagaatctttacgataaggtccgactacagcttaaggataatgcaaaagagctgggaaacggttgtttcgagttctatcacaaatgtaataatgaatgtatggaaagtgtaagaaacgggacgtatgactacccgcagtattcagaagaagcaagattaaaaagagaggaaataagtggggtaaaactggaatcaataggagtctaccaaatactgtcgatttattcaacagtggcgagttccctagtgctggcaatcatgatggctggtctatctttatggatgtgttccaacgggtcgttacagtgcagaatttgcatttga

>H5N1_A/Muscovy duck/Vietnam/HU3-579/2015

cttgtcaaaagcgatcatatttgcattggttatcatgcaaataactcgacagagcaggttgacacaataatggaaaagaacgttactgttacacatgcccaagacatactggaaaagacacacaacgggaaactctgcgatctaaatggagtgaagcctctgattttaaaagattgtagtgtagcaggatggctcctcggaaacccattgtgtgacgaattcacaaatgtgccagaatggtcttacatagtagagaaggccaatccagccaatgacctctgttacccagggaatttcaatgattatgaagaattgaaacacctattgagcaggataaaccattttgagaaaatacagatcatccccaaagattcttggtcaaatcatgaagcctcattgggggtgagcgcagcatgttcataccaaggaaattcctccttcttcagaaatgtggtgtggcttatcaaaaagaacaatgcatacccaacaataaagaaaggctacaataacgccaatcgagaagatctcttgatcctgtgggggatccaccatcctaatgatgaggcagagcagacaaggctctaccaaaacccagctacctatatttccattgggacttcaacactgaaccagagattggtaccaaaaatagccactagatccaaaataaacgggcaaagtggcaggatagatttcttctggacaattttaaaaccgaatgacacaatccatttcgaaagtaatggaaatttcattgctccagaatatgcatacaaaattgtcaagaagggagactccacaatcatgagaagtgaagtggaatatggtaattgcaacaccaggtgtcagactccaataggggcgataaactctagtatgccattccacaacatacaccctctcactatcggagaatgtcccaaatatgtgaaatcaaacaaattagtccttgcaactgggctcagaaatagtcctcaaagagagagaagaagaaaaGGGagaggactgtttggagctatagcaggttttatagagggaggatggcagggaatggtagatggttggtatgggtaccaccacagtaatgaacaggggagtggttatgctgcagacaaagaatctactcaaaaggcgatagacggagtcaccaataaggtcaattcgatcattgacaaaatgaacactcagtttgaggctgtaggaagggaatttaataacttagagaggagaatagaaaatttaaacaagaagatggaagacggattcctagatgtctggacttataatgctgaacttctggttctcatggagaatgagagaactctagacttccatgactcaaatgtcaagaatctttacgataaggtccgactacagcttaaggataatgcaaaagagctgggaaacggttgtttcgagttctatcacaaatgtaataatgaatgtatggaaagtgtaagaaacgggacgtatgactacccgcagtattcagaagaagcaagattaaaaagagaggaaataagtggggtaaaactggaatcaataggagtctaccaaatactgtcgatttattcaacagtggcgagttccctagtgctggcaatcatgatggctggtctatctttatggatgtgttccaacgggtcgttacagtgcagaatttgcatttga

>H5N1_A/Muscovy duck/Vietnam/HU3-584/2015

cttgtcaaaagcgatcatatttgcattggttatcatgcaaataactcgacagagcaggttgacacaataatggaaaagaacgttactgttacacatgcccaagacatactggaaaagacacacaacgggaaactctgcgatctaaatggagtgaagcctctgattttaaaagattgtagtgtagcaggatggctcctcggaaacccattgtgtgacgaattcacaaatgtgccagaatggtcttacatagtagagaaggccaatccagccaatgacctctgttacccagggaatttcaatgattatgaagaattgaaacacctattgagcaggataaaccattttgagaaaatacagatcatccccaaagattcttggtcaaatcatgaagcctcattgggggtgagcgcagcatgttcataccaaggaaattcctccttcttcagaaatgtggtgtggcttatcaaaaagaacaatgcatacccaacaataaagaaaggctacaataacgccaatcgagaagatctcttgatcctgtgggggatccaccatcctaatgatgaggcagagcagacaaggctctaccaaaacccagctacctatatttccattgggacttcaacactgaaccagagattggtaccaaaaatagccactagatccaaaataaacgggcaaagtggcaggatagatttcttctggacaattttaaaaccgaatgacacaatccatttcgaaagtaatggaaatttcattgctccagaatatgcatacaaaattgtcaagaagggagactccacaatcatgagaagtgaagtggaatatggtaattgcaacaccaggtgtcagactccaataggggcgataaactctagtatgccattccacaacatacaccctctcactatcggagaatgtcccaaatatgtgaaatcaaacaaattagtccttgcaactgggctcagaaatagtcctcaaagagagagaagaagaaaaGGGagaggactgtttggagctatagcaggttttatagagggaggatggcagggaatggtagatggttggtatgggtaccaccacagtaatgaacaggggagtggttatgctgcagacaaagaatctactcaaaaggcgatagacggagtcaccaataaggtcaattcgatcattgacaaaatgaacactcagtttgaggctgtaggaagggaatttaataacttagagaggagaatagaaaatttaaacaagaagatggaagacggattcctagatgtctggacttataatgctgaacttctggttctcatggagaatgagagaactctagacttccatgactcaaatgtcaagaatctttacgataaggtccgactacagcttaaggataatgcaaaagagctgggaaacggttgtttcgagttctatcacaaatgtaataatgaatgtatggaaagtgtaagaaacgggacgtatgactacccgcagtattcagaagaagcaagattaaaaagagaggaaataagtggggtaaaactggaatcaataggagtctaccaaatactgtcgatttattcaacagtggcgagttccctagtgctggcaatcatgatggctggtctatctttatggatgtgttccaacgggtcgttacagtgcagaatttgcatttga

>H5N1_A/Muscovy duck/Vietnam/HU3-589/2015

cttgtcaaaagcgatcatatttgcattggttatcatgcaaataactcgacagagcaggttgacacaataatggaaaagaacgttactgttacacatgcccaagacatactggaaaagacacacaacgggaaactctgcgatctaaatggagtgaagcctctgattttaaaagattgtagtgtagcaggatggctcctcggaaatccatggtgtgacgaattcaccaatgtgccagaatggtcttacatagtagagaaggccaatccagccaatgacctctgttacccagggaatttcaacgattatgaagaattgaaacacctattgagcaggataaaccattttgagaaaatacagatcatccccaaagattcttggtcaaatcatgaagcctcattgggggtgagcgcagcatgttcataccaaggaaattcctccttcttcagaaatgtggtgtggcttatcaaaaagaacaatgcatacccaacaataaagaaaggctacaataacaccaatcgagaagatctcttgatcctgtgggggatccaccatcctaatgatgaggcagagcagacaaggctctaccaaaacccagctacctatatttccattgggacttcaacactgaaccagagattggtaccaaaaatagccactagatccaaaataaacgggcaaagtggcaggatagatttcttctggacaattttaaaaccgaatgacacaatccatttcgaaagtaatggaaatttcattgctccagaatatgcatacaaaattgtcaagaagggagactccacaatcatgagaagtgaagtggaatatggtaattgcaacaccaggtgtcagactccaataggggcgataaactctagtatgccattccacaacatacaccctctcactatcggagaatgtcccaaatatgtgaaatcaaacaaattagtccttgcaactgggctcagaaatagtcctcaaagagagagaagaagaaaaGGGagaggactgtttggagctatagcaggttttatagagggaggatggcagggaatggtagatggttggtatgggtaccaccacagtaatgaacaggggagtggttatgctgcagacaaagaatctactcaaaaggcgatagacggagttaccaataaggtcaattcgatcattgacaaaatgaacactcagtttgaggctgtaggaagggaatttaataacttagagaggagaatagaaaatttaaacaagaagatggaagacggattcctagatgtctggacttataatgctgaacttctggttctcatggagaatgagagaactctagacttccatgactcaaatgtcaagaacctttacgataaggtccgactacagcttaaggataatgcaaaagagctgggaaacggttgtttcgagttctatcacaaatgtaataatgaatgtatggaaagtgtaagaaacgggacgtatgactacccgcagtattcagaagaagcaagattaaaaagagaggaaataagtggagtaaaactggaatcaataggagtctaccaaatactgtcgatttattcaacagtggcgagttccctagtgctggcaatcatgatggctggtctatctttatggatgtgttccaacgggtcgttacagtgcagaatttgcatttga

>H5N1_A/Muscovy duck/Vietnam/HU3-759/2015

cttgtcaaaagcgaccatatttgcattggttatcatgcaaataactcgacagagcaggttgacacaataatggaaaagaacgttactgttacacatgcccaagacatactggaaaagacacacaacgggaagctctgcgatataaatggagtgaagcctctgattttaaaagattgtagtgtagcaggatggctcctcggaaatccattgtgtgacgaattcaccaatgtaccagaatggtcttacatagtagagaaggccaatccagccaatgacctctgttacccagggaatttcaacgattatgaagaattgaaacacctattgagcaggataaaccattttgagaaaatacagatcatccccaaagattcttggtcagatcatgaagcctcattgggggtgagcgccgcatgttcataccagggaaattcctccttcttcagaaatgtggtgtggcttatcaaaaagaacaatgcatacccaacaataaagaaaggctacaacaacaccaatcgagaagatctcttgatactgtgggggatccaccatcctaatgatgaggcagagcagacaaggctctaccaaaacccaactacctatatttccattgggacttcaacactaaaccagagattggtaccaaaaatagccactagatccaaaataaacgggcaaagtggcaggatagatttcttctggacaattttaaaaccgaatgacacaatccacttcgagagtaatggaaatttcattgctccagaatatgcatacaaaattgtcaagaagggagactccacaatcatgagaagtgaagtgaaatatggtaactgcaacaccaggtgtcagaccccaataggggcaataaactctagtatgccattccacaacatacaccctctcactatcggagaatgtcccaaatatgtgaaatcaaacaaattagtccttgcaactgggctcagaaatagtcctcaaagagagagaagaagaaaaGGGagaggactgtttggagctatagcaggttttatagagggaggatggcaaggaatggtagatggttggtatgggtaccaccacagcaatgaacaggggagtggttacgctgcagacagagaatctactcaaaaggcgatagacggggtcaccaataaggtcaattcgatcattgacaaaatgaacactcagtttgaggctgtaggaagggaatttaataacttagagaggagaatagaaaatttaaacaagaagatggaagacggattcctagatgtctggacttataatgctgaacttctggttctcatggagaatgagagaactctagacttccatgactcaaatgtcaagaacctttacgataaggtccgactacagcttaaggataatgcaaaagagctgggaaacggttgtttcgagttctatcacaaatgtaataatgaatgtatggaaagtgtaagaaacgggacgtatgactacccgcagtattcagaagaagcaagattaaaaagagaggaaataagtggagtaaaactggaatcaataggaatctaccaaatactgtcaatttattcaacagtggcgagttccctagtgctggcaatcatgatggctggtctatctttatggatgtgttccaacgggtcgttacagtgcagaatttgcatttga

>H5N1_A/Muscovy duck/Vietnam/HU3-835/2015

cttgtcaaaagcgaccatatttgcattggttatcatgcaaataactcgacagagcaggttgacacaataatggaaaagaacgttactgttacacatgcccaagacatactggaaaagacacacaacgggaagctctgcgatctaaatggagtgaagcctctgattttaaaagattgtagtgtagcaggatggctcctcggaaatccattgtgtgacgaattcaccaatgtaccagaatggtcttacatagtagagaaggccaatccagccaatgacctctgttacccagggaatttcaacgattatgaagaattgaaacacctattgagcaggataaaccattttgagaaaatacagatcatccccaaagattcttggtcagatcatgaagcctcattgggggtgagcgccgcatgttcataccagggaaattcctccttcttcagaaatgtggtgtggcttatcaaaaagaacaatgcatacccaacaataaagaaaggctacaacaacaccaatcgagaagatctcttgatactgtgggggatccaccatcctaatgatgaggcagagcagacaaggctctaccaaaacccaactacctatatttccattgggacttcaacactaaaccagagattggtaccaaaaatagccactagatccaaaataaacgggcaaagtggcaggatagatttcttctggacaattttaaaaccgaatgacacaatccacttcgagagtaatggaaatttcattgctccagaatatgcatacaaaattgtcaagaagggagactccacaatcatgagaagtgaagtgaaatatggtaactgcaacaccaggtgtcagaccccaataggggcaataaactctagtatgccattccacaacatacaccctctcactatcggagaatgtcccaaatatgtgaaatcaaacaaattagtccttgcaactgggctcagaaatagtcctcaaagagagagaagaagaaaaGGGagaggactgtttggagctatagcaggttttatagagggaggatggcaaggaatggtagatggttggtatgggtaccaccacagcaatgaacaggggagtggttacgctgcagacagagaatctactcaaaaggcgatagacggggtcaccaataaggtcaattcgatcattgacaaaatgaacactcagtttgaggctgtaggaagggaatttaataacttagagaggagaatagaaaatttaaacaagaagatggaagacggattcctagatgtctggacttataatgctgaacttctggttctcatggagaatgagagaactctagacttccatgactcaaatgtcaagaacctttacgataaggtccgactacagcttaaggataatgcaaaagagctgggaaacggttgtttcgagttctatcacaaatgtaataatgaatgtatggaaagtgtaagaaacgggacgtatgactacccgcagtattcagaagaagcaagattaaaaagagaggaaataagtggagtaaaactggaatcaataggaatctaccaaatactgtcaatttattcaacagtggcgagttccctagtgctggcaatcatgatggctggtctatctttatggatgtgttccaacgggtcgttacagtgcagaatttgcatttga

>H5N1_A/Muscovy duck/Vietnam/HU3-Z10/2015

cttgtcaaaagcgaccatatttgcgttggttatcatgcaaataactcgacagagcaggttgacacaataatggaaaagaacgttactgttacacatgcccaagacatactggaaaagacacacaacgggaagctctgcgatctaaatggagtgaagcctctgattttaaaagattgtagtgtagcaggatggctcctcggaaatccattgtgtgacgaattcaccaatgtaccagaatggtcttacatagtagagaaggccaatccagccaatgacctctgttacccagggaatttcaacgattatgaagaattgaaacacctattgagcaggataaaccattttgagaaaatacagatcatccccaaagattcttggtcagatcatgaagcctcattgggggtgagcgccgcatgttcataccagggaaattcctccttcttcagaaatgtggtgtggcttatcaaaaagaacaatgcatacccaacaataaagaaaggctacaataacaccaatcgagaagatctcttgatactgtgggggatccaccatcctaatgatgaggcagagcagacaaggctctaccaaaacccaactacctatatttccattgggacttcaacactaaaccagagattggtaccaaaaatagccactagatccaaaataaacgggcaaagtggcaggatagatttcttctggacaattttaaaaccgaatgacacaatccacttcgagagtaatggaaatttcattgctccagaatatgcatacaaaattgtcaagaagggagactccacaatcatgagaagtgaagtgaaatatggtaactgcaacaccaggtgtcagaccccaataggggcaataaactctagtatgccgttccacaacatacaccctctcactatcggagaatgtcccaaatatgtgaaatcaaacaaattagtccttgcaactgggctcagaaatagtcctcaaagagagagaagaagaaaaGGGagaggactgtttggagctatagcaggttttatagagggaggatggcaaggaatggtagatggttggtatgggtaccaccacagcaatgaacaggggagtggttacgctgcagacagagaatctactcaaaaggcgatagacggggtcaccaataaggtcaattcgatcattgacaaaatgaacactcagtttgaggctgtaggaagggaatttaataacttagagaggagaatagaaaatttaaacaagaagatggaagacggattcctagatgtctggacttataatgctgaacttctggttctcatggagaatgagagaactctagacttccatgactcaaatgtcaagaacctttacgataaggtccgactacagcttaaggataatgcaaaagagctgggaaacggttgtttcgagttctatcacaaatgtaataatgaatgtatggaaagtgtaagaaacgggacgtatgactacccgcagtattcagaagaagcaagattaaaaagagaggaaataagtggagtaaaactggaatcaataggaatctaccaaatactgtcaatttattcaacagtggcgagttccctagtgctggcaatcatgatggctggtctatctttatggatgtgttccaacgggtcgttacagtgcagaatttgcatttga

>H5N1_A/Muscovy duck/Vietnam/HU3-Z2/2015

cttgtcaaaagcgaccatatttgcattggttatcatgcaaataactcgacagagcaggttgacacaataatggaaaagaacgttactgttacacatgcccaagacatactggaaaagacacacaacgggaagctctgcgatctaaatggagtgaagcctctgattttaaaagattgtagtgtagcaggatggctcctcggaaatccattgtgtgacgaattcaccaatgtaccagaatggtcttacatagtagagaaggccaatccagccaatgatctctgttacccagggaatttcaacgattatgaagaattgaaacacctattgagcaggataaaccattttgagaaaatacagatcatccccaaagattcttggtcagatcatgaagcctcattgggggtgagcgccgcatgttcataccagggaaattcctccttcttcagaaatgtggtgtggcttatcaaaaagaacaatgcatacccaacaataaagaaaggctacaataacaccaatcgagaagatctcttgatactgtgggggatccaccatcctaatgatgaggcagagcagacaaggctctaccaaaacccaactacctatatttccattgggacttcaacactaaaccagagattggtaccaaaaatagccactagatccaaaataaacgggcaaagtggcaggatagatttcttctggacaattttaaaaccgaatgacacaatccacttcgagagtaatggaaatttcattgctccagaatatgcatacaaaattgtcaagaagggagactccacaatcatgagaagtgaagtgaaatatggtaactgcaacaccaggtgtcagaccccaataggggcaataaactctagtatgccattccacaacatacaccctctcactatcggagaatgtcccaaatatgtgaaatcaaacaaattagtccttgcaactgggctcagaaatagtcctcaaagagagagaagaagaaaaGGGagaggactgtttggagctatagcaggttttatagagggaggatggcaaggaatggtagatggttggtatgggtaccaccacagcaatgaacaggggagtggttacgctgcagacagagaatctactcaaaaggcgatagacggggtcaccaataaggtcaattcgatcattgacaaaatgaacactcagtttgaggctgtaggaagggaatttaataacttagagaggagaatagaaaatttaaacaagaagatggaagacggattcctagatgtctggacttataatgctgaacttctggttctcatggagaatgagagaactctagacttccatgactcaaatgtcaagaacctttacgataaggtccgactacagcttaaggataatgcaaaagagctgggaaacggttgtttcgagttctatcacaaatgtaataatgaatgtatggaaagtgtaagaaacgggacgtatgactacccgcagtattcagaagaagcaagattaaaaagagaggaaataagtggagtaaaactggaatcaataggaatctaccaaatactgtcaatttattcaacagtggcgagttccctagtgctggcaatcatgatggctggtctatctttatggatgtgttccaacgggtcgttacagtgcagaatttgcatttga

>H5N1_A/Muscovy duck/Vietnam/LBM113/2012

cttgtcaaaagcgatcatatttgcattggttatcatgcaaataactcgacagagcaggttgacacaataatggaaaagaacgttactgttacacatgcccaagacatactggaaaagacacacaacgggaagctctgcgatctaaatggagtgaagcctctgattttaaaagattgtagtgtagcaggatggctcctcggaaacccaatgtgtgacgaattcatcaatgtgccagaatggtcttacatagtagagaaggccaatccagccaatgacctctgttacccaggaaacttcaacgattatgaagaattgaaacacctattgagcaggataaaccattttgagaaaatacagatcatccccaaagattcttggtcagatcatgaagcctcattgggggtgagtgcagcatgtccataccagggaaattcctccttcttcagaaatgtggtatggcttatcaaaaaggacaatgcatacccaacaataaagaaaggctacaataataccaatcaagaagatctcttggtactgtgggggattcaccatcctaatgatgaggcagagcagacaaagctctatcaaaacccaaccacctatatttccattgggacatcaacactgaaccagagattggtaccaaaaatagccactaggtccaaaataaacgggcaaagtggcaggatagatttcttctggacaattttaaaaccgaatgatgcaatccacttcgagagtaatggaaatttcattgctccagaatatgcatacaaaattgtcaagaaaggagactcaacaattatgaaaagtgaagtggaatatggtaactgcaacaccaggtgtcagactccgataggggcgataaactccagtatgccattccacaacatacaccctctcaccatcggagaatgtcccaaatacgtgaaatcaaacaaactagtccttgcgactgggctcagaaatagtcctcaaagagagagaagaagaaaaGGGagaggattgtttggagctatagcaggttttatagagggaggatggcagggaatggtagatggttggtatgggtaccaccacagcaatgagcaggggagtgggtacgcggcagacaaagaatctactcaaaaggcaatagacggagtcaccaataaggtcaactcgatcattgacaaaatgaacactcagtttgaggccgtaggaagggaatttaataacttagagaggagaatagagaatttgaacaagaagatggaagacggattcctagatgtctggacttataatgctgaacttctggttctcatggaaaatgagagaactctagacttccatgactcaaatgtcaggaacctttacgacaaggtcagactacagctcaaggataatgcaaaagagctgggtaacggttgtttcgagttctatcacaaatgtaataatgaatgtatggaaagtgtaagaaacggaacgtatgactacccgcagtattcagaagaagcaagattaaaaagagaggaaataagtggagtaaaattggaatcaataggaatctaccaaatactgtcaatttattcaacagtggcgagttccctagtactggcaatcatgatggctggtctatctttatggatgtgttccaacggttcgttacagtgcagaatttgcatttga

>H5N1_A/Mute_swan/Netherlands/21037283-002/2021

cttgttaaaagtgatcagatttgcattggttaccatgcaaacaattcgacagagaaggttgacacgataatggaaaagaacgtcactgttacacatgcccaagacatactggaaaaaacacacaacgggaagctctgtgatttaaatggggtgaagcctctgattttaaaggattgtagtgtagctggatggctcctcggaaacccaatgtgcgacgaattcatcagagtgccggaatggtcctacatagtggagcgggctaatccagctaatgacctctgttacccagggagcctcaatgactatgaagaactgaaacacctgttgagcagaataaaccattttgagaagattctgatcatccccaagagctcctggccaaatcatgaaacatcactaggggtgagcgcagcttgtccataccagggagcgccctcctttttcagaaatgtggtgtggcttatcaaaaagaacgatgcatacccaacaataaagataagctacaataataccaatcgggaagatctcttgatactgtgggggattcatcattccaacaatgcagaagaacagacaaatctctataaaaacccaaccacctacatttcagttggaacatcaactttaaaccagaggttggtaccaaaaatagctactagatcccaagtaaacgggcaacgtggaagaatggacttcttctggacaattttaaaaccagatgatgcaatccatttcgagagtaatggaaatttcattgctccagaatatgcatataaaattgtcaagaaaggggactcaacaattatgaaaagtggagtggaatatggccactgcaacaccaaatgtcaaaccccagtaggagcgataaattctagtatgccattccacaacatacatcctctcaccattggggaatgccccaaatacgtgaagtcaaacaagttggtccttgcgactgggctcagaaatagtcctctaagagaaaagagaagaaaaGGGagaggcctgtttggggcgatagcagggtttatagagggaggatggcagggaatggttgatggttggtatgggtaccatcatagcaatgagcaggggagtgggtacgctgcagacaaagaatccacccaaaaggcaatagatggagttaccaataaggtcaactcaatcattgacaaaatgaacactcaatttgaggcagttggaagggagtttaataatttagaaaggaggatagagaatttgaacaagaaaatggaagacggattcctagatgtctggacctataatgctgaacttctagttctcatggaaaacgagaggactctagatttccatgattcaaatgtcaagaacctttacgacaaagtcagactacagcttagggataatgcaaaggagctgggtaatggctgtttcgaattctatcacaaatgcgataatgaatgtatggaaagtgtgagaaatgggacgtatgactaccctcagtattcagaagaagcaagattaaaaagagaagaaataagcggagtgaaattagaatcaataggaacttaccagatactgtcaatttattcaacagcggcgagttccctagcactggcaatcatgatagctggtctatctttatggatgtgctccaatgggtcgttacagtgcagaatttgcatttag

>H5N1_A/Oystercatcher/Netherlands/1/2022

ctagttaaaagtgatcagatttgcattggttaccatgcaaacaattcgacagagcaggttgacacgataatggaaaagaacgtcactgttacacatgcccaagacatactggaaaaaacacacaacgggaagctctgtgatttaaatggggtgaagcctctgattttaaaggattgtagtgtagctggatggctcctcggaaacccaatgtgcgacgaattcatcagagtgccggaatggtcctacatagtggagcgggctaatccagctaatgacctctgttacccagggagcctcaatgactatgaagaactgaaacacctgttgagcagaataaatcattttgagaagattcttatcatccccaagagttcctggccaaatcatgaaacatcactaggggtaagcgcagcttgtccataccagggagcgccctcctttttcagaaatgtggtgtggcttatcaaaaagaacgatgcatacccaacaataaagataagctacaataataccaatcgggaagatctcttgatactgtgggggattcatcattccaacaatgcagaagaacagacaaatctctataaaaacccaaccacctacatttcagttggaacatcaactttaaaccagaggttggtaccaaaaatagctactagatcccaagtaaacgggcaacgtggaagaatggacttcttctggacaattttaaaaccagatgatgcaatccatttcgagagtaatggaaatttcattgctccagaatatgcatataaaattgtcaagaaaggggactcaacaattatgaaaagtggagtggaatatggccactgcaacaccaaatgtcaaaccccagtaggagcgataaattctagtatgccattccacaacatacatcctctcaccattggggaatgccccaaatacgtgaagtcaaacaagttggtccttgcgactgggctcagaaatagtcctctaagagaaaagagaagaaaaGGGagaggcctgtttggggcgatagcagggtttatagagggaggatggcagggaatggttgatggttggtatgggtaccatcatagcaatgagcaggggagtgggtacgctgcagacaaagaatccacccaaaaggcaatagatggagttaccaataaggtcaactcaatcattgacaaaatgaacactcaatttgaggcagttggaagggagtttaataacttagaaaggaggatagagaatttgaacaagaaaatggaagacggattcctagatgtctggacctataatgctgaacttctagttctcatggaaaacgagaggactctagatttccatgattcaaatgtcaagaacctttacgacaaagtcagactacagcttagggataatgcaaaggagctgggtaatggctgtttcgaattctatcacaaatgcgataatgaatgtatggaaagtgtgagaaatgggacgtatgactaccctcagtattcagaagaagcaagattaaaaagagaagaaataagcggagtgaaattagaatcaataggaacttaccagatactgtcaatttattcaacagcggcgagttccctagcactggcaatcatgatagctggtctatctttatggatgtgctccaatgggtcgttacagtgcagaatttgcatttag

>H5N1_A/Pigeon/Egypt/RIMD18/2009

cttgttaaaagtgatcagatttgcattggttaccatgcaaacaactcaacagagcaggttgacacaataatggaaaagaacgtcactgttacacacgctcaagacatactggaaaagacacacaacgggaaactctgcgatctagatggagtgaagcctctaattttaagagattgtagtgtagctggatggctcctcgggaacccaatgtgtgacgaattcctcaatgtgtcggaatggtcttacatagtggagaagatcaatccagccaatgacctctgttacccagggaatttcaacaactatgaagaactgaaacacctattgagcagaataaaccgttttgagaaaattcagatcatccccaaaagttcttggccagatcatgaagcctcattaggagtgagctcagcatgtcaataccagggaggaccctccttttatagaaatgtggtatggcttatcaaaaaggacaatgcatacccaacaataaagaaaagttaccataataccaataaagaagatcttttggtactgtgggggattcaccatccaaataatgaggaagagcagacaaagctctatcaaaacccaactacctatatttccgttgggacatcaacactaaaccagagattggtaccaaagatagctactagatctaaggtaaacgggcaaagtggaagggtggagttcttttggacaattttaaaatcgaatgatgcaataaactttgagagtaatggaaatttcattgctccagaaaatgcatacaaaattgtcaagaaaggggactcaacaattatgaaaagtgagttggaatatggtaactgcaacaccaagtgtcaaactccaataggggcgataaactccagtatgccattccacaacatccaccctctcaccatcggggaatgccccaaatatgtgaagtcaaacagattagtccttgctactgggctcagaaatagccctcaaggagagagaagaagaaaaaagagaggactatttggagctatagcaggttttatagagggaggatggcagggaatggtagatggttggtatgggtaccaccatagcaacgagcaggggagtgggtacgctgcagacaaagaatccactcaaaaggcaatagatggagtcaccaataaagtcaactcgatcattgacaaaatgaacactcagtttgaggctgttggaaaggaatttaataacttagaaaggagaatagaaaatttaaacaagaagatggaagacggattcctagatgtctggacttataatgctgaacttctggttctcatggaaaatgagagaactctagactttcatgactcaaatgtcaagaacctttacgacaaggtgcgactacagcttagggataacgcaaaggagcttggtaacggttgtttcgagttctatcacagatgtgataatgaatgtatggaaagtgtaagaaacggaacgtatgactacccgcagtattctgaagaagcaagattaaaaagagaggaaataagtggagtaaagttggagtcaataggaacttaccaaatactgtcaatttactcaacagtggcaagttccctagcactggcaatcatggtggctggtctatttttatggatgtgctccaatggatcgttacaatgcagaatttgcatttga

>H5N1_A/Pigeon/Egypt/RIMD20/2009

cttgttaaaagtgatcagatttgcattggttaccatgcaaacaactcaacagagcaggttgacacaataatggaaaagaacgtcactgttacacacgctcaagacatactggaaaagacacacaacgggaaactctgcgatctagatggagtgaagcctctaattttaagagattgtagtgtagctggatggctcctcgggaacccaatgtgtgacgaattcctcaatgtgtcggaatggtcttacatagtggagaagatcaatccagccaatgacctctgttacccagggaatttcaacaactatgaagaactgaaacacctattgagcagaataaaccgttttgagaaaattcagatcatccccaaaagttcttggccagatcatgaagcctcattaggagtgagctcagcatgtcaataccagggaggaccctccttttatagaaatgtggtatggcttatcaaaaaggacaatgcatacccaacaataaagaaaagttaccataataccaataaagaagatcttttggtactgtgggggattcaccatccaaataatgaggaagagcagacaaagctctatcaaaacccaactacctatatttccgttgggacatcaacactaaaccagagattggtaccaaagatagctactagatctaaggtaaacgggcaaagtggaagggtggagttcttttggacaattttaaaatcgaatgatgcaataaactttgagagtaatggaaatttcattgctccagaaaatgcatacaaaattgtcaagaaaggggactcaacaattatgaaaagtgagttggaatatggtaactgcaacaccaagtgtcaaactccaataggggcgataaactccagtatgccattccacaacatccaccctctcaccatcggggaatgccccaaatatgtgaagtcaaacagattagtccttgctactgggctcagaaatagccctcaaggagagagaagaagaaaaaagagaggactatttggagctatagcaggttttatagagggaggatggcagggaatggtagatggttggtatgggtaccaccatagcaacgagcaggggagtgggtacgctgcagacaaagaatccactcaaaaggcaatagatggagtcaccaataaagtcaactcgatcattgacaaaatgaacactcagtttgaggctgttggaaaggaatttaataacttagaaaggagaatagaaaatttaaacaagaagatggaagacggattcctagatgtctggacttataatgctgaacttctggttctcatggaaaatgagagaactctagactttcatgactcaaatgtcaagaacctttacgacaaggtgcgactacagcttagggataacgcaaaggagcttggtaacggttgtttcgagttctatcacagatgtgataatgaatgtatggaaagtgtaagaaacggaacgtatgactacccgcagtattctgaagaagcaagattaaaaagagaggaaataagtggagtaaagttggagtcaataggaacttaccaaatactgtcaatttactcaacagtggcaagttccctagcactggcaatcatggtggctggtctatttttatggatgtgctccaatggatcgttacaatgcagaatttgcatttga

>H5N1_A/Sanderling/Netherlands/1/2022

cttgttaaaagtgatcagatttgcattggttaccatgcaaacaattcgacagagcaggttgacacgataatggaaaagaacgtcactgttacacatgcccaagacatactggaaaaaacacacaacgggaagctctgtgatttaaatggggtgaagcctctgattttaaaggattgtagtgtagctggatggctcctcggaaacccaatgtgcgacgaattcatcagagtgccggaatggtcctacatagtggagcgggctaatccagccaatgacctctgttacccagggagcctcaatgactatgaagaactgaaacacctgttgagcagaataaatcattttgagaagattctgatcatccccaagagttcctggccaaatcatgaaacatcactaggggtgagcgcagcttgtccataccagggagcgccctcctttttcagaaatgtgttgtggcttatcaaaaagaacgatgcatacccaacaataaagataagctacaataataccaatcgggaagatctcttgatactgtgggggattcatcattccaacaatgcagaagagcagacaaatctctataaaaacccaaccacctacatttcagttggaacatcaactttaaaccagaggttggtaccaaaaatagctactagatcccaagtaaacgggcaacgtggaagaatggacttcttctggacaattttaaaaccagatgatgcaatccatttcgagagtaatggaaatttcattgcaccagaatatgcatataaaattgtcaagaaaggggactcaacaattatgaaaagtggagtggaatatggccactgcaacaccaaatgtcaaaccccagtaggagcgataaattctagtatgccattccacaacatacatcctctcaccattggggaatgccccaaatacgtgaagtcaaacaagttggtccttgcgactgggctcagaaatagtcctctaagagaaaagagaagaaaaGGGagaggcctgtttggggcaatagcagggtttatagagggaggatggcagggaatggttgatggttggtatgggtaccatcatagcaatgagcaggggagtgggtacgctgcagacaaagaatccacccaaaaggcaatagatggagttaccaataaggtcaactcaatcattgacaaaatgaacactcaatttgaggcagttggaagggagtttaataacttagaaaggaggatagagaatttgaacaagaaaatggaagacggattcctagatgtctggacctataatgctgaacttctagttctcatggaaaacgagaggactctagatttccatgattcaaatgtcaagaacctttacgacaaagtcagactacagcttagggacaatgcaaaggagctgggtaatggatgtttcgaattctatcacaaatgcgataatgaatgtatggaaagtgtgagaaatgggacgtatgactaccctcagtattcagaagaagcaagattaaaaagagaagaaataagcggagtgaaattagaatcaataggaatttaccagatactgtcaatttattcaacagcggcgagttccctagcactggcaatcatgatagctggtctatctttatggatgtgctccaatgggtcgttacagtgcagaatttgcatttag

>H5N1_A/Swiftlet/Vietnam/NCVD-3000/2013

cttgtcaaaagcgatcatatttgcattggttatcatgcaaataactcgacagagcaggttgacacaataatggaaaagaacgttactgttacacatgcccaagacatactggaaaagacacacaacgggaagctctgcgatctaaatggagtgaagcctctgattttaaaagattgtagtgtagcaggatggctcctcggaaatccattgtgtgacgaattcaccaatgtgccagaatggtcttacatagtagagaaggccaatccagccaatgatctctgttacccagggaatttcaacgattatgaagaattgaaacacctattgagcaggataaaccattttgagaaaatacagatcatccccaaagattcttggccagatcatgaagcctcattgggggtgagcgcagcatgttcataccagggaaattcctccttcttcagaaatgtggtgtggctcatcaaaaaggacaatgcatacccaacaataaagaaaggctacaataataccaaccgagaagatctcttgatactgtgggggatccaccatcctaatgatgaggcagagcagacaaggctctaccaaaacccaactacctatatttccattgggacttcaacactaaaccagagattggtaccaaaaatagccactagatccaaaataaacgggcaaagtggcaggatagatttcttctggacaattttaaaaccgaatgacgcaatccacttcgagagtaatggaaatttcattgctccggaatatgcatacaaaattgtcaagaagggagactccacaatcatgagaagtgaagtggaatatggtaactgcaacaccaggtgtcagactccaataggggcgataaactctagtatgccattccacaacatacaccctctcaccatcggagaatgtcccaaatatgtgaaatcaaacaaattagtccttgcaactgggctcagaaatagtcctcaaagagagagaagaagaaaaGGGagaggactgtttggagctatagcaggttttatagagggaggatggcagggaatggtagatggttggtatgggtaccaccacagcaatgaacaggggagtggttacgctgcagacaaagaatctactcaaaaggcgatagacggagtcaccaataaggtcaattcgatcattgacaaaatgaacactcagtttgaggctgtaggaagggaatttaataacttagagaggagaatagaaaatttaaacaagaagatggaagacggattcctagatgtctggacttataatgctgaacttctggttctcatggagaatgagagaactctagacttccatgactcaaatgtcaagaacctttacgataaggtccgactacagcttaaggataatgcaaaagagctgggaaacggttgtttcgagttctatcacaaatgtaataatgaatgtatggaaagtgtaagaaacgggacgtatgactacccgcagtattcagaagaagcaagattaaaaagagaggaaataagtggagtaaaactggaatcaataggaatctaccaaatactgtcaatttattcaacagtggcgagttccctagtgctggcaatcatgatggctggtctatctttatggatgtgttccaacgggtcgttacagtgcagaatttgcatttga

>H5N1_A/Turkey/Hungary/16603/2021_(H5N1)

cttgttaaaagtgatcagatttgcattggttaccatgcaaacaattcgacagagcaagttgacacgataatggaaaagaacgtcactgttacacatgcccaagacatactggaaaaaacacacaacgggaagctctgtgatctaaatggggtgaagcctctgattttaaaggattgtagtgtagctggatggctcctcggaaacccaatgtgcgacgaattcatcagagtgccggaatggtcctacatagtggagcgggctaatccagctaatgacctctgttacccagggagcctcaatgactatgaagaactgaaacacctgttgagcagaataaatcattttgagaagattctgatcatccccaagagttcctggccaaatcatgaaacatcactaggggtgagcgcagcttgtccataccagggagcgccctcctttttcagaaatgtggtgtggcttatcaaaaagaacgatgcatacccaacaataaaaataagctacaataataccaatcgggaagatctcttgatactgtgggggattcatcattccaacaatgcagaagagcagacaaatctctataaaaacccaaccacctacatttcggttggaacatcaactttaaaccagaggttggtaccaaaaatagctactagatcccaagtaaacgggcaacgtggaagaatggacttcttctggacaattttaaaaccagatgatgcaatccatttcgagagtaatggaaatttcattgctccagaatatgcatacaaaattgtcaagaaaggggactcaacaattatgaaaagtggagtggaatatggccactgcaacaccaaatgtcaaaccccagtaggagcgataaattctagtatgccattccacaacatacatcctctcaccattggggaatgccccaaatacgtgaagtcaaacaagttggtccttgcgactgggctcagaaatagtcctctaagagaaaagagaagaaaaGGGagaggcctgtttggggcgatagcagggtttatagagggaggatggcagggaatggttgatggttggtatgggtaccatcatagcaatgagcaggggagtgggtacgctgcagacaaagaatccacccaaaaggcaatagatggagttaccaataaggtcaactcaatcattgacaaaatgaacactcaatttgaggcagttggaagggagtttaataacttagaaaggaggatagagaatttgaacaagaaaatggaagacggattcctagatgtctggacctataatgctgaacttctagttctcatggaaaatgagaggactctagatttccatgattcaaatgtcaagaacctttacgacaaagtcagactacagcttagggataatgcaaaggagctgggtaacggctgtttcgaattctatcacaaatgcgataatgaatgtatggaaagtgtgagaaatgggacgtatgactaccctcagtattcagaagaagcaagattaaaaagagaagaaataagcggagtgaaattagaatcaataggaacttaccagatactgtcaatttattcaacagcggcgagttccctagcactggcaatcatgatggctggtctatctttatggatgtgctccaatgggtcgttacagtgcagaatttgcatttag

>H5N1_A/Turkey/Sweden/SVA211212SZ0001/FB301013-IP-2/M-2021

cttgttaaaagtgatcagatttgcattggttaccatgcaaacaattcgacagagcaggttgacacgataatggaaaagaacgtcactgttacacatgcccaagacatactggaaaaaacacacaacgggaagctctgtgatttaaatggggtgaagcctctgattttaaaggattgtagtgtagctggatggctcctcggaaacccaatgtgcgacgaattcatcagagtgccggaatggtcctacatagtggagcgggctaatccagccaatgacctctgttacccagggagcctcaatgactatgaagaactgaaacacctgttgagcagaataaatcattttgagaagattctgatcatccccaagagttcctggccaaatcatgaaacatcactaggggtgagcgcagcttgtccataccagggagcgccctcctttttcagaaatgtggtgtggctaatcaaaaagaacgatgcatacccaacaataaagataagctacaataataccaatcgggaagatctcttgatactgtgggggattcatcattccaacaatgcagaagagcagacaaatctctataaaaacccaaccacctacatttcagttggaacatcaactttaaaccagaggttggtaccaaaaatagctactagatcccaagtaaacgggcaacgtggaagaatggacttcttctggacaattttaaaaccagatgatgcaatccatttcgagagtaatggaaatttcattgcaccagaatatgcatataaaattgtcaagaaaggggactcaacaattatgaaaagtggagtggaatatggccactgcaacaccaaatgtcaaaccccagtaggagcgataaattctagtatgccattccacaacatacatcctctcaccattggggaatgccccaaatacgtgaagtcaaacaagttggtccttgcgactgggctcagaaatagtcctctaagagaaaagagaagaaaaGGGagaggcctgtttggggcgatagcagggtttatagagggaggatggcagggaatggttgatggttggtatgggtaccatcatagcaatgggcaggggagtgggtacgctgcagacaaagaatccacccaaaaggcaatagatggagttaccaataaggtcaactcaatcattgacaaaatgaacactcaatttgaggcagttggaagggagtttaataacttagaaaggaggatagagaatttgaacaagaaaatggaagacggattcctagatgtctggacctataatgctgaacttctagttctcatggaaaacgagaggactctagatttccatgattcaaatgtcaagaacctttacgacaaagtcagactacagcttagggataatgcaaaggagctgggtaatggctgtttcgaattctatcacaaatgcgataatgaatgtatggaaagtgtgagaaatgggacgtatgactaccctcagtattcagaagaagcaagattaaaaagagaagaaataagcggagtgaaattagaatcaataggaacttaccagatactgtcaatttattcaacagcggcgagttccctagcactggcaatcatgatagctggtctatctttatggatgtgctccaatgggtcgttacagtgcagaatttgcatttag

>H5N1_A/White-Tailed_Eagle/Sweden/SVA210528SZ0223/KN002027/AB-2021

cttgttaaaagtgatcagatttgcattggttaccatgcaaacaattcgacagagcaagttgacacgataatggaaaagaacgtcactgttacacatgcccaagacatactggaaaaaacacacaacgggaagctctgtgatctaaatggggtgaagcctctgattttaaaggattgtagtgtagctggatggctcctcggaaacccaatgtgcgacgaattcatcagagttccggaatggtcctacatagtggagcgggctaatccagctaatgacctctgttacccagggagcctcaatgactatgaagaactgaaacacctgttgagcagaataaatcattttgagaagattctgatcatccccaatagttcctggccaaatcatgaaacatcactaggggtgagcgcagcttgtccataccagggagcgccctcctttttcagaaatgtggtgtggcttatcaaaaagaacgatgcatacccaacaataaagataagctacaataataccaatcgggaagatctcttgatactgtgggggattcatcattccaacaatgcagaagagcagacaaatctctacaaaaacccaaccacctacatttcagttggaacatcaactttaaaccaaaggttggtaccaaaaatagctactagatcccaagtaaacgggcaacgtggaagaatggacttcttctggacaattttaaaaccagatgatgcaatccatttcgagagtaatggaaatttcattgctccagaatatgcatacaaaattgtcaagaaaggggactcaacaattatgaaaagtggagtggaatatggccactgcaacaccaaatgtcaaaccccagtaggagcgataaattctagtatgccattccacaacatacatcctctcaccattggggaatgccccaaatacgtgaagtcaaacaagttggtccttgcgactgggctcagaaatagtcctctaagagaaaagagaagaaaaGGGagaggcctatttggggcgatagcagggtttatagagggaggatggcagggaatggttgatggttggtatgggtaccatcatagcaatgagcaggggagtgggtacgctgcagacaaagaatctacccaaaaggcaatagatggagttaccaataaggtcaactcaatcattgacaaaatgaacactcaatttgaggcagttggaagggagtttaataacttagaaaggaggatagagaatttgaacaagaaaatggaagacggattcctagatgtctggacctataatgctgaacttctagttctcatggaaaacgagaggactctagatttccatgattcaaatgtcaagaacctttacgacaaagtcagactacagcttagggataatgcaaaggagctgggtaacggctgtttcgaattctatcacaaatgcgataatgaatgtatggaaagtgtgagaaatgggacgtatgactaccctcagtattcagaagaagcaagattaaaaagagaagaaataagcggagtgaaattagaatcaataggaacttaccagatactgtcaatttattcaacagcggcaagttccctagcactggcaatcatgatggctggtctatctttatggatgtgctccaatgggtcgttacagtgcagaatttgcatttag

>H5N1_A/Whooper_swan/Scotland/056219/2021

cttgttaaaagtgatcagatttgcattggttaccatgcaaacaattcgacagagcaggttgacacgataatggaaaagaacgtcactgttacacatgcccaagacatactggaaaaaacacacaacgggaagctctgtgatttaaatggggtgaagcctctgattttaaaggattgtagtgtagctggatggctcctcggaaacccaatgtgcgacgaattcatcagagtgccggaatggtcctacatagtggagcgggctaatccagccaatgacctctgttacccagggagcctcaatgactatgaagaactgaaacacctgttgagcagaataaatcattttgagaagattctgatcatccccaagagttcctggccaaatcatgaaacatcactaggggtgagcgcagcttgtccataccagggagcgccctcctttttcagaaatgtgttgtggcttatcaaaaagaacgatgcatacccaacaataaagataagctacaataataccaatcgggaagatctcttgatattgtgggggattcatcattccaacaatgcagaagagcagacaaatctctataaaaacccaaccacctacatttcagttggaacatcaactttaaaccagaggttggtaccaaaaatagctactagatcccaagtaaacgggcaacgtggaagaatggacttcttctggacaattttaaaaccagatgatgcaatccatttcgagagtaatggaaatttcattgcaccagaatatgcatataaaattgtcaagaaaggggactcaacaattatgaaaagtggagtggaatatggccactgcaacaccaaatgtcaaaccccagtaggagcgataaattctagtatgccattccacaacatacatcctctcaccattggggaatgccccaaatacgtgaagtcaaacaagttggtccttgcgactgggctcagaaatagtcctctaagagaaaagagaagaaaaGGGagaggcctgtttggggcgatagcagggtttatagagggaggatggcagggaatggttgatggttggtatgggtaccatcatagcaatgagcaggggagtgggtacgctgcagacaaagaatccacccaaaaggcaatagatggagttaccaataaggtcaactcaatcattgacaaaatgaacactcaatttgaggcagttggaagggagtttaataacttagaaaggaggatagagaatttgaacaagaaaatggaagacggattcctagatgtctggacctataatgctgaacttctagttctcatggaaaacgagaggactctagatttccatgattcaaatgtcaagaacctttacgacaaagtcagactacagcttagggacaatgcaaaggagctgggtaatggctgtttcgaattctatcacaaatgcgataatgaatgtatggaaagtgtgagaaatgggacgtatgactaccctcagtattcagaagaagcaagattaaaaagagaagagataagcggagtgaaattagaatcaataggaacttaccagatactgtcaatttattcaacagcggcgagttccctagcactggcaatcatgatagctggtctatctttatggatgtgctccaatgggtcgttacagtgcagaatttgcatttag

>H5N1_A/Wild_goose/Italy/21VIR10193/2021

cttgttaaaagtgatcagatttgcattggttaccatgcaaacaattcgacagagcaggttgacacgataatggaaaagaacgtcactgttacacatgcccaagacatactggaaaaaacacacaacgggaagctctgtgatttaaatggggtgaagcctctgattttgaaggattgtagtgtagctggatggctcctcggaaacccaatgtgcgacgaattcatcagagtgccagaatggtcctacatagtggagcgggctaatccagccaatgacctctgttacccagggagcctcaatgactatgaagaactgaaacacctgttgagcagaataaatcattttgagaagattctgatcatccccaagagttcctggccaaatcatgaaacatcactaggggtgagcgcagcttgtccataccagggagcgccctcctttttcagaaatgtggtgtggcttatcaaaaagaacgatgcatacccaacaataaagataagctacaataataccaatcgggaagatctcttgatactgtgggggattcatcattccaacaatgcagaagagcagacaaatctctataaaaacccaaccacctacatttcagttggaacatcaactttaaaccagaggttggtaccaaaaatagctactagatcccaagtaaacgggcaacgtggaagaatggacttcttctggacaattttaaaaccagatgatgcaatccatttcgagagtaatggaaatttcattgcaccagaatatgcatataaaatcgtcaagaaaggggactcaacaattatgaaaagtggagtggaatatggccactgcaacaccaaatgtcaaaccccagtaggagcgataaattctagtatgccattccacaacatacatcctctcaccattggggaatgccccaaatacgtgaagtcaaacaagttggtccttgcgactgggctcagaaatagtcctctaagagaaaagagaagaaaaGGGagaggcctgtttggggcgatagcagggtttatagagggaggatggcagggaatggttgatggttggtatgggtaccatcatagcaatgagcaggggagtgggtacgctgcagacaaagaatccacccaaaaggcaatagatggagttaccaataaggtcaactcaatcattgacaaaatgaacactcaatttgaggcagttggaagggagtttaataacttagaaaggaggatagagaatttgaacaagaaaatggaagacggattcctagatgtctggacctataatgctgaacttctagttctcatggaaaacgagaggactctagatttccatgattcaaatgtcaagaacctttacgacaaagtcagactacagcttagggataatgcaaaggagctgggtaatggctgtttcgaattctatcacaaatgcgataatgaatgtatggaaagtgtgagaaatgggacatatgactaccctcagtattcagaagaagcaagattaaaaagagaagaaataagcggagtgaaattagaatcaataggaacttaccagatactgtcaatttattcaacagcggcgagttccctagcactggcaatcatgatagctggtctatctttatggatgtgctccaatgggtcgttacagtgcagaatttgcatttag

>H5N1_A/Withe-tiled_eagle/Estonia/TA2124126-1_21VIR10433-11/2021

cttgttaaaagtgatcagatttgcattggttaccatgcaaacaattcgacagagcaggttgacacgataatggaaaagaacgtcactgttacacatgcccaagacatactggaaaaaacacacaacggaaaactctgtgatttaaatggggtgaagcctctgattttaaaggattgtagtgtagctggatggctcctcggaaacccaatgtgcgacgaattcatcagagtgccggaatggtcctacatagtggagcgggctaatccagccaatgacctctgttacccagggagcctcaatgactatgaagaactgaaacacctgttgagcagaataaatcattttgagaagattctgatcatccccaagagttcctggccaaatcatgaaacatcactaggggtgagcgcagcttgtccataccagggagcgccctcctttttcagaaatgtggtgtggcttatcaaaaagaacgatgcatacccaacaataaagataagctacaataataccaatcgggaagatctcttgatactgtgggggattcatcattccaacaatgcagaagagcagacaaatctctataaaaacccaaccacctacatttcagttggaacatcaactttaaaccagaggttggtaccaaaaatagctactagatcccaagtaaacgggcaacgtggaagaatggacttcttctggacaattttaaaaccagatgatgcaatccatttcgagagtaatggaaatttcattgcaccagaatatgcatataaaattgtcaagaaaggggactcaacaattatgaaaagtggagtggaatatggccactgcaacaccaaatgtcaaaccccagtaggagcgataaattctagtatgccattccacaacatacatcctctcaccattggggaatgccccaaatacgtgaagtcaaacaagttggtccttgcgactgggctcagaaatagtcctctaagagaaaagagaagaaaaGGGagaggcctgtttggggcgatagcagggtttatagagggaggatggcagggaatggttgatggttggtatgggtaccatcatagcaatgagcaggggagtgggtacgctgcagacaaagaatccacccaaaaggcaatagatggagttaccaataaggtcaactcaatcattgacaaaatgaacactcaatttgaggcagttggaagggagtttaataacttagaaaggaggatagagaatttgaacaagaaaatggaagacggattcctagatgtctggacctataatgctgaacttctagttctcatggaaaacgagaggactctagatttccatgattcaaatgtcaagaacctttacgacaaagtcagactacagcttagggataatgcaaaggagctgggtaatggctgtttcgaattctatcacaaatgcgataatgaatgtatggaaagtgtgagaaatgggacgtatgactaccctcagtattcagaagaagcaagattaaaaagagaagaaataagcggagtgaaattagaatcaataggaacttaccagatactgtcaatttattcaacagcggcgagttccctagcactggcaatcatgatagctggtctatctttatggatgtgctccaatgggtcgttacagtgcagaatttgcatttag

>H5N1_A/avian/Hong Kong/0719/2007

cttgttaaaagtgatcagatttgcattggttaccatgcaaacaactcgacagagcaggttgacacaataatggaaaagaacgttactgttacacatgctcaagatatactggaaaagacacacaacgggaagctctgcgatctagatggagtgaagcctctgattttaagagattgtagtgtagctggatggctcctcggaaacccaatgtgtgacgaattcatcaatgtgccggaatggtcttacatagtggagaaggccaacccagccaatgacctctgttacccagggaatttcaacgactatgaagaactgaaacacctattgagcaggataaaccattttgagaaaattcagatcatccccaaaagttcttggtccgatcatgaagcctcatcaggggtgagctcagcatgtccataccagggaacgccctcctttttcagaaatgtggtatggcttatcaaaaagaacaatacatacccaacaataaagagaagctacaataataccaaccaggaagatcttttgatactgtgggggattcatcattctaatgatgcagcagagcagacaaagctctatcaaaacccaaccacctatatttccgttgggacatcaacactgaaccagagattggtaccaaaaatagctactagatccaaagtaaacgggcaaagtggaaggatggatttcttctggacaattttaaaaccgaatgatgcaatcaacttcgagagtaatggaaatttcattgctccagaatatgcatacaaaattgtcaagaaaggggactcagcaattatgaaaagtgaggtggaatatggtaactgcaacaccaagtgtcaaactccaataggggcgataaactctagtatgccattccacaacatacaccctctcaccatcggggaatgccccaaatatgtgaaatcaaacaaattagtccttgcgactgggctcagaaatagtcctctaagagaaagaagaagaaaaGGGagaggactatttggagctatagcaggttttatagagggcggatggcagggaatggtagatggttggtatgggtttcaccatagcaatgagcaggggagtgggtacgctgcagacaaagaatccactcaaaaggcaatagatggagtcaccaataaggtcaactcgatcattgacaaaatgaacactcagtttgaggccgttggaagggaatttaataacttagagaggagaatagagaatttaaacaagaaaatggaagacggattcctagatgtctggacttataatgctgaacttctggttctcatggaaaatgagagaactctagacttccatgattcaaatgtcaagaacctttacgacaaggtccgactacagcttagggataatgcaaaggagctgggtaacggttgtttcgagttctatcacaaatgtgataatgaatgcatggaaagtgtaagaaacggaacgtatgactacccgcagtattcagaagaagcaagattaaaaagagaggaaataagtggagtaaaattggaatcaataggaacttaccaaatactgtcaatttattcaacagttgcgagttctctagcactggcaatcatggtggctggtctatctttatggatgtgctccaatgggtcgttacaatgcagaatttgcatttga

>H5N1_A/avian/Hong Kong/1993/2007

cttgttaaaagtgatcagatttgcattggttaccatgcaaacaactcgacagagcaggttgacacaataatggaaaagaacgttactgttacacatgctcaagatatactggaaaagacacacaacgggaagctctgcgatctagatggagtgaagcctctgattttaagagattgtagtgtagctggatggctcctcggaaacccaatgtgtgacgaattcatcaatgtgccggaatggtcttacatagtggagaaggccaacccagccaatgacctctgttacccagggaatttcaacgactatgaagaactgaaacacctattgagcaggataaaccattttgagaaaattcagatcatccccaaaagttcttggtccgatcatgaagcctcatcaggggtgagctcagcatgtccataccagggaacgccctcctttttcagaaatgtggtatggcttatcaaaaagaacaatacatacccaacaataaagagaagctacaataataccaaccaggaagatcttttgatactgtgggggattcatcattctaatgatgcagcagagcagacaaagctctatcaaaacccaaacacctatatttccgttgggacatcaacactgaaccagagattggtaccaaaaatagctactagatccaaagtaaacgggcaaagtggaaggatggatttcttctggacaattttaaaaccgaatgatgcaatcaacttcgagagtaatggaaatttcattgctccagaatatgcatacaaaattgtcaagaaaggggactcagcaattatgaaaagtgaggtggaatatggtaactgcaacaccaagtgtcaaactccaataggggcgataaactctagtatgccattccacaacatacaccctctcaccatcggggaatgccccaaatatgtgaaatcaaacaaattagtccttgcgactgggctcagaaatagtcctctaagagaaagaagaagaaaaGGGagaggactatttggagctatagcaggttttatagagggcggatggcagggaatggtagatggttggtatgggtttcaccatagcaatgagcaggggagtgggtacgctgcagacaaagaatccactcaaaaggcaatagatggagtcaccaataaggtcaactcgatcattgacaaaatgaacactcagtttgaggccgttggaagggaatttaataacttagagaggagaatagagaatttaaacaagaaaatggaagacggattcctagatgtctggacttataatgctgaacttctggttctcatggaaaatgagagaactctagacttccatgattcaaatgtcaagaacctttacgacaaggtccgactacagcttagggataatgcaaaggagctgggtaacggttgtttcgagttctatcacaaatgtgataatgaatgcatggaaagtgtaagaaacggaacgtatgactacccgcagtattcagaagaagcaagattaaaaagagaggaaataagtggagtaaaattggaatcaataggaacttaccaaatactgtcaatttattcgacagttgcgagttctctagcactggcaatcatggtggctggtctatctttatggatgtgctccaatgggtcgttacaatgcagaatttgcatttga

>H5N1_A/avian/Hong Kong/2372/2007

cttgttaaaagtgatcagatttgcattggttaccatgcaaacaactcgacagagcaggttgacacaataatggaaaagaacgttactgttacacatgctcaagatatactggaaaagacacacaacgggaagctctgcgatctagatggagtgaagcctctgattttaagagattgtagtgtagctggatggctcctcggaaacccaatgtgtgacgaattcatcaatgtgccggaatggtcttacatagtggagaaggccaacccagccaatgacctctgttacccagggaatttcaacgactatgaagaactgaaacacctattgagcaggataaaccattttgagaaaattcagatcatccccaaaagttcttggtccgatcatgaagcctcatcaggggtgagctcagcatgtccataccagggaacgccctcctttttcagaaatgtggtatggcttatcaaaaagaacaatacatacccaacaataaagagaagctacaataataccaaccaggaagatcttttgatactgtgggggattcatcattctaatgatgcagcagagcagacaaagctctatcaaaacccaaccacctatatttccgttgggacatcaacactgaaccagagattggtaccaaaaatagctactagatccaaagtaaacgggcaaagtggaaggatggatttcttctggacaattttaaaaccgaatgatgcaatcaacttcgagagtaatggaaatttcattgctccagaatatgcatacaaaattgtcaagaaaggggactcagcaattatgaaaagtgaggtggaatatggtaactgcaacaccaagtgtcaaactccaataggggcgataaactctagtatgccattccacaacatacaccctctcaccatcggggaatgccccaaatatgtgaaatcaaacaaattagtccttgcgactgggctcagaaatagtcctctaagagaaagaagaagaaaaGGGagaggactatttggagctatagcaggttttatagagggcggatggcagggaatggtagatggttggtatgggtttcaccatagcaatgagcaggggagtgggtacgctgcagacaaagaatccactcaaaaggcaatagatggagtcaccaataaggtcaactcgatcattgacaaaatgaacactcagtttgaggccgttggaagggaatttaataacttagagaggagaatagagaatttaaacaagaaaatggaagacggattcctagatgtctggacttataatgctgaacttctggttctcatggaaaatgagagaactctagacttccatgattcaaatgtcaagaacctttacgacaaggtccgactacagcttagggataatgcaaaggagctgggtaacggttgtttcgagttctatcacaaatgtgataatgaatgcatggaaagtgtaagaaacggaacgtatgactacccgcagtattcagaagaagcaagattaaaaagagaggaaataagtggagtaaaattggaatcaataggaacttaccaaatactgtcaatttattcgacagttgcgagttctctagcactggcaatcatggtggctggtctatctttatggatgtgctccaatgggtcgttacaatgcagaatttgcatttga

>H5N1_A/bar-headed goose/Mongolia/1/2005

cttgttaaaagtgatcagatttgcattggttaccatgcaaacaactcgacagagcaggttgacacaataatggaaaagaacgtcactgttacacacgcccaagacatactggaaaagacacacaacgggaaactctgcgatctagatggagtgaagcctctaattttaagagattgtagtgtagctggatggctcctcgggaacccaatgtgtgacgaattcctcaatgtgccggaatggtcttacatagtggagaagatcaatccagccaatgacctctgttacccagggaatttcaacgactatgaagaactgaaacacctattgagcagaataaaccattttgagaaaattcagatcatccccaaaagttcttggtcagatcatgaagcctcatcaggggtgagctcagcatgtccataccagggaaggtcctccttttttagaaatgtggtatggcttatcaaaaaggacaatgcatacccaacaataaagagaagttacaataataccaaccaagaagatcttttggtactgtgggggattcaccatccaaatgatgcggcagagcagacaaggctctatcaaaacccaaccacctatatttccgttgggacatcaacactaaaccagagattggtaccaaaaatagctactagatccaagataaacgggcaaagtggaaggatggagttcttttggacaattttaaaaccgaatgatgcaataaactttgagagtaatggaaatttcattgctccagaaaatgcatacaaaattgtcaagaaaggggactcaacaattatgaaaagtgaattggaatatggtaactgcaacaccaagtgtcaaactccaataggggcgataaactctagtatgccattccacaacatccaccctctcaccatcggggaatgccccaaatatgtgaaatcaaacagattagtccttgcgactgggctcagaaatagccctcaaggagagagaagaagaaaaaagagagggctatttggagctatagcaggttttatagagggaggatggcagggaatggtagatggttggtatgggtaccaccatagcaacgagcaggggagtgggtacgctgcagacaaagaatccactcaaaaggcaatatatggagtcaccaatacggtcaactcgatcattgacaaaatgaacactcaatttgaggctgttggaagggaatttaataacttagaaaggagaatagaaaatttaaacaagaagatggaagacggattcctagatgtctggacttataatgctgaacttctggttctcatggaaaatgagagaactctagactttcatgactcaaatgtcaagaacctttacgacaaggtccgactacagcttagggataatgcaaaggaacttggtaacggttgtttcgagttctatcacagatgtgataatgaatgtatggaaagtgtaagaaacggaacgtatgactacccgcagtattcagaagaagcaagattaaaaagagaggaaataagtggagtaaaattggaatcaataggaacttaccaaatactgtcaatttattcaacagtggcgagctccctagcactggcaatcatggtggctggtctatctttatggatgtgctccaatggatcgttacaatgcagaatttgcatttga

>H5N1_A/bar-headed goose/Mongolia/X53/2009

cttgttaaaagcgatcatatttgcattggttatcatgcaaataactcgacagagcaggttgacacaataatggaaaagaacgttactgttacacatgcccaagacatactggaaaagacacacaacgggaagctctgcgatctaaatggagtgaagcctctgattttaaaagattgtagtgtagcgggatggctcctcggaaacccattgtgtgacgaattcatcaatgtgccagaatggtcttacatagtagagaaggccaatccagccaatgacctctgttacccagggaatttcaacgattatgaagaattgaaacacctattgagcaggataaaccattttgagaaaatacagatcatccccaaagattcttggtcagatcatgaagcctcattgggggtgagcgcagcatgttcataccagggaaattcctccttcttcagaaatgtggtatggcttatcaaaaaggacaatgcatacccaacaataaagaaaggctacaataataccaaccaagaagatctcttggtactgtgggggattcaccatcctaatgatgaggcagagcagacaaggctctatcaaaacccaaccacctatatttccattgggacatcaacactaaaccagagattggtaccaaaaatagccactagatccaaaataaacgggcaaagtggcaggatagatttcttctggacaattttaaaaccgaatgatgcaatccacttcgagagtaatggaaatttcattgctccagaatatgcatacaaaattgtcaagaaaggagactccacaattatgaaaagtgaagtggaatatggtaactgcaacaccaggtgtcagactccgataggggcgataaactctagtatgccattccacaacatacaccctctcaccatcggagaatgtcccaaatatgtgaaatcaaacaaattagtccttgcgactgggctcagaaatagtcctcaaagagagagaagaagaaaaGGGagaggactgtttggagctatagcaggttttatagagggaggatggcagggaatggtagatggttggtatgggtaccaccacagcaatgagcaggggagtgggtacgctgcagacaaagaatctactcaaaaggcaatagacggagtcaccaataaggtcaactcgatcattgacaaaatgaacactcagtttgaggccgtaggaagggaatttaataacttagagaggagaatagagaatttaaacaagaagatggaagacggattcctagatgtctggacttataatgctgaacttctggttctcatggaaaatgagagaactctagacttccatgactcaaatgtcaagaacctttacgataaggtcagactacagcttaaggataatgcaaaagagttgggtaacggttgtttcgagttctatcacaaatgtaataatgaatgtatggaaagtgtaagaaacggaacgtatgactacccgcagtattcagaagaagcaagattaaaaagagaggaaataagtggagtaaaattggaatcaataggaatctaccaaatactgtcaatttattcaacagtggcgagttccctagtgctggcaatcatgatggctggtctatctttatggatgtgttccaacgggtcgttacagtgcagaatttgcatttga

>H5N1_A/bar-headed goose/Qinghai/1-HVRI/2006

cttgttaaaagtgatcagatttgcattggttaccatgcaaacaactcgacagagcaggttgacacaataatggaaaagaacgtcactgttacacacgcccaagacatactggaaaagacacacaacgggaagctctgcgatctagatggagtgaagcctctaattttaagagattgtagtgtagctggatggctcctcgggaacccaatgtgtgatgaattcctcaatgtgccggaatggtcttacatagtggagaagatcaatccagccaatgacctctgttacccagggaatttcaacgactatgaagaactgaaacacctattgagcagaataaaccattttgagaaaattcagatcatccccaaaagttcttggtcagatcatgaagcctcatcaggggtgagctcagcatgtccataccagggaaggtcctccttttttagaaatgtggtatggcttatcaaaaaggacaatgcatacccaacaataaagagaagttacaataataccaaccaagaagatcttttggtactgtgggggattcaccatccaaatgatgcggcagagcagacaaagctctatcaaaacccaaccacctatatttccgttgggacatcaacactaaaccagagattgataccaaaaatagctactagatccaaggtaaacgggcaaagtggaaggatggagttcttttggacaattttaaaaccgaatgatgcaataaactttgagagtaatggaaatttcattgctccagaaaatgcatacaaaattgtcaagaaaggggactcaacaattatgaaaagtgaattggaatatggtaactgcaacaccaagtgtcaaactccaataggggcgataaactctagtatgccattccacaacatccaccctctaaccatcggggaatgccccaaatatgtgaaatcaaacagattaatccttgcgactgggctcagaaatagccctcaaggagagagaagaagaaaaaagagaggactatttggagctatagcaggttttatagaaggaggatggcagggaatggtagatggttggtatgggtaccaccatagcaacgagcaggggagtgggtacgctgcagacaaagaatccactcaaaaggcaatagatggagtcaccaataaggtcaactcgatcattgacaaaatgaacactcagtttgaggccgttggaagggaatttaataacttagaaaggagaatagaaaatttaaacaagaagatggaagacggattcctagatgtctggacttataatgctgaacttctggttctcatggaaaatgagagaactctagactttcatgactcaaatgtcaagaacctttacgacaaggtccgactacagcttagggataatgcaaaggagcttggtaacggttgtttcgagttctaccacagatgtgataatgaatgtatggaaagtgtaagaaacggaacgtatgactacccgcagtattcagaagaaacaagactaaaaagagaggaaataagtggagtaaaattggaatcaataggaacctaccaaatactgtcaatttattcaacagtggcgagctccctagcactggcaatcatggtggctggtctatctttatggatgtgctccaatggatcgttacaatgcagaatttgcatttga

>H5N1_A/bar-headed goose/Qinghai/3/2005

cttgttaaaagtgatcagatttgcattggttaccatgcaaacaactcgacagagcaggttgacacaataatggaaaagaacgtcactgttacacacgcccaagacatactggaaaagacacacaacgggaagctctgcgatctagatggagtgaagcctctaattttaagagattgtagtgtagctggatggctcctcgggaacccaatgtgtgacgaattcctcaatgtgccggaatggtcttacatagtggagaagatcaatccagccaatgacctctgttacccagggaatttcaacgactatgaagaactgaaacacctattgagcagaataaaccattttgagaaaattcagatcatccccaaaagttcttggtccaatcatgaagcctcatcaggggtgagctcagcatgtccataccatggaaggtcctcctttttcagaaatgtggtatggcttatcaaaaagaacaatgcatacccaacaataaagagaagttacaataataccaaccaagaagatcttttggtactgtgggggattcaccatccaaatgatgcggcagagcagacaaagctctatcaaaacccaaccacctatatttccgttgggacatcaacactaaaccagagattggtaccaaaaatagctactagatccaaagtaaacgggcaaagtggaaggatggagttcttttggacaattttaaaaccgaatgatgccataaactttgagagtaatggaaatttcattgctccagaaaatgcatacaaaattgtcaagaaaggggactcaacaattatgaaaagtgaattggaatatggtaactgcaacaccaagtgtcaaactccaataggggcgataaactctagtatgccattccacaacatccaccctctcaccatcggggaatgccccaaatatgtgaaatcaaacagattaatccttgcgactgggctcagaaatagccctcaaagagagagaagaagaaaaaagagaggactatttggagctatagcaggttttatagaaggaggatggcagggaatggtagatggttggtatgggtaccaccatagcaacgagcaggggagtgggtacgctgcagacaaagaatccactcaaaaggcaatagatggagtcaccaataaggtcaactcgatcattgacaaaatgaacactcagtttgaggccgttggaagggaatttaataacttagaaaggagaatagagaatttaaacaagaagatggaagacggattcctagatgtctggacttataatgctgaacttctggttctcatggaaaatgagagaactctagactttcatgactcaaatgtcaagaacctttacgacaaggtccgactacagcttagggataatgcaaaggagcttggtaacggttgtttcgagttttatcacagatgtgataatgaatgtatggaaagtgtaagaaacggaacgtatgattacccgcagtattcagaagaagcaagactaaaaagagaggaaataagtggagtaaaattggaatcaataggaacttaccaaatactgtcaatttattcaacagtggcgagctccctagcactggcaatcatggtggctggtctatctttatggatgtgctccaatggatcgttacaatgcagaatttgcatttga

>H5N1_A/bar-headed goose/Tibet/8/2006

cttgttaaaagtgatcagatttgcattggttaccatgcaaacaactcgacagagcaggttgacacaataatggaaaagaacgtcactgttacacacgcccaagacatactggaaaagacacacaacgggaagctctgcgatctagatggagtgaagcctctaattttaagagattgtagtgtagctggatggctcctcgggaacccaatgtgtgacgaattcctcaatgtgccggaatggtcttacatagtggagaagatcaatccagccaatgacctctgttacccagggaatttcaacgactatgaagaactgaaacacctattgagcagaataaaccattttgagaaaattcagatcatccccaaaagttcttggtcagatcatgaagcctcatcaggggtgagctcagcatgtccataccagggaaggtcctccttttttagaaatgtggtatggcttatcaaaaaggacaatgcatacccaacaataaagagaagttacaataataccaaccaagaagatcttttggtactgtgggggattcaccatccaaatgatgcggcagaacagacaaagctctatcaaaacccaaccacctatatttccgttgggacctcaacattaaaccagagattgataccaaaaatagctactagaaccaaggtaaacgggcaaggtggaaggatggatttcttttggacaattttaaaaccgaatgatgcaataaactttgagagtaatggaaatttcattgctccagaaaatgcatacaaaattgtcaagaaaggggactcaacaattatgaaaagtgaattggaatatggtaactgcaacaccaagtgtcaaactccaataggggcgataaactctagtatgccattccacaacatccaccctctaaccatcggggaatgccccaaatatgtgaaatcaaacagattagtccttgcgactgggctcagaaatagccctcaaggagagagaagaagaaaaaagagaggactatttggagctatagcaggttttatagagggaggatggcagggaatggtagatggttggtatgggtaccaccatagcaacgagcaggggagtgggtacgctgcagacaaagaatccactcaaaaggcaatagatggagtcaccaataaggtcaactcgatcattgacaaaatgaacactcagtttgaggccgttggaagggaatttaataacttagaaaggagaatagaaaatttaaacaagaagatggaagacggattcctagatgtctggacttataatgctgaacttctggttctcatggaaaatgagagaactctagactttcatgactcaaatgtcaagaacctttacgacaaggtccgactacagcttagggataatgcaaaggagcttggtaacggttgtttcgagttctatcacagatgtgataatgaatgtatggaaagtgtaagaaacggaacgtatgactacccgcagtattcagaagaagcaagattaaaaagagaggaaataagtggagtaaaattggaatcaataggaacctaccaaatactgtcaatttattcaacagtggcgagctccctagcactggcaatcatggtggctggtctatctttatggatgtgctccaatggatcgttacaatgcagaatttgcatttga

>H5N1_A/barn swallow/Hong Kong/1161/2010

cttgttaaaagcgatcatatttgcattggttaccatgcaaacaactcgacagagcaggttgacacaataatggaaaagaacgttactgttacacatgctcaagacatactggagaagacacacaatgggaagctctgcgatctaaatggagtgaagcctctgattttaaaagattgtagtgtagcaggatggctcctcgggaacccgatgtgtgacgaattcatcgatgtgccagaatggtcttacatagtagagaaggccaatccagccaatgacctctgttacccagggaatttcaacgattatgaagaattaaaacacctattgagcagaataaaccattttgagaaaatacggatcatccccaaagattcttggccagatcatgaagcctcattgggggtgagcgcagcatgttcataccagggaaattcctcctttttcagaaatgtggtatggcttctcaaaaaggacaatgcatacccaataataaagaaaagctacaataataccaataaagaagatctcttggtactgtgggggatccaccatcctaatgatgaggcagagcagacaaggctctatcaaaacccaaccacctatgtttccattgggacatcaacactaaaccagagattggtaccaagaatagctactagatccaaagtaaacgggcaaagtggaaggatagatttcttctggacaattttaaaaccgaatgatgcaatcaacttcgagagtaatggaaatttcattgctccagaatatgcatacaaaatagtcaagaaaggagactcaacaattatgagaagtgaagtggaatatggtaactgcagcaccaggtgtcagactccgatgggggcgataaattctagtatgccattccacaacatacaccctctcaccatcggagaatgtcccaaatatgtgaaatcaaacaaattagtccttgcgactgggctcagaaatagtcctcaaatagaaagaagaagaaggaaaagggggctgtttggagctatagcaggttttatagagggaggatggcagggaatggtagatggttggtatgggtaccaccacagcaatgagcaggggagtggatatgctgcagacaaagaatccactcaaaaggcaatagatggggtcaccaataaagtcaactcgatcattgacaaaatgaacactcagtttgaggcagtaggaagggaatttaataacttagagaggagaatagagaatttaaacaagaagatggaagacggattcctagatgtctggacttataatgctgaactcctggttctaatggaaaatgagagaactctagacttccatgactcaaatgtcaagaacctttacgacaaggtcagactacagcttagggataatgcaaaagagctgggtaacggttgtttcgagttctatcacaaatgtgataatgaatgtatggaaagtgtaagaaacggaacgtatgactacccgcagtattcagaagaagcaagattaaaaagagaggaaataagtggagtaaaattggaatcaataggaacttaccaaatattgtcaatttattcaacagtggcgagttccctagtactggcaatcatggtggctggtctatctttatggatgtgttccaacgggtcgttacaatgcagaatttgcatttga

>H5N1_A/barnacle/goose_Denmark/19027-1.02/2021-04-18

cttgttaaaagtgatcagatttgcattggttaccatgcaaacaattcgacagagcaagttgacacgataatggaaaagaacgtcactgttacacatgcccaagacatactggaaaaaacacacaacgggaagctctgtgatctaaatggggtgaagcctctgattttaaaggattgtagtgtagctggatggctcctcggaaacccaatgtgcgacgaattcatcagagtgccggaatggtcctacatagtggagcgaactaatccagctaatgacctctgttacccagggagcctcaatgactatgaagaactgaaacacctgttgagcagaataaatcattttgagaagattctgatcatccccaagagttcctggccaaatcatgaaacatcactaggggtgagcgcagcttgtccataccagggagcgccctcctttttcagaaatgtggtgtggcttatcaaaaagaacgatgcatacccaacaataaagataagctacaataataccaatcgggaagatctcttgatactgtgggggattcatcattccaacaatgcagaagagcagacaaatctctacaaaaacccaaccacctacatttcagttggaacatcaactttaaaccagaggttggtaccaaaaatagctactagatcccaagtaaatgggcaacgtggaagaatggacttcttctggacaattttaaaaccagatgatgcaatccatttcgagagtaatggaaatttcattgctccagaatatgcatacaaaattgtcaagaaaggggactcaacaattatgaaaagtggagtggaatatggccactgcaacaccaaatgtcaaaccccagtaggagcgataaattctagtatgccattccacaacatacatcctctcaccattggggaatgccccaaatacgtgaagtcaaacaagttggtccttgcgactgggctcagaaatagtcctctaagagaaaagagaagaaaaGGGagaggcctgtttggggcgatagcagggtttatagagggaggatggcagggaatggttgatggttggtatgggtaccatcatagcaatgagcaggggagtgggtacgctgcagacaaagaatccacccaaaaggcaatagatggagttaccaataaggtcaactcaatcattgacaaaatgaacactcaatttgaggcagttggaagggagtttaataacttagaaaggaggatagagaatttgaacaagaaaatggaagacggattcctagatgtctggacctataatgctgaacttctagttctcatggaaaacgagaggactctagatttccatgattcaaatgtcaagaacctttacgacaaagtcagactacagcttagggataatgcaaaggagctgggtaacggctgtttcgaattctatcacaaatgcgataatgaatgtatggaaagtgtgagaaatgggacgtatgactaccctcagtattcagaagaagcaagattaaaaagagaagaaataagcggagtgaaattagaatcaataggaacttaccagatactgtcaatttattcaacagcggcgagttccctagcactggcaatcatgatggctggtctatctttatggatgtgctccaatgggtcgttacagtgcagaatttgcatttag

>H5N1_A/barnacle_goose/Denmark/17572-1.01/2021-03-01

cttgttaaaagtgatcagatttgcattggttaccatgcaaacaattcgacagagcaagttgacacgataatggaaaagaacgtcactgttacacatgcccaagacatactggaaaaaacacacaacgggaagctctgtgatctaaatggggtgaagcctctgattttaaaggattgtagtgtagctggatggctcctcggaaacccaatgtgcgacgaattcatcagagtgccggaatggtcctacatagtggagcggactaatccagctaatgacctctgttacccagggagcctcaatgactatgaagaactgaaacacctgttgagcagaataaatcattttgagaagattctgatcatccccaagagttcctggccaaatcatgaaacatcactaggggtgagcgcagcttgtccataccagggagcgccctcctttttcagaaatgtggtgtggcttatcaaaaagaacgatgcatacccaacaataaagataagctacaataataccaatcgggaagatctcttgatactgtgggggattcatcattccaacaatgcagaagagcagacaaatctctacaaaaacccaaccacctacatttcagttggaacatcaactttaaaccagaggttggtaccaaaaatagctactagatcccaagtaaacgggcaacgtggaagaatggacttcttctggacaattttaaaaccagatgatgcaatccatttcgagagtaatggaaatttcattgctccagaatatgcatacaaaattgtcaagaaaggggactcaacaattatgaaaagtggagtggaatatggccactgcaacaccaaatgtcaaaccccagtaggagcgataaattctagtatgccattccacaacatacatcctctcaccattggggaatgccccaaatacgtgaagtcaaacaagttggtccttgcgactgggctcagaaatagtcctctaagagaaaagagaagaaaaGGGagaggcctgtttggggcgatagcagggtttatagagggaggatggcagggaatggttgatggttggtatgggtaccatcatagcaatgagcaggggagtgggtacgctgcagacaaagaatccacccaaaaggcaatagatggagttaccaataaggtcaactcaatcattgacaaaatgaacactcaatttgaggcagttggaagggagtttaataacttagaaaggaggatagagaatttgaacaagaaaatggaagacggattcctagatgtctggacctataatgctgaacttctagttctcatggaaaacgagaggactctagatttccatgattcaaatgtcaagaacctttacgacaaagtcagactacagcttagggataatgcaaaggaactgggtaacggctgtttcgaattctatcacaaatgcgataatgaatgtatggaaagtgtgagaaatgggacgtatgactaccctcagtattcagaagaagcaagattaaaaagagaagaaataagcggagtgaaattagaatcaataggaacttaccagatactgtcaatttattcagcagcggcgagttccctagcactggcaatcatgatggctggtctatctttatggatgtgctccaatgggtcgttacagtgcagaatttgcatttag

>H5N1_A/barnacle_goose/Denmark/24273-1.02/2021-10-26

cttgttaaaagtgatcagatttgcattggttaccatgcaaacaattcgacagagcaggttgacacgataatggaaaagaacgtcactgttacacatgcccaagacatactggaaaaaacacacaacgggaagctctgtgatttaaatggggtgaagcctctgattttaaaggattgtagtgtagctggatggctcctcggaaacccaatgtgcgacgaattcatcagagtgccggaatggtcctacatagtggagcgggctaatccagccaatgacctctgttacccagggagcctcaatgactatgaagaactgaaacacctgttgagcagaataaatcattttgagaagattctgatcatccccaagagttcctggccaaatcatgaaacatcactaggggtgagcgcagcttgtccataccagggagcgccctcctttttcagaaatgtgttgtggcttatcaaaaagaacgatgcatacccaacaataaagataagttacaataataccaatcgggaagatctcttgatactgtgggggattcatcattccaacaatgcagaagagcagacaaatctctataaaaacccaaccacctacatttcagttggaacatcaactttaaaccagaggttggtaccaaaaatagctactagatcccaagtaaacgggcaacgtggaagaatggacttcttctggacaattttaaaaccagatgatgcaatccatttcgagagtaatggaaatttcattgcaccagaatatgcatataaaattgtcaagaaaggggactcaacaattatgaaaagtggagtggaatatggccactgcaacaccaaatgtcaaaccccagtaggagcgataaattctagtatgccattccacaacatacatcctctcaccattggggaatgccccaaatacgtgaagtcaaacaagttggtccttgcgactgggctcagaaatagtcctctaagagaaaagagaagaaaaGGGagaggcctgtttggggcgatagcagggtttatagagggaggatggcagggaatggttgatggttggtatgggtaccatcatagcaatgagcaggggagtgggtacgctgcagacaaagaatccacccaaaaggcaatagatggagttaccaataaggtcaactcaatcattgacaaaatgaacactcaatttgaggcagttggaagggagtttaataacttagaaaggaggatagagaatttgaacaagaaaatggaagacggattcctagatgtctggacctatagtgctgaacttctagttctcatggaaaacgagaggactctagatttccatgattcaaatgtcaagaacctttacgacaaagtcagactacagcttagggacaatgcaaaggagctgggtaatggctgtttcgaattctatcacaaatgcgataatgaatgtatggaaagtgtgagaaatgggacgtatgactaccctcagtattcagaagaagcaagattaaaaagagaagaaataagcggagtgaaattagaatcaataggaacttaccagatactgtcaatttattcaacagcggcgagttccctagcactggcaatcatgatagctggtctatctttatggatgtgctccaatgggtcgttacagtgcagaatttgcatttag

>H5N1_A/barnacle_goose/Denmark/24342-1.02/2021-10-30

cttgttaaaagtgatcagatttgcattggttaccatgcaaacaattcgacagagcaagttgacacgataatggaaaagaacgtaactgttacacatgcccaagacatactggaaaaaacacacaacgggaagctctgtgatctaaatggggtgaagcctctgattttaaaggattgtagtgtagctggatggctcctcggaaacccaatgtgcgacgaattcatcagagtgccggaatggtcctacatagtggagcgggctaatccagctaatgacctctgttacccagggagcctcaatgactatgaagaactgaaacacctgttgagcagaataaatcattttgagaagattctgatcatccccaagagttcctggccaaatcatgaaacatcactaggggtgagcgcagcttgtccataccagggagcgccctcctttttcagaaatgtggtgtggcttatcaaaaagaacgatgcatacccaacaataaagataagctacaataataccaatcgggaagatctcttgatactgtgggggattcatcattccaacaatgcagaagagcagacaaatctctacaaaaacccaaccacctacatttcagttggaacatcaactttaaaccagaggttggtaccaaaaatagctactagatcccaagtaaacgggcaacgtggacgaatggacttcttctggacaattttaaaaccagatgatgcaatccatttcgagagtaatggaaatttcattgctccagaatatgcatacaaaattgtcaagaaaggggactcaacaattatgaaaagtggagtggaatatggccactgcaacaccaaatgtcaaaccccagtaggagcgataaattctagtatgccattccacaacatacatcctctcaccattggggaatgccccaaatacgtgaagtcaaacaagttggtccttgcgactgggcttagaaatagtcctctaagagaaaagagaagaaaaGGGagaggcctgtttggggcgatagcagggtttatagagggaggatggcagggaatggttgatggttggtatgggtaccatcatagcaatgagcaggggagtgggtacgctgcagacaaagaatccacccaaaaggcaatagatggagttaccaataaggtcaactcaatcattgacaaaatgaacactcaatttgaggcagttggaagggagtttaataacttagaaaggaggatagagaatttgaacaagaaaatggaagacggattcctagatgtctggacctataatgctgaacttctagttctcatggaaaacgagaggactctagatttccatgattcaaatgtcaagaacctttacgacaaagtcagactacagcttagggataatgcaaaggagctgggtaacggctgtttcgaattctatcacaaatgcgataatgaatgtatggaaagtgtgagaaatgggacgtatgactaccctcagtattcagaagaagcaagattaaaaagagaagaaataagcggagtgaaattagaatcaataggaacttaccagatactgtcaatttattcaacagcggcaagttccctagcactggcaatcatgatggctggtctatctttatggatgtgctccaatgggtcgttacagtgcagaatttgcatttag

>H5N1_A/barnacle_goose/Finland//6378_21VIR7689-7/2021

cttgttaaaagtgatcagatttgcattggttaccatgcaaacaattcgacagagcaagttgacacgataatggaaaagaacgtcactgttacacatgcccaagacatactggaaaaaacacacaacgggaagctctgtgatctaaatggggtgaagcctctgattttaaaggattgtagtgtagctggatggctcctcggaaacccaatgtgcgacgaattcatcagagtgccggaatggtcctacatagtggagcgggctaatccagctaatgacctctgttacccagggagcctcaatgactatgaagaactgaaacacctgttgagcagaataaatcattttgagaagattctgatcatccccaagagttcttggccaaatcatgaaacatcactaggggtgagcgcagcttgtccataccagggagcgccctcctttttcagaaatgtggtgtggcttatcaaaaagaacgatgcatacccaacaataaagataagctacaataataccaatcgggaagatctcttgatactgtgggggattcatcattccaacaatgcagaagagcagacaaatctctacaaaaacccaaccacctacatttcagttggaacatcaactttaaaccagaggttggtaccaaaaatagctactagatcccaagtaaacgggcaacgtggaagaatggacttcttctggacaattttaaaaccagatgatgcaatccatttcgagagtaatggaaatttcattgctccagaatatgcatacaaaattgtcaagaaaggggactcaacaattatgaaaagtggagtggaatatggccactgcaacaccaaatgtcaaaccccagtaggagcgataaattctagtatgccattccacaacatacatcctctcaccattggggaatgccccaaatacgtgaagtcaaacaagttggtccttgcgactgggcttagaaatagtcctctaagagaaaagagaagaaaaGGGagaggcctgtttggggcgatagcagggtttatagagggaggatggcagggaatggttgatggttggtatgggtaccatcatagcaatgagcaggggagtgggtacgctgcagacaaagaatccacccaaaaggcaatagatggagttaccaataaggtcaactcaatcattgacaaaatgaacactcaatttgaggcagttggaagggagtttaataacttagaaaggagaatagagaatttgaacaagaaaatggaagacggattcctagatgtctggacctataatgctgaacttctagttctcatggaaaacgagaggactctagatttccatgattcaaatgtcaagaacctttacgacaaagtcagactacagcttagggataatgcaaaggagctgggtaacggctgtttcgaattctatcacaaatgcgataatgaatgtatggaaagtgtgagaaatgggacgtatgactaccctcagtattcagaagaagcaagattaaaaagagaagaaataagcggagtgaaattagaatcaataggaacttaccagatactgtcaatttattcaacagcggcaagttccctagcactggcaatcatgatggctggtctatctttatggatgtgctccaatgggtcgttacagtgcagaatttgcatttag

>H5N1_A/barnacle_goose/Finland/6247_21VIR7689-6/2021

cttgttaaaagtgatcagatttgcattggttaccatgcaaacaattcgacagagcaagttgacacgataatggaaaagaacgtcactgttacacatgcccaagacatactggaaaaaacacacaacgggaagctctgtgatctaaatggggtgaagcctctgattttaaaggattgtagtgtagctggatggctcctcggaaacccaatgtgcgacgaattcatcagagtgccggaatggtcctacatagtggagcgggctaatccagctaatgacctctgttacccagggagcctcaatgactatgaagaactgaaacacctgttgagcagaataaatcattttgagaagattctgatcatccccaagagttcctggccaaatcatgaaacatcactaggggtgagcgcagcttgtccataccaaggagcgccctcctttttcagaaatgtggtgtggcttatcaaaaagaacgatgcatacccaacaataaagataagctacaataataccaatcgggaagatctcttgatactgtgggggattcatcattccaacaatgcagaagagcagacaaatctctacaaaaacccaaccacctacatttcagttggaacatcaactttaaaccagaggttggtaccaaaaatagctactagatcccaagtaaacgggcaacgtggaagaatggacttcttctggacaattttaaaaccagatgatgcaatccatttcgagagtaatggaaatttcattgctccagaatatgcatacaaaattgtcaagaaaggggactcaacaattatgaaaagtggagtggaatatggccactgcaacaccaaatgtcaaaccccagtaggagcgataaattctagtatgccattccacaacatacatcctctcaccattggggaatgccccaaatacgtgaagtcaaacaagttggtccttgcgactgggcttagaaatagtcctctaagagaaaagagaagaaaaGGGagaggcctgtttggggcgatagcagggtttatagagggaggatggcagggaatggttgatggttggtatgggtaccatcatagcaatgagcaggggagtgggtacgctgcagacaaagaatccacccaaaaggcaatagatggagttaccaataaggtcaactcaatcattgacaaaatgaacactcaatttgaggcagttggaagggagtttaataacttagaaaggaggatagagaatttgaacaagaaaatggaagacggattcctagatgtctggacctataatgctgaacttctagttctcatggaaaacgagaggactctagatttccatgattcaaatgtcaagaacctttacgacaaagtcagactacagcttagggataatgcaaaggagctgggtaacggctgtttcgaattctatcacaaatgcgataatgaatgtatggaaagtgtgagaaatgggacgtatgactaccctcagtattcagaagaagcaagattaaaaagagaagaaataagcggagtgaaattagaatcaataggaacttaccagatactgtcaatttattcaacagcggcaagttccctagcactggcaatcatgatggctggtctatctttatggatgtgctccaatgggtcgttacagtgcagaatttgcatttag

>H5N1_A/barnacle_goose/Finland/6955_21VIR7689-9/2021

cttgttaaaagtgatcagatttgcattggttaccatgcaaacaattcgacagagcaagttgacacgataatggaaaagaacgtcactgttacacatgcccaagacatactggaaaaaacacacaacgggaagctctgtgatctaaatggggtgaagcctctgattttaaaggattgtagtgtagctggatggctcctcggaaacccaatgtgcgacgaattcatcagagtgccggaatggtcctacatagtggagcgggctaatccagctaatgacctctgttacccagggagcctcaatgactatgaagaactgaaacacctgttgagcagaataaatcattttgagaagattctgatcatccccaagagttcctggccaaatcatgaaacatcactaggggtgagcgcagcttgtccataccagggagcgccctcctttttcagaaatgtggtgtggcttatcaaaaagaacgatgcatacccaacaataaagataagctacaataataccaatcgggaagatctcttgatactgtgggggattcatcattccaacaatgcagaagagcagacaaatctctacaaaaacccaaccacctacatttcagttggaacatcaactttaaaccagaggttggcaccaaaaatagctactagatcccaagtaaacgggcaacgtggaagaatggacttcttctggacaatcttaaaaccagatgatgcaatccatttcgagagtaatggaaatttcattgctccagaatatgcatacaaaattgtcaagaaaggggactcaacaattatgaaaagtggagtggaatatggccactgcaacaccaaatgtcaaaccccagtaggagcgataaattctagtatgccattccacaacatacatcctctcaccattggggaatgccccaaatacgtgaagtcaaacaagttggtccttgcgactgggctcagaaatagtcctctaagagaaaggagaagaaaaGGGagaggcctgtttggggcgatagcagggtttatagagggaggatggcagggaatggttgatggttggtatgggtaccatcatagcaatgagcaggggagtgggtacgctgcagacaaagaatccacccaaaaggcaatagatggagttaccaataaggtcaactcaatcattgacaaaatgaacactcaatttgaggcagttggaagggagtttaataacttagaaaggaggatagagaatttgaacaagaaaatggaagacggattcctagatgtctggacctataatgctgaacttctagttctcatggaaaacgagaggactctagatttccatgattcaaatgtcaagaacctttacgacaaagtcagactacagcttagggataatgcaaaggagctgggtaacggctgtttcgaattctatcacaaatgtgataatgaatgtatggaaagtgtgagaaatgggacgtatgactaccctcagtattcagaagaagcaagattaaaaagagaagaaataagcggagtgaaattagaatcaataggaacttaccagatactgtcaatttattcaacagcggcaagttccctagcactggcaatcatgatggctggtctatctttatggatgtgctccaatgggtcgttacagtgcagaatttgcatttag

>H5N1_A/barnacle_goose/Germany-NI/AI01605/2021

cttgttaaaagtgatcagatttgcattggttaccatgcaaacaattcgacagagcaagttgacacgataatggaaaagaacgtcactgttacacatgcccaagacatactggaaaaaacacacaacgggaagctctgtgatctaaatggggtgaagcctctgattttaaaggattgtagtgtagctggatggctcctcggaaacccaatgtgcgacgaattcatcagagtgccggaatggtcctacatagtggagcgggctaatccagctaatgacctctgttacccagggagcctcaatgactatgaagaactgaaacacctgttgagcagaataaatcattttgagaagattctgatcatccccaagagttcctggccaaatcatgaaacatcactaggggtgagcgcagcttgtccataccagggagcgccctcctttttcagaaatgtggtgtggcttatcaaaaagaacgatgcatacccaacaataaagataagctacaataataccaatcgggaagatctcttgatactgtgggggattcatcattccaacaatgcagaagagcagacaaatctctacaaaaacccaaccacctacatttcagttggaacatcaactttaaaccagaggttggcaccaaaaatagctactagatcccaagtaaacgggcaacgtggaagaatggacttcttctggacaattttaaaaccagatgatgcaatccatttcgagagtaatggaaatttcattgctccagaatatgcatacaaaattgtcaagaaaggggactcaacaattatgaaaagtggagtggaatatggccactgcaacaccaaatgtcaaaccccagtaggagcgataaattctagtatgccattccacaacatacatcctctcaccattggggaatgccccaaatacgtgaagtcaaacaagttggtccttgcgactgggctcagaaatagtcctctaagagaaaagagaagaaaaGGGagaggcctgtttggggcgatagcagggtttatagagggaggatggcagggaatggttgatggttggtatgggtaccatcatagcaatgagcaggggagtgggtacgctgcagacaaagaatccacccaaaaggcaatagatggagttaccaataaggtcaactcaatcattgacaaaatgaacactcaatttgaggcagttggaagggagtttaataacttagaaaggaggatagagaatttgaacaagaaaatggaagacggattcctagatgtctggacctataatgctgaacttctagttctcatggaaaacgagaggactctagatttccatgattcaaatgtcaagaacctttacgacaaagtcagactacagcttagggataatgcaaaggagctgggtaacggctgtttcgaattctatcacaaatgcgataatgaatgtatggaaagtgtgagaaatgggacgtatgactaccctcagtattcagaagaagcaagattaaaaagagaagaaataagcggagtgaaattagaatcaataggaacttaccagatactgtcaatttattcaacagcggcaagttccctagcactggcaatcatgatggctggtctatctttatggatgtgctccaatgggtcgttacagtgcagaatttgcatttag

>H5N1_A/barnacle_goose/Germany-NI/AI03914/2021

cttgttaaaagtgatcagatttgcattggttaccatgcaaacaattcgacagagcaagttgacacgataatggaaaagaacgtcactgttacacatgcccaagacatactggaaaaaacacacaacgggaagctctgtgatctaaatggggtgaagcctctgattttaaaggattgtagtgtagctggatggctcctcggaaacccaatgtgcgacgaattcatcagagtgccggaatggtcctacatagtggagcggactaatccagctaatgacctctgttacccagggagcctcaatgactatgaagaactgaaacacctgttgagcagaataaatcattttgagaagattctgatcatccccaagagttcctggccaaatcatgaaacatcactaggggtgagcgcagcttgtccataccagggagcgccctcctttttcagaaatgtggtgtggcttatcaaaaagaacgatgcatacccaacaataaagataagctacaataataccaatcgggaagatctcttgatactgtgggggattcatcattccaacaatgcagaagagcagacaaatctctacaaaaacccaaccacctacatttcagttggaacatcaactttaaaccagaggttggtaccaaaaatagctactagatcccaagtaaacgggcaacgtggaagaatggacttcttctggacaattttaaaaccagatgatgcaatccatttcgagagtaatggaaatttcattgctccagaatatgcatacaaaattgtcaagaaaggggactcaacaattatgaaaagtggagtggaatatggccactgcaacaccaaatgtcaaaccccagtaggagcgataaattctagtatgccattccacaacatacatcctctcaccattggggaatgccccaaatacgtgaagtcaaacaagttggtccttgcgactgggctcagaaatagtcctctaagagaaaagagaagaaaaGGGagaggcctgtttggggcgatagcagggtttatagagggaggatggcagggaatggttgatggttggtatgggtaccatcatagcaatgagcaggggagtgggtacgctgcagacaaagaatccacccaaaaggcaatagatggagttaccaataaggtcaactcaatcattgacaaaatgaacactcaatttgaggcagttggaagggagtttaataacttagaaaggaggatagagaatttgaacaagaaaatggaagacggattcctagatgtctggacctataatgctgaacttctagttctcatggaaaacgagaggactctagatttccatgattcaaatgtcaagaacctttacgacaaagtcagactacagcttagggataatgcaaaggagctgggtaacggctgtttcgaattctatcacaaatgcgataatgaatgtatggaaagtgtgagaaatgggacgtatgactacccccagtattcagaagaagcaagattaaaaagagaagaaataagcggagtgaaattagaatcaataggaacttaccagatactgtcaatttattcaacagcggcgagttccctagcactggcaatcatgatggctggtctatctttatggatgtgctccaatgggtcgttacagtgcagaatttgcatttag

>H5N1_A/barnacle_goose/Germany-NI/AI03980/2021

cttgttaaaagtgatcagatttgcattggttaccatgcaaacaattcgacagagcaagttgacacgataatggaaaagaacgtcactgttacacatgcccaagacatactggaaaaaacacacaacgggaagctctgtgatctaaatggggtgaagcctctgattttaaaggattgtagtgtagctggatggctcctcggaaacccaatgtgcgacgaattcatcagagtgccggaatggtcctacatagtggagcggactaatccagctaatgacctctgttacccagggagcctcaatgactatgaagaactgaaacacctgttgagcagaataaatcattttgagaagattctgatcatccccaagagttcctggccaaatcatgaaacatcactaggggtgagcgcagcttgtccataccagggagcgccctcctttttcagaaatgtggtgtggcttatcaaaaagaacgatgcatacccaacaataaagataagctacaataataccaatcgggaagatctcttgatactgtgggggattcatcattccaacaatgcagaagagcagacaaatctctacaaaaacccaaccacctacatttcagttggaacatcaactttaaaccagaggttggtaccaaaaatagctactagatcccaagtaaacgggcaacgtggaagaatggacttcttctggacaattttaaaaccagatgatgcaatccatttcgagagtaatggaaatttcattgctccagaatatgcatacaaaattgtcaagaaaggggactcaacaattatgaaaagtggagtggaatatggccactgcaacaccaaatgtcaaaccccagtaggagcgataaattctagtatgccattccacaacatacatcctctcaccattggggaatgccccaaatacgtgaagtcaaacaagttggtccttgcgactgggctcagaaatagtcctctaagagaaaagagaagaaaaGGGagaggcctgtttggggcgatagcagggtttatagagggaggatggcagggaatggttgatggttggtatgggtaccatcatagcaatgagcaggggagtgggtacgctgcagacaaagaatccacccaaaaggcaatagatggagttaccaataaggtcaactcaatcattgacaaaatgaacactcaatttgaggcagttggaagggagtttaataacttagaaaggaggatagagaatttgaacaagaaaatggaagacggattcctagatgtctggacctataatgctgaacttctagttctcatggaaaacgagaggactctagatttccatgattcaaatgtcaagaacctttacgacaaagtcagactacagcttagggataatgcaaaggaactgggtaacggctgtttcgaattctatcacaaatgcgataatgaatgtatggaaagtgtgagaaatgggacgtatgactaccctcagtattcagaagaagcaagattaaaaagagaagaaataagcggagtgaaattagaatcaataggaacttaccagatactgtcaatttattcaacagcggcgagttccctagcactggcaatcatgatggctggtctatctttatggatgtgctccaatgggtcgttacagtgcagaatttgcatttag

>H5N1_A/barnacle_goose/Netherlands/21021591-001/2021

cttgttaaaagtgatcagatttgcattggttaccatgcaaacaattcgacagagcaagttgacacgataatggaaaagaacgtcactgttacacatgcccaagacatactggaaaaaacacacaacgggaagctctgtgatctaaatggggtgaagcctctgattttaaaggattgtagtgtagctggatggctcctcggaaacccaatgtgcgacgaattcatcagagtgccggaatggtcctacatagtggagcgggctaatccagctaatgacctctgttacccagggagcctcaatgactatgaagaactgaaacacctgttgagcagaataaatcattttgagaagattctgatcatccccaagagttcctggccaaatcatgaaacatcactaggggtgagcgcagcttgtccataccagggagcgccctcctttttcagaaatgtggtgtggcttatcaaaaagaacgatgcatacccaacaataaagataagctacaataataccaatcgggaagatctcttgatactgtgggggattcatcattccaacaatgcagaagagcagacaaacctctacaaaaacccaaccacctacatttcagttggaacatcaactttaaaccagaggttggtaccaaaaatagctactagatcccaagtaaacgggcaacgtggaagaatggacttcttctggacaattttaaaaccagatgatgcaatccatttcgagagtaatggaaatttcattgctccagaatatgcatacaaaattgtcaagaaaggggactcaacaattatgaaaagtggagtggaatatggccactgcaacaccaaatgtcaaaccccagtaggagcgataaattctagtatgccattccacaacatacatcctctcaccattggggaatgccccaaatacgtgaagtcaaacaagttggtccttgcgactgggctcagaaatagtcctctaagagaaaagagaagaaaaGGGagaggcctgtttggggcgatagcagggtttatagagggaggatggcagggaatggttgatggttggtatgggtaccatcatagcaatgagcaggggagtgggtacgctgcagacaaagaatccacccaaaaggcaatagatggagttaccaataaggtcaactcaatcattgacaaaatgaacactcaatttgaggcagttggaagggagtttaacaacttagaaaggaggatagagaatttgaacaagaaaatggaagacggattcctagatgtctggacctataatgctgaacttctagttctcatggaaaacgagaggactctagatttccatgattcaaatgtcaagaacctttacgacaaagtcagactacagcttagggataatgcaaaggagctgggtaacggctgtttcgaattctatcacaaatgcgataatgaatgtatggaaagtgtgagaaatgggacgtatgactaccctcagtattcagaagaagcaagattaaaaagagaagaaataagcggagtgaaattagaatcaataggaacttaccagatactgtcaatttattcaacagcggcgagttccctagcactggcaatcatgatggctggtctatctttatggatgtgctccaatgggtcgttacagtgcagaatttgcatttag

>H5N1_A/barnacle_goose/Netherlands/21022611-001/2021

cttgttaaaagtgatcagatttgcattggttaccatgcaaacaattcgacagagcaagttgacacgataatggaaaagaacgtcactgttacacatgcccaagacatactggaaaaaacacacaacgggaagctctgtgatctaaatggggtgaagcctctgattttaaaggattgtagtgtagctggatggctcctcggaaacccaatgtgcgacgaattcatcagagtgccggaatggtcctacatagtggagcgggctaatccagctaatgacctctgttacccagggagcctcaatgactatgaagaactgaaacacctgttgagcagaataaatcattttgagaagattctgatcatccccaagagttcctggccaaatcatgaaacatcactaggggtgagcgcagcttgtccataccagggagcgccctcctttttcagaaatgtggtgtggcttatcaaaaagaacgatgcatacccaacaataaagataagctacaataataccaatcgggaagatctcttgatactgtgggggattcatcattccaacaatgcagaagagcagacaaatctctacaaaaacccaaccacctacatttcagttggaacatcaactttaaaccagaggttggtaccaaaaatggctactagatcccaagtaaacgggcaacgtggaagaatggacttcttctggacaattttaaaaccagatgatgcaatccatttcgagagtaatggaaatttcattgctccagaatatgcatacaaaattgtcaagaaaggggactcaacaattatgaaaagtggagtggaatatggccactgcaacaccaaatgtcaaaccccagtaggagcgataaattctagtatgccattccacaacatacatcctctcaccattggggaatgccccaaatacgtgaagtcaaacaagttggtccttgcgactgggctcagaaatagtcctctaagagaaaagagaagaaaaGGGagaggcctgtttggggcgatagcagggtttatagagggaggatggcagggaatggttgatggttggtatgggtaccatcatagcaatgagcaggggagtgggtacgctgcagacaaagaatccacccaaaaggcaatagatggagttaccaataaggtcaactcaatcattgacaaaatgaacactcaatttgaggcagttggaagggagtttaataacttagaaaggaggatagagaatttgaacaagaaaatggaagacggattcctagatgtctggacctataatgctgaacttctagttctcatggaaaacgagaggactctagatttccatgattcaaatgtcaagaacctttacgacaaagtcagactacagcttagggataatgcaaaggagctgggtaacggctgtttcgaattctatcacaaatgcgataatgaatgtatggaaagtgtgagaaatgggacgtatgactaccctcagtattcagaagaagcaagattaaaaagagaagaaataagcggagtgaaattagaatcaataggaacttaccagatactgtcaatttattcaacagcggcgagttccctagcactggcaatcatgatggctggtctatctttatggatgtgctccaatgggtcgttacagtgcagaatttgcatttag

>H5N1_A/barnacle_goose/Netherlands/21024066-001/2021

cttgttaaaagtgatcagatttgcattggttaccatgcaaacaattcgacagagcaagttgacacgataatggaaaagaacgtcactgttacacatgcccaagacatactggaaaaaacacacaacgggaagctctgtgatctaaatggggtgaagcctctgattttaaaggattgtagtgtagctggatggctcctcggaaacccaatgtgcgacgaattcatcagagtgccggaatggtcctacatagtggagcgggctaatccagctaatgacctctgttacccagggagcctcaatgactatgaagagctgaaacacctgttgagcagaataaatcattttgagaagattctgatcatccccaagagttcctggccaaatcatgaaacatcactaggggtgagcgcagcttgtccataccagggagcgccctcctttttcagaaatgtggtgtggcttatcaaaaagaacgatgcatacccaacaataaagataagctacaataataccaatcgggaagatctcttgatactgtgggggattcatcattctaacaatgcagaagagcagacaaatctctacaaaaacccaaccacctacatttcagttggaacatcaactttaaaccagaggttggtaccaaaaatagctactagatcccaagtaaacgggcaacgtggaagaatggacttcttctggacaattttaaaaccagatgatgcaatccatttcgagagtaatggaaatttcattgctccagaatatgcatacaaaattgtaaagaaaggggactcaacaattatgaaaagtggagtggaatatggccactgcaacaccaaatgtcaaaccccagtaggagcgataaattctagtatgccattccacaacatacatcctctcaccattggggaatgccccaaatacgtgaagtcaaacaagttggtccttgcgactgggctcagaaatagtcctctaagagaaaagagaagaaaaGGGagaggcctgtttggggcgatagcagggtttatagagggaggatggcagggaatggttgatggttggtatgggtaccatcatagcaatgagcaggggagtgggtacgctgcagacaaagaatccacccaaaaggcaatagatggagttaccaataaggtcaactcaatcattgacaaaatgaacactcaatttgaggcagttggaagggagtttaataacttagaaaggaggatagagaatttgaacaagaaaatggaagacggattcctagatgtctggacctataatgctgaacttctagttctcatggaaaacgagaggactctagatttccatgattcaaatgtcaagaacctttacgacaaagtcagactacagcttagggataatgcaaaggagctgggtaacggctgtttcgaattctatcacaaatgcgataatgaatgtatggaaagtgtgagaaatgggacgtatgactaccctcagtattcagaagaagcaagattaaaaagagaagaaataagcggagtgaaattagaatcaataggaacttaccagatactgtcaatttattcaacagcggcgagttccctagcactggcaatcatgatggctggtctatctttatggatgtgctccaatgggtcgttacagtgcagaatttgcatttag

>H5N1_A/barnacle_goose/Netherlands/21025769-002/2021

cttgttaaaagtgatcagatttgcattggttaccatgcaaacaattcgacagagcaagttgacacgataatggaaaagaacgtcactgttacacatgcccaagacatactggaaaaaacacacaacgggaagctctgtgatctaaatggggtgaagcctctgattttaaaggattgtagtgtagctggatggctcctcggaaacccaatgtgcgacgaattcatcagagtgccggaatggtcctacatagtggagcggactaatccagctaatgacctctgttacccagggagcctcaatgactatgaagaactgaaacacctgttgagcagaataaatcattttgagaagattctgatcatccccaagagttcctggccaaatcatgaaacatcactaggggtgagcgcagcttgtccataccagggagcgccctcctttttcagaaatgtggtgtggcttatcaaaaagaacgatgcatacccaacaataaagataagctacaataataccaatcgggaagatctcttgatactgtgggggattcatcattccaacaatgcagaagagcagacaaatctctacaaaaacccaaccacctacatttcagttggaacatcaactttaaaccagaggttggtaccaaaaatagctactagatcccaagtaaacgggcaacgtggaagaatggacttcttctggacaattttaaaaccagatgatgcaatccatttcgagagtaatggaaatttcattgctccagaatatgcatacaaaattgtcaagaaaggggactcaacaattatgaaaagtggagtggaatatggccactgcaacaccaaatgtcaaaccccagtaggagcgataaattctagtatgccattccacaacatacatcctctcaccattggggaatgccccaaatacgtgaagtcaaacaagttggtccttgcgactgggctcagaaatagtcctctaagagaaaagagaagaaaaGGGagaggcctgtttggggcgatagcagggtttatagagggaggatggcagggaatggttgatggttggtatgggtaccatcatagcaatgagcaggggagtgggtacgctgcagacaaagaatccacccaaaaggcaatagatggagttaccaataaggtcaactcaatcattgacaaaatgaacactcaatttgaggcagttggaagggagtttaataacttagaaaggaggatagagaatttgaacaagaaaatggaagacggattcctagatgtctggacctataatgctgaacttctagttctcatggaaaacgagaggactctagatttccatgattcaaatgtcaagaacctttacgacaaagtcagactacagcttagggataatgcaaaggagctgggtaacggctgtttcgaattctatcacaaatgcgataatgaatgtatggaaagtgtgagaaatgggacgtatgactaccctcagtattcagaagaagcaagattaaaaagagaagaaataagcggagtgaaattagaatcaataggaacttaccagatactgtcaatttattcaacagcggcgagttccctagcactggcaatcatgatggctggtctatctttatggatgtgctccaatgggtcgttacagtgcagaatttgcatttag

>H5N1_A/barnacle_goose/Netherlands/21027016-002/2021

cttgttaaaagtgatcagatttgcattggttaccatgcaaacaattcgacagagcaagttgacacgataatggaaaagaacgtcactgttacacatgcccaagacatactggagaaaacacacaacgggaagctctgtgatctaaatggggtgaagcctctgattttaaaggattgtagtgtagctggatggctcctcggaaacccaatgtgcgacgaattcatcagagtgccggaatggtcctacatagtggagcgggctaatccagctaatgacctatgttacccagggagcctcaatgactatgaagaactgaaacacctgttgagcagaataaatcattttgagaagattctgatcatccccaagagttcctggccaaatcatgaaacatcactaggggtgagcgcagcttgtccataccagggagcgccctcctttttcagaaatgtggtgtggcttatcaaaaagaacgatgcatacccaacaataaagataagctacaataataccaatcgggaagatctcttgatactgtgggggattcatcattccaacaatgcagaagagcagacaaatctctacaaaaacccaaccacctacatttcagttggaacatcaactttaaaccagaggttggtaccaaaaatagctactagatcccaagtaaacgggcaacgtggaagaatggacttcttctggacaattttaaaaccagatgatgcaatccatttcgagagtaatggaaatttcattgctccagaatatgcatacaaaattgtcaagaaaggggactcaacaattatgaaaagtggagtggaatatggccactgcaacaccaaatgtcaaaccccagtaggagcgataaattctagtatgccattccacaacatacatcctctcaccattggggaatgccccaaatacgtgaagtcaaacaagttggtccttgcgactgggctcagaaatagtcctctaagagaaaagagaagaaaaGGGagaggcctgtttggggcgatagcagggtttatagagggaggatggcagggaatggttgatggttggtatgggtaccatcatagcaatgagcaggggagtgggtacgctgcagacaaagaatccacccaaaaggcaatagatggagttaccaataaggtcaactcaatcattgacaaaatgaacactcaatttgaggcagttggaagggagtttaataacttagaaaggaggatagagaatttgaacaagaaaatggaagacggattcctagatgtctggacctataatgctgaacttctagttctcatggaaaacgagaggactctagatttccatgattcaaatgtcaagaacctttacgacaaagtcagactacagcttagggataatgcaaaggagctgggtaacggctgtttcgaattctatcacaaatgcgataatgaatgtatggaaagtgtgagaaatgggacgtatgactaccctcagtattcagaagaagcaagattaaaaagagaagaaataagcggagtgaaattagaatcaataggaacttaccagatactgtcaatttattcaacagcggcaagttccctagcactggcaatcatgatggctggtctatctttatggatgtgctccaatgggtcgttacagtgcagaatttgcatttag

>H5N1_A/barnacle_goose/Netherlands/21027357-002/2021

cttgttaaaagtgatcagatttgcattggttaccatgcaaacaattcgacagagcaagttgacacgataatggaaaagaacgtcactgttacacatgcccaagacatactggaaaaaacacacaacgggaagctctgtgatctaaatggggtgaagcctctgattttaaaggattgtagtgtagctggatggctcctcggaaacccaatgtgcgacgaattcatcagagtgccggaatggtcctacatagtggagcgggctaatccagctaatgacctctgttacccagggagcctcaatgactatgaagaactgaaacacctgttgagcagaataaatcattttgagaagattctgatcatccccaagagttcctggccaaatcatgaaacatcactaggggtgagcgcagcttgtccataccagggagcgccctcctttttcagaaatgtggtgtggcttatcaaaaagaacgatgcatacccaacaataaagataagctacaataataccaatcgggaagatctcttgatactgtgggggattcatcattccaacaatgcagaagagcagacaaatctctacaaaaacccaaccacctacatttcagttggaacatcaactttaaaccagaggttggcaccaaaaatagctactagatcccaagtaaacgggcaacgtggaagaatggacttcttctggacaatcttaaaaccagatgatgcaatccatttcgagagtaatggaaatttcattgctccagaatatgcatacaaaattgtcaagaaaggggactcaacaattatgaaaagtggagtggaatatggccactgcaacaccaaatgtcaaaccccagtaggagcgataaattctagtatgccattccacaacatacatcctctcaccattggggaatgccccaaatacgtgaagtcaaacaagttggtccttgcgactgggctcagaaatagtcctctaagagaaaagagaagaaaaGGGagaggcctgtttggggcgatagcagggtttatagagggaggatggcagggaatggttgatggttggtatgggtaccatcatagcaatgaacaggggagtgggtacgctgcagacaaagaatccacccaaaaggcaatagatggagttaccaataaggtcaattcaatcattgacaaaatgaacactcaatttgaggcagttggaagggagtttaataacttagaaaggaggatagagaatttgaacaagaaaatggaagacggattcctagatgtctggacctataatgctgaacttctagttctcatggaaaacgagaggactctagatttccatgattcaaatgtcaagaacctttacgacaaagtcagactacagcttagggataatgcaaaggagctgggtaacggctgtttcgaattctatcacaaatgtgataatgagtgtatggaaagtgtgagaaatgggacgtatgactaccctcagtattcagaagaagcaagattaaaaagagaagaaataagcggagtgaaattagaatcaataggaacgtaccagatactgtcaatttattcaacagcggcaagttccctagcactggcaatcatgatggctggtctatctttatggatgtgctccaatgggtcgttacagtgcagaatttgcatttag

>H5N1_A/barnacle_goose/Netherlands/21028196-002/2021

cttgttaaaagtgatcagatttgcattggttaccatgcaaacaattcgacagagcaagttgatacgataatggaaaagaacgtcactgttacacatgcccaagacatactggaaaaaacacacaacgggaagctctgtgatctaaatggggtgaagcctctgattttaaaggattgtagtgtagctggatggctcctcggaaacccaatgtgcgacgaattcatcagagtgccggaatggtcctacatagtggagcgggctaatccagctaatgacctctgttacccagggagcctcaatgactatgaagaactgaaacacctgttgagcagaataaatcattttgagaagattctgatcatccccaagagttcctggccaaatcatgaaacatcactaggggtgagcgcagcttgtccataccagggagcgccctcctttttcagaaatgtggtgtggcttatcaaaaagaacgatgcatacccaacaataaagataagctacaataataccaatcgggaagatctcttgatactgtgggggattcatcattccaacaatgcagaagagcagacaaatctctacaaaaacccaaccacctacatttcagttggaacatcaactttaaaccagaggttggcaccaaaaatagctactagatcccaagtaaacgggcaacgtggaagaatggacttcttctggacaattttaaaaccagatgatgcaatccatttcgagagtaatggaaatttcattgctccagaatatgcatacaaaattatcaagaaaggggactcaacaattatgaaaagtggagtggaatatggccactgcaacaccaaatgtcaaaccccagtaggagcgataaattctagtatgccattccacaacatacatcctctcaccattggggaatgccccaaatacgtgaagtcaaacaagttggtccttgcgactgggctcagaaatagtcctctaagagaaaagagaagaaaaGGGagaggcctgtttggggcgatagcagggtttatagagggaggatggcagggaatggttgatggttggtatgggtaccatcatagcaatgagcaggggagtgggtacgctgcagacaaagaatccacccaaaaggcaatagatggagttaccaataaggtcaactcaatcattgacaaaatgaacactcaatttgaggcagttggaagggagtttaataacttagaaaggaggatagagaatttgaacaagaaaatggaagacggattcctagatgtctggacctataatgctgaacttctagttctcatggaaaacgagaggactctagatttccatgattcaaatgtcaagaacctttacgacaaagtcagactacagcttagggataatgcaaaggagctgggtaacggctgtttcgaattctatcacaaatgcgataatgaatgtatggaaagtgtgagaaatgggacgtatgactaccctcagtattcagaagaagcaagattaaaaagagaagaaataagcggagtgaaattagaatcaataggaacttaccagatactgtcaatttattcaacagcggcaagttccctagcactggcaatcatgatggctggtctatctttatggatgtgctccaatgggtcgttacagtgcagaatttgcatttag

>H5N1_A/barnacle_goose/Netherlands/21028534-002/2021

cttgttaaaagtgatcagatttgcattggttaccatgcaaacaattcgacagagcaagttgacacgataatggaaaagaacgtcactgttacacatgcccaagacatactggagaaaacacacaacgggaagctctgtgatctaaatggggtgaagcctctgattttaaaggattgtagtgtagctggatggctcctcggaaacccaatgtgcgacgaattcatcagagtgccggaatggtcctacatagtggagcgggctaatccagctaatgacctctgttacccagggagcctcaatgactatgaagaactgaaacacctgttgagcagaataaatcattttgagaagattctgatcatccccaagagttcctggccaaatcatgaaacatcactaggggtgagcgcagcttgtccataccagggagcgccctcctttttcagaaatgtggtgtggcttatcaaaaagaacgatgcatacccaacaataaagataagctacaataataccaatcgggaagatctcttgatactgtgggggattcatcattccaacaatgcagaagagcagacaaatctctacaaaaacccaaccacctacatttcagttggaacatcaactttaaaccagaggttggtaccaaaaatagctactagatcccaagtaaacgggcaacgtggaagaatggacttcttctggacaattttaaaaccagatgatgcaatccatttcgagagtaatggaaatttcattgctccagaatatgcatacaaaattgtcaagaaaggggactcaacaattatgaaaagtggagtggaatatggccactgcaacaccaaatgtcaaaccccagtaggagcgataaattctagtatgccattccacaacatacatcctctcaccattggggaatgccccaaatacgtgaagtcaaacaagttggtccttgcgactgggctcagaaatagtcctctaagagaaaagagaagaaaaGGGagaggcctgtttggggcgatagcagggtttatagagggaggatggcagggaatggttgatggttggtatgggtaccatcatagcaatgagcaggggagtgggtacgctgcagacaaagaatccacccaaaaggcaatagatggagttaccaataaggtcaactcaatcattgacaaaatgaacactcaatttgaggcagttggaagggagtttaataacttagaaaggaggatagagaatttgaacaagaaaatggaagacggattcctagatgtctggacctataatgctgaacttctagttctcatggaaaacgagaggactctagatttccatgactcaaatgtcaagaacctttacgacaaagtcagactacagcttagggataatgcaaaggagctgggtaacggctgtttcgaattctatcacaaatgcgataatgaatgtatggaaagtgtgagaaatgggacgtatgactaccctcagtattcagaagaagcaagattaaaaagagaagaaataagcggagtgaaattagaatcaataggaacttaccagatactgtcaatttattcaacagcggcaagttccctagcactggcaatcatgatggctggtctatctttatggatgtgctccaatgggtcgttacagtgcagaatttgcatttag

>H5N1_A/barnacle_goose/Sweden/SVA210511SZ0567/FB001840/M-2021

cttgttaaaagtgatcagatttgcattggttaccatgcaaacaattcgacagagcaagttgacacgataatggaaaagaacgtcactgttacacatgcccaagacatactggaaaaaacacacaacgggaagctctgtgatctaaatggggtgaagcctctgattttaaaggattgtagtgtagctggatggctcctcggaaacccaatgtgcgacgaattcatcagagtgccggaatggtcctacatagtggagcgggctaatccagctaatgacctctgttacccagggagcctcaatgactatgaagaactgaaacacctgttgagcagaataaatcattttgagaaaattctgatcatccccaaaagttcctggccaaatcatgaaacatcactaggggtgagcgcagcttgtccataccagggagcgccctcctttttcagaaatgtggtgtggcttatcaaaaagaacgatgcatacccaacaataaagataagctacaataataccaatcgggaagatctcttgatactgtgggggattcatcattccaacaatgcagaagagcagacaaatctctacaaaaacccaaccacctacatttcagttggaacttcaactttaaaccagaggttggtaccaaaaatagctactagatcccaagtaaacgggcaacgtggaagaatggacttcttctggacaattttaaaaccagatgatgccatccatttcgagagtaatggaaatttcattgctccagaatatgcatacaaaattgtcaagaaaggggactcaacaattatgaaaagtggagtggaatatggccactgcaacaccaaatgtcaaaccccagtaggagcgataaattctagtatgccattccacaacatacatcctctcaccattggggaatgccccaaatacgtgaagtcaaacaagttggtccttgcgactgggctcagaaatagtcctctaagagaaaagagaagaaaaGGGagaggcctgtttggggcgatagcagggtttatagagggaggatggcagggaatggttgatggttggtatgggtaccatcatagcaatgagcaggggagtgggtacgctgcagacaaagaatccacccaaaaggcaatagatggagttaccaataaggtcaactcaatcattgacaaaatgaacactcaatttgaggcagttggaagggagtttaataacttagaaaggaggatagagaatttgaacaagaaaatggaagacggattcctagatgtctggacctataatgctgaacttctagttctcatggaaaacgagaggactctagatttccatgattcaaatgtcaagaacctttacgacaaagtcagactacagcttagggataatgcaaaggagctgggtaacggctgtttcgaattctatcacaaatgcgataatgaatgtatggaaagtgtgagaaatgggacgtatgactaccctcagtattcagaagaagcaagattaaaaagagaagaaataagcggagtgaaattagaatcaataggaacttaccagatactgtcaatttattcaacagcggcaagttccctagcactggcaatcatgatggctggtctatctttatggatgtgctccaatgggtcgttacagtgcagaatttgcatttag

>H5N1_A/barnacle_goose/Sweden/SVA211102SZ0402/FB004395/M-2021

cttgttaaaagtgatcagatttgcattggttaccatgcaaacaattcgacagagcaagttgacacgataatggaaaagaacgtaactgttacacatgcccaagacatactggaaaaaacacacaacgggaagctctgtgatctaaatggggtgaagcctctgattttaaaggattgtagtgtagctggatggctcctcggaaacccaatgtgcgacgaattcatcagagtgccggaatggtcctacatagtggagcgggctaatccagctaatgacctctgttacccagggagcctcaatgactatgaagaactgaaacacctgttgagcagaataaatcattttgagaagattctgatcatccccaagagttcctggccaaatcatgaaacatcactaggggtgagcgcagcttgtccataccagggagcgccctcctttttcagaaatgtggtgtggcttatcaaaaagaacgatgcatacccaacaataaagataagctacaataataccaatcgggaagatctcttgatactgtgggggattcatcattccaacaatgcagaagagcagacaaatctctacaaaaacccaaccacctacatttcagttggaacatcaactttaaaccagaggttggtaccaaaaatagctactagatcccaagtaaacgggcaacgtggacgaatggacttcttctggacaattttaaaaccagatgatgcaatccatttcgagagtaatggaaatttcattgctccagaatatgcatacaaaattgtcaagaaaggggactcaacaattatgaaaagtggagtggaatatggccactgcaacaccaaatgtcaaaccccagtaggagcgataaattctagtatgccattccacaacatacatcctctcaccattggggaatgccccaaatacgtgaagtcaaacaagttggtccttgcgactgggcttagaaatagtcctctaagagaaaagagaagaaaaGGGagaggcctgtttggggcgatagcagggtttatagagggaggatggcagggaatggttgatggttggtatgggtaccatcatagcaatgagcaggggagtgggtacgctgcagacaaagaatccacccaaaaggcaatagatggagttaccaataaggtcaactcaatcattgacaaaatgaacactcaatttgaggcagttggaagggagtttaataacttagaaaggaggatagagaatttgaacaagaaaatggaagacggattcctagatgtctggacctataatgctgaacttctagttctcatggaaaacgagaggactctagatttccatgattcaaatgtcaagaacctttacgacaaagtcagactacagcttagggataatgcaaaggagctgggtaacggctgtttcgaattctatcacaaatgcgataatgaatgtatggaaagtgtgagaaatgggacgtatgactaccctcagtattcagaagaagcaagattaaaaagagaagaaataagcggagtgaaattagaatcaataggaacttaccagatactgtcaatttattcaacagcggcaagttccctagcactggcaatcatgatggctggtctatctttatggatgtgctccaatgggtcgttacagtgcagaatttgcatttag

>H5N1_A/barnacle_goose/Sweden/SVA211111SZ0376/FB004496/2021

cttgttaaaagtgatcagatttgcattggttaccatgcaaacaattcgacagagaaggttgacacgataatggaaaagaacgtcactgttacacatgcccaagacatactggaaaaaacacacaacgggaagctctgtgatttaaatggggtgaagcctctgattttaaaggattgtagtgtagctggatggctcctcggaaacccaatgtgcgacgaattcatcagagtgccggaatggtcctacatagtggagcgggctaatccagctaatgacctctgttacccagggagcctcaatgactatgaagaactgaaacacctgttgagcagaataaaccattttgagaagattctgatcatccccaagagttcctggccaaatcatgaaacatcactaggggtgagcgcagcttgtccataccagggagcgccctcctttttcagaaatgtggtgtggcttatcaaaaagaacgatgcatacccaacaataaagataagctacaataataccaatcgggaagatctcttgatactgtgggggattcatcattccaacaatgcagaagaacagacaaatctctataaaaacccaaccacctacatttcagttggaacatcaactttaaaccagaggttggtaccaaaaatagctactagatcccaagtaaacgggcaacgtggaagaatggacttcttctggacaattttaaaaccagatgatgcaatccatttcgagagtaatggaaatttcattgctccagaatatgcatataaaattgtcaagaaaggggactcaacaattatgaaaagtggagtggaatatggccactgcaacaccaaatgtcaaaccccagtaggagcgataaattctagtatgccattccacaacatacatcctctcaccattggggaatgccccaaatacgtgaagtcaaacaagttggtccttgcgactgggctcagaaatagtcctctaagagaaaagagaagaaaaGGGagaggcctgtttggggcgatagcagggtttatagagggaggatggcagggaatggttgatggttggtatgggtaccatcatagcaatgagcaggggagtgggtacgctgcagacaaagaatccacccaaaaggcaatagatggagttaccaataaggtcaactcaatcattgacaaaatgaacactcaattcgaggcagttggaagggagtttaataacttagaaaggaggatagagaatttgaacaagaaaatggaagacggattcctagatgtctggacctataatgctgaacttctagttctcatggaaaacgagaggactctagatttccatgattcaaatgtcaagaacctttacgacaaagtcagactacagcttagggataatgcaaaggagctgggtaatggctgtttcgaattctatcacaaatgcgataatgaatgtatggaaagtgtgagaaatgggacgtatgactaccctcagtattcagaagaagcaagattaaaaagagaagaaataagcggagtgaaattagaatcaataggaacttaccagatactgtcaatttattcaacagcggcgagttccctagcactggcaatcatgatagctggtctatctttatggatgtgctccaatgggtcgttacagtgcagaatttgcatttag

>H5N1_A/bean goose/Tyva/10/2009

cttgttaaaagcgatcatatttgcattggttatcatgcaaataactcgacagagcaggttgacacaataatggaaaagaacgttactgttacacatgcccaagacatactggaaaagacacacaacgggaagctctgcgatctaaatggagtgaagcctctgattttaaaagattgtagtgtagcgggatggctcctcggaaacccattgtgcgacgaattcatcaatgtgccagaatggtcttacatagtagagaaggccaatccagccaatgacctctgttacccagggaatttcaacgattatgaagaattgaaacacctattgagcaggataaaccattttgagaaaatacagatcatccccaaagattcttggtcagatcatgaagcctcattgggggtgagcgcagcatgttcataccagggaaattcctccttcttcagaaatgtggtatggcttatcaaaaaggacaatgcatacccaacaataaagaaaggctacaataataccaaccaagaagatctcttggtactgtgggggattcaccatcctaatgatgaggcagagcagacaaggctctatcaaaacccaaccacctatatttccattgggacatcaacactaaaccagagattggtaccaaaaatagccactagatccaaaataaacgggcaaagtggcaggatagatttcttctggacaattttaaaaccgaatgatgcaatccacttcgagagtaatggaaatttcattgctccagaatatgcatacaaaattgtcaagaaaggagactccacaattatgaaaagtgaagtggaatatggtaactgcaacaccaggtgtcagactccgataggggcgataaactctagtatgccattccacaacatacaccctctcaccatcggagaatgtcccaaatatgtgaaatcaaacaaattagtccttgcgactgggctcagaaatagtcctcaaagagagagaagaagaaaaGGGagaggactgtttggagctatagcaggttttatagagggaggatggcagggaatggtagatggttggtatgggtaccaccacagcaatgagcaggggagtgggtacgctgcagacaaagaatctactcaaaaggcaatagacggagtcaccaataaggtcaactcgatcattgacaaaatgaacactcagtttgaggccgtaggaagggaatttaataacttagagaggagaatagagaatttaaacaagaagatggaagacggattcctagatgtctggacttataatgctgaacttctggttctcatggaaaatgagagaactctagacttccatgactcaaatgtcaagaacctttacgataaggtcagactacagcttaaggataatgcaaaagagttgggtaacggttgtttcgagttctatcacaaatgtaataatgaatgtatggaaagtgtaagaaacggaacgtatgactacccgcagtattcagaagaagcaagattaaaaagagaggaaataagtggagtaaaattggaatcaataggaatctaccaaatactgtcaatttattcaacagtggcgagttccctagtgctggcaatcatgatggctggtctatctttatggatgtgttccaacgggtcgttacagtgcagaatttgcatttga

>H5N1_A/bean_goose/Sweden/SVA211111SZ0372/FB004482/2021

cttgttaaaagtgatcagatttgcattggttaccatgcaaacaattcgacagagcaggttgacacgataatggaaaagaacgtcactgttacacatgcccaagacatactggaaaaaacacacaacgggaagctctgtgatttaaatggggtgaagcctctgattttaaaggattgtagtgtagctggatggctcctcggaaacccaatgtgcgacgaattcatcagagtgccggaatggtcctacatagtggagcgggataatccagccaatgacctctgttacccagggagcctcaatgactatgaagaactgaaacacctgttgagcagaataaatcattttgagaagattctgatcatccccaagagttcctggccaaatcatgaaacatcactaggggtgagcgcagcttgtccataccagggagcgccctcctttttcagaaatgtggtgtggcttatcaaaaagaacgatgcatacccaacaataaagataagctacaataataccaatcgggaagatctcttgatactgtgggggattcatcattccaacaatgcagaagagcagacaaatctctataaaaacccaaccacctacatttcagttggaacatcaactttaaaccagaggttggtaccaaaaatagctactagatcccaagtaaacgggcaacgtggaagaatggacttcttctggacaattttaaaaccagatgatgcaatccatttcgagagtaatggaaatttcattgcaccagaatatgcatacaaaattgtcaagaaaggggactcaacaattatgaaaagtggagtggaatatggccactgcaacaccaaatgtcaaaccccagtaggagcgataaattctagtatgccattccacaacatacatcctctcaccattggggaatgccccaaatacgtgaagtcaaacaagttagtccttgcgactgggctcagaaatagtcctctaagagaaaagagaagaaaaGGGagaggcctgtttggggcgatagcagggtttatagagggaggatggcagggaatggttgatggttggtatgggtaccatcatagcaatgagcaggggagtgggtacgctgcagacaaagaatccacccaaaaggcaatagatggagttaccaataaggtcaactcaatcattgacaaaatgaacactcaatttgaggcagttggaagggagtttaataacttagaaaggaggatagagaatttgaacaagaaaatggaagacggattcctagatgtctggacctataatgctgaacttctagttctcatggaaaacgagaggactctagatttccatgattcaaatgtcaagaacctttacgacaaagtcagactacagcttagggataatgcaaaggagctgggcaatggctgtttcgaattctatcacaaatgcgataatgaatgtatggaaagtgtgagaaatgggacgtatgactacccccagtattcagaagaagcaagattaaaaagagaagaaataagcggagtgaaattagaatcaataggaacttaccagatactgtcaatttattcaacagcggcgagttccctagcactggcaatcatgatagctggtctatctttatggatgtgctccaatgggtcgttacagtgcagaatttgcatttag

>H5N1_A/black duck/Tennessee/17OS0306/2017

attgtcaaaggtgaccaaatctgcattggttatcatgcaaacaattcaacagagcaggttgatacaatcatggagaagaatgtgacggtcacacatgctcaggatatactggaaaaagagcacaatggaaaactttgcagtattaaaggagtgaggcccctcattctgaaggattgcagtgtagctggatggcttcttggaaacccaatgtgtgatgaatttctgaatgtaccggaatggtcatacatcgtggaaaaagataatccagtctatggcctgtgctatccagggaacttcaatgattacgaagaactgaagcatttaatgagcagcacaaaccattttgagaaaattcagataattcctaggagttcttggtccaatcatgatgcctcatcaggagtaagttcggcatgcccatacaatggcagatcttcctttttcaggaatgtggtgtggttgatcaagaagaacaatgcgtacccaacaataaagaggacctacaacaacaccaatgtagaagatcttttaataatatggggaattcaccatcctaatgatgcagctgaacaaacaaagctctatcagaactcgaacacttatgtgtctgtaggaacatcaacactgaatcagagatcaatcccagaaatagccactagacccaaagtgaacgggcaaagtggaagaatggaatttttctggacaatactgaggccgaacgatgcaatcagctttgaaagcaatggaaattttatagcccctgaatatgcgtacaagattgtcaagaaaggagattcagcaatcatgaggagtgaattggagtatggtaactgtgacaccaaatgtcagaccccagtgggagctataaattccagtatgcccttccacaatgttcatcctcttaccattggggagtgccccaagtatgtcaaatcggacaagctggtccttgcaacaggactaagaaacgtcccccaaagagaaacaGGGGGGGGGGGGagaggcctatttggagcaatagcaggattcatagaaggaggatggcaaggaatggttgatggatggtacggataccatcatagcaatgagcagggaagtggatatgctgcagacaaagaatctacccagaaagcaatcgatgggatcactaataaagtaaactcaatcattgacaaaatgaacactcaatttgaagccgctgggaaagaattcaacaacctggaaagaagaatagaaaatttgaataagaaaatggaagatgggtttttagatgtatggacttacaatgcagaacttcttgtgctcatggaaaatgagagaactttggacttccatgattcaaatgtcaagaacctatatgataaggtccgactccagctgagggacaatgcaaaagaattgggcaacggatgctttgaattctaccacaagtgtgacaatgaatgcatggaaagtgtgagaaatggaacgtatgactatccgcaatattcggaagaatcaagactgaacagggaggaaatagacggagtcaaattggaatcaatgggcacctatcagatactatcaatctactcaacagtggcgagttccctagcactggcaatcatgatagctggtctatctttttggatgtgctccaatggatcattgcagtgcagaatttgcatctga

>H5N1_A/black swan/Yamaguchi/1/2011

cttgttaaaagcgatcatatttgcattggttatcatgcaaataactcgacagagcaggttgacacaataatggaaaagaacgttactgttacacatgcccaagacatactggaaaagacacacaacgggaagctctgcgatctaaatggagtgaagcctctgattttaaaagattgtagtgtagcgggatggctcctcggaaacccattgtgtgacgaattcatcaatgtgccagaatggtcttacatagtagagaaggccaagccagccaatgacctctgttacccagggaatttcaacgattatgaagaattgaaacacctattgagcaggataaaccattttgagaaaatacagatcatccccaaagactcttggtcagatcatgaagcctcattgggggtgagcgcagcatgttcataccagggaaattcctccttcttcagaaatgtggtatggcttatcaaaaaggacaatgcatacccaacaataaagaaaggctacaataataccaaccaagaagatctcttggtactgtgggggattcaccatcctaatgatgaggcagagcagacaaggctctatcaaaacccaaccacctatatttccattgggacatcaacactaaaccagagattggtaccaaaaatagccactagatccaaaataaacgggcaaagtggcaggatagatttcttctggacaattttaaaaccgaatgatgcaatccacttcgagagtaatggaaatttcattgctccagaatatgcatacaaaattgtcaagaaaggagactccacaattatgaaaagtgaagtggaatatggtaactgcaacaccaggtgtcagactccgataggggcgataaactctagtatgccattccacaacatacaccctctcaccatcggagaatgtcccaaatatgtgaaatcaaacaaattagtccttgcgactgggctcagaaatagtcctcaaagagagagaagaagaaaaGGGagaggactgtttggagctatagcaggttttatagagggaggatggcagggaatggtagatggttggtatgggtaccaccacagcaatgagcaggggagtgggtacgctgcagacaaagaatctactcaaaaggcaatagacggagtcaccaataaggtcaattcgatcattgacaaaatgaacactcagtttgaggccgtaggaagggaatttaataacttagagaggagaatagagaatttaaacaagaagatggaagacggattcctagatgtttggacttataatgctgaacttctggttctcatggaaaatgagagaactctagatttccatgactcaaatgtcaagaacctttacgataaggtcagactacagcttaaggataatgcaaaagagttgggtaacggttgtttcgagttctatcacaaatgtaataatgaatgtatggaaagtgtaagaaacggaacgtatgactacccgcagtattcagaagaagcaagactaaaaagagaggaaataagtggagtaaaattggaatcaataggaatctaccaaatactgtcaatttattcaacagtggcgagttccctagtgctggcaatcatgatggctggtctgtctttatggatgtgttccaacggatcgttacagtgcagaatttgcatttga

>H5N1_A/black-crowned night heron/Hong Kong/659/2008

cttgttaaaagcgatcatatttgcattggttatcatgcaaacaactcgacagagcaggttgacacaataatggaaaagaacgttactgttacacatgcccaagacatactggaaaagacacacaacgggaagctctgcgatctaaatggagtgaagcctctgattttaaaagattgtagtgtagcaggatggctcctcggaaacccaatgtgtgacgaattcatcaatgtgccagaatggtcttacatagtagagaaggccaatccagccaatgacctctgttacccagggaatttcaacgattatgaagaattgaaacacctattgagcaggataaaccattttgagaaaatacagatcatccccaaagattcttggtcagatcatgaagcctcattgggggtgagcgcagcatgtccataccagggaaattcctcctttttcagaaatgtggtatggcttatcaaaaaggacaatgcatacccaacaataaagaaaagctacaataataccaaccaagaagatctcttggtactgtggggaattcaccatcctaatgatgaggcagaacagacaaggctctatcaaaacccaaccacctatatttccattgggacatcaacactaaaccagagattggtaccaaaaatagccacgagatccaaagtaaacgggcaaagtggaaggatagatttcttctggacaattttaaaaccgaatgatgcaatcaacttcgagagtaatggaaatttcattgctccagaatatgcatacaaagttgtcaagaaaggagactcaacaattatgaaaagtgaagtggaatatggtaactgcaacaccaggtgtcagactccgatgggggcgataaactctagtatgccattccacaacatacaccctctcaccatcggagaatgtcccaaatatgtgaaatcaaacaaattagtccttgcgactgggctcagaaatagtcctcaaagagagagaagaagaaaaGGGagaggactgtttggagctatagcaggttttatagagggaggatggcagggaatggtagatggttggtatgggtaccaccacagcaawaggcaggggagtgggtacgctgcagacaaagaatccactcaaaaggcaatagacggagtcaccaataaggtcaactcgatcattgacaaaatgaacactcagtttgaggccgtaggaagggaatttaataacttagagaggagaatagagaatttaaacaagaagatggaagacggattcctagatgtctggacttataatgctgaacttctggttctcatggaaaatgagagaactctagacttccatgattcaaatgtcaagaacctttacgacaaggtcagactacagcttagggataatgcaaaagagctgggtaacggttgtttcgagttctatcacaaatgtaataatgaatgtatggaaagtgtaagaaacggaacgtatgactacccgcagtattcagaagaagcaagattaaaaagagaggaaataagtggagtaaaattggaatcaataggaacataccaaatactgtcaatttattcaacagtggcgagttccctagtactggcaatcatgatggctggtctatctttatggatgtgttccaacgggtcgttacagtgcagaatttgcatttga

>H5N1_A/black-headed_gull/England/306270/2022

cttgttaaaagtgatcagatttgcattggttaccatgcaaacaattcgacagagcaggttgacacgataatggaaaagaacgtcactgttacacatgcccaagacatactggaaaaaacacacaacgggaagctctgtgatttaaatggggtgaagcctctgattttaaaggattgtagtgtagctggatggctcctcggaaacccaatgtgcgacgaattcatcagagtgccggaatggtcctacatagtggagcgggctaatccagccaatgacctctgttacccagggagcctcaatgactatgaagaactgaaacacctgttgagcagaataaatcattttgagaagattctgatcatccccaagagttcctggccaaatcatgaaacatcactaggggtgagcgcagcttgtccataccagggagcgccctccttttttagaaatgtgttgtggcttatcaaaaagaacgatgcatacccaacaataaagataagctacaataataccaatcgggaagatctcttgatactgtgggggattcatcattccaacaatgcagaagagcagacaaatctctataaaaacccaaccacctacatttcagttggaacatcaactttaaaccagaggttggtaccaaaaatagctactagatcccaagtaaacgggcaacgtggaaggatggacttcttctggacaattttaaaaccagatgatgcaatccatttcgagagtaatggaaatttcattgcaccagaatatgcatataaaattgtcaagaaaggggactcaacaattatgaaaagtggagtggaatatggccactgcaacaccaaatgtcaaaccccagtaggagcgataaattctagtatgccattccacaacatacatcctctcaccattggggaatgccccaaatacgtgaagtcaaacaagttggtccttgcgactgggctcagaaatagtcctctaagagaaaagagaagaaaaGGGagaggcctgtttggggcgatagcagggtttatagagggaggatggcagggaatggttgatggttggtatgggtaccatcatagcaatgagcaggggagtgggtacgctgcagacaaagaatccacccaaaaggcaatagatggagttaccaataaggtcaactcaatcattgacaaaatgaacactcaatttgaggcagttggaagggagtttaataacttagaaaggaggatagagaatttgaacaagaaaatggaagacggattcctagatgtctggacctataatgctgaacttctagttctcatggaaaacgagaggactctagatttccatgattcaaatgtcaagaacctttacgacaaagtcagactacagcttagggacaatgcaaaggagctgggtaatggctgtttcgaattctatcacaaatgcgataatgaatgtatggaaagtgtgagaaatgggacgtatgactaccctcagtattcagaagaagcaagattaaaaagagaagaaataagcggagtgaaattagaatcaataggaatttaccagatactgtcaatttattcaacagcggcgagttccctagcactggcaatcatgatagctggtctatctttatggatgtgctccaatgggtcgttacagtgcagaatttgcatttag

>H5N1_A/black-headed_gull/England/388256/2022

cttgttaaaagtgatcagatttgcattggttaccatgcaaacaattcgacagagcaggttgacacgataatggaaaagaacgtcactgttacacatgcccaagacatactggaaaaaacacacaacgggaagctctgtgatttaaatggggtgaagcctctgattttaaaggattgtagtgtagctggatggctcctcggaaacccaatgtgtgacgaattcatcagagtgccggaatggtcctacatagtggagcgggctaatccagccaatgacctctgttacccagggagcctcaatgactatgaagaattgaaacacctgttgagcagaataaatcattttgagaagattctgatcatccccaagagttcctggccaaatcatgaaacatcactaggggtgagcgcagcttgtccataccagggagcgccctcctttttcagaaatgtgttgtggcttatcaaaaagaacgatgcatacccaacaataaagataagctacaataataccaatcgggaagatctcttgatattgtgggggattcatcattccaacaatgcagaagagcagacaaatctctataaaaacccaaccacctacatttcagttggaacatcaactttaaaccagaggttggtaccaaaaatagctactagatcccaagtaaacgggcaacgtggaagaatggacttcttctggacaattttaaaaccagatgatgcaatccatttcgagagtaatggaaatttcattgcaccagaatatgcatataaaattgtcaagaaaggggactcaacaattatgaaaagtggagtggaatatggccactgcaacaccaaatgtcaaaccccagtaggagcgataaattctagtatgccattccacaacatacatcctctcaccattggggaatgccccaaatacgtgaagtcaaacaagttggtccttgcgactgggctcagaaatagtcctctaagagaaaagagaaggaaaGGGagaggcctgtttggggcgatagcagggtttatagagggaggatggcagggaatggttgatggttggtatgggtaccatcatagcaatgagcaggggagtgggtacgctgcagacaaagaatccacccaaaaggcaatagatggagttaccaataaggtcaactcaatcattgacaaaatgaacactcaatttgaggcagttggaagggagtttaataacttagaaaggaggatagagaatttgaacaagaaaatggaagacggattcctagatgtctggacctataatgctgaacttctagttctcatggaaaacgagaggactctagatttccatgattcaaatgtcaagaacctttacgacaaagtcagactacagcttagggacaatgcaaaggagctgggtaatggctgtttcgaattctatcacaaatgcgataatgaatgtatggaaagtgtgagaaatgggacgtatgactaccctcagtattcagaagaagcaagattaaaaagagaagaaataagcggagtgaaattagaatcaataggaacttaccagatactgtcaatttattcaacagcggcgagttccctagcactggcaatcatgatagctggtctatctttatggatgtgctccaatgggtcgttacagtgcagaatttgcatttag

>H5N1_A/blue-winged teal/Texas/16-031241-1/2016

attgtcaaaggtgaccaaatctgcattggttatcatgcaaacaattcaacagagcaggttgatacaatcatggagaagaatgtgacggtcacacatgctcaggatatactggaaaaagagcacaatggaaaactttgcagtattaaaggggtgaggcccctcattctgaaggattgcagtgtagctggatggcttcttggaaacccaatgtgtgatgaattcctgaatgtaccggaatggtcatacatcgtggaaaaagataatccagtcaatggcctgtgctatccaggggacttcagtgattacgaagaactgaagcatttaatgagcagcacaaaccattttgagaaaattcaaataattcctaggagttcttggtccaatcatgatgcctcatcaggagtaagttcggcatgcccatacaatggcagatcttcctttttcaggaatgtggtgtggttgatcaagaagaacaatgcgtacccaacaataaagaggacctacaacaacaccaatgtagaagaccttttaataatatggggaattcaccatcctaatgatgcagctgaacaaacaaagctctatcagaactcgaacacttatgtgtctgtaggaacatcaacactgaatcagagatcaatcccagaaatagccactagacccaaagtaaacgggcaaagtggaagaatggaatttttctggacaatactgaggccgaacgatgcaatcagctttgaaagcaatgggaattttatagcccctgaatatgcgtacaaaattgtcaagaaaggagattcagcaatcatgaggagtgaattggggtatggtaactgtgacaccaaatgtcagaccccagtgggagctataaattccagcatgcccttccacaatgttcatcctcttaccattggggagtgccccaagtatgtcaaatcggacaagctggtccttgcaacaggactaagaaacgtcccccaaagagaaacaGGGGGGGGGGGGagaggcctatttggagcaatagcaggattcatagaaggaggatggcaaggaatggttgatggatggtacgggtaccatcatagcaatgagcagggaagtggatatgctgcagacaaagaatctacccagaaagcaatcgatgggatcaccaataaagtaaactcaatcattgacaaaatgaacactcaattcgaagccgtcgggaaagaattcaacaacctggaaaggagaatagaaaatttgaataggaaaatggaagatgggtttttagatgtatggacttacaatgcagaacttcttgtgctcatggaaaatgagagaactttggacttccatgattcaaatgtcaagaacctatatgagaaggtccgactccagctgagagacaatgcaaaagaattgggcaacggatgctttgaattctaccacaagtgtgacaatgaatgcatggaaagtgtgagaaatggaacgtatgactatccgcaatattcggaagaatcaagactgaacagggaggaaatagacggagtcaaattggaatcaatggccacctatcagatactatcaatctactcaacagtggcgagttccctagcactggcaatcatgatagctggtctatctttttggatgtgctccaacggatcattgcagtgcagaatttgcatctga

>H5N1_A/brent_goose/Ireland/033257_22VIR1325-9/2021

cttgttaaaagtgatcagatttgcattggttaccatgcaaacaattcgacagagaaggttgacacgataatggaaaagaacgtcactgttacacatgcccaagacatactggaaaaaacacacaacgggaagctctgtgatttaaatggggtgaagcctctgattttaaaggattgtagtgtagctggatggctcctcggaaacccaatgtgcgacgaattcatcagagtgccggaatggtcctacatagtggagcgggctaatccagctaatgacctctgttacccagggagcctcaatgactatgaagaactgaaacacctgttgagcagaataaaccattttgagaagattctgatcatccccaagagttcctggccaaatcatgaaacatcactaggggtgagcgcagcttgtccataccagggagcgccctcctttttcagaaatgtggtgtggcttatcaaaaagaacgatgcatacccaacaataaagataagctacaataataccaatcgggaagatctcttgatactgtgggggattcatcattccaacaatgcagaagaacagacaaatctctataaaaacccaaccacctacatttcagttggaacatcaactttaaaccagaggttggtaccaaaaatagctactagatcccaagtaaacgggcaacgtggaagaatggacttcttctggacaattttaaaaccagatgatgcaatccatttcgagagtaatggaaatttcattgctccagaatatgcatataaaattgtcaagaaaggggactcaacaattatgaaaagtggagtggaatatggccactgcaacaccaaatgtcaaaccccagtaggagcgataaattctagtatgccattccacaacatacatcctctcaccattggggaatgccccaaatacgtgaagtcaaacaagttggtccttgcgactgggctcagaaatagtcctctaagagaaaagagaagaaaaGGGagaggcctgtttggggcgatagcagggtttatagagggaggatggcagggaatggttgatggttggtatgggtaccatcatagcaatgagcaggggagtgggtacgctgcagacaaagaatccacccaaaaggcaatagatggagttaccaataaggtcaactcaatcattgacaaaatgaacactcaatttgaggcagttggaagggagtttaataacttagaaaggaggatagagaatttgaacaagaaaatggaagacggattcctagatgtctggacctataatgctgaacttctagttctcatggaaaacgagaggactctagatttccatgattcaaatgtcaagaacctttacgacaaagtcagactacagcttagggataatgcaaaggagctgggtaatggctgtttcgaattctatcacaaatgcgataatgaatgtatggaaagtgtgagaaatgggacgtatgactaccctcagtattcagaagaagcaagattaaaaagagaagaaataagcggagtgaagttagaatcaataggaacttaccagatactgtcaatttattcaacagcggcgagttccctagcactggcaatcatgatagctggtctatctttatggatgtgctccaatgggtcgttacagtgcagaatttgcatttag

>H5N1_A/broiler/Ireland/033734_22VIR1325-20/2021

cttgttaaaagtgatcagatttgcattggttaccatgcaaacaattcgacagagcaggttgacacgataatggaaaagaacgtcactgttacacatgcccaagacatactggaaaaaacacacaacgggaagctctgtgatttaaatggggtgaagcctctgattttaaaggattgtagtgtagctggatggctcctcggaaacccaatgtgcgacgaattcatcagagtgccggaatggtcctacatagtggagcgggctaatccagccaatgacctctgttacccagggagcctcaatgactatgaagaactgaaacacctgttgagcagaataaatcattttgagaagattctaatcatccccaagagttcctggccaaatcatgaaacatcactaggggtgagcgcagcttgtccataccagggagcgccctcctttttcagaaatgtgttgtggcttatcaaaaagaacgatgcatacccaacaataaagataagctacaataataccaatcgggaagatctcttgatattgtgggggattcatcattccaacaatgcagaagagcagacaaatctctataaaaacccaaccacctacatttcagttggaacatcaactttaaaccagaggttggtaccaaaaatagctactagatcccaagtaaacgggcaacgtggaagaatggacttcttctggacaattttaaaaccagatgatgcaatccatttcgagagtaatggaaatttcattgcaccaaaatatgcatataaaattgtcaagaaaggggactcaacaattatgaaaagtggagtggaatatggccactgcaacaccaaatgtcaaaccccagtaggagcgataaattctagtatgccattccacaacatacatcctctcaccattggggaatgccccaaatacgtgaagtcaaacaagttggtccttgcgactgggctcagaaatagtcctctaagagaaaagagaagaaaaGGGagaggcctgtttggggcgatagcagggtttatagagggaggatggcagggaatggttgatggttggtatgggtaccatcatagcaatgagcaggggagtgggtacgctgcagacaaagaatccacccaaaaggcaatagatggagttaccaataaggtcaactcaatcattgacaaaatgaacactcaatttgaggcagttggaagggagtttaataacttagaaaggaggatagagaatttgaacaagaaaatggaagacggattcctagatgtctggacctataatgctgaacttctagttctcatggaaaacgagaggactctagatttccatgattcaaatgtcaagaacctttacgacaaagtcagactacagcttagggacaatgcaaaggagctgggtaatggctgtttcgaattctatcacaaatgcgataatgaatgtatggaaagtgtgagaaatgggacgtatgactaccctcagtattcagaagaagcaagattaaaaagagaagaaataagcggagtgaaattagaatcaataggaacttaccagatactgtcaatttattcaacagcggcgagttccctagcactggcaatcatgatagctggtctatctttatggatgtgctccaatgggtcgttacagtgcagaatttgcatttag

>H5N1_A/brown-headed gull/Qinghai/19/2009

cttgttaaaagcgatcatatttgcattggttatcatgcaaataactcgacagagcaggttgacacaataatggaaaagaacgttactgtcacacatgcccaagacatactggagaagacacacaacgggaatctctgcgatctaaatggagtgaagcctctgattttaaaagattgtagtgtagcgggatggctcctcggaaacccattgtgtgacgaattcatcaatgtgccagaatggtcttacatagtagagaaggccaatccagccaatgacctctgttacccagggaatttcaacgattatgaagaattgaaacacctattgagcaggataaaccattttgagaaaatacagatcatccccaaagattcttggtcagatcatgaagcctcattgggggtgagcgcagcatgtccataccagggaaattcctccttcttcagaaatgtggtatggcttatcaaaaaggacaatgcatatccaacaataaagaaaggctacaataataccaaccaagaagatctcttggtactgtgggggattcaccatcctaatgatgaggcagagcacacaaggctctatcaaaacccaaccacctatatttccattgggacatcaacactaaaccagagattggtaccaaaaatagccactagatccaaaataaacgggcaaagtggcaggatagatttcttctggacaattttaaaaccgaatgatgcaatccacttcgagagtaatggaaatttcattgctccagaatatgcatacaaaattgtcaagaaaggagactccacaattatgaaaagtgaagtggaatatggtaactgcaacaccaggtgtcagactccgataggggcgataaactctagtatgccattccacaacatacaccctctcaccatcggagaatgtcccaaatatgtgaaatcaaacaaattagtccttgcgactgggctcagaaatagtcctcaaagagagagaagaagaaaaGGGagaggactgtttggagctatagcaggttttatagagggaggatggcagggaatggtagatggttggtatgggtaccaccacagcaatgagcaggggagtgggtacgctgcagacaaagaatctactcaaaaggcaatagacggagtcaccaataaggtcaactcgatcattgacaaaatgaacactcagtttgaggccgtaggaagggaatttaataacttagagaggagaatagagaatttaaacaagaagatggaagacggattcctagatgtctggacttataatgctgaacttctggttctcatggaaaatgagagaactctagacttccatgactcaaatgtcaagaacctttacgataaggtcagactacagcttaaggataatgcaaaagagttgggtaacggttgtttcgagttctatcacaaatgtaataatgaatgtatggaaagtgtaagaaacggaacgtatgactacccgcagtattcagaagaagcaagattaaaaagagaggaaataagtggagtaaaattggaatcaataggaatctaccaaatactgtcaatttattcaacagtggcgagttccctagtgctggcaatcatgatggctggtctatctttatggatgtgctccaacgggtcgttacagtgcagaatttgcatttga

>H5N1_A/brown-headed gull/Qinghai/9/2009

gttgttaaaagcgatcatatttgcattggttatcatgcaaataactcgacagagcaggttgacacaataatggaaaagaacgttactgttacacatgcccaagacatactggaaaagacacacaacgggaagctctgcgatctaaatggagtgaagcctctgattttaaaagattgtagtgtagcgggatggctcctcggaaacccattgtgtgacgaattcatcaatgtgccagaatggtcttacatagtagagaaggccaatccagccaatgacctctgttacccagggaatttcaacgattatgaagaattgaaacacctattgagcaggataaaccattttgagaaaatacagatcatccccaaagattcttggtcagatcatgaagcctcattgggggtgagcgcagcatgtccataccagggaaattcctccttcttcagaaatgtggtatggcttatcaaaaaggacaatgcatatccaacaataaagaaaggctacaataataccaaccaagaagatctcttggtactgtgggggattcaccatcctaatgatgaggcagagcagacaaggctctatcaaaacccaaccacctatatttccattgggacatcaacactaaaccagagattggtaccaaaaatagccactagatccaaagtaaacgggcaaagtggcaggatagatttcttctggacaattttaaaaccgaatgatgcaatccacttcgagagtaatggaaatttcattgctccagaatatgcatacaaaattgtcaagaaaggagactccacaattatgaaaagtgaagtggaatatggtaactgcaacaccaggtgtcagactccgataggggcgataaactctagtatgccattccacaacatacaccctctcaccatcggagaatgtcccaaatatgtgaaatcaaacaaattagtccttgcgactgggctcagaaatagtcctcaaagagagagaagaagaaaaGGGagaggactgtttggagctatagcaggttttatagagggaggatggcagggaatggtagatggttggtatgggtaccaccacagcaatgagcaggggagtgggtacgctgcagacaaagaatctactcaaaaggcaatagacggagtcaccaataaggtcaactcgatcattgacaaaatgaacactcagtttgaggccgtaggaagggaatttaataacttagagaggagaatagagaatttaaacaagaagatggaagacggattcctagatgtctggacttataatgctgaacttctggttctcatggaaaatgagagaactctagacttccatgactcaaatgtcaagaacctttacgataaggtcagactacagcttaaggataatgcaaaagagttgggtaacggttgtttcgagttctatcacaaatgtaataatgaatgtatggaaagtgtaagaaacggaacgtatgactacccgcagtattcagaagaagcaagattaaaaagagaggaaataagtggagtaaaattggaatcaataggaatctaccaaatactgtcaatttattcaacagtggcgagttccctagtgctggcaatcatgatggctggtctatctttatggatgtgttccaacgggtcgttacagtgcagaatttgcatttga

>H5N1_A/buzzard/Ireland/000656_22VIR1325-12/2022

cttgttaaaagtgatcagatttgcattggttaccatgcaaacaattcgacagagcaggttgacacgataatggaaaagaatgtcactgttacacatgcccaagacatactggaaaaaacacacaacgggaagctctgtgatttaaatggggtgaagcctctgattttaaaggattgtagtgtagctggatggctcctcggaaacccaatgtgcgacgaattcatcagagtgccggaatggtcctacatagtggagcgggctaatccagccaatgacctctgttacccagggagcctcaatgactatgaagaactgaaacacctgttgagcagaataaatcattttgagaagattctgatcatccccaagagttcctggccaaatcatgaaacatcactaggggtgagcgcagcttgtccataccagggagcgccctcctttttcagaaatgtgttgtggcttatcaaaaagaacgatgcatacccaacaataaagataagctacaataataccaatcgggaagatctcttgatattgtgggggattcatcattccaacaatgcagaagagcagacaaatctctataaaaacccaaccacctacatttcagttggaacatcaactttaaaccagaggttggtaccaaaaatagctactagatcccaagtaaacgggcaacgtggaagaatggacttcttctggacaattttaaaaccagatgatgcaatccatttcgagagtaatggaaatttcattgcaccagaatatgcatataaaattgtcaagaaaggggactcaacaattatgaaaagtggagtggaatatggccactgcaacaccaaatgtcaaaccccagtaggagcgataaattctagtatgccattccacaacatacatcctctcaccattggggaatgccccaaatacgtgaagtcaaacaagttggtccttgcgactgggctcagaaatagtcctctaagagaaaagagaagaaaaGGGagaggcctgtttggggcgatagcagggtttatagagggaggatggcagggaatggttgatggttggtatggrtaccatcatagcaatgagcaggggagtgggtacgctgcagacaaagaatccacccaaaaggcaatagatggagttaccaataaggtcaactcaatcattgacaaaatgaacactcaatttgaggcagttggaagggagtttaataacttagaaaggaggatagagaatttgaacaagaaaatggaagacggattcctagatgtctggacctataatgctgaacttctagttctcatggaaaacgagaggactctagatttccatgattcaaatgtcaagaacctttacgacaaagtcagactacagcttagggacaatgcaaaggagctgggtaatggctgtttcgaattctatcacaaatgcgataatgaatgtatggaaagtgtgagaaatgggacgtatgactaccctcagtattcagaagaagcaagattaaaaagagaagaaataagcggagtgaaattagaatcaataggaacttaccagatactgtcaatttattcaacagcggcgagttccctagcactggcaatcatgatagctggtctatctttatggatgtgctccaatgggtcgttacagtgcagaatttgcatttag

>H5N1_A/buzzard/Italy/21VIR11899-5/2021

cttgttaaaagtgatcagatttgcattggttaccatgcaaacaattcgacagagcaggttgacacgataatggaaaagaacgtcactgttacacatgcccaagacatactggaaaaaacacacaacgggaagctctgtgatttaaatggggtgaagcctctggttttaaaggattgtagtgtagctggatggctcctcggaaacccaatgtgcgacgaattcatcagagtgccggaatggtcctacatagtggagcgggctaatccagctaatgacctctgttacccagggagcctcaatgactatgaagaactgaaacacctgttgagcagaataaatcattttgagaagattctgatcatccccaagagttcctggccaaatcatgaaacatcactaggggtgagcgcagcttgtccataccagggagcgccctcctttttcagaaatgtggtgtggcttatcaaaaagaacgatgcatacccaacaataaagataagctacaataataccaatcgggaagatctcttgatactgtgggggattcatcattccaacaatgcagaagagcagacaaatctctataaaaacccaaccacctacatttcagttggaacatcaactttaaaccagaggttggtaccaaaaatagctactagatcccaagtaaacgggcaacgtggaagaatggacttcttctggacaattttaaaaccagatgatgcaatccatttcgagagtaatggaaatttcattgctccagaatatgcatataaaattgtcaagaaaggggactcaacaattatgaaaagtggagtggaatatggccactgcaacaccaaatgtcaaaccccagtaggagcgataaattctagtatgccattccacaacatacatcctctcaccattggggaatgccccaaatacgtgaagtcaaacaagttggtccttgcgactgggctcagaaatagtcctctaagagaaaagagaagaaaaGGGagaggcctgtttggggcgatagcagggtttatagagggaggatggcagggaatggttgatggttggtatgggtatcatcatagcaatgagcaggggagtgggtacgctgcagacaaagaatccacccaaaaggcaatagatggagttaccaataaggtcaactcaatcattgacaaaatgaacactcaatttgaggcagttggaagggagtttaataacttagaaaggaggatagaaaatttgaacaagaaaatggaagacggattcctagatgtctggacctataatgctgaacttctagttctcatggaaaacgagaggactctagatttccatgattcgaatgtcaagaacctttacgacaaagtcagactacagcttagggataatgcaaaggagctgggtaatggctgtttcgaattctatcacaaatgcgataatgaatgtatggaaagtgtgagaaatgggacgtatgactaccctcagtattcagaagaagcaagattaaaaagagaagaaataagcggagtgaaattagaatcaataggaacttaccagatactgtcaatttattcaacagcggcgagttccctagcactggcaatcatgatagctggtctatctttatggatgtgctccaatgggtcgttacagtgcagaatttgcatttag

>H5N1_A/chicken/Afghanistan/1207/2006

cttgttaaaagtgatcagatttgcattggttaccatgcaaacaactcgacagagcaggttgacacaataatggaaaagaacgtcactgttacacacgcccaagacatactagaaaagacacacaacgggaagctctgcgatctagacggagtgaagcctctaattttaagagattgtagtgtagctggatggctcctcgggaatccaatgtgtgacgaattcctcaatgtgccggaatggtcttacatagtggagaagatcaatccagccaatgacctctgttacccagggaatttcaacgactatgaagaactgaaacacctattgagcagaataaaccattttgagaaaattcagatcatccccaaaagttcttggtcagatcatgaagcatcatcaggggtgagctcagcatgtccataccagggaaggtcctccttttttagaaatgtggtatggcttatcaaaaagaacgatgcatacccaacaataaagagaagttacaataataccaaccaagaagatcttttggtactgtgggggattcaccatccaaatgatgcggcagagcagacaaggctctatcaaaacccaaccacctatatttccgttgggacatcaacactaaaccagagattggtaccaaaaatagctactagatccaaggtaaacgggcaaagtggaaggatggagttcttttggacaattttaaaaccgaatgatgcaataaactttgagagtaatggaaatttcattgctccagaaaatgcatacaaaattgtcaagaaaggggactcaacaatcatgaaaagtgaattggaatatggtaactgcaacaccaagtgtcaaactccaataggggcgataaactctagtatgccattccacaacatccaccctctcaccatcggggaatgccccaaatatgtgaaatcaaacagattagtccttgcgactgggctcagaaatagccctcaaggagagagaagaagaaaaaagagaggactatttggagctatagcaggttttatagagggaggatggcagggaatggtagatggttggtatgggtaccaccatagcaacgagcaggggagtgggtacgctgcagacaaagaatccactcaaaaggcaatagatggagtcaccaataaggtcaactcgatcattgacaaaatgaacactcagtttgaggccgttggaagggaatttaataacttagaaaggagaatagaaaatttaaacaagaagatggaagacggatttctagatgtctggacttataatgctgaacttctggttctcatggaaaatgagagaactctagactttcatgactcaaatgtcaagaacctttacgacaaggtccgactacagcttagggataatgcaaaggagcttggtaacggttgtttcgagttctatcacagatgtgataatgaatgtatggaaagtgtaagaaacggaacgtatgactacccgcagtattcagaagaagcaagattaaaaagagaggaaataagtggagtaaaattggaatcaataggaacttatcaaatactgtcaatttattcaacagtggcgagctccctagcactggcaatcatggtggctggtctatctttatggatgtgctccaatggatcgttacaatgcagaatttgcatttga

>H5N1_A/chicken/Afghanistan/1573-47/2006

cttgttaaaagtgatcagatttgcattggttaccatgcaaacaactcgacagagcaggttgacacaataatggaaaagaacgtcactgttacacacgcccaagacatactggaaaagacacacaacgggaagctctgcgatctagacggagtgaagcctctaattttaagagattgtagtgtagctggatggctcctcgggaatccaatgtgtgacgaattcctcaatgtgccggaatggtcttacatagtggagaagatcaatccagccaatgacctctgttacccagggaatttcaacgactatgaagaactgaaacacctattgagcagaataaaccattttgagaaaattcagatcatccccaaaagttcttggtcagatcatgaagcctcatcaggggtgagctcagcatgtccataccagggaaggtcctccttttttagaaatgtggtatggcttatcaaaaagaacgatgcatacccaacaataaagagaagttacaataataccaaccaagaagatcttttggtactgtgggggattcaccatccaaatgatgcggcagagcagacaaggctctatcaaaacccaaccacctatatttccgttgggacatcaacactaaaccagagattggtaccaaaaatagctactagatccaaggtaaacgggcaaagtggaaggatggagttcttttggacaattttaaaaccgaatgatgcaataaactttgagagtaatggaaatttcattgctccagaaaatgcatacaaaattgtcaagaaaggggactcaacaatcatgaaaagtgaattggaatatggtaactgcaacaccaagtgtcaaactccaataggggcgataaactctagtatgccattccacaacatccaccctctcaccatcggggaatgccccaaatatgtgaaatcaaacagattagtccttgcgactgggctcagaaatagccctcaaggagagagaagaagaaaaaagagaggactatttggagctatagcaggttttatagagggaggatggcagggaatggtagatggttggtatgggtaccaccatagcaacgagcaggggagtgggtacgctgcagacaaagaatccactcaaaaggcaatagatggagtcaccaataaggtcaactcgatcattgacaaaatgaacactcagtttgaggccgttggaagggaatttaataacttagaaaggagaatagaaaatttaaacaagaagatggaagacggatttctagatgtctggacttataatgctgaacttctggttctcatggaaaatgagagaactctagactttcatgactcaaatgtcaagaacctttacgacaaggtccgactacagcttagggataatgcaaaggagcttggtaacggttgtttcgagttctatcacagatgtgataatgaatgtatggaaagtgtaagaaacggaacgtatgactacccgcagtattcagaagaagcaagattaaaaagagaggaaataagtggagtaaaattggaatcaataggaacttatcaaatactgtcaatttattcaacagtggcgagctccctagcactggcaatcatggtggctggtctatctttatggatgtgctccaatggatcgttacaatgcagaatttgcatttga

>H5N1_A/chicken/Afghanistan/1573-65/2006

cttgttaaaagtgatcagatttgcattggttaccatgcaaacaactcgacagagcaggttgacacaataatggaaaagaacgtcactgttacacatgcccaagacatactggaaaagacacacaacgggaagctctgcgatctagacggagtgaagcctctaattttaagagattgtagtgtagctggatggctcctcgggaatccaatgtgtgacgaattcctcaatgtgccggaatggtcttacatagtggagaagatcaatccagccaatgacctctgttacccagggaatttcaacgactatgaagaactgaaacacctattgagcagaataaaccattttgagaaaattcagatcatccccaaaagttcttggtcagatcatgaagcctcatcaggggtgagctcagcatgtccataccagggaaggtcctccttttttagaaatgtggtatggcttatcaaaaagaacgatgcatacccaacaataaagagaagttacaataataccaaccaagaagatcttttggtactgtgggggattcaccatccaaatgatgcggcagagcagacaaggctctatcaaaacccaaccacctatatttccgttgggacatcaacactaaaccagagattggtaccaaaaatagctactagatccaaggtaaacgggcaaagtggaaggatggagttcttttggacaattttaaaaccgaatgatgcaataaactttgagagtaatggaaatttcattgctccagaaaatgcatacaaaattgtcaagaaaggggactcaacaatcatgaaaagtgaattggaatatggtaactgcaacaccaagtgtcaaactccaataggggcgataaactctagtatgccattccacaacatccaccctctcaccatcggggaatgccccaaatatgtgaaatcaaacagattagtccttgcgactgggctcagaaatagccctcaaggagagagaagaagaaaaaagagaggactatttggagctatagcaggttttatagagggaggatggcagggaatggtagatggttggtatgggtaccaccatagcaacgagcaggggagtgggtacgctgcagacaaagaatccactcaaaaggcaatagatggagtcaccaataaggtcaactcgatcattgacaaaatgaacactcagtttgaggccgttggaagggaatttaataacttagaaaggagaatagaaaatttaaacaagaagatggaagacggatttctagatgtctggacttataatgctgaacttctggttctcatggaaaatgagagaactctagactttcatgactcaaatgtcaagaacctctacgacaaggtccgactacagcttagggataatgcaaaggagcttggtaacggttgtttcgagttctatcacagatgtgataatgaatgtatggaaagtgtaagaaacggaacgtatgactacccgcagtattcagaagaagcaagattaaaaagagaggaaataagtggagtaaaattggaatcaataggaacttatcaaatactgtcaatttattcaacagtggcgagctccctagcactggcaatcatggtggctggtctatctttatggatgtgctccaatggatcgttacaatgcagaatttgcatttga

>H5N1_A/chicken/Afghanistan/1573-7/2006

cttgttaaaagtgatcagatttgcattggttaccatgcaaacaactcgacagagcaggttgacacaataatggaaaagaacgtcactgttacacacgcccaagacatactggaaaagacacacaacgggaagctctgcgatctagacggagtgaagcctctaattttaagagattgtagtgtagctggatggctcctcgggaatccaatgtgtgacgaattcctcaatgtgccggaatggtcttacatagtggagaagatcaatccagccaatgacctctgttacccagggaatttcaacgactatgaagaactgaaacacctattgagcagaataaaccattttgagaaaattcagatcatccccaaaagttcttggtcagatcatgaagcctcatcaggggtgagctcagcatgtccataccagggaaggtcctccttttttagaaatgtggtatggcttatcaaaaagaacgatgcatacccaacaataaagagaagttacaataataccaaccaagaagatcttttggtactgtgggggattcaccatccaaatgatgcggcagagcagacaaggctctatcaaaacccaaccacctatatttccgttgggacatcaacactaaaccagagattggtaccaaaaatagctactagatccaaggtaaacgggcaaagtggaaggatggagttcttttggacaattttaaaaccgaatgatgcaataaactttgagagtaatggaaatttcattgctccagaaaatgcatacaaaattgtcaagaaaggggactcaacaatcatgaaaagtgaattggaatatggtaactgcaacaccaagtgtcaaactccaataggggcgataaactctagtatgccattccacaacatccaccctctcaccatcggggaatgccccaaatatgtgaaatcaaacagattagtccttgcgactgggctcagaaatagccctcaaggagagagaagaagaaaaaagagaggactatttggagctatagcaggttttatagagggaggatggcagggaatggtagatggttggtatgggtaccaccatagcaacgagcaggggagtgggtacgctgcagacaaagaatccactcaaaaggcaatagatggagtcaccaataaggtcaactcgatcattgacaaaatgaacactcagtttgaggccgttggaagggaatttaataacttagaaaggagaatagaaaatttaaacaagaagatggaagacggatttctagatgtctggacttataatgctgaacttctggttctcatggaaaatgagagaactctagactttcatgactcaaatgtcaagaacctttacgacaaggtccgactacagcttagggataatgcaaaggagcttggtaacggttgtttcgagttctatcacagatgtgataatgaatgtatggaaagtgtaagaaacggaacgtatgactacccgcagtattcagaagaagcaagattaaaaagagaggaaataagtggagtaaaattggaatcaataggaacttatcaaatactgtcaatttattcaacagtggcgagctccctagcactggcaatcatggtggctggtctatctttatggatgtgctccaatggatcgttacaatgcagaatttgcatttga

>H5N1_A/chicken/Afghanistan/1573-92/2006

cttgttaaaagtgatcagatttgcattggttaccatgcaaacaactcgacagagcaggttgacacaataatggaaaagaacgtcactgttacacacgcccaagacatactggaaaagacacacaacgggaagctctgcgatctagacggagtgaagcctctaattttaagagattgtagtgtagctggatggctcctcgggaatccaatgtgtgacgaattcctcaatgtgccggaatggtcttacatagtggagaagatcaatccagccaatgacctctgttacccagggaatttcaacgactatgaagaactgaaacacctattgagcagaataaaccattttgagaaaattcagatcatccccaaaagttcttggtcagatcatgaagcctcatcaggggtgagctcagcatgtccataccagggaaggtcctccttttttagaaatgtggtatggcttatcaaaaagaacgatgcatacccaacaataaagagaagttacaataataccaaccaagaagatcttttggtactgtgggggattcaccatccaaatgatgcggcagagcagacaaggctctatcaaaacccaaccacctatatttccgttgggacatcaacactaaaccagagattggtaccaaaaatagctactagatccaaggtaaacgggcaaagtggaaggatggagttcttttggacaattttaaaaccgaatgatgcaataaactttgagagtaatggaaatttcattgctccagaaaatgcatacaaaattgtcaagaaaggggactcaacaatcatgaaaagtgaattggaatatggtaactgcaacaccaagtgtcaaactccaataggggcgataaactctagtatgccattccacaacatccaccctctcaccatcggggaatgccccaaatatgtgaaatcaaacagattagtccttgcgactgggctcagaaatagccctcaaggagagagaagaagaaaaaagagaggactatttggagctatagcaggttttatagagggaggatggcagggaatggtagatggttggtatgggtaccaccatagcaacgagcaggggagtgggtacgctgcagacaaagaatccactcaaaaggcaatagatggagtcaccaataaggtcaactcgatcattgacaaaatgaacactcagtttgaggccgttggaagggaatttaataacttagaaaggagaatagaaaatttaaacaagaagatggaagacggatttctagatgtctggacttataatgctgaacttctggttctcatggaaaatgagagaactctagactttcatgactcaaatgtcaagaacctttacgacaaggtccgactacagcttagggataatgcaaaggagcttggtaacggttgtttcgagttctatcacagatgtgataatgaatgtatggaaagtgtaagaaacggaacgtatgactacccgcagtattcagaagaagcaagattaaaaagagaggaaataagtggagtaaaattggaatcaataggaacttatcaaatactgtcaatttattcaacagtggcgagctccctagcactggcaatcatgatggctggtctatctttatggatgtgctccaatggatcgttacaatgcagaatttgcatttga

>H5N1_A/chicken/Aichi/2/2011

cttgttaaaagcgatcatatttgcattggttatcatgcaaataactcgacagagcaggttgacacaataatggaaaagaacgttactgttacacatgcccaagacatactggaaaagacacacaacgggaagctctgcaatctaaatggagtgaagcctctgattttaaaagattgtagtgtagcgggatggctcctcggaaacccattgtgtgacgaattcatcaatgtgccagaatggtcttacatagtagagaaggccaagccagccaatgacctctgttacccagggaatttcaacgattatgaagaattgaaacacctattgagcaggataaaccattttgagaaaatacagatcatccccaaagactcttggtcagatcatgaagcctcattgggggtgagcgcagcatgttcataccagggaaattcctccttcttcagaaatgtggtatggcttatcaaaaaggacaatgcatacccaacaataaagaaaggctacaataataccaaccaagaagatctcttggtactgtgggggattcaccatcctaatgatgaggcagagcagacaaggctctatcaaaacccaaccacctatatttccattgggacatcaacactaaaccagagattggtaccaaaaatagccactagatccaaaataaacgggcaaagtggcaggatagatttcttctggacaattttaaaaccgaatgatgcaatccacttcgagagtaatggaaatttcattgctccagaatatgcatacaaaattgtcaagaaaggagactccacaattatgaaaagtgaagtggaatatggtaactgcaacaccaggtgtcagactccgataggggcgataaactctagtatgccattccacaacatacaccctctcaccatcggagaatgtcccaaatatgtgaaatcaaacaaattagtccttgcgactgggctcagaaatagtcctcaaagagagagaagaagaaaaGGGagaggactgtttggagctatagcaggttttatagagggaggatggcagggaatggtagatggttggtatgggtaccaccacagcaatgagcaggggagtgggtacgctgcagacaaagaatctactcaaaaggcaatagacggagtcaccaataaggtcaactcgatcattgacaaaatgaacactcagtttgaggccgtaggaagggaatttaataacttagagaggagaatagagaatttaaacaagaagatggaagacggattcctagatgtttggacttataatgctgaacttctggttctcatggaaaatgagagaactctagatttccatgactcaaatgtcaagaacctttacgataaggtcagactacagcttaaggataatgcaaaagagttgggtaacggttgtttcgagttctatcacaaatgtaataatgaatgtatggaaagtgtaagaaacggaacgtatgactacccgcagtattcagaagaagcaagactaaaaagagaggaaataagtggagtaaaattggaatcaataggaatctaccaaatactgtcaatttattcaacagtggcgagttccctagtgctggcaatcatgatggctggtctgtctttatggatgtgttccaacggatcgttacagtgcagaatttgcatttga

>H5N1_A/chicken/Aichi/T1/2011

cttgttaaaagcgatcatatttgcattggttatcatgcaaataactcgacagagcaggttgacacaataatggaaaagaacgttactgttacacatgcccaagacatactggaaaagacacacaacgggaagctctgcgatctaaatggagtgaagcctctgattttaaaagattgtagtgtagcgggatggctcctcggaaacccattgtgtgacgaattcatcaatgtgccagaatggtcttacatagtagagaaggccaatccagccaatgacctctgttacccagggaatttcaacgattatgaagaattgaaacacctattgagcaggataaaccattttgagaaaatacggatcatccccaaagactcttggtcagatcatgaagcctcattgggggtgagcgcagcatgttcataccagggaaattcctccttcttcagaaatgtggtatggcttatcaaaaaggacaatgcatacccaacaataaagaaaggctacaataataccaaccaagaagatctcttggtactgtgggggattcaccatcctaatgatgaggcagagcagacaaggctctatcaaaacccaaccacctatatttccattgggacatcaacactaaaccagagattggtaccaaaaatagccactagatccaaaataaacgggcaaagtggcaggatagatttcttctggacaattttaaaaccgaatgatgcaatccacttcgagagcaatggaaatttcattgctccagaatatgcatacaaaattgtcaaaaaaggagactccacaattatgaaaagtgaagtggaatatggtaactgcaacaccaggtgtcagactccgataggggcgataaactctagtatgccattccacaacatacaccctctcaccatcggagaatgtcccaaatatgtgaaatcaaacaaattagtccttgcgactgggctcagaaatagtcctcaaagagagagaagaagaaaaGGGagaggactgtttggagctatagcaggttttatagagggaggatggcagggaatggtagatggttggtatgggtaccaccacagcaatgagcaggggagtgggtacgctgcagacaaagaatctactcaaaaggcaatagacggagtcaccaataaggtcaactcgatcattgacaaaatgaacactcagtttgaagccgtaggaagggaatttaataacttagagaggagaatagagaatttaaacaagaagatggaagacggattcctagatgtttggacttataatgctgaacttctggttctcatggaaaatgagagaactctagatttccatgactcaaatgtcaagaacctttacgataaggtcagactacagcttaaggataatgcaaaagagttgggtaacggttgtttcgagttctatcacaaatgtaataatgaatgtatggaaagtgtaagaaacggaacgtatgactacccgcagtattcagaagaagcaagactaaaaagagaggaaataagtggagtaaagttggaatcaataggaatctaccaaatactgtcaatttattcaacagtggcgagttccctagtgctggcaatcatgatggctggtctgtctttatggatgtgttccaacggatcgttacagtgcagaatttgcatttga

>H5N1_A/chicken/Anhui/39/2004

cttgttaaaagtgatcagatctgcattggttaccatgcaaacaactcgacagagcaggttgacacaataatggaaaagaatgttactgttacacatgcccaagatatactggaaaagacacacaacgggaagctctgcgacctagatggagtgaagcctctaattttgagagattgtagtgtagctggatggctcctcgggaatccaatgtgtgacgaatttatcaatgtaccggaatggtcttacatagtggagaaggccagtccagccaatgacctctgttacccaggggatttcaacgactatgaagaactgaaacacctattgagcagaataaaccattttgagaaaattcagatcatccccaaaagttcttggtccaatcatgaagcctcatcaggggtgagctcagcatgtccataccatgggaagccctcctttttcagaaatgtggtatggcttatcaaaaagaacaatacatacccaacaataaagaggagctacaataataccaaccaagaagatcttttggtactgtgggggattcaccatcctaatgatgcggcagagcagacaaagctctatcaaaacccaaccacctatatttccgttggaacatcgacactaaaccagagattggtaccaaaaatagctactaaaaccaaagtaaacgggcaaagtggaaggatggagttcttctggacaattttaaaaccgaatgatgcaatcaacttcgagagtaatggaaatttcattgctccagaatatgcatacaaaattgtcaagaaaggggactcagcaattatgaaaagtgaattggaatatggtaactgcaacaccaagtgtcaaactccaatgggggcgataaactctagtatgccattccacaacatacaccctctcaccatcggggaatgccccaaatatgtgaaatcaaacagattagtccttgcaactgggctcagaaatagcccccaaagagagggaagaagaaaaaagagaggactatttggagctatagcaggttttatagagggaggatggcaaggaatggtaaatggttggtatggataccatcatagcaatgagcaggggagtgggtacgctgcagacaaagaatccactcaaaaggcaatagatggagtcaccaacaaggtcaactcgatcattgacaaaatgaacactcagtttgaggccgttggaagggaatttaataacttagaaaggagaatagagaatttaaacaagaagatggaagacggattcctagatgtctggacttataatgctgaacttctggttctcatggaaaatgagagaactctagactttcatgactcaaatgtcaagaacctttacgacaaggtccgactacagcttagggataatgcaaaagagctgggtaacggttgtttcgagttctatcacaaatgtgataatgaatgtatggaaagtgtaagaaacggaacgtatgactacccgcagtattcagaagaagcaagactaaaaagagaggaaataagtggagtaaaatcggaatcaataggaacttaccaaatactgtcaatttattcaacagtggcgagttccctagcactggcaatcatggtagctggtctatctttatggatgtgctccaatggttcgttacaatgcagaatttgcatttga

>H5N1_A/chicken/Anhui/QD1/2014

cttgttaaaagtgatcagatttgcattggttaccatgcaaataactcgacagagcaggttgacacgataatggaaaaaaacgtcactgttacacatgcccaagacatactggaaaagacacacaacgggaggctctgcgatctgaatggagtgaaacctctgattttaaaggattgtagtgtagctggatggctccttggaaacccaatgtgcgacgagttcatcagagtgccggaatggtcttacatagtggagagggctaatccatccaatgacctctgttacccagggaacctcaatgactatgaagaactgaaacacctattgagcagaataaatcattttgagaagactctgatcatccccaagagttcttggcccgatcatgaaacatcattaggggtgagcgcagcatgtccataccagggaatgccctcctttttcagaaatgtggtatggcttatcaagaagaacgatacatacccaacaataaagatgagctacaataataccaatagggaagatcttttgatactgtgggggattcatcattccaacaacgcagcagagcagacaaatctctataaaaacccaaccacctatgtttccgttgggacatcaacattaaaccagagattggtgcccaaaatagctactagatcccaagtaaacgggcaacgtggaagaatggatttcttctggacaattttaaaaccgaatgatgcaatccacttcgagagtaatggaaattttattgctccagaatatgcatacaaaattgtcaagaaaggggactcaacaatcatgaaaagtgaaatggagtatggccactgcaacaccaaatgtcaaactccaataggggcgataaactctagtatgccattccacaatatacaccctctcaccatcggggaatgccccaaatacgtgaaatcaaacaaattagtccttgcgactgggctcagaaatagtcctctaagagagaggagaagaaaaGGGagaggactatttggagctatagcagggtttatagagggaggatggcaaggaatggtagatggttggtatgggtaccaccatagcaatgaacaggggagtgggtacgctgcagacaaagaatccacccaaaaggcaatagatggagttaccaataaggtcgactcgatcattgacaagatgaacactcaatttgaggccgttggaagggaatttaataacttagaacggagaatagagaatttaaataagaaaatggaagacggattcctagatgtctggacttataatgctgaacttctagttctcatggaaaatgagagaactctagatttccatgactcaaatgtcaagaacctttacgacaaagtccgactacagcttagggataatgcaaaggagctgggtaacggttgtttcgagttctatcacaaatgtgataatgaatgtatggaaagtgtaagaaatgggacgtatgactaccctcagtattcagaagaagcaagattaaaaagagaagaaataagcggagtgaaattggaatcaataggaacttaccaaatactgtcaatttattcaacagtggcgagttccctagcactggcaatcattgtggctggtctatctttatggatgtgctccaatgggtcgttacaatgcagaatttgcatttga

>H5N1_A/chicken/BC/FAV2/2015

cttgccaaaagtgatcagatttgcattggttaccatgcaaacaactcaacaaagcaggttgacacgataatggagaaaaacgtcactgttacacatgcccaagacatactggaaaagacacacaacgggaagctctgcgatcttaatggagtgaagcccctgattctaaaggattgtagcgtagctgggtggctccttggaaatccaatgtgcgacgagtttatcagggtaccggaatggtcttacatcgtggagagggctaacccagccaacgacctctgttacccagggactctcaatgactatgaggaactgaaacacctattgagcagaataaatcattttgagaaaactctgatcatccccaggagttcttggcccaatcacgaaacatcattaggggtgagcgcagcatgtccataccagggagcatcctcatttttcagaaatgtggtatggctcatcaaaaagaacgatgcatacccgacaataaagataagctacaataataccaatcgggaagatcttttgatactgtgggggattcatcattccaacaatgcagcagagcagacaaatctttataaaaacccagacacttatgtttccgttgggacatcaacattaaaccagagattggtgccaaaaatagctactagatcccaagtaaacgggcagagtggaagaatggatttcttctggacaattttaaaaccgaatgatgcaatccactttgagagtaatggaaatttcattgctccagaatatgcatacaaaattgtcaagaaaggggactcaacaattatgaaaagtgaaatggagtatggccactgcaacaccaaatgtcaaactccaataggggctataaactctagcatgccattccacaatatacaccctctcaccatcggggaatgccccaaatacgtgaagtcaaacaaattagtccttgcgactgggctcagaaatagtcctctaagagaaagaagaagaaaaGGGagaggactatttggagctatagcagggtttatagagggaggatggcagggaatggtagacggttggtatgggtatcatcatagcaatgagcaggggagtgggtacgctgcagacaaagaatccacccaaaaggcaatagatggagttaccaataaggtcaactcaatcattgacaaaatgaacactcaatttgaggccgttggaagggaatttaataacttagaaaggagaatagagaatttaaacaagaaaatggaagacggattcctagatgtctggacttataatgctgaacttttagttctcatggaaaatgagagaactctagatttccatgactcaaacgtcaagaacctttacgacaaagtccgactacagcttagggataatgcaaaggagctgggtaatggttgtttcgagttctatcacaaatgtgataacgaatgtatggagagcgtaagaaatgggacgtatgactaccctaagtattcagaagaagcaatattaaaaagagaagaaataagcggagtgaaattagaatcaataggaacttaccagatactgtcaatttattcaacagtggcgagttccctagcactggcaatcatagtggctggtttatctttatggatgtgctctaatgggtcgttacaatgcagaatttgcatctga

>H5N1_A/chicken/Bac Lieu/07-10/2007

cttgttaaaagtgatcagatttgcattggttaccatgcaaacaactcgacagagcaggttgacacaataatggaaaagaacgttactgttacacatgcccaagacatactggaaaagacacataacgggaagctctgtgatctagatggagtgaagcctctaattttgagagattgtagtgtagctggatggcttctcggaaacccaatgtgtgacgagttcatcaatgtgccggaatggtcttacatagtggagaaggccaatccagtcaatgacctctgttacccaggagttttcaatgactatgaagaattgaaacacctattgagcagaataaaccattttgagaaaattcagatcatccccaaaagttcttggcccagtcatgaagcctcattgggggtgagcgcagcatgtccataccagggaaagtcctcttttttcagaaatgtggtatggcttatcaaaaagaacagtacatacccaacaataaagaggagctacaataataccaaccaagaagatcttttggtaatgtgggggatccaccatcctaatgatgcggcagagcagacaaagctctatcaaaatccaaccacctatatctccgttgggacatcaacactaaaccagagattgacaccaagaatagctactagatccaaagtaaacgggcaaagtgggaggatggagttcttctggacaattttaaaaccaaatgatgcaatcaacttcgagagtaatggaaatttcattgctccagaatatgcatacaaaattgtcaagaaaggggactcaacaattatgaaaagtgaattggaatatggtaactgcaacaccaagtgtcaaactccaatgggggcgataaactctagtatgccattccacaatatacatcctctcactattggggaatgccccaaatatgtgaaatcaaacagattagtccttgcgactgggctcagaaatagccctcaaagagagggaagaagaaaaaagagaggattatttggagctatagcaggttttatagagggaggatggcagggaatggtagatggttggtatgggtaccaccatagcaatgagcaggggagtgggtacgctgcagacaaagaatccactcaaaaggctatagatggagtcaccaataaggtcaactcgatcattgacaaaatgaacactcagtttgaggccgttggaagggaatttaacaacttagaaagaagaatagagaatttaaacaagaagatggaagacgggttcctagatgtctggacttataatgctgaacttctggttctcatggaaaatgagagaactctagacttccatgactcaaatgtaaagaacctttacgacaaggtccgattacagctcagggataatgcaaaggagctgggtaacggttgtttcgagttctatcacaaatgtgataatgaatgtatggaaagtgtgagaaacgggacgtatgactacccgcagtattcagaagaagcaagattaaaaagagaggaaataagtggagtgaaattggaatcgataggaatttaccaaatactgtcaatttattctacagtggcgagttccctagcactggcaatcatggtagctggtctatccttatggatgtgctccaatgggtcgttacaatgcagaatttgcatttga

>H5N1_A/chicken/Bac Lieu/1214/2007

cttgttaaaagtgatcagatttgcattggttaccatgcaaacaactcgacagagcaggttgacacaataatggaaaagaacgttactgttacacatgcccaagacatactggaaaagacacataacgggaagctctgtgatctggatggagtgaagcctctaattttgagagattgtagtgtagctggatggcttctcggaaacccaatgtgtgacgaattcatcaatgtgccggaatggtcttacatagtggagaaggccaatccagtcaatgacctctgttacccaggagttttcaatgactatgaagaattgaaacacctattgagcagaataaaccattttgagaaaattcagatcatccccaaaagttcttggcccagtcatgaagcctcattgggggtgagcgcagcatgtccataccagggaaagtcctcttttttcagaaatgtggtatggcttatcaaaaagggcagtacatacccaacaataaagaggagttacaataataccaaccaagaagatcttttggtaatgtgggggatccaccatcctaatgatgcggcagagcagacaaagctctatcaaaatccaaccacctatatctccgttgggacatcaacactaaaccagagattgacaccaagaatagctactagatccaaagtaaacgggcaaagtgggaggatggagttcttctggacaattttaaaaccgaatgatgcaatcaacttcgagagtaatggaaatttcattgctccagaatatgcatacaaaattgtcaagaaaggggactcaacaattatgaaaagtgaattggaatatggtaactgcaacaccaagtgtcaaactccaatgggggcgataaactctagtatgccattccacaatatacatcctctcactattggggaatgccccaaatatgtgaaatcaaacagattagtccttgcgactgggctcagaaatagccctcaaagagagggaagaagaaaaaagagaggattatttggagctatagcaggttttatagagggaggatggcagggaatggtagatggttggtatgggtaccaccatagcaatgagcaggggagtgggtacgctgcagacaaagaatccactcaaaaggctatagatggagtcaccaataaggtcaactcgatcattgacaaaatgaacactcagtttgaggccgttggaagggaatttaacaacttagaaagaagaatagagaatttaaacaagaagatggaagacgggttcctagatgtctggacttataatgctgaacttctggttctcatggaaaatgagagaactctagacttccatgactcaaatgtaaagaacctttacgacaaggtccgattacagcttagggataatgcaaaggagctgggtaacggttgtttcgagttctatcacaaatgtgataatgaatgtatggaaagtgtgagaaacgggacgtatgactacccgcagtattcagaagaagcaagattaaaaagagaggaaataagtggagtgaaattggaatcgataggaatttaccaaatactgtcaatttattctacagtggcgagttccctagcactggcaatcatggtagctggtctatccttatggatgtgctccaatgggtcgttacaatgcagaatttgcatttga

>H5N1_A/chicken/Bali/U8661/2009

cttgttaaaagtgatcagatttgcattggttatcatgcaaacaattcaacagagcaggttgacacaatcatggaaaagaacgttactgttacacatgcccaagacatactggaaaagacacacaacgggaagctctgcgatctagatggagtgaagcctctaattttaagagattgtagtgtagctggatggctcctcgggaacccaatgtgtgacgaattcatcaatgtaccggaatggtcttacatagtggagaaggccaatccaaccaatgacctctgttacccagggagtttcaacgactatgaagaactgaaacatctattgagcagaataaaccattttgaaaaaattcaaatcatccccaaaaattcttggtccgatcatgaagcctcatcaggagtgagcgcagcatgtccatacctgggaagtccctccttttttagaaatgtggtatggcttatcaaaaagaacagtacatacccaacaataaagaagagctacaataataccaaccaagaagatcttttggtactgtggggaattcaccatcctaatgatgcggcagagcagacaaggctatatcaaaacccaaccacctatatttccattgggacatcaacactaaaccagagattggtgccaaaaatagctactagatccaaagtaaacgggcaaagtggaaggatggagttcttctggacaattttaaaacctaatgacgcaatcaatttcgagagtaatggaaatttcattgctccagaatatgcatacaaaattgtcaaaaaaggggactcagcaattatgaaaagtgaattggaatatggtaattgcaacaccaagtgtcaaactccaatgggggcgataaactctagtatgccattccacaacatacaccctctcaccatcggggaatgccccaaatatgtaaagtcaaacagattagtccttgcaacagggctcagaaatagccctcaaagagaaagcagaagaaaaaagagaggactatttggagctatagcaggttttatagagggaggatggcagggaatggtagatggttggtatgggtaccaccatagcaatgagcaggggagtgggtacgctgcagacaaagaatccactcaaaaggcagtagatggagtcaccaataaggtcaactcaattattgacaaaatgaacactcagtttgaggctgttggaagggaatttaataacttagagaggagaatagagaatttaaacaagaagatggaagacgggtttctggatgtttggacttataatgctgaacttctggttctcatggaaaatgagagaactttagactttcatgattcaaatgttaagaacctctacgacaaggtccggctacagcttagggataatgcaaaggagctgggtaacggttgtttcgagttctatcacaaatgtgataatggatgtatggaaagtataagaaacggaacgtacaactatccgcagtattcagaagaagcaagattaaaaagagaggaaataaatggggtaaaattggaatcaataggaacttaccaaatattgtcaatttattcaacagtggcaagttccctagcactggcaatcatgatggctggtctatctttatggatgtgctccaatggatcgttacagtgcagaatttgcatttga

>H5N1_A/chicken/Bali/UT2091/2005

cttgttaaaagtgatcagatttgcattggttaccatgcaaacaattcaacagagcaggttgacacaataatggaaaagaacgttactgttacacatgcccaagacatactggaaaaaacacacaacgggaagctctgcgatctagatggagtgaagcctctaattttaagagattgtagtgtagctggatggctcctcgggaacccaatgtgtgacgaattcatcaatgtaccggaatggtcttacatagtggagaaggccaatccagccaatgacctctgttacccagggagtttcaacgactatgaagaactgaaacacctattgagcagaataaaccattttgagaaaattcagatcatccccaaaagttcttggtccgaccatgaagcctcatcaggagtgagctcagcatgtccatacctgggaagtccctccttttttagaaatgtggtatggattatcaaaaagaacagtacatacccaacaataaagaaaagctacaataataccaaccaagaagatcttttggtactgtgggggattcaccatcctaatgatgcggcagagcagacaaggctatatcaaaacccaaccacctatatttccgttgggacatcaacactaaaccagagatcggtaccaaaaatagctactagatccaaagtaaacgggcaaagtggaaggatggagttcttctggacaattttaaaaccgaatgatgcaatcaacttcgagagtaatggaaatttcattgctccagaatatgcatacaaaattgtcaagaaaggggactcagcaattatgaagagtgaattggaatatggtaattgcaacaccaagtgtcaaactccaatgggggcgataaactctagtatgccattccacaacatacaccctctcaccatcggggaatgccccaaatatgtgaaatcaaacagattagtccttgcaacagggctcagaaatagccctcaaagagagagaagaagaaaaaagagaggactatttggagctatagcaggttttatagagggaggatggcagggaatggtagatggttggtatgggtaccaccatagcaatgagcaggggagtgggtacgctgcagacaaagaatccactcaaaaggcaatagatggagtcaccaataaggtcaactcaatcattgacaaaatgaacactcagtttgaggccgttggaagggaatttaataacttagaaaggagaatagagaatttaaacaagaagatggaagacggatttctagatgtctggacttataatgccgaacttctggttctcatggaaaatgagagaactctagactttcatgactcaaatgttaagaacctctacgacaaggtccgactacagcttagggataatgcaaaggagctgggtaacggttgtttcgagttctatcacaaatgtgataatgaatgtatggaaagtataagaaacggaacgtataactacccgcagtattcagaagaagcaagattaaaaagagaggaaataagtggagtaaaattggaatcaataggaacttaccaaatactgtcaatttattcaacagtggcgagttccctagcactggcaatcatgatggctggtctatctttatggatgtgctccaatggatcgttacaatgcagaatttgcatttga

>H5N1_A/chicken/Bali/UT2092/2005

cttgttaaaagtgatcagatttgcattggttaccatgcaaacaattcaacagagcaggttgacacaataatggaaaagaacgttactgttacacatgcccaagacatactggaaaaaacacacaacgggaagctctgcgatctagatggagtgaagcctctaattttaagagattgtagtgtagctggatggctcctcgggaacccaatgtgtgacgaattcatcaatgtaccggaatggtcttacatagtggagaaggccaatccagccaatgacctctgttacccagggagtttcaacgactatgaagaactgaaacacctattgagcagaataaaccattttgagaaaattcagatcatccccaaaagttcttggtccgaccatgaagcctcatcaggagtgagctcagcatgtccatacctgggaagtccctccttttttagaaatgtggtatggattatcaaaaagaacagtacatacccaacaataaagaaaagctacaataataccaaccaagaagatcttttggtactgtgggggattcaccatcctaatgatgcggcagagcagacaaggctatatcaaaacccaaccacctatatttccgttgggacatcaacactaaaccagagatcggtaccaaaaatagctactagatccaaagtaaacgggcaaagtggaaggatggagttcttctggacaattttaaaaccgaatgatgcaatcaacttcgagagtaatggaaatttcattgctccagaatatgcatacaaaattgtcaagaaaggggactcagcaattatgaagagtgaattggaatatggtaattgcaacaccaagtgtcaaactccaatgggggcgataaactctagtatgccattccacaacatacaccctctcaccatcggggaatgccccaaatatgtgaaatcaaacagattagtccttgcaacagggctcagaaatagccctcaaagagagagaagaagaaaaaagagaggactatttggagctatagcaggttttatagagggaggatggcagggaatggtagatggttggtatgggtatcaccatagcaatgagcaggggagtgggtacgctgcagacaaagaatccactcaaaaggcaatagatggagtcaccaataaggtcaactcaatcattgacaaaatgaacactcagtttgaggccgttggaagggaatttaataacttagaaaggagaatagagaatttaaacaagaagatggaagacggatttctagatgtctggacttataatgccgaacttctggttctcatggaaaatgagagaactctagactttcatgactcaaatgttaagaacctctacgacaaggtccgactacagcttagggataatgcaaaggagctgggtaacggttgtttcgagttctatcacaaatgtgataatgaatgtatggaaagtataagaaacggaacgtataactacccgcagtattcagaagaagcaagattaaaaagagaggaaataagtggagtaaaattggaatcaataggaacttaccaaatactgtcaatttattcaacagtggcgagttccctagcactggcaatcatgatggctggtctatctttatggatgtgctccaatggatcgttacaatgcagaatttgcatttga

>H5N1_A/chicken/Bangladesh/15078/2012

cttgttaaaagcgatcatatttgcattggttatcatgcaaataactcgacaaagcaggttgacacaataatggaaaagaacgttactgttacacatgcccaagacatactggaaaagacacacaacgggaagctctgtgatctaaatggagtgaaacctctgattttaaaagattgtagtgtagcaggatggctcctcggaaacccaatgtgtgacgaattcatcaatgtaccagaatggtcttacatagtagagaaggccaatccagtcaatggcctctgttacccagggagtttcaacgattatgaagaattgaagcacctattgagcaggataaaccattttgagaaaatacggatcatccccaaagattcttggccagatcatgaagcctcattgggggtgagtgcagcatgtccataccagggaaattcctccttcttcagaaatgtggtatggcttatcaaaaaggacaatgcatacccaacaataaagaaaagctacaataataccaatcaagaagatctcttggtactgtgggggattcaccatcctaatgatgaagcagagcagacaaggctctatcaaaacccaaccacctatatttccattgggacatcaacactaaacctgagattggtaccaaaaatagccactagatccaaaataaacgggcaaagtggcaggatagatttcttttggacaattttaaaaccgaatgatgcaatccacttcgagagtaacggaaatttcattgctccagagtatgcatacaaaattgtcaagaaaggagactcaacaattatgaaaagtgaagtggaatatggtaactgcaacaccaggtgtcagactcctataggggcgataaactctagtatgccattccacaacatacatcccctcaccatcggagaatgtcccaaatatgtgaaatcaaacaaattagtccttgcgactgggctcagaaatagtcctcaaagagagagaagaagaaaaGGGagaggattgtttggagctatagcagggtttatagagggaggatggcagggaatggtagatggttggtatgggtaccaccacagcaacgagcaggggagtgggtacgctgcagacaaagaatctactcaaaaggcaatagacggagtcaccaataaggtcaactcgatcattgacaaaatgaacactcagtttgaggccgtaggaagggagtttaataacttagagagaagaatagagaatttgaacaagaagatggaagacggattcttagatgtctggacttataatgctgaacttctggttctcatggaaaatgagagaactctagacttccatgactcaaatgtcaggaacctttacgacaaggtcagactgcagcttaaggacaatgcaaaagagatgggtaacggttgtttcgagttctatcacaaatgtgataatgaatgtatggaaagtgtaagaaacggaacgtatgactacccgcagtactcagaagaagcaagattaaaaagagaagaaataagtggagtaaagttggaatcaataggaatttaccaaatattgtcaatttattcaacagtggcgagttccctagtactggcaatcatgatggctggtctatctttatggatgtgttccaacgggtcgttacaatgcagaatctgcatttga

>H5N1_A/chicken/Bangladesh/15083/2012

cttgttaaaagcgatcatatttgcattggttatcatgcaaataactcgacagagcaggttgacacaataatggaaaagaacgttactgttacacatgcccaagacatactggaaaagacacacaacgggaagctctgcgatctaaatggggtgaagcctctgattttaaaagattgtagtgtagcaggatggctactcggaaacccattgtgtgacgaattcatcaatgtaccagaatggtcttacatagtagagaaggccaatccagccaatggcctctgttacccagggaatttcaacgattatgaagaattgaaacacctattgagtaggataaaccattttgagaaaatacagatcatccccaaagattcttggtcagatcatgaagcctcattgggggtgagcgcagcatgttcataccagggaaattcctccttcttcagaaatgtggtatggcttatcaaaaaggacaatgcatacccaacaataaagaaagactacaataataccaaccgagaagatctcttggtactttgggggatccaccatcctaatgatgaggcagagcagacgaggctctatcaaaacccaaccacctatatttccattgggacatcaacactaaaccagagattggtaccaaaaatagccactagatccaaaataaacgggcaaagtggcaggatagatttcttctggacaattttaaaaccgaatgatgcaatccacttcgagagtaatggaaatttcattgctccagaatatgcatacaaaattgtcaagaaaggagactccacaattatgagaagtgaagtggaatatggtaactgcaacaccaggtgtcagactccgataggggcgataaactctagtatgccattccataacatacaccccctcaccatcggagaatgtcccaaatatgtgaaatcaaacaaattagtccttgcgactgggctcagaaatagtcctcaaagagagagaagaagaaaaGGGagaggactgtttggagctatagcaggttttatagagggaggatggcagggaatggtagatggttggtatgggtaccatcacagcaatgagcaggggagtgggtacgctgcagacaaagaatctactcaaaaggcaatagacggagtcaccaataaggtcaactcgatcattgacaaaatgaacactcagtttgaggccgtaggaagggaatttaataacttagagaggagaatagagaatttaaacaagaagatggaagacggattcctagacgtctggacttataatgctgaacttctggttctcatggaaaatgagagaactctagacttccatgactcaaatgtcaagaacctttacgataaggtcagactacagcttaaggataatgcaaaagagctgggtaacggttgtttcgagttctatcacaaatgtaataatgaatgtatggaaagtgtaagaaacggaacgtatgactacccgcagtattcagaagaagcaagattaaaaagagaggaaataagtggagtaaaattggaatcaataggagtctaccaaatactgtcaatttattcaacagtggcgagttccctagtgctggcaatcatgatggctggtctatctttatggatgtgttccaacgggtcgttacagtgcagaatttgcatttga

>H5N1_A/chicken/Bangladesh/18061/2012

cttgttgaaagcgatcatatttgcattggttatcatgcaaataactcgacaaaccaggttgatacaataatggaaaagaacgttactgttacacatgcccaagacatactggaaaagacacacaacgggaagctctgcgatctaaatggagtgaaacctctgattttaaaagattgtagtgtagcaggatggctcctcggaaacccaatgtgtgacgaattcatcaatgtgccagaatggtcttacatagtagagaaggccaatccagccaatggcctctgttacccagggactttcaacgattatgaggaattgaaacacctattgagcaggataaaccattttgagaaaatacggatcatccccaaagattcttggtcagatcatgaagcctcattgggggtgagtgcagcatgtccataccagggaaattcctccttcttcaggaatgtggtatggcttatcaaaaaggacaatgcatacccaacaataaagaaaagctacaataataccaaccaagaagatctcttggtactgtgggggattcaccatcctaatgatgaagcagagcagacagggctctatcaaaacccaaccacctatatttccattgggacatcaacactaaaccagagattggtaccaaaaatagctactagatccaaaataaacgggcaaagtggcaggatagatttcttctggacaatattaaaaccgaatgatgcaatccacttcgagagtaacggaaatttcattgctccagagtatgcatacaaaattgtcaagaaaggagactcaacaattatgaaaagtgaagtggaatatggtaactgcaacaccaggtgtcagactcctataggggcgataaactctagtatgccattccacaacatacatcccctcaccatcggagaatgtcccaaatatgtgaaatcaaacaaattagtccttgcgactgggctcagaaatagtcctcaaagagagagaagaaggaaaGGGagagggttgtttggagctatagcgggatttatagagggaggatggcagggaatggtagatggctggtatgggtaccaccacagcaatgagcaggggagtgggtacgctgcagacaaagaatctactcaaaaggcaatagacggagtcaccaataaggtcaactcgatcatcgacaaaatgaacactcagtttgaagccgtaggaagggagtttaataacttagagaggagaatagagaatttgaacaagaagatggaagacggattcctagatgtctggacttataatgctgaacttctggttctcatggaaaatgagagaactctagacttccatgactcaaatgtcaggaacctttacgacaaggtcagactacagcttaaggacaatgcaaaagagctgggtaacggctgtttcgagttctatcacaaatgtaataatgaatgtatggaaagtgtaagaaacggaacgtatgactacccgcattattcagaagaagcaagattaaaaagagaagaaataaatggggtaaagttggaatcaataggaatttaccaaatattgtcaatttattcaacagcggcgagttccctagtactggcaatcatgatggctggtctatctttatggatgtgttccaacgggtcgttacagtgcagaatttgcatttga

>H5N1_A/chicken/Bangladesh/19D1833/2022

cttgttaaaagcgatcatatctgcattggttatcatgcaaataactcgacaaagcaggttgacacaataatggaaaagaacgttactgttacacatgcccaagacatactggaaaaaacacacaatgggaagctctgcgatctaaatggagtgaaacctctgattttaaaggattgtagtgtagcaggatggctcctcggaaacccaatgtgtgacgaattcatcaatgtaccagaatggtcttacatagtagagaaggccaatccagccaatggcctctgttacccagggagtttcaacgattatgaagagttgaaacacctattgagcaggataaaccattttgagaaaatacggatcatccccaaagattcttggtcaaatcatgaagcctcattgggggtgagtgcagcatgtccatatcagggaaattcctccttctttagaaatgtggtatggcttatcaaaaaggacaatgcatacccaacaataaagaaaagctacaataataccaaccaagaagatctcttaatactgtgggggatccatcaccctaatgatgaagcagagcagacaaggctctatcaaaacccaaccacctacatttccattggaacatcaacactaaaccaaagattggtaccaaaaatagccactagatccaaaataaacgggcaaagtggcaggatagatttcttctggacgattctaaaaccgaatgatgcaatccacttcgaaagtaacggaaacttcatagctccagaatatgcatacaaaattgtcaaaaaaggagactcaacaattatgaaaagtgaagtggaatatggaaattgcaataccaggtgccagactcctataggggcgataaactctagtatgccattccataacatacatcccctcaccattggagaatgtcccaaatatgtgaaatcaaacaaattggtccttgcgactgggctcagaaatagtcctcaaaaagaaaggagaagaaaaGGGagaggattgtttggagctatagcagggtttatagaaggaggatggcaggggatggtagatggctggtatgggtaccaccacagcaatragcaggggagtgggtacgctgcagacaaagaatctacccaaaaggcaatagacggggtcaccaataaggtcaactcgatcattgacaaaatgaacacccagtttgaggccgtgggaagggagttcaatagcttagagaggagaatagaaaatttgaacaagaagatggaagacggatttctagatgtctggacttacaatgcagaactcctggttctcatggaaaatgagagaactctagacttccatgactcaaatgtcaggaatctttacgacaaagtcagactacagcttaaggacaatgcaaaagagctgggtaatggttgtttcgagttctatcacaaatgtgataatgaatgtatggaaagtgtaagaaacggaacgtatgattacccacagtattcagaagaagcaagattaaaaagagaagaaataagtggagtaaagttggaatcaataggaatttaccaaatattgtcaatttattcaacagtggcaagttccctagtactggcaatcatgatggctggtctatctttatggatgtgttccaacgggtcgttgcagtgcagaatttgcatttaa

>H5N1_A/chicken/Bangladesh/19D1836/2022

cttgttaaaagcgatcatatctgcattggttatcatgcaaataactcgacaaagcaggttgacacaataatggaaaagaacgttactgttacacatgcccaagacatactggaaaaaacacacaatgggaagctctgcgatctaaatggagtgaaacctctgattttaaaggattgtagtgtagcaggatggctcctcggaaacccaatgtgtgacgaattcatcaatgtaccagaatggtcttacatagtagagaaggccaatccagccaatggcctctgttacccagggagtttcaacgattatgaagagttgaaacacctattgagcaggataaaccattttgagaaaatacggatcatccccaaagattcttggtcaaatcatgaagcctcattgggggtgagtgcagcatgtccatatcagggaaattcctccttctttagaaatgtggtatggcttatcaaaaaggacaatgcatacccaacaataaagaaaagctacaataataccaaccaagaagatctcttaatactgtgggggatccatcaccctaatgatgaagcagagcagacaaggctctatcaaaacccaaccacctacatttccattggaacatcaacactaaaccaaagattggtaccaaaaatagccactagatccaaaataaacgggcaaagtggcaggatagatttcttctggacgattctaaaaccgaatgatgcaatccacttcgaaagtaacggaaacttcatagctccagaatatgcatacaaaattgtcaaaaaaggagactcaacaattatgaaaagtgaagtggaatatggaaattgcaataccaggtgccagactcctataggggcgataaactctagtatgccattccataacatacatcccctcaccattggagaatgtcccaaatatgtgaaatcaaacaaattggtccttgcgactgggctcagaaatagtcctcaaaaagaaaggagaagaaaaGGGagaggattgtttggagctatagcagggtttatagaaggaggatggcaggggatggtagatggctggtatgggtaccaccacagcaatragcaggggagtgggtacgctgcagacaaagaatctacccaaaaggcaatagacggggtcaccaataaggtcaactcgatcattgacaaaatgaacacccagtttgaggccgtgggaagggagttcaatagcttagagaggagaatagaaaatttgaacaagaagatggaagacggatttctagatgtctggacttacaatgcagaactcctggttctcatggaaaatgagagaactctagacttccatgactcaaatgtcaggaatctttacgacaaagtcagactacagcttaaggacaatgcaaaagagctgggtaatggttgtttcgagttctatcacaaatgtgataatgaatgtatggaaagtgtaagaaacggaacgtatgattacccacagtattcagaagaagcaagattaaaaagagaagaaataagtggagtaaagttggaatcaataggaatttaccaaatattgtcaatttattcaacagtggcaagttccctagtactggcaatcatgatggctggtctatctttatggatgtgttccaacgggtcgttgcagtgcagaatttgcatttaa

>H5N1_A/chicken/Bangladesh/22478/2014

cttgttaaaagcgatcatatttgcattggttatcatgcaaataactcgacaaagcaggttgacacaataatggaaaagaacgttactgttacacatgcccaagacatactggaaaagacacacaatgggaagctctgcgatctaaatggagtgaaacctctgattttaaaagattgtagtgtagcaggatggctcctcggaaacccaatgtgtgacgaattcatcaatgtgccagaatggtcttacatagtagagaaggccaatccagccaatggcctctgttacccagggaatctcaacgattatgaagaattgaaacacctattgagcaggataaaccattttgagaaaatacggatcatccccaaagatttttggtcagatcatgaagcctcattgggggtgagtgcagcatgtccataccagggaaattcctccttcttcagaaatgtggtgtggcttatcaaaaaggacaatacatacccaacaataaagaaaagctacaataataccaaccaagaagatctcttgatactgtgggggattcaccaccctaatgatgaagcagagcagacaaggctctatcaaaacccgaccacctatatttccattgggacatcaacactaaaccagagattggtaccaaaaatagccactagatccaaaataaacgggcaaagtggcaggatagatttcttctggacaattttaaaaccgaaagatgcaatccacttcgagagtaacggaaatttcattgctccagagtatgcatacaaaattgtcaagaaaggagactcaacaattatgagaagtgaagtggaatatggtaattgcaacaccaggtgtcagactcctataggggcgataaactctagtatgccattccacaacatacatcccctcaccatcggagaatgtcccaaatatgtgaaatcaaacaaattagtccttgcgactgggctcagaaataatcctcaaagagagagaagaagaaaaGGGagaggattgtttggagctatagcagggtttatagagggaggatggcaggggatggtagatggttggtatgggtaccaccacagcaacgagcaggggagtgggtacgctgcagacaaagaatctactcaaaaggcaatagacggagtcaccaataaggtcaactcgatcattgataaaatgaacactcagtttgaggccgtaggaagggagtttaataacttagagaggagaatagagaatttgaacaagaagatggaagacggattcctagatgtctggacttataatgcagaacttctggttctcatggaaaatgagagaactctagacttccatgactcaaatgtcaggaatctttacgacaaggtcagactacagcttaaggacaatgcgaaagagctgggtaatggttgtttcgagttctatcacaaatgtaataatgaatgtatggaaagtgtaagaaacggaacgtatgactacccgcagtattcagaagaagcaagattaaaaagagaagaaataagtggagtaaagttggaatcaataggaatttaccaaatattgtcaatttattcaacagcggcaagttccctagtactggcaatcatgatggctggtctatctttatggatgtgttccaacgggtcgttacagtgcagaatttgcatttga

>H5N1_A/chicken/Bangladesh/22482/2014

cttgttaaaagcgatcatatttgcattggttatcatgcaaataactcgacaaagcaggttgacacaataatggaaaagaacgttactgttacacatgcccaagacatactggaaaagacacacaatgggaagctctgcgatctaaatggagtgaaacctctgattttaaaagattgtagtgtagcaggatggctcctcggaaacccaatgtgtgacgaattcatcaatgtgccagaatggtcttacatagtagagaaggccaatccagccaatggcctctgttacccagggaatctcaacgattatgaagaattgaaacacctattgagcaggataaaccattttgagaaaatacggatcatccccaaagatttttggtcagatcatgaagcctcattgggggtgagtgcagcatgtccataccagggaaattcctccttcttcagaaatgtggtgtggcttatcaaaaaggacaatacatacccaacaataaagaaaagctacaataataccaaccaagaagatctcttgatactgtgggggattcaccaccctaatgatgaagcagagcagacaaggctctatcaaaacccgaccacctatatttccattgggacatcaacactaaaccagagattggtaccaaaaatagccactagatccaaaataaacgggcaaagtggcaggatagatttcttctggacaattttaaaaccgaaagatgcaatccacttcgagagtaacggaaatttcattgctccagagtatgcatacaaaattgtcaagaaaggagactcaacaattatgagaagtgaagtggaatatggtaattgcaacaccaggtgtcagactcctataggggcgataaactctagtatgccattccacaacatacatcccctcaccatcggagaatgtcccaaatatgtgaaatcaaacaaattagtccttgcgactgggctcagaaataatcctcaaagagagagaagaagaaaaGGGagaggattgtttggagctatagcagggtttatagagggaggatggcaggggatggtagatggttggtatgggtaccaccacagcaacgagcaggggagtgggtacgctgcagacaaagaatctactcaaaaggcaatagacggagtcaccaataaggtcaactcgatcattgataaaatgaacactcagtttgaggccgtaggaagggagtttaataacttagagaggagaatagagaatttgaacaagaagatggaagacggattcctagatgtctggacttataatgcagaacttytggttctcatggaaaatgagagaactctagacttccatgactcaaatgtcaggaatctttacgacaaggtcagactacagcttaaggacaatgcgaaagagctgggtaatggttgtttcgagttctatcacaaatgtaataatgaatgtatggaaagtgtaagaaacggaacgtatgactacccgcagtattcagaagaagcaagattaaaaagagaagaaataagtggagtaaagttggaatcaataggaatttaccaaatattgtcaatttattcaacagcggcaagttccctagtactggcaatcatgatggctggtctatctttatggatgtgttccaacgggtcgttacagtgcagaatttgcatttga

>H5N1_A/chicken/Bangladesh/23974/2014

cttgytaaaagcgatcatatctgcattggttatcatgcaaataactctacaaagcaggttgacacaataatggaaaagaacgttactgttacacaggcccaagacatactggaaaagacacacaatggraagctctgcgatctaaatggagtgaaacctctgattttaaaagattgtagtgtagcaggatggctcctcggaaacccaatgtgtgacgaattcaycaatgtgccagaatggtcttacatagtagagaaggccaatccagtcaatggcctctgttacccaggwaatttcaacgattatgaagaattgaaacatctattgagcaggataaaccattttgagaaaatacggattatccccaaagattcttggtcagatcatgaagcctcactgggggtgagtgcagcatgtccataccagggaaattcctccttcttcagaaatgtggtgtggcttatcaaaaaggacaatacataccctacaataaagaaaagctacaataataccaaccaagaagatctcttgatactgtgggggattcaccaccctaatgatgaagcagagcagacaaggctctatcaaaacccaaccacctacatttccattgggacatcaacactaaacctgagattggtgccaaaaatagccactagatccaaaataaacgggcaaagtggcaggatagatttcttctggacaattttaaaaccgaatgatgcaatccacttcgagagtaacggaaatttcattgctccagagtatgcatacaagattgtcaagaaaggagactcaacaattatgaaaagtgaagtggaatatggcaattgcaacaccaggtgtcagactcctataggggcgataaactctagtatgccattccacaacatacaccccctcaccatcggagaatgtcccaaatatgtgaaatcaaacaaattagtccttgcgactgggctcagaaatagtcctcaaagagagagaagaagaaaaGGGagaggattgtttggggctatagcagggtttatagagggaggatggcaggggatggtagatggttggtatgggtaccaccacagcaacgagcaggggagtgggtacgctgcagacaaagaatctactcaaaaggcaatagacggagtcaccaataaggtcaactcgattattgacaagatgaacacccagtttgaggcagtaggaagggagtttaacaacttagagaggagaatagagaatttgaacaagaagatggaagacggattcctagatgtctggacttataatgcagaacttctggttctcatggaaaatgagagaactctagacttccatgactcaaatgtcaggaatctttacgacaaggtcagactacaacttaaggacaatgcaaaagagctgggtaacggttgtttcgagttctatcacaaatgtgataatgaatgtatggaaagtgtaaaaaacggaacttatgactacccacagtattcaaaagaagcaagattaaaaagagaagaaataagtggagtaaagttggaatcaataggaatttaccaaatattgtcaatttattcaacagtggcaagttccctagtactggcaatcatgatggctggtctatcgttatggatgtgttccaacggatcgttacagtgcagaatttgcatttga

>H5N1_A/chicken/Bangladesh/30065/2016

cttgttaaaagcgatcatatctgcattggttatcatgcaaataactcgacaaaccaggttgacacaataatggaaaagaacgttactgttacacatgcccaagacatactggaaaagacacacaatgggaagctctgcgatctaaatggagtgaaacctctgattttaaaagattgtagtgtagcaggatggctcctcggaaacccaatgtgtgacgaattcatcaatgtgccagaatggtcttacatagtagagaaggccaatccagccaatggcctctgttacccagggaatttcaacgattatgaagaattgaaacacctattgagcaggataaaccattttgagaaaatacggatcatccccaaagattcttggtcagatcatgaagcctcactgggggtgagtgcagcatgtccatatcagggaaattcctccttcttcagaaacgtggtatggcttatcaaaaaggacaatacatacccaacaataaagaaaagctacaataataccaaccaagaagatctcttgatactgtgggggattcatcaccctaatgatgaagcagagcagacaaggctctatcaaaacccaaccacctacatttccattggaacatcaacactaaaccaaagattggtaccaaaaatagccactagatccaaaataaacggacaaagtggcaggatagatttcttctggacaattctaaaaccgaatgatgcaatccacttcgagagtaacggaaacttcattgctccagagtatgcatacaaaattgtcaaaaagggagactcaacaattatgaaaagtgaagtggaatatggaaattgcaataccaggtgccagactcctataggggcgataaactctagtatgccattccacaacatacatcccctcaccatcggagaatgccccaaatatgtgaaatcaaacaaattagtccttgcgactgggctcagaaatagtcctcaaagagagagaagaagaaaaGGGagaggattgtttggagctatagcagggtttatagagggaggatggcagggaatggtagatggttggtatgggtaccaccacagcaatgagcaggggagtgggtacgctgcagacaaagaatctacccaaaaggcaatagacggggtcaccaataaggtcaactcgatcattgacaaaatgaacacccagtttgaggccgtaggaagggagtttaatagcttagagaggagaatagaaaatttgaacaagaagatggaagacggatttctagatgtctggacttacaatgcagaacttctggttctcatggaaaatgagagaactctagacttccatgactcaaatgtcaggaatctttacgacaaggtcagactacagcttaaggacaatgcaaaagagctgggtaatggttgtttcgagttctatcacaaatgtgataatgaatgtatggaaagtgtaagaaacggaacgtatgattacccacagtattcagaagaatcaagattaaaaagagaagaaataagtggagtaaagttggaatcaataggaatttaccaaatattgtcaatttattcaacagtggcaagttccctagtactggcaatcatgatggctggtctatctttatggatgtgttccaacgggtcgttacagtgcagaatttgcatttga

>H5N1_A/chicken/Bangladesh/32747/2017

cttgttaaaagcgatcatatctgcattggttatcatgcaaataactcgacaaagcaggttgacacaataatggaaaagaacgttactgttacacatgcccaagacatactggaaaagacacacaatgggaagctctgcgatctaaatggagtgaaacctctgattttaaaggattgtagtgtagcaggatggctcctcggaaacccaatgtgtgacgaattcatcaatgtgccagaatggtcttacatagtagagaaggccaatccagccaatggcctctgttacccagggactttcaacgattatgaagaattgaaacacctattgagcaggataaaccattttgagaaaatacgggtcatccccagagattcttggtcagatcatgaagcctcactgggggtgagtgcagcctgcccataccagggaaattcctccttcttcagaaacgtggtatggcttatcaaaaaggacaatacatacccaacaataaagaaaagctacaataataccaaccaagaagatctcttgatactgtgggggattcatcaccctaatgatgaagcagagcagacaaggctctatcaaaacccgaccacctacatttccattggaacatcaacactaaaccaaagattggtaccaaaaatagccactagatccaaaataaacgggcaaagtggcaggatagatttcttctggacaattctaaaaccgaatgatgcaatccacttcgagagtaacggaaacttcattgctccagagtatgcatacaaaattatcaaaaagggagactcaacaattatgaaaagtgaagtggaatatggaaattgcaataccaggtgccagacccctataggggcgataaactctagtatgccattccacaacatacatcccctcaccatcggagaatgtcccaaatatgtgaaatcaaacaaattagtccttgcgactgggctcagaaatagccctcaaagagaaagaagaagaaaaGGGagaggattgtttggagctatagcaggatttatagagggaggatggcaggggatggtagatggttggtatgggtaccaccacagcaatgagcaggggagtgggtacgctgcagacaaagaatctacccaaaaggcaatagacggggtcactaataaggtcaactcgatcattgacaaaatgaacacccaatttgaggccgtaggaagggagtttaataacttagagaggagaatagaaaatttgaacaagaagatggaagacggatttctagatgtctggacttacaatgcagaacttctggttctcatggaaaatgagagaactctagacttccatgactcaaatgtcaggaatctttacgacaaggtcagactacagcttaaggacaatgcaaaagagctgggtaatggttgtttcgagttctatcacaaatgtgataatgaatgtatggaaagtgtaagaaacggaacgtatgattacccacagtattcagaagaatcaagattaaaaagagaagaaataagtggggtaaagttggaatcaataggaatttaccaaatattgtcaatttattcaacagtggcaagttccctagtactggcaatcatgatggctggtctatctttatggatgtgttccaacgggtcgttacagtgcagaatttgcatttga

>H5N1_A/chicken/Bangladesh/33167/2017

cttgttaaaagcgatcatatctgcattggttatcatgcaaataactcgacaaagcaggttgacacaataatggaaaagaacgttactgttacacatgcccaagacatactggaaaagacacacaatgggaagctctgcgatctgaatggagtgaaacctctgattttaaaagattgtagtgtagcaggatggctcctcgggaacccaatgtgtgacgaattcatcaatgtgccagaatggtcttacatagtagagaaggccaatccagccaatggcctctgttacccagggaatttcaacgattatgaagaattgaaacacctattgagcaggataaaccattttgagaaaatacgtatcatccccagagattcttggtcagatcatgaagcctcacttggggtgagcgcagcatgtccataccagggaaattcctccttcttcagaaatgtggtatggcttatcaaaaaggacaatacatacccaacaataaagaaaagttacaacaataccaaccaagaagatctcttgatactgtgggggattcatcaccctaatgatgaagcagagcagacaaggctctatcaaaacccaaccacctacatttccattggaacatcaacactaaaccaaagattggtaccaaaaatagccactagatccaaaataaacgggcaaagtggcagaatagatttcttctggacaattctaaaaccgaatgatgcaatccacttcgagagtaacggaaacttcattgctccagagtatgcatacaaaattgttaaaaagggagactcaacaattatgaaaagtgaagtggaatatggaaattgcaataccaggtgccagactcctataggggcgataaactctagtatgccattccacaacatacatcccctcaccattggagaatgtcccaaatatgtgaaatcaaacaaattagtccttgcgactgggctcagaaatagtcctcaaagagagagaagaagaaaaGGGagaggattgtttggagctatagcagggtttatagagggaggatggcaggggatggtagatggttggtatgggtaccaccacagcaatgagcaggggagtgggtacgctgcagacaaagaatctacccaaaaggcaatagacggggtcaccaataaggttaactcgatcattgacaaaatgaacacccagtttgaggccgtaggaagggagtttaatagcttagagaggagaatagaaaatttgaacaagaagatggaagacggatttctagatgtctggacttacaatgcagaacttctggttctcatggaaaatgagagaactctagacttccatgactcaaatgtcaggaatctttacgacaaggtcagactacagcttaaggacaatgcaaaagagctgggtaatggttgtttcgagttctatcacaaatgtgataatgaatgtatggaaagtgtaagaaacggaacgtatgattacccacagtattcagaagaatcaagattaaaaagagaagaaataagtggagtaaagttggagtcaataggaatttaccaaatattgtcaatttattcaacagtggcaagttccctagtactggcaatcatgatggctggtctatctttatggatgtgttccaacgggtcgttacagtgcagaatttgcatttga

>H5N1_A/chicken/Bangladesh/33395/2017

cttgttaaaagcgatcatatctgcattggttaccatgcaaataactcgacaaagcaggttgacacaataatggaaaagaacgttactgttacacatgcccaagacatactggaaaagacacacaatgggaagctctgcgatctaaatggagtgaaacctctgattttaaaggattgtagtgtagcaggatggctcctcggaaacccaatgtgtgacgaattcatcaatgtgccagaatggtcttacatagtagagaaggccaatccagccaatggcctctgttacccagggaatttcaacgattatgaagaattgaaacacctattgagcaggataaaccattttgagaaaatacggatcatccccaaagattcttggtcagatcatgaagcctcactgggggtgagtgcagcatgtccataccagggaaatccctccttcttcagaaatgtggtatggcttatcaaaaaggacaatacatacccaacaataaagaaaagctacaataataccaaccaagaagatctcttgatactgtgggggatccatcaccccaatgatgaagcagagcagacaaggctctatcaaaacccaatcacctacatttccattggaacatcaacactaaaccaaagattggtaccaaaaatagccactagatccaaaataaacgggcaaagtggcaggatagatttcttctggacaattctaaaaccaaatgatgcaatccacttcgagagcaacggaaacttcattgctccagagtatgcatacaaaattgtcaaaaagggggactcaacaattatgagaagtgaagtggaatatggaaattgcaacaccaggtgccagactcctataggggcgataaactctagtatgccattccataacatacatcccctcaccatcggagaatgtcccaaatatgtgaaatcaaacaaattagtccttgcgactgggctcagaaatagtcctcaaagagagagaagaagaaaaGGGagaggattgtttggagctatagcagggtttatagagggaggatggcaggggatggtagatggttggtatgggtaccaccacaacaatgagcaggggagtgggtacgctgcagacaaagaatctacccaaaaggcaatagacggggtcaccaataaggtcaactcgatcattgacaaaatgaacatccagtttgaggccgtaggaagggagttcaatagcttagagaggagaatagaaaatttgaacaagaagatggaagacggatttctagatgtctggacttacaatgcagaacttctggttctcatggaaaatgagaggactctagacttccatgactcaaatgtcaggaatctttacgacaaggtcagactacagcttaaggacaatgcaaaggagctgggtaatggttgttttgagttctatcacaaatgtgataatgaatgtatggaaagtgtaagaaacggaacgtatgattacccaaagtattcagaagaatcaagattaaaaagagaagaaataagtggagtaaagttggaatcaataggaatttaccaaatattgtcaatttattcaacagtggcaagttctctagtactggcaatcatgatggctggtctatctctatggatgtgttccaacgggtcgttacagtgcagaatttgcatttga

>H5N1_A/chicken/Bangladesh/34265/2017

cttgtcaaaagcgatcatatctgcattggttatcatgcaaataactcgacgaagcaggttgacacaataatggaaaagaacgttactgttacacatgcccaagacatactggaaaagacacataatgggaagctctgcgatctaaatggagtgaaacctctgattttaaaagattgtagtgtagcaggatggctcctcggaaacccaatgtgtgacgaattcatcaatgtgccagaatggtcttacatagtagagaaggccaatccagccaatggcctctgttacccagggaatttcaacgattatgaagaattgaaacacctattgagcaggataaaccattttgagaaaatacggatcatccccaaagattcttggtcagatcatgaagcctcactgggggtgagtgcagcatgtccataccagggaaattcctccttcttcagaaatgtgatatggcttatcaaaaagaacaatgcatacccaacaataaagagaagctacaataataccaaccaagaagatctcttgatactgtgggggattcatcaccctaatgatgaagcagagcagacaaggctctatcaaaacccaaccacctacatttccattggaacatcaacactaaaccaaagattggtaccaaaaatagccactagatccaaaataaacgggcaaagtggcaggatagatttcttctggacaattctaaaaccgaatgatgcaatccacttcgagagtaacggaaacttcattgctccagaatatgcatacaaaattgtcaaaaaaggagactcaacaattatgaaaagtgaagtgaaatatggaaattgcaataccaggtgccagactcctataggggcgataaactctagtatgccattccataacatacatcccctcaccattggagaatgtcccaaatatgtgaaatcaaacaaattagtccttgcgactgggctcagaaatagtcctcaaaaagaaagaagaagaagaGGGagaggattgtttggagctatagcagggtttatagaaggaggatggcaggggatggtagatggttggtatgggtaccaccacagcaatgagcaggggagtgggtacgctgcagacaaagaatctacccaaaaggcaatagacggggtcaccaataaggtcaactcgatcattgacaaaatgaacacccagtttgaagccgtaggaagggagttcaataacttagagaggagaatagaaaatttgaacaagaagatggaagacggatttctagatgtctggacttacaatgcagaactcctggttctcatggaaaatgagagaactctagacttccatgactcaaatgtcaggaatctttacgacaaggtcagactacagcttaaggacaatgcaaaagagctgggtaatggttgtttcgagttctatcacaaatgtgataatgaatgtatggaaagtgtaagaaacggaacgtatgattacccacagtattcagaagaagcaagattaaaaagagaagaaataagtggagtaaagttggaatcaataggaatttaccaaatattgtcaatttattcaacagtggcaagttccctagtactggcaatcatgatggctggtctatctttatggatgtgttccaacgggtcgttacagtgcagaatttgcatttga

>H5N1_A/chicken/Bangladesh/35579/2018

cttgttaaaagcgatcatatctgcattggttatcatgcaaataactcgacaaagcaggttgacacaataatggaaaagaacgttactgttacacatgcccaagacatactggaaaagacacacaatgggaagctctgcgatctaaatggagtgaaacctctgattttaaaagattgtagtgtagcaggatggctcctcggaaacccaatgtgtgacgaattcatcaatgtgccagaatggtcttacatagtagagaaggccaatccagccaatggcctctgttacccaggaaatttcaacaattatgaagaattgaaacacctattgagtaggataaaccattttgagaaaatacggatcatccccaaagactcttggtcagatcatgaagcctcactgggggtgagtgcagcatgtccataccagggaaattcctccttctttagaaatgtggtatggcttatcaaaaagaacaatgcatacccaacaataaagaaaagctacaataataccaaccaagaagatctcttgatactgtgggggattcatcaccctaatgatgaagcagagcagacaaggatctatcaaaacccaaccacctacatttccattggaacatcaacactaaaccaaagattggtaccaagaatagccactagatccaaaataaacgggcaaagtggcaggatagatttcttctggacaattctaaaaccgaatgatgcaatccacttcgagagtaacgggaacttcattgctccagaatatgcatacaaaattgtcaaaaaaggagactcaacaattatgaaaagtgaagtggaatatggaaattgcaataccaggtgccagactcctataggggcgataaactctagtatgccatttcataacatacatcccctcaccattggagaatgtcccaaatatgtgaaatcaaacaaattagtccttgcgactgggctcagaaatagtcctcaaaaagaaagaagaagaaaaGGGagaggattgtttggagctatagcagggtttatagaaggaggatggcaggggatggtagatggttggtatgggtaccaccacagcaatgagcaggggagtgggtacgctgcagacaaagaatctacccaaaaggcaatagacggggtcaccaataaggtcaactcgatcattgacaaaatgaacacccagtttgaggccgttggaagggagttcaataacttagagaggagaatagaaaatttgaacaagaagatggaagatggatttctagatgtctggacttacaatgcagaactcctggttctcatggaaaatgagagaactctagacttccatgactcaaatgtcaggaatctttacgacaaggtcagactacagcttaaggacaatgcaaaagagctgggtaatggttgtttcgagttctatcacaaatgtgataatgaatgtatggaaagtgtaagaaacggaacgtatgattacccacagtattcagaagaagcaagattaaaaagagaagaaataagtggagtaaagttggaatcaataggaatttaccaaatattgtcaatttattcaacagtggcaagttccctagtactggcaatcatgatggctggtctatctttatggatgtgttccaacgggtcgttacagtgcagaatttgcatttga

>H5N1_A/chicken/Bangladesh/40446/2019

cttgttaaaagcgatcatatctgcattggttatcatgcaaataactcgacaaagcaggttgacacaataatggaaaagaacgttactgttacacatgcccaagacatactggaaaagacacacaacgggaagctctgcgatctaaatggagtgaaacctctgattttaaaagattgtagtgtagcaggatggctcctcggaaatccaatgtgtgacgaattcatcaatgtgccagaatggtcttacattgtagagaaggccaatccagccaatggcctctgttacccaggaaatttcaacgattatgaagaattgaaacacctattgagcaggataaaccattttgagaaaatacggatcatccccaaagattcttggtcagatcatgaagcctcactgggggtgagtgcagcatgtccataccagggaaattcctccttctttagaaatgtggtatggcttatcaaaaagaacaattcatacccaacaataaagaaaagctacaataataccaaccaagaagatctcttaatactgtgggggattcatcaccctaatgatgaagcagagcagacaaagctctatcaaaacccaaccacctacatttccattggaacatcaacactaaaccaaagattgatacccagaatagccactagatccaaaataaacgggcaaagtggcaggatagatttcttctggacaattctaaaaccgaatgatgcaatccacttcgagagtaacggaaacttcattgctccagaatatgcatacaaaattgtcaaaaaaggagactcaacaattatgaaaagtgaagtggaatatggaaattgcaataccaggtgccagactcctataggggcgataaactctagtatgccatttcataacatacatcccctcaccattggagaatgtcccaaatatgtgaaatcaaacaaattagtccttgcgacagggctcagaaatagtcctcaaaaagaaagaagaagaaaaGGGagaggattgtttggagctatagcagggtttatagaaggaggatggcaggggatggtagatggttggtatgggtaccaccatagcaatgagcaggggagtgggtacgctgcagacaaagaatctacccaaaaggcaatagacggggtcaccaataaggtcaactcgatcattgacaaaatgaacacccagtttgaggccgtaggaagggagttcaataacttagagagaagaatagaaaatttgaacaagaagatggaagacggatttctagatgtctggacttacaatgcagaactcttggttctcatggaaaatgagagaactctagacttccatgactcaaatgtcaggaatctttacgacaaggtcagactacagcttaaagacaatgcaaaagagctgggtaatggttgtttcgagttctatcacaaatgtgataatgaatgtatggaaagtgtaagaaacggaacatatgattacccacagtattcagaagaagcaagattaaaaagagaagaaataagtggagtaaagttggaatcaataggagtttaccaaatattgtcaatttattcaacagtggcaagttccctagtactggcaatcatgatggctggtctatctttatggatgtgttccaacggttcgttacagtgcagaatttgcatttga

>H5N1_A/chicken/Bangladesh/NRL-AI-1613/2017

cttgttaaaagcgatcatatctgcattggttatcatgcaaataactcgacaaagcaggttgacacaataatggaaaagaacgttactgttacacatgcccaagacatactggaaaagacacacaatgggaagctctgcgatctaaatggagtgaaacctctgattttaaaagattgtagtgtagcaggatggctcctcggaaacccaatgtgtgacgaattcatcaatgtgccagaatggtcttacatagtagagaaggccaatccagccaatgacctttgttacccagggaatttcaacgattatgaggaattgaagcacctattaagcaggataaaccattttgagaaaatacggatcatccccaaagattcttggtcagatcatgaagcctcactgggggtgagtgcagcatgtccataccagggaaattcctccttcttcagaaatgtggtatggcttatcaaaaaggacaatacatacccaacaataaagaaaagctacaataataccaaccaagaagatctcttgatactgtgggggattcatcaccctaatgatgaagcagagcagataaggctctatcaaaacccaaccacctacatttccattggaacatcaacactaaaccaaagattggtaccaaaaatagccactagatccaaaataaacgggcaaagtggcaggatagatttcttctggacaattctaaaaccgaatgatgcaatccacttcgagagcaacggaaacttcattgctccagagtatgcatacaaaattgtcaaaaagggggactcaacaattatgaaaagtgaagtggaatatggaaattgcaataccaggtgccagactcctataggggcgataaactctagtatgccattccacaacatacatcccctcaccatcggagaatgtcccaaatatgtgaaatcaaacaaattagtccttgcgactgggctcagaaatagtcctcaaagagagagaagaagaaaaGGGagaggattgtttggagctatagcaggatttatagagggaggatggcaggggatggtagatggttggtatgggtaccaccacagcaatgagcaggggagtgggtacgctgcagacaaagaatctacccaaaaggcaatagacggggtcaccaataaggtcaactcgatcattgacaaaatgaacacccagtttgaggccgtaggaagggagtttaatagcttagagaggagaatagaaaatttgaacaagaagatggaagacggatttctcgatgtctggacttacaatgcagaacttctggttctcatggaaaatgagagaactctagacttccatgactcaaatgtcaggaatctttacgacaaggtcagactacagcttaaggacaatgcaaaagagctgggtaatggttgtttcgagttctatcacaagtgtgataatgaatgtatggaaagtgtaagaaacggaacgtatgattacccgcagtattcagaagaatcaagattaaaaagagaagaaataagtggagtaaagttggaatcaataggaatttaccaaatattgtcaatttattcaacagtggcaagttccctagtactggcaatcatgatggctggtctatctttatggatgtgttccaacgggtcgttacagtgcagaatttgcatttga

>H5N1_A/chicken/Bangladesh/NRL-AI-3237/2017

cttgttaaaagcgatcatatctgcattggttatcatgcaaataactcgacaaagcaggttgacacaataatggaaaagaacgttactgttacacatgcccaagacatactggaaaagacacacaatgggaagctctgcgatctaaatggagtgaaacctctgattttaaaggattgtagtgtagcaggatggctcctcggaaacccaatgtgtgacgaattcatcaatgtgccagaatggtcttacatagtagagaaggccaatccagccaatggcctctgttacccagggactttcaacgattatgaagaattgaaacacctattgagcaggataaaccattttgagaaaatacgggtcatccccagagattcttggtcagatcatgaagcctcactgggggtgagtgcagcatgcccataccagggaaattcctccttcttcagaaacgtggtatggcttatcaaaaaggacaatacatacccaacaataaagaaaagctacaataataccaaccaagaagatctcttgatactgtgggggattcatcaccctaatgatgaagcagagcagacaaggctctatcaaaacccgaccacctacatttccattggaacatcaacactaaaccaaagattggtaccaaaaatagccactagatccaaaataaacgggcaaagtggcaggatagatttcttctggacaattctaaaaccgaatgatgcaatccacttcgagagtaacggaaacttcattgctccagagtatgcatacaaaattatcaaaaagggagactcaacaattatgaaaagtgaagtggaatatggaaattgcaataccaggtgccagacccctataggggcgataaactctagtatgccattccacaacatacatcccctcaccatcggagaatgtcccaaatatgtgaaatcaaacaaattagtccttgcgactgggctcagaaatagtcctcaaagagaaagaagaagaaaaGGGagaggattgtttggagctatagcaggatttatagagggaggatggcaggggatggtagatggttggtatgggtaccaccacagcaatgagcaggggagtgggtacgctgcagacaaagaatctacccaaaaggcaatagacggggtcactaataaggtcaactcgatcattgacaaaatgaacacccaatttgaggccgtaggaagggagtttaataacttagagaggagaatagaaaatttgaacaagaagatggaagacggatttctagatgtctggacttacaatgcagaacttctggttctcatggaaaatgagagaactctagacttccatgactcaaatgtcaggaatctttacgacaaggtcagactacagcttaaggacaatgcaaaagagctgggtaatggttgtttcgagttctatcacaaatgtgataatgaatgtatggaaagtgtaagaaacggaacgtatgattacccacagtattcagaagaatcaagattaaaaagagaagaaataagtggggtaaagttggaatcaataggaatttaccaaatattgtcaatttattcaacagtggcaagttccctagtactggcaatcatgatggctggtctatctttatggatgtgttccaacgggtcgttacagtgcagaatttgcatttga

>H5N1_A/chicken/Bangladesh/NRL-AI-8251/2017

cttgttaaaagcgatcatatctgcattggttatcatgcaaataactcgacaaagcaggttgacacaataatggaaaagaacgttactgttacacatgcccaagacatactggaaaagacacacaatgggaagctctgcgatctaaatggagtgaaacctctgattttaaaagattgtagtgtagcaggatggctcctcggaaacccaatgtgtgacgaattcatcaatgtgccagaatggtcttacatagtagagaaggccaatccagccaatggcctctgttacccagggaatttcaacgattatgaagaattgaaacacctattgagcaggataaaccattttgagaaaatacggatcatccccaaagattcttggtcagatcatgaagcctcactgggggtgagtgcagcatgcccataccagggaaattcctccttcttcagaaacgtggtatggcttattaaaaaggacaatacatacccaacaataaagaaaagctacaacaataccaaccgagaagatctcttgatactgtgggggattcatcaccctaatgatgaagcagagcagacaaggctctatcaaaacccaaccacttacatttccattggaacatcaacactaaatcaaagattggtaccaaaaatagccactagatccaaaataaacgggcaaagtggcaggatagatttcttctggacaattctaaaaccgaatgatgcaatccacttcgagagtaacggaaacttcattgctccagagtatgcatacaaaattgtcaaaaagggggactcaacaattatgaaaagtgaagtggaatatggaaattgcaataccaggtgccagactcctataggggcgataaactctagtatgccattccacaacatacatcccctcaccatcggagaatgtcccaaatatgtgaaatcaaacaaattagtccttgcgactgggctcagaaatagtcctcaaagagaaagaagaagaaaaGGGagaggattgtttggagctatagcagggtttatagagggaggatggcaggggatggtagatggttggtatgggtatcaccacagcaatgagcaggggagtgggtacgctgcagacaaagaatctacccaaaaggcaatagacggggtcaccaataaggtcaactcgatcattgacaaaatgaacacccagttcgaggccgtaggaagggagtttaatagcttagagaggagaatagaaaatctgaacaagaagatggaagacggatttctagatgtctggacttacaatgcagaactcctggttctcatggaaaatgagagaactctagacttccatgactcaaatgtcaggaatctttacgacaaggtcagactacagcttaaggacaatgcaaaagagttgggtaatggttgtttcgagttctatcacaaatgtgataatgaatgtatggaaagtgtaagaaacggaacatatgattacccacagtattcagaagaatcaagattaaaaagagaagaaataagtggagtaaagttggaatcaataggaatttaccaaatattgtcaatttattcaacagtggcaagttccctagtactggcaatcatgatggctggtctatctttatggatgtgttccaacgggtcgttacagtgcagaatttgcatttga

>H5N1_A/chicken/Bangladesh/NRL-AI-8468/2017

cttgttaaaagcgatcatatctgcattggttatcatgcaaacaactcgacaaagcaggttgacacaataatggaaaagaacgttactgttacacatgcccaagacatactggaaaagacacacaatgggaagctctgcgatctgaatggagtgaaacctctgattttaaaagattgtagtgtagcaggatggctcctcggaaacccaatgtgtgacgaattcatcaatgtgccagagtggtcttacatagtagagaaggccaatccagccaatggcctctgttacccagggaatttcaacgattatgaagaattgaaacacctattgagcaggataaaccattttgagaaaatacggatcatccccaaagattcttggtcagatcatgaagcctcactgggggtgagtgcagcatgtccataccagggaaattcctccttctttagaaatgtggtatggcttatcaaaaagaacaatgcatacccaacaataaagaaaagctacaataataccaaccaagaagatctcttgatattgtgggggatccatcaccctaatgatgaagcagagcagacaaggctctatcaaaacccaaccacctacatttccattggaacatcaacactaaaccaaagattggtaccaaaaatagccactagatccaaaataaacgggcaaagtggcagggtagatttcttctggacaattctaaaaccgaatgatgcaattcacttcgagagtaacggaaacttcattgctccagaatatgcatacaaaattgtcaaaaaaggagactcaacaattatgaaaagtgaagtggaatatggaaattgcaataccaggtgccagactcctataggggcgataaactctagtatgccattccataacatacaccccctcaccattggagaatgtcccaaatatgtgaaatcaaacaaattagtccttgcgactgggctcagaaataatcctcaaagagaaagaagaagaaaaGGGagaggattgtttggagctatagcagggtttatagagggaggatggcaggggatggtagatggttggtatgggtaccaccacagcaatgagcaagggagtgggtacgctgcagacaaagaatctacccaaaaggcaatagacggggtcaccaataaggtcaactcgatcattgacaaaatgaacacccagtttgaggccgtaggaagggagttcaataacttagagaggagaatagaaaatttgaacaagaagatggaagacggatttctagatgtctggacttacaatgcagaacttctggttctcatggaaaatgagagaactctagacttccatgactcaaatgtcaggaatctttacgacaaggtcagactacagcttaaggacaatgcaaaagagctgggtaatggttgtttcgagttctatcacaaatgtgataatgaatgtatggaaagtgtaagaaacggaacgtatgattacccacagtattcagaagaagcaagattaaaaagagaggaaataagtggagtaaagttggagtcaataggaatttaccaaatattgtcaatttattcaacagtggcaagttccctagtactggcaatcatgatggctggtctatctttatggatgtgttccaacgggtcgttacagtgcagaatttgcatttga

>H5N1_A/chicken/Banten/UT6025/2006

cttgttaaaagtgatcagatttgcattggttaccatgcaaacaattcaacagagcaggttgacacaataatggaaaagaacgttactgttacacatgcccaagacatactggaaaagacacacaacgggaagctctgcgatctagatggagtgaaacctctaattttaagagattgtagtgtagctggatggctcctcgggaacccaatgtgtgacgaattcatcaatgtaccggaatggtcttacatagtggagaaggccaatccagccaatgacctctgttacccagggagtttcaacgactatgaagaactgaaacacctattgagcagaataaaccattttgagaaaattcagatcatccccaaaagttcttggtccgatcatgaagcctcatcaggagtgagctcagcatgcccatacctgggaagtccctccttttttagaaatggggtatggcttatcaaaaagaacaatacatacccaacaataaagaaaagctacaataataccaatcaagaagatcttttggtactgtgggggattcaccatcctaatgatgcagcagagcaaacaaggctatatcaaaacccaaccacctatatttccgttgggacatcaacactaaatcagagatcggtaccaaaaatagctactagatccaaagtaaacgggcaaagtggaaggatggagttcttctggacaattttaaaaccgaatgatgcaatcaacttcgagagtaatggaaatttcattgctccagaatatgcatacaaaattgtcaagaaaggggactcagcaattatgaaaagtgaattggaatatggtaattgcaacaccaagtgtcaaactccaatgggggcgataaactctagtatgccattccacaacatacaccctctcaccatcggggaatgccccaaatatgtgaaatcaaacagattagtccttgcaacagggctcagaaatagccctcaaagggagagaagaagaaaaaagagaggactatttggagctatagcaggttttatagagggaggatggcagggaatggtagatggttggtatgggtaccatcatagcaatgagcaggggagtgggtacgctgcagacaaagaatccactcaaaaggcaatagatggagtcaccaataaggtcaactcaatcattgacaaaatgaacactcagtttgaggccgttggaagggaatttaataacttagaaaggagaatagagaatttaaacaagaagatggaagacggatttctagatgtctggacttataatgccgaacttctggttctcatggaaaatgagagaactctagactttcatgactcaaatgttaagaacctctatgacaaggtccgactacagcttagggataatgcaaaggagctgggtaacggttgtttcgagttctatcacaaatgtgataatggatgtatggaaagtataagaaacggaacgtataactacccgcagtattcagaagaagcaagattaaaaagagaggaaataagtggagtaaaattggaatcaataggaacttatcaaatactgtcaatttattcaacagtggcgagttccctagcactggcaatcatgatggctggtctatctttatggatgtgttccaatggatcgttacaatgcagaatttgcatttga

>H5N1_A/chicken/Bhutan/1029/2012

cttgttaaaagcgatcatatttgcattggttatcatgcaaataactcgacaaagcaggttgacacaataatggaaaagaacgttactgttacacatgcccaagacatactggaaaagacacacaacgggaagctctgcgatctaaatggagtgaaacctctgattttaaaggattgtagtgtagcaggatggctcctcggaaacccaatgtgtgacgaattcatcaatgtgccagaatggtcttacatagtagagaaggccaatccagccaatggcctctgttacccagggaatttcaacgattatgaagaattgaaacacctattgagcaggataaaccattttgagaaaatacggatcatccccaaagattcttggtcagatcatgaagcctcattgggggtgagtgcagcatgtccataccagggaaattcctccttcttcagaaatgtggtatggcttatcaaaaaggacaatgcatacccaacaataaagaaaagctacaacaataccaaccaagaagatcttttggtcctgtgggggattcaccatcctaacgatgaagcagagcagacaatgctctatcaaaacccaaccacctatatttccattgggacatcaacactaaaccagagattggtgccaaaaatagccactagatccaaaataaacgggcaaagcggcaggatagatttcttctggacaattttaaaaccgaatgatgcaatccacttcgagagtaacggaaatttcattgctccagagtatgcatacaaaattgtcaagaaaggagactcaacaattatgaaaagtgaagtggaatatggtaactgcaacaccaggtgtcagactcctataggggcgataaactctagtatgccattccacaacatacatcccctcaccatcggagaatgtcccaaatatgtgaaatcaaacaaattagtccttgcgactgggctcagaaatagtcctcaaagagagagaagaagaaaaGGGagaggattgtttggagctatagcagggtttatagagggaggatggcaaggaatggtagatggttggtatgggtaccaccacagcaacgagcaggggagtgggtacgctgcagacaaagaatctactcaaaaggcaatagacggagtcaccaataaggtcaactcgatcattgacaaaatgaacactcagtttgaggccgtaggaagggagtttaataacttagagaggagaatagagaatttgaacaagaagatggaagacggattcctagatgtctggacttataatgctgaacttctggttctcatggaaaatgagagaactctagacttccatgactcaaatgtcaggaacctttacgacaaggtcagactacagcttaaggacaatgcaaaagaattgggtaacggttgtttcgagttctatcacaaatgtaataatgaatgtatggaaagtgtaagaaacggaacgtatgactacccgcagtattcagaagaagcaagattaaaaagagaagaaataagtggagtaaagttggaatcaataggaatttaccaaatattgtcaatttattcaacagtagcgagttccctagtactggcaatcatgatggctggtctatctttatggatgtgttccaacgggtcgttacagtgcagaatttgcatttga

>H5N1_A/chicken/Bhutan/1031/2012

cttgttaaaagcgatcatatttgcattggttatcatgcaaataactcgacaaagcaggttgacacaataatggaaaagaacgttactgttacacatgcccaagacatactggaaaagacacacaacgggaagctctgcgatctgaatggagtgaaacctctgattttaaaagattgtagtgtagcaggatggctcctcggaaacccaatgtgtgacgaattcatcaatgtgccagaatggtcttacatagtagagaaggccaatccagccaatggcctctgttacccagggaatttcaacgattatgaagaattggaacacctattgagcaggataaaccattttgagaaaatacggatcatccccaaagattcttggtcagaccatgaagcctcattgggggtgagtgcagcatgtccataccagggaaattcttccttcttcagaaatgtggtatggcttaccaaaaaggacaatgcatacccaacaataaagaaaagctacaacaataccaaccaagaagatctcttggtactgtgggggattcaccatcctaacgatgaagcagagcagacaaggctctatcaaaacccaaccacctatatttccattgggacatcaacactaaaccagagattggtgccaaaaatagccactagatccaaaataaacgggcaaagcggcaggatagatttcttctggacaattttaaaaccgaatgatgcaatccacttcgagagtaacggaaatttcattgctccagagtatgcatacaaaattgtcaagaaaggggactcaacaattatgaaaagtgaagtggaatatggtaactgcaacaccaggtgtcagactcctataggggcgataaactctagtatgccattccacaacatacatcccctcaccatcggagaatgtcccaaatatgtgaaatcaaacaaattagtccttgcgactgggctcagaaatagtcctcaaagagagagaagaagaaaaGGGagaggattgtttggagctatagcagggtttatagagggaggatggcagggaatggtagatggttggtatgggtaccaccacagcaacgagcaggggagtgggtacgctgcagacaaagaatctactcaaaaggcaatagacggagtcaccaataaggtcaactcgatcattgacaaaatgaacactcagtttgaggccgtaggaagggagtttaataacttagagaggagaatagagaatttgaacaagaagatggaagacggattcctagatgtctggacttataatgctgaacttctggttctcatggaaaatgagagaactctagacttccatgactcaaatgtcaggaacctttacgacaaggtcagactacagcttaaggacaatgcaaaagagttgggtaacggttgtttcgagttctatcacaaatgtgataatgaatgtatggaaagtgtaagaaacggaacgtatgactacccacagtattcagaagaagcaagattaaaaagagaagaaataagtggagtaaagttggaatcaataggaatttaccaaatattgtcaatttattcaacagtggcgagttccctagtactggcaatcatgatggctggtctatccttatggatgtgttccaacgggtcgttacagtgcagaatttgcatttga

>H5N1_A/chicken/Bhutan/248015/2010

cttgttaaaagtgatcagatttgcattggttaccatgcaaacaactcgacagagcaggttgacacaataatggaaaagaacgtcactgttacacacgcccaagacatactggaaaaaacacacaacgggaagctctgtgatctagacggagtgaagcctctaattttgagagattgtagtgtagctggatggctcctcgggaatccaatgtgtgacgaattcctcaatgtgcccgaatggtcttacatagtggaaaagatcaatccagccaatgacctctgttacccagggaatttcaacgactatgaagaactgaaacacctactgagcagaataaaccattttgagaaaattcagatcatctccaaaagttcttggtcagatcatgaagcctcatcaggggtgagctcagcatgcccataccagggaaggtcctccttttttagaaatgtggtatggcttatcaaaaagaacgatgcatacccaacaataaagataagttacaataataccaaccaagaagatcttttggtattgtgggggattcaccatccaaatgatgctgcagagcagacaaaactttatcaaaacccaaccacctatatttctgttgggacatcaacactaaacctgagattggtaccaaaaatagctactagatccaaagtaaacgggcaaagtggaaggatggagttcttttggacaattttaaaaccgaatgatacaataaattttgagagtaatggaaatttcattgctccagaaaatgcatacaaaattgtcaagaaaggggactcaacgatcatgaaaagtgaattggaatatggtaactgcaacaccaagtgtcaaactccagtaggagcgataaactctagtatgccattccacaacatccaccctctcaccataggggaatgccccaaatatgtgaaatcaaacagattagtccttgcgactgggctcagaaatagccctcaaggagagagaagaagaaaaaagagaggactatttggagctatagcaggttttatagagggaggatggcagggaatggtagatggttggtatgggtaccaccatagtaacgagcaggggagtgggtacgctgcagacaaagaatccactcaaaaggcaatagatggagtcaccaataaggtcaactcgatcattgacaaaatgaacactcagtttgaggccgttggaagggaatttaataacttagaaaggagaatagagaatttaaacaagaagatggaagacggatttctagatgtctggacttataatgctgaacttctggttctcatggaaaatgagagaactctagactttcatgactcaaatgtcaagaatctttacgacaaggtccgactacagcttagagataatgcaaaggagcttggtaacggttgtttcgagttctaccacagatgtgataatgaatgtatggaaagtgtaagaaacggaacgtatgactatcctcagtattcagaagaatcaagattaaaaagagaggaaataagtggagtaaaattggagtcaataggaacttatcaaatactgtcaatttattcaacagtggcgagctccctagtgctggcaatcatggtggctggtctatctttatggatgtgctccaatggatcgttacaatgcagaatttgcatttga

>H5N1_A/chicken/Bhutan/258/2012

cttgttaaaagcgatcatatttgcattggttatcatgcaaataactcgacaaagcaggttgacacaataatggaaaagaacgttactgttacacatgcccaagacatactggaaaagacacacaacgggaagctctgcgatctaaatggagtgaaacctctgattttaaaagattgtagtgtagcaggatggctcctcggaaacccaatgtgtgacgaattcatcaatgtgccagaatggtcttacatagtagagaaggccaatccagccaatggcctctgttacccagggaatttcaacgattatgaagaattgaaacacctattgagcaggataaaccattttgagaaaatacggatcatccccaaagattcttggtcagatcatgaagcctcattgggggtgagtgcagcatgtccataccagggaaattcctccttcttcagaaatgtggtatggcttatcaaaaaggacaatgcatacccaacaataaagaaaagctacaacaataccaaccaagaagatcttttggtactgtgggggattcaccatcctaacgatgaaacagagcagacagggctctatcaaaacccaaccacctatatttccattgggacatcaacactaaaccagagattggtgccaaaaatagccactagatccaaaataaacgggcaaagcggcaggatagatttcttctggacaattttaaaaccgaatgatgcaatccacttcgagagtaacggaaatttcattgctccagagtatgcatacaaaattgtcaagaaaggagactcaacaattatgaaaagtgaagtggaatatggtaactgcaacaccaggtgtcagactcctataggggcgataaactctagtatgccattccacaacatacatcccctcaccatcggagaatgtcccaaatatgtgaaatcaaacaaattagtccttgcgactgggctcagaaatagtcctcaaagagagagaagaagaaaaGGGagaggattgtttggagctatagcagggtttatagagggaggatggcagggaatggtagatggttggtatgggtaccaccacagcaacgagcaggggagtgggtacgctgcagacaaagaatctactcaaaaggcaatagacggagtcaccaataaggtcaactcgatcattgacaagatgaacactcagtttgaggccgtaggaagggagtttaataatttagagaggagaatagagaatttgaacaagaagatggaagacggattcctagatgtctggacttataatgctgaacttctggttctcatggaaaatgagagaactctagacttccatgactcaaatgtcaggaacctttacgacaaggtcagactacagcttaaggacaatgcaaaagaattgggtgacggttgtttcgagttctatcacaaatgtaataatgaatgtatggaaagtgtaagaaacggaacgtatgactacccgcagtattcagaagaagcaagattaaaaagagaagaaataagtggagtaaagttggaatcaataggaatttaccaaatattgtcaatttattcaacagtagcgagttccctagtactggcaatcatgatggctggtctatctttatggatgtgttccaacgggtcgttacagtgcagaatttgcatttga

>H5N1_A/chicken/Bhutan/298/2012

cttgttaaaagcgatcatatttgcattggttatcatgcaaataactcgacaaagcaggttgacacaataatggaaaagaacgttactgttacacatgcccaagacatactggaaaagacacacaacgggaagctctgcgatctaaatggagtgaaacctctgattttaaaagattgtagtgtagcaggatggctcctcggaaacccaatgtgtgacgaattcatcaatgtgccagaatggtcttacatagtagagaaggccaatccagccaatggcctctgttacccagggaatttcaacgattatgaagaattgaaacacctattgagcaggataaaccattttgagaaaatacggatcatccccaaagattcttggtcagatcatgaagcctcattgggggtgagtgcagcatgtccataccagggaaattcctccttcttcagaaatgtggtatggcttatcaaaaaggacaatgcatacccaacaataaagaaaagctacaacaataccaaccaagaagatcttttggtactgtgggggattcaccatcctaacgatgaaacagagcagacagggctctatcaaaacccaaccacctatatttccattgggacatcaacactaaaccagagattggtgccaaaaatagccactagatccaaaataaacgggcaaagcggcaggatagatttcttctggacaattttaaaaccgaatgatgcaatccacttcgagagtaacggaaatttcattgctccagagtatgcatacaaaattgtcaagaaaggagactcaacaattatgaaaagtgaagtggaatatggtaactgcaacaccaggtgtcagactcctataggggcgataaactctagtatgccattccacaacatacatcccctcaccatcggagaatgtcccaaatatgtgaaatcaaacaaattagtccttgcgactgggctcagaaatagtcctcaaagagagagaagaagaaaaGGGagaggattgtttggagctatagcagggtttatagagggaggatggcagggaatggtagatggttggtatgggtaccaccacagcaacgagcaggggagtgggtacgctgcagacaaagaatctactcaaaaggcaatagacggagtcaccaataaggtcaactcgatcattgacaaaatgaacactcagtttgaggccgtaggaagggagtttaataatttagagaggagaatagagaatttgaacaagaagatggaagacggattcctagatgtctggacttataatgctgaacttctggttctcatggaaaatgagagaactctagacttccatgactcaaatgtcaggaacctttacgacaaggtcagactacagcttaaggacaatgcaaaagaattgggtgacggttgtttcgagttctatcacaaatgtaataatgaatgtatggaaagtgtaagaaacggaacgtatgactacccgcagtattcagaagaagcaagattaaaaagagaagaaataagtggagtaaagttggaatcaataggaatttaccaaatattgtcaatttattcaacagtagcgagttccctagtactggcaatcatgatggctggtctatctttatggatgtgttccaacgggtcgttacagtgcagaatttgcatttga

>H5N1_A/chicken/Bhutan/317/2012

cttgttaaaagcgatcatatttgcattggttatcatgcaaataactcgacaaagcaggttgacacaataatggaaaagaacgttactgtcacacatgcccaagacatactggaaaagacacacaacgggaagctctgcgatctaaatggagtgaaacctctgattttaaaagattgtagtgtagcaggatggctcctcggaaacccaatgtgtgacgaattcatcaatgtgccagaatggtcttacatagtagagaaggccaatccagccaatggcctctgttacccagggaatttcaacgattatgaagaattgaaacacctattgagcaggataaaccattttgagaaaatacggatcatccccaaagattcttggtcagatcatgaagcctcattgggggtgagtgcagcatgtccataccagggaaattcctccttcttcagaaatgtggtatggcttatcaaaaaggacaatgcatacccaacaataaagaaaagctacaacaataccaaccaagaggatcttttggtactgtgggggattcaccatcctaacgatgaagcagagcagacaaggctctatcaaaacccaaccacctatatttccattgggacatcaacactaaaccagagattggtgccaaaaatagccactagatccaaaataaacgggcaaagcggcaggatagatttcttctggacaattttaaaaccgaatgatgcaatccacttcgagagtaacggaaatttcattgctccagagtatgcatacaaaattgtcaagaaaggagactcaacaattatgaaaagtgaagtggaatatggtaactgcaacaccaggtgtcagactcctataggggcgataaactctagtatgccattccacaacatacatcccctcaccatcggagaatgtcccaaatatgtgaaatcaaacaaattagtccttgcgactgggctcagaaatagtcctcaaagagagagaagaagaaaaGGGagaggattgtttggagctatagcagggtttatagagggaggatggcagggaatggtagatggttggtatgggtaccaccacagcaacgagcaggggagtgggtacgctgcagacaaagaatctactcaaaaggcaatagacggagtcaccaataaggtcaactcgatcattgacaaaatgaacactcagtttgaggccgtaggaagggagtttaataacttagagaggagaatagagaatttgaacaagaagatggaagacggattcctagatgtctggacttataatgctgaacttctggttctcatggaaaatgagagaactctagacttccatgactcaaatgtcaggaacctttacgacaaggtcagactacagcttaaggacaatgcaaaagaattgggtaacggttgtttcgagttctatcacaaatgtaataatgaatgtatggaaagtgtaagaaacggaacgtatgactacccgcagtattcagaagaagcaagattaaaaagagaagaaataagtggagtaaagttggaatcaataggaatttaccaaatattgtcaatttattcaacagtagcgagttccctagtactggcaatcatgatggctggtctatctttatggatgtgttccaacgggtcgttacagtgcagaatttgcatttga

>H5N1_A/chicken/Bhutan/331/2012

cttgttaaaagcgatcatatttgcattggttatcatgcaaataactcgacaaagcaggttgacacaataatggaaaagaacgttactgttacacatgcccaagacatactggaaaagacacacaatgggaagctctgcgatctaaatggagtgaaacctctgattttaaaagattgtagtgtagcaggatggctcctcggaaacccaatgtgtgacgaattcatcaatgtgccagaatggtcttacatagtagagaaggccaatccagccaatggcctctgttacccagggaatttcaacgattatgaagaattgaaacacctattgagcaggataaaccattttgagaaaatacggatcatccccaaagattcttggtcagatcatgaagcctcattgggagtgagtgcagcatgtccataccagggaaattcctccttcttcagaaatgtagtatggcttatcaaaaaggacaatgcatacccaacaataaagaaaagctacaacaataccaaccaagaagatctcttggtactgtgggggattcaccatcctaatgatgaagcagagcagacaaggctctatcaaaacccaaccacctatatttccattgggacatcaacactaaaccagagattggtaccaaaaatagccactagatccaaaataaacgggcaaagcggcagaatagatttcttctggacaattttaaaaccgaatgatgcaatccacttcgagagtaacggaaatttcattgctccagagtatgcatacaaaattgtcaagaaaggagactcaacaattatgaaaagtgaagtggaatatggtaactgcaacaccaggtgtcagactcctataggggcgataaactctagtatgccattccacaacatacatcccctcaccatcggagaatgtcccaaatatgtgaaatcaaacaaattagtccttgcgactgggctcagaaatagccctcaaagagagagaagaagaaaaGGGagaggattgtttggagctatagcaggttttatagagggaggatggcagggaatggtagatggttggtatgggtaccaccacagcaacgagcaggggagtgggtacgctgcagacaaagaatctacccaaaaggcaatagacggagtcaccaataaggtcaactcgatcattgacaaaatgaacacccagtttgaggccgtaggaagggagtttaataacttagagaggagaatagagaatttgaacaagaagatggaagacggattcctagatgtctggacttataatgctgaacttctggttctcatggaaaatgagagaactctagacttccatgactcaaatgtcaggaacctttacgacaaggtcagactacagcttaaggacaatgcaaaagagttgggtaacggttgtttcgagttctatcacaaatgtaataatgaatgtatggaaagtgtaagaaacggaacgtatgactacccgcagtattcagaagaagcaagattaaaaagagaagaaataagtggagtaaagttggaatcaataggaatttaccaaatattgtcaatttattcaacagtggcgagttccctagtactggcaatcatgatggctggtctatctttatggatgtgttccaacgggtcgttacagtgcagaatttgcatttga

>H5N1_A/chicken/Bhutan/347/2012

cttgttaaaagcgatcatatttgcattggttatcatgcaaataactcgacaaagcaggttgacacaataatggaaaagaacgttactgttacacatgcccaagacatactggaaaagacacacaacgggaagctctgcgatctaaatggagtgaaacctctgattttaaaagattgtagtgtagcaggatggctcctcggaaacccaatgtgtgacgaattcatcaatgtgccagaatggtcttacatagtagagaaggccaatccagccaatggcctctgttacccagggaatttcaacgattatgaagaattgaaacacctattgagcaggataaaccattttgagaaaatacggatcatccccaaagattcttggtcagatcatgaagcctcattgggggtgagtgcagcatgtccataccagggaaattcctccttcttcagaaatgtggtatggcttatcaaaaaggacaatgcatacccaacaataaagaaaagctacaacaataccaaccaagaagatcttttggtactgtgggggattcaccatcctaacgatgaagcagagcagacaatgctctatcaaaacccaaccacctatatttccattgggacatcaacactaaaccagagattggtgccaaaaatagccactagatccaaaataaacgggcaaagcggcaggatagatttcttctggacaattttaaaaccgaatgatgcaatccacttcgagagtaacggaaatttcattgctccagagtatgcatacaaaattgtcaagaaaggagactcaacaattatgaaaagtgaagtggaatatggtaactgcaacaccaggtgtcagactcctataggggcgataaactctagtatgccattccacaacatacatcccctcaccatcggagaatgtcccaaatatgtgaaatcaaacaaattagtccttgcgactgggctcagaaatagtcctcaaagagagagaagaagaaaaGGGagaggattgtttggagctatagcagggtttatagagggaggatggcagggaatggtagatggttggtatgggtaccaccacagcaacgagcaggggagtgggtacgctgcagacaaagaatctactcaaaaggcaatagacggagtcaccaataaggtcaactcgatcattgacaaaatgaacactcagtttgaggccataggaagggagtttaataacttagagaggagaatagagaatttgaacaagaagatggaagacggattcctagatgtctggacttataatgctgaacttctggttctcatggaaaatgagagaactctagacttccatgactcaaatgtcaggaacctttacgacaaggtcagactacagcttaaggacaatgcaaaagaattgggtaacggttgtttcgagttctatcacaaatgtaataatgaatgtatggaaagtgtaagaaacggaacgtatgactacccgcagtattcagaagaagcaagattaaaaagagaagaaataagtggagtaaagttggaatcaataggaatttaccaaatattgtcaatttattcaacagtagcgagttccctagtactggcaatcatgatggctggtctatctttatggatgtgttccaacgggtcgttacagtgcagaatttgcatttga

>H5N1_A/chicken/Bhutan/367/2012

cttgttaaaagcgatcatatttgcattggttatcatgcaaataactcgacaaagcaggttgacacaataatggaaaagaacgttactgttacacatgcccaagacatactggaaaagacacacaacgggaagctctgcgatctaaatggagtgaaacctctgattttaaaagattgtagtgtagcaggatggctcctcggaaacccaatgtgtgacgaattcatcaatgtgccagaatggtcttacatagtagagaaggccaatccagccaatggcctctgttacccagggaatttcaacgattatgaagaattgaaacacctattgagcaggataaaccattttgagaaaatacagatcatccccaaagattcttggtcagatcatgaagcctcattgggggtgagtgcagcatgtccataccagggaaattcctccttcttcagaaatgtggtatggcttatcaaaaaggacaatgcatacccaacaataaagaaaagctacaacaataccaaccaagaagatcttttggtactgtgggggattcaccatcctaacgatgaagcagagcagacaaggctctatcaaaacccaaccacctatatctccattgggacatcaacactaaaccagagattggtgccaaaaatagccactagatccaaaataaacgggcaaagcggcaggatagatttcttctggacaattttaaaaccgaatgatgcaatccacttcgagagtaacggaaatttcattgctccagagtatgcatacaaaattgtcaagaaaggagactcaacaattatgaaaagtgaagtggaatatggtaactgcaacaccaggtgtcagactcctataggggcgataaactctagtatgccattccacaacatacatcccctcaccatcggagaatgtcccaaatatgtgaaatcaaacaaattagtccttgcgactgggctcagaaatagtcctcaaagagagagaagaagaaaaGGGagaggattgtttggagctatagcagggtttatagagggaggatggcagggaatggtagatggttggtatgggtaccaccacagcaacgagcaggggagtgggtacgctgcagacaaagaatctactcaaaaggcaatagacggagtcaccaataaggtcaactcgatcattgacaaaatgaacactcagtttgaggctgtaggaagggagtttaataacttagagaggagaatagagaatttgaacaagaagatggaagacggattcctagatgtctggacttataatgctgaacttctggttctcatggaaaatgagagaactctagacttccatgactcaaatgtcaggaacctttacgacaaggtcagactacagcttaaggacaatgcaaaagaattgggtaacggttgtttcgagttctatcacaaatgtaataatgaatgtatggaaagtgtaagaaacggaacgtatgactacccgcagtattcagaagaagcaagattaaaaagagaagaaataagtggagtaaagttggaatcaataggaatttaccaaatattgtcaatttattcaacagtagcgagttccctagtactggcaatcatgatggctggtctatctttatggatgtgttccaacgggtcgttacagtgcagaatttgcatttga

>H5N1_A/chicken/Bhutan/406/2012

cttgttaaaagcgatcatatttgcattggttatcatgcaaataactcgacaaagcaggttgacacaataatggaaaagaacgttactgttacacatgcccaagacatactggaaaagacacacaacgggaagctctgcgatctaaatggagtgaaacctctgattttaaaagattgtagtgtagcaggatggctcctcggaaacccaatgtgtgacgaattcatcaatgtgccagaatggtcttacatagtagagaaggccaatccagccaatggcctctgttacccagggaatttcaacgattatgaagaattgaaacacctattgagcaggataaaccattttgagaaaatacggatcatccccaaagattcttggtcagatcatgaagcttcattgggggtgagtgcagcatgtccataccagggaaattcctccttcttcagaaatgtggtatggcttatcaaaagggacaatgcatacccaacaataaagaaaagctacaacaataccaaccaagaagatcttttggtactgtgggggattcaccatcctaacgatgaagcagagcagacaatgctctatcaaaacccaaccacctatatttccattgggacatcaacactaaaccagagattggtgccaaaaatagccactagatccaaaataaacgggcaaagcggcaggatagatttcttctggacaattttaaaaccgaatgatgcaatccacttcgagagtaacggaaatttcattgctccagagtatgcatacaaaattgtcaagaaaggagactcaacaattatgaaaagtgaagtggaatatggtaactgcaacaccaggtgtcagactcctataggggcgataaactctagtatgccattccacaacatacatcccctcaccatcggagaatgtcccaaatatgtgaaatcaaacaaattagtccttgcgactgggctcagaaatagtcctcaaagagagagaagaagaaaaGGGagaggattgtttggagctatagcagggtttatagagggaggatggcagggaatggtagatggttggtatgggtaccaccacagcaacgagcaggggagtgggtacgctgcagacaaagaatctactcaaaaggcaatagacggagtcaccaataaggtcaactcgatcattgacaaaatgaacactcagtttgaggccgtaggaagggagtttaataacttagagaggagaatagagaatttgaacaagaagatggaagacggattcctagatgtctggacttataatgctgaacttctggttctcatggaaaatgagagaactctagacttccatgactcaaatgtcaggaacctttacgacaaggtcagactacagcttaaggacaatgcaaaagaattgggtaacggttgtttcgagttctatcacaaatgtaataatgaatgtatggaaagtgtaagaaacggaacgtatgactacccgcagtattcagaagaagcaagattaaaaagagaagaaataagtggagtaaagttggaatcaataggaatttaccaaatattgtcaatttattcaacagtagcgagttccctagtgctggcaatcatgatggctggtctatctttatggatgtgttccaacgggtcgttacagtgcagaatttgcatttga

>H5N1_A/chicken/Bhutan/413/2012

cttgttaaaagcgatcatatttgcattggttatcatgcaaataactcgacaaagcaggttgacacaataatggaaaagaacgttactgttacacatgcccaagacatactggaaaagacacacaacgggaagctctgcgatctaaatggagtgaaacctctgattttaaaagattgtagtgtagcaggatggctcctcggaaacccaatgtgtgacgaattcatcaatgtgccagaatggtcttacatagtagagaaggccaatccagccaatggcctctgttacccagggaatttcaacgattatgaagaattgaaacacctattgagcaggataaaccattttgagaaaatacggatcatccccaaagattcttggtcagatcatgaagcttcattgggggtgagtgcagcatgtccataccagggaaattcctccttcttcagaaatgtggtatggcttatcaaaagggacaatgcatacccaacaataaagaaaagctacaacaataccaaccaagaagatcttttggtactgtgggggattcaccatcctaacgatgaagcagagcagacaatgctctatcaaaacccaaccacctatatttccattgggacatcaacactaaaccagagattggtgccaaaaatagccactagatccaaaataaacgggcaaagcggcaggatagatttcttctggacaattttaaaaccgaatgatgcaatccacttcgagagtaacggaaatttcattgctccagagtatgcatacaaaattgtcaagaaaggagactcaacaattatgaaaagtgaagtggaatatggtaactgcaacaccaggtgtcagactcctataggggcgataaactctagtatgccattccacaacatacatcccctcaccatcggagaatgtcccaaatatgtgaaatcaaacaaattagtccttgcgactgggctcagaaatagtcctcaaagagagagaagaagaaaaGGGagaggattgtttggagctatagcagggtttatagagggaggatggcaaggaatggtagatggttggtatgggtaccaccacagcaacgagcaggggagtgggtacgctgcagacaaagaatctactcaaaaggcaatagacggagtcaccaataaggtcaactcgatcattgacaaaatgaacactcagtttgaggccgtaggaagggagtttaataacttagaaaggagaatagagaatttgaacaagaagatggaagacggattcctagatgtctggacttataatgctgaacttctggttctcatggaaaatgagagaactctagacttccatgactcaaatgtcaggaacctttacgacaaggtcagactacagcttaaggacaatgcaaaagaattgggtaatggttgtttcgagttctatcacaaatgtaataatgaatgtatggaaagtgtaagaaacggaacgtatgactacccgcagtattcagaagaagcaagattaaaaagagaagaaataagtggagtaaagttggaatcaataggaatttaccaaatattgtcaatttattcaacagtagcgagttccctagtactggcaatcatgatggctggtctatctttatggatgtgttccaacgggtcgttacagtgcagaatttgcatttga

>H5N1_A/chicken/Bhutan/415/2012

cttgttaaaagcgatcatatttgcattggttaccatgcaaataactcgacaaagcaggttgacacaataatggaaaagaacgttactgttacacatgcccaagacatactggaaaagacacacaacgggaagctctgcgatctaaatggagtgaaacctctgattttaaaagattgtagtgtagcaggatggctcctcggaaacccaatgtgtgacgaattcatcaacgtgcccgaatggtcttacatagtagagaaggccaatccagccaatggcctctgttacccagggaatttcaacgattatgaagaattgaaacacctattgagcaggataaaccattttgagaaaatacggatcatccccaaagattcttggtcagatcatgaaacctcattgggggtgagtgcagcatgtccataccagggaaattcctccttcttcagaaatgtggtatggcttatcaaaaaggacaatgcatacccaacaataaagaaaagctacaacaataccaaccaagaagatcttttggtactgtgggggattcaccatcctaacgatgaagcagagcagacaaggctctatcaaaacccaaccacctatatttccattgggacatcaacactaaaccagagattggtgccaaaaatagccactagatccaaaataaacgggcaaagcggcaggatagatttcttctggacaattttaaaaccgaatgatgcaatccacttcgagagtaacggaaatttcattgctccagagtatgcatacaaaattgtcaagaaaggagactcaacaattatgaaaagtgaagtggaatatggtaactgcaacaccaggtgtcagactcctataggggcgataaactctagtatgccattccacaacatacatcccctcaccatcggagaatgtcccaaatatgtgaaatcaaacaaattagtccttgcgactgggctcagaaatagtcctcaaagagagagaagaagaaaaGGGagaggattgtttggagctatagcagggtttatagagggaggatggcaaggaatggtagatggttggtatgggtaccaccacagcaacgagcaggggagtgggtacgctgcagacaaagaatctactcaaaaggcaatagacggagtcaccaataaggtcaactcgatcattgacaaaatgaacactcagtttgaggccgtaggaagggagtttaataacttagaaaggagaatagagaatttgaacaagaagatggaagacggattcctagatgtctggacttataatgctgaacttctggttctcatggaaaatgagagaactctagacttccatgactcaaatgtcaggaacctttacgacaaggtcagactacagcttaaggacaatgcaaaagaattgggtaatggttgtttcgagttctatcacaaatgtaataatgaatgtatggaaagtgtaagaaacggaacgtatgactacccgcagtattcagaagaagcaagattaaaaagagaagaaataagtggagtaaagttggaatcaataggaatttaccaaatattgtcaatttattcaacagtagcgagttccctagtactggcaatcatgatggctggtctatctttatggatgtgttccaacgggtcgttacagtgcagaatttgcatttga

>H5N1_A/chicken/Bhutan/505/2012

cttgttaaaagcgatcatatttgcattggttatcatgcaaataactcgacaaagcaggttgacacaataatggaaaaaaacgttactgttacacatgcccaagacatactggaaaagacacacaacgggaagctctgcgatctaaatggagtgaaacctctgattttaaaagattgtagtgtagcaggatggctcctcggaaacccaatgtgtgacgaattcatcaatgtgccagaatggtcttacatagtagagaaggccaatccagccaatggcctctgttacccagggaatttcaacgattatgaagaattgaaacacctattgagcaggataaaccattttgagaaaatacggatcatccccaaagattcttggtcagatcatgaagccttcttgggggtgagtgcagcatgtccataccagggaaattcctccttcttcagaaatgtggtatggcttatcaaaaaggacaatgcatacccaacaataaagaaaagctacaacaataccaaccaagaagatcttttagtactgtgggggattcaccatcctaacgatgaagcagagcagacaatgctctatcaaaacccaaccacctatatttccattgggacatcaacactaaaccagagattggtgccaaaaatagccactagatccaaaataaacgggcaaagcggcaggatagatttcttctggacaattttaaaaccgaatgatgcaatccacttcgagagtaacggaaatttcattgctccagagtatgcatacaaaattgtcaagaaaggagactcaacaattatgaaaagtgaagtggaatatggtaactgcaacaccaggtgtcagactcctataggggcgataaactctagtatgccattccacaacatacatcccctcaccatcggagaatgtcccaaatatgtgaaatcaaacaagttagtccttgcgactgggctcagaaatagtcctcaaagagagagaagaagaaaaGGGagaggattgtttggagctatagcagggtttatagagggaggatggcagggaatggtagatggttggtatgggtaccaccacagcaacgagcaggggagtgggtacgctgcagacaaagaatctactcaaaaggcaatagacggagtcaccaataaggtcaactcgatcattgacaaaatgaacactcagtttgaggccataggaagggagtttaataacttagagaggagaatagagaatttgaacaagaagatggaagacggattcctagatgtctggacttataatgctgaacttctggttctcatggaaaatgagagaactctagacttccatgactcaaatgtcaggaacctttacgacaaggtcagactacagcttaaggacaatgcaaaagaattgggtaacggttgtttcgagttctatcacaaatgtaataatgaatgtatggaaagtgtaagaaacggaacgtatgactacccgcagtattcagaagaagcaagattaaaaagagaagaaataagtggagtaaagttggaatcaataggaatttaccaaatattgtcaatttattcaacagtagcgagttccctagtactggcaatcatgatggctggtctatctttatggatgtgttccaacgggtcgttacagtgcagaatttgcatttga

>H5N1_A/chicken/Bhutan/507/2012

cttgttaaaagcgatcatatttgcattggttatcatgcaaataactcgacaaagcaggttgacacaataatggaaaagaacgttactgttacacatgcccaagacatactggaaaagacacacaacgggaagctctgcgatctaaatggagtgaaacctctgattttaaaagattgtagtgtagcaggatggctcctcggaaacccaatgtgtgacgaattcatcaatgtgccagaatggtcttacatagtagagaaggccaatccagccaatggcctctgttacccagggaatttcaacgattatgaagaattgaaacacctattgagcaggataaaccattttgagaaaatacggatcatccccaaagattcttggtcagatcatgaagcctctttgggggtgagtgcagcatgtccataccagggaaattcctccttcttcagaaatgtggtatggcttatcaaaaaggacaatgcatacccaacaataaagaaaagctacaacaataccaaccaagaagatcttttggtactgtgggggattcaccatcctaacgatgaagcagagcagacaatgctctatcaaaacccaaccacctatatttccattgggacatcaacactaaaccagagattggtgccaaaaatagccactagatccaaaataaacgggcaaagcggcaggatagatttcttctggacaattttaaaaccgaatgatgcaatccacttcgagagtaacggaaatttcattgctccagagtatgcatacaaaattgtcaagaaaggagactcaacaattatgaaaagtgaagtggaatatggtaactgcaacaccaggtgtcagactcctataggggcgataaactctagtatgccattccacaacatacatcccctcaccatcggagaatgtcccaaatatgtgaaatcaaacaaattagtccttgcgactggactcagaaatagtcctcaaagagagagaagaagaaaaGGGagaggattgtttggagctatagcagggtttatagagggaggatggcagggaatggtagatggttggtatgggtaccaccacagcaacgagcaggggagtgggtacgctgcagacaaagaatctactcaaaaggcaatagacggagtcaccaataaggtcaactcgatcattgacaaaatgaacactcagtttgaggccataggaagggagtttaataacttagagaggagaatagagaatttgaacaagaagatggaagacggattcctagatgtctggacttataatgctgaacttctggttctcatggaaaatgagagaactctagacttccatgactcaaatgtcaggaacctttacgacaaggtcagactacagcttaaggacaatgcaaaagaattgggtaacggttgtttcgagttctatcacaaatgtaataatgaatgtatggaaagtgtaagaaacggaacgtatgactacccgcagtattcagaagaagcaagattaaaaagagaagaaataagtggagtaaagttggaatcaataggaatttaccaaatattgtcaatttattcaacagtagcgagttccctagtactggcaatcatgatggctggtctatctttatggatgtgttccaacgggtcgttacagtgcagaatttgcatttga

>H5N1_A/chicken/Bhutan/933/2012

cttgttaagagcgatcatatttgcattggttatcatgcaaataactcgacaaagcaggttgacacaataatggaaaagaacgttactgttacacatgcccaagacatactggaaaagacacacaacgggaagctctgcgatctaaatggagtgaaacctctgattttaaaagattgtagtgtagcaggatggctcctcggaaacccaatgtgtgatgaattcatcaatgtgccagaatggtcttacatagtagagaaggccaatccagccaatggcctctgttacccagggaatttcaacgattatgaagaattgaaacacctattgagcaggataaaccattttgagaaaatacggatcatccccaaagattcttggtcagatcatgaagccttcttgggggtgagtgcagcatgcccataccagggaaattcctctttcttcagaaatgtggtatggcttatcaaaaaggacaatgcatacccaacaataaagaaaagctacaacaataccaaccaagaagatcttttggtactgtgggggattcaccatcctaacgatgaagcagagcagacaatgctctatcaaaacccaaccacctatatttccattgggacatcaacactaaatcagagattggtgccaaaaatagccactagatccaaaataaacgggcaaagcggcaggatagatttcttctggacaattttaaaaccgaatgatgcaatccacttcgagagtaacggaaatttcattgctccagagtatgcatacaaaattgtcaagaaaggagactcaacaattatgaaaagtgaagtggaatatggtaactgcaacaccaggtgtcagactcctataggggcgataaactctagtatgccattccacaacatacatcccctcaccatcggagaatgtcccaaatatgtgaaatcaaacaaattagtccttgcgactgggctcagaaatagtcctcaaagagagagaagaagaaaaGGGagaggattgtttggagctatagcagggtttatagagggaggatggcagggaatggtagatggttggtatgggtaccaccacagcaacgagcaggggagtgggtacgctgcagacaaagaatctactcaaaaggcaatagacggagtcaccaataaggtcaactcgatcattgacaaaatgaacactcagtttgaggccgtaggaagggagtttaataacttagagaggagaatagagaatttgaacaagaagatggaagacggattcctagatgtctggacttataatgctgaacttctggttctcatggaaaatgagagaactctagacttccatgactcaaatgtcaggaacctttacgacaaggtcagactacagcttaaggacaatgcaaaagaattgggcaacggctgtttcgagttctatcacaaatgtaataatgagtgtatggaaagtgtaagaaacggaacgtatgactacccgcagtattcagaagaagcaagattaaaaagagaagaaataagtggagtaaagttggaatcaataggaatttaccaaatattgtcaatttattcaacagtagcgagttccctagtactggcaatcatgatggctggtctatctttatggatgtgttccaacgggtcgttacagtgcagaatttgcatttga

>H5N1_A/chicken/Bhutan/934/2012

cttgttaagagcgatcatatttgcattggttatcatgcaaataactcgacaaagcaggttgacacaataatggaaaagaacgttactgttacacatgcccaagacatactggaaaagacacacaacgggaagctctgcgatctaaatggagtgaaacctctgattttaaaagattgtagtgtagcaggatggctcctcggaaacccaatgtgtgatgaattcatcaatgtgccagaatggtcttacatagtagagaaggccaatccagccaatggcctctgttacccagggaatttcaacgattatgaagaattgaaacacctattgagcaggataaaccattttgagaaaatacggatcatccccaaagattcttggtcagatcatgaagccttcttgggggtgagtgcagcatgcccataccagggaaattcctctttcttcagaaatgtggtatggcttatcaaaaaggacaatgcatacccaacaataaagaaaagctacaacaataccaaccaagaagatcttttggtactgtgggggattcaccatcctaacgatgaagcagagcagacaatgctctatcaaaacccaaccacctatatttccattgggacatctacactaaatcagagattggtgccaaaaatagccactagatccaaaataaacgggcaaagcggcaggatagatttcttctggacaattttaaaaccgaatgatgcaatccacttcgagagtaacggaaatttcattgctccagagtatgcatacaaaattgtcaagaaaggagactcaacaattatgaaaagtgaagtggaatatggtaactgcaacaccaggtgtcagactcctataggggcgataaactctagtatgccattccacaacatacatcccctcaccatcggagaatgtcccaaatatgtgaaatcaaacaaattagtccttgcgactgggctcagaaatagtcctcaaagagagagaagaagaaaaGGGagaggattgtttggagctatagcagggtttatagagggaggatggcagggaatggtagatggttggtatgggtaccaccacagcaacgagcaggggagtgggtacgctgcagacaaagaatctactcaaaaggcaatagacggagtcaccaataaggtcaactcgatcattgacaaaatgaacactcagtttgaggccgtaggaagggagtttaataacttagagaggagaatagagaatttgaacaagaagatggaagacggattcctagatgtctggacttataatgctgaacttctggttctcatggaaaatgagagaactctagacttccatgactcaaatgtcaggaacctttacgacaaggtcagactacagcttaaggacaatgcaaaagaattgggcaacggctgtttcgagttctatcacaaatgtaataatgagtgtatggaaagtgtaagaaacggaacgtatgactacccgcagtattcagaagaagcaagattaaaaagagaagaaataagtggagtaaagttggaatcaataggaatttaccaaatattgtcaatttattcaacagtagcgagttccctagtactggcaatcatgatggctggtctatctttatggatgtgttccaacgggtcgttacagtgcagaatttgcatttga

>H5N1_A/chicken/Burkina Faso/15VIR1774-2/2015

cttgtcaaaagcgatcatatttgcattggttatcatgcaaataactcgacagagcaggttgacacaataatggaaaagaacgttactgttacacatgcccaagacatactggaaaagacacacaacgggaagctctgcgatctaaatggagtgaagcctctgattttaaaagattgtagtgtagcaggatggctcctcggaaatccattgtgtggcgaattcaccaatgtgccagaatggtcttacatagtagagaaggccaatccagccaatgacctctgttatccagggaatttcaacgattatgaggaactaaaacacctattgagcaggataaaccattttgagaaaatacagatcatccccaaagattcttggtcagatcatgaagcctcattgggggtgagcgcagcatgttcatatcagggaaattcctccttcttcagaaatgtggtgtggcttatcaaaaaggacaatgcatacccaacaataaagaaaggctacaataataccaaccgagaagatctcttgatactgtgggggatccaccatcctaatgatgaagcagagcagacaaagctctatcaaaacccaactacctatatttccattgggacttcaacactaaaccagagattggtaccaaaaatagccactagatccaaaataaacgggcaaagtggcaggatagatttcttctggacaattttaaaaccgaatgacgcaatccatttcgagagtaatggaaatttcattgctccagaatatgcatacaaaattgtcaagaaaggagactccacaatcatgagaagtgaggtggaatatggtaactgcaacaccaggtgtcagactccaataggggcgataaactctagcatgccattccacaacatacaccctctcactatcggagaatgtcccaaatatgtgaaatcaaacaaattagtccttgcaactgggctcagaaatagtcctcaaagagagagaagaagaaaaGGGagaggactgtttggagctatagcaggttttatagagggaggatggcagggaatggtagatggttggtatgggtaccaccacagcaatgaacaaggaagtggttacgctgcagacaaagaatctactcaaaaggcgatagacggagtcaccaataaggtcaattcaatcattgacaaaatgaacactcagtttgaggctgtaggaaaagaatttaataacttagagaggagaatagaaaatttaaacaagaagatggaagacggattcctagatgtctggacttataatgctgaacttctggttctcatggagaatgagagaactctagacttccatgactcaaatgtcaagaacctttacgataaggtccgactacagctcaaggataatgcaaaagagctgggaaacggttgtttcgagttctatcacaaatgtaataatgaatgtatggaaagtgtaagaaacgggacatatgactacccgcagtattcagaagaagcaagattaaaaagagaggaaataagtggagtaaaactggaatcaataggagtctaccaaatactgtcaatttattcaacagtggcgagttccctagtgctggcaatcatgatggctggtctatctttatggatgtgttccaacgggtcgttacagtgcagaatttgcatttga

>H5N1_A/chicken/Burkina Faso/15VIR1774-33/2015

cttgtcaaaagcgatcatatttgcattggttatcatgcaaataactcgacagagcaggttgacacaataatggaaaagaacgttactgttacacatgcccaaaacatactggaaaagacacacaacgggaagctctgcgatctaaatggagtgaagcctctgattttaaaagattgtagtgtagcaggatggctcctcggaaatccattgtgtggcgaattcaccaatgtgccagaatggtcttacatagtagagaaggccaatccagccaatgacctctgttatccagggaatttcaacgattatgaggaactaaaacacctattgagcaggataaaccattttgagaaaatacagatcatccccaaagattcttggtcagatcatgaagcctcattgggggtgagcgcagcatgttcatatcagggaaattcctccttcttcagaaatgtggtgtggcttatcaaaaaggacaatgcatacccaacaataaagaaaggctacaataataccaaccgagaagatctcttgatactgtgggggatccaccatcctaatgatgaagcagagcagacaaagctctatcaaaacccaactacctatatttccattgggacttcaacactaaaccagagattggtaccaaaaatagccactagatccaaaataaacgggcaaagtggcaggatagatttcttctggacaattttaaaaccgaatgacgcaatccatttcgagagtaatggaaatttcattgctccagaatatgcatacaaaattgtcaagaaaggagactccacaatcatgagaagtgaggtggaatatggtaactgcaacaccaggtgtcagactccaataggggcgataaactctagcatgccattccacaacatacaccctctcactatcggagaatgtcccaaatatgtgaaatcaaacaaattagtccttgcaactgggctcagaaatagtcctcaaagagagagaagaagaaaaGGGagaggactgtttggagctatagcaggttttatagagggaggatggcagggtatggtagatggttggtatgggtaccaccacagcaatgaacaaggaagtggttacgctgcagacaaagaatctactcaaaaggcgatagacggagttaccaataaggtcaattcaatcattgacaaaatgaacactcagtttgaggctgtaggaaaagaatttaataacttagagaggagaatagaaaatttaaacaagaagatggaagacggattcctagacgtctggacttataatgctgaacttctggttctcatggagaatgagagaactctagacttccatgactcaaatgtcaagaacctttacgataaggtccgactacagctcaaggataatgcaaaagagctgggaaacggttgtttcgagttctatcacaaatgtaataatgaatgtatggaaagtgtacgaaacgggacgtatgactacccacagtattcagaagaagcaagattaaaaagagaggaaataagtggagtaaaactggaatcaataggagtctaccaaatactgtcaatttattcaacagtggcgagttccctagtgctggcaatcatgatggctggtctatctttatggatgtgttccaacgggtcgttacagtgcagaatttgcatttga

>H5N1_A/chicken/Burkina Faso/15VIR1774-37/2015

cttgtcaaaagcgatcatatttgcattggttatcatgcaaataactcgacagagcaggttgacacaataatggaaaagaacgttactgttacacatgcccaagacatactggaaaagacacacaacgggaagctctgcgatctaaatggagtgaagcctctgattttaaaagattgtagtgtagcaggatggctcctcggaaatccattgtgtgccgaattcaccaatgtgccagaatggtcttacatagtagagaaggccaatccagccaatgacctctgttatccagggaatttcaacgattatgaggaactaaaacacctattgagcaggataaaccattttgagaaaatacagatcatccccaaagattcttggtcagatcatgaagcttcattgggggtgagcgcagcatgttcatatcagggaaattcctccttcttcagaaatgtggtgtggcttatcaaaaaggacaatgcatacccaacaataaagaaaggctacaataataccaaccgagaagatctcttgatactgtgggggatccaccatcctaatgatgaagcagagcagacaaagctctatcaaaacccaactacctatatttccattgggacttcaacactaaaccagagattggtaccaaaaatagccactagatccaaaataaacgggcaaagtggcaggatagatttcttctggacaattttaaaaccgaatgacgcaatccatttcgagagtaatggaaatttcattgctccagaatatgcatacaaaattgtcaagaaaggagactccacaatcatgagaagtgaggtggaatatggtaactgcaacaccaggtgtcagactccaataggggcgataaactctagcatgccattccacaacatacaccctctcactatcggagaatgtcccaaatatgtgaaatcaaacaaattagtccttgcaactgggctcagaaatagtcctcaaagagagagaagaagaaaaGGGagaggactgtttggagctatagcaggttttatagagggaggatggcagggaatggtagatggttggtatgggtaccaccacagcaatgaacaaggaagtggttacgctgcagacaaagaatctactcaaaaggcgatagacggagtcaccaataaggtcaattcaatcattgacaaaatgaacactcagtttgaggctgtagggaaagaatttaataacttagagaggagaatagaaaatttaaacaagaagatggaagacggattcctagatgtctggacttataatgctgaacttctggttctcatggagaatgagagaactctagacttccacgactcaaatgtcaagaacctttacgataaggtccgactacagctcaaggataatgcaaaagagctgggaaacggttgtttcgagttctatcacaaatgtaataatgaatgtatggaaagtgtaagaaacgggacgtatgactacccgcagtattcagaagaagcaagattaaaaagagaggaaataagtggagtaaaactggaatcaataggagtctaccaaatactgtcaatttattcaacagtggcgagttccctagtgctggcaatcatgatggctggtctatctttatggatgtgttccaacgggtcgttacagtgcagaatttgcatttga

>H5N1_A/chicken/Burkina Faso/15VIR1774-4/2015

cttgtcaaaagcgatcatatttgcattggttatcatgcaaataactcgacagagcaggttgacacaataatggaaaagaacgttactgttacacatgcccaagacatactggaaaagacacacaacgggaagctctgcgatctaaatggagtgaagcctctgattttaaaagattgtagtgtagcaggatggctcctcggaaatccattgtgtggcgaattcaccaatgtgccagaatggtcttacatagtagagaaggccaatccagccaatgacctctgttatccagggaatttcaacgattatgaggaactaaaacacctattgagcaggataaaccattttgagaaaatacagatcatccccaaagattcttggtcagatcatgaagcctcattgggggtgagcgcagcatgttcatatcagggaaattcctccttcttcagaaatgtggtgtggcttatcaaaaaggacaatgcatacccaacaataaagaaaggctacaataataccaaccgagaagatctcttgatactgtgggggatccaccatcctaatgatgaagcagagcagacaaagctctatcaaaacccaactacctatatttccattgggacttcaacactaaaccagagattggtaccaaaaatagccactagatccaaaataaacgggcaaagtggcaggatagatttcttctggacaattttaaaaccgaatgacgcaatccatttcgagagtaatggaaatttcattgctccagaatatgcatacaaaattgtcaagaaaggagactccacaatcatgagaagtgaggtggaatatggtaactgcaacaccaggtgtcagactccaataggggcgataaactctagcatgccattccacaacatacaccctctcactatcggagaatgtcccaaatatgtgaaatcaaacaaattagtccttgcaactgggctcagaaatagtcctcaaagagagagaagaagaaaaGGGagaggactgtttggagctatagcaggttttatagagggaggatggcagggaatggtagatggttggtatgggtaccaccacagcaatgaacaaggaagtggttacgctgcagacaaagaatctactcaaaaggcgatagacggagtcaccaataaggtcaattcaatcattgacaaaatgaacactcagtttgaggctgtaggaaaagaatttaataacttagagaggagaatagaaaatttaaacaagaagatggaagacggattcctagatgtctggacttataatgctgaacttctggttctcatggagaatgagagaactctagacttccatgactcaaatgtcaagaacctttacgataaggtccgactacagctcaaggataatgcaaaagagctgggaaacggttgtttcgagttctatcacaaatgtaataatgaatgtatggaaagtgtaagaaacgggacgtatgactacccgcagtattcagaagaagcaagattaaaaagagaggaaataagtggagtaaaactggaatcaataggagtctaccaaatactgtcaatttattcaacagtggcgagttccctagtgctggcaatcatgatggctggtctatctttatggatgtgttccaacgggtcgttacagtgcagaatttgcatttga

>H5N1_A/chicken/Ca Mau/1180/2006

cttgttaaaagtgatcagatttgcattggttaccatgcaaacaactcgacagagcaggttgacacaataatggaaaagaacgttactgttacacatgcccaagacatactggaaaagacacataacgggaagctctgtgatctagatggagtgaagcctctaattttgagagattgtagtgtagctggatggcttctcggaaacccaatgtgtgacgagttcatcaatgtgccggaatggtcttacatagtggagaaggccaatccagtcaatgacctctgttacccaggagttttcaatgactatgaagaattgaaacacctattgagcagaataaaccattttgagaaaattcagatcatccccaaaagttcttggcccagtcatgaagcctcattgggggtgagcgcagcatgtccataccagggaaagtcctcttttttcagaaatgtggtatggcttatcaaaaagaacagtacatacccaacaataaagaggagctacaataataccaaccaagaagatcttttggtaatgtgggggatccaccatcctaatgatgcggcagagcagacaaagctctatcaaaatccaaccacctatatctccgttgggacatcaacactaaaccagagattgacaccaagaatagctactagatccaaagtaaacgggcaaagtgggaggatggagttcttctggacaattttaaaaccaaatgatgcaatcaacttcgagagtaatggaaatttcattgctccagaatatgcatacaaaattgtcaagaaaggggactcaacaattatgaaaagtgaattggaatatggtaactgcaacaccaagtgtcaaactccaatgggggcgataaactctagtatgccattccacaatatacatcctctcactattggggaatgccccaaatatgtgaaatcaaacagattagtccttgcgactgggctcagaaatagccctcaaagagagggaagaagaaaaaagagaggattatttggagctatagcaggttttatagagggaggatggcagggaatggtagatggttggtatgggtaccaccatagcaatgagcaggggagtgggtacgctgcagacaaagaatccactcaaaaggctatagatggagtcaccaataaggtcaactcgatcattgacaaaatgaacactcagtttgaggccgttggaagggaatttaacaacttagaaagaagaatagagaatttaaacaagaagatggaagacgggttcctagatgtctggacttataatgctgaacttctggttctcatggaaaatgagagaactctagacttccatgactcaaatgtaaagaacctttacgacaaggtccgattacagctcagggataatgcaaaggagctgggtaacggttgtttcgagttctatcacaaatgtgataatgaatgtatggaaagtgtgagaaacgggacgtatgactacccgcagtattcagaagaagcaagattaaaaagagaggaaataagtggagtgaaattggaatcgataggaatttaccaaatactgtcaatttattctacagtggcgagttccctagcactggcaatcatggtagctggtctatccttatggatgtgctccaatgggtcgttacaatgcagaatttgcatttga

>H5N1_A/chicken/Cambodia/083LC/2011

cttgttaaaagtgaccagatttgcattggctaccatgcaaacaactcgacaaagcaggttgacacgataatggaaaagaacgttactgttacacatgcccaagacatactagaaaagacacacaacggaaagctctgtgacttagatggagttaggcctctaattttgagagattgtagtgtagctggatggcttctcggaaacccaatgtgtgacgaattcatcaatgtaccagaatggtcttatatagtggagaaggccaatccaatcaatgacctctgttacccaggagttttcaatgactatgaagaattgaaacacctattgagcagaataaaccattttgagaaaattcagatcatccccaagagttcttggcctagtcatgaagcctcattgggggtgagcgcagcatgtccatacctgggaaagtcctcttttttccgaaatgtggtgtggcttatcaaaaagaacagtacatacccaacaataaagaggagttacaataataccaaccaagaagatcttttggtaatgtgggggatccaccatcctaatgatgcagcagaacagacaaaactctatcaaaatccaaccacctatatctccgttgggacgtcaacactaaaccagagattgacaccaagaatagctactagatccaaagtaaacggacaaagtgggaggatggagttcttttggacaatcttaaaaccgaatgatgcaatcaacttcgagagtaatggaaatttcattgctccagaatatgcatacaaaattgtcaagaaaggggactcagcaattatgagaagtgaattggaatatggtaactgcaacaccaagtgtcaaactccaatgggggcaataaactctagtatgccattccacaatatacatcctctcactattggagaatgccccaaatatgtgaaatcaaccagattagtccttgcaactgggctcagaaatagccctcaaagagaggaaagaagaaaaaagagaggattatttggagctatagcaggatttatagaaggaggatggcaggggatggtagatggttggtatgggtaccatcatagcaatgagcagggaagcgggtacgctgcagacaaagaatccactcaaaaggctatagatggagtcaccaataaggtcaactcgatcattgacaaaatgaacactcagtttgaggccgttggaagggaatttaacaacttagaaagaagaatagagaatttaaacaagaagatggaagacgggttcctagatgtctggacttataatgctgaacttctggttctcatggaaaatgagagaactctagacttccatgactcaaatgtaaagaacctttacgacaaggtccgattacagcttagggataatgcaaaggagctgggtaacggttgttttgagttctatcacaaatgtgataatgaatgcatggaaagtgtgagaaacgggacatatgactacccgcattattcagaagaagcaagattaaaaagagaggagataagtggagtgaaattggaatcgataggagtttaccaaatactgtcaatttattctacagtggcgagttccctagcactggcaatcatggtagctggtctatccttatggatgtgctccaatgggtcgttacaatgcagaatttgcatttga

>H5N1_A/chicken/Cambodia/TKCMB5T/2010

cttgttaaaagtgatcagatttgcattggttatcatgcaaacaactcgacaaagcaggttgacacgataatggaaaagaacgttactgttacacatgcccaagacatactagaaaagacacataacggaaagctctgtgacctagatggagttaggcctctaattttgagagattgtagtgtagctggatggcttctcggaaacccaatgtgtgacgaattcatcaatgtgccagaatggtcttatatagtggagaaggccaatccagtcaatgacctctgttacccaggagttttcaatgactatgaagaattgaaacacctattgagcagaataaaccattttgagaaaattcagatcatccccaagagttcttggcccagtcatgaagcctcattgggggtgagcgcagcatgtccataccagggaaagtcctcttttttccgaaatgtggtgtggcttatcaaaaagaacagtacatacccaacaataaagaggagttacaataataccaaccaagaagatcttttggtaatgtgggggatccaccatcctaatgatgcagcagagcagacaaagctctatcaaaatccaaccacctatatctccgttgggacgtcaacgctaaaccagagattgacaccaagaatagctactagatccaaagtaaacgggcaaagtgggaggatggagttcttttggacaatcttaaaaccgaatgatgcaatcaacttcgagagtaatggaaatttcattgctccagaatatgcatacaaaattgtcaagaaaggggactcaacaattatgaaaagtgaattggaatatggtaactgcaacaccaagtgtcaaactccaatgggggcgataaactctagtatgccattccacaatatacatcctctcactattggagaatgccccaaatatgtgaaatcaaccagattagtccttgcgactgggctcagaaatagccctcaaagagagggaagaagaaaaaagagaggattatttggagctatagcagggtttatagagggaggatggcaggggatggtagatggttggtatgggtaccatcatagcaatgagcagggaagcgggtacgctgcagacaaagaatccactcaaaaggctatagatggagtcaccaataaggtcaactcgatcattgacaaaatgaacactcagtttgaggccgttggaagggaatttaacaacttagaaagaagaatagagaatttaaacaagaagatggaagacgggttcctagatgtctggacttataatgctgaacttctggttctcatggaaaatgagagaactctagacttccatgactcaaatgtaaagaacctttacgacaaggtccgattacagcttagggataatgcaaaggagctgggtaacggttgtttcgagttctatcacaaatgtgataatgaatgcatggaaagtgtgagaaacgggacgtatgactacccgcagtattcagaagaagcaagattaaaaagagaggagataagtggagtgaaattggaatcgataggagtttaccaaatactgtcaatttattctacagtggcgagttccctagcactggcaatcatggtagctggtctatccttatggatgtgctccaatgggtcgttacaatgcagaatctgcatttga

>H5N1_A/chicken/Cambodia/X0815301/2013

cttgttaaaagtgaccagatttgcattggctaccatgcaaacaactcgacagagcaggttgacacgataatggaaaagaacgttactgttacacatgcccaagacatactagaaaagacacataacggaaagctctgtgacttagatggagttaggcctctaattttgagagattgtagtgtagctggatggcttctcggaaacccaatgtgtgacgaattcatcaatgtgccagaatggtcttatatagtggagaaggccaatccagtcaatgacctctgctacccaggagttttcaatgactatgaagaattgaaacacctattgagcagaataaaccattttgagaaaattcagatcatccccaagaattcttggcccagtcatgaagcctcattgggggtgagcgcagcatgtccataccaggggcagtcctcttttttccgaaatgtggtgtggcttatcaaaaagaacaatacatacccaacaataaagaggagttacaataataccaaccaagaagatcttttggtaatgtgggggatccaccatcctaatgatgcagtagaacagacaaaactctatcaaaatccaaccacctatatctccgttgggacgtcaacattaaaccagagattgacaccaagaatagctactagatccaaagtaaacggacaaagtgggaggatggagttcttttggacaatcttaaaaccgaatgatgcaatcaacttcgagagtaatggaaatttcatcgctccagaatatgcatacaaaattgtcaagaaaggggactcaacaattatgagaagtgaactggaatatggtaactgcaacaccaagtgtcaaactccaatgggggcgataaactctagtatgccattccacaatatacatcctctcactattggagaatgccccaaatatgtgaaatcaaccagattagtccttgcgactgggctcagaaatagccctcaaagagaggaaagaagaaaaaagagaggattatttggagctatagcagggtttatagaaggaggatggcaggggatggtagatggttggtatgggtaccatcatagcaacgagcagggaagcgggtacgctgcagacaaagaatccactcaaaaggctatagatggagtcaccaataaggtcaactcgatcattgacaaaatgaacactcagtttgaggctgttgggagggaatttaacaacttagaaagaagaatagagaatttaaacaagaagatggaagacgggttcctagatgtttggacttataatgctgaacttctggttctcatggaaaatgagagaactctagacttccatgactcaaatgtaaagaacctttacgacaaggtccgattacagcttagggataatgccaaggagctgggtaacggttgttttgagttctatcacaaatgtgataatgaatgtatggaaagtgtgagaaatgggacatatgactatccgcagtattcagaagaagcaagattaaaaagagaggagataagtggagtgaaattggaatcgataggagtttaccaaatactgtcaatttattctacagtggcgagttccctagcactggcaatcatggtagctggtctatccttatggatgtgctccaatgggtcgttacaatgcagaatttgcatttga

>H5N1_A/chicken/Cambodia/Z207W17M1/2015

cttgtcaaaagcgatcatatttgcattggttatcatgcaaataactcgacagagcaggttgacacaataatggaaaagaacgttactgttacacatgcccaagacatactggaaaagacacacaacgggaagctctgcgatctaaatggagtgaagcctctgattttaaaagattgtagtgtagcaggatggctcctcggaaatccattgtgtgacgaattcaccaatgtgccagaatggtcctacatagtagagaaggccagtccagccaatgacctctgttacccagggaatttcaacgattatgaagaattgaagcacctattgagcaggataaaccattttgagaaaatacagatcatccccataaattcttggtcagatcatgaagcctcattgggggtgagcgcagcatgttcataccagggaaattcctccttcttcagaaatgtggtgtggcttatcaaaaagaacaatgcatacccaacaataaagaaaggttacaataacaccaaccgagaagatctcttgatactgtgggggatccaccatcctaatgatgaggaggaacagacaaggctctaccaaaacccaactacctatatttccattgggacttcaacactaaaccagagattggtaccaaaaatagccactagatccaaaataaacgggcaaagtggcaggatagatttcttctggacaattttaaaaccgaatgacacaatccatttcgagagtaatggaaatttcattgctccagaatatgcatacaaaattgtcaagaagggagactccacaatcatgagaagtgaagtggaatatggtaactgcaacaccaggtgtcagactccaataggggcgataaactctagtatgccattccacaacatacaccctctcactatcggagaatgtcccaaatatgtgaaatcaaacaaattagtccttgcaactgggctcagaaatagtcctcaaagagagagaagaagaagaGGGagaggactgtttggagctatagcaggttttatagagggaggatggcagggaatggtagatggttggtatgggtaccatcacagcaatgaacaggggagtggctacgctgcagacaaggaatctactcaaaaggcgatagacggagtcaccaataaggtcaattcgatcattgacaaaatgaacactcagtttgaggctgtagggagggaatttaataacttagagaggagaatagaaaatttaaacaagaagatggaagatggattcctggatgtctggacttataatgctgaacttctggttctcatggagaatgagagaacactagacttccatgactcaaatgtaaaaaacctttacgataaggtacgactacagcttaaggataatgcaaaagaactgggaaacggttgtttcgagttctatcacaaatgtaataatgaatgtatggaaagtgttagaaacgggacgtatgactacccgcagtattcagaagaagcaagattaaaaagagaggaaataagtggagtaaaactggaatcaataggaatctaccaaatactgtcaatttattcaacagtggcgagttccctagtgctggcaatcatgatggctggtctatccttatggatgtgttccaacgggtcgttacagtgcagaatttgcatttga

>H5N1_A/chicken/Cambodia/Z850W49M1/2015

cttgtcaaaagcgatcatatttgcattggttatcatgcaaataactcgacagagcaggttgacacaataatggaaaagaacgttactgttacacatgcccaggacatactggaaaagacacacaatgggaagctctgcgatctaaacggagtgaagcctctgattttaaaagattgtagtgtagcaggatggctcctcggaaatccattgtgtgacgaattcaccaatgtgccagaatggtcctacatagtagagaaggccaacccagccaatgacctctgttacccagggaatttcaacgattatgaagaattgaagcacctattgagcaggataaaccattttgagaaaatacagatcatccccaaagattcttggtcaaatcatgaagcctcattgggggtgagcgcagcatgttcataccagggaaattcctccttcttcagaaatgtggtgtggcttatcaaaaagaacaatacatacccaacaataaagaaaggctacaataacatcaaccgagaagatctcttgatactgtgggggatccaccatcctaataatgaggaggaacaaacaaagctctaccaaaacctagatacctatgtttccattgggacttcaacactaaaccagagattggtaccaaaaatagccactagacccaaaataaacgggcaaagtggcaggatagatttcttctggacaattttaaaaccgaatgacacaatccacttcgagagtaatggaaatttcattgctccagaatatgcatacaaaattgtcaagaagggagactccacaatcatgagaagtgaagtggaatatggtaactgcaacaccaggtgtcagactccaataggggcaataaactctagtatgccattccacaacatacaccctctcactatcggagaatgtcccaaatatgtgaaatcaaacaaattagtccttgcaactgggctcagaaatagtcctcaaagagagagaagaagaaaaGGGagaggactgtttggagctatagcaggttttatagagggaggatggcagggaatggtagacggttggtatgggtaccaccacagtaatgaacaggggagtggttacgctgcagacaaggaatctactcaaaaggcgatggacggagtcaccaataaggtcaattcgatcattgacaaaatgaacactcagtttgaggctgtagggagggaatttaataacttagagaggagaatagaaaatttaaacaaaaagatggaagatggattcctagatgtctggacttataatgctgaacttctggttctcatggaaaatgagagaacactagacttccatgactcaaatgtaaaaaacctttacgataaggtccgactacagcttaaggataatgcaaaagaactgggaaacggttgtttcgagttctatcacaaatgtaataatgaatgtatggaaagtgttagaaacgggacgtatgactacccgcagtattcagaagaagcaagattaaaaagagaggaaataagtggagtaaaactggaatcaataggaatctaccaaatactgtcaatttattcaacagtggctagttccctagtgctggcaatcatgatggctggtctatccttatggatgtgttccaacgggtcgttacagtgcagaatttgcatttga

>H5N1_A/chicken/Cameroon/16VIR3791-12/2016

cttgttaaaagcgatcatatttgcattggttatcatgcaaataattcgacagagcaggttgacacaataatggaaaagaacgttactgttacacatgcccaagacatactggaaaagacacacaacggaaagctctgcgatctaaatggagtgaagcctctgattttaaaagactgtagtgtagcaggatggctcctcggaaatccattgtgtggcgaattcaccaatgtgccagaatggtcctacatagtagagaaggccaatccagccaatgatctctgttacccagggaatttcaacgattatgaggaactaaaacatctattgagcaggataaaccattttgagaaaatacagatcatccccaaagattcttggtcagatcatgaagcctcattgggggtgagcgcagcatgttcataccagggaaattcctccttcttcggaaatgtggtgtggcttatcaagaagaacaatgcatacccaacaataaaaaaaggctacaataataccaaccgagaagatctcttgatactgtgggggatccaccatcctaatgatgaagcagagcagacaaagctctatcaaaacccaactacctatatttccgttgggacttcaacactaaaccagagattggtaccaaaaatagccactagatccaaaataaacgggcaaagtggcaggatagatttcttctggacaattttaaaaccgaatgacacaatccatttcgagagtaatggaaatttcattgctccagaatatgcatacaaaattgtcaagaaaggagactccacaatcatgagaagtgaggtagaatatggtaactgcaacaccaggtgtcaaactccaataggggcgataaactctagcatgccattccacaacatacaccctctcactatcggagaatgtcccaaatatgtgaaatcaaacaaattagtccttgcaactgggctcagaaatagtcctcaaagagagagaagaagaaaaGGGagaggactgtttggagctatagcaggttttatagagggaggatggcagggaatggtagatggttggtatgggtaccaccacagcaatgaacaagggagtggttacgctgcagacaaagaatctactcaaaaggcgatagacggagtcaccaataaggtcaattcaatcattgacaaaatgaacactcagtttgaggctgtaggaaaggaatttaataacttagagaggagaatagaaaatttaaacaagaagatggaagacggattcctagatgtctggacttataatgctgaacttctggttctcatggagaatgagagaactctagacttccatgactcaaatgtcaagaacctttacgacaaggtccgactacagcttaaggataatgcaaaagagctgggaaacggttgtttcgagttctatcacaaatgtaataatgaatgtatggaaagtgtgagaaacgggacgtatgactatccgcagtattcagaagaagcaagattaaaaagagaggaaataagtggagtaaaattggaatcaataggaatctaccaaatactgtcaatttattcaacagtggcgagttccctagtgctggcaatcatgatggctggtctatctttatggatgtgttccaacgggtcgttacagtgcagaatttgcatttga

>H5N1_A/chicken/Cameroon/16VIR3791-16/2016

cttgtcaaaagcgatcatatttgcattggttatcatgcaaataattcgacagagcaggttgacacaataatggaaaagaacgttactgttacacatgcccaagacatactggaaaagacacacaacggaaagctctgcgatctaaatggagtgaagcctctgattttaaaagactgtagtgtagcaggatggctcctcggaaatccatggtgtggcgaattcaccaatgtgccagaatggtcctacatagtagagaaggccaatccagccaatgatctctgttacccagggaatttcaacgattatgaggaactaaaacatctattgagcaggataaaccattttgagaaaatacagatcatccccaaagattcttggtcagatcatgaagcctcattgggggtgagcgcagcatgttcataccagggaaattcctccttcttcggaaatgtggtgtggcttatcaagaagaacaatgcatacccaacaataaaaaaaggctacaataataccaaccgagaagatctcttgatactgtgggggatccaccatcctaatgatgaagcagagcagacaaagctctatcaaaacccaactacctatatttccgttgggacttcaacactaaaccagagattggtaccaaaaatagccactagatccaaaataaacgggcaaagtggcagaatagatttcttctggacaattttaaaaccgaatgacacaatccatttcgagagtaatggaaatttcattgctccagaatatgcatacaaaattgtcaagaaaggagactccacaatcatgagaagtgaggtagaatatggtaactgcaacaccaggtgtcaaactccaataggggcaataaactctagcatgccattccacaacatacaccctctcactatcggagaatgtcccaaatatgtgaaatcaaacaaattagtccttgcaactgggctcagaaatagtcctcaaagagagagaagaagaaaaGGGagaggactgtttggagctatagcaggttttatagagggaggatggcagggaatggttgatggttggtatgggtaccaccacagcaatgaacaagggagtggttacgctgcagacaaagaatctactcaaaaggcgatagacggagtcaccaataaggtcaattcaatcattgacaaaatgaacactcagtttgaggctgtaggaaaggaatttaataacttagagaggagaatagaaaatttaaacaagaagatggaagacggattcctagatgtctggacttataatgctgaacttctggttctcatggagaatgagagaactctagacttccatgactcaaatgtcaagaacctttacgacaaggtccgactacagcttaaggataatgcaaaagagctgggaaacggttgtttcgagttctatcacaaatgtaataatgaatgtatggaaagtgtgagaaacgggacgtatgactatccgcagtattcagaagaagcaagattaaaaagagaggaaataagtggagtaaaattggaatcaataggaatctaccaaatactgtcaatttattcaacagtggcgagttccctagtgctggcaatcatgatggctggtctatctttatggatgtgttccaacgggtcgttacagtgcagaatttgcatttga

>H5N1_A/chicken/Cameroon/16VIR3791-22/2016

cttgtcaaaagcgatcatatttgcattggttatcatgcaaataattcgacagagcaggttgacacaataatggaaaagaacgttactgttacacatgcccaagacatactggaaaagacacacaacggaaagctctgcgatctaaatggagtgaagcctctgattttaaaagactgtagtgtagcaggatggctcctcggaaatccattgtgtggcgaattcaccaatgtgccagaatggtcctacatagtagagaaggccaatccagccaatgatctctgttacccagggaatttcaacgattatgaggaactaaaacatctattgagcaggataaaccattttgagaaaatacagatcatccccaaagattcttggtcagatcatgaagcctcattgggggtgagcgcagcatgttcataccagggaaattcctccttcttcggaaatgtggtgtggcttatcaagaagaacaatgcatacccaacaataaaaaaaggctacaataataccaaccgagaagatctcttgatactgtgggggatccaccatcctaatgatgaagcagagcagacaaagctctatcaaaacccaactacctatatttccgttgggacttcaacactaaaccagagattggtaccaaaaatagccactagatccaaaataaacgggcaaagtggcagaatagatttcttctggacaattttaaaaccgaatgacacaatccatttcgagagtaatggaaatttcattgctccagaatatgcatacaaaattgtcaagaaaggagactccacaatcatgagaagtgaggtagaatatggtaactgcaacaccaggtgtcaaactccaataggggcaataaactctagcatgccattccacaacatacaccctctcactatcggagaatgtcccaaatatgtgaaatcaaacaaattagtccttgcaactgggctcagaaatagtcctcaaagagagagaagaagaaaaGGGagaggactgtttggagctatagcaggttttatagagggaggatggcagggaatggttgatggttggtatgggtaccaccacagcaatgaacaagggagtggttacgctgcagacaaagaatctactcaaaaggcgatagacggagtcaccaataaggtcaattcaatcattgacaaaatgaacactcagtttgaggctgtaggaaaggaatttaataacttagagaggagaatagaaaatttaaacaagaagatggaagacggattcctagatgtctggacttataatgctgaacttctggttctcatggagaatgagagaactctagacttccatgactcaaatgtcaagaacctttacgacaaggtccgactacagcttaaggataatgcaaaagagctgggaaacggttgtttcgagttctatcacaaatgtaataatgaatgtatggaaagtgtgagaaacgggacgtatgactatccgcagtattcagaagaagcaagattaaaaagagaggaaataagtggagtaaaattggaatcaataggaatctaccaaatactgtcaatttattcaacagtggcgagttccctagtgctggcaatcatgatggctggtctatctttatggatgtgttccaacgggtcgttacagtgcagaatttgcatttga

>H5N1_A/chicken/Cao Bang/20/2007

cttgttaaaagtgatcagatttgcattggttaccatgcaaacaactcgacagagcaggttgacacaataatggaaaagaacgttactgttacacatgcccaagatatactggaaaagacacacaacgggaagctctgcgatctagatggagtgaagcctctaattttaagagattgtagtgtagctggatggctcctcggaaacccaatgtgtgacgaattcatcaatgtgccggaatggtcttacatagtggagaaggccaacccagccaatgacctctgttacccagggaatttcaacgactatgaagaactgaaacacctattgagcagaataaaccattttgagaaaattcagatcatccccaaaagttcttggtccgatcatgaagcctcattaggggtgagctcagcatgtccataccagggaacgccctcctttttcagaaatgtggtatggcttatcaaaaagaacaatacatacccaacaataaagaaaagctacaataataccaaccaggaagatcttttgatactgtgggggattcatcattctaatgatgcggcagagcagacaaagctctatcaaaacccaaccacttatatttccgttgggacatcaacactaaaccagagattggtaccaaaaatagccactagatccaaagtaaacgggcaaagtggaaggatggatttcttctggacaattttaaaaccgaatgatgcaatcaacttcgagagtaatggaaatttcattgctccagaatatgcatacaaaattgtcaagaaaggggactcagcaattatgaaaagtgaagtggaatatggtaactgcaacaccaagtgtcaaactccaataggggcgataaactctagtatgccattccacaacatacaccctctcaccatcggggaatgccccaaatacgtgaaatcaaacaaattagtccttgcgactgggctcagaaatagtcctctaagagaaagaagaagaaaaGGGagaggactatttggagctatagcaggttttatagagggaggatggcagggaatggtagatggttggtatgggtaccaccatagcaatgagcaggggagtgggtacgctgcagacaaagaatccactcaaaaggcaataratggagtcaccaataaggtcaactcgatcattgacaaaatgaacactcagtttgaggccgttggaagggaatttaataacttagaaaggagaatagagaatttaaacaagaaaatggaagacggattcctagatgtctggacttataatgctgaacttctggttctcatggaaaacgagagaactctagacttccatgactcaaatgtcaagaacctttacgacaaggtccgactacagcttagggataatgcaaaggagctgggtaacggttgtttcgagttctatcacaaatgtaataatgaatgcatagaaagtgtaaaaaacggaacgtatgactacccacagtattcagaagaagcaagattaaaaagagaggaaataagtggagtaaaattggaatcaataggaacttaccaaatactgtcaatttattcaacagttgcgagttccctagcactggcaatcatggtggctggtctatctttatggatgtgctccaatgggtcgttacaatgcagaatttgcatttga

>H5N1_A/chicken/Central Java/UT3091/2005

cttgttaaaagtgatcagatttgcattggttaccatgcaaacaattcaacagagcaggttgacacaataatggaaaagaacgttactgttacacatgcccaagacatactggaaaagacacacaacgggaagctttgtgatctagatggagtgaagcctctaattttaagagattgtagtgtagctggatggctcctcgggaacccaatgtgtgacgaattcatcaatgtaccggaatggtcttacatagtggagaaggccaatccagccaatgacctctgctacccagggaatttcaatgactatgaagaactgaaacacctattgagcagaataaaccattttgagaaaattcagatcatccccaaaagttcttggtccgatcatgaagcctcatcaggggtgagctcagcatgtccatacctgggaacgccctccttttttagaaatgtggtatggcttatcaaaaagaacagtacatacccaacaataaaaagaagctacaataataccaaccaagaagatcttttggtactgtgggggattcaccatcctaatgatgcggcagagcaaacgaggctatatcaaaatccaaccacctatatttccgttgggacatcaacactgaaccagagattggtaccaaaaatagctaccagatccaaagtaaacggacaaagtggaaggatggagttcttctggacaattttaaaacctaatgatgcaatcaacttcgagagtaatggaaatttcattgctccagaatatgcctacaaaattgtcaagaaaggggactcagcaattatgaaaagtgaattggaatatggcaactgcaacaccaagtgtcaaactccaatgggggcgataaactctagtatgccattccacaacatacaccctctcaccatcggggagtgccccaaatatgtgaaatcaaacagattagtccttgcgactgggctcagaaatagccctcaaagagagGGGagaagaaaaaaaagaggactatttggagctatagcaggttttatagagggaggatggcagggaatggtagatggttggtatgggtaccaccatagcaatgagcagggaagtgggtacgctgcagacaaagaatccactcaaaaggcaatagatggagtcaccaataaggtcaactcgatcattgacaaaatgaacactcagtttgaggccgttggaagggaatttaataacttagaaaggagaatagagaatttaaacaagaagatggaagacggattcctagatgtctggacttataatgctgaacttctggttctcatggaaaatgagagaactctagactttcatgactcaaatgttaagaacctctacgacaaggtccgactacagcttaaggataatgcaaaggagctgggtaacggttgtttcgagttctatcacaaatgtgatgatgaatgtatggaaagtgtaagaaatgggacgtataactacccgcagtattcagaagaagcaagattaaaaagagaggaaataagtggggtaaaattggaatcaataggaatttaccaaatactgtcaatttattcaacagtagcgagttccctagcactggcaatcatgatggctggtctatctttatggatgtgctccaatggatcgttacaatgcagaatttgcatttga

>H5N1_A/chicken/Chiba/1/2011

cttgttaaaagcgatcatatttgcattggttatcatgcaaataactcgacagagcaggttgacacaataatggaaaagaacgttactgttacacatgcccaagacatactggaaaagacacacaacgggaagctctgcgatctaaatggagtgaagcctctgattttaaaagattgtagtgtagcgggatggctcctcggaaacccattgtgtgacgaattcatcaatgtgccagaatggtcttacatagtagagaaggccaagccagccaatgacctctgttacccagggaatttcaacgattatgaagaattgaaacacctattgagcaggataaaccattttgagaaaatacagatcatccccaaagactcttggtcagaacatgaagcctcattgggggtgagcgcagcatgttcataccagggaaattcctccttcttcagaaatgtggtatggcttatcaaaaaggacaatgcatacccaacaataaagaaaggctacaataataccaaccaagaagatctcttggtactgtgggggattcaccatcctaatgatgaggcagagcagacaaggctctatcaaaacccaaccacctatatttccattgggacatcaacactaaaccagagattggtaccaaaaatagccactagatccaaaataaacgggcaaagtggcaggatagatttcttctggacaattttaaaaccgaatgatgcaatccacttcgagagtaatggaaatttcattgctccagaatatgcatacaaaattgtcaagaaaggagactccacaattatgaaaagtgaagtggaatatggtaactgcaacaccaggtgtcagactccgataggggcgataaactctagtatgccattccacaacatacaccctctcaccatcggagaatgtcccaaatatgtgaaatcaaacaaattagtccttgcgactgggctcagaaatagtcctcaaagagagagaagaagaGGGaaaagaggactgtttggagctatagcaggttttatagagggaggatggcagggaatggtagatggttggtatgggtaccaccacagcaatgagcaggggagtgggtacgctgcagacaaagaatctactcaaaaggcaatagacggagtcaccaataaggtcaactcgatcattgacaaaatgaacactcagtttgaggccgtaggaagggaatttaataacttagagaggagaatagagaatttaaacaagaagatggaagacggattcctagatgtttggacttataatgctgaacttctggttctcatggaaaatgagagaactctagatttccatgactcaaatgtcaagaacctttacgataaggtcagactacagcttaaggataatgcaaaagagttgggtaacggttgtttcgagttctatcacaaatgtaataatgaatgtatggaaagtgtaagaaacggaacgtatgactacccgcagtattcagaagaagcaagactaaaaagagaggaaataagtggagtaaaattggaatcaataggaatctaccaaatactgtcaatttattcaacagtggcgagttccctagtgctggcaatcatgatggctggtctgtctttatggatgtgttccaacggatcgttacagtgcagaatttgcatttga

>H5N1_A/chicken/China/E93/2012

cttgttaaaagtgatcagatttgcgtgggctaccatgcaaacaactcgacagagcaggttgacacaataatggaaaagaacgttactgttacacatgctcaagacatactggagaagacacacaacgggaaactctgcaacctaaatggagtgaagcctttaattttggaagattgtagtgtagctggatggctcctcggaaacccaatgtgtgacaaatttctcaatgtgtcagaatggtcttacatagtggagaaggccagtccatccaatggcctttgttacccaggggatttcaatgattatgaagaactgaaacacctattgagcagaataaaccattttgagaaaattgagatcatctccaaaagtcattggtccaatcataatacctca---ggggtgagctcagcttgttcctatctggagaatccctcttttttcaggaatgtagtatggcttaccaaaaagaacaatacatacccaccaataaaggtgaactacaccaatgcccaccaaaaagatcttttggtactgtggggaatccaccaccccaataacgaggcagagcagaaaatgatctatcaaaacctaaacacttatgtttccgttggaacatcaacattaaaccagagattggtacccaaaatagctactaggtccaaagtgaacgggcaaagtggaagaatagacttcttctggacaattttaaagccgaatgatactatcaatttcgatagtaatggaaatttcattgccccaaaatatgcatacaaaattgtcaaggaaggggactcagcaattatgaaaagtgaattgaaatatggtaactgcaacaccaaatgccaaactccaataggggcgataaattctagtatgccattccacaacatacatcctctcaccatcggggaatgccccaaatatgtgaaatcaaacaggttagtcctcgcgactggactcagaaatgcccctcaaatagagggaagaagaagaaaaagaggactatttggagccatagcagggtttatagagggaggatggcagggaatggtagatggctggtatgggtaccaccatagtaatgagcagggaagtggatacgctgcagacaaagaatccactcaaaaagcagtagatggaatcaccaataagatcaactcgatcattgacaaaatgaacactcagtttgaggccgttggaagggaatttaataacttagaaaggagaatagaaaatttaaataaaaagatggaggacggattcctagatgtctggacttataacgctgaacttctggttctcatggaaaatgagagaactctagacttccatgactcaaatttcaagaatctgtatgaaaaggttcgactacagcttagggataatgcaaaggagctgggtaacggttgcttcgagttctaccacaaatgtgataatgaatgtatggaaagtgtaaagaacgggacgtatgactacccgcaatattcagaagaagcaagactaaacagagaggaaataagtggagtaaaattggaatcaactgtaacttaccaaatactgtcaatttattcaacagtggcgagttccctagtactggcaatcatggtggctggtctatctttatggatgtgctccaatggatcgttacaatgcagaatttgcatttga

>H5N1_A/chicken/China/E96/2012

cttgttaaaagtgatcagatttgcgtgggctaccatgcaaacaactcgacagagcaggttgacacaataatggaaaagaacgttactgttacacatgctcaagacatactggagaagacacacaacgggaaactctgcaacctaaatggagtgaagcctttaattttggaagattgtagtgtagctggatggctcctcggaaacccaatgtgtgacaaatttctcaatgtgtcagaatggtcttacatagtggagaaggccagtccagccaatggcctttgttacccaggggatttcaatgattatgaagaactgaaacacctattgagcagaataaaccattttgggaaaattgagatcatctccaaaagtcattggtccaatcataatacctca---ggggtgagctcagcttgttcctatctggagaatccctcttttttcaggaatgtagtatggcttaccaaaaagaataatacatacccaccaataaaggtgaactacaccaatgcccaccaaaaagatcttttggtactgtggggaatccaccaccccaataacgaggcagagcagaaaatgatctatcaaaacctaaacacttatgtttccgttggaacatcaacattaaaccagagattggtacccaaaatagctactaggtccaaagtgaacgggcaaagtggaagaatagacttcttctggacaattttaaagccgaatgatactatcaatttcgatagtaatggaaatttcattgccccaaaatatgcatacaaaattgtcaaggaaggggactcagcaattattaaaagtgaattgaaatatggtaactgcaacaccaaatgccaaactccaataggggcgataaattctagtatgccatttcacaacatacatcctctcaccatcggggaatgccccaaatatgtgaaatcaaacaggttagtcctcgcgactggactcagaaatgcccctcaaatagagggaagaagaagaaaaagaggactatttggagccatagcagggtttatagagggaggatggcagggaatggtagatggctggtatgggtaccaccatagtaatgagcagggaagtggatacgctgcagacaaagaatccactcaaaaagcagtagatggaatcaccaataagatcaactcgatcattgacaaaatgaacactcagtttgaggccgttggaagggaatttaataacttagaaaggagaatagaaaatttaaataaaaagatggaggacggattcctagatgtctggacttataacgctgaacttctggttctcatggaaaatgagagaactctagacttccatgactcaaatgtcaagaatctgtatgaaaaggttcgattacagcttagagataatgcaaaggagctgggtaacggttgcttcgagttctaccacaaatgtgataatgaatgtatggaaagtgtaaagaacgggacgtatgactacccgcaatattcagaagaagcaagactaaacagagaggaaataagtggagtaaaattggaatcaactgtaacttaccaaatactgtcaatttattcaacagtggcgagttccctagtactggcaatcatggtggctggtctatctttatggatgtgctccaatggatcgttacaatgcagaatttgcatttga

>H5N1_A/chicken/Connecticut/22-006118-001/2022

cttgttaaaagtgatcagatttgcattggttaccatgcaaacaattcgacagagcaagttgacacgataatggaaaagaacgtcactgttacacatgcccaagacatactggaaaaaacacacaacgggaagctctgtgatctaaatggggtgaagcctctgattttaaaggattgtagtgtagctggatggctcctcggaaacccaatgtgcgacgaattcatcagagtgccggaatggtcctacatagtggagcgggctaacccagctaatgacctctgttacccagggagcctcaatgactatgaagaactgaaacacatgttgagcagaataaatcattttgagaagattctgatcatccccaagagttcctggccaaatcatgaaacatcactaggggtgagcgcagcttgtccataccagggagcgccctcctttttcagaaatgtggtgtggcttatcaaaaagaacgatgcatacccaacaataaagataagctacaataataccaatcgggaagatctcttgatactgtgggggattcatcattccaacaatgcagaagagcagacaaatctctacaaaaacccaaccacctacatttcagttggaacatcaactttaaaccagaggttggcaccaaaaatagctactagatcccaagtaaacgggcaacgtggaagaatggacttcttctggacaatcttaaaaccagatgatgcaatccatttcgagagtaatggaaatttcattgctccagaatatgcatacaaaattgtcaagaaaggggactcaacaattatgaaaagtggagtggaatatggccactgcaacaccaaatgtcaaaccccagtaggtgcgataaattctagtatgccattccacaacatacatcctctcaccattggggaatgccccaaatacgtgaaatcaaacaagttggtccttgcgactgggctcagaaatagtcctctaagagaaaagagaagaaagGGGagaggcctgtttggggcgatagcagggtttatagagggaggatggcagggaatggttgatggttggtatgggtaccatcatagcaatgagcaggggagtgggtacgctgcggacaaagaatccacccaaaaggcaatagatggagttaccaataaggtcaattcaatcattgacaaaatgaacactcaatttgaggcagttggaagggagtttaataacttagaaaggagaatagagaatttgaacaagaaaatggaagacggattcctagatgtctggacctataatgctgaacttctagttctcatggaaaacgagaggactctagatttccatgattcaaatgtcaagaacctttacgacaaagtcagattacagcttagggataatgcaaaggagctgggtaacggctgtttcgaattctatcacaaatgtgataatgaatgtatggaaagtgtgagaaatgggacgtatgactaccctcagtattcagaagaagcaagattaaaaagagaagaaataagcggagtgaaattagaatcagtaggaacttaccagatactgtcaatttattcaacagcggcaagttccctagcactggcaatcatgatggctggtctatctttatggatgtgctccaatgggtcgttacagtgcagaatttgcatttag

>H5N1_A/chicken/Cote d'Ivoire/Viro001-100/2016

cttgtcaaaagcgatcatatttgcattggttatcatgcaaataactcaacggagcaggttgacacaataatggaaaagaacgttactgttacacatgcccaagacatactggaaaagacacacaacgggaagctctgtgatctaaatggagtgaagcctctgattttaaaagattgtagtgtagcaggatggctcctcggaaatccatcgtgtggcgaattcaccaatgtgccagaatggtcttacatagtagagaaggccaatccagccaatgacctctgttatccagggaatttcaacgattatgaggaactaaaacacctattgagcaggataaaccattttgagaaaatacagatcatccccaaagactcttggtcagatcatgaagcctcattgggggtgagcgcagcatgttcatatcagggaaattcctccttcttcagaaatgtggtgtggcttatcaaaaaggacaatgcatacccaacaataaagaaaggctacaataataccaaccgagaagatctcttgatactgtgggggatccaccatcctaatgatgaagcagagcagacaaagctctatcaaaacccaactacctatgtttccgttgggacttcaacactaaaccagagattggtgccaaagatagccactagatccaaaataaacgggcaaagtggcaggatagatttcttctggacaattttaaaaccgaatgacgcaatccatttcgagagtaatggaaatttcattgctccagaatatgcatacaaaattgtcaagaaaggagactccacaatcatgagaagtgaggtggaatatggtaactgcaacaccaggtgtcagactccaataggggcgataaactctagcatgccattccacaacatacaccctctcactatcggagaatgtcccaaatatgtgaaatcaaacaaattagtccttgcaactgggctcagaaatagtcctcaaagagagagaagaagaaaaGGGagaggactgtttggagctatagcaggttttatagagggaggatggcagggaatggtagatggttggtatgggtaccaccacagcaatgaacaaggaagtggttacgctgcagacaaagaatctactcaaaaggcgatagacggaatcaccaataaggtcaattcaatcattgacaaaatgaacactcagtttgaggctgtaggaaaagaatttaataacttagaaaggagaatagaaaatttaaacaagaagatggaagacggattcctagatgtctggacttataatgctgaacttctggttctcatggagaacgaaagaactctagacttccatgactcaaatgtcaagaacctttacgataaggtccgactacagctcaaggataatgcaaaagagctgggaaacggttgtttcgagttctatcacaaatgtaataatgaatgtatggaaagtgtaagaaacgggacgtatgactacccgcagtattcagaagaagcaagattaaaaagagaggaaataagtggagtaaaactggaatcaataggagtctaccaaatactgtcaatttattcaacagtggcgagttccctagtgctggcaatcatgatggctggtctatctttatggatgtgttccaacgggtcgttacagtgcagaatttgcatttga

>H5N1_A/chicken/Cote d'Ivoire/Viro005-89/2016

cttgtcaaaagcgatcatatttgcattggttatcatgcaaataactcaacggagcaggttgacacaataatggaaaagaacgttactgttacacatgcccaagacatactggaaaagacacacaacgggaagctctgcgatctaaatggagtgaagcctctgattttaaaagattgtagtgtagcaggatggctcctcggaaatccatcgtgtggcgaattcaccaatgtgccagaatggtcttacatagtagagaaggccaatccagccaatgacctctgttatccagggaatttcaacgattatgaggaactaaaacacctattgagcaggataaaccattttgagaaaatacagatcatccccaaagactcttggtcagatcatgaagcctcattgggggtgagcgcagcatgttcatatcagggaaattcctccttcttcagaaatgtggtgtggcttatcaaaaaggacaatgcatacccaactataaagaaaggctacaataataccaaccgagaagatctcttgatactgtgggggatccaccatcctaatgatgaagcagagcagacaaagctctatcaaaacccaactacctatatttccgttgggacttcaacactaaaccagagattggtgccaaagatagccactagatctaaaataaacgggcaaagtggcaggatagatttcttctggacaattttaaaaccgaatgacgcaatccatttcgagagtaatggaaatttcattgctccagaatatgcatacaaaattgtcaagaaaggagactccacaatcatgagaagtgaggtggaatatggtaactgcaacaccaggtgtcagactccaataggggcgataaactctagcatgccattccacaacatacaccctctcactatcggagaatgtcccaaatatgtgaaatcaaacaaattagtccttgcaactgggctcagaaatagtcctcaaagagagagaagaggaacaGGGagaggactgtttggagctatagcaggttttatagagggaggatggcagggaatggtagatggttggtatgggtaccaccacagcaatgaacaaggaagtggttacgctgcagacaaagaatctactcaaagggcgatagacggagtcaccaataaggtcaattcaatcattgacaaaatgaacactcagtttgaggctgtaggaaaagaatttaataacttagaaaggagaatagaaaatttaaacaagaagatggaagacggattcctagatgtctggacttataatgctgaacttctggttctcatggaaaacgagagaactctagacttccatgactcaaatgtcaagaacctttacgataaggtccgattacagctcaaggataatgcaaaagagctgggaaacggttgtttcgagttctatcacaaatgtaataatgaatgtatggaaagtgtaagaaacgggacgtatgactacccgcagtattcagaagaagcaagattaaaaagagaggaaataagtggagtaaaactggaatcaataggagtctaccaaatactgtcaatttattcaacagtggcgagttccctagtgctggcaatcatgatggctggtctatctttatggatgtgttccaacgggtcgttacagtgcagaatttgcatttga

>H5N1_A/chicken/Cote d'Ivoire/Viro008-14/2016
[truncated: 6,265,687 more chars]
