# Supplementary material for: Phylodynamics and Molecular Mutations of the Hemagglutinin Affecting Global Transmission and Host Adaptation of H5Nx Viruses
Source: Transbound Emerg Dis. 2023 Apr 14;2023:8855164. doi: 10.1155/2023/8855164 (PMC12017097; doi:10.1155/2023/8855164)
Supplement: Supplementary Materials — Supplemental Table 1: the number of the H5Nx HA sequences by subtype collected. Supplemental Table 2: the number of the H5Nx HA sequences by subtype and isolation year used for the phylogenetic analysis. Supplemental Table 3: amino acid mutations in the HA globular head region of reference human-isolated H5Nx viruses by clade. Supplemental Table 4: summary of natural selection pressure profiles of the H5Nx HAs by clade. Supplemental Data 1: initial sequence set of H5Nx HAs. Supplemental Data 2: selected sequence set of H5Nx HAs. Supplemental Data 3: sequence set of human-isolated H5Nx HAs. Supplemental Figure 1: the proportion of amino acid mutations around the HA globular head region of clade 2.3.4.4 H5Nx viruses by subtype and collection year. (a) The proportion of amino acid mutations around the HA globular head region of clade 2.3.4.4 H5Nx viruses is presented by each subtype (Supplemental Table 1). (b) The H5Nx HA sequences are divided into six periods; (a) 1997–2004 (n = 147), (b) 2005–2008 (n = 533), (c) 2009–2012 (n = 425), (d) 2013–2016 (n = 1082), (e) 2017–2020 (n = 842) and 2021-2022 (n = 860). Supplemental Figure 2: the proportion of the I155T and T160A mutations and molecular interactions of the HA globular head region of H5Nx viruses. (a) The proportion of the I155T (blue) and T160A (magenta) mutations in avian (dashed lines) and human-isolated H5Nx viruses (solid lines) is presented by each period (years). (b) Using the HA structure of VN1194, the receptor-binding pocket in H5 HA contains a conserved floor of residues Y95, W153, H183, Y195, and E190 (pale yellow), and Q226 and G228 (orange) that interact with α2,3 SA receptors. Residues 155 and 160 are colored blue and magenta. Supplemental Figure 3: structural analysis of the HA globular head region of human-isolated H5Nx viruses. Using the HA structure of VN1194, molecular interactions of the HA globular head region residues are estimated; (a) A/Nepal/19FL1997/2019 (H5N1) (NP19FL1197) in subclade 2. [file 8855164.f1.zip › dataS2_revision.docx]

>H5N6_2344g_A_chicken_Vietnam_Raho4_Cd_20_421_2020

atggagaaaatagtgcttcttcttgcagtggttagccttgtcaaaagtgatcagatttgcattggttaccatgcaaataactcgacagaacaggttgacacgataatggaaaaaaacgtcactgttacacatgcccaagacatactagaaaagacacacaacgggaggctctgcgatttgaatggagtgaaacctctgattttaaaggattgtagtgtagctggatggctccttggaaaccctatgtgcgacgagttcatcagagtgccggaatggtcctacatagtggagagggctaacccgccccacgacctctgttaccccgggaacctcaacgactatgaagaactgaaacatctattgagtagaataaatcattttgagaaaactctgatcatccccaaaagttcttggcccaatcatgaaacatcgttaggagtgagcgctgcatgccaataccagggaatgccttcctttttcagaaatgtggtatggctcatcaagaagaacgatgcatacccaacaatagagatgagctacaataataccaacagtgaagatcttttgatactgtgggggattcatcattctaacaacgcagcagaacaaacaaatctctataaaaacccaaccacctatgtttccgttgggacatcaacattaaaccagagattggtacccaaaatagctactagatcccaagtaaacgggcaacgtggaagaatggatttctactggacaattttaaaaccgaatgatgcaatccacttcgagagtaatggaaattttattgctccagaatatgcatacaaaattgtcaagaaaggggactcaacaatcatgaaaagtgagatggaatatggccgttgcaacaccaaatgccaaactccaataggggcgataaactctagtatgccattccacaatatacaccctctcacaatcggggaatgccccaaatatgtaaaatcaaacaaattagtacttgcgactgggctcagaaatagccccctaagagagGGGaggagaagaaaaagaggactatttggagctatagcaggatttatagagggaggatggcaaggaatggtagatggttggtatggataccaccatagcaatgaacagggaagtgggtacgctgccgacaaagaatccacccaaaaggcaatagatggagttaccaataaggtcaactcgatcattgaaaagatgaacactcaatttgaggccgttgggagggaatttaataacttagaaaggagaatagagaatttaaacaagaaaatggaagacggattcctggatgtctggacttataatgcggaacttctagttctcatggaaaatgagagaaccctagatttccatgactcaaatgtcaagaacctttatgacaaagtccgactacagcttagggacaatgcaaaggagctgggtaatggttgctttgagttctatcacaaatgtgataatgaatgtatggaaagtgtaagaaatggaacatataactaccctcagtactcagaagaagcaagattgaaaagagaagaaataagcggagtgaaattggaatcaataggaacttaccagatactgtcaatttattcaacagtggcgagttccctagcactggcaatcattgtggctggtctatctttatggatgtgttccaatgggtcactacaatgcagaatttgcatc

>H5N6_2344e_A_duck_Hyogo_1_2016

atggagaaaatagtgcttcttcttgcagtggttagccttgttaaaagtgatcagatttgcattggttaccatgcaaacaactcgacagagcaggttgacacgataatggaaaaaaacgtcactgttacacatgcccaagacatactggaaaagacacacaacgggaggctctgcgatctgaatggagtgaaacctctgattttaaaggattgtagtgtagctggatggcttcttggaaacccaatgtgcgacgaattcatcagagtgccggaatggtcttacatagtggagaggactaacccagccaatgacctctgttacccagggaacctcaatgactatgaagaactgaaacacctattgagcagaataaatcattttgagaagactctgatcatccccaagagttcttggcccaatcatgaaacatcaGGGggggtgagcgcagcatgcccataccagggagtgccctcctttttcagaaatgtggtatggcttaccaagaagaacgatgcatacccaacaataaagatgagctacaataataccaatggggaagatcttttgatactgtgggggattcatcattccaacaatgcagcagagcagacaaatctctataaaaacccaaccacctatgtttccgttgggacatcaacattaaaccagagattggtgccaaaaatagctactagatcccaagtaaacgggcaacaaggaagaatggatttcttctggacaattttaaaaccgaatgatgcaatccactttgagagtaatggaaattttattgctccagaatatgcatacaaaatagtcaagaaaggggactcaacaattatgaaaagtgaaatggaatatggccactgcaacaccaaatgtcaaactccaataggggcgataaactctagtatgccattccacaatatacaccctctcaccatcggggagtgccccaaatacgtgaaatcaaacaaattagtccttgcgactggactcagaaatagtcctttaagagaaGGGagaagaagaaaaagaggactatttggagctatagcagggttcatagagggaggatggcaaggaatggtagatggttggtatgggtaccaccatagcaatgaacaggggagtgggtacgctgcagacagagaatccacccaaaaggcaatagatggagttaccaataaggtcaactcgataatcgacaaaatgaacactcaatttgaggccgttggaagggagtttaataacttagaacggagaatagagaatttaaataagaaaatggaagacggattcctagatgtctggacttacaatgctgaacttttagttctcatggaaaatgagagaactttagattttcacgattcaaatgtaaagaacctttatgacaaagtcagactacagcttagggataatgcaaaggagctaggtaatggttgtttcgagttctatcataaatgtgataatgaatgtatggaaagtgtaagaaatgggrcgtatgactatccccagtattcagaagaggcaagattaaaaagggaagaaataagcggagtgaaattggaatcaataggaacttaccaaatactgtcaatttattcaacagtggcgagttccctagcactggcaatcattgtggctggtctatctttatggatgtgctccaatgggtcgttacaatgcagaatttgcatt

>H5N6_2344f_A_chicken_Vietnam_NCVD_15A59_2015

atggagaaaatagtgcttcttcttgcattggttagccttgttaaaagtgatcagatttgcattggttaccatgcaaacaactcgacagagcaggttgacacaataatggaaaaaaacgtcactgttacacatgcccaagacatactggaaaagacacacaacgggaggctctgcgatctgaatggagtgaaacctctgatcttaaaggattgtagtgtagctggatggcttcttggaaacccaatgtgcgacgagttcatcagagtgccggaatggtcttacatagtggagagggctaacccagccaatgacctctgttacccagggaatctcaatgactatgaagaactgaaacacttattgagcagaataaatcattttgagaagactctgatcatccccaagagttcttggcccaatcatgaaacatcattgggggtgagcgcagcatgtccataccagggaatgccctcctttttcagaaatgtggtatggcttaccaagaagaacgatgcatacccaacaataaaggtgagctacaataataccaatagggaagatcttttgatactgtgggggattcatcattccaacaatgcagcagagcagacaaatctctataaaaacccaaccacctatgtttccgttgggacatcaacattaaaccagagattggtgcccaaaatagctactagatcccaagtaaacgggcaacgtggaagaatggatttcttctggacaattttaaaaccgaatgatgcaatccacttcgagagtaatggaaattttattgctccagaatatgcatacaaaattgtcaagaaaggggactcaacaattatgaaaagtgaaatggaatacggccactgcaacaccaaatgtcaaactccaataggggcgataaactctagtatgccattccacaatatacaccctctcactatcggggagtgccccaaatacgtgaaatcaaacaaattagtccttgcgactgggctcagaaatagtcctctaagagaaGGGagaagaagaaaaagagggctatttggagctattgcaggttttatagagggaggctggcagggaatggtagatggttggtatgggtaccaccatagcaatgaacaggggagtgggtacgctgcagacagagaatccacccaaaaggcaatagatggagttaccaataaggtcaactcgatcattgacaaaatgaacactcaatttgaggccgttggaagggaatttaataacttagaacggagaatagagaatttaaataagaaaatggaagacggattcctagatgtctggacttataatgctgaacttttagttctcatggaaaatgagagaactctagatttccatgactcaaatgtcaagaacctttatgacaaagtccgactacagcttagggataatgcaaaggagctgggtaatggttgtttcgagttctatcacaaatgtgataatgaatgtatggaaagtgtaagaaatgggacgtatgactacccccaatattcagaagaagcaagattaaaaagggaagaaataagcggagtgaaactggaatcaataggaacttaccaaatactgtcaatttattcaacagtggcaagttccctaacactggcaatcattgtggctggtctatctttatggatgtgctccaatgggtcgttacaatgcagaatttgcatt

>H5N1_A_England_215201407_2021

atggagaacatagtacttcttcttgcaatagttaaccttgttaaaagtgatcagatttgcattggttaccatgcaaacaattcgacagagcaagttgacacgataatggaaaagaacgtcactgttacacatgcccaagacatactggaaaaaacacacaacgggaagctctgtgatctaaatggggtgaagcctctgattttaaaggattgtagtgtagctggatggctcctcggaaacccaatgtgcgacgaattcatcagagtgccggaatggtcctacatagtggagcgggctaatccagctaatgacctctgttacccagggagcctcaatgactatgaagaactgaaacacctgttgagcagaataaatcattttgagaagattctgatcatccccaagagttcctggccaaatcatgaaacatcactaggggtgagcgcagcttgtccataccagggaacgccctcctttttcagaaatgtggtgtggcttatcaaaaagaacgatgcatacccaacaataaagataagctacaataataccaatcgggaagatctcttgatactgtgggggattcatcattccaacaatgcagaagagcagacaaatctctacaaaaacccaaccacctacatttcagttgggacatcaactttaaaccagaggttggtaccaaaaatagctactagatcccaagtaaacgggcaacgtggaagaatggacttcttctggacaattttaaaaccagatgatgcaatccatttcgagagtaatggaaatttcattgctccagaatatgcatacaaaattgtcaagaaaggggactcaacaattatgaaaagtggagtggaatatggccactgcaacaccaaatgtcaaaccccagtaggagcgataaattctagtatgccattccacaacatacatcctctcaccattggggaatgccccaaatacgtgaagtcaaacaagttggtccttgcgactgggcttagaaatagtcctctaagagaaGGGaagagaagaaaaagaggcctgtttggggcgatagcagggtttatagagggaggatggcagggaatggttgatggttggtatgggtaccatcatagcaatgagcaggggagtgggtacgctgcagacaaagaatccacccaaaaggcaatagatggagttaccaataaggtcaactcaatcattgacaaaatgaacactcaatttgaggcagttggaagggagtttaataacttagaaaggaggatagagaatttgaacaagaaaatggaagacggattcctagatgtctggacctataatgctgaacttctagttctcatggaaaacgagaggactctagatttccatgattcaaatgtcaagaacctttacgacaaagtcagactacagcttagggataatgcaaaggagctgggtaacggctgtttcgaattctatcacaaatgcgataatgaatgtatggaaagtgtgagaaatgggacgtatgactaccctcagtattcagaagaagcaagattaaaaagagaagaaataagcggagtgaaattagaatcaataggaacttaccagatactgtcaatttattcaacagcggcaagttccctagcactggcaatcatgatggctggtctatctttatggatgtgctccaatgggtcgttacagtgcagaatttgcatt

>H5N1_A_duck_Ireland_036646_22VIR1325_18_2021

atggagaacatagtacttcttcttgcaacagttagccttgttaaaagtgatcagatttgcattggttaccatgcaaacaattcgacagagcaggttgacacgataatggaaaagaacgtcactgttacacatgcccaagacatactggaaaaaacacacaacgggaagctctgtgatttaaatggggtgaagcctctgattttaaaggattgtagtgtagctggatggctcctcggaaacccaatgtgcgacgaattcatcagagtgccggaatggtcctacatagtggagcgggctaatccagccaatgacctctgttacccaggaagcctcaatgactatgaagaactgaaacacctgttgagcagaataaatcattttgagaagattctgatcatccccaagagttcctggccaaatcatgaaacatcactaggggtgagcgcagcttgtccataccagggagcgccctcctttttcagaaatgtgttgtggcttatcaaaaagaacgatgcatacccaacaataaagataagctacaataataccaatcgggaagatctcttgatattgtgggggattcatcattccaacaatgcagaagagcagacaaatctctataaaaacccaaccacctacatttcagttggaacatcaactttaaaccagaggttggtaccaaaaatagctactagatcccaagtaaacgggcaacgtggaagaatggacttcttctggacaattttaaaaccagatgatgcaatccatttcgagagtaatggaaatttcattgcaccaaaatatgcatataaaattgtcaagaaaggggactcaacaattatgaaaagtggagtggaatatggccactgcaacaccaaatgtcaaaccccagtaggagcgataaattctagtatgccattccacaacatacatcctctcaccattggggaatgccccaaatacgtgaagtcaaacaagttggtccttgcgactgggctcagaaatagtcctctaagagaaGGGaagagaagaaaaagaggcctgtttggggcgatagcagggtttatagagggaggatggcagggaatggttgatggttggtatgggtaccatcatagcaatgagcaggggagtgggtacgctgcagacaaagaatccacccaaaaggcaatagatggagttaccaataaggtcaactcaatcattgacaaaatgaacactcaatttgaggcagttggaagggagtttaataacttagaaaggaggatagagaatttgaacaagaaaatggaagacggattcctagatgtctggacctataatgctgaacttctagttctcatggaaaacgagaggactctagatttccatgattcaaatgtcaagaacctttacgacaaagtcagactacagcttagggacaatgcaaaggagctgggtaatggctgtttcgaattctatcacaaatgcgataatgaatgtatggaaagtgtgagaaatgggacgtatgactaccctcagtattcagaagaagcaagattaaaaagagaagaaataagcggagtgaaattagaatcaataggaacttaccagatactgtcaatttattcaacagcggcgagttccctagcactggcaatcatgatagctggtctatctttatggatgtgctccaatgggtcgttacagtgcagaatttgcatt

>H5N1_A_chicken_England_000187_2022

atggagaacatagtacttcttcttgcaacagttagccttgttaaaagtgatcagatttgcattggttaccatgcaaacaattcgacagagcaggttgacacgataatggaaaagaacgtcactgttacacatgcccaagacatactggaaaaaacacacaacgggaagctctgtgatttaaatggggtgaagcctctgattttaaaggattgtagtgtagctggatggctcctcggaaacccaatgtgcgacgaattcatcagagtgccggaatggtcctacatagtggagcgggctaatccagccaatgacctctgttacccagggagcctcaatgactatgaagaactgaaacacctgttgagcagaataaatcattttgagaagattctgatcatccccaagagttcctggccaaatcatgaaacatcactaggggtgagcgcagcttgtccataccagggagcgccctcctttttcagaaatgtgttgtggcttatcaaaaagaacgatgcatacccaacaataaagataagctacaataataccaatcgggaagatctcttgatattgtgggggattcatcattccaacaatgcagaagagcagacaaatctctataaaaacccaaccacctacatttcagttggaacatcaactttaaaccagaggttggtaccaaaaatagctactagatcccaagtaaacgggcaacgtggaagaatggacttcttctggacaattttaaaaccaggtgatgcaatccatttcgagagtaatggaaatttcattgcaccagaatatgcatataaaattgtcaagaaaggggactcaacaattatgaaaagtggagtggaatatggccactgcaacaccaaatgtcaaaccccagtaggagcgataaattctagtatgccattccacaacatacatcctctcaccattggggaatgccccaaatacgtgaagtcaaacaagttggtccttgcgactgggctcagaaatagtcctctaagagaaGGGaagagaagaaaaagaggcctgtttggggcgatagcagggtttatagagggaggatggcagggaatggttgatggctggtatgggtaccatcatagcaatgagcaggggagtgggtacgctgcagacagagaatccacccaaaaggcaatagatggggttaccaataaggtcaactcaatcattgacaaaatgaacactcaatttgaggcagttggaagggagtttaataactttgaaaggaggatagagaatttgaacaagaaaatggaagacggattcctagatgtctggacctataatgctgaacttctagttctcatggaaaacgagaggactctagatttccatgattcaaatgtcaagaacctttacgacaaagtcagactacagcttagggacaatgcaaaggagctgggtaatggctgtttcgaattctatcacaaatgcgataatgaatgtatggaaagtgtgagaaatgggacgtatgactaccctcagtattcagaagaagcaagattaaaaagagaagaaataagcggagtgaaattagaatcaataggaacttaccagatactgtcaatttattcaacagcggcgagttccctagcactggcaatcatgatagctggtctatctttatggatgtgctccaatgggtcgttacagtgcagaatttgcatt

>H5N1_A_Sanderling_Netherlands_1_2022

atggagaacatagtacttcttcttgcaacagttagccttgttaaaagtgatcagatttgcattggttaccatgcaaacaattcgacagagcaggttgacacgataatggaaaagaacgtcactgttacacatgcccaagacatactggaaaaaacacacaacgggaagctctgtgatttaaatggggtgaagcctctgattttaaaggattgtagtgtagctggatggctcctcggaaacccaatgtgcgacgaattcatcagagtgccggaatggtcctacatagtggagcgggctaatccagccaatgacctctgttacccagggagcctcaatgactatgaagaactgaaacacctgttgagcagaataaatcattttgagaagattctgatcatccccaagagttcctggccaaatcatgaaacatcactaggggtgagcgcagcttgtccataccagggagcgccctcctttttcagaaatgtgttgtggcttatcaaaaagaacgatgcatacccaacaataaagataagctacaataataccaatcgggaagatctcttgatactgtgggggattcatcattccaacaatgcagaagagcagacaaatctctataaaaacccaaccacctacatttcagttggaacatcaactttaaaccagaggttggtaccaaaaatagctactagatcccaagtaaacgggcaacgtggaagaatggacttcttctggacaattttaaaaccagatgatgcaatccatttcgagagtaatggaaatttcattgcaccagaatatgcatataaaattgtcaagaaaggggactcaacaattatgaaaagtggagtggaatatggccactgcaacaccaaatgtcaaaccccagtaggagcgataaattctagtatgccattccacaacatacatcctctcaccattggggaatgccccaaatacgtgaagtcaaacaagttggtccttgcgactgggctcagaaatagtcctctaagagaaGGGaagagaagaaaaagaggcctgtttggggcaatagcagggtttatagagggaggatggcagggaatggttgatggttggtatgggtaccatcatagcaatgagcaggggagtgggtacgctgcagacaaagaatccacccaaaaggcaatagatggagttaccaataaggtcaactcaatcattgacaaaatgaacactcaatttgaggcagttggaagggagtttaataacttagaaaggaggatagagaatttgaacaagaaaatggaagacggattcctagatgtctggacctataatgctgaacttctagttctcatggaaaacgagaggactctagatttccatgattcaaatgtcaagaacctttacgacaaagtcagactacagcttagggacaatgcaaaggagctgggtaatggatgtttcgaattctatcacaaatgcgataatgaatgtatggaaagtgtgagaaatgggacgtatgactaccctcagtattcagaagaagcaagattaaaaagagaagaaataagcggagtgaaattagaatcaataggaatttaccagatactgtcaatttattcaacagcggcgagttccctagcactggcaatcatgatagctggtctatctttatggatgtgctccaatgggtcgttacagtgcagaatttgcatt

>H5N1_A_goose_Tyumen_33_52V_2021

atggagaacatagtacttcttcttgcaacatttagccttgttaaaagtgatcagatttgcattggttaccatgcaaacaattcgacagagcaggttgacacgataatggaaaagaacgtcactgttacacatgcccaagacatactggaaaaaacacacaacgggaagctctgtgatttaaatggggtgaagcctctgattttaaaggattgtagtgtagctggatggctcctcggaaacccaatgtgcgacgaattcatcagagtgccggaatggtcctacatagtggagcgggctaatccagccaatgacctctgttacccagggagcctcaatgactatgaagaactgaaacacctgttgagcagaataaatcattttgagaagattctgatcatccccaagagttcctggccaaatcatgaaacatcactaggggtgagcgcagcttgtccataccagggagcgccctcctttttcagaaatgtggtgtggcttatcaaaaagaacgatgcatacccaacaataaagataagctacaacaataccaatcgggaagatctcttgatactgtgggggattcatcattccaacaatgcagaagagcagacaaatctctataaaaacccaaccacctacatttcagttggaacatcaactttaaaccagaggttggtaccaaaaatagctactagatcccaagtaaacgggcaacgtggaagaatggacttcttctggacaattttaaaaccagatgatgcaatccatttcgagagtaatggaaatttcattgcaccagaatatgcatataaaattgtcaagaaaggggactcaacaattatgaaaagtggagtggaatatggccactgcaacaccaaatgtcaaaccccagtaggagcgataaattctagtatgccattccataacatacatcctctcaccattggggaatgccccaaatacgtgaagtcaaacaagttggtccttgcgactgggctcagaaatagtcctctaagagaaGGGaagagaagaaaaagaggcctgtttggggcgatagcagggtttatagagggaggatggcagggaatggttgatggttggtatgggtaccatcatagcaatgagcaggggagtgggtacgctgcagacaaagaatccacccaaaaggcaatagatggagttaccaataaggtcaactcaatcattgacaaaatgaacactcaatttgaggcagttggaagggagtttaataacttagaaaggaggatagagaatttgaacaagaaaatggaagacggattcctagatgtctggacctataatgctgaacttctagttctcatggaaaacgagaggactctagatttccatgattcaaatgtcaagaacctttacgacaaagtcagactacagcttagggataatgcaaaggagctgggtaatggctgtttcgaattctatcacaaatgcgataatgaatgtatggaaagtgtgagaaatgggacgtatgactaccctcagtattcagaagaagcaagattaaaaagagaagaaataagcggagtgaaattagaatcaataggaacttaccagatactgtcaatttattcaacagcggcgagttccctagcactggcaatcatgatagctggtctatctttatggatgtgctccaatgggtcgttacagtgcagaatttgcatt

>H5N2_A_chicken_Poland_H182_22VIR2515_1_2022

atggagaacatagtacttcttcttgcaacagttagccttgttaaaagtgatcagatttgcattggttaccatgcaaacaattcgacagagcaggttgacacgataatggaaaagaacgtcactgttacacatgcccaagacatactggaaaaaacacacaacgggaagctctgtgatttaaatggggtgaagcctctgattttaaaggattgtagtgtagctggatggctcctcggaaacccaatgtgcgacgaattcatcagagtgccggaatggtcctacatagtggagcgagctaatccagccaatgacctctgttacccaggaagcctcaatgactatgaagaactgaaacacctgttgagcagaataaatcattttgagaagattctgatcatccccaagagttcctggccaaatcatgaaacatcactaggggtgagcgcagcttgtccataccagggagcgccctcctttttcagaaatgtggtgtggcttatcaaaaagaacgatgcatacccaacaataaagataagctacaataataccaatcgggaagatctcttgatactatgggggattcatcattccaacaatgcagaagagcagacaaatctctataaaaacccaaccacctacatttcagttggaacatcaactttaaaccagaggttggtaccaaaaatagctactagatcccaagtaaacgggcaacgtggaagaatggacttcttctggacaattttaaaaccagatgatgcaatccatttcgagagtaatggaaatttcattgcaccagaatatgcatataaaattgtcaagaaaggggactcaacaattatgaaaagtggagtggaatatggccactgcaacaccaaatgtcaaaccccagtaggagcgataaattctagtatgccattccacaacatacatcctctcaccattggggaatgccccaaatacgtgaagtcaaacaagttggtacttgcgactgggctcagaaatagtcatctaagagaaGGGaagagaagaaaaagaggcctgtttggggcgatagcagggtttatagagggaggatggcagggaatggttgatggttggtatgggtaccatcatagcaatgagcaggggagtgggtacgctgcagataaagaatccacccaaaaggcaatagatggagttaccaataaggtcaactcaatcattgacaaaatgaacactcaatttgaggcagttggaagggagtttaataacttagaaagaaggatagagaatttgaacaagaaaatggaagacggattcctagatgtctggacctataatgctgaacttctagttctcatggaaaacgagaggactctagatttccatgattcaaatgtcaagaacctttacgacaaagtcagactacagcttagggataatgcaaaggagctgggtaatggctgtttcgaattctatcacaaatgcgataatgaatgtatggaaagtgtgagaaatgggacgtatgactaccctcagtattcagaagaagcaagattaaaaagagaagaaataagcggagtgaaattagaatcaataggaacttaccagatactgtcaatttattcaacagcggcgagttccctagcactggcaatcatgatagctggtctatctttatggatgtgctccaatgggtcgttacagtgcagaatttgcatt

>H5N2_A_swan_Germany_BW_AI00997_2022

atggagaacatagtacttcttcttgcaacagttagccttgttaaaagtgatcagatttgcattggttaccatgcaaacaattcgacagagcaggttgacacgataatggaaaagaacgtcactgttacacatgcccaagacatactggaaaaaacacacaacgggaagctctgtgatttaaatggggtgaagcctctgattttaaaggattgtagtgtagctggatggctcctcggaaacccaatgtgcgacgaattcatcagagtgccggaatggtcctacatagtggagcgggctaatccagccaatgacctctgttacccaggaagcctcaatgactatgaagaactgaaacacctgttgagcagaataaatcattttgagaagattctgatcatccccaagagttcctggccaaatcatgaaacatcactaggggtgagcgcagcttgtccataccagggagcgccctcctttttcagaaatgtggtgtggcttatcaaaaagaacgatgcatacccaacaataaagataagctacaataataccaatcgggaagatctcttgatactatgggggattcatcattccaacaatgcagaagagcagacaaatctctataaaaacccaaccacctacatttcagttggaacatcaactttaaaccagaggttggtaccaaaaataactactagatcccaagtaaacgggcaacgtggaagaatggacttcttctggacaattttaaaaccagatgatgcaatccatttcgagagtaatggaaatttcattgcaccagaatatgcatataaaattgtcaagaaaggggactcaacaattatgaaaagtggagtggaatatggccactgcaacaccaaatgtcaaaccccagtaggagcgataaattctagtatgccattccacaacatacatcctctcaccattggggaatgccccaaatacgtgaagtcaaacaagttggttcttgcgactgggctcagaaatagtcctctaagagaaGGGaagagaagaaaaagaggcctgtttggggcgatagcagggtttatagagggaggatggcagggaatggttgatggttggtatgggtaccatcatagcaatgagcaggggagtgggtacgctgcagataaagaatccacccaaaaggcaatagatggagttaccaataaggtcaactcaatcattgacaaaatgaacactcaatttgaggcagttggaagggagtttaataacttagaaagaaggatagagaatttgaacaagaaaatggaagacggattcctagatgtctggacctataatgctgaacttctagttctcatggaaaacgagaggactctagatttccatgattcaaatgtcaagaacctttacgacaaagtcagactacagcttagggataatgcaaaggagctgggtaatggctgtttcgaattctatcacaaatgcgataatgaatgtatggaaagtgtgagaaatgggacgtatgactaccctcagtattcagaagaagcaagattaaaaagagaagaaataagcggagtgaaattagaatcaataggaacttaccagatactgtcaatttattcaacagcggcgagttccctagcactggcaatcatgatagctggtctatctttatggatgtgctccaatgggtcgttacagtgcagaatttgcatt

>H5N1_A_swan_France_21P012384_2021

atggagaacatagtacttcttcttgcaacagttagccttgttaaaagtgatcagatttgcattggttaccatgcaaacaattcgacagagcaggttgacacgataatggaaaagaacgtcactgttacacatgcccaagacatactggaaaaaacacacaacgggaagctctgtgatttaaatggggtgaagcctctgattttaaaggattgtagtgtagctggatggctcctcggaaacccaatgtgcgacgaattcatcagagtgccggaatggtcctacatagtggagcgggctaatccagccaatgacctctgttacccagggagcctcaatgactatgaagaactgaaacacctgttgagcagaataaatcattttgagaagattctgatcatccccaagagttcctggccaaatcatgaaacatcactaggggtgagcgcagcttgtccataccagggagcgccctcctttttcagaaatgtggtgtggcttatcaaaaagaacgatgcatacccaacaataaagataagctacaataataccaatcgggaagatctcttgatattgtgggggattcatcattccaacaatgcagaagagcagacaaatctctataaaaacccaaccacctacatttcagttggaacatcaactttaaaccagaggttggtaccaaaaatagctactagatcccaagtaaacgggcaacgtggaagaatggacttcttctggacaattttaaaaccagatgatgcaatccatttcgagagtaatggaaatttcattgcaccagaatatgcatataaaattgtcaagaaaggggactcaacaattatgaaaagtggagtggaatatggccactgcagcaccaaatgtcaaaccccagtaggagcgataaattctagtatgccattccacaacatacatcctctcaccattggggaatgccccaaatacgtgaagtcaaacaagttggtccttgcgactgggcttagaaatagtcctctaagagaaGGGaagagaagaaaaagaggcctgtttggggcgatagcagggtttatagagggaggatggcagggaatggttgatggttggtatgggtaccatcatagcaatgagcaggggagtgggtacgccgcagacaaagaatccacccaaaaggcaatagatggagttaccaataaggtcaactcaatcattgacaaaatgaacactcaatttgaggcagttggaagggagtttaataacttagaaaggaggatagagaatttgaacaagaaaatggaagacggattcctagatgtctggacctataatgctgaacttctagttctaatggaaaacgagaggactctagatttccatgattcaaatgtcaagaacctttacgacaaagtcagactacagcttagggataatgcaaaggagctgggtaatggctgtttcgaattctatcacaaatgcgataatgaatgtatggaaagtgtgagaaatgggacgtatgactaccctcagtattcagaagaagcaagattaaaaagagaagaaataagcggagtgaaattagaatcaataggaacttaccagatactgtcaatttattcaacagcggcgagttccctagcactggcaatcatgatagctggtctatctttatggatgtgctccaatgggtcgttacagtgcagaatttgcatt

>H5N1_A_Anser_albifrons_Belgium_15465_0010_2021

atggagaacatagtacttcttcttgcaacagttagccttgttaaaagtgatcagatttgcattggttaccatgcaaacaattcgacagagcaggttgacacgataatggaaaagaacgtcactgttacacatgcccaagacatactggaaaaaacacacaacgggaagctctgtgatttaaatggggtgaagcctctgattttaaaagattgtagtgtagctggatggctcctcggaaacccaatgtgcgacgaattcatcagagtgccggaatggtcctacatagtggagcgggctaatccagccaatgacctctgttacccagggagcctcaatgactatgaagaactgaaacacctgttgagcagaataaatcattttgagaagattctgatcatccccaagagttcctggccaaatcatgaaacatcactaggggtgagcgcagcttgtccataccagggagcgccctcctttttcagaaatgtggtgtggcttatcaaaaagaacgatgcatacccaacaataaagataagctacaataataccaatcgggaagatctcttgatactgtgggggattcatcattccaacaatgcagaagagcagacaaatctctataaaaacccaaccacctacatttcagttggaacatcaactttaaaccagaggttagtaccaaaaatagctactagatcccaagtaaacgggcaacgtggaagaatggacttcttctggacaattttaaaaccagatgatgcaatccatttcgagagtaatggaaatttcattgcaccagaatatgcatataaaattgtcaagaaaggggactcaacaattatgaaaagtggagtggaatatggccactgcaacaccaaatgtcaaaccccagtaggagcgataaattctagtatgccattccacaacatacatcctctcaccattggggaatgccccaaatacgtgaagtcaaacaagttggtccttgcgactgggctcagaaatagtcttctaagagaaGGGaagagaagaaaaagaggcctgtttggggcgatagcagggtttatagagggaggatggcagggaatggttgatggttggtatgggtaccatcatagcaatgagcaggggagtgggtacgctgcagacaaagaatccacccaaaaggcaatagatggagttaccaataaggtcaactcaatcattgacaaaatgaacactcaatttgaggcagttggaagggagtttaataacttagaaaggaggatagagaatttgaacaagaaaatggaagacggattcctagatgtctggacctataatgctgaacttctagttctcatggaaaacgagaggactctagatttccatgattcaaatgtcaagaacctttacgacaaagtcagactacagcttagggataatgcaaaggagctgggtaatggctgtttcgaattctatcacaaatgcgataatgaatgtatggaaagtgtgagaaatgggacgtatgactaccctcagtattcagaagaagcaagattaaaaagagaagaaataagcggagtgaaattagaatcaataggaacttaccagatactgtcaatttattcaacagcggcgagttccctagcactggcaatcatgatagctggtctatctttatggatgtgctccaatgggtcgttacagtgcagaatttgcatt

>H5N1_A_mute_swan_Croatia_100_2021

atggagaacatagtacttcttcttgcaacagttagccttgttaaaagtgatcagatttgcattggttaccatgcaaacaattcgacagagcaggttgacacgataatggaaaagaacgtcactgttacacatgcccaagacatactggaaaaaacacacaacgggaagctctgtgatttaaatggggtgaagcctctgattttaaaggattgtagtgtagctggatggctcctcggaaacccaatgtgcgacgaattcatcagagtgccggaatggtcctacatagtggagcgggctaatccagccaatgacctctgttacccagggagcctcaatgactatgaagaactgaaacacctgttgagcagaataaatcattttgagaagattctgatcatccccaagagttcctggccaaatcatgaaacatcactaggggtgagcgcagcttgtccataccagggagcgccctcctttttcagaaatgtggtgtggcttatcaaaaagaacgatgcatacccaacaataaagataagctacaataataccaatcgggaagatctcttgatactgtgggggattcatcattccaacaatgcagaagagcagacaaatctctataaaaacccaaccacctacatttcagttggaacatcaactttaaaccagaggttggtaccaaaaatagctactagatcccaagtaaacgggcaacgtggaagaatggacttcttctggacaattttaaaaccagatgatgcaatccatttcgagagtaatggaaatttcattgcaccagaatatgcatataaaattgtcaagaaaggggactcaacaattatgaaaagtggagtggaatatggccactgcaacaccaaatgtcaaaccccagtaggagcgataaattctagtatgccattccacaacatacatcctctcaccattggggaatgccccaaatacgtgaagtcaaacaagttggtccttgcgactgggctcagaaatagtcctctaagagaaGGGaagagaagaaaaagaggcctgtttggggcgatagcagggtttatagagggaggatggcagggaatggttgatggttggtatgggtaccatcatagcaatgagcaggggagtgggtacgctgcagacaaagaatccacccaaaaggcaatagatggagttaccaataaggtcaactcaatcattgacaaaatgaatactcaatttgaggcagttggaagggagtttaataacttagaaaggaggatagagaatttgaacaagaaaatggaagacggattcctagatgtctggacctataatgctgaacttctagttctcatggaaaacgagaggaccctagatttccatgattcaaatgtcaagaacctttacgacaaagtcagactacagcttaggaataatgcaaaggagctgggtaatggctgtttcgaattctatcacaaatgcgataatgaatgtatggaaagtgtgagaaatgggacgtatgactaccctaagtattcagaagaagcaagattaaaaagagaagaaataagcggagtgaaattagaatcaataggaacttaccagatactgtcaatttattcaacagcggcgagttccctagcactggcaatcatgatagctggtctatctttatggatgtgctccaatgggtcgttacagtgcagaatttgcatt

>H5N1_A_hen_Bulgaria_722_1_22VIR778_1_2021

atggagaacatagtacttcttcttgcaacagttagccttgttaaaagtgatcagatttgcattggttaccatgcaaacaattcgacagagcaggttgacacgataatggaaaagaacgtcactgttacacatgcccaagacatactggaaaaaacacacaacgggaagctctgtgatttaaatggggtgaagcctctgattttaaaggattgtagtgtagctggatggctcctcggaaacccaatgtgcgacgaattcatcagggtgccggaatggtcctacatagtggagcgggctaatccagccaatgacctctgttacccagggagcctcaatgactatgaagaactgaaacacctgttgagcagaataaatcattttgagaagattctgatcatccccaagagttcctggccaaatcatgaaacatcactaggggtgagcgcagcttgtccataccagggagcgccctcctttttcagaaatgtggtgtggcttgtcaaaaagaacgatgcatacccaacaataaagataagctacaataatacaaatcgggaagatctcttgatactgtgggggattcatcattccaacaatgcagaagagcagacaaatctctataaaaacccaaccacctacatttcagttggaacatcaactttaaaccagaggttggtaccaaaaatagctactagatcccaagtaaacgggcaacgtggaagaatggacttcttctggacaattttaaaaccagatgatgcaatccatttcgagagtaatggaaatttcattgcaccagaatatgcatataaaattgtcaagaaaggggactcaacaattatgaaaagtggagtggaatatggccactgcaacaccaaatgtcaaaccccagtaggagcgataaattctagtatgccattccacaacatacatcctctcaccattggggaatgccccaaatacgtgaagtcaaacaagttggtccttgcgactgggctcagaaatagtcctctaagagaaGGGaagagaagaaaaagaggcctgtttggggcgatagcagggtttatagagggaggatggcagggaatggttgatggttggtatgggtaccatcatagcaatgagcaggggagtgggtacgctgcagacaaagaatccacccaaaaggcaatagatggagttaccaataaggtcaactcaatcattgacaaaatgaacactcaatttgaggcagttggaagggagtttaataacttagaaaggaggatagagaatttgaacaagaaaatggaagacggattcctagatgtctggacctataatgctgaacttctagttctcatggaaaacgagaggactctagatttccatgattcaaatgtcaagaacctttacgacaaagtcagactacagctcagggataatgcaaaggagctgggtaatggctgtttcgaattctatcacaaatgcgataatgaatgtatggaaagtgtgaaaaatgggacgtatgactaccctcattattcagaagaagcaagattaaaaagagaagaaataagcggagtgaaattagaatcaataggaacttaccagatactgtcaatttattcaacagcggcgagttccctagcactggcaatcatgatagctggtctatctttatggatgtgctccaatgggtcgttacagtgcagaatttgcatt

>H5N1_A_swan_Romania_10455_22VIR2749_4_2022

atggagaacatagtactacttcttgcaatagttagccttgttaaaagtgatcagatttgcattggttaccatgcaaacaattcgacagagcaggttgacacgataatggaaaagaacgtcactgttacacatgcccaagacatactggaaaaaacacacaacgggaagctctgtgatttaaatggggtgaagcctctgattttaaaggattgtagtgtagctggatggctcctcggaaacccaatgtgcgacgaattcaycagagtgccggaatggtcctacatagtggagcgggctaatccagccaatgacctctgttacccagggagtctcaatgactatgaagaactgaaacacctgttgagcagaataaatcattttgagaagattctgatcatccccaagagttcttggccaaatcatgaaacatcactaggggtgagcgcagcttgtccataccagggagcgccctcctttttcagaaatgtggtgtggcttatcaaaaagaacgatgcatacccaacaataaagataagctacaataataccaatcgggaagatctcttgatactgtgggggattcatcattccaacaatgcagaagagcagacaaatctctataaaaacccaaccacctacatttcagttggaacatcaactttaaaccagaggttggtaccaaaaatagctactagatcccaagtaaacgggcaacgtggaagaatggacttcttctggacaattttaaaaccagatgatgcaatccatttcgagagtaatggaaatttcattgttccagaatatgcatataaaattgtcaagaaaggggactcaacaattatgaaaagtggagtggaatatggccactgcaacaccaaatgtcaaaccccagtaggagcgataaattctagtatgccattccacaacatacatcctctcaccattggggaatgccccaaatacgtgaagtcaaacaagttggtccttgcgactgggctcagaaatagtcctctaagagaaGGGaaaagaagaaaaagaggcctgtttggggcgatagcagggtttatagagggaggatggcagggaatggttgatggttggtatgggtaccatcatagcaatgagcaggggagtgggtacgctgcagacaaagaatccacccaaaaggcaatagatggagttaccaataaggtcaactcaatcattgacaaaatgaacactcaatttgaggcagttggaagggagtttaataacttagaaaggaggatagagaatttgaacaagaaaatggaagacggattcctagatgtctggacctataatgctgaacttctagttctcatggaaaacgagaggactctagatttccatgattcaaatgtaaagaacctttacgacaaagtcagactacagcttagggataatgcaaaggagctgggtaatggctgttttgaattctatcacaaatgcgataatgaatgtatggaaagtgtgagaaatgggacgtatgactaccctcagtattcagaagaagcaagattaaaaagagaagaaataagcggagtgaaattagaatcaataggaacttaccagatactgtcaatttattcaacagcggcgagttccctagcactggcaatcatgatagctggtctatctttatggatgtgctccaatgggtcgttacagtgcagaatttgcatt

>H5N1_A_turkey_Spain_455_96_22VIR2142_24_2022

atggagaacatagtacttcttcttgcaatagttagccttgttaaaagtgatcagatttgcattggttaccatgcaaacaattcgacagagaaggttgacacgataatggaaaagaacgtcactgttacacatgcccaagacatactggaaaaaacacacaacgggaagctctgtgatttaaatggggtgaagcctctgattttgaaggattgtagtgtagctggatggctcctcggaaacccaatgtgcgacgaattcatcagagtgccggaatggtcctacatagtggagcgggctaatccagctaatgacctctgttacccagggagcctcaatgactatgaagaactgaaacacctgttgagcagaataaaccattttgagaagattctgatcatccccaagagttcctggccaaatcatgaaacatcactaggggtgagcgcagcttgtccataccagggagcgccctcctttttcagaaatgtggtgtggcttatcaaaaagaacgatgcatacccaacaataaagataagctacaataataccaatcgggaagatctcttgatactgtgggggattcatcattccaacaatgcagaagaacagacaaatctctataaaaacccaaccacctacatttcagttggaacatcaactttgaaccagaggttggtaccaaaaatagctactagatcccaagtaaacgggcaacgtggaagaatggacttcttctggacaattttaaaaccagatgatgcaatccatttcgagagtaatggaaatttcattgctccagaatatgcatataaaattgtcaagaaaggggactcaacaattatgaaaagtggagtggaatatggccactgcaacaccaaatgtcaaaccccagtaggagcgataaattctagtatgccattccacaacatacatcctctcaccattggggaatgccccaaatacgtgaagtcaaacaagttggtccttgcgactgggctcagaaatagtcctctaagagaaGGGaagagaagaaaaagaggcctgtttggggcgatagcagggtttatagagggaggatggcagggaatggttgatggttggtatgggtaccatcatagcaatgagcaggggagtgggtacgctgcagacaaagaatccacccaaaaggcaatagatggagttaccaataaggtcaactcaatcattgacaaaatgaacactcaatttgaggcagttggaagggagtttaataacttagaaaggaggatagagaatttgaacaagaaaatggaagacggattcctagatgtctggacctataatgccgaacttctagttctcatggaaaacgagaggactctagatttccatgattcaaatgtcaagaacctttacgacaaagtcagactacagcttagggataatgcaaaggagctgggtaatggctgtttcgaattctatcacaaatgcgataatgaatgtatggaaagtgtgagaaatgggacgtatgactaccctcagtattcagaagaagcaagatcaaaaagagaagaaataagcggagtgaaattagaatcaataggaacttaccagatactgtcaatttattcaacagcggcgagttccctagcactggcaatcatgatagctggtctatctttatggatgtgctccaatgggtcgttacagtgcagaatttgcatt

>H5N1_A_guineafowl_Scotland_054471_2021

atggagaacatagtacttcttcttgcaatagttagccttgttaaaagtgatcagatttgcattggttaccatgcaaacaattcgacagagaaggttgacacgataatggaaaagaacgtcactgttacacatgcccaagacatactggaaaaaacacacaacgggaagctctgtgatttaaatggggtgaagcctctgattttaaaggattgtagtgtagctggatggctcctcggaaacccaatgtgcgacgaattcatcagagtgccggaatggtcctacatagtggagcgggctaatccagctaatgacctctgttacccagggagcctcaatgactatgaagaactgaaacacctgttgagcagaataaaccattttgagaagattctgatcatccccaagagttcctggccaaatcatgaaacatcactaggggtgagcgcagcttgtccataccagggagcgccctcctttttcagaaatgtggtgtggcttatcaaaaagaacgatgcatacccaacaataaagataagctacaataataccaatcgggaagatctcttgatactgtgggggattcatcattccaacaatgcagaagaacagacaaatctctataaaaacccaaccacctacatttcagttggaacatcaactttaaaccagaggttggtaccaaaaatagctactagatcccaagtaaacgggcaacgtggaagaatggacttcttctggacaattttaaaaccagatgatgcaatccatttcgagagtaatggaaatttcattgctccagaatatgcatataaaattgtcaagaaaggggactcaacaattatgaaaagtggagtggaatatggccactgcaacaccaaatgtcaaaccccagtaggagcgataaattctagtatgccattccacaacatacatcctctcaccattggggaatgccccaaatacgtgaagtcaaacaagttggtccttgcgactgggctcagaaatagtcctctaagagaaGGGaagagaagaaaaagaggcctgtttggggcgatagcagggtttatagagggaggatggcagggaatggttgatggttggtatgggtaccatcatagcaatgagcaggggagtgggtacgctgcagacaaagaatccacccaaaaggcaatagatggagttaccaataaggtcaactcaatcattgacaaaatgaacactcaatttgaggcagttggaagggagtttaataacttagaaaggaggatagagaatttgaacaagaaaatggaagacggattcctagatgtctggacctataatgctgaacttctagttctcatggaaaacgagaggactctagatttccatgattcaaatgtcaagaacctttacgacaaagtcagactacagcttagggataatgcaaaggagctgggtaatggctgtttcgaattctatcacaaatgcgataatgaatgtatggaaagtgtgagaaatgggacgtatgactaccctcagtattcagaagaagcaagattaaaaagagaagaaataagcggagtgaaattagaatcaataggaacttaccagatactgtcaatttattcaacagcggcgagttccctagcactggcaatcatgatagctggtctatctttatggatgtgctccaatgggtcgttacagtgcagaatttgcatt

>H5N1_A_goose_France_21P013228_2021

atggagaacatagtacttcttcttgcaatagttagccttgtcaaaagtgatcagatttgcattggttaccatgcaaacaattcgacagagaaggttgacacgataatggaaaagaacgtcactgttacacatgcccaagacatactggaaaaaacacacaacgggaagctctgtgatttaaatggggtgaagcctctgattttaaaggattgtagtgtagctggatggctcctcggaaacccaatgtgcgacgaattcatcagagtgccggaatggtcctacatagtggagcgggctaatccagctaatgacctctgttacccagggagcctcaatgactatgaagaactgaaacacctgttgagcagaataaaccattttgagaagattctgatcatccccaagagttcctggccaaatcatgaaacatcactaggggtgagcgcagcttgtccataccagggagcgccctcctttttcagaaatgtggtgtggcttatcaaaaagaacgatgcatacccaacaataaagataagctacaataataccaatcgggaagatctcttgatactgtgggggattcatcattccaacaatgcagaagaacagacaaatctctataaaaacccaaccacctacatttcagttggaacatcaactttaaaccagaggttggtaccaaaaatagctactagatcccaagtaaacgggcaacgtggaagaatggacttcttctggacaattttaaaaccagatgatgcaatccatttcgagagtaatggaaatttcattgctccagaatatgcatataaaattgtcaagaaaggggactcaacaattatgaaaagtggagtggaatatggccactgcaacaccaaatgtcaaaccccagtaggagcgataaattctagtatgccattccacaacatacatcctctcaccattggggaatgccccaaatacgtgaagtcaaacaagttggtccttgcgactgggctcagaaatagtcctctaagagaaGGGaagagaagaaaaagaggcctgtttggggcgatagcagggtttatagagggaggatggcagggaatggttgatggttggtatgggtaccatcatagcaatgagcaggggagtgggtacgctgcagacaaagaatccacccaaaaggcaatagatggagttaccaataaggtcaactcaatcattgacaaaatgaacactcaatttgaggcagttggaagggagtttaataacttagaaaggaggatagagaatttgaacaagaaaatggaagacggattcctagatgtctggacctataatgctgaacttctagttctcatggaaaacgagaggactctagatttccatgattcaaatgtcaagaacctttacgacaaagtcagactacagcttagggataatgcaaaggagctgggtaatggctgtttcgaattctatcacaaatgcgataatgaatgtatggaaagtgtgagaaatgggacgtatgactaccctcagtattcagaagaagcaagattaaaaagagaagaaataagcggagtgaaattagaatcaataggaacttaccagatactgtcaatttattcaacagcggcgagttccctagcactggcaatcatgatagctggtctatctttatggatgtgctccaatgggtcgttacagtgcagaatttgcatt

>H5N1_A_Mallard_Netherlands_4_2021

atggagaacatagtacttcttcttgcaatagttagccttgttaaaagtgatcagatttgcattggttaccatgcaaacaattcaacagagaaggttgacacgataatggaaaagaacgtcactgttacacatgcccaagacatactggaaaaaacacacaacgggaagctctgtgatttaaatggggtgaagcctctgattttaaaggattgtagtgtagctggatggctcctcggaaacccaatgtgcgacgaattcatcagagtgccggaatggtcctacatagtggagcgggctaatccagctaatgacctctgttacccagggagcctcaatgactatgaagaactgaaacacctgttgagcagaataaaccattttgagaagattctgatcatccccaagagttcctggccaaatcatgaaacatcactaggggtgagcgcagcttgtccataccagggagcgccctccttcttcagaaatgtggtgtggcttatcaaaaagaacgatgcatacccaacaataaagataagctacaataataccaatcgggaagatctcttgatactgtgggggattcatcattccaacaatgcagaagaacagacaaatctctataaaaacccaaccacctacatttcagttggaacatcaactttaaaccagaggttggtaccaaaaatagctactagatcccaagtaaacgggcaacgtggaagaatggacttcttctggacaattttaaaaccagatgatgcaatccatttcgagagtaatggaaatttcattgctccagaatatgcatataaaattgtcaagaaaggggactcaacaattatgaaaagtggagtggaatatggccactgcaacaccaaatgtcaaaccccagtaggagcgataaattctagtatgccattccacaacatacatcctctcaccattggggaatgccccaaatacgtgaagtcaaacaagttggtccttgcgactgggctcagaaatagtcctctaagagaaGGGaagagaagaaaaagaggcctgtttggggcgatagcagggtttatagagggaggatggcagggaatggttgatggttggtatgggtatcatcatagcaatgagcaggggagtgggtacgctgcagacaaagaatccacccaaaaggcaatagatggagttaccaataaggtcaactcaatcattgacaaaatgaacactcaatttgaggcagttggaagggagtttaataacttagaaaggaggatagagaatttgaacaagaaaatggaagacggattcctggatgtctggacctataatgctgaacttctagttctcatggaaaacgagaggactctagatttccatgattcaaatgtcaagaacctttacgacaaagtcagactacagcttagggataatgcaaaggagctgggtaatggctgtttcgaattctatcacaaatgcgataatgaatgtatggaaagtgtgagaaatgggacgtatgactaccctcagtattcagaagaagcaagattaaaaagagaagaaataagcggagtgaaattagaatcaataggaacttaccagatactgtcaatttattcaacagcggcgagttccctagcactggcaatcatgatagctggtctatctttatggatgtgctccaatgggtcgttacagtgcagaatttgcatt

>H5N1_A_greylag_goose_Denmark_24343_1_02_2021

atggagaacatagtacttcttcttgcaatagttagccttgttaaaagtgatcagatttgcattggttaccatgcaaacaattcgacagagaaggttgacacgataatggaaaagaacgtcactgttacacatgcccaagacatactggaaaaaacacacaacgggaagctctgtgatttaaatggggtgaagcctctgattttaaaggattgtagtgtagctggatggctcctcggaaacccaatgtgcgatgaattcatcagagtgccggaatggtcctacatagtggagcgggctaatccagctaatgacctctgttacccagggagcctcaatgactatgaagaactgaaacacctgttgagcagaataaaccattttgagaagattctgatcatccccaagagttcctggccaaatcatgaaacatcactaggggtgagcgcagcttgtccataccagggagcgccctcctttttcagaaatgtggtgtggcttatcaaaaagaacgatgcatacccaacaataaagataagctacaataataccaatcgggaagatctcttgatactgtgggggattcatcattccaacaatgcagaagaacagacaaatctctataaaaacccaaccacctacatttcagttggaacatcaactttaaaccagaggttggtaccaaaaatagctactagatcccaagtaaacgggcaacgcggaagaatggacttcttctggacaattttaaaaccagatgatgcaatccatttcgagagtaatggaaatttcattgctccagaatatgcatataaaattgtcaagaaaggggactcaacaattatgaaaagtggagtggaatatggccactgcaacaccaaatgtcaaaccccagtaggagcgataaattctagtatgccattccacaacatacatcctctcaccattggggaatgccccaaatacgtgaagtcaaacaagttggtccttgcgactgggctcagaaatagtcctctaagagaaGGGaagagaagaaaaagaggcctgtttggggcgatagcagggtttatagagggaggatggcagggaatggttgatggttggtatgggtaccatcatagcaatgagcaggggagtgggtacgctgcagacaaagaatccacccaaaaggcaatagatggagttaccaataaggtcaactcaatcattgacaaaatgaacactcaatttgaggcagttggaagggagtttaataacttagaaaggaggatagagaatttgaacaagaaaatggaagacggattcctagatgtctggacctataatgctgaacttctagttctcatggaaaacgagaggactctagatttccatgattcaaatgtcaagaacctttacgacaaagtcagactacagcttagggataatgcaaaggagctgggtaatggctgtttcgaattctatcacaaatgcgataatgaatgtatggaaagtgtgagaaatgggacgtatgactaccctcagtattcagaagaagcaagattaaaaagagaagaaataagcggagtgaaattagaatcaataggaacttaccagatactgtcaatttattcaacagcggcgagttccctagcactggcaatcatgatagctggtctatctttatggatgtgctccaatgggtcgttacagtgcagaatttgcatt

>H5N1_A_mute_swan_Croatia_101_2021

atggagaacatagtacttcttcttgcaatagttagccttgttaaaagtgatcagatttgcattggttaccatgcaaacaattcgacagagcaggttgacacgataatggaaaagaacgtcactgttacacatgcccaagacatactggaaaaaacacacaacgggaagctctgtgatttaaatggggtgaagcctctgatcttaaaggattgtagtgtagctggatggctcctcggaaacccaatgtgcgacgaattcatcagagtgccggaatggtcctacatagtggagcgggctaatccagctaatgacctctgttacccagggagcctcaatgactatgaagaactgaaacacctgttgagcagaataaatcattttgagaagattctgatcatccccaagagttcctggccaaatcatgaaacatcactaggggtgagcgcagcttgtccataccagggagctccctcctttttcagaaatgtggtgtggcttatcaaaaagaacgatgcatacccaacaataaagataagctacaataataccaatcgggaagatctcttgatactgtgggggattcatcattccaacaatgcagaagaacagacaaatctctataaaaacccaaccacctacatttcagttggaacatcaactttaaaccagaggttggtaccaaaaatagctactagatcccaagtaaacgggcaacgtggaagaatggacttcttctggacaattttaaaaccagatgatgcaatccatttcgagagtaatggaaatttcattgctccagaatatgcatataaaattgtcaagaaaggggactcaacaattatgaaaagtggagtggaatatggccactgcaacaccaaatgtcaaaccccagtaggagcgataaattctagtatgccattccacaacatacatcctctcaccattggggaatgccccaaatacgtgaagtcaaacaagttggtccttgcgactgggctcagaaatagtcctctaagagaaGGGaagagaagaaaaagaggcctgtttggggcgatagcagggtttatagagggaggatggcagggaatggttgatggttggtatgggtaccatcatagcaatgagcaggggagtgggtacgctgcagacaaagaatccacccaaaaggcaatagatggagttaccaataaggtcaactcaatcattgacaaaatgaacactcaatttgaggcagttggaagggagtttaataacttagaaaggaggatagagaatttgaacaagaaaatggaagacggattcctagatgtctggacctataatgctgaacttctagttctcatggaaaacgagaggactctagatttccatgattcaaatgtcaagaacctttacgacaaagtcagactacagcttagggataatgcaaaggagctgggtaatggctgtttcgaattctatcacaaatgcgataatgaatgtatggaaagtgtgagaaatgggacgtatgactaccctcagtattcagaagaagcaagattaaaaagagaagaaataagcggagtgaaattagaatcaataggaacttaccagatactgtcaatttattcaacagcggcgagttccctagcactggcaatcatgatagctggtctatctttatggatgtgctccaatgggtcgttacagtgcagaatttgcatt

>H5N1_A_Gallus_gallus_Belgium_3194_0001_2022

atggagaacatagtacttcttcttgcaatagttagccttgttaaaagtgatcagatttgcattggttaccatgcaaacaattcgacagagcaggttgacacgataatggaaaagaacgtcactgttacacatgcccaagacatactggaaaaaacacacaacgggaagctctgtgatttaaatggggtgaagcctctgattttaaaggattgtagtgtagctggatggctcctcggaaacccaatgtgcgacgaattcatcagagtgccggaatggtcctacatagtggagcgggctaatccagctaatgacctctgttacccagggagcctcaatgactatgaagaactgaaacacctgttgagcagaataaatcattttgagaagattcttatcatccccaagagttcctggccaaatcatgaaacatcactaggggtgagcgcagcttgtccataccagggagcgccctcctttttcagaaatgtggtgtggcttatcaaaaagaacgatgcatatccaacaataaagataagctacaataataccaatcgggaagatctcttgatactgtgggggattcatcattccaacaatgcagaagaacagacaaatctctataaaaatccaaccacctacatttcagttggaacatcaactttaaaccagaggttggtaccaaaaatagctactagatcccaagtaaacgggcaacgtggaagaatggacttcttctggacaattttaaaaccagatgatgcaatccatttcgagagtaatggaaatttcattgctccagaatatgcatataaaattgtcaagaaaggggactcaacaattatgaaaagtggagtggaatatggccactgcaacaccaaatgtcaaaccccagtaggagcgataaattctagtatgccattccacaacatacatcctctcaccattggggaatgccccaaatacgtgaagtcaaacaagttggtccttgcgactgggctcagaaatagtcctctaagagaaGGGaagagaagaaaaagaggcctgtttggggcgatagcagggtttatagagggaggatggcagggaatggttgatggttggtatgggtaccatcatagcaatgagcaggggagtgggtacgctgcagacaaagaatccacccaaaaggcaatagatggagttaccaataaggtcaactcaatcattgacaaaatgaacactcaatttgaggcagttggaagggagtttaataacttagaaaggaggatagagaatttgaacaagaaaatggaagacggattcctagatgtctggacctataatgctgaacttctagttctcatggaaaacgagaggactctagatttccatgattcaaatgttaagaacctttacgacaaagtcagaatacagcttagggataatgcaaaggagctgggtaatggctgtttcgaattctatcacaaatgcgataatgaatgtatggaaagtgtgagaaatgggacgtatgactaccctcagtattcagaagaagcaagattaaagagagaagaaataagcggagtgaaattagaatcaataggaacttaccagatactgtcaatttattcaacagcggcgagttccctagcactggcaatcatgatagctggtctatctttatggatgtgctccaatgggtcgttacagtgcagaatttgcatt

>H5N1_A_duck_Saratov_29_03V_2021

atggagaacatagtacttcttcttgcaatagttagccttgttaaaagtgatcagatttgcattggttaccatgcaaacaattcgacagagcaggttgacacgataatggaaaagaacgtcactgttacacatgcccaagacatactggaaaaaacacacaacgggaagctctgtgatttaaatggggtgaagcctctgattttaaaggattgtagtgtagctggatggctcctcggaaacccaatgtgcgacgaattcatcagagtgccggaatggtcctacatagtggagcgggctaatccagctaatgacctctgttacccagggagcctcaatgactatgaagaactgaaacacctgttgagcagaataaatcattttgagaagattctgatcatccccaagagttcctggccaaatcatgaaacatcactaggggtgagcgcagcttgtccataccagggagcgccctcctttttcagaaatgtggtgtggcttatcaaaaagaacgatgcatacccaacaataaagataagctacaataataccaatcgggaagatctcttgatactgtgggggattcatcattccaacaatgcagaagaacagacaaatctctataaaaacccaaccacctacatttcagttggaacatcaactttaaaccagaggttggtaccaaaaatagctactagatcccaagtaaacgggcaacgtggaagaatggacttcttctggacaattttaaaaccagatgatgcaatccatttcgagagtaatggaaatttcattgctccagaatatgcatataaaattgtcaagaaaggggactcaacaattatgaaaagtggagtggaatatggccactgcaacaccaaatgtcaaaccccagtaggagcgataaattctagtatgccattccacaacatacatcctctcaccattggggaatgccccaaatacgtgaagtcaaacaaattggtccttgcgactgggctcagaaatagtcctctaagagaaGGGaagagaagaaaaagaggcctgtttggggcgatagcagggtttatagagggaggatggcagggaatggttgatggttggtatgggtaccatcatagcaatgagcaggggagtgggtacgctgcagacaaagaatccacccaaaaggcaatagatggagttaccaataaggtcaactcaatcattgacaaaatgaacactcaatttgaggcagttggaagggagtttaataatttagaaaggaggatagagaatttgaacaagaaaatggaagacggattcctagatgtctggacctataatgctgaacttctagttctcatggaaaacgagaggactctagatttccatgattcaaatgtcaagaacctttacgacaaagtcagactacagcttagggataatgcaaaggagctgggtaatggctgtttcgaattctatcacaaatgcgataatgaatgtatggaaagtgtgagaaatgggacgtatgactaccctcagtattcagaagaagcaagattaaaaagagaagaaataagcggagtgaaattagaatcaataggaacttaccagatactgtcaatttattcaacagcggcgagttccctagcactggcaatcatgatagctggtctatctttatggatgtgctccaatgggtcgttacagtgcagaatttgcatt

>H5N3_A_Mallard_Netherlands_17_2021

atggagaacatagtacttcttcttgcaatagttagccttgttaaaagtgatcagatttgcattggttaccatgcaaacaattcgacagagcaggttgacacgataatggaaaagaacgtcactgttacacatgcccaagacatactggaaaaaacacacaacgggaagctctgtgatttaaatggggtgaagcctctgattttaaaggattgtagtgtagctggatggctcctcggaaacccaatgtgcgacgaattcatcagagtgccggaatggtcctacatagtggagcgggctaatccagctaatgacctctgttacccagggagcctcaatgactatgaagaactgaaacacctgttgagcagaataaatcattttgagaagattctgatcatccccaagagttcctggccaaatcatgaaacatcactaggggtgagcgcagcttgtccataccagggagcgccctcctttttcagaaatgtggtgtggcttatcaaaaagaacgatgcatacccaacaataaagataagctacaataataccaatcgggaagatctcttgatactgtgggggattcatcattccaacaatgcagaagagcagacaaatctctataaaaacccaaccacctacatttcagttggaacatcaactttaaaccagaggttggtaccaaaaatagctactagatcccaagtaaacgggcaacgtggaagaatggacttcttctggacaattttaaaaccagatgatgcaatccatttcgagagtaatggaaatttcattgctccagaatatgcatataaaattgtcaagaaaggggactcaacaattatgaaaagtggagtggaatatggccactgcaacaccaaatgtcaaaccccagtaggagcgataaattctagtatgccattccacaacatacatcctctcaccattggggaatgccccaaatacgtgaagtcaaacaagttggtccttgcgactgggctcagaaatagtcctctaagagaaGGGaagagaagaaaaagaggcctgtttggggcgatagcagggtttatagagggaggatggcagggaatggttgatggttggtatgggtaccatcatagcaatgagcaggggagtgggtacgctgcagacaaagaatccacccaaaaggcaatagatggagttaccaataaggtcaactcaatcattgacaaaatgaacactcaatttgaggcagttggaagggagtttaataacttagaaaggaggatagagaatttgaacaagaaaatggaagacggattcctagatgtctggacctataatgctgaacttctagttctcatggaaaacgagaggactctagatttccatgattcaaatgtcaagaacctttacgacaaagtcagactacagcttagggataatgcaaaggagctgggtaatggctgtttcgaattctatcacaaatgcgataatgaatgtatggaaagtgtgagaaatgggacgtatgactaccctcagtattcagaagaagcaagattaaaaagagaagaaataagcggagtgaaattagaatcaataggaacttaccagatactgtcaatttattcaacagcggcgagttccctagcactggcaatcatgatagctggtctatctttatggatgtgctccaatgggtcgttacagtgcagaatttgcatt

>H5N1_A_duck_Italy_21VIR10447_2021

atggagaacatagtacttcttcttgcaatagttagccttgttaaaagtgatcagatttgcattggttaccatgcaaacaattcgacagagcaggttgacacgataatggaaaagaacgtcactgttacacatgcccaagacatactggaaaaaacacacaacgggaagctctgtgatttaaatggggtgaagcctctgattttaaaggattgtagtgtagctggatggctcctcggaaacccaatgtgcgacgaattcatcagagtgccggaatggtcctacatagtggagcgggctaatccagctaatgacctctgttacccagggagcctcaatgactatgaagaactgaaacacctgttgagcagaataaatcattttgagaagattctgatcatccccaagagttcctggccaaatcatgaaacatcactaggggtgagcgcagcttgtccataccagggagcgccctcctttttcagaaatgtggtgtggcttatcaaaaagaacgatgcatacccaacaataaagataagctacaataataccaatcgggaagatctcttgatactgtgggggattcatcattccaacaatgcagaagagcagacaaatctctataaaaacccaaccacctacatttcagttggaacatcaactttaaaccagaggttggtaccaaaaatagctactagatcccaagtaaacgggcaacgtggaagaatggacttcttctggacaattttaaaaccagatgatgcaatccatttcgagagtaatggaaatttcattgctccagaatatgcatataaaattgtcaagaaaggggactcaacaattatgaaaagtggagtggaatatggccactgcaacaccaaatgtcaaaccccagtaggagcgataaattctagtatgccattccacaacatacatcctctcaccattggggaatgccccaaatacgtgaagtcaaacaagttggtccttgcgactgggctcagaaatagtcctctaagagaaGGGaagagaagaaaaagaggcctgtttggggcgatagcagggtttatagagggaggatggcagggaatggttgatggttggtatgggtaccatcatagcaatgagcaggggagtgggtacgctgcagacaaagaatccacccaaaaggcaatagatggagttaccaataaggtcaactcaatcattgacaaaatgaacactcaatttgaggcagttggaagggagtttaataacttagaaaggaggatagaaaatttgaacaagaaaatggaagacggattcctagatgtctggacctataatgctgaacttctagttctcatggaaaacgagaggactctagatttccatgattcgaatgtcaagaacctttacgacaaagtcagactacagcttagggataatgcaaaggagctgggtaatggctgtttcgaattctatcacaaatgcgataatgaatgtatggaaagtgtgagaaatgggacgtatgactaccctcagtattcagaagaagcaagattaaaaagagaagaaataagcggagtgaaattagaatcaataggaacttaccagatactgtcaatttattcaacagcggcgagttccctagcactggcaatcatgatagctggtctatctttatggatgtgctccaatgggtcgttacagtgcagaatttgcatt

>H5N1_A_chicken_Poland_H1940_N_2021

atggagaacatagtacttcttcttgcaatagttagccttgttaaaagtgatcagatttgcattggttaccatgcaaacaattcgacagagcaggttgacacgataatggaaaagaacgtcactgttacacatgcccaagacatactggaaaagacacacaacgggaagctctgtgatttaaatggggtgaagcctctgattttaaaggattgtagtgtagctggatggctcctcggaaacccaatgtgcgacgaattcatcagagtgccggaatggtcctacatagtggagcgggctaatccagctaatgacctctgttacccagggagcctcaatgactatgaagaactgaaacacctgttgagcagaataaatcattttgagaagattctgatcatccccaagagttcctggccaaatcatgaaacatcactaggggtgagcgcagcttgtccataccagggagcgccctcctttttcagaaatgtggtgtggcttatcaaaaagaacgatgcatacccaacaataaagataagctacaataataccaatcgggaagatctcttgatactgtgggggattcatcattccaacaatgcagaagagcagacaaatctctataaaaacccaaccacctacatttcagttggaacatcaactttaaaccagaggttggtaccaaaaatagctactagatcccaagtaaacgggcaacgtggaagaatggacttcttctggacaattttaaaaccagatgatgcaatccatttcgagagtaatggaaatttcattgctccagaatatgcatataaaattgtcaagaaaggggactcaacaattatgaaaagtggagtggaatatggccactgcaacaccaaatgtcaaaccccagtaggagcgataaattctagtatgccattccacaacatacatcctctcaccattggggaatgccccaaatacgtgaagtcaaacaagttggtccttgcgactgggctcagaaatagtcctctaagagaaGGGaagagaagaaaaagaggcctgtttggggcgatagcagggtttatagagggaggatggcagggaatggttgatggttggtatgggtaccatcatagcaatgagcaggggagtgggtacgctgcagacaaagaatccacccaaaaggcaatagatggagttaccaataaggtcaactcaatcattgacaaaatgaacactcaatttgaggcagttggaagggagtttaataatttagaaaggaggatagagaatttgaacaagaaaatggaagacggattcctagatgtctggacctataatgctgaacttctagttctcatggaaaacgagaggactctagatttccatgattcaaatgtcaagaacctttacgacaaagtcagactacagcttaaggataatgcaaaggagctgggtaatggctgtttcgaattctatcacaaatgcgataatgaatgtatggaaagtgtgagaaatgggacgtatgactaccctcagtattcagaagaagcaagattaaaaagagaagaaataagcggagtgaaattagaatcaataggaacttaccagatactgtcaatttattcaacagcggcgagttccctagcactggcaatcatgatagctggtctatctttatggatgtgctccaatgggtcgttacagtgcagaatttgcatt

>H5N1_A_swan_Slovenia_2049_22VIR777_3_2021

atggagaacatagtacttcttcttgcaatagttagccttgttaaaagtgatcagatttgcattggttaccatgcaaacaattcgacagagcaggttgacacgataatggaaaagaacgtcactgttacacatgcccaagacatactggaaaaaacacacaacgggaagctctgtgatttaaatggggtgaaacctctgattttaaaggattgtagtgtagctggatggctcctcggaaacccaatgtgcgacgaattcatcagagtgccggaatggtcctacatagtggagcgggctaatccagctaatgacctctgttacccagggagcctcaatgactatgaagaactgaaacacctgttgagcagaataaatcattttgagaagattctgatcatccccaagagttcctggccaaatcatgaaacatcactaggggtgagcgcagcttgtccataccagggagcgccctcctttttcagaaatgtggtgtggcttatcaaaaagaacgatgcatacccaacaataaagataagctacaataataccaatcgggaagatctcttgatactttgggggattcatcattccaacaatgcagaagagcagacaaatctctataaaaacccaaccacctacatttcagttggaacatcaactttaaaccagaggttggtaccaaaaatagctactagaycccaagtaaacgggcaacgtggaagaatggacttcttctggacaattttaaaaccagatgatgcaatccatttcgagagtaatggaaatttcattgctccagaatatgcatataaaattgtcaagaaaggggactcaacaattatgaaaagtggagtggaatatggccactgcaacaccaaatgtcaaaccccagtaggagcgataaattctagtatgccattccacaacatacatcctctcaccattggggaatgccccaaatacgtgaagtcaaacaagttggtccttgcgactgggctcagaaatagtcctctaagagaaGGGaagagaagaaaaagaggcctgtttggggcgatagcagggtttatagagggaggatggcagggaatggttgatggttggtatgggtaccatcatagcaatgagcaggggagtgggtacgctgcagacaaagaatccacccaaaaggcaatagatggagttaccaataaggtcaactcaatcattgacaaaatgaacactcaatttgaggcagttggaagggagtttaataacttagaaaggaggatagagaatttgaacaagaaaatggaagacggattcctagacgtctggacctataatgctgaacttctagttctcatggaaaacgagaggactctagatttccatgattcaaatgtcaagaacctttacgacaaagtcagactacagcttagggataatgcaaaggagctgggtaatggctgtttcgaattctatcacaaatgcgataatgaatgtatggaaagtgtgagaaatgggacgtatgactaccctcagtattcagaagaagcaagattaaaaagagaagaaataagcggagtgaaattagaatcaataggaacttatcagatactgtcaatttattcaacagcggcgagttccctagcactggcaatcatgatagctggtctatctttatggatgtgctccaatgggtcgttacagtgcagaatttgcatt

>H5N1_A_chicken_Czech_Republic_61_1_2021

atggagaacatagtacttcttcttgcaatagttagccttgttaaaagtgatcagatttgcattggttaccatgcaaacaattcgacagagcaggttgacacgataatggaaaagaacgtcactgttacacatgcccaagacatactggaaaaaacacacaacgggaagctctgtgatttaaatggggtgaagcctctgattttaaaggattgtagtgtagctggatggctcctcggaaacccaatgtgcgacgaattcatcagagtgccggaatggtcctacatagtggagcgggctaatccagctaatgacctctgttacccagggagcctcaatgactatgaagaactgaaacacctgttgagcagaataaatcattttgagaagattctgatcatccccaagagttcctggccaaatcatgaaacatcactaggggtgagcgcagcttgtccttaccagggagcgccctcctttttcagaaatgtggtgtggcttatcaaaaagaacgatgcatacccaacaataaagataagctacaataataccaatcgggaagatctcttgatactgtgggggattcatcattccaacaatgcagaagagcagacaaatctctataaaaacccaaccacctacatttcagttggaacatcaactttaaaccagaggttggtaccaaaaatagctactagatcccaagtaaacgggcaacgtggaagaatggacttcttctggacaattttaaaaccagatgatgcaatccatttcgagagtaatggaaatttcgttgctccagaatatgcatataaaattgtcaagaaaggggactcaacaattatgaaaagtggagtggaatatggccactgcaacaccaaatgtcaaaccccagtaggagcgataaattctagtatgccattccacaacatacatcctctcaccattggggaatgccccaaatacgtgaagtcaaacaagttggtccttgcgactgggctcagaaatagtcctctaagagaaGGGaagagaagaaaaagaggcctgtttggggcgatagcagggtttatagagggaggatggcagggaatggttgatggttggtatgggtaccatcatagcaatgagcaggggagtgggtacgctgcagacaaagaatccacccaaaaggcaatagatggagttaccaataaggtcaactcaatcattgacaaaatgaacactcaatttgaggcagttggaagggagtttaataacttagaaaggaggatagagaatttgaacaagaaaatggaagacggattcctagatgtctggacctataatgctgaacttctagttctcatggaaaacgagaggactctagatttccatgattcaaatgtcaagaacctttacgacaaagtcagactacagcttagggataatgcaaaggagctgggtaatggctgtttcgaattctatcacaaatgcgataatgaatgtatggaaagtgtgagaaatgggacgtatgactaccctcagtattcagaagaagcaagattaaaaagagaagaaataagcggagtgaaattagaatcaataggaacttaccagatactgtcaatttattcaacagcggcgagttccctagcactggcaatcatgatagctggtctatctttatggatgtgctccaatgggtcgttacagtgcagaatttgcatt

>H5N1_A_chicken_Iowa_22_007376_001_original_2022

atggagaacatagtactacttcttgcaatagttagccttgttaaaagtgatcagatttgcattggttaccatgcaaacaattcgacagagcaagttgacacgataatggaaaagaacgtcactgttacacatgcccaagacatactggaaaaaacacacaacgggaagctctgtgatctaaatggggtgaagcctctgattttaaaggattgtagtgtagctggatggctcctcggaaacccaatgtgcgacgaattcatcagagtgccggaatggtcctacatagtggagcgggctaacccagctaatgacctctgttacccagggagcctcaatgactatgaagaactgaaacacatgttgagcagaataaatcattttgagaagattctgatcatccccaagagttcctggccaaatcatgaaacatcactaggggtgagcgcagcttgtccataccagggagcgccctcctttttcagaaatgtggtgtggcttatcaaaaagaacgatgcatacccaacaataaagataagctacaataataccaatcgggaagatctcttgatactgtgggggattcatcattccaacaatgcagaagagcagacaaatctctacaaaaacccaaccacctacatttcagttggaacatcaactttaaaccagaggttggcaccaaaaatagctactagatcccaagtaaacgggcaacgtggaagaatggacttcttctggacaatcttaaaaccagatgatgcaatccatttcgagagtaatggaaatttcattgctccagaatatgcatacaaaattgtcaagaaaggggactcaacaattatgaaaagtggagtggaatatggccactgcaacaccaaatgtcaaaccccagtaggtgcgataaattctagtatgccattccacaacatacatcctctcaccattggggaatgccccaaatacgtgaagtcaaacaagttggtccttgcgactgggctcagaaatagtcctctaagagaaGGGaagagaagaaaaagaggcctgtttggggcgatagcagggtttatagagggaggatggcagggaatggttgatggttggtatgggtaccatcatagtaatgagcaggggagtgggtacgctgcggacaaagaatccacccaaaaggcaatagatggagttaccaataaggtcaactcaatcattgacaaaatgaacactcaatttgaggcagttggaagggagtttaataacttagaaaggaggatagagaatttgaacaagaaaatggaagacggattcctagatgtctggacctataatgctgaacttctagttctcatggaaaacgagaggactctagatttccatgattcaaatgtcaagaacctttacgacaaagtcagattacagcttagggataatgcaaaggagctgggtaacggctgtttcgaattctatcacaaatgtgataatgaatgtatggaaagtgtgagaaatgggacgtatgactaccctcagtattcagaagaagcaagattaaaaagagaagaaataagcggagtgaaattagaatcagtaggaacttaccagatactgtcaatttattcaacagcggcaagttccctagcactggcaatcatgatggctggtctatctttatggatgtgctccaatgggtcgttacagtgcagaatttgcatt

>H5N1_A_chicken_Maryland_22_006578_001_2022

atgaagaacatagtactacttcttgcaatagttagccttgttaaaagtgatcagatttgcattggttaccatgcaaacaattcgacagagcaagttgacacgataatggaaaagaacgtcactgttacacatgcccaagacatactggagaaaacacacaacgggaagctctgtgatctaaatggggtgaagcctctgattttaaaggattgtagtgtagctggatggctcctcggaaacccaatgtgcgacgaattcatcagagtgccggaatggtcctacatagtggagcgggctaacccagctaatgacctctgttacccagggagcctcaatgactatgaagaactgaaacacatgttgagcagaataaatcattttgagaagattctgatcatccccaagagttcctggccaaatcatgaaacatcactaggggtgagcgcagcttgtccataccagggagcgccctcctttttcagaaatgtggtgtggcttatcaaaaagaacgatgcatacccaacaataaagataagctacaataataccaatcgggaagatctcttgatactgtgggggattcatcattccaacaatgcagaagagcagacaaatctctacaaaaacccaaccacctacatttcagttggaacatcaactttaaaccagaggttggcaccaaaaatagctactagatcccaagtaaacgggcaacgtggaagaatggacttcttctggacaatcttaaaaccagacgatgcaatccatttcgagagtaatggaaatttcattgctccagaatatgcatacaaaattgtcaagaaaggggactcaacaattatgaaaagtggagtggaatatggccactgcaacaccaaatgtcaaaccccagtaggtgcgataaattctagtatgccattccacaacatacatcctctcaccattggggaatgccccaaatacgtgaagtcaaacaagttggtccttgcgactgggctcagaaatagtcctctaagagaaGGGaggagaagaaaaagaggcctgtttggggcgatagcagggtttatagagggaggatggcagggaatggttgatggttggtatgggtaccatcatagcaatgagcaggggagtgggtacgctgcggacaaagaatccacccaaaaggcaatagatggagttaccaataaggtcaactcaatcattgacaaaatgaacactcaatttgaggcagttggaagggagtttaataacttagaaaggaggatagagaatttgaacaagaaaatggaagacggattcctagatgtctggacctataatgctgaacttctagttctcatggaaaacgagaggactctagatttccatgattcaaatgtcaagaacctttacgacaaagtcagattacagcttagggataatgcaaaggagctgggtaacggctgtttcgaattctatcacaaatgtgataatgaatgtatggaaagtgtgagaaatgggacgtatgactaccctcagtattcagaagaagcaagattaaaaagagaagaaataagcggagtgaaattagaatcagtaggaacttaccagatactgtcaatttattcaacagcggcaagttccctagcactggcaatcatgatggctggtctatctttatggatgtgctccaatgggtcgttacagtgcagaatttgcatt

>H5N1_A_turkey_Indiana_22_005328_001_2022

atggagaacatagtactacttcttgcaatagttagccttgttaaaagtgatcagatttgcattggttaccatgcaaacaattcgacagagcaagttgacacgataatggaaaagaatgtcactgttacacatgcccaagacatactggaaaaaacacacaacgggaagctctgtgatctaaatggggtgaagcctctgattttaaaggattgtagtgtagctggatggctcctcggaaacccaatgtgcgacgaattcatcagagtgccggaatggtcctacatagtggagcgggctaacccagctaatgacctctgttacccagggagcctcaatgactatgaagaactgaaacacatgttgagcagaataaatcattttgagaagattctgatcattcccaagagttcctggccaaatcatgaaacatcactaggggtgagcgcagcttgtccataccagggagcgccctcctttttcagaaatgtggtgtggcttatcaaaaagaacgatgcatacccaacaataaagataagctacaataataccaatcgggaagatctcttgatactgtgggggattcatcattccaacaatgcagaagagcagacaaatctctacaaaaacccaaccacctacatttcagttggaacatcaactttaaaccagaggttggcaccaaaaatagctactagatcccaagtaaacgggcaacgtggaagaatggacttcttctggacaatcttaaaaccagatgatgcaatccatttcgagagtaatggaaatttcattgctccagaatatgcatacaaaattgtcaagaaaggggactcaacaattatgaaaagtggagtggaatatggccactgcaacaccaaatgtcaaaccccagtaggtgcgataaattctagtatgccattccacaacatacatcctctcaccattggggaatgccccaaatacgtgaagtcaaacaagttggtccttgcgactgggctcagaaatagtcctctaagagaaGGGaagagaagaaaaagaggcctgtttggggcgatagcagggtttatagagggaggatggcagggaatggttgatggttggtatgggtaccatcatagcaatgagcaggggagtgggtacgctgcggacaaagaatccacccaaaaggcaatagatggagttaccaataaggtcaactcaatcattgacaaaatgaacactcaatttgaggcagttggaagggagtttaataacttagaaaggaggatagagaatttgaacaagaaaatggaagacggattcctagatgtctggacctataatgctgaacttctagttctcatggaaaacgagaggactctagatttccatgattcaaatgtcaagaacctttacgacaaagtcagattacagcttagggataatgcaaaggagctgggtaacggctgtttcgaattctatcacaaatgtgataatgaatgtatggaaagtgtgagaaatgggacgtatgactaccctcagtattcagaagaagcaagattaaaaagagaagaaataagcggagtgaaattagaatcagtaggaacttaccagatactgtcaatttattcaacagcggcaagttccctagcactggcaatcatgatggctggtctatctttatggatgtgctccaatgggtcgttacagtgcagaatttgcatt

>H5N1_A_turkey_Kentucky_22_004546_001_2022

atggagaacatagtactacttcttgcaatagttagccttgttaaaagtgatcagatttgcattggttaccatgcaaacaattcgacagagcaagttgacacgataatggaaaagaacgtcactgttacacatgcccaagacatactggaaaaaacacacaacgggaagctctgtgatctaaatggggtgaagcctctgattttaaaggattgtagtgtagctggatggctcctcggaaacccaatgtgcgacgaattcatcagagtgccggaatggtcctacatagtggagcgggctaacccagctaatgacctctgttacccagggagcctcaatgactatgaagaactgaaacacatgttgagcagaataaatcattttgagaagattctgatcattcccaagagttcctggccaaatcatgaaacatcactaggggtgagcgcagcttgtccataccagggagcgccctcctttttcagaaatgtggtgtggcttatcaaaaagaacgatgcatacccaacaataaagataagctacaataataccaatcgggaagatctcttgatactgtgggggattcatcattccaacaatgcagaagagcagacaaatctctacaaaaacccaaccacctacatttcagttggaacatcaactttaaaccagaggttggcaccaaaaatagctactagatcccaagtaaacgggcaacgtggaagaatggacttcttctggacaatcttaaaaccagatgatgcaatccatttcgagagtaatggaaatttcattgctccagaatatgcatacaaaattgtcaagaaaggggactcaacaattatgaaaagtggagtggaatatggccactgcaacaccaaatgtcaaaccccagtaggtgcgataaattctagtatgccattccacaacatacatcctctcaccattggggaatgccccaaatatgtgaagtcaaacaagttggtccttgcgactgggctcagaaatagtcctctaagagaaGGGaagagaagaaaaagaggcctgtttggggcgatagcagggtttatagagggaggatggcagggaatggttgatggttggtatgggtaccatcatagcaatgagcaggggagtgggtacgctgcggacaaagaatccacccaaaaggcaatagatggagttaccaataaggtcaactcaatcattgacaaaatgaacactcaatttgaggcagttggaagggagtttaataacttagaaaggaggatagagaatttgaacaagaaaatggaagacggattcctagatgtctggacctataatgctgaacttctagttctcatggaaaacgagaggactctagatttccatgattcaaatgtcaagaacctttacgacaaagtcagattacagcttagggataatgcaaaggagctgggtaacggctgtttcgaattctatcacaaatgtgataatgaatgtatggaaagtgtgagaaatgggacgtatgactaccctcagtattcagaagaagcaagattaaaaagagaagaaataagcggagtgaaattagaatcagtaggaacttaccagatactgtcaatttattcaacagcggcaagttccctagcactggcaatcatgatggctggtctatctttatggatgtgctccaatgggtcgttacagtgcagaatttgcatt

>H5N1_A_chicken_Germany_NI_AI01599_2021

atggagaacatagtacttcttcttgcaatagttagccttgttaaaagtgatcagatttgcattggttaccatgcaaacaattcgacagagcaagttgacacgataatggaaaagaacgtcactgttacacatgcccaagacatactggaaaaaacacacaacgggaagctctgtgatctaaatggggtgaagcctctgattttaaaggattgtagtgtagctggatggctcctcggaaacccaatgtgcgacgaattcatcagagtgccggaatggtcctacatagtggagcgggctaatccagctaatgacctctgttacccagggagcctcaatgactatgaagaactgaaacacctgttgagcagaataaatcattttgagaagattctgatcatccccaagagttcctggccaaatcatgaaacatcactaggggtgagcgcagcttgtccataccagggagcgccctcctttttcagaaatgtggtgtggcttatcaaaaagaacgatgcatacccaacaataaagataagctacaataataccaatcrggaagatctcttgatactgtgggggattcatcattccaacaatgcagaagagcagacaaatctctacaaaaacccaaccacctacatttcagttggaacatcaactttaaaccagaggttggcaccaaaaatagctactagatcccaagtaaacgggcaacgtggaagaatggacttcttctggacaattttaaaaccagatgatgcaatccatttcgagagtaatggaaatttcattgctccagaatatgcatacaaaattgtcaagaaaggggactcaacaattatgaaaagtggagtggaatatggccactgcaacaccaaatgtcaaaccccagtaggagcgataaattctagtatgccattccacaacatacatcctctcaccattggggaatgccccaaatacgtgaagtcaaacaagttggtccttgcgactgggctcagaaatagtcctctaagagaaGGGaagagaagaaaaagaggcctgtttggggcgatagcagggtttatagagggaggatggcagggaatggttgatggttggtatgggtaccatcatagcaatgagcaggggagtgggtacgctgcagacaaagaatccacccaaaaggcaatagatggagttaccaataaggtcaactcaatcattgacaaaatgaacactcaatttgaggcagttggaagggagtttaataacttagaaaggaggatagagaatttgaacaagaaaatggaagacggattcctagatgtctggacctataatgctgaacttctagttctcatggaaaacgagaggactctagatttccatgattcaaatgtcaagaacctttacgacaaagtcagactacagcttagggataatgcaaaggagctgggtaacggctgtttcgaattctatcacaaatgcgataatgaatgtatggaaagtgtgagaaatgggacgtatgactaccctcagtattcagaagaagcaagattaaaaagagaagaaataagcggagtgaaattagaatcaataggaacttaccagatactgtcaatttattcaacagcggcaagttccctagcactggcaatcatgatggctggtctatctttatggatgtgctccaatgggtcgttacagtgcagaatttgcatt

>H5N1_A_barnacle_goose_Finland_6247_21VIR7689_6_2021

atggagaacatagtacttcttcttgcaatagttagccttgttaaaagtgatcagatttgcattggttaccatgcaaacaattcgacagagcaagttgacacgataatggaaaagaacgtcactgttacacatgcccaagacatactggaaaaaacacacaacgggaagctctgtgatctaaatggggtgaagcctctgattttaaaggattgtagtgtagctggatggctcctcggaaacccaatgtgcgacgaattcatcagagtgccggaatggtcctacatagtggagcgggctaatccagctaatgacctctgttacccagggagcctcaatgactatgaagaactgaaacacctgttgagcagaataaatcattttgagaagattctgatcatccccaagagttcctggccaaatcatgaaacatcactaggggtgagcgcagcttgtccataccaaggagcgccctcctttttcagaaatgtggtgtggcttatcaaaaagaacgatgcatacccaacaataaagataagctacaataataccaatcgggaagatctcttgatactgtgggggattcatcattccaacaatgcagaagagcagacaaatctctacaaaaacccaaccacctacatttcagttggaacatcaactttaaaccagaggttggtaccaaaaatagctactagatcccaagtaaacgggcaacgtggaagaatggacttcttctggacaattttaaaaccagatgatgcaatccatttcgagagtaatggaaatttcattgctccagaatatgcatacaaaattgtcaagaaaggggactcaacaattatgaaaagtggagtggaatatggccactgcaacaccaaatgtcaaaccccagtaggagcgataaattctagtatgccattccacaacatacatcctctcaccattggggaatgccccaaatacgtgaagtcaaacaagttggtccttgcgactgggcttagaaatagtcctctaagagaaGGGaagagaagaaaaagaggcctgtttggggcgatagcagggtttatagagggaggatggcagggaatggttgatggttggtatgggtaccatcatagcaatgagcaggggagtgggtacgctgcagacaaagaatccacccaaaaggcaatagatggagttaccaataaggtcaactcaatcattgacaaaatgaacactcaatttgaggcagttggaagggagtttaataacttagaaaggaggatagagaatttgaacaagaaaatggaagacggattcctagatgtctggacctataatgctgaacttctagttctcatggaaaacgagaggactctagatttccatgattcaaatgtcaagaacctttacgacaaagtcagactacagcttagggataatgcaaaggagctgggtaacggctgtttcgaattctatcacaaatgcgataatgaatgtatggaaagtgtgagaaatgggacgtatgactaccctcagtattcagaagaagcaagattaaaaagagaagaaataagcggagtgaaattagaatcaataggaacttaccagatactgtcaatttattcaacagcggcaagttccctagcactggcaatcatgatggctggtctatctttatggatgtgctccaatgggtcgttacagtgcagaatttgcatt

>H5N1_A_chicken_Wales_053969_2021

atggagaacatagtacttcttcttgcaatagttagccttgttaaaagtgatcagatttgcattggttaccatgcaaacaattcgacagaacaagttgacacgataatggaaaagaacgtcactgttacacatgcccaagacatactggaaaaaacacacaacgggaagctctgtgatctaaatggggtgaagcctctgattttaaaggattgtagtgtagctggatggctcctcggaaacccaatgtgcgacgaattcatcagagtgccggaatggtcctacatagtggagcgggctaatccagctaatgacctctgttacccagggagcctcaatgactatgaagaactgaaacacctgttgagcagaataaatcattttgagaagattctgatcatccccaagagttcctggccaaatcatgaaacatcactaggggtgagcgcagcttgtccataccagggagcgccctcctttttcagaaatgtggtgtggcttatcaaaaagaacgatgcatacccaacaataaagataagctacaataataccaatcgggaagatctcttgatactgtgggggattcatcattccaacaatgcagaagagcagacaaatctctacaaaaacccaaccacctacatttcagttggaacatcaactttaaaccagaggttggtaccaaaaatagctactagatcccaagtaaacgggcaacgtggaagaatggacttcttctggacaattttaaaaccagatgatgcaatccatttcgagagtaatggaaatttcattgctccagaatatgcatacaaaattgtcaagaaaggggactcaacaattatgaaaagtggagtggaatatggccactgcaacaccaaatgtcaaaccccagtaggagcgataaattctagtatgccattccacaacatacatcctctcaccattggggaatgccccaaatacgtgaagtcaaacaaattggtccttgcgactgggcttagaaatagtcctctaagagaaGGGaagagaagaaaaagaggcctgtttggggcgatagcagggtttatagagggaggatggcagggaatggttgatggttggtatgggtaccatcatagcaatgagcaggggagtgggtacgctgcagacaaagaatccacccaaaaggcaatagatggagttaccaataaggtcaactcaatcattgacaaaatgaacactcaatttgaggcagttggaagggagtttaataacttagaaaggaggatagagaatttgaacaagaaaatggaagacggattcctagatgtctggacctataatgctgaacttctagttctcatggaaaacgagaggactctagatttccatgattcaaatgtcaagaacctttacgacaaagtcagactacagcttagggataatgcaaaggagctgggtaacggctgtttcgaattctatcacaaatgcgataatgaatgtatggaaagtgtgagaaatgggacgtatgactaccctcagtattcagaagaagcaagattaaaaagagaagaaataagcggagtgaaattagaatcaataggaacttaccagatactgtcaatttattcaacagcggcaagttccctagcactggcaatcatgatggctggtctatctttatggatgtgctccaatgggtcgttacagtgcagaatttgcatt

>H5N1_A_chicken_England_053052_2021

atggagaacatagtacttcttcttgcaatagttaaccttgttaaaagtgatcagatttgcattggttaccatgcaaacaattcgacagagcaagttgacacgataatggaaaagaacgtcactgttacacatgcccaagacatactggaaaaaacacacaacgggaagctctgtgatctaaatggggtgaagcctctgattttaaaggattgtagtgtagctggatggctcctcggaaacccaatgtgcgacgaattcatcagagtgccggaatggtcctacatagtggagcgggctaatccagctaatgacctctgttacccagggagcctcaatgactatgaagaactgaaacacctgttgagcagaataaatcattttgagaagattctgatcatccccaagagttcctggccaaatcatgaaacatcactaggggtgagcgcagcttgtccataccagggaacgccctcctttttcagaaatgtggtgtggcttatcaaaaagaacgatgcatacccaacaataaagataagctacaataataccaatcgggaagatctcttgatactgtgggggattcatcattccaacaatgcagaagagcagacaaatctctacaaaaacccaaccacctacatttcagttggaacatcaactttaaaccagaggttggtaccaaaaatagctactagatcccaagtaaacgggcaacgtggaagaatggacttcttctggacaattttaaaaccagatgatgcaatccatttcgagagtaatggaaatttcattgctccagaatatgcatacaaaattgtcaagaaaggggactcaacaattatgaaaagtggagtggaatatggccactgcaacaccaaatgtcaaaccccagtaggagcgataaattctagtatgccattccacaacatacatcctctcaccattggggaatgccccaaatacgtgaagtcaaacaagttggtccttgcgactgggcttagaaatagtcctctaagagaaGGGaagagaagaaaaagaggcctgtttggggcgatagcagggtttatagagggaggatggcagggaatggttgatggttggtatgggtaccatcatagcaatgagcaggggagtgggtacgctgcagacaaagaatccacccaaaaggcaatagatggagttaccaataaggtcaactcaatcattgacaaaatgaacactcaatttgaggcagttggaagggagtttaataacttagaaaggaggatagagaatttgaacaagaaaatggaagacggattcctagatgtctggacctataatgctgaacttctagttctcatggaaaacgagaggactctagatttccatgattcaaatgtcaagaacctttacgacaaagtcagactacagcttagggataatgcaaaggagctgggtaacggctgtttcgaattctatcacaaatgcgataatgaatgtatggaaagtgtgagaaatgggacgtatgactaccctcagtattcagaagaagcaagattaaaaagagaagaaataagcggagtgaaattagaatcaataggaacttaccagatactgtcaatttattcaacagcggcaagttccctagcactggcaatcatgatggctggtctatctttatggatgtgctccaatgggtcgttacagtgcagaatttgcatt

>H5N1_A_gull_Estonia_TA2113284_4_21VIR7512_8_2021

atggagaacatagtacttcttcttgcaatagttagccttgttaaaagtgatcagatttgcattggttaccatgcaaacaattcgacagagcaagttgacacgataatggaaaagaacgtcactgttacacatgcccaagacatactggaaaaaacacacaacgggaagctctgtgatctaaatggggtgaagcctttgattttaaaggattgtagtgtagctggatggctcctcggaaacccaatgtgcgacgaattcatcagagtgccggaatggtcctacatagtggagcgggctaatccagctaatgacctctgttacccagggagcctcaatgactatgaagaactgaaacacctgttgagcagaataaatcattttgagaagattctgatcatccccaagagttcctggccaaatcatgaaacatcactaggggtgagcgcagcttgtccataccagggagcgccctcctttttcagaaatgtggtgtggcttatcaaaaagaacgatgcatacccaacaataaagataagctacaataataccaatcgggaagatctcttgatactgtgggggattcatcattccaacaatgcaaaagagcagacaaatctctacaaaaacccaaccacctacatttcagttggaacatcaactttgaaccagaggttggtaccaaaaatagctactagatcccaagtaaacgggcaacgtggaagaatggacttcttctggacaattttaaaaccagatgatgcaatccatttcgagagtaatggaaatttcattgctccagaatatgcatacaaaattgtcaagaaaggggactcaacaattatgaaaagtggagtggaatatggccactgcaacaccaaatgtcaaaccccagtaggagcgataaattctagtatgccattccacaacatacatcctctcaccattggggaatgccccaaatacgtgaagtcaaacaagttggtccttgcgactgggctcagaaatagtcctctaagagaaGGGaagagaagaaaaagaggcctgtttggggcgatagcagggtttatagagggaggatggcagggaatggttgatggttggtatgggtaccatcatagcaatgagcaggggagtgggtacgctgcagacaaagaatccacccaaaaggcaatagatggagttaccaataaggtcaactcaatcattgacaaaatgaacactcaatttgaggcagttggaagggagtttaataacttagaaaggaggatagagaatttgaacaagaaaatggaagacggattcctagatgtctggacctataatgctgaacttctagttctcatggaaaacgagaggactctagatttccatgattcaaatgtcaagaacctttacgacaaagtcagactacagcttagggataatgcaaaggagctgggtaacggctgtttcgaattctatcacaaatgcgataatgaatgtatggaaagtgtgagaaatgggacgtatgactaccctcagtattcagaagaagcaagattaaaaagagaagaaataagcggagtgaaattagaatcaataggaacttaccagatactgtcaatttattcaacagcggcaagttccctagcactggcaatcatgatggctggtctatctttatggatgtgctccaatgggtcgttacagtgcagaatttgcatt

>H5N1_A_chicken_Netherlands_20019879_001005_2020

atggagaacatagtacttcttcttgcaatagttagccttgttaaaagtgatcagatttgcattggttaccatgcaaacaattcgacagagcaagttgacacgataatggaaaagaacgtcactgttacacatgcccaagacatactggaaaaaacacacaacgggaagctctgtgatctaaatggggtgaagcctctgattttaaaggattgtagtgtagctggatggctcctcggaaacccaatgtgcgacgaattcatcagagtgccggaatggtcctacatagtggagcgggctaatccagctaatgacctctgttacccagggagcctcaatgactatgaagaactgaaacacctgttgagcagaataaatcattttgagaagattctgatcatccccaagagttcctggccaaatcatgaaacatcactaggggtgagcgcagcttgtccataccagggagcgccctcctttttcagaaatgtggtgtggcttatcaaaaagaacgatgcatacccaacaataaagataagctacaataataccaatcgggaagatctcttgatactgtgggggattcatcattccaacaatgcagaagagcagacaaatctctacaaaaacccaaccacctacatttcagttggaacatcaactttaaaccagaggttggtaccaaaaatagctactagatcccaagtaaacgggcaacgtggaagaatggacttcttctggacaattttaaaaccagatgatgcaatccatttcgagagtaatggaaatttcattgctccagaatatgcatacaaaattgtcaagaaaggggactcaacaattatgaaaagtggagtggaatatggccactgcaacaccaaatgtcaaaccccagtaggagcgataaattctagtatgccattccacaacatacatcctctcaccattggggaatgccccaaatacgtgaagtcaaacaagttggtccttgcgactgggctcagaaatagtcctctaagagaaGGGaagagaagaaaaagaggcctgtttggggcgatagcagggtttatagagggaggatggcagggaatggttgatggttggtatgggtaccatcatagcaatgagcaggggagtgggtacgctgcagacaaagaatccacccaaaaggcaatagatggagttaccaataaggtcaactcaatcattgacaaaatgaacactcaatttgaggcagttggaagggagtttaataacttagaaaggaggatagagaatttgaacaagaaaatggaagacggattcctagatgtctggacctataatgctgaacttctagttctcatggaaaacgagaggactctagatttccatgattcaaatgtcaagaacctttacgacaaagtcagactacagcttagggataatgcaaaggagctgggtaacggctgtttcgaattctatcacaaatgcgataatgaatgtatggaaagtgtgagaaatgggacgtatgactaccctcagtattcagaagaagcaagattaaaaagagaagaaataagcggagtgaaattagaatcaataggaacttaccagatactgtcaatttattcaacagcggcgagttccctagcactggcaatcatgatggctggtctatctttatggatgtgctccaatgggtcgttacagtgcagaatttgcatt

>H5N1_A_swan_Netherlands_20017772_002_2020

atggagaacatagtacttcttcttgcaatagttagccttgttaaaagtgatcagatttgcattggttaccatgcaaacaattcgacagagcaagttgacacgataatggaaaagaacgtcactgttacacatgcccaagacatactggaaaaaacacacaacgggaagctctgtgatctaaatggggtgaagcctctgattttaaaggattgtagtgtagctggatggctcctcggaaacccaatgtgcgacgaattcatcagagtgccggaatggtcctacatagtggagcgggctaatccagctaatgacctctgttacccagggagcctcaatgactatgaagaactgaaacacctgttgagcagaataaatcattttgagaagattctgatcatccccaagagttcctggccaaatcatgaaacatcactaggggtgagcgcagcttgtccataccagggagcgccctcctttttcagaaatgtggtgtggcttatcaaaaagaacgatgcatacccaacaataaagataagctacaataataccaatcgggaagatctcttgatactgtgggggattcatcattccaacaatgcagaagagcagacaaatctctacaaaaacccaaccacctacatttcagttggaacatcaactttaaaccagaggttggtaccaaaaatagctactagatcccaagtaaacgggcaacgtggaagaatggacttcttctggacaattttaaaaccagatgatgcaatccatttcgagagtaatggaaatttcattgctccagaatatgcatacaaaattgtcaagaaaggggactcaacaattatgaaaagtggagtggaatatggccactgcaacaccaaatgtcaaaccccagtaggagcgataaattctagtatgccattccacaacatacatcctctcaccattggggaatgccccaaatacgtgaagtcaaacaagttggtccttgcgactgggctcagaaatagtcctctaagagaaGGGaagagaagaaaaagaggcctgtttggggcgatagcagggtttatagagggaggatggcagggaatggttgatggttggtatgggtaccatcatagcaatgagcaggggagtgggtacgctgcagacaaagaatccacccaaaaggcaatagatggagttaccaataaggtcaactcaatcattgacaaaatgaacactcaatttgaggcagttggaagggagtttaataacttagaaaggaggatagagaatttgaacaagaaaatggaagacggattcctagatgtctggacctataatgctgaacttctagttctcatggaaaacgagaggactctagatttccatgattcaaatgtcaagaacctttacgacaaagtcagactacagcttagggataatgcaaaggagctgggtaacggctgtttcgaattctatcacaaatgcgataatgaatgtatggaaagtgtgagaaatgggacgtatgactaccctcagtattcagaagaagcaagattaaaaagagaagaaataagcggagtgaaattagaatcaataggaacttaccagatactgtcaatttattcaacagcggcgagttccctagcactggcaatcatgatggctggtctatctttatggatgtgctccaatgggtcgttacagtgcagaatttgcatt

>H5N5_A_mute_swan_Austria_21013162_21VIR1085_5_2021

atggagaacatagtacttcttcttgcagtagttagccttgttaaaagtgatcagatttgcattggttaccatgcaaacaattcgacagagcaagttgacacgataatggaaaagaacgtcactgttacacatgcccaagacatactggaaaaaacacacaacgggaagctctgtgatctaaatggggtgaagcctctgattttaaaggattgtagtgtagctggatggctcctcggaaacccaatgtgcgacgaattcatcagagtgccggaatggtcctacatagtggagcgggctaatccagctaatgacctctgttacccagggagcctcaatgactatgaagaactgaaacacctgttgagcagaataaatcattttgagaagattctgatcatccccaagagttcctggccaaatcatgaaacatcactaggggtgagcgcagcttgtccataccagggagcgccctcctttttcagaaatgtggtgtggcttatcaaaaagaacgatgcatacccaacaataaagataagctacaataataccaatcgggaagatctcttgatactgtgggggattcatcattccaacaatgcagaagagcagacaaatctctataaaaacccaaccacctacatttcagttggaacatcaactttaaaccagaggttggtaccaaaaatagctactagatcccaagtaaacgggcaacgtggaagaatggacttcttctggacaattttaaaaccagatgatgcaatccatttcgagagtaatggaaatttcattgctccagaatatgcatacaaaattgtcaagaaaggggactcaacaattatgaaaagtggagtggaatatggccactgcaacaccaaatgtcaaaccccagtaggagcgataaattctagtatgccattccacaacatacatcctctcaccattggggaatgccccaaatacgtgaagtcaaacaagttggtccttgcgactgggctcagaaatagtcctctaagagaaGGGaagagaagaaaaagaggcctgtttggggcgatagcagggtttatagagggaggatggcagggaatggttgatggttggtatggataccatcatagcaatgagcaggggagtgggtacgctgcagacaaagaatccacccaaaaggcaatagatggagttaccaataaggtcaactcaatcattgacaaaatgaacactcaatttgaggcagttggaagggagtttaataacttagaaaggaggatagagaatttgaacaagaaaatggaagacggattcctagatgtctggacctataatgctgaacttctagttctcatggaaaacgagaggactctagatttccatgattcaaatgtcaagaacctttacgacaaagtcagactacagcttagggataatgcaaaggagctgggtaacggctgtttcgaattctatcacaaatgcgataatgaatgtatggaaagtgtgagaaatgggacgtatgactaccctcagtattcagaagaagcaagattaaaaagagaagaaataagcggagtgaaattagaatcaataggaacttaccagatactgtcaatttattcaacagcggcgagttccctagcactggcaatcatgatggctggtctatctttatggatgtgctccaatgggtcgttacagtgcagaatttgcatt

>H5N5_A_mute_swan_Czech_Republic_4099_2021

atggagaacatagtacttcttcttgcagtagttagccttgttaaaagtgatcagatttgcattggttaccatgcaaacaattcgacagagcaagttgacacgataatggaaaagaacgtcactgttacacatgcccaagacatactggaaaaaacacacaacgggaagctctgtgatctaaatggggtgaagcctctgattttaaaggattgtagtgtagctggatggctcctcggaaacccaatgtgcgacgaattcatcagagtgccggaatggtcctacatagtggagcgggctaatccagctaatgacctctgttacccagggagcctcaatgactatgaagaactgaaacacctgttgagcagaataaatcattttgagaagattctgatcatccccaagagttcctggccaaatcatgaaacatcactaggggtgagcgcagcttgtccataccagggagcgccctcctttttcagaaatgtggtgtggcttatcaaaaagaacgatgcatacccaacaataaagataagctacaataataccaatcgggaagatctcttgatactgtgggggattcatcattccaacaatgcagaagagcagacaaatctctataaaaacccaaccacctacatttcagttggaacatcaactttaaaccagaggttggtaccaaaaatagctactagatcccaagtaaacgggcaacgtggaagaatggacttcttctggacaattttaaaaccagatgatgcaatccatttcgagagtaatggaaatttcattgctccagaatatgcatacaaaattgtcaagaaaggggactcaacaattatgaaaagtggagtggaatatggccactgcaacaccaaatgtcaaaccccagtaggagcgataaattctagtatgccattccacaacatacatcctctcaccattggggaatgccccaaatacgtgaagtcaaacaagttggtccttgcgactgggctcagaaatagtcctctaagagaaGGGaagagaagaaaaagaggcctgtttggggcgatagcagggtttatagagggaggatggcagggaatggttgatggttggtatggataccatcatagcaatgagcaggggagtgggtacgctgcagacaaagaatccacccaaaaggcaatagatggagttaccaataaggtcaactcaatcattgacaaaatgaacactcaatttgaggcagttggaagggagtttaataacttagaaaggaggatagagaatttgaacaagaaaatggaagacggattcctagatgtctggacctataatgctgaacttctagttctcatggaaaacgagaggactctagatttccatgattcaaatgtcaagaacctttacgacaaagtcagactacagcttagggataatgcaaaggagctgggtaacggctgtttcgaattctatcacaaatgcgataatgaatgtatggaaagtgtgagaaatgggacgtatgactaccctcagtattcagaagaagcaagattaaaaagagaagaaataagcggagtgaaattagaatcaataggaacttaccagatactgtcaatttattcaacagcggcgagttccctagcactggcaatcatgatggctggtctatctttatggatgtgctccaatgggtcgttacagtgcagaatttgcatt

>H5N1_A_chicken_Senegal_21VIR1084_3_2021

atggagaacatagtacttcttcttgcaatagttagccttgttaaaagtgatcagatttgcattggttaccatgcaaacaattcgacagagcaagttgacacgataatggaaaagaacgtcactgttacacatgcccaagacatactggaaaaaacacacaacgggaagctctgtgatctaaatggggtgaagcctctgattttaaaggattgtagtgtagctggatggctcctcggaaacccaatgtgcgacgaattcatcagagtgccggaatggtcctacatagtggagcgggctaatccagctaatgacctctgttacccagggagcctcaatgactatgaagaactgaaacacctgttgagcagaataaatcattttgagaagattctgatcatccccaagagttcctggccaaatcatgaaacatcactaggggtgagcgcagcttgtccataccagggagcgccctcctttttcagaaatgtggtgtggcttatcaaaaagaacgatgcatacccaacaataaagataagctacaataataccaatcgggaagatctcttgatactgtgggggattcatcattccaacaatgcagaagagcagacaaatctctataaaaacccaaccacctacatttcggttggaacatcaactttaaaccagaggttggtaccaaaaatagctactagatcccaagtaaacgggcaacgtggaagaatggacttcttctggacaattttaaaaccagatgatgcaatccatttcgagagtaatggaaatttcattgctccagaatatgcatacaaaattgtcaagaaaggggactcaacaattatgaaaagtggagtggaatatggccactgcaacaccaaatgtcaaaccccagtaggagcgataaattctagtatgccattccacaacatacatcctctcaccattggggaatgccccaaatacgtgaagtcaaacaagttggtccttgcgactgggctcagaaatagtcctctaagagaaGGGaagagaagaaaaagaggcctgtttggggcgatagcagggtttatagagggaggatggcagggaatggttgatggttggtatgggtaccatcatagcaatgagcaggggagtgggtacgctgcagacaaagaatccacccaaaaggcaatagatggagttaccaataaggtcaactcaatcattgacaaaatgaacactcaatttgaggcagttggaagggagtttaataacttagaaaggaggatagagaatttgaacaagaaaatggaagacggattcctagatgtctggacctataatgctgaacttctagttctcatggaaaacgagaggactctagatttccatgattcaaatgtcaagaacctttacgacaaagtcagactacagcttagggataatgcaaaggaactgggtaacggctgtttcgaattctatcacaaatgcgataatgaatgtatggaaagtgtgagaaatgggacgtatgactactctcagtattcagaagaagcaagattaaaaagagaagaaataagcggagtgaaattagaatcaataggaacttaccagatactgtcaatttattcaacagcggcgagttccctagcactggcaatcatgatggctggtctatctttatggatgtgctccaatgggtcgttacagtgcagaatttgcatt

>H5N1_A_chicken_Niger_22VIR1409_23_2022

atggagaacatagtacttcttcttgcaatagttagccttgttaaaagtgatcagatttgcattggttaccatgcaaacaattcgacagagcaagttgacacgataatggaaaagaacgttactgttacccatgcccaagacatactggaaaaaacacacaacgggaagctctgtgatctaaatggagtgaagcctctgattttaaaggattgtagtgtagctggatggctcctcggaaacccaatgtgcgacgaattcatcagagtgccggaatggtcctacatagtggagcgggctaatccagctaatgacctctgttacccagggagcctcaatgactatgaagaactgaaacacctgttgagcagaataaatcattttgagaagattctgatcatccccaagagttcctggccaaatcatgaaacatcgctaggggttagtgcagcttgtccataccagggagcgccctcctttttcagaaatgtggtatggcttatcaaaaagaacgattcatacccaacaataaagataagctacaataataccaatcaggaagatctcttgatactgtgggggattcatcattccaacaatgcagaagagcagacaaatctctataaaaacccaaccacctacatttcagttggaacatcaactttaaaccagaggttggtaccaaaaatagctactagatcccaagtaaacgggcaacgtggaagaatggacttcttctggacaattttaaaaccagatgatgcaatccatttcgagagtaacggaaatttcattgctccagaatatgcatacaaaattgtcaagaaaggggactcaacaattatgaaaagtggagtggaatatggcaactgcaacaccaaatgtcaaaccccagtaggagcgataaattctagtatgccattccacaacatacatcctctcaccattggggaatgccccaaatacgtgaagtcaaacaagttggtccttgcgactgggctcagaaatagtcctctaagagaaGGGaagagaagaaaaagaggcctgtttggggcgatagcagggtttatagagggaggatggcagggaatggttgatggttggtatgggtaccatcatagcaatgagcaggggagtgggtacgctgcagacaaagaatccacccaaaaggcaatagatggagttaccaataaggtcaactcaatcattgacaaaatgaacactcaatttgaggcagttggaagggagtttaataacttagaaaggaggatagagaatctgaacaagaaaatggaagacggattcctagatgtctggacctataatgctgaacttctagttctcatggaaaacgagaggactctagatttccatgattcaaatgtcaagaacctttacgacaaagtcagactacagcttagagataatgcaaaggagctgggtaacggctgtttcgaattctatcacaaatgcgataatgaatgtatggaaagtgtgagaaatgggacgtatgactaccctcagtattcagaagaagcaagattaaaaagagaagaaataagcggagtaaaattagaatcaataggaacttaccagatactgtcaatttattcaacagcggcgagttccctagcactggcaatcatgatggctggtctatctttatggatgtgctccaatgggtcgttacagtgcagaatttgcatt

>H5N8_A_chicken_Northern_Ireland_2021_000067_21VIR114_19_2021

atggagaacatagtacttcttcttgcaatagttagccttgttaaaagtgatcagatttgcattggttaccatgcaaacaattcgacagagcaagttgacacgataatggaaaagaacgtcactgttacacatgcccaagacatactggaaaaaacacacaacgggaagctctgtgatctaaatggggtgaagcctctgattttaaaggattgtagtgtagctggatggctcctcggaaacccaatgtgcgacgaattcatcagagtgccggaatggtcctacatagtggagcgggctaatccagctaatgacctctgttacccagggagtctcaatgactatgaagaactgaaacacctgttgagcagaataaatcattttgagaagattctgatcatccccaagagttcctggccaaatcatgaaacatcactaggggtgagcgcagcttgtccataccagggagcgccctcctttttcagaaatgtggtgtggcttatcaaaaagaacgatgcatacccaacaataaagataagctacaataataccaatcgggaagatctcttgatactgtgggggattcatcattccaacaatgcagaagagcagacaaatctctataaaaacccaaccacctacatttcagttggaacatcaactttaaaccagaggttggtgccaaaaatagctactagatcccaagtaaacgggcaacgtggaagaatggacttcttctggacaattttaaaaccggatgatgcaatccatttcgagagtaatggaaatttcattgctccagaatatgcatacaaaattgtcaagaaaggggactcaacaattatgaaaagtggagtggaatatggccactgcaacaccaaatgtcaaaccccagtaggagcgataaattctagtatgccattccacaacatacatcctctcaccattggggaatgccccaaatacgtgaagtcaaacaagttggtccttgcgactgggctcagaaataatcctctaagagaaGGGaagagaagaaaaagaggcctgtttggggcgatagcaggatttatagagggaggatggcagggaatggttgatggttggtatgggtaccatcatagcaatgagcaggggagtgggtacgctgcagacaaagaatccacccaaaaggcaatagatggagttaccaataaggtcaactcaatcattgacaaaatgaacactcaatttgaggcagttggaagggagtttaataacttagaaaggaggatagagaatttgaacaagaaaatggaagacggattcctagatgtctggacctataatgctgaacttctagttctcatggaaaacgagaggactctagatttccatgattcaaatgtcaagaacctttacgacaaagtcagactacagcttagggataatgcaaaggagctgggtaacggctgtttcgaattctatcacaaatgcgataatgaatgtatggaaagtgtgagaaatgggacgtatgactaccctcagtattcagaagaagcaagattaaaaagagaagaaataagcggagtgaaattagaatcaataggaacttaccagatactgtcaatttattcaacagcggcgagttccctagcactggcaatcatgatggctggtctatctttatggatgtgctccaatgggtcgttacagtgcagaatttgcatt

>H5N8_A_Numenius_arquata_Belgium_11956_003_2020

atggagaacatagtacttcttcttgcaatagttagccttgttaaaagtgatcagatttgcattggttaccatgcaaacaattcgacagagcaagttgacacgataatggaaaagaacgtcactgttacacatgcccaagacatactggaaaaaacacacaacgggaagctctgtgatctaaatggggtgaagcctctgattttaaaggattgtagtgtagctggatggctcctcggaaacccaatgtgcgacgaattcatcagagtgccggaatggtcctacatagtggagcgggctaatccagctaatgacctctgttacccagggagtctcaatgactatgaagaactgaaacacctgttgagcagaataaatcattttgagaagattctgatcatccccaagagttcctggccaaatcatgaaacatcactaggggtgagcgcagcttgtccataccagggagcgccctcctttttcagaaatgtggtgtggcttatcaaaaagaacgatgcatacccaacaataaagataagctacaataataccaatcgggaagatctcttgatactgtgggggattcatcattccaacaatgcagaagagcagacaaatctctataaaaacccaaccacctacatttcagttggaacatcaactttaaaccagaggttggtgccaaaaatagctactagatcccaagtaaacgggcaacgtggaagaatggacttcttctggacaattttaaaaccggatgatgcaatccatttcgagagtaatggaaatttcattgctccagaatatgcatacaaaattgtcaagaaaggggactcaacaattatgaaaagtggagtggaatatggccactgcaacaccaaatgtcaaaccccagtaggagcgataaattctagtatgccattccacaacatacatcctctcaccattggggaatgccccaaatacgtgaagtcaaacaagttggtccttgcgactgggctcagaaataatcctctaagagaaGGGaagagaagaaaaagaggcctgtttggggcgatagcaggatttatagagggaggatggcagggaatggttgatggttggtatgggtaccatcatagcaatgagcaggggagtgggtacgctgcagacaaagaatccacccaaaaggcaatagatggagttaccaataaggtcaactcaatcattgacaaaatgaacactcaatttgaggcagttggaagggagtttaataacttagaaaggaggatagagaatttgaacaagaaaatggaagacggattcctagatgtctggacctataatgctgaacttctagttctcatggaaaacgagaggactctagatttccatgattcaaatgtcaagaacctttacgacaaagtcagactacagcttagggataatgcaaaggagctgggtaacggctgtttcgaattctatcacaaatgcgataatgaatgtatggaaagtgtgagaaatgggacgtatgactaccctcagtattcagaagaagcaagattaaaaagagaagaaataagcggagtgaaattagaatcaataggaacttaccagatactgtcaatttattcaacagcggcgagttccctagcactggcaatcatgatggctggtctatctttatggatgtgctccaatgggtcgttacagtgcagaatttgcatt

>H5N8_A_turkey_England_039352_2020

atggagaacatagtacttcttcttgcaatagttagccttgttaaaagtgatcagatttgcattggttaccatgcaaacaattcgacagagcaagttgacacgataatggaaaagaacgtcactgttacacatgcccaagacatactggaaaaaacacacaacgggaagctctgtgatctaaatggggtgaagcctctgattttaaaggattgtagtgtagctggatggctcctcggaaacccaatgtgcgacgaattcatcagagtgccggaatggtcctacatagtggagcgggctaatccagctaatgacctctgttacccagggagtctcaatgactatgaagaactgaaacacctgttgagcagaataaatcattttgagaagattctgatcatccccaagagttcctggccaaatcatgaaacatcactaggggtgagcgcagcttgttcataccagggagcgccctcctttttcagaaatgtggtgtggcttatcaaaaagaacgatgcatacccaacaataaagataagctacaataataccaatcgggaagatctcttgatactgtgggggattcatcattccaacaacgcagaagagcagacaaatctctataaaaacccaaccacctacatttcagttggaacatcaactttaaaccagaggttggtgccaaaaatagctactagatcccaagtaaacgggcaacgtggaagaatggacttcttctggacaattttaaaaccggatgatgcaatccatttcgagagtaatggaaatttcattgctccagaatatgcatacaaaattgtcaagaaaggggactcaacaattatgaaaagtggagtggaatatggccactgcaacaccaaatgtcaaaccccagtaggagcgataaattctagtatgccattccacaacatacatcctctcaccattggggaatgccccaaatacgtgaagtcaaacaagttggtccttgcgactgggctcagaaataatcctctaagagaaGGGaagagaagaaaaagaggcctgtttggggcgatagcaggatttatagagggaggatggcagggaatggttgatggttggtatgggtaccatcatagcaatgagcaggggagtgggtacgctgcagacaaagaatccacccaaaaggcaatagatggagttaccaataaggtcaactcaatcattgacaaaatgaacactcaatttgaggcagttggaagggagtttaataacttagaaaggaggatagagaatttgaacaagaaaatggaagacggattcctagatgtctggacctataatgctgaacttctagttctcatggaaaacgagaggactctagatttccatgattcaaatgtcaagaacctttacgacaaagtcagactacagcttagggataatgcaaaggagctgggtaacggctgtttcgaattctatcacaaatgcgataatgaatgtatggaaagtgtgagaaatgggacgtatgactaccctcagtattcagaagaagcaagattaaaaagagaagaaataagcggagtgaaattagaatcaataggaacttaccagatactgtcaatttattcaacagcggcgagttccctagcactggcaatcatgatggctggtctatctttatggatgtgctccaatgggtcgttacagtgcagaatttgcatt

>H5N8_A_crow_Kazakhstan_15_20_B_Talg_4_2020

atggagaacatagtacttcttcttgcaatagttagccttgttaaaagtgatcagatttgcattggttaccatgcaaacaattcgacagagcaagttgacacgataatggaaaagaacgtcactgttacacatgcccaagacatactggaaaaaacacacaacgggaagctctgtgatctaaatggggtgaagcctctgattttaaaggattgtagtgtagctggatggctcctcggaaacccaatgtgcgacgaattcatcagagtgccggaatggtcctacatagtggagcgggctaatccagctaatgacctctgttacccagggagcctcaatgactatgaagaactgaaacacctgttgagcagaataaatcattttgagaagattctgatcatccccaagagttcctggccaaatcatgaaacatcactaggggtgagcgcagcttgtccataccagggagcgccctcctttttcagaaatgtggtgtggcttatcaaaaagaacgatgcatacccaacaataaagataagctacaataataccaatcgggaagatctcttgatactgtgggggattcatcattccaacaatgcagaagagcagacaaatctctataaaaacccaaccacctacatttcagttggaacatcaactttaaaccagaggttggtaccaaaaatagctactagatcccaagtaaacgggcaacgtggaagaatggacttcttctggacaattttaaaaccggatgatgcaatccatttcgagagtaatggaaatttcattgctccagaatatgcatacaaaattgtcaagaaaggggactcaacaattatgaaaagtggagtggaatatggccactgcaacaccaaatgtcaaaccccagtaggagcgataaattctagtatgccattccacaacatacatcctctcaccattggggaatgccccaaatacgtgaagtcaaacaagttggtccttgcgactgggctcagaaatagtcctctaagagaaGGGaagagaagaaaaagaggcctgtttggggcgatagcagggtttatagagggaggatggcagggaatggttgatggttggtatgggtaccatcatagcaatgagcaggggagtgggtacgctgcagacaaagaatccacccaaaaggcaatagatggagttaccaataaggtcaactcaatcattgacaaaatgaacactcaatttgaggcagttggaagggagtttaataacttagaaaggaggatagagaatttgaacaagaaaatggaagacggattcctagatgtctggacctataatgctgaacttctagttctcatggaaaacgagaggactctagatttccatgattcaaatgtcaagaacctttacgacaaagtcagactacagcttagggataatgcaaaggagctgggtaacggctgtttcgaattctatcacaaatgcgataatgaatgtatggaaagtgtgagaaatgggacgtatgactaccctcagtattcagaagaagcaagattaaaaagagaagaaataagcggagtgaaattagaatcaataggaacttaccagatactgtcaatttattcaacagcggcgagttccctagcactggcaatcatgatggctggtctatctttatggatgtgctccaatgggtcgttacagtgcagaatttgcatt

>H5N6_A_Hunan_10117_2021

atggagaacatagtacttcttcttgcaatagttagccttgttaaaagtgatcagatttgcattggttaccatgcaaacaattcgacagagcaagttgacacgataatggaaaagaacgtcactgttacacatgcccaagacatactggaaaaaacacacaacgggaagctctgtgatctaaatggggtgaagcctctgattttaaaggattgtagtgtggctggatggctcctcggaaacccaatgtgcgacgaattcatcagagtgccggaatggtcctacatagtggagagggctaatccagctaatgacctctgttacccagggagcctcaatgactatgaagaactgaaacacctgttgagcagaataaatcattttgagaagattctgatcatccccaagagttcatggccaaaccatgaaacatcactaggggtgagcgcagcttgtccataccagggagcgccctcctttttcagaaatgtggtgtggcttatcaaaaagaacgatgcatacccaacaataaagataagctacaataataccaatcgggaagatctcttgatactgtgggggattcatcattccaacaatgcagaagagcagataaatctctataaaaacccaaccacctacatttcagttggaacatcaactttaaaccagaggttggtaccaaaaatagctactagatcccaagtaaacgggcaacgtggaagaatggacttcttctggacaattttaaaaccggatgatgcaatccatttcgagagtaatggaaatttcattgctccagaatatgcatacaaaattgtcaagaaaggggactcaacaattatgaaaagtggagtggaatatggccactgcaacaccaaatgtcaaaccccagtaggagcgataaattctagtatgccattccacaacatacatcctctcaccattggggaatgccccaaatacgtgaaatcaaacaagttggtccttgcgactgggctcagaaatagtcctctaagagaaGGGaagagaagaaaaagaggcctgttcggggcgatagcagggtttatagagggaggatggcagggaatggttgatggttggtatgggtaccaccatagcaatgagcaggggagtgggtacgctgcagacaaagaatccacccaaaaggcaatagatggagttaccaataaggtcaactcaatcattgacaaaatgaacactcaatttgaggcagttggaagggagtttaataacttagaaaggaggatagagaatttgaacaagaaaatggaagacggattcctagatgtatggacctataatgctgaacttctagttctcatggaaaacgagaggactctagatttccatgattcaaatgtcaagaacctttacgacaaagtcagactacagcttagggataatgcaaaggagctgggtaacggctgtttcgaattctatcacaaatgcgataatgaatgtatggaaagtgtgagaaatgggacgtatgactaccctcagtattcagaagaagcaaggttaaaaagagaagaaataagcggagtgaaattagaatcaataggaacttaccagatactgtcaatttattcaacagcggcgagttccctagcactggcaatcatgatagctggtctatctttatggatgtgctccaatgggtcgttacagtgcagaatttgcatt

>H5N6_A_Hangzhou_01_2021

atggagaacatagtatttcttcttgcaatagttagccttgttaaaagtgatcagatttgcattggttaccatgcaaacaattcgacagagcaagttgacacgataatggaaaagaacgtcactgttacacatgcccaagacatactggaaaaaacacacaacgggaagctctgtgatctaaatggggtgaagcctctgattttaaaggattgtagtgtggctggatggctcctcggaaacccaatgtgcgacgaattcatcagagtgccggaatggtcctacatagtggagagggctaatccagctaatgacctctgttacccagggagcctcaatgactatgaagaactgaaacatctgttgagcagaataaatcattttgagaagattctgatcatccccaagagttcatggccaaatcatgaaacatcactaggggtgagtgcagcttgtccataccagggagcgccctcctttttcagaaatgtggtgtggcttatcaaaaagaacgatgcatacccaacaataaagataagctacaataataccaatcgggaagatctcttgatactgtgggggattcatcattccaacaatgcagaagagcagataaacctctataaaaactcaaccacctacatttcagttggaacatcaactttaaaccagaggttggtaccaaaaatagctactagatcccaagtaaacgggcaacgtggaagaatggacttcttctggacaattttaaaaccggatgatgcaatacatttcgagagtaatggaaatttcattgctccagaatatgcatacaaaattgtcaagaaaggggactcaacaattatgaaaagtggagtggaatatggccactgcaacaccaaatgtcaaaccccagtaggagcgataaattctagtatgccattccacaacatacatcctctcaccattggggaatgccccaaatacgtgaaatcaaacaagttggtccttgcgactgggctcagaaatagtcctctaagagaaGGGaagagaagaaaaagaggcctgttcggggcgatagcagggtttatagagggaggatggcagggaatggttgatggttggtatgggtaccaccatagcaatgagcaggggagtgggtacgctgcagacaaagaatccacccaaaaggcaatagatggagttaccaataaggtcaactcaatcattgacaaaatgaacactcaatttgaggcagttggaagggagtttaataacttagaaaggaggatagagaatttgaacaagaaaatggaagacggattcctagatgtatggacctataatgctgaacttctagttctcatggaaaacgagaggactctagatttccatgattcaaatgtcaagaacctttacgacaaagtcagactacagcttagggataatgcaaaggagctgggtaacggctgtttcgaattctatcacaaatgcgataatgaatgtatggaaagtgtgagaaacgggacgtatgactaccctcagtattcagaagaagcaaggttaaaaagagaagaaataagcggggtgaaattagaatcaataggaacttaccagatactgtcaatttattcaacagcggcgagttccctagcactggcaatcatgatagctggtctatctttatggatgtgctccaatgggtcgttacagtgcagaatttgcatt

>H5N8_A_whooper_swan_Shanxi_4_2_2020

atggagaacatagtacttcttcttgcaatagttagccttgttaaaagtgatcagatttgcattggttaccatgcaaacaattcgacagagcaagttgacacgataatggaaaagaacgtcactgttacacatgcccaagacatactggaaaaaacacacaacgggaagctctgtgatctaaatggggtgaagcctctgattttaaaggattgtagtgtagctggatggctcctcggaaacccaatgtgcgacgaattcatcagagtgccggaatggtcctacatagtggagagggctaatccagctaatgacctctgttacccagggagcctcaatgactatgaagaactgaaacacctgttgagcagaataaatcattttgagaagattctgatcatccccaagagttcatggccaaaccatgaaacatcactaggggtgagcgcagcttgtccataccagggagcgccctcctttttcagaaatgtggtgtggcttatcaaaaagaacgatgcatacccaacaataaagataagctacaataataccaatcgggaagatctcttgatactgtgggggattcatcattccaacaatgcagaagagcagataaatctctataaaaacccaaccacctacatttcagttggaacatcaactttaaaccagaggttggtaccaaaaatagctactagatcccaagtaaacgggcaacgtggaagaatggacttcttctggacaattttaaaaccggatgatgcaatccatttcgagagtaatggaaatttcattgctccagaatatgcatacaaaattgtcaagaaaggggactcaacaattatgaaaagtggagtggaatatggccactgcaacaccaaatgtcaaaccccagtaggagcgataaattctagtatgcccttccacaacatacatcctctcaccattggggaatgccccaaatacgtgaaatcaaacaagttggtccttgcgactgggctcagaaatagtcctctaagagaaGGGaagagaagaaaaagaggcctgttcggggcgatagcagggtttatagagggaggatggcagggaatggttgatggttggtatgggtaccaccatagcaatgagcaggggagtgggtacgctgcagacaaagaatccacccaaaaggcaatagatggagttaccaataaggtcaactcaatcattgacaaaatgaacactcaatttgaggcagttggaagggagtttaataacttagaaaggaggatagagaatttgaacaagaaaatggaagacggattcctagatgtctggacctataatgctgaacttctagttctcatggaaaacgagaggactctagatttccatgattcaaatgtcaagaacctttacgacaaagtcagactacagcttagggataatgcaaaggagctgggtaacggctgtttcgaattctatcacaaatgcgataatgaatgtatggaaagtgtgagaaatgggacgtatgactaccctcagtattcagaagaagcaagattaaaaagagaagaaataagcggagtgaaattagaatcaataggaacttaccagatactgtcaatttattcaacagcggcgagttccctagcactggcaatcatgatggctggtctatctttatggatgtgctccaatgggtcgttacagtgcagaatttgcatt

>H5N8_A_chicken_Vietnam_HU14_LB11_2021

atggagaacatagtacttcttcttgcaatagttagccttgttaaaagtgatcagatttgcattggttaccatgcaaacaattcgacagagcaagttgacacgataatggaaaagaacgtcactgttacacatgcccaagacatactggaaaaaacacacaacgggaagctctgtgatctaaatggggtgaagcctctgattttaaaggattgtagtgtagctggatggctcctcggaaacccaatgtgtgacgaattcatcagagtgccggaatggtcctacatagtggagagggctaatccagctaatgacctctgttacccagggagcctcaatgactatgaagaactgaaacacctattgagcagaataaatcattttgagaagattctgatcatccccaagagttcatggccaaaccatgaaacatcactaggggtgagcgcagcttgtccataccagggagcgccctcctttttcagaaatgtggtgtggcttatcaaaaagaacaatgcatacccaacaataaagataagctacaataataccaatcgggaagatctcttgatactgtgggggattcatcattccaacaatgcagaagagcagataaatctctataaaaacccaaccacctacatttcagttggaacatcaactttaaaccagaggttggtaccaaaaatagctactagatcccaagtaaacgggcaacgtggaagaatggacttcttctggacaattttgaaaccggatgatgcaatccatttcgagagtaatggaaatttcattgctccagaatatgcatacaaaattgtcaagaaaggggactcaacaattatgaaaagtggagtggaatatggccactgcaacaccaaatgtcaaaccccagtaggagcgataaattctagtatgccattccacaacatacaccctctcaccattggggaatgccccaaatacgtgaaatcaaacaagttggtccttgcgactgggctcagaaatagtcctctaagagaaGGGaagagaagaaaaagaggcctgttcggggcgatagcagggtttatagagggaggatggcagggaatggttgatggttggtatgggtaccaccatagcaatgagcaggggagtgggtacgctgcagacaaagactccacccaaaaggccatagatggagttaccaataaggtcaactcaatcattgacaaaatgaacactcaatttgaggcagttggaagggagtttaataacttagaaaggaggatagagaatttgaacaagaaaatggaagacggattcctagatgtctggacctataatgctgaacttctagttctcatggaaaacgagaggactctagatttccatgattcaaatgtcaagaacctttacgacaaagtcagactacagcttagggataatgcaaaggagctgggtaacggctgtttcgaattctatcacaaatgcgataatgaatgtatggaaagtgtgagaaatgggacgtatgactaccctcagtattcagaagaagcaagattaaaaagagaagaaataagcggagttaaattagaatcaataggaacttaccagatactgtcaatttattcaacagcggcgagttccctagcactggcaatcatgatggctggtctatctttatggatgtgctccaatgggtcgttacagtgcagaatttgcatt

>H5N8_A_duck_Guangxi_S11043_2021

atggagaacatagtacttcttcttgcaatagttagccttgttaaaagtgatcagatttgcattggttaccatgcaaacaattcgacagagcaagttgacacgataatggaaaagaacgtcactgttacacatgcccaagacatactggaaaaaacacacaacgggaagctctgtgatctaaatggggtgaagcctctgattttaaaggattgtagtgtagctggatggctcctcggaaacccaatgtgcgacgaattcatcagagtgccggaatggtcctacatagtggagagggctaatccagctaatgacctctgttacccagggagcctcaatgactatgaagaactgaaacacctgttgagcagaataaatcattttgagaagattctgatcatccccaagagttcatggccaaaccatgaaacatcactaggggtgagcgcagcttgtccataccagggagcgccctcttttttcagaaatgtggtgtggcttatcaaaaagaacgatgcatacccaacaataaagataagctacaataataccaatcgggaagatctcttgatactgtgggggattcatcattccaacaatgcagaagagcagataaatctctataaaaacccaaccacctacatttcagttggaacatcaactttaaaccagaggttggtaccaaaaatagctactagatcccaagtaaacgggcaacgtggaagaatggacttcttctggacaattttaaaaccggatgatgcaatccatttcgagagtaatggaaatttcattgctccagaatatgcatacaaaattgtcaagaagggggactcaacaattatgaaaagtggagtggaatatggccactgcaacaccaaatgtcaaaccccagtaggagcgataaattctagtatgccattccacaacatacatcctctcaccattggggaatgccccaaatacgtgaaatcaaacaagttggtccttgcgactgggctcagaaatagtcctctaagagaaGGGaagagaagaaaaagaggcctgttcggggcgatagcagggtttatagagggaggatggcagggaatggttgatggttggtatgggtaccaccatagcaatgagcaggggagtgggtacgctgcagacaaagaatccacccaaaaggccatagatggagttaccaataaggtcaactcaatcattgacaaaatgaacactcaatttgaggcagttggaagggagtttaataacttagaaaggaggatagagaatttgaacaagaaaatggaagacggattcctagatgtctggacctataatgctgaacttctagttctcatggaaaacgaaaggactctagatttccatgattcaaatgtcaagaacctttacgacaaagtcagactacagcttagggataatgcaaaggagctgggtaacggctgtttcgaattctatcacaaatgcgataatgaatgtatggaaagtgtgagaaatgggacgtatgactaccctcagtattcagaagaagcaagattaaaaagagaagaaataagcggagtgaaattagaatcaataggaacttaccagatactgtcaatttattcaacagcggcgagttccctagcactggcaatcatgatggctggtctatctttatggatgtgctccaatgggtcgttacagtgcagaatttgcatt

>H5N8_A_tundra_swan_Hubei_BQ6_2020

atggagaacatagtgcttcttcttgcaatagttagccttgttaaaagtgatcagatttgcattggttaccatgcaaacaattcgacagagcaagttgacacgataatggaaaagaacgtcactgttacacatgcccaagacatactggaaaaaacacacaacgggaagctctgtgatctaaatggggtgaagcctctgattttaaaggattgtagtgtagctggatggctcctcggaaacccaatgtgcgacgaattcatcagagtgccggaatggtcctacatagtggagagggctaatccagctaatgacctctgttacccagggagcctcaatgactatgaagaactgaaacacctgttgagcagaataaatcattttgagaagattctgatcatccccaagagttcatggccaaaccatgaaacatcactaggggtgagcgcagcttgtccataccagggagcgccctcctttttcagaaatgtggtgtggcttatcaaaaagaacgatgcatacccaacaataaagataagctacaataataccaatcgggaagatctcttgatactgtgggggattcatcattccaacaatgcagaagagcagataaatctctataaaaacccaaccacctacatttcagttggaacttcaactttaaaccagaggttggtaccaaaaatagctactagatcccaagtaaacgggcaacgtggaagaatggacttcttctggacaattttaaaaccggatgatgcaatccatttcgagagtaatggaaatttcattgctccagaatatgcatacaaaattgtcaagaaaggggactcaacaattatgaaaagtggagtggaatatggccactgcaacaccaaatgtcaaaccccagtaggagcgataaattctagtatgccattccacaacatacatcctctcaccattggggaatgccccaaatacgtgaaatcaaacaagttggtccttgcgactgggctcagaaatagttctctaagagaaGGGaagagaagaaaaagaggcctgttcggggcgatagcagggtttatagagggaggatggcagggaatggttgatggttggtatgggtaccaccatagcaatgagcaggggagtgggtacgctgcagacaaagaatccacccaaaaggcaatagatggagttaccaataaggtcaactcaatcattgacaaaatgaacactcaatttgaggcagttggaagggagtttaataacttagaaaggaggatagagaatttgaacaagaaaatggaagacggattcctagatgtctggacctataatgctgaacttctagttctcatggaaaacgagaggactctagatttccatgattcaaatgtcaagaacctttacgacaaagtcagactacagcttagggataatgcaaaggagctgggtaacggctgtttcgaattctatcacaaatgcgataatgaatgtatggaaagtgtgagaaatgggacgtatgactaccctcagtattcagaagaagcaagattaaaaagagaagaaataagcggagtgaaattagaatcaataggaacttaccagatactgtcaatttattcaacagcggcgagttccctagcactggcaatcatggtggctggtctatctttatggatgtgctccaatgggtcgttacagtgcagaatttgcatt

>H5N8_A_whooper_swan_Henan_SM16_2020

atggagaacatagtgcttcttcttgcaatagttagccttgttaaaagtgatcagatttgcattggttaccatgcaaacaattcgacagagcaagttgacacgataatggaaaagaacgtcactgttacacatgcccaagacatactggaaaaaacacacaacgggaagctctgtgatctaaatggggtgaagcccctgattttaaaggattgtagtgtagctggatggctcctcggaaacccaatgtgcgacgaattcatcagagtgccggaatggtcctacatagtggagagggctaatccagctaatgacctctgttacccagggagcctcaatgactatgaagaactgaaacacctgttgagcagaataaatcattttgagaagattctgatcatccccaaaagttcatggccaaaccatgaaacatcactaggggtgagcgcagcttgtccataccagggagcgccctcctttttcagaaatgtggtgtggcttatcaaaaagaacgatgcatacccaacaataaagataagctacaataataccaatcgggaagatctcttgatactgtgggggattcatcattccaacaatgcagaagagcagataaatctctataaaaacccaaccacctacatttcagttggaacatcaactttaaaccagaggttgataccaaaaatagctactagatcccaagtaaacgggcaacgtggaagaatggacttcttctggacaattttaaaaccggatgatgcaatccatttcgagagtaatggaaatttcattgctccagaatatgcatacaaaattgtcaagaaaggggactcaacaattatgaaaagtggagtggaatatggccactgcaacaccaaatgtcaaaccccagtaggagcgataaattctagtatgccattccacaacatacatcctctcaccattggggaatgccccaaatacgtgaaatcaaacaagttggtccttgcgactgggctcagaaatagtcctctaagagaaGGGaagagaagaaaaagaggcctgttcggggcgatagcagggtttatagagggaggatggcagggaatggttgatggttggtatgggtaccaccatagcaatgagcaggggagtgggtacgctgcagacaaagaatccacccaaaaggcaatagatggagttaccaataaggtcaactcaatcattgacaaaatgaacactcaatttgaggcagttggaagggagtttaataacttagaaaggaggatagagaatttgaacaagaaaatggaagacggattcctagatgtctggacctataatgctgaacttctagttctcatggaaaacgagaggactctagatttccatgattcaaatgtcaagaacctttacgacaaagtcagactacagcttagggataatgcaaaggagctgggtaacggctgtttcgaattctatcacaaatgcgataatgaatgtatggaaagtgtgagaaatgggacgtatgactaccctcagtattcagaagaagcaagattaaaaagagaagaaataagcggagtgaaattagaatcaataggaacttaccagatactgtcaatttattcaacagcggcgagttccctagcactggcaatcatgatggctggtctatctttatggatgtgctccaatgggtcgttacagtgcagaatttgcatt

>H5N8_A_mute_swan_Shandong_1_2021

atggagaacatagtacttcttcttgcaatagttagccttgttaaaagtgatcagatttgcattggttaccatgcaaacaattcgacagagcaagttgacacgataatggaaaagaacgtcactgttacacatgcccaagacatactggaaaaaacacacaacgggaagctctgtgatctaaatggggtgaagcctctgattttaaaggattgtagtgtagctggatggctcctcggaaacccaatgtgcgacgaattcatcagagtgccggaatggtcctacatagtggagagggctaatccagctaatgacctctgttacccagggagcctcaatgactatgaagaactgaaacacctgttgagcagaataaatcattttgagaagattctgatcatccccaagagttcatggccaaaccatgaaacttcactaggggtgagcgcagcttgtccataccagggagcgccctcctttttcagaaatgtggtgtggcttatcaaaaagaacgatgcatacccaacaataaagataagctacaataataccaatcgggaagatctcttgatactgtgggggattcatcattccaacaatgcagaagagcagatgaatctctataaaaacccaaccacctacatttcagttggaacatcaactttaaaccagaggttggtaccaaaaatagctacgagatcccaagtaaacgggcaacgtggaagaatggacttcttctggacaattttaaaaccggatgatgcaatccatttcgagagtaatggaaatttcattgctccagaatatgcatacaaaattgtcaagaaaggggactcaacaattatgaaaagtggagtggaatatggccactgcaacaccaaatgtcaaaccccagtaggagcgataaattctagtatgccattccacaacatacatcctctcaccattggggaatgccccaaatacgtgaaatcaaacaagttggtacttgcgactgggctcagaaatagtcctctaagagaaGGGaagagaagaaaaagaggcctgttcggggcgatagcagggtttatagagggaggatggcagggaatggttgatggttggtatgggtaccaccatagcaatgagcaggggagtgggtacgctgcagacaaagaatccacccaaaaggcaatagatggagttaccaataaggtcaactcaatcattgacaaaatgaacactcagtttgaggcagttggaagggagtttaataacttagaaaggaggatagagaatttgaacaagaaaatggaagacggattcctagatgtctggacctataatgctgaacttctagttctcatggaaaacgagaggactctagatttccatgattcaaatgtcaagaacctttacgacaaagtcagactacagcttagggataatgcaaaggagctgggtaacggctgtttcgaattctatcacaaatgcgataatgaatgtatggaaagtgtgagaaatgggacgtatgactaccctcagtattcagaagaagcaagattaaaaagagaagaaataagcggagtgaaattagaatcaataggaacttaccagatactgtcaatttattcaacagcggcgagttccctagcactggcaatcatgatggctggtctatctttatggatgtgctccaatgggtcgttacagtgcagaatttgcatt

>H5N6_A_Sichuan_06681_2021

atggagaacatagtacttcttcttgcaatagttagccttgttaaaagtgatcagatttgcattggttaccatgcaaacaattcgacagagcaagttgacacgataatggaaaagaacgtcactgttacacatgcccaagacatactggaaaaaacacacaacgggaagctctgtgatctgaatggggtgaagcctctgattttaaaggattgtagtgtagctggatggctcctcggaaacccaatgtgcgatgaattcatcagagtgccggaatggtcctacatagtggagagggctaatccagctaatgacctctgttacccagggagcctcaatgactatgaagaactgaaacacctgttgagcagaataaatcattttgagaagattctgatcatccccaaggggtcctggccaaaccatgaaacatcactaggggtgagcgcagcttgtccataccagggagcgccctcctttttcagaaatgtggtgtggcttgtcaaaaagaacgatgcatacccaacaataaagataagctacaataataccaatcgggaagatctcttgatactgtgggggattcaccattccaacaatgcagaagagcagataaatctctataaaaacccaaccacctacatttcagttggaacatcaactttaaaccagaggttggtaccaaaaatagctactagatcccaagtaaacgggcaacgtggaagaatggacttcttctggacaattttaaaaccggatgatgcaatccatttcgagagtaacggaaatttcattgctccagaatatgcatacaaaattgtcaagaaaggggactcaacaattatgaaaagtggagtggaatatggccactgcaacaccaaatgtcaaaccccaataggagcgataaattctagtatgccattccacaacatacatcctctcaccattggggaatgccccaaatacgtgaaatcaaacaagttagtccttgcgactgggctcagaaatagtcctctaagagaaGGGaagagaagaaaaagaggcctgtttggggcgatagcagggtttatagagggaggatggcagggaatggttgatggttggtatgggtaccaccatagcaatgagcaggggagtgggtacgctgcagacaaagaatccacccaaaaggcaatagatggagttaccaataaggtcaactcaatcattgacaaaatgaacactcaatttgaggcagttggaagggagtttaataacttagaaaggaggatagagaatttgaacaagaaaatggaagacggattcctagatgtctggacctataatgctgaacttctagttctcatggaaaacgagaggactctagatttccatgattcaaatgtcaagaacctttacgacaaagtcagactacagcttagggataatgcaaaggagctgggtaacggctgtttcgaattctatcacaaatgcgataatgaatgtatggaaagtgtgagaaatgggacgtatgactaccctcagtattcagaagaagcaagattaaaaagagaggaaataagcggagtgaaattagaatcaataggaacttaccagatactgtcaatttattcaacagcggcgagttccctagcactggcaatcatgatggctggtctatctttatggatgtgctccaatgggtcgttacagtgcagaatttgcatt

>H5N6_A_Chongqing_02_2021

atggagaacatagtacttcttcttgcgatagttagccttgttaaaagtgatcagatttgcattggttaccatgcaaacaattcgacagagcaagttgacacgataatggaaaagaacgtcactgttacacatgcccaagacatactggaaaaaacacacaacgggaagctctgtgatctgaatggggtgaagcctctgattttaaaggattgtagtgtagctggatggctcctcggaaacccaatgtgcgacgaattcatcagagtgccggaatggtcctacatagtggagagggctaatccagctaatgacctctgttacccagggagcctcaatgactatgaagaactgaaacacctgttgagcagaataaatcattttgagaagattctgatcatccccaagagttcctggccaaaccatgaaacatcactaggggtgagcgcagcttgtccataccagggagcgccctcctttttcagaaatgtggtgtggcttatcaaaaagaacgatgcatacccaacaataaagataagctacaataataccaatcgggaagatctcttgatactgtgggggattcaccactccaacaatgcagaagagcagataaatctctataaaaacccaaccacctacatttcagttggaacatcaactttaaaccagaggttggtaccaaaaatagctactagatcccaagtaaacgggcaacgtggaagaatggacttcttctggacaattttaaaaccggatgatgcaatccatttcgagagtaacggaaatttcattgctccagaatatgcatacaaaattgtcaagaaaggggactcaacaattatgaaaagtggagtggaatatggccactgcaacaccaaatgtcaaaccccagtaggagcgataaattctagtatgccattccacaacatacatcctctcaccattggggaatgccccaaatacgtgaaatcaaacaagttagtccttgcgactgggctcagaaatagtcctctaagagaaGGGaagagaagaaaaagaggcctgtttggggcgatagcagggtttatagagggaggatggcagggaatggttgatggttggtatgggtaccaccacagcaatgagcaggggagtgggtacgctgcagacaaagaatccacccaaaaggcaatagatggagttaccaataaggtcaactcaatcattgacaaaatgaacactcaatttgaggcagttggaagggagtttaataacttagaaaggaggatagagaatttgaacaagaaaatggaagacggattcctagatgtctggacctataatgctgaacttctagttctcatggaaaacgagaggactctagatttccatgattcaaatgtcaagaacctttacgacaaagtcagactacagcttagggataatgcaaaggagctgggtaacggctgtttcgaattctatcacaaatgcgataatgaatgtatggaaagtgtgagaaatgggacgtatgactaccctcagtattcagaagaagcaagattaaaaagagaggaaataagcggagtgaaattagaatcaataggaacttaccagatactgtcaatttattcaacagcggcgagttccctagcactggcaatcatgatggctggtctatctttatggatgtgctccaatgggtcgttacagtgcagaatttgcatt

>H5N8_A_goose_Henan_S1315_2021

atggagaacatagtacttcttcttgcaatagttagccttgttaaaagtgatcagatttgcaatggttaccatgcaaacaattcgacagagcaagttgacacgataatggaaaagaacgtcactgttacacatgcccaagacatactggaaaaaacacacaacgggaagctctgtgatctaaatggggtgaagcctctgattttaaaggattgtagtgtagctggatggctcctcggaaacccaatgtgcgacgaattcatcagagtgccggaatggtcctacatagtggagagggctaacccagctaatgacctctgttacccagggagcctcaatgactatgaagaactgaaacacctgttgagcagaataaatcattttgagaagattctgatcatccccaagagttcctggccaaaccatgaaacatcactaggggtgagcgcagcttgtccataccagggagcgccctcctttttcagaaatgtggtgtggcttatcaaaaagaacgatgcatacccaacaataaagataagctacaataataccaatcgggaagatctcttgatactgtgggggattcaccattccaacaatgcagaagagcagataaatctctataaaaacccaaccacctacatttcagttggaacatcaactttaaaccagaggttggtaccaaaaatagctactagatcccaagtaaacgggcaacgtggaagaatggacttcttctggacaattttaaaaccgaatgatgcaatccatttcgagagtaatggaaatttcattgctccagaatatgcatacaaaattgtcaagaaaggggactcaacaattatgaaaagtggagtggaatatggccattgcaacaccaaatgtcaaaccccagtaggagcgataaattctagtatgccattccacaacatacatcctctcaccattggggaatgccccaaatacgtgaaatcaaacaagttggtccttgcgactgggctcagaaatagtcctctaagagaaGGGaagagaagaaaaagaggcctgtttggggcgatagcagggtttatagagggaggatggcagggaatggttgatggttggtatgggtaccaccatagcaatgagcaggggagtgggtacgctgcagacaaagaatccacccaaaaggcaatagatggagttaccaataaagtcaactcaatcattgacaaaatgaacactcaatttgaggcagttggaagggagtttaataacttagaaaggaggatagagaatttgaacaagaaaatggaagacggattcctagatgtctggacctataatgctgaacttctggttctcatggaaaacgagaggactctagatttccatgattcaaatgtcaagaacctttacgacaaagtcagactacagcttagggataatgcaaaggagctgggtaacggctgtttcgaattctatcacaaatgcgataatgaatgtatggaaagtgtgagaaatgggacgtatgactaccctcagtattcagaagaagcaagattaaaaagagaagaaataagcggagtgaaattagaatcaataggaacttaccagatactgtcaatttattcaacagcggcgagttccctagcactggcaatcatgatggctggtctatctttatggatgtgctccaatgggtcgttacagtgcagaatttgcatt

>H5N8_A_whooper_swan_Shandong_SC198_2021

atggagaacatagtacttcttcttgcaatagttagccttgttaaaagtgatcagatttgcattggttaccatgcaaacaattcgacagagcaagttgacacgataatggaaaagaacgtcactgttacacatgcccaagacatactggaaaaaacacacaacgggaagctctgtgatctaaatggggtgaagcctctgattttaaaggattgtagtgtagcgggatggctcctcggaaacccaatgtgcgacgaattcatcagagtgccggaatggtcctacatagtggagagggctaatccagctaatgacctctgttacccagggagcctcaatgactatgaagaactgaaacacctgttgagcagaataaatcattttgagaagattctgatcatccccaagagttcctggccaaaccatgaaacatcactaggggtgagcgcagcttgtccataccagggagcgccctcctttttcagaaatgtggtgtggcttatcaaaaagaacgatgcatacccaacaataaagataagctacaataataccaatcgggaagatctcttgatactgtgggggattcaccattccaacaatgcagaagagcagataaatctctataaaaacccaaccacctacatttcagttggaacatcaactttaaaccagaggttggtaccaaaaatagctactagatcccaagtaaacgggcaacgtggaagaatggacttcttctggacaattttaaaaccggatgatgcaatccatttcgagagtaatggaaatttcattgctccagaatatgcatacaaaattgtcaagaaaggggactcaacaattatgaaaagtggagtggaatatggccactgcaacaccaaatgtcaaaccccagtaggagcgataaattctagtatgccattccacaacatacatcctctcaccattggggaatgccccaaatacgtgaaatcaaacaagttggtccttgcgactgggctcagaaatagtcctctaagagaaGGGaagagaagaaaaagaggcctgtttggggcgatagcagggtttatagagggaggatggcagggaatggttgatggttggtatgggtaccaccatagcaatgagcaggggagtgggtacgctgcagacaaagaatccacccaaaaggcaatagatggagttaccaataaggtcaactcaatcattgacaaaatgaacactcaatttgaggcagttggaagggagtttaataacttagaaaggaggatagagaatttgaacaagaaaatggaagacggattcctagatgtctggacctataatgctgaacttctagttctcatggaaaacgagaggactctagatttccatgattcaaatgtcaagaacctttacgacaaagtcagactacagcttagggataatgcaaaggagctgggtaacggctgtttcgaattctatcacaaatgcgataatgaatgtatggaaagtgtgagaaatgggacgtatgactaccctcagtattcagaagaagcaagattaaaaagagaagaaataagcggagtgagattagaatcaataggaacttaccagatactgtcaatttattcaacagcggcgagttccctagcactggcaatcatgatggctggtctatctttatggatgtgctccaatgggtcgttacagtgcagaatttgcatt

>H5N8_A_goose_Liaoning_S1266_2021

atggagaacatagtacttcttcttgcaatagttagccttgttaaaagtgatcagatttgcattggttaccatgcaaacaattcgacagaacaagttgacacgataatggaaaagaacgtcactgttacacatgcccaagacatactggaaaaaacacacaacgggaagctctgtgatctaaacggggtgaagcctctgattttaaaggattgtagtgtagctggatggctcctcggaaacccaatgtgcgacgaattcatcagagtgccggaatggtcctacatagtggagagggctaatccagctaatgacctctgttacccagggagtctcaatgactatgaagaactgaaacacctgttgagcagaataaatcattttgagaagattctgatcatccccaagagttcctggccaaaccatgaaacatcactaggggtgagcgcagcttgtccataccagggagcgccctcctttttcagaaatgtggtgtggcttatcaaaaagaacgatgcatacccaacaataaagataagctacaataataccaaccgggaagatctcttgatactgtgggggattcaccattccaacaatgcagaagagcagataaatctctataaaaacccaaccacctacatttcagttggaacatcaactttaaaccagaggttggtaccaaaaatggctactagatcccaagtaaacgggcaacgtggaagaatggacttcttctggacaattttaaaaccggatgatgcaatccatttcgagagtaatggaaatttcattgctccagaatatgcatacaaaattgtcaagaaaggggactcaacaattatgaaaagtggagtggaatatggccactgcaacaccaaatgtcaaaccccaataggagcgataaattctagtatgccattccacaacatacatcctctcaccattggggaatgccccaaatacgtgaaatcaaacaagttggtccttgcgactgggctcagaaatagtcctctaagagaaGGGaagagaagaaaaagaggcctgtttggggcgatagcagggtttatagagggaggatggcagggaatggttgatggttggtatgggtaccaccatagcaatgagcaggggagtgggtacgctgcagacaaagattccacccaaaaggcaataaatggagttaccaataaggtcaactcaatcattgacaaaatgaacactcaatttgaggcagttggaagggagtttaataacttagaaaggaggatagagaatttgaacaagaaaatggaagacggattcctagatgtctggacctataatgctgaacttctagttctcatggaaaacgagaggactctagatttccatgattcaaatgtcaagaacctttacgacaaagtcagactacagcttagggataatgcaaaggagctgggtaacggctgtttcgaattctatcacaaatgcgataatgaatgtatggaaagtgtgagaaatgggacgtatgactaccctcagtattcagaagaagcaagattaaaaagagaagaaataagcggggtgaaattagaatcaataggaacttaccagatactgtcaatttattcaacagcggcgagttccctagcactggcaatcatgatggctggtctatctttatggatgtgctccaatgggtcgttacagtgcagaatttgcatt

>H5N8_A_duck_Hebei_S1070_2021

atggagaacatagtacttcttcttgcaatagttagccttgttaaaagtgatcagatttgcattggttaccatgcaaacaattcgacagagcaagttgacacgataatggaaaagaacgtcactgttacacatgcccaagacatactggaaaaaacacacaacgggaagctctgtgatctaaatggggtgaagcctctgattttaaaggattgtagtgtagctggatggctcctcggaaacccaatgtgcgacgaattcatcagagtgccggaatggtcctacatagtggagagggctaatccagctaatgacctctgttacccagggagcctcaatgactatgaagaactgaaacacctgttgagcagaataaatcattttgagaagattctgatcatccccaagagttcctggccaaaccatgaaacatcactaggggtgagcgcagcttgtccataccagggagcgccctcctttttcagaaatgtggtgtggcttatcaaaaagaacgatgcatacccaacaataaagataagctacaagaataccaatcgggaagatctcttgatactgtgggggattcaccattccaacaatgcagaagagcagataaatctttataaaaacccaaccacctacatttcagttggaacatcaactttaaaccagaggttggtaccaaaaatagctactagatcccaagtaaacgggcaacgtggaagaatggacttcttctggacaattttaaaaccggatgatgcaatccatttcgagagtaatggaaatttcattgctccagaatatgcatacaaaattgtcaagaaaggggactcaacaattatgaaaagtggagtggaatatggccactgcaacaccaaatgtcaaaccccagtaggagcgataaattctagtatgccattccacaacatacatcctctcaccattggggaatgccccaaatacgtgaaatcaaacaagttggtccttgcgactgggctcagaaatagtcctctaagagaaGGGaagagaagaaaaagaggcctgtttggggcgatagcagggtttatagagggaggatggcagggaatggtttatggttggtatgggtaccaccataacaatgagcaggggagtgggtacgctgcagacaaagaatccacccaaaaggcaatagatggagttaccaataaggtcaactcaatcattgacaaaatgaacactcaatttgaggcagttggaagggagtttaataacttagaaaggaggatagagaatttgaacaagaaaatggaagacggattcctagatgtctggacctataatgctgaacttctagttctcatggaaaacgagaggactctagatttccatgattcaaatgtcaagaacctttacgacaaagtcagactacagcttagggataatgcaaaggagctgggtaacggctgtttcgaattctaccacaaatgcgataatgaatgtatggaaagtgtgagaaatgggacgtatgactaccctcagtattcagaagaagcaagattaaaaagagaagaaataagcggactgaaattagaatcaatagggacttaccagatactgtcaatttattcaacagcggcgagttccctagcactggcaatcatgatggctggtctatctttatggatgtgctccaatgggtcgttacagtgcagaatttgcatt

>H5N8_A_duck_Korea_H549_2020

atggagaacatagtacttcttcttgcaatagttagccttgttaaaagtgatcagatttgcattggttaccatgcaaacaattcgacagagcaagttgacacgataatggaaaagaacgtcactgttacacatgcccaagacatactggaaaaaacacacaacgggaagctctgtgatctaaatggggtgaagcctctgattttaaaggattgtagtgtagctggatggctcctcggaaacccaatgtgcgacgaattcatcagagtgccggaatggtcctacatagtggagcgggctaatccagctaatgacctctgttacccagggagcctcaatgactatgaagaactgaaacacctgttgagcagaataaatcattttgagaagattctgatcatccccaagagttcctggccaaaccatgaaacatcactaggggtgagcgcagcttgtccataccagggagcgccctcctttttcagaaatgtggtgtggcttatcaaaaagaacgatgcatacccaacaataaagataagctacaataataccaatcgggaagatctcttgatactgtgggggattcaccattccaacaatgcagaagagcagataaatctctataaaaacccaaccacctacatttcagttggaacatcaactttaaaccagaggttggtaccaaaaatagctactagatcccaagtaaacgggcaacgtggaagaatggacttcttctggacaattttaaaaccggatgatgcaatccatttcgagagtaatggaaatttcattgctccagaatatgcatacaaaattgtcaagaaaggggactcaacaattatgaaaagtggagtggaatatggccactgcaacaccaaatgtcaaaccccagtaggagcgataaattctagtatgccattccacaacatacatcctctcaccattggggaatgccccaaatacgtgaaatcaaacaagttggtccttgcgactgggctcagaaatagtcctataagagaaGGGaagagaagaaaaagaggcctgtttggggcgatagcagggtttatagagggaggatggcagggaatggttgatggttggtatgggtaccaccatagcaatgagcaggggagtgggtacgctgcagacaaagaatccacccaaaaggcaatagatggagttaccaataaggtcaactcaatcattgacaaaatgaacactcaatttgaggcagttggaagggagtttaataacttagaaaggaggatagagaatttgaacaagaaaatggaagacggattcctagatgtctggacctataatgctgaacttctagttctcatggaaaacgagaggactctagatttccatgattcaaatgtcaagaacctttacgacaaagtcagactacagcttagggataatgcaaaggagctgggtaacggctgtttcgaattctatcacaaatgcgataatgaatgtatggaaagtgtgagaaatgggacgtatgactaccctcagtattcagaagaagcaagattaaaaagagaagaaataagcggagtgaaattagaatcaataggaacttaccagatactgtcaatttattcaacagcggcgagttccctagcactggcaatcatgatggctggtctatctttatggatgtgctccaatgggtcgttacagtgcagaatttgcatt

>H5N5_A_whooper_swan_Romania_10122_21VIR2593_23_2021

atggagaacatagtacttcttcttgcaatagttagccttgttaaaagtgatcagatttgcattggttaccatgcaaacaattcgacagagcaagttgacacgataatggaaaagaacgtcactgttacacatgcccaagacatactggaaaaaacacacaacgggaagctctgtgatctaaatggggtgaagcctctgattttaaaggattgtagtgtagctggatggctcctcggaaacccaatgtgcgacgaattcatcagagtgccggaatggtcctacatagtggagagggctaatccagctaatgacctctgctacccagggagtctcaatgactatgaagaactgaaacacctgttgagcagaataaatcattttgagaagattctgatcatccccaagagttcctggccaaaccatgaaacatcactaggggtgagcgcagcttgtccataccagggagcgccctcctttttcagaaatgtggtgtggcttatcaaaaagaacgatgcatacccaacaataaagataagctacaataataccaatcgggaagatctcttgatactgtgggggattcatcattccaacaatgcagaagagcagacaaacctctataaaaacccaaccacctacatttcagttggaacatcaactttaaaccagaggttggtaccaaaaatagctactagatcccaagtaaacgggcaacgtggaagaatggacttcttctggacaattttaaaaccggatgatgcaatccatttcgagagtaatggaaatttcattgctccagaatatgcatacaaaattgtcaagaaaggggactcaacaattatgaaaagtggagtggaatatggccactgcaacaccaaatgtcaaaccccagtaggagcgataaattctagtatgccattccacaacatacatcctctcaccattggggaatgccccaaatacgtgaagtcaaacaagttggtccttgcgactgggctcagaaatagtcctctaagagaaGGGaagagaagaaaaagaggcctgtttggggcgatagcagggtttatagagggaggatggcagggaatggttgatggttggtatgggtaccaccatagcaatgagcaggggagtgggtacgctgcagacaaagaatccacccaaaaggcaatagatggagttaccaataaggtcaactcaatcattgacaaaatgaacactcaatttgaggcagttggaagggagtttaataacttagaaaggaggatagagaatttgaacaagaaaatggaagacggattcctagatgtctggacctataatgctgaacttctagttctcatggaaaacgagaggactctagatttccatgattcaaatgtcaagaacctttacgacaaagtcagactacagcttagggataatgcaaaggagctgggtaacggatgtttcgaattctaccacaaatgcgataatgaatgtatggaaagtgtgagaaatgggacgtatgactaccctcagtattcagaagaagcaagattaaaaagagaagaaataagcggagtgaaattagaatcaataggaacttaccagatactgtcaatttattcaacagcggcgagttccctagcactggcaatcatgatggctggtctatctttatggatgtgctccaatgggtcgttacagtgcagaatttgcatt

>H5N5_A_grey_heron_Bulgaria_223_21VIR4270_2_2021

atggagaacatagtacttcttcttgcaatagttagccttgttaaaagtgatcagatttgcattggttaccatgcaaacaattcgacagagcaagttgacacgataatggaaaagaacgtcactgttacacatgcccaagacatactggaaaaaacacacaacgggaagctctgtgatctaaatggggtgaagcctctgattttaaaggattgtagtgtagctggatggctcctcggaaacccaatgtgcgacgaattcatcagagtgccggaatggtcctacatagtggagagagctaatccagctaatgacctctgctacccagggagtctcaatgactatgaagaactgaaacacctgttgagcagaataaatcattttgagaagattctgatcatccccaagagttcctggccaaaccatgaaacatcactaggggtgagcgcagcttgtccataccagggagcgccctcctttttcagaaatgtggtgtggcttatcaaaaagaacgatgcatacccaacaataaagataagctacaataataccaatcgggaagatctcttgatactgtgggggattcatcattccaacaatgcagaagagcagacaaatctctataaaaacccaaccacctacatttccgttggaacatcaactttaaaccagaggttggtaccaaaaatagctactagatcccaagtaaacgggcaacgtggaagaatggacttcttctggacaattttaaaaccggatgatgcaatccatttcgagagtaatggaaatttcattgctccagaatatgcatacaaaattgtcaagaaaggggactcaacaattatgaaaagtggagtggaatatggccactgcaacaccaaatgtcaaaccccagtaggagcgataaattctagtatgccattccacaacatacatcctctcaccattggggaatgccccaaatacgtgaagtcaaacaagttggtccttgcgactgggctcagaaatagtcctctaagagaaGGGaaaagaagaaaaagaggcctgtttggggcgatagcagggtttatagagggaggatggcagggaatggttgatggttggtatgggtaccaccatagcaatgagcaggggagtgggtacgctgcagacaaagaatccacccaaaaggcaatagacggagttaccaacaaggtcaactcaatcattgacaaaatgaacactcaatttgaggcagttggaagggagtttaataacttagaaaggaggatagagaatttgaacaagaaaatggaagacggattcctagatgtctggacctataatgctgaacttctagttctcatggaaaacgagaggactctagatttccatgattcaaatgtcaagaacctttacgacaaagtcagactacagcttagggataatgcaaaggagctgggtaacggctgtttcgaattctaccacaaatgcgataatgaatgtatggaaagtgtgagaaatgggacgtatgactaccctcagtattcagaagaagcaagattaaaaagagaagaaataagcggagtgaaattagaatcaataggaacttaccagatactgtcaatttattcaacagcggcgagttccctagcactggcaatcatgatggctggtctatctttatggatgtgctccaatgggtcgttacagtgcagaatttgcatt

>H5N8_2344b_A_Astrakhan_3212_2020

atggagaacatagtacttcttcttgcaatagttagccttgttaaaagtgatcagatttgcattggttatcatgcaaacaattcgacagagcaagttgacacgataatggaaaagaacgtcactgttacacatgcccaagacatactggaaaaaacacacaacgggaagctctgtgatctaaatggggtgaagcctctgattttaaaggattgtagtgtagctggatggctcctcggaaacccaatgtgcgacgaattcatcagagtgccggaatggtcctacatagtggagagggctaatccagctaatgacctctgctacccagggagcctcaatgactatgaagaactgaaacacctgttgagcagaataaatcattttgagaagattctgattatccccaagagttcctggccaaaccatgaaacatcactaggggtgagcgcagcttgtccataccagggagcgccctcctttttcagaaatgtggtgtggcttatcaaaaagaacgatgcatacccaacgataaagataagctacaataataccaatcgggaagatctcttgatactgtgggggattcatcattccaacaatgcagaagagcagacaaatctctataaaaacccaaccacctacatttcagttggaacatcaactttaaaccagaggttggtaccaaaaatagctactagatcccaagtaaacgggcaacgtggaagaatggacttcttctggacaattttaaaaccggatgatgcaatccatttcgagagtaatggaaatttcattgctccagaatatgcatacaaaattgtcaagaaaggggactcaacaattatgaaaagtggagtggaatatggccactgcaacaccaaatgtcaaaccccagtaggagcgataaattctagtatgccattccacaacatacatcctctcaccattggggaatgccccaaatacgtgaagtcaaacaagttggtccttgcgactgggctcagaaatagtcctctaagagaaGGGaagagaagaaaaagaggcctgtttggggcgatagcagggtttatagagggaggatggcagggaatggttgatggttggtatgggtaccaccatagcaatgagcaggggagtgggtacgctgcagacaaagaatccacccaaaaggcaatagatggagttaccaataaggtcaactcaatcattgacaaaatgaacactcaatttgaggcagttggaagggagtttaataacttagaaaggaggatagagaatttgaacaagaaaatggaagacggattcctagatgtctggacctataatgctgaacttctagttctcatggaaaacgagaggactctagatttccatgattcaaatgtcaagaacctttacgacaaagtcagactacagcttagggataatgcaaaggagctgggtaacggctgtttcgaattctaccacaaatgcgataatgaatgtatggaaagtgtgagaaatgggacgtatgactaccctcagtattcagaagaagcaagattaaaaagagaagaaataagcggagtgaaattagaatcaataggaacttaccagatactgtcaatttattcaacagcggcgagttccctagcactggcaatcatgatggctggtctatctttatggatgtgctccaatgggtcgttacagtgcagaatttgcatt

>H5N8_A_chicken_Astrakhan_2171_1_2020

atggagaacatagtacttcttcttgcaatagttagccttgttaaaagtgatcagatttgcattggttatcatgcaaacaattcgacagagcaagttgacacgataatggaaaagaacgtcactgttacacatgcccaagacatactggaaaaaacacacaacgggaagctctgtgatctaaatggggtgaagcctctgattttaaaggattgtagtgtagctggatggctcctcggaaacccaatgtgcgacgaattcatcagagtgccggaatggtcctacatagtggagagggctaatccagctaatgacctctgctacccagggagcctcaatgactatgaagaactgaaacacctgttgagcagaataaatcattttgagaagattctgattatccccaagagttcctggccaaaccatgaaacatcactaggggtgagcgcagcttgtccataccagggagcgccctcctttttcagaaatgtggtgtggcttatcaaaaagaacgatgcatacccaacgataaagataagctacaataataccaatcgggaagatctcttgatactgtgggggattcatcattccaacaatgcagaagagcagacaaatctctataaaaacccaaccacctacatttcagttggaacatcaactttaaaccagaggttggtaccaaaaatagctactagatcccaagtaaacgggcaacgtggaagaatggacttcttctggacaattttaaaaccggatgatgcaatccatttcgagagtaatggaaatttcattgctccagaatatgcatacaaaattgtcaagaaaggggactcaacaattatgaaaagtggagtggaatatggccactgcaacaccaaatgtcaaaccccagtaggagcgataaattctagtatgccattccacaacatacatcctctcaccattggggaatgccccaaatacgtgaagtcaaacaagttggtccttgcgactgggctcagaaatagtcctctaagagaaGGGaagagaagaaaaagaggcctgtttggggcgatagcagggtttatagagggaggatggcagggaatggttgatggttggtatgggtaccaccatagcaatgagcaggggagtgggtacgctgcagacaaagaatccacccaaaaggcaatagatggagttaccaataaggtcaactcaatcattgacaaaatgaacactcaatttgaggcagttggaagggagtttaataacttagaaaggaggatagagaatttgaacaagaaaatggaagacggattcctagatgtctggacctataatgctgaacttctagttctcatggaaaacgagaggactctagatttccatgattcaaatgtcaagaacctttacgacaaagtcagactacagcttagggataatgcaaaggagctgggtaacggctgtttcgaattctaccacaaatgcgataatgaatgtatggaaagtgtgagaaatgggacgtatgactaccctcagtattcagaagaagcaagattaaaaagagaagaaataagcggagtgaaattagaatcaataggaacttaccagatactgtcaatttattcaacagcggcgagttccctagcactggcaatcatgatggctggtctatctttatggatgtgctccaatgggtcgttacagtgcagaatttgcatt

>H5N8_A_mallard_Georgia_DT_22362_2020

atggagaacatagtacttcttcttgcaatagttagccttgttaaaagtgatcagatttgcattggttaccatgcaaacaattcgacagagcaagttgacacgataatggaaaagaacgtcactgttacacatgcccaagacatactggaaaaaacacacaacgggaagctctgtgatctaaatggggtgaagcctctgattttaaaggattgtagtgtagctggatggctcctcggaaacccaatgtgcgacgaattcatcagagtgccggaatggtcctacatagtggagagggctaatccatctaatgacctctgttacccagggagcctcaatgactatgaagaactgaaacacctgttgagcagaataaatcattttgagaagattctgatcatccccaagagttcctggccaaaccatgaaacatcactaggggtgagcgcagcttgtccataccagggagcgccctcctttttcagaaatgtggtgtggcttatcaaaaagaacgatgcatacccaacaataaagataagctacaataataccaatcgggaagatctcttgatactgtgggggattcatcattccaacaatgcagaagagcagacaaatctctataaaaacccaaccacctacatttcagttggaacatcaactttaaaccagaggttggtaccaaaaatagctactagatcccaagtaaacgggcaacgtggaagaatggacttcttctggacaattttaaaaccggatgatgcaatccatttcgagagtaatggaaatttcattgctccagaatatgcatacaaaattgtcaagaaaggggactcaacaattatgaaaagtggagtggaatatggccactgcaacaccaaatgtcaaaccccagtaggagcgataaattctagtatgccattccacaacatacatcctctcaccattggggaatgccccaaatacgtgaagtcaaacaagttggtccttgcgactgggctcagaaatagtcctctaagagaaGGGaagagaagaaaaagaggcctgtttggggcgatagcagggtttatagagggaggatggcagggaatggttgatggttggtatgggtaccaccatagcaatgagcagggaagtgggtacgctgcagacaaagagtccacccaaaaggcaatagatggagttaccaataaggtcaactcaatcattgacaaaatgaacactcaatttgaggcagttggaagggagtttaataacttagaaaggaggatagagaatttgaacaagaaaatggaagacggattcctagatgtctggacctataatgctgaacttctagttctcatggaaaacgaaaggactctagatttccatgattcaaatgtcaagaacctttacgacaaagtcagactacagcttagggataatgcaaaggagctgggtaatggctgtttcgaattctatcacaaatgcgataatgaatgtatggaaagtgtgagaaatgggacgtatgactaccctcagtattcagaagaagcaagattaaaaagagaagaaataagcggagtgaaattagaatcaataggaacttaccagatactgtcaatttattcaacagcggcgagttccctagcactggcaatcatgatggctggtctatctttatggatgtgctccaatgggtcgttacagtgcagaatttgcatt

>H5N3_A_green_winged_teal_Georgia_DT_22894_2020

atggagaacatagtacttcttcttgcaatagttagccttgttaaaagtgatcagatttgcattggttaccatgcaaacaattcgacagagcaagttgacacgataatggaaaagaacgtcactgttacacatgcccaagacatactggaaaaaacacacaacgggaagctctgtgatctaaatggggtgaagcctctgattttaaaggattgtagtgtagctggatggctcctcggaaacccaatgtgcgacgaattcatcagagtgccggaatggtcctacatagtggagagggctaatccatctaatgacctctgttacccagggagcctcaatgactatgaagaactgaaacacctgttgagcagaataaatcattttgagaagattctgatcatccccaagagttcctggccaaaccatgaaacatcactaggggtgagcgcagcttgtccataccagggagcgccctcctttttcagaaatgtggtgtggcttatcaaaaagaacgatgcatacccaacaataaagataagctacaataataccaatcgggaagatctcttgatactgtgggggattcatcattccaacaatgcagaagagcagacaaatctctataaaaacccaaccacctacatttcagttggaacatcaactttaaaccagaggttggtaccaaaaatagctactagatcccaagtaaacgggcaacgtggaagaatggacttcttctggacaattttaaaaccggatgatgcaatccatttcgagagtaatggaaatttcattgctccagaatatgcatacaaaattgtcaagaaaggggactcaacaattatgaaaagtggagtggaatatggccactgcaacaccaaatgtcaaaccccagtaggagcgataaattctagtatgccattccacaacatacatcctctcaccattggggaatgccccaaatacgtgaagtcaaacaagttggtccttgcgactgggctcagaaatagtcctctaagagaaGGGaagagaagaaaaagaggcctgtttggggcgatagcagggtttatagagggaggatggcagggaatggttgatggttggtatgggtaccaccatagcaatgagcagggaagtgggtacgctgcagacaaagagtccacccaaaaggcaatagatggagttaccaataaggtcaactcaatcattgacaaaatgaacactcaatttgaggcagttggaagggagtttaataacttagaaaggaggatagagaatttgaacaagaaaatggaagacggattcctagatgtctggacctataatgctgaacttctagttctcatggaaaacgaaaggactctagatttccatgattcaaatgtcaagaacctttacgacaaagtcagactacagcttagggataatgcaaaggagctgggtaacggctgtttcgaattctatcacaaatgcgataatgaatgtatggaaagtgtgagaaatgggacgtatgactaccctcagtattcagaagaagcaagattaaaaagagaagaaataagcggagtgaaattagaatcaataggaacttaccagatactgtcaatttattcaacagcggcgagttccctagcactggcaatcatgatggctggtctatctttatggatgtgctccaatgggtcgttacagtgcagaatttgcatt

>H5N8_A_chicken_Czech_Republic_1566_1_2021

atggagaacatagtacttcttcttgcaatagttagccttgttaaaagtgatcagatttgcattggttaccatgcaaacaattcgacagagcaagttgacacgataatggaaaagaacgtcactgttacacatgcccaagacatactggaaaaaacacacaacgggaagctctgtgatctaaatggggtgaagcctctgattttaaaggattgtagtgtagctggatggctcctcggaaacccaatgtgcgacgaattcatcagagtgccggaatggtcctacatagtggagagggctaatccatctaatgacctctgttacccagggagcctcaatgactatgaagaactgaaacacctgttgagcagaataaatcattttgagaagattctgatcatccccaagagttcctggccaaaccatgaaacatcactaggggtgagcgcagcttgtccataccagggagcgccctcctttttcagaaatgtggtgtggcttatcaaaaagaacgatgcatacccaacaataaagataagctacaataataccaatcaggaagatctcttgatactgtgggggattcatcattccaacaatgcagaagagcagacaaatctctataaaaacccaaccacctacatttcagttggaacatcaactttaaaccagaggttggtaccaaaaatagctactagatcccaagtaaacgggcaacgtggaagaatggacttcttctggacaattttaaaaccggatgatgcaatccatttcgagagtaatggaaatttcattgctccagaatatgcatacaaaattgtcaagaaaggggactcaacaattatgaaaagtggagtggaatatggccactgcaacactaaatgtcaaaccccagtaggagcgataaattctagtatgccattccacaacatacatcctctcaccattggggaatgccccaaatacgtgaagtcaaacaagttggtccttgcgactgggctcagaaatagtcctctaagagaaGGGaagagaagaaaaagaggcctgtttggggcgatagcagggtttatagagggaggatggcagggaatggttgatggttggtatgggtaccaccatagcaatgagcaggggagtgggtacgctgcagacaaagaatccacccaaaaggcaatagatggagttaccaataaggtcaactcaatcattgacaaaatgaacactcaatttgaggcagttggaagggagtttaataacttagaaaggaggatagagaatttgaacaagaaaatggaagacggattcctagatgtctggacctataatgctgaacttctagttctcatggaaaacgaaaggactctagatttccatgattcaaatgtcaagaacctttacgacaaagtcagactacagcttagggataatgcaaaggaactgggtaacggctgtttcgaattctatcacaaatgcgataatgaatgtatggaaagtgtgagaaatgggacgtatgactaccctcagtattcaggagaagcaagattaaaaagagaagaaataagcggagtgaaactagaatcaataggaacttaccagatactgtcaatttattcaacagcggcgagttccctagcactggcaatcatgatggctggtctatctttatggatgtgctccaatgggtcgttacagtgcagaatttgcatt

>H5N8_A_chicken_Krasnodar_334_02_2021

atggagaacatagtacttcttcttgcaatagttagccttgttaaaagtgatcagatttgcattggttaccatgcaaacaattcgacagagcaagttgacacgataatggaaaagaacgtcactgttacacatgcccaagacatactggaaaaaacacacaacgggaagctctgtgatctaaatggggtgaagcctctgattttaaaggattgtagtgtagctggatggctcctcggaaacccaatgtgcgacgaattcatcagagtgccggaatggtcctacatagtggagagggctaatccagctaatgacctctgttacccagggagcctcaatgactatgaagaactgaaacacctgttgagcagaataaatcattttgagaagattctgatcatccccaagagttcctggccaaatcatgaaacatcactaggggtgagcgcagcttgtccataccagggagcgccctcctttttcagaaatgtggtgtggcttatcaaaaagaacgatgcatacccaacaataaagataagctacaataataccaatcgggaagatctcttgatactgtgggggattcatcattccaacaatgcagaagagcagacaaatctctataaaaacccaaccacctacatttcagttggaacatcaactttaaaccagaggttggtaccaaaaatagctactagatcccaagtaaacgggcaacgtgggagaatggacttcttctggacaattttaaaaccggatgatgcaatccatttcgagagtaatggaaatttcattgctccagaatatgcatacaaaattgtcaagaaaggggactcaacaattatgaaaagtggagtggagtatggccactgcaacgccaaatgtcaaaccccagtaggagcgataaattctagtatgccattccacaacatacatcctcttaccattggggaatgccccaaatacgtgaagtcaaacaagttggtccttgcgactgggctcagaaatagtcctctaagagaaGGGaagagaagaaaaagaggcctgtttggggcgatagcagggtttatagagggaggatggcagggaatggttgatggttggtatgggtaccaccatagcaatgagcaggggagtgggtacgctgcagacaaagaatccacccaaaaggcaatagatggagttaccaataaggtcaactcaatcattgacaaaatgaacactcaatttgaggcagtcggaagggagtttaataacttagaaaggaggatagagaatttgaacaagaaaatggaagacggattcctagatgtctggacctataatgctgaacttctagttctcatggaaaacgagaggactctagatttccatgattcaaatgtcaaaaacctttacgacaaagtcagactacagcttagggataatgcaaaggagctgggtaacggctgtttcgaattctatcacaaatgcgataatgaatgtatggaaagtgtgagaaatgggacgtatgactaccctcagtattcagaagaagcaagattaaaaagagaagaaataagcggagtgaaattagaatcaataggaacttaccagatactgtcaatttattcaacagcggcgagttccctagcactggcaatcatgatggctggtctatctttatggatgtgctccaatgggtcgttacagtgcagaatttgcatt

>H5N8_A_chicken_Kosovo_82_21VIR5162_1_2021

atggagaacatagtacttcttcttgcaatagttagccttgttaaaagtgatcagatttgcattggttaccatgcaaacaattcgacagagcaagttgacacgataatggaaaagaacgtcactgttacacatgcccaagacatactggaaaaaacacacaacgggaagctctgtgatctaaatggggtgaagcctctgattttaaaggattgtagtgtagctggatggctcctcggaaacccaatgtgcgacgaattcatcagagtgccggaatggtcctacatagtggagagggctaatccagctaatgacctctgttacccagggagcctcaatgactatgaagaactgaaacacctgttgagcagaataaatcattttgagaagattctgatcatccccaagagttcctggccaaatcatgaaacatcactgggggtgagcgcagcttgtccataccagggagcgccctcctttttcagaaatgtggtgtggcttatcaaaaagaacgatgcatacccaacaataaagataagctacaataataccaatcgggaagatctcttgatactgtgggggattcatcattccaacaatgcagaagagcagacaaatctctataaaaacccaaccacctacatttcagttggaacatcaactttaaaccagaggttggtaccaaaaatagctactagatcccaagtaaacgggcaacgtgggagaatggacttcttctggacaattttaaaaccggatgatgcaatccatttcgagagtaatggaaatttcattgctccagaatatgcatacaaaattgtcaagaaaggggactcaacaattatgaaaagtggagtggagtatggccactgcaacaccaaatgtcaaaccccagtaggagcgataaattctagtatgccattccacaacatacatcctctcaccattggggaatgccccaaatacgtgaagtcaaacaagttggtccttgcgactgggctcagaaatagtcctctaagagaaGGGaagagaagaaaaagaggcctgtttggggcgatagcagggtttatagagggaggatggcagggaatggttgatggttggtatgggtaccaccatagcaatgagcaggggagtgggtacgctgcagacaaagaatccacccaaaaggcaatagatggagttaccaataaggtcaactcaatcattgacaaaatgaacactcaatttgaggcagttggaagggagtttaataacttagaaaggaggatagagaatttgaacaagaaaatggaagacggattcctagatgtctggacctataatgctgaacttctagttctcatggaaaacgagaggactctagatttccatgattcaaatgtcaaaaacctttacgacaaagtcagactacagcttagggataatgcaaaggagctgggtaacggctgtttcgaattctatcacaaatgcgataatgaatgtatggaaagtgtgagaaatgggacgtatgactaccctcagtattcagaagaagcaagattaaaaagagaagaaataagcggagtgaaattagaatcaataggaacttaccagatactgtcaatttattcaacagcggcgagttccctagcactggcaatcatgatggctggtctatctttatggatgtgctccaatgggtcgttacagtgcagaatttgcatt

>H5N8_A_mute_swan_Croatia_14_2021

atggagaacatagtacttcttcttgcaatagttagccttgttaaaagtgatcagatttgcattggttaccatgcaaacaattcgacagagcaagttgacacgataatggaaaagaacgtcactgttacacatgcccaagacatactggaaaaaacacacaacgggaagctctgtgatctaaatggggtgaagcctctgattttaaaggattgtagtgtagctggatggctcctcggaaacccaatgtgcgacgaattcatcagagtgccggaatggtcctacatagtggagagggctaatccagctaatgacctctgttacccagggagcctcaatgactatgaagaactgaaacacctgttgagcagaataaatcattttgagaagattctgatcatccccaagagttcctggccaaatcatgaaacatcactaggggtgagcgcagcttgtccataccagggagcgccctcctttttcagaaatgtggtgtggcttatcaaaaagaacgatgcatacccaacaataaagataagctacaataataccaatcgggaagatctcttgatactgtgggggattcatcattccaacaatgcagaagagcagacaaatctctataaaaacccaaccacctacatttcagttggaacatcaactttaaaccagaggttggtaccaaaaatagctactagatcccaagtaaacgggcaacgtgggagaatggacttcttctggacaattttaaaaccggatgatgcaatccatttcgagagtaatggaaatttcattgctccagaatatgcatacaaaattgtcaagaaaggggactcaacaattatgaaaagtggagtggagtatggccactgcaacaccaaatgtcaaaccccagtaggagcgataaattctagtatgccattccacaacatacatcctctcaccattggggaatgccccaaatacgtgaagtcaaacaagttggtccttgcgactgggctcagaaatagtcctctaagagaaGGGaagagaagaaaaagaggcctgtttggggcgatagcagggtttatagagggaggatggcagggaatggttgatggttggtatgggtaccaccatagcaatgagcaggggagtgggtacgctgcagacaaagaatccacccaaaaggcaatagatggagttaccaataaggtcaactcaatcattgacaaaatgaacactcaatttgaggcagttggaagggagtttaataacttagaaaggaggatagagaatttgaacaagaaaatggaagacggattcctagatgtctggacctataatgctgaacttctagttctcatggaaaacgagaggactctagatttccatgattcaaatgtcaaaaacctttacgacaaagtcagactacagcttagggataatgcaaaggagctgggtaacggctgtttcgaattctatcacaaatgcgataatgaatgtatggaaagtgtgagaaatgggacgtatgactaccctcagtattcagaagaagcaagattaaaaagagaagaaataagcggagtgaaattagaatcaataggaacttaccagatactgtcaatttattcaacagcggcgagttccctagcactggcaatcatgatggctggtctatctttatggatgtgctccaatgggtcgttacagtgcagaatttgcatt

>H5N8_A_chicken_Bulgaria_275_4_21VIR4270_6_2021

atggagaacatagtacttcttcttgcaatagttagccttgttaaaagtgatcagatttgcattggttaccatgcaaacaattcgacagagcaagttgacacgataatggaaaagaacgtcactgttacacatgcccaagacatactggaaaaaacacacaacgggaagctctgtgatctaaatggggtgaagcctctgattttaaaggattgtagtgtagctggatggctcctcggaaacccaatgtgcgacgaattcatcagagtgccggaatggtcctacatagtggagagggctaatccagctaatgacctctgttacccagggagcctcaatgactatgaagaactgaaacacctgttgagcagaataaatcattttgagaaaattctgatcatccccaagagttcctggccaaatcatgaaacatcactaggggtgagcgcagcttgtccataccagggagcgccctcctttttcagaaatgtggtgtggcttatcaaaaagaatgatgcatacccaacaataaagataagctacaataataccaatcgggaagatctcttgatactgtgggggattcatcattccaacaatgcagaagagcagacaaatctctataaaaacccaaccacctacatttcagttggaacatcaactttaaaccagaggttggtaccaaaaatagctactagatcccaagtaaacgggcaacgtgggagaatggacttcttctggacaattttaaaaccggatgatgcaatccatttcgagagtaatggaaatttcattgctccagaatatgcatacaaaattgtcaagaaaggggactcaacaattatgaaaagtggagtggagtatggccactgtaacaccaaatgtcaaaccccagtaggagcgataaattctagtatgccattccacaacatacatcctctcaccattggggaatgccccaaatacgtgaagtcaaacaagttggtccttgcgactgggctcagaaataatcctctaagagaaGGGaagagaagaaaaagaggcctgtttggggcgatagcagggtttatagagggaggatggcagggaatggttgatggttggtatgggtaccaccatagcaatgagcaggggagtgggtacgctgcagacaaagaatccacccaaaaggcaatagatggagttaccaataaggtcaactcaatcattgacaaaatgaacactcaatttgaggcagttggaagggagtttaataacttagaaaggaggatagagaatttgaacaagaaaatggaagacggattcctagatgtctggacctataatgctgaacttctagttctcatggaaaacgagaggactctagatttccatgattcaaatgtcaaaaacctttacgacaaagtcagactacagcttagggataatgcaaaggagctgggtaacggctgtttcgaattctatcacaaatgcgataatgaatgtatggaaagtgtgagaaatgggacgtatgactaccctcagtattcagaagaagcaagattaaaaagagaagaaataagcggagtgaaattagaatcaataggaacttaccagatactgtcaatttattcaacagcggcgagttccctagcactggcaatcatgatggctggtctatctttatggatgtgctccaatgggtcgttacagtgcagaatttgcatt

>H5N8_A_chicken_Albania_2574_21VIR5387_1_2021

atggagaacatagtacttcttcttgcaatagttagccttgttaaaagtgatcagatttgcattggttaccatgcaaacaattcgacagagcaagttgacacgataatggaaaagaacgtcactgttacacatgcccaagacatactggaaaaaacacacaacgggaagctctgtgatctaaacggggtgaagcctctgattttaaaggattgtagtgtagctggatggctcctcggaaacccaatgtgcgacgaattcatcagagtgccggaatggtcctacatagtggagagggctaatccagctaatgacctctgttacccagggagcctcaatgactatgaagaactgaaacacctgttgagcagaataaatcattttgagaagattctgatcatccccaagagttcctggccaaatcatgaaacatcactaggggtgagcgcagcttgtccataccagggagcgccctcctttttcagaaatgtggtgtggcttatcaaaaagaacgatgcatacccaacaataaagataagctacaataataccaatcgggaagatctcttgatactgtgggggattcatcattccaacaatgcagaagagcagacaaatctctataaaaacccaaccacctacatttcagttggaacatcaactttaaaccagaggttggtaccaaaaatagctactagatcccaagtaaacgggcaacgtgggagaatggacttcttctggacaattttaaaaccggatgatgcaatccatttcgagagtaatggaaatttcattgctccagaatatgcatacaaaattgtcaagaaaggggactcaacaattatgaaaagtggagtggagtatggccactgcaacaccaaatgtcaaaccccagtaggagcgataaattctagtatgccattccacaacatacatcctctcaccattggggaatgccccaaatacgtgaagtcaaacaagttggtccttgcgactgggctcagaaatagtcctctaagagaaGGGaagagaagaaaaagaggcctgtttggggcgatagcagggtttatagagggaggatggcagggaatggttgatggttggtatgggtaccaccatagcaatgagcaggggagtgggtacgctgcagacaaagaatccacccaaaaggcaatagatggagttaccaataaggtcaactcaatcattgacaaaatgaacactcaatttgaggcagttggaagggagtttaataacttagaaaggaggatagagaatttgaacaagaaaatggaagacggattcctagatgtctggacctataatgctgaacttctagttctcatggaaaacgagaggactctagatttccatgattcaaatgtcaaaaacctttacgacaaagtcagactacagcttagggataatgcaaaggagctgggtaacggctgtttcgaattctatcacaaatgcgataatgaatgtatggaaagtgtgagaaatgggacgtatgactaccctcagtattcagaagaagcaagattaaaaagagaagaaataagcggagtgaaattagaatcaatagggacttaccagatactgtcaatttattcaacagcggcgagttccctagcactggcaatcatgatggctggtctatctttatggatgtgctccaatgggtcgttacagtgcagaatttgcatt

>H5N8_A_mute_swan_Austria_21051907_21VIR3291_6_2021

atggagaacatagtacttcttcttgcaatagttagccttgttaaaagtgatcagatttgcattggttaccatgcaaacaattcgacagagcaagttgacacgataatggaaaagaacgtcactgttacacatgcccaagacatactggaaaaaacacacaacgggaagctctgtgatctaaatggggtgaagcctctgattttaaaggattgtagtgtagctggatggctcctcggaaacccaatgtgcgacgaattcatcagagtgccggaatggtcctacatagtggagagggctaatccagctaatgacctctgttacccagggagcctcaatgactatgaagaactgaaacacctgttgagcagaataaatcattttgagaagattctgatcatccccaagagttcctggccaaatcatgaaacatcactaggggtgagcgcagcttgtccataccagggagcgccctcctttttcagaaatgtggtgtggcttatcaaaaagaacgatgcatacccaacaataaagataagctacaataataccaatcgggaagatctcttgatactgtgggggattcatcattccaacaatgcagaagagcagacaaatctctataaaaacccaaccacctacatttcagttggaacatcaactttaaaccagaggttggtaccaaaaatagctactagatcccaagtaaacgggcaacgtgggagaatggacttcttctggacaattttaaaaccggatgatgcaatccatttcgagagtaatggaaatttcattgctccagaatatgcatacaaaattgtcaagaaaggggactcaacaattatgaaaagtggagtggaatatggccactgcaacaccaaatgtcaaaccccagtaggagcgataaattctagtatgccattccacaacatacatcctctcaccattggggaatgccccaaatacgtgaagtcaaacaagttggtccttgcgactgggctcagaaatagtcctctaagagaaGGGaagagaagaaaaagaggcctgtttggggcgatagcagggtttatagagggaggatggcagggaatggttgatggttggtatgggtaccaccatagcaatgagcaggggagtgggtacgctgcagacaaagaatccacccaaaaggcaatagatggtgttaccaataaggtcaactcaatcattgacaaaatgaacactcaatttgaggcagttggaagggagtttaataacttagaaaggaggatagagaatttgaacaagaaaatggaagacggattcctagatgtctggacctataatgctgaacttctagttctcatggaaaacgagaggactctagatttccatgattcaaatgtcaaaaacctttacgacaaagtcagactacagcttagggataatgcaaaggagctgggtaacggctgtttcgaattctatcacaaatgcgataatgaatgtatggaaagtgtgagaaatgggacgtatgactaccctcagtattcagaagaagcaagattaaaaagagaagaaataagcggagtgaaactagaatcaataggaacttaccagatactgtcaatttattcaacagcggcgagttccctagcactggcaatcatgatggctggtctatctttatggatgtgctccaatgggtcgttacagtgcagaatttgcatt

>H5N8_A_chicken_Germany_NI_AI02954_2021

atggagaacatagtacttcttcttgcaatagttagccttgttaaaagtgatcagatttgcattggctaccatgcaaacaattcgacagagcaagttgacacgataatggaaaagaacgtcactgttacacatgcccaagacatactggaaaaaacacacaacgggaagctctgtgatctaaatggggttaagcctctgattttaaaggattgtagtgtagctggatggctcctcggaaacccaatgtgcgacgaattcatcagagtgccggaatggtcctacatagtggagagggctaatccagctaatgacctctgttacccagggagcctcaatgactatgaagaactgaaacacctgttgagcagaataaatcattttgagaagattctgatcatccccaagagttcctggccaaatcatgaaacatcactaggggtgagcgcagcttgtccataccagggagcgccctcctttttcagaaatgtggtgtggcttatcaaaaagaacgatgcatacccaacaataaagataagctacaataataccaatcrggaagatctcttgatactgtgggggattcatcattccaacaatgcagaagagcagacaaatctctataaaaacccaaccacctacatttcagttggaacatcaactttaaaccagaggttggtaccaaaaatagctactagatcccaagtaaacgggcaacgtgggagaatggacttcttctggacaattttaaaaccggatgatgcaatccatttcgagagtaatggaaatttcattgctccagaatatgcatacaaaattgtcaagaaaggggactcaacaattatgaaaagtggagtggaatatggccactgcaacaccaaatgtcaaaccccagtaggagcgataaattctagtatgccattccacaacatacatcctctcaccattggggaatgccccaaatacgtgaagtcaaacaagttggtccttgcgactgggctcagaaatagtcctctaagagaaGGGaagagaagaaaaagaggcctgtttggggcgatagcagggtttatagagggaggatggcagggaatggttgatggttggtatgggtaccaccatagcaatgagcaggggagtgggtacgctgcagacaaagaatccacccaaaaggcaatagatggagttaccaataaggtcaactcaatcattgacaaaatgaacactcaatttgaggcagttggaagggagtttaataacttagaaaggaggatagagaatttgaacaagaaaatggaagacggattcctagatgtctggacctataatgctgaacttctagtcctcatggaaaacgagaggactctagatttccatgattcaaatgtcaaaaacctttacgacaaagtcagactacagcttagggataatgcaaaggagctgggtaacggctgtttcgaattctatcacaaatgcgataatgaatgcatggaaagtgtgagaaatgggacgtatgactaccctcagtattcagaagaagcaagattaaaaagagaagaaataagcagagtgaaattagaatcaataggaacttaccagatactgtcaatttattcaacagcggcgagttccctagcactggcaatcatgatggctggtctatctttatggatgtgctccaatgggtcgttacagtgcagaatttgcatt

>H5N8_A_turkey_Norway_FU496_2020

atggagaacatagtacttcttcttgcaatagttagccttgttaaaagtgatcagatttgcattggttaccatgcaaacaattcgacagagcaagttgacacgataatggaaaagaacgtcactgttacacatgcccaagacatactggaaaaaacacacaacgggaagctctgtgatctaaatggggtgaagcctctgattttaaaggattgtagtgtagctggatggctcctcggaaacccaatgtgcgacgaattcatcagagtgccggaatggtcctacatagtggagagggctaatccagctaatgacctctgttacccagggagcctcaatgactatgaagaactgaaacacctgttgagcagaataaatcattttgagaagattctgatcatccccaagagttcctggccaaatcatgaaacatcactaggggtgagcgcagcttgtccataccagggagcgccctcctttttcagaaatgtggtgtggcttatcaaaaagaacgatgcatacccaacaataaagataagctacaataataccaatcgggaagatctcttgatactgtgggggattcatcattccaacaatgcagaagagcagacaaatctctataaaaacccaaccacctacatttcagttggaacatcaactttaaaccagaggttggtaccaaaaatagctactagatcccaagtaaacgggcaacgtggaagaatggacttcttctggacaattttaaaaccggatgatgcaatccatttcgagagtaatggaaatttcattgctccagaatatgcatacaaaattgtcaagaaaggggactcaacaattatgaaaagtggagtggaatatggccactgcaacaccaaatgtcaaaccccagtaggagcgataaattctagtatgccattccacaacatacatcctctcaccattggggaatgccccaaatacgtgaagtcaaacaagttggtccttgcgactgggctcagaaatagtcctctaagagaaGGGaagagaagaaaaagaggcctgtttggggcgatagcagggtttatagagggaggatggcagggaatggttgatggttggtatgggtaccaccatagcaatgagcaggggagtggttacgctgcagacaaagaatccacccaaaaggcaatagatggagttaccaataaggtcaactcaatcattgacaaaatgaacactcaatttgaggcagttggaagggagtttaataacttagaaaggaggatagagaatttgaacaagaaaatggaagacggattcctagatgtctggacctataatgctgaacttctagttctcatggaaaatgagaggactctagatttccatgattcaaatgtcaaaaacctttacgacaaagtcagactacagcttagggataatgcaaaggagctgggtaacggctgtttcgaattctatcacaaatgcgataatgaatgtatggaaagtgtgagaaatgggacgtatgactaccctcagtattcagaagaagcaagattaaaaagagaagaaataagcggagtgaaattagaatcaataggaacttaccagatactgtcaatttattcaacagcggcgagttccctagcactggcaatcatgatggctggtctatctttatggatgtgctccaatgggtcgttacagtgcagaatttgcatt

>H5N8_A_goose_Romania_10205_t5_21VIR2593_8_2021

atggagaacatagtacttcttcttgcaatagttagccttgttaaaagtgatcagatttgcattggttaccatgcaaacaattcgacagagcaagttgacacgataatggaaaagaacgtcactgttacacatgcccaagacatactggaaaaaacacacaacgggaagctctgtgatctaaatggggtgaagcctctgattttaaaggattgtagtgtagctggatggctcctcggaaacccaatgtgcgacgaattcatcagagtgccggaatggtcctacatagtggagagggctaatccagctaatgacctctgttacccagggagcctcaatgactatgaagaactgaaacacctgttgagcagaataaatcattttgagaagattctgatcatccccaaaagttcctggccaaatcatgaaacatcactaggggtgagcgcagcttgtccataccagggagcaccctcctttttcagaaatgtggtgtggcttatcaaaaagaacgatgcatacccaacaataaagataagctacaataataccaatcgggaagatctcttgatactgtgggggattcatcattccaacaatgcagaagagcagacaaatctctataaaaacccaaccacctacatttcagttggaacatcaactttaaaccagaggttggtaccaaaaatagctactagatcccaagtaaacgggcaacgtggaagaatggacttcttctggacaattttaaaaccggatgatgcaatccatttcgagagtaatggaaatttcattgctccagaatatgcatacaaaattgtcaagaaaggggactcaacaattatgaaaagtggagtggaatatggccactgcaacaccaaatgtcaaaccccagtaggagcgataaattctagtatgccattccacaacatacatcctctcaccattggggaatgccccaaatacgtgaagtcaaacaagttggtccttgcgactgggctcagaaatagtcctctaagagaaGGGaagagaagaaaaagaggcctgtttggggcgatagcagggtttatagagggaggatggcagggaatggttgatggttggtatgggtaccaccatagcaatgagcaggggagtgggtacgctgcagacaaagaatccacccaaaaggcaatagatggagttaccaataaggtcaactcaatcattgacaaaatgaacactcaatttgaggcagttggaagagagtttaataacttagaaaggaggatagagaatttgaacaagaaaatggaagacggattcctagatgtctggacctataatgctgaacttctagttctcatggaaaacgagaggactctagatttccatgattcaaatgtcaaaaacctttacgacaaagtcagactacagcttagggataatgcaaaggagcttggtaacggctgtttcgaattctatcacaaatgcgataatgaatgtatggaaagtgtgagaaatgggacgtatgactaccctcagtattcagaagaagcaagattaaaaagagaagaaataagcggagtgaaattagaatcaataggaacttaccagataatgtcaatttattcaacagcggcgagttccctagcactggcaatcatgatggctggtctatctttatggatgtgctccaatgggtcgttacagtgcagaatttgcatt

>H5N8_A_duck_Romania_10206_21VIR849_4_2021

atggagaacatagtacttcttcttgcaatagttagccttgttaaaagtgatcagatttgcattggttaccatgcaaacaattcgacagagcaagttgacacgataatggaaaagaacgtcactgttacacatgcccaagacatactggaaaaaacacacaacgggaagctctgtgatctaaatggggtgaagcctctgattttaaaggattgtagtgtagctggatggctcctcggaaacccaatgtgcgacgaattcatcagagtgccggaatggtcctacatagtggagagggctaatccagctaatgacctctgttacccagggagcctcaatgactatgaagaactgaaacacctgttgagcagaataaatcattttgagaagattctgatcatccccaaaagttcctggccaaatcatgaaacatcactaggggtgagcgcagcttgtccataccagggagcaccctcctttttcagaaatgtggtgtggcttatcaaaaagaacgatgcatacccaacaataaagataagctacaataataccaatcgggaagatctcttgatactgtgggggattcatcattccaacaatgcagaagagcagacaaatctctataaaaacccaaccacctacatttcagttggaacatcaactttaaaccagaggttggtaccaaaaatagctactagatcccaagtaaacgggcaacgtggaagaatggacttcttctggacaattttaaaaccggatgatgcaatccatttcgagagtaatggaaatttcattgctccagaatatgcatacaaaattgtcaagaaaggggactcaacaattatgaaaagtggagtggaatatggccactgcaacaccaaatgtcaaaccccagtaggagcgataaattctagtatgccattccacaacatacatcctctcaccattggggaatgccccaaatacgtgaagtcaaacaagttggtccttgcgactgggctcagaaatagtcctctaagagaaGGGaagagaagaaaaagaggcctgtttggggcgatagcagggtttatagagggaggatggcagggaatggttgatggttggtatgggtaccaccatagcaatgagcaggggagtgggtacgctgcagacaaagaatccacccaaaaggcaatagatggagttaccaataaggtcaactcaatcattgacaaaatgaacactcaatttgaggcagttggaagagagtttaataacttagaaaggaggatagagaatttgaacaagaaaatggaagacggattcctagatgtctggacctataatgctgaacttctagttctcatggaaaacgagaggactctagatttccatgattcaaatgtcaaaaacctttacgacaaagtcagactacagcttagggataatgcaaaggagcttggtaacggctgtttcgaattctatcacaaatgcgataatgaatgtatggaaagtgtgagaaatgggacgtatgactaccctcagtattcagaagaagcaagattaaaaagagaagaaataagcggagtgaaattagaatcaataggaacttaccagataatgtcaatttattcaacagcggcgagttccctagcactggcaatcatgatggctggtctatctttatggatgtgctccaatgggtcgttacagtgcagaatttgcatt

>H5N8_A_chicken_Denmark_14819_6_2020

atggagaacatagtacttcttcttgcaatagttagccttgttaaaagtgatcagatttgcattggttaccatgcaaacaattcgacagagcaagttgacacgataatggaaaagaacgtcactgttacacatgcccaagacatactggaaaaaacacacaacgggaagctctgtgatctaaatggggtgaagcctctgattttaaaggattgtagtgtagctggatggctcctcggaaacccaatgtgcgacgaattcatcagagtgccggaatggtcctacatagtggagagggctaatccagctaatgacctctgttacccagggagcctcaatgactatgaagaactgaaacacctgttgagcagaataaatcattttgagaagattctgatcatccccaagagttcctggccgaatcatgaaacatcactaggggtgagcgcagcttgcccataccagggagcgccctcctttttcagaaatgtggtgtggcttatcaaaaagaacgatgcatacccaacaataaagataagctacaataataccaatcgggaagatctcttgatactgtgggggattcatcattccaacaatgcagaagagcagacaaatctctataaaaacccaaccacctacatttcagttggaacatcaactttaaaccagaggttggtaccaaaaatagctactagatcccaagtaaacgggcaacgtggaagaatggacttcttctggacaattttaaaaccggatgatgcaatccatttcgagagtaatggaaatttcattgctccagaatatgcatacaaaattgtcaagaaaggggactcaacaattatgaaaagtggagtggaatatggccactgcaacaccaaatgtcaaaccccagtaggagcgataaattctagtatgccattccacaacatacatcctctcaccattggggaatgccccaaatacgtgaaatcaaacaagttggtccttgcgactgggctcagaaatagtcctctaagagaaGGGaagagaagaaaaagaggcctgtttggggcgatagcagggtttatagagggaggatggcagggaatggttgatggttggtatgggtaccaccatagcaatgagcaggggagtgggtacgctgcagacaaagaatccacccaaaaggcaatagatggagttaccaataaggtcaactcaatcattgacaaaatgaacactcaatttgaggcagttggaagggagtttaataacttagaaaggaggatagagaatttgaacaagaaaatggaagacggattcctagatgtctggacctataatgctgaacttctagttctcatggaaaacgagaggactctagatttccatgattcaaatgtcaaaaacctttacgacaaagtcagactacagcttagggataatgcaaaggagctgggtaacggctgtttcgaattctatcacaaatgcgataatgaatgtatggaaagtgtgagaaatgggacgtatgactaccctcagtattcagaagaagcaagattaaaaagagaagaaataagcggagtgaaattagaatcaataggaacttaccagatactgtcaatttattcaacagcggcgagttccctagcactggcaatcatgatggctggtctatctttatggatgtgctccaatgggtcgttacagtgcagaatttgcatt

>H5N5_A_black_necked_grebe_Kalmykia_78_1V_2021

atggagaacatagtacttcttcttgcaatagttagccttgttaaaagtgatcagatttgcattggttaccatgcaaacaattcgacagagcaagttgacacgataatggaaaagaacgtcactgttacacatgcccaagacatactggaaaaaacacacaacgggaagctctgtgatctaaatggggtgaagcctctgattttaaaggattgtagtgtagctggatggctcctcggaaacccaatgtgcgacgaattcatcagagtgccggaatggtcttacatagtggagagggctaatccagctaatgacctctgctacccagggagcctcaatgactatgaagaactgaaacacctgttgagccgaataaatcattttgagaagattctgatcatccccaagggttcctggccaaatcatgaaacatcactaggggtgagcgcagcttgtccataccagggagcgccctcctttttcagaaatgtggtgtggcttatcaaaaagaacgatgcatacccaacaataaagataagctacaataataccaatcgggaagatctcttgatactgtgggggattcatcattccaacaatgcagaagagcagacaaatctctataaaaacccaaccacctacatttcagttggaacatcaactttaaaccagaggttggtaccaaaaatagctactagatcccaagtaaacgggcaacgtggaagaatggacttcttctggacaattttaaaaccggatgatgcaatccatttcgagagtaatggaaatttcattgctccagaatatgcatacaaaattgtcaagaaaggggactcaacaattatgaaaagtggagtggaatatggccactgcaacaccaaatgtcaaaccccagtaggagcgataaattctagtatgccattccacaacatacatcctctcaccattggggaatgccccaaatacgtgaagtcaaacaagttggtccttgcaactgggctcagaaatagtcctctaagagaaGGGaagagaagaaaaagaggcctgtttggggcgatagcagggtttatagagggaggatggcagggaatggttgatggttggtatgggtaccaccatagcaatgagcaggggagtgggtacgctgcagacaaagaatccacccaaaaggcaatagatggagttaccaataaggtcaactcaattattgacaaaatgaacactcaatttgaggcagttggaagggagtttaataacttagaaaggaggatagagaatttgaacaagaaaatggaagacggattcctagatgtctggacctataatgctgaacttctagttctcatggaaaacgagaggactctagatttccatgattcaaatgtcaagaacctttacgacaaagtcagactacagcttagggataatgcaaaggagctgggtaacggctgtttcgaattctatcacaaatgcgataatgaatgtatggaaagtgtgagaaatgggacgtatgactactctcagtattcagaagaagcaagattaaaaagagaagaaataagcggagtgaaattagaatcaataggaacttaccagatactgtcaatttattcaacagcggcgagttccctagcactggcaatcatgatggctggtctatctttatggatgtgctccaatgggtcgttacagtgcagaatttgcatt

>H5N5_A_goose_Russian_Federation_Omsk_1680_6_2020

atggagaacatagtacttcttcttgcaatagttagccttgttaaaagtgatcagatttgcattggttaccatgcaaacaattcgacagagcaagttgacacgataatggaaaagaacgtcactgttacacatgcccaagacatactggaaaaaacacacaacgggaagctctgtgatctaaatggggtgaagcctctgattttaaaggattgtagtgtagctggatggctcctcggaaacccaatgtgcgacgaattcatcagagtgccggaatggtcctacatagtggagagggctaatccagctaatgacctctgttacccagggagcctcaatgactatgaagaactgaaacacctgttgagcagaataaatcattttgagaagattctgatcatccccaagagttcctggccaaatcatgaaacagcactaggagtgagcgcagcttgtccataccagggagcgccctcctttttcagaaatgtggtgtggattatcaaaaagaacgatgcatacccaacaataaagataagctacaataataccaatcgggaagatctcttgatactgtgggggattcatcattccaacaatgcagaagagcagacaaatctctataaaaacccaaccacctacatttcagttggaacatcaactttaaaccagaggttggtaccaaaaatagctactagatcccaagtaaacgggcaacgtggaagaatggacttcttctggacaattttaaaaccggatgatgcaatccatttcgagagtaatgggaatttcattgctccagaatatgcatacaaaattgtcaagaaaggggactcaacaattatgaaaagtggagtggaatatggccactgcaacaccaaatgtcaaaccccagtaggagcgataaattctagtatgccattccacaacatacatcctctcaccattggggaatgccccaaatacgtgaagtcaaacaagttggtccttgcgactgggctcagaaatagtcctctaagagaaGGGaagagaagaaaaagaggcctgtttggggcgatagcagggtttatagagggaggatggcagggaatggttgatggttggtatgggtaccaccatagcaatgagcaggggagtgggtacgctgcagacaaagaatccacccaaaaggcaatagatggagttaccaataaggtcaactcaatcattgacaaaatgaacactcaatttgaggcagttggaagggagtttaataacttagaaaggaggatagagaatttgaacaagaaaatggaagacggattcctagatgtctggacctataatgctgaacttctagttctcatggaaaacgagaggactctagatttccatgattcaaatgtcaagaacctttacgacaaagtcagactacagcttagggataatgcaaaggagctgggtaacggctgtttcgaattctatcacaaatgcgataatgaatgtatggaaagtgtgagaaatgggacgtatgactaccctcagtattcagaagaagcaagattaaaaagagaagaaataagcggagtgaaattagaatcaataggaacttaccagatactgtcaatttattcaacagcggcgagttccctagcactggcaatcatgttggctggtctatctttatggatgtgctccaatgggtcgttacagtgcagaatttgcatt

>H5N8_A_chicken_Netherlands_20016978_001_2020

atggagaacatagtacttcttcttgcaatagttagccttgttaaaagtgatcagatttgcattggttaccatgcaaacaattcgacagagcaagttgacacgataatggaaaagaacgtcactgttacacatgcccaagacatactggaaaaaacacacaacgggaagctctgtgatctaaatggagtgaagcctctgattttaaaggattgtagtgtagctggatggctcctcggaaacccaatgtgcgacgaattcatcagagtgccggaatggtcctacatagtggagagggctaatccagctaatgacctctgttacccagggagcctcaatgactatgaagaactgaaacacctgttgagcagaataaatcattttgagaagattctgatcatccccaagagttcctggccaaatcatgaaacagcactaggagtgagcgcagcttgtccataccagggagcgccctcctttttcagaaatgtggtgtggcttatcaaaaagaacgatgcatacccaacaataaagataagctacaataataccaatcgggaagatctcttgatactgtgggggattcatcattccaacaatgcagaagagcagacaaatctctataaaaacccagccacctacatttcagttggaacatcaactttaaaccagaggttggtaccaaaaatagctactagatcccaagtaaacgggcaacgtggaagaatggacttcttctggacaattttaaaaccggatgatgcaatccatttcgagagtaatggaaatttcattgctccagaatatgcatacaaaattgtcaagaaaggggactcaacaattatgaaaagtggagtggaatatggccactgcaacaccaaatgtcaaaccccagtaggagcgataaattctagtatgccattccacaacatacatcctctcaccattggggaatgccccaaatacgtgaagtcaaacaagttagtccttgcgactgggctcagaaatagtcctctaagagaaGGGaagagaagaaaaagaggcctgtttggggcgatagcagggtttatagagggaggatggcagggaatggttgatggttggtatgggtaccaccatagcaatgagcaggggagtgggtacgctgcagacaaagaatccacccaaaaggcaatagatggagttaccaataaggtcaactcaatcattgacaaaatgaacactcaatttgaggcagttggaagggagtttaataacttagaaaggaggatagagaatttgaacaagaaaatggaagacggattcctagatgtctggacctataatgctgaacttctagttctcatggaaaacgagaggactctagatttccatgattcaaatgtcaagaacctttacgacaaagtcagactacagcttagggataatgcaaaggagctgggtaacggctgtttcgaattctatcacaaatgcgataatgaatgtatggaaagtgtgagaaatgggacgtatgactaccctcagtattcagaagaagcaagattaaaaagagaagaaataagcggagtgaaattagaatcaataggaacttaccagatactgtcaatttattcaacagcggcgagttccctagcactggcaatcatgatggctggtctatctttatggatgtgctccaatgggtcgttacagtgcagaatttgcatt

>H5N8_A_duck_Chelyabinsk_1207_1_2020

atggagaacatagtacttcttcttgcaatagttagccttgttaaaagtgatcagatttgcattggttaccatgcaaacaattcgacagagcaagttgacacgataatggaaaagaacgtcactgttacacatgcccaagacatactggaaaaaacacacaacgggaagctctgtgatctaaatggggtgaagcctctgattttaaaggattgtagtgtagctggatggctcctcggaaacccaatgtgcgacgaattcatcagagtgccggaatggtcctacatagtggagagggctaatccagctaatgacctctgttacccagggagcctcaatgactatgaagaactgaaacacctgttgagcagaataaatcattttgagaagattctgatcatccccaagagttcctggccaaatcatgaaacagcactaggggtgagcgcagcttgtccataccagggagcgccctcctttttcagaaatgtggtgtggcttatcaaaaagaacgatgcatacccaacaataaagataagctacaataataccaatcgggaagatctcttgatactgtgggggattcatcattccaacaatgcagaagagcagacaaatctctataaaaacccaaccacctacatttcagttggaacatcaactttaaaccagaggttggtaccaaaaatagctactagatcccaagtaaacgggcaacgtggaagaatggacttcttctggacaattttaaaaccggatgatgcaatccatttcgagagtaatggaaatttcattgctccagaatatgcatacaaaattgtcaagaaaggggactcaacaattatgaaaagtggagtggaatatggccactgcaacaccaaatgtcaaaccccagtaggagcgataaattctagtatgccattccacaacatacatcctctcaccattggggaatgccccaaatacgtgaagtcaaacaagttggtccttgcgactgggctcagaaatagtcctctaagagaaGGGaagagaagaaaaagaggcctgtttggggcgatagcagggtttatagagggaggatggcagggaatggttgatggttggtatgggtaccaccatagcaatgagcaggggagtgggtacgctgcagacaaagaatccacccaaaaggcaatagatggagttaccaataaggtcaactcaatcattgacaaaatgaacactcaatttgaggcagttggaagggagtttaataacttagaaaggaggatagagaatttgaacaagaaaatggaagacggattcctagatgtctggacctataatgctgaacttctagttctcatggaaaacgagaggactctagatttccatgattcaaatgtcaagaacatttacgacaaagtcagactacagcttagggataatgcaaaggagctgggtaacggctgtttcgaattctatcacaaatgcgataatgaatgtatggaaagtgtgagaaatgggacgtatgactaccctcagtattcagaagaagcaagattaaaaagagaagaaataagcggagtgaaattagaatcaataggaacttaccagatactgtcaatttattcaacagcggcgagttccctagcactggcaatcatgatggctggtctatctttatggatgtgctccaatgggtcgttacagtgcagaatttgcatt

>H5N8_A_pheasant_Finland_499_21VIR7689_1_2021

atggagaacatagtacttcttcttgcaatagttagccttgttaaaagtgatcagatttgcattggttaccatgcaaacaattcgacagagcaagttgacacgataatggaaaagaacgtcactgttacacatgcccaagacatactggaaaaaacacacaacgggaagctctgtgatctaaatggggtgaagcctctgattttaaaggattgtagtgtagctggatggctcctcggaaacccaatgtgcgacgaattcatcagagtgccggaatggtcctacatagtggagagggctaatccagctaatgacctctgttacccagggagcctcaatgactatgaagaactgaaacacctgttgagcagaataaatcattttgagaagattctgatcatccccaagagttcctggccaaatcatgaaacatcactaggggtgagcgcagcttgtccataccagggagcgccctcctttttcagaaatgtggtgtggcttatcaaaaagaacgatgcatacccaacaataaagataagctacaataataccaatcgggaagatctcttgatactgtgggggattcatcattccaacaatgcagaagagcagacaaatctctataaaaacccaaccacctacatttcagttggaacatcaactttaaaccagaggttggtaccaaaaatagctactagatcccaagtgaacgggcaacgtggaagaatggacttcttctggacaattttaaaaccggatgatgcaatccatttcgagagtaatggaaatttcattgctccagaatatgcatacaaaattgtcaagaaaggggactcaacaattatgaaaagtggagtggaatatggccactgcaacaccaaatgtcaaactccagtaggagcgataaattctagtatgccattccacaacatacatcctctcaccattggggaatgccccaaatacgtgaagtcaaacaagttggtccttgcgactgggctcagaaatagtcctctaagagaaGGGaagagaagaaaaagaggcctgtttggggcgatagcagggtttatagagggaggatggcagggaatggttgatggttggtatgggtaccaccatagcaatgagcaggggagtgggtacgctgcagacaaagaatccacccaaaaggcaatagatggagttaccaataaggtcaactcaataattgacaaaatgaacactcaatttgaggcagttggaagggagtttaataacttagaaaggaggatagagaatttgaacaagaaaatggaagacggattcctagatgtctggacctataatgctgaacttctagttctcatggaaaacgagaggactctagatttccatgattcaaacgtcaagaacctttacgacaaagtcagactacagctcagggataatgcaaaggagctgggtaacggctgttttgaattctatcacaaatgcgataatgaatgtatggaaagtgtgagaaatgggacgtatgactaccctcagtattcagaagaagcaagattaaaaagagaagaaataagcggagtgaaattagaatcaataggaacttaccagatactgtcaatttattcaacagcggcgagttccctagcactggcaatcatgatggctggtctatctttatggatgtgctccaatgggtcgttacagtgcagaatttgcatt

>H5N8_A_wigeon_Latvia_23903_2021

atggagaacatagtacttcttcttgcaatagttagccttgttaaaagtgatcagatttgcattggttaccatgcaaacaattcgacagagcaagttgacacgataatggaaaagaacgtcactgtcacacatgcccaagacatactggaaaaaacacacaacgggaagctctgtgatctaaatggggtgaagcctctgattttaaaggattgtagtgtagctggatggctcctcggaaacccaatgtgcgacgaattcatcagagtgccggaatggtcctacatagtggagagggctaatccagctaatgacctctgttacccagggagcctcaatgactatgaagaactgaaacacctgttgagcagaataaatcattttgagaagattctgatcatccccaagagttcctggccaaatcatgaaacatcactaggggtgagcgcagcttgtccataccagggagcgccctcctttttcagaaatgtggtgtggcttatcaaaaagaacgatgcatacccaacaataaagataagctacaataataccaatcgggaagatctcttgatactgtgggggattcatcattccaacaatgcagaagagcagacaaatctctataaaaacccaaccacctacatttcagttggaacatcaactttaaaccagaggttggtaccaaaaatagctactagatcccaagtgaacgggcaacgtggaagaatggacttcttctggacaattttaaaaccggatgatgcaatccatttcgagagtaatggaaatttcattgctccagaatatgcatacaaaattgtcaagaaaggggactcaacaattatgaaaagtggagtggaatatggccactgcaacaccaaatgtcaaactccagtaggagcgataaattctagtatgccattccacaacatacatcctctcaccattggggaatgccccaaatacgtgaagtcaaacaagttggtccttgcgactgggctcagaaatagtcctctaagagaaGGGaagagaagaaaacgaggcctgtttggggcgatagcagggtttatagagggaggatggcagggaatggttgatggttggtatgggtaccaccatagcaatgagcaggggagtgggtacgctgcagacaaagaatccacccaaaaggcaatagatggagttaccaataaggtcaactcaataattgacaaaatgaacactcaatttgaggcagttggaagggagtttaataacttagaaaggaggatagagaatttgaacaagaaaatggaagacggattcctagatgtctggacctataatgctgaacttctagttctcatggaaaacgagaggactctagatttccatgattcaaacgtcaagaacctttacgacaaagtcagactacagctcagggataatgcaaaggagctgggtaacggctgtttcgaattctatcacaaatgcgataatgaatgtatggaaagtgtgagaaatgggacgtatgactaccctcagtattcagaagaagcaagattaaaaagagaagaaataagcggagtgaaattagaatcaataggaacttaccagatactgtcaatttattcaacagcggcgagttccctagcactggcaatcatgatggctggtctatctttatggatgtgctccaatgggtcgttacagtgcagaatttgcatt

>H5N8_A_swan_Lithuania_1298PG1_21VIR2606_3_2021

atggagaacatagtacttcttcttgcaatagttagccttgttaaaagtgatcagatttgcattggttaccatgcaaacaattcgacagaacaagttgacacgataatggaaaagaacgtcactgttacacatgcccaagacatactggaaaaaacacacaacgggaagctctgtgatctaaatggggtgaagcctctgattttaaaggattgtagtgtagctggatggctcctcggaaacccaatgtgcgacgaattcatcagagtgccggaatggtcctacatagtggagagggctaatccagctaatgacctctgttacccagggagcctcaatgactatgaagaactgaaacacctgttgagcagaataaatcattttgagaagattctgatcatccccaagagttcctggccaaatcatgaaacatcactaggggtgagcgcagcttgtccataccagggagcgccctcctttttcagaaatgtggtgtggcttatcaaaaagaacgatgcgtacccaacaataaagataagctacaataataccaatcgggaagatctcttgatactgtgggggattcatcattccaacaatgcagaagagcagacaaatctctataaaaacccaaccacctacatttcagttggaacatcaactttaaaccagaggttggtaccaaaaatagctactagatcccaagtgaacgggcaacgtggaagaatggacttcttctggacaattttaaaaccggatgatgcaatccatttcgagagtaatggaaatttcattgctccagaatatgcatacaaaattgtcaagaaaggggactcaacaattatgaaaagtggagtggaatatggccactgcaacaccaaatgtcaaactccagtaggagcgataaattctagtatgccattccacaacatacatcctctcaccattggggaatgccccaaatacgtgaagtcaaacaagttggtccttgcgactgggctcagaaatagtcctctaagagaaGGGaagagaagaaaaagaggcctgtttggggcgatagcagggtttatagagggaggatggcagggaatggttgatggttggtatgggtaccaccatagcaatgagcaggggagtgggtacgctgcagacaaagaatccacccaaaaggcaatagatggagttaccaataaggtcaactcaataattgacaaaatgaacactcaatttgaggcagttggaagggagtttaataacttagaaaggaggatagagaatttgaacaagaaaatggaagacggattcctagatgtctggacctataatgctgaacttctagttctcatggaaaacgagaggactctagatttccatgattcaaacgtcaagaacctttacgacaaagtcagactacagctcagggataatgcaaaggagctgggtaacggctgtttcgaattctatcacaaatgcgataatgaatgtatggaaagtgtgagaaatgggacgtatgactaccctcagtattcagaagaagcaagattaaaaagagaaaaaataagcggagtgaaattagaatcaataggaacttaccagatactgtcaatttattcaacagcggcgagttccctagcactggcaatcatgatggctggtctatctttatggatgtgctccaatgggtcgttacagtgcagaatttgcatt

>H5N8_A_chicken_Luxembourg_21168413_2021

atggagaacatagtacttcttcttgcaatagttagccttgttaaaagtgatcagatttgcattggttaccatgcaaacaattcgacagagcaagttgacacgataatggaaaagaacgtcactgttacacatgcccaagacatactggaaaaaacacacaacgggaagctctgtgatctaaatggggtgaagcctctgattttaaaagattgtagtgtagctggatggctcctcggaaacccaatgtgcgacgaattcatcagagtgccggaatggtcctacatagtggagagggctaatccagcyaatgacctctgttacccagggagcctcaatgactatgaagaactgaaacacctgttgagcagaataaatcattttgagaagattctgatcatccccaagagttcctggccaaatcatgaaacatcactaggggtgagcgcagcttgtccataccagggagcgccctcctttttcagaaatgtggtgtggcttatcaaaaagaacgatgcatacccaacaataaagataagctacaataataccaatcgggaagatctcttgatactgtgggggattcatcattccaacaatgcagaagagcagacaaatctctataaaaacccaaccacctacatttcagttggaacatcaactttaaaccagaggttggtaccaaaaatagctactagatcccaagtgaacgggcaacgtggaagaatggacttcttctggacaattttaaaaccggatgatgcaatccatttcgagagtaatggaaatttcattgctccagaatatgcctacaaaattgtcaagaaaggggactcaacaattatgaaaagtggagtggaatatggccactgcaacaccaaatgtcaaactccagtaggagcgataaattctagtatgccattccacaacatacatcctctcaccattggggaatgccccaaatacgtgaagtcaaacaagttggtccttgcgactgggctcagaaatagtcctctaagaggaGGGaagagaagaaaaagaggcctgtttggggcgatagcagggtttatagagggaggatggcagggaatggttgatggttggtatgggtaccaccatagcaatgagcaggggagtgggtacgctgcagacaaagaatccacccaaaaggcaatagatggagttaccaataaggtcaactcaataattgacaaaatgaacactcaattcgaggcagttggaagggagtttaataacttagaaaggaggatagagaatttgaacaagaaaatggaagacggattcctggatgtctggacctataatgctgaacttctagttctcatggaaaacgagaggactctagatttccatgattcaaacgtcaagaacctttacgacaaagtcagactacagctcagggataatgcaaaggagctgggtaacggctgtttcgaattctatcacaaatgcgataatgaatgtatggaaagtgtgagaaatgggacgtatgactaccctcagtattcagaagaagcaagattaaaaagagaagaaataagcggagtgaaattagaatcaataggaacttaccagatactgtcaatttattcaacagcggcgagttccctagcactggcaatcatgatggctggtctatctttatggatgtgctccaatgggtcgttacagtgcagaatttgcatt

>H5N8_A_mute_swan_Estonia_TA2106419_1_21VIR7512_3_2021

atggagaacatagtacttcttcttgcaatagttagccttgttaaaagtgatcagatttgcattggttaccatgcaaacaattcgacagagcaagttgacacgataatggaaaagaacgtcactgttacacatgcccaagacatactggagaaaacacacaacgggaagctctgtgatctaaatggggtgaagcctctgattttaaaggattgtagtgtagctggatggctcctcggaaacccaatgtgcgacgaattcatcagagtgccggaatggtcctacatagtggagagggctaatccagctaatgacctctgttacccagggagcctcaatgactatgaagaactgaaacacctgttgagcagaataaatcattttgagaagattctgatcatccccaagagttcctggccaaatcatgaaacatcactaggggtgagcgcagcttgtccataccagggagcgccctcctttttcagaaatgtggtgtggcttatcaaaaagaacgatgcatacccaacaataaagataagctacaataataccaatcgggaagatctcttgatactgtgggggattcatcattccaacaatgcagaagagcagacaaatctctataaaaacccaaccacctacatttcagttggaacatcaactttaaaccagaggttggtaccaaaaatagctactagatcccaagtgaacgggcaacgtggaagaatggacttcttctggacaattttaaaaccggatgatgcaatccatttcgagagtaatggaaatttcattgctccagaatatgcatacaaaattgtcaagaaaggggactcaacaattatgaaaagtggagtggaatatggccactgcaacaccaaatgtcaaactccagtaggagcgataaattctagtatgccattccacaacatacatcctctcaccattggggaatgccccaaatacgtgaagtcaaacaagttggtccttgcgactgggctcagaaatagtcctctaagagaaGGGaagagaagaaaaagaggcctgtttggggcgatagcagggtttatagagggaggatggcagggaatggttgatggttggtatgggtaccaccatagcaatgagcaggggagtgggtacgctgcagacaaagaatccacccaaaaggcaatagatggagttaccaataaggtcaactcaataattgacaaaatgaacactcaatttgaggcagttggaagggagtttaataacttagaaaggaggatagagaatttgaacaagaaaatggaagacggattcctagatgtctggacctataatgctgaacttctagttctcatggaaaacgagaggactctagatttccatgattcaaacgtcaagaacctttacgacaaagtcagactacagctcagggataatgcaaaggagctgggtaacggctgtttcgaattctatcacaaatgcgataatgaatgtatggaaagtgtgagaaatgggacgtatgactaccctcagtattcagaagaagcaagattaaaaagagaagaaataagcggagtgaaattagaatcaataggaacttaccagatactgtcaatttattcaacagcggcgagttccctagcactggcaatcatgatggctggtctatctttatggatgtgctccaatgggtcgttacagtgcagaatttgcatt

>H5N5_A_tufted_duck_Poland_MB061_2021

atgaagaacatagtacttcttcttgcaatagttagccttgttaaaagtgatcagatttgcattggttaccatgcaaacaattcgacagagcaagttgacacgataatggaaaagaacgtcactgttacacatgcccaagacatactggaaaaaacacacaacgggaagctctgtgatctaaatggggtgaagcctctgattttaaaggattgtagtgtagctggatggctcctcggaaacccaatgtgcgacgaattcatcagagtgccggaatggtcctacatagttgagagggctaatccagctaatgacctctgttacccagggagcctcaatgactatgaagaactgaaacacctgttgagcagaataaatcattttgagaagattctgatcatccccaagagttcctggccaaatcatgaaacatcactaggggtgagcgcagcttgtccataccagggagcgccatcctttttcagaaatgtggtgtggcttatcaaaaagaacgatgcatacccaacaataaagataagctacaataataccaatcgggaagatctcttgatactgtgggggattcatcattccaacaatgcagaagagcagacaaatctctataaaaacccaaccacctacatttcagttggaacatcaactttaaaccagaggttggtaccaaaaatagctactagatcccaagtgaacgggcaacgtggaagaatggacttcttctggacaattttaaaaccggatgatgcaatccatttcgagagtaatggaaatttcattgctccagaatatgcatacaaaattgtcaagaaaggggactcaacaattatgaaaagtggagtggaatatggccactgcaacaccaaatgtcaaactccagtaggagcgataaactctagtatgccattccacaacatacatcctctcaccattggggaatgccccaaatacgtgaagtcaaacaagttggtccttgcgactgggctcagaaatagtcctctaagagaaGGGaagagaagaaaaagaggtctgtttggggcgatagcagggtttatagagggaggatggcagggaatggttgatggttggtatgggtaccaccatagcaatgagcaggggagtgggtacgctgcagacaaagaatccacccaaaaggcaatagatggagttaccaataaggtcaactcaataattgacaaaatgaacactcaatttgaggcagttggaagggagtttaataaattagaaaggaggatagagaacttgaacaagaaaatggaagacgggttcctagatgtctggacctataatgctgaacttctagttctcatggaaaacgagaggactctagatttccatgattcaaacgtcaagaacctttacgacaaagtcagactacagctcagggataatgcaaaggagctgggtaacggctgtttcgaattctatcacaaatgcgataatgaatgtatggaaagtgtgagaaatgggacgtatgactaccctcagtattcagaagaagcaagattaaaaagagaagaaataagcggagtgaaattagaatcaataggaacttaccagatactgtcaatttattcaacagcggcgagttccctagcactggcaatcatgatggctggtctatctttatggatgtgctccaatgggtcgttacagtgcagaatttgcatt

>H5N1_A_quail_Korea_H526_2021

atggagaacatagtacttcttcttgcaatagttagccttgttaaaagtgatcagatttgcattggttaccatgcaaacaattcgacagagcaagttgacacgataatggaaaagaacgtcactgttacacatgcccaagacatactggaaaaaacacacaacgggaagctctgtgatctaaatggggtgaagcctctgattttaaaggattgtagtgtagctggatggctcctcggaaacccaatgtgcgacgaattcatcagagtgccggaatggtcctacatagtggagagggctaatccgactaatgacctctgttacccagggagcctcaatgactatgaagaattgaaacacctgttgagcagaataaatcattttgagaagattctgatcatccccaagaattcctggccaaatcatgaaacatcactaggggtgagtgcagcttgtccataccagggagcgccctcctttttcagaaatgtggtgtggcttatcaaaaagaacgatgcatacccaacaataaagataagctacaataataccaatcgggaagatctcttgatactgtgggggattcatcattccaacaatgcagaagagcagacagatctctacaaaaacccaaccacctacatttcagttggaacatcaactttaaaccagaggttggtaccaaaaatagctactagatcccaagtaaacgggcaacgtggaagaatggacttcttctggacaattttaaaaccggatgatgcaatccatttcgagagtaatggaaatttcattgctccagaatatgcatacaaaattgtcaagaaaggggactcaacaattatgaaaagtggagtggaatatggccactgcaacaccaaatgtcaaaccccagtaggagcgataaattctagtatgccattccacaacatacatcctctcaccattggggaatgccccaaatacgtgaagtcaaacaagttggtccttgcgactgggctcagaaatagtcctctaagagaaGGGaggagaagaaaaagaggcctatttggggcgatagcagggtttatagagggaggatggcagggaatggttgatggttggtatgggtaccatcatagcaatgagcaggggagtgggtacgctgcagacaaagaatccacccaaaaggcaatagatggagttaccaataaggtcaactcaataattgacaaaatgaacactcaatttgaggcagttggaagggagtttaataacttagaaaggaggatagagaatttgaacaagaaaatggaagacggattcctagatgtatggacctataatgctgaacttctagttctcatggaaaatgagaggactctagatttccatgattcaaatgtcaagaacctttacgacaaagtcagactacagcttagggataatgcaaaggagctgggtaacggctgtttcgaattctatcacaaatgcgataacgaatgtatggaaagtgtgagaaatgggacgtatgactaccctcagtattcagaagaagcaagattaaaaagagaagaaataagcggagtgaaattagaatcaataggaacttaccagatactgtcaatttattcaacagcggcgagttccctagcactggcaatcatgatggctggtctttctttatggatgtgctccaatgggtcgttacagtgcagaatttgcatt

>H5N8_A_common_teal_Italy_20VIR7608_73_2020

atggagaacatagtacttcttcttgcaatagttagccttgttaaaagtgatcagatttgcattggttaccatgcaaacaattcgacagagcaagttgacacgataatggaaaagaacgtcactgttacacatgcccaagacatactggaaaaaacacacaacgggaagctctgtgatctaaatggggtgaagcctctgattttaaaggattgtagtgtagctggatggctcctcggaaacccaatgtgcgacgaattcatcagagtgccggaatggtcctacatagtggagagggctaatccagctaatgacctctgttacccagggagcctcaatgactatgaagaactgaaacacctgttgagcagaataaatcattttgagaagattctgatcatccccaaaagttcctggccaaatcatgaaacatcactaggggtgagcgcagcttgtccataccagggagcgccctcctttttcagaaatgtggtgtggcttatcaaaaagaacgatgcatacccaacaataaagataagctacaataataccaatcgggaagatctcttgatactgtgggggattcatcattccaacaatgcagaagagcagacaaatctctataaaaacccaaccacctacatttcagttggaacatcaactttaaaccagaggttggtgccaaaaatagctactagatcccaagtaaacgggcaacgtgggagaatggacttcttctggacaattttaaaaccggatgatgcaatccatttcgagagtaatggaaatttcattgctccagaatatgcatacaaaattgtcaagaaaggggactcaacaattatgaaaagtggagtggaatatggccactgcaacaccaaatgtcaaaccccagtaggagcgataaactctagtatgccattccacaacatacatcctctcaccattggggaatgccccaaatacgtgaagtcaaacaagttggtccttgcgaccgggctcagaaatagtcctctaagagaaGGGaagagaagaaaaagaggcctgtttggggcgatagcagggtttatagagggaggatggcagggaatggttgatggttggtatgggtaccaccatagcaatgagcaggggagtgggtacgctgcagacaaagaatccacccaaaaggcaatagatggtgttaccaataaggtcaactcaataattgacaaaatgaacactcaatttgaggcagttggaagggagtttaataacttagaaaggaggatagagaatttgaacaagaaaatggaagacggattcctagatgtctggacctataatgctgaacttctagttctcatggaaaacgagaggactctagatttccatgattcaaatgtcaagaacctttacgacaaggtcagactacagcttagggataatgcaaaggagctgggtaacggctgtttcgaattctatcacaaatgcgataatgaatgtatggaaagtgtgagaaatgggacgtatgactaccctcagtattcagaagaagcaagattaaaaagagaagaaataagcggagtgaaattagaatcaataggaacttaccagatactgtcaatttattcaacagcggcgagttccctagcactggcaatcatgatggctggtctatctttatggatgtgctccaatgggtcgttacagtgcagaatttgcatt

>H5N8_A_chicken_Italy_21VIR1293_9_2021

atggagaacatagtacttcttcttgcaatagttagccttgttaaaagtgatcagatttgcattggttaccatgcaaacaattcgacagagcaagttgacacgataatggaaaagaacgtcactgttacacatgcccaagacatactggaaaaaacacacaacgggaagctctgtgatctaaatggggtgaagcctctgattttaaaggattgtagtgtggctggatggctcctcggaaacccaatgtgcgacgaattcatcagagtgccggaatggtcctacatagtggagagggctaatccagctaatgacctctgttacccagggagcctcaatgactatgaagaactgaaacacctgttgagcagaataaatcattttgagaagattctgatcattcccaaaagttcctggccaaatcatgaaacatcactaggggtgagcgcagcttgtccataccagggagcgccctcctttttcagaaatgtggtgtggcttatcaaaaagaacgatgcatacccaacaataaagataagctacaataataccaatcgggaagatctcttgatactgtgggggattcatcattccaacaatgcagaagagcagacaaatctctataaaaacccaaccacctacatttcagttggaacatcaactttaaaccagaggttggtgccaaaaatagctactagatcccaagtaaacgggcaacgtgggagaatggacttcttctggacaattttaaaaccggatgatgcaattcatttcgagagtaatggaaatttcattgctccagaatatgcatacaaarttgtcaagaaaggggactcaacaattatgaaaagtggagtggaatatggccactgcaacaccaaatgtcaaaccccagtaggagcgataaattctagtatgccattccacaacatacatcctctcaccattggggaatgccccaaatacgtgaagtcaaacaagttggtccttgcgactgggctcagaaatagtcctctaagagaaGGGaagagaagaaaaagaggcctgtttggggcgatagcagggtttatagagggaggatggcaaggaatggttgatggttggtatgggtaccaccatagcaatgagcaggggagtgggtacgctgcagacaaagaatccacccaaaaggcaatagatggtgttaccaataaggtcaactcaataattgacaaaatgaacactcaatttgaggcagttggaagggagtttaataacttagaaaggaggatagagaatttgaacaagaaaatggaagacggattcctagatgtctggacctataatgctgaacttctagttctcatggaaaacgagaggactctagatttccatgattcaaatgtcaagaacctttacgacaaggtcagactacagcttagggataatgcaaaggagctgggtaacggctgtttcgaattctatcacaaatgcaataatgaatgtatggaaagtgtgagaaatgggacgtatgactaccctcagtattcagaagaagcaagattaaaaagagaagaaataagcggagtgaaattagaatcaataggaacttaccagatactgtcaatttattcaacagcggcgagttccctagcactggcaatcatgatggctggtctatctttatggatgtgctccaatgggtcgttacagtgcagaatttgcatt

>H5N3_A_common_buzzard_Netherlands_21021023_002_2021

atggagaacatagtacttcttcttgcaatagttagccttgttaaaagtgatcagatttgcattggttaccatgcaaacaattcgacagagcaagttgacacgataatggaaaagaacgtcactgttacacatgcccaagacatactggaaaaaacacacaacgggaagctctgtgatctaaatggggtgaagcctctgattttaaaggattgtagtgtagctggatggctcctcggaaacccaatgtgcgacgaattcataagagtgccggaatggtcctacatagtggagagggctaatccagctaatgacctctgttacccagggagcctcaatgactatgaagaactgaaacacctgttgagcagaataaatcattttgagaagattctgatcatccccaagagttcctggcaaaatcatgaaacatcactaggggtgagcgcagcttgtccataccagggagcgccctcctttttcagaaatgtggtgtggcttatcaaaaagaacgatgcatacccaacaataaagataagctacaataataccaatcgggaagatctcttgatactgtgggggatccatcattccaacaatgcagaagaacagacaaatctctataaaaacccaaccacctacatttcagttggaacatcaactttaaaccagaggttggtaccaaaaatagctactagatcccaagtaaacgggcaacgtggaagaatggacttcttctggacaattttaaaaccggatgatgcaatccatttcgagagtaatggaaatttcattgctccagaatatgcatacaaaattgtcaagaaaggggactcaacaattatgaaaagtggagtggaatatggccactgcaacaccaaatgtcaaaccccagtaggtgcgataaattctagtatgccattccacaacatacatcctctcaccattggggaatgccccaaatacgtgaagtcaaacaagttggtccttgcgactgggctcagaaatagtcctctaagagaaGGGaagagaagaaaaagaggcctgtttggggcgatagcagggtttatagagggaggatggcagggaatggttgatggttggtatgggtaccaccatagcaatgagcaggggagtgggtacgctgcagacaaagaatccacccaaaaggcaatagatggagttaccaataaggtcaactcaatcattgacaaaatgaacactcaatttgaggcagttggaagggagtttaataacttagaaaggaggatagagaatttgaacaagaaaatggaagacggattcctagatgtctggacctataatgctgaacttctagttctcatggaaaacgagaggactctagatttccatgattcaaatgtcaagaacctttacgacaaagtcagactacagcttagggataatgcaaaggagctgggtaacggctgtttcgaattctatcacaaatgcgataatgaatgtatggaaagtgtgagaaatgggacgtatgactaccctcagtattcagaagaagcaagattaaaaagagaagaaataagcggagtgaaattagaatcaataggaacttaccagatactgtcaatttattcaacagcggcgagttccctagcactggcaatcatgatggctggtctatctttatggatgtgctccaatgggtcgttacagtgcagaatttgcatt

>H5N3_A_curlew_France_21P003648_2021

atggagaacatagtacttcttcttgcaatagttagccttgttaaaagtgatcagatttgcattggttaccatgcaaacaattcgacagagcaagttgacacgataatggaaaagaacgtcactgttacacatgcccaagacatactggaaaaaacacacaacgggaagctctgtgatctaaatggggtgaagcctctgattttaaaggattgtagtgtagctggatggctcctcggaaacccaatgtgcgacgaattcatcagagtgccggaatggtcctacatagtggagagggctaatccagctaatgacctctgttacccagggagcctcaatgactatgaagaactgaaacacctgttgagcagaataaatcattttgagaagattctgatcatccccaagagttcctggcaaaatcatgaaacatcactaggggtgagcgcagcttgtccataccagggagcgccctcctttttcagaaatgtggtgtggcttatcaaaaagaacgatgcatacccaacaataaagataagctacaataataccaatcgggaagatctcttgatactgtgggggatccatcattccaacaatgcagaagagcagacaaatctctataaaaacccaaccacctacatttcagttggaacatcaactttaaaccagaggttggtaccaaaaatagctactagatcccaagtaaacgggcaacgtggaagaatggacttcttctggacaattttaaaaccggatgatgcaatccatttcgagagtaatggaaatttcattgctccagaatatgcatacaaaattgtcaagaaaggggactcaacaattatgaaaagtggagtggaatatggccactgcaacaccaaatgtcaaaccccagtaggtgcgataaattctagtatgccattccacaacatacatcctctcaccattggggaatgccccaaatacgtgaagtcaaacaagttggtccttgcgactgggctcagaaatagtcctctaagagaaGGGaagagaagaaaaagaggcctgtttggggcgatagcagggtttatagagggaggatggcagggaatggttgatggttggtatgggtaccaccatagcaatgagcaggggagtggatacgctgcagacaaagaatccacccaaaaggcaatagatggagttaccaataaggtcaactcaatcattgacaaaatgaacactcaatttgaggcagttggaagggagtttaataacttagaaaggaggatagagaatttgaacaagaaaatggaagacggattcctagatgtctggacctataatgctgaacttctagttctcatggaaaacgagaggactctagatttccatgattcaaatgtcaagaacctttacgacaaagtcagactacagcttagggataatgcaaaggagctgggtaacggctgtttcgaattctatcacaaatgcgataatgaatgtatggaaagtgtgagaaatgggacgtatgactaccctcagtattcagaagaagcaagattaaaaagagaagaaataagcggagtgaaattagaatcaataggaacttaccagatactgtcaatttattcaacagcggcgagttccctagcactggcaatcatgatggctggtctatctttatggatgtgctccaatgggtcgttacagtgcagaatttgcatt

>H5N3_A_common_kestrel_Denmark_16023_01_2021

atggagaacatagtacttcttcttgcaatagttagccttgttaaaagtgatcagatttgcattggttaccatgcaaacaattcgacagagcaagttgacacgataatggaaaagaacgtcactgttacacatgcccaagacatactggaaaaaacacacaacgggaagctctgtgatctaaatggggtgaagcctctgattttaaaggattgtagtgtagctggatggctcctcggaaacccaatgtgcgacgaattcatcagagtgccggaatggtcctacatagtggagagggctaatccagctaatgacctctgttacccagggagcctcaatgactatgaagaactgaaacacctgttgagcagaataaatcattttgagaagattctgatcatccccaagagctcctggcaaaatcatgaaacatcactaggggtgagcgcagcttgtccataccagggagcgccctcctttttcagaaatgtggtgtggcttatcaaaaagaacgatgcatacccaacaataaagataagctacaataataccaatcgggaagatctcttgatactgtgggggatccatcattccaacaatgcagaagagcagacaaatctctataaaaacccaaccacctacatttcagttggaacatcaactttaaaccagaggttggtaccaaaaatagctactagatcccgagtaaacgggcaacgtggaagaatggacttcttctggacaattttaaaaccggatgatgcaatccatttcgagagtaatggaaatttcattgctccagaatatgcatacaaaattgtcaagaaaggggactcaacaattatgaaaagtggagtggaatatggccactgcaacaccaaatgtcaaaccccagtaggtgcgataaattctagtatgccattccacaacatacatcctctcaccattggggaatgccccaaatacgtgaagtcaaacaagttggtccttgcgactgggctcagaaatagtcctctaagagaaGGGaagagaagaaaaagaggcctgtttggggcgatagcagggtttatagagggaggatggcagggaatggttgatggttggtatgggtaccaccatagcaatgagcaggggagtgggtacgctgcagacaaagaatccacccaaaaggcaatagatggagttaccaataaggtcaactcaatcattgacaaaatgaacactcaatttgaggcagttggaagggagtttaataacttagaaaggaggatagagaatttgaacaagaaaatggaagacggattcctagatgtctggacctataatgctgaacttctagttctcatggaaaacgagaggactctagatttccatgattcaaatgtcaagaacctttacgacaaagtcagactacagcttagggataatgcaaaggagctgggtaacggctgtttcgaattctatcacaaatgcgataatgaatgtatggaaagtgtgagaaatgggacgtatgactaccctcagtattcagaagaagcaagattaaaaagagaagaaataagcggagtgaaattagaatcaataggaacttaccagatactgtcaatttattcaacagcggcgagttccctagcactggcaatcatgatggctggtctatctttatggatgtgctccaatgggtcgttacagtgcagaatttgcatt

>H5N5_A_mute_swan_Wales_048068_2020

atggagaacatagtacttcttcttgcaatagttagccttgttaaaagtgatcagatttgcattggttaccatgcaaacaattcgacagagcaagttgacacgataatggaaaagaacgtcactgttacacatgcccaagacatactggaaaaaacacacaacgggaagctctgtgatctaaatggggtgaagcctctgattttaaaggattgtagtgtagctggatggctcctcggaaacccaatgtgcgacgaattcatcagagtgccggaatggtcctacatagtggagagggctaatccagctaatgacctctgttacccagggagcctcaatgactatgaagaactgaaacacctgttgagcagaataaatcattttgagaagattctgatcatccccaagagttcctggcaaaatcatgaaacatcactaggggtgagcgcagcttgtccataccagggagcgccctcctttttcagaaatgtggtgtggcttatcaaaaagaacgatgcatacccaacaataaagataagctacaataataccaatcgggaagatctcttgatactgtgggggatccatcattccaacaatgcagaagagcagacaaatctctataaaaacccaaccacctacatttcagttggaacatcaactttaaaccagaggttggtaccaaaaatagctactagatcccaagtaaacgggcaacgtggaagaatggacttcttctggacaattttaaaaccggatgatgcaatccatttcgagagtaatggaaatttcattgctccagaatatgcatacaaaattgtcaagaaaggggactcaacaattatgaaaagtggagtggaatatggccactgcaacaccaaatgtcaaaccccagtaggtgcgataaattctagtatgccattccacaacatacatcctctcaccattggggaatgccccaaatacgtgaagtcaaacaagttggtccttgcgactgggctcagaaatagtcctctaagagaaGGGaagagaagaaaaagaggcctgtttggggcgatagcagggtttatagagggaggatggcagggaatggttgatggttggtatgggtaccaccatagcaatgagcaggggagtgggtacgctgcagacaaagaatccacccaaaaggcaatagatggagttaccaataaggtcaactcaatcattgacaaaatgaacactcaatttgaggcagttggaagggagtttaataacttagaaaggaggatagagaatttgaacaagaaaatggaagacggattcctagatgtctggacctataatgctgaacttctagttctcatggaaaacgagaggactctagatttccatgattcaaatgtcaagaacctttacgacaaagtcagactacagcttagggataatgcaaaggagctgggtaacggctgtttcgaattctatcacaaatgcgataatgaatgtatggaaagtgtgagaaatgggacgtatgactaccctcagtattcagaagaagcaagattaaaaagagaagaaataagcggagtgaaattagaatcaataggaacttaccagatactgtcaatttattcaacagcggcaagttccctagcactggcaatcatgatggctggtctatctttatggatgtgctccaatgggtcgttacagtgcagaatttgcatt

>H5N5_A_buzzard_Germany_MV_AI02166_2020

atggagaacatagtacttcttcttgcaatagttagccttgttaaaagtgatcagatttgcattggttaccatgcaaacaattcgacagagcaagttgacacgataatggaaaagaacgtcactgttacacatgcccaagacatactggaaaaaacacacaacgggaagctctgtgatctaaatggggtgaagcctctgattttaaaggattgtagtgtagctggatggctcctcggaaacccaatgtgcgacgaattcatcagagtgccggaatggtcctacatagtggagagggctaatccagctaatgacctctgttacccagggagcctcaatgactatgaagaactgaaacacctgttgagcagaataaatcattttgagaagattctgatcatccccaagagttcctggcaaaatcatgaaacatcactaggggtgagcgcagcttgtccataccagggagcgccctcctttttcagaaatgtggtgtggcttatcaaaaagaacgatgcatacccaacaataaagataagctacaataataccaatcgggaagatctcttgatactgtgggggatccatcattccaacaatgcagaagagcagacaaatctctataaaaacccaaccacctacatttcagttggaacatcaactttaaaccagaggttggtaccaaaaatagctactagatcccaagtaaacgggcaacgtggaagaatggacttcttctggacaattttaaaaccggatgatgcaatccatttcgagagtaatggaaatttcattgctccagaatatgcatacaaaattgtcaagaaaggggactcaacaattatgaaaagtggagtggaatatggccactgcaacaccaaatgtcaaaccccagtaggtgcgataaattctagtatgccattccacaacatacatcctctcaccattggggaatgccccaaatacgtgaagtcaaacaagttggtccttgcgactgggctcagaaatagtcctctaagagaaGGGaagagaagaaaaagaggcctgtttggggcgatagcagggtttatagagggaggatggcagggaatggttgatggttggtatgggtaccaccatagcaatgagcaggggagtgggtacgctgcagacaaagaatccacccaaaaggcaatagatggagttaccaataaggtcaactcaatcattgacaaaatgaacactcaatttgaggcagttggaagggagtttaataacttagaaaggaggatagagaatttgaacaagaaaatggaagacggattcctagatgtctggacctataatgctgaacttctagttctcatggaaaacgagaggactctagatttccatgattcaaatgtcaagaacctttacgacaaagtcagactacagcttagggataatgcaaaggagctgggtaacggctgtttcgaattctatcacaaatgcgataatgaatgtatggaaagtgtgagaaatgggacgtatgactaccctcagtattcagaagaagcaagattaaaaagagaagaaataagcggagtgaaattagaatcaataggaacttaccagatactgtcaatttattcaacagcggcgagttccctagcactggcaatcatgatggctggtctatctttatggatgtgctccaatgggtcgttacagtgcagaatttgcatt

>H5N3_A_red_knot_Germany_SH_AI03421_2020

atggagaacatagtacttcttcttgcaatagttagccttgttaaaagtgatcagatttgcattggttaccatgcaaacaattcgacagagcaagttgacacgataatggaaaagaacgtcactgttacacatgcccaagacatactggaaaaaacacacaacgggaagctctgtgatctaaatggggtgaagcctctgattttaaaggattgtagtgtagctggatggctcctcggaaacccaatgtgcgacgaattcatcagagtgccggaatggtcctacatagtggagagggctaatccagctaatgacctctgttacccaggaagcctcaatgactatgaagaactgaaacacctgttgagcagaataaatcattttgagaagattctgatcatccccaagagttcctggcaaaatcatgaaacatcactaggggtgagcgcagcttgtccataccagggagcaccctcctttttcagaaatgtggtgtggcttatcaaaaagaacgatgcatacccaacaataaagataagctacaataataccaatcgggaagatctcttgatactgtgggggatccatcattccaacaatgcagaagagcagacaaatctctataaaaacccaaccacctacatttcagttggaacatcaactttaaaccagaggttggtaccaaaaatagctactagatcccaagtaaacgggcaacgtggaagaatggacttcttctggacaattttaaaaccggatgatgcaatccatttcgagagtaatggaaatttcattgctccagaatatgcatacaaaattgtcaagaaaggggactcaacaattatgaaaagtggagtggaatatggccactgcaacaccaaatgtcaaaccccagtaggtgcgataaattctagtatgccattccacaacatacatcctctcaccattggggaatgccccaaatacgtgaagtcaaacaagttggtccttgcgactgggctcagaaatagtcctctaagagaaGGGaagagaagaaaaagaggcctgtttggggcgaaagcagggtttatagagggaggatggcagggaatggttgatggttggtatgggtaccaccatagcaatgagcaggggagtgggtacgctgcagacaaagaatccacccaaaaggcaatagatggagttaccaataaggtcaactcaatcattgacaaaatgaacactcaatttgaggcagttggaagggagtttaataacttagaaaggaggatagagaatttgaacaagaaaatggaagacggattcctagatgtctggacctataatgctgaacttctagttctcatggaaaacgagaggactctagatttccatgattcaaatgtcaagaacctttacgacaaagtcagactacagcttagggataatgcaaaggagctgggtaacggctgtttcgaattctatcacaaatgcgataatgaatgtatggaaagtgtgagaaatgggacgtatgactaccctcagtattcagaagaagcaagattaaaaagagaagaaataagcggagtgaaattagaatcaataggaacttaccagatactgtcaatttattcaacagcggcgagttccctagcactggcaatcatgatggctggtctatctttatggatgtgctccaatgggtcgttacagtgcagaatttgcatt

>H5N5_A_Gallus_gallus_Belgium_12168_002_2020

atggagaacatagtacttcttcttgcaatagttagccttgttaaaagtgatcagatttgcattggttaccatgcaaacaattcgacagagcaagttgacacgataatggaaaagaacgtcactgttacacatgcccaagacatactggaaaaaacacacaacgggaagctctgtgatctaaatggggtgaagcctctgattttaaaggattgtagtgtagctggatggctcctcggaaacccaatgtgcgacgaattcatcagagtgccggaatggtcctacatagtggagagggccaatccagctaatgacctctgttacccagggagcctcaatgactatgaagaactgaaacacctgttgagcagaataaatcattttgagaagattctgatcatccccaagagttcctggcaaaaccatgaaacatcactaggggtgagcgcagcttgtccataccagggagcgccctcctttttcagaaatgtggtgtggcttgtcaaaaagaacgatgcatacccaacaataaagataagctacaataataccaatcgggaagatctcttgatactgtgggggatccatcattccaacaatgcagaagagcagacaaatctctataaaaacccaaccacctacatttcagttggaacatcaactttaaaccagaggttggtaccaaaaatagctactagatcccaagtaaacgggcaacgtggaagaatggacttcttctggacaattttaaaaccggatgatgcaatccatttcgagagtaatggaaatttcattgctccagaatatgcatacaaaattgtcaagaaaggggactcaacaattatgaaaagtggagtggaatatggccactgcaacaccaaatgtcaaaccccagtaggtgcgataaattctagtatgccattccacaacatacatcctctcaccattggggaatgccccaaatacgtgaagtcaaacaagttggtccttgcgactgggctcagaaatagtcctctaagagaaGGGaagagaagaaaaagaggcctgtttggggcgatagcagggtttatagagggaggatggcagggaatggttgatggttggtatgggtaccaccatagcaatgagcaggggagtgggtacgctgcagacaaagaatccacccaaaaggcaatagatggagttaccaataaggtcaactcaatcattgacaaaatgaacactcaatttgaggcagttggaagggagtttaataacttagaaaggaggatagagaatttgaacaagaaaatggaagacggattcctagatgtctggacctataatgctgaacttctagttctcatggaaaacgagaggactctagatttccatgattcaaatgtcaagaacctttacgacaaagtcagactacagcttagggataatgcaaaggagctgggtaacggctgtttcgaattctatcacaaatgcgataatgaatgtatggaaagtgtgagaaatgggacgtatgactaccctcagtattcagaagaagcaagattaaaaagagaagaaataagcggagtgaaattagaatcaataggaacttaccagatactgtcaatttattcaacagcggcgagttccctagcactggcaatcatgatggctggtctatctttatggatgtgctccaatgggtcgttacagtgcagaatttgcatt

>H5N4_A_Laridae_Germany_SH_AI01498_2021

atggagaacatagtacttcttcttgcaatagtcagccttgttaaaagtgatcagatttgcattggttaccatgcaaacaattcgacagagcaagttgacacgataatggaaaagaacgtcactgttacacatgcccaagacatactggaaaaaacacacaacgggaagctctgtgatctgaatggggtgaagcctctgattttaaaggattgtagtgtagctggatggctcctcggaaacccaatgtgcgacgaattcatcagagtgccggaatggtcctacatagtggagagggctaatccagttaatgacctctgttacccagggagcctcaatgactatgaagaactgaaacacctgttgagcagaataaatcattttgagaagattctgatcatccccaagagttcctggccaaatcatgaaacatcactaggggtgagcgcagcttgtccataccagggagcgccctcctttttcagaaatgtggtgtggcttatcaaaaagaacgatacatacccaacaataaagataagctacaataataccaatcgggaagatctcttgatactgtgggggattcatcattccaacaatgcagaagagcagacaaatctctataaaaacccaaccacctacatttcagttggaacatcaactttaaaccagaggttggtaccaaaaatagctactagatcccaagtaaacgggcaacgtggaagaatggacttcttctggacaattttaaaaccggatgatgcaatccatttcgagagtaatggaaatttcattgctccagaatatgcatacaaaattgtcaaggaaggggactcaacaattatgaaaagtggagtggaatatggccactgcaacaccaaatgtcagaccccagtaggagcgataaattctagtatgccattccacaacatacatcctctcaccattggggaatgccccaaatacgtgaagtcaaacaaattggtccttgcgactgggctcagaaatagtcctctaagggaaGGGaagagaagaaaaagaggcctgtttggggcgatagcagggtttatagagggaggatggcagggaatggttgatggttggtatgggtaccaccatagcaatgagcaggggagtgggtacgctgcagacaaagaatccacccaaaaggcaatagatggaattaccaataaggtcaactcaatcattgacaaaatgaacactcaatttgaggcagttggaagggagtttaataacttagaaaggaggatagagaatttgaacaagaaaatggaagacggattcctagatgtctggacctataatgctgaacttctagttctcatggaaaacgagaggactctagatttccatgattcgaatgtcaagaacctttacgacaaagtcagactacagcttagggataatgcaaaggagctgggtaacggctgtttcgaattctatcacaaatgcgataatgaatgtatggaaagtgtgagaaatgggacgtatgactaccctcagtattcggaagaagcaagattaaaaagagaagaaataagcggagtgaaattagaatcaataggaacttaccagatactgtcaatttattcaacagcggcgagttccctagcactggcaatcatgatggctggtctatctttatggatgtgctccaatgggtcgttacagtgcagaatttgcatt

>H5N8_A_chicken_Iraq_1_2020

atggagaacatagtacttcttcttgcaatagttagccttgttaaaagtgatcagatttgcattggttaccatgcaaacaattcgacagagcaagttgacacgataatggaaaagaacgtcactgttacacatgcccaagacatactggaaaaaacacacaacgggaagctctgtgatctaaatggggtgaagcctctgattttaaaggattgtagtgtagctggatggctcctcggaaacccaatgtgcgacgaattcatcagagtgccggaatggtcctacatagtggagagggctaatccagctaatgacctctgttacccagggagcctcaatgactatgaagaactgaaacacctgttgagcagaataaatcattttgagaagattctgatcatccccaagagttcctggtccaatcatgaaacatcactaggggtgagcgcagcttgtccataccagggagcgccctcctttttcagaaatgtggtgtggcttatcaaaaagaacgatgcatacccaacaataaagataagctacaataataccaatcgggaagatctcttgatactgtgggggattcatcattccaacaatgcagaagagcagacaaatctctataaaaacccaaccacctacatttcagttggaacatcaactttaaaccagaggttggtaccaaaaatagctactagatcccaagtaaacgggcaacgtggaagaatggacttcttctggacaattttaaaaccggatgatgcaatccatttcgagagtaatggaaatttcattgctccagaatatgcatacaaaattgtcaagaaaggggactcaacaattatgaaaagtggagtggaatatggccactgcaacaccaaatgtcaaaccccagtaggagcgataaattctagtatgccattccacaacatacatcctctcaccattggggaatgccccaaatacgtgaagtcaaacaagttggtccttgcgactgggctcagaaatagtcctcttagagaaGGGaagagaagaaaaagaggcctgtttggggcgatagcagggtttatagagggaggatggcagggaatggttgatggttggtatgggtaccaccatagcaatgagcaggggagtgggtacgctgcagacaaagaatccacccaaaaggcaatagatggagtcaccaataaggtcaactcaatcattgacaaaatgaacactcaatttgaggcagttggaagggagtttaataacttagaaaggaggatagagaatttgaacaagaaaatggaagacggatttctagatgtctggacctataatgctgaacttctagttctcatggaaaacgagagaactctagatttccatgattcaaatgtcaagaacctttacgacaaagtcagactgcagcttagggataatgcaaaggagctgggtaacggctgtttcgaattctatcacaaatgcgataatgaatgtatggaaagtgtgagaaatgggacgtatgactaccctcagtattcagaagaagcaagattaaaaagagaagaaataagcggagtgaaattagaatcaataggaacttaccagatactgtcaatttattcaacagcggcgagttccctagcactggcaatcatgatggctggtctatctttatggatgtgctccaatgggtcgttacagtgcagaatttgcatt

>H5N6_A_Anas_platyrhynchos_Korea_W612_2017

atggagaacatagtgcttcttcttgcaatagttagccttgttaaaagtgatcagatttgcattggttaccatgcaaacaactcgacagagcaagttgacacgataatggaaaagaacgtcactgttacacatgcccaagacatactggagaaaacacacaacgggaagctctgcgatctaaatggagtgaagcctctgattttaaaggattgtagtgtagctggatggctcctcggaaacccaatgtgcgacgaattcatcagagtgccggaatggtcttacatagtggagagggataatccagctaatgacctctgttacccagggagcctcaatgactatgaagaactgaaacacctgttgagcagaataaatcattttgagaagattctgatcatccccaagagttcttggcccaatcatgaaacatcattaggggtgagcacagcttgtccataccagggagcgccctcctttttcagaaatgtggtatggcttatcaaaaagaacgatgcataccccacaataaagataagctacaataataccaatcgggaagatctcttgatactgtgggggattcatcattccaacaatgcagaagagcagacaaatctctataaaaacccaaccacctatatttcagttggaacatcaacattaaaccagagattggtaccaaaaatagctactagatcccaagtaaacgggcaacgtggaagaatggacttcttctggacaattttaaaaccgaatgatgcaattcatttcgagagtaatggaaatttcattgctccagaatatgcatacaaaattgtcaagaaaggggactcaacaattatgaaaagtggagtggaatatggccactgcaacaccaaatgtcaaaccccagtaggagcgataaactctagtatgccgttccacaatatacatcctctcaccattggggaatgccccaaatacgtgaagtcaaacaagttggtccttgcaactgggctcagaaatagtcctctaagagaaGGGaagagaagaaaaagagggctgtttggggcgatagcaggttttatagagggaggatggcagggaatggttgatggttggtatggctaccaccatagcaatgagcaggggagtgggtacgctgcagacaaagagtccacccaaaaggcaatagatggagttaccaataaggtcaactcgatcattgacaaaatgaacactcaatttgaggcagttggaagggagtttaataacttagaaaggaggatagagaatttgaacaagaaaatggaagacggattcctagatgtctggacctataatgctgaacttctagttctcatggaaaacgagaggactctagatttccatgactcaaatgtcaagaacctttacgacaaagtcagactgcagcttagggataatgcaaaggagctgggtaacggttgtttcgaattctatcacaaatgtgataatgaatgtatggaaagtgtgagaaatgggacgtatgactaccctcagtactcagaagaagcaagattaaaaagagaagaaataagcggagttaaattagaatcaataggaacttaccaaatactgtcaatttattcaacagtggcgagttccctagcactggcaatcatggtggctggtctatctttatggatgtgctccaatgggtcgttacagtgcagaatttgcatt

>H5N6_A_duck_Nigeria_SK28T_19VIR8424_2_2019

atggagaacatagtgcttcttcttgcaatagttagccttgttaaaagtgatcagatttgcattggttaccatgcaaacaactcgacagagcaagttgacacgataatggaaaagaacgtcactgttacacatgcccaagacatactagaaaaaacacacaacgggaagctctgcgatctaaatggagtgaagcctctgattttaaatgattgtagtgtagctggatggctcctcggaaacccaatgtgcgacgaattcatcagagtgccggaatggtcttacatagtggagagggataatccagctgatgacctctgttacccagggagcctcaatgattatgaagaactgaaacacctgttgagcagaataaatcattttgagaagattctgatcatccccaagagttcttggcccaatcatgaaacatcattaggggtgagtgcagcttgtccataccaggggacgccctcctttttcagaaacgtggtatggcttatcaaaaagaacgatgcataccccacaataaagatgagctacaataataccaatcgggaagatctcttgatactgtgggggattcatcattccaacaatgcagaagagcagacaaatctctataaaaacccaaccacctatatttcagttggaacatcaacattaaaccagagattggtaccaaaaatagctaccagatcccaagtaaacgggcaacgtggaagaatggacttcttctggacaattttaaaaccgaatgatgcaatccatttcgagagtaatggaaatttcattgctccagaatatgcatacaaaattgtcaagaaaggggactcaacaattatgaaaagtggagtggaatatggccactgcaacaccaaatgtcaaaccccagtaggagcgataaactctagtatgccgttccacaatatacatcctctcaccattggggaatgccccaaatacgtgaagtcaaacaagttggtccttgcgactgggcttagaaatagtcctctaagagaaGGGaagagaagaaaaagagggctgtttggggcgatagcagggtttatagagggaggatggcagggaatggttgatggttggtatggctaccaccatagcaatgagcaggggagtgggtacgctgcagacaaagagtccacccaaaaggcaatagatggagttaccaataaggtcaactcgatcattgacaaaatgaacactcaatttgaggcaattggaagggagtttaataacttagagaggaggatagagaatttgaacaagaaaatggaagacggattcctagatgtctggacctataatgctgaacttctagttctcatggaaaacgagaggactctagatttccatgactcaaatgtcaagaacctttacgacaaagtcagactgcagcttagggacaatgcaaaggagctgggtaacggttgtttcgaattctatcacaaatgtgataatgaatgtatggaaagtgtgagaaatgggacgtatgactaccctcagtactcagaagaagcaagattaaaaagagaagaaataagcggagttaaattagaatcaataggaacttaccaaatactgtcagtttattcaacagtggcgagttccctagcactggcaatcatggtggctggtctatctttgtggatgtgctccaatgggtcgttacagtgcagaatttgcatt

>H5N8_A_swan_Krasnodar_44_2017

atggagaacatagtgcttcttcttgcaatagttagccttgttaaaagtgatcagatttgcattggttaccatgcaaacaactcgacagagcaagttgacacgataatggaaaagaacgtcactgttacacatgcccaagacatactggaaaaaacacacaacgggaagctctgcgatctaaatggggtgaagcctctgattttaaaggattgtagtgtagctggatggctcctcggaaacccaatgtgcgacgaattcatcagagtgccggaatggtcttacatagtggagagggctaatccagctaatgacctctgttacccagggagcctcaatgactatgaagaactgaaacacctgttgagcagaataaatcattttgagaagattctgatcatccccaagagttcttggcccaatcatgaaacatcattaggggtgagcgcagcttgtccataccagggaacgccctcctttttcagaaatgtggtatggcttatcaaaaagaacgatgcatacccaacaataaagataagctacaataataccaatcgggaagatctcttgatactgtggggaatccatcattccaacaatgcagaagagcagacaaatctctataaaaacccaaccacctatatttcagttggaacatcaacattaaaccagagattggtaccaaaaatagctactagatcccaagtaaacgggcaacgtggaagaatggacttcttctggacaattttaaaaccgaatgatgcaatccatttcgagagtaatggaaatttcattgctccagaatatgcatacaaaattgtcaagaaaggggactcaacaattatgaaaagtggagtggaatatggccactgcaacaccaaatgtcaaaccccagtaggagcgataaactctagtatgccgttccacaatatacatcctctcaccatcggggaatgccccaaatacgtgaagtcaaacaagttggtccttgcgactgggctcagaaatagtcctctaaaagaaGGGaagagaagaaaaagagggctgtttggggctatagcaggttttatagagggaggatggcagggaatggttgatggttggtatgggtaccaccatagcaatgagcaggggagtgggtacgctgcagacaaagaatccacccaaaaggcaatagatggagttaccaataaggtcaactcgatcattgacaaaatgaacactcaatttgaggcagttggaagggagtttaataacttagaaaggaggatagagaatttgaacaagaaaatggaagacggattcctagatgtctggacctataatgctgaacttctagttctcatggaaaacgagaggactctagatttccatgactcaaatgtcaagaacctttacgacaaagtcagactgcagcttagggataatgcaaaggagctgggtaacggttgtttcgaattctatcacaaatgtgataatgaatgtatggaaagtgtgagaaatgggacgtatgactaccctcattattcagaagaagcaagattaaaaagagtagaaacaagcggagtgaaattagaatcaataggaacttaccaaatactgtcaatttattcaacagtggcgagttccctagcactggcaatcatggtggctggtctatctttatggatgtgctccaatgggtcgttacagtgcagaatttgcatt

>H5N8_A_chicken_Moscow_94_2017

atggagaacatagtgcttcttcttgcaatagttagccttgttaaaagtgatcagatttgcattggttaccatgcaaacaactctacagagcaagttgacacgataatggaaaagaacgtcactgttacacatgcccaagacatactggaaaaaacacacaacgggaagctctgcgatctaaatggggtgaagcctctgattttaaaggattgtagtgtagctggatggctcctcggaaacccaatgtgcgacgaattcatcagagtgccggaatggtcttacatagtggagagggctaatccagttaatgacctctgttacccagggagcctcaatgactatgaagaactgaaacacctgttgagcagaataaatcattttgagaagattctgatcatccccaagagttcttggaccaatcatgaaacatcattaggggtgagcgcagcttgtccataccaggggacgccctcctttttcagaaatgtggtatggcttatcaaaaagaacgatgcatacccaacaataaagataagctacaataataccaatcgggaagatctcttgatactgtggggaatccatcattccaacaatgcagaagagcagacaaatctctataaaaacccaaccacctatatttcagttggaacatcaacattaaaccagagattggtaccaaaaatagctactagatcccaagtaaatgggcaacgtggaagaatggacttcttctggacaattttaaaaccgaatgatgcaatccatttcgagagtaatggaaatttcattgctccagaatatgcatacaaaattgtcaagaaaggggactcaacaattatgaaaagtggagtggaatatggccactgcaacaccaaatgtcaaaccccagtaggagcgataaactctagtatgccgttccacaatatacatcctctcaccatcggggaatgccccaaatacgtgaagtcaaacaagttggtccttgcgactgggctcagaaatagtcctctaagagaaGGGaagagaagaaaaagagggctgtttggggctatagcaggttttatagagggaggatggcagggaatggttgatggttggtatgggtaccaccatagcaatgagcaggggagtgggtacgctgcagacaaagaatccacccaaaaggcaatagatggagtcaccaataaggtcaactcgatcattgacaaaatgaacactcaatttgaggcagttggaagggagtttaataacttagaaaggaggatagagaatttgaacaagaaaatggaagacggattcctagatgtctggacctataatgctgaacttctagttctcatggaaaacgagaggactctagatttccatgactcaaatgtcaagaacctttacgacaaagtcagactgcagcttagggataatgcaaaggagctgggtaacggttgtttcgaattctatcacaaatgtgataatgaatgtatggaaagtgtgagaaatgggacgtatgactaccctcagtattcagaagaagcaagattaaaaagagtagaaacaagcggagtgaaattagaatcaataggaacttaccaaatactgtcaatttattcaacagtggcgagttccctagcactggcaatcatggtggctggtctatctttatggatgtgctccaatgggtcgttacaatgcagaatttgcatt

>H5N8_A_cattle_egret_Monofiya_VRLCU_2019

atggagaacatagtgcttcttcttgcaatagttagccttgttaaaagtgatcagatttgcattggttaccatgcaaacaactcgacagagcaagttgacacgataatggaaaagaacgtcactgttacacatgcccaagacatactggagaaaacacacaacgggaagctctgcgatctaaatggggtgaagcctctgattttaaaggattgtagtgtagctggatggctcctcggaaacccaatgtgcgacgaattcatcagagtgccggaatggtcttacatagtggagagggctaatccagctaatgacctctgttacccagggagcctcaatgactatgaagaactgaaacacctgttgagcagaataaatcattttgagaagattctgatcatccccaagagctcttggcccaatcatgaaacatcattaggggtgagcgcagcttgtccataccagggaacgccttcctttttcagaaatgtggtatggcttatcaaaaagaacgatgcatacccaacaataaagataagctacaataataccaatcgggaagatctcttgatactgtggggaatccatcattccaacaatgcagaagagcagacaaatctctataaaaacccaaccacctatatttcagttggaacatcaacattaaaccagagattggtaccaaaaatagctactagatcccaagtaaacgggcaacgtggaagaatggacttcttctggacaattttaaaaccgaatgatgcaatccatttcgagagtaatggaaatttcattgctccagaatatgcatacaaaattgtcaagaaaggggactcaacaattatgaaaagtgaagtggaatatggccactgcaacaccaaatgtcaaaccccagtaggagcgataaactctagtatgccgttccacaatatacatcctctcaccatcggggaatgccccaaatacgtgaagtcaaacaagttggtccttgcgactgggctcagaaatagtcctctaagagagGGGaagagaagaaaaagagggctgtttggggctatagcaggttttatagaaggaggatggcaaggaatggttgatggttggtatgggtaccaccatagcaatgagcaggggagtgggtacgccgcagacaaagaatccacccaaaaggcaatagatggagttaccaataaggtcaactcgatcattgacaaaatgaacactcaatttgaggcagttggaagggagtttaataacttagaaaggaggatagaaaatttgaacaagaaaatggaagacggattcctagatgtctggacctataatgctgaacttctagttctcatggaaaacgagaggactctagatttccatgactcaaatgtcaagaacctttacgacaaagtcagactgcagcttagggataatgcaaaggagctgggtaacggttgtttcgaattctatcacaaatgtgataatgaatgtatggaaagtgtgagaaatgggacgtatgactaccctcagtattcagaagaagcaagattaaaaagagaagaaacaagcggagtgaaattagaatcaataggaacttaccaaatactgtcaatttattcaacagtggcgagttccctagcactggcaatcatggtggctggtctgtctttatggatgtgctccaatgggtcgctacagtgcagaatttgcatt

>H5N8_A_swan_Voronezh_2_2017

atggagaacatagtgcttcttcttgcaatagttagccttgttaaaagtgatcagatttgcattggttaccatgcaaacaactcgacagagcaagttgacacgataatggaaaagaacgtcactgttacacatgcccaagacatactggaaaaaacacacaacgggaagctctgcgatctaaatggggtgaagcctctgattttaaaggattgtagtgtagctggatggctcctcggaaacccaatgtgcgacgaattcatcagcgtgccggaatggtcttacatagtggagagggctaatccagctaatgacctctgttacccagggagcctcaatgactatgaagaactgaaacacctgttgagcagaataaatcattttgagaagattctgatcatccccaagagttcttggcccaaccatgaaacatcattaggggtgagcgcagcctgtccataccagggaacgccctcctttttcagaaatgtggtatggcttatcaaaaagaacgatgcatacccaacaataaagataagctacaataataccaatcgggaagatctcttgatactgtggggaattcatcattccaacaatgcagaagagcagacaaatctctataaaaacccaaccacctatatttcagttggaacatcaacattaaaccagagattggtaccaaaaatagctactagatcccaagtaaacggccaacgtggaagaatggacttcttctggacaattttaaaaccgaatgatgcaatccatttcgagagtaatggaaatttcattgctccagaatatgcatacaaaattgtcaagaaaggggactcaacaattatgaaaagtggagtggaatatggccactgcaacaccaaatgtcaaaccccagtaggagcgataaactctagtatgccgttccacaatatacatcctctcaccatcggggaatgccccaaatacgtgaagtcaaacaagttggtccttgcgactgggctcagaaatagtcctctaagagaaGGGaagagaagaaaaagagggctgtttggggctatagcaggttttatagagggaggatggcagggaatggttgatggttggtatgggtaccaccatagcaatgagcaggggagtgggtacgctgcagacaaagaatccacccaaaaggcaatagatggagttaccaataaggtcaactcgatcattgacaaaatgaacactcaatttgaggcagttggaagggagtttaataacttagaaaggaggatagagaatttgaacaagaaaatggaagacggattcctagatgtctggacctataatgctgaacttctagttctcatggaaaacgagaggactctagatttccatgactcaaatgtcaagaacctttacgacaaagtcagactgcagcttagggataatgcaaaggagctgggtaatggttgtttcgaattctatcacaaatgtgataatgaatgtatggaaagtgtgagaaatgggacgtatgactaccctcagtattcagaagaagcaagattaaaaagagaagaaacaagcggagtgaaattagaatcaataggaacttaccaaatactgtcaatttattcaacagtggcgagttccctagcactggcaatcatggtggctggtctatctttatggatgtgctccaatgggtcgttacagtgcagaatttgcatt

>H5N8_A_Cygnus_olor_Belgium_2967_2017

atggagaacatagtgcttcttcttgcaatagttagccttgttaaaagtgatcagatttgcattggttaccatgcaaacaactcgacagagcaagttgacacgataatggaaaagaacgtcactgttacacatgcccaagacatactggaaaaaacacacaacgggaagctctgcgatctaaatggggtgaagcctctgattttaaaggattgtagtgtagctggatggctcctcggaaacccaatgtgcgacgaattcatcagagtgccggaatggtcttacatagtggagagggctaacccagctaatgacctctgttacccagggagcctcaatgactatgaagaactgaaacacctgttgagcagaataaatcattttgagaagattctgatcatccccaagagttcttggcccaatcatgaaacatcattaggggtgagcgcagcttgtccataccagggaacgccctcctttttcagaaatgtggtatggcttatcaaaaagaacgatgcatacccaacaataaagataagctacaataataccaatcgggaagatctcttgatactgtgggggattcatcattccaacaatgcagaagagcagacaaatctctataaaaacccaaccacctatatttcagttggaacatcaacattaaaccagagattggtaccaaaaatagctactagatcccaagtaaacgggcaacgtggaagaatggacttcttctggacaattttaaaaccggatgatgcaatccacttcgagagtaatggaaatttcattgctccagaatatgcatacaaaattgtcaagaaaggggactcaacaattatgaaaagtggagtggaatatggccactgcaacaccaaatgtcaaaccccagtaggagcgataaactctagtatgccgttccacaatatacatcctctcaccatcggggaatgccccaaatacgtgaagtcaaacaagttggtccttgcgactgggctcagaaatagtcctctaaaagaaGGGaagagaagaaaaagagggctgtttggggctatagcaggttttatagagggaggatggcagggaatggttgatggttggtatgggtaccaccatagcaatgagcaggggagtgggtacgctgcagacaaagaatccacccaaaaggcaatagatggagttaccaataaggtcaactcgatcattgacaaaatgaacactcaatttgaggcagttggaagggagtttaataacttagaaaggaggatagagaatttgaacaagaaaatggaagacggattcctagatgtctggacctataatgctgaacttctagttctcatggaaaacgagaggactctagatttccatgactcaaatgtcaagaacctttacgacaaagtcagactgcagcttagggataatgcaaaggagctgggtaacggttgtttcgaattctatcacaaatgtgataatgaatgtatggaaagtgtgagaaatgggacgtatgactaccctcagtattcagaagaagcaagattaaaaagagaagaaacaagcggagtgaaattagaatcaataggaacttaccaaatactgtcaatttattcaacagtggcgagttccctagcactggcaatcatggtggctggtctatctttatggatgtgctccaatgggtcgttacagtgcagaatttgcatt

>H5N8_A_goose_Kalmykia_813_2016

atggagaacatagtgcttcttcttgcaatagttagccttgttaaaagtgatcagatttgcattggttaccatgcaaacaactcgacagagcaagttgacacgataatggaaaagaacgtcactgttacacatgcccaagacatactggaaaaaacacacaacgggaagctctgcgatctaaatggggtgaagcctctgattttaaaggattgtagtgtagctggatggctcctcggaaacccaatgtgcgacgaattcatcagagtgccggaatggtcttacatagtggagagggctaatccagctaatgacctctgttacccagggagcctcaatgactatgaagaactgaaacacctgttgagcagaataaatcattttgagaagattctgatcatccccaagagttcttggcccaatcatgaaacatcattaggggtgagcgcagcttgtccataccagggaacgccctcctttttcagaaatgtggtatggcttatcaaaaagaacgatgcatacccaacaataaaaataagctacaataataccaatcgggaagatctcttgatactgtgggggattcatcattccaacaatgcagaagagcagacaaatctctataaaaacccaaccacctatatttcagttggaacatcaacattaaaccagagattggtaccaaaaatagctactagatcccaagtaaacgggcaacgtggaagaatggacttcttctggacaattttaaaaccgaatgatgcaatccatttcgagagtaatggaaatttcattgctccagaatatgcatacaaaattgtcaagaaaggggactcaacaattatgaaaagtggagtggaatatggccactgcaacaccaaatgtcaaaccccagtaggagcgataaactctagtatgccgttccacaatatacatcctctcaccatcggggaatgccccaaatacgtgaagtcaaacaagttggtccttgcgactgggctcagaaatagtcctctaagagaaGGGaagagaagaaaaagagggctgtttggggctatagcaggttttatagagggaggatggcagggaatggttgatggttggtatgggtaccaccatagcaatgagcaggggagtgggtacgctgcagacaaagagtccacccaaaaggcaatagatggagttaccaataaggtcaactcgatcattgacaaaatgaacactcaatttgaggcagttggaagggagtttaataacttagaaaggaggatagagaatttgaacaagaaaatggaagacggattcctagatgtctggacctataatgctgaacttctagttctcatggaaaacgagaggactctagatttccatgactcaaatgtcaagaacctttacgacaaagtcagactgcagcttagggataatgcaaaggagctgggtaacggttgtttcgaattctatcacaaatgtgataatgaatgtatggaaagtgtgagaaatgggacgtatgactaccctcagtattcagaagaagcaagattaaaaagagtagaaacaagcggagtgaaattagaatcaataggaacttaccaaatactgtcaatttattcaacagtggcgagttccctagcactggcaatcatggtggctggtctatctttatggatgtgctccaatgggtcgttacagtgcagaatttgcatt

>H5N6_A_jungle_crow_Hyogo_2803E022_2018

atggagaacatagtgcttcttctcgcaataattagccttgttaaaagtgatcagatttgcattggttaccatgcaaacaactcgacagagcaagttgacacgataatggaaaagaacgtcactgttacacatgcccaagacatactggaaaaaacacacaacgggaagctctgcgatctaaatggggtgaagcctctgattttaaaggattgtagtgtagctggatggctcctcgggaacccaatgtgcgacgaattcatcagagtgccggaatggtcttacatagtggagagggctaatccagctaatgacctctgttacccagggagtctcaatgactatgaagaactgaaacacctgttgagcagaataaatcattttgagaagattctgatcatccccaagagttcttggcccaatcatgaaacatcattaggggtgagcgcagcttgtccataccagggaacaccctcctttttcagaaatgtggtatggcttatcaataagaacgatgcatacccaacaataaagataagctacaataataccaatcgggaagatctcttgatactgtgggggattcatcatcccaacaatgcggaagagcagacaaatctttataaaaacccaaccacctatatttcagttggaacatcaacattaaaccagagattggtaccaaaaatagctactagatcccaagtaaacgggcaacgtggaagaatggacttcttctggacaattttaaaaccgaatgatgcaatccatttcgagagtaatggaaatttcattgctccagaatatgcatacaaaattgtcaagaaaggggactcaacaattatgaaaagtggagtggaatatggccactgcaacaccaaatgtcaaaccccagtaggagcgataaactctagtatgccgttccacaatatacatcctctcaccatcggggaatgccccaaatacgtgaagtcaaacaagttggtccttgcgactgggctcagaaatagtcctctaagagaaGGGaggagaagaaagagagggctgtttggggctatagcaggttttatagagggaggatggcagggaatggttgatggttggtatgggtaccaccatagcaatgagcagggaagtggatacgctgcagacaaagaatccacccaaaaggcaatagatggagttaccaataaggtcaactcgatcattgacaaaatgaacactcaatttgaggcagttggaagggagtttaataacttagaaaggaggatagagaatttgaacaagaaaatggaagacggattcctagatgtctggacctataatgctgaacttctagttctcatggaaaacgagaggactctagatttccatgactcaaatgtcaagaacctttacgacaaagtcagactgcagcttagggataatgcaaaggagctgggtaacggttgtttcgaattctatcacaaatgtgataatgaatgtatggaaagtgtgagaaatgggacgtatgactaccctcagtattcagaagaagcaagattaaaaagagaagaaataagcggagtgaaattagaatcaataggaacttaccaaatactgtcaatttattcaacagtggcgagttccctagcactggcaatcatggtggctggtctatctttatggatgtgctccaatgggtcgttacagtgcagaatttgcatt

>H5N6_A_wild_duck_South_Korea_1801_2018

atggagaacatagtgcttcttcttgcaataattagccttgttaaaagtgatcagatttgcattggttaccatgcaaacaactcgacagagcaagttgacacgataatggaaaagaacgtcactgttacacatgcccaagacatactggaaaaaacacacaacgggaagctctgcgatctaaatggggtgaagcctctgattttaaaggattgtagtgtagctggatggctcctcgggaacccaatgtgcgacgaattcatcagagtgccggaatggtcttacatagtggagagggctaatccagctaatgacctctgttacccagggagtctcaatgactatgaagaactgaaacacctgttgagcagaataaatcattttgagaagattctgatcatccccaagagttcttggcccaatcatgaaacatcattaggggtgagcgcagcttgtccataccagggaacaccctcctttttcagaaatgtggtatggcttatcaataagaacaatgcatacccaacaataaagataagctacaataataccaatcgggaagatctcttgatactgtgggggattcatcatcccaacaatgcggaagagcagacaaatctttataaaaacccaaccacctatatttcagttggaacatcaacattaaaccagagattggtaccaaaaatagctactagatccccagtaaacgggcaacgtggaagaatggacttcttctggacaattttaaaaccgaatgatgcaatccatttcgagagtaatggaaatttcattgctccagaatatgcatacaaaattgtcaagaaaggggactcaacaattatgaaaagtggggtggaatatggccactgcaacaccaaatgtcaaaccccagtaggagcgataaactctagtatgccgttccacaatatacatcctctcaccatcggggaatgccccaaatacgtgaagtcaaacaagttggtccttgcgactgggctcagaaatagtcctctaagagaaGGGaggagaagaaagagagggctgtttggggctatagcaggttttatagagggaggatggcagggaatggttgatggttggtatgggtaccaccatagcaatgagcagggaagtggatacgctgcagacaaagaatccacccaaaaggcaatagatggagttaccaataaggtcaactcgatcattgacaaaatgaacactcaatttgaggcagttggaagggagtttaataacttagaaaggaggatagagaatttgaacaagaaaatggaagacggattcctagatgtctggacctataatgctgaacttctagttctcatggaaaacgagaggactctagatttccatgactcaaatgtcaagaacctttacgacaaagtcagactgcagcttagggataatgcaaaggagctgggtaacggttgtttcgaattctatcacaaatgtgataatgaatgtatggaaagtgtgagaaatgggacgtatgactaccctcagtattcagaagaagcaagattaaaaagagaagaaataagcggagtgaaattagaatcaataggaacttaccaaatactgtcaatttattcaacagtggcgagttccctagcactggcaatcatggtggctggtctatctttatggatgtgctccaatgggtcgttacagtgcagaatttgcatt

>H5N6_A_tufted_duck_Shimane_3211TY001_2017

atggagaacatagtgcttcttcttgcaataattagccttgttaaaagtgatcagatttgcattggttaccatgcaaacaactcgacagagcaagttgacacgataatggaaaagaacgtcactgttacacatgcccaagacatactggaaaaaacacacaacgggaagctctgcgatctaaatggggtgaagcctctgattttaaaggattgtagtgtagctggatggctcctcgggaacccaatgtgcgacgaattcatcagagtgccggaatggtcttacatagtggagagggctaatccagctaatgacctctgttacccagggagtctcaatgactatgaagaactgaaacacctgttgagcagaataaatcattttgagaagattctgatcatccccaagagttcttggcccaatcatgaaacatcattaggggtgagcgcagcttgtccataccagggaacaccctcctttttcagaaatgtggtatggcttatcaataagaacgatgcatacccaacaataaagataagctacaataataccaatcgggaagatctcttgatactgtgggggattcatcatcccaacaatgcggaagagcagacaaatctttataaaaacccaaccacctatatttcagttggaacatcaacattaaaccagagattggtaccaaaaatagctactagatcccaagtaaacgggcaacgtggaagaatggacttcttctggacaattttaaaaccgaatgatgcaatccatttcgagagtaatggaaatttcattgctccagaatatgcatacaaaattgtcaagaaaggggactcaacaattatgaaaagtgaagtggaatatggccactgcaacaccaaatgtcaaaccccagtaggagcgataaactctagtatgccgttccataatatacatcctctcaccatcggggaatgccccaaatacgtgaagtcaaacaagttggtccttgcgactgggctcagaaatagtcctctaagagaaGGGaggagaagaaagagagggctgtttggggctatagcaggttttatagagggaggatggcagggaatggttgatggttggtatgggtaccaccatagcaatgagcagggaagtggatacgctgcagacaaagaatccacccaaaaggcaatagatggagttaccaataaggtcaactcgatcattgacaaaatgaacactcaatttgaggcagttggaagggagtttaataacttagaaaggaggatagagaatttgaacaagaaaatggaagacggattcctagatgtctggacctataatgctgaacttctagttctcatggaaaacgagaggactctagatttccatgactcaaatgtcaagaacctttacgacaaagtcagactgcagcttagggataatgcaaaggagctgggtaacggttgtttcgaattctatcacaaatgtgataatgaatgtatggaaagtgtgagaaatgggacgtatgactaccctcagtattcagaagaagcaagattaaaaagagaagaaataagcggagtgaaattagaatcaataggaacttaccaaatactgtcaatttattcaacagtggcgagttccctagcactggcaatcatggtggctggtctatctttatggatgtgctccaatgggtcgttacagtgcagaatttgcatt

>H5N8_A_Buteo_buteo_Belgium_3022_2017

atggagaacatggtgcttcttcttgcaatagttagccttgttaaaagtgatcagatttgcattggttaccatgcaaacaactcgacagagcaagttgacacgataatggaaaagaacgtcactgttacacatgcccaagacatactggaaaaaacacacaacgggaagctctgcgatctaaatggggtgaagcctctgattttaaaggattgtagtgtagctggatggctcctcggaaacccaatgtgcgacgaattcatcagagtgccggaatggtcttacatagtggagagggctaacccaactaatgacctctgttacccagggagcctcaatgactatgaagaactgaaacacctgttgagcagaataaatcattttgagaagattcagatcatccccaaaagttcttggcccaatcatgaaacatcattaggggtaagcgcagcttgtccatatcagggaacgccctcctttttcagaaatgtggtatggcttatcaaaaagaacgatgcatacccaacaataaagataagctacaataataccaatcgggaagatctcttgatactgtgggggattcatcattccaacaatgcagaagagcagacaaatctctacaaaaacccaaccacctatatttcagttggaacatcaacattaaaccagagattggtaccaaaaatagctactagatcccaagtaaacgggcaacgtggaagaatggacttcttctggacaattttaaaaccgaatgatgcaatccacttcgagagtaatggaaatttcattgctccagaatatgcatacaaaattgtcaagaaaggggactcaacaattatgaaaagtggagtggaatatggccactgcaacaccaaatgtcaaaccccagtaggagcgataaactctagtatgccgttccacaatatacatcctctcaccatcggggaatgccccaaatacgtgaagtcaaacaagttggtccttgcgactgggctcagaaatagtcctctaagagaaGGGaagagaagaaaaagagggctgtttggggctatagcaggttttatagagggaggatggcagggaatggttgatggttggtatgggtaccaccatagcaatgagcaggggagtgggtacgctgcagacaaagaatccacccaaaaggcaatagatggagttaccaataaggtcaactcgatcattgacaaaatgaacactcaatttgaggcagttggaagggagtttaataacttagaaaggaggatagagaatttgaacaagaaaatggaagacggattcctagatgtctggacctataatgctgaacttctagttctcatggaaaatgaaaggactctagatttccatgactcaaatgtcaagaacctttacgacaaagtcagactgcagcttagggataatgcaaaggagctgggtaacggttgtttcgagttctatcacaaatgtgataatgaatgcatggaaagtgtgagaaatgggacgtatgactaccctcagtattcagaagaagcaagattaaaaagagaagaaataggcggagtgaaactagaatcaataggaacttaccaaatactgtcaatttattcaacagtggcgagttccctagcactggcaatcatggtggctggtctatctttatggatgtgctccaatgggtcgttacagtgcagaatttgcatt

>H5N8_A_Anas_platyrhynchos_Belgium_1899_2017

atggagaacatggtgcttcttcttgcaatagttagccttgttaaaagtgatcagatttgcattggttaccatgcaaacaactcgacagagcaagttgacacgataatggaaaagaacgtcactgttacacatgcccaagacatactggaaaaaacacacaacgggaagctctgcgatctaaatggggtgaagcctctgattttaaaggattgtagtgtagctggatggctcctcggaaacccaatgtgcgacgaattcatcagagtgccggaatggtcttacatagtggagagggctaacccaactaatgacctctgttacccagggagcctcaatgactatgaagaactgaaacacctgttgagcagaataaatcattttgagaagattcagatcatccccaaaagttcttggcccaatcatgaaacatcattaggggtaagcgcagcttgtccatatcagggaacgccctcctttttcagaaatgtggtatggcttatcaaaaagaacgatgcatacccaacaataaagataagctacaataataccaatcgggaagatctcttgatactgtgggggattcatcattccaacaatgcagaagagcagacaaatctctacaaaaacccaaccacctatatttcagttggaacatcaacattaaaccagagattggtaccaaaaatagctactagatcccaagtaaacgggcaacgtggaagaatggacttcttctggacaattttaaaaccgaatgatgcaatccacttcgagagtaatggaaatttcattgctccagaatatgcatacaaaattgtcaagaaaggggactcaacaattatgaaaagtggagtggaatatggccactgcaacaccaaatgtcaaaccccagtaggagcgataaactctagtatgccgttccacaatatacatcctctcaccatcggggaatgccccaaatacgtgaagtcaaacaagttggtccttgcgactgggctcagaaatagtcctctaagagaaGGGaagagaagaaaaagagggctgtttggggctatagcaggttttatagagggaggatggcagggaatggttgatggttggtatgggtaccaccatagcaatgagcaggggagtgggtacgctgcagacaaagaatccacccaaaaggcaatagatggagttaccaataaggtcaactcgatcattgacaaaatgaacactcaatttgaggcagttggaagggagtttaataacttagaaaggaggatagagaatttgaacaagaaaatggaagacggattcctagatgtctggacctataatgctgaacttctagttctcatggaaaatgaaaggactctagatttccatgactcaaatgtcaagaacctttacgacaaagtcagactgcagcttagggataatgcaaaggagctgggtaacggttgtttcgagttctatcacaaatgtgataatgaatgcatggaaagtgtgagaaatgggacgtatgactaccctcagtattcagaagaagcaagattaaaaagagaagaaataggcggagtgaaactagaatcaataggaacttaccaaatactgtcaatttattcaacagtggcgagttccctagcactggcaatcatggtggctggtctatctttatggatgtgctccaatgggtcgttacagtgcagaatttgcatt

>H5N8_A_duck_Bulgaria_78_4t_20VIR1416_3_2020

atggagaaaatagtgcttcttcttgcaatagttagccttgttgaaagtgatcagatttgcattggttaccatgcaaacaactcgacagagcaagttgacacgataatggaaaagaacgtcactgttacacatgcccaagacatactggaaaaaacacacaacgggaagctctgcgatctaaatggggtgaagcctctgattttaaaggattgtagtgtagctggatggctcctcgggaacccaatgtgcgacgaattcatcagagtgccggaatggtcctacatagtggagagggctaacccagccaatgacctctgttacccagggagcctcaatgactatgaagaactgaaacacctgttgagcagaataaatcattttgagaagattctgatcatccccaagagttcttggcccaatcatgaaacatcattaggggtaagcgcagcttgtccatatcagggaacgccctcctttttcagaaatgtggtatggcttatcaaaaagaacgatgcatacccaacaataaagataagctacaataataccaatcgggaagatctcttgatactgtgggggattcatcattccaacaatgcagaagagcagacaaatctctataaaaacccaaccacctatatttcagttggaacatcaacattaaaccagagattggtaccaaaaatagctactagatcccaagtaaacgggcagcgtggaagaatggacttcttctggacaattttaaaaccgaatgatgcaatccacttcgagagtaatggaaatttcattgctccagaatatgcatacaaaattgtcaagaaaggggactcaacaattatgaaaagtggagtggaatatggccactgcaacaccaaatgtcaaaccccagtaggagcgataaactctagtatgccgttccacaatatacatcctctcaccatcggggagtgccccaaatacgtgaagtcaaacaagttggtccttgcgactgggctcagaaatagtcctctaagagaaGGGaagaggaggaaaagagggctgtttggggctatagcaggttttatagagggaggatggcagggaatggttgatggttggtatgggtaccaccatagcaatgagcaggggagtgggtacgctgcagacaaagaatccacccaaaaggcaatagatggagttaccaataaggtcaactcgatcattgacaaaatgaacactcaatttgaggcagttggaagggagtttaataacttagaaaggagaatagagaatttgaacaagaaaatggaagacggattcctagatgtctggacctataatgctgaacttctagttctcatggaaaacgagaggactctagatttccatgactcaaatgtcaagaacctttacgacaaagtcagactgcagcttagggataatgcaaaggagctgggtaacggttgtttcgagttctatcacaaatgtgataatgaatgtatggaaagtgtgagaaacgggacgtatgactaccctcagtattcagaagaagcaagattaaaaagagaagaaataggcggagtgaaattagaatcaataggaacttaccaaatactgtcaatttattcaacagtggcgagttccctagcactggcaatcatggtggctggtctatctttatggatgtgctccaatgggtcgttacagtgcagaatttgcatt

>H5N8_A_whooper_swan_Fukushima_0701B002_2021

atggagaacatagtgcttcttcttgcaatagttagccttgttaaaagtgatcaaatttgcattggttaccatgcaaacaactcgacagaacaagtcgacacgataatggaaaagaacgtcactgttacacatgcccaagacatactggaaaaagcacacaacgggaagctctgcgatttaaatggggtgaaacctctggttttaaatgattgtagtgtagctggatggctcctcggaaacccaatgtgcgacgaattcatcagagtgccggaatggtcttacatagtagagaggactaacccagctaatgacctctgttacccagggagcctcaatgactatgaagaactaaaacacctgttgagcagaataaaacattttgagaagattctgatcatccccaagagttcttggcccaatcatgaaacatcattgggggtgagcgcagcttgtccataccagggaacgccctcctttttcagaaatgtggtatggcttatcaaaaagaatgatgcatacccaacaataaagataaactacaataataccaatcgggaagatctcttgatactgtgggggattcatcattccaacaatgaagaagagcagacaaatctatataaaaacccaaccacctatatttcagttgggacatcaacgttaaaccagagattggtaccaaaaatagctactagatcccaagtaaatgggcagcggggaagaatggacttcttctggacaattttaaaaccaaatgatgcaatccacttcgagagtaatggaaatttcattgctccagaatatgcatacaaaattgtcaagaaaggggactcaacaattatgaaaagtgaagtggaatatggccactgcaacaccaaatgtcaaaccccagtaggagcgataaactctagtatgccattccacaatatacatcctctcaccatcggggaatgccccaaatacgtgaagtcaaacaagttggtccttgcaactgggctcagaaatagtcctctaagagaaGGGaagagaagaaaaagagggctgtttggggcgatagcaggtttcatagagggaggatggcagggaatggttgatggttggtatgggtaccaccatagcaatgagcagggaagtgggtacgctgcagacaaggaatccacccaaaaggcaatagatggagttaccaataaggtcaactcgatcattaacaaaatgaacactcaatttgaggcagttggaagggagtttaataacttagaaaggaggatagagaatttgaacaagaaaatggaagacgggttcctagatgtctggacctataatgctgaacttttagttctcatggaaaacgagaggactctagatttccatgactcaaatgtcaagaacctttacgacaaagtcagactgcagcttagggataatgcaaaggagctgggtaacggttgtttcgagttctatcacaaatgtgataatgaatgtatggaaagtgtgagaaatgggacgtattactaccctcagtattcagaagaagcaagattaaaaagggaagaaataagcggggtgaaattggaatcaataggaacttaccaaatactgtcaatttattcaacagtggcgagttccctagcactggcaatcatggtggctggtctatctttatggatgtgttccaatgggtcgttacagtgcagaatttgcctt

>H5N8_A_mandarin_duck_Korea_WA877_2020

atggagaacatagtgcttcttcttgcaatagttagccttgttaaaagtgatcaaatttgcattggttaccatgcaaacaactcgacagaacaagtcgacacgataatggaaaagaacgtcactgttacacatgcccaagacatactggaaaaagcacacaacgggaagctctgcgatttaaatggggtgaaacctctggttttaaatgattgtagtgtagctggatggctcctcggaaacccaatgtgcgacgaattcatcagagtgccggaatggtcttacatagtagagaggactaacccagctaatgacctctgttacccagggagcctcaatgactatgaagaactaaaacacctgttgagcagaataaaacattttgagaagattctgatcatccccaagagttcttggcccaatcatgaaacatcattgggggtgagcgcagcttgtccataccagggaacgccctcctttttcagaaatgtggtatggcttatcaaaaagaatgatgcatacccaacaataaagataaactacaataataccaatcgggaagatctcttgatactgtgggggattcatcattccaacaatgaagaagagcagacaaatctatataaaaacccaaccacctatatttcagttgggacatcaacgttaaaccagagattggtaccaaaaatagctactagatcccaagtaaatgggcagcggggaagaatggacttcttctggacaattttaaaaccaaatgatgcaatccacttcgagagtaatggaaatttcattgctccagaatatgcatacaaaattgtcaagaaaggggactcaacaattatgaaaagtgaagtggaatatggccactgcaacaccaaatgtcaaaccccaataggagcgataaactctagtatgccattccacaatatacatcctctcaccatcggggaatgccccaaatacgtgaagtcaaacaagttggtccttgcaactgggctcagaaatagtcctctaagagaaGGGaagagaagaaaaagagggctgtttggggcgatagcaggtttcatagagggaggatggcagggaatggttgatggttggtatgggtaccaccatagcaatgagcagggaagtgggtacgctgcagacaaggaatccacccaaaaggcaatagatggagttaccaataaggtcaactcgatcattaacaaaatgaacactcaatttgaggcagttggaagggagtttaataacttagaaaggaggatagagaatttgaacaagaaaatggaagacgggttcctagatgtctggacctataatgctgaacttttagttctcatggaaaacgagaggactctagatttccatgactcaaatgtcaagaacctttacgacaaagtcagactgcagcttagggataatgcaaaggagctgggtaacggttgtttcgagttctatcacaaatgtgataatgaatgtatggaaagtgtgagaaatgggacgtattactaccctcagtattcagaagaagcaagattaaaaagggaagaaataagcggagtgaaattggaatcaataggaacttaccaaatactgtcaatttattcaacagtggcgagttccctagcactggcaatcatggtggctggtctatctttatggatgtgttccaatgggtcgttacagtgcagaatttgcatt

>H5N8_A_chicken_Korea_H450_2020

atggagaacatagtgcttcttcttgcaatagttagccttgttaaaagtgatcaaatttgcattggttaccatgcaaacaactcgacagaacaagtcgacacgataatggaaaagaacgtcactgttacacatgcccaagacatactggaaaaagcacacaacgggaagctctgcgatttaaatggggtgaaacctctggttttaaatgattgtagtgtagctggatggctcctcggaaacccaatgtgcgacgaattcatcagagtgccggaatggtcttacatagtagagaggactaacccagctaatgacctctgttacccagggagcctcaatgactatgaagaactaaaacacctgttgagcagaataaaacattttgagaagattctgatcatccccaagagttcttggcccaatcatgaaacatcattgggggtgagcgcagcttgttcataccagggaacgccctcctttttcagaaatgtggtatggcttatcaaaaagaatgatgcatacccaacaataaagataaactacaataataccaatcgggaagatctcttgatactgtgggggattcatcattccaacaatgaagaagagcagacaaatctatataaaaacccaaccacctatatttcagttgggacatcaacgttaaaccagagattggtaccaaaaatagctactagatcccaagtaaatgggcagcggggaagaatggacttcttctggacaattttaaaaccaaatgatgcaatccacttcgagagtaatggaaatttcattgctccagaatatgcatacaaaattgtcaagaaaggggactcaacaattatgaaaagtgaagtggaatatggccactgcaacaccaaatgtcaaaccccagtaggagcgataaactctagtatgccattccacaatatacatcctctcaccatcggggaatgccccaaatacgtgaagtcaaacaagttggtccttgcaactgggctcagaaatagtcctctaagagaaGGGaagagaagaaaaagagggctgtttggggcgatagcaggtttcatagagggaggatggcagggaatggttgatggttggtatgggtaccaccatagcaatgagcagggaagtgggtacgctgcagacaaggaatccacccaaaaggcaatagatggagttaccaataaggtcaactcgatcattaacaaaatgaacactcaatttgaggcagttggaagggagtttaataacttagaaaggaggatagagaatttgaacaagaaaatggaagacgggttcctagatgtctggacctataatgctgaacttttagttctcatggaaaacgagaggactctagatttccatgactcaaatgtcaagaacctttacgacaaagtcagactgcagcttagggataatgcaaaggagctgggtaacggttgtttcgagttctatcacaaatgtgataatgaatgtatggaaagtgtgagaaatgggacgtattactaccctcagtattcagaagaagcaagattaaaaagggaagaaataagcggagtgaaattggaatcaataggaacttaccaaatactgtcaatttattcaacagtggcgagttccctagcactggcaatcatggtggctggtctatctttatggatgtgttccaatgggtcgttacagtgcagaatttgcatt

>H5N8_A_duck_Korea_H385_2020

atggagaacatagtgcttcttcttgcaatagttagccttgttaaaagtgatcaaatttgcattggttaccatgcaaacaactcgacagaacaagtcgacacgataatggaaaagaacgtcactgttacacatgcccaagacatactggaaaaagcacacaacgggaagctctgcgatttaaatggggtgaaacctctggttttaaatgattgtagtgtagctggatggctcctcggaaacccaatgtgcgacgaattcatcagagtgccggaatggtcttacatagtagagaggactaacccagctaatgacctctgttacccagggagcctcaatgactatgaagaactaaaacacctgttgagcagaataaaacattttgagaagattctgatcatccccaagagttcttggcccaatcatgaaacatcattgggggtgagcgcagcttgtccataccagggaacgccctcctttttcagaaatgtggtatggcttatcaaaaagaatgatgcatacccaacaataaagataaactacaataataccaatcgggaagatctcttgatactgtgggggattcatcattccaacaatgaagaagagcagacaaatctatataaaaacccaaccacctatatttcagttgggacatcaacgttaaaccagagattggtaccaaaaatagctactagatcccaagtaaatgggcagcggggaagaatggattttttctggacaattttaaaaccaaatgatgcaatccacttcgagagtaatggaaatttcattgctccagaatatgcatacaaaattgtcaagaaaggggactcaacaattatgaaaagtgaagtggaatatggccactgcaacaccaaatgtcaaaccccagtaggagcgataaactctagtatgccattccacaatatacatcctctcaccatcggggaatgccccaaatacgtgaagtcaaacaagttggtccttgcaactgggctcagaaatagtcctctaagagaaGGGaagagaagaaaaagagggctgtttggggcgatagcaggtttcatagagggaggatggcagggaatggttgatggttggtatgggtaccaccatagcaatgagcagggaagtgggtacgctgcagacaaggaatccacccaaaaggcaatagatggagttaccaataaggtcaactcgatcattaacaaaatgaacactcaatttgaggcagttggaagggagtttaataacttagaaaggaggatagagaatttgaacaagaaaatggaagacgggttcctagatgtctggacctataatgctgaacttttagtcctcatggaaaacgagaggactctagatttccatgactcaaatgtcaagaacctttacgacaaagtcagactgcagcttagggataatgcaaaggagctgggtaacggttgtttcgagttctatcacaaatgtgataatgaatgtatggaaagtgtgagaaatgggacgtattactaccctcagtattcagaagaagcaagattaaaaagggaggaaataagcggagtgaaattggaatcaataggaacttaccaaatactgtcaatttattcaacagtggcgagttccctagcactggcaatcatggtggctggtctatctttatggatgtgttccaatgggtcgttacagtgcagaatttgcatt

>H5N2_A_whooper_swan_Shanxi_SX116_2020

atggagaacatagtgcttcttcttgcaatagttagccttgttaaaagtgatcaaatttgcattggttaccatgcaaacaactcgacagaacaagtcgacacgataatggaaaagaacgtcactgttacacatgcccaagacatactggaaaaagcacacaacgggaagctctgcgatttaaatggggtgaaacctctggttttaaatgattgtagtgtagctggatggctcctcggaaacccaatgtgcgacgaattcatcagagtgccggaatggtcttacatagtagagaggactaacccagctaatgacctctgttacccagggagcctcaatgactatgaagaactaaaacacctgttgagcagaataaaacattttgagaagattctgatcatccccaagagttcttggcccaatcatgaaacatcattgggggtgagcgcagcttgtccataccagggaacgccctcctttttcaggaatgtggtatggctcatcaaaaagaatgatgcatacccaacaataaagataaactacaataataccaatcgggaagatctcttgatactgtgggggattcatcattccaacaatgaagaagagcagacaaatctatataaaaacccaaccacctatatttcagttgggacatcaacgttaaaccagagattggtaccaaaaatagctactagatcccaagtaaatgggcagcggggaagaatggacttcttctggacaattttaaaaccaaatgatgcaatccacttcgagagtaatggaaatttcattgctccagaatatgcatacaaaattgtcaagaaaggggactcaacaattatgaaaagtgaagtggaatatggccactgcaacaccaaatgtcaaaccccagtaggagcgataaactctagtatgccattccacaatatacatcctctcaccatcggggaatgccccaaatacgtgaagtcaaacaagttggtccttgcaactgggctcagaaatagtcctctaagagaaGGGaagagaagaaaaagagggctgttcggggcgatagcaggtttcatagagggaggatggcagggaatggttgatggttggtatgggtaccaccatagcaatgagcagggaagtgggtacgctgcagacaaggaatccacccaaaaggcaatagatggagttaccaataaggtcaactcgatcattaacaaaatgaacactcaatttgaggcagttggaagggagtttaataacttagaaaggaggatagagaatttgaacaagaaaatggaagacgggttcctagatgtctggacctataatgctgaacttttagttcttatggaaaacgagaggactctagatttccatgactcaaatgtcaagaacctttacgacaaagtcagactgcagcttagggataatgcaaaggagctgggtaacggttgtttcgagttctatcacaaatgtgataatgaatgtatggaaagtgtgagaaatgggacgtattactaccctcagtattcagaagaagcaagattaaaaagggaagaaataagcggagtgaaattggaatcaataggaacttaccaaatactgtcaatttattcaacagtggcgagttccctagcactggcaatcatggtggctggtctatctttatggatgtgttccaatgggtcgttacagtgcagaatttgcatt

>H5N8_A_turkey_Czech_Republic_3071_2020

atggagaacatagtgcttcttctcgcaatagttagccttgttaaaagtgatcaaatttgcattggttaccatgcaaacaactcgacagaacaagtcgacacgataatggaaaagaacgtcactgttacacatgcccaagacatactggaaaaagcacacaacgggaagctctgcgatttaaatggggtgaagcctctggttttaaatgattgtagtgtagctggatggctcctcggaaacccaatgtgcgacgaattcatcagagtgccagaatggtcttacatagtagagaggactaacccagctaatgacctctgttacccaggaagyctcaatgactatgaagaactaaaacacctgttgagcagaataaatcattttgagaagattctgatcatccccaagagttcttggcccaatcatgaaacatcattaggggtgagcgcagcttgtccataccagggaacgccctcctttttcagaaatgtggtatggcttatcaaaaagaatgatgcatacccaacaataaagataaactacaataataccaatcgggaagatctcttgatactgtgggggattcatcattccaacaatgaagaagagcagacaaatctatataaaaacccaaccacctatatttcagttgggacatcaacattaaaccagagattggtaccaaaaatagctactagatcccaagtaaatgggcagcggggaagaatggacttcttctggacaattttaaaaccaaatgatgcaatccacttcgagagtaatggaaatttcattgctccagaatatgcatacaaaattgtcaagaaaggggactcaacaattatgaaaagtgaagtggaatatggccactgcaacaccaaatgtcaaaccccagtaggagcgataaactctagtatgccatttcacaatatacatcctctcaccatcggggaatgccccaaatacgtgaagtcaaacaagttggtccttgcaactgggctcagaaatagtcctctaagagaaGGGaagagaagaaaaagagggctgtttggggcgatagcaggtttcatagagggaggatggcagggaatggttgatggttggtatgggtaccaccatagcaatgagcagggaagtgggtacgctgcagacaaggaatccacccaaaaggcaatagatggagttaccaataaggtcaactcgatcattaacaaaatgaacactcaatttgaggcagttggaagggagtttaataacttagaaaggaggatagagaatttgaacaagaaaatggaagacgggttcctagatgtctggacctataatgctgaacttttagttctcatggaaaacgagaggactctagatttccatgactcaaatgtcaagaacctttacgaaaaagtcagactgcagcttagggataatgcaaaggagctgggtaacggttgtttcgagttctatcacaaatgtgataatgaatgtatggaaagtgtgagaaatgggacgtattactaccctcagtattcagaagaagcaaaattaaaaagagaagaaataagcggagtgaaattagaatcaataggaacttaccaaatactgtcaatttattcaacagtggcgagttccctagcactggcaatcatggtggctggtctatctttatggatgtgttccaatgggtcgttacagtgcagaatttgcatt

>H5N8_A_chicken_Slovakia_Pah_14_2020

atggagaacatagtgcttcttcttgcaatagttagccttgttaaaagtgatcaaatttgcattggttaccatgcaaacaactcgacagaacaagtcgacacgataatggaaaagaacgtcactgttacacatgcccaagacatactggaaaaagcacacaacgggaagctctgcgatttaaatggggtgaagcctctggttttaaatgattgtagtgtagctggatggctcctcggaaacccaatgtgcgacgaattcatcagagtgccggaatggtcttacatagtagagaggactaacccagctaatgacctctgttacccagggagcctcaatgactatgaagaactaaaacacctgttgagcagaataaatcattttgagaagattctgatcatccccaagagttcttggcccaatcatgaaacatcattaggggtgagcgcagcttgtccataccagggaacgccctcctttttcagaaatgtggtatggcttatcaaaaagaatgatgcatacccaacaataaagataaactacaataataccaatcgggaagatctcttgatactgtgggggattcatcattccaacaatgaagaagagcagacaaatctatataaaaacccaaccacctatatttcagttgggacatcaacattaaaccagagattggtaccaaaaattgctactagatcccaagtaaatgggcagcggggaagaatggacttcttctggacaattttaaaaccaaatgatgcaatccacttcgagagtaatggaaatttcattgctccagaatatgcatacaaaattgtcaagaaaggggactcaacaattatgaaaagtgaagtggaatatggccactgcaacaccaaatgtcaaaccccagtaggagcgataaactctagtatgccattccacaatatacatcctctcaccatcggggaatgccccaaatacgtgaagtcaaacaagttggtccttgcaactgggctcagaaatagtcctctaagagaaGGGaagagaagaaaaagagggctgtttggggcgatagcaggtttcatagagggaggatggcagggaatggttgatggttggtatgggtaccaccatagcaatgagcagggaagtgggtacgctgcagacaaggaatccacccaaaaggcaatagatggagttaccaataaggtcaactcgatcattaacaaaatgaacactcaatttgaggcagttggaagggagtttaataacttagaaaggaggatagagaatttgaacaagaaaatggaagacgggttcctagatgtctggacctataatgctgaacttttagttctcatggaaaacgagaggactctagatttccatgactcaaatgtcaagaacctttacgacaaagtcagactgcagcttagggataatgcaaaggagctgggtaacggttgtttcgagttctatcacaaatgtgataatgaatgtatggaaagtgtgagaaatgggacgtattactaccctcagtattcagaagaagcaagattaaaaagagaagaaataagcggagtgaaattagaatcaataggaacttaccaaatactgtcaatttattcaacagtggcgagttccctagcactggcaatcatggtggctggtctatctttatggatgtgttccaatgggtcgttacagtgcagaatttgcatt

>H5N8_A_Goose_Hungary_19953_2020

atggagaacatagtgcttcttcttgcaatagttagccttgttaaaagtgatcaaatttgcattggttaccatgcaaacaactcaacagaacaagtcgacacgataatggaaaagaacgtcactgttacacatgcccaagacatactggaaaaagcacacaacgggaagctctgcgatttaaatggggtgaagcctctggttttaaatgattgtagtgtagctggatggctcctcggaaacccaatgtgcgacgaattcatcagagtgccggaatggtcttacatagtagagaggactaacccagctaatgacctctgttacccagggagcctcaatgactatgaagaactaaaacacctgttgagcagaataaatcattttgagaagattctgatcatccccaagagttcttggcccaatcatgaaacatcattaggggtgagcgcagcttgtccataccagggaacgccctcctttttcagaaatgtggtatggcttatcaaaaagaatgatgcatacccaacaataaagataaactacaataataccaatcgggaagatctcttgatactgtgggggattcatcattccaacaatgaagaagagcagacaaatctatataaaaacccaaccacctatatttcagttgggacatcaacattaaaccagagattggtaccaaaaatagctactagatcccaagtaaatgggcagcggggaagaatggacttcttctggacaattttaaaaccaaatgatgtaatccacttcgagagtaatggaaatttcattgctccagaatatgcatacaaaattgtcaagaaaggggactcaacaattatgaaaagtgaagtggaatatggccactgcaacaccaaatgtcaaaccccagtaggagcgataaactctagtatgccattccacaatatacatcctctcaccatcggggaatgccccaaatacgtgaagtcaaacaagttggtccttgcaactgggctcagaaatagtcctctaagagaaGGGaagagaagaaaaagagggctgtttggggcgatagcaggtttcatagagggaggatggcagggaatggttgatggttggtatgggtaccaccatagcaatgagcagggaagtgggtacgctgcagacaaggaatccacccaaaaggcaatagatggagttaccaataaggtcaactcgatcattaacaaaatgaacactcaatttgaggcagttggaagggagtttaataacttagaaaggaggatagagaatttgaacaagaaaatggaagacgggttcctagatgtctggacctataatgctgaacttttagttctcatggaaaacgagaggactctagatttccatgactcaaatgtcaagaacctttacgacaaagtcagactgcagcttagggataatgcaaaggagctgggtaacggttgtttcgagttctatcacaaatgtgataatgaatgtatggaaagtgtgagaaatgggacgtattactaccctcagtattcagaagaagcaagattaaaaagagaagaaataagcggagtgaaattagaatcaataggaacttaccaaatactgtcaatttattcaacagtggcgagttccctagcactggcaatcatggtggctggtctatctttatggatgtgttccaatgggtcgttacagtgcagaatttgcatt

>H5N8_A_chicken_Cameroon_17RS1661_1_2017

atggagaacatagtgcttcttcttgcaatagttagccttgttaaaagtgatcagatttgcattggttaccatgcaaacaactcgacagagcaagttgacacgataatggaaaagaacgtcactgttacacatgcccaagacatactggaaaaaacacacaacgggaagctctgcgatctaaatggggtgaagcctctgattttaaaggattgtagtgtagctggatggctcctcggaaacccaatgtgcgacgaattcatcagagtgccggaatggtcttacatagtggagagagctgacccagctaatgacctctgttacccagggagcctcaatgactatgaagaactgaaacacctgttgagcagaataaatcattttgagaagattctgatcatccccaagagttcttggcccaatcatgaaacatcattaggggtgagcgcagcttgtccataccagggaacgccctcctttttcagaaatgtggtatggcttatcaaaaagaacgatgcatacccaacaataaagataagctacaataataccaatcgggaagatctcttgatactgtgggggattcatcattccaacaatgcagaagagcagacaaatctctataaaaacccaaccacctatatttcagttggaacatcaacattaaaccagagattggtaccaaaaatagctactagatcccaagtaaacgggcaacggggaagaatggacttcttctggacaattttaaaaccgaatgatgcaatccacttcgagagtaatggaaatttcattgctccagaatatgcatacaaaattgtcaagaaaggggactcaacaattatgaaaagtgaagtggaatatggccactgcaacaccaaatgtcaaaccccagtaggagcgataaactctagtatgccattccacaatatacatcccctcaccatcggggaatgccccaaatacgtgaagtcaaacaagttggtccttgcgactgggctcagaaatagtcctctaagagaaGGGaagagaagaaaaagagggctgtttggggctatagcaggttttatagagggaggatggcagggaatggttgatggttggtatgggtaccaccatagcaatgagcagggaagtgggtacgctgcagacaaagaatccacccaaaaggcaatagatggagttaccaataaggtcaactcgatcattgacaaaatgaacactcaatttgaggcagttggaagggagtttaataacttagaaaggaggatagagaatttgaacaagaaaatggaagacggattcctagatgtctggacctataatgctgaacttctagttctcatggaaaatgagaggactctagatttccatgactcaaatgtcaagaacctttacgacaaagtcagactgcagcttagggataatgcaaaggagctgggtaacggttgtttcgagttctatcacaaatgtgataatgaatgtatggaaagtgtgagaaatgggacgtatgactaccctcagtattcagaagaagcaagattaaaaagagaagaaataagcggagtgaaattagaatcaataggaacttaccaaatactgtcaatttattcaacagtggcgagttccctagcactggcaatcatggtggctggtctatctttatggatgtgctccaatgggtcgttacagtgcagaatttgcatt

>H5N8_A_green_winged_teal_Egypt_877_2016

atggagaacatagtacttcttcttgcaatagttagccttgttaaaagtgatcagatttgcattggttaccatgcaaacaactcgacagagcaagttgacacgataatggaaaagaacgtcactgttacacatgcccaagacatactggaaaagacacacaacgggaagctctgcgatctaaatggggtgaagcctctgattttaaaggattgtagtgtagctggatggctcctcggaaacccaatgtgcgacgaattcatcagagtgccggaatggtcttacatagtggagagggctaacccagctaatgacctctgttacccagggagcctcaatgactatgaagaactgaaacacctgttgagcagaataaatcattttgagaagattctgatcatccccaagagttcttggcccaatcatgaaacatcattaggggtgagcgcagcttgtccataccagggaacgccctcctttttcagaaatgtggtatggcttatcaaaaagaacgatgcatacccaacaataaagataagctacaataataccaatcgggaagatctcttgatactgtgggggattcatcattccaacaatgcagaagagcagacaaatctctataaaaacccaaccacctatatttcagttggaacatcaacattaaaccagagattggtaccaaaaatagctactagatcccaagtaaacgggcaacggggaagaatggacttcttctggacaattttaaaaccgaatgatgcaatccacttcgagagtaatggaaatttcattgctccagaatatgcatacaaaattgtcaagaaaggggactcaacaattatgaaaagtgaagtggaatatggccactgcaacaccaaatgccaaaccccagtaggagcaataaactctagtatgccattccacaatatacatcctctcaccatcggggaatgccccaaatacgtgaagtcaaacaagttggtccttgcgactgggctcagaaatagtcctctaagagaaGGGaagagaagaaaaagagggctgtttggggctatagcaggttttatagagggaggatggcagggaatggttgatggttggtatgggtaccaccatagcaatgagcagggaagtgggtacgctgcagacaaagaatccacccaaaaggcaatagatggagttaccaataaggtcaactcgatcattgacaaaatgaacactcaatttgaggcagttggaagggagtttaataacttagaaaggaggatagagaatttgaacaagaaaatggaagacggcttcctagatgtctggacctataatgctgaacttctagttctcatggaaaacgagaggactctagatttccatgactcaaatgtcaagaacctatacgacaaagtcagactgcagcttagggataatgcaaaggagctgggtaacggttgtttcgagttctatcacaaatgtgataatgaatgtatggaaagtgtgagaaatgggacgtatgactaccctcagtattcagaagaagcaagattaaaaagagaagaaataagcggagtgaaattagaatcaataggaacttaccaaatactgtcaatttattcaacagtggcgagttccctagcactggcaatcatggtggctggtctatctttatggatgtgctccaatgggtcgttacagtgcagaatttgcatt

>H5N8_A_grey_heron_W779_2017

atggagaacatagtgcttcttcttgcaatagttagccttgttaaaggtgatcagatttgcattggttaccatgcaaacaactcgacagagcaagttgacacgataatggaaaagaacgtcactgttacacatgcccaagacatactggaaaaaacacacaacgggaagctctgcgatctaaatggggtgaagcctctgattttaaaggattgtagtgtagctggatggctcctcggaaacccaatgtgcgacgaattcatcagagtgccggaatggtcttacatagtggagagggctaacccagctaatgacctctgttacccagggagcctcaatgactatgaagaactgaaacacctgttgagcagaataaatcattttgagaagattctgatcatccccaagagttcttggcccaatcatgaaacatcattaggggtgagcgcagcttgtccataccagggaacgccctcctttttcagaaatgtggtatggcttatcaaaaagaacgatgcatacccaacaataaagataagctacaataataccaatcgggaagatctcttgatactgtgggggattcatcattccaacaatgcagaagagcagacaaatctctataagaacccaaccacctatatttcagttggaacatcaacattaaaccagagattggtaccaaaaatagctactagatcccaagtaaacgggcaacggggaagaatggacttcttctggacaattttaaaaccgaatgatgcaatccactttgagagtaatggaaatttcattgctccagaatatgcatacaaaattgtcaagaaaggggactcaacaattatgaaaagtgaagtggaatatggccactgcaacaccaaatgtcaaaccccaataggagcgataaactctagtatgccattccacaatatacatcctctcaccatcggggaatgccccaaatatgtgaagtcaaacaagttggtccttgcgactgggctcagaaatagtcctctaagagaaGGGaagagaagaaaaagagggctgtttggggctatagcaggttttatagagggaggatggcagggaatggttgatggttggtatgggtaccaccatagcaatgagcagggaagtgggtacgctgcagacaaagaatccacccaaaaggcaatagatggagttaccaataaggtcaactcgatcattgacaaaatgaacactcaatttgaggcagttggaagggagtttaataacttagaaaggaggatagagaatttgaacaagaaaatggaagacggattcctagatgtctggacctataatgctgaacttctagttctcatggaaaacgagaggactctagatttccatgactcaaatgtcaagaacctttacgacaaagtcagactgcagcttagggataatgcaaaggagctgggtaacggttgtttcgagttctatcacaaatgtgataatgaatgtatggaaagtgtgagaaatgggacgtatgactaccctcagtattcagaagaagcaagattaaaaagagaagaaataagcggagtgaaattagaatcaataggaacttaccaaatactgtcaatttattcaacagtggcgagttccctagcactggcaatcatggtggctggtctatctttatggatgtgctccaatgggtcgttacagtgcagaatttgcatt

>H5N8_A_common_teal_Korea_W547_2016

atggagaaaatagtgcttcttcttgcaatagttagccttgttaaaggtgatcagatttgcattggttaccatgcaaacaactcgacagagcaagttgacacgataatggaaaagaacgtcactgttacacatgcccaagacatactggaaaaaacacacaacgggaagctctgcgatctaaatggggtgaagcctctgattttaaaggattgtagtgtagctggatggctcctcggaaacccaatgtgcgacgaattcatcagagtgccggaatggtcttacatagtggagagggctaacccagctaatgacctctgttacccagggagcctcaatgactatgaagaactgaaacacctgttgagcagaataaatcattttgagaagattctgatcatccccaagagttcttggcccaatcatgaaacatcattaggggtgagcgcagcttgtccataccagggaacgccctcctttttcagaaatgtggtatggcttatcaaaaagaacgatgcatacccaacaataaagataagctacaataataccaatcgggaagatctcttgatactgtgggggattcatcattccaacaatgcagaagagcagacaaatctctataagaacccaaccacctatatttcagttggaacatcaacattaaaccagagattggtaccaaaaatagctactagatcccaagtaaacgggcaacggggaagaatggacttcttctggacaattttaaaaccgaatgatgcaatccacttcgagagtaatggaaatttcattgctccagaatatgcatacaaaattgtcaagaaaggggactcaacaattatgaaaagtgaagtggaatatggccactgcaacaccaaatgtcaaaccccagtaggagcgataaactctagtatgccattccacaatatacatcctctcaccatcggggaatgccccaaatatgtgaagtcaaacaagttggtccttgcgactgggctcagaaatagtcctctaagagaaGGGaagagaagaaaaagagggctgtttggggctatagcaggttttatagagggaggatggcagggaatggttgatggttggtatgggtaccaccatagcaatgagcagggaagtgggtacgctgcagacaaagaatccacccaaaaggcaatagatggagttaccaataaggtcaactcgatcattgacaaaatgaacactcaatttgaggcagttggaagggagtttaataacttagaaaggaggatagagaatttgaacaagaaaatggaagacggattcctagatgtctggacctataatgctgaacttctagttctcatggaaaacgagaggactctagatttccatgactcaaatgtcaagaacctttacgacaaagtcagactgcagcttagggataatgcaaaggagctgggtaacggttgtttcgagttctatcacaaatgtgataatgaatgtatggaaagtgtgagaaatgggacgtatgactaccctcagtattcagaagaagcaagattaaaaagagaagaaataagcggagtgaaattagaatcaataggaacttaccaaatactgtcaatttattcaacagtggcgagttccctagcactggcaatcatggtggctggtctatctttatggatgtgctccaatgggtcgttacagtgcagaatttgcatt

>H5N8_A_grey_headed_gull_Uganda_MUWRP_538_2017

atggagaacatagtgcttcttcttgcaatagttagccttgttaaaagtgatcagatttgcattggttaccatgcaaacaactcgacagagcaagttgacacgataatggaaaagaacgtcactgttacacatgcccaagacatactggaaaaaacacacaacgggaagctctgcgatctaaatggggtgaaacctctgattttaaaggattgtagtgtagctggatggctcctcggaaacccaatgtgcgacgaattcatcagagtgccggaatggtcttacatagtggagagggctaacccagctaatgacctctgttacccagggagcctcaatgactatgaagaactgaaacacctgttgagcagaataaatcattttgagaagattctgatcatccccaagagttcttggcccaatcatgaaacatcattaggggtgagcgcagcttgtccataccagggaacaccctcctttttcagaaatgtggtatggcttatcaaaaagaacgatgcatacccaacaataaagataagctacaataataccaatcgggaagatctcttgatactgtgggggattcatcattccaacaatgcagaagagcagacaaatctctataaaaacccaaccacctatatctcagttggaacatcaacattaaaccagagattggtaccaaaaatagctactagatcccaagtaaacgggcaacgtggaagaatggacttcttctggacaattttaaaaccgaatgatgcaatccatttcgagagtaatggaaatttcattgctccagaatatgcatacaaaattgtcaagaaaggggactcaacaattatgaaaagtgaagtggaatatggccactgcaacaccagatgtcaaaccccagtaggagcgataaactctagtatgccattccacaatatacatcctctcaccatcggggaatgccccaaatacgtgaagtcaaacaagttggtccttgcgactgggctcagaaatagtcctctaagagaaGGGaagagaaggaaaagagggctgtttggggctatagcaggttttatagagggaggatggcagggaatggttgatggttggtatgggtaccaccatagcaatgagcaggggagtgggtacgctgcggacaaagaatccacccaaaaggcaatagatggagttaccaataaggtcaactcgatcattgacaaaatgaacactcaatttgaggcagttgtaagggagtttaataacttagaaaggaggatagagaatttgaacaagaaaatggaagacggattcctagatgtctggacctataatgctgaacttctagttctcatggaaaacgagaggactctagatttccatgactcaaatgtcaagaacctttacgacaaagtcagactgcagcttagggataatgcaaaggagctgggtaacggttgtttcgagttctatcacaaatgtgataatgaatgtatggaaagtgtgagaaatgggacgtatgactaccctcagtattcagaagaagcaagattaaaaagagaagaaataagcggagtgaaattagaatcaataggaacttaccaaatactgtcaatttattcaacagtggcgagttccctagcactggcaatcatggtggctggtctatctttatggatgtgctccaatgggtcgttacagtgcagaatttgcatt

>H5N8_A_duck_Cameroon_17RS1661_3_2017

atggagaacatagtgcttcttcttgcaatagttagccttgttaaaagtgatcagatttgcattggttaccatgcaaacaactcgacagagcaagttgacacgataatggaaaagaacgtcactgttacacatgcccaagacatactggaaaaaacacacaacgggaagctctgcgatctaaatggggtgaaacctctgattttaaaggattgtagtgtagctggatggctcctcggaaacccaatgtgcgacgaattcaccagagtgccggaatggtcttacatagtggaaagggctaacccagctaatgacctctgttacccagggagcctcaatgactatgaagaactgaaacacctgttgagcagaataaatcattttgagaagattctgatcatccccaagagttcttggcccaatcatgaaacatcattaggggtgagcgcagcttgtccataccagggaacgccctcctttttcagaaatgtggtatggcttatcaaaaagaacgatgcatacccaacaataaagataagctacaataataccaatcgggaagatctcttgatactgtgggggattcatcattccaacaatgcagaagagcagacaaatctctataaaaacccaaccacctatatttcagttggaacatcaacattaaaccagagattggtaccaaaaatagctactagatcccaagtaaacgggcaacgcggaagaatggacttcttctggacaattttaaaaccgaatgatgcaatccatttcgagagtaatggaaatttcattgctccagaatatgcatacaaaattgtcaagaaaggggactcaacaattatgaaaagtgaagtggaatatggccactgcaacaccagatgtcaaaccccagtaggagcgataaactctagtatgccattccacaatatacatcctctcaccatcggggaatgccccaaatacgtgaagtcaaacaagttggtccttgcgactgggctcagaaatagtcctctaagagaaGGGaagagaaggaaaagagggctgtttggggctatagcaggttttatagagggaggatggcagggaatggttgatggttggtatgggtaccaccatagcaatgagcaggggagtgggtacgctgcagacaaagaatccacccaaaaggcaatagatggagttaccaataaggtcaactcgatcattgacaaaatgaacactcaatttgaggcagttggaagggagtttaataacttagaaaggaggatagagaatttgaacaagaaaatggaagacggattcctagatgtctggacctataatgctgaacttctagttctcatggaaaacgagaggactctagatttccatgactcaaatgtcaagaatctttacgacaaagtcagactgcagcttagggataatgcaaaggagctgggtaacggttgtttcgagttctatcacaaatgtgataatgaatgtatggaaagtgtgagaaatgggacgtatgactaccctcagtattcagaagaggcaagattaaaaagagaagaaataagcggagtgaaattagaatcaataggaacttaccaaatactgtcaatttattcaacagtggcgagttccctagcactggcaatcatggtggctggtctatctttatggatgtgctccaatgggtcgttacagtgcagaatttgcatt

>H5N8_A_duck_Democratic_Republic_of_the_Congo_17RS882_29_2017

atggagaacatagtgcttcttcttgcaatagttagccttgttaaaagtgatcagatttgcattggttaccatgcaaacaactcgacagagcaagttgacacgataatggaaaagaacgtcactgttacacatgcccaagacatactggaaaaaacacacaacgggaagctctgcgatctaaatggggtgaaacctctgattttaaaggattgtagtgtagctggatggctcctcggaaacccaatgtgcgacgaattcatcagagtgccggaatggtcttacatagtggagagggctaacccagctaatgacctctgttacccagggagcctcaatgactatgaagaactgaaacacctgttgagcagaataaatcattttgagaagattctgatcatccccaagagttcttggcccaatcatgaaacatcattaggggtgagcgcagcttgtccataccagggaacgccctcctttttcagaaatgtggtatggcttatcaaaaagaatgatgcatacccaacaataaagataagctacaataataccaatcgggaagatctcttgatactgtgggggattcatcattccaacaatgcagaagagcagacaaatctctataaaaacccaaccacctatatttcagttggaacatcaacattaaaccagagattggtaccaaaaatagctactagatcccaagtaaacgggcaacgtggaagaatggacttcttctggacaattttaaaaccgaatgatgcaatccatttcgagagtaatgggaatttcattgctccagaatatgcatacaaaattgtcaagaaaggggactcaacaattatgaaaagtgaagtggaatatggccactgcaacaccagatgtcaaaccccagtaggagcgataaactctagtatgccattccacaatatacatcctctcaccatcggggaatgccccaaatacgtgaagtcaaacaagttggtccttgcgactgggctcagaaatagtcctctaagagaaGGGaagagaaggaaaagagggctgtttggggctatagcaggttttatagagggaggatggcagggaatggttgatggttggtatgggtaccaccatagcaatgagcaggggagtgggtacgctgcagacaaagaatccacccaaaaggcaatagatggagttaccaataaggtcaactcgatcattgacaaaatgaacactcaatttgaggcagttggaagggagtttaataatttagagaggaggatagagaatttgaacaagaaaatggaagacggattcctagatgtctggacctataatgctgaacttctagttctcatggaaaacgagaggactctagatttccatgactcaaatgtcaagaacctttacgacaaagtcagactgcagcttagggataatgcaaaggagctgggtaacggttgtttcgagttctatcacaaatgtgataatgaatgtatggaaagtgtgagaaatgggacgtatgactaccctcagtattcagaagaagcaagattaaaaagagaagaaataagcggagtgaaattagaatcaataggaacttaccaaatactgtcaatttattcaacagtggcgagttccctaacactggcaatcatggtggctggtctatctttatggatgtgctccaatgggtcgttacagtgcagaatttgcatt

>H5N8_A_Cygnus_atratus_Hubei_HF_1_2016

atggagaaaatagtgcttcttcttgcaatagttagccttgttaaaagtgatcagatttgcattggttaccatgcaaacaactcgacagagcaagttgacacgataatggaaaagaacgtcactgttacacatgcccaagacatactggaaaaaacacacaacgggaagctctgcgatctaaatggggtgaaacccctgattttaaaggattgtagtgtagctggatggctcctcggaaacccaatgtgcgacgaattcatcagagtgccggaatggtcttacatagtggagagggctaacccagttaatgacctctgttacccagggagcctcaatgactatgaagaactgaaacacctgttgagcagaataaatcattttgagaagattctgatcatccccaagagttcttggcccaatcatgaaacatcattaggggtgagcgcagcttgtccataccagggaacgccctcctttttcagaaatgtggtatggcttatcaaaaagaacgatgcatacccaacaataaagataagctacaataataccaatcgggaagatctcttgatactgtgggggattcatcattccaacaatgcagaagagcagacaaatctctataaaaacccaaccacctatatttcagttggaacatcaacattaaaccagagattggtaccaaaaatagctactagatcccaagtaaacgggcaacgtggaagaatggacttcttctggacaattttaaaaccgaatgatgcaatccatttcgagagtaatggaaatttcattgctccagaatatgcatacaaaattgtcaagaaaggggactcaacaattatgaaaagtgaagtggaatatggccactgcaacaccaaatgtcaaaccccagtaggagcgataaactctagtatgccattccacaatatacatcctctcaccatcggggaatgccccaaatacgtgaagtcaaacaagttggtccttgcgactgggctcagaaatagtcctctaagagaaGGGaagagaagaaaaagagggctgtttggggctatagcaggttttatagagggaggatggcagggaatggttgacggttggtatgggtaccaccatagcaatgagcaggggagtgggtacgctgcagacaaagaatccacccaaaaggcaatagatggagttaccaataaggtcaactcgatcattgacaaaatgaacactcaatttgaggcagttggaagggagtttaataacttagaaaggaggatagagaatttgaacaagaaaatggaagacggattcctagatgtctggacctataatgctgaacttctagttctcatggaaaacgagaggactctagatttccatgactcaaatgtcaagaacctttacgacaaagtcagactgcagcttagggataatgcaaaggagctgggtaacggttgtttcgagttctatcacaaatgtgataatgaatgtatggaaagtgtgaaaaatgggacgtatgactaccctcagtattcagaagaagcaagattaaaaagagaagaaataagcggagtgaaattagaatcaataggaacttaccaaatactgtcaatttattcaacagtggcgagttccctagcactggcaatcatggtggctggtctatctttatggatgtgctccaatgggtcgttacagtgcagaatttgcatt

>H5N6_2344b_A_Fujian_Sanyuan_21099_2017

atggaggacatagtgcttcttcttgcaatagttagccttgttaaaagtgatcagatttgcattggttaccatgcaaacaactcgacagagcaagttgacacgataatggaaaagaacgtcactgttacacatgcccaagacatactggaaaaaacacacaatgggaagctctgcgatctaaatggggtgaaacccctgattttaaaggattgtagtgtagctggatggctcctcggaaacccaatgtgcgacgaattcatcagagtgccggaatggtcttacatagtggagagggctaacccagctaatgacctctgttacccagggagcctcaatgactatgaagaactgaaacacctgttgagcagaataaatcattttgagaagattctgatcatccccaagagttcttggcccaatcatgaaacatcattaggggtgagtgcagcttgtccataccaggggacgccctcctttttcagaaatgtggtatggcttatcaaaaagaacgatgcatacccaacaataaagataagctacaataataccaatcgggaagatctcttgatactgtgggggattcatcattccaacaatgcagaagagcagacaaatctctataaaaacccaaccacctatatttcagttggaacatcaacattaaaccagagattggtaccaaaaatagctactagatcccaagtaaacgggcaacgtggaagaatggacttcttctggacaattttaaaaccgaatgatgcaatccatttcgagagtaatggaaatttcattgctccagaatatgcatacaaaattgtcaagaaaggggactcaacaattatgaaaagtgaagtggaatatggccactgcaacaccaaatgtcaaaccccagtaggagcgataaactctagtatgccattccacaatatacatcctctcaccatcggggaatgccccaaatacgtgaagtcaaacaagttggtccttgcgactgggctcagaaatagtcctctaagagaaGGGaagagaagaaaaagagggctgtttggggctatagcaggttttatagagggaggatggcagggaatggttgatggttggtatgggtaccaccatagcaatgagcaggggagtgggtacgctgcagacaaagaatccacccaaaaggcaatagatggagttaccaataaggtcaactcgatcattgacaaaatgaacactcaatttgaggcagttggaagggagtttaataacttagaaaggaggatagagaatttgaacaagaaaatggaagacggattcctagatgtctggacctataatgctgaacttctagttctcatggaaaacgagaggactctagatttccatgactcaaatgtcaagaacctttacgacaaagtcagactgcagcttagggataatgcaaaggagctgggtaacggttgtttcgagttctatcacaaatgtgataatgaatgtatggaaagtgtgaaaaatgggacgtatgactaccctcagtattcagaagaagcaagattaaaaagagaagaaataagcggagtgaaattagaatcaataggaacttaccaaatactgtcaatttattcaacagtggcgagttccctagcactggcaatcatggtggctggtctatctttatggatgtgctccaatgggtcgttacagtgcagaatttgcatt

>H5N8_A_Broiler_chicken_Giza_VRLCU_2019

atggagaacatagtgcttcttcttgcaatagttagccttgttaaaagtgatcagatttgcattggttaccatgcaaacaactcgacagagcaagttgacacgataatggaaaagaacgtcactgttacacatgcccaagacatactggaaaaaacacacaacgggaagctctgcgatctaaatggggtgaaacctctgattttaaaggattgtagtgtagctggatggctcctcggaaacccaatgtgcgacgaattcatcagagtgccggaatggtcttacatagtggagagggctaacccagctaatgacctctgttacccagggagcctcaatgactatgaagaactgaaacacctgttgagcagaataaatcattttgagaaaattctgatcatccccaagagctcttggcccaatcatgaaacatcattaggggtgagcgcagcttgtccataccagggaacgccctcctttttcagaaatgtggtatggcttatcaaaaagaacgatgcatatccaacaataaagataagctacaataataccaatcgggaagatctcttgatactgtgggggattcatcattccaacaatgcagaagagcagacaaatctctataaaaacccaaccacctatatttcagttggaacatcaacattaaaccagagattggtaccaaaaatagctactagatcccaagtaaacgggcaacgtggaagaatggacttcttttggacaattttaaaaccgaatgatgcaatccatttcgagagtaatggaaatttcattgctccagaatatgcatacaaaattgtcaagaaaggggactcaacaattatgaaaagtgaagtggaatatggccactgcaacaccaaatgtcaaaccccagtaggagcgataaactctagtatgccattccacaatatacatcctctcaccatcggggaatgccccaaatacgtgaagtcaaacaagttggtccttgcgactgggctcagaaatagtcctctaagagaaGGGaagagaagaaaaagagggctgtttggggctatagcaggttttatagagggaggatggcagggaatggttgatggttggtatgggtaccaccatagcaatgagcaggggagtgggtacgccgcagacaaagaatccacccaaaaggcaatagatggagttaccaataaggtcaactcgatcattgacaaaatgaacactcaatttgaggcagttggaagggagtttaataacttagaaaggaggatagaaaatttgaacaagaaaatggaagacggattcctagatgtctggacctataatgctgaacttctagttctcatggaaaacgagaggactctagatttccatgactcaaatgtcaagaacctttacgacaaagtcagactgcagcttagggataatgcaaaggagctgggtaacggttgtttcgagttctatcacaaatgtgataatgaatgtatggaaagtgtgagaaatgggacgtatgactaccctcagtattcagaagaagcaagattaaaaagagaagaaataagcggagtgaaattagaatcaataggaacttaccaaatactgtcaatttattcaacagtggcgagttccctagcactggcaatcatggtggctggtctatctttatggatgtgctccaatgggtcgttacagtgcagaatttgcatt

>H5N8_A_chicken_Egypt_Q13941B_2017

atggagaacatagtgcttcttcttgcaatagttagccttgttaaaagtgatcagatttgcattggttaccatgcaaacaactcgacagagcaagttgacacgataatggaaaagaacgtcactgttacacatgcccaagacatactggaaaaaacacacaacgggaagctctgcgatctaaatggggtgaaacctctgattttaaaggattgtagtgtagctggatggctcctcggaaacccaatgtgcgacgaattcatcagagtgccggaatggtcctacatagtggagagggctaacccagctaatgacctctgttacccagggagcctcaatgattatgaagaactgaagcacctgttgagcagaataaatcattttgagaaaattctgatcatccccaagagctcttggcccaatcatgaaacatcattaggggtgagcgcagcttgtccataccagggaacgccttcctttttcagaaatgtggtatggcttatcaaaaagaacgatgcatatccaacaataaagataagctacaataataccaatcgggaagatctcttgataatgtgggggattcatcattccaacaatgcagaagagcagacaaatctctataaaaacccaaccacctatatttcagttggaacatcaacattaaaccagagattggtaccaaaaatagctactagatcccaagtaaacgggcaacgtggaagaatggacttcttctggacaattttaaaaccgaatgatgcaatccatttcgagagtaatggaaatttcattgctccagaatatgcatacaaaattgtcaagaaaggggactcaacaattatgaaaagtgaagtggaatatggccactgcaacaccaaatgtcaaaccccagtaggagcgataaactctagtatgccattccacaatatacatcctctcaccatcggggaatgccccaaatacgtgaagtcaaacaagttggtccttgcgactgggctcagaaatagtcctctaagagaaGGGaagagaagaaaaagagggctgtttggggctatagcaggttttatagagggaggatggcagggaatggttgatggttggtatgggtaccaccatagcaatgagcaggggagtgggtacgccgcagacaaagaatccacccaaaaggcaatagatggagttaccaataaggtcaactcgatcattgacaaaatgaacactcaatttgaggcagttggaagggagtttaataacttagaaaggaggatagagaatttgaacaagaaaatggaagacggattcctagatgtctggacctataatgctgaacttctagttctcatggaaaacgagaggactctagatttccatgactcaaatgtcaagaacctttacgacaaagtcagactgcagcttagggataatgcaaaggagctgggtaacggttgtttcgagttctatcacaaatgtgataatgaatgtatggaaagtgtgagaaatgggacgtatgactaccctcagtattcagaagaagcaagattaaaaagagaagaaataagcggagtgaaattagaatcaataggaacttaccaaatactgtcaatttattcaacagtggcgagttccctagcactggcaatcatggtggctggtctgtctttatggatgtgctccaatgggtcgttacagtgcagaatttgcatt

>H5N8_A_goose_Zhejiang_925104_2014

atggagaaaatagtgcttcttcttgcaatagttagccttgttaaaagtgatcagatttgcattggttaccatgcaaacaactcgacagagcaggttgacacgataatggaaaagaacgtcactgttacacatgcccaagacatactggaaaagacacacaacgggaagctctgcgatctaaatggagtgaagcctctggttttaaaggattgtagtgtagctggatggctcctcggaaacccaatgtgcgacgaattcatcagggtgccggaatggtcttacatagtggagagggctaacccagccaatgacctctgttacccagggagcctcaatgactatgaagaactgaaacacctattgagcagaataaatcattttgagaagattctgatcatccccaagagttcttggcccaatcatgaaacatcattaggggtgagcgcagcttgtccataccagggaacgccctcctttttcagaaatgtggtatggcttatcaaaaagaacgatgcatacccaacaataaagataagctacaataataccaatcgggaagatcttttgatactgtgggggattcatcattccaacaatgcagcagagcagacaaatctctataaaaacccaaccacctatatttccgttgggacatcaacattaaaccagagattggtaccaaaaatagctactagatcccaagtaaacgggcaacgtggaagaatggacttcttctggacaattttaaaaccgaatgatgcaatccacttcgagagtaatggaaatttcattgctccagaatatgcatacaaaattgtcaagaaaggggactcaacaattatgaaaagtgaagtggaatatggccactgcaacaccaaatgtcaaactccagtaggagcgataaactctagtatgccattccacaatatacatcctctcaccatcggggaatgccccaaatacgtgaagtcaaacaaattggtccttgcgactgggctcagaaatagtcctctaagagaaGGGaagagaagaaaaagagggctgtttggggctatagcaggttttatagagggaggatggcagggaatggtagatggttggtatgggtaccaccatagcaatgagcaggggagtgggtacgctgcagacaaagaatccacccaaaaggcaatagatggagttaccaataaggtcaactcgatcattgacaaaatgaacactcaatttgaggccgttggaagggaatttaataacttagaaaggaggatagagaatttgaacaagaaaatggaagacggattcctagatgtctggacttataatgctgaacttctagttctcatggaaaatgagaggactctagatttccatgactcaaatgtcaagaacctttacgacaaagtccgactacagcttagggataatgcaaaggagctgggtaatggttgtttcgagttctatcacaaatgtgataatgaatgtatggaaagtgtaagaaatgggacgtatgactaccctcagtattcagaagaagcaagattaaaaagagaagaaataagcggagtgaaattagaatcaataggaacttaccaaatactgtcaatttattcaacagtggcgagttccctagcactggcaatcatggtggctggtctatctttatggatgtgctccaatgggtcgttacagtgcagaatttgcatt

>H5N8_A_duck_Zhejiang_6D18_2013

atggagaaaatagtgcttcttcttgcaatagttagccttgttaaaagtgatcagatttgcattggttaccatgcaaacaactcgacagagcaggttgacacgataatggaaaagaacgtcactgttacacatgcccaagacatactggaaaagacacacaacgggaagctctgcgatctaaatggggtgaagcctctgattttaaaggattgtagtgtagctggatggctcctcggaaacccaatgtgcgacgaattcatcagggtgccggaatggtcttacatagtagagagggctaacccagccaatgacctctgttacccagggagcctcaatgactatgaagaactgaaacacctattgagcagaataaatcattttgagaagattctgattatccccaagagttcttggcccgatcatgaaacatcattaggggtgagcgcagcatgtccataccagggaacgccctcctttttcagaaatgtgatatggcttatcaaaaagaacgatgcatacccaacaataaagataagctacaataataccaatcgggaagatattttgatactgtggggggttcatcattccaacaatgcagcagagcagacaaatctctataaaaacccaaccacctatgtttccgttgggacatcaacattaaaccagagattggtaccgaaaatagctactagatcccaagtaaacgggcaacgtggaagaatggatttcttctggacaattttaaaaccgaatgatgcaatccacttcgagagtaatggaaatttcattgctccagaatatgcatacaaaattgtcaagaaaggggactcaacaattatgaaaagtgaagtggaatatggccactgcaacaccaaatgtcaaactccagtaggggcgataaactctagtatgccattccacaacatacatcctctcaccatcggggaatgccccaaatacgtgaagtcaaacaaattagtccttgcgactgggctcagaaatagtcctctaagagaaGGGaagagaagaaaaagagggctgtttggggctatagcaggttttatagagggaggatggcagggaatggtagatggttggtatgggtaccaccatagcaatgagcaggggagtgggtacgctgcagacaaagaatccacccaaaaggcaatagatggagttaccaataaggtcaactcgatcattgacaaaatgaacactcaatttgaggccgttggaagggaatttaataacttagaaaggaggctagagaatttaaacaagaaaatggaagacggattcctagatgtctggacttataatgctgaacttctagttctcatggaaaatgagaggactctagatttccatgactcaaatgtcaagaacctttacgacaaagtccgactacagcttagggataatgcaaaggagctgggtaatggttgtttcgagttctatcacaaatgtgataatgaatgtatggaaagtgtaagaaatgggacgtatgactaccctcagtattcagaagaagcaagattaaaaagagaagaaataagcggagtgaaattagaatcaataggaacttaccaaatactgtcaatttattcaacagtggcgagttccctagcactggcaatcatggtggctggtctatctttatggatgtgctccaatgggtcgttacagtgcagaatttgcatt

>H5N8_A_duck_Eastern_China_JY_2014

atggagaaaatagtgcttcttcttgcaatagttagccttgttaaaagtgatcagatttgcattggttaccatgcaaacaactcgacagagcaggttgacacgataatggaaaagaacgtcactgttacacatgcccaagacatactggaaaagacacacaacgggaagctctgcgatctaaatggggtgaagcctctgattttaaaggattgtagtgtagctggatggctcctcggaaacccaatgtgcgatgaattcatcagggtgccggaatggtcttacatagtggagagggctaacccagccaatgacctctgttacccagggagcctcaatgactatgaagaactgaaacacctattgagcagaataaatcattttgagaagattctgatcatccccaagagttcttggcccgatcatgaaacatcattaggggtgagcgcagcatgtccataccagggaacgccctcctttttcagaaatgtggtatggcttatcaaaaagaacgatgcatacccaacaataaagataagctacaataataccaatcgggaagatcttttgatactgtggggggttcatcattccaacaatgcagcagagcagacaaatctctataaaaacccaaccacctatgtttccgttgggacatcaacattaaaccagagattggtaccaaaaatagctactagatcccaagtaaacgggcaacgtggaagaatggatttcttctggacaattttaaaaccgaatgatgcaatccacttcgagagtaatggaaatttcattgctccagaatatgcatacaaaattgtcaagaaaggggactcaacaattatgaaaagtgaggtggaatatggccactgcaacaccaaatgtcaaactccagtaggggcgataaactctagcatgccattccacaatatacatcctctcaccatcggggaatgccccaaatacgtgaagtcaaacaaattagtccttgcgactgggctcagaaatagtcctctaagagaaGGGaagagaagaaaaagagggctgtttggggctatagcaggttttatagagggaggatggcagggaatggtagatggttggtatgggtaccaccatagcaatgagcaggggagtgggtacgctgcagacaaagaatccacccaaaaggcaatagatggagttaccaataaggtcaactcgatcattgacaaaatgaacactcaatttgaggccgttggaagggaatttaataacttagaaaggaggctagagaatttaaacaagaaaatggaagacggattcctagatgtctggacttataacgctgaacttctagttctcatggaaaatgagaggactctagatttccatgactcaaatgtcaagaacctttacgacaaagtccgactacagctcagggataatgcaaaggagctgggtaatggttgtttcgagttttatcacaaatgtgataatgaatgtatggaaagtgtaagaaatgggacgtatgactaccctcagtattcagaagaagcaagattaaaaagagaagaaataagcggagtgaaattagaatcaataggaacttaccaaatactgtcaatttattcaacagtggcgagttccctagcactggcaatcatggtggctggtctatctttatggatgtgctccaatgggtcgttacagtgcagaatttgcatt

>H5N8_A_goose_Shandong_WFSG1_2014

atggagaaaatagtgcttcttcttgcactggttagccttgttaaaagtgatcagatttgcattggttaccatgcaaacaactcgacagagcaggttgacacgataatggaaaagaacgtcactgttacacatgcccaagacatactggaaaagacacacaacgggaagctctgcgatctaaatggggtgaagcctctgattttaaaggattgtagtgtagctggatggctcctcggaaacccaatgtgcgacgaattcatcagggtgccggaatggtcttacatagtggagagggctaacccagccaatgacctctgttacccagggagcctcaatgactatgaagaactgaaacacctattgagcagaataaatcattttgagaagattctgatcatccccaagagttcttggcccgatcatgaaacatcattaggggtgagcgcagcatgtccataccagggaacgccctcctttttcagaaatgtggtatggcttatcaaaaagaacgatgcatacccaacaataaagataagctacaataataccaatcgggaagatcttttgatactgtggggggttcatcattccaacaatgcagcagagcagacaaatctctataaaaacccaaccacctatgtttccgttgggacatcaacattaaaccagagattggtaccaaaaatagctactagatcccaagtaaacgggcaacgtggaagaatggatttcttctggacaattttaaaaccgaatgatgcaatccacttcgagagtaatggaaatttcattgctccagaatatgcatacaaaattgtcaagaaaggggactcaacaattatgaaaagtgaagtggaatatggccactgcaacaccaaatgtcaaactccagtaggggcgataaactctagtatgccattccacaatatacatcctctcaccatcggggaatgccccaaatacgtgaagtcaaacaaattagtccttgcgactgggctcagaaatagtcctctaagagaaGGGaagagaagaaaaagagggctgtttggggctatagcaggttttatagagggaggatggcagggaatggtagatggttggtatgggtaccaccatagcaatgagcaggggagtgggtacgctgcagacaaagaatccacccaaaaggcaatagatggagttaccaataaggtcaactcgatcattgacaaaatgaacactcaatttgaggccgttgggagggaatttaataacttagaaaggaggctagagaatttaaacaagaaaatggaagacggattcctagatgtctggacttataatgctgaacttctagttctcatggaaaatgagaggactctagatttccatgactcaaatgtcaagaacctttacgacaaagtccgactacagcttagggataatgcaaaggagctgggtaatggttgtttcgagttctatcacaaatgtgataatgaatgtatggaaagtgtaagaaatgggacgtatgactaccctcagtattcagaagaagcaagattaaaaagagaagaaataagcggagtgaaattagaatcaataggaacttaccaaatactgtcaatttattcaacagtggcgagttccctagcactggcaatcatggtggctggtctatctttatggatgtgcgccaatgggtcgttacagtgcagaatttgcatt

>H5N8_2344b_A_breeder_duck_Korea_Gochang1_2014

atggagaaaatagtgcttcttcttgcaatagttagccttgttaaaagtgatcagatttgcattggttaccatgcaaacaactcgacagagcaggttgacacgataatggaaaagaacgtcactgttacacatgcccaagacatactggaaaagacacacaacgggaagctctgcgatctaaatggggtgaagcctctgattttaaaggattgtagtgtagctggatggctcctcggaaacccaatgtgcgacgaattcatcagggtgccggaatggtcttacatagtggagagggctaacccagccaatgacctctgttacccagggagcctcaatgactatgaagaactgaaacacctattgagcagaataaatcattttgagaagattctgatcatccccaagagttcttggcccgatcatgaaacatcattaggggtgagcgcagcatgtccataccagggaacgccctcctttttcagaaatgtggtatggcttatcaaaaagaacgatgcatacccaacaataaagataagctacaataataccaatcgggaagatcttttgatactgtgggggattcatcattccaacaatgcagcagagcagacaaatctctataaaaacccaaccacctatgtttccgttgggacatcaacattaaaccagagattggtaccaaaaatagctactagatcccaagtaaacgggcaacgtggaagaatggatttcttctggacaattttaaaaccgaatgatgcaatccacttcgagagtaatggaaatttcattgctccagaatatgcatacaaaattgtcaagaaaggggactcaacaattatgaaaagtgaagtggaatatggccactgcaacaccaaatgtcaaactccagtaggggcgataaactctagtatgccattccacaatatacatcctctcaccatcggggaatgccccaaatacgtgaagtcaaacaaattggtccttgcgactgggctcagaaataatcctctaagagaaGGGaagagaagaaaaagagggctgtttggggctatagcaggttttatagagggaggatggcagggaatggtagatggttggtatgggtaccaccatagcaatgagcaggggagtgggtacgctgcagacaaagaatccacccaaaaggcaatagatggagttaccaataaggtcaactcgatcattgacaaaatgaacactcaatttgaggccgttggaagggaatttaataacttagaaaggaggctagagaatttaaacaagaaaatggaagacggattcctagatgtctggacttataatgctgaacttctagttctcatggaaaatgagaggactctagatttccatgactcaaatgtcaagaacctttacgacaaagtccgactacagcttagggataatgcaaaggagctgggtaatggttgtttcgagttctatcacaaatgtgataatgaatgtatggaaagtgtaagaaatgggacgtatgactaccctcagtattcagaagaagcaagattaaaaagagaagaaataagcggagtgaaattagaatcaataggaacttaccaaatactgtcaatttattcaacagtggcgagttccctagcactggcaatcatggtggctggtctatctttatggatgtgctccaatgggtcgttacagtgcagaatttgcatt

>H5N8_A_duck_Zhejiang_925019_2014

atggagaaaatagtgcttcttcttgcaatagttagccttgttaaaagtgatcagatttgcattggttatcatgcaaacaactcgacagagcaggttgacacgataatggaaaagaacgtcactgttacacatgcccaagacatactggaaaagacacacaacgggaagctctgcgatctaaatggggtgaagcctctggttttaaaggattgtagtgtagctggatggctcctcggaaacccaatgtgcgacgaattcatcagggtgccggaatggtcttacatagtggagagggctaacccagccaatgacctctgttacccagggagcctcaatgactatgaagaactgaaacacctattgagcagaataaatcattttgagaagattctgatcattcccaagagttcttggctcgatcatgaaacatcattaggggtgagcgcagcatgtccataccagggaacgccctcctttttcagaaatgtggtatggcttatcaaaaagaacgatgcatacccaacaataaagataagctacaataataccaatcgggaagatcttttgatactgtgggggattcatcattccaacaatgaagcagagcagacaaatctctataaaaacccaaccacctatgtttccgttgggacatcaacattaaaccagagattggtaccaaaaatagctactagatcccaagtaaacgggcaacgtggaagaatggatttcttctggacaattttaaaaccgaatgatgcaatccacttcgagagtaatggaaatttcattgctccagaatatgcatacaaaattgtcaagaaaggggactcaacaattatgaaaagtgaagtggaatatggccactgcaacaccaaatgtcaaactccagtaggggcgataaactctagtatgccattccacaatatacatcctctcaccatcggggaatgccccaaatacgtgaaatcaaacaaattggtccttgcgactgggctcaggaatagtcctctaagagaaGGGaggagaagaaaaagagggctgtttggggctatagcaggttttatagaaggaggatggcagggaatggtagatggttggtatgggtaccaccatagcaatgagcaggggagtgggtacgctgcagacaaagaatccacccaaaaggcaatagatggagttaccaataaggtcaactcgatcattgacaaaatgaacactcaatttgaggccgttggaagggaatttaataacttagaaaggaggatagagaatttaaacaagaaaatggaagacggattcctagatgtctggacttataatgctgaacttctagttctcatggaaaatgagaggactctagatttccatgactcaaatgtcaagaacctttacgacaaagtccgactacagcttagggataatgcaaaggagctgggtaatggttgtttcgagttctatcacaaatgtgataatgaatgtatggaaagtgtaaaaaatgggacgtatgactaccctcagtattcagaagaggcaagattaaaaagagaagaaataagcggagtgaaattagaatcaataggaacttaccaaatactgtcaatttattcaacaggggcgagttccctagcactggcaatcatgggtgctggtctatccttatggatgtgctccaatgggtcgttacagtgcagaatttgcatt

>H5N8_A_duck_Zhejiang_W24_2013

atggagaaaatagtgcttcttcttgcaataattagccttgttaaaagtgatcagatttgcattggttaccatgcaaacaactcgacagagcaggttgacacgataatggagaagaacgtcactgttacacatgcccaagacatactggaaaagacacacaacgggaagctctgcgatctaaatggggtgaagcctctggttttaaaggattgtagtgtagctggatggctcctcggaaacccaatgtgcgacgaattcatcagggtgccggaatggtcttacatagtggagagggctaacccagccaatgacctctgttacccagggagcctcaatgactatgaagaactgaaacacctattgagcagaataaatcattttgagaagattctgatcatccccaagagttcttggctcgatcatgaaacatcattaggggtgagcgcagcatgcccataccagggaacgccctcctttttcagaaatgtggtatggcttatcaaaaagaacgatgcatacccaacaataaagataagctacaataataccaatcgggaagatcttttgatactgtgggggattcatcattccaacaatgcagcagagcagacaaatctctataaaaacccaaccacctatgtttccgttgggacatcaacattaaaccagagattggtaccaaaaatagctactagatcccaagtaaacgggcaacgtggaagaatggatttcttctggacaattttaaaaccgaatgatgcaatccacttcgagagtaatggaaatttcattgctccagaatatgcatacaaaatagtcaagaaaggggactcaacaattatgaaaagtgaagtggaatatggccactgcaacaccaaatgtcaaactccagtaggggcgataaactctagtatgccattccacaatatacatcctctcaccatcggggaatgccccaaatacgtgaagtcaaacaaattggtccttgcgactgggctcagaaatagtcctctaagagaaGGGaagagaagaaaaagagggctgtttggggctatagcaggttttatagaaggaggatggcagggaatggtagatggttggtatgggtaccaccatagcaatgagcaggggagtgggtacgctgcagacaaagaatccacccaaaaggcaatagatggagttaccaataaggtcaactcgatcattgacaaaatgaacactcaatttgaggccgttggaagggaatttaataacttagaaaggaggatagagaatttaaacaagaaaatggaagacggattcctagatgtctggacttataatgctgaacttctagttctcatggaaaatgagaggactctagatttccatgactcaaatgtcaagaacctttacgacaaagtccgactacagcttagggataatgcaaaggagctgggtaatggttgtttcgagttctatcacaaatgtgataatgaatgtatggaaagtgtaaaaaatgggacgtatgactaccctcagtattcagaagaagcaagattaaaaagagaagaaataagcggagtgaaattagaatcaataggaacttaccaaatactgtcaatttattcaacagtggcgagttccctagcactggcaatcatggtggctggtctatctttatggatgtgctccaatgggtcgttacagtgcagaatttgcatt

>H5N6_A_duck_Wenzhou_YHQL22_2014

atggagaaaatagtgcttcttcttgcaatagttagccttgttaaaagtgatcagatttgcattggttaccatgcaaacaactcgacagagcaggttgacacgataatggaaaagaacgtcactgttacacatgcccaagacatactggaaaaaacacacaacgggaagctctgcgatctaaatggagtgaagcctctggttttaaaggattgtagtgtagctggatggctcctcggaaacccaatgtgcgacgagttcatcagggtgccggaatggtcttacatagtggagagggctaatccagccaatgacctctgttacccagggagcctcaatgactatgaagaactgaaacatctattgagcagaataaatcattttgagaagattctgatcatccccaagagttcttggcccgatcatgaaacatcattaggggtgagcgcagcatgtccataccagggaacgccctcctttttcagaaatgtggtatggcttatcaaaaagaacgatgcatacccaacaataaagataagctacaataataccaatcgggaagatcttttgatactgtgggggattcatcattctaacaatgcagcagagcagacaaatctctataaaaacccaaccacttatgtttccgttgggacatcaacattaaaccagagattggtaccaaaaatagctactagatcccaagtaaacgggcaacgtggaagaatggatttcttctggacaattttaaaaccgaatgatgcaatccacttcgagagtaatggaaatttcattgctccagaatatgcatacaaaattgtcaagaaaggggactcaacaattatgaaaagtgaagtggaatatggccactgcaacaccaaatgtcaaactccagtaggggcgataaactctagtatgccattccacaatatacatcctctcaccatcggagaatgccccaaatacgtgaagtcaaacaaattagtccttgcgactgggctcagaaataatcccctaagagaaGGGaagagaagaaaaagagggctgtttggggctatagcagggtttatagagggaggatggcagggaatggtagatggttggtatgggtaccaccatagcaatgagcaggggagtgggtacgctgcagacaaagaatccacccaaaaggcaatagatggagttaccaataaggtcaactcgatcattgacaaaatgaacactcaatttgaggctgttggaagggaatttaataacttagaaaggaggatagagaatttaaacaagaaaatggaagacggattcctagatgtctggacttataatgctgaacttctagttctcatggaaaatgagaggactctagatttccatgactcaaatgtcaagaacctttacgacaaagtccgactacagcttagagataatgcaaaagagctgggtaatggttgtttcgagttctatcacaaatgcgataatgaatgtatggaaagtgtaagaaatgggacgtatgactaccctcagtattcagaagaagcaagattaaaaagagaagaaataagcggagtgaaattagaatcaataggaacttaccaaatactgtcaatttattcaacagtggcgagttccctagcactggcaatcatggtggctggtctatctttatggatgtgctccaatgggtcgttacagtgcagaatttgcatt

>H5N8_A_duck_Eastern_China_L0611_2011

atggagaaaatagtgcttcttcttgcaatagtaagccttgttaaaagtgatcagatttgcattggttaccatgcaaacaactcgacagagcaggttgacacgataatggaaaagaacgtcactgttacacatgcccaagacatactggaaaagacacacaacgggaagctctgcgatctaaatggagtgaagcctctgattttaaaggattgtagtgtagctggatggctcctcggaaacccaatgtgcgacgagttcatcagggtgccggaatggtcttacatagtggagagggctaacccagccaatgacctctgttacccagggagcctcaatgactatgaagaactgaaacacctattgagcagaataaatcattttgagaaaattctgatcatccccaagagttcttggcccgatcatgaaacatcattaggggtgagcgcagcatgtccataccagggaacgccctcctttttcagaaatgtggtatggcttatcaaaaagaacgatgcatacccaacaataaagataagctacaacaataccaatcgggaagatcttttgatactgtgggggattcatcattccaacaatgcagcagagcagacaaatctctataaaaacccaaccacctatgtttccgttgggacatcaacattaaaccagagattggtaccaaaaatagctactagatcccaagtaaacgggcaacgtggaagaatggatttcttctggacaattttaaaaccgaatgatgcaatccacttcgagagtaatggaaatttcattgctccaaaatatgcatacaaaattgtcaagaaaggggactcaacaattatgaaaagtgaagtggaatatggccactgcaacaccaaatgtcaaactccaataggggcgataaactctagtatgccattccacaatatacaccctctcaccatcggggaatgccccaaatacgtgaagtcaaacaaattagtccttgcgactgggctcagaaatagtcctctaagagaaGGGaagagaagaaaaagaggactatttggggctatagcagggtttatagagggaggatggcagggaatggtagatggttggtatgggtaccaccatagcaatgagcaggggagtgggtacgctgcagacaaagaatccacccaaaaggcaatagatggagttaccaataaggtcaactcgatcattgacaaaatgaacactcaatttgaggccgttggaagggaatttaataacttagaaaggagaatagagaatttaaacaagaaaatggaagacggattcctagatgtctggacttataatgctgaacttctagttctcatggaaaatgagaggactctagatttccatgactcaaatgtcaagaacctttacgacaaagtccgactacagcttagggataatgcaaaggagctgggtaatggttgtttcgagttctatcacaaatgtgataatgaatgtatggaaagtgtaagaaatgggacgtatgactaccctcagtattcagaagaagcaagattaaaaagagaagaaataagcggagtgaaattagaatcaataggaacttaccaaatactgtcaatttattcaacagtggcgagttccctagcactggcaatcatggtggctggtctatctttatggatgtgctccaatgggtcgttacaatgcagaatttgcatt

>H5N8_A_duck_Shandong_Q1_2013

atggagaaaatagtgcttcttcttgcaatagttagccttgttaaaagtgatcagatttgcattggttaccatgcaaacaactcaacagagcaggttgacacgataatggaaaagaacgtcactgttacacatgcccaagacatactggaaaagacacacaacgggaagctctgcgatctaaatggagtgaagcctctgattttaaaggattgtagtgtagcaggatggctcctcggaaacccaatgtgcgacgagttcatcagggtgccggaatggtcttacatagtggagagagctaacccagccaatgacctctgttaccctgggagcctcaatgactatgaagaactgaaacacctattgagcagaataaatcattttgagaaaattctgatcatccccaagagttcttggcccgatcatgaaacatcattaggggtgagcgcagcatgtccataccagggaaagccctcctttttcagaaatgtagtatggcttatcaaaaggaacgatgcatacccaacaataaagataagctacaataataccaatcgggaagatcttttgatactgtgggggattcaccattccaacaatgcagcagagcagacaaatctctataaaaatccaaccacctatgtttccgttgggacatcaacattaaaccagagattggtaccaaaaatagctactagatcccaagtaaacgggcaacgtggaagaatggatttcttctggacaattttaaaaccgaatgatgcaatccacttcgagagtaatggaaatttcattgctccagaatatgcatacaaaattgtcaagaaaggggactcaacaattatgaaaagtgaaatggaatatggccactgcaacaccaaatgtcaaactccaataggggcgataaactctagtatgccattccacaatatacaccctctcaccatcggggaatgccccaaatacgtgaaatcaaacaaattagtccttgcgactgggctcaggaatagtcctctaagagaaGGGaagagaagaaaaagaggactatttggagctatagcaggatttatagagggaggatggcagggaatggtagatggttggtatgggtaccaccatagcaatgaacaggggagtgggtacgctgcagacaaagaatccacccaaaaggcaatagatggagtcaccaacaaggtcaactcgatcattgacaaaatgaacactcaatttgaggccgttggaagggaatttaataacttagaaaggagaatagagaatttaaacaagaaaatggaagacggattcctagatgtctggacttataatgctgaacttctagttctcatggaaaatgagagaactctagatttccatgattcaaatgtcaggaacctttacgacaaagtccgactacagcttagggataatgcaaaggagctgggtaatggttgtttcgagttctatcacaaatgtgacaatgaatgtatggaaagtgtaagaaatgggacgtatgactaccctcagtattcagaagaagcaagattaaaaagagaagaaataagcggagtgaaattagaatcaataggaacttaccaaatactgtcaatttattcaacagtggcgagttccctagcactggcaatcatggtggctggtctatctttatggatgtgctccaatgggtcgttacaatgcagaatttgcatt

>H5N8_A_goose_Guangdong_s13124_2013

atggagaaaatagtgcttcttcttgcaatagttagccttgttaaaagtgatcagatttgcattggttaccatgcaaacaactcgacagagcaggttgacacgataatggagaagaacgtcactgttacacatgcccaagacatactggaaaagacacataacgggaagctttgcgatctaaatggagtgaaacctctgattttaaaggattgtagtgtagcaggttggctcctaggaaacccaatgtgcgacgagttcatcagggtgccggaatggtcttacatagtggagagggctaacccagccaatgacctctgttacccagggagcctcaatgactatgaagaactgaaacatctattgagcagaataaatcattttgagaaaattctgatcatacccaagagttcttggcccaatcatgaaacatcattaggggtgagcgcagcatgtccataccagggaaagccctcctttttcagaaatgtggtatggcttatcaaaaagaacgatgcatacccaacaataaagataagctacaataataccaatcgggaagatcttttgatactgtgggggattcaccattccaacaatgcagcagagcagacaaatctctataaaaacccaaccacctatgtttccgttgggacatcaacattaaaccagagattggtaccaaaaatagctactagatcccaagtaaacgggcaacgtggaagaatggatttcttctggacaattttaaaaccgaatgatgcaatccacttcgagagtaatggaaatttcattgctccagaatatgcatacaaaattgtcaagaaaggggactcaacaattatgaaaagtgaagtggaatatggccactgcaacaccaaatgtcaaactccaataggggcgataaactctagtatgccattccacaatatacaccctctcaccatcggggaatgccccaaatacgtgaagtcaaacaaattagtccttgcgactgggctcaggaatagtcctctaagagagGGGaagagaagaaaaagaggactatttggagctatagcagggtttatagagggaggatggcagggaatggtagatggttggtatgggtaccaccatagcaatgagcaggggagtgggtacgctgcagacaaagaatccacccaaaaggcaatagatggagttaccaacaaggtcaactcgatcattgacaaaatgaacactcaatttgaggccgttggaagggaatttaataacttagaaaggagaatagagaatttaaacaagaaaatggaagacggattcctagatgtctggacttataatgctgaacttctagttctcatggaaaatgagagaactctagatttccatgattcaaatgtcaggaacctttacgacaaagtccgactacagcttaaggataatgcaaaggagctgggtaatggttgtttcgagttctatcacaaatgtgataatgaatgtatggaaagtgtaagaaatgggacgtatgactaccctcagtattcagaagaagcaagattaaaaagagaagaaataagcggagtgaaattagaatcaataggaacttaccaaatactgtcaatttattcaacagtggcgagttccctagcactggcaatcatggtggctggtctatctttatggatgtgctccaatgggtcgttacaatgcagaatttgcatt

>H5N8_A_duck_Eastern_China_L1120_2012

atggagaaaatagtgcttcttcttgcaatagttagccttgttaaaagtgatcagatttgcattggttaccatgcaaacaactcgacagagcaggttgacacgataatggaaaagaacgtcactgttacacatgcccaagacatactggaaaaaacacacaacgggaagctctgcgatctaaatggagtgaagcctctgattttaaaggattgtagtgtagcaggatggctcctcggaaacccaatgtgcgacgagttcatcagggtgccggaatggtcttacatagtggagagggctaacccagccaatgacctctgttacccagggagcctcaatgactatgaagaactgaaacacctattgagcagaataaatcattttgagaaaattctgatcatccccaagagttcttggcccgatcatgaaacatcattaggggtgagcgcagcatgtccataccagggaacgccctcctttttcagaaatgtggtatggcttatcaaaaagaacgatgcatacccaacaataaagataagctacaataataccaatcgggaagatcttttgatactgtgggggattcaccattccaacaatgcagcagagcagacaaatctctataaaaacccaaccacctatgtttccgttgggacatcaacattaaaccagagattggtaccaaaaatagctactagatcccaagtaaacgggcaacgtggaagaatggatttcttctggacaattttaaaaccgaatgatgcaatccacttcgagagtaatggaaatttcattgctccagaatatgcatacaaaattgtcaagaaaggggactcaacaattatgaaaagtgaagtggaatatggccactgcaacaccaaatgtcaaactccaataggggcgataaactctagtatgccattccacaatttacaccctctcaccatcggggaatgccccaaatacgtgaaatcaaacaaattagtccttgcgactgggctcaggaatagtcctctaagagagGGGaagagaagaaaaagaggactatttggagctatagcagggtttatagagggaggatggcagggaatggtagatggttggtatgggtaccaccatagcaatgaacaggggagtgggtacgctgcagacaaagaatccacccaaaaggcaatagatggagttaccaacaaggtcaactcgatcattgacaaaatgaacactcaatttgaggccgttggaagggaatttaataacttagaaaggagaatagagaatttaaacaagaaaatggaagacggattcctagatgtctggacttataatgctgaacttctagttctcatggaaaatgagagaactctagatttccatgattcaaatgtcaggaacctttacgacaaagtccgactacagcttagggataatgcaaaggagctgggtaatggttgtttcgagttctatcacaaatgtgataatgaatgtatggaaagtgtaagaaatgggacgtatgactaccctcagtattcagaagaagcaagattaaaaagagaagaaataagcggagtgaaattagaatcaataggaacttaccaaatactgtcaatttattcaacagtggcgagttccctagcactggcaatcatggtggctggtctatctttatggatgtgctccaatgggtcgttacaatgcagaatttgcatt

>H5N6_A_wild_duck_South_Korea_1920_2019

atggagaaaatagtgcttcttcttgcagtggttagccttgttaaaagtgatcagatttgcattggttaccatgcaaacaactcgacagagcaggttgacacgataatggaaaaaaacgtcactgttacacatgctcaagacatactggaaaagacacacaacgggaggctctgcgatctgaatggagtgaaacctctgattttgaaggattgtagtgtagctggatggcttcttggaaacccaatgtgcgacgaattcatcagagtgccggaatggtcttacatagtggagagggctaacccagccaatgacctctgttacccagggaacctcaatgactatgaagaactgaaacacctattgagcagaataaatcattttgagaagactctgatcatccccaagagttcttggcccaatcatgaaacatcaGGGggggtgagctcagcatgcccataccagggagtgccctcctttttcagaaatgtggtatggcttaccaagaagaacgatgcatacccaacaataaagatgagctacaataataccaatggggaagatcttttgatactgtgggggattcatcattccaacaatgcagcagagcagacaaatctctataaaaacccaaccacctatgtttccgttgggacatcaacattaaaccagaaattggtgccaaaaatagctactagatcccaagtaaacgggcaacaaggaagaatggatttcttctggacaattttaaaaccgaatgatgcaatccactttgagagtaatggaaattttattgctccagaatatgcatacaaaatagtcaagaaaggggactcaacaattatgaaaagtgaaatggaatatggccactgcaacaccaaatgtcaaactccaataggggcgataaactctagtatgccattccacaatatacaccctctcaccatcggggagtgccccaaatacgtgaaatcaaacaaattagtccttgcgactggactcagaaatagtcctttaagagaaGGGagaagaagaaaaagaggactatttggagctatagcagggttcatagagggaggatggcaaggaatggtagatggttggtatgggtaccaccatagcaatgaacaggggagtgggtacgctgcagacagagaatccacccaaagggcaatagatggagttaccaataaggtcaactcgataatcgacaaaatgaacactcaatttgaggccgttggaagggagtttaataacttagaacggagaatagagaatttaaataagaaaatggaagacggattcctagatgtctggacttacaatgctgaacttttagttctcatggaaaatgagagaactttagattttcacgattcaaatgtaaaaaacctttatgacaaagtccgactacagcttagggataatgcaaaggagctaggtaatggttgtttcgagttctatcataaatgtgataatgaatgtatggaaagtgtaagaaatgggacgtatgactatccccagtattcagaagaagcaagattaaaaagggaagaaataagcggagtgaaattggaatcaataggaacttaccaaatactgtcaatttattcaacagtggcgagttccctaacactggcaatcattgtggctggtctatctttatggatgtgctccaatgggtcgttacaatgcagaatttgcatt

>H5N6_A_whooper_swan_Korea_Gangjin_48_2016

atggagaaaatagtgcttcttcttgcagtggttagccttgttaaaagtgatcagatttgcattggttatcatgcaaacaactcgacagagcaggttgacacgataatggaaaaaaacgtcactgttacacatgcccaagacatactggaaaagacacacaacgggaggctctgcgatctgaatggagtgaaacctctgattttgaaggattgtagtgtagctggatggcttcttggaaacccaatgtgcgacgaattcatcagagtgccggaatggtcttacatagtggagagggctaacccagccaatgacctctgttacccagggaacctcaatgactatgaagaactgaaacacctattgagcagaataaatcattttgagaagactctgatcatccccaagagttcttggcccaatcatgaaacatcaGGGggggtgagctcagcatgcccataccagggagtgccctcctttttcagaaatgtggtatggcttaccaagaagaacgatgcatacccaacaataaagatgagctacaataataccaatggggaagatcttttgatactgtgggggattcatcattccaacaatgcagcagagcagacaaatctctataaaaacccaaccacctatgtttccgttgggacatcaacattaaaccagagattggtgccaaaaatagctactagatcccaagtaaacgggcaacaaggaagaatggatttcttctggacaattttaaaaccgaatgatgcaatccactttgagagtaatggaaattttattgctccagaatatgcatacaaaatagtcaagaaaggggactcaacaattatgaaaagtgaaatggaatatggccactgcaacaccaaatgtcaaactccaataggggcgataaactctagtatgccattccacaatatacaccctctcaccataggggagtgccccaaatacgtgaaatcaaacaaattagtccttgcgactggactcagaaatagtcctttaagagaaGGGagaagaagaaaaagaggactatttggagctatagcagggttcatagagggaggatggcaaggaatggtagatggttggtatgggtaccaccatagcaatgaacaggggagtgggtacgctgcagacagagaatccacccaaagggcaatagatggagttaccaataaagtcaactcgataatcgacaaaatgaacactcaatttgaggccgttggaagggagtttaataacttagaacggagaatagagaatttaaataagaaaatggaagacggattcctagatgtctggacttacaatgctgaacttttagttctcatggaaaatgagagaactttagattttcacgattcaaatgtaaaaaacctttatgacaaagtccgactacagcttagggataatgcaaaggagctaggtaatggttgtttcgagttctatcataaatgtgataatgaatgtatggaaagtgtaagaaatgggacgtatgactatccccagtattcagaagaagcaagattaaaaagggaagaaataagcggagtgaaattggaatcaataggaacttaccaaatactgtcaatttattcaacagtggcgagttccctagcactggcaatcattgtggctggtctatctttatggatgtgctccaatgggtcgttacaatgcagaatttgcatt

>H5N6_A_coot_Shiga_2501T010_2017

atggagaaaatagtgcttcttcttgcagtggttagccttgttaaaagtgatcagatttgcattggttaccatgcaaacaactcgacagagcaggttgacacgataatggaaaaaaacgtcactgttacacatgcccaagacatactggaaaagacacacaacgggaggctctgcgatctgaatggagtgaaacctctgattttaaaggattgtagtgtagctggatggcttcttggaaacccaatgtgcgacgaattcatcagagtgccggaatggtcttacatagtggagagggctaacccagccaatgacctctgttacccagggaacctcaatgactatgaagaactgaaacacctattgagcagaataaatcattttgagaagactctgatcatccccaagagttcttggcctaatcatgaaacatcaGGGggggtgagctcagcatgcccataccagggagtgccctcctttttcagaaatgtggtatggcttaccaagaagaacgatgcatacccaacaataaagatgagctacaataataccaatggggaagatcttttgatactgtgggggattcatcattccaacaatgcagcagagcagacaaatctctataaaaacccaaccacctatatttccgttgggacatcaacattaaaccagagattggtgccaaaaatagctactagatcccaagtaaacgggcaacaaggaagaatggatttcttctggacaattttaaaaccgaatgatgcaatccactttgagagtaatggaaattttattgctccagaatatgcatacaaaatagtcaagaaaggggactcaacaattatgaaaagtgaaatggaatatggccactgcaacaccaaatgtcaaactccaataggggcgataaactctagtatgccattccacaatatacaccctctcaccatcggggagtgccccaaatacgtgaaatcaaacaaattagtccttgcgactggactcagaaatagtcctttaagagaaGGGagaagaagaaaaagaggactatttggagctatagcagggttcatagagggaggatggcaaggaatggtagatggttggtatgggtaccaccatagcaatgaacaggggaatgggtacgctgcagacagagaatccacccaaaaggcaatagatggagttaccaataaggtcaactcgataatcgacaaaatgaacactcaatttgaggccgttggaagggagtttaataacttagaacggagaatagagaatttaaataagaaaatggaagacggattcctagatgtctggacttacaatgctgaacttttagttctcatggaaaatgagagaactttagattttcacgattcaaatgtaaaaaacctttatgacaaagtccgactacagcttagggataatgcaaaggagctaggtaatggttgtttcgagttctatcataaatgtgataatgaatgtatggaaagtgtaagaaatgggacgtatgactatccccagtattcagaagaagcaagattaaaaagggaagaaataagcggagtgaaattggaatcaataggaacttaccaaatactgtcaatttattcaacagtggcgagttccctagcactggcaatcattgtggctggtctatctttatggatgtgctccaatgggtcgttacaatgcagaatttgcatt

>H5N6_A_pintail_Hokkaido_X8_2016

atggagaaaatagtgcttcttcttgcagtggttagccttgttaaaagtgatcagatttgcattggttaccatgcaaacaactcgacagagcaggttgacacgataatggaaaaaaacgtcactgttacacatgcccaagacatactggaaaggacacacaacgggaggctctgcgatctgaatggagtgaaacctctgattttaaaggattgtagtgtagctggatggcttcttggaaacccaatgtgcgacgaattcatcagagtgccggaatggtcttacatagtggagaggactaacccagccaatgacctctgttacccagggaacctcaatgactatgaagaactgaaacacctattgagcagaataaatcattttgagaagactcagatcatccccaagagttcttggcccaatcatgaaacatcaGGGggggtgagcgcagcatgcccataccagggagtgccctcctttttcagaaatgtggtatggcttaccaagaaaaacgatgcatacccaacaataaagatgagctacaataataccaatggggaagatcttttgatactgtgggggattcatcattccaacaatgcagcagagcagacaaatctctataaaaacccaaccacctatgtttccgttgggacatcaacattaaaccagagattggcgccaaaaatagctactagatcccaagtgaacgggcaacaaggaagaatggatttcttctggacaattttaaagccgaatgatgcaatccactttgagagtaatggaaattttattgctccagaatatgcatataaaatagtcaagaaaggggactcaacaattatgaaaagtgaaatggaatatggccactgcaacaccaaatgtcaaactccaataggggcgataaactctagtatgccattccacaatatacaccctctcaccatcggggagtgccccaaatacgtgaaatcaaacaaattagtccttgcgactggactcagaaatagtcctttaagagaaGGGagaagaagaaaaagaggactatttggagctatagcagggttcatagagggaggatggcaaggaatggtagatggttggtatgggtaccaccatagcaatgaacaggggagtgggtacgctgcagacagagaatccacccaaaaggcaatagatggagttaccaataaggtcaactcgataatcgacaaaatgaacactcaatttgaggccgttggaagggagtttaataacttagaacggagaatagagaatttaaataagaaaatggaagacggattcctagatgtctggacttacaatgctgaacttttagttctcatggaaaatgagagaactttagattttcacgattcaaatgtaaaaaacctttatgacaaagtccgactacagcttagggataatgcaaaggagctaggtaatggttgtttcgagttctatcataaatgtgataatgaatgtatggagagtgtaaggaatgggacgtatgactatccccagtattcagaagaagcaagattaaaaagggaagaaataagcggagtgaaattggaatcaataggaacttaccaaatactgtcaatttattcaacagtggcgagttccctagcactggcaatcattgtggctggtctatctttatggatgtgctccaatgggtcgttacaatgcagaatttgcatt

>H5N6_A_whooper_swan_Fukushima_1_2016

atggagaaaatagtacttcttcttgcagtggttagccttgttaaaagtgatcagatttgcattggttaccatgcaaacaactcgacagagcaggttgacacgataatggaaaaaaacgtcactgttacacatgcccaagacatactggaaaagacacacaacgggaggctctgcgatctgaatggagtgaaacctctgattttaaaggattgtagtgtagctggatggcttcttggaaacccaatgtgcgacgaattcatcagagtgccggaatggtcttacatagtggagaggactaacccagtcaatgacctctgttacccagggaacctcaatgactatgaagaactgaaacacctattgagcagaataaatcattttgagaagactctgatcatccccaagagttcttggtccaatcatgaaacatcaGGGggggcgagcgcagcatgcccataccagggagtgccctcctttttcagaaatgtggtatggcttaccaagaagaacgatgcatacccaacaataaagatgagctacaataataccaatggggaagatcttttgatactgtgggggattcatcattccaacaatgcagcagagcagacaaatctctataaaaacccaaccacctatgtttccgttgggacatcaacattaaaccagagattggtgccaaaaatagctactagatcccaagtaaacgggcaacaaggaagaatggatttcttctggacaattttaaaaccgaatgatgcaatccactttgagagtaatggaaattttattgctccagaatatgcatacaaaatagtcaagaaaggggactcaacaattatgaaaagtgaaatggaatatggccactgcaacaccaaatgtcaaactccaataggggcgataaactctagtatgccattccacaatatacaccctctcaccatcggggagtgccccaaatacgtgaaatcaaacaaattagtccttgtgactggactcagaaatagtcctttaagagaaGGGagaagaagaaaaagaggactatttggagctatagcagggttcatagagggaggatggcaaggaatggtagatggttggtatgggtaccaccatagcaatgaacaggggagtgggtacgctgcagacagagaatccacccaaaaggcaatagatggagttaccaataaggtcaactcgataatcgacaaaatgaacactcaatttgaggccgttggaagggagtttaataacttagaacggagaatagagaatttaaataagaaaatggaagacggattcctagacgtctggacttacaatgctgaacttttagttctcatggaaaatgagagaactttagattttcacgattcaaatgtaaaaaacctttatgacaaagtccgactacagcttagggataatgcaaaggagctaggtaatggttgtttcgagttctatcataaatgtgataatgaatgtatggaaagtgtaagaaatgggacgtatgactatccccagtattcagaagaagcaagattaaaaagggaagaaataagcggggtgaaattggaatcaataggaacttaccaaatactgtcaatttattcaacagtagcgagttccctagcactggcaatcattgtggctggtctatctttatggatgtgctccaatgggtcgttacaatgcagaatttgcatt

>H5N6_A_peregrine_falcon_Niigata_12_2017

atggagaaaatagtacttcttcttgcagtggttagccttgttaaaagtgatcagatttgcattggttaccatgcaaacaactcgacagagcaggttgacacgataatggaaaaaaacgtcactgttacacatgcccaagacatactggaaaagacacacaacgggaggctctgcgatctgaatggagtgaaacctctgattttaaaggattgtagtgtagctggatggcttcttggaaacccaatgtgcgacgaattcatcagagtgccggaatggtcttacatagtggagaggactaacccagccaatgacctctgttacccagggaacctcaatgactatgaagaactgaaacacctattgagcagaataaatcattttgagaagactctgatcatccccaagagttcttggcccaatcatgaaacatcaGGGggggcgagcgcagcatgcccataccagggagtgccctcctttttcagaaatgtggtatggcttaccaagaagaacgatgcatacccaacaataaagatgagctacaataataccaatggggaagatcttttgatactgtgggggattcatcattccaacaatgcagcagagcagacaaatctctataaaaacccaaccacctatgtttccgttgggacatcaacattaaaccagagattggtgccaaaaatagctactagatcccaagtaaacgggcaacaaggaagaatggatttcttctggacaattttaaaaccgaatgatgcaattcactttgagagtaatggaaattttattgctccagaatatgcatacaaaatagtcaagaaaggggactcaacaattatgaaaagtgaaatggaatatggccactgcaacaccaaatgtcaaactccaataggggcgataaactctagtatgccattccacaatatacaccctctcaccatcggggagtgccccaaatacgtgaaatcaaacaaattagtccttgcgactggactcagaaatagtcctttaagagaaGGGagaagaagaaaaagaggactatttggagctatagcagggttcatagagggaggatggcaaggaatggtagatggttggtatgggtaccaccatagcaatgaacaggggagtgggtacgctgcagacagagaatccacccaaaaggcaatagatggagttaccaataaggtcaactcgataatcgacaaaatgaacactcaatttgaggccgttggaagggagtttaataacttagaacggagaatagagaatttaaataagaaaatggaagacggattcctagatgtctggacttacaatgctgaacttttagttctcatggaaaatgagagaactttagattttcacgattcaaatgtaaaaaacctttatgacaaagtccgactacagcttagggataatgcaaaggagctaggtaatggttgtttcgagttctatcataaatgtgataatgaatgtatggaaagtgtaagaaatgggacgtatgactatccccagtattcagaagaagcaagattaaaaagggaagaaataagcggagtgaaattggaatcaataggaacttaccaaatactgtcaatttattcaacagtggcgagttccctagcactggcaatcattgtggctggtctatctttatggatgtgctccaatgggtcgttacaatgcagaatttgcatt

>H5N6_A_white_fronted_goose_Miyagi_1_2016

atggagaaaatagtgcttcttcttgcagtggttagccttgttaaaagtgatcagatttgcattggttaccatgcaaacaactcgacagagcaggttgacacgataatggaaaaaaacgtcactgttacacatgcccaagacatactggaaaagacacacaacgggaggctctgcgatctgaatggagtgaaacctctgattttaaaggattgtagtgtagctggatggcttcttggaaacccaatgtgcgacgaattcatcagagtgccggaatggtcttacatagtggagaggactaacccagccaatgacctctgttacccagggaacctcaatgactatgaagaactgaaacacctattgagcagaataaatcattttgagaagactctgatcatccccaagagttcttggcccaatcatgaaacatcaGGGggggtgagcgcagcatgcccataccagggagtgccctcctttttcagaaatgtggtatggcttaccaagaagaacgatgcatacccaacaataaagatgagctacaataataccaatggggaagatcttttgatactgtgggggattcatcattccaacaatgcagcagagcagacaaatctctataaaaacccaaccacctatgtttccgttgggacatcaacattaaaccagagattggtgccaaaaatagctactagatcccaagtaaacgggcaacaaggaagaatggatttcttctggacaattttaaaaccgaatgatgcaatccactttgagagtaatggaaattttattgctccagaatatgcatacaaaatagtcaagaaaggggactcaacaattatgaaaagtgaaatggaatatggccactgcaacaccaaatgtcaaactccaataggggcgataaactctagtatgccattccacaatatacaccctctcaccattggggagtgccccaaatacgtgaaatcaaacaaattagtccttgcaactggactcagaaatagtcctttaagagaaGGGagaagaagaaaaagaggactatttggagctatagcagggttcatagagggaggatggcaaggaatggtagatggttggtatgggtaccaccatagcaatgaacaggggagtgggtacgctgcagacagagaatccacccaaaaggcaatagatggagttaccaataaggtcaactcgataatcgacaaaatgaacactcaatttgaggccgttggaagggagtttaataacttagaacggagaatagagaatttaaataagaaaatggaagacggattcctagatgtctggacttacaatgctgaacttttagttctcatggaaaatgagagaactttagattttcacgattcaaatgtaaaaaacctttatgacaaagtccgactacagcttagggataatgcaaaggagctaggtaatggttgtttcgagttctatcataaatgtgataatgaatgtatggaaagtgtaagaaatgggacgtatgactatccccagtattcagaagaagcaagattaaaaagggaagaaataagcggagtgaaattggaatcaataggaacttaccaaatactgtcaatttattcaacagtggcgagttccctagcactggcaatcattgtggctggtctatctttatggatgtgctccaatgggtcgttacaatgcagaatttgcatt

>H5N6_A_duck_Tottori_E10_2016

atggagaaaatagtgcttcttcttgcagtggttagccttgttaaaagtgatcagatttgcattggttaccatgcaaacaactcgacagagcaggttgacacgataatggaaaaaaacgtcactgttacacatgcccaagacatactggaaaagacacacaacgggaggctctgcgatctgaatggagtgaaacctctgattttaaaggattgtagtgtagctggatggcttcttggaaacccaatgtgcgacgaattcatcagagtgccggaatggtcttacatagtggagaggactaacccagccaatgacctctgttacccagggaacctcaatgactatgaagaactgaaacacctattgagcagaataaatcattttgagaagactctgatcatccccaagagttcttggcccaatcatgaaacatcaGGGggggtgagcgcagcatgcccataccagggagtgccctcctttttcagaaatgtggtatggcttaccaagaagaacgatgcatacccaacaataaagatgagctacaataataccaatggggaagatcttttgatactgtgggggattcatcattccaacaatgcagcagagcagacaaatctctataaaaacccaaccacctatgtttccgttgggacatcaacattgaaccagagattggtgccaaaaatagctactagatcccaagtaaacgggcaacaaggaagaatggatttcttctggacaattttaaaaccgaatgatgcaatccactttgagagtaatggaaattttattgctccagaatatgcatacaaaatagtcaagaaaggggactcaacaattatgaaaagtgaaatggaatatggccactgcaacaccaaatgtcaaactccaataggggcgataaactctagtatgccattccacaatatacaccctctcaccattggggagtgccccaaatacgtgaaatcaaacaaattagtccttgcgactggactcagaaatagtcctttaagagaaGGGagaagaagaaaaagaggactatttggagctatagcagggttcatagagggaggatggcaaggaatggtagatggttggtatgggtaccaccatagcaatgaacaggggagtgggtacgctgcagacagagaatccacccaaaaggcaatagatggagttaccaataaggtcaactcgataatcgacaaaatgaacactcaatttgaggccgttggaagggagtttaataacttagaacggagaatagagaatttaaataagaaaatggaagacggattcctagatgtctggacttacaatgctgaacttttagttctcatggaaaatgagagaactttagattttcacgattcaaatgtaaaaaacctttatgacaaagtccgactacagcttagggataatgcaaaggagctaggtaatggttgtttcgagttctatcataaatgtgataatgaatgtatggaaagtgtaagaaatgggacgtatgactatccccagtattcagaagaagcaagattaaaaagggaagaaataagcggagtgaaattggaatcaataggaacttaccaaatactgtcaatttattcaacagtggcgagttccctagcactggcaatcattgtggctggtctatctttatggatgtgctccaatgggtcgttacaatgcagaatttgcatt

>H5N6_A_greater_scaup_Aichi_2301H050_2017

atggagaaaatagtgcttcttcttgcagtggttagccttgttaaaagtgatcagatttgcattggttaccatgcaaacaactcgacagagcaggttgacacgataatggaaaaaaacgtcactgttacacatgcccaagacatactggaaaagacacacaacgggaggctctgcgatctgaatggagtgaaacctctgattttaaaggattgtagtgtagctggatggcttcttggaaacccaatgtgcgacgaattcatcagagtgccggaatggtcttacatagtggagaggactaacccagtcaatgacctctgttacccagggaacctcaatgactatgaagaactgaaacacctattgagcagaataaatcattttgagaagactctgatcatccccaagagttcttggcccaatcatgaaacatcaGGGggggcgagctcagcatgcccatatcagggagtgccctcctttttcagaaatgtggtatggcttaccaagaagaacgatgcatacccaacaataaagatgagctacaataataccaatggggaggatcttttgatactgtgggggattcatcattccaacaatgcagcagagcagacaaatctctataaaaacccaaccacctatgtttccgttgggacatcaacactaaaccagagattggtgccaaaaatagctactagatcccaagtaaacgggcaacaaggaagaatggatttcttctggacaattttaaaaccgaatgatgcaatccactttgagagtaatggaaattttattgctccagaatatgcatacaaaatagtcaagaaaggggactcaacaattatgaaaagtgaaatggaatatggccactgcaacaccaaatgtcaaactccaataggggcgataaactctagtatgccattccacaatatacaccctctcaccattggggagtgccccaaatacgtgaaatcaaacaaattagtccttgcgactggactcagaaatagtccttcaagagaaGGGagaagaagaaaaagaggactatttggagctatagcggggttcatagaaggaggatggcaaggaatggtagatggttggtatgggtaccaccatagcaatgaacaggggagtgggtacgctgcagacagagaatccacccaaaaggcaatagatggagttaccaataaggtcaactcgataatcgacaaaatgaacactcaatttgaggccgttggaagggagtttaataacttagaacggagaatagagaatttaaataagaaaatggaagacggattcctagatgtctggacttacaatgctgaacttttagttctcatggaaaatgagagaactttagattttcacgattcaaatgtaaaaaacctttatgacaaagtccgactacagcttagggataatgcaaaggaactaggtaatggttgtttcgagttctatcataaatgtgataatgaatgtatggaaagtgtgagaaatgggacgtatgactatccccagtattcagaagaagcaagattaaaaagggaagaaataagcggagtgaaattggaatcaataggaacttaccaaatactgtcaatttattcaacagtggcgagttccctagcactggcaatcattgtggctggtctatctttatggatgtgctccaatgggtcgttacaatgcagaatttgcatt

>H5N6_A_mute_swan_Hyogo_2801ITM001_2017

atggagaaaatagtgcttcttcttgcagtggttagccttgttaaaggtgatcagatttgcattggttaccatgcaaacaactcgacagagcaggttgacacgataatggaaaaaaacgtcactgttacacatgcccaagacatactggaaaagacacacaacgggaggctctgcgatctgaatggagtgaaacctctgattttaaaggattgtagtgtagctggatggcttcttggaaacccaatgtgcgacgaattcatcagagtgccggaatggtcttacatagtggagaggactaacccagtcaatgacctctgttacccagggaacctcaatgactatgaagaactgaaacacctattgagcagaataaatcattttgagaagactctgatcattcccaagagttcttggcccaatcatgaaacatcaGGGggggtgagctcagcatgcccatatcagggagtgccctcctttttcagaaatgtggtatggcttaccaagaagaacgatgcatacccaacaataaagatgagctacaataataccaatggggaagatcttttgatactgtgggggattcatcattccaacaatgcagcagagcagacaaatctctataaaaacccaaccacctatatttccgttgggacatcaacattaaaccagaggttggtgccaaaaatagctactagatcccaagtaaacgggcaacaagggagaatggatttcttctggacaattttaaaaccgaatgatgcaatccactttgagagtaatggaaattttattgctccagaatatgcatacaaaatagtcaagaaaggggactcaacaattatgaaaagtgaaatggaatatggccactgcaacaccaaatgtcaaactccaataggggcgataaactctagtatgccattccacaatatacaccctctcaccattggggagtgccccaaatacgtgaaatcaaacaaattagtccttgcgactggactcagaaatagtccttcaagagaaGGGagaagaagaaaaagaggactatttggagctatagcagggttcatagaaggaggatggcaaggaatggtagatggttggtatgggtaccaccatagcaatgaacaggggagtgggtacgctgcagacagagaatccacccaaaaggcaatagatggagttaccaataaggtcaactcgataatcgacaaaatgaacactcaatttgaggccgttggaagggagtttaataacttagaacggagaatagagaatttaaataagaaaatggaagacggattcctagatgtctggacttacaatgctgaacttttagttctcatggaaaatgagagaactttagattttcacgattcaaatgtaaaaaacctttatgacaaagtccgactacagcttagggataatgcaaaggaactaggtaatggttgtttcgagttctatcataaatgtgataatgaatgtatggaaagtgtgagaaatgggacgtatgactatccccagtattcagaagaagcaagattaaaaagggaagaaataagcggagtgaaattggaatcaataggaacttaccaaatactgtcaatttattcaacagtggcgagttccctagcactggcaatcattgtggctggtctatctttatggatgtgctccaatgggtcgttacaatgcagaatttgcatt

>H5N6_A_wigeon_Aichi_2301H025_2017

atggagagaatagtgcttcttcttgcagtggttagccttgttaaaagtgatcagatttgcattggttaccatgcaaacaactcgacagagcaggttgacacgataatggaaaaaaacgtcactgttacacatgcccaagacatactggaaaagacacacaacgggaggctctgcgatctgaatggagtgaaacctctgattttaaaggattgtagtgtagctggatggcttcttggaaacccaatgtgcgacgaattcatcagagttccggaatggtcttacatagtggagaggactaacccagccaatgacctctgttacccagggaacctcaatgactatgaagaactgaaacacctattgagcagaataaatcattttgagaagactctgatcatccccaagagttcttggcccaatcatgaaacatcaGGGggggtgagcgcagcatgcccataccagggagtgccctcctttttcagaaatgtggtatggcttaccaagaagaacgatgcatacccaacaataaagatgagctacaataataccaatggggaagatcttttgatactgtgggggattcatcattccaacaatgcagcagagcagacaaatctctataaaaacccaaccacctatgtttccgttgggacatcaacattaaaccagagattggtgccaaaaatagctactagatcccaagtaaacgggcaacaaggaagaatggatttcttctggacaattttaaaaccgaatgatgcaatccactttgagagtaatggaaattttattgctccagaatatgcatacaaaatagtcaagaaaggggactcaacaattatgaaaagtgaaatggaatatggccactgcaacaccaaatgtcaaactccaataggggcgataaactctagtatgcccttccacaatatacaccctctcaccattggggagtgccccaaatacgtgaaatcaaacaaattagtccttgcgactggactcagaaatagtcctttaagagaaGGGagaagaagaaaaagaggactatttggagctatagcagggttcatagagggaggatggcaaggaatggtagatggttggtatgggtaccaccatatcaatgaacaggggagtgggtacgctgcagacagagaatccacccaaaaggcaatagatggagttaccaataaggtcaactcgataatcgacaaaatgaacactcaatttgaggccgttggaagggagtttaataacttagaacggagaatagagaatttaaataagaaaatggaagacggattcctagatgtctggacttacaatgctgaacttttagttctcatggaaaatgagagaactttagattttcacgattcaaatgtaaaaaacctttatgacaaagtccgactacagcttagggataatgcaaaggagctaggtaatggttgtttcgagttctatcataaatgtgataatgaatgtatggaaagtgtaagaaatgggacgtatgactatccccagtattcagaagaagcaagattaaaaagggaagaaataagcggagtgaaattggaatcaataggaacttaccaaatactgtcaatttattcaacagtggcgagttccctagcactggcaatcattgtggctggtctatctttatggatgtgctccaatgggtcgttacaatgcagaatttgcatt

>H5N6_A_black_swan_Aichi_2312T001_2016

atggagaaaatagtgcttcttcttgcagtggttagccttgttaaaagtgatcagatttgcattggttaccatgcaaacaactcgacagagcaggttgacacgataatggaaaaaaacgtcactgttacacatgcccaagacatactggaaaagacacacaacgggaggctctgcgatctgaatggagtgaaacctctgattttaaaggattgtagtgtagctggatggctccttggaaacccaatgtgcgacgaattcatcagagtgccggaatggtcttacatagtggagaggactaacccagccaatgacctctgttacccagggaacctcaatgactatgaagaactgaaacacctattgagcagaataaatcattttgagaagactctgatcatccccaagagttcttggcccaatcatgaaacatcaGGGggggtgagcgcagcatgcccataccagggagtgccctcctttttcagaaatgtggtatggcttaccaagaagaacgatgcatatccaacaataaagatgagctacaataataccaatggggaagatcttttgatactgtgggggattcatcattccaacaatgcagcagagcagacaaatctctataaaaacccaaccacctatgtttccgttgggacatcaacattaaaccagagattggtgccaaaaatagctactagatcccaagtaaacgggcaacaaggaagaatggatttcttctggacaattttaaaaccgaatgatgcaatccactttgagagtaatggaaattttattgctccagaatatgcatacaaaatagtcaagaaaggggactcaacaattatgaaaagtgaaatggaatatggccactgcaacaccaaatgtcaaactccaataggggcgataaactctagtatgccattccacaatatacaccctctcaccatcggggagtgccccaaatacgtgaaatcaaacaaattagtccttgcgactggactcagaaatagtcctttaagagaaGGGagaagaagaaaaagaggactatttggagctatagcagggttcatagagggaggatggcaaggaatggtagatggttggtatgggtaccaccatagcaatgaacaggggagtgggtacgctgcagacagagaatccacccaaaaggcaatagatggagttaccaataaggtcaactcgataatcgacaaaatgaacactcaatttgaggccgtcggaagggagtttaataacttagaacggagaatagagaatttaaataagaaaatggaagacggattcctagatgtctggacttacaatgctgaacttttagttctcatggaaaatgagagaactttagattttcacgattcaaatgtaaagaacctttatgacaaagtccgactacagcttagggataatgcaaaggagctaggtaatggttgtttcgagttctatcataaatgtgataatgaatgtatggaaagtgtaagaaatgggacgtatgactatccccagtattcagaagaggcaagattaaaaagggaagaaataagcggagtgaaattggaatcaataggaacttaccaaatactgtcaatttattcaacagtggcgagttccctagcactggcaatcattgtggctggtctatctttatggatgtgctccaatgggtcgttacaatgcagaatttgcatt

>H5N6_A_chicken_Hokkaido_002_2017

atggagaaaatagtgcttcttcttgcagtggttagccttgttaaaagtgatcagatttgcattggttaccatgcaaacaactcgacagagcaggttgacacgataatggaaaaaaacgtcactgttacacatgcccaagacatactggaaaagacacacaacgggaggctctgcgatctgaatggagtgaaacctctgattttaaaggattgtagtgtagctggatggcttcttggaaacccaatgtgcgacgaattcatcagagtgccggaatggtcttacatagtggagaggactaacccagccaatgacctctgttacccagggaacctcaatgactatgaagaactgaaacatctattgagcagaataaatcattttgagaagactctgatcatccccaagagttcttggcccaatcatgaaacatcaGGGggggtgagcgcagcatgcccataccagggagtgccctcctttttcagaaatgtggtatggcttaccaagaagaacgatgcatatccaacaataaagatgagctacaataataccaatggggaagatcttttgatactgtgggggattcatcattccaacaatgcagcagagcagacaaatctctataaaaacccaaccacctatgtttccgttgggacatcaacattaaaccagagattggtgccaaaaatagctactagatcccaagtaaacgggcaacaaggaagaatggatttcttctggacaattttaaaaccgaatgatgcaatccactttgagagtaatggaaattttattgctccagaatatgcatacaaaatagtcaagaaaggggactcaacaattatgaaaagtgaaatggaatatggccactgcaacaccaaatgtcaaactccaataggggcgataaactctagtatgccattccacaatatacaccctctcaccatcggggagtgccccaaatacgtgaaatcaaacaaattagtccttgcgactggactcagaaatagtcctttaagagaaGGGagaagaagaaaaagaggactatttggagctatagcagggttcatagagggaggatggcaaggaatggtagatggttggtatgggtaccaccatagcaatgaacaggggagtgggtacgctgcagacagagaatccacccaaaaggcaatagatggagttaccaataaggtcaactcgataatcgacaaaatgaacactcaatttgaggccgttggaagggagtttaataacttagaacggagaatagagaatttaaataagaaaatggaagacggattcctagatgtctggacttacaatgctgaacttttagttctcatggaaaatgagagaactttagattttcacgattcaaatgtaaagaacctttatgacaaagtccgactacagcttagggataatgcaaaggagctaggtaatggttgtttcgagttctatcataaatgtgataatgaatgtatggaaagtgtaagaaatgggacgtatgactatccccagtattcagaagaggcaagattaaaaagggaagaaataagcggagtgaaattggaatcaataggaacttaccaaatactgtcaatttattcaacagtggcgagttccctagcactggcaatcattgtggctggtctatctttatggatgtgctccaatgggtcgttacaatgcagaatttgcatt

>H5N6_A_cackling_goose_Aichi_2312T020_2016

atggagaaaatagtgcttcttcttgcagtggttagccttgttaaaagtgatcagatttgcattggttaccatgcaaacaactcgacagagcaggttgacacgataatggaaaaaaacgtcactgttacacatgcccaagacatactggaaaagacacacaacgggaggctctgcgatctgaatggagtgaaacctctgattttaaaggattgtagtgtagctggatggcttcttggaaacccaatgtgcgacgaattcatcagagtgccggaatggtcttacatagtggagaggactaacccagccaatgacctctgttacccagggaacctcaatgactatgaagaactgaaacacctattgagcagaataaatcattttgagaagactctgatcatccccaagagttcttggcccaatcatgaaacatcaGGGggggtgagcgcagcatgcccataccagggagtgccctcctttttcagaaatgtggtatggcttaccaagaagaacgatgcatatccaacaataaagatgagctacaataataccaatggggaagatcttttgatactgtggggaattcatcattccaacaatgcagcagagcagacaaatctctataaaaacccaaccacctatgtttccgttgggacatcaacattaaaccagagattggtgccaaaaatagctactagatcccaagtaaacgggcaacaaggaagaatggatttcttctggacaattttaaaaccgaatgatgcaatccactttgagagtaatggaaattttattgctccagaatatgcatacaaaatagtcaagaaaggggactcaacaattatgaaaagtgaaatggaatatggccactgcaacaccaaatgtcaaactccaataggggcgataaactccagtatgccattccacaatatacaccctctcaccatcggggagtgccccaaatacgtgaaatcaaacaaattagtccttgcgactggactcagaaatagtcctttaagagaaGGGagaagaagaaaaagaggactatttggagctatagcagggttcatagagggaggatggcaaggaatggtagatggttggtatgggtaccaccatagcaatgaacaggggagtgggtacgctgcagacagagaatccacccaaaaggcaatagatggagttaccaataaggtcaactcgataatcgacaaaatgaacactcaatttgaggccgttggaagggagtttaataacttagaacggagaatagagaatttaaataagaaaatggaagacggattcctagatgtctggacttacaatgctgaacttttagttctcatggaaaatgagagaactttagattttcacgattcaaatgtaaagaacctttatgacaaagtccgactacagcttagggataatgcaaaggagctaggtaatggttgtttcgagttctatcataaatgtgataatgaatgtatggaaagtgtaagaaatgggacgtatgactatccccagtattcagaagaggcaagattaaaaagggaagaaataagcggagtgaaattggaatcaataggaacttaccaaatactgtcaatttattcaacagtggcgagttccctagcactggcaatcattgtggctggtctatctttatggatgtgctccaatgggtcgttacaatgcagaatttgcatt

>H5N6_A_whooper_swan_Hokkaido_X13_2017

atggagaaaatagtgcttcttcttgcagtggttagccttgttaaaagtgatcagatttgcattggttaccatgcaaacaactcgacagagcaggttgacacgataatggaaaaaaacgtcactgttacacatgcccaagacatactggaaaagacacacaacgggaggctctgcgatctgaatggagtgaaacctctgattttaaaggattgtagtgtagctggatggcttcttggaaacccaatgtgcgacgaattcatcagagtgccggaatggtcttacatagtggagaggactaacccagccaatgacctctgttacccagggaacctcaatgactatgaagaactgaaacacctattgagcagaataaatcattttgagaagactctgatcatccccaagagttcttggcccaatcatgaaacatcaGGGggggtgagcgcagcatgcccataccagggagtgccctcctttttcagaaatgtggtatggcttaccaagaagaacgatgcatatccaacaataaagatgagctacaataataccaatggggaagatcttttgatactgtgggggattcatcattccaacaatgcagcagagcagacaaatctctataaaaacccaaccacctatgtttccgttgggacatcaacattaaaccagagattggtgccaaaaatagctactagatcccaagtaaacgggcaacaaggaagaatggatttcttctggacaattttaaaaccgaatgatgcaatccactttgagagtaatggaaattttattgctccagaatatgcatacaaaatagtcaagaaaggggactcaacaattatgaaaagtgaaatggaatatggccactgcaacaccaaatgtcaaactccaataggggcgataaactctagtatgccattccacaatatacaccctctcaccatcggggagtgccccaaatacgtgaaatcaaacaaattagtccttgcgactggactcagaaatagtcatttaagagaaGGGagaagaagaaaaagaggactatttggagctatagcagggttcatagagggaggatggcaaggaatggtagatggttggtatgggtaccaccatagcaatgaacaggggagtgggtacgctgcagacagagaatccacccaaaaggcaatagatggagttaccaataaggtcaactcgataatcgacaaaatgaacactcaatttgaggccgttggaagggagtttaataacttagaacggagaatagagaatttaaataagaaaatggaagacggattcctagatgtctggacttacaatgctgaacttttagttctcatggaaaatgagagaactttagattttcacgattcaaatgtaaagaacctttatgacaaagtccgactacagcttagggataatgcaaaggagctaggtaatggttgtttcgagttctatcataaatgtgataatgaatgtatggaaagtgtaagaaatgggacgtatgactatccccagtattcagaagaggcaagattaaaaagggaagaaataagcggagtgaaattggaatcaataggaacttaccaaatattgtcaatttattcaacagtggcgagttccctagcactggcaatcattgtggctggtctatctttatggatgtgctccaatgggtcgttacaatgcagaatttgcatt

>H5N6_A_whooper_swan_Tochigi_1_2017

atggagaaaatagtgcttcttcttgcagtggttagccttgttaaaagtgatcagatttgcattggttaccatgcaaacaactcgacagagcaggttgacacgataatggaaaaaaacgtcactgttacacatgcccaagacatactggaaaagacacacaacgggaggctctgcgatctgaatggagtgaaacctctgattttaaaggattgtagtgtagctggatggcttcttggaaacccaatgtgcgacgaattcatcagagtgccggaatggtcttacatagtggagaggactaacccagccaatgacctctgttacccagggaacctcaatgactatgaagaactgaaacacctattgagcagaataaatcattttgagaagactctgatcatccccaagagttcttggcccaatcatgaaacatcaGGGggggtgagcgcagcatgctcataccagggagtgccctcctttttcagaaatgtggtatggcttaccaagaagaacgatgcatatccaacaataaagatgagctacaataataccaatggggaagatcttttgatactgtgggggattcatcattccaacaatgcagcagagcagacaaatctctataaaaacccaaccacctatgtttccgttgggacatcaacattaaaccagagattggtgccaaaaatagctactagatcccaagtaaacgggcaacaaggaagaatggatttcttctggacaattttaaaaccgaatgatgcaatccactttgagagtaatggaaattttattgctccagaatatgcatacaaaatagtcaagaaaggggactcaacaattatgaaaagtgaaatggaatatggccactgcaacaccaaatgtcaaactccaataggggcgataaactctagtatgccattccacaatatacaccctctcaccatcggggagtgccccaaatacgtgaaatcaaacaaattagtccttgcgactggactcagaaatagtcctttaagagaaGGGagaagaagaaaaagaggactatttggagctatagcagggttcatagagggaggatggcaaggaatggtagatggttggtatgggtaccaccatagcaatgaacaggggagtgggtacgctgcagacagagaatccacccaaaaggcaatagatggagttaccaataaggtcaactcgataatcgacaaaatgaacactcaatttgaggccgttggaagggagtttaataacttagaacggagaatagagaatttaaataagaaaatggaagacggattcctagatgtctggacttacaatgctgaacttttagttctcatggaaaatgagagaactttagattttcacgattcaaatgtaaagaacctttatgacaaagtccgactacagcttagggataatgcaaaggagctaggtaatggttgtttcgagttctatcataaatgtgataatgaatgtatggaaagtgtaagaaatgggacgtatgactatccccagtattcagaagaggcaagattaaaaagggaagaaataagcggagtgaaattggaatcaataggaacttaccaaatactgtcaatttattcaacagtggcgagttccctagcactggcaatcattgtggctggtctatctttatggatgtgttccaatgggtcgttacaatgcagaatttgcatt

>H5N6_A_Northern_Pintail_Tottori_b37_2016

atggagaaaatagtgcttcttcttgcagtggttagccttgttaaaagtgatcagatttgcattggttaccatgcaaacaactcgacagagcaggttgacacgataatggaaaaaaacgtcactgttacacatgcccaagacatactggaaaagacacacaacgggaggctctgcgatctgaatggagtgaaacctctgattttaaaggattgtagtgtagctggatggcttcttggaaacccaatgtgcgacgaattcatcagagtgccggaatggtcttacatagtggagaggactaacccagccaatgacctctgttacccagggaacctcaatgactatgaagaactgaaacacctattgagcagaataaatcattttgagaagactctgatcatccccaagagttcttggcccaatcatgaaacatcaGGGggggtgagcgcagcatgcccataccagggagtgccctcctttttcagaaatgtggtatggcttaccaagaagaacgatgcatatccaacaataaagatgagctacaataataccaatggggaagatcttttgatactgtgggggattcatcattccaacaatgcagcagagcagacaaatctctataaaaacccaaccacctatgtttccgttgggacaacaacagtaaaccagagattggtgccaaaaatagctactagatcccaagtaaacgggcaacaaggaagaatggatttcttctggacaattttaaaaccgaatgatgcaatccactttgagagtaatggaaattttattgctccagaatatgcatacaaaatagtcaagaaaggggactcaacaattatgaaaagtgaaatggaatatggccactgcaacaccaaatgtcaaactccaataggggcgataaactctagtatgccattccacaatatacaccctctcaccatcggggagtgccccaaatacgtgaaatcaaacaaattagtccttgcgactggactcagaaatagtcctttaagagaaGGGagaagaagaaaaagaggactatttggagctatagcagggttcatagagggaggatggcaaggaatggtagatggttggtatgggtaccaccatagcaatgaacaggggagtgggtacgctgcagacagagaatccacccaaaaggcaatagatggagttaccaataaggtcaactcgataatcgacaaaatgaacactcaatttgaggccgttggaagggagtttaataacttagaacggagaatagagaatttaaataagaaaatggaagacggattcctagatgtctggacttacaatgctgaacttttagttctcatggaaaatgagagaactttagattttcacgattcaaatgtaaagaacctttatgacaaagtccgactacagcttagggataatgcaaaggagctaggtaatggttgtttcgagttctatcataaatgtgataatgaatgtatggaaagtgtaagaaatgggacgtatgactatccccagtattcagaagaggcaagattaaaaagggaagaaataagcggagtgaaattggaatcaataggaacttaccaaatactgtcaatttattcaacagtggcgagttccctagcactggcaatcattgtggctggtctatctttatggatgtgctccaatgggtcgttacaatgcagaatttgcatt

>H5N6_A_tundra_swan_Niigata_1_2016

atggagaaaatagtgcttcttcttgcagtggttagccttgttaaaagtgatcagatttgcattggttaccatgcaaacaactcgacagagcaggttgacacgataatggaaaaaaacgtcactgttacacatgcccaagacatactagaaaagacacacaacgggaggctctgcgatctgaatggagtgaaacctctgattttaaaggattgtagtgtagctggatggcttcttggaaacccaatgtgcgacgaattcatcagagtgccggaatggtcttacatagtggagaggactaacccagccaatgacctctgttacccagggaacctcaatgattatgaagaactgaaacacctattgagcagaataaatcattttgagaagactctgatcatccacaagagttcttggcccaatcatgaaacatcaGGGggggtgagcgcagcatgcccataccagggagtgccctcctttttcagaaatgtggtatggcttaccaagaagaacgatgcatacccaccaataaagatgagctacaataataccaatggggaagatcttttgatactgtgggggattcatcattccaacaatgcagcagagcagacaaatctctataaaaacccaaccacctatgtttccgttgggacatcaacattaaaccagagattggtgccaaaaatagctactagatcccaattaaacgggcaacaaggaagaatggatttcttctggacaattttaaaaccgaatgatgcaatccactttgagagtaatggaaattttattgctccagaatatgcatacaaaatagtcaagaaaggggactcaacaattatgaaaagtgaaatggaatatggccactgcaacaccaaatgtcaaactccaataggggcgataaactctagtatgccattccacaatatacaccctctcaccatcggggagtgccccaaatacgtgaaatcaaacaaattagtccttgcgactggactcagaaatagtcctttaagagaaGGGagaagaagaaaaagaggactatttggagctatagcagggttcatagagggaggatggcaaggaatggtagatggttggtatgggtaccaccatagcaatgaacaggggagtgggtacgctgcagacagagaatccacccaaaaggcaatagatggagttaccaataaggtcaactcgataatcgacaaaatgaacactcaatttgaggccgttggaagggagtttaataacttagaacggagaatagagaatttaaataagaaaatggaagacggattcctagatgtctggacttacaatgctgaacttttagttctcatggaaaatgagagaactttagattttcacgattcaaatgtaaagaacctttatgacaaagtccgactacagcttagggataatgcaaaggagctaggtaatggttgtttcgagttctatcataaatgtgataatgaatgtatggaaagtgtaagaaatgggacgtatgactatccccagtattcagaagaggcaagattaaaaagggaagaaataagcggagtgaaattggaatcaataggaacttaccaaatactgtcaatttattcaacagtggcgagttccctagcactggcaatcattgtggctggtctatctttatggatgtgctccaatgggtcgttacaatgcagaatttgcatt

>H5N6_2344h_A_Guangdong_18SF020_2018

atggagaaaatagtacttcttctttcagtggttggccttgttaaaagtgatcagatttgcattggttaccatgcaaacaactcgacagagcaggttgacacaataatggaaaaaaacgtcactgtcacgcatgcccaagacatactggaaaagacacacaacgggaagctctgcgatctgaatggagtgaaacctctggttttaaagaattgtagtgtagctggatggcttcttggaaacccaatgtgcgacgagttcatcagcgtgccggaatggtcttatatagtggagagggctaacccagccaatgacctctgttacccagggaacctcaatgactatgaagaactgaaacacctattgagcagaataaatcattttgagaagactcagatcatccccaagaggtcttggtccaatcatGGGacatcatcaggagtgagcgcagcatgtccataccaaggggtggcctccttttttagaaatgtggtatggcttaccaagaagaatgatgcatacccgacaataaagatgagctacaataataccaacaaagaagatcttttgatactgtggggaatccatcattccaacagtgcagaggagcagacagatctctacaagaacccaaccacctatgtttccgttgggacatcaacactaaaccagaggttggtgccaaaaatagctactagatcccaagtaaatgggcaacgtggaagaatggatttcttctggacaattttaagaccgaatgatgcaatccacttcgagagtaatgggaattttatcgctccagaatatgcatacaaaattatcaagacaggagactcaacaattatgaaaagtgaaatagaatatggcaactgcaacaccaagtgtcaaactccaataggggcgataaactctagtatgccattccacaatatacatcctctcactatcggggagtgccccaaatatgtgaaatcaaacaaattagtccttgcgactgggctcagaaatagtcccctaagagaaGGGagaagaagaaaaagaggactgtttggagctatagcagggtttatagagggaggatggcaaggaatggtagatggttggtatgggtaccaccatagtaatgaacaggggagtgggtatgctgcagacagagaatccacccaaaaggcaatagatggagtcaccaacaaggtcaactcgataattgacaaaatgaacactcaatttgaggccgttggaagagaatttaatagcttagaacggagaatagagaatttaaataagaaaatggaagacggattcctagatgtctggacttataacgctgaacttttagttctcatggaaaatgagagaactctagatttccatgactcaaatgtcaagaacctttatgacaaagtccgactacagcttagggataatgcaaaggagctgggtaatggttgtttcgagttctatcacaaatgtgataatgaatgtatggaaagtgtaagaaatggaacgtatgactacccccagtactcagaagaagcaagattaaaaagggaagaaataagcggagtgaaattggaatcaataggaacttaccaaatactgtcaatttattcaacagtggcgagttccctagtactggcaatcattatggctggtctatctttatggatgtgctccaatgggtcgttacaatgcagaatttgcatt

>H5N6_A_whooper_swan_Iwate_1_2016

atggagaaaatagtgcttcttcttgcagtggttagccttgttaaaagtgatcagatttgcattggttaccatgcaaacaactcgacagagcaggttgacacgataatggaaaaaaacgtcactgttacacatgcccaagacatactggaaaaaacacacaacgggaggctctgcgatctgaatggagtgaaacctctgattttaaaggattgtagtgtagctggatggcttcttggaaacccaatgtgcgacgaattcatcagagtgccggaatggtcttacatagtggagaggactaacccagccaatgacctctgttacccagggaacctcaatgactatgaagaactgaaacacctattgagcagaataaatcattttgagaagactctgatcatccccaagagttcttggcccaatcatgaaacatcaGGGggggtgagcgcagcatgcccataccagggagtgccctcctttttcagaaatgtggtatggcttaccaagaagaacgatgcatacccaacaataaagatgagctacaataataccaatggggaagatcttttgatactgtgggggattcatcattccaacaatgcagcagagcagacaaatctctataaaaacccaaccacctatgtttccgttgggacatcaacattaaaccagagattggtgccaaaaatagctactagatcccaagtaaacgggcaacaaggaagaatggatttcttctggacaattttaaaaccgaatgatgcaatccactttgagagtaatggaaattttattgctccagaatatgcatacaaaatagtcaagaaaggggactcaacaattatgaaaagtgaaatggaatatggccactgcaacaccaaatgtcaaactccaataggggcgataaactctagtatgccattccacaatatacaccctctcaccatcggggagtgccccaaatacgtgaaatcaaacaaattagtccttgcgactggactcagaaatagtcctttaagagaaGGGagaagaagaaaaagaggactatttggagctatagcagggttcatagagggaggatggcaaggaatggtagatggttggtatgggtaccaccatagcaatgaacaggggagtgggtacgctgcagacagagaatccacccaaaaggcaatagatggagttaccaataaggtcaactcgataatcgacaaaatgaacactcaatttgaggccgttggaagggagtttaataacttagaacggagaatagagaatttaaataagaaaatggaagacggattcctagatgtctggacttacaatgctgaacttttagttctcatggaaaatgagagaactttagattttcacgattcaaatgtaaagaacctttatgacaaagtccgactacagcttagggataatgcaaaggagctaggtaatggttgtttcgagttctatcataaatgtgataatgaatgtatggaaagtgtaagaaatgggacgtatgactatccccagtattcagaagaggcaagattaaaaagggaagaaataagcggagtggaattggaatcaataggaacttaccaaatactgtcaatttattcaacagtggcgagttccctagcactggcaatcattgtggctggtctatctttatggatgtgctccaatgggtcgttacaatgcagaatttgcatt

>H5N6_A_common_teal_Korea_W558_2017

atggagaaaatagtgcttcttcttgcagtggttagccttgttaaaagtgatcagatttgcattggttaccatgcaaacaactcgacagagcaggttgacacgataatggaaaaaaacgtcactgttacacatgcccaagacatactggaaaagacacacaacgggaggctctgcgatctgaatggagtgaaacctctgattttaaaggattgtagtgtagctggatggcttcttggaaacccaatgtgcgacaaattcatcagagtgccggaatggtcttacatagtggagaggaataacccagccaatgacctctgttacccagggaacctcaatgactatgaagaactgaaacacctattgagcagaataaatcattttgagaagactctgatcatccccaagagttcttggcccaatcatgaaacatcaGGGggggtgagcgcagcatgcccataccagggagtgccctcctttttcagaaatgtggtatggcttaccaagaagaacgatgcatacccaacaataaagatgagctacaataataccaatggggaagatcttttgatactgtgggggattcatcattccaacaatgcagcagagcagacaaatctctataaaaacccaaccacctatgtttccgttgggacatcaacattaaaccagagattggtgccaaaaatagctactagatcccaagtaaacgggcaacaaggaagaatggatttcttctggacaattttaaaaccgaatgatgcaatccactttgagagtaatggaaattttattgctccagaatatgcatacaaaatagtcaagaagggggactcaacaattatgaaaagtgaaatggaatatggccactgcaacaccaaatgtcaaactccaataggggcgataaactctagtatgccattccacaatatacaccctctcaccatcggggagtgccccaaatacgtgaaatcaaacaaattagtccttgcgactggactcagaaatagtcctttaagagaaGGGagaagaagaaaaagaggactatttggagctatagcagggttcatagagggaggatggcaaggaatggtagatggttggtatgggtaccaccatagcaatgaacaggggagtgggtacgctgcagacagagaatccacccaaaaggcaatagatggagttaccaataaggtcaactcgataatcgacaaaatgaacactcaatttgaggccgttggaagggagtttaataacttagaacggagaatagagaatttaaataagaaaatggaagacggattcctagatgtctggacttacaatgctgaacttttagttctcatggaaaatgagagaactttagattttcacgattcaaatgtaaagaacctttatgacaaagtccgactacagcttagggataatgcaaaggagctaggtaatggttgtttcgagttctatcataaatgtgataatgaatgtatggaaagtgtaagaaatgggacgtatgactatccccagtattcagaagaagcaaaattaaaaagggaagaaataagcggagtgaaattggaatcaataggaacttaccaaatattgtcaatttattcaacagtggcgagttccctagcactggcaatcattttggctggtctatctttatggatgtgctccaatgggtcgttacaatgcagaatttgcatt

>H5N6_A_chicken_Vietnam_QuangBinh_BT1113_2017

atggagaaaatagtgcttcttcttgcagtggttagccttgttaaaagtgatcagatttgcattggttaccatgcaaacaactcgacagagcaggttgacacgataatggaaaaaaacgtcactgttacacatgcccaagacatactggaaaagacacacaacgggaggctctgcgatctgaatggagtgaaacctctgattttaaaggattgtagtgtagctggatggcttcttggaaacccaatgtgcgacgaattcatcagagtgccggaatggtcttacatagtggagaggactaacccagccaatgacctctgttacccagggaacctcaatgactatgaagaactgaaacacctattgagcagaataaatcattttgagaagactctgatcatccccaagagttcttggcccaatcatgaaacatcatcaggggtgagcgcagcatgcccataccagggagtgccctcctttttcagaaatgtggtatggcttaccaagaagaacgatgcatacccaacaataaagatgagctacaataataccaatggggaagatcttttgatactgtgggggattcatcattccaacaatgaagcagagcagacaaatctctataaaaacccaaccacctatgtttccgttgggacatcaacattaaaccagagattggtgccaaaaatagctactagatcccaagtaaacgggcaacaaggaagaatggatttcttctggacaattttaaaaccgaatgatgcaatccactttgagagtaatggaaattttattgctccagaatatgcatacaaaatagtcaagaaaggggactcaacaattatgaaaagtgaaatggaatatggccactgcaacaccaaatgtcaaactccaataggggcgataaactctagtatgccattccacaatatacaccctctcaccatcggggagtgccccaaatacgtgaaatcaaacaaattagtccttgcgactggactcagaaatagtcctttaagagaaGGGagaagaagaaaaagaggactatttggagctatagcagggttcatagagggaggatggcaaggaatggtagatggttggtatgggtaccaccatagcaatgaacaggggagtgggtacgctgcagacagagaatccacccaaaaggcaatagatggagttaccaataaggtcaactcgataatcgacaaaatgaacactcaatttgaggccgttgggagggagtttaataacttagaacggagaatagagaatttaaataagaaaatggaagacggattcctagatgtctggacttacaatgctgaacttttagttctcatggaaaatgagagaactttagattttcacgattcaaatgtaaagaacctttatgacaaagtccgactacagcttagggataatgcaaaggagctaggtaatggttgtttcgagttctatcataaatgtgataatgaatgtatggaaagtgtaagaaatgggacgtatgactatccccagtattcagaagaagcaagattaaaaagggaagaaataagcggagtgaaattggaatcaataggaacttaccaaatactgtcaatttattcaacagtggcgagttccctagcactggcaatcattgtggctggtctatctttatggatgtgctccaatgggtcgttacaatgcagaatttgcatt

>H5N6_A_snowy_owl_Akita_0051D007_2016

atggagaaaatagtgcttcttcttgcagtggtcagccttgttaaaagtgatcagatctgcattggttaccatgcaaacaactcgacagagcaggttgacacgataatggaaaaaaacgtcactgttacacatgcccaagacatactggaaaagacacacaacgggaggctctgcgatctgaatggagtgaaacctctgattctaaaggattgtagtgtagctggatggcttcttggaaacccaatgtgcgacgaattcatcagagtgccggaatggtcttacatagtggagaggactaacccagccaatgacctctgttacccagggaacctcaatgactatgaagaactgaaacacctattgagcagaataaatcattttgagaagactctgatcatccccaagagttcttggcccaatcatgaaacatcaGGGggggtgagcgcagcatgcccataccagggagtgccctcctttttcagaaatgtggtatggcttaccaagaagaacgatgcatacccaacaataaagatgagctacaataataccaatggggaagatcttttgatactgtgggggattcatcattccaacaatgcagcagagcagataaatctctataaaaacccaaccacctatgtttccgttgggacatcaacattaaaccagagattggtgccaaaaatagctactagatcccaagtaaacgggcaacaaggaagaatggatttcttctggacaattttaaaaccgaatgatgcaatccactttgagagtaatggaaattttattgctccagaatatgcatacaaaatagtcaagaaaggggactcaacaattatgaaaagtgaaatggaatatggccactgcaacaccaaatgtcaaactccaataggggcgataaactctagtatgccattccacaatatacaccctctcaccatcggggagtgccccaaatacgtgaaatcaaacaaattagtccttgcgactggactcagaaatagtcctttaagagaaGGGagaagaagaaaaagaggactatttggagctatagcagggttcatagagggaggatggcaaggaatggtagatggttggtatgggtaccaccatagcaatgaacaggggagtgggtacgctgcagacagagaatccacccaaaaggcaatagatggagttaccaataaggtcaactcgataatcgacaaaatgaacactcaatttgaggccgttggaagggagtttaataacttagaacggagaatagagaatttaaataagaaaatggaagacggattcctagatgtctggacttacaatgctgaacttttagttctcatggaaaatgagagaactttagattttcacgattcaaatgtaaagaacctttatgacaaagtccgactacagcttagggataatgcaaaggagctaggtaatggttgtttcgagttctatcataaatgtgataatgaatgtatggaaagtgtaagaaatgggacgtatgactatccccagtattcagaagaagcaagattaaaaagggaagaaataagcggagtgaaattggaatcaataggaacttaccaaatactgtcaatttattcaacagtggcgagttccctagcactggcaatcattgtggctggtctatctttatggatgtgctccaatgggtcgttacaatgcagaatttgcatt

>H5N6_A_black_swan_Akita_2_2016

atggagaaaatagtgcttcttcttgcagtggtcagccttgttaaaagtgatcagatctgcattggttaccatgcaaacaactcgacagagcaggttgacacgataatggaaaaaaacgtcactgttacacatgcccaagacatactggaaaagacacacaacgggaggctctgcgatctgaatggagtgaaacctctgattctaaaggattgtagtgtagctggatggcttcttggaaacccaatgtgcgacgaattcatcagagtgccggaatggtcttacatagtggagaggactaacccagccaatgacctctgttacccagggaacctcaatgactatgaagaactgaaacacctattgagcagaataaatcattttgagaagactctgatcatccccaagagttcttggcccaatcatgaaacatcaGGGggggtgagcgcagcatgcccataccagggagtgccctcctttttcagaaatgtggtatggcttaccaagaagaacgatgcatacccaacaataaagatgagctacaataataccaatggggaagatcttttgatactgtgggggattcatcattccaacaatgcagcagagcagataaatctctataaaaacccaaccacctatgtttccgttgggacatcaacattaaaccagagattggtgccaaaaatagctactagatcccaagtaaacgggcaacaaggaagaatggatttcttctggacaattttaaaaccgaatgatgcaatccactttgagagtaatggaaattttattgctccagaatatgcatacaaaatagtcaagaaaggggactcaacaattatgaaaagtgaaatggaatatggccactgcaacaccaaatgtcaaactccaataggggcgataaactctagtatgccattccacaatatacaccctctcaccatcggggagtgccccaaatacgtgaaatcaaacaaattagtccttgcgactggactcagaaatagtcctttaagagaaGGGagaagaagaaaaagaggactatttggagctatagcagggttcatagagggaggatggcaaggaatggtagatggttggtatgggtaccaccatagcaatgaacaggggagtgggtacgctgcagacagagaatccacccaaaaggcaatagatggagttaccaataaggtcaactcgataatcgacaaaatgaacactcaatttgaggccgttggaagggagtttaataacttagaacggagaatagagaatttaaataagaaaatggaagacggattcctagatgtctggacttacaatgctgaacttttagttctcatggaaaatgagagaactttagattttcacgattcaaatgtaaagaacctttatgacaaagtccgactacagcttagggataatgcaaaggagctaggtaatggttgtttcgagttctatcataaatgtgataatgaatgtatggaaagtgtaagaaatgggacgtatgactatccccagtattcagaagaagcaagattaaaaagggaagaaataagcggagtgaaattggaatcaataggaacttaccaaatactgtcaatttattcaacagtggcgagttccctagcactggcaatcattgtggctggtctatctttatggatgtgctccaatgggtcgttacaatgcagaatttgcatt

>H5N6_A_chicken_Miyazaki_21_2016

atggagaaaatagtgcttcttcttgcagtggttagccttgttaaaagtgatcagatctgcattggttaccatgcaaacaactcgacagagcaggttgacacgataatggaaaaaaacgtcactgttacacatgcccaagacatactggaaaagacacacaacgggaggctctgcgatctgaatggagtgaaacctctgattctaaaggattgtagtgtagctggatggcttcttggaaacccaatgtgcgacgaattcatcagagtgccggaatggtcttacatagtggagaggactaacccagccaatgacctctgttacccagggaacctcaatgactatgaagaactgaaacacctattgagcagaataaatcattttgagaagactctgatcatccccaagagttcttggcccaatcatgaaacatcaGGGggggtgagcgcagcatgcccataccagggagtgccctcctttttcagaaatgtggtatggcttaccaagaagaacgatgcatacccaacaataaagatgagctacaataataccaatggggaagatcttttgatactgtgggggattcatcattccaacaatgcagcagagcagacaaatctctataaaaacccaaccacctatgtttccgttgggacatcaacattaaaccagagattggtgccaaaaatagctactagatcccaagtaaacgggcaacaaggaagaatggatttcttctggacaattttaaaaccgaatgatgcaatccactttgagagtaatggaaattttattgctccagaatatgcatacaaaatagtcaagaaaggggactcaacaattatgaaaagtgaaatggaatatggccactgcaacaccaaatgtcaaactccaataggggcgataaactctagtatgccattccacaatatacaccctctcaccatcggggagtgccccaaatacgtgaaatcaaacaaattagtccttgcgactggactcagaaatagtcctttaagagaaGGGagaagaagaaaaagaggactatttggagctatagcagggttcatagagggaggatggcaaggaatggtagatggttggtatgggtaccaccatagcaatgaacaggggagtgggtacgctgcagacagagaatccacccaaaaggcaatagatggagttaccaataaggtcaactcgataatcgacaaaatgaacactcaatttgaggccgttggaagggagtttaataacttagaacggagaatagagaatttaaataagaaaatggaagacggattcctagatgtctggacttacaatgctgaacttttagttctcatggaaaatgagagaactttagattttcacgattcaaatgtaaagaacctttatgacaaagtccgactacagcttagggataatgcaaaggagctaggtaatggttgtttcgagttctatcataaatgtgataatgaatgtatggaaagtgtaagaaatgggacgtatgactatccccagtattcagaagaagcaagattaaaaagggaagaaataagcggagtgaaattggaatcaataggaacttaccaaatactgtcaatttattcaacagtggcgagttccctagcactggcaatcattgtggctggtctatctttatggatgtgctccaatgggtcgttacaatgcagaatttgcatt

>H5N6_A_white_naped_crane_China_ya3_2018

atggagaaaatagtgcttcttcttgcagtggttagccttgtcaaaagtgatcagatttgcattggttaccatgcaaacaactcgacagagcaggttgacacgataatggaaaaaaacgtcactgttacacatgcccaagacatactggaaaagacacacaacggaaggctctgcgatctgaatggagtaaaacctctgattttaaaggattgtagtgtagctggatggcttcttggaaacccaatgtgcgacgaattcatcagggtgccggaatggtcttacatagtggagaggactaacccaaccaatgacctctgttacccagggaacctcaatgactatgaagaactgaaacacctattgagcagaataaatcattttgagaagactctgatcatccccaatagttcttggcccaatcatgaaacatcaGGGggggtgagcgcagcgtgcccataccagggagtgccctccttttacagaaatgtggtatggcttaccaagaagaatgatgcatacccaacaataaagatgagctacaataataccaatggggaagatcttttaatactgtgggggattcatcattccaacaatgcagaagagcagacaaaactctataaaaatccaaccacctatgtttccgttgggacatcaacattaaaccagagattggtgccaaaaatagctactagatcccaagtaaacgggcaacaaggaagaatggatttcttctggactattttaaaaccgaatgatgcaatccactttgagagtaatggaaattttattgctccagagtatgcatacaaaatagtcaagaaaggagactcaacaattatgaaaagtgaaatggaatatggccactgcaacaccaaatgtcaaactccaatcggggcgataaactctagtatgccattccacaatatacaccctctcaccatcggggagtgccctaaatacgtgaaatcaaacaaattagtccttgcgactgggctcagaaatagtccttcaagagaaGGGaagagaagaaaaagaggactatttggagctatagcagggttcatagagggaggatggcaaggaatggtagatggttggtatgggtaccatcacagcaatgaacaggggagtgggtacgctgcagacagagaatccacccaaaaggcaatagatggagttaccaataaggtcaactcgataattgacaaaatgaacactcaatttgaggccgttggaagggagtttaataacttagaacggaggatagagaatttaaataagaaaatggaagacggattcctagatgtctggacttacaatgctgaacttttagttctcatggaaaatgagagaactttagatttccatgattcaaatgtcaaaaacctttatgacaaagtccgactacagcttagggataatgtaaaagagctgggtaatggttgtttcgagttctatcacaaatgtgatgatgaatgtatggaaagcgtaagaaatgggacgtatgactatccccagtattcagaagaagcaagattaaaaagggaagaaataagcggagtgaaattggaatcaataggaacttatcaaatactgtcaatttattcaacagtggcgagttccctagcactggcaatcattgtggctggtctatctttatggatgtgctccaatgggtcgttacaatgcagaatttgcatt

>H5N6_A_duck_Guangzhou_41227_2014

atggagaaaatagtgcttcttcttgcagtggttagccttgttaaaagtgatcagatttgcattggttaccatgcaaacaactcgacagagcaggttgacacgataatggaaaaaaacgtcactgttacacatgcccaagacatactggaaaagacacacaacgggaggctctgcgatctgaatggagtgaaacctctgattttaaaggattgtagtgtagctggatggcttcttggaaacccaatgtgcgacgagttcatcagagtgccggaatggtcttacatagtggagagggctaacccagccaatgacctctgttacccagggaatctcaatgactatgaagaactgaaacacctattgagcagaataaatcattttgagaagactctgatcatccccaagagttcttggcccaatcatgaaacatcattaggggtgagtgcagcatgtccataccagggagtgccctcctttttcagaaatgtggtatggcttaccaagaagaacgatgcatacccaacaataaagatgagctacaataataccaatagggaagatcttttgatactgtgggggattcatcattccaacaatgcagcagagcagacaaatctctataaaaacccaaccacctatgtttccgttgggacatcaacattaaaccagagattggtgccaaaaatagctactagatcccaagtaaacgggcaacgtggaagaatggatttcttctggacaattttaaaaccgaatgatgcaatccacttcgagagtaatggaaattttattgctccagaatatgcatacaaaatagtcaagaaaggggactcaacaattatgaaaagtgaaatggaatatggccactgcaacaccaaatgtcaaactccaataggggcgataaactctagtatgccattccacaatatacaccctctcaccatcggggagtgccccaaatacgtgaaatcaaacaaattagtccttgcgactgggctcagaaatagtcctctaagagaaGGGagaagaagaaaaagaggactatttggagctatagcagggttcatagagggaggatggcaaggaatggtagatggttggtatgggtaccaccatagcaatgaacaggggagtgggtacgctgcagacagagaatccacccaaaaggcaatagatggagttaccaataaggtcaactcgataattgacaaaatgaacactcaatttgaggccgttggaagggagtttaataacttagaacggagaatagagaatttaaataagaaaatggaagacggattcctagatgtctggacttacaatgctgaacttttagttctcatggaaaatgagagaactctagatttccatgattcaaatgtcaagaacctttatgacaaagtccgactacagcttagggataatgcaaaggagctgggtaatggttgtttcgagttctatcacaaatgtgataatgaatgtatggaaagtgtaagaaatgggacgtatgactacccccagtattcagaagaagcaagattaaaaagggaagaaataagcggagtgaaattggaatcaataggaacttaccaaatactgtcaatttattcaacagtggcgagttccctaacactggcaatcattgtggctggtctatctttatggatgtgctccaatgggtcgttacaatgcagaatttgcatt

>H5N6_A_peregrine_falcon_HK_4955_2015

atggaggaaatagtgcttcttcttgcagtggttagccttgttaaaagtgatcagatttgcattggttaccatgcaaacaactcaacagagcaggttgacacgataatggaaaaaaacgtcactgttacacatgcccaagacatactggaaaagacacacaacgggaggctctgcgatctgaatggagtgaaacctctgattttaaaggattgtagtgtagctggatggcttcttggaaacccaatgtgcgacgagttcatcagagtgccggaatggtcttacatagtggagagggctaacccagtcaatgacctctgttacccagggaacctcaatgactatgaagaactgaaacacctattgagcagaataaatcattttgagaagactctgatcatccccaagagttcttggcccaatcatgaaacatcattaggggtgagcgcagcatgtccataccagggaatgccctcctttttcagaaatgtgatatggcttaccaagaagaacgatgcatacccaacaataaagatgagctacaataataccaatagggaagatcttttgatactgtgggggattcatcattccaacaatgcagcagagcagacaaatctctataaaaacccaaccacctatgtttccgttgggacatcaacattaaatcagagattggtgccaaaaatagctactagatcccaagtaaacgggcaacgtggaagaatggatttcttctggacgattttaaaaccgaatgatgcaatccacttcgagagtaatggaaattttattgctccagaatatgcatacaaaattgtcaagaaaggggactcaacaattatgaaaagtgaaatggaatatggccactgcaacaccaaatgtcaaactccaataggggcgataaactctagtatgccattccacaatatacaccctctcactatcggggagtgccccaaatacgtgaaatcaaacaaattagtccttgcaactgggctcagaaatagtcctctaagagaaGGGagaagaagaaaaagaggactatttggagctatagcagggttcatagagggaggatggcaaggaatggtagatggttggtatgggtaccaccatagcaatgaacaggggagtgggtacgctgcagacagagaatccacccaaaaggcaatagatggagttaccaataaggtcaactcgataattgacaaaatgaacactcaatttgaggccgttggaagggagtttaataacttagaacggagaatagagaatttaaataagaaaatggaagacggattcctagatgtctggacttataatgctgaacttttagttctcatggaaaatgagagaactctagatttccatgattcaaatgtcaagaacctttatgacaaagtccgactacagcttagggataatgcaaaggagctgggtaatggttgtttcgagttctatcacaaatgtgataatgaatgtatggaaagtgtaagaaatgggacgtatgactacccccagtattcagaagaagcaagattaaaaagggaagaaataagcggagtgaaattggaatcaataggaacttaccaaatactgtcaatttattcaacagtggcgagttccctagcactggcaatcattgtggctggtctatctttatggatgtgctccaatgggtcgttacaatgcagaatttgcatt

>H5N2_A_chicken_Zhejiang_514135_2015

atggagaaaatagtgcttcttcttgcagtggttagccttgttaaaagtgatcagatttgcattggttaccatgcaaacaactcgacagagcaggttgacacgataatggaaaaaaacgtcactgttacacatgcccaagacatactggaaaagacacacaacgggaggctctgcgatctgaatggagtgaaacctctgattttaaaggattgtagtgtagctggatggcttcttgggaacccaatgtgcgacgagttcatcagagtgccggaatggtcttacatagtggagagggctaacccagccaatgacctctgttacccagggaacctcaatgactatgaagaactgaaacacctattgagcagaataaatcattttgagaagactctgatcatccccaagagttcttggcccaatcatgaaacatcattaggggtgagcgcagcatgtccataccagggaatgccctcctttttcagaaatgtggtatggcttaccaaaaagaacgatgcatacccaacaataaagatgaactacaataataccaatggggaagatcttttgatactgtgggggattcatcattccaacaatgcagcagagcagacaaatctctataaaaacccaaccacctatgtttccgttgggacatcaacattaaaccagagattggtgccaaaaatagctactagatcccaagtaaacgggcaacgtggaagaatggatttcttctggacaattttaaaaccgaatgatgcaatccacttcgagagtaatggaaattttattgctccagaatatgcatacaaaattgtcaagaaaggggactcaacaattatgaaaagtgaaatggaatatggccactgcaacaccaaatgtcaaactccaataggggcgataaactctagtatgccattccacaatatacaccctctcactatcggggagtgccccaaatacgtgaaatcaaacaaattagtccttgcgactgggctcagaaatagtcctctaagagaaGGGagaagaagaaaaagaggactatttggagctatagcagggttcatagagggaggatggcaaggaatggtagatggttggtatggataccaccatagcaatgaacaggggagtgggtacgctgcagacagagaatccacccaaaaggcaatagatggagttaccaataaggtcaactcgataattgacaaaatgaacactcaatttgaggccgttggaagggagtttaataacttagaacggagaatagagaatttaaataagaaaatggaagacggattcctagatgtctggacttacaatgccgaacttctagttctcatggaaaatgagagaactctagatttccatgattcaaatgtcaagaacctttatgataaagtccgactacagcttagggataatgcaaaggagctgggtaatggttgtttcgagttctatcacaaatgtgataatgaatgtatggaaagtgtaagaaatgggacgtatgactacccccagtattcagaagaagcaagattaaaaagggaagaaataagcggagtgaaattggaatcaataggaacttaccaaatactgtcaatttattcaacagtggcgagttccctagcactggcaatcattgtggctggtctatcgttatggatgtgctccaatgggtcgttacaatgcagaatttgcatt

>H5N6_A_duck_Vietnam_LBM758_2014

atggagaaaatagtgcttcttcttgcagtggttagccttgttaaaagtgatcagatttgcattggttaccatgcaaacaactcgacagagcaggttgacacgataatggaaaagaacgtcactgttacacatgcccaagacatactggaaaagacacacaacgggaggctctgcgatctgaatggagtgaaacctctgattttaaaggattgtagtgtagctggatggcttcttggaaacccaatgtgcgacgagttcatcagagtgccggaatggtcttacatagtggagagggctaacccagccaatgacctctgttacccagggaacctcaatgactatgaagaactgaaacacctattgagcagaataaatcattttgagaagactctgatcatccccaagagttcttggcccaatcatgaaacatcattaggggtgagcgcagcatgtccataccagggaatgccctcctttttcagaaatgtggtatggcttaccaagaagaacgatgcatacccaacaataaagatgagctacaataataccaatagggaagatcttttgatactgtgggggattcatcattccaacaatgcagcagagcagacaaatctctataaaaacccaaccacctatgtttccgttgggacatcaacattaaaccagagattggtgccaaaaatagctactagatcccaagtaaacgggcaacgtggaagaatggatttcttctggacaattttaaaaccgaatgatgcaatccacttcgagagtaatggaaattttattgctccagaatatgcatacaaaattgtcaagaaaggggactcaacaattatgaaaagtgaaatggaatatggccactgcaacaccaaatgtcaaactccaataggggcgataaactctagtatgccattccacaatatacaccctctcactatcggggagtgccccaaatacgtgaaatcaaacaaattagtccttgcgactgggctcagaaatagtcctctaagagaaGGGagaagaagaaaaagaggactatttggagctatagcagggttcatagagggaggatggcaaggaatggtagatggttggtatgggtaccaccatagcaatgaacaggggagtgggtacgctgcagacagagaatccacccaaaaggcaatagatggagttaccaataaggtcaactcgataattgacaaaatgaacactcaatttgaggccgttggaagggagtttaataaattagaacggagaatagagaatttaaataagaaaatggaagacggattcctagatgtctggacttacaatgctgaacttttagttctcatggaaaatgagagaactctagatttccatgattcaaatgtcaagaacctttatgacaaagtccgactacagcttagggataatgcaaaggagctgggtaatggttgtttcgagttctatcacaaatgtgataatgaatgtatggaaagtgtaagaaatgggacgtatgactacccccagtattcagaagaatcaagattaaagagggaagaaataagcggagtgaaattggaatcaataggaacttaccaaatactgtcaatttattcaacggtggcgagttccctagcactggcaatcattgtggctggtctatctttatggatgtgctccaatgggtcgttacaatgcagaatttgcatt

>H5N6_2344d_A_Guangzhou_39715_2014

atggagaaaatagtgcttcttcttgcagtggttagccttgttaaaagtgatcagatttgcattggttaccatgcaaacaactcgacagagcaggttgacacgataatggaaaaaaacgtcactgttacacatgcccaagacatactggaaaagacacacaacgggaggctctgcgatctgaatggagtgaaacctctgattttaaaggattgtagtgtagctggatggcttcttggaaacccaatgtgcgacgagttcatcagagtgccggaatggtcttacatagtggagagggctaacccagccaatgacctctgttacccagggaacctcaatgactatgaagaactgaaacacctattgagcagaataaatcattttgagaagactctgatcatccccaagagttcttggcccaatcatgaaacatcattaggggtgagcgcagcatgtccataccagggaatgccctcctttttcagaaatgtggtatggcttaccaagaagaacgatacatacccaacaataaagatgagctacaataataccaatagggaagatcttttgatactatgggggattcatcattccaacaatgcagcagagcagacaaatctctataaaaatccaaccacctatgtttccgttgggacagcaacattaaaccagagattggtgccaaaaatagctactagatcccaagtaaacgggcaacgtggaagaatggatttcttctggacaattttaaaaccgaatgatgcaatccacttcgagagtaatggaaattttattgctccagaatatgcatacaaaattgtcaagaaaggggactcaacaattatgaaaagtgaaatggaatatggccactgcaacaccaaatgtcaaactccaataggggcgataaactctagtatgccattccacaatatacaccctctcactatcggggagtgccccaaatacgtgaaatcaaacaaattagtccttgcgactgggctcagaaatagtcctctaagagaaGGGagaagaagaaaaagaggactatttggagctatagcagggtttatagagggaggatggcaaggaatggtagatggttggtatgggtaccaccatagcaatgaacaggggagtgggtacgctgcagacagagaatccacccaaaaggcaatagatggagttaccaataaggtcaactcgataattgacaaaatgaacactcaatttgaggccgttggaagggagtttaataacttagaacggagaatagagaacttaaataagaaaatggaagacggattcctagatgtctggacttataatgctgaacttttagttctcatggaaaatgagagaactctagatttccatgactcaaatgtcaagaacctttatgacaaagtccgactacagcttagggataatgcaaaggagctgggtaatggttgtttcgagttctatcacaaatgtgataatgaatgtatggaaagtgtaagaaatgggacgtatgactacccccagtattcagaagaagcaagattaaaaagggaagaaataagcggagtgaaattggaatcaataggaacttaccaaatactgtcaatttattcaacagtggcgagttccctagcactggcaatcattgtggctggtctatctttatggatgtgctccaatgggtcgttacaatgcagaatttgcatt

>H5N6_A_Syrrhaptes_paradoxus_Guangdong_ZH283_2015

atggagaaaatagtgcttcttcttgcagtggttagccttgttaaaagcgatcagatttgcattggttaccatgcaaacaactcgacagagcaggttgacacgataatggaaaaaaacgtcactgttacacatgcccaagacatactggaaaagacacacaacgggaggctctgcgatctgaatggagtgaaacctctgattttaaaggattgtagtgtagctgggtggcttcttggaaacccaatgtgcgacgagttcatcagagtaccggaatggtcttacatagtggagagggctaacccagccaatgacctctgttacccagggaacctcaatgactatgaagaactgaaacacctattgagcagaataaatcattttgagaaaactctgatcatccccaagagttcttggcccaatcatgaaacatcattaggggtgagcgcagcatgtccataccagggagtgccctcttttttcagaaatgtggtatggcttaccaagaagaacgatgcatacccaacaataaagatgagctacaataataccaatggggaagatcttttgatattgtgggggattcatcattccaacaatgcagcagagcagacaaatctctataaaaacccaaccacctatgtttccgttgggacatcaacattaaaccagagattggtgccaaaaatagctactagatcccaagtaaacgggcaacgtggaagaatggatttcttctggacgattttaaaaccgaatgatgcaatccacttcgagagtaatggaaattttattgctccagaatatgcatacaaaattgtcaagaaaggagactcaacaattatgaaaagtgaaatggaatatggccactgcaacaccaaatgtcaaactccaataggggcgataaactctagtatgccattccacaatatacaccctctcactatcggggagtgccccaaatacgtgaaatcaaacaaattagtccttgcgactgggctcagaaatagtcctctaagagaaGGGagaagaaggaaaagaggactatttggagctatagcagggtttatagagggaggatggcaaggaatggtagatggttggtatgggtaccaccatagcaatgaacaggggagtgggtacgctgcagacagagaatccacccaaaaggcgatagatggagttaccaataaggtcaactcgataattgacaaaatgaacactcaatttgaggccgttggaagggaatttaataacttagaacggagaatagagaatttaaataagaaaatggaagacggattcctagatgtctggacttataatgctgaacttttagttcttatggaaaatgagagaactctagatttccatgactcaaatgtcaagaacctttatgacaaagtccgactacagcttagggataatgcaaaggagctgggtaatggttgtttcgagttctatcacaaatgtgataatgaatgtatggaaagtgtaagaaatgggacgtatgactacccccagtattcagaagaagcaagattaaaaagggaagaaataagcggagtgaaattggaatcaataggaacttaccaaatactgtcaatttattcaacagtggcgagttccctagcactggcaatcattgtggctggtctatctttatggatgtgctccaatgggtcgttacaatgcagaatttgcatt

>H5N6_2344d_A_Hubei_29578_2016

atggagaaaatagtgcttcttcttgcagtggttagccttgtcaaaagtgatcagatttgcattggttaccatgcaaacaactcgactgagcaggttgacacgataatggaaaaaaacgtcactgttacacatgctcaagacatactggaaaagacacacaacgggaagctctgcgatctgaatggagtgaaacctctgattttaaaggattgtagtgtagctggatggcttcttggaaacccaatgtgcgacgagttcatcagagtgccggaatggtcttacatagtggaaagggctaacccagccaatgacctctgttacccagggaacctcaatgactatgaagaactgaaacacctattgagcagaataaatcatttcgagaagactctgatcatccccaagagttcttggcccaatcatGGGacatcatcaggggtgagcgcagcatgtccatacctgggaaagccctcctttttcagaaatgtggtatggcttaccaagaagaacgatgcatacccaacaataaaaatgagttacaataacaccaatagggaagatcttttgatactgtgggggattcatcattccaataatgcagaagagcagacaaatctctataaaaacccaaccacttatgtttccgttgggacatcaacattaaaccagagagtggtgccaaaaatagctactagatcccaagtaaacgggcaaagtggaagaatggatttcttctggacaattttaaaaccggatgatgcaatccacttcgagagtaatggaaattttattgctccagaatatgcatacaaaattgtcaagaaaggggactcaacaattatgaaaagtgaaatggaatatggcaattgcaacaccaaatgtcaaactccaataggggcgataaactctagtatgccattccacaatatacaccctctcactatcggggagtgccccaaatacgtgaaatcaaacaaattagtccttgcgactgggctcagaaatagtcctctaagagaaGGGaggagaagaaaaagaggactatttggggccatagcagggtttatagagggaggatggcaagggatggtagatggttggtatgggtaccaccatagcaatgaacaagggagtgggtatgctgcagacagagaatccacccaaaaggcaatagatggagttaccaataaggtcaactcgataattgacaaaatgaacactcaatttgaggccgttggaagggaatttaataacttagaacggagaatagagaatttaaataagaaaatggaagacggattcctagatgtctggacttataatgctgaacttttagttctcatggaaaatgagagaactctagatttccatgactcaaatgtcaagaacctttatgacaaagtccgactacagcttagggataatgcaaaggagctgggtaatggttgtttcgagttctatcacaaatgtgataatgaatgtatggaaagtgtgagaaatgggacgtatgactacccccagtattcagaagaagcaagattaaaaagggaagaaataagcggagtgaaattggaatcaataggaacttaccaaatactgtcaatttattcaacagtggcgggttccctagcactggcaatcattgtggctggtctatctttatggatgtgctccaatgggtcgttacaatgcagaatttgcatt

>H5N6_A_chicken_Ha_Tinh_514VTC_2021

atggagaaagtagtatttcttctttcaatggttggccttgttaaaagtgatcagatttgcattggttaccatgcaaacaactcaacagagcaggttgacacaataatggaaaaaaacgtcactgttacgcatgcccaagacatactggaaaagacacacaacgggaagctctgcgatctgaatggagtgaaacctctgattttaaagaattgtagtgtagctggatggcttcttggaaacccaatgtgcgacgagttcatcagcgtaccggaatggtcttacatagtggagagaactgacccagccaatgatctctgttacccagggaccctcaatgactatgaagaactgaaacacctattgagcagaataaatcattttgagaagactcagatcatccccaaaaggtcttggtccaatcatGGGacatcatcaggagtgagcgcagcatgtccatacgcaggggtggcctccttttttagaaatgtggtatggcttaccaagaagaatgatgcatacccaacaataaagaagagctacaataataccaacaaagaagatcttttgatactgtgggggatccatcattccaacagtgcagaggagcaggcagatctctacaagaacccaaccacctatgtttccgttgggacatcaacactaaaccagaagttggtgccaaaaatagctactagatcccaagtaaatgggcaacgtggaagaatggatttcttctggacaatcttaagacggaatgatgcaatccacttcgagagtaatggaaattttatcgctccagaatatgcatacaaaattatcaagacaggagactcaacaattatgaaaagtgaaatagaatatggcaactgcaacaccaagtgtcaaactccaataggggcgataaactctagtatgccattccacaatatacatcctctcaccatcggggagtgccccaaatatgtgaaatcaaacaaattagttcttgcgactgggctcagaaatagtcccctaagagaaGGGagaagaagaaaaagaggactgtttggagctatagcagggtttatagagggaggatggcaaggaatgatagatggttggtatgggtaccatcatagtaatgaacaggggagtgggtacgctgcagacagagaatccacccaaaaggcaatagatggagtcaccaacaaggtcaattcgataattgacaaaatgaacactcaatttgaggctgttggaagagaatttaatagcttagagcggagaatagagaatttaaataagaaaatggaagacggattcctagatgtctggacttataatgctgaacttttagttctcatggaaaatgagagaactctagatttccatgattcaaatgtcaagaacctttatgacaaagtccgactacagcttagggataatgcaaaggagctgggtaatggttgtttcgagttctatcacaaatgtgataatgaatgtatggaaagtgtgagaaatggaacgtacgactaccctcagtactcagaagaagcaagattaaaaagggaagaaataagtggagtgaaattggaatcaatgggaacttaccaaatactgtcaatttattccacagtggcgagcaccctagtactggcaatcattgtggctggtctatctttatggatgtgctccaatgggtcgttacaatgcagaatttgcatt

>H5N6_A_chicken_Nghe_An_7007VTC_2020

atggagaaaatggtacttcttcttttaatggttggccttgttaaaagtgatcagatttgcattggttaccatgcaaacaactcaacagagcaggttgacacaataatggaaaaaaacgtcactgttacgcatgcccaagacatactggaaaagacacacaacgggaagctctgcgatctgaatggggtgaaacctctgattttaaagaattgtagtgtagctggatggcttcttggaaacccaatgtgcgacgagttcatcagcgtaccggaatggtcttacatagtggagagagctgacccagccaatgatctctgttacccagggaccctcaatgactatgaagaactgaaacacctattgagcagaataaatcattttgagaagactcagatcatccccaagaggtcttggtccaatcatGGGacatcatcaggagtgagcgcagcatgtccatacgcaggggtggcctccttttttagaaatgtggtatggcttaccaagaagaatgatgcatacccaacaataaagaagagctacaataataccaacaaagaagatcttttgatactgtgggggatccatcattccaacagtgcagaggagcaggcagatctctacaagaacccaaccacctatgtttccgttgggacgtcaacactaaaccagaagttggtgccaaaaatagctactagatctcaagtaaatgggcaacgtggaagaatggatttcttctggacaatcttaagacggaatgatgctatccacttcgagagtaatgggaattttatcgctccagaatatgcatacaaaattatcaagacaggagactcaacaattatgaaaagtgaaatagaatatggcaactgcaacaccaagtgtcaaactccaataggggcgataaactctagtatgccattccacaatatacatcctctcactatcggggagtgccccaaatatgtgaaatcaaacaaattagttcttgcgactgggctcagaaatagtcctctaagagaaGGGagaagaagaaaaagaggactgtttggagctatagcagggtttatagagggaggatggcaaggaatggtagacggttggtatgggtaccaccatagtaatgaacaggggagtgggtacgctgcagacagagaatccacccaaaaggcaatagatggagtcaccaacaaggtcaactcgataatcgacaaaatgaacactcaatttgaggccgttggaagagaatttaatagcttagaacggagaatagagaatttaaataagaaaatggaagacggattcctagatgtctggacttataatgctgaacttttagttctcatggaaaatgagagaactctagatttccatgattcaaatgtcaagaacctttatgacaaagtccgactacagcttagggataatgcaaaggagctgggtaatggttgtttcgagttctatcacaaatgtgataatgaatgtatggaaagtgtgagaaatggaacgtacgactaccctcagtactcagaagaagcaagattaaaaagggaagaaataagtggagtgaaattggaatcaatgggaacttaccaaatactgtcaatttattccacagtggcgagctccctagtgctggcaatcattgtggctggtctgtctttatggatgtgctccaatgggtcgttacaatgcagaatttgcatt

>H5N6_A_chicken_Quang_Tri_V4S4VTC_2020

atggagaaaatagtacttcttctttcaatggttggccttgttaaaagtgatcagatttgcattggttaccatgcaaacaactcaacagagcaggttgacacaataatggaaaaaaacgtcactgttacgcatgcccaagacatactggaaaagacacacaacgggaagctctgcgatctgaatggagtgaaacctctgattttaaagaattgtagtgtagctggatggcttcttggaaacccaatgtgcgacgagttcatcagcgtaccggaatggtcttacatagtggagagggctgacccagccaatgatctctgttacccagggaccctcaatgactatgaagaactgaaacacctattgagcagaataaatcattttgagaagactcagatcatccccaagaggtcttggtccaatcatGGGacatcatcaggagtgagcgcagcatgtccatacgcaggagtggcctccttttttagaaatgtggtatggcttaccaagaagaatgatgcatacccaacaataaagaagagctacaataataccaacaaagaagatcttttgatactgtgggggatccatcattccaacagtgcagaggagcaggcagatctctacaagaacccagccacttatgtttccgttgggacatcaacactaaaccagaagttggtgccaaaaatagctactagatcccaagtaaatgggcaacgtggaagaatggatttcttttggacaatcttaagacggaatgatgcaatccacttcgaaagtaatgggaattttatcgctccagaatatgcatacaaaattatcaagacaggagactcaacaattatgaaaagtgaaatagaatatggcaactgcaacaccaagtgtcaaactccaataggggcgataaactctagtatgccattccacaatatacatcctctcactatcggggagtgccccaaatatgtgaaatcaaacaaattagttcttgcgactgggctcagaaatagtcctctaagagaaGGGagaagaagaaaaagagggctgtttggagctatagcagggtttatagaaggaggatggcaaggaatggtagacggttggtatgggtaccaccatagtaatgaacaggggagtgggtacgctgcagacagagaatccacccaaaaggcaatagatggagtcaccaacaaagtcaactcgataattgacaaaatgaacactcaatttgaggccgttggaagagaatttaatagcttagaacggagaatagagaatttaaataagaaaatggaagacggattcctagatgtttggacttataatgctgaacttttagttctcatggaaaatgagagaactctagatttccatgattcaaatgtcaggaacctttatgacaaagtccgactacagcttagggataacgcaaaggagctgggcaatggttgtttcgagttctatcacaaatgtgataatgaatgtatggaaagtgtgagaaatggaacgtacgactaccctcagtactcagaagaagcaagattaaaaagagaagaaataagtggagtgaaattggaatcaatggggacttaccaaatactgtcaatttattccaccgtggcgagctctctagtactggcaatcattgtggctggtctatctttatggatgtgctccaatgggtcgttacaatgcagaatttgcatt

>H5N6_A_Anhui_2021_00011_2020

atggagaaaatagtacttcttctttcagtggttggccttgtcaaaagtgatcagatctgcattggttaccatgcaaacaactcgacagagcaggttgacacaataatggaaaaaaacgtcactgttacgcatgcccaagacatactggaaaagacacacaacgggaagctctgtgatctgaatggagtgaaacctctgattttaaagaattgtagtgtggctggatggcttcttggaaacccaatgtgcgatgagttcatcaacgtaccggaatggtcttatatagtggagagggccaacccagccaatgacctctgttacccagggaacctcaatgactatgaagaactgaaacacctattgagcagaataaatcattttgagaagactcagatcatccccaaggagtcttggtccaatcatacaacatcaGGGggagtgagcgcagcatgtccataccaaggagtggcctccttttttagaaatgtggtatggcttaccaagaagaatgatgcatacccgacaataaagaagagctacaataataccaataaagaggaccttttgatactatggggaattcatcattccaacagtgtagaggagcagacagatctctacaagaacccaaccacctatgtttccgttgggacatcaacgctaaatcagaggttggtgccaaaaatagctaccagatcccaagtaaatgggcaacgtggaagaatggatttcttctggacaattttaagaccgaatgatgcaatccacttcgagagtaatggaaattttatcgctccagaatatgcatacaaaatcatcaagacaggagactcaacaattatgaaaagtgaaatagaatatggcgactgcaacagcaagtgtcaaactccgataggggcgataaactctagtatgccattccacaatatacaccctctcactatcggggagtgccccaaatatgtgaaatcaaacaaattagtccttgcgactgggctcagaaatagtcctctaagggaaGGGagaagaaggaaaagaggactgtttggagctatagcaggatttatagagggaggatggcaaggaatggtagatggttggtatgggtaccatcatagtaatgaacaggggagtgggtacgctgcagacagagaatccactcaaaaggcaatagatggggtcaccaacaaggtcaactcgataatagacaaaatgaacactcaatttgaggccgttggaagagaatttaacagcttagaacggagaatagagaatttaaataagaaaatggaagacggattcctagatgtctggacttataatgctgaacttttagttctcatggaaaatgagagaactctagattttcatgactcaaatgtcaagaacctttatgacaaagtccgactacagcttagggataatgcaaaggagctgggtaatggttgtttcgaattctatcacaaatgtgataatgaatgtatggaaagtgtaagaaatggaacgtatgactacccccagtactcagaggaggcaagattaaaaagggaagaaataagcggagtgaaattggaaacaataggaactttccaaatactgtcaatttattcaacagtggcgagttccctagtactggcaatcattatggctggtctatctttatggatgtgctccaatgggtcgttacaatgcagaatttgcatt

>H5N6_A_Chongqing_00013_2021

atgaagaaaatagtacttcttctttcagtggttggccttgttaaaagtgatcagatctgcattggttaccatgcaaacaactcgacagagcaggttgacacaataatggaaaaaaacgtcactgttacgcatgcccaagacatactggaaaagacacacaacgggaagctctgtgatctgaatggagtgaaacctctgattttaaagaattgtagtgtggctggatggcttcttggaaacccaatgtgcgatgagttcatcagcgtaccggaatggtcttatatagtggagagggctaacccagccaatgacctctgttacccagggaacctcaatgactatgaagaactgaaacacctattgagcagaataaatcattttgagaagactcagatcatccccaaggagtcttggtccaatcatacaacatcaGGGggagtgagcgcagcatgtccataccaaggagtggcctccttttttagaaatgtggtatggcttaccaagaagaatgatgcatacccgacaataaagaagagctacaataataccaataaagaggaccttctgatactatggggaattcatcactccaacagtgtagaggagcagacagttctctacaagaacccaaccacctatgtttccgttgggacatcaacactaaatcagaggttggtgccaaaaatagctaccagatcccaagtaaatgggcaacgtggaagaatggatttcttctggacaattttaagaccgaatgatgcaatccacttcgagagtaatggaaattttatcgctccagaatatgcatacaaaatcatcaagacaggagactcaacaattatgaaaagtgaaatagaatatggcgactgcaacagcaagtgtcaaactccgataggggcgataaactctagtatgccattccacaacatacaccctctcactatcggggagtgccccaaatatgtgaaatcaaacaaattagtccttgcgactgggctcagaaatagtcctctaagggaaGGGagaagaaggaaaagaggactgtttggagctatagcaggatttatagagggaggatggcaaggaatgatagatggttggtatgggtaccatcatagtaatgaacaggggagtgggtacgctgcagacagagaatccactcaaaaggcaatagatggggtcaccaacaaggtcaactcgataatagacaaaatgaacactcaatttgaggccgttggaagagaatttaacagcttagaacggagaatagagaatttaaataagaaaatggaagacggattcctagatgtctggacttataatgctgaacttttagttctcatggaaaatgagagaactctagatttccatgactcaaatgtcaagaacctttatgacaaagtccgactacagcttagggataatgcaaaggaactgggtaatggttgtttcgaattctatcacaaatgtgataatgaatgtatggaaagtgtaagaaatggaacgtataactacccccagtactcagaggaggcaagattaaaaagggaagaaataagcggagtgaaattggaaacaataggaactttccaaatactgtcaatttattcaacagtggcgagttccctagtactggcaatcattgtggctggtctatctttatggatgtgctccaatgggtcgttacaatgcagaatttgcatt

>H5N6_A_Whooper_swan_Mongolia_25_2020

atggagaaaatagtacttcttttttcagtggttagccttgttaaaagtgatcagatttgcattggttaccatgcaaacaactcgacagagcaggttgacacaataatggaaaaaaacgtcactgttacgcatgcccaagacatactggaaaagacacacaacgggaagctctgcgatctgaatggagtaaaaccactgattttaaaggattgtagtgtagctggatggcttcttggaaacccaatgtgcgacgagttcatcagtgtaccggaatggtcttatatagtggagagggctaacccagccaatgacctctgttacccggggaacctcaatgactatgaagagctgaaacacctattgagcagaataaatcattttgagaagactcagatcatccccaagaggtcttggtccaatcatGGGacatcatcaggggtgagcgcagcatgtccataccaaggagtggcctccttttttagaaatgtggtatggcttaccaagaagaatgatgcatacccgacaataaagatgagctacaataataccaataaagaagatcttttgatactgtggggaatccatcattccaacagtgcagaggagcagataagtctctacaagaacccaaccacctatgtttccgttgggacatcaacattaaaccagaggttggtgccaaaaatagctactagatcccaagtaaatgggcaacgtggaagaatggatttcttctggacaattttaagaccgagtgatgcaatccacttcgagagtaatggaaattttattgctccagaatatgcatacaaaattatcaagacaggagactcaacaattatgaaaagtgaattagaatatggcaactgcaacaccaagtgtcaaactccaataggggcgataaactctagtatgccattccacaatatacatcctctcaccatcggggagtgccccaaatatgtaaaatcaaacaaattagtccttgcgactgggctcagaaatagtcctctaagagaaGGGagaagaagaaaaagaggactgtttggagccatagcagggtttatagagggaggatggcaaggaatggtagatggttggtatgggtaccaccatagtaatgagcaggggagtggatacgctgcagacagagaatccacccaaaaggcaatagatggagtcaccaacaaggtcaattcgataattgacaaaatgaacactcaatttgaggccgttggaagagaatttaacagcttagaacggagaatagagaatttaaacaagaaaatggaagacggattcctagatgtctggacttataatgctgaacttttggttctcatggaaaatgagagaactctagatttccatgactcaaatgtcaagaacctttatgacaaagtccgactacagcttagggataatgcaaaggagctgggtaatggttgtttcgagttctatcacaaatgtgataatgaatgtatggaaagtgtaagaaatggaacgtatgactacccccagtactcagaagaagcaagattaaaaagggaggaaataagcggagtgaaattggaatcaataggaacttaccaaatactgtcaatttattcaacagtggcgagttccctagtactggcaatcattgtggctggtctatctttatggatgtgctccaatgggtcgttacaatgcagaatttgcatt

>H5N6_A_Whooper_Swan_Khuvsgul_4_2020

atggagaaaatagtacttcttttttcagtggttagccttgttaaaagtgatcagatttgcattggttaccatgcaaacaactcgacagagcaggttgacacaataatggaaaaaaacgtcactgttacgcatgcccaagacatactggaaaagacacacaacgggaagctctgcgatctgaatggagtaaaaccactgattttaaaggattgtagtgtagctggatggcttcttggaaacccaatgtgcgacgagttcatcagtgtgccggaatggtcttatatagtggagagggctaacccagccaatgacctctgttacccggggaacctcaatgactatgaagagctgaaacacctattgagcagaataaatcattttgagaagactcagatcatccccaagaggtcttggtccaatcatGGGacatcatcaggggtgagcgcagcatgtccataccaaggagtggcctccttttttagaaatgtggtatggcttaccaagaagaatgatgcatacccgacaataaagatgagctacaataataccaataaagaagatcttttgatactgtggggaatccatcattccaacagtgcagaggagcagataagtctctacaagaacccaaccacctatgtttccgttgggacatcaacattaaaccagaggttggtgccaaaaatagctactagatcccaagtaaatgggcaacgtggaagaatggatttcttctggacaattttaagaccgagtgatgcaatccacttcgagagtaatggaaattttattgctccagaatatgcatacaaaattatcaagacaggagactcaacaattatgaaaagtgaattagaatatggcaactgcaacaccaagtgtcaaactccaataggggcgataaactctagtatgccattccacaatatacatcctctcaccatcggggagtgccccaaatatgtgaaatcaaacaaattagtcctcgcgactgggctcagaaatagtcctctaagagaaGGGagaagaagaaaaagaggactgtttggagccatagcagggtttatagagggaggatggcaaggaatggtagatggttggtatgggtaccaccatagtaatgagcaggggagtggatacgctgcagacagagaatccactcaaaaggcaatagatggagtcaccaacaaggtcaattcaataattgacaaaatgaacactcaatttgaggccgttggaagagaatttaacagcttagaacggagaatagagaatttaaacaagaaaatggaagacggattcctagatgtctggacttataatgctgaacttttggttctcatggaaaatgagagaactctagatttccatgattcaaatgtcaagaacctttatgacaaagtccgactacagcttagggataatgcaaaggagctgggtaatggttgtttcgagttctatcacaaatgtgataatgaatgtatggaaagtgtaagaaatggaacgtatgactacccccagtactcagaagaagcaagattaaaaagggaggaaataagcggagtgaaattggaatcaataggaacttaccaaatactgtcaatttattcaacagtggcgagttccctagtactggcaatcattgtggctggtctatctttatggatgtgctccaatgggtcgttacaatgcagaatttgcatt

>H5N6_A_duck_Bangladesh_44469_2020

atggagaaaacaatacttcttttttcagtggttagccttgttaaaagtgatcagatttgcattggttaccatgcaaacaactcgacagagcaggttgacacaataatggaaaaaaacgtcactgttacgcatgcccaagacatactggaaaagacacacaacgggaagctctgcgatctgaatggagtaaaaccactgattttaaaggattgtagtgtagctggatggcttcttggaaacccaatgtgcgacgagttcatcagtgtgccggaatggtcttatatagtggagagggctaacccagccaatgacctctgttacccggggaacctcaatgactatgaagagctgaaacacctattgagcagaataaatcattttgagaagactcagatcatccccaagaggtcttggtccaatcatGGGacatcatcaggggtgagcgcagcatgtccataccaaggagtggcctccttttttagaaatgtggtatggcttaccaagaagaatgatgcatacccgacaataaagatgagctacaataataccaataaagaagatcttttgatactgtggggaatccatcattccaacagtgcagaggagcagataagtctctacaagaacccaaccacctatgtttccgttgggacatcaacattaaaccagaggttggtgccaaaaatagctactagatcccacgtaaatgggcaacgtggaagaatggatttcttctggacaattttaagaccgagtgatgcaatccacttcgagagtaatggaaattttattgctccagaatatgcatacaaaattatcaagacaggagactcaacaattatgaaaagtgaattagaatatggcaactgcaacaccaagtgtcaaactccaataggggcgataaactctagtatgccattccacaatatacatcctctcaccatcggggagtgccccaaatatgtgaaatcaaacaaattagtccttgcgactgggctcagaaatagtcctctaagagaaGGGagaagaagaaaaagaggactgtttggagccatagcagggtttatagagggaggatggcaagggatggtagatggttggtatgggtaccaccatagtaatgagcaggggagtggatacgctgcagacagagaatccacccaaaaggcaatagatggagtcaccaacaaggtcaattcgataattgacaaaatgaacactcaatttgaggccgttggaagagaatttaacagcttagaacggagaatagagaatttaaacaagaaaatggaagacggattcctagatgtctggacttataatgctgaacttttggttctcatggaaaatgagagaactctagatttccatgactcaaatgtcaagaacctttatgacaaagtccgactacagcttagggataatgcaaaggagctgggtaatggttgtttcgagttctatcacaaatgtgataatgaatgtatggaaagtgtaagaaatggaacgtatgactacccccagtactcagaagaagcaagattaaaaagggaggaaataagcggagtgaaattggaatcaataggaacttaccaaatactgtcaatttattcaacagtggcgagttccctagtactggcaatcattgtggctggtctatctttatggatgtgctccaatgggtcgttacaatgcagaatttgcatt

>H5N6_2344d_A_Jiangsu_1_2020

atggagaaaatagtacttattctttcagtggttagccttgttaaaagtgatcagatttgcattggctaccatgcaaacaactcgacagagcaggttgacacaataatggaaaaaaacgtcactgttacgcatgcccaagacatactggaaaagacacacaacgggaagctctgcgatctgaatggggtgaaacctctgattttgaaggattgtagtgtagctggatggcttcttggaaacccaatgtgcgacgagttcatcagagtgccggaatggtcttatatagtggagaaggctaacccagccaatgacctctgctacccagggaacctcaatgactatgaagaactgaaacacctattgagcagaataaatcactttgagaagactcagatcatccccaagagttcttggcccaatcatGGGacatcatcaggggtgagcgcagcttgtccataccaaggagtgccctccttctttagaaatgtggtatggcttaccaagaagaatgatgcatacccgacaataaagataagctacaataataccaatagtgaagatcttttgatactgtggggaatccatcattccaacaatgcagaagagcagacaaaactctacaagaacccaaccacctatatttccgttgggacatcaacattaaaccagaggttggtgccaaaaatagctactagatcccaagtaaatgggcaaggtggaagaatggatttcttctggacaattttaagaccgaatgatgtaatccacttcgagagtaatggaaattttatcgctccagaatatgcatacaaaattgtcaagacaggagactcaacaattatgaaaagtgaaatagaatatggcaactgcgacaccaagtgtcaaactccaataggggcgataaactctagtatgccattccacaatatacaccctctcactatcggggagtgccccaaatatgtgaaatcaaacaaattaatccttgcgactgggctcagaaacagtcctctaagagaaGGGagaagaagaaaaagaggactgtttggagctatagcaggatttatagagggaggatggcaaggaatggtagatggttggtatgggtaccaccatagtaatgaacaggggagtgggtacgctgcagacagagaatccacccaaaaggcaatagatggagtcaccaacaaggtcaactcgataattgacaaaatgaacactcaatttgaggccgttggaagagaatttaatagcttagaacggagaatagagaatttgaataagaaaatggaagacggattcctagatgtctggacttataatgctgaacttttagttctcatggaaaatgagagaactctagatttccatgactcaaatgtcaagaacctttatgacaaagtccgactacagcttagggataatgcaaaggaactgggtaatggttgtttcgagttctatcacaaatgtgataatgaatgtatggaaagtgtaagaaatggaacgtatgactacccccagtactcagaagaagcaagattaaaaagggaagaaataagcggaataaaattggaatcaataggaacttaccaaatactgtcaatttattcaacagtggcgagttccctagcactggcaatcattgtggctggtctatctttatggatgtgctcaaatgggtcgttacaatgcagaatttgcatt

>H5N6_A_goose_Guangdong_QY01_2016

atggggaaaattgtacttcttctttcagtggttagccttgttaaaagtgatcagatttgcattggttaccatgcaaacaactcaacagagcaggttgacacaataatggaaaaaaacgtcactgttacacatgcccaagacatactggaaaagacacacaacgggaaactctgcgatctgaatggagtgaaacctctgattttaaaggattgtagtgtagctggatggcttcttggaaacccaatgtgcgacgagttcatcagagtgccggaatggtcttatatagtggagagggctaacccagccaatgacctctgttacccagggaacctcaatgactatgaagaactgaaacacctattgagcagaataaatcattttgagaagactctgatcatccccaagagttcttggcccaatcatGGGacatcatcaggggtgagcgcagcatgtccataccagggaatgccctccttttttagaaatgtggtatggcttaccaagaagaatgatgcatacccgacaataaagatgagctacaataataccaatagagaagatcttttgatactgtggggaattcatcattccaacaatgcagaagagcagacaaatctctacaaaaacccaaccacctatgtttccgttgggacatcaacattaaaccagaggtcggtgccaaaaatagctactagatcccaagtaaatgggcaacgtggaagaatggatttcttctggacaattttaagaccgaatgatgcaatccacttcgaaagtaatggaaattttattgctccagaatatgcatacaaaattatcaagacaggagactcaacaattatgaaaagtgaaatggaatatggcaactgcaacaccaagtgtcaaactccaataggggcgataaactctagtatgccattccacaatatacatcctctaactatcggggagtgccccaaatacgtgaaatcaaacaaattagtccttgcgactgggctcagaaatagtcctctaagagaaGGGagaagaagaaaaagaggactatttggagctatagcagggtttatagagggaggatggcaaggaatggtagatggttggtatgggtaccaccatagtaatgaacaggggagtgggtacgctgcagacagagaatccacccaaaaggcaatagatggagttaccaacaaggtcaactcgataattgacaaaatgaacactcaatttgaggccgttggaagagaatttaataatttagaacgaagaatagagaatttaaataagaaaatggaagacggattcctagatgtctggacttataatgctgaacttctagttctcatggaaaatgagagaactctagatttccatgactcaaatgtcaagaacctttatgacaaagtccgactacagcttagggataatgcaaaggagctgggtaatggttgtttcgagttctatcacaaatgtgataatgaatgtatggaaagtgtaagaaatgggacgtatgactacccccagtactcagaagaagcaagattaaaaagggaagaaataagcggagtgaaattggaatcaataggaacttaccaaatactgtcaatttattcaacagtggcgagttccctagcactggcaatcattgtggctggtctatctttatggatgtgctccaatgggtcgttacaatgcagaatttgcatt

>H5N6_A_chicken_Ganzhou_GZ27_2015

atggagaaaatagtacttcttctttcagtggttagccttgttaaaagtgatcagatttgcattggttaccatgcaaacaactcgacagagcaggttgacacaataatggaaaaaaacgtcactgttacacatgcccaagacatactggaaaagacacacaacgggaagctctgcgatctgaatggagtgaaacctctgattttaaaggattgtagtgtagctggatggcttcttggaaacccaatgtgcgacgagttcatcagagtgccggaatggtcttatatagtggagagggctaacccagccaatgacctctgttacccagggaacctcaatgactatgaagaactgaaacacctattgagcagaataaatcattttgagaagactctgatcatccccaagagttcttggcccaatcatGGGacatcatcaggggtgagcgcagcatgtccataccagggaatgccctccttttttagaaatgtggtatggcttaccaagaagaatgatgcatacccgacaataaagatgagctacaataataccaatagagaagatcttttgatactgtggggaattcatcattccaacaatgcagaagagcagacaaatctctacaaaaacccaaccacctatgtttccgttgggacatcaacattaaaccagaggttggtgccaaaaatagctactagatcccaagtaaatgggcaacgtggaagaatggatttcttctggacaattttaagaccgaatgatgcaatccacttcgagagtaatggaaattttattgctccagaatatgcatacaaaattatcaagacaggggactcaacaattatgaaaagtgaaatggaatatggcaactgcaacaccaagtgtcaaactccaataggggcgataaactctagtatgccattccacaatatacatcctctcactatcggggagtgccccaaatacgtgaaatcaaacaaattagtccttgcgactgggctcagaaatagtcctctaagagaaGGGagaagaagaaaaagaggactatttggagctatagcagggtttatagagggaggatggcaaggaatggtagatggttggtatgggtaccaccatagcaatgaacaggggagtgggtacgctgcagacagagaatccacccaaaaggcaatagatggagttaccaacaaggtcaactcgataattgacaaaatgaacactcaatttgaggccgttggaagagaatttaataacttagaacggagaatagagaatttaaataagaaaatggaagacggattcctagatgtctggacttataatgctgaacttttagttctcatggaaaatgagagaactctagatttccatgactcaaatgtcaagaacctttatgacaaagtccgactacagcttagggataatgcaaaggagctgggtaatggttgtttcgagttctatcacaaatgtgataatgaatgtatggaaagtgtaagaaatgggacgtatgactacccccagtactcagaagaagcaagattaaaaagggaagaaataagcggagtgaaattggaatcaataggaacttaccaaatactgtcaatttattcaacagtggcgagttccctagcactggcaatcattgtggctggtctatctttatggatgtgctccaatgggtcgttacaatgcagaatttgcatt

>H5N6_A_Pavo_cristatus_Jiangxi_JA1_2016

atggagaaaatagtgcttcttctttcagtggttagccttgttaaaagtgatcagatttgcattggttaccatgcaaacaactcgacagagcaggttgacacaataatggaaaaaaacgtcactgttacacatgcccaagacatactggaaaagacacacaacgggaagctctgcgatctgaatggagtgaaacctctgattttaaaggattgtagtgtagctggatggcttcttggaaacccaatgtgcgacgagttcatcagagtgccggaatggtcttatatagtggagagggctaacccagccaatgacctctgttacccagggaacctcaatgactatgaagaactgaaacacctattgagcagaataaatcattttgagaagactctgatcatccccaagagttcttggcccaatcatGGGacatcatcaggggtgagcgcagcatgtccataccagggaatgccctccttttttagaaatgtggtatggcttaccaagaagaatgatgcatacccgacaataaagatgagctacaataataccaatagagaagatctcttgatactgtggggaattcatcattccaacaatgcagaagagcagacaaatctctacaaaaacccaaccacctatgtttccgttgggacatcaacattaaaccagaggttggtgccaaaaatagctactagatcccaagtaaatgggcaacgtggaagaatggatttcttctggacaattttaagaccgaatgatgcaatccacttcgagagtaatggaaattttattgctccagaatatgcatacaaaattgtcaagacaggggactcaacaattatgaaaagtgaaatggaatatggcaactgcaacgccaagtgtcaaactccaataggggcgataaactctagtatgccattccacaatatacatcctctcactatcggggagtgccccaaatacgtgaaatcaaacaaattagtccttgcgactgggctcagaaatagtcctctaagagaaGGGagaagaagaaaaagaggactatttggagctatagcagggtttatagagggaggatggcaaggaatggtagatggttggtatgggtaccaccatagtaatgaacaggggagtgggtacgctgcagacagagaatccacccaaaaggcaatagatggagttaccaacaaggtcaactcgataattgacaaaatgaacactcaatttgaggccgttggaagagaatttaataacttagaacggagaatagagaatttaaataagaaaatggaagacggattcctagatgtctggacttataatgctgaacttttagttctcatggaaaatgagagaactctagatttccatgactcaaatgtcaagaacctttatgacaaagtccgactacagcttagggataatgcaaaggagctgggtaatggttgtttcgagttctatcacaaatgtgataatgaatgtatggaaagtgtaagaaatgggacgtatgactacccccagtactcagaagaagcaagattaaaaagggaagaaataagcggagtgaaattggaatcaataggaacttaccaaatactgtcaatttattcaacagtggcgagttccctagcactggcaatcattgtggctggtctatctttatggatgtgctccaatgggtcattacaatgcagaatttgcatt

>H5N2_A_chicken_Zhejiang_194_2016

atggagaaaatagtgcttcttcttgcagtggttagccttgttaaaagtgatcagatttgcattggttaccatgcaaacaactcgacagagcaggttgacacgataatggaaaaaaacgtcactgttacacatgcccaagacatactggaaaagacacacaacgggaggctctgcgatctgaatggagtgaaacctctgattttaaaggattgtagtgtagctggatggcttcttggaaacccaatgtgcgacgagttcatcagagtgccggaatggtcttatatagtggagagggctaacccagccaatgacctctgttacccagggaatctcaatgactatgaagaactgaaacacctattgagcagaataaatcattttgagaagactctgatcatccccaagagttcttggcccaatcatgaaacatcattaggggtgagcgcagcatgtccataccagggagtgccctcctttttcagaaatgtagtatggcttaccaagaagaatgatgcatacccaacaataaaggtgagctacaataacaccaatagggaagatcttttgatactgtggggaattcatcattccaacaatgcagcagagcagacaaatctctataaaaacccaaccacctatgtttccgttgggacatcaacattaaaccagaggttggtgccaaaaatagctactagatcccaagtaaacgggcaacgtggaagaatggatttcttctggacaattttaaaaccgaatgatgcaatccacttcgagagtaatggaaattttattgctccagaatatgcatacaaaattgtcaagacaggggactcaacaattatgaaaagtgaaatggaatatggccactgcaacaccaaatgtcaaactccaataggggcgataaactccagtatgccattccacaatatacaccctctcactatcggggagtgccccaaatacgttaaatcaaacaaattagtccttgcgactgggctcagaaatagtcctctaagagaaGGGagaagaagaaaaagaggactatttggagctatagcagggtttatagagggaggatggcaaggaatggtagatggttggtatgggtaccaccatagcaatgaacaggggagtgggtacgccgcagacagagaatccacccaaaaggcaatagatggagttaccaataaggtcaactcgataattgacaaaatgaacactcaatttgaggccgttggaagggaatttaataacttagaacggagaatagagaatttaaataagaaaatggaagacggattcctagatgtctggacttataatgctgaacttttggttctcatggaaaatgagagaactctagatttccatgactcaaatgtcaagaacctttatgacaaagtccgactacagcttagggataatgcaaaggagctgggtaatggttgtttcgagttctatcacaaatgtgataatgaatgtatggaaagtgtaagaaatgggacgtatgattacccccagtattcagaagaagcaagattaaaaagggaagaaataagcggagtgaaattggaatcaataggaacttaccaaatactgtcaatttattcaacagtggcgagttccttagcactggcaatcattgtggctggtctatccttatggatgtgctccaatgggtcgttacaatgcagaatttgcatt

>H5N6_A_duck_Hubei_ZYSYF1_2015

atggagaaaatagtgcttcttcttgcagtggttagccttgttaaaagtgatcagatttgcattggttaccatgcaaacaactcgacagagcaagttgacacgataatggaaaaaaacgtcactgttacacatgcccaagacatactggaaaagacacacaacgggaagctctgcgatctgaatggagtgaaacctctgattttaaaggattgtagtgtagctggatggcttcttggaaacccaatgtgcgacgagttcatcagagtgccggaatggtcttacatagtggagagggctaacccagccaatgacctctgttacccagggaacctcaatgactatgaagaactgaaacacctattgagcagaataaatcattttgagaagactctgatcatccccaagagttcttggcccaatcatgaaacatcattaggggtgagcgcagcatgtccataccagggagtgccctcctttttcagaaatgtggtatggcttaccaagaagaatgatgcatacccaacaataaaggtgagctacaataacaccaatagggaagatcttttgatactgtggggaattcatcattccaacaatgcagcagagcagacaaatctctataaaaacccaaccacctatgtttccgttgggacatcaacattaaaccagaggttggtgccaaaaatagctactagatcccaagtaaacgggcaacgtggaagaatggatttcttctggacaattttaaaaccgaaagatgcaatccacttcgagagtaatggaaattttattgctccagaatatgcatacaaaattgtcaagacaggggactcaacaattatgaaaagtgaaatggaatatggccactgcaacaccaaatgtcaaactccaataggggcgataaactccagtatgccattccacaatatacaccctctcactatcggggagtgccccaaatacgtgaaatcaaacaaattagtccttgcgactgggctcagaaatagtcctctaagagaaGGGagaagaagaaaaagaggactatttggagctatagcagggtttatagagggaggatggcaaggaatggtagatggttggtatgggtaccaccatagcaatgaacaggggagtgggtacgccgcagacagagaatccacccaaaaggcaatagatggagttaccaataaggtcaactcgataattgacaaaatgaacactcaatttgaggccgttggaagggaattcaataacttagaacggagaatagagaatttaaataagaaaatggaagacggattcctagatgtctggacttataatgctgaacttttagttctcatggaaaatgagagaactctagatttccatgactcaaatgtcaagaacctttatgacaaagtccgactacagcttagggataatgcaaaggagctgggtaatggttgtttcgagttctatcacaaatgtgataatgaatgtatggaaagtgtaagaaatgggacgtatgattacccccagtattcagaagaagcaagattaaaaagggaagaaataagcggagtgaaattggaatcaataggaacttaccaaatactgtcaatttattcaacagtggcgagttccttagcactggcaatcattgtggctggtctatctttatggatgtgctccaatgggtcgttacaatgcagaatttgcatt

>H5N6_A_Gadwall_Ningxia_472_7_2015

atggagaaaatagtgcttcttcttgcagtggttagccttgttaaaagtgatcagatttgcattggttaccatgcaaacaactcgacagagcaggttgacacgataatggaaaaaaacgtcactgttacacatgcccaagacatactggaaaagacacacaacgggaggctctgcgatctgaatggagtgaaacctctggttttaaaggattgtagtgtagctggatggcttcttggaaacccaatgtgcgatgagttcatcagagtgccggaatggtcttacatagtggagagggcaaacccagctaatgacctctgttacccagggaacctcaatgactatgaagaactaaaacacctattgagcagaataaatcattttgagaagactctgatcatccccaagagttcttggcccaatcatgaaacatcattaggggtgagcgcagcatgtccataccagggaatgccctccttttttagaactgtggtatggcttaccaagaagaacgatgcatacccaacaataaagatgagctacaataataccaatagggaagatcttttgatactgtggggaattcatcattccaacaatgcagcagagcagacaaatctctataaaaacccaaccacatatgtttccgttgggacatcaacattaaaccagaggttggtgccaaaaatagctactagatcccaagtaaatgggcaacgtggaagaatggatttcttctggacaattttaaagccgaatgatgcaatccacttcgagagtaatggaaattttattgctccagaatatgcatacaaaattgtcaagaaaggggactcaacaattatgaaaagtgaaatggaatatggccactgcaacaccaaatgtcaaactccaataggggcaataaactctagcatgccattccacaatatacaccctctcactatcggggagtgccccaaatacgtgaaatcaaacaaattagtccttgcgactgggctcagaaatagtcctctaagagaaGGGagaagaagaaaaagaggactatttggagctatagcagggtttatagagggaggatggcaaggaatggtagatggttggtatgggtaccaccatagcaacgaacaggggagtgggtacgctgcagacagagaatccacccaaaaggcaatagatggagttaccaataaggtcaactcgataattgacaaaatgaacactcaatttgaggccgttggaagggaatttaataacttagaacggagaatagagaatttaaataagaaaatggaagacggattcctagatgtctggacttataatgctgaacttttagttctcatggaaaatgagagaactctagatttccatgactcaaatgtcaagaacctttatgacaaagtccgactacagcttagggataatgcaaaggagctgggtaatggttgtttcgagttctatcacaaatgtgataatgaatgtatggaaagtgtaagaaatgggacgtatgactacccccagtattcagaagaagcaagattaaaaagggaagagataagcggagtgaaattggaatcgataggaacttaccaaatattgtcaatttattcaacagtggcgagttccctagtgctggcaatcattgtggctggtctatctttatggatgtgctccaatgggtcgttacaatgcagaatttgcatt

>H5N6_A_duck_Laos_XBY004_2014

atggagaaaatggtgcttcttcttgcagtggttagccttgttaaaagtgatcagatttgcattggttaccatgcaaacaactcgacagagcaggttgacacgataatggaaaaaaacgtcactgttacacatgcccaagacatactggaaaagacacacaacgggagactctgcgatctgaatggagtgaaacctctgattttaaaggattgtagtgtagctggatggcttcttggaaacccaatgtgtgacgagttcatcagagtgccggaatggtcttacatagtggagagggctaacccagccaatgacctctgttatccagggaacctcaatgactatgaagaactgaaacacctattgagcagaataaatcattttgagaagactctgatcatccccaagagttcttggcccaatcatgaaacatcattaggggtgagcgcagcatgtccataccagggaatgccctcctttttcagaaatgtggtatggcttaccaagaagaacgatgcatacccaacaataaagatgagctacaataataccaatagggaagatcttttgatactgtgggggattcatcattccaacaatgcagcagagcagacaaatctctataaaaacccaaccacctatgtttccgttgggacatcaacattaaaccagagattggagccaaaaatagctactagatcccaagtaaacgggcaacgtggaagaatggatttcttctggaccattttaaaaccgaatgatgcaatccacttcgagagtaatggaaattttattgctccagaatatgcatacaaaattgtcaagaaaggggactcaacaattatgaaaagtgaaatggaatatggccactgcaacaccaaatgtcaaactccaataggggcgataaactctagtatgccattccacaatatacaccctctcactatcggggagtgccccaaatacgtgaaatcgaacaaattagtccttgcgactgggctcagaaatagtcctctaagagaaGGGagaagaagaaaaagaggactatttggagctatagcagggtttatagagggaggatggcaaggaatggtagatggttggtatgggtaccaccatagcaatgaacaggggagtgggtacgctgcagacagagaatccacccaaaaggcaatagatggagttaccaataaggtcaactcgataatagacaaaatgaacactcaatttgaggccgttggaagggaatttaataacttagaacggagaatagagaatttaaataagaaaatggaagacggattcctagatgtctggacttataatgctgaacttttagttctcatggaaaatgagagaactctagatttccatgactcaaatgtcaagaacctttatgacaaagtccgactacagcttagggataatgcaaaggagctgggtaatggttgtttcgagttctatcacaaatgtgataatgaatgtatggaaagtgtaagaaatgggacgtatgactacccccagtattcagaagaagcaagattaaaaagggaagaaataagcggagtgaaattggaatcaataggaacttaccaaatactgtcaatttattcaacagtggcgagttccctagcactggcaatcattgtggctggtctatctttatggatgtgctccaatgggtcgttacaatgcagaatttgcatt

>H5N6_A_Yunnan_0127_2015

atggagaaaatggtgcttcttcttgcagtggttagccttgttaaaagtgatcagatttgcattggttaccatgcaaacaactcgacagagcaggttgacacgataatggaaaaaaacgtcactgttacacatgcccaagacatactggaaaagacacacaacgggagactctgcgatctgaatggagtgaaacctctgattttaaaggattgtagtgtagctggatggctccttggaaacccaatgtgtgacgagttcatcagagtgccggaatggtcttacatagtggagagggctaatccagccaatgacctctgttatccagggaacctcaatgactatgaggaactgaaacacctattgagcagaataaatcattttgagaagactctgatcatccccaagagttcttggcccaatcatGGGacatcatcaggggtgagtgcagcatgtccataccagggaatgccctcctttttcagaaatgtggtatggcttaccaaaaagaacgatgcatacccaacaataaagatgagctacaataatacctatagggaagatcttttgatactgtgggggattcatcattccaacaatgcagcagagcagacaaatctctataaaaacccaaccacctatgtttccgttgggacatcaacattaaaccagagattggagccaaaaatagctactagatcccaagtaaacgggcaacgtggaagaatggatttcttctggaccattttaaaaccgaatgatgcaatccacttcgagagtaatggaaattttattgctccagaatatgcatacaaaattgtcaagaaaggggactcaacaattatgaaaagtgaaatggaatatgggcactgcaacaccaaatgtcaaactccaataggggcgataaactctagtatgccattccacaatatacaccctctcactatcggggagtgccccaaatacgtgaaatcgaacaaattagtccttgcgactgggctcagaaatagtcctctaagagaaGGGagaagaagaaaaagaggactatttggagctatagcagggtttatagagggaggatggcaaggaatggtagatggttggtatgggtaccaccatagcaatgaacaggggagtgggtacgctgcagacagagaatccacccaaaaggcaatagatggagttaccaataaggtcaactcgataatagacaaaatgaacactcaatttgaggccgttggaagagaatttaataacttagaacggagaatagagaatttaaataagaaaatggaagacggattcttagatgtctggacttataatgctgaacttttagttctcatggaaaatgagagaactctagatttccatgactcaaatgtcaagaacctttatgacaaggtccgactacagcttagggataatgcaaaggagctgggtaatggttgtttcgagttctatcacaaatgtgataatgaatgtatggaaagtgtaagaaatgggacgtatgactacccccagtattcagaagaagcaaggttaaaaagggaagaaataagcggggtgaaattggaatcaataggaacttaccaaatactgtcaatttattcaacagtggcgagttccctagcactggcaatcattgtggctggtctatctttatggatgtgctccaatgggtcgttacaatgcagaatttgcatt

>H5N6_A_duck_Japan_AQ_HE72_2015

atggagaaaatagtgctttttcttgcagtggttagccttgttgaaagtgatcagatttgcattggttaccatgcaaacaactcaacagagcaggttgacacgataatggaaaaaaacgtcactgttacacatgcccaagacatactggaaaagacacacaacgggaggctctgcgatctgaatggagtgaaacctctgattttaaaggattgtagtgtagctggatggcttcttggaaacccaatgtgcgacgagttcatcagagtgcctgaatggtcttacatagtggagagggctaacccagccaatgacctctgttacccagggaacctcaatgactatgaagaactgaaacacctattgagcagaataaatcattttgagaagactcagatcatccccaggagttcttggcccaatcatgaaacatcattaggggtgagcgcagcttgtccataccagggaatgccctcctttttcagaaatgtggtatggcttaccaagaagaacgatgcatacccaacaataaagatgagctacaataataccaatagggaagatcttttgatactgtgggggattcatcattctaacaatgcagcagagcagacaaatctctataaaaacccaaacacctatgtttctgttgggacatcaacattaaaccagagattggtgccaaagatagctactagatcccaagtaaacgggcaacgtggaagaatggatttcttctggacaattttaaaaccgaatgatgcaatccacttcgagagtaatggaaattttattgctccagaatatgcatacaaaattgtcaagaaaggggactcaacaattatgaaaagtgaaatggaatatggccactgcaacaccaaatgtcaaactccaataggggcgataaactctagtatgccattccacaatatacaccctctcactatcggggagtgccccaaatacgtgaaatcaaacaaattagtccttgcgactgggctcagaaatagtcctctaagagaaGGGaggagaagaaaaagaggactatttggagctatagcagggtttatagagggaggatggcaaggaatggtagatggttggtatgggtaccaccatagcaatgaacaggggagtgggtacgctgcagacagagaatccacccaaaaggcaatagatggagttactaataaggtcaactcgataattgacaaaatgaacactcaatttgaggccgttggaagggaatttaataacttagaacggagaatagagaatttaaataagaaaatggaagacggattcttagatgtttggacttataatgctgaacttttagttctcatggaaaatgagagaactctagatttccatgactcaaatgtcaagaacctttatgacaaagtccgactacagcttagggataatgcaaaggagctgggcaatggttgtttcgagttctatcacaaatgtgataatgaatgtatggaaagtgtaagaaatgggacgtatgactacccccagtattcagaagaagcaagattaaaaagggaagaaataagcggagtgaaattggaatcaataggaacttaccaaatactgtcaatttattcaacagtggcgagttccctagcactggcaatcattgtggctggtctatctttatggatgtgctccaatgggtcgttacaatgcagaatttgcatt

>H5N6_A_goose_Zhejiang_727092_2014

atggagaaaatagtgcttcttcttgcagtggttagccttgttaaaagtgatcagagttgcattggttaccatgcaaacaactcgacagtgcaagttgacacgataatggaaaaaaacgtcactgttacacatgcccaagacatactggaaaagacacacaacgggaagctctgcgatctgaatggagtgaaacctctgattttaaaggattgtagtgtagctggatggcttcttggaaacccaatgtgcgacgagttcatcagagtgccggaatggtcttacatagtggagagggctaacccagccaatgacctctgttacccagggaacctcaatgactatgaagaactgaaacacctattgagcagaataaatcattttgagaagactctgatcatccccaagagttcttggcccaatcatgaaacatcattaggggtgagcgcagcatgtccataccagggaatgccctcctttttcagaaatgtggtatggcttaccaagaagaacgatgcatacccaacaataaagatgagctacaataataccaatagggaagatcttttgatactgtgggggattcatcattccaacaatgcagcagagcagacaaatctctataaaaacccaaccacctatgtttccgttgggacatcaacattaaaccagagattggtgccaaaaatagctactagatcccaagtaaacgggcaacgtggaagaatggatttcctctggacaattttaaaaccgaatgatgcaatccacttcgagagtaatggaaattttattgctccagaatatgcatacaaaattgtcaagaaaggggactcaacaattatgaaaagtgaaatggaatatggccactgcaacaccaaatgtcaaactccaataggggcgataaactctagtatgccattccacaatatacatcctctcactatcggggagtgccccaaatacgtgaaatcaaacaaattagtccttgcgactgggctcagaaatagtcctctaagagaaGGGagaagaagaaaaagaggactatttggagctatagcagggtttatagagggaggatggcaaggaatggtagatggttggtatgggtaccaccatagcaatgaacaggggagtgggtatgctgcagacaaagaatccacccaaaaggcaatagatggagttaccaataaggtcaactcgataattgacaaaatgaacactcaatttgaggccgttggaagggaatttaataacttagaacggagaatagagaatttaaataagaaaatggaagacggattcctagatgtctggacttataatgctgaacttttagttctcatggaaaatgagagaactctagatttccatgactcaaatgtcaagaacctttatgacaaagtccgactacagcttagggataatgcaaaggagctgggtaatggttgtttcgagttctatcacaaatgtgataatgaatgtatggaaagtgtaagaaatgggacgtatgactacccccagtattcagaagaagcaagattaaaaagggaagaaataagcggagtgaaattggaatcaataggaacttaccaaatactgtcaatttattcaacagtggcgagttccctagcactggcaatcattgtggctggtctatctttatggatgtgctccaatgggtcgttacaatgcagaatttgcatt

>H5N6_A_duck_Zhejiang_727158_2014

atggagataatagtgcttcttcttgcagtggttagccttgttaaaagtgatcagatttgcattggttaccatgcaaacaactcgacagagcaagttgacacgataatggaaaaaaacgtcactgttacacatgcccaagacatactggaaaagacacacaacgggaagctctgcgatctgaatggagtgaaacctctgattttaaaggattgtagtgtagctggatggcttcttggaaacccaatgtgcgacgagttcatcagagtgccggaatggtcttacatagtggagagggctaacccagccaatgacctctgttacccagggaacctcaatgactatgaagaactgaaacacctattgagcagaataaatcattttgagaagactctgatcatccccaagagttcttggcccaatcatgaaacatcattaggggtgagcgcagcatgtccataccagggaatgccctcctttttcagaaatgtggtatggcttaccaagaagaacgatgcatacccaacaataaagatgagctacaataataccaatagggaagatcttttgatactgtgggggattcatcattccaacaatgcagcagagcagacaaatctctataaaaacccaaccacctatgtttccgttgggacatcaacattaaaccagagattggtgccaaaaatagctactagatcccaagtaaacgggcaacgtggaagaatggatttcttctggacaattttaaaaccgaatgatgcaatccacttcgagagtaatggaaattttattgctccagaatatgcatacaaaattgtcaagaaaggggactcaacaattatgaagagtgaaatggaatatggccactgcaacaccaaatgtcaaactccaataggggcgataaactctagtatgccattccacaatatacatcctctcactatcggggagtgccccaaatacgtgaaatcaaacaaattagtgcttgcgactgggctcagaaatagtcctctaagagaaGGGagaagaagaaaaagaggactatttggagctatagcagggtttatagagggaggatggcaaggaatggtagatggttggtatgggtaccaccatagcaatgaacaggggagtgggtatgctgcagacagagaatccacccaaaaggcaatagatggagttaccaataaggtcaactcgataattgacaaaatgaacactcaatttgaggccgttggaagggaatttaataacttagaacggagaatagagaatttaaataagaaaatggaagacggattcctagatgtctggacttataatgctgaacttttagttctcatggaaaatgagagaactctagatttccatgactcaaatgtcaagaacctttatgacaaagtccgactacagcttagggataatgcaaaggagctgggtaatggttgtttcgagttctatcacaaatgtgataatgaatgtatggaaagtgtaagaaatgggacgtatgactacccccagtattcagaagaagcaagattaaaaagggaagaaataagcggagtgaaattggaatcaataggaacttaccaaatactgtcaatttattcaacagtggcgagttccctagcactggcaatcattgtggctggtctatctttatggatgtgctccaatgggtcgttacaatgcagaatttgcatt

>H5N2_A_chicken_Zhejiang_727079_2014

atggagaaaatagtgcttcttcttgcagtggttagccttgttaaaagtgatcagatttgcattggttaccatgcaaacaactcgacagagcaagttgacacgataatggaaaaaaacgtcactgttacacatgcccaagacatactggaaaagacacacaacgggaagctctgcgatctgaatggagtgaaacctctgattttaaaggattgtagtgtagctggatggcttcttggaaacccaatgtgcgacgagttcatcagagtgccggaatggtcttacatagtggagagggctaacccagccaatgacctctgttacccagggaacctcaatgactatgaagaactgaaacacctattgagcagaataaatcattttgagaagactctgatcatccccaagagttcttggcccaatcatgaaacatcattaggggtgagcgcagcatgtccataccagggaatgccctcctttttcagaaatgtggtatggcttaccaagaagaacgatgcatacccaacaataaagatgagctacaataataccaatagggaagatcttttgatactgtgggggattcatcattccaacaatgcagcagagcagacaaatctctataaaaacccaaccacctatgtttccgttgggacatcaacattaaaccagagattggtgccaaaaatagctactagatcccaagtaaacgggcaacgtggaagaatggatttcttctggacaattttaaaaccgaatgatgcaatccacttcgagagtaatggaaattttattgctccagaatatgcatacaaaattgtcaagaaaggggactcaacaattatgaaaagtgaaatggaatatggccactgcaacaccaaatgtcaaactccaataggggcgataaactctagtatgccattccacaatatacatcctctcactatcggggagtgccccaaatacgtgaaatcaaacaaattagtccttgcgactgggctcagaaatagtcctctaagagaaGGGagaagaagaaaaagaggactatttggagctatagcagggtttatagagggaggatggcaaggaatggtagatggttggtatgggtaccaccatagcaatgaacaggggagtgggtatgctgcagacagagaatccacccaaaaggcaatagatggagttaccaataaggtcaactcgataattgacaaaatgaacactcaatttgaggccgttggaagggaatttaataacttagaacggagaatagagaatttaaataagaaaatggaagacggattcctagatgtctggacttataatgctgaacttttagttctcatggaaaatgagagaactctagatttccatgactcaaatgtcaagaacctttatgacaaagtccgactacagcttagggataatgcaaaggagctgggtaatggttgtttcgagttctatcccaaatgtgataatgaatgtatggaaagtgtaagaaatgggacgtatgactacccccagtattcagaagaagcaagattaaaaagggaagaaataagcggagtgaaattggaatcaataggaacttaccaaatactgtcaatttattcaacagtggcgagttccctagcactggcaatcattgtggctggtctatctttatggatgtgctccaatgggtcgttacaatgcagaatttgcatt

>H5N2_A_duck_Zhejiang_727041_2014

atggagaaaatagtgcttcttcttgcagtggttagccttgttaaaagtgatcagatttgcattggttaccatgcaaacaactcgacagagcaagttgacacgataatggaaaaaaacgtcactgttacacatgcccaagacatactggaaaagacacacaacgggaagctctgcgatctgaatggagtgaaacctctgattttaaaggattgtagtgtagctggatggcttcttggaaacccaatgtgcgacgagttcatcagagtgccggaatggtcttacatagtggagagagctaacccagccaatgacctctgttacccagggaacctcaatgactatgaagaactgaaacacctattgagcagaataaatcattttgagaagactctgatcatccccaagagttcctggcccaatcatgaaacatcattaggagtgagcgcagcatgtccataccagggaatgccctcctttttcagaaatgtggtatggcttaccaagaagaacgatgcatacccaacaataaagatgagctacaataataccaatagggaagatcttttgatactgtgggggattcatcattccaacaatgcagcagagcagacaaatctctataaaaacccaaccacctatgtttccgttgggacatcaacattaaaccagagattggtgccaaaaatagctactagatcccaagtaaacgggcaacgtggaagaatggatttcttctggacaattttaaaaccgaaagatgcaatccacttcgagagtaatggaaattttattgctccagaatatgcatacaaaattgtcaagaaaggggactcaacaattatgaaaagtgaaatagaatatgggcactgcaacaccaaatgtcaaactccaataggggcgataaactctagtatgccattccacaatatacatcctctcactatcggggagtgccccaaatacgtgaaatcaaacaaattagtccttgcgactgggctcagaaatagtcctctaagagaaGGGagaagaagaaaaagaggactatttggagctatagcagggtttatagagggaggatggcaaggaatggtagatggttggtatgggtaccaccatagcaatgaacaggggagtgggtatgctgcagacagagaatccacccaaaaggcaatagatggagttaccaataaggtcaactcgataattgacaaaatgaacactcaatttgaggccgttggaagggaatttaataacttagaacggagaatagagaatttaaataagaaaatggaagacggattcctagatgtctggacttataatgctgaacttttagttctcatggaaaatgagagaactctagatttccatgactcaaatgtcaagaacctttatgacaaagtccgactacagcttagggataatgcaaaggagctgggtaatggttgtttcgagttctatcacaaatgtgataatgaatgtatggaaagtgtaagaaatgggacgtatgactacccccagtattcagaagaagcaagattaaaaagggaagaaataagcggagtgaaattggaatcaataggaacttaccaaatactgtcaatttattcaacagtggcgagttccctagcactggcaatcattgtggctggtctatctttatggatgtgctccaatgggtcgttacaatgcagaatttgcatt

>H5N6_A_chicken_Zhejiang_727026_2014

atggagaaaatagtgcttcttcttgcagtggttagccttgttaaaagtgatcagatttgcattggttaccatgcaaacaactcgacagagcaagttgacacgataatggaaaaaaacgtcactgttacacatgcccaagacatactggaaaagacacacaacgggaagctctgcgatctgaatggagtgaaacctctgattttaaaggattgtagtgtagctggatggcttcttggaaacccaatgtgcgacgagttcatcagagtgccggaatggtcttacatagtggagagagctaacccagccaatgacctctgttacccagggaacctcaatgactatgaagaactgaaacacctattgagcagaataaatcattttgagaagactctgatcatccccaagagttcttggcccaatcatgaaacatcattaggggtgagcgcagcatgtccataccagggaatgccctcctttttcagaaatgtggtatggcttaccaagaagaacgatgcatacccaacaataaagatgagctacaataataccaatagggaagatcttttgatactgtgggggattcatcattccaacaatgcagcagagcagacaaatctctataaaaacccaaccacctatgtttccgttgggacatcaacattaaaccagagattggtgccaaaaatagctactagatcccaagtaaacgggcaacgtggaagaatggatttcttctggacaattttaaaaccgaatgatgcaatccacttcgagagtaatggaaattttattgctccagaatatgcatacaaaattgtcaagaaaggggactcaacaattatgaaaagtgaaatggaatatggccactgcaacaccaaatgtcaaactccaataggggcgataaactctagtatgccattccacaatatacatcctctcactatcggggagtgccccaaatacgtgaaatcaaacaaattagtccttgcgactgggctcagaaatagtcctctaagagaaGGGagaagaagaaaaagaggactatttggagctatagcagggtttatagagggaggatggcaaggaatggtagatggttggtatgggtaccaccatagcaatgaacaggggagtgggtatgctgcagacagagaatccacccaaaaggcaatagatggagttaccaataaggtcaactcgataattgacaaaatgaacactcaatttgaggccgttggaagggaatttaataacttagaacggagaatagagaatttaaataagaaaatggaagacggattcctagatgtctggacttataatgctgaacttttagttctcatggaaaatgagagaactctagatttccatgactcaaatgtcaagaacctttatgacaaagtccgactacagcttagggataatgcaaaggagctgggtaatggttgtttcgagttctatcacaaatgtgataatgaatgtatggaaagtgtaagaaatgggacgtatgactacccccagtattcagaagaagcaagattaaaaagggaagaaataagcggagtgaaattggaatcaataggaacttaccaaatactgtcaatttattcaacagtggcgagttccctagcactggcaatcattgtggctggtctatctttatggatgtgctccaatgggtcgttacaatgcagaatttgcatt

>H5N6_A_chicken_Guangdong_GD1602_2016

atggagaaaatagtgcttcttcttgcagtggttagccttgttaaaggtgatcagatttgcattggttaccatgcaaacaactcgactgagcaggttgacacgataatggaaaaaaacgtcactgttacacatgctcaagacatactagaaaagacacataacgggaagctctgcgatctgaatggagtgaaacctctgattttaaaggattgtagtgtagctggatggcttcttggaaacccaatgtgcgacgagttcatcagagtgccggaatggtcttacatagtggaaagggctaacccagccaatgacctctgttacccagggaacctcaatgactatgaagaactgaaacacctattgagcagaataaatcatttcgagaagactctgatcatccccaagagttcttggcccaatcatacaacatcaGGGggggtgagcgcagcatgtccatacctgggaaagccctcctttttcagaaatgtggtatggcttaccaagaagaacgatgcatacccaacaataaaaatgagctacaataacaccaatagggaagatcttttgatactgtgggggattcatcattccaacaatgcagaagagcagacaaatctctataaaaatccaaccacctatgtttccgttgggacatcaacattaaaccagagagtggtgccaaaaatagctactagatcccaagtaaacgggcaaagtggaagaatggatttcttctggacaattttaaaaccggatgatgcaatccacttcgagagtaatggaaattttattgctccagaatatgcatacaaaattgtcaagaaaggggactcaacaattatgaaaagtgaaatggaatatggcaattgcaacaccaaatgtcaaactccaataggggcgataaactctagtatgccattccacaatatacaccctctcactatcggggagtgccccaaatatgtgaaatcaaacaaattagtccttgcgactgggctcagaaatagtcctctaagagaaGGGagaagaagaaaaagaggattatttggagccatagcagggtttatagagggaggatggcaaggaatggtagatggttggtatgggtaccaccatagcaatgcacaagggagtgggtatgctgcagacagagaatccacccaaaaggcaatagatggagttaccaataaggtcaactcgataattgacaaaatgaacactcaatttgaggccgttggaagggaatttaataacttagaacggagaatagagaatttaaataagaaaatggaagacggattcctagatgtctggacttataatgctgaacttttagttctcatggaaaatgagagaactctagatttccatgactcaaatgtcaagaacctttatgacaaagtccgactacagcttagggataatgcaaaggagctgggtaatggttgtttcgagttctatcacaaatgtgataatgaatgtatggaaagtgtgaggaatgggacgtatgactacccccagtattcagaagaagcaagattaaaaagggaagaaataagcggagtgaaattggaatcaataggaacttaccaaatactgtcaatttattcaacagtggcgggttccctagcactggcaatcattgtggctggtctatctttatggatgtgctccaatgggtcgttacaatgcagaatttgcatt

>H5N6_A_chicken_Henan_YB0597_2016

atggagaaaatagtgcttcttcttgcagtggttagccttgtcaaaggtgatcagatttgcattggttaccatgcaaacaactcgactgagcaggttgacacgataatggaaaaaaacgtcactgttacacatgctcaagacatactggaaaagacacacaacgggaagctctgcgatctgaatggagtgaaacctctgattttaaaggattgtagtgtagctggatggcttcttggaaacccaatgtgcgacgagttcatcagagtgccggaatggtcttacatagtggaaagggctaacccagccaatgacctctgttacccagggaacctcaatgactatgaagaactgaaacacctattgagcagaataaatcatttcgagaagactctgatcatccccaagagttcttggcccaatcatGGGacatcatcaggggtgagcgcagcatgtccatacctgggaaagccctcctttttcagaaatgtggtatggcttaccaagaagaacgatgcatacccaacaataaaaatgagctacaataacaccaatagggaagatcttttgatactgtgggggattcatcattccaataatgcagaagagcagacaaatctctataaaaacccaaccacttatgtttccgttgggacatcaacattaaaccagagagtggtgccaaaaatagctactagatcccaagtaaacgggcaaagtggaagaatggatttcttctggacaattttaaaaccggatgatgcaatccacttcgagagtaatggaaattttattgctccagaatatgcatacaaaattgtcaagaaaggggactcaacaattatgaaaagtgaaatggaatatggcaattgcaacaccaaatgtcaaactccaataggggcgataaactctagtatgccattccacaatatacaccctctcactatcggggagtgccccaaatacgtgaaatcaaacaaattagtccttgcgactgggctcagaaatagtcctctaagagaaGGGagaagaagaaaaagaggactatttggggccatagcagggtttatagagggaggatggcaaggaatggtagatggttggtatgggtaccaccatagcaatgaacaagggagtgggtatgctgcagacagagaatccacccaaaaggcaatagatggagttaccaataaggtcaactcgataattgacaaaatgaacactcaatttgaggccgttggaagggaatttaataacttagaacggagaatagagaatttaaataagaaaatggaagacggattcctagatgtctggacttacaatgctgaacttttagttctcatggaaaatgagagaactctagatttccatgactcaaatgtcaagaacctttatgacaaagtccgactacagcttagggataatgcaaaggagctgggtaatggttgtttcgagttctatcacaaatgtgataatgaatgtatggaaagtgtgagaaatgggacgtatgactacccccagtattcagaagaagcaagattaaaaagggaagaaataagcggagtgaaattggaatcaataggaacttaccaaatactgtcaatttattcaacagtggcgggttccctagcactggcaatcattgtggctggtctatctttatggatgtgctccaatgggtcgttacaatgcagaatttgcatt

>H5N6_A_oriental_magpie_robin_HK_6154_2015

atggagaaaatagtgcttcttcttgcagtggttagccttgttaaaggtgatcagatttgcattggttaccatgcaaacaactcgactgagcaggttgacacgataatggaaaaaaacgtcactgttacacatgctcaagacatactggaaaagacacacaacggaaagctctgcgatctgaatggagtgaaacctctgattttaaaggattgtagtgtagctggatggcttcttggaaacccaatgtgcgacgagttcatcagagtgccggaatggtcttacatagtggaaagggctaacccagccaatgacctctgttacccagggaacctcaatgactatgaagaactgaaacacctattgagcagaataaatcattttgagaagactctgatcatccccaagagttcttggcccaatcacGGGacatcatcaggggtgagcgcagcatgtccataccagggaatgccctcctttttcagaaatgtggtatggcttaccaagaagaacgatgcatacccaacaataaagatgagctacaataacaccaatagggaagatcttttgatactatgggggattcatcattccaacaatgcagcagagcagacaaatctctataaaaacccaaccacctatgtttccgttgggacatcaacattaaaccagagattggtgccaaaaatagctactagatcccaagtaaacgggcaaagtggaagaatggatttcttctggacaattttaaaaccgaatgatgcaatccacttcgagagtaatggaaattttattgctccagaatatgcatacaaaattgtcaagaaaggggactcaacaattatgaaaagtgaaatggaatatggctactgcaacaccaaatgtcaaactccaataggggcgataaactctagtatgccattccacaatatacaccctctcactatcggggagtgccccaaatacgtgaaatcaaacaaattagtccttgcgactgggctcagaaatagtcctctaagagaaGGGagaagaagaaaaagaggactatttggagccatagcagggtttatagagggaggatggcaaggaatggtagatggttggtatgggtaccaccatagcaatgaacaagggagtgggtatgctgcagacagagaatccacccaaaaggcaatagatggagttaccaataaggtcaactcgataattgacaaaatgaacactcaatttgaggccgttggaagggaatttaataacttagaacggagaatagagaatttaaataagaaaatggaagacggattcctagatgtctggacttataatgctgaacttttagttctcatggaaaatgagagaactctagatttccatgactcaaatgtcaagaacctttatgacaaagtccgactacagcttagggataatgcaaaggagctgggtaatggttgtttcgagttctatcacaaatgtgataatgaatgtatggaaagtgtgagaaatgggacgtatgactacccccagtattcagaagaagcaagattaaaaagggaagaaataagcggagtgaaattggaatcaataggaacttaccaaatactgtcaatttattcaacagtggcgggttccctagcactggcaatcattgtggctggtctatctttatggatgtgctccaatgggtcgttacaatgcagaatttgcatt

>H5N6_A_wild_pigeon_Jilin_CC01_2014

atggagaaaatagtgcttctcctcgcagtggttagccttgttaaaagtgatcagatttgcattggttaccatgcaaacaactcgacagagcaggttgacacgataatggaaaaaaacgtcactgttacacatgcccaagacatactggaaaagacacacaacgggaagctctgcgatctgaatggagtgaaacctctgattttaaaggattgtagtgtagctggatggcttcttggaaacccaatgtgcgacgagttcatcagagtgccggaatggtcttacatagtggagagggctaacccagccaatgacctctgttacccaggaaacctcaatgactatgaagaactgaaacacctattgagcagaataaatcattttgagaagactatgatcatccccaagagttcttggcccaatcatgaaacatcattaggggtgagcgcagcatgtccataccagggaatgccctcctttttcagaaatgtggtatggcttaccaagaagaacgatgcatacccaacaataaagatgagctacaataataccaatagggaagatcttttggtactgtgggggattcatcattccaacaatgcagcagagcagacaaatctctataaaaacccaaccacctatgtttccgttgggacatcaacattaaaccagagattggtgccaaaaatagctactagatcccaagtaaacgggcaacgtggaagaatggatttcttctggacaattttaaaaccgaatgatgcaatccacttcgagagtaatggaaattttattgctccagaatatgcatacaaaattgtcaagaaaggggactcaacaattatgaaaagtgaaatggagtatggccactgcaacaccaaatgtcaaactccaataggggcgataaactctagtatgccattccacaatatacaccctctcactatcggggagtgccccaaatacgtgaaatcaaacaaattagtccttgcgactgggctcagaaatagtcctctaagagaaGGGagaagaagaaaaagaggactatttggagctatagcagggtttatagagggaggatggcaaggaatggtagatggttggtatgggtaccaccatagtaatgaacaggggagtgggtatgctgcagacagagaatccacccaaaaggcaatagatggagttaccaataaggtcaactcgataattgacaaaatgaacactcaatttgaggccgttggaagggaatttaataacttagaacggagaatagagaatttaaataagaaaatggaagacggattcctagatgtctggacttataatgctgaacttttagttctcatggaaaatgagagaactctagatttccatgactcaaatgtcaagaacctttatgacaaagtccgactacagcttagagataatgcaaaggagctaggtaatggttgtttcgagttctatcacaaatgtgataatgaatgtatggaaagtgtaagaaatgggacatatgactacccccagtattcagaagaagcaagattaaaaagggaagaaataagcggagtgaaattggaatcaataggaacttaccaaatactgtcaatttattcaacagtggcgagttccctagtactggcaatcattgtggctggtctatctttatggatgtgctccaatgggtcgttacaatgcagaatttgcatt

>H5N6_A_duck_Vietnam_LBM835_2015

atggagaaaatagtgcttcttcttgcagtggttagccttgttaaaagtgatcagatttgcattggttaccatgcaaacaactcaacagagcaggttgacacgataatggaaaaaaacgtcactgttacacatgcacaagacatactggaaaagacacacaacgggaggctctgcgatctgaatggagtgaaacctctgattttaaaggattgtagtgtagctggatggcttcttggaaacccaatgtgcgacgagttcatcagagtgccggaatggtcctacatagtggaaagggctaacccagccaatgacctctgttacccagggaatctcaatgactatgaagaactgaaacacttattgagcagaataaatcattttgagaagactctgatcatccccaagagttcttggcccaatcatgaaacatcattaggggtgagcgcagcttgtccataccagggaatgccctcctttttcagaaatgtggtatggcttaccaagaagaacgatgcatacccaacaataaagatgagctacaataataccaatagggaagatcttttgatactgtgggggattcatcatcccaacaatgaagcggagcaaacaagtatctataaaaatccaaacacctatgtttccgttgggacatcaacattaaaccagagattggtgccaaaaatagctactagatcccaagtaaacgggcaacgtggaagaatggatttcttctggacaattttaaaaccgaatgatgcaatccacttcgagagtaatggaaattttattgctccagaatatgcatacaaaattgtcaagaaaggggactcaacaattatgaaaagtgaaatagaatacggctactgcaacaccaaatgtcaaactccaataggggcgataaactctagtatgccattccacaatatacaccctctcactatcggggagtgccccaaatacgtgaaatcaaacaaattagtccttgcaactgggctcagaaatagtcctctaagagaaGGGagaagaagaaaaagagggctatttggagctatagcaggttttatagagggaggatggcaaggaatggtagatggttggtatgggtatcaccatagcaatgaacaggggagtgggtacgctgcagacagagaatccacccaaaaggcaatagatggagttaccaataaggtcaactcgatcattgacaaaatgaacactcaatttgaggccgttggaagggaattcaataacttagaacggagaatagagaatttaaataagaaaatggaagacggattcctagatgtctggacttataatgctgaacttttagttctcatggaaaatgagagaactctagatttccatgactcaaatgtcaagaacctttatgacaaagtccgactacagcttagggataatgcaaaggagctgggtaatggttgtttcgagttctatcacaaatgtgataatgaatgtatggaaagtgtaagaaatgggacgtacgactacccccaatattcagaagaagcaagattaaaaagggaagaaataagcggagtgaaactggaatcaataggaacttaccaaatactgtcaatttattcaacagtggcgagttccctaacactggcaatcatcgtggctggtctatctttatggatgtgctccaatgggtcgttacaatgcagaatttgcatt

>H5N6_A_duck_Vietnam_LBM806_2015

atggagaaaatagtgcttcttcttgcagtggttagccttgttaaaagtgatcagatttgcattggttaccatgcaaacaactcgacagagcaggttgacacgataatggaaaaaaacgtcactgttacacatgcccaagacatactggaaaagacacacaacgggaggctctgcgatctgaatggagtgaaacctctgattttaaaggattgtagtgtagctggatggcttcttggaaacccaatgtgcgacgagttcatcagagtgccggaatggtcctacatagtggaaagggctaacccagccaatgacctctgttacccagggaatctcaatgactatgaagaactgaaacacttattgagcagaataaatcattttgagaagactctgatcatccccaagagttcttggcccaatcatgaaacatcattaggggtgagcgcagcttgttcataccagggaatgccctcctttttcagaaatgtggtatggcttaccaagaagaacgatgcatacccaacaataaagatgagctacaataataccaatagggaagatcttttgatactgtgggggattcatcatcccaacaatgaagcagagcaaaaaagtatctataaaaatccaaccacctatgtttccgttgggacatcaacattaaaccagagattggtgccaaaaatagctactagatcccaagtaaacgggcaacgtggaagaatggatttcttctggacaattttaaaaccgaatgatgcaatccacttcgagagtaatggaaattttattgctccagaatatgcatacaaaattgtcaagaaaggggactcaacaattatgaaaagtgaaatggaatacggctactgcaacaccaaatgtcaaactccaataggggcgataaactctagtatgccattccacaatatacaccctctcactatcggggagtgccccaaatacgtgaaatcaaacaaattagtccttgcgactgggctcagaaatactcctctaagagaaGGGagaagaagaaaaagaggactatttggagctatagcaggttttatagagggaggatggcaaggaatggtagatggttggtatgggtaccaccatagcaatgaacaggggagtgggtacgctgcagacagagaatccacccaaaaggcaatagatggagttaccaataaggtcaactcgatcattgacaaaatgaacactcaatttgaggccgttggaagggaattcaataacttagaacggagaatagagaatttaaataagaaaatggaagacggattcctagatgtctggacttataatgctgaacttttagttctcatggaaaatgagagaactctagatttccatgactcaaatgtcaagaacctttatgacaaagtccgactacagcttagggataatgcaaaggagctgggtaatggttgtttcgagttctatcacaaatgtgataatgaatgtatggaaagtgtaagaaatgggacgtacgactacccccaatattcagaagaagcaagattaaaaagggaagaaataagcggagtgaaactggaatcaataggaacttaccaaatactgtcaatttattcaacagtggcgagttccctaacactggcaatcatcgtggctggtctatctttatggatgtgctccaatgggtcgttgcaatgcagaatttgcatt

>H5N6_A_muscovy_duck_Quang_Ninh_254_2016

atggagaaaatagtgcttcttcttgcagtggttagccttgttaaaagtgatcagatttgcattggttaccatgcaaacaactcgacagagcaggttgacacgataatggaaaaaaacgtcactgttacacatgcccaagacatactggaaaagacacacaacgggaggctctgcgatctgaatggagtgaaacctctgattttaaaggattgtagtgtagctggatggcttcttggaaacccaatgtgcgacgagttcaccagagtgccggaatggtcctacatagtggaaagggctaacccagccaatgacctctgttacccagggaatctcaatgactatgaagaactgaaacacttattgagcagaataaatcattttgagaagactctgatcatccccaagagttcttggcccaatcatgaaacatcattaggggtgagcgcagcttgtccatacttgggaatgccctcctttttcagaaatgtggtatggcttaccaaaaagaacgatgcatacccaacaataaagatgagctacaataataccaatagggaagatcttttgatactgtgggggattcatcatcccaacaatgaagcagagcaaacaagtatctataaaaatccaaccacctatgtttccgttgggacatcaacattaaaccagagattggtgccaaaaatagctactagatcccaagtaaacgggcaacgtggaagaatggatttcttctggacaattttaaaaccgaatgatgcaatccacttcgagagtaatggaaattttattgctccagaatatgcatacaaaattgtcaagaaaggggactcaacaattatgaaaagtgaaatggaatacggctactgcaacaccaaatgtcaaactccaataggggcgataaactctagtatgccattccacaatatacaccctctcactatcggggagtgccccaaatacgtgaaatcaaacaaattagtccttgcgactgggctcagaaatagtcctctaagagaaGGGagaagaagaaaaagaggactatttggagctatagcaggttttatagagggaggatggcaaggaatggtagatggttggtatgggtaccaccatagcaatgaacaggggagtgggtacgctgcagacagagaatccacccaaaaggcaatagatggagttaccaataaggtcaactcgatcattgacaaaatgaacactcaatttgaggccgttggaagggaattcaataacttagaacggagaatagagaatttaaataagaaaatggaagacggattcctagatgtctggacttataatgctgaacttttagttctcatggaaaatgagagaactctagatttccatgactcaaatgtcaagaacctttatgacaaagtccgactacagcttagggataatgcaaaggagctgggtaatggttgtttcgagttctatcacaaatgtgataatgaatgtatggaaagtgtaagaaatgggacgtacgactacccccaatattcagaagaagcaagattaaaaagggaagaaataagcggagtgaaactggaatcaataggaacttaccaaatactgtcaatttattcaacagtggcgagttctctaacactggcaatcatcgtggctggtctatctttatggatgtgctccaatgggtcgttacaatgcagaatttgcatt

>H5N6_A_quail_Vietnam_CVVI_03_2015

atggagaaaataatgcttcttcttgcagtggttagccttgttaaaagtgatcaaatttgcattggttaccatgcaaacaactcgacagagcaggttgacacgataatggaaaaaaacgtcactgttacacatgcccaagacatactggaaaagacacacaacgggaggctctgcgatctgaatggagtgaaacctctgattttaaaggattgtagtgtagctggatggcttcttggaaacccaatgtgcgacgagttcattagagtgccggaatggtcctacatagtggaaagggctaacccagccaatgacctctgttacccagggaatctcaatgactatgaagaactgaaacacttgttgagcagaataaatcattttgagaagactctgatcatccccaagagttcttggcccaatcatgaaacatcattaggggtgagcgcagcttgtccataccagggaatgccctcctttttcagaaatgtggtatggcttaccaagaagaacgatgcatacccaacaataaagatgagctacaataataccaatagggaagatcttttgatactgtgggggattcatcatcccaacagtgaagcagagcaaacaagtatctataaaaatccaaccacctatgtttccgttgggacatcaacattaaaccagagattggtgccaaaaatagctactagatcccaagtaaacgggcaacgtggaagaatggatttcttctggacaattttaaaaccgaatgatgcaatccacttcgagagtaatggaaattttattgctccagaatatgcatacaaaattgtcaagaaaggggactcaacaattatgcaaagtgaaatggaatacggctactgcaacaccaaatgtcaaactccaataggggcgataaactctagtatgccattccacaatatacaccctctcactatcggggagtgccccaaatacgtgaaatcaaacaaattagtccttgcgactgggctcagaaatagtcctctaagagaaGGGagaagaagaaaaagaggactatttggagctatagcaggttttatagagggaggatggcaaggaatggtagatggttggtatgggtaccaccatagcaatgaacaggggagtgggtacgctgcagacagagaatccacccaaaaggcaatagatggagttaccaataaggtcaactcgatcattgacaaaatgaacactcaatttgaggccgttggaagggaattcaataacttagaacggagaatagagaatttaaataagaaaatggaagacggattcctagatgtctggacttataatgctgaacttttagttctcatggaaaatgagagaactctagatttccatgactcaaatgtcaagaacctttatgacaaagtccgactacagcttagggataatgcaaaggagctgggtaatggttgtttcgagttctatcacaaatgtgataatgaatgtatggaaagtgtaagaaatgggacgtacgactacccccaatattcagaagaagcaagattaaaaagggaagaaataagcggagtgaaactggaatcaataggaacttaccaaatactgtcaatttattcaacagtggcgagttccctaacactggcaatcatcgtggctggtctatctttatggatgtgctccaatgggtcgttacaatgcagaatttgcatt

>H5N6_A_chicken_Vietnam_QuangBinh_BD1113_2017

atggagaaaatagtgcttcttcttgcagtggttagccttgttaaaagtgatcagatttgcattggttaccatgcaaacaactcgacagagcaggttgacacgataatggaaaaaaacgtcactgttacacatgcccaagacatactggaaaagacacacaacgggaggctctgcgatctgaatggagtgaaacctctgattttaaaggattgtagtgtagctggatggcttcttggaaacccaatgtgcgacgaattcatcagagtgccggaatggtcttacatagtggagaggactaacccagccaatgacctctgttacccagggaacctcaatgactatgaagaactgaaacacctattgagcagaataaatcattttgagaagactctgatcatccccaagagttcttggcccaatcatgaaacatcatcaggggtgagcgcagcatgcccataccagggagtgccctcctttttcagaaatgtggtatggcttaccaagaagaacgatgcatacccaacaataaagatgagctacaataataccaatggggaagatcttttgatactgtgggggattcatcattccaacaatgcagcagagcagacaaatctctataaaaacccaaccacctatgtttccgttgggacatcaacattaaaccagagattggtgccaaaaatagctactagatcccaagtaaacgggcaacaaggaagaatggatttcttctggacaattttaaaaccgaatgatgcaatccactttgagagtaatggaaattttattgctccagaatatgcatacaaaatagtcaagaaaggggactcaacaattatgaaaagtgaaatggaatatggccactgcaacaccaaatgtcaaactccaataggggcgataaactctagtatgccattccacaatatacaccctctcactatcggggagtgccccaaatacgtgaaatcaaacaaattagtccttgcgactgggctcagaaatagtcctctaagagaaGGGagaagaagaaaaagaggactatttggagctatagcaggttttatagagggaggatggcaaggaatggtagatggttggtatgggtatcaccatagcaatgaacaggggagtgggtacgctgcagacagagaatccacccaaaaggcaatagatggagttaccaataaggtcaactcgatcattgacaaaatgaacactcaatttgaggccgttggaagggaattcaataacttagaacggagaatagagaatttaaataagaaaatggaagacggattcctagatgtctggacttataatgctgaacttttagttctcatggaaaatgagagaactctagatttccatgactcaaatgtcaagaacctttatgacaaagtccgactacagcttagggataatgcaaaggagctgggtaatggttgtttcgagttctatcacaaatgtgataatgaatgtatggaaagtgtaagaaatgggacgtacgactacccccaatattcagaagaagcaagattaaaaagggaagaaataagcggagtgaaactggaatcaataggaacttaccaaatactgtcaatttattcaacagtggcgagttccctaacactggcaatcatcgtggctggtctatctttatggatgtgctccaatgggtcgttacaatgcagaatttgcatt

>H5N6_A_duck_Wuhan_WHYF03_2015

atggagaaaatagtgcttcttcttgcagtggttagccttgttaaaagtgatcagatttgcattggttaccatgcaaacaactcgacagagcaggttgacacgataatggaaaaaaacgtcactgttacacatgcccaagacatactggaaaagacacacaacgggaggctctgcgatctgaatggagtgaaacctctgattttaaaggattgtagtgtagctggatggcttcttggaaacccaatgtgcgacgagttcatcagggtgccggaatggtcttacatagtggagagggctaacccagccaatgacctctgttacccagggaacctcaatgactatgaagaactgaaacacttattgagcagaataaatcattttgagaagactctgatcatccccaagagttcttggcccaatcatgaaacatcattaggggtgagcgcagcatgtccataccagggaatgccctcctttttcagaaatgtggtatggcttaccaagaagaacgatgcatacccaacaataaagatgagctacaataataccaatagggaagatcttttgatactgtgggggattcatcattccaacaatgcagcagagcagacaaatctctataaaaacccaaccacctatgtttccgttgggacatcaacattaaaccagagattggtgccaaaaatagctactagatcccaagtaaacgggcaacgtggaagaatggatttcttctggacaattttaaaaccgaatgatgcaatccatttcgagagtaatggaaactttattgctccagaatatgcatacaaaattgtcaagaaaggggactcaacaattatgaaaagtgaaatggaatacggccactgcaacaccaaatgtcaaactccaataggggcgataaactctagtatgccattccacaatatacaccctctcactatcggggagtgccccaaatacgtgaaatcaaacaaattagtccttgcgactgggctcagaaatagtcctctaagagaaGGGagaagaagaaaaagaggactatttggagctatagcaggttttatagagggaggatggcaaggaatggtagatggttggtatgggtaccaccatagcaatgaacaggggagtgggtacgctgcagacagagaatccacccaaaaggcaatagatggagttaccaataaggtcaactcgatcattgacaaaatgaacactcaatttgaagccgttggaagggaatttaataacttagaacggagaatagagaatttaaataagaaaatggaagacggattcctagatgtctggacttataatgctgaacttttagttctcatggaaaatgagagaactctagatttccatgactcaaatgtcaagaacctttatgacaaagtccgactacagcttagggataatgcaaaggagctgggtaatggttgtttcgagttctatcacaaatgtgataatgaatgtatggaaagtgtaagaaatgggacgtatgactacccccaatactcagaagaagcaagattaaaaagggaagaaataagcggagtgaaactggaatcaataggaacttaccaaatactgtcaatttattcaacagtggcgagttccctaacactggcaatcattgtggctggtctatctttatggatgtgctccaatgggtcgttacaatgcagaatttgcatt

>H5N6_A_chicken_Wuhan_WHYJ02_2015

atggagaaaatagtgcttcttcttgcagtggttagccttgttaaaagtgatcagatttgcattggttaccatgcaaacaactcgacagagcaggttgacacgataatggaaaaaaacgtcactgttacacatgcccaagacatactggaaaagacacacaacgggaggctctgcgatctgaatggagtgaaacctctgattttaaaggattgtagtgtagctggatggcttcttggaaacccaatgtgcgacgagttcatcagggtgccggaatggtcttacatagtggagagggctaacccagccaatgacctctgttacccagggaacctcaatgactatgaagaactgaaacacttattgagcagaataaatcattttgagaagactctgatcatccccaagagttcttggcccaatcatgaaacatcattaggggtgagcgcagcatgtccataccagggaatgccctcctttttcagaaatgtggtatggcttaccaagaagaacgatgcatacccaacaataaagatgagctacaataataccaatagggaagatctcttggtactgtgggggattcatcattccaacaatgcagcagagcagacaaatctctataaaaacccaaccacctatgtttccgttgggacatcaacattaaaccagagattggtgccaaaaatagctactagatcccaagtaaacgggcaacgtggaagaatggatttcttctggacaattttaaaaccgaatgatgcaatccatttcgagagtaatggaaactttattgctccagaatatgcatacaaaattgtcaagaaaggggactcaacaattatgaaaagtgaaatggaatacggccactgcaacaccaaatgtcaaactccaataggggcgataaactctagtatgccattccacaatatacaccctctcactatcggggagtgccccaaatacgtgaaatcaaacaaattagtccttgcgactgggctcagaaatagtcctctaagagaaGGGagaagaagaaaaagaggactatttggagctatagcaggttttatagagggaggatggcaaggaatggtagatggttggtatgggtaccaccatagcaatgaacaggggagtgggtacgctgcagacagagaatccacccaaaaggcaatagatggagttaccaataaggtcaactcgatcattgacaaaatgaacactcaatttgaagctgttggaagggaatttaataacttagaacggagaatagagaatttaaataagaaaatggaagacggattcctagatgtctggacttataatgctgaacttttagttctcatggaaaatgagagaactctagatttccatgactcaaatgtcaagaacctttatgacaaagtccgactacagcttagggataatgcaaaggagctgggtaatggttgtttcgagttctatcacaaatgtgataatgaatgtatggaaagtgtaagaaatgggacgtatgactacccccaatattcagaagaagcaagattaaaaagggaagaaataagcggagtgaaactggaatcaataggaacttaccaaatactgtcaatttattcaacagtggcgagttccctaacactggcaatcattgtggctggtctatctttatggatgtgctccaatgggtcgttacaatgcagaatttgcatt

>H5N6_A_chicken_Nha_Trang_122_2015

atggagaaaatagtgcttcttcttgcattggttagccttgttaaaagtgatcagatttgcattggttaccatgcaaacaactcgacagagcaggttgacacaataatggaaaaaaacgtcactgttacacatgcccaagacatactggaaaagacacacaacgggaggctctgcgatctgaatggagtgaaacctctgattttaaaggattgtagtgtagctggatggcttcttggaaacccaatgtgcgacgagttcatcagagtgccggaatggtcttacatagtggagagggctaacccagccaatgacctctgttacccagggaatctcaatgactatgaagaactgaaacacttattgagcagaataaatcattttgagaagactctgatcatccccaagagttcttggcccaatcatgaaacatcattgggggtgagcgcagcatgtccataccagggaatgccctcctttttcagaaatgtggtatggcttaccaagaagaacgatgcatacccaacaataaaggtgagctataataataccaatagggaagatcttttgatattgtgggggattcatcattccaacaatgcagcagagcagacaaatctctataaaaacccagccacctatgtttccgttgggacatcaacattaaaccagagattggtgcccaaaatagctactagatcccaagtaaacgggcaacgtggaagaatggatttcttctggacaattttaaaaccgaatgatgcaatccacttcgagagtaatggaaattttattgctccagaatatgcatacaaaattgtcaagaaaggggactcaacaattatgaaaagtgaaatggaatacggccactgcaacaccaaatgtcaaactccaataggggcgataaactctagtatgccattccacaatatacaccctctcactatcggggagtgccccaaatacgtgaaatcaaacaaattagtccttgcgactgggctcagaaatagtcctctaagagaaGGGagaagaagaaaaagagggctatttggagctatagcaggttttatagagggaggctggcagggaatggtagatggttggtatgggtaccaccatagcaatgaacaggggagtgggtacgctgcagacagagaatccacccaaaaggcaatagatggagttaccaataaggtcaactcgatcattgacaaaatgaacactcaatttgaggccgttggaagggaatttaataacttagaacggagaatagagaatttaaataagaaaatggaagacggattcctagatgtctggacttataatgctgaacttttagttctcatggaaaatgaaagaactctagatttccatgactcaaatgtcaagaacctttatgacaaagtccgactacagcttagggataatgcaaaggagctgggtaatggttgtttcgagttctatcacaaatgtgataatgaatgtatggaaagtgtaagaaatgggacgtatgactacccccaatattcagaagaagcaagattaaaaagggaagaaataagcggagtgaaactggaatcaataggaacttaccaaatactgtcaatttattcaacagtggcaagttccctaacactggcaatcattgtggctggtctatctttatggatgtgctccaatgggtcgttacaatgcagaatttgcatt

>H5N6_A_chicken_Dongguan_4259_2013

atggagaaaatagtgcttcttcttgcagtggttagccttgttaaaagtgatcagatttgcattggttaccatgcaaacaactcgacagagcaggttgacacgataatggaaaaaaacgtcactgttacacatgcccaagacatactggaaaagacacacaacgggaggctctgcgatctgaatggagtgaaacctctgattttaaaggattgtagtgtagctggatggcttcttggaaacccaatgtgcgacgagttcatcagagtgccggaatggtcttacatagtggagagggctaacccagccaatgacctctgttacccagggaacctcaatgactatgaagaactgaaacacctattgagcagaataaatcattttgagaagactctgatcatccccaagagttcttggcccaatcatgaaacatcattaggagtgagcgcagcatgtccatacctgggaatgccctcctttttcagaaatgtggtatggcttaccaagaagaacgatgcatacccaacaataaagatgagctacaataataccaatagggaagatcttttgatactgtgggggattcatcattccaacaatgcagcagagcagacaaatctctataaaaacccaaccacctatgtttccgttgggacatcaacattaaaccagagattggtgccaaaaatagctactagatcccaagtaaacgggcaacgtggaagaatggatttcttctggacaattttaaaaccgaatgatgcaatccacttcgagagtaatggaaattttattgctccagaatatgcatacaaaattgtcaagaaaggggactcaacaattatgaaaagtgaaatggaatatggccactgcaacaccaaatgtcaaactccaataggggcgataaactctagtatgccattccacaatatacaccctctcactatcggggagtgccccaaatacgtgaaatcaaacaaattagtccttgcgactgggctcagaaatagtcctctaagagaaGGGagaagaagaaaaagaggactatttggagctatagcagggtttatagagggaggatggcaaggaatggtagatggttggtatgggtaccaccatagcaatgaacaggggagtgggtacgctgcagacagagaatccacccaaaaggcaatagatggagttaccaataaggtcaactcgatcattgacaaaatgaacactcaatttgaggccgttggaagggaatttaataacttagaacggagaatagagaatttaaataagaaaatggaagacggattcctagatgtctggacttataatgctgaacttttagttctcatggaaaatgagagaactctagatttccatgactcaaatgtcaagaacctttatgacaaagtccgactacagcttagggataatgcaaaggagctgggtaatggttgtttcgagttctatcacaaatgtgataatgaatgtatggaaagtgtaagaaatgggacgtatgactacccccagtattcagaagaagcaagattaaaaagggaagaaataagcggggtgaaattggaatcaataggaacttaccaaatactgtcaatttattcaacagtggcgagttccctagcactggcaatcattgtggctggtctatctttatggatgtgctccaatgggtcgttacaatgcagaatttgcatt

>H5N6_A_chicken_Taishun_TS2_2016

atggagaaaatagtgcttcttcttgcagtggttagccttgttaaaagtgatcagatttgcattggttaccatgcaaacaactcgacagagcaggttgacacgataatggaaaaaaacgtcactgttacacatgcccgagacatactggaaaagacacacaacgggaggctctgcgatctgaatggagtgaaacctctgattttaaaggattgtagtgtagctggatggcttcttggaaatccaatgtgcgacgagttcaccagagtgccggagtggtcttacatagtggagagggctaacccagccaatgacctctgttacccagggaacttcaatgactatgaagaactgaaacacctattgagtagaataaatcattttgagaagactctgatcatccccaagagttcctggcccaatcatgaaacatcatcaggggtgagcgcagcatgtccataccagggaatgccctcctttttcagaaatgtggtatggcttaccaagaagaacgatgcatacccaacaataaagatgagctacaataataccaatatggaagatcttttgatactgtgggggattcatcattccaataatgcagcagagcagacagatctctataaaaacccaaccacctatgtttccgttgggacatcaacattaaaccagagattggtgccaaaaatagctactagatcccaagtaaacgggcaacgtggaagaatggatttcttctggacaattttaaaaccgaatgatgcaatccacttcgagagtaatggaaattttattgctccagaatatgcatacaaaattgtcaagaaaggggactcaacaattatgaaaagtgaaatggaatatggccactgcaacaccaaatgtcaaactccaataggggcgataaactctagtttgccattccacaatatacaccctctcactatcggggagtgccctaaatacgtgaaatcaaacaaattagtccttgcgactgggctcagaaatagtcctctaagagaaGGGagaagaagaaaaagaggactatttggagctatagcagggtttatagagggaggatggcaaggaatggtagatggttggtatgggtaccaccatagcaatgaacaggggagtgggtacgctgcagacagagaatccactcaaaaggcaatagatggagttaccaataaggtcaactcgatcattgacaaaatgaacactcaatttgaggccgttggaagggaatttaataacttagaacggagaatagagaatttaaataagaaaatggaagacggattcctagatgtctggacttataatgctgaacttctagttctcatggaaaatgagagaactctagattttcatgactcaaatgtcaagaacctttatgacaaagtccgactacagcttagggataatgcaaaggaactgggtaatggttgtttcgagttctatcacaaatgtgataatgaatgtatggaaagtgtgagaaatgggacgtatgactacccccagtattcagaagaagcaagattaaaaagggaagaaataagcggagtgaaattggagacaataggaacttaccaaatactgtcaatttattcaacagtggcgagttccctagcactggcaatcatcgtggctggtctatctttatggatgtgctccaatgggtcgttacaatgcagaatttgcatt

>H5N6_A_chicken_Jiangxi_NCDZT1126_2014

atggagaaaatagtgcttcttcttgcagtggttagccttgttaaaagtgatcagatttgcattggttaccatgcaaacaactcgacagagcaggttgacacgataatggaaaaaaacgtcactgttacacatgcccaagacatactggaaaagacacacaacgggaggctctgcgatctgaatggagtgaaacctctgattttaaaggattgtagtgtagctggatggcttcttggaaacccaatgtgcgacgagttcatcagagtgccggaatggtcttacatagtggagagggctaacccagccaatgacctctgttacccagggaacctcaatgactatgaagaactgaaacacctattgagcagaataaatcattttgagaagactctgatcatccccaagagttcctggcccaatcatgaaacatcattaggggtgagcgcagcatgtccataccagggaatgccctcctttttcagaaatgtggtatggcttaccaagaagaacgatgcgtacccaacaataaagatgagctacaataataccaatagggaagatcttttgatactgtgggggattcatcattccaacaatgcagcagagcagacaaatctctataaaaacccaaccacctatgtttccgttgggacatcaacattaaaccagagattggtgccaaaaatagctactagatcccaagtaaacgggcaacgtggaagaatggatttcttctggacaattttaaaaccgaatgatgcaatccacttcgagagtaatggaaattttattgctccagaatatgcatacaaaattgtcaagaaaggggactcaacaattatgaaaagtgaaatggaatatgggcactgcaacaccaaatgtcaaactccaataggggcgataaactctagtatgccattccacaatatacaccctctcactatcggggagtgccctaaatacgtgaaatcaaacaaattagtccttgcgactgggctcagaaatagtcctctaagagaaGGGagaagaagaaaaagaggactatttggagctatagcagggtttatagagggaggatggcaaggaatggtagatggttggtatgggtaccatcatagcaatgaacaggggagtgggtacgctgcagacagagaatccacccaaaaggcaatagatggagttaccaataaggtcaactcgatcattgacaaaatgaacactcaatttgaggccgttggaagggaatttaataacttagaacggagaatagagaatttaaataagaaaatggaagacggattcctagatgtctggacttataatgctgaacttctagttctcatggaaaatgagagaactctagattttcatgactcaaatgtcaagaacctttatgacaaagtccgactacagcttagggataatgcaaaggagctgggtaatggttgtttcgagttctatcacaaatgtgataatgaatgtatggaaagtgtaagaaatgggacgtatgactacccccagtattcagaagaagcaagattaaaaagggaagaaataagcggagtgaaattggaatcaataggaacttaccaaatactgtcaatttattcaacagtggcgagttccctagcactggcaatcattgtggctggtctatctttatggatgtgctccaatgggtcgttacaatgcagaatttgcatt

>H5N6_2344d_A_Changsha_1_2014

atggagaaaatagtgcttcttcttgcagtggttagccttgttaaaagtgatcagatttgcattggttaccatgcaaacaactcgacagagcaggttgacacgataatggaaaaaaacgtcactgttacacatgcccaagacatactggaaaagacacacaacgggaggctctgcgatctgaatggagtgaaacctctgattttgaaggattgtagtgtagctggatggcttcttggaaacccaatgtgcgacgagttcatcagagtgccggaatggtcttacatagtggagagggctaacccagccaatgacctctgttacccagggaacctcaatgactatgaagaactgaaacacctattgagcagaataaatcattttgagaagactctgatcatccccaagagttcttggcccaatcatGGGacatcatcaggggtgagcgcagcatgtccataccagggaacgccctcctttttcagaaatgtggtatggcttaccaagaagaacgatgcatacccaacaataaagatgagctacaataataccaatagggaagatcttttgatactgtgggggattcatcattccaacaatgcagcagagcagacaaatctctataaaaacccaaccacctatgtttccgttgggacatcaacattaaaccagagattggtgccaaaaatagctactagatcccaagtaaacgggcaacgtggaagaatggatttcttctggacaattttaaaaccgaatgatgcaatccacttcgagagtaatggaaattttattgctccagaatatgcatacaaaattgtcaagaaaggggactcaacaattatgaaaagtgaaatggaatatggccactgcaacaccaaatgtcaaactccaataggggcgataaactctagtatgccattccacaatatacatcctctcactatcggggagtgccccaaatacgtgaaatcaaacaaattagtccttgcgactgggctcagaaatagtcctctaagagaaGGGagaagaagaaaaagaggactatttggagctatagcagggtttatagagggaggatggcaaggaatggtagatggttggtatgggtaccaccatagcaatgaacaggggagtgggtacgctgcagacagagaatccacccaaaaggcaatagatggagttaccaataaggtcaactcgatcattgacaaaatgaacactcaatttgaggccgttggaagggaatttaataacttagaacggagaatagagaatttaaataagaaaatggaagacggattcctagatgtctggacttataatgctgaacttctagttctcatggaaaatgagagaactctagatttccatgactcaaatgtcaagaacctttatgacaaagtccgactgcagcttagggataatgcaaaggagctgggtaatggttgtttcgagttctatcacaaatgtgataatgaatgtatggaaagtgtaagaaatgggacgtatgactacccccagtattcagaagaagcaagattaaaaagggaagaaataagcggagtgaaattggaatcaataggaacttaccaaatactgtcaatttattcaacagtggcgagttccctagcactggcaatcattgtggctggtctatctttatggatgtgctccaatgggtcgttacaatgcagaatttgcatt

>H5N6_A_goose_Eastern_China_S0513_2013

atggagaaaatagtgcttcttcttgcagtggttagccttgttaaaagtgatcagatttgcattggttaccatgcaaacaactcgacagagcaggttgacacgataatggaaaaaaacgtcactgttacacatgcccaagacatactggaaaagacacacaacgggaggctctgcgatctgaatggagtgaaacctctgattttaaaggattgtagtgtagctggatggcttcttggaaacccaatgtgcgacgagttcatcagagtgccggaatggtcttacatagtggagagggctaacccagccaatgacctctgttacccagggaacctcaatgactatgaagaactgaaacacctattgagcagaataaatcattttgagaagactctgatcatccccaagagttcttggcccaatcatgaaacatcattaggggtgagcgcagcatgtccataccagggaatgccctcctttttcagaaatgtggtatggcttaccaagaagaacaatgcatacccaacaataaagatgagctacaataataccaatagggaagatcttttgatactgtgggggattcatcattccaacaatgcagcagagcagacaaatctctataaaaacccaaccacctatgtttccgttgggacatcaacattaaaccagagattggtgccaaaaatagctactagatcccaagtaaacgggcaacgtggaagaatggatttcttctggacaattttaaaaccgaatgatgcaatccacttcgagagtaatggaaattttattgctccagaatatgcatacaaaattgtcaagaaaggggactcaacaattatgaaaagtgaaatggaatatggccactgcaacaccaaatgtcaaactccaataggggcgataaactctagtatgccattccacaatatacaccctctcactatcggggagtgccccaaatacgtgaaatcaaacaaattagtccttgcgactgggctcagaaatagtcctctaagagaaGGGagaagaagaaaaagaggactatttggagctatagcagggtttatagagggaggatggcaaggaatggtagatggttggtatgggtaccaccatagcaatgaacaggggagtgggtacgctgcagacagagaatccacccaaaaggcaatagatggagttaccaataaggtcaactcgatcattgacaaaatgaacactcaatttgaggccgttggaagggaatttaataacttagaacggagaatagagaatttaaataagaaaatggaagacggattcctagatgtctggacttataatgctgaacttctagttctcatggaaaatgagagaactctagatttccatgactcaaatgtcaagaacctttatgacaaagtccgactacagcttagggataatgcaaaggagctgggtaatggttgtttcgagttctatcacaaatgtgataatgaatgtatggaaagtgtaagaaatgggacgtatgactacccccagtattcagaagaagcaagattaaaaagggaagaaataagcggagtgaaattggaatcaataggaacttaccaaatactgtcaatttattcaacagtggcgagttccctagcactggcaatcattgtggctggtctatctttatggatgtgctccaatgggtcgttacaatgcagaatttgcatt

>H5N6_A_duck_Guangdong_GD01_2014

atggagaaaatagtgcttcttcttgcagtggttagccttgttaaaagtgatcagatttgcattggttaccatgcaaacaactcgacagagcaggttgacacgataatggaaaaaaacgtcactgttacacatgcccaagacatactggaaaagacacacaacgggaggctctgcgatctgaatggagtgaaacctctgattttaaaggattgtagtgtagctggatggcttcttggaaacccaatgtgcgacgagttcatcagagtgccggaatggtcttacatagtggagagggctaacccagccaatgacctctgttacccagggaacctcaatgactatgaagaactgaaacacctattgagcagaataaatcattttgagaagactctgatcatccccaagagttcttggcccaatcatgaaacatcattaggggtgagcgcagcatgtccataccagggaatgccctcctttttcagaaatgtggtatggcttaccaagaagaacaatgcatacccaacaataaagatgagctacaataataccaatagggaagatcttttgatactgtgggggattcatcattccaacaatgcagcagagcagacaaatctctataaaaacccaaccacctatgtttccgttgggacatcaacattaaaccagagattggtgccaaaaatagctactagatcccaagtaaacgggcaacgtggaagagtggatttcttctggacaattttaaaaccgaatgatgcaatccacttcgagagtaatggaaattttattgctccagaatatgcatacaaaattgtcaagaaaggggactcaacaattatgaaaagtgaaatggaatatggccactgcaacaccaaatgtcaaactccaataggggcgataaactctagtatgccattccacaatatacaccctctcactatcggggagtgccccaaatacgtgaaatcaaacaaattagtccttgcgactgggctcagaaatagtcctctaagagaaGGGagaagaagaaaaagaggactatttggagctatagcagggtttatagagggaggatggcaaggaatggtagatggttggtatgggtaccaccatagcaatgaacaggggagtgggtacgctgcagacagagaatccacccaaaaggcaatagatggagttaccaataaggtcaactcgatcattgacaaaatgaacactcaatttgaggccgttggaagggaatttaataacttagaacggagaatagagaatttaaataagaaaatggaagacggattcctagatgtctggacttataatgctgaacttctagttctcatggaaaatgagagaactctagatttccatgactcaaatgtcaagaacctttatgacaaagtccgactacagcttagggataatgcaaaggagctgggtaatggttgtttcgagttctatcacaaatgtgataatgaatgtatggaaagtgtaagaaatgggacgtatgactacccccagtattcagaagaagcaagattaaaaagggaagaaataagcggagtgaaattggaatcaataggaacttaccaaatactgtcaatttattcaacagtggcgagttccctagcactggcaatcattgtggctggtctatctttatggatgtgctccaatgggtcgttacaatgcagaatttgcatt

>H5N6_A_duck_Dongguan_3069_2013

atggagaaaatagtgcttcttcttgcagtggttagccttgttaaaagtgatcagatttgcattggttaccatgcaaacaactcgacagagcaggttgacacgataatggaaaaaaacgtcactgttacacatgcccaagacatactggaaaagacacacaacgggaggctctgcgatctgaatggagtgaaacctctgattttaaaggattgtagtgtagctggatggcttcttggaaacccaatgtgcgacgagttcatcagagtgccggaatggtcttacatagtggagagggctaacccagccaatgacctctgttacccagggaacctcaatgactatgaagaactgaaacacctattgagcagaataaatcattttgagaagactctgatcatccccaagagttcttggcccaatcatgaaacatcattaggggtgagcgcagcatgtccataccagggaatgccctcctttttcagaaatgtggtatggcttaccaagaagaacgatgcatacccaacaataaagatgagctacaataataccaatagggaagatcttttgatactgtgggggattcatcattccaacaatgcagcagagcagacaaatctctataaaaacccaaccacctatgtttccgttgggacgtcaacattaaaccagagattggtgccaaaaatagctactagatcccaagtaaacgggcaacgtggaagaatggatttcttctggacaattttaaaaccgaatgatgcaatccacttcgagagtaatggaaattttattgctccagaatatgcatacaaaattgtcaagaaaggggactcaacaattatgaaaagtgaaatggaatatggccactgcaacaccaaatgtcaaactccaataggggcgataaactctagtatgccattccacaatatacaccctctcactatcggggagtgccccaaatacgtgaaatcaaacaaattagtccttgcgactgggctcagaaatagtcctctaagagaaGGGagaagaagaaaaagaggactatttggagctatagcagggtttatagagggaggatggcaaggaatggtagatggttggtatgggtaccaccatagcaatgaacaggggagtgggtacgctgcagacagagaatccacccaaaaggcaatagatggagttaccaataaggtcaactcgatcattgacaaaatgaacactcaatttgaggccgttggaagggaatttaataacttagaacggagaatagagaatttaaataagaaaatggaagacggattcctagatgtctggacttataatgctgaacttctagttctcatggaaaatgagagaactctagatttccatgactcaaatgtcaagaacctttatgacaaagtccgactacagcttagggataatgcaaaggagctgggtaatggttgtttcgagttctatcacaaatgtgataatgaatgtatggaaagtgtaagaaatgggacgtatgactacccccagtattcagaagaagcaagattaaaaagggaagaaataagcggagtgaaattggaatcaataggaacttaccaaatactgtcaatttattcaacagtggcgagttccctagcactggcaatcattgtggctggtctatctttatggatgtgctccaatgggtcgttacaatgcagaatttgcatt

>H5N6_A_duck_Vietnam_HU12_1473_2019

atggagaaaatagtgcttcttcttgcagtgattagccttgtcaaaagtgatcagatttgcattggttaccatgcaaataactcaacagagcaggttgacacgataatggaaaaaaacgtcactgttacacatgcccaagacatactagaaaagacacacaacgggaggctctgcgatttgaatggagtgagacctctgattttaaaggattgtagtgtagctggatggctccttggaaacccgatgtgcgacgagttcatcagagtgccggaatggtcctacatagtggagagggctaacccgccccatgacctctgttaccccgggaacctcaacgactatgaagaactgaaacatctattgagcagaataaatcattttgagaaaactctgatcatccccaaaagttcttggcccaatcatgaaacatcgttaggagtgagcactgcatgccaataccagggaatgccttcctttttcagaaatttggtatggctcatcaagaagaacgatgcatacccaacaataaagatgagctacaataacaccaatagtgaagatcttttgatactgtgggggattcatcatcctaacaacgcagcagaacaaacaaatatctataaaaacccaaccacctatgtttccgttgggacatcaacattaaaccaaagattggtacccaaaatagctactagatcccaagtaaacgggcaacgtggaagaatggatttctactggacaattttaaaaccgaatgatgcaatccacttcgagagtaatggaaattttattgctccagaatatgcatacaaaattgtcaagaaaggggactcaacaatcatgaaaagtgagatggaatatggcaattgcaacaccaaatgccaaactccaataggggcgataaactctagtatgccattccacaatatacaccctctcacaatcggggaatgccccaaatacgtaaaatcaaacaaattagtacttgcgactgggctcagaaatagccccctaagggagGGGagaagaagaaaaagaggactatttggagctatagcaggatttatagagggaggatggcaaggaatggtagatggttggtatgggtatcaccatagcaatgaacaggggagtgggtacgctgcagacagagaatccacccaaaaggcaatagatggagttaccaataaggtcaactcgatcattgagaagatgaacactcaatttgaggccgttgggagggaatttaataacttagaaaggagaatagagaatttaaacaagaaaatggaagacgggttcctggatgtctggacttataatgcggaacttctagttctcatggaaaatgagagaaccctagatttccatgactcaaatgtcaagaacctttatgacaaagtccgactacagcttagggacaatgcaaaggagctgggtaatggttgctttgagttctatcacaaatgtgataatgaatgtatggaaagtgtaagaaatgggacatataactaccctcagtactcagaagaagcaagattgaaaagagaagaaataagcggagtgaaattggaatcaataggaacttaccagatactgtcaatttattcaacagtggcgagttccctagcactggcaatcattgtggctggtctatctttatggatgtgttccaatgggtcactacaatgcagaatttgcatc

>H5N6_A_duck_Vietnam_HU12_971_2019

atggagaaaatagtgcttcttcttgcagtgattagccttgtcaaaagtgatcagatttgcattggttaccatgcaaataactcaacagagcaggttgacacgataatggaaaaaaacgtcactgttacacatgcccaagacatactagaaaagacacacaacgggaggctctgcgatttgaatggagtgaaacctctgattttaaaggattgtagtgtagctggatggctccttggaaacccgatgtgcgacgagttcatcagagtgccggaatggtcctacatagtggagagggctaacccgccccatgacctctgttaccccgggaacctcaacgactatgaagaactgaaacatctattgagcagaataaatcattttgagaaaactctgatcatccccaaaagttcttggcccaatcatgaaacatcgttaggagtgagcgctgcatgccaataccagggaatgccttcctttttcagaaatttggtatggctcatcaagaagaacgatgcatacccaacaataaagatgagctacaataacaccaatggtgaagatcttttgatactgtgggggattcatcattctaacaacgcagcagaacaaacagatctctataaaaacccaaccacctatgtttccgttgggacatcaacattaaaccaaagattggtacccaaaatagctactagatcccaagtaaacgggcaacgtggaagaatggatttctactggacaattttaaaaccgaatgatgcaatccacttcgagagtaatggaaattttattgctccagaatatgcatacaaaattgtcaagaaaggggactcaacaatcatgaaaagtgagatggaatatggcaattgcaacacaaaatgccaaactccaataggggcgataaactctagtatgccattccacaatatacaccctctcacaatcggggaatgccccaaatacgtaaaatcaaacaaattagtacttgcgactgggctcagaaatagccccctaagagagGGGagaagaagaaaaagaggactatttggagctatagcaggatttatagagggaggatggcaaggaatggtagatggttggtatgggtatcaccatagcaatgaacaggggagtgggtacgctgcagacagagaatccacccaaaaggcaatagatggagttaccaataaggtcaactcgatcattgagaagatgaacactcaatttgaggccgttgggagggaatttaataacttagaaaggagaatagagaatttaaacaagaaaatggaagacgggttcctggatgtctggacttataatgcggaacttctagttctcatggaaaatgagagaaccctagatttccatgactcaaatgtcaagaacctttatgataaagtccgactacagcttagggacaatgcaaaggagctgggtaatggttgctttgagttctatcacaaatgtgataatgaatgtatggaaagtgtaagaaatgggacatataactaccctcagtactcagaagaagcaagattgaaaagagaagaaataagcggagtgaaattggaatcaataggaacttaccagatactgtcaatttattcaacagtggcgagttccctagcactggcaatcattgtggctggtctatctttatggatgtgttccaatgggtcactacaatgcagaatttgcatc

>H5N6_A_duck_Vietnam_HU13_65_2019

atggagaaaatagtgcttcttcttgcagtgattagccttgtcaagagtgatcagatttgcattggttaccatgcaaataactcgacagagcaggttgacacgataatggaaaaaaacgtcactgttacacatgcccaagacatactagaaaagacacacaacgggaggctctgcgatttgaatggagtgaaacctctgattttaaaggattgtagtgtagctggatggctccttggaaacccgatgtgcgacgagttcatcagagtgccggaatggtcctacatagtggagagggctaacccgccccacgacctctgttaccccgggaacctcaacgactatgaagaactgaagcatctattgagcagaataaatcattttgagaaaactctgataatccccaaaagttcttggcccaatcatgaaacatcattaggagtgagcgctgcatgccaataccagggaatgccttcctttttcagaaatgtggtatggctcatcaagaagaacgatgcatacccaacaatagagatgagctacaataataccaatagtgaagatcttttgatactgtgggggattcatcattctaacaacgcagcagaacaaacaaatctctataaaaacccaaccacctatgtttccgttgggacatcaacattaaaccagagattggtacccaaaatagctactagatcccaagtaaacgggcaacgtggaagaatggatttctactggacaattttaaaaccgaatgatgcaatccacttcgagagtaatggaaattttattgctccagaatatgcatacaaaattgtcaagaaaggggactcaacaatcatgaaaagtgagatggaatatggccattgcaacaccaaatgccaaactccaataggggcgataaactctagtatgccattccacaatatacaccctctcacaatcggggaatgccccaaatacgtaaaatcaaacaaattagtacttgcaactgggctcagaaatagccccctaagagagGGGaggagaagaaaaagaggactatttggagctatagcaggatttatagagggaggatggcaaggaatggtagatggttggtatgggtaccaccatagcaatgaacagggaagtgggtacgctgcagacaaagaatccacccaaaaggcaatagatggagttaccaataaggtcaactcgatcattgaaaagatgaacactcaatttgaggccgttgggagggaatttaataacttagaaagaagaatagagaatttaaacaagaaaatggaagacggtttcctggatgtctggacttataatgcggaacttctagttctcatggaaaatgagaggaccctagatttccatgactcaaatgtcaagaacctttatgacaaggtccgactacagcttagggacaatgcaaaggagctgggtaatggttgctttgagttctatcacaaatgtgataatgaatgtatggaaagtgtaagaaatgggacatataactaccctcagtactctgaagaagcaagattgaaaagagaagaaataagcggagtgaaattggagtcaataggaacttaccagatactgtcaatttattcaacagtggcgagttccctagcactggcaatcattgtggctggtctatctttatggatgtgttccaatgggtcactacaatgcagaatttgcatc

>H5N6_A_goose_Yangzhou_YZ587_2016

atggagaaaatagtgcttcttcttgcagtggttagccttgttaaaagtgatcagatttgcattggttaccatgcaaataactcgacagagcaggttgacacgataatggaaaaaaacgtcactgttacacatgcccaagacatactagaaaagacacacaacgggaggctctgcgatctgaatggagtgaaacctctgattttaaaggattgtagtgtagctggatggctccttggaaacccaatgtgcgacgagttcatcagagtgccggagtggtcttacatagtggagagggctaacccgcccaatgacctctgttaccccgggaacctcaacgactatgaagaactgaaacatctattgagcagaataaatcattttgagaagactctgatcatccccaagagttcttggcccaatcatgaaacatcattaggagtgagcgcagcatgtcaataccagggaatgccctcctttttcagaaatgtggtatggctcctcaagaagaacgatgcatacccaacaataaagatgagctacaataataccaatagtgaagatcttttgatactgtgggggattcatcattctaacaacgcagcagagcagacaaatctctataaaaacccgaccacctatgtttccgttgggacatcaacattaaaccagagattggtgcccaaaatagctactagatcccaagtaaacgggcaacgtggaagaatggatttcttctggacaattttaaaaccgaatgatgcaatccacttcgagagtaatggaaattttattgctccagaatatgcatacaaaattgtcaagaaaggggactcaacaatcatgaaaagtgaaatggaatatggccattgcaacaccaaatgtcaaactccaataggggcgataaactctagtatgccattccacaatatacaccctctcacaatcggggaatgtcccaaatacgtgaaatcaaacaaattagtacttgcgactgggctcagaaatagtcctctaagggagGGGaggagaagaaaaagaggactatttggagctatagcagggtttatagagggaggatggcaaggaatggtagatggttggtatgggtaccaccatagcaatgaacagggaagtgggtacgctgcagacaaagaatccacccaaaaggcaatagatggagttaccaataaggtcaactcgatcattgacaagatgaacactcaatttgaggccgttggaagggaatttaataacttagaacggagaatagagaatttaaacaagaaaatggaagacggattcctagatgtctggacttataatgcggaacttctagttctcatggaaaatgagagaaccctagatttccatgactcaaatgtcaagaacctttatgacaaagtccgactacagcttagggataatgcaaaggagctgggtaatggttgctttgagttctatcacaaatgtgataatgaatgtatggaaagtgtaagaaatgggacatatgactaccctcagtattcagaagaagcaagattgaaaagagaagaaataagcggagtgaaattggaatcaataggaacttaccagatactgtcaatttattcaacagtggcgagttccctagcactggcaatcattgtggctggtctatctttatggatgtgctccaatgggtcattacaatgcagaatttgcatt

>H5N6_A_chicken_Japan_AQ_HE144_2015

atggagaaaatggtgcttcttcttgcagtggttagccttgttaaaagtgatcagatttgcattggttaccatgcaaataactcgacagagcaggttgacacgataatggaaaaaaacgtcactgttacacatgcccaagacatactggaaaagacacacaacgggagactctgcgatctgaatggagtgaaacctctgattttaaaggattgtagtgtagctggatggctccttggaaacccaatgtgcgacgagttcatcagagtgccggaatggtcttacatagtggagagggctaacccacccaatgacctctgttaccccgggaacctcaacgactatgaagaactgaaacatctattgagcagaataaatcattttgagaagactctgatcatccccaagagctcttggcccaatcatgaaacatcattaggggtgagcgcagcatgtccataccagggaatgccctcctttttcagaaatgtggtatggctcatcaagaagaacgatgcatacccaacaataaagatgagctacaataataccaatagtgaagatcttttgatactgtgggggattcatcattccaacaacgcagcagagcagacagatctctataaaaacccaaccacctatgtttccgttgggacatcaacattaaaccagagattggtgcccaaaatagctactagatcccaagtaaacgggcaacgtggaagaatggatttcttctggacaattttaaaaccgaatgatgcaatccacttcgagagtaatggaaattttattgctccagaatatgcatacaaaattgtcaagaaaggggactcaacaatcatgaaaagtgaaatggaatatggccattgcaacaccaaatgtcaaactccaataggggcgataaactctagtatgccattccacaatatacaccctctcacaatcggggaatgccccaaatacgtgaaatcaaacaaattagtccttgcgactgggctcagaaatagtcctctaagggagGGGaggagaagaaaaagaggactatttggagctatagcagggtttatagagggaggatggcaagggatggtagatggttggtatgggtaccaccatagcaatgaacaggggagtgggtacgctgcagacaaagaatccacccaaaaggcaatagatggagttaccaataaggtcaactcgatcattgacaagatgaacactcaatttgaggccgttggaagggaatttaataacttggaacggagaatagagaatttaaataagaaaatggaagacggattcctagatgtctggacttataatgcggaacttctagttctcatggaaaatgagagaaccctagatttccatgactcaaatgtcaagaacctttatgacaaagtccgactacagcttagggataatgcaaaggagctgggtaatggttgttttgagttctatcacaaatgtgataatgaatgtatggaaagtgtaagaaatgggacgtatgactaccctcagtattcagaagaagcaagattgaagagagaagaaataagcggagtgaaattggaatcaataggaacttaccagatactgtcaatttattcaacagtggcgagttccctagcactggcaatcattgtggctggtctatctttatggatgtgctccaatgggtcattacaatgcagaatttgcatt

>H5N6_A_goose_Hunan_118_2014

atggagaaaatagtgcttcttcttgcagtggttagccttgttaaaagtgatcagatttgcattggttaccatgcaaataactcgacagagcaggttgacacgataatggaaaaaaacgtcactgttacacatgcccaagacatactggaaaagacacacaacgggaggctctgcgatctgaatggagtgaaacctctgattttaaaggattgtagtgtagctggatggctccttggaaacccaatgtgcgacgagttcatcagagtgccggaatggtcttacatagtggagagggctaacccatccaatgacctctgttacccagggaacctcaatgactatgaagaactgaaacatctattgagcagaataaatcattttgagaagactctgatcatccccaagagttcttggtccaatcatgaaacatcattaggggtgagcgcagcatgtccataccagggaatgccctcctttttcagaaatgtggtatggctcatcaagaagaacgatgcatacccaacaataaagatgagctacaataataccaatagtgaagatcttttgatactgtgggggattcatcattccaacaacgcagcagagcagacaaatctctataaaaacccaaccacctatgtttcagttgggacatcaacattaaaccagagattggtgcccaaaatagctactagatcccaagtaaacgggcaacgtggaagaatggatttcttctggacaattttaaaaccgaatgatgcaatccacttcgagagtaatggaaattttattgctccagaatatgcatacaaaattgtcaagaaaggggactcaacaatcatgaaaagtgaaatggaatatggccattgcaacaccaaatgtcaaactccaataggggcgataaactctagtatgccattccacaatatacaccctctcacaatcggggaatgccccaaatacgtgaaatcaaacaaattagtccttgcgactgggctcagaaatagtcctctaagggagGGGaggagaagaaaaagaggactatttggagctatagcagggtttatagagggaggatggcaaggaatggtagatggttggtatgggtaccaccatagcaatgaacaggggagtgggtacgctgcagacaaagaatccacccaaaaggcaatagatggagttaccaataaggtcaactcgatcattgacaagatgaacactcaatttgaggccgttggaagggaatttaataacttagaacggagaatagagaatttaaataagaaaatggaagacggattcctagatgtctggacttataatgcggaacttctagttctcatggaaaatgagagaactctagatttccatgactcaaatgtcaagaacctttatgacaaagtccggctacagcttagggataatgcaaaggagctgggtaatggttgtttcgagttctatcacaaatgtgataatgaatgtatggaaagtgtaagaaatgggacgtatgactaccctcagtattcagaagaagcaagattgaaaagagaagaaataagcggagtgaaattggaatcaataggaacttaccagatactgtcaatttattcaacagtggcgagttccctagcactggcaatcattgtggctggtctatctttatggatgtgctccaatgggtcattacaatgcagaatttgcatt

>H5N1_A_muscovy_duck_Vietnam_LBM636_2014

atggagaaaatagtgcttcttcttgcagtggttagccttgttaaaagtgatcagatttgcattggttaccatgcaaataactcgacagagcaggttgacacgataatggaaaaaaacgtcactgttacacatgcccaagacatactggaaaagacacacaacgggaggctctgcgatctgaatggagtgaaacctctgattttaagggattgtagtgtagctggatggctccttggaaacccaatgtgcgacgagttcatcagagtgccggaatggtcttacatagtggagagggctaacccatccaatgacctctgttacccggggaacctcaatgactatgaagaactgaaacacctattaagcagaataaatcattttgagaagactctgatcatccccaagagttcttggcccgatcatgaaacatcattaggggtgagcgcagcatgtccataccagggaatgccctcctttttcagaaatgtggtatggcttatcaagaagaacgatacatacccaacaataaagataagctacaataataccaatagggaagatcttttgatactgtgggggattcatcattccaacaacgcagcagagcagacagagctctataaaaacccaaacacctatgtttccgttgggacatcaacattaaaccagagattggtgcccaaaatagctactagatcccaagtaaacgggcaacgtggaagaatggatttcttctggacaattttaaaaccgaatgatgcaatccacttcgagagtaatggaaattttattgctccagagtatgcatacaaaattgtcaagaaaggggactcaacaatcatgaaaagtgaaatggaatatggccactgcaacaccaaatgtcaaactccaataggggcgataaactctagtatgccattccacaatatacaccctctcactatcggggaatgccccaaatacgtgaaatcaaacaaattagtccttgcgactgggctcagaaataatcctctaagagagGGGaggagaagaaaaagaggactatttggagctatagcagggtttatagagggaggatggcaagggatggtagatggttggtatgggtaccaccatagcaatgaacaggggagtgggtacgctgcagacaaagaatccacccaaaaggcaatagatggagttaccaataaggtcaactcgatcattgacaagatgaacactcaatttgaggccgttggaagggaatttaataacttggaacggagaatagagaatttaaataagaaaatggaagacggattcctagatgtctggacttataatgctgaacttctagttctcatggaaaatgagagaactctagatttccatgactcaaatgtcaagaacctttacgacaaagtccgactacagcttagggataatgcaaaggagctgggtaatggttgtttcgagttctatcacaaatgtgataatgaatgtatggaaagtgtaagaaatggtacgtatgactaccctcagtattcagaagaagcaagattaaaaagagaagaaataagcggagtgaaattggaatcaataggaacttaccaaatactgtcaatttattcaacagtggcgagttccctagcactggcaatcattgtggctggtctatctttatggatgtgctccaatgggtcgttacaatgcagaatttgcatt

>H5N1_A_duck_Vietnam_LBM638_2014
[truncated: 888,848 more chars]
