# Supplementary material for: Phylodynamics and Molecular Mutations of the Hemagglutinin Affecting Global Transmission and Host Adaptation of H5Nx Viruses
Source: Transbound Emerg Dis. 2023 Apr 14;2023:8855164. doi: 10.1155/2023/8855164 (PMC12017097; doi:10.1155/2023/8855164)
Supplement: Supplementary Materials — Supplemental Table 1: the number of the H5Nx HA sequences by subtype collected. Supplemental Table 2: the number of the H5Nx HA sequences by subtype and isolation year used for the phylogenetic analysis. Supplemental Table 3: amino acid mutations in the HA globular head region of reference human-isolated H5Nx viruses by clade. Supplemental Table 4: summary of natural selection pressure profiles of the H5Nx HAs by clade. Supplemental Data 1: initial sequence set of H5Nx HAs. Supplemental Data 2: selected sequence set of H5Nx HAs. Supplemental Data 3: sequence set of human-isolated H5Nx HAs. Supplemental Figure 1: the proportion of amino acid mutations around the HA globular head region of clade 2.3.4.4 H5Nx viruses by subtype and collection year. (a) The proportion of amino acid mutations around the HA globular head region of clade 2.3.4.4 H5Nx viruses is presented by each subtype (Supplemental Table 1). (b) The H5Nx HA sequences are divided into six periods; (a) 1997–2004 (n = 147), (b) 2005–2008 (n = 533), (c) 2009–2012 (n = 425), (d) 2013–2016 (n = 1082), (e) 2017–2020 (n = 842) and 2021-2022 (n = 860). Supplemental Figure 2: the proportion of the I155T and T160A mutations and molecular interactions of the HA globular head region of H5Nx viruses. (a) The proportion of the I155T (blue) and T160A (magenta) mutations in avian (dashed lines) and human-isolated H5Nx viruses (solid lines) is presented by each period (years). (b) Using the HA structure of VN1194, the receptor-binding pocket in H5 HA contains a conserved floor of residues Y95, W153, H183, Y195, and E190 (pale yellow), and Q226 and G228 (orange) that interact with α2,3 SA receptors. Residues 155 and 160 are colored blue and magenta. Supplemental Figure 3: structural analysis of the HA globular head region of human-isolated H5Nx viruses. Using the HA structure of VN1194, molecular interactions of the HA globular head region residues are estimated; (a) A/Nepal/19FL1997/2019 (H5N1) (NP19FL1197) in subclade 2. [file 8855164.f1.zip › dataS3_revision.docx]

>H5N1_A/Alberta/01/2014

tatcatgcaaataactcgacagagcaggttgacacaataatggaaaagaacgttactgttacacatgcccaagacatactggaaaagacacacaacgggaagctctgcgatctaaatggagtgaagcctctgattttaaaagattgtagtgtagcaggatggctcctcggaaatccattgtgtgacgaattcaccaatgtgccagaatggtcttacatagtagagaaggccaatccagccaatgacctctgttacccagggaatttcaacgattatgaggaactaaaacacctattgagcaggataaaccattttgagaaaatacagatcatccccaaagattcttggtcagatcatgaagcctcattgggggtgagcgcagcatgttcataccagggaaattcctccttcttcagaaatgtggtgtggcttatcaaaaaggacaatgcatacccaacaataaagaaaggctacaataataccaaccgagaagatctcttgatactgtgggggatccaccatcctaatgatgaagcagagcagacaaagctctatcaaaacccaactacctatatttccattgggacttcaacactaaaccagagattggtaccaaaaatagccactagatccaaaataaacaggcaaagtggcaggatagatttcttctggacaattttaaaaccgaatgacgcaatccatttcgagagtaatggaaatttcattgctccagaatatgcatacaaaattgtcaagaagggagactccacaatcatgagaagtgaggtggaatatggtaactgcaacaccaggtgtcagactccaataggggcgataaactctagcatgccattccacaacatacaccctctcactatcggagaatgtcccaaatatgtgaaatcaaacaaattagtccttgcaactgggctcagaaatagtcctcaaagagagagGGGaagaagaaaaaga

>H5N1_A/Anhui/1/2005

taccatgcaaacaactcgacagagcaggttgacacaataatggaaaagaacgttactgttacacatgcccaagacatactggaaaagacacacaacgggaagctctgcgatctagatggagtgaagcctctgattttaagagattgtagtgtagctggatggctcctcggaaacccaatgtgtgacgaattcatcaatgtgccggaatggtcttacatagtggagaaggccaacccagccaatgacctctgttacccagggaatttcaacgactatgaagaactgaaacacctattgagcagaataaaccattttgagaaaattcagatcatccccaaaagttcttggtccgatcatgaagcctcatcaggggtgagctcagcatgtccataccagggaacgccctcctttttcagaaatgtggtatggcttatcaaaaagaacaatacatacccaacaataaagagaagctacaataataccaaccaggaagatcttttgatactgtgggggattcatcattctaatgatgcggcagagcagacaaagctctatcaaaacccaaccacctatatttccgttgggacatcaacactaaaccagagattggtaccaaaaatagctactagatccaaagtaaacgggcaaagtggaaggatggatttcttctggacaattttaaaaccgaatgatgcaatcaacttcgagagtaatggaaatttcattgctccagaatatgcatacaaaattgtcaagaaaggggactcagcaattgttaaaagtgaagtggaatatggtaactgcaacacaaagtgtcaaactccaataggggcgataaactctagtatgccattccacaacatacaccctctcaccatcggggaatgccccaaatatgtgaaatcaaacaaattagtccttgcgactgggctcagaaatagtcctctaagagaaagGGGaagaagaaaaaga

>H5N1_A/Anhui/1/2006

taccatgcaaacaactcgacagagcaggttgacacaataatggaaaagaacgttactgttacacatgcccaagacatactggaaaagacacacaacgggaagctctgcgatctagatggagtgaagcctctgattttaagagattgtagtgtagctggatggctcctcggaaacccaatgtgtgacgaattcatcaatgtgccggaatggtcttacatagtggagaaggccaacccagccaatgacctctgttacccagggaatttcaacgactatgaagaactgaaacacctattgagcagaataaaccattttgagaaaattcagatcatccccaaaagttcttggtccgatcatgaagcctcatcaggggtgagctcagtatgtccataccagggaacgccctcctttttcagaaatgtggtatggcttatcaaaaagaacaatacatacccaacaataaagagaagctacaataataccaaccaggaagatcttttgatactgtggggaattcatcattctaatgatgcggcagagcagacaaagctctatcaaaacccaaccacctatatttccgttgggacatcaacactaaacctgagattggtaccaaaaatagctactagatccaaagtaaacgggcaaagtggaaggatggatttcttctggacaattttaaaaccgagtgatgcaatcaacttcgagagtaatggaaatttcattgctccagaatatgcatacaaaattgtcaagaaaggggactcagcaattatgaaaagtgaagtggaatatggtaactgcaacaccaagtgtcaaactccaataggggcgataaactctagtatgccattccacaacatacaccctctcaccatcggggaatgccccaaatatgtgaaatcaaacaaattagtccttgcgactgggctcagaaatagtcctctaagagagagGGGaagaagaaaaaga

>H5N1_A/Anhui/1/2007

taccatgcaaacaactcgacagagcaggttgacacaataatggaaaagaacgttactgttacacatgcccaagacatactggaaaagacacacaacgggaagctctgcgatctagatggagtgaagcctctgattttaagagattgtagtgtagctggatggctcctcggaaacccaatgtgtgacgagttcatcaatgtgccggaatggtcttacatagtggagaaggccaacccggccaatgacctctgttacccagggaatttcaacgactatgaagaactgaaacacctattgagcagaataaaccattttgagaaaattcagatcatccccaaaagttcttggtccgatcatgacacctcatcaggggtgagctcagcatgtccataccagggaacgccctcctttttcagaaatgtggtatggcttatcaaaaagaacaatacatacccaacaataaagagaagctacaataataccaacaaggaagatcttttgatactgtgggggattcatcattctaatgatgcggcagagcagacaaagctctatcaaaatccaaccacctatatttccgttgggacatcaacactaaaccagagattggtaccaaaaatagctactagatccaaagtaaacgggcaaagtggaaggatggatttcttctggacaattttaaaaccgaatgatgcaatcaacttcgagagtaatggaaatttcattgctccagaatatgcatacaaaattgtcaagaaaggggactcagcaattatgaaaagtgaagtagaatatggtaactgcaatacaaagtgtcaaactccaataggggcgataaactctagtatgccattccacaacatacaccctctcaccatcggggaatgccccaagtatgtgaaatcaaacaaattagtccttgcgactgggctcagaaatagtcctctaagagaaagGGGaagaagaaaaaga

>H5N1_A/Anhui/2/2005

taccatgcaaacaactcgacagagcaggttgacacaataatggaaaagaacgttactgttacacatgcccaagacatactggaaaagacacacaacgggaagctctgcgatctagatggagtgaagcctctgattttaagagattgtagtgtagctggatggctcctcggaaacccaatgtgtgacgaattcatcaatgtgccggaatggtcttacatagtggagaaggccaacccagccaatgacctctgttacccagggaatttcaacgactatgaagaactgaaacacctattgagcagaataaaccattttgagaaaattcagatcatccccaaaagttcttggtccgatcatgaagcctcatcaggggtgagctcagcatgtccataccagggaacgccctcctttttcagaaatgtggtatggcttatcaaaaagaacaatacatacccaccaataaagagaagctacaataataccaaccaggaagatcttttgatactgtgggggattcatcattctaatgatgcggcagagcagacaaagctctatcaaaacccaaccacctatatttccgttgggacatcaacactaaaccagagattggtaccaaaaatagctactagatccaaagtaaacgggcgaagtggaaggatggatttcttctggacaattttaaaaccgaatgatgcaatcaacttcgagagtaatggaaatttcattgctccagaatatgcatacaaaattgtcaagaaaggggactcagcaattatgaaaagtgaagtggaatatggtaactgcaacacaaagtgtcaaactccaataggggcgataaactctagtatgccattccacaacatacaccctctcaccatcggggaatgccccaaatatgtgaaatcaaacaaattagtccttgcgactgggctcagaaatagtcctctaagagaaagGGGaagaagaaaaaga

>H5N1_A/Azerbaijan/008-208/2006

taccatgcaaacaactcgacagagcaggttgacacaataatggaaaagaacgtcactgttacacacgcccaagatatactggaaaaggcacacaacgggaagctctgcgatctagacggagtgaagcctctaattttaagagattgtagtgtagctggatggctcctcgggaacccaatgtgtgacgaattcctcaatgtgccggaatggtcttacatagtggagaagatcaatccagccaatgacctctgttacccagggaatttcaacgactatgaagaactgaaacacctattgagcagaataaaccattttgagaaaattcagatcatccccaaaagttcttggtcagatcatgaagcctcatcaggggtgagctcagcatgtccataccagggaaggtcctccttttttagaaatgtggtatggcttatcaaaaagaacgatgcatacccaacaataaagagaagttacaataataccaaccaagaagatcttttggtactgtgggggattcaccatccaaatgatgcggcagagcagacaaggctctatcaaaacccaaccacctatatttccgttgggacatcaacactaaaccagagattggtaccaaaaatagctactagatccaaggtaaacgggcaaagtggaaggatggagttcttttggacaattttaaaaccgaatgatgcaataaactttgagagtaatggaaatttcattgctccagaaaatgcatacaaaattgtcaagaaaggggactcaacaatcatgaaaagtgaattggaatatggtaactgcaacaccaagtgtcaaactccaataggggcgataaactctagtatgccattccacaacatccaccctctcaccatcggggaatgccctaaatatgtgaaatcaaacagattagtccttgcgactgggctcagaaatagccctcaaggagagagaagaagaaaaaagaga

>H5N1_A/Azerbaijan/011-162/2006

taccatgcaaacaactcgacagagcaggttgacacaataatggaaaagaacgtcactgttacacacgcccaagatatactggaaaaggcacacaacgggaagctctgcaatctagacggagtgaagcctctaattttaagagattgtagtgtagctggatggctcctcgggaacccaatgtgtgacgaattcctcaatgtgccggaatggtcttacatagtggagaagatcaatccagccaatgacctctgttacccagggaatttcaacgactatgaagaactgaaacacctattgagcagaataaaccattttgagaaaattcagatcatccccaaaagttcttggtcagatcatgaagcctcatcaggggtgagctcagcatgtccataccagggaaggtcctccttttttagaaatgtggtatggcttatcaaaaagaacgatgcatacccaacaataaagagaagttacaataataccaaccaagaagatcttttggtactgtgggggattcaccatccaaaagatgcggcagagcagacaaggctctatcaaaacccaaccacctatatttccgttgggacatcaacactaaaccagagattggtaccaaaaatagctactagatccaaggtaaacgggcaaagtggaaggatggagttcttttggacaattttaaaaccgaatgatgcaataaactttgagagtaatggaaatttcattgctccagaaaatgcatacaaaattgtcaagaaaggggactcaacaatcatgaaaagtgaattggaatatggtaactgcaacaccaagtgtcaaactccaataggggcgataaactctagtatgccattccacaacatccaccctctcaccatcggggaatgccccaaatatgtgaaatcaaacagattagtccttgcgactgggctcagaaatagccctcaaggagagagaagaagaaaaaagaga

>H5N1_A/Bangladesh/207095/2008

taccatgcaaacaactcgacagagcaggttgacacaataatggaaaagaacgtcactgttacacacgcccaagacatactggaaaagacacacaacgggaagctctgtgatctagacggagtgaagcctctaattttgagagattgtagtgtagctggatggctcctcgggaatccaatgtgtgacgaattcctcaatgtgccggaatggtcttacatagtggaaaagatcaatccagccaatgacctctgttacccagggaatttcaacgactatgaagaactgaaacacctattgagcagaataaaccattttgagaaaattcagatcatccccaaaagttcttggtcagatcatgaagcctcatcaggggtgagctcagcatgtccataccagggaaggtcctccttttttagaaatgtggtatggcttatcaaaaagaacgatgcatacccaacaataaagataagttacaataataccaaccaagaagatcttttggtattgtgggggattcaccatccaaatgatgcggcagagcagacaagactttatcaaaacccaaccacctatatttccgttgggacatcaacactaaacctgagattggtaccaaaaatagctactagatccaaggtaaacgggcaaagtggaaggatggagttcttttggacaattttaaaaccgaatgatgcaataaattttgagagtaatggaaatttcattgctccagaaaatgcatacaaaattgtcaagaaaggggactcaacgatcatgaaaagtgaattggaatatggtaactgcaacaccaagtgtcaaactccagtaggggcgataaactctagtatgccattccacaacatccaccctctcaccataggggaatgccccaaatatgtgaaatcaaacagattagtccttgcgactgggctcagaaatagccctcaaggagagagaagaagaaaaaagaga

>H5N1_A/Bangladesh/3233/2011

taccatgcaaacaactcgacagagcaggttgacacaataatggaaaagaacgtcactgttacacacgcccaagacatactggaaaagacacacaacgggaagctctgtgatctagacggagtgaagcctctaattttgagagattgtagtgtagctggatggctcctcgggaatccaatgtgtgacgaattcctcaatgtgccggaatggtcttacatagtggaaaagatcaatccagccaatgacctctgttacccagggaatttcaacgactatgaagaactgaaacacctactgagcagaatcaaccattttgagaaaattcagatcatccccaaaagttcttggtcagatcatgaagcctcatcaggggtgagctcagcatgtccgtaccagggaaggtcctccttttttagaaatgtggtatggcttatcaaaaagaacgatgcatacccaacaataaagataagttacaataataccaaccaagaagatcttttggtattgtgggggattcaccatccaaatgatgcggcagagcagacaaaactttatcaaaatccaaccacctatatttccgttgggacatcaacacttaatctgagattggtaccaaaaatagctactagatccaaagtaaacgggcaaagtggaaggatggagttcttttggacaattttaaaaccaaatgatgcaataaattttgagagtaatggaaatttcattgctccagaaaatgcatacaaaattgtcaagaaaggggactcaacgatcatgaaaagtgaattggaatatggcaactgcaacaccaagtgtcaaactccagtaggggcgataaactctagtatgccattccacaacatccaccctctcaccataggggaatgccccaaatatgtgaaatcaaacagattagttcttgcgactgggctcagaaatagccctcaaggagaaagaagaagaaaaaagaga

>H5N1_A/Bangladesh/5487/2011

taccatgcaaacaactcgacagagcaggttgacacaataatggaaaagaacgtcactgttacacacgcccaagacatactggaaaagacacacaacgggaagctctgtgatctagacggagtgaagcctctaattttgagagattgtagtgtagctggatggctcctcgggaatccaatgtgtgacgaattcctcaatgtgccggaatggtcttacatagtggaaaagatcaatccagccaatgacctctgttacccagggaatttcaacgactatgaagaactgaaacacctactgagcagaatcaaccattttgagaaaattcagatcatccccaaaagttcttggtcagatcatgaagcctcatcaggggtgagctcagcatgtccgtaccagggaaggtcctccttttttagaaatgtggtatggcttatcaaaaagaacgatgcatacccaacaataaagataagttacaataataccwaccaagaagatcttttggtattgtgggggattcaccatccaaatgatgcggcagagcagacaaaactttatcaaaatccaaccacctatatttccgttgggacatcaacacttaatctgagattggtaccaaaaatagctactagatccaaagtaaacgggcaaagtgggaggatggagttcttttggacaattttaaaaccaaatgatgctataaattttgagagtaatggaaatttcattgctccagaaaatgcatacaaaattgtcaagaaaggggactcaacgatcatgaaaagtgaattggaatatggcaactgcaacaccaagtgtcaaactccagtaggggcgataaactctagtatgccattccacaacatccatcctctcaccataggggaatgccccaaatatgtgaaatcaaacagattagtccttgcgactgggctcagaaatagccctcaaggagaaagaagaagaaaaaagaga

>H5N1_A/Beijing/01/2003

tcccatgcaaacaactcgacagagcaggttgacacaataatggaaaagaacgttactgttacacatgcccaagacatactggaaaagacacacaacgggaagctctgcgacctagatggagtaaagcctctaattttgagagattgtagtgtagctggatggctcctcggaaacccaatgtgtgacgaattcatcaatgtgccggagtggtcttacatagtggagaaggccagtccagccaatgacctctgttacccaggggatttcaatgactatgaagaactgaaacacctattgagcagaataaaccattttgagaaaattcagatcatccccaaaagttcttggtccaatcatgaagcctcatcaggggtgagctcagcatgtccctacctggggaggccctcctttttcagaaatgtggtatggcttatcaaaaagaataatacatacccaacaataaagaggagctacaataataccaaccaagaagatcttttggtactgtgggggattcaccatcccaatgatgaggcagagcagataaagctctatcaaaacccaaccacctatatttccgttggaacatcaacactaaaccagagattggtaccaaaaatagctactagatccaaagtaaacgggcaaagtggaagaatggagttcttctgggcaattttaaagccgaatgatgctatcaatttcgagagtaatggaaatttcattgctccagaatatgcatacaaaattgtcaagaaaggggactctgcaattatgaaaagtgaattggaatatggtaactgcaacaccaagtgtcaaactccaatgggggcgataaattctagtatgccattccacaacatacaccctctcaccatcggggaatgccccaaatatgtgaaatcaaacagattagtccttgcgactggactcagaaatgcccctcaaagagagggaagaagaaaaaagaga

>H5N1_A/Beijing/1/2009

taccatgcaaacaactcgacagagcaggttgacacaataatggaaaagaacgttactgttacacatgcccaagacatactggaaaagacacataacgggaagctctgcgatctagatggagtgaagcctctaattttaagagattgtagtgtagctggatggctcctcggaaacccaatgtgtgacgaattcatcaatgtgccggaatggtcttacatagtggagaaggccaacccagccaatgacctctgttacccagggaatttcaacgactatgaagaactgaaacacctattgagcagaataaaccattttgagaaaattcagatcatccccaaaagttcttggtccgatcatgaagcctcatcaggggtgagctcagcatgtccataccagggaacgccctcctttttcagaaatgtggtatggcttatcaaaaagaacaatacatacccaacaataaagagaagctacaataataccaaccaggaaaatcttttgatactgtgggggattcatcattctaatgatgcggcagagcagataaagctctatcaaaatccaaccacctatgtttccattgggacatcaacactaaaccagagattggtaccaaaaatagccactagatccaaagtaaacgggcaaagtgggaggatggatttcttctggacaattttaaaaccgaatgatgcaatcaacttcgagagtaatggaaatttcattgctccagaatatgcatacaaaattgtcaaggaaggagactcagcaattatgaaaagtgaagtggaatatggtaactgcaacaccaagtgtcaaactccaataggggcgataaactctagtatgccattccacaacatacaccctctcaccatcggggaatgccccaaatatgtgaaatcaaacaaattagtccttgctactgggctcagaaatagtcctctaagagaaagGGGaagaagaaaaaga

>H5N1_A/Cambodia/408008/2005

taccatgcaaacaactcgacagagcaggttgacacaataatggaaaggaacgttactgttacacatgcccaagacatactggaaaagacacataacgggaagctctgcgatctagatggagtgaagcctctaattttgagagattgtagtgtagctggatggctcctcggaaacccaatgtgtgacgaattcatcaatgtgccggaatggtcttacatagtggagaaggccaatccagtcaatgacctctgttacccaggggatttcaatgactatgaagaattgaaacacctattgagcagaataaaccattttgagaaaattcagatcatccccaaaagttcttggtccagtcatgaagcctcattgggggtgagcgcagcatgtccataccagggaaagtcctcctttttcagaaatgtggtatggcttatcaaaaagaacagtacatacccaacaataaagaggagctacaataataccaaccaagaagatcttttggtaatgtgggggatccaccatcctaatgatgcggcagagcagacaaagctctatcaaaacccaaccacctatatttccgttgggacatcaacactaaatcagagattggtaccaagaatagctactagatccaaagtaaacgggcaaagtgggaggatggagttcttctggacaattttaaaaccgaatgatgcaatcaacttcgagagtaatggaaatttcattgctccagaatatgcatacaaaattgtcaagaaaggggactcaacaattatgaaaagtgaattggaatatggtaactgcaacaccaagtgtcaaactccaatgggggcgataaactctagtatgccattccacaatatacaccctctcaccatcggggaatgccccaaatatgtgaaatcaaacagattagtccttgcgactgggctcagaaatagccctcaaagagagagaagaagaaaaaagaga

>H5N1_A/Cambodia/JP52a/2005

taccatgcaaacaactcgacagagcaggttgacacaataatggaaaagaacgttactgttacacatgcccaagacatactggaaaagacccacaacgggaagctctgtgatctagatggagtgaagcctctaattttgagagattgtagtgtagctggatggctcctcggaaacccaatgtgtgacgaattcatcaatgtgccggaatggtcttacatagtggagaaggccaatccagtcaatgacctctgttacccaggggatttcaatgactatgaagaattgaaacacctattgagcagaataaaccattttgagaaaattcagatcatccccaaaagttcttggtccagtcatgaagcctcattaggggtgagctcagcatgtctataccagggacagtcctcctttttcagaaatgtggtatggcttatcaaaaagaacagtacatacccaacaataaagaggagctacaataataccaaccaagaagatcttttggtaatgtgggggattcaccaccctaatgatgcggcagagcagataaagctctatcaaaacccaaccacctatatttccgttgggacatcaacactaaaccagagattggtaccaagaatagctactagatccaaagtaaacgggcaaagtgggaggatggagttcttctggacaattttaaaaccgaatgatgcaatcaacttcgagagtaatggaaatttcattgctccagaatatgcatacaaaattgtcaagaaaggggactcaacaattatgaaaagtgaattggaatatggtaactgcaacaccaagtgtcaaactccaatgggggcgataaactctagtatgccattccacaatatacaccctctcaccatcggggaatgccccaaatatgtgaaatcaaacagattagtccttgcgactgggctcagaaatagccctcaaagagagagaagaagaaaaaagaga

>H5N1_A/Cambodia/P0322095/2005

taccatgcaaacaactcgacagagcaggttgacacaataatggaaaagaatgtcactgttacacatgcccaagacatactggaaaggacacacaacgggaagctctgcgatctagatggagtgaagcctctaattttgagagattgtagtgtagctggatggctcctcggaaacccaatgtgtgacgaattcatcaatgtgccggaatggtcttacatagtggagagggccaatccagtcaatggcctctgttacccaggggatttcaatgactatgaagaattgaaacacctattgagcagaataaaccattttgagaaaattcaaatcatccccaaaagttcttggtccagtcatgaagcctcattaggggtgagctcagcatgtccatacctgggaaagccctcctttttcagaaatgtggtatggcttatcaaaaagaacagtacatacccaacaataaagaggagctacaataataccaaccaagaagatcttttagtaatgtgggggattcaccatcctaatgatgcggcagagcagacaaagctctatcaaaacccaaccacctatatttccgttgggacatcaacactaaaccagagattggtaccaagaatagctactagatccaaagtaaatgggcaaagtgggaggatggagttcttctggacaattttaaaaccgaatgatgcaatcaacttcgagagtaatggaaatttcattgctccagaatatgcatacaaaattgtcaagaaaggggactcaacaattatgaaaagtgaattggaatatggtaactgcaacaccaagtgtcaaactccaatgggggcgataaactctagtatgccattccacaatatacaccctctcaccatcggggaatgccccaaatatgtgaaatcaaacagattagtccttgcaactgggctcagaaatagccctcaaagagagagaagaagaaaaaagaga

>H5N1_A/Cambodia/Q0321176/2006

taccatgcaaacaactcgacagagcaggttgacacaataatggaaaagaacgttactgttacacatgcccaagacatactggaaaagacacacaacgggaagctctgcgatctagatggagtgaagcctctaattttgagagattgtagtgtagctggatggctcctcggaaacccaatgtgtgacgaattcatcaatgtgccggaatggtcttacatagtggagaaggccaatccggtcaatgacctctgttacccaggggatttcaatgactatgaagaattgaaacacctattaagcagaataaaccattttgagaaaattcagatcatccccaaaagttcttggtccagccatgaagcctcattgggggtgagcgcagcatgtccataccagggaaagtcctcctttttcagaaatgtggtatggcttatcaaaaagaacagtacatacccaacaataaagaggagctacaataataccaaccaagaagatctgttggtaatgtgggggatccaccatcctaatgatgaggcagagcagacaaaactctatcaaaacccaatcacctatatttccgttgggacatcaacactaaaccagagattggtaccaagaatagctactagatccaaagtaaacgggcaaagtgggaggatggagttcttttggacaattttaaaaccgaatgatgcaatcaacttcgagagtaatggaaatttcattgctccagaatatgcatacaaaattgtcaagaaaggggactcaacaattatgaaaagtgaattggaatatggtaactgcaacaccaagtgtcaaactccaatgggggcgataaactctagtatgccattccacaatatacaccctctcaccatcggggaatgccccaaatatgtgaaatcaaacagattagtccttgcgactgggctcagaaatagccctcaaagagagagGGGaagaaaaaagaga

>H5N1_A/Cambodia/Q0405047/2006

taccatgcaaacaactcgacagagcaggttgacacaataatggaaaagaacgttactgttacacatgcccaagacatactggaaaagacacataacgggaagctctgcgatctagatggagtgaagcctctaattttgagagattgtagtgtagctggatggctcctcggaaacccaatgtgtgacgaattcatcaatgtgccggaatggtcttacatagtggagaaggccaatccagtcaatgacctctgttacccaggggatttcaatgactatgaagaattgaaacacctattgagcagaataaaccatttcgagaaaattcagatcatccccaaaagttcttggtccagtcatgaagcctcattgggggtgagcgcagcatgtccataccagggaaagtcctcctttttcagaaatgtggtatggcttatcaaaaagaacagtacatacccaacaataaagaggagctacaataataccaaccaagaagatcttttggtaatgtgggggatccaccatcctaatgatgcggcagagcagacaaagctctatcaaaacccaaccacctatatttccgttgggacatcaacactaaaccagagattggtaccaagaatagctactagatccaaagtaaacgggcaaagtgggaggatggagttcttctggacaattttaaaaccgaatgatgcaatcaactttgagagtaacgggaatttcattgctccagaatatgcatacaaaattgtcaagaaaggggactcaacaattatgaaaagtgaattggaatatggtaactgcaacaccaagtgtcaaactccaatgggggcgataaactctagtatgccattccacaatatacaccctctcaccatcggggaatgccccaaatatgtgaaatcaaacagattagtccttgcgactgggctcagaaatagccctcaaagagagagaagaagaaaaaagaga

>H5N1_A/Cambodia/R0405050/2007

taccatgcaaacaactcgacagagcaggttgacacaataatggaaaagaacgttactgttacacatgcccaagacatactggaaaagacacataacgggaagctctgtgatctagatggagtgaagcctctaatcttgagagattgtagtgtagctggatggctcctcggaaacccaatgtgtgacgaattcatcaatgtgccggaatggtcttatatagtggagaaggccaatccagtcaatgacctctgttacccaggggatttcaatgactatgaagaattgaaacacctattgagcagaataaaccattttgagaaaattcagatcattcccaaaagttcttggcccagtcatgaagcctcattgggggtgagcgcagcatgtccataccaggggaagtcctcttttttcagaaatgtggtatggcttatcaaaaagaacaacacatacccaacaataaagaggagctacaataataccaaccaagaagatcttttggtaatgtgggggatccaccatcctaatgatgcggcagagcagacaaatctctatcaaaatccaaccacctatatttccgttgggacatcaacactaaaccagagattgacaccaagaatagctactagatccaaagtaaacgggcaaagtgggaggatggagttcttctggacaattttaaaaccgaatgatgcaatcaacttcgagagtaatggaaacttcattgctccagagtatgcatacaaaattgtcaagaaaggggactcaacaattatgaaaagtgaattagaatatggtaactgcaacaccaagtgtcaaactccaatgggggcgataaactctagtatgccattccacaatatacatcctctcaccattggggaatgccccaaatatgtgaaatcaaacagattagtccttgcgactgggctcagaaatagccctcaaagagagggaagaagaaaaaagaga

>H5N1_A/Cambodia/S1211394/2008

taccatgcaaacaactcgacagagcaggttgacacgataatggaaaagaacgttactgttacacatgcccaagacatactggaaaagacacataacgggaagctctgcgacctagatggagttaggcctctaattttgagagattgtagtgtagctggatggcttctcggaaacccaatgtgtgacgaattcatcaatgtgccagaatggtcttacatagtggagaaggccaatccagtcaatgacctctgttacccaggagttttcaacgactacgaagaattgaaacacctattgagcagaataaaccattttgagaaaattcagatcatccccaaaagttcttggcccagtcatgaagcctcattgggggtgagcgcagcatgtccataccagggaaagtcctcttttttcagaaatgtggtatggcttattaaaaagaacagtacatacccaacaataaagaggagttacaataataccgaccaagaagatcttttggtaatgtgggggatccaccatcctaatgatgcggcagagcagacaaagctctatcaaaatccaaccacctatatctccgttgggacatcaacgctaaaccagagattgacaccaagaatagctactagatccaaagtaaacgggcaaagtgggaggatggagttcttctggacaattttaaaaccgaatgatgcaatcaacttcgagagtaatggaaatttcattgctccagaatatgcatacaaaattgtcaagaaaggggactcaacaattatgaaaagtgaattggaatatggtaactgcaacaccaagtgtcaaactccaatgggggcgataaactctagtatgccattccacaatatacatcctctcactattggggaatgccccaaatatgtgaaatcaaacagattagtccttgcgactgggctcagaaatagccctcaaagagagggaagaagaaaaaagaga

>H5N1_A/Cambodia/U0417030/2010

taccatgcaaacaactcgacaaagcaggttgacacgataatggaaaagaacgttactgttacacatgcccaagacatactagaaaagacacataacggaaagctctgtgacctagatggagttaggcctctaattttgagagattgtagtgtagctggatggcttctcggaaacccaatgtgtgacgaattcatcaatgtgccagaatggtcttatatagtggagaaggccaatccagtcaatgacctctgttacccaggagttttcaatgactatgaagaattgaaacacctattgagcagaataaaccattttgagaaaattcagatcatccccaagagttcttggcccagtcatgaagcctcattgggggtgagcgcagcatgtccataccagggaaagtcctcttttttccgaaatgtggtgtggcttatcaaaaagaacagtacatacccaacaataaagaggagttacaataataccaaccaagaagatcttttggtaatgtgggggatccaccatcctaatgatgcagcagagcagacaaagctctatcaaaatccaaccacctacatctccgttgggacgtcaacgctaaaccagagattgacaccaagaatagctactagatccaaagtaaacgggcaaagtgggaggatggagttcttttggacaatcttaaaaccgaatgatgcaatcaacttcgagagtaatggaaatttcattgccccagaatatgcatacaaaattgtcaagaaaggggactcaacaattatgaaaagtgaattggaatatggtaactgcaacaccaagtgtcaaactccaatgggggcgataaactctagtatgccattccacaatatacatcctctcactattggagaatgccccaaatatgtgaaatcaaccagattagtccttgcgactgggctcagaaatagccctcaaagagagggaagaagaaaaaagaga

>H5N1_A/Cambodia/V0203306/2011

taccatgcaaacaactcgacaaagcaggttgacacgataatggaaaagaacgttactgttacacatgcccaagacatactagaaaagacacataacggaaagctctgtgacttagatggagttaggcctctaattttgagagattgtagtgtagctggatggcttctcggaaacccaatgtgtgacgaattcatcaatgtgccagaatggtcttatatagtggagaaggccaatccagtcaatgacctctgttacccaggagttttcaatgactatgaagaattgaaacacctattgagcagaataaaccattttgagaaaattcagatcatccccaagagttcttggcccagtcatgaagcctcattgggggtgagcgcagcatgtccataccagggaaagtcctcttttttccgaaatgtggtgtggctcatcaaaaagaacagtacatacccaacaataaagaggagttacaataataccaaccaagaagatcttttggtaatgtgggggatccaccatcctaatgatgcagcagaacagacaaaactctatcaaaatccaaccacctatatctccgttgggacgtcaacactaaaccagagattgacaccaagaatagctactagatccaaagtaaacggacaaagtgggaggatggagttcttttggacaatcttaaaaccgaatgatgcaatcaacttcgagagtaatggaaatttcattgctccagaatatgcatacaaaattgtcaagaaaggggactcaacaattatgaaaagtgaattggaatatggtaactgcaacaccaagtgtcaaactccaatgggggcgataaattctagtatgccattccacaatatacatcctctcactattggagaatgccccaaatatgtgaaatcaaccagattagtccttgcgactgggctcagaaatagccctcaaagagaggaaagaagaaaaaagaga

>H5N1_A/Cambodia/V0219301/2011

taccatgcaaacaactcgacaaagcaggttgacacgataatggaaaagaacgttactgttacacatgcccaagacatactagaaaagacacataacggaaagctctgtgacttagatggagtcagacctctaattttgagagattgtagtgtagctggatggcttctcggaaacccaatgtgtgacgaattcatcaatgtgccagaatggtcttatatagtggagaaggccaatccagtcaatgacctctgttacccaggagttttcaatgactatgaagaattgaaacacctattgagcagaataaaccattttgagaaaattcagatcatccccaagagttcttggcccagtcatgaagcctcattgggggtgagcgcagcatgtccataccagggaaagtcctcttttttccgaaatgtggtgtggcttatcaaaaagaacagtacatacccaacaataaagaggagttacaataataccaaccaagaagatcttttggtaatgtgggggatccaccatcctaatgatgcagcagaacagacaaaactctatcaaaatccaaccacctatatctccgttgggacgtcaacactaaaccagagattgacaccaagaatagctactagatccaaagtaaacggacaaagtgggaggatggagttcttttggacaatcttaaaaccgaatgatgcaatcaacttcgagagtaatggaaatttcattgctccagaatatgcatacaaaattgtcaagaaaggggactcaacaattatgagaagtgaattggaatatggtaactgcaacaccaagtgtcaaactccaatgggggcgataaactctagtatgccattccacaatatacatcctctcactattggagaatgccccaaatatgtgaaatcaaccagattagtccttgcgactgggctcagaaatagccctcaaagagaggaaagaagaaaaaagaga

>H5N1_A/Cambodia/V0401301/2011

taccatgcaaacaactcgacagagcaggttgacacgataatggaaaagaacgttactgttacacatgcccaagacatactggaaaagacacataacgggaagctctgtgacctagatggaattaggcctctaattttgagggattgtagtgtagctggatggcttctcggaaacccaatgtgtgacgaattcatcaatgtgccagaatggtcttacatagtggagaaggccaatccagtcaatgacctctgttacccaggagttttcaatgactatgaagaattgaaacacctattgagccgaataaaccattttgagaaaattcagatcatccccaagagttcttggcccagtcatgaagcctcattgggggtgagcgcagtatgtccataccagggaaagtcctcttttttcagaaatgtggtatggcttatcaaaaagaacagtacatacccgacaataaagaggagttacaataatacaggccaagaagatcttttggtaatgtgggggatccaccatcctaatgatgcggcagagcaaacaaagctctaccaaaatccaaccacctatatctccgttgggacatcaacgctaaaccagagattgacaccaagaatagctactagatccaaagtaaacgggcaaagtgggaggatggagttcttctggacaattttaaaaccgaatgatgcaatcaactttgagagtaatggaaatttcattgctccagaatatgcatacaaaattgtcaagaaaggggactcaacaattatgaaaagtgaattggaatatggcaactgcaacaccaagtgtcaaactccaatgggagcgataaactctagtatgccattccacaatatacatcctctcactattggggaatgccccaaatatgtgaaatcaaacagactagtacttgcgaccgggctcagaaatagccctcaaagagtgggaagaagaaaaaagaga

>H5N1_A/Cambodia/V0417301/2011

taccatgcaaacaactcgacaaagcaggttgacacgataatggaaaagaacgttactgttacacatgcccaagacatactagaaaagacacataacggaaagctctgtgacttagatggagttaggcctctaattttgagagattgtagtgtagctggatggcttctcggaaacccaatgtgtgacgaattcatcaatgtgccagaatggtcttatatagtggagaaggccaatccagtcaatgacctctgttacccaggagttttcaatgactatgaagaattgaaacacctattgagcagaataaaccattttgagaaaattcagatcatccccaagagttcttggcccagtcatgaagcctcattgggggtgagcgcagcatgtccataccagggaaagtcctcttttttccgaaatgtggtgtggctcatcaaaaagaacagtacatacccaacaataaagaggagttacaataataccaaccaagaagatcttttggtaatgtgggggatccaccatcctaatgatgcagcagaacagacaaaactctatcaaaatccaaccacctatatctccgttgggacgtcaacactaaaccagagattgacaccaagaatagctactagatccaaagtaaacggacaaagtgggaggatggagttcttttggacaatcttaaaaccgaatgatgcaatcaacttcgagagtaatggaaatttcattgctccagaatatgcatacaaaattgtcaagaaaggggactcaacaattatgaaaagtgaattggaatatggtaactgcaacaccaagtgtcaaactccaatgggggcgataaattctagtatgccattccacaatatacatcctctcactattggagaatgccccaaatatgtgaaatcaaccagattagtccttgcgactgggctcagaaatagccctcaaagagaggaaagaagaaaaaagaga

>H5N1_A/Cambodia/V0606311/2011

taccatgcaaacaactcgacagagcaggttgacacgataatggaaaagaacgttactgttacacatgcccaagacatactggaaaagacacataacgggaagctctgtgacctagatggaattaggcctctaattttgagggattgcagtgtagctggatggctcctcggaaacccaatgtgtgacgaattcatcaatgtgccagaatggtcttacatagtggagaaggccaatccagtcaacgacatctgttacccaggagttttcaatgactatgaagaattgaaacacctactgagccgaataaaccattttgagaaagttcagatcatccccaagagttcttggcccagtcatgaagcctcattgggggtgagcgcagcatgtccataccagggaaagtcctcttttttcagaaatgtggtatggcttatcaaaaagaacagtacatacccaacaataaagaggagttacaataatacaggccaagaagatcttttgataatgtgggggatccaccatcctaatgatgcggcagagcaaacaaaactctaccaaaatccaaccacctatatctccgttgggacatcaacgctaaaccagagattgacaccaagaatagctactagatccaaagtaaacgggcaaaatgggaggatggagttcttctggacaattttaaaaccgaatgatgcaatcaacttcgagagtaatggaaatttcattgctccagaatatgcatacaaaattgtcaagaaaggggactcaacaattatgaaaagtgaattggaatatggcaactgcaacaccaggtgtcaaactccaatgggggcgataaactctagtatgccattccacaatatacatcctctcactattggggaatgccccaaatatgtgaaatcaaacagactagtacttgcgaccgggctcagaaatagccctcaaagagagggaagaagaaaaaagaga

>H5N1_A/Cambodia/V0719348/2011

taccatgcaaacaactcgacaaagcaggttgacacgataatggaaaagaacgttactgttacacatgcccaagacatactagaaaagacacacaacggaaagctctgtgacttagatggagttaggcctctaattttgagagattgtagtgtagctggatggcttctcggaaacccaatgtgtgacgaattcatcaatgtgccagaatggtcttatatagtggagaaggccaatccaatcaatgacctctgttacccaggagttttcaatgactatgaagaattgaaacacctattgagcagaataaaccattttgagaaaattcagatcatccccaagagttcttggcctagtcatgaagcctcattgggggtgagcgcagcatgtccataccagggaaagtcctcttttttccgaaatgtggtgtggcttatcaaaaagaacagtacatacccaacaataaagaggagttacaataataccaaccaagaagatcttttggtaatgtgggggatccaccatcctaatgatgcagcagaacagacaaaactctatcaaaatccaaccacctatatctccgttgggacgtcaacactaaaccagagattgacaccaagaatagctactagatccaaagtaaacggacaaagtgggaggatggagttcttttggacaatcttaaaaccgaatgatgcaatcaacttcgagagtaatggaaatttcattgctccagaatatgcatacaaaattgtcaagaaaggggactcaacaattatgagaagtgaattggaatatggtaactgcaacaccaagtgtcaaactccaatgggggcaataaactctagtatgccattccacaatatacatcctctcactattggagaatgccccaaatatgtgaaatcaaccagattagtcctagcaactgggctcagaaatagccctcaaagagaggaaagaagaaaaaagaga

>H5N1_A/Cambodia/V0813302/2011

taccatgcaaacaactcgacaaagcaggttgacacgataatggaaaagaacgttactgttacacatgcccaagacatactagaaaagacacacaacggaaagctctgtgacttagatggagttaggcctctaattttgagagattgtagtgtagctggatggcttctcggaaacccaatgtgtgacgaattcatcaatgtgccagaatggtcttatatagtggagaaggccaatccaatcaatgacctctgttacccaggagttttcaatgactatgaagaattgaaacacctattgagcagaataaaccattttgagaaaattcagatcatccccaagagttcttggcctagtcatgaagcctcattgggggtgagcgcagcatgtccataccagggaaagtcctcttttttccgaaatgtggtgtggcttatcaaaaagaacagtacatacccaacaataaagaggagttacaataataccaaccaagaagatcttttggtaatgtgggggatccaccatcctaatgatgcagcagaacagacaaaactctatcaaaatccaaccacctatatctccgttgggacgtcaacactaaaccagagattgacaccaagaatagctactagatccaaagtaaacggacaaagtgggaggatggagttcttttggacaatcttaaaaccgaatgatgcaatcaacttcgagagtaatggaaatttcattgctccagaatatgcatacaaaattgtcaagaaaggggactcaacaattatgagaagtgaattggaatatggtaactgcaacaccaagtgtcaaactccaatgggggcaataaactctagtatgccattccacaatatacatcctctcactattggagaatgccccaaatatgtgaaatcaaccagattagtccttgcaactgggctcagaaatagccctcaaagagaggaaagaagaaaaaagaga

>H5N1_A/Cambodia/W0112303/2012

taccatgcaaacaactcgacaaagcaggttgacacgataatggaaaagaacgttactgttacacatgcccaagacatactagaaaagacacacaacggaaagctctgtgacttagatggagttaggcctctaattttgagagattgtagtgtagctggatggcttctcggaaacccaatgtgtgacgaattcatcaatgtgccagaatggtcttatatagtggagaaggccaatccaatcaatgacctctgttacccaggagttttcaatgattatgaagaattgaaacacctattgagcagaataaaccattttgagaaaattcagatcatccccaagagttcttggcctagtcatgaagcctcattgggggtgagcgcagcatgtccataccagggaaagtcctcttttttccgaaatgtggtgtggcttatcaaaaagaacagtacatacccaacaataaagaggagttacaataatactaaccaagaagatcttttggtaatgtgggggatccaccatcctaatgatgcagcagaacagacaaaactctatcaaaacccaaccacctatatctccgttgggacgtcaacactaaaccagagattgacaccaagaatagctactagatccaaagtaaacggacaaagtgggaggatggagttcttttggacaatcttaaaaccgaatgatgcaatcaacttcgagagtaatggaaatttcattgctccagaatatgcatacaaaattgtcaagaaaggggactcaacaattatgagaagtgaattggaatatggtaactgcaacaccaagtgtcaaactccaatgggggcaataaactctagtatgccattccacaatatacatcctctcactattggagaatgccccaaatatgtgaaatcaaccagattagtccttgcaactgggctcagaaatagccctcaaagagaggaaagaagaaaaaagaga

>H5N1_A/Cambodia/W0329318/2012

taccatgcaaacaactcgacaaagcaggttgacacgataatggaaaagaacgttactgttacacatgcccaagacatactagaaaagacacacaacggaaagctctgtgacttagatggagttaggcctctaattttgagagattgtagtgtagctggatggcttctcggaaacccaatgtgtgacgaattcatcaatgtgccagaatggtcttatatagtggagaaggccaatccaatcaatgacctctgttacccaggagttttcaatgactatgaagaattgaaacacctattgagcagaataaaccattttgagaaaattcagatcatccccaagagttcttggcctagtcatgaagcctcattgggggtgagcgcagcatgtccataccagggaaagtcctcttttttccgaaatgtggtgtggcttatcaaaaagaacagtacatacccaacaataaagaggagttacaataatactaaccaagaagatcttttggtaatgtgggggatccaccatcctaatgatgcagcagaacagacaaaactctatcaaaatccaaccacctatatctccgttgggacgtcaacactaaaccagagattgacaccaagaatagctactagatccaaagtaaacggacaaagtgggaggatggagttcttttggacgatcttaaaaccgaatgatgcaatcaacttcgagagtaatggaaatttcattgctccagaatatgcatacaaaattgtcaagaaaggggactcaacaattatgagaagtgaattggaatatggtaactgcaacaccaagtgtcaaactccaatgggggcaataaactctagtatgccattccacaatatacatcctctcactattggagaatgccccaaatatgtgaaatcaaccagattagtccttgcaactgggctcagaaatagccctcaaagagaggaaagaagaaaaaagaga

>H5N1_A/Cambodia/W0526301/2012

taccatgcaaacaactcgacagagcaggttgacacgataatggaaaagaacgttactgttacacatgcccaagacatactagaaaagacacataacggaaagctctgtgacttagatggagttaggcctctaattttgagagattgtagtgtagctggatggcttctcggaaacccaatgtgtgacgaattcatcaatgtgccagaatggtcttatatagtggagaaggccaatccagtcaatgacctctgttacccaggagttttcaatgactatgaagaattgaaacacctattgagcagaataaaccattttgagaaaatccagatcatccccaagagttcttggcccagtcatgaagcctcattgggggtgagcgcagcatgtccataccaggggcagtcctcttttttccgaaatgtggtgtggcttatcaaaaagaacagtacatacccaacaataaagaggagttacaataataccaaccaagaagaccttttggtaatgtgggggatccaccatcccaatgatgcagcagaacagacaaaactctatcaaaatccaaccacctatatctccgttgggacgtcaacactaaaccagagattgacaccaagaatagctactagatccaaagtaaacggacaaagtgggaggatggagttcttttggacaatcttaaaaccgaatgatgcaatcaacttcgagagtaatggaaatttcattgctccagaatatgcatacaaaattgtcaagaaaggggactcaacaattatgagaagtgaattggaatatggtaactgcaacaccaagtgtcaaactccaatgggggcgataaactctagcatgccattccacaatatacatcctctcactattggagaatgccccaaatatgtgaaatcaaccagattagtccttgcgactgggctcagaaatagccctcaaagagaggaaagaagaaaaaagaga

>H5N1_A/Cambodia/X0121311/2013

taccatgcaaacaactcgacagagcaggttgacacgataatggaaaagaacgttactgttacacatgcccaagacatactagaaaagacacataacggaaagctctgtgacttagatggagttaggcctctaattttgagagattgtagtgttgctggatggcttctcggaaacccaatgtgtgacgaattcatcaatgtgccagaatggtcttatatagtggagaaggccaatccagtcaatgacctctgttacccaggagttttcaatgactatgaagaattgaaacacctattgagcagaataaaccattttgagaaaattcagatcatccccaagagttcttggcccagtcatgaagcctcattgggggtgagcgcagcatgtccataccaggggcagtcctcttttttcagaaatgtggtgtggcttatcaaaaagaacaatacatacccaacaataaagaggagttacaataataccaaccaagaagatcttttggtaatgtgggggatccaccatcctaatgatgcagcagaacagacaaaactctatcaaaatccaaccacctatatctccgttgggacgtcaacattaaaccagagattgacaccaagaatagctactagatccaaagtaaacggacgaagtgggaggatggagttcttttggacaatcttaaaaccgaatgatgcaatcaacttcgagagtaatggaaatttcatcgctccagaatatgcatacaaaattgtcaagaaaggggactcaacaattatgagaagtgaactggaatatggtaactgcaacaccaagtgtcaaactccaatgggggcgataaactctagtatgccattccacaatatacatcctctcactattggagaatgccccaaatatgtgaaatcaaccagattagtccttgctactgggctcagaaatagccctcaaagagaggaaagaagaaaaaagaga

>H5N1_A/Cambodia/X0123311/2013

tatcatgcaaacaactcgacagagcaggttgacacgataatggaaaagaacgttactgttacacatgcccaagacatactagaaaagacacataacggaaagctctgtgacttagatggagttaggcctctaattttgagagattgtagtgtagctggatggcttctcggaaacccaatgtgtgacgaattcatcaatgtgccagaatggtcttatatagtggagaaggccaatccagtcaatgacctctgttacccaggagttttcaatgactatgaagaattgaaacacctattgagcagaataaaccattttgagaaaattcagatcatccccaagagttcttggcccagtcatgaagcctcattgggggtgagcgcagcatgtccataccaggggcagtcctctttcttccgaaatgtggtgtggcttatcaaaaagaacaatacatacccaacaataaagaggagttacaataataccaaccaagaagatcttttggtaatgtgggggatccaccatcctaatgatgcagcagaacagacaaaactctatcaaaatccaaccacctatatctccgttgggacgtcaacattaaaccagagattgacaccaagaatagctactagatccaaagtaaacggacaaagtgggaggatggagttcttttggacaatcttaaaaccgaatgatgcaatcaacttcgagagtaatggaaatttcattgctccagaatatgcatacaaaattgtcaagaaaggggactcaacaattatgagaagtgaactggaatatggtaactgcaacaccaagtgtcaaactccaatgggggcgataaactctagtatgccattccacaatatacatcctctcactattggagaatgtcccaaatatgtgaaatcaaccagattagtccttgcgactgggctcagaaatagccctcaaagagaggaaagaagaaaaaagaga

>H5N1_A/Cambodia/X0123312/2013

taccatgcaaacaactcgacagagcaggttgacacgataatggaaaagaacgttactgttacacatgcccaagacatactagaaaagacacataacggaaagctctgtgacttagatggagttaggcctctaattttgagagattgtagtgtagctggatggcttctcggaaacccaatgtgtgacgaattcatcaatgtgccagaatggtcttatatagtggagaaggccaatccagtcaatgacctctgctacccaggagttttcaatgactatgaagaattgaaacacctattgagcagaataaaccattttgagaaaattcagatcatccccaagagttcttggcccagtcatgaagcctcattgggggtgagcgcagcatgtccataccaggggcagtcctcttttttccgaaatgtggtgtggcttatcaaaaagaacaatacatacccaacaataaagaggagttacaataataccaaccaagaagatcttttggtaatgtgggggatccaccatcctaatgatgcagcagcacagacaaaactctatcaaaatccaaccacctatatctccgttgggacgtcaacattaaaccagagattgacaccaagaatagctactagatccaaagtaaacggacaaagtgggaggatggagttcttttggacaatcttaaaaccgaatgatgcaatcaacttcgagagtaatggaaatttcatcgctccagaatatgcatacaaaattgtcaagaaaggggactcaacaattatgagaagtgaactggaatatggtaactgcaacaccaagtgtcaaactccaatgggggcgataaactctagtatgccattccacaatatacatcctctcactattggagaatgccccaaatatgtgaaatcaaccagattagtccttgcgactgggctcagaaatagccctcaaagagaggaaagaagaaaaaaaaga

>H5N1_A/Cambodia/X0125302/2013

taccatgcaaacaactcgacagagcaggttgacacgataatggaaaagaacgttactgttacacatgcccaagacatactagaaaagacacataacggaaagctctgtgacttagatggagttaggcctctaattttgagagattgtagtgtagctggatggcttctcggaaacccaatgtgtgacgaattcatcaatgtgccagaatggtcttatatagtggagaaggccaatccagtcaatgacctctgttacccaggagttttcaatgactatgaagaattgaaacacctattgagcagaataaaccattttgagaaaattcagatcatccccaagagttcttggcccagtcatgaagcctcattgggggtgagcgcagcatgtccataccaggggcagtcctcttttttccgaaatgtggtgtggcttatcaaaaagaacaatacatacccaacaataaagaggagttacaataataccaaccaagaagatcttttggtaatgtgggggatccaccatcctaatgatgcagcagaacagacaaaactctatcaaaatccaaccacctatatctccgttgggacgtcaacattaaaccagagactgacaccaagaatagctactagatccaaagtaaacggacgaagtgggaggatggagttcttttggacaatcttaaaaccgaatgatgcaatcaacttcgagagtaatggaaatttcatcgctccagaatatgcatacaaaattgtcaagaaaggggactcaacaattatgagaagtgaactggaatatggtaactgcaacaccaagtgtcaaactccaatgggggcgataaactctagtatgccattccacaatatacatcctctcactattggagaatgccccaaatatgtgaaatcaaccagattagtccttgcaactgggctcagaaatagccctcaaagagaggaaagaagaaaaaagaga

>H5N1_A/Cambodia/X0128304/2013

taccatgcaaacaactcgacagagcaggttgacacaataatggaaaagaacgttactgttacacatgcccaagacatactagaaaagacacataacggaaagctctgtgacttagatggagttaggcctctaattttgagagattgtagtgtagctggatggcttctcggaaacccaatgtgtgacgaattcatcaatgtgccagaatggtcttatatagtggagaaggccaatccagtcaatgacctctgttacccaggagttttcaatgactatgaagaattgaaacacctattgagcagaataaaccattttgagaaaattcagatcatccccaagagttcttggcccagtcatgaagcctcattgggggtgagcgcagcatgtccataccaggggcagtcctcttttttccgaaatgtggtgtggcttatcaaaaagaacaatacatacccaacaataaagaggagttacaataataccaaccaagaagatcttttggtaatgtgggggatccaccatcctaatgatgcagcagaacagacaaaactctatcaaaatccaaccacctatatctccgttgggacgtcaacattaaaccagagattgacaccaagaatagctactagatccaaagtaaacggactaagtgggaggatggagttcttttggacaatcttaaaatcgaatgatgcaatcaacttcgagagtaatggaaatttcatcgctccagaatatgcatacaaaattgtcaagaaaggggactcaacaattatgagaagtgaactggaatatggtaactgcaacaccaagtgtcaaactccaatgggggcgataaactctagtatgccattccacaatatacatcctctcactattggagaatgccccaaatatgtgaaatcaaccagactagtccttgcgactgggctcagaaatagccctcaaagagaggaaagaagaaaaaagaga

>H5N1_A/Cambodia/X0207301/2013

taccatgcaaacaactcgacagagcaggttgacacgataatggaaaagaacgttactgttacacatgcccaagacatactagaaaagacacataacggaaagctctgtgacttagatggagttaggcctctaattttgagagattgtagtgtagctggatggcttctcggaaacccaatgtgtgacgaattcatcaatgtgccagaatggtcttatatagtggagaaggccaatccagtcaatgacctctgctacccaggagttttcaatgactatgaagaattgaaacacctattgagcagaataaaccattttgagaaaattcagatcatccccaagagttcttggcccagtcatgaagcctcattgggggtgagcgcagcatgtccataccaggggcagtcctcttttttccgaaatgtggtgtggcttatcaaaaagaacaatacatacccaacaataaagaggagttacaataataccaaccaagaagatcttttggtaatgtgggggatccaccatcctaatgatgcagcagaacagacaaaactctatcaaaatccaaccacctatatctccgttgggacgtcaacattaaaccagagattgacaccaagaatagctactagatccaaagtaaacggacaaagtgggaggatggagttcttttggacaatcttaaaaccgaatgatgcaatcaacttcgagagtaatggaaatttcatcgctccagaatatgcatacaaaattgtcaagaaaggggactcaacaattatgagaagtgaactggaatatggcaactgcaacaccaagtgtcaaactccaatgggggcgataaactctagtatgccattccacaatatacatcctctcactattggagaatgccccaaatatgtgaaatcaaccagattagtccttgcgactgggctcagaaatagccctcaaagagaggaaagaagaaaaaagaga

>H5N1_A/Cambodia/X0212301/2013

taccatgcaaacaactcgacagagcaggttgacacgataatggaaaagaacgttactgttacacatgcccaagacatactagaaaagacacataacggaaagctctgtgacttagatggagttaagcctctaattttgagagattgtagtgttgctggatggcttctcggaaacccaatgtgtgacgaattcatcaatgtgccagaatggtcttatatagtggagaaggccaatccagtcaatgacctctgttacccaggagttttcaatgactatgaagaattgaaacacctattgagcagaataaaccattttgagaaaattcagatcatccccaagagttcttggcccagtcatgaagcctcattgggggtgagcgcagcatgtccataccaggggcagtcctcttttttccgaaatgtggtgtggcttatcaaaaagaacaatacatacccaataataaagaggagttacaataataccaaccaagaagatcttttggtaatgtgggggatccaccatcctaatgatgcagcagaacagacaaaactctatcaaaatccaaccacctatatctccgttgggacgtcaacattaaaccagagattgacaccaagaatagctactagatccaaagtaaacggacaaagtgggaggatggagttcttttggacaatcttaaaaccgaatgatgcaatcaacttcgagagtaatggaaatttcatcgctccagaatatgcatacaaaattgtcaagaaaggggactcaacaattatgagaagtgaactggaatatggtaactgcaacaccaagtgtcaaactccaatgggggcgataaactctagtatgccattccacaatatacatcctctcactattggagaatgccccaaatatgtgaaatcaaccagattagtccttgctactgggctcagaaatagccctcaaagagaggaaagaagaaaaaagaga

>H5N1_A/Cambodia/X0215301/2013

taccatgcaaacaactcgacagagcaggttgacacgataatggaaaagaacgttactgttacacatgcccaagacatactagaaaagacacataacggaaagctctgtgacttagatggagttaagcctctaattttgagagattgtagtgttgctggatggcttctcggaaacccaatgtgtgacgaattcatcaatgtgccagaatggtcttatatagtggagaaggccaatccagtcaatgacctctgttacccaggagttttcaatgactatgaagaattgaaacacctattgagcagaataaaccattttgagaaaattcagatcatccccaagagttcttggcccagtcatgaagcctcattgggggtgagcgcagcatgtccataccaggggcagtcctcttttttccgaaatgtggtgtggcttatcaaaaagaacaatacatacccaataataaagaggagttacaataataccaaccaagaagatcttttggtaatgtgggggatccaccatcctaatgatgcagcagaacagacaaaactctatcaaaatccaaccacctatatctccgttgggacgtcaacattaaaccagagattgacaccaagaatagctactagatccaaagtaaacggacaaagtgggaggatggagttcttttggacaatcttaaaaccgaatgatgcaatcaacttcgagagtaatggaaatttcatcgctccagaatatgcatacaaaattgtcaagaaaggggactcaacaattatgagaagtgaactggaatatggtaactgcaacaccaagtgtcaaactccaatgggggcgataaactctagtatgccattccacaatatacatcctctcactattggagaatgccccaaatatgtgaaatcaaccagattagtccttgctactgggctcagaaatagccctcaaagagaggaaagaagaaaaaagaga

>H5N1_A/Cambodia/X0219301/2013

taccatgcaaacaactcgacagagcaggttgacacgataatggaaaagaacgttactgttacacatgcccaagacatactagaaaagacacataacggaaagctctgtgacttagatggagttaggcctctaattttgagagattgtagtgtagctggatggcttctcggaaacccaatgtgtgacgaattcatcaatgtgccagaatggtcttatatagtggagaaggccaatccagtcaatgacctctgttacccaggagttttcaatgactatgaagaattgaaacacctattgagcagaataaaccattttgagaaaattcagatcatccccaagagttcttggcccagtcatgaagcctcattgggggtgagtgcagcatgtccataccaggggcagtcctcttttttccgaaatgtggtgtggcttatcaaaaagaacaatacatacccaacaataaagaagagttacaataataccaaccaagaagatcttttggtaatgtgggggatccaccatcctaatgatgcagcagaacagacaaaactctatcaaaatccaaccacctatatctccgttgggacgtcaacattaaaccagagattgacaccaagaatagctactagatccaaagtaaacggacaaagtgggaggatggagttcttttggacaatcttaaaaccgaatgatgcaatcaacttcgagagtaatggaaatttcatcgctccagaatatgcatacaaaattgtcaagaaaggggactcaacaattatgagaagtgaactggaatatggtaactgcaacaccaagtgtcaaactccaatgggggcgataaactctagtatgccattccacaatatacatcctctcactattggagaatgccccaaatatgtgaaatcaaccagattagtccttgcgactgggctcagaaatagccctcaaagagaggaaagaagaaaaaagaga

>H5N1_A/Cambodia/X0502302/2013

taccatgcaaacaactcgacagagcaggttgacacgataatggaaaagaacgttactgttacacatgcccaagacatactagaaaagacacataacggaaagctctgtgacttagatggagttaggcctctaattttgagagattgtagtgtagctggatggcttctcggaaacccaatgtgtgacgaattcatcaatgtgccagaatggtcttatatagtggagaaggccaatccagtcaatgacctctgttacccaggagttttcaatgactatgaagaattgaaacacctattgagcagaataaaccattttgagaaaattcagatcatccccaagagttcttggcccagtcatgaagcctcattgggggtgagcgcagcatgtccataccaggggcagtcctcttttttccgaaatgtggtatggcttatcaaaaagaacaatacatacccaacaataaagaggagttacaataataccaaccaagaagatcttttggtaatgtgggggatccaccatcctaatgatgcagcagaacagacaaaactctatcaaaatccaaccacctatatctccgttgggacgtcaacattaaaccagagattgacaccaagaatagctactagatccaaagtaaacggacaaagtgggaggatggagttcttttggacaatcttaaaaccgaatgatgcaatcaacttcgagagtaatggaaatttcatcgctccagaatatgcatacaaaattgtcaagaaaggggactcaacaattatgagaagtgaactggaatatggtaactgcaacaccaagtgtcaaactccaatgggggcgataaactctagtatgccattccacaatatacatcctctcactattggagaatgccccaaatatgtgaaatcaaccagattagtccttgcgactgggctcagaaatagccctcaaagagaggaaagaagaaaaaagaga

>H5N1_A/Cambodia/X0628313/2013

taccatgcaaacaactcgacagagcaggttgacacgataatggaaaagaacgttactgttacacatgcccaagacatactagaaaagacacataacggaaagctctgtgacttagatggagttaggcctctaattttgagagattgtagtgtagctggatggcttctcggaaacccaatgtgtgacgaattcatcaatgtgccagaatggtcttatatagtggagaaggccaatccagtcaatgacctctgctacccaggagttttcaatgactatgaagaattgaaacacctattgagcagaatcaaccattttgagaaaattcagatcatccccaagagttcttggcccagtcatgaagcctcattgggggtgagcgcagcatgtccataccaggggcagtcttcttttttccgaaatgtggtgtggcttgtcaaaaagaacaatacatacccaacaataaagaggagttacaataataccaaccaagaagatcttttggtaatgtgggggatccaccatcctaatgatgcagcagaacagacaaaactctatcaaaatccaaccacctatattttcgttgggacgtcaacattaaaccagagattgacaccaagaatagctactagatccaaagtaaacggacaaagtgggaggatggagttcttttggacaatcttaaaaccgaatgatgcaatcaacttcgagagtaatggaaatttcatcgctccagaatatgcatacaaaattgtcaagaaaggggactcaacaattatgagaagtgaactggaatatggtaactgcaacaccaagtgtcaaactccaatgggggcgataaactctagtatgccattccacaatatacatcctctcactattggagaatgccccaaatatgtgaaatcaaccagattagttcttgcgactgggctcagaaatagccctcaaagagaggaaagaagaaaaaagaga

>H5N1_A/Cambodia/X0808305/2013

taccatgcaaacaactcgacagagcaggttgacacgataatggaaaagaacgttactgttacacatgcccaagacatactagaaaagacacataacggaaagctctgtgacttagatggagttaggcctctaattttgagagatggtagtgtagctggatggcttctcggaaacccaatgtgtgacgaattcatcaatgtgccagaatggtcttatatagtggagaaggccaatccagtcaatgacctctgctacccaggagttttcaatgactatgaagaattgaaacacctattgagcagaataaaccattttgagaaaattcagatcatccccaagaattcttggcccagtcatgaagcctcattgggggtgagcgcagcatgtccataccaggggcagtcctcttttttccgaaatgtggtgtggcttatcaaaaagaacaatacatacccaacaataaagaggagttacaataataccaaccaagaagatcttttggtaatgtgggggatccaccatcctaatgatgcagtagaacagacaaaactctatcaaaatccaaccacctatatctccgttgggacgtcaacattaaaccagagattgacaccaagaatagctactagatccaaagtaaacggacaaagtgggaggatggagttcttttggacaatcttaaaaccgaatgatgcaatcaacttcgagagtaatggaaatttcatcgctccagaatatgcatacaaaattgtcaagaaaggggactcaacaattatgagaagtgaactggaatatggtaactgcaacaccaagtgtcaaactccaatgggggcgataaactctagtatgccattccacaatatacatcctctcactattggagaatgccccaaatatgtgaaatcaaccagattagtccttgcgactgggctcagaaatagccctcaaagagaggaaagaagaaaaaagaga

>H5N1_A/Cambodia/X0810301/2013

taccatgcaaacaactcgacagagcaggttgacacgataatggaaaagaacgttactgttacacatgcccaagacatactagaaaagacacataacggaaagctctgtgacttagatggagttaggcctctaattttgagagattgtagtgtagctggatggcttctcggaaacccaatgtgtgacgaattcatcaatgtgccagaatggtcttatatagtggagaaggccaatccagtcaatgacctctgctacccaggagttttcaatgactatgaagaattgaaacacctattgagcagaataaaccattttgagaaaattcagatcatccccaagaattcttggcccagtcatgaagcctcattgggggtgagcgcagcatgtccataccaggggcagtcctcttttttccgaaatgtggtgtggcttatcaaaaagaacaatacatacccaacaataaagaggagttacaataataccaaccaagaagatcttttggtaatgtgggggatccaccatcctaatgatgcagtagaacagacaaaactctatcaaaatccaaccacctatatctccgttgggacgtcaacattaaaccagagattgacaccaagaatagctactagatccaaagtaaaaggactaagtgggaggatggagttcttttggacaatcttaaaaccgaatgatgcaatcaacttcgagagtaatggaaatttcatcgctccagaatatgcatacaaaattgtcaagaaaggggactcaacaattatgagaagtgaactggaatatggtaactgcaacaccaagtgtcaaactccaatgggggcgataaactctagtatgccattccacaatatacatcctctcactattggagaatgccccaaatatgtgaaatcaaccagattagtccttgcgactgggctcagaaatagccctcaaagagaggaaagaagaaaaaagaga

>H5N1_A/Cambodia/X0817302/2013

taccatgcaaacaactcgacagagcaggttgacacgataatggaaaagaacgttactgttacacatgcccaagacatactagaaaagacacataacggaaagctctgtgacttagatggagttaggcctctaattttgagagattgtagtgtagctggatggcttctcggaaacccaatgtgtgacgaattcatcaatgtgccagaatggtcttatatagtggagaaggccaatccagtcaatgacctctgctacccaggagttttcaatgactatgaagaattgaaacacctattgagcagaataaaccattttgagaaaattcagatcatccccaagaattcttggcccagtcatgaagcctcattgggggtgagcgcagcatgtccataccaggggcagtcctcttttttccgaaatgtggtgtggcttatcaaaaagaacaatacatacccaacaataaagaggagttacaataataccaaccaagaagatcttttggtaatgtgggggatccaccatcctaatgatgcagtagaacagacaagactctatcaaaatccaaccacctatatctccgttgggacgtcaacattaaaccagagattgacaccaagaatagctactagatccaaagtaaacggacaaagtgggaggatggagttcttttggacaatcttaaaaccgaatgatgcaatcaacttcgagagtaatggaaatttcatcgctccagaatatgcatacaaaattgtcaagaaaggggactcaacaattatgagaagtgaactggaatatggtaactgcaacaccaagtgtcaaactccaatgggggcgataaactctagtatgccattccacaatatacatcctctcactattggagaatgccccaaatatgtgaaatcaaccagattagtccttgcgactgggctcagaaatagccctcaaagagaggaaagaagaaaaaagaga

>H5N1_A/Cambodia/X0828324/2013

taccatgcaaacaactcgacagagcaggttgacacgataatggaaaagaacgttactgttacacatgcccaagacatactagaaaagacacataacggaaagctctgtgacttaaatggagttaggcctctaattttgagagattgtagtgtggctggatggcttctcggaaacccaatgtgtgacgaattcatcaatgtgccagaatggtcttatatagtggagaaggccaatccagtcaatgacctctgttacccaggagttttcaatgactatgaagaattgaaacacctattgagcagaataaaccattttgagaaaattcagatcatccccaagagttcttggcccagtcatgaagcctcattgggggtgagcgcagcatgtccataccaggggcagtcctcttttttccgaaatgtggtgtggcttatcaaaaagaacaatacatacccaacaataaagaggagttacaataataccaaccaagaagatcttttggtaatgtgggggatccaccatcctaatgatgcagcagaacagacaaaactctatcaaaatccaaccacctatatctccgttgggacgtcaacattaaaccagagattgacaccaagaatagctactagatccaaagtaaacggacaaagtgggaggatggagttcttttggacaatcttaaaaccgaatgatgcaatcaacttcgagagtaatggaaatttcatcgctccagaatatgcatacaaaattgtcaagaaaggggactcaacaattatgagaagtgaactggaatatggtaactgcaacaccaagtgtcaaactccaatgggggcgataaactctagtatgccattccacaatatacatcctctcactattggagaatgccccaaatatgtgaaatcaaccagattagtccttgcgactgggctcagaaatagccctcaaagagaggaaagaagaaaaaagaga

>H5N1_A/Cambodia/X0913301/2013

taccatgcaaacaactcgacagagcaggttgacacgataatggaaaagaacgttactgttacacatgcccaagacatactagaaaaaacacataacggaaagctctgtgacttagatggagttaggcctctaattttgagagattgtagtgtagctggatggcttctcggaaacccaatgtgtgacgaattcatcaatgtgccagaatggtcttatatagtggagaaggccaatccagtcaatgacctctgctacccaggagttttcaatgactatgaagaattgaaacacctattgagcagaataaaccattttgagaaaattcagatcatccccaagagttcttggcccagtcatgaagcctcattgggggtgagcgcagcatgtccataccaggggcagtcctcttttttccgaaatgtggtgtggcttatcaaaaagaacaatacatacccaacaataaaaaggagttacaataataccaaccaagaagatcttttggtaatgtgggggatccaccatcctaatgatgcagcagaacagacaaaactctatcaaaatccaaccacctatatctccgttgggacgtcaacattaaaccagagattgacaccaagaatagctactagatccaaagtaaacggacaaagtgggaggatggagttcttttggacaatcttaaaaccgaatgatgcaatcaacttcgagagtaatggaaatttcatcgctccagaatatgcatacaaaattgtcaagaaaggggactcaacaattatgagaagtgaactggaatatggtaactgcaacaccaagtgtcaaactccaatgggggcgataaactctagtatgccattccacaatatacatcctctcactattggagaatgccccaaatatgtgaaatcaaccagattagtccttgcgactgggctcagaaatagccctcaaagagaggaaagaagaaaaaagaga

>H5N1_A/Cambodia/X0916322/2013

taccatgcaaacaactcgacagagcaggttgacacgataatggaaaagaacgttactgttacacatgcccaagacatactagaaaagacacataacggaaagctctgtgacttagatggagttaggcctctaattttgagagattgtagtgtagctggatggcttctcggaaacccaatgtgtgacgaattcatcaatgtgccagaatggtcttatatagtggagaaggccaatccagtcaatgacctctgctacccaggagttttcaatgactatgaagaattgaaacacctattgagcagaataaaccattttgagaaaattcagatcatccccaagaattcttggcccagtcatgaagcctcattgggggtgagcgcagcatgtccataccaggggcagtcctcttttttccgaaatgtggtgtggcttatcaaaaagaacaatacatacccaacaataaagaggagttacaataataccaaccaagaagatcttttggtaatgtgggggatccaccatcctaatgatgcagcagaacaaacaaaactctatcaaaatccaaccacctatatctccgttgggacgtcaacattaaaccagagattgacaccaagaatagctactagatccaaagtaaacggacaaagtgggaggatggagttcttttggacaatcttaaaaccgaatgatgcaatcaacttcgagagtaatggaaatttcatcgctccagaatatgcatacaaaattgtcaagaaaggggactcaacaattatgagaagtgaactggaatatggtaactgcaacaccaagtgtcaaactccaatgggggcgataaactctagtatgccattccacaatatacatcctctcactattggagaatgccccaaatatgtgaaatcaaccagattagtccttgcgactgggctcagaaatagccctcgaagagaggaaagaagaaaaaagaga

>H5N1_A/Cambodia/X1024307/2013

taccatgcaaacaactcgacagagcaggttgacacgataatggaaaagaacgttactgttacacatgcccaagacatactagaaaagacacataacggaaagctctgtgacttagatggagttaggcctctaattttgagagattgtagtgtagctggatggcttctcggaaacccaatgtgtgacgaattcatcaatgtgccagaatggtcttatatagtggagaaggccaatccagtcaatgacctctgctaccccggagttttcaatgactatgaagaattgaaacacctattgagcagaataaaccattttgagaaaattcagatcatccccaaggattcttggcccagtcatgaagcctcattgggggtgagcgcagcatgtccataccaggggcagtcctcttttttccgaaatgtggtgtggcttatcaaaaagaacaatacatacccaacaataaagaggagttacaataataccaaccaagaagatcttttggtaatgtgggggatccaccatcctaatgatgcagcagaacagacaaaactctatcaaaatccaaccacctatatctctgttgggacgtcaacattaaaccagagattgacaccaagaatagctactagatccaaagtaaacggacaaagtgggaggatggagttcttttggacaatcttaaaaccgaatgatgcaatcaacttcgagagtaatggaaatttcatcgctccagaatatgcatacaaaattgtcaagaaaggggactcaacaattatgagaagtgaactggaatatggtaactgcaacaccaagtgtcaaactccaatgggggcgataaactctagtatgccattccacaatatacatcctctcactattggagaatgccccaaatatgtgaaatcaaccagattagtccttgcgactgggctcagaaatagccctcaaagagaggaaagaagaaaaaagaga

>H5N1_A/Cambodia/X1030304/2013

taccatgcaaacaactcgacagagcaggttgacacgataatggaaaagaacgttactgttacacatgcccaagacatactagaaaagacacataatggaaagctctgtgacttagatggagttaggcctctaattttgagagattgtagtgtagctggatggcttctcggaaacccaatgtgtgacgaattcatcaatgtgccagaatggtcttatatagtggagaaggccaatccagtcaatgacctctgctacccaggagttttcaatgactatgaagaattgaaacacctattgagcagaataaaccattttgagaaaattcagatcatccccaagaattcttggcccagtcatgaagcctcattgggggtgagcgcagcatgtccatacctggggcagtcctcttttttccgaaatgtggtgtggcttatcaaaaagaacaatacatacccaacaataaagaggagttacaataataccaaccaagaagatcttttggtaatgtgggggatccaccatcctaatgatgcagcagaacagacaaaactctatcaaaatccaaccacctatatctccgttgggacgtcaacattaaaccagagattgacaccaagaatagctactagatccaaagtaaacggacaaagtgggaggatggagttcttttggacaatcttaaaaccgaatgatgcaatcaacttcgagagtaatggaaatttcatcgctccagaatatgcatacaaaattgtcaagaaaggggactcaacaattatgagaagtgaactggaatatggtaactgcaacaccaagtgtcaaactccaatgggggcgataaactctagtatgccattccacaatatacatcctctcactattggagaatgccccaaatatgtgaaatcaaccagattagtccttgcgactgggctcagaaatagccctcaaagagaggaaagaagaaaaaagaga

>H5N1_A/Cambodia/X1107305/2013

taccatgcaaacaactcgacagagcaggttgacacgataatggaaaagaacgttactgttacacatgcccaagacatactagaaaagacacataacggaaagctctgtgacttagatggagttaggcctctaattttgagagattgtagtgtggctggatggcttctcggaaacccaatgtgtgacgaattcatcaatgtgccagaatggtcttatatagtggagaaggccaatccagtcaatgacctctgttacccaggagttttcaatgactatgaagaattgaaacacctattgagcagaataaaccattttgagaaaattcagatcatccccaagagttcttggcccagtcatgaagcctcattgggggtgagcgcagcatgtccataccaggggcagtcttcttttttccgaaatgtggtgtggcttatcaaaaagaacaatacatacccaacaataaagaggagttacaataataccaaccaagaagatcttttgttaatgtgggggatccaccatcctaatgatgcagcagaacagacaaaactctatcaaaatccaaccacctatatctccgttgggacgtcaacattaaaccagagattgacaccaagaatagctactagatccaaagtaaacggacaaagtgggaggatggagttcttttggacaatcttaaaaccgaatgatgcaatcaacttcgagagtaatggaaatttcatcgctccagaatatgcatacaaaattgtcaagaaaggggactcaacaattatgagaagtgaactggaatatggtaactgcaacaccaagtgtcaaactccagtgggggcgataaactctagtatgccattccacaatatacatcctctcactattggagaatgccccaaatatgtgaaatcaaccagattagtccttgcgactgggctcagaaatagccctcaaagagaggaaagaagaaaaaagaga

>H5N1_A/Cambodia/X1108306/2013

taccatgcaaacaactcgacagagcaggttgacacgataatggaaaagaacgttactgttacacatgcccaagacatactagaaaagacacataacggaaagctctgtgacttagatggagttaggcctctaattttgagagattgtagtgtggctggatggcttctcggaaacccaatgtgtgacgaattcatcaatgtgccagaatggtcttatatagtggagaaggccaatccagtcaatgacctctgttacccaggagttttcaatgactatgaagaattgaaacacctattgagcagaataaaccattttgagaaaattcagatcatccccaagagttcttggcccagccatgaagcctcattgggggtgagcgcagcatgtccataccaggggcagtcttcttttttccgaaatgtggtgtggcttatcaaaaagaacaatacatacccaacaataaagaggagttacaataataccaaccaagaagatcttttggtaatgtgggggatccaccatcctaatgatgcagcagaacagacaaaactctatcaaaatccaaccacctatatctccgttgggacgtcaacattaaaccagagattgacaccaagaatagctactagatccaaagtaaacggacaaagtgggaggatggagttcttttggacaatcttaaaaccgaatgatgcaatcaacttcgagagtaatggaaatttcatcgctccagaatatgcatacaaaattgtcaagaaaggggactcaacaattatgagaagtgaactggaatatggtaactgcaacaccaagtgtcaaactccagtgggggcgataaactctagtatgccattccacaatatacatcctctcactattggagaatgccccaaatatgtgaaatcaaccagattagtccttgcgactgggctcagaaatagccctcaaagagaggaaagaagaaaaaagaga

>H5N1_A/Cambodia/Y0203301/2014

taccatgcaaacaactcgacagagcaggttgacacgataatggaaaagaacgttagtgttacacatgcccaagacatactagaaaagacacataacggaaagctctgtgacttagatggagttaggcctctaattttgagagattgtagtgtggctggatggcttctcggaaacccaatgtgtgacgaatttatcaatgtgccagaatggtcttatatagtggagaaggccaatccagtcaatgacctctgttacccaggagttttcaatgactatgaagaattgaaacacctattgagcagaataaaccattttgagaaaattcagatcatccccaagagttcttggcccagtcatgaagcctcattgggggtgagcgcagcatgtccataccaggggcagtcctcttttttccgaaatgtggtgtggcttatcaaaaagaacaatacatacccaacaataaagaggagttacaataataccaaccaagaagatcttttggtaatgtgggggatccaccatcctaatgatgcagcagaacagacaaaactctatcaaaatccaaccacctatatctccgttgggacgtcaacattaaaccagagattgacaccaagaatagctactagatccaaagtaaacggacgaagtgggaggatggagttcttttggacaatcttaaaaccgaatgatgcaatcaacttcgagagtaatggaaatttcatcgctccagaatatgcatacaaaattgtcaagaaaggggactcaacaattatgagaagtgaactggagtatggtaactgcaacaccaagtgtcaaactccaatgggggcgataaactctagtatgccattccacaatatacatcctctcactattggagaatgccccaaatatgtgaaatcaaccagattagtccttgcgactgggctcagaaatagccctcaaagagaggaaagaagaaaaaagaga

>H5N1_A/Cambodia/Y0208301/2014

ttccacgcaaacatctcgacagagcaggttgacacgataatggaaaagaacgttactgttacacatgcccaagacatactagaaaagacacataacggaaagctctgtgacttagatggagttaggcctctaattttgagagattgtagtgtagctggatggcttctcggaaacccaatgtgtgacgaattcatcaatgtgccagaatggtcttatatagtggagaaggccaatccagtcaatgacctctgctacccaggagttttcaatgactatgaagaattgaaacacctattgagcagaataaaccattttgagaaaattcagatcatccccaagagttcttggcccagtcatgaagcctcattgggggtgagcgcagcatgtccataccaggagcagccctcttttttccgaaatgtggtgtggcttatcaaaaagaacaatacatacccaacaataaagaggagttacaataataccaaccaagaagatcttttggtaatgtggggaatccaccatccaaatgatgcagcagaacagacaaaactctatcaaaatccaaccacctatatctccgttgggacgtcaacattaaaccagagattgacaccaagaatagctactagatccaaagtaaacggacgaagtgggaggatggagttcttttggacaatcttaaaaccgaatgatgcaatcaacttcgagagtaatggaaatttcatcgctccagaatatgcatacaaaattgtcaagaaaggggactcaacaattatgagaagtgaactggaatatggtaactgcaacaccaagtgtcaaactccgatgggggcgataaactctagtatgccattccacaatatacatcctctcactattggagaatgccccaaatatgtgaaatcaaccagattagtccttgcgactgggctcagaaatagccctcaaagagaggaaagaagaaaaaagaga

>H5N1_A/Cambodia/Y0214301/2014

taccatgcaaacaactcgacagagcaggttgacacgataatggaaaagaacgttactgttacacatgcccaagacatactagaaaagacacataacggaaagctctgtgacttagatggagttaggcctctaattttgagagattgtagtgtggctggatggcttctcggaaacccaatgtgtgacgaattcatcaatgtgccagaatggtcttatatagtggagaaggccaatccagtcaatgacctctgttacccaggagttttcaatgactatgaagaattgaaacacctattgagcagaataaaccattttgagaaaattcagatcatccccaagagttcttggcccagtcatgaagcctcattgggagtgagcgcagcatgtccataccaggggcagtcttcttttttccgaaatgtggtgtggcttatcaaaaagaacaatacatacccaacaataaagaggagttacaataataccaaccaagaagatcttttggtaatgtgggggatccaccatcctaatgatgcagcagaacagacaaaactctatcaaaatccaaccacctatatctccgttgggacgtcaacattaaaccagagattgacaccaagaatagctactagatccaaagtaaacggacaaagtgggaggatggagttcttttggacaatcttaaaaccgaatgatgcaatcaacttcgagagtaatggaaatttcatagctccagaatatgcatacaaaattgtcaagaaaggggactcaacaattatgagaagtgaactggaatatggtaactgcaacaccaagtgtcaaactccgatgggggcgataaactctagtatgccattccacaatatacatcctctcactattggagaatgccccaaatatgtgaaatcaaccagattagtccttgcgactgggctcagaaatagccctcaaagagaggaaagaagaaaaaagaga

>H5N1_A/Cambodia/Y0219302/2014

tatcatgcaaataactcgacagagcaggtggacacaataatggaaaagaacgttactgttacacatgcccaagacatactggaaaagacacacaacgggaagctctgcgatctaaatggagtgaagcctctgattttaaaagattgtagtgtagcaggatggctcctcggaaatccattgtgtgacgaattcaccaatgtgccagagtggtcttacatagtagagaaggccaatccagccaatgacctctgttacccagggaatttcaacgattatgaagaattgaaacacctattgagcaggataaaccattttgagaaaatacagatcatccccaaagattcttggtcagatcatgaagcctcattgggggtgagcgcagcatgttcataccagggaaattcctccttcttcagaaatgtggtatggcttctcaaaaaggacaatgcatacccaacaataaagaaaggctacaataataccaaccgagaagatctcttgatactgtgggggatccaccatcctaatgatgaggcagagcagacaaggctctaccaaaacccaactacctatatttccattgggacttcaacactaaaccagagattggtaccaaaaatagccactagatccaaaataaacgggcaaagtggcaggatagatttcttctggacaattttaaaaccgaatgacgcaatccacttcgagagtaatggaaatttcattgctccagaatatgcatacaaaattgtcaagaagggagactccacaatcatgagaagtgaagtggaatatggtaactgcagcaccaggtgtcagactccaataggagcgataaactctagtatgccattccacaacatacaccctctcaccatcggagaatgtcccaaatatgtgaaatcaaacaaattagtccttgcaactgggctcagaaatagtcctcaaagagagagGGGaagaagaaaaaga

>H5N1_A/Cambodia/Y0304301/2014

taccatgcaaacaactcgacagagcaggttgacacgataatggaaaagaacgttactgttacacatgcccaagacatactagaaaagacacataacggaaagctctgtgacttagatggagttaggcctctaattttgagagattgtagtgtggctggatggcttctcggaaacccaatgtgtgacgaattcatcaatgtgccagaatggtcttatatagtggagaaggccaatccagtcaatgacctctgttacccaggagttttcaatgactatgaagaattgaaacacctattgagcagaataaaccattttgagaaaattcagatcatccccaagagttcttggcccagtcatgaagcctcattgggagtgagcgcagcatgtccataccaggggcagtcttcttttttccgaaatgtggtgtggcttatcaaaaagaacaatacatacccaacaataaagaggagttacaataataccaaccaagaagatcttttggtaatgtgggggatccaccatcctaatgatgcagcagaacagacaaaactctatcaaaatccaaccacctatatctccgttgggacgtcaacattaaaccagagattgacaccaagaatagctactagatccaaagtaaacggacaaagtgggaggatggagttcttttggacaatcttaaaaccgaatgatgcaatcaacttcgagagtaatggaaatttcatagctccagaatatgcatacaaaattgtcaagaaaggggactcaacaattatgagaagtgaactggaatatggtaactgcaacaccaagtgtcaaactccagtgggggcgataaactctagtatgccattccacaacatacatcctctcactattggagaatgccccaaatatgtgaaatcaaccagattagtccttgcgactgggctcagaaatagccctcaaagagaggaaagaagaaaaaagaga

>H5N1_A/Cambodia/Y0305306/2014

taccatgcaaacaactcgacagagcaggttgacccgataatggaaaagaacgttactgttacacatgcccaagacatcctagaaaagacacataacggaaagctctgtgacttagatggagttaggcctctaattttgagagattgtagtgtggctggatggcttctcggaaacccaatgtgtgacgaattcatcaatgtgccagaatggtcttatatagtggagaaggccaatccagccaatgacctctgttacccaggagttttcaatgactatgaagaattgaaacacctattgagcagaataaaccattttgagaaaattcagatcatccccaagagttcttggcccagtcatgaagcctcattgggggtgagcgcagcatgtccataccaggggcagtcttcttttttccgaaatgtggtgtggcttatcaaaaagaacaatacatacccaacaataaagaggagttacaataataccaaccaagaagatcttttggtaatgtgggggatccaccatcctaatgatgcagcagaacagacaaaactctatcaaaatccaaccacctatatctccgttgggacgtcaacattaaaccagagattgacaccaagaatagctactagatccaaagtaaacggacaaagtgggaggatggagttcttttggacaatcttaaaaccgaatgatgcaatcaacttcgagagtaatggaaatttcatcgctccagaatatgcatacaaaattgtcaagaaaggggactcaacaattatgagaagtgaactggaatatggtaactgcaacaccaagtgtcaaactccagtgggggcgataaactctagtatgccattccacaatatacatcctctcactattggagaatgccccaaatatgtgaaatcaaccagattagtccttgcgactgggctcagaaatagccctcaaagagaggaaagaagaaaaaagaga

>H5N1_A/Cambodia/Y0307302/2014

taccatgcaaacaactcgacagagcaggttgacacaataatggaaaagaacgttactgttacacatgcccaagacatactagaaaagacacataacggaaagctctgtgacttagatggagttaggcctctaattttgagagattgtagtgtggctggatggcttctcggaaacccaatgtgtgacgaattcatcaatgtgccagaatggtcttatatagtggagaaggccaatccagtcaatgacctctgttacccaggagttttcaatgactatgaagaattgaaacacctattgagcagaataaaccattttgagaaaattcagatcatccccaagagttcttggcccagtcatgaagcctcattgggggtgagcgcagcatgtccataccaggggcagtcttcttttttccgaaatgtggtgtggcttatcaaaaagaacaatacatacccaacaataaagaggagttacaataataccaaccaagaagatcttttggtaatgtgggggatccaccatcctaatgatgcagcagaacagacaaaactctatcaaaatccaaccacctatatctccgttgggacgtcaacattaaaccagagattgacaccaagaatagctactagatccaaagtaaacggacaaagtgggaggatggagttcttttggacaatcttaaaaccgaatgatgcaatcaacttcgagagtgatggaaatttcatcgctccagaatatgcatacaaaattgtcaagaaaggggactcaacaattatgagaagtgaactggaatatggtaactgcaacaccaagtgtcaaactccagtgggggcgataaactctagtatgccattccacaatatacatcctctcactattggagaatgccccaaatatgtgaaatcaaccagattagtccttgcgactgggctcagaaatagccctcaaagagaggaaagaagaaaaaagaga

>H5N1_A/Cambodia/Y0314301/2014

taccatgcaaacaactcgacagagcaggttgacacgataatggaaaagaacgttactgttacacatgcccaagacatactagaaaagacacataacggaaagctctgtgacttagatggagttaggcctctaattttgagagattgtagtgtggctggatggcttctcggaaacccaatgtgtgacgaattcatcaatgtgccagaatggtcttatatagtggagaaggccaatccagtcaatgacctctgttacccaggagttttcaatgactatgaagaattgaaacacctattgagcagaataaaccattttgagaaaattcagatcatccccaagagttcttggcccagtcatgaagcctcattgggggtgagcgcagcatgtccataccaggggcagtcttcttttttccgaaatgtggtgtggcttatcaaaaagaacaatacatacccaacaataaagaggagttacaataataccaaccaagaagatcttttggtaatgtgggggatccaccatcctaatgatgcagcagaacagacaaaactctatcaaaatccaaccacctatatctccgtcgggacgtcaacattaaaccagagattgacaccaagaatagctactagatccaaagtaaacggacaaagtgggaggatggagttcttttggacaatcttaaaaccgaatgatgcaatcaacttcgagagtaatggaaatttcatcgctccagaatatgcatacaaaattgtcaagaaaggggactcaacaattatgagaagtgaactggaatatggtaactgcaacaccaagtgtcaaactccagtgggggcgataaactctagtatgccattccacaatatacatcctctcactattggagaatgccccaaatatgtgaaatcaaccagattagtccttgcgactgggctcagaaatagccctcaaagagaggaaagaagaaaaaagaga

>H5N1_A/China/2006

taccatgcaaacaactcgacagagcaggttgacacaataatggaaaagaacgttactgttacacatgcccaagatatactggaaaagacacacaacgggaagctctgcgatctagatggagtgaagcctctgattttaagagattgtagtgtagctggatggctcctcggaaacccaatgtgtgacgaattcatcaatgtgccggaatggtcttacatagtggagaaggccaacccagccaatgacctctgttacccagggaatttcaacgactatgaagaactgaaacacctattgagcagaataaaccattttgagaaaattcagatcatccccaaaagttcttggtccgatcatgaagcctcatcaggggtgagctcagcatgtccataccagggaacgccctcctttttcagaaatgtgatatggcttatcaaaaagaacaatacatacccaacaataaagagaagctacaataataccaaccaggaagatcttttgatactgtgggggattcatcattctaatgatgcggcagagcagacaaagctctatcaaaacccaaccacctatatttccgttgggacatcaacacttaaccagagattggtaccgaaaatagctactagatccaaagtaaacgggcaaagtggaaggatggatttcttctggacaattttaaaaccaaatgatgcaatcaacttcgagagtaatggaaatttcattgctccagaatatgcatacaaaattgtcaagaaaggggactcagcaattctgaaaagtgaagtggaatatggtaactgcaacaccaagtgtcaaactccaataggggcgataaactctagtatgccattccacaacatacaccctctcactatcggggaatgccccaaatatgtgaaatcaaacaaattagtccttgcgactgggctcagaaatagtcctctaagagaaagGGGaagaagaaaaaga

>H5N1_A/China/GD01/2006

taccatgcaaacaactcgacagagcaggttgacacaataatggaaaagaacgttactgttacacatgcccaagacatactggaaaagacacacaacgggaagctctgcgatctagatggagtgaagcctctgattttaagagattgtagtgtagctggatggctcctcggaaacccaatgtgtgacgaattcatcaatgtgccggaatggtcttacatagtggagaaggccaacccagccaatgacctctgttacccagggaatttcaacgactatgaagaactgaaacacctattgagcagaataaaccattttgagaaaattcagatcatctccaaaagttcttggtccgatcatgaagcctcatcaggggtgagctcagcatgtccataccagggaacgccctcctttttcagaaatgtggtatggcttatcaaaaagaacaatacatacccaacaataaagagaagctacaataataccaaccaggaagatcttttgatactgtggggaattcatcattctaataatgcggcagagcagacaaagctctatcaaaacccaaccacctatatttccgttgggacatcaacactaaacctgagattggtaccaaaaatagctactagatccaaagtaaacgggcaaagtggaaggatggatttcttctggacaattttaaaaccgaatgatgcaatcaacttcgagagtaatggaaatttcattgctccagaatatgcatacaaaattgtcaagaaaggggactcagcaattatgaaaagtgaagtggaatatggtaactgcaacaccaagtgtcaaactccaataggggcgataaactctagtatgccattccacaacatacaccctctcaccatcggggaatgccccaaatatgtgaaatcaaacaaattagtccttgcgactgggctcagaaatagtcctctaagagagagGGGaagaagaaaaaga

>H5N1_A/Djibouti/5691NAMRU3/2006

taccatgcaaacaactcgacagagcaggttgacacaataatggaaaagaacgtcactgttacacacgcccaagacatactggaaaagacacacaacgggaaactctgcgatctagatggagtgaagcctctaattttaagagattgtagtgtagctggatggctcctcgggaacccaatgtgtgacgaattcctcaatgtgccggaatggtcttacatagtggagaagatcaatccagccaatgacctctgttacccagggaatttcaacgactatgaagaactgaaacacctattgagcagaataaaccattttgagaaaattcagatcatccccaaaagttcttggtcagatcatgaagcctcatcgggagtgagctcagcatgtccataccagggaagatcctccttttttagaaatgtggtatggcttatcaaaaaggacaatgcatacccaacaataaagagaagttacaataataccaaccaagaagatcttttggtactgtgggggattcaccatccaaatgatgcggcagagcagacaaggctctatcaaaacccaactacctatatttccgttgggacatcaacactaaaccagagattggtaccaaaaatagctactagatctaaggtaaacgggcaaagtggaaggatggagttcttttggacaattttaaaatcgaatgatgcaataaactttgagagtaatggaaatttcattgctccagaaaatgcatacaaaattgtcaagaaaggggactcaacaattatgaaaagtgagttggaatatggtaactgcaacaccaagtgtcaaactccaataggggcgataaactccagtatgccattccacaacatccaccctctcaccatcggggaatgccccaaatatgtgaaatcaaacagattagtccttgctactgggctcagaaatagccctcaaggagagagaagaagaaaaaagaga

>H5N1_A/Egypt/0636-NAMRU3/2007

taccatgcaaacaactcgacagagcaggttgacacaataatggaaaagaacgtcactgttacacacgcccaagacatactggaaaagacacacaacgggaaactctgcaatctaaatggagtgaagcctctaattttaagagattgtagtgtagctggatggctcctcgggaacccaatgtgtgacgaattcctcaatgtgccggaatggtcttacatagtggagaagatcaatccagccaatgacctctgttatccagggaatttcaacgactatgaagaactgaaacacctattgagcagaataaaccattttgagaaaattcagatcatccccaaaaattcttggtcagatcatgaagccGGGtcaggagtgagctcagcatgtccataccagggaagatcctccttttttagaaatgtggtatggcttaccaaaaaggacaatgcatacccaacaataaagagaagttacaataataccaaccaagaagatcttttggtactatgggggattcaccatccaaatgatgcggcagagcagacaaggctctatcaaaacccaactacctatatttccgttgggacatcaacactaaaccagagattggtaccaaaaatagctgctagatctaaggtaaacgggcaaagtggaaggatggagttcttttggacaattttaaaatcgaatgatgcaataaactttgagagtaatggaaatttcattgctccagaaaatgcatacaaaattgtcaagaaaggggactcaacaattatgaaaagtgagttggaatatggtaactgcaacaccaagtgtcagactccaataggggcgataaactccagtatgccattccacaacatccaccctctcaccatcggggaatgccccaaatatgtgaaatcaaacagattagtccttgctactgggctcagaaatagccctcaaggagagagaagaagaaaaaagaga

>H5N1_A/Egypt/10211-NAMRU3/2007

taccatgcaaacaactcgacagaacaggttgacacaataatggaaaagaacgtcactgttacacacgcccaagacatactggaaaagacacacaacgggaagctctgcgatctagatggagtgaagcctctaattttaagagattgtagtgtagctggatggctcctcggaaacccaatgtgtgacgaattcctcaatgtgccggaatggtcttacatagtggagaagatcaatccagccaatgacctctgttacccaggggatttcaacgactatgaagaactgaaacacctgttgagcagaataaaccattttgagaaaattcagatcatccccaaaagttcttggtcagatcatgaagcatcgtcaggagtgagctcagcatgtccataccagggaagatcctccttttttagaaatgtggtatggcttatcaaaaaggacaatgcatacccaacaataaagagaagttacaataataccaaccaagaagatcttttggtactgtgggggattcaccatccgaatgatgcggcagagcagacaaggctctatcaaaacccaactacctatatttccgttgggacatcaacactaaaccagagattggtaccaaaaatagctactagatctaaggtaaatgggcaaagtggaaggatggagttcttttggacaattttaaaaccgaatgatgcaataaactttgagagtaatggaaatttcattgctccagaaaatgcatacaaaattgtcaagaaaggggactcaacaattatgaaaagtgagttggaatatggtaactgcaacaccaagtgtcaaactccaataggggcgataaactccagtatgccattccacaacatccaccctctcaccatcggggaatgccccaaatatgtgaaatcaagcagattagtccttgctactgggctcagaaatagccctcaaggagagagaagaagaaaaaagaga

>H5N1_A/Egypt/10215-NAMRU3/2007

taccatgcaaacaactcgacagagcaggttgacacaataatggaaaagaacgtcactgttacacacgcccaagacatactggaaaagacacacaatgggaaactctgcgatctagatggagttaagcctctaattttaagagattgtagtgtagctggatggctcctcgggaatccaatgtgtgacgaattcctcaatgtgccggaatggtcttacatagtggagaagatcaatccagccaatgacctctgttacccagggaatttcaacgactatgaagaactgaaacacctattgagcagaataaaccattttgagaaaattcagatcatccccaaaagttcttggtcaaatcatgaagcctcatcaggagtgagctcagcatgtccataccagggaagatcctccttttttagaaatgtggtatggcttatcaaaaaggacaatgcatacccaacaataaagataagttacaataataccaaccaagaagatcttttggtactgtgggggattcaccatccaaatgatgcggcagagcagacaaggctctatcaaaacccaactacctacatttccgttgggacatcaacattaaaccagagattggtaccaaaaatagctactagatccaaggtaaacgggcagagtggaaggatggagttcttttggacaattttaaaatcgaatgatgcaataaactttgaaagtaatggaaatttcattgctccagaaaatgcatacaaaattgtcaagaaaggggactcaacaattatgaaaagtgagttggaatatggtaactgcaacaccaagtgtcaaactccaataggggcgataaactccagtatgccgtttcacaacatccaccctctcaccatcggggaatgccccaaatatgtgaaatcaaacagattagtccttgctactgggctcagaaatagccctcaaggagagagaagaagaaaaaagaga

>H5N1_A/Egypt/10216-NAMRU3/2007

taccatgcaaacaactcgacagagcaggttgacacaataatggaaaagaacgtcactgttacacacgcccaagacatactggaaaagacacacaatgggaaactctgcgatctagatggagttaagcctctaattttaagagattgtagtgtagctggatggctcctcgggaacccaatgtgtgacgaattcctcaatgtgccggaatggtcttacatagtggagaagatcaatccagccaatgacctctgttacccagggaatttcaacgattatgaagaactgaaacacctattgagcagaataaaccattttgagaaaattcagatcatccccaaaagttcttggtcaaatcatgaagcctcatcaggggtgagctcagcatgtccataccagggaagatcctccttttttagaaatgtggtatggcttatcaaaaaggacaatgcatacccaacaataaagataagttacaataataccaaccaagaagatcttttggtactgtgggggattcaccatccaaatgatgcggcagagcagacaaggctctatcaaaacccaactacctatatttccgttgggacatcaacattaaaccagagattggtaccaaaaatagctactagatccaaggtaaacgggcaaagtggaaggatggagttcttttggacaattttaaaatcgaatgatgcaataaactttgaaagtaatggaaatttcattgctccagaaaatgcatacaaaattgtcaagaaaggggactcaacaattatgaaaagtgagttggaatatggtaactgcaacaccaagtgtcaaactccaataggggcgataaactccagtatgccatttcacaatatccaccctctcaccatcggggaatgccccaaatatgtgaaatcaaacagattagtccttgctactgggctcagaaatagccctcaaggagagagaagaagaaaaaagaga

>H5N1_A/Egypt/10217-NAMRU3/2007

taccatgcaaacaactcgacagagcaggttgacacaataatggaaaagaacgtcactgttacacacgcccaagacatactggaaaagacacacaacgggaaactctgcgatctagatggagttaagcctctaattttaagagattgtagtgtagctggatggctcctcgggaacccaatgtgtgacgaattcctcaatgttccggaatggtcttacatagtggagaagatcaatccagccaatgacctctgttacccagggaatttcaatgactatgaagaactgaagcacctattgagcagaataaaccattttgagaaaattcagatcatccccaaaagttcttggtcaaatcatgaagcctcatcaggagtgagctcagcatgtccataccagggaagatcctccttttttagaaatgtggtatggcttatcaaaaagggcaatgcatacccaacaataaagataagttacaataataccaaccaagaagatcttttggtactgtgggggattcaccatccaaatgatgcggcagagcagacaaggctctatcaaaacccaactacctatatttccgttgggacatcaacattaaaccagagattggtaccaaaaatagccactagatctaaggtaaacgggcaaaatggaaggatggagttcttttggacaattttaaaatcgaatgatgcaataaactttgagagtaatggaaatttcattgctccagaaaatgcatacaaaattgtcaagaaaggggattcaacaattatgaaaagtgagttggaatatggtaactgcaacaccaagtgtcaaactccaataggggcgataaactccagtatgccattccacaacatccaccctctcaccatcggggaatgccccaaatatgtgaaatcaaacagattagtccttgctactgggctcagaaatagccctcaaggagagagaagaagaaaaaagaga

>H5N1_A/Egypt/12374-NAMRU3/2006

taccatgcaaacaactcgacagagcaggttgacacaataatggaaaagaacgtcactgttacacacgcccaagacatactggaaaagacacacaacgggaaactctgcgatctagatggagtgaagcctctaattttaagagattgtagtgtagctgggtggctcctcgggaacccaatgtgtgacgaattcctcaatgtaccggaatggtcttacatagtggagaagatcaatccagccaatgacctctgttacccagggaatttcaacgactatgaagaactgaaacacctattgagcagaataaaccattttgagaaaattcagatcatccccaaaagttcttggtcagaccatgaagcctcatcaggagtgagctcagcatgtccataccagggaagatcctccttttttagaaatgtggtatggcttatcaaaaaggacaatgcatacccaacaataaagagaagttacaataataccaaccaagaagatcttttagtactgtgggggattcaccatcctaatgatgcggcagagcagacaaggctctatcaaaacccaactacctatatttccgttgggacatcaacactaaaccagagattggtaccaaaaatagctactagatctaaggtaaacgggcaaagtggaaggatagagttcttttggacaattttaaaatcgaatgatgcaataaactttgagagtaatggaaatttcattgctccagaaaatgcatacaaaattgtcaagaaaggggactcaacaattatgaaaagtgagttggaatatggtaactgcaacaccaagtgtcaaactccaataggggcgataaactccagtatgccattccacaacatccaccctctcaccatcggggaatgccccaaatatgtgaaatcaaacagattagtccttgctactgggctcagaaatagccctcaaggagagagaagaagaaaaaagaga

>H5N1_A/Egypt/1394-NAMRU3/2007

taccatgcaaacaactcgacagaacaggttgacacaataatggaaaagaacgtcactgttacgcacgcccaagacatactggaaaagacacacaacgggaaactctgcaatctagatggagtgaagcctctaattttaagagattgtagtgtagctggatggctcctcgggaacccaatgtgtgacgaattcctcaatgtgccggaatggtcttacatagtggagaagatcaatccagccaatgacctctgttatccagggaatttcaacgactatgaagaactgaaacacctattgagcagaataaaccattttgagaaaattcagatcatccccaaaaattcttggtcagatcatgaagccGGGtcaggagtgagctcagcatgcccataccagggaagatcctccttttttagaaatgtggtatggcttaccaaaagggacaatgcatacccaacaataaagagaagttacaataataccaaccaagaagatcttttggtactgtgggggattcaccatccaaatgatgcggcagagcagasaaggctctatcaaaacccaactacctatatttccgttgggacatcaacactaaaccagagattggtaccaaaaatagctactagatctaaggtaaacgggcaaartggaaggatggagttcttttggacaattttaaaatcgaatgatgcaataaactttgagagtaatggaaatttcattgctccagaaaatgcatacaaaattgtcaagaaaggggactcaacaattatgaaaagtgagttggaatatggtaactgcaacaccaagtgtcagactccaataggggcgataaactccagtatgccattccacaacatccaccctctcaccatcggggaatgccccaaatatgtgaaatcaaacagattagtccttgctactgggctcagaaatagccctcaaggagagagaagaagaaaaaagaga

>H5N1_A/Egypt/14724-NAMRU3/2006

taccatgcaaacaactcgacagagcaggttgacacaataatggaaaagaacgtcactgttacacacgcccaagacatactggaaaagacacacaacgggaaactctgcgatctagatggagtgaagcctctaattttaagagattgtagtgtagctggatggctcctcgggaacccaatgtgtgacgaattcctcaatgtgccggaatggtcttacatagtggagaagatcaatccagccaatgacctctgttacccagggaatttcaacgactatgaagaactgaaacacctattgagcagaataaaccattttgagaaaattcagatcatccccaaaagttcttggtcagatcatgaaacctcatcaggagtgagctcagcatgtccataccagggaagatcctccttttttagaaatgtggtatggcttatcaaaaaggacaatgcatacccaacaataaagagaagttacaataataccaaccaagaagatcttttggtactgtgggggattcaccatccaaatgatgcggcagagcagacaaggctctatcaaaacccaactacctatatttccgttgggacatcaacactaaaccagagattggtaccaaaaatagctactagatctaagataaacgggcaaagtggaaggatagagttcttttggacaattttaaaatcgaatgatgcaataaactttgagagtaatggaaatttcattgctccagaaaatgcatacaaaattgtcaagaaaggggactcaacaattatgaaaagtgaattggaatatggtaactgcaacaccaagtgtcaaactccaataggggcgataaactccagtatgccattccacaacatccaccctctcaccatcggggaatgccccaaatatgtgaaatcaaacagattagtccttgctactgggctcagaaatagccctcaaggagagagaagaagaaaaaagaga

>H5N1_A/Egypt/14725-NAMRU3/2006

taccatgcaaacaactcgacagagcaggttgacacaataatggaaaagaacgtcactgttacacacgcccaagacatactggaaaagacacacaacgggaaactctgcgatctagatggagtgaagcctctaattttaagagattgtagtgtagctggatggctcctcgggaacccaatgtgtgacgaattcctcaatgtgccggaatggtcttacatagtggagaagatcaatccagccaatgacctctgttacccagggaatttcaacgactatgaagaactgaaacacctattgagcagaataaaccattttgagaaaattcagatcatccccaaaagttcttggtcagatcatgaaacctcatcaggagtgagctcagcatgtccataccagggaagatcctccttttttagaaatgtggtatggcttatcaaaaaggacaatgcatacccaacaataaagagaagttacaataataccaaccaagaagatcttttggtactgtgggggattcaccatccaaatgatgcggcagagcagacaaggctctatcaaaacccaactacctatatttccgttgggacatcaacactaaaccagagattggtaccaaaaatagctactagatctaagataaacgggcaaagtggaaggatagagttcttttggacaattttaaaatcgaatgatgcaataaactttgagagtaatggaaatttcattgctccagaaaatgcatacaaaattgtcaagaaaggggactcaacaattatgaaaagtgaattggaatatggtaactgcaacaccaagtgtcaaactccaataggggcgataaactccagtatgccattccacaacatccaccctctcaccatcggggaatgccccaaatatgtgaaatcaaacagattagtccttgctactgggctcagaaatagccctcaaggagagagaagaagaaaaaagaga

>H5N1_A/Egypt/1604-NAMRU3/2007

taccatgcaaacaactcgacagagcaggttgacacaataatggaaaagaacgtcactgttacacacgcccaagacatactggaaaagacacacaacgggaaactctgcgatctagacggagtgaagcctctaattttaagagattgtagtgtagctggatggctcctcgggaacccaatgtgtgacgaattcctcaatgtgccagaatggtcttacatagtggagaagatcaatccagccaatgacctctgttacccagggaatttcaacgactatgaagaactgaaacacctattgagcagaataaaccattttgagaaaattcagatcatccccaaaagttcttggtcagatcatgaagcctcatcaggagtgagctcagcatgtccataccagggaagatcctccttttttagaaatgtggtatggcttatcaaaaaggacaatgcatacccaacaataaagagaagttacaataataccaaccaagaagatcttttggtactgtgggggattcaccatccaaatgatgcggcagagcagacaaggctctatcaaaacccaactacctatatttccgttgggacctcaacactaaaccagagattggtaccaaaaatagctactagatctaaggtaaacgggcaaagtggaaggattgagttcttttggacaattttaaaatcgaatgatgcaataaactttgagagtaatggaaatttcattgctccagaaaatgcatacaaaattgtcaagaaaggggactcaacaattatgaaaagtgagttggaatatggtaactgcaacaccaagtgtcaaactccaataggggcgataaactccagtatgccattccacaacatccaccctctcaccatcggggaatgccccaaatatgtgaaatcaaacagattagtccttgctactgggctcagaaatagccctcaaggagagagaagaagaaaaaagaga

>H5N1_A/Egypt/1731-NAMRU3/2007

taccatgcaaacaactcgacagagcaggttgacacaataatggaaaagaacgtcactgttacacacgcccaagacatactggaaaagacacacaacgggaaactctgcgatctagatggagtgaagcctctaattttaagagattgtagtgtagctggatggctcctcgggaacccaatgtgtgacgaattcctcaatgtgccggaatggtcttacatagtggagaagatcaatccagccaatgacctctgttacccagggaatttcaacaactatgaagaactgaaacacctattgagcagaataaaccattttgagaaaattcagatcatccccaaaagttcttggtcagatcatgaagcctcatcaggagtgagctcagcatgtccataccaggggagatcctccttttttagaaatgtggtatggcttatcaaaaaggacaatgcatacccaacaataaagagaagttacaataataccaaccaagaagatcttttgatactgtgggggattcaccatccaaatgatgcggcagagcagacaaggctctatcaaaacccaactacctatatttccgttgggacatcaacactaaaccagagattggtaccaaaaatagctactagatctaaggtaaacggacaaagtggaaggatggagttcttttggacaattttaaaatcgaatgatgcaataaactttgagagtaatggaaatttcattgctccagaaaatgcatacaaaattgtcaagaaaggggactcaacaattatgaaaagtgagttggaatatggtaactgcaacaccaagtgtcaaactccaataggggcgataaactccagtatgccattccacaacatccaccctctcaccatcggggaatgtcccaaatatgtgaaatcaaacagattagtccttgctactgggctcagaaatagccctcaaggagagagaagaagaaaaaagaga

>H5N1_A/Egypt/17692/2010

taccatgcaaacaactcgacagagcaggttgacacaataatggaaaagaacgtcactgttacacacgcccaagacatactggaaaagacacacaacgggaaactctgcaatctagatggagtgaagcctctaattttaagagattgtagcgtagccggatggctcctcgggaacccaatgtgcgacgaattcctcaatgtgccggaatggtcttacatagtggagaagatcaatccagccaatgacctctgttatccagggaatttcaacgactatgaagaactgaaacacttattaagcagaataaaccattttgagaaaattcagatcatccccaaaagttcttggtcagatcatgaagccGGGtcaggagtgagctcagcatgtccataccagggaagatcctccttttttagaaatgtggtatggcttaccaaaaaggacaatgcatacccaacaataaagagaagttacaataataccaaccaagaagatcttttggtactatgggggattcaccacccaaatgatgcagcagagcagacaaggctttatcaaaacccaactacctatatttccgttgggacatcaacactaaaccagagattggtaccaaaaatagctactagatctaaggtaaacgggcaaagtggaaggatggagttcttttggacaattttaaaatcgaatgatgcaataaactttgagagtaatggaaatttcattgctccagaaaatgcatacaaaattgtcaagaaaggggactcaacaattatgaaaagtgagttggaatatggtaactgcaacaccaagtgtcagactccaataggggcgataaactccagtatgccattccacaacatccaccctctcaccatcggggaatgccccaaatatgtgaaatcaaacagattagtccttgctactgggctaagaaatagccctcaaggagagaggagaagaaaaaagaga

>H5N1_A/Egypt/1902-NAMRU3/2007

taccatgcaaacaactcgacagagcaggttgacacaataatggaaaagaacgtcactgttacacacgcccaagacatactggaaaagacacacaacgggaaactctgcgatctagatggagtgaagcctctaattttaagagattgtagtgtagctggatggctcctcgggaacccaatgtgtgacgaattcctcaatgtgccggaatggtcttacatagtggagaagatcaatccagccaatgacctctgttacccagggactttcaacgactacgaagaactgaaacacctattgagcagaataaaccattttgagaaaattcagatcatccccaaaagttcttggtcagatcatgaagcctcatcaggagtgagctcagcatgtccacaccagggaagatcctccttttttagaaatgtggtatggcttatcaaaaagaacaatgcatacccaacaataaagataagttacaataataccaaccaagaagatcttttggtactgtgggggattcaccatccaaatgatgcggcagagcagacaaggctctatcaaaacccaactacctatatttccgttgggacatcaacactaaaccagagattggtaccaaaaatagctactagatctaaagtaaacgggcaaagtggaaggatggagttcttttggacaattttaaaatcgaatgatgcaataaactttgagagtaatggaaatttcattgctccagaaaatgcatacaaaattgtcaagaaaggggactcaacaattatgaaaagtgagttggaatatggtaactgcaacaccaagtgtcaaactccaataggggcgataaactccagtatgccattccataacatccaccctctcaccatcggggaatgccccaaatatgtgaaatcaaacagattagtccttgctactgggctcagaaatagccctcaaggagagagaagaagaaaaaagaga

>H5N1_A/Egypt/1980-NAMRU3/2008

taccatgcaaacaactcgacagaacaggttgacacaataatggaaaagaacgtcactgttacacacgcccaagacatactggaaaagacacacaacgggaagctctgcgatctagatggagtgaagcctctaattttaagagattgtagtgtagctggatggctcctcgggaacccaatgtgtgacgaattcctcaatgtgccggaatggtcttacatagtggagaagatcaatccagccaatgacctctgttacccaggggatttcaacgactatgaagaactgaaacacctgttgagcagaataaaccattttgagaaaattcagatcatccccaaaagttcttggtcagatcatgaagcatcgtcaggagtgagctcagcatgtccataccagggaagatcctccttttttagaaatgtggtatggcttatcaaaaaggaaaatgcatacccaacaataaagagaagttacaataataccaaccaagaagatcttttggtactgtgggggattcaccatccgaatgatgcggcagagcagacaaggctctatcaaaacccaactacctatatttccgttgggacatcaacactaaaccagagattggtaccaaaaatagctactagatctaaggtaaatgggcaaagtggaaggatggagttcttttggacaattttaaaaccgaatgatgcaataaactttgagagtaatggaaatttcattgctccagaaaatgcatacaaaattgtcaagaaaggggactcaacaattatgaaaagtgagttggaatatggtaactgcaacaccaagtgtcaaactccaataggggcgataaactccagtatgccattccacaatatccaccctctcaccatcggggaatgccccaaatatgtgaaatcaaacagattagtccttgctactgggctcagaaatagccctcaaggagagagaagaagaaaaaagaga

>H5N1_A/Egypt/2256-NAMRU3/2007

taccatgcaaacaactcgacagagcaggttgacacaataatggaaaagaacgtcactgttacacacgcccaagacatactggaaaagacacacaacgggaaactctgcgatctagatggagtgaagcctctaattttaagagattgtagtgtagctggatggctcctcgggaacccaatgtgtgacgaattcctcaatgtgccggaatggtcttacatagtggagaagatcaatccagccaatgacctctgttacccagggaatttcaacgactatgaagaactgaaacacctattgagcagaataaaccattttgagaaaattcagatcatccccaaaagttcttggtcagatcatgaagcctcatcaggagtgagctcagcatgtccataccagggaagatcctccttttttagaaatgtggtatggcttatcaaaaaggacaatgcatacccaacaataaagagaagttacaataataccaaccaagaagatcttttggtactgtgggggattcaccatccaaatgatgcggcagagcagacaaggctctatcaaaacccaactacctatatttccgttgggacatcaacactaaaccagagattggtaccaaaaatagctactagatctaaggtaaacgggcaaagtggaaggatggagttcttttggacaattttaaaatcgaatgatgcaataaactttgagagtaatggaaatttcattgctccagaaaatgcatacaaaattgtcaagaaaggggactcaacaattatgaaaagtgagttggaatatggtaactgcaacaccaagtgtcaaactccaataggggcgataaactccagtatgccattccacaacattcaccctctcaccatcggggaatgccccaaatatgtgaaatcaaacagattagtccttgctactgggctcagaaatagccctcaaggagagagaagaagaaaaaagaga

>H5N1_A/Egypt/2289-NAMRU3/2008

taccatgcaaacaactcgacagagcaggttgacacaataatggaaaagaacgtcactgttacacacgcccaagacatactggaaaagacacacaacgggaaactctgcaatctagatggagtgaagcctctaattttaagagattgtagtgtagctggatggctcctcgggaatccaatgtgtgacgaattcctcaatgtgccggaatggtcttacatagtggagaagatcaatccagccaatgacctctgttatccaggaaatttcaacgactatgaagaactgaaacacctattgagcagaataaaccattttgagaaaattcagatcatccccaaaaattcttggtcagatcatgaagccGGGtcaggagtgagctcagcatgtccataccagggaagatcctccttttttagaaatgtggtatggcttaccaaaaaggacaatgcatacccaacaataaagagaagttacaataataccaaccaagaagatcttttggtactgtgggggattcaccatccaaatgatgcggcagagcagacaaggctctatcaaaacccaactacctatatttccgttgggacatcaacactaaaccagagattggtaccaaaaatagctactagatctaaggtaaacgggcaaattggaaggatggagttcttttggacaattttaaaatcgaatgatgcaataaactttgagagtaatggaaatttcattgctccagaaaatgcatacaaaattgtcaagaaaggggactcaacaattatgaaaagtgagttggaatatggtaactgcaacaccaagtgtcagactccaataggggcgataaactccagtatgccattccacaacatccaccctctcaccatcggggaatgccccaaatatgtgaaatcaaacagattagtccttgctactgggctcagaaatagccctcagggagagagaagaagaaaaaagaga

>H5N1_A/Egypt/2321-NAMRU3/2007

taccatgcaaacaactcgacagaacaggttgacacaataatggaaaagaacgtcactgttacacacgcccaagacatactggaaaagacacacaacgggaaactctgcgatctagatggagtgaagcctctaattttaagagattgtagtgtagctggatggctcctcgggaacccaatgtgtgacgaattcctcaatgtgccggaatggtcttacatagtggagaagatcaatccagccaatgacctctgttacccaggggatttcaacgactatgaagaactgaaacacctattgagcagaataaaccattttgagaaaattcagatcatccccaaaagttcttggtcagattatgaagcctcatcaggagtgagctcagcatgtccataccagggaagatcctccttttttagaaatgtggtatggcttatcaaaaagaacaatgcatacccaacaataaagagaagttacaataataccaaccaagaggatcttttggtactgtgggggattcaccatccgaatgatgcggcagagcagataaggctctatcaaaacccaactacctatatttccgttgggacatcaacactaaaccagagattggtaccaaaaatagctactagatctaaggtaaatgggcaaagtggaaggatggagttcttttggacaattttaaaatcgaatgatgcaataaactttgagagtaatggaaatttcattgctccagaatatgcatacaaaattgtcaagaaaggggactcaacaattatgaaaagtgagttggaatatggtaactgcaacaccaaatgtcaaactccaataggggcgataaactccagtatgccattccacaacatccaccctctcaccatcggggaatgccccaaatatgtgaaatcaaacagattagtccttgctactgggctcagaaatagccctcaaggagagagaagaagaagaaagaga

>H5N1_A/Egypt/2331-NAMRU3/2007

taccatgcaaacaactcgacagaacaggttgacacaataatggaaaagaacgtcactgttacacacgcccaagacatactggaaaagacacacaacgggaaactctgcgatctagatggagtgaagcctctaattttaagagattgtagtgtagctggatggctcctcgggaacccaatgtgtgacgaattcctcaatgtgccggaatggtcttacatagtggagaagatcaatccagccaatgacctctgttacccaggggatttcaacgactatgaagaactgaaacacctattgagcagaataaaccattttgagaaaattcagatcatccccaaaagttcttggtcagattatgaagcctcatcaggagtgagctcagcatgtccataccagggaagatcctccttttttagaaatgtggtatggcttatcaaaaagaacaatgcatacccaacaataaagagaagttacaataataccaaccaagaggatcttttggtactgtgggggattcaccatccgaatgatgcggcagagcagataaggctctatcaaaacccaactacctatatttccgttgggacatcaacactaaaccagagattggtaccaaaaatagctactagatctaaggtaaatgggcaaagtggaaggatggagttcttttggacaattttaaaatcgaatgatgcaataaactttgagagtaatggaaatttcattgctccagaatatgcatacaaaattgtcaagaaaggggactcaacaattatgaaaagtgagttggaatatggtaactgcaacaccaaatgtcaaactccaataggggcgataaactccagtatgccattccacaacatccaccctctcaccatcggggaatgccccaaatatgtgaaatcaaacagattagtccttgctactgggctcagaaatagccctcaaggagagagaagaagaagaaagaga

>H5N1_A/Egypt/2472-NAMRU3/2008

taccatgcaaacaactcgacagaacaggtagacacaataatggaaaagaacgtcactgttacacacgcccaagacatactggaaaagacacacaacgggaagctctgcgatctagatggggtgaagcctctaattttaagagattgtagtgtagctggatggctcctcgggaacccaatgtgtgacgaattcctcaatgtgccggaatggtcttacatagtggagaagatcaatccagccaatgacctctgttacccaggggatttcaacgactatgaagaactgaaacacctgttgagcagaataaaccattttgagaaaattcagatcattcccaaaagttcttggtcagatcatgaagcatcgtcaggagtgagctcagcatgtccataccagggaagatcctccttttttagaaatgtggtatggcttatcaaaaaggacaatgcatacccaacaataaagagaagttacaataataccaaccaagaagatcttttggtactgtgggggattcaccatccgaatgatgcggcagagcagacaaggctctatcaaaacccaactacctatatttccgttgggacatcaacactaaaccagagattggtaccaaaaatagctactagatctaaggtaaatgggcaaagtggaaggatggagttcttttggacaattttaaaaccgaatgatgcaataaactttgagagtaatggaaatttcattgctccagaaaatgcatacaaaattgtcaagaaaggggactcaacaattatgaaaagtgagttggaatatggtaactgcaacaccaagtgtcaaactccaataggggcgataaactccagtatgccattccacaacatccaccctctcaccatcggggaatgccccaaatatgtgaaatcaaacagattagtccttgctactgggctcagaaatagccctcaaggggagagaagaagaaaaaagaga

>H5N1_A/Egypt/2514-NAMRU3/2008

taccatgcaaacaactcgacagaacaggttgacacaataatggaaaagaacgtcactgttacacacgcccaagacatactggaaaagacacacaacgggaagctctgcgatctagatggagtgaagcctctaattttaagagattgtagtgtagctggatggctcctcgggaacccaatgtgtgacgaattcctcaatgtgccggaatggtcttacatagtggagaagatcaatccagccaatgacctctgttacccaggggacttcaacgactatgaagaactgaaacacctgttgagcagaataaaccattttgagaaaatccagatcatccccaaaagttcttggtcagatcatgaagcatcgtcaggagtgagctcagcatgtccataccagggaagatcctccttttttagaaatgtggtatggcttatcaaaaaggacaatgcatacccaacaataaagagaagttacaataataccaaccaagaagatcttttggtactgtgggggattcaccatccgaaagatgcggcagagcagacaaggctctatcaaaacccaattacctatatttccgttgggacatcaacactaaaccagagattggtaccaaaaatagctactagatctaaggtaaatgggcaaagtggaaggatggagttcttttggacaattttaaaaccgaatgatgcaataaactttgagagtaatggaaatttcattgctccagaaaatgcatacaaaattgtcaagaaaggggactcaacaattatgaaaagtgagttggaatatggtaactgcaacaccaagtgtcaaactccaataggggcgataaactccagtatgccattccacaacatccaccctctcaccatcggggaatgccccaaatatgtgaaatcaaacagattagtccttgctactgggctcagaaatagccctcaaggagagagaagaagaaaaaagaga

>H5N1_A/Egypt/2546-NAMRU3/2008

taccatgcaaacaactcgacagagcaggttgacacaataatggaaaagaacgtcactgttacacacgcccaagacatactggaaaagacacacaacgggaaactctgcaatctagatggagtgaagcctctaattttaagagattgtagtgtagctggatggctcctcgggaatccaatgtgtgacgaattcctcaatgtgccggaatggtcgtacatagtggagaagatcaatccagccaatgacctctgttatccagggaatttcaacgactatgaagaactgaaacacctattgagcagaataaaccattttgagaaaattcagatcatccccaaaaattcttggtcagatcatgaagccGGGtcaggagtgagctcagcatgtccataccagggaagatcctccttttttagaaatgtggtatggcttaccaaaaaggacaatgcctacccaacaataaagagaagttacaataataccaaccaagaagatcttttggtactgtgggggattcaccatccaaatgatgcggcagagcagacaaggctctatcaaaacccaactacctatatttccgttgggacatcaacactaaaccagagattggtaccaaaaatagctactagatctaaggtaaacgggcaaaatggaaggatggagttcttttggacaattttaaaatcgaatgatgcaataaactttgagagtaatggaaatttcattgctccagaaaatgcatacaaaattgtcaagaaaggggactcaacaattatgaaaagtgagttggaatatggtaactgcaacaccaagtgtcagactccaataggggcgataaactccagtatgccattccacaacatccaccctctcaccatcggggaatgccccaaatatgtgaaatcaaacagattagtccttgctactgggctcagaaatagccctcagggagagagaagaagaaaaaagaga

>H5N1_A/Egypt/2616-NAMRU3/2007

taccatgcaaacaactcgacagaacaggttgacacaataatggaaaagaacgtcactgttacacacgcccaagacatactggaaaagacacacaacgggaaactctgcgatctagatggagtgaagcctctaattttaagagattgtagtgtagctggatggctcctcgggaacccaatgtgtgacgaattcctcaatgtgccggaatggtcttacatagtggagaagatcaatccagccaatgacctctgttacccaggggatttcaacgactatgaagaactgaaacacctattgagcagaataaaccattttgagaaaattcagatcatccccaaaagttcttggtcagattatgaagcctcatcaggagtgagctcagcatgtccataccagggaagatcctccttttttagaaatgtggtatggcttatcaaaaagaacaatgcatacccaacaataaagagaagttacaataataccaaccaagaggatcttttggtactgtgggggattcaccatccgaatgatgcggcagagcagataaggctctatcaaaacccaactacctatatttccattgggacatcaacactaaaccagagattggtaccaaaaatagctactagatctaaggtaaatgggcaaagtggaaggatggagttcttttggacaattttaaaatcgaatgatgcaataaactttgagagtaatggaaatttcattgctccagaatatgcatacaaaattgtcaagaaaggggactcaacaattatgaaaagtgagttggaatatggtaactgcaacaccaaatgtcaaactccaataggggcgataaactccagtatgccattccacaacatccaccctctcaccatcggggaatgccccaaatatgtgaaatcaaacagattagtccttgctactgggctcagaaatagccctcaaggagagagaagaagaagaaagaga

>H5N1_A/Egypt/2620-NAMRU3/2007

taccatgcaaacaactcgacagaacaggttgacacaataatggaaaagaacgtcactgttacacacgcccaagacatactggaaaagacacacaacgggaaactctgcgatctagatggagtgaagcctctaattttaagagattgtagtgtagctggatggctcctcgggaacccaatgtgtgacgaattcctcaatgtgccggaatggtcttacatagtggagaagatcaatccagccaatgacctctgttacccaggggatttcaacgactatgaagaacttaaacacctattgagcagaataaaccattttgagaaaattcagatcatccccaaaagttcttggtcagattatgaagcctcatcaggagtgagctcagcatgtccataccagggaagatcctccttttttagaaatgtggtatggcttatcaaaaagaacaatgcatacccaacaataaagagaagttacaataataccaaccaagaggatcttttggtactgtgggggattcaccatccgaatgatgcggcagagcagacaaggctctatcaaaacccaactacctatatttccgttgggacatcaacactaaaccagagattggtaccaaaaatagctactagatctaaggtaaatgggcaaagtggaaggatggagttcttttggacaattttaaaatcgaatgatgcaataaactttgagagtaatggaaatttcattgctccagaatatgcatacaaaattgtcaagaaaggggactcaacaattatgaaaagtgagttggaatatggtaactgcaacaccaaatgtcaaactccaataggggcgataaactccagtatgccattccacaacatccaccctctcaccatcggggaatgccccaaatatgtgaaatcaaacagattagtccttgctactgggctcagaaatagccctcaaggagagagaagaagaagaaagaga

>H5N1_A/Egypt/2621-NAMRU3/2007

taccatgcaaacaactcgacagagcaggttgacacaataatggaaaagaacgtcactgttacacacgcccaagacatactggaaaagacacacaacgggaaactctgcaatctagatggagtgaagcctctaattttaagagattgtagtgtagctggatggctcctcgggaacccaatgtgtgacgaattcctcaatgtgccggaatggtcttacatagtggagaagatcaatccagccaatgacctctgttatccagggaatttcaacgactatgaagaactgaaacacctattgagcagaataaaccattttgagaaaattcagatcatccccaaaaattcttggtcagatcatgaagccGGGtcaggagtgagctcagcatgtccataccagggaagatcctccttttttagaaatgtggtatggcttaccaaaaaggacaatgcatacccaacaataaagagaagttacaataataccaaccaagaagatcttttggtactatgggggattcaccatccaaatgatgcggcagagcagacaaggctttatcaaaacccaactacctatatttccgttgggacatcaacactaaaccagagattggtaccaaaaatagctactagatctaaggtaaacgggcaaagtggaaggatggagttcttttggacaattttaaaatcgaatgatgcaataaactttgagagtaatggaaatttcattgctccagaaaatgcatacaaaattgtcaagaaaggggactcaacaattatgaaaagtgagttggaatatggtaactgcaacaccaagtgtcagactccaataggggcgataaactccagtatgccattccacaacatccaccctctcaccatcggggaatgccccaaatatgtgaaatcaaacagattagtccttgctactgggctcagaaatagccctcaaggagagagaagaagaaaaaagaga

>H5N1_A/Egypt/2629-NAMRU3/2007

taccatgcaaacaactcgacagagcaggttgacacaataatggaaaagaacgtcactgttacacacgcccaagacatactggaaaagacacacaacgggaaactctgcaatctagatggagtgaagcctctaattttaagagattgtagtgtagctggatggctcctcgggaacccaatgtgtgacgaattcctcaatgtgccggaatggtcttacatagtggagaagatcaatccagccaatgacctctgttatccagggaatttcaacgactatgaagaactgaaacacctattgagcagaataaaccattttgagaaaattcagatcatccccaaaaattcttggtcagatcatgaagccGGGtcaggagtgagctcagcatgtccataccagggaagatcctccttttttagaaatgtggtatggcttaccaaaaaggacaatgcatacccaacaataaagagaagttacaataataccaaccaagaagatcttttggtactatgggggattcaccatccaaatgatgcggcagagcagacaaggctttatcaaaacccaactacctatatttccgttgggacatcaacactaaaccagagattggtaccaaaaatagctactagatctaaggtaaacgggcaaagtggaaggatggagttcttttggacaattttaaaatcgaatgatgcaataaactttgagagtaatggaaatttcattgctccagaaaatgcatacaaaattgtcaagaaaggggactcaacaattatgaaaagtgagttggaatatggtaactgcaacaccaagtgtcagactccaataggggcgataaactccagtatgccattccacaacatccaccctctcaccatcggggaatgccccaaatatgtgaaatcaaacagattagtccttgctactgggctcagaaatagccctcaaggagagagaagaagaaaaaagaga

>H5N1_A/Egypt/2630-NAMRU3/2007

taccatgcaaacaactcgacagagcaggttgacacaataatggaaaagaacgtcactgttacacacgcccaagacatactggaaaagacacacaacgggaaactctgcgatctagatggagtgaagcctctaattttaagagattgtagtgtagctggatggctcctcgggaacccaatgtgtgacgaattcctcaatgtgccggaatggtcttacatagtggagaagatcaatccagccaatgacctctgttacccagggaatttcaacgactatgaagaactgaaacacctattgagcagaataaaccattttgagaaaattcagatcatccccaaaagttcttggtcagatcatgaagcctcatcaggagtgagctcagcatgtccataccagggaagatcctccttttttagaaatgtagtatggcttataaaaaaggacaatgcatacccaacaataaagagaagttacaataataccaaccaagaagatcttttggtactgtggggaatccaccatccaaatgatgcggcagagcagacaaggctctatcaaaacccaactacctatatttccgttgggacatcaacactaaaccagagattggtgccaaagatagctactagatctaaggtaaacgggcaaagtggaaggatggagttcttttggacaattttaaaatcgaatgatgcaataaactttgagagtaatggaaatttcattgctccagaaaatgcatacaaaattgtcaagaaaggggactcaacaattatgaaaagtgagttggaatatggtaactgcaacaccaagtgtcaaactccaataggggcgataaactccagtatgccattccacaacatccaccctctcaccatcggggaatgccccaaatatgtgaaatcaaacagattagtccttgctactgggctcagaaatagccctcaaggagagagaagaagaaaaaagaga

>H5N1_A/Egypt/2631-NAMRU3/2007

taccatgcaaacaactcgacagagcaggttgacacaataatggaaaagaacgtcactgttacacacgcccaagacatactggaaaagacacacaacgggaaactctgcaatctagatggagtgaagcctctaattttaagagattgtagtgtagctggatggctcctcgggaacccaatgtgtgacgaattccttaatgtgccggaatggtcttacatagtggagaagatcaatccagccaatgacctctgttatccagggaatttcaacgactatgaagaactgaaacacctattgagcagaataaaccattttgagaaaattcagatcatccccaaaaattcttggtcagatcatgaagccGGGtcaggagtgagctcagcatgtccataccagggaagatcctccttttttagaaatgtagtatggcttaccaaaaaggacaatgcatacccaacaataaagagaagttacaataataccaaccaagaagatcttttggtactgtgggggattcaccatccaaatgatgcggcagagcagacaaggctctatcaaaacccaactacctatatttccgttgggacatcaacactaaaccagagattggtaccaaaaatagctactagatctaaggtaaacgggcaaagtggaaggatggagttcttttggacaattttaaaatcgaatgatgcaataaactttgagagtaatggaaatttcattgctccagaaaatgcatacaaaattgtcaagaaaggggactcaacaattatgaaaagtgagttggaatatggtaactgcaacaccaagtgtcagactccaataggggcgataaactccagtatgccattccacaacatccaccctctcaccatcggggaatgccccaaatatgtgaaatcaaacagattagtccttgctactgggctcagaaatagccctcaaggagagagaagaagaaaaaagaga

>H5N1_A/Egypt/2750-NAMRU3/2007

taccatgcaaacaactcgacagaacaggttgacacaataatggaaaagaacgtcactgttacacacgcccaagacatactggaaaagacacacaacgggaaactctgcgatctagatggagtgaagcctctaattttaagagattgtagtgtagctggatggctcctcgggaacccaatgtgtgacgaattcctcaatgtgccggaatggtcttacatagtggagaagatcaatccagccaatgacctctgttacccaggggatttcaacgactatgaagaactgaaacacctattgagcagaataaaccattttgagaaaattcagatcatccccaaaagttcttggtcagatcatgaagcctcatcaggagtgagctcagcatgtccataccagggaagatcctccttttttagaaatgtggtatggcttatcaaaaagaacaatgcatacccaacaataaagagaagttacaataataccaaccaagaggatcttttggtactgtgggggattcaccatccgaatgatgcggcagagcagacaaggctctatcaaaacccaactacctatatttccgttgggacatcaacactaaaccagagattggtaccaaaaatagctactagatctaaggtaaatgggcaaagtggaaggatggagttcttttggacaattttaaaatcgaatgatgcaataaactttgagagtaatggaaatttcattgctccagaatatgcatacaaaattgtcaagaaaggggactcaacaattatgaaaagtgagttggaatatggtaactgcaacaccaaatgtcaaactccaataggggcgataaactccagtatgccattccacaacatccaccctctcaccatcggggaatgccccaaatatgtgaaatcaaacaggttagtccttgctactgggctcagaaatagccctcaaggagagagaagaagaaaaaagaga

>H5N1_A/Egypt/2751-NAMRU3/2007

taccatgcaaacaactcgacagagcaggttgacacaataatggaaaagaacgtcactgttacacacgcccaagacatactggaaaagacacacaacgggaaactctgcgatctagatggagttaagcctctaattttaagagattgtagtgtagctggatggctcctcgggaacccaatgtgtgacgaattcctcaatgtgccggaatggtcttacatagtggagaagatcaatccagccaacgacctctgttacccagggaatttcaacgactatgaagaactgaaacacctattaagcagaataaaccattttgaaaaaattcagatcatccccaaaagttcttggtcaaatcatgaagcctcatcaggagtgagctcagcatgtccataccagggaagatcctccttttttagaaatgtggtatggcttatcaaaaaggacaatgcatacccaacaataaagataagttacaataataccaaccaagaagatcttttggtactgtgggggattcaccatccaaawgatgaggcagagcagacaaggctctatcaaaacccaactacctatatttccgttgggacatcaacattaaaccagagattggtaccaaaaatagctactagatctaaggtaaacgggcaaagtggaaggatggagttcttttggacaattttaaaatcgaatgatgcaataaactttgagagtaatggaaatttcattgctccagaaaatgcatacaaaattgtcaagaaaggggactcaacaattatgaaaagtgagttggaatatggtaactgcaacaccaagtgtcaaactccaataggggcgataaactccagtatgccattccacaacatccaccctctcaccatcggggaatgccccaaatatgtgaaatcaaacagattagtccttgctactgggctcagaaatagccctcaaggagagagaagaagaaaaaagaga

>H5N1_A/Egypt/2782-NAMRU3/2006

taccatgcaaacaactcgacagagcaggttgacacaataatggaaaagaacgtcactgttacacacgcccaagacatactggaaaagacacacaacgggaaactctgcgatctagatggagtgaagcctctaattttaagagattgtagtgtagctggatggctcctcgggaacccaatgtgtgacgaattcctcaatgtgccggaatggtcttacatagtggagaagatcaatccagccaatgacctctgttacccagggaatttcaacgactatgaagaactgaaacacctattgagcagaataaaccattttgagaaaattcagatcatccccaaaagttcttggtcagatcatgaagcctcatcaggagtgagctcagcatgtccataccagggaagatcctccttttttagaaatgtggtatggcttatcaaaaaggacaatgcatacccaacaataaagagaagttacaataataccaaccaagaagatcttttggtactgtgggggattcaccatccgaatgatgcggcagagcagacaaggctctatcaaaacccaactacctatatttccgttgggacatcaacactaaaccagagattggtaccaaaaatagctactagatctaaggtaaacgggcaaagtggaaggatggagttcttttggacaattttaaaatcgaatgatgcaataaactttgagagtaatggaaatttcattgctccagaaaatgcatacaaaattgtcaagaaaggggactcaacaattatgaaaagtgagttggaatatggtaactgcaacaccaagtgtcaaactccaataggggcgataaactccagtatgccattccacaacatccaccctctcaccatcggggaatgccccaaatatgtgaaatcaaacagattaatccttgctactgggctcagaaatagccctcaaggagagagaagaagaaaaaagaga

>H5N1_A/Egypt/2991-NAMRU3/2006

taccatgcaaacaactcgacagagcaggttgacacaataatggaaaagaacgtcactgttacacacgcccaagacatactggaaaagacacacaacgggaaactctgcgatctagatggagtgaagcctctaattttaagagattgtagtgtagctggatggctcctcgggaacccaatgtgtgacgaattcctcaatgtgccggaatggtcttacatagtggagaagatcaatccagccaatgacctctgttacccagggaatttcaacgactatgaagaactgaaacacctattgagcagaataaaccattttgagaaaattcagatcatccccaaaagttcttggtcagatcatgaagcctcatcaggagtgagctcagcatgtccataccagggaagatcctccttttttagaaatgtggtatggcttatcaaaaaggacaatgcatacccaacaataaagagaagttacaataataccaaccaagaagatcttttggtactgtgggggattcaccatccaaatgatgcggcagagcagacaaggctctatcaaaacccaactacctatatttccgttgggacatcaacactaaaccagagattggtaccaaaaatagctactagatctaaggtaaacgggcaaagtggaaggatggagttcttttggacaattttaaaatcgaatgatgcaataaactttgagagtaatggaaatttcattgctccagaaaatgcatacaaaattgtcaagaaaggggactcaacaattatgaaaagtgagttggaatatggtaactgcaacaccaagtgtcaaactccaataggggcgataaactccagtatgccattccacaacatccaccctctcaccatcggggaatgccccaaatatgtgaaatcaaacagattagtccttgctactgggctcagaaatagccctcaaggagagagaagaagaaaaaagaga

>H5N1_A/Egypt/2992-NAMRU3/2006

taccatgcaaacaactcgacagagcaggttgacacaataatggaaaagaacgtcactgttacacacgcccaagacatactggaaaagacacacaacgggaaactctgcgatctagatggagtgaagcctctaattttaagagattgtagtgtagctggatggctcctcgggaacccaatgtgtgacgaattcctcaatgtgccggaatggtcttacatagtggagaagatcaatccagccaatgacctctgttacccagggaatttcaacgactatgaagaactgaaacacctattgagcagaataaaccattttgagaaaattcagatcatccccaaaagttcttggtcagatcatgaagcctcatcaggagtgagctcagcatgtccataccagggaagatcctccttttttagaaatgtggtatggcttatcaaaaaggacaatgcatacccaacaataaagagaagttacaataataccaaccaagaagatcttttggtactgtgggggattcaccatccaaatgatgcggcagagcagacaaggctctatcaaaacccaactacctatatttccgttgggacatcaacactaaaccagagattggtaccaaaaatagctactagatctaaggtaaacgggcaaagtggaaggatggagttcttttggacaattttaaaatcgaatgatgcaataaactttgagagtaatggaaatttcattgctccagaaaatgcatacaaaattgtcaagaaaggggactcaacaattatgaaaagtgagttggaatatggtaactgcaacaccaagtgtcaaactccaataggggcgataaactccagtatgccattccacaacatccaccctctcaccatcggggaatgccccaaatatgtgaaatcaaacagattagtccttgctactgggctcagaaatagccctcaaggagagagaagaagaaaaaagaga

>H5N1_A/Egypt/3158-NAMRU3/2008

taccatgcaaacaactcgacagaacaggtagacacaataatggaaaagaacgtcactgttacacacgcccaagacatactggaaaagacacacaacgggaagctctgcgatctagatggggtgaagcctctaattttaagagattgtagtgtagctggatggctcctcgggaacccaatgtgtgacgaattcctcaatgtgccggaatggtcttacatagtggagaagatcaatccagccaatgacctctgttacccaggggatttcaacgactatgaagaactgaaacacctgttgagcagaataaaccattttgagaaaattcagatcattcccaaaagttcttggtcagatcatgaagcatcgtcaggagtgagctcagcatgtccataccagggaagatcctccttttttagaaatgtggtatggcttatcaaaaaggacaatgcatacccaacaataaagagaagttacaataataccaaccaagaagatcttttggtactgtgggggattcaccatccgaatgatgcggcagagcagacaaggctctatcaaaacccaactacctatatttccgttgggacatcaacactaaaccagagattggtaccaaaaatagctactagatctaaggtaaatgggcaaagtggaaggatggagttcttttggacaattttaaaaccgaatgatgcaataaactttgagagtaatggaaatttcattgctccagaaaatgcatacaaaattgtcaagaaaggggactcaacaattatgaaaagtgagttggaatatggtaactgcaacaccaagtgtcaaactccaataggggcgataaactccagtatgccattccacaacatccaccctctcaccatcggggaatgccccaaatatgtgaaatcaaacagattagtccttgctactgggctcagaaatagccctcaaggggagagaagaagaaaaaagaga

>H5N1_A/Egypt/321-NAMRU3/2007

taccatgcaaacaactcgacagagcaggttgacacaataatggaaaagaacgtcactgttacacacgcccaagacatactggaaaagacacacaacgggaaactctgcgatctagatggagtgaagcctctaattttaagagattgtagtgtagctggatggctcctcgggaacccaatgtgtgacgaattcctcaatgtgccggaatggtcttacatagtggagaagatcaatccagccaatgacctctgttacccaggggatttcaacgactatgaagaactgaaacacctattgagcagaataaaccattttgagaaaattcagatcatccccaaaagttcttggtcagattatgaagcctcatcaggagtgagctcagcatgtccataccagggaagatcctccttttttagaaatgtggtatggcttatcaaaaagaacaatgcatacccaacaataaagagaagttacaataataccaaccaagaggatcttttggtactgtgggggattcaccatccgaatgatgcggcagagcagataaggctctatcaaaacccaactacctatatttccgttgggacatcaacactaaaccagagattggtaccaaaaatagctactagatctaaggtaaatgggcaaagtggaaggatggagttcttttggacaattttaaaatcgaatgatgcaataaactttgagagtaatggaaatttcattgctccagaatatgcatacaaaattgtcaagaaaggggactcaacaattatgaaaagtgagttggaatatggtaactgcaacaccaaatgtcaaactccaataggggcgataaactccagtatgccattccacaacatccaccctctcaccatcggggaatgccccaaatatgtgaaatcaaacagattagtccttgctactgggctcagaaatagccctcaaggagagagaagaagaagaaagaga

>H5N1_A/Egypt/321/2007

taccatgcaaacaactcgacagagcaggttgacacaataatggaaaagaacgtcactgttacacacgcccaagacatactggaaaagacacacaacgggaaactctgcgatctagatggagtgaagcctctaattttaagagattgtagtgtagctggatggctcctcgggaacccaatgtgtgacgaattcctcaatgtgccggaatggtcttacatagtggagaagatcaatccagccaatgacctctgttacccaggggatttcaacgactatgaagaactgaaacacctattgagcagaataaaccattttgagaaaattcagatcatccccaaaagttcttggtcagattatgaagcctcatcaggagtgagctcagcatgtccataccagggaagatcctccttttttagaaatgtggtatggcttatcaaaaagaacaatgcatacccaacaataaagagaagttacaataataccaaccaagaggatcttttggtactgtgggggattcaccatccgaatgatgcggcagagcagataaggctctatcaaaacccaactacctatatttccgttgggacatcaacactaaaccagagattggtaccaaaaatagctactagatctaaggtaaatgggcaaagtggaaggatggagttcttttggacaattttaaaatcgaatgatgcaataaactttgagagtaatggaaatttcattgctccagaatatgcatacaaaattgtcaagaaaggggactcaacaattatgaaaagtgagttggaatatggtaactgcaacaccaaatgtcaaactccaataggggcgataaactccagtatgccattccacaacatccaccctctcaccatcggggaatgccccaaatatgtgaaatcaaacagattagtccttgctactgggctcagaaatagccctcaaggagagagaagaagaagaaagaga

>H5N1_A/Egypt/3300-NAMRU3/2008

taccatgcaaacaactcaacagagcaggttgacacaataatggaaaagaacgtcactgttacacacgctcaagacatactggaaaagacacacaacgggaaactctgcgatctagatggagtgaagcctctaattttaagagattgtagtgtagctggatggctcctcgggaacccaatgtgtgacgaattcctcaatgtgtcggaatggtcttacatagtggagaagatcaatccagccaatgacctctgttatccagggaatttcaacaactatgaagaactgaaacacctattgagcagaataaaccgttttgagaaaattcagatcatccccaaaagttcttggccagatcatgaagcctcattaggagtgagctcagcatgtccataccagggaggaccctccttttatagaaatgtggtatggcttatcaaaaagaacaatacatacccaacaataaagaaaagttaccataataccaatcaagaagatcttttggtgctgtgggggattcaccatccaaatgatgaggcagagcagacaaggatctataaaaacccaactacctatatttccgttgggacatcaacactaaaccagagattggtaccaaagatagccactagatctaaggtaaacgggcaaagtggaagggtggagttcttttggacaattttaaaatcaaatgatacaataaactttgagagtaatggaaatttcattgctccagaaaatgcatacaaaattgtcaagaaaggggactcaacaattatgaaaagtgagttggaatatggtaactgcaacaccaagtgtcaaactccaataggggcgataaactccagtatgccattccacaacatccaccctctcaccatcggggaatgccccaaatatgtgaaatcaaacagattagtccttgctactgggctcagaaatagccctcaaggagagagaagaagaaaaaagaga

>H5N1_A/Egypt/3401-NAMRU3/2008

taccatgcaaacaactcgacagagcaggttgacacaataatggaaaagaacgtcactgttacacacgcccaagacatactggaaaagacacacaacgggaaactctgcgatctaaatggagttaagcctctaattttaagagattgtagtgtagctggatggctcctcgggaacccaatgtgtgacgaattcctcaatgttccggaatggtcttacatagtggagaagatcaatccagccaatgacctctgttacccagggaatttcaatgactatgaagaactgaaacacctattgagcagaataaaccattttgagaaaattcagatcatccccaaaagttcttggtcaaatcatgaagcctcatcaggagtgagctcagcatgtccataccagggaagatcctccttttttagaaatgtggtatggcttatcaaaaagggcaatgcatacccaacaataaagataagttacaataataccaaccaagaagatcttttggtactgtgggggattcaccatccaaatgatgcggcagagcagacaaggctctatcaaaacccaactacctatatttccgttgggacatcaacattaaaccagagattggtaccaaaaatagctactagatctaaggtaaacgggcaaagtggaaggatggagttcttttggacaattttaaaatcgaatgatgcaataaactttgagagtaatggaaatttcattgctccagaaaatgcatacaaaattgtcaagaaaggagattcaacaattatgaaaagtgagttggaatatggtaactgcaacaccaagtgtcaaactccaataggggcgataaactccagtatgccattccacaacatccaccctctcaccatcggggaatgccccaaatatgtgaaatcaaacagattagtccttgctactgggctcagaaatagccctcaaggagagagaagaagaaaaaagaga

>H5N1_A/Egypt/3458-NAMRU3/2006

taccatgcaaacaactcgacagagcaggttgacacaataatggaaaagaacgtcactgttacacacgcccaagacatactggaaaagacacacaacgggaaactctgcgatctagatggagtgaagcctctaattttaagagattgtagtgtagctggatggctcctcgggaacccaatgtgtgacgaattcctcaatgtgccggaatggtcttacatagtggagaagatcaatccagccaatgacctctgttacccagggaatttcaacgactatgaagaactgaaacacctattgagcagaataaaccattttgagaaaattcagatcatccccaaaagttcttggtcagatcatgaagcctcatcgggagtgagctcagcatgtccataccagggaagatcctccttttttagaaatgtggtatggcttatcaaaaaggacaatgcatacccaacaataaagagaagttacaataataccaaccaagaagatcttttggtactgtgggggattcaccatccaaatgatgcggcagagcagacaaggctctatcaaaacccaactacctatatttccgttgggacatcaacactaaaccagagattggtaccaaaaatagctactagatctaaggtaaacgggcaaagtggaaggatggagttcttttggacaattttaaaatcgaatgatgcaataaactttgagagtaatggaaatttcattgctccagaaaatgcatacaaaattgtcaagaaaggggactcaacaattatgaaaagtgagttggaatatggtaactgcaacaccaagtgtcaaactccaataggggcgataaactccagtatgccattccacaacatccaccctctcaccatcggggaatgccccaaatatgtgaaatcaaacagattagtccttgctactgggctcagaaatagccctcaaggagagagaagaagaaaaaagaga

>H5N1_A/Egypt/4081-NAMRU3/2007

taccatgcaaacaactcgacagaacaggttgacacaataatggaaaagaacgtcactgttacacacgcccaagacatactggaaaagacacacaacgggaaactctgcgatctagatggagtgaagcctctaattttaagagattgtagtgtagctggatggctcctcgggaacccaatgtgtgacgaattcctcaatgtgccggaatggtcttacatagtggagaagatcaatccagccaatgacctctgttacccaggggatttcaacgactatgaagaactgaaacacctattgagcagaataaaccattttgagaaaatccagatcatccccaaaagttcttggtcagatcatgaagcctcatcaggagtgagctcagcatgtccataccagggaagatcctccttttttagaaatgtggtatggcttatcaaaaaggacaatgcatacccaacaataaagagaagttacaataataccaaccaagaagatcttttggtactgtgggggattcaccatccgaatgatgcggcagagcagacaaggctctatcaaaacccaactacctatatttccgttgggacatcaacactaaaccagagattggtaccaaaaatagctactagatctaaggtaaatgggcaaagtggaaggatggagttcttttggacaattttaaaatcgaatgatgcaataaactttgagagtaatggaaatttcattgctccagaaaatgcatacaaaattgtcaagaaaggggactcaacaattatgaaaagtgagttggaatatggtaactgcaacaccaagtgtcaaactccaataggggcgataaactccagtatgccattccacaacatacaccctctcaccatcggggaatgccccaaatatgtgaaatcaaacagattagtccttgctactgggctcagaaatagccctcaaggagagagaagaagaaaaaagaga

>H5N1_A/Egypt/4082-NAMRU3/2007

taccatgcaaacaactcgacagaacaggttgacacaataatggaaaagaacgtcactgttacacacgcccaagacatactggaaaagacacacaacgggaaactctgcgatctagatggagtgaagcctctaattttaagagattgtagtgtagctggatggctcctcgggaacccaatgtgtgacgaattcctcaatgtgccggaatggtcttacatagtggagaagatcaatccagccaatgacctctgttacccaggggatttcaacgactatgaagaactgaaacacctattgagcagaataaaccattttgagaaaatccagatcatccccaaaagttcttggtcagatcatgaagcctcatcaggagtgagctcagcatgtccataccagggaagaccctccttttttagaaatgtggtatggcttatcaaaaaggacaatgcatacccaacaataaagagaagttacaataataccaaccaagaagatcttttggtactgtgggggattcaccatccgaatgatgcggcagagcagacaaggctctatcaaaacccaactacctatatttccgttgggacatcaacactaaaccagagattggtaccaaaaatagctactagatctaaggtaaatgggcaaagtggaaggatggagttcttttggacaattttaaaatcgaatgatgcaataaactttgagagtaatggaaatttcattgctccagaaaatgcatacaaaattgtcaagaaaggggactcaacaattatgaaaagtgagttggaatatggtaactgcaacaccaagtgtcaaactccaataggggcgataaactccagtatgccattccacaacatacaccctctcaccatcggggaatgccccaaatatgtgaaatcaaacagattagtccttgctactgggctcagaaatagccctcaaggagagagaagaagaaaaaagaga

>H5N1_A/Egypt/4226-NAMRU3/2007

taccatgcaaacaactcgacagaacaggttgacacaataatggaaaagaacgtcactgttacacacgcccaagacatactggaaaagacacacaacgggaaactctgcgatctagatggagtgaagcctctaattttaagagattgtagtgtagctggatggctcctcgggaacccaatgtgtgacgaattcctcaatgtgccggaatggtcttacatagtggagaagatcaatccagccaatgacctctgttacccaggggatttcaacgactatgaagaactgaaacacctattgagcagaataaaccattttgagaaaatccagatcatccccaaaagttcttggtcagatcatgaagcctcatcaggagtgagctcagcatgtccataccagggaagatcctccttttttagaaatgtggtatggcttatcaaaaaggacaatgcatacccaacaataaagagaagttacaataataccaaccaagaagatcttttggtactgtgggggattcaccatccgaatgatgcggcagagcagacaaggctctatcaaaacccaactacctatatttccgttgggacatcaacactaaaccagagattggtaccaaaaatagctactagatctaaggtaaatgggcaaagtggaaggatggagttcttttggacaattttaaaatcgaatgatgcaataaactttgagagtaatggaaatttcattgctccagaaaatgcatacaaaattgtcaagaaaggggactcaacaattatgaaaagtgagttggaatatggtaactgcaacaccaagtgtcaaactccaataggggcgataaactccagtatgccattccacaacatacaccctctcaccatcggggaatgccccaaatatgtgaaatcaaacagattagtccttgctactgggctcagaaatagccctcaaggagagagaagaagaaaaaagaga

>H5N1_A/Egypt/4935-NAMRU3/2009

taccatgcaaacaactcgacagagcaggttgacacaataatggaaaagaacgtcactgttacacacgcccaagacatactggaaaagacacacaacgggaaactctgcaatctagatggagtgaagcctctaattttaagagattgtagtgtagccggatggctcctcgggaacccaatgtgcgacgaattcctcaatgtgccggaatggtcttacatagtggagaagatcaatccagccaatgacctctgttatccagggaatttcaacgactatgaagaactgaaacacctattgagcagaataaaccattttgagaaaattcagatcatccccaaaaattcttggtcagatcatgaagccGGGtcaggagtgagctcagcatgtccataccagggaagatcctccttttttagaaatgtggtatggcttaccaaaaaggacaatgcatacccaacaataaagagaagttacaataataccaaccaagaagatcttttggtactatgggggattcaccatccaaatgatgcggcagagcagacaaggctttatcaaaacccaactacctatatttccgttgggacatcaacactaaaccagagattggtaccaaaaatagctactagatctaaggtaaacggacaaagtggaaggatggagttcttttggacaattttaaaatcgaatgatgcaataaactttgagagtaatggaaatttcattgctccagaaaatgcatacaaaattgtcaagaaaggggactcaacaattatgaaaagtgagttggaatatggtaactgcaacaccaagtgtcagactccaataggggcgataaactccagtatgccattccacaacatccaccctctcaccatcggggaatgccccaaatatgtgaaatcaaacagattagtccttgctactgggctcagaaatagccctcaaggagagagaagaagaaaaaagaga

>H5N1_A/Egypt/5494-NAMRU3/2006

taccatgcaaacaactcgacagagcaggttgacacaataatggaaaagaacgtcactgttacacacgcccaagacatactggaaaagacacacaacgggaaactctgcgatctagatggagtgaagcctctaattttaagagattgtagtgtagctggatggctcctcgggaacccaatgtgtgacgaattcctcaatgtgccggaatggtcttacatagtggagaagatcaatccagccaatgacctctgttacccagggaatttcaacgactatgaagaactgaaacacctattgagcagaataaaccattttgagaaaattcagatcatccccaaaagttcttggtcagatcatgaagcctcatcaggagtgagctcagcatgtccataccagggaagatcctccttttttagaaatgtggtatggcttatcaaaaaggacaatgcatacccaacaataaagagaagttacaataataccaaccaagaagatcttttggtactgtgggggatccaccatccaaatgatgcggcagagcagacaaggctctatcaaaacccaactacctatatttccgttgggacatcaacactaaaccagagattggtaccaaaaatagctactagatctaaggtaaacgggcaaagtggaaggatggagttcttttggacaattttaaaatcgaatgatgcaataaactttgagagtaatggaaatttcattgctccagaaaatgcatacaaaattgtcaagaaaggggactcaacaattatgaaaagtgagttggaatatggtaactgcaacaccaagtgtcaaactccaataggggcgataaactccagtatgccattccacaacatccaccctctcaccatcggggaatgccccaaatatgtgaaatcaaacagattagtccttgctactgggctcagaaatagccctcaaggagagagaagaagaaaaaagaga

>H5N1_A/Egypt/5614-NAMRU3/2006

taccatgcaaacaactcgacagagcaggttgacacaataatggaaaagaacgtcactgttacacgcgcccaagacatactggaaaagacacacaacgggaaactctgcgatctagatggagtgaagcctctaattttaagagattgtagtgtagctggatggctcctcgggaacccaatgtgtgacgaattcctcaatgtgccggaatggtcttacatagtggagaagatcaatccagccaatgacctctgttacccagggaatttcaacgactatgaagaactgaaacacctattgagcagaataaaccattttgagaaaattcagatcatccccaaaagttcttggtcagatcatgaagcctcatcaggagtgagctcagcatgtccataccagggaagatcctccttttttagaaatgtggtatggcttatcaaaaaggacaatgcatacccaacaataaagagaagttacaataataccaaccaagaagatcttttgatactgtgggggattcaccatccaaatgatgcggcagagcagacaaggctctatcaaaacccaactacctatatttccgttgggacatcaacactaaaccagagattggtaccaaaaatagctactagatctaaggtaaacgggcaaagtggaaggatggagttcttttggacaattttaaaatcgaatgatgcaataaactttgagagtaatggaaatttcattgctccagaaaatgcatacaaaattgtcaagaaaggggactcaacaattatgaaaagtgagttggaatatggtaactgcaacaccaagtgtcaaactccaataggggcgataaactccagtatgccattccacaacatccaccctctcaccatcggggaatgccccaaatatgtgaaatcaaacagattagtccttgctactgggctcagaaatagccctcaaggagagagaagaagaaaaaaaaga

>H5N1_A/Egypt/6251-NAMRU3/2007

taccatgcaaacaactcgacagagcaggttgacacaataatggaaaagaacgtcactgttacacacgcccaagacatactggaaaagacacacaacgggaaactctgcgatctagatggagttaagcctctaattttaagagattgtagtgtagctggatggctcctcgggaacccaatgtgtgacgaattcctcaatgttccggaatggtcttacatagtggagaagatcaatccagccaatgacctctgttacccagggaatttcaatgactatgaagaactgaaacacctattgagcagaataaaccattttgagaaaattcagatcatccccaaaagttcttggtcaaatcatgaagcctcatcaggagtgagctcagcatgtccataccagggaagatcctccttttttagaaatgtggtatggcttatcaaaaagggcaatgcatacccaacaataaagataagttacaataataccaaccaagaagatcttttggtactgtgggggattcaccatccaaatgatgcggcagagcagacaaggctctatcaaaacccaactgcctatatttccgttgggacatcaacattaaaccagagattggtaccaaaaatagctactagatctaaggtaaacgggcaaagtggaaggatggagttcttttggacaattttaaaatcgaatgatacaataaactttgagagtaatggaaatttcattgctccggaaaatgcatacaaaattgtcaagaaaggggactcaacaattatgaaaagtgagttggaatatggtaactgtaacaccaagtgtcaaactccaataggggcgataaactccagtatgccattccacaacatccaccctctcaccatcggggaatgccccaaatatgtgaaatcaaacagattagtccttgctactgggctcagaaatagccctcaaggagagagaagaagaaaaaagaga

>H5N1_A/Egypt/682/2015

taccatgcaaacaactcgacagagcaggttgacacaataatggaaaagaatgtcactgttacacacgcccaagacatactggaaaagacacacaacgggaaactctgcaatctagatggagtgaagcctctcattttgagagattgtagtgtagctggatggctcctcgggaacccaatgtgcgatgaattcctcaatgtgccggaatggtcttacatagtggagaaaatcaatccagccaatgacctctgttatccagggaatttcaacgactatgaagaactgaaacacctattgagcagaataaaccattttgagaaaattcagatcattcccaaagattcttggtcagatcatgaagccGGGtcgggagtgagctcagcatgctcataccaaggaagatcctccttttttagaaatgttgtatggcttaccaaaaagaacgatgcatacccaacaataaagaaaagttacaataatactaaccaagaagatcttttggtactatgggggattcaccacccaaatgatgctgcagagcagacaaggctttatcaaaacccaactacctatatctccgttgggacatcaacactaaaccagagattggtacccaaaatagctactagatctaaagtaaacgggcaaagtggaaggatggagttcttttggacaattttaaaatcgaatgatgcaataaactttgagagcaatggaaacttcattgctccagaaaatgcatacaaaattgtcaagaaaggagattcaacaattatgaaaagtgagttggaatatagtaactgcaacaccaagtgtcagactccaataggggcgataaactccagtatgccattccacaacatccaccctctcaccatcggggaatgccccaaatatgtgaaatcaaacagattagtccttgctactgggctcaggaatagccctcaaggagagaaaagaagaaaaaagaga

>H5N1_A/Egypt/7021-NAMRU3/2006

taccatgcaaacaactcgacagagcaggttgacacaataatggaaaagaacgtcactgttacacacgcccaagacatactggaaaagacacacaacgggaaactctgcgatctagatggagtgaagcctctaattttaagagattgtagtgtagctggatggctcctcgggaacccaatgtgtgacgaattcctcaatgtgccggaatggtcttacatagtggagaagatcaatccagccaatgacctctgttacccagggaatttcaacgactatgaagaactgaaacacctattgagcagaataaaccattttgagaaaattcagatcatccccaaaagttcttggtcagatcatgaagcctcatcaggagtgagctcagcatgtccataccagggaagatcctccttttttagaaatgtggtatggcttatcaaaaaggacaatgcatacccaacaataaagagaagttacaataataccaaccaagaagatcttttggtactgtgggggattcaccatccgaatgatgcggcagagcagacaaggctctatcaaaacccaactacctatatttccgttgggacatcaacactaaaccagagattggtaccaaaaatagctactagatctaaggtaaacgggcaaagtggaaggatggagttcttttggacaattttaaaatcgaatgatgcaataaactttgagagtaatggaaatttcattgctccagaaaatgcatacaaaattgtcaagaaaggggactcaacaattatgaaaagtgagttggaatatggtaactgcaacaccaagtgtcaaactccaataggggctataaactccagtatgccattccacaacatccaccctctcaccatcggggaatgccccaaatatgtgaaatcaaacagattaatccttgctactgggctcagaaatagccctcaaggagagagaagaagaaaaaagaga

>H5N1_A/Egypt/902782/2006

taccatgcaaacaactcgacagagcaggttgacacaataatggaaaagaacgtcactgttacacacgcccaagacatactggaaaagacacacaacgggaaactctgcgatctagatggagtgaagcctctaattttaagagattgtagtgtagctggatggctcctcgggaacccaatgtgtgacgaattcctcaatgtgccggaatggtcttacatagtggagaagatcaatccagccaatgacctctgttacccagggaatttcaacgactatgaagaactgaaacacctattgagcagaataaaccattttgagaaaattcagatcatccccaaaagttcttggtcagatcatgaagcctcatcaggagtgagctcagcatgtccataccagggaagatcctccttttttagaaatgtggtatggcttatcaaaaaggacaatgcatacccaacaataaagagaagttacaataataccaaccaagaagatcttttggtactgtgggggattcaccatccgaatgatgcggcagagcagacaaggctctatcaaaacccaactacctatatttccgttgggacatcaacactaaaccagagattggtaccaaaaatagctactagatctaaggtaaacgggcaaagtggaaggatggagttcttttggacaattttaaaatcgaatgatgcaataaactttgagagtaatggaaatttcattgctccagaaaatgcatacaaaattgtcaagaaaggggactcaacaattatgaaaagtgagttggaatatggtaactgcaacaccaagtgtcaaactccaataggggcgataaactccagtatgccattccacaacatccaccctctcaccatcggggaatgccccaaatatgtgaaatcaaacagattaatccttgctactgggctcagaaatagccctcaaggagagagaagaagaaaaaagaga

>H5N1_A/Egypt/902786/2006

taccatgcaaacaactcgacagagcaggttgacacaataatggaaaagaacgtcactgttacacacgcccaagacatactggaaaagacacacaacgggaaactctgcgatctagatggagtgaagcctctaattttaagagattgtagtgtagctggatggctcctcgggaacccaatgtgtgacgaattcctcaatgtgccggaatggtcttacatagtggagaagatcaatccagccaatgacctctgttacccagggaatttcaacgactatgaagaactgaaacacctattgagcagaataaaccattttgagaaaattcagatcatccccaaaagttcttggtcagatcatgaagcctcatcaggagtgagctcagcatgtccataccagggaagatcctccttttttagaaatgtggtatggcttatcaaaaaggacaatgcatacccaacaataaagagaagttacaataataccaaccaagaagatcttttggtactgtgggggattcaccatccaaatgatgcggcagagcagacaaggctctatcaaaacccaactacctatatttccgttgggacatcaacactaaaccagagattggtaccaaaaatagctactagatctaaggtaaacgggcaaagtggaaggatggagttcttttggacaattttaaaatcgaatgatgcaataaactttgagagtaatggaaatttcattgctccagaaaatgcatacaaaattgtcaagaaaggggactcaacaattatgaaaagtgagttggaatatggtaactgcaacaccaagtgtcaaactccaataggggcgataaactccagtatgccattccacaacatccaccctctcaccatcggggaatgccccaaatatgtgaaatcaaacagattagtccttgctactgggctcagaaatagccctcaaggagagagaagaagaaaaaagaga

>H5N1_A/Egypt/9538-NAMRU3/2009

taccatgcaaacaactcgacagagcaggttgacacaataatggaaaagaacgtcactgttacacacgcccaagacatactggaaaagacacacaacgggaaactctgcaatctagatggagtgaagcctctaattttaagagattgtagtgtagctggatggctcctcgggaacccaatgtgcgacgaattcctcaatgtgccggaatggtcttacatagtggagaagatcaatccagccaatgacctctgttatccagggaatttcaacgactatgaagaactgaaacacctattgagcagaataaaccattttgagaaaattcagatcattcccaaaaattcttggtcagatcatgaagccGGGtcaggagtgagctcagcatgtccataccagggaagatcctccttttttagaaatgtggtatggcttaccaaaaagaacaatgcatacccaacaataaagaaaagttacaataataccaaccaagaagatcttttggtactatgggggattcaccatccaaatgatgcggcagagcagacaaggctttatcaaaacccaactacctatatttccgttgggacatcaacactaaaccagagattggtacccaaaatagctactagatctaaggtaaacgggcaaagtggaaggatggagttcttttggacaattttaaaatcgaatgatgcaataaactttgagagtaatggaaatttcattgctccagaaaatgcatacaaaattgtcaagaaaggggactcaacaattatgaaaagtgagttggaatatggtaactgcaacaccaagtgtcagactccaataggggcgataaactccagtatgccattccacaacatccaccctctcaccatcggggaatgccccaaatatgtgaaatcaaacagattagtccttgctactgggctcagaaatagccctcaaggagagagaagaagaaaaaagaga

>H5N1_A/Egypt/MOH-NRC-7271/2014

taccatgcaaacaactcgacagagcaggttgacacaataatggaaaagaatgtcactgttacacacgcccaagacatactggaaaagacacacaacgggaaactctgcaatctagatggagtgaagcctctcattttgagagattgtagtgtagctggatggctcctcgggaacccaatgtgcgatgaattcctcaatgtgccggaatggtcttacatagtggagaaaatcaatccagccaatgacctctgttatccagggaatttcaacgactatgaagaactgaaacacctattgagcagaataaaccattttgagaaaattcagatcattcccaaagattcttggtcagatcatgaagccGGGtcgggagtgagctcagcatgcccataccaaggaagatcctccttttttagaaatgttgtatggcttaccaaaaagaacgatgcatacccaacaataaagaaaagttacaataatactaaccaagaagatcttttggtactatgggggattcaccacccaaatgatgctgcagagcagacaaggctttatcaaaacccaactacctatatctccgttgggacatcaacactaaaccagagattggtacccaaaatagctactagatctaaggtaaacgggcaaagtggaaggatggagttcttttggacaattttaaaatcgaatgatgcaataaactttgagagcaatggaaacttcattgctccagaaaatgcatacaaaattgtcaagaaaggagattcaacaattatgaaaagtgagttgggatatagtaactgcaacaccaagtgtcagactccaataggggcgataaactccagtatgccattccacaacatccaccctctcaccatcggggaatgccccaaatatgtgaaatcaaacaaattagtccttgctactgggctcaggaatagccctcaaggagagaaaagaagaaaaaagaga

>H5N1_A/Egypt/MOH-NRC-7305/2014

taccatgcaaacaactcgacagagcaggttgacacaataatggaaaagaatgtcactgttacacacgcccaagacatactggaaaagacacacaacgggaaactctgcaatctagatggagtgaagcctctcattttgagagattgtagtgtagctggatggctcctcgggaacccaatgtgcgatgaattcctcaatgtgccggaatggtcttacatagtggagaaaatcaatccagccaatgacctctgttatccagggaatttcaacgactatgaagaactgaaacacctattgagcagaataaaccattttgagaaaattcagatcattcccaaagattcttggtcagatcatgaagccGGGtcgggagtgagctcagcatgcccataccaaggaagatcctccttttttagaaatgttgtatggcttaccaaaaagaacgatgcatacccaacaataaagaaaagttacaataatactaaccaagaagatcttttggtactatgggggattcaccatccaaatgatgctgcagagcagacaaggctttatcaaaacccaactacctatatctccgttgggacatcaacactaaaccagagattggtacccaaaatagctactagatctaaggtaaacgggcaaagtggaaggatggagttcttttggacaattttaaaatcgaatgatgcaataaactttgagagcaatggaaacttcattgctccagaaaatgcatacaaaattgtcaagaaaggagattcaacaattatgaaaagtgagttggaatatagtaactgcaacaccaagtgtcagactccaataggggcgataaactccagtatgccattccacaacatccaccctctcaccatcggggaatgccccaaatatgtgaaatcaaacagattagtccttgctactgggctcaggaatagccctcaaggagagaaaagaagaaaaaagaga

>H5N1_A/Egypt/MOH-NRC-8434/2014

taccatgcaaacaactcgacagagcaggttgacacaataatggaaaagaacgtcactgttacacacgcccaagacatactggaaaagacacacaacgggaaactctgcaatctagatggagtgaagcctctcattttaagagattgtagtgtagctggatggctcctcgggaacccaatgtgcgatgaattcctcaatgtgccggaatggtcttacatagtggagaaaatcaatccagccaatgacctctgttatccagggaatttcaacgactatgaagaactgaaacacctattgagcagaataaaccattttgagaaaattcagatcattcccaaagattcctggtcagatcatgaaaccGGGtcaggagtgagctcagcatgcccataccaaggaagatcctccttttttagaaatgttgtgtggcttaccaaaaagaacgatgcatacccaacaataaagaaaagttacaataatactaaccaagaagatcttttggtactatgggggattcaccatccaaatgatgctgcagagcagacaaggctttatcaaaacccaactacctacatctccgttgggacatcaacactaaaccagagattggtacccaaaatagctaccagatctaaggtaaacgggcaaaatggaaggatggagttcttttggacaattttaaaatcgaatgatgcaataaactttgagagcaatgggaacttcattgctccagaaaatgcatacaaaattgtcaagaaaggagattcaacaattatgagaagtgagttggaatatagtaactgcaacaccaagtgtcagactccaataggggcgataaactccagtatgccattccacaacattcaccctctcaccatcggggaatgccccaaatatgtgaaatcaaacagattagtccttgctactgggctcaggaatagccctcaaggagagaaaagaagaaaaaagaga

>H5N1_A/Egypt/N00001/2009

taccatgcaaacaactcgacagagcaggttgacacaataatggaaaagaacgtcactgttacacacgcccaagacatactggaaaagacacacaacgggaaactctgcaatctagatggagtgaagcctctaattttaagagattgtagtgtagccggatggctcctcgggaacccaatgtgcgacgaattccttaatgtgccggaatggtcttacatagtggagaagatcaatccagccaatgacctctgttatccagggaatttcaacgactatgaagaactgaaacacctattgagcagaataaaccattttgagaaaattcagatcatccccaaaaattcttggtcagatcatgaagccGGGtcaggagtgagctcagcatgtccataccagggaagaccctccttttttagaaatgtggtatggcttaccaaaaaggacaatgcatacccaacaataaagagaagttacaataataccaaccaagaagatcttttggtactatgggggattcaccatccamatgatgcggcagagcagacaaggctttatcaaaacccaactacctatatttccgttgggacatcaacactaaaccagagattggtaccaaaaatagctactagatctaaggtaaacgggcaaagtggaaggatggagttcttttggacaattttaaaatcgaatgatgcaataaactttgagagtaatggaaatttcattgctccagaaaatgcatacaaaattgtcaagaaaggggactcaacaattatgaaaagtgagttggaatatggtaactgcaacaccaagtgtcagactccaataggggcgataaactccagtatgccattccacaacatccaccctctcaccatcggggaatgccccaaatatgtgaaatcaaacagattagtccttgctactgggctcagaaatagccctcaaggagagagaagaagaaaaaagaga

>H5N1_A/Egypt/N00002/2011

taccatgcaaacaactcgacagagcaggttgacacaataatggaaaagaacgtcactgttacacacgtccaagacatactggaaaagacacacaacgggaaactctgcaatctagatggagtgaagcctctaattttaagagattgtagtgtagctggatggctcctcgggaacccaatgtgcgacgaattcctcaatgtgccggaatggtcttacatagtggagaagatcaatccagccaatgacctctgttatccagggaatttcaacgactatgaagaactgaaacacctattgagcagaataaaccattttgagaaaattcagatcattcccaaagattcttggtcagatcatgaagccGGGtcaggagtgagctcagcatgtccataccagggaagatcctccttttttagaaatgtggtatggcttaccaaaaagaacaatgcatacccaacaataaagaaaagttacaataataccaaccaagaagatcttttggtactatgggggattcaccatccaaatgatgctgcagatcagacaaggctttatcaaaacccaactacctatatttccgttgggacatcaacactaaaccagagattggtacccaaaatagctactagatctaaggtaaacgggcaaagtggaaggatggagttcttttggacaattttaaaatcgaatgatgcaataaactttgagagtaatgggaacttcattgctccagaaaatgcatacaaaattgtcaagaaaggggactcaacaattatgaaaagtgagttggaatatagtaactgcaacaccaagtgtcagactccaataggggcgataaactccagtatgccattccacaacatccaccctctcaccatcggggaatgccccaaatatgtgaaatcaaacagattagtccttgctactgggctcagaaatagccctcaaggagagaaaagaagaaaaaagaga

>H5N1_A/Egypt/N00166/2011

taccatgcaaacaactcgacagagcaggttgacacaataatggaaaagaacgtcactgttacacacgcccaagacatactggaaaagacacacaacgggaaactctgcaatctagatggagtgaagcctctaattttaagagattgtagtgtagctggatggctcctcgggaacccaatgtgcgacgaattcctcaatgtgccggaatggtcttacatagtggaaaagatcaatccagccaatgacctctgttatccagggaatttcaacgactatgaagaactgaaacacctattgagcagaataaaccattttgagaaaattcagatcattcccaaagattcttggtcagatcatgaagccGGGtcaggagtgagctcagcatgtccataccagggaagatcctccttttttagaaatgtggtatggcttaccaaaaagaacgatgcatacccaacaataaagaaaagttacaataataccaaccaagaagatcttttggtactatgggggattcaccatccaaatgatgctgcagagcagacaaggctttatcaaaacccaactacctatatttccgttgggacatcaacactaaaccagagattggtacccaaaatagctactagatctaaggtaaacgggcaaagtggaaggatggagttcttttggacaattttaaaatcgaatgatgcaataaactttgagagtaatggaaacttcattgctccagaaaatgcatacaaaattgtcaagaaaggggactcaacaattatgaaaagtgagttggaatatagtaactgcaacaccaagtgtcagactccaataggggcgataaactccagtatgccattccacaacatccaccctctcaccatcggggaatgccccaaatatgtgaaatcaaacagattagtccttgctactgggctcagaaatagccctcaaggagagaaaagaagaaaaaagaga

>H5N1_A/Egypt/N00269/2010

taccatgcaaacaactcgacagagcaggttgacacaataatggaaaagaacgtcactgttacacacgcccaagacatactggaaaagacacacaacgggaaactctgcaatctagatggagtgaagcctctaattttaagagattgtagtgtagccggatggctcctcgggaacccaatgtgcgacgaattcctyaatgtgccggaatggtcttacatagtggagaagatcaatccagccaatgacctctgttatccagggaatttcaacgactatgaagaactgaaacacctattgagcagaataaaccattttgagaaaattcagatcatccccaaaaattcttggtcagatcatgaagccGGGtcaggagtgagctcagcatgtccataccagggaagatcctccttttttagaaatgtggtatggcttaccaaaaaggacaatgcatacccaacaataaagagaagttacaataataccaaccaagaagatcttttggtactatgggggattcaccacccaaatgatgcgacagagcagacaaggctttatcaaaacccaactacctatatttccgttgggacatcaacactaaaccagagattggtaccaaagatagctactagatctaaggtaaacgggcaaartggaaggatggagttcttttggacaattttaaaatcgaatgatgcaataaactttgagagtaatggaaatttcattgctccagaaaatgcatacaaaattgtcaagaaaggggactcaacaattatgaaaagtgagttggaatatggtaactgcaacaccaagtgtcagactccaataggggcgataaactccagtatgccattccacaacatccaccctctcaccatcggggaatgccccaaatatgtgaaatcaaacagattagtccttgctactgggctcagaaatagccctcaaggagagagaagaagaaaaaagaga

>H5N1_A/Egypt/N00270/2010

taccatgcaaacaactcaacagagcaggttgacacaataatggaaaagaacgtcactgttacacacgcccaagacatactggaaaaaacacacaacgggaaactctgcaatctagatggagtggagcctctaattttaagagattgtagtgtagctggatggctcctcgggaacccaatgtgcgacgaattcctcaatgtgccggaatggtcttacatagtggagaagatcaatccagccaatgacctctgttatccagggaatttcaacgactatgaagaactgaaacacctattgagcagaataaaccattttgagaaaattcagatcattcccaaaaattcttggtcagatcatgaagccGGGtcaggagtgagctcagcatgtccataccagggaagatcctccttttttagaaatgtggtatggcttaccaaaaagaacaatgcatacccaacaataaagaaaagttacaataataccaaccaagaagatcttttggtactatgggggattcaccatccaaatgatgcggcagagcagacaaggctttatcaaaacccaactacctatatttccgttgggacatcaacactaaaccagagattggtaccaaaaatagctactagatctaaggtaaacgggcaaagtggaaggatggagttcttttggacaattttaaaatcgaatgatgtaataaactttgagagtaatggaaatttcattgctccagaaaatgcatacaaaattgtcaagaaaggggactcaacaattatgaaaagtgagttggaatatggtaactgcaacaccaagtgtcagactccaataggggcgataaactccagtatgccattccacaacatccaccctctcaccatcggggaatgccccaaatatgtgaaatcagacagactagtccttgctactgggctcagaaatagccctcaaggagagagaagaagaaaaaagaga

>H5N1_A/Egypt/N00585/2009

taccatgcaaacaactcgacagagcaggttgacacaataatggaaaagaacgtcactgttacacacgcccaagacatactggaaaagacacacaacgggaaactctgcaatctagatggagtgaagcctctaattttaagagattgtagtgtagctggatggctcctcgggaacccaatgtgcgacgaattcctcaatgtgccggaatggtcttacatagtggagaagatcaatccagccaatgacctctgttatccagggaatttcaacgactatgaagaactgaaacacctattgagcagaataaaccattttgagaaaattcagatcattcccaaaaattcttggtcagatcatgaagccGGGtcaggagtgagctcagcatgtccataccagggaagatcctccttttttagaaatgtggtatggcttaccaaaaagaacaatgcatacccaacaataaagagaagttacaataataccaaccaagaagatcttttggtactatgggggattcaccatccaaatgatgcggcagagcagacaaggctttatcaaaacccaactacctatatttccgttgggacatcaacactaaaccagagattggtaccaaaaatagctactagatctaaggtaaacgggcaaagtggaaggatggagttcttttggacaattttaaaatcgaatgatgcaataaactttgagagtaatggaaatttcattgctccagaaaatgcatacaaaattgtcaagaaaggggactcaacaattatgaaaagtgagttggaatatggtaactgcaacaccaagtgtcagactccaataggggcgataaactccagtatgccattccacaacatccaccctctcaccatcggggaatgccccaaatatgtgaaatcaaacagattagtccttgctactgggctcagaaatagccctcaaggagagagaagaagaaaaaagaga

>H5N1_A/Egypt/N00605/2009

taccatgcaaacaactcgacagagcaggttgacacaataatggaaaagaacgtcactgttacacacgcccaagacatactggaaaagacacacaacgggaaactctgcaatctagatggagtgaagcctctaattttaagagattgtagtgtagccggatggctcctcgggaacccaatgtgcgacgaattcctcaatgtgccggaatggtcttacatagtggagaagatcaatccagccaatgacctctgttatccagggaatttcaacgactatgaagaactgaaacacctattgagcagaataaaccattttgagaaaattcagatcatccccaaaaattcttggtcagatcatgaagccGGGtcaggagtgagctcagcatgtccataccagggaagatcctccttttttagaaatgtggtatggcttaccaaaaagaacaatgcatacccaacaataaagagaagttacaataataccaaccaagaagatcttttggtactatgggggattcaccatccaaatgatgcggcagagcagacaaggctttatcaaaacccaactacctatatttccgttgggacatcaacactaaaccagagattggtaccaaaaataactactagatctaaggtaaacgggcaaagtggaaggatggagttcttttggacaattttaaaatcgaatgatgcaataaactttgagagtaatggaaatttcattgctccagaaaatgcatacaaaattgtcaagaaaggggactcaacaattatgaaaagcgagttggaatatggtaactgcaacaccaagtgtcagactccaataggggcgataaactccagtatgccattccacaacatccaccctctcaccatcggggaatgccccaaatatgtgaaatcaaacagattagtccttgctactgggctcagaaatagccctcaaggagagagaagaagaaaaaagaga

>H5N1_A/Egypt/N00606/2009

taccatgcaaacaactcgacagagcaggttgacacaataatggaaaagaacgtcactgttacacacgcccaagacatactggaaaagacacacaacgggaaactctgcaatctagatggagtgaagcctctaattttaagagattgtagtgtagctggatggctcctcgggaacccaatgtgtgacgaattcctcaatgtgccggaatggtcttacatagtggagaagatcaatccagccaatgacctctgttatccagggaatttcaacgactatgaagaactgaaacatctattgagcagaataaaccattttgagaaaattcagatcatccccaaaaattcttggtcagatcatgaagccGGGtcaggagtgagctcagcatgtccataccagggaaggtcctccttttttagaaatgtggtatggcttaccaaaaaggacaatgcatacccaacaataaagagaagttacaataataccaaccaagaagatcttttggtactatggggaattcaccatccaaatgatgcggcagagcagacaaggctttatcaaaacccaactacctatatttccgttgggacatcaacactaaaccagagattgataccaaaaatagctactagatctaaggtaaacgggcaaagtggaaggatggagttcttttggacaattttaaaatcgaatgatgcaataaactttgagagtaatggaaatttcattgctccagaaaatgcatacaaaattgtcaagaaaggggactcaacaattatgaaaagtgagttggaatatggtaactgcaacaccaagtgtcagactccaataggggcgataaactccagtatgccattccacaacatccaccctctcaccatcggggaatgccccaaatatgtgaaatcaaacagattagtccttgctactgggctaagaaatagccctcaaggagagagaagaagaaaaaagaga

>H5N1_A/Egypt/N00951/2012

taccatgcaaacaactcgacagagcaggttgacacaataatggaaaagaacgtcactgttacacacgcccaagacatactggaaaagacacacaacgggaaactctgcaatctagatggagtgaagcctctaattttaagagattgtagtgtagctggatggctcctcgggaacccaatgtgcgacgaattcctcaatgtgccggaatggtcttacatagtggagaagatcaatccagccaatgacctctgttatccagggaatttcaacgactatgaagaactgaaacacctattgagcagaataaaccattttgagaaaattcagatcattcccaaagattcttggtcagatcatgaagccGGGtcaggagtgagctcagcatgtccataccagggaagatcctccttttttagaaatgtggtatggcttaccaaaaagaacgatgcatacccgacaataaagaaaagttacaataataccaaccaagaagatcttttggtactatgggggattcaccatccaaatgatgctgcagagcagacaaggctttatcaaaacccaactacctatatttccgttgggacatcaacactaaaccagagattggtacccaaaatggctactagatctaaggtaaacgggcaaaatggaaggatggagttcttttggacaattttaaaatcgaatgatgcaataaactttgagagtaatggaaacttcattgctccagaaaatgcatacaaaattgtcaagaaaggggactcaacaattatgaaaagtgagttggaatatagtaactgcaacaccaagtgtcagactccaataggggcgataaactccagtatgccattccacaacatccaccctctcaccatcggggagtgccccaaatatgtgaaatcaaacagattagtccttgctactgggctcagaaacagccctcaaggagagaaaagaagaaaaaagaga

>H5N1_A/Egypt/N01310/2009

taccatgcaaacaactcgacagagcaggttgacacaataatggaaaagaacgtcactgttacacacgcccaagacatactggaaaagacacacaacgggaaactctgcaatctagatggagtgaagcctctaattttaagagattgtagtgtagccggatggcttctcgggaacccaatgtgcgacgaattcctcaatgtgccggaatggtcttacatagtggagaagatcaatccagccaatgacctctgttatccagggaatttcaacgactatgaagaactgaaacacctattgagcagaataaaccattttgagaaaattcagatcatccccaaaaattcttggtcagatcatgaagccGGGtcaggagtgagctcagcatgtccataccagggaagatcctccttttttagaaatgtggtatggcttaccaaaaaggacaatgcatacccaacaataaagagaagttacaataataccaaccaagaagatcttttggtactatgggggattcaccatccaaatgatgcggcagagcagacaaggctttatcaaaacccaactacctatatttccgttgggacatcaacactaaaccagagattggtaccaaaaatagctactagatctaaggtaaacggacaaagtggaaggatggagttcttttggacaattttaaaatcgaatgatgcaataaactttgagagtaatggaaatttcattgctccagaaaatgcatacaaaattgtcaagaaaggggactcaacaattatgaaaagtgagttggaatatggtaactgcaacaccaagtgtcagactccaataggggcgataaactccagtatgccattccacaacatccaccctctcaccatcggggaatgccccaaatatgtgaaatcaaacagattagtccttgctactgggctcagaaatagccctcaaggagagagaagaagaaaaaagaga

>H5N1_A/Egypt/N01360/2010

taccatgcaaacaactcgacagagcaggttgacacaataatggaaaagaacgtcactgttacacacgcccaagacatactggaaaagacacacaacgggaaactctgcaacctagatggagtgaagcctctaattttaagagattgtagtgtagccggatggctcctcgggaacccaatgtgcgacgaattcctcaatgtgccagaatggtcttacatagtggagaagatcaatccagccaatgacctctgttatccagggaatttcaacgactatgaagaactgaaacacctattgagcagaataaaccattttgagaaaattcagatcatccccaaaaattcttggtcagatcatgaagccGGGtcaggagtgagctcagcatgtccataccagggaaaatcctccttttttagaaatgtagtatggcttaccaaaaaggacaatgcatacccaacaataaagagaagttacaataataccaaccaagaagatcttttggtactatgggggattcaccatcyaaatgatgcggcagagcagacaaggctttaccaaaacccaactacctatatttccgttgggacatcaacactaaaccagagattggcaccaaaaatagctactagatctaaggtaaacgggcaaagtggaaggatggagttcttttggacaatcttaaaatcgaatgatgcaataaactttgagagtaatggaaatttcattgctccagaaaatgcatacaaaattgtcaagaaaggggactcaacaattatgaaaagtgagttggaatatagtaactgcaacacaaagtgccagactccaataggggcgataaactccagtatgccattccacaacatccaccctctcaccatcggggaatgccccaaatacgtgaaatcaaacagattagtccttgctactgggctcagaaatagccctcaaggagagagaagaagaaaaaagaga

>H5N1_A/Egypt/N01400/2012

taccatgcaaacaactcgacagagcaggttgacacaataatggaaaagaacgtcactgttacacacgcccaagacatactggaaaagacacacaacgggaaactctgcaatctagatggagtgaagcctctaattttaagagattgtagtgtagctggatggctcctcgggaacccaatgtgcgacgaattcctcaatgtgccggaatggtcttacatagtggagaagatcaatccagccaatgacctctgttatccagggaatttcaacgactatgaagaactgaaacacctgttgagcagaataaaccattttgagaaaattcagatcattcccaaagattcttggtcagatcatgaagccGGGtcaggagtgagctcagcatgtccataccagggaagatcctccttttttagaaatgtggtatggcttaccaaaaagaacgatgcatacccaacaataaagaaaagttacaataataccaatcaagaagatcttttggtactatgggggattcaccatccaaatgatgctgcagagcagacaagactttaccaaaacccaactacctatatttctgttgggacatcaacactaaaccagagattggtacccaaaatagctactagatctaaggtaaacgggcaaagtggaaggatggagttcttttggacaattttaaaatcgaatgatgcaataaactttgagagtaatggaaacttcattgctccagaaaatgcatacaaaattgtcaagaaaggggactcaacaattatgaaaagtgagttggaatatagtaactgcaacaccaagtgccagactccaataggggcgataaattccagtatgccattccacaacatccaccctctcaccatcggggaatgccccaaatatgtgaaatcaaacagattagtccttgctactgggctcagaaatagccctcaaggagagaaaagaagaaaaaagaga

>H5N1_A/Egypt/N01644/2010

taccatgcaaacaactcgacagagcaggttgacacaataatggaaaagaacgtcactgttacacacgcccaagacatactggaaaagacacacaacgggaaactctgcaatctagatggagtgaagcctctaattttaagagattgtagcgtagccggatggctcctcgggaacccaatgtgcgacgaattcctcaatgtgccggaatggtcttacatagtggagaagatcaatccagccaatgacctctgttatccagggaatttcaacgactatgaagaactgaaacacctattgagcagaataaaccattttgagaaaattcagatcatccccaaaagttcttggtcagatcatgaagccGGGtcaggagtgagctcagtatgtccataccagggaagatcctccttttttagaaatgtggtatggcttaccaaaaaggacaatgcatacccaacaataaagagaagttacaataataccaaccaagaagatcttttggtactatgggggattcaccatccaaatgatgcggcagagcagacaaggctttatcaaaacccaactacctatatttccgttgggacatcaacactaaaccagagattggtaccaaaaatagctactagatctaaggtaaacgggcaaagtggraggatggagttcttttggacaattttaaaatcgaatgatgcaataaactttgagagtaatggaaatttcattgctccagaaaatgcatacaaaattgtcaagaaaggggactcaacaattatgaaaagtgagttggaatatggtaactgcaacaccaagtgtcagactccaataggggcgataaactccagtatgccattccacaacatccaccctctcaccatcggggaatgccccaaatatgtgaaatcaaacagattagtccttgctactgggctaagaaatagccctcaaggagagagaagaagaaaaaagaga

>H5N1_A/Egypt/N01753/2014

taccatgcaaacaactcaacagagcaggttgacacaataatggaaaagaacgtcactgttacacacgcccaagacatactggaaaagacacacaatgggaaactctgcaatctagatggagtgaagcctctaattttaagagattgtagtgtagctggatggctcctcgggaacccaatgtgcgacgaattcctcaatgtgccggaatggtcttacatagtggaaaggatcaatccagccaatgacctctgttatccagggaatttcaacgactatgaagaactgaaacacctattgagcagaataaaccactttgagaaaattcagatcattcccaaggattcttggtcaaatcatgaagccGGGtcaggagtgagctcagcatgtccataccagggaagatcctccttttttagaaatgtggtatggcttaccaaaaagaacgatgcatacccaacaataaagaaaagttacaataatactaaccaagaagatcttttggtactatgggggattcaccatccaaatgatgccgcagagcagacaaggctttatcaaaaccyaactacctatatttccgttgggacatcaacaataaaccagagattggtacccaaaatagctactagatctaaggtaaacgggcaaagtggaaggatggagttcttttggacaattttaaaatcgaatgatgcaataaactttgagagtaatggaaacttcattgctccagaaaatgcatacaaaattgtcaagaaaggggactcaacaattatgaaaagtgagttggaatatagtaactgcaacaccaagtgtcagactccaataggggcgataaactccagtatgccattccacaacatccaccctctcaccatcggggaatgccccaaatatgtgaaatcaaacagattagtccttgctactgggctcagaaatagccctcaaggagagaaaagaagaaaaaagaga

>H5N1_A/Egypt/N01754/2014

taccatgcaaacaactcgacagagcaggttgacacaataatggaaaagaacgtcactgttacccacgcccaagacatactggaaaagacacacaacgggaaactctgcaatctagatggagtgaagcctctaattttaagagattgtagtgtagctggatggctcctcgggaacccaatgtgcgacgaattcctcaatgtgccggaatggtcttacatagtggagaagatcaatccagccaatgacctctgttacccagggaatttcaacgactatgaagaactgaaacacctattgagcagaataaaccattttgagaaaattcagatcattcccaaagattcttggtcagatcatgaagctGGGtcaggagtgagctcagcatgtccataccagggaagatcctccttttttagaaatgtggtatggcttaccaaaaagaacgatgcatacccaacaataaagaaaagttacaataatactaaccaagaagatcttttggtactatgggggatacaccatccaaatgatgctgcagagcagacaaggctttatcaaaacccaactacctatatttccgttgggacatcaacactaaaccagagattggtacccaaaatagctactagatctaaggtaaacgggcaaagtggaaggatggagttcttttggacaattttaaaatcgaatgatgcaataaactttgagagtaatggaaacttcattgctccagaaaatgcatacaaaattgtcaagaaaggggactcaacaattatgaaaagtgagttggaatatagtaactgcaacaccaagtgtcagactccaataggggcgataaactccagtatgccattccacaacatccaccctctcaccatcggggaatgccccaaatatgtgaaatcaaacagattagtccttgctactgggctcagaaatagccctcaaggagaaaaaagaagaaaaaagaga

>H5N1_A/Egypt/N01982/2010

taccatgcaaacaactcgacagagcaggttgacacaataatggaaaagaacgtcactgttacacacgcccaagacatactggaaaagacacacaacgggaaactctgcaatctagatggagtgaagcctctaattttaagagattgtagtgtagctggatggctcctcgggaacccaatgtgcgacgaattcctcaatgtgccggaatggtcttacatagtggagaagatcaatccagccaatgacctctgttatccagggaatttcaacgactatgaagaactgaaacacctattgagcagaataaaccattttgagaaaattcagatcatccccaaagattcttggtcagatcatgaagccGGGtcaggagtgagctcagcatgtccataccagggaagatcctccttttttagaaatgtggtatggcttaccaaaaagaacaatgcatacccaacaataaagaaaagttacaataataccaaccaagaagatcttttggtactatgggggattcaccatccaaatgatgcggcagagcagacaaggctttatcaaaacccaactacctatatttccgttgggacatcaacactaaaccagagattggtacccaaaatagctactagatctaaggtaaacgggcaaagtggaaggatggagttcttttggacaattttaaaatcgaatgatgcaataaactttgagagtaatggaaatttcattgctccagaaaatgcatacaaaattgtcaagaaaggggactcaacaattatgaaaagtgagttggaatatggtaactgcaacaccaagtgtcagactccaataggggcgataaactccagtatgccattccacaacatccaccctctcaccatcggggaatgccccaaatatgtgaaatcaaacagattagtccttgctactgggctcagaaatagccctcaaggagagagaagaagaaaaaagaga

>H5N1_A/Egypt/N02038/2010

taccatgcaaacaactcaacagagcaggttgacacaataatggaaaagaacgtcactgttacacacgcccaagacatactggaaaagacacacaacgggaaactctgcaatctagatggagtgaagcctctaattttaagagattgtagtgtagctggatggctcctcgggaacccaatgtgcgacgaattcctcaatgtgccggaatggtcttacatagtggagaagatcaatccagccaatgacctctgttatccagggaatttcaacgactatgaagaactgaaacacctattgagcagaataaaccattttgagaaaattcagatcattcccaaaaattcttggtcagatcatgaagccGGGtcaggagtgagctcagcatgtccataccagggaagatcctccttttttagaaatgtggtatggcttacccaaaagaacaatgcatacccaacaataaagaaaagttacaataataccaaccaagaagatcttttggtactatgggggattcaccatccaaatgatgcggcagagcagacaaggctttatcaaaacccaactacctatatttccgttgggacatcaacactaaaccagagattggtaccaaaaatagctactagatctaaagtaaacgggcaaagtggaaggatggagttcttttggacaattttaaaatcgaatgatgtaataaactttgagagtaatggaaatttcattgctccagaaaatgcatacaaaattgtcaagaaaggggactcaacaattatgaaaagtgagttggaatatggtaactgcaacaccaagtgtcagactccaataggggcgataaactccagtatgccattccacaacatccaccctctcaccatcggggaatgccccaaatatgtgaaatcaaacagattagtccttgctactgggctcagaaatagccctcaaggagagagaagaagaaaaaagaga

>H5N1_A/Egypt/N02039/2009

taccatgcaaacaactcgacagagcaggttgacacaataatggaaaagaacgtcactgttacacacgcccaagacatactggaaaagacacacaacgggaaactctgcaatctagatggagtgaagcctctaattttaagagattgtagtgtagccggatggctcctcgggaacccaatgtgcgacgaattcctcaatgtgccggaatggtcttacatagtggagaagatcaatccagccaatgacctctgttatccagggaatttcaacgactatgaagaactgaaacacctattgagcagaataaaccattttgagaaaattcagatcatccccaaaaattcttggtcagatcatgaagccGGGtcaggaatgagctcagcatgtccataccagggaagatcctccttttttagaaatgtggtatggcttaccaaaaaggacaatgcatacccaacaataaagagaagttacaataataccaaccaagaagatcttttggtactatgggggattcaccatccaaatgatgcggcagagcagacaaggctttatcaaaacccaactacctatatttccgttgggacatcaacactaaaccagagattggtaccaaaaatagctactagatctaaggtaaacggacaaagtggaaggatggagttcttttggacaattttaaaatcgaatgatgcaataaactttgagagtaatggaaatttcattgctccagaaaatgcatacaaaattgtcaagaaaggggactcaacaattatgaaaagtgagttggaatatggtaactgcaacaccaagtgtcagactccaataggggcgataaactccagtatgccattccacaacatccaccctctcaccatcggggaatgccccaaatatgtgaaatcaaacagattagtccttgctactgggctcagaaatagccctcaaggagagagaagaagaaaaaagaga

>H5N1_A/Egypt/N02127/2010

taccatgcaaacaactcgacagagcaggttgacacaataatggaaaagaacgtcactgttacacacgcccaagacatactggaaaagacacacaacgggaaactctgcaatctagatggagtgaagcctctaattttaagagattgtagtgtagctggatggctcctcgggaacccaatgtgcgacgaattcctcaatgtgccggaatggtcttacatagtggagaagatcaatccagccaatgacctctgttatccagggaatttcaacgactatgaagaactgaaacacctattgagcagaataaaccattttgagaaaattcagatcattcccaaaggttcttggtcagatcatgaagccGGGtcaggagtgagctcagcatgtccataccagggaagatcctccttttttagaaatgtggtatggcttaccaaaaagaacaatgcatacccaacaataaagaaaagttacaataataccaaccaagaagatcttttggtactatgggggattcaccatccaaatgatgcggcagagcagacaaggctttatcaaaacccaactacctatatttccgttgggacatcaacactaaaccagagattggtaccccaaatagctactagatctaaggtaaacgggcaaagtggaaggatggagttcttttggacaattttaaaatcgaatgatgcaataaactttgagagtaatggaaatttcattgctccagaaaatgcatacaaaattgtcaagaaaggggactcaacaattatgaaaagtgagttggaatatggtaactgcaacaccaagtgtcagactccaataggggcgataaactccagtatgccattccacaacatccaccctctcaccatcggggaatgccccaaatatgtgaaatcaaacagattagtccttgctactgggctcagaaatagccctcaaggagagagaagaagaaaaaagaga

>H5N1_A/Egypt/N02137/2012

taccatgcaaacaactcgacagagcaggttgacacaataatggaaaagaacgtcactgttacacacgcccaagacatactggaaaagacacacaacgggaaactctgcaatctagatggagtgaagcctctaattttaagagattgtagtgtagctggatggctccttgggaacccaatgtgcgacgaattcctcaatgtgccggaatggtcttacatagtggagaagatcaatccagccaatgacctctgttatccagggaatttcaacgactatgaagaactgaaacacctattgagcagaataaaccattttgagaaaattcagatcattcccaaagattcttggtcagatcatgaagtcGGGtcaggagtgagctcagcatgtccataccagggaagatcctcattttttagaaatgtggtatggcttaccaaaaagaacgatgcatacccaacaataaagaaaagttacaataataccaaccaagaagatcttttggtactatgggggattcaccatccaaatgatgctgcagagcagacaaggctttatcaaaacccaactacctatatttccgttgggacatcaacactaaaccagagattggtacccaaaatagctactagatctaaggtaaacgggcaaaatggaaggatggagttcttttggacaattctaaaatcgaatgatgcaataaactttgagagtaatggaaacttcattgctccagaaaatgcatacaaaattgtcaagaaaggggactcaacaattatgaaaagtgagttggaatatagtaactgcaacaccaagtgtcagactccaataggggcaataaactccagtatgccattccacaacatccaccctctcaccatcggggaatgccccaaatatgtgaaatcaaacagattagtccttgctactgggctcagaaatagccctcaaggagagaaaagaagaaaaaagaga

>H5N1_A/Egypt/N02407/2009

taccatgcaaacaactcgacagagcaggttgacacaataatggaaaagaacgtcactgttacacacgcccaagacatactggaaaagacacacaacgggaaactctgcaatctagatggagtgaagcctctaattttaagagattgtagtgtagccggatggctcctcgggaacccaatgtgcgacgaattcctcaatgtgccggaatggtcttacatagtggagaagatcaatccagccaatgacctctgttatccagggaatttcaacgactatgaagaactgaaacacctattgagcagaataaaccattttgagaaaattcagatcatccccaaaaattcttggtcagatcatgaagccGGGtcaggagtgagctcagcatgtccataccagggaagatcctccttttttagaaatgtggtatggcttaccaaaaaggacaatgcatacccaacaataaaaagaagttacaataataccaaccaagaagatcttttggtactatgggggattcaccatccaaatgatgcggcagagcagacaaggctttatcaaaacccaactacctatatttccgttgggacatcaacactaaaccagagattggtaccaaaaatagctactagatctaaggtaaacggacaaagtggaaggatggagttcttttggacaattttaaaatcgaatgatgcaataaactttgagagtaatggaaatttcattgctccagaaaatgcatacaaaattgtcaagaaaggggactcaacaattatgaaaagtgagttggaatatggtaactgcaacaccaagtgtcagactccaataggggcgataaactccagtatgccattccacaacatccaccctctcaccatcggggaatgccccaaatatgtgaaatcaaacagattagtccttgctactgggctcagaaatagccctcaaggagagagaagaagaaaaaagaga

>H5N1_A/Egypt/N02554/2010

taccatgcaaacaactcgacagagcaagttgacacaataatggaaaagaacgtcactgttacacacgcccaagacatactggaaaagacacacaacgggaaactctgcaatctagatggagtgaagcctctaattttaagagattgtagtgtagctggatggctcctcgggaacccaatgtgcgacgaattcctcaatgtgccggaatggtcttacatagtggagaagatcaatccatccaatgacctctgttatccagggaatttcaacgactatgaagaactgaaacacctattgagcagaataaaccattttgagaaaattcagatcattcccaaaaattcttggtcagatcatgaagccGGGtcaggagtgagctcagcatgtccataccagggaagatcctccttttttagaaatgtggtatggcttaccaaaaagaacaatgcatacccaacaataaagaaaagttacaataataccaaccaagaagatcttttggtactatgggggattcaccatccaaatgatgaggcagagcagacaagtctttatcaaaacccaactacctatatttccgttgggacatcaacactaaaccagagattggtaccaaaaatagctactagatctaaggtaaatgggcaaagtggaaggatggagttcttttggacaattttaaaatcgaatgatgcaataaactttgagagtaatggaaatttcattgctccagaaaatgcatacaaaattgtcaagaaaggggactcaacaattatgaaaagtgaattggaatatggcaactgcaacaccaagtgtcagactccaataggggcgataaactccagtatgccattccacaacatccaccctctcaccatcggggaatgccccaaatatgtgaaatcaaacagattagtccttgctactgggctcagaaatagccctcaaggagagagaagaagaaaaaagaga

>H5N1_A/Egypt/N02563/2009

taccatgcaaacaactcgacagagcaggttgacacaataatggaaaagaacgtcactgttacacacgcccaagacatactggaaaagacacacaacgggaaactctgcaatctagatggagtgaagcctctaattttaagagattgtagtgtagccggatggctcctcgggaacccaatgtgcgacgaattcctcaatgtgccggaatggtcttacatagtggagaagatcaatccagccaatgacctctgttatccagggaatttcaacgactatgaagaactgaaacacctattgagcagaataaaccattttgagaaaattcagatcatccccaaaaattcttggtcagatcatgaagccGGGtcaggagtgagctcagcatgtccataccagggaagatcctccttttttagaaatgtggtatggcttaccaaaaagaacaatgcatacccaacaataaagagaagttacaataataccaaccaagaagatcttttggtactatgggggattcaccatccaaatgatgcggaagagcagacaaggctttatcaaaacccaactacctatatttccgttgggacatcaacactaaaccagagattggtaccaaaaatagctactagatctaaggtaaacgggcaaagtgggaggatggagttcttttggacaattttaaaatcgaatgatgcaataaactttgagagtaatggaaatttcattgctccagaaaatgcatacaaaattgtcaagaaaggggactcaacaattatgaaaagtgagttggaatatggtaactgcaacaccaagtgtcagactccaataggggcgataaactccagtatgccattccacaacatccaccctctcaccatcggggaatgccccaaatatgtgaaatcaaacagattagtccttgctactgggctcagaaatagccctcaaggagagagaagaagaaaaaagaga

>H5N1_A/Egypt/N02752/2009

taccatgcaaacaactcgacagagcaggttgacacaataatggaaaagaacgtcactgttacacacgcccaagacatactggaaaagacacacaacggaaaactctgcaatctagatggagtgaagcctctaattttaagagattgtagtgtagctggatggctcctcgggaacccaatgtgcgacgaattcctcaatgtgccggaatggtcttacatagtggagaagatcaatccagccaatgacctctgttatccagggaatttcaacgactatgaagaactgaaacacctattgagcagaataaaccattttgagaaaatccagatcatccccaaaaattcttggtcagatcatgaagccGGGtcaggagtgagctcagcatgtccataccagggaagatcctccttttttagaaatgtggtatggcttaccaaaaaggacaatgcatacccaacaataaagagaagttacaataataccaaccaagaagatcttttggtactatgggggattcaccatccaaatgatgcggcagagcagacaaggctttatcaaaacccaactacctatatttccgttgggacatcaacactaaaccagagattgataccaaaaatagctactagatctaaggtaaacgggcaaagtggaaggatggagttcttttggacaattttaaaaccgaatgatgcaataaattttgagagtaatggaaatttcattgctccagaaaatgcatacaaaattgtcaagaaaggggactcaacaattatgaaaagtgagttggaatatggtaactgcaacaccaagtgtcagactccaataggggcgataaactccagtatgccattccacaacatccaccctctcaccatcggggaatgccccaaatatgtgaaatcaaacagattagtccttgctactgggctaagaaatagccctcaaggagagagaagaagaaaaaagaga

>H5N1_A/Egypt/N02770/2010

taccatgcaaacaactcgacagagcaggttgacacaataatggaaaagaacgtcactgttacacacgcccaagacatactggaaaagacacacaacgggaaactctgcaatctagatggagtgaagcctctaattttaagagattgtagtgtagctggatggctcctagggaacccaatgtgcgacgaattcctcaatgtgccggaatggtcttacatagtggagaagatcaatccaaccaatgacctctgttatccagggaatttcaacgactatgaagaactgaaacacctattgagcagaataaaccattttgagaaaattcagatcattcccaaaaattcttggtcagatcatgaagccGGGtcaggagtgagctcagcatgtccataccagggaagatcctccttttttagaaatgtggtatggcttaccaaaaagaacaatgcatacccaacaataaagaaaagttacaataataccaaccaagaagatcttttggtattatgggggattcaccatccaaatgatgcggcagagcagacaaggctttatcaaaacccaactacctatatttccgttgggacatcaacactaaaccagagattggtacccaaaatagctactagatctaaggtaaacgggcaaagtggaaggatggagttcttttggacaattttaaaatcgaatgatgcaataaactttgagagtaatggaaatttcattgctccagaaaatgcatacaaaattgtcaagaaaggggactcaacaattatgaaaagtgagttggaatatggtaactgcaacaccaagtgtcagactccaataggggcgataaactccagtatgccattccacaacatccaccctctcaccatcggggaatgccccaaatatgtgaaatcaaacagattagtccttgctactgggctcagaaatagccctcaaggagagagaagaagaaaaaagaga

>H5N1_A/Egypt/N03071/2010

taccatgcaaacaactcgacagagcaagttgacacaataatggaaaagaacgtcactgttacacacgcccaagacatactggaaaagacacacaacgggaaactctgcaatctagatggagtgaagcctctaattttaagagattgtagtgtagctggatggctcctcgggaacccaatgtgcgacgaattcctcaatgtgccggaatggtcttacatagtggagaagatcaatccatccaatgacctctgttatccagggaatttcaacgactatgaagaactgaaacacctattgagcagaataaaccattttgagaaaattcagatcattcccaaaaattcttggtcagatcatgaagccGGGtcaggagtgagctcagcatgtccataccagggaagatcctccttttttagaaatgtggtatggcttaccaaaaagaacaatgcatacccaacaataaagaaaagttacaataataccaaccaagaagatcttttggtactatgggggattcaccatccaaatgatgaggcagagcagacaaggctttatcaaaacccaactacctatatttccgttgggacatcaacactaaaccagagattggtaccaaaaatagctactagatctaaggtaaacgggcaaagtggaaggatggagttcttttggacaattttaaaatcgaatgatgcaataaactttgagagtaatggaaatttcattgctccagaaaatgcatacaaaattgtcaagaaaggggactcaacaattatgaaaagtgaattggaatatggcaactgcaacaccaagtgtcagactccaataggggcgataaactccagtatgccattccacaacatccaccctctcaccatcggggaatgccccaaatatgtgaaatcaaacagattagtccttgctactgggctcagaaatagccctcaaggagagagaagaagaaaaaagaga

>H5N1_A/Egypt/N03072/2010

taccatgcaaacaactcgacagagcaggttgacacaataatggaaaagaacgtcactgttacacacgcccaagacatactggaaaagacacacaacgggaaactctgcaatctagatggagtgaagcctctaattttaagagattgtagcgtagccggatggctcctcgggaacccaatgtgcgacgaattcctcaatgtgccggaatggtcttacatagtggagaagatcaatccagccaatgacctctgttatccagggaatttcaacgactatgaagaactgaaacacctattgagcagaataaaccattttgagaaaattcagatcatccccaaaagttcttggtcagatcatgaagccGGGtcaggagtgagctcagcatgtccataccagggaagatcctccttttttagaaatgtggtatggcttaccaaaaaggacaatgcatacccaacaataaagagaagttacaataataccaaccaagaagatcttttggtactatgggggattcaccatccaaatgatgcggcagagcagacaaggctttatcaaaacccaactacctatatttccgttgggacatcaacactaaaccagagattggtaccaaaaatagctactagatctaaggtaaacgggcaaagtggaaggatggagttcttttggacaattttaaaatcgaatgatgcaataaactttgagagtaatggaaatttcattgctccagaaaatgcatacaaaattgtcaagaaaggggactcaacaattatgaaaagtgagttggaatatggtaactgcaacaccaagtgtcagactccaataggggcgataaactccagtatgccattccacaacatccaccctctcaccatcggggaatgccccaaatatgtgaaatcaaacagattagtccttgctactgggctaagaaatagccctcaaggagagagaagaagaaaaaagaga

>H5N1_A/Egypt/N03228/2009

taccatgcaaacaactcgacagagcaggttgacacaataatggaaaagaacgtcactgttacacacgcccaagacatactggaaaagacacacaacgggaaactctgcaatctagatggagtgaagcctctaattttaagagattgtagtgtagccggatggctcctcgggaacccaatgtgcgacgaattcctcaatgtgccggaatggtcttacatagtggagaagatcaatccagccaatgacctctgttatccagggaatttcaacgactatgaagaactgaaacacctattgagcagaataaaccattttgagaaaattcagatcatccccaaaaattcttggtcagatcatgaagccGGGtcaggagtgagctcagcatgtccataccagggaaggtcctccttttttagaaatgtggtatggcttaccaaaaaggacaatgcatacccaacaataaagagaagttacaataatacaaaccaagaagatcttttggtactatgggggattcaccatccaaatgatgcggcagagcagacaaggctttatcaaaacccaactacctatatttccgttgggacatcaacactaaaccagagattggtaccaaaaatagctactagatctaaggtaaacggacaaaatggaaggatggagttcttttggacaattttaaaatcgaatgatgcaataaactttgagagtaatggaaatttcattgctccagaaaatgcatacaaaattgtcaagaaaggggactcaacaattatgaaaagtgagttggaatatggtaattgcaacaccaagtgtcagactccaataggggcgataaactccagtatgccattccacaacatccaccccctcaccatcggggaatgccccaaatatgtgaaatcaaacagattagtccttgctactgggctcagaaatagccctcaaggagagagaagaagaaaaaagaga

>H5N1_A/Egypt/N03272/2009

taccatgcaaacaactcgacagagcaggttgacacaataatggaaaagaacgtcactgttacacacgcccaagacatactggaaaagacacacaacgggaaactctgcaatttagatggagtgaagcctctaattttaagagattgtagtgtagccggatggctcctcgggaacccaatgtgcgacgaattcctcaatgtgccggaatggtcttacatagtggagaagatcaatccagccaatgacctctgttatccagggaatttcaacgactatgaagaactgaaacacctattgagcagaataaaccattttgagaaaattcagatcatccccaaaaattcttggtcagatcatgaagccGGGtcaggagtgagctcagcatgtccataccagggaaggtcctccttttttagaaatgtggtatggcttaccaaaaaggacaatgcatacccaacaataaagagaagttacaataatacaaaccaagaagatcttttggtactatgggggattcaccatccaaatgatgcggcagagcagacaaggctttatcaaaacccaactacctatatttccgttgggacatcaacactaaaccagagattggtaccaaaaatagctactagatctaaggtaaacggacaaagtggaaggatggagttcttttggacaattttaaaatcgaatgatgcaataaactttgagagtaatggaaatttcattgctccagaaaatgcatacaaaattgtcaagaaaggggactcaacaattatgaaaagtgagttggaatatggtaattgcaacaccaagtgtcagactccaataggggcgataaactccagtatgccattccacaacatccaccccctcaccatcggggaatgccccaaatatgtgaaatcaaacagattagtccttgctactgggctcagaaatagccctcaaggagagagaagaagaaaaaagaga

>H5N1_A/Egypt/N03434/2009

taccatgcaaacaactcgacagaacaggttgacacaataatggaaaagaacgtcactgttacacacgcccaagacatactggaaaagacacacaacgggaagctatgcgatctagatggagtgaagcctctaattttaagagattgtagtgtagctggatggctcctcgggaacccaatgtgtgacgaattcctcaatgtgccggaatggtcttacatagtggagaagatcaatccagccaatgacctctgttacccaggggatttcaacgactatgaagaactgaaacacctattgagcagaataaaccattttgagaaaattcagatcatccccaaaagttcttggtcagatcatgaagcatcgtcaggagtgagctcagcatgtccataccagggaagatcctccttttttagaaatgtggtatggcttatcaaaaaggacaatgcatacccaacaataaagagaagttacaataataccaaccaagaagatcttttggtactgtgggggattcaccatccgaatgatgcggcagagcagacaaggctctatcataacccaactacctatatttccgttgggacatcaacactaaaccagagattggtaccaaaaatagctactagatctaagataaatgggcaaagtggaaggatggagttcttttggacaattttaaaaccgaatgatgcaataaactttgagagtaatggaaatttcattgctccagaaaatgcatacaaaattgtcaagaaaggggactcaacaattatgaaaagtgagttggaatatggtaactgcaacaccaagtgtcaaactccaataggggcgataaactccagtatgccattccacaacatccaccctctcaccatcggggaatgccccaaatatgtgaaatcaagcagattagtccttgctactgggctcagaaatagtcctcaaggaaagagtagaagaaaaaagaga

>H5N1_A/Egypt/N03438/2009

taccatgcaaacaactcgacagagcaggttgacacaataatggaaaagaacgtcactgttacacacgcccaagacatactggaaaagacacacaacgggaaactctgcaatctagatggagtgaagcctctaattttaagagattgtagtgtagccggatggctcctcgggaacccaatgtgcgacgaattcctcaatgtgccggaatggtcttacatagtggagaagatcaatccagccaatgacctctgttatccagggaatttcaacgactatgaagaactgaaacacctattgagcagaataaaccattttgagaaaattcagatcatccccaaaaattcttggtcagatcatgaagccGGGtcaggagtgagctcagcatgtccataccagggaagatcctccttttttagaaatgtggtatggcttaccaaaaaggacaatgcatacccaacaataaagagaagttacaataataccaaccaagaagatcttttggtactatgggggattcaccatccaaatgatgcggcagagcagacaaggctttatcaaaacccaactacctatatttccgttgggacatcaacactaaaccagagattggtaccaaaaatagctactagatctaaggtaaacgggcaaagtggaaggatggagttcttttggacaattttaaaatcgaatgatgcaataaactttgagagtaatggaaatttcattgctccagaaaatgcatacaaaattgtcaagaaaggggactcaacaattatgaaaagtgagttggaatatggtaactgcaacaccaagtgtcagactccaataggggcgataaactccagtatgccattccacaacatccaccctctcaccatcggggaatgccccaaayatgtgaaatcaaacagattagtccttgctactgggctcagaaatagtcctcaaggagagagaagaagaaaaaagaga

>H5N1_A/Egypt/N03439/2009

taccatgcaaacaactcgacagagcaggttgacacaataatggaaaagaacgtcactgttacacacgcccaagacatactggaaaagacacacaacgggaaactctgcaatctagatggagtgaagcctctaattttaagagattgtagtgtagccggatggctcctcgggaacccaatgtgcgacgaattcctcaatgtgccggaatggtcttacatagtggagaagatcaatccagccaatgacctctgttatccagggaatttcaacgactatgaagaactgaaacacctattgagcagaataaaccattttgagaaaattcagatcatccccaaaaattcttggtcagatcatgaagccGGGtcaggagtgagctcagcatgtccataccagggaagatcctccttttttagaaatgtggtatggcttaccaaaaaggacaatgcatacccaacaataaagagaagttacaataataccaaccaagaagatcttttggtactatgggggattcaccatccaaatgatgcggcagagcagacaaggctttatcaaaacccaactacctatatttccgttgggacatcaacactaaaccagagattggtaccaaaaatagctactagatctaaggtaaacggacaaagtggaaggatggagttcttttggacaattttaaaatcgaatgatgcaataaactttgagagtaatggaaatttcattgctccagaaaatgcatacaaaattgtcaagaaaggggactcaacaattatgaaaagtgagttggaatatggtaactgcaacaccaagtgtcagactccaataggggcgataaactccagtatgccattccacaacatccaccctctcaccatcggggaatgccccaaatatgtgaaatcaaacagattagtccttgctactgggctcagaaatagccctcaaggagagagaagaagaaaaaagaga

>H5N1_A/Egypt/N03450/2009

taccatgcaaacaactcgacagagcaggttgacacaataatggaaaagaacgtcactgttacacacgcccaagacatactggaaaagacacacaacgggaaactctgcaatctagatggagtgaagcctctaattttaagagattgtagtgtagctggatggctcctcgggaacccaatgtgtgacgaattcctcaatgtgccggaatggtcttacatagtggagaagatcaatccagccaatgacctctgttatccagggaatttcaacgactatgaagaactgaaacacctattgagcagaataaaccattttgagaaaattcagatcatccccaaaaattcttggtcagatcatgaagccGGGtcaggagtgagctcagcatgtccataccagggaagatcctccttttttagaaatgtggtatggcttacaaaaaagaacaatgcatacccaacaataaagagaagttacaataataccaaccaagaagatcttttggtactatgggggattcaccatccaaatgatgcgacagagcagacaaggctttatcaaaacccaactacctatatttccgttgggacatcaacactaaaccagagattgataccaaaaatagctactagatctaaggtaaacgggcaaagtggaaggatggagttcttttggacaattttaaaatcgaatgatgcaataaactttgagagtaatggaaatttcattgctccagaaaatgcatacaaaattgtcaagaaaggggactcaacaattatgaaaagtgagttggaatatggtaactgcaacaccaagtgtcagactccaataggggcgataaactccagtatgccattccacaacatccaccccctcaccatcggggaatgccccaaatatgtgaaatcaaacagattagtccttgctaccgggctaagaaatagccctcaagaagggagaagaagaaaaaagaga

>H5N1_A/Egypt/N0423/2011

taccatgcaaacaactcgacagagcaggttgacacaataatggaaaagaatgtcactgttacacacgcccaagacatactggaaaagacacacaacgggaaactctgcaatctagatggagtgaagcctctaattttaagagattgtagcgtagccggatggctcctcgggaacccaatgtgcgacgaattcctcaatgtgccggaatggtcttacatagtggagaagatcaatccagccaatgacctctgttatccagggaatttcaacgactatgaagaactgaaacacctattgagcagaataaaccattttgagaaaattcagatcatccccaaaagttcttggtcagatcatgaagccGGGtcaggagtgagctcagcatgtccataccagggaagatcctccttttttagaaatgtggtatggcttaccaaaaaggacaatgcatacccaacaataaagagacgttacaataataccaaccaagaagatcttttggtactatgggggattcaccatccaaatgatgcggcagagcagacaaggctttatcaaaacccaactacctatatttccgttgggacatcaacactaaaccagagattggtaccaaaaatagctactagatctaaggtaaacgagcaaagtggaaggatggagttcttttggacaattttaaaatcgaatgatgcaataaactttgagagtaatggaaatttcattgctccagaaaatgcatacaaaattgtcaagaaaggggactcaacaattatgaaaagtgagttggaatatggtaactgcaacaccaagtgtcagactccaataggggcgataaactccagtatgccattccacaacatccaccctctcaccatcggggaatgccccaaatatgtaaaatcaaacagattagtccttgctactgggctaagaaatagccctcaaggagagaggagaagaaaaaagaga

>H5N1_A/Egypt/N04284/2011

taccatgcaaacaactcgacagagcaggttgacacaataatggaaaagaacgtcactgttacacacgcccaagacatactggaaaagacacacaacgggaaactctgcaatctagatggagtgaagcctctaattttaagagattgtagtgtagctggatggctcctcgggaacccaatgtgcgacgaattcctcaatgtgccggaatggtcttacatagtggagaagatcaatccagccaatgacctctgttatccagggaatttcaacgactatgaagaactgaaacacctattgagcagaataaaccattttgagaaaattcagatcattcccaaagattcttggtcagatcatgaagccGGGtcaggagtgagctcagcatgtccataccagggaaggtcctccttttttagaaatgtggtatggcttaccaaaaagaacgatgcatacccaacaataaagaaaagttacaataataccaaccaagaagatcttttggtactatgggggattcaccatccaaatgatgctgcagagcagacaaggctttatcaaaacccaactacctatatttccgttgggacatcaacactaaaccagagattggtacccaaaatagctactagatctaaggtaaacgggcaaagtggaaggatggagttcttttggacaattttaaaatcgaatgatgcaataaactttgagagtaatggaaacttcattgctccagaaaatgcatacaaaattgtcaagaaaggggactcaacaattatgaaaagtgagttggaatatagtaactgcaacaccaagtgtcagactccaataggggcgataaactccagtatgccattccacaacattcaccctctcaccatcggggaatgccccaaatatgtgaaatcaaacagattagtccttgctactgggctcagaaatagccctcaaggagagaaaagaagaaaaaagaga

>H5N1_A/Egypt/N04285/2011

taccatgcaaacaactcgacagagcaggttgacacaataatggaaaagaacgtcactgttacacacgcccaagacatactggaaaagacacacaacgggaaactctgcaacctagatggagtgaagcctctaattttaagagattgcagtgtagctggatggctcctcgggaacccaatgtgcgacgaattcctcaatgtgccggaatggtcctacatagtggagaagatcaatccagccaatgacctctgttatccagggaatttcaacgactatgaagaactgaaacacctattgagcagaataaaccattttgagaaaattcagatcattcccaaagattcttggtcagatcatgaagccGGGtcaggagtgagctcagcatgtccataccagggaagatcctccttttttagaaatgtggtatggcttaccaaaaagaacgatgcatacccaacaataaagaaaagttacaataataccaaccaagaagatcttttggtactatggggaattcaccatccaaatgatgctgcagagcagacaaggctttatcaaaacccaactacctatatttccgttgggacatcaacactaaaccagagattggtacccaaaatagctactagatctaaggtaaacgggcaaagtggaaggatggagttcttttggacaattttaaaatcgaatgatgcaataaactttgagagtaatggaaacttcattgctccagaaaatgcatacaaaattgtcaagaaaggggactcaacaattatgaaaagtgagttggaatatagtaactgcaacaccaagtgtcagactccaataggggcgataaactccagtatgccattccacaacatccaccctctcaccatcggggaatgccccaaatatgtgaaatcaaacagattagtccttgctactgggctcagaaatagccctcaaggagagaaaagaagaaaaaagaga

>H5N1_A/Egypt/N04286/2011

taccatgcaaacaactcgacagagcaggttgacacaataatggaaaagaacgtcactgttacacacgcccaagacatactggaaaagacacacaacgggaaactatgcaatctagatggagtgaagcctctaattttaagagattgtagtgtagctggatggctcctcgggaacccaatgtgcgacgaattcctcaatgtgccggaatggtcttacatagtggagaagatcaatccagccaatgacctctgttatccagggaatttcaacgactatgaagaactgaaacacctattgagcagaataaaccattttgagaaaattcagatcattcccaaagattcttggtcagatcatgaaaccGGGtcaggagtgagctcagcatgtccataccagggaagatcctccttttttagaaatgtggtatggcttaccaaaaagaacgatgcatacccaacaataaagaaaagttacaataataccaaccaagaagatcttttggtactatgggggattcaccatccaaatgatgctgcagagcagacaaggctttatcaaaacccaactacctatatttccgttgggacatcaacactaaaccagagattggtacccaaaatagctactagatctaaagtaaacgggcaaagtggaaggatggagttcttttggacaattttaaaatcgaatgatgcaataaactttgagagtaatggaaacttcattgctccagaaaatgcatacaaaattgtcaagaaaggggactcaacaattatgaaaagtgagttggaatatagtaactgcaacaccaagtgtcagactccaataggggcgataaactccagtatgccattccacaacatccaccctctcaccatcggggaatgccccaaatatgtgaaatcaaacagattagtccttgctactgggctcagaaatagccctcaaggagagaaaagaagaaaaaagaga

>H5N1_A/Egypt/N04287/2011

taccatgcaaacaactcgacagagcaggttgacacaataatggaaaagaacgtcactgttacacacgcccaagacatactggaaaagacacacaacgggaaactctgcaatctaggtggagtgaagcctctaattttaagagattgtagtgtagctggatggctcctcgggaacccaatgtgcgacgaattcctcaatgtgccggaatggtcttacatagtggagaagatcaatccagccaatgacctctgttatccagggaatttcaacgactatgaagaactgaaacacctattgagcagaataaaccattttgagaaaattcagatcattcccaaagattcttggtcagatcatgaagccGGGtcaggagtgagctcagcatgtccataccagggaagatcctccttttttagaaatgtgatatggcttaccaaaaagaacgatgcatacccaacaataaagaaaagttacaataataccaaccaagaagatcttttggtactatgggggattcaccatccaaatgatgctgcagagcagacaaggctttatcaaaacccaactacctatatttccgttgggacagcaacactaaaccagagattggtacccaaaatagctactagatctaaggtaaacgggcaaagtggaaggatggagttcttttggacaattttaaaatcgaatgatgcaataaactttgagagtaatggaaacttcattgctccagaaaatgcatacaaaattgtcaagaaaggggactcaacaattatgaaaagtgagttggaatatagtaactgcaacaccaagtgtcagactccaataggggcgataaactccagtatgccattccacaacatccaccctctcaccatcggggaatgccccaaatatgtgaaatcaaacagattagtccttgctactgggctcagaaatagccctcaaggagagaaaagaagaaaaaagaga

>H5N1_A/Egypt/N04288/2011

taccatgcaaacaactcgacagagcaggttgacacaataatggaaaagaacgtcactgttacacacgcccaagacatactggaaaagacacacaacgggaaactctgcaatctagatggagtgaagcctctaattttaagagattgtagtgtagctggatggctcctcgggaacccaatgtgcgacgaattcctcaatgtgccggaatggtcttacatagtggagaagatcaatccagccaatgacctctgttatccagggaatttcaacgactatgaagaactgaaacacctattgagcagaataaaccattttgagaaaattcagatcattcccaaagattcttggtcagatcatgaaaccGGGtcaggagtgagctcagcatgtccataccagggaagatcctccttttttagaaatgtggtatggcttaccaaaaagaacgatgcatacccaacaataaagaaaagttacaataataccaaccaagaagatcttttggtactatgggggattcaccatccaaatgatgctgcagagcagacaaggctttatcaaaacccaactacctatatttccgttgggacatcaacactaaaccagagattggtacccaaaatagctactagatctaaagtaaacgggcaaagtggaaggatggagttcttttggacaattttaaaatcgaatgatgcaataaactttgagagtaatggaaacttcattgctccagaaaatgcatacaaaattgtcaagaaaggggactcaacaattatgaaaagtgagttggaatatagtaactgcaacaccaagtgtcagactccaataggggcgataaactccagtatgccattccacaacatccaccctctcaccatcggggaatgccccaaatatgtgaaatcaaacagattagtccttgctactgggctcagaaatagccctcaaggagagaaaagaagaaaaaagaga

>H5N1_A/Egypt/N04316/2009

taccatgcaaacaactcgacagagcaggttgacacaataatggaaaagaacgtcactgttacacacgcccaagacatactggaaaagacacacaacgggaaactctgcaatctagatggagtgaagcctctaattttaagagattgtagtgtagccggatggctcctcgggaacccaatgtgcgacgaattcctcaatgtgccggaatggtcttacatagtggagaagatcaatccagccaatgacctctgttatccagggaatttcaacgactatgaagaactgaaacacctattgagcagaataaaccattttgagaaaattcagatcatccccaaaaattcttggtcagatcatgaagccGGGtcaggagtgagctcagcatgtccataccagggaagatcctccttttttagaaatgtggtatggcttaccaaaaagaacaatgcatacccaacaataaagagaagttacaataataccaaccaagaagatcttttggtactatgggggattcaccatccaaatgatgcggaagagcagacaaggctttatcaaaacccaactacctatatttccgttgggacatcaacactaaaccagagattggtaccaaaaatagctactagatctaaggtaaacgggcaaagtggaaggatggagttcttttggacaattttaaaatcgaatgatgcaataaactttgagagtaatggaaatttcattgctccagaaaatgcatacaaaattgtcaagaaaggggactcaacaattatgaaaagtgagttggaatatggtaactgcaacaccaagtgtcagactccaataggggcgataaactccagtatgccattccacaacatccaccctctcaccatcggggaatgccccaaatatgtgaaatcaaacagattagtccttgctactgggctcagaaatagccctcaaggagagagaagaagaaaaaagaga

>H5N1_A/Egypt/N04394/2009

taccatgcaaacaactcgacagagcaggttgacacaataatggaaaagaacgtcactgttacacacgcccaagacatactggaaaagacacacaacgggaaactctgcaatctagatggagtgaagcctctaattttaagagattgtagtgtagctggatggctcctcgggaacccaatgtgcgacgaattcctcaatgtgccggaatggtcttacatagtggagaagatcaatccagccaatgacctctgttatccagggagtttcaacgactatgaagaactgaaacacctattgagcagaataaaccattttgagaaaattcagatcatccccaagaattcttggtcagatcatgaagccGGGtcaggagtgagctcagcatgtccataccagggaagatcctccttttttagaaatgtggtatggcttaccaaaaaggacaatgcatacccaacaataaagagaagttacaataacaccaaccaagaagatcttttggtactatgggggattcaccatccaaatgatgcggcagagcaaacaaggctttatcaaaacccaactacctatatttccgttggaacatcaacactaaaccagagattggtaccaaaaattgctactagatctaaggtaaacgggcaaagtggaaggatggagttcttttggacaattttaaaaccgaatgatgcaataaactttgaaagtaatggaaatttcattgctccagaaaatgcatacaaaattgtcaagaaaggggactcaacaattatgaaaagtgagttggaatatggtaactgcaacaccaagtgtcagactccaataggggcgataaactccagtatgccattccacaacatccaccctctcaccatcggggaatgccccaaatatgtgaaatcaaacagattaatccttgctactgggctcagaaatagccctcaaggagagagaagaagaaaaaagaga

>H5N1_A/Egypt/N04395/2009

taccatgcaaacaactcgacagagcaggttgacacaataatggaaaagaacgtcactgttacacacgcccaagacatactggaaaagacacacaacgggaaactctgcaatctagatggagtgaagcctctaattttaagagattgtagtgtagccggatggctcctcgggaacccaatgtgcgacgaattcctcaatgtgccggaatggtcttacatagtggagaagatcaatccagccaatgacctctgttatccagggaatttcaacgactatgaagaactgaaacacctattgagcagaataaaccattttgagaaaattcagatcatccccaaaaattcttggtcagatcatgaagccGGGtcaggagtgagctcagcatgtccataccagggaagatcctccttttttagaaatgtggtatggcttaccaaaaaggacaatgcatacccaacaataaagagaagttacaataataccaaccaagaagatcttttggtactatgggggattcaccatccaaatgatgcggcagagcagacaaggctttatcaaaacccaactacctatatttccgttgggacatcaacactaaaccagagattggtaccaaaaatagctactagatctaaggtaaacggacaaagtggaaggatggagttcttttggacaattttaaaatcgaatgatgcaataaactttgagagtaatggaaatttcattgctccagaaaatgcatacaaaattgtcaagaaaggggactcaacaattatgaaaagtgagttggaatatggtaactgcaacaccaagtgtcagactccaataggggcgataaactccagtatgccattccacaacatccaccctctcaccatcggggaatgccccaaatatgtgaaatcaaacagattagtccttgctactgggctcagaaatagccctcaaggagagagaagaagaaaaaagaga

>H5N1_A/Egypt/N04396/2009

taccatgcaaacaactcgacagagcaggttgacacaataatggaaaagaacgtcactgttacacacgcccaagacatactggaaaagacacacaacgggaaactctgcaatctagatggagtgaagcctctaattttaagagattgtagtgtagccggatggctcctcgggaacccaatgtgcgacgaattcctcaatgtgccggaatggtcttacatagtggagaagatcaatccagccaatgacctctgttatccagggaatttcaacgactatgaagaactgaaacacctattgagcagaataaaccattttgagaaaattcagatcatccccaaaaattcttggtcagatcatgaagccGGGtcaggagtgagctcatcatgtccataccagggaagatcctccttttttagaaatgtggtatggcttaccaaaaaggacaatgcatacccaacaataaagagaagttacaataataccaaccaagaagatcttttggtactatgggggattcaccatccaaatgatgcggcagagcagacaaggctttatcaaaacccaactacctatatttccgttgggacatcaacactaaaccaaagattggtaccaaaaatagctactagatctaaggtaaacggacaaagtggaaggatggagttcttttggacaattttaaaatcgaatgatgcaataaactttgggagtaatggaaatttcattgctccagaaaatgcatacaaaattgtcaagaaaggggactcaacaattatgaaaagtgagttggaatatggtaactgcaacaccaagtgtcagactccaataggggcgataaactccagtatgccattccacaacatccaccctctcaccatcggggaatgccccaaatatgtgaaatcaaacagattagtccttgctactgggctcagaaatagccctcaaggagagagaagaagaaaaaagaga

>H5N1_A/Egypt/N04434/2010

taccatgcaaacaactcgacagagcaggttgacacaataatggaaaagaacgttactgttacacacgcccaagacatactggaaaggacacacaacgggaaactctgcaatctagatggagtgaagcctctaattttaagagattgtagcgtagccggatggctcctcgggaacccaatgtgcgacgaattcctcaatgtgccggaatggtcttacatagtggagaagatcaatccagccaatgacctctgttatccagggaatttcaacgactatgaagaactgaaacacctattgagcagaataaaccattttgagaaaattcagatcatccccaaaagttcttggtcagatcatgaagccGGGtcaggagtgagctcagcatgtccataccagggaagatcctccttttttagaaatgtggtatggcttaccaaaaaggacaatgcatacccaacaataaagagaagttacaataataccaaccaagaagatcttttggtactatgggggattcaccatccaaatgatgcggcagagcagacaaggctttatcaaaacccaactacctatatttccgttgggacatcaacactaaaccagagattggtaccaaaaatagctactagatctaaggtaaacgggcaaagtggaaggatggagttcttttggacaattttaaaatcgaatgatgcaataaactttgagagtaatggaaatttcattgctccagaaaatgcatacaaaattgtcaagaaaggggactcaacaattatgaaaagtgagttggaatatggtaactgcaacaccaagtgtcagactccaataggggcgataaactccagtatgccattccacaacatccaccctctcaccatcggggaatgccccaaatatgtgaaatcaaacagattagtccttgctactgggctaagaaatagccctcaaggagagagaagaagaaaaaagaga

>H5N1_A/Egypt/N04526/2009

taccatgcaaacaactcgacagaacaggttgacacaataatggaaaagaacgtcactgttacacacgcccaagacatactggaaaagacacacaacgggaagctatgtgatctagatggagtgaagcctctaattttaagagattgtagtgtagctggatggctcctcgggaacccaatgtgtgacgaattcctcaatgtgccggaatggtcttacatagtggagaagatcaatccagccaatgacctctgttacccaggggatttcaacgactatgaagaactgaaacacctattgagcagaataaaccattttgagaaaattcagatcatccccaaaagttcttggtcagatcatgaagcatcgtcaggagtgagctcagcatgtccataccagggaagatcctccttttttagaaatgtggtatggcttatcaaaaaggacaatgcatacccaacaataaagagaagttacaataataccaaccaagaagatcttttggtactgtgggggattcaccatccgaatgatgcggcagagcagacaaggctctatcataacccaactacctatatttccgttgggacatcaacactaaaccagagattggtaccaaaaatagctactagatctaaggtaaatgggcaaagtggaaggatggagttcttttggacaattttaaaaccgaatgatgcaataaactttgagagtaatggaaatttcattgctccagaaaatgcatacaaaattgtcaagaaaggggactcaacaattatgaaaagtgagttggaatatggtaactgcaacaccaagtgtcaaactccaataggggcgataaactccagtatgccattccacaacatccaccctctcaccatcggggaatgccccaaatatgtgaaatcaagcagattagtccttgctactgggctcagaaatagtcctcaaggaaagaatagaagaaaaaagaga

>H5N1_A/Egypt/N04527/2009

taccatgcaaacaactcgacagagcaggttgacacaataatggaaaagaacgtcactgttacacacgcccaagacatactggaaaagacacacaacgggaaactatgcaatctagatggagtgaagcctctaattttaagagattgtagtgtagctggatggctcctcgggaacccaatgtgtgacgaattcctcaatgtgccggaatggtcttacatagtggagaagatcaatccagccaatgacctctgttatccagggaatttcaacgactatgaagaactgaaacacctattgagcagaataaaccattttgagaaaattcagatcatccccaaaaattcttggtcagatcatgaagccGGGtcaggagtgagctcagcatgtccataccagggaagatcctccttttttagaaatgtggtatggcttacaaaaaaggacaatgcatacccaacaataaagagaagttacaataataccaaccaagaagatcttttggtactatgggggattcaccatccaaatgatgcgacagagcagacaaggctttatcaaaacccaactacctatatttccgttgggacatcaacactaaaccagagattgataccaaaaatagctactagatctaaggtaaacgggcaaagtggaaggatggagttcttttggacaattttaaaatcgaatgatgcaataaactttgagagtaatggaaatttcattgctccagaaaatgcatacaaaattgtcaagaaaggggactcaacaattatgaaaagtgagttggaatatggtaactgcaacaccaagtgtcagactccaataggggcgataaactccagtatgccattccacaacatccaccccctcaccatcggggaatgccccaaatatgtgaaatcaaacagattagtccttgctactgggctaagaaatagccctcaagaagagagaagaagaaaaaagaga

>H5N1_A/Egypt/N04822/2009

taccatgcaaacaactcgacagaacaggttgacacaataatggaaaagaacgtcactgttacacacgcccaagacatactggaaaagacacacaacgggaagctatgcgatctagatggagtgaagcctctaattttaagagattgtagtgtagctggatggctcctcgggaatccaatgtgtgacgaattcctcaatgtgccggaatggtcttacatagtggagaagatcaatccagccaatgacctctgttacccaggggatttcaacgactatgaagaactgaaacacctattgagcagaataaaccattttgagaaaattcagatcatccccaaaagttcttggtcagatcatgaagcatcgtcaggagtgagctcagcatgtccataccagggaggatcctccttttttagaaatgtggtatggcttatcaaaaaggacaatgcatacccaacaataaagagaagttacaataataccaaccaagaagatcttttggtactgtgggggattcaccatccgaatgatgcggcggagcagacaaggctctatcataacccaactacctatatttccgttgggacatcaacactaaaccagagattggtaccaaaaatagctactagatctaaggtaaatgggcaaagtggaaggatggagttcttttggacaattttaaaaccgaatgatgcaataaactttgagagtaatggaaatttcattgctccagaaaatgcatataaaattgtcaagaaaggggactcaacaattatgaaaagtgagttggaatatggtaactgcaacaccaagtgtcaaactccaataggggcgataaactccagtatgccattccacaacatccaccctctcaccatcggggaatgccccaaatatgtgaaatcaagcagattagtccttgctactgggctcagaaatagtcctcaaggaaagagtagaagaaaaaagaga

>H5N1_A/Egypt/N04823/2009

taccatgcaaacaactcgacagagcaggttgacacaataatggaaaagaacgtcactgttacacacgcccaagacatactggaaaagacacacaacgggaaactctgcaatctagatggagtgaagcctctaattttaagagattgtagtgtagccggatggctcctcgggaacccaatgtgcgacgaattcctcaatgtgccggaatggtcttacatagtggagaagatcaatccagccaatgacctctgttatccagggaatttcaacgactatgaagaactgaaacacctattgagcagaataaaccattttgagaaaattcagatcatccccaaaaattcttggtcagatcatgaagccGGGtcaggagtgagctcagcatgtccataccagggaagatcctccttttttagaaatgtggtatggcttaccaaaaagaacagtgcatacccaacaataaagagaagttacaataataccaaccaagaagatcttttggtactatgggggattcaccatccaaatgatgcggcagagcagacaaggctttatcaaaacccaactacctatatttccgttgggacatcaacactaaaccagagattggtaccaaaaatagctactaggtctaaggtaaacgggcaaagtggaaggatggagttcttttggacaattttaaaatcgaatgatgcaataaactttgagagtaatggaaatttcattgctccagaaaatgcatacaaaattgtcaagaaaggggactcaacaattatgaaaagtgagttggaatatagtaactgcaacaccaagtgtcagactccaataggggcgataaactccagtatgccattccacaacatccaccctctcaccatcggggaatgccccaaatatgtgaaatcaaacagattagtccttgctactgggctcagaaatagccctcaaggagagagaagaagaaaaaagaga

>H5N1_A/Egypt/N04830/2009

taccatgcaaacaactcgacagagcaggttgacacaataatggaaaagaacgtcactgttacacacgcccaagacatactggaaaagacacacaacgggaaactttgcaatctagatggaatgaagcctctaattttaagagattgtagtgtagccggatggctcctcgggaacccaatgtgcgacgaattcctcaatgtgccagaatggtcttacatagtggagaagattaatccagccaatgacctctgttatccagggaatttcaacgactatgaagaactgaaacacctattgagcagaataaaccattttgagaaaattcagatcatccccaaaaattcttggtcagatcatgaagccGGGtcaggagtgagctcagcatgtccataccagggaagatcctccttttttagaaatgtggtatggcttaccaaaaaggacaatgcatacccaacaataaagagaagttacaataataccaaccaagaagatcttttggtactatggggaattcaccatccaaatgatgcggcagagcagacaaggctttatcaaaacccaactacctatatttccgttgggacatcaacactaaaccagagattggtaccaaaaatagctactagatctaaggtaaacgggcaaagtggaaggatggagttcttttggacaattttaaaatcgaatgatgcaataaactttgagagtaatggaaatttcattgctccagaaaatgcatacaaaattgtcaagaaaggggactcaacaattatgaaaagtgagttggaatatagtaactgcaacaccaaatgtcagactccaataggggcgataaactccagtatgccattccacaacatccaccctctcaccatcggggaatgccccaaatatgtgaaatcaaacagattagtccttgctactgggctcagaaatagccctcaaggagagagaagaagaaaaaagaga

>H5N1_A/Egypt/N04915/2014

taccatgcaaacaactcgacagagcaggttgacacaataatggaaaagaatgtcactgttacacacgcccaagacatactggaaaagacacacaacgggaaactctgcaatctagatggagtgaagcctctcattttgagagattgtagtgtagctggatggctcctcgggaacccaatgtgcgatgaattcctcaatgtgccggaatggtcttacatagtggagaaaatcaatccagccaatgacctctgttatccagggaatttcaacgactatgaagaactgaaacacctattgagcagaataaaccattttgagaaaattcagatcattcccaaagattcttggtcagatcatgaagccGGGtcgggagtgagctcagcatgcccataccaaggaagatcctccttttttagaaatgttgtatggcttaccaaaaagaacgatgcatacccaacaataaagaaaagttacaataatactaaccaagaagatcttttggtactatgggggattcaccatccaaatgatgctgcagagcagacaaggctttatcaaaacccaactacctatatctccgttgggacatcaacactaaaccagagattggtacccaaaatagctactagatctaaggtaaacgggcaaagtggaaggatggagttcttttggacaattttaaaatcgaatgatgcaataaactttgagagcaatggaaacttcattgctccagaaaatgcatacaaaattgtcaagaaaggagattcaacaattatgaaaagtgagttggaatatagtaactgcaacaccaagtgtcagactccaataggggcgataaactccagtatgccattccacaacatccaccctctcaccatcggggaatgccccaaatatgtgaaatcaaacagattagtccttgctactgggctcaggaatagccctcaaggagagaaaagaagaaaaaagaga

>H5N1_A/Egypt/N04915/2014__NIBRG-306

taccatgcaaacaactcgacagagcaggttgacacaataatggaaaagaatgtcactgttacacacgcccaagacatactggaaaagacacacaacgggaaactctgcaatctagatggagtgaagcctctcattttgagagattgtagtgtagctggatggctcctcgggaacccaatgtgcgatgaattcctcaatgtgccggaatggtcttacatagtggagaaaatcaatccagccaatgacctctgttatccagggaatttcaacgactatgaagaactgaaacacctattgagcagaataaaccattttgagaaaattcagatcattcccaaagattcttggtcagatcatgaagccGGGtcgggagtgagctcagcatgcccataccaaggaagatcctccttttttagaaatgttgtatggcttaccaaaaagaacgatgcatacccaacaataaagaaaagttacaataatactaaccaagaagatcttttggtactatgggggattcaccatccaaatgatgctgcagagcagacaaggctttatcaaaacccaactacctatatctccgttgggacatcaacactaaaccagagattggtacccaaaatagctactagatctaaggtaaacgggcaaagtggaaggatggagttcttttggacaattttaaaatcgaatgatgcaataaactttgagagcaatggaaacttcattgctccagaaaatgcatacaaaattgtcaagaaaggagattcaacaattatgaaaagtgagttggaatatagtaactgcaacaccaagtgtcagactccaataggggcgataaactccagtatgccattccacaacatccaccctctcaccatcggggaatgccccaaatatgtgaaatcaaacagattagtccttgctactgggctcaggaatagccctcaacgagagagtagaagaaaaaagcga

>H5N1_A/Egypt/N04979/2009

taccatgcaaacaactcgacagaacaggttgacacaataatggaaaagaacgtcactgttacacacgcccaagacatactggaaaagacacacaacgggaagctatgcgatctagatggagtgaagcctctaattttaagagattgtagtgtagctggatggctcctcgggaacccaatgtgtgacgaattcctcaatgtgccggaatggtcttacatagtggagaagatcaatccagccaatgacctctgttacccaggggatttcaacgactatgaagaactgaaacacctattgagcagaataaaccattttgagaaaattcagatcatccccaaaagttcttggtcagatcatgaagcatcgtcaggagtgagctcagcatgtccataccagggaagatcctccttttttagaaatgtggtatggcttatcaaaaaggacaatgcatacccaacaataaagagaagttacaataataccaaccaagaagatcttttggtactgtgggggattcaccatccgaatgatgcggcagagcagacaaggctctatcataacccaactacctatatttccgttgggacatcaacactaaaccagagattggtaccaaaaatagctactagatctaaggtaaatgggcaaagtggaaggatggagttcttttggacaattttaaaaccgaatgatgcaataaactttgagagtaatggaaatttcattgctccagaaaatgcatacaaaattgttaagaaaggggactcaacaattatgaaaagtgagttggaatatggtaactgcaacaccaagtgtcaaactccaataggggcgataaactccagtatgccattccacaacatccaccctctcaccatcggggaatgccccaaatatgtgaaatcaagcagactagtccttgctactgggctcagaaatagtcctcaaggaaagagtagaagaaaaaagaga

>H5N1_A/Egypt/N05056/2009

taccatgcaaacaactcgacagagcaggttgacacaataatggaaagaaacgtcactgttacacacgcccaggacatactggaaaagacacacaacgggaaactctgcaatctagatggagtgaagcctctaattttaagagattgtagtgtagctggatggctcctcgggaacccaatgtgtgacgaattcctcaatgtgccggaatggtcttacatagtggagaagatcaatccagccaatgacctctgttatccagggaatttcaacgactatgaagaactgaaacacctattgagcagaataaaccattttgagaaaattcagatcatccccaaaaattcttggtcagatcatgaagccGGGtcaggagtgagctcagcatgtccataccagggaagatcctccttttttagaaatgtggtatggcttacccaaaaggacaatgcatacccaacaataaagagaagttacaataataccaaccaagaagatcttttggtactatgggggattcaccatccaaatgatgcggcagagcagacaaggctttatcaaaacccaactacctatatttccgttgggacatcaacactaaaccagagattgataccaaaaatagctactagatccaaggtaaacgggcaaagtggaaggatggagttcttttggacaattttaaaatcgaatgatgcaataaactttgagagtaatggaaatttcattgctccagaaaatgcatacaaaattgtcaagaaaggggactcaacaattatgaaaagtgagttggaatatggtaactgcaacaccaagtgtcagactccaataggggcgataaactccagtatgccattccacaacatccaccctctcaccatcggggaatgtcccaaatatgtgaaatcaaacagattagtccttgctactgggctaagaaatagccctcaaggagagagaagaagaaaaaagaga

>H5N1_A/Egypt/N0544/2011

taccatgcaaacaactcgacagagcaggttgacacaataatggaaaagaacgtcactgttacacacgcccaagacatactggaaaagacacacaacgggaaactctgcaatctagatggagtgaagcctctaattttaagagattgtagtgtagctggatggctcctcgggaacccaatgtgcgacgaattcctcaatgtgccggaatggtcttacatagtggagaagatcaatccagccaatgacctctgttatccagggaatttcaacgactatgaagaactgaaacacctattgagcagaataaaccattttgagaaaattcagatcattcccaaagattcttggtcagatcatgaagccGGGtcaggagtgagctcagcatgtccataccagggaaggtcctccttttttagaaatgtggtatggcttaccaaaaagaacgatgcatacccaacaataaagaaaagttacaataataccaaccaagaagatcttttggtactatgggggattcaccatccaaatgatgctgcagagcagacaaggctttatcaaaacccaactacctatatttccgttgggacatcaacactaaaccagagattggtacccaaaatagctactagatctaaggtaaacgggcaaagtggaaggatggagttcttttggacaattttaaaatcgaatgatgcaataaactttgagagtaatggaaacttcattgctccagaaaatgcatacaaaattgtcaagaaaggggactcaacaattatgaaaagtgagttggaatatagtaactgcaacaccaagtgtcagactccaataggggcgataaactccagtatgccattccacaacattcaccctctcaccatcggggaatgccccaaatatgtgaaatcaaacagattagtccttgctactgggctcagaaatagccctcaaggagagaaaagaagaaaaaagaga

>H5N1_A/Egypt/N05860/2011

taccatgcaaacaactcgacagagcaggttgacacaataatggaaaagaacgtcactgttacacacgcccaagacatactggaaaagacacacaacgggaaactctgcaatctagatggagtgaagcctctaattttaagagattgtagtgtagctggatggctcctcgggaacccaatgtgcgacgaattcctcaatgtgccggaatggtcttacatagtggagaagatcaatccagccaatgacctctgttatccagggaatttcaacgactatgaagaactgaaacacctattgagcagaataaaccattttgagaaaattcagatcattcccaaagattcttggtcagatcatgaagccGGGtcaggagtgagctcagcatgtccataccagggaagatcctccttttttagaaatgtggtatggcttaccaaaaagaacgatgcatacccaacaataaagaaaagttacaataataccaaccaagaagatcttttggtactatgggggattcaccatccaaatgatgctgcagagcagacaaggctttatcaaaacccaactacctatatttccgttgggacatcaacactaaaccagagattggtacccaaaatagctactagatctaaggtaaacgggcaaagtggaaggatggagttctyttggacaattttaaaatcgaatgatgcaataaactttgagagtaatggaaacttcattgctccagaaaatgcatacaaaattgtcaagaaaggggactcaacaattatgaaaagtgagttggaatatagtaactgcaacaccaagtgtcagactccaataggggcgataaactccagtatgccattccacaacatccaccctctcaccatcggggaatgccccaaatatgtgaaatcaaacagattagtccttgctactggactcagaaatagccctcaaggagagaaaagaagaaaaaagaga

>H5N1_A/Egypt/N05912/2009

taccatgcaaacaactcgacagagcatgttgacacaataatggaaaagaatgtcactgttacacacgcccaagacatactggaaaagacacacaacgggaaactctgcaatctagatggagtgaagcctctaattttaagagattgtagtgtagctggatggctcctcgggaacccaatgtgcgacgaattcctcaatgtgccggaatggtcttacatagtggagaagatcaatccagccaatgacctctgttatccagggaatttcaacgactatgaagaactgaaacacctattgagcagaataaaccattttgagaaaattcagatcattcccaaaaattcttggtcagatcatgaagccGGGtcaggagtgagctcagcatgtccataccagggaagatcctccttttttagaaatgtggtatggcttaccaaaaagaacaatgcatacccaacaataaagaaaagttacaataataccaaccaagaagatcttttggtactatgggggattcaccatccaaatgatggggcagagcagacaaggctttatcaaaacccaactacctatatttccgttgggacatcaacactaaaccagagattggtaccaaaaatagctactagatctaaggtaaacgggcaaagtggaaggatggagttcttttggacaattttaaaatcgaatgatgcaataaactttgagagtaacggaaatttcattgctccagaaaatgcatacaaaattgtcaagaaaggggactcaacaattatgaaaagtgagttggaatatggtaactgcaacaccaagtgtcagactccaataggggcgataaactccagtatgccattccacaacatccaccctctcaccatcggggaatgccccaaatatgtgaaatcaaacagattagtccttgctactgggctcagaaatagccctcaaggagagagaagaagaaaaaagaga

>H5N1_A/Egypt/N0677/2011

taccatgcaaacaactcgacagagcaggttgacacaataatggaaaagaacgtcactgttacacacgcccaagacatactggaaaagacacacaacgggaaactctgcaatctagatggagtgaagcctctaattttaagagattgtagtgtagctggatggctcctcgggaacccaatgtgcgacgaattccgcaatgtgccggaatggtcttacatagtggaaaagatcaatccagccaatgacctctgttatccagggaatttcaacgactatgaagaactgaaacacctattgagcagaataaaccattttgagaaaattcagatcattcccaaagattcttggtcagatcatgaagccGGGtcaggagtgagctcagcatgtccataccagggaagatcctccttttttagaaatgtggtatggcttaccaaaaagaacgatgcatacccaacaataaagaaaagttacaataataccaaccaagaagatcttttggtactatgggggattcaccatccaaatgatgctgcagagcagacaaggctttatcaaaacccaactacctatatttccgttgggacatcaacactaaaccagagattggtacccaaaatagctactagatctaaggtaaacgggcaaagtggaaggatggagttcttttggacaattttaaaatcgaatgatgcaataaactttgagagtaatggaaacttcattgctccagaaaatgcatacaaaattgtcaagaaaggggactcaacaattatgaaaagtgagttggaatatagtaactgcaacaccaagtgtcagactccaataggggcgataaactccagtatgccattccacaacatccaccctctcaccatcggggaatgccccaaatatgtgaaatcaaacagattagtccttgctactgggctcagaaatagccctcaaggagagaaaagaagaaaaaagaga

>H5N1_A/Egypt/N07392/2009

taccatgcaaacaactcaacagagcaggttgacacaataatggaaaagaacgtcactgttacacacgcccaagacatactggaaaagacacacaacgggaaactctgcaatctagatggagtgaagcctctaattttaagagattgtagtgtagctggatggctcctcgggaacccaatgtgcgacgaattcctcaatgtgccggaatggtcttacatagtggagaagatcaatccagccaatgacctctgttatccagggaatttcaacgactatgaagaactgaaacacctattgagcagaataaaccattttgagaaaattcagatcattcccaaaaattcttggtcagatcatgaagccGGGtcaggagtgagctcagcatgtccataccagggaagatcctccttttttagaaatgtggtatggcttaccaaaaagaacaatgcatacccaacaataaagaaaagttacaataataccaaccaagaagatcttttggtactatgggggattcaccatccaaatgatgcggcagagcagacaaggctttatcaaaacccaactacctatatttccgttgggacatcaacactaaaccagagattggtaccaaaaatagctactagatctaaggtaaacgggcaaagtggaaggatggagttcttttggacaattttaaaatcgaatgatgcaataaactttgagagtaatggaaatttcattgctccagaaaatgcatacaaaattgtcaagaaaggggactcaacaattatgaaaagtgagttggaatatggtaactgcaacaccaagtgtcagactccaataggggcgataaactccagtatgccattccacaacatccaccctctcaccatcggggaatgccccaaatatgtgaaatcaaacagattagtccttgctactgggctcagaaatagccctctaggagagagaagaagaaaaaagaga

>H5N1_A/Egypt/N07908/2009

taccatgcaaacaactcgacagagcatgttgacacaataatggaaaagaatgtcactgttacacacgcccaagacatactggaaaagacacacaacgggaaactctgcaatctagatggagtgaagcctctaattttaagagattgtagtgtagctggatggctcctcgggaacccaatgtgcgacgaattcctcaatgtgccggaatggtcttacatagtggagaagatcaatccagccaatgacctctgttatccagggaatttcaacgactatgaagaactgaaacacctattgagcagaataaaccattttgagaaaattcagatcattcccaaaaattcttggtcagatcatgaagccGGGtcaggagtgagctcagcatgtccataccagggaagatcctccttttttagaaatgtggtatggcttaccaaaaagaacaatgcatacccaacaataaagaaaagttacaataataccaaccaagaagatcttttggtactatgggggattcaccatccaaatgatggggcagagcagacaaggctttatcaaaacccaactacctatatttccgttgggacatcaacactaaaccagagattggtaccaaaaatagctactagatctaaggtaaacgggcaaagtggaaggatggagttcttttggacaattttaaaatcgaatgatgcaataaactttgagagtaacggaaatttcattgctccagaaaatgcatacaaaattgtcaagaaaggggactcaacaattatgaaaagtgagttggaatatggtaactgcaacaccaagtgtcagactccaataggggcgataaactccagtatgccattccacaacatccaccctctcaccatcggggaatgccccaaatatgtgaaatcaaacagattagtccttgctactgggctcagaaatagccctcaaggagagagaagaagaaaaaagaga

>H5N1_A/Egypt/N08835/2009

taccatgcaaacaactcaacagagcaggttgacacaataatggaaaagaacgtcactgttacacacgcccaagacatactggaaaagacacacaacgggaaactctgcaatctagatggagtgaagcctctaattttaagagattgtagtgtagctggatggctcctcgggaacccaatgtgcgacgaattcctcaatgtgccggaatggtcttacatagtggagaagatcaatccagccaatgacctctgttatccagggaatttcaacgactatgaagaactgaaacacctattgagcagaataaaccattttgagaaaattcagatcattcccaaaaattcttggtcagatcatgaagccGGGtcaggagtgagctcagcatgtccataccagggaagatcctccttttttagaaatgtggtatggcttaccaaaaagaacaatgcatacccaacaataaagaaaagttacaataataccaaccaagaagatcttttggtactatgggggattcaccatccaaatgatgcggcagagcagacagggctttatcaaaacccaactacctatatttccgttgggacatcaacactaaaccagagattggtaccaaaaatagctactagatctaaggtaaacgggcaaagtggaaggatggagttcttttggacaattttaaaatcgaatgatgtaataaactttgagagtaatggaaatttcattgctccagaaaatgcatacaaaattgtcaagaaaggggactcaacaattatgaaaagtgagttggaatatggtaactgcaacaccaagtgtcagactccaataggggcgataaactccagtatgccattccacaacatccaccctctcaccatcggggaatgccccaaatatgtgaaatcaaacagattagtccttgctactgggctcagaaatagccctcaaggagagagaagaagaaaaaagaga

>H5N1_A/Egypt/N08932/2010

taccatgcaaacaactcgacagagcaggttgacacaataatggaaaagaacgtcactgttacacacgcccaagacatactggaaaagacacacaacgggaaactctgcaatctagatggagtgaagcctctaattttaagagattgtagtgtagctggatggctcctcgggaacccaatgtgcgacgaattcctcaatgtgccggaatggtcttacatagtggagaagatcaatccagccaatgacctctgttatccagggaatttcaacgactatgaagaactgaaacacctattgagcagaataaaccattttgagaaaattcagatcattcccaaagattcttggtcagatcatgaagccGGGtcaggagtgagctcagcatgtccataccagggaagatcctccttttttagaaatgtggtatggcttaccaaaaagaacgatgcatacccaacaataaagaaaagttacaataataccaaccaagaagatcttttggtactatgggggattcaccatccaaatgatgctgcagagcagacaaggctttatcaaaacccaactacctatatttccgttgggacatcaacactaaaccagagattggtacccaaaatagctactagatctaaggtaaacgggcaaagtggaaggatggagttcttttggacaattttaaaatcgaatgatgcaataaactttgagagtaatggaaacttcattgctccagaaaatgcatacaaaattgtcaagaaaggggactcaacaattatgaaaagtgagttggaatatagtaactgcaacaccaagtgtcagactccaataggggcgataaactccagtatgccattccacaacatccaccctctcaccmtcggggaatgccccaaatatgtgaaatcaaacagattagtccttgctactgggctcagaaatagccctcaaggagagagaagaagaaaaaagaga

>H5N1_A/Egypt/N09174/2009

taccatgcaaacaactcgacagagcaggttgacacaataatggaaaagaacgtcactgttacacacgcccaagacatactggaaaagacacacaacgggaaactctgcaatctagatggagtgaagcctctaattttaagagattgtagtgtagctggatggctcctcgggaacccaatgtgcgacgaattcctcaatgtgccggaatggtcttacatagtggagaagatcaatccagccaatgacctctgttatccagggaatttcaacgactatgaagaactgaaacacctattgagcagaataaaccattttgagaaaattcagatcattcccaaaaattcttggtcagatcatgaagccGGGtcaggagtgagctcagcatgtccataccagggaagatcctccttttttagaaatgtggtatggcttaccaaaaagaacaatgcatacccaacaataaagaaaagttacaataataccaaccaagaagatcttttggtactatgggggattcaccatccaaatgatgcggcagagcagacaaggctttatcaaaacccaactacctatatttccgttgggacatcaacactaaaccagagattggtacccaaaatagctactagatctaaggtaaacgggcaaagtggaaggatggagttcttttggacaattttaaaatcgaatgatgcaataaactttgagagtaatggaaatttcattgctccagaaaatgcatacaaaattgtcaagaaaggggactcaacaattatgaaaagtgagttggaatatggtaactgcaacaccaagtgtcagactccaataggggcgataaactccagtatgccattccacaacatccaccctctcaccatcggggaatgccccaaatatgtgaaatcaaacagattagtccttgctactgggctcagaaatagccctcaaggagagagaagaagaaaaaagaga

>H5N1_A/Egypt/N09407/2010

taccatgcaaacaactcgacagagcaggttgacacaataatggaaaagaacgtcactgttacacacgcccaagacatactggaaaagacacacaacgggaaactctgcaatctagatggagtgaagcctctaattttaagagattgtagtgtagctggatggctcctcgggaacccaatgtgcgacgaattcctcaatgtgccggaatggtcttacatagtggagaagatcaatccagccaatgacctctgttatccagggaatttcaacgactatgaagaactgaaacacctattgagcagaataaaccattttgagaaaattcagatcattcccaaagattcttggtcagatcatgaagccGGGtcaggagtgagctcagcatgtccataccagggaagatcctccttttttagaaatgtggtatggcttaccaaaaagaacaatgcatacccaacaataaagaaaagttacaataataccaaccaagaagatcttttggtactatgggggattcaccatccaaatgatgcggcagagcagacaaggctttatcaaaacccaactacctatatttccgttgggacatcaacactaaaccagagattggtacccaaaatagctactagatctaaggtaaacgggcaaagtggaaggatggagttcttttggacaattttaaaatcgaatgatgcaataaactttgagagtaatggaaatttcattgctccagaaaatgcatacaaaattgtcaagaaaggggactcaacaattatgaaaagtgagttggaatatggtaactgcaacaccaagtgtcagactccaataggggcgataaactccagtatgccattccacaacatccaccctctcaccatcggggaatgccccaaatatgtgaaatcaaacagattagtccttgctactgggctcagaaatagccctcaaggagagagaagaagaaaaaagaga

>H5N1_A/Egypt/N09539/2009

taccatgcaaacaactcgacagagcaggttgacacaataatggaaaagaacgtcactgttacacatgcccaagacatactggaaaagacacacaacgggaaactctgcaatctagatggagtgaagcctctaattttaagagattgtagtgtagctggatggctcctcgggaacccaatgtgcgacgaattcctcaatgtgccggaatggtcttacatagtggagaagatcaatccagccaatgacctctgttatccagggaatttcaacgactatgaagaactgaaacacctattgagcagaataaaccattttgagaaaattcagatcattcccaaaaattcttggtcagatcatgaagccGGGtcaggagtgagctcagcatgtccataccagggaagatcctccttttttagaaatgtggtatggcttaccaaaaagaacaatgcatacccaacaataaagaaaagttacaataataccaaccaagaagatcttttggtactatgggggattcaccatccaaatgatgcggcagagcagacaaggctttatcaaaacccaactacctatatttccgttgggacatcaacactaaaccagagattggtaccaaaaatagctactagatctaaggtaaacgggcaaagtggaaggatggagttcttttggacaattttaaaatcgaatgatgcaataaactttgagagtaatggaaatttcattgctccagaaaatgcatacaaaattgtcaagaaaggggactcaacaattatgaaaagtgagttggaatatggtaactgcaacaccaagtgtcagactccaataggggcgataaactccagtatgccattccacaacatccaccctctcaccatcggggaatgccccaaatatgtgaaatcaaacagattagtccttgctactgggctcagaaatagccctcaaggagagagaagaagaaaaaagaga

>H5N1_A/Egypt/N09966/2011

taccatgcaaacaactcgacagagcaggttgacacgataatggaaaagaacgtcactgttacacacgcccaagacatactggaaaaaacacacaacgggaaactctgcaatctagatggagtgaagcctctaattttaagagattgtagtgtagctggatggctcctcgggaacccaatgtgcgacgaattcctcaatgtgccggaatggtcttacatagtggagaagatcaatccagctaatgacctctgttatccagggaatttcaatgactatgaagaactgaaacacctattgagcagaataaaccattttgagaaaattcagatcattcccaaagattcttggtcagatcatgaagccGGGtcaggagtgagctcagcatgtccataccagggaagatcctccttttttagaaatgtggtatggcttaccaaaaagaacgatgcatacccaacaataaagaaaagttacaataataccaaccaagaagatcttttggtactatgggggattcaccatccaaatgatgctgcagagcagacaaggctttatcaaaacccaactacctatatttccgttgggacatcaacactaaaccagagattggtacccaaaatagctactagatctaaggtaaacgggcaaagtggaaggatggagttcttttggacaattttaaaatcgaatgatgcaataaactttgagagtaatgggaacttcattgctccagaaaatgcatacaaaattgtcaagaaaggggactcaacaattatgaaaagtgagttggaatatagtaactgcaacaccaagtgtcagactccaataggggcgataaactccagtatgccattccacaacatccaccctctcaccatcggggaatgccccaaatatgtgaaatcaaacagattagtccttgctactgggctcagaaatagccctcaaggagagaaaagaagaaaaaagaga

>H5N1_A/Egypt/N10621/2011

taccatgcaaacaactcgacagagcaggttgacacaataatggaaaagaacgtcactgttacacacgcccaagacatactggaaaagacacacaacgggaaactctgcaatctagatggagtgaagcctctaattttaagagattgtagtgtagctggatggctcctcgggaacccaatgtgcgacgaattcctcaatgtgccggaatggtcttacatagtggagaagatcaatccagccaatgacctctgttatccagggaatttcaacgactatgaagaactgaaacacctattgagcagaataaaccattttgagaaaattcagatcattcccaacgattcttggtcagatcatgaagccGGGtcaggagtgagctcagcatgtccataccagggaagatcctccttttttagaaatgtggtatggcttaccaaaaagaacgatgcatacccgacaataaagaaaagttacaataataccaaccaagaagatcttttggtactatgggggattcaccatccaaatgatgctgcagagcagacaaggctttatcaaaacccaactacctatatttccgttgggacatcaacactaaaccagagattggtacccaaaatagctactagatctaaggtaaacgggcaaagtggaaggatggagttcttttggacaattttaaaatcgaatgatgcaataaattttgagagtaatggaaacttcattgctccagaaaatgcatacaaaattgtcaagaaaggggactcaacaattatgaaaagtgagttggaatatagtaactgcaacaccaagtgtcagactccaataggggcgataaactccagtatgccattccacaacatccaccctctcaccatcggggaatgccccaaatatgtgaaatcaaacagattagtccttgctactgggctcagaaatagccctcaaggagagaaaagaagaaaaaagaga

>H5N1_A/Egypt/N10954/2010

taccatgcaaacaactcgacagagcaggttgacacaataatggaaaagaacgtcactgttacacacgcccaagacatactggaaaagacacacaacgggaaactctgcaatctagatggagtgaagcctctaattttaagagattgtagtgtagctggatggctcctcgggaacccaatgtgcgacgaattcctcaatgtgccggaatggtcttacatagtggagaagatcaatccagccaatgacctctgttatccagggaatttcaacgactatgaagaactgaaacacctattgagcagaataaaccattttgagaaaattcagatcattcccaaagattcttggtcagatcatgaagccGGGtcaggagtgagctcagcatgtccataccagggaagatcctccttttttagaaatgtggtatggcttaccaaaaagaacgatgcatacccaacaataaagaaaagttacaataataccaaccaagaagatcttttggtactatgggggattcaccatccaaatgatgctgcagagcagacaaggctttatcaaaacccaactacctatatttccgttgggacatcaacactaaaccagagattggtacccaaaatagctactagatctaaggtaaacgggcaaaatggaaggatggagttcttttggacaattttaaaatcgaatgatgcaataaattttgagagtaatggaaacttcattgctccagaaaatgcatacaaaattgtcaagaaaggggactcaacaattatgaaaagtgagttggaatatagtaactgcaacaccaagtgtcagactccaataggggcgataaactccagtatgccattccacaacatccaccctctcaccatcggggaatgccccaaatatgtgaaatcaaacagattagtccttgctactgggctcagaaatagccctcaaggagagaaaagaagaaaaaagaga

>H5N1_A/Egypt/N11126/2011

taccatgcaaacaactcgacagagcaggttgacacaataatggaaaagaacgtcactgttacacacgcccaagacatactggaaaagacacacaacgggaaactctgcaatctagatggagtgaagcctctaattttaagagattgtagtgtagctggatggctcctcgggaacccaatgtgcgacgaattcctcaatgtgccggaatggtcttacatagtggagaagatcaatccagccaatgacctctgttatccagggaatttcaacgactatgaagaactgaaacacctgttgagcagaataaaccattttgagaaaattcagatcattcccaaagattcttggtcagatcatgaagccGGGtcaggagtgagctcagcatgtccataccagggaagatcctccttttttagaaatgtggtatggcttaccaaaaagaacgatgcatacccaacaataaagaaaagttacaataataccaatcaagaagatcttttggtactatgggggattcaccatccaaatgatgctgcagagcagacaagactttaccaaaacccaactacctatatttccgttgggacatcaacactaaaccagagattggtacccaaaatagctactagatctaaggtaaacgggcaaaatggaaggatggagttcttttggacaattttaaaatcgaatgatgcaataaactttgagagtaatggaaacttcattgctccagaaaatgcatacaaaattgtcaagaaaggggactcaacaattatgaaaagtgagttggaatatagtaactgcaacaccaaatgccagactccaataggggcgataaattccagtatgccattccacaacatccaccctctcaccatcggggaatgccccaaatatgtgaaatcaaacagattagtccttgctactgggctcagaaatagccctcaaggagagaaaagaagaaaaaagaga

>H5N1_A/Egypt/N11470/2011

taccatgcaaacaactcgacagagcaggttgacacaataatggaaaagaacgtcactgttacacacgcccaagacatactggaaaagacacacaacgggaaactctgcaatctagatggagtgaagcctctaattttaagagattgtagtgtagctggatggctcctcgggaacccaatgtgcgacgaattcctcaatgtgccggaatggtcttacatagtggagaagatcaatccagccaatgacctctgttatccagggaatttcaacgactatgaagaactgaaacacctgttgagcagaataaaccattttgagaaaattcagatcattcccaaagattcttggtcagatcatgaagccGGGtcaggagtgagctcagcatgtccataccagggaagatcctccttttttagaaatgtggtatggcttaccaaaaagaacgatgcatacccaacaataaagaaaagttacaataataccaatcaagaagatcttttggtactatgggggattcaccatccaaatgatgctgcagagcagacaagactttaccaaaacccaactacctatatttccgttgggacatcaacactaaaccagagattggtacccaaaatagctactagatctaaggtaaacgggcaaagtggaaggatggagttcttttggacaattttaaaatcgaatgatgcaataaactttgagagtaatggaaacttcattgctccagaaaatgcatacaaaattgtcaagaaaggggactcaacaattatgaaaagtgagttggaatatagtaactgcaacaccaaatgccagactccaataggggcgataaattccagtatgccattccacaacatccaccctctcaccatcggggaatgccccaaatatgtgaaatcaaacagattagtccttgctactgggctcagaaatagccctcaaggagagaaaagaagaaaaaagaga

>H5N1_A/Egypt/N11981/2009

taccatgcaaacaactcgacagagcaggttgacacaataatggaaaagaacgtcactgttacacacgcccaagacatactggaaaagacacacaatgggaaactctgcaatctagatggagtgaagcctctaattttaagagattgtagtgtagccggatggctcctcgggaatccaatgtgcgacgagttcctcaatgtgccggaatggtcttacatagtggaaaagatcaatccaaccaatgacctctgttatccagggaatttcaacgactatgaagaactgaaacacctattgagcagaataaaccattttgagaaaattcagatcatccccaaaaattcttggtcagatcatgaagccGGGtcaggagtgagctcagcatgtccataccagggaagatcctccttttttagaaatgtggtatggcttaccaaaaagaacaatgcatacccaacaataaagagaagttacaataataccaaccaagaagatcttttggtactatgggggattcaccatccaaatgatgcggcagagcagacaaggctttatcaaaacccaactacctatatttccgttgggacatcaacactaaaccagagattggtaccaaaaatagctactagatctaaggtaaacgggcaaagtggaaggatggaattcttttggacaattttaaaatcgaatgatgcaataaactttgagagtaatggaaatttcattgctccagaaaatgcatacaaaattgtcaagaaaggggactcaacaattatgaaaagtgagttggaatatggtaactgcaacaccaagtgtcagactccaataggggcgataaactccagtatgccattccacaacatccaccctctcaccatcggggaatgccccaaatatgtgaaatcaaacagattagtccttgctactgggctcagaaatagccctcaaggagagagaagaagaaaaaagaga

>H5N1_A/Egypt/N14976/2011

taccatgcaaacaactcgacagagcaggttgacacaataatggaaaagaacgtcactgttacacacgcccaagacatactggaaaagacacacaacgggaaactctgcaatctagatggagtgaagcctctaattttaagagattgtagtgtagctggatggctcctcgggaacccaatgtgcgacgaattcctcaatgtgccggaatggtcttacatagtggagaagatcaatccagccaatgacctctgttatccagggaatttcaacgactatgaagaactgaaacacctgttgagcagaataaaccattttgagaaaattcagatcattcccaaagattcttggtcagatcatgaagccGGGtcaggagtgagctcagcatgtccataccagggaagatcctccttttttagaaatgtggtatggcttaccaaaaagaacgatgcatacccaacaataaagaaaagttacaataataccaatcaagaagatcttttggtactatgggggattcaccatccaaatgatgctgcagagcagacaagactttaccaaaacccaactacctatatttccgttgggacatcaacactaaaccagagattggtacccaaaatagctactagatctaaggtaaacgggcaaagtggaaggatggagttcttttggacaattttaaaatcgaatgatgcaataaactttgagagtaatggaaacttcattgctccagaaaatgcatacaaaattgtcaagaaaggggactcaacaattatgaaaagtgagttggaatatagtaactgcaacaccaaatgccagactccaataggggcgataaattccagtatgccattccacaacatccaccctctcaccatcggggaatgccccaaatatgtgaaatcaaacagattagtccttgctactgggctcagaaatagccctcaaggagagaaaagaagaaaaaagaga

>H5N1_A/Egypt/N15262/2009

taccatgcaaacaactcgacagagcaggttgacacaataatggaaaagaacgtcactgttacacacgcccaagacatactggaaaagacacacaacgggaaactctgcaatctagatggagtgaagcctctaattttaagagattgtagtgtagccggatggctcctcgggaacccaatgtgcgacgaattcctcaatgtgccggaatggtcttacatagtggagaagatcaatccagccaatgacctctgttatccagggaatttcaacgactatgaagaactgaaacacctattgagcagaataaaccattttgagaaaattcagatcatccccaaaaattcttggtcagatcatggagccGGGtcaggagtgagctcagcatgtccataccagggaagatcctccttttttagaaatgtggtatggcttaccaaaaagaacaatgcatacccaacaataaagaaaagttacaataataccaaccaagaagatcttttggtactatgggggattcaccatccaaatgatgcggaagagcagacaaggctttatcaaaacccaactacctatatttccgttgggacatcaacactaaaccagagattggtaccaaaaatagctactagatctaaggtaaacgggcaaagtggaaggatggaattcttttggacaattttaaaatcgaatgatgcaataaactttgagagcaatggaaatttcattgctccagaaaatgcatacaaaattgtcaagaaaggggactcaacaattatgaaaagtgagttggaatatgataactgcaacaccaagtgtcagactccaataggggcgataaactccagtatgccattccacaacatccaccctctcaccatcggggaatgccccaaatatgtgaaatcaaacaaattagtccttgctactgggctcagaaatagccctcaaggagagaaaagaagaaaaaagaga

>H5N1_A/Egypt/N16789/2010

taccatgcaaacaactcgacagagcaggttgacacaataatggaaaagaacgtcactgttacacacgcccaagacatactggaaaagacacacaacgggaaactctgcaatctagatggagtgaagcctctaattttaagagattgtagtgtagctggatggctcctcggaaacccaatgtgcgacgaattcctcaatgtgccggaatggtcttacatagtggagaagaacaatccagccaatgacctctgttatccagggaatttcaacgactatgaagaactgaaacacctattgagcagaataaaccattttgagaaaattcagatcattcccaaagattcttggtcagatcatgaagccGGGtcaggagtgagctcagcatgtccataccagggaagatcctccttttttagaaatgtggtatggcttaccaaaaagaacgatgcatacccaacaataaagaaaagttacaataataccaaccaagaagatcttttggtactatgggggattcaccatccaaatgatgctgcagagcagacaaggctttatcaaaatccaactacctatatttccgttgggacatcaacactaaaccagagattggtacccaaaatagctactagatctaaggtaaacgggcaaagtggaaggatggagttcttttggacaattttaaaatcgaatgatgcaataaactttgagagtaatggaaatttcattgctccagaaaatgcatacaaaattgtcaagaaaggggactcaacaattatgaaaagtgagttggaatatagtaactgcaacaccaagtgtcagactccaataggggcgataaactccagtatgccattccacaacatccaccctctcaccatcggggaatgccccaaatatgtgaaatcaaacagattagtccttgctactgggctcagaaatagtcctcaaggagagaggagaagaaaaaagaga

>H5N1_A/Egypt/N6322/2011

taccatgcaaacaactcgacagagcaggttgacacaataatggaaaagaacgtcactgttacacacgcccaagacatactggaaaagacacacaacgggaaactctgcaacctagatggagtgaagcctctaattttaagagattgcagtgtagctggatggctcctcgggaacccaatgtgcgacgaattcctcaatgtgccggaatggtcctacatagtggagaagatcaatccagccaatgacctctgttatccagggaatttcaacgactatgaagaactgaaacacctattgagcagaataaaccattttgagaaaattcagatcattcccaaagattcttggtcagatcatgaagccGGGtcaggagtgagctcagcatgtccataccagggaagatcctccttttttagaaatgtggtatggcttaccaaaaagaacgatgcatacccaacaataaagaaaagttacaataataccaaccaagaagatcttttggtactatggggaattcaccatccaaatgatgctgcagagcagacaaggctttatcaaaacccaactacctatatttccgttgggacatcaacactaaaccagagattggtacccaaaatagctactagatctaaggtaaacgggcaaagtggaaggatggagttcttttggacaattttaaaatcgaatgatgcaataaactttgagagtaatggaaacttcattgctccagaaaatgcatacaaaattgtcaagaaaggggactcaacaattatgaaaagtgagttggaatatagtaactgcaacaccaagtgtcagactccaataggggcgataaactccagtatgccattccacaacatccaccctctcaccatcggggaatgccccaaatatgtgaaatcaaacagattagtccttgctactgggctcagaaatagccctcaaggagagaaaagaagaaaaaagaga

>H5N1_A/Egypt/N6658/2011

taccatgcaaacaactcgacagagcaggttgacacaataatggaaaagaacgtcactgttacacacgcccaagacatactggaaaagacacacaacgggaaactctgcaatctagatggagtgaagcctctaattttaagagattgtagtgtagctggatggctcctcgggaacccaatgtgcgacgaattcctcaatgtgccggaatggtcttacatagtggagaagatcaatccagccaatgacctctgttatccagggaatttcaacgactatgaagaactgaaacacctattgagcagaataaaccattttgagaaaattcagatcattcccaaagattcttggtcagatcatgaagccGGGtcaggagtgagctcagcatgtccataccagggaaggtcctccttttttagaaatgtggtatggcttaccaaaaagaacgatgcatacccaacaataaagaaaagttacaataataccaaccaagaagatcttttggtactatgggggattcaccatccaaatgatgctgcagagcagacaaggctttatcaaaacccaactacctatatttccgttgggacatcaacactaaaccagagattggtacccaaaatagctaatagatctaaggtaaacgggcaaagtggaaggatggagttcttttggacaattttaaaatcgaatgatgcaataaactttgagagtaatggaaacttcattgctccagaaaatgcatacaaaattgtcaagaaaggggactcaacaattatgaaaagtgagttggaatatagtaactgcaacaccaagtgtcagactccaataggggcgataaactccagtatgccattccacaacattcaccctctcaccatcggggaatgccccaaatatgtgaaatcaaacagattagtccttgctactgggctcagaaatagccctcaaggagagaaaagaagaaaaaagaga

>H5N1_A/Egypt/N6774/2011

taccatgcaaacaactcgacagagcaggttgacacaataatggaaaagaacgtcactgttacacacgcccaagacatactggaaaagacacacaacgggaaactctgcaatctagatggagtgaagcctctaattttaagagattgtagtgtagctggatggctcctcgggaacccaatgtgcgacgaattcctcaatgtgccggaatggtcttacatagtggagaagatcaatccagccaatgacctctgttatccagggaatttcaacgactatgaagaactgaaacacctattgagcagaataaaccattttgagaaaattcagatcattcccaaagattcttggtcagatcatgaagccGGGtcaggagtgagctcagcatgtccataccagggaaggtcctccttttttagaaatgtggtatggcttaccaaaaagaacgatgcgtacccaacaataaagaaaagttacaataataccaaccaagaagatcttttggtactatgggggattcaccatccaaatgatgctgcagagcagacaaggctttatcaaaacccaactacctatatttccgttgggacatcaacactaaaccagagattggtacccaaaatagctactagatctaaggtaaacgggcaaagtggaaggatggagttcttttggacaattttaaaatcgaatgatgcaataaactttgagagtaatggaaacttcattgctccagaaaatgcatacaaaattgtcaagaaaggggactcaacaattatgaaaagtgagttggaatatagtaactgcaacaccaagtgtcagactccaataggggcgataaactccagtatgccattccacaacattcaccctctcaccatcggggaatgccccaaatatgtgaaatcaaacagattagtccttgctactgggctcagaaatagccctcaaggagggaaaagaagaaaaaagaga

>H5N1_A/Egypt/N6828/2011

taccatgcaaacaactcgacagagcaggttgacacaataatggaaaagaacgtcactgttacacacgcccaagacatactggaaaagacacacaacgggaaactctgcaatctagatggagtgaaacctctaattttaagagattgtagtgtagctggatggctcctcgggaacccaatgtgcgacgaattcctcaatgtgccggaatggtcttacatagtggaaaagatcaatccagccaatgacctctgttatccagggaatttcaacgactatgaagaactgaaacacctattgagcagaataaaccattttgagaaaattcagatcattcccaaagattcttggtcagatcatgaaaccGGGtcaggagtgagctcagcatgtccataccagggaagatcctccttttttagaaatgtggtatggcttaccaaaaagaacgatgcatacccaacaataaagaaaagttacaataataccaaccaagaagatcttttggtactatggggaattcaccatccaaatgatgctgcagagcagacaaggctttatcaaaacccaactacctatgtttccgttgggacatcaacactaaaccagagattggtacccaaaatagctactagatctaaggtaaacgggcaaagtgggaggatggagttcttttggacaattttaaaatcgaatgatgcaataaactttgagagtaatggaaacttcattgctccagaaaatgcatacaaaattgtcaagaaaggggactcaacaattatgaaaagtgagttggaatatagtaactgcaacaccaagtgtcagactccaataggggcgataaactccagtatgccattccacaacatccaccctctcaccatcggggaatgccccaaatatgtgaaatcaaacagattagtccttgctactgggctcagaaatagccctcaaggagagaaaagaagaaaaaagaga

>H5N1_A/Egypt/N7562/2011

taccatgcaaacaactcgacagagcaggttgacacaataatggaaaagaacgtcactgttacacacgcccaagacatactggaaaagacacacaacgggaaactctgcaatctagatggagtgaagcctctaattttaagagattgtagtgtagctggatggctcctcgggaacccaatgtgcgacgaattcctcaatgtgccggaatggtcttacatagtggaaaagatcaatccagccaatgacctctgttatccagggaatttcaacgactatgaagaactgaaacacctattgagcagaataaaccattttgagaaaattcagatcattcccaaagattcttggtcagatcatgaagccGGGtcaggagtgagctcagcatgtccataccagggaagatcctccttttttagaaatgtggtatggcttaccaaaaagaacgatgcatacccaacaataaagaaaagttacaataataccaaccaagaagatcttttggtactatgggggattcaccatccaaatgatgctgcagagcagacaaggctttatcaaaacccaactacctatatttccgttgggacatcaacactaaaccagagattggtacccaaaatagctactagatctaaggtaaacgggcaaagtggaaggatggagttcttttggacaattttaaaatcgaatgatgcaataaactttgagagtaatggaaacttcattgctccagaaaatgcatacaaaattgtcaagaaaggggactcaacaattatgaaaagtgagttggaatatagtaactgcaacaccaagtgtcagactccaataggggcgataaactccagtatgccattccacaacatccaccctctcaccatcggggaatgccccaaatatgtgaaatcaaacagattagtccttgctactgggctcagaaatagccctcaaggagagaaaagaagaaaaaagaga

>H5N1_A/Egypt/N7592/2011

taccatgcaaacaactcgacagagcaggttgacacaataatggaaaagaacgtcactgttacacacgcccaagacatactggaaaagacacacaacgggaaactctgcaatctagatggrgtgaagcctctaattttaagagattgtagtgtagctggatggctcctcgggaacccaatgtgcgacgaattcctcaatgtgccggaatggtcttacatagtggagaagatcaatccagccaatgacctctgttatccagggaatttcaacgactatgaagaactgaaacacctattgagcagaataaaccattttgagaaaattcagatcattcccaaagattcttggtcagatcatgaagccGGGtcaggagtgagctcagcatgtccataccagggaaggtcctccttttttagaaatgtggtatggcttaccaaaaagaacgatgcatacccaacaataaagaaaagttacaataataccaaccaagaagatcttttggtactatgggggattcaccatccaaatgatgctgcagagcagacaaggctttatcaaaacccaactacctatatttccgttgggacatcaacactaaaccagagattggtacccaaaatagctactagatctaaggtaaacgggcaaagtggaagaatggagttcttttggacaattttaaaatcgaatgatgcaataaactttgagagtaatggaaacttcattgctccagaaaatgcatacaaaattgtcaagaaaggggactcaacaattatgaaaagtgagttggaatatagtaactgcaacaccaagtgtcagactccaataggggcgataaactccagtatgccattccacaacattcaccctctcaccatcggggaatgccccaaatacgtgaaatcaaacagattagtccttgctactgggctcagaaatagccctcaaggagagaaaagaagaaaaaagaga

>H5N1_A/Egypt/N7724/2011

taccatgcaaacaactcgacagagcaggttgacacaataatggaaaagaacgtcactgttacacacgcccaagacatactggaaaagacacacaacgggaaactctgcaatctagatggagtgaagcctctaattttaagagattgtagtgtagctggatggctcctcgggaacccaatgtgcgacgaattcctcaatgtgccggaatggtcttacatagtggagaagatcaatccagccaatgacctctgttatccagggaatttcaacgactatgaagaactgaaacacctattgagcagaataaaccattttgagaaaattcagatcattcccaaagattcttggtcagatcatgaagccGGGtcaggagtgagctcagcatgtccataccagggaaggtcctccttttttagaaatgtggtatggcttaccaaaaagaacgatgcatacccaacaataaagaaaagttacaataataccaaccaagaagatcttttggtactatgggggattcaccatccaaatgatgctgcagagcagacaaggctttatcaaaacccaactacctatatttccgttgggacatcaacactaaaccagagattggtacccaaaatagctactagatctaaggtaaacgggcaaaatggaaggatggagttcttttggacaattttaaaatcgaatgatgcaataaactttgagagtaatggaaayttcattgctccagaaaatgcatacaaaattgtcaagaaaggggactcaacaattatgaaaagtgagttggaatatagtaactgcaacaccaagtgtcagactccaataggggcgataaactccagtatgccattccacaacattcaccctctcaccatcggggaatgccccaaatatgtgaaatcaaacagattagtccttgctactgggctcagaaatagccctcaaggagagaaaagaagaaaaaagaga

>H5N1_A/England/215201407/2021

taccatgcaaacaattcgacagagcaagttgacacgataatggaaaagaacgtcactgttacacatgcccaagacatactggaaaaaacacacaacgggaagctctgtgatctaaatggggtgaagcctctgattttaaaggattgtagtgtagctggatggctcctcggaaacccaatgtgcgacgaattcatcagagtgccggaatggtcctacatagtggagcgggctaatccagctaatgacctctgttacccagggagcctcaatgactatgaagaactgaaacacctgttgagcagaataaatcattttgagaagattctgatcatccccaagagttcctggccaaatcatgaaacatcactaggggtgagcgcagcttgtccataccagggaacgccctcctttttcagaaatgtggtgtggcttatcaaaaagaacgatgcatacccaacaataaagataagctacaataataccaatcgggaagatctcttgatactgtgggggattcatcattccaacaatgcagaagagcagacaaatctctacaaaaacccaaccacctacatttcagttgggacatcaactttaaaccagaggttggtaccaaaaatagctactagatcccaagtaaacgggcaacgtggaagaatggacttcttctggacaattttaaaaccagatgatgcaatccatttcgagagtaatggaaatttcattgctccagaatatgcatacaaaattgtcaagaaaggggactcaacaattatgaaaagtggagtggaatatggccactgcaacaccaaatgtcaaaccccagtaggagcgataaattctagtatgccattccacaacatacatcctctcaccattggggaatgccccaaatacgtgaagtcaaacaagttggtccttgcgactgggcttagaaatagtcctctaagagaaGGGaagagaagaaaaaga

>H5N1_A/Fujian/1/2005

taccatgcaaacaactcgacagagcaggttgacacaataatggaaaagaacgttactgttacacatgcccaagacatactggaaaagacacacaacgggaagctctgcgatctagatggagtgaagcctctgattttaagagattgtagtgtagctggatggctcctcggaaacccaatgtgtgacgaattcatcaatgtgccggaatggtcttacatagtggagaaggccaacccagccaatgacctctgttacccagggaatttcaacgactatgaagaactgaaacacctattgagcagaataaaccattttgagaaaattcagatcatccccaaaagttcttggtccgatcatgaagcctcatcaggggtgagctcagcatgtccataccagggaacgccctcctttttcagaaatgtggtatggcttatcaaaaagaacaatacatacccaacaataaagagaagctacaataataccaaccaggaagatcttttgatactgtgggggattcatcattctaatgatgcggcagagcagacaaagctctatcaaaacccaaccacctatatttccgttgggacatcaacactaaaccagagattggtaccaaaaatagctactagatccaaagtaaacgggcaaagtggaaggatggatttcttctggacaattttaaaaccaaatgatgcaatcaacttcgagagtaatggaaatttcattgctccagaatatgcatacaaaattgtcaagaaaggggactcagcaattataaaaagtgaagtggaatatggtaactgcaacaccaagtgtcaaactccaataggggcgataaactctagtatgccattccacaacatacaccctctcaccatcggggaatgcccaaaatatgtgaaatcaaacaaattagtccttgcgactgggctcagaaatagtcctctaagagaaagGGGaagaagaaaaaga

>H5N1_A/Fujian/1/2007

taccatgcaaacaactcgacagagcaggttgacacaataatggaaaagaacgttactgttacacatgcccaagatatactggaaaagacacacaacgggaagctctgcgatctagatggagtgaagcctctgattttaagagattgtagtgtagctggatggctcctcggaaacccaatgtgtgacgaattcatcaatgtgccggaatggtcttacatagtggagaaggccaacccagccaatgacctctgttacccagggaatttcaacgactatgaagaactgaaacacctattgagcagaataaatcattttgagaaaattcagatcatccccaaaagttcttggtccgatcatgaagcctcattgggggtgagctcagcatgtccataccagggaatgccctcctttttcagaaatgtggtatggcttatcaaaaagaacaatacatacccaacaataaagagaagctacaataataccaaccaggaagatcttttgatactgtgggggattcatcattctaatgatgcggcagagcagacaaagctctatcaaaacccaaccacttatatttccgttgggacgtcaacactaaaccagagattggtaccaaaaatagctactagatccaaagtaaacgggcaaagtggaaggatggatttcttctggacaattttaaaaccaaatgatgcaatcaacttcgagagtaatggaaatttcattgctccagaatatgcatacaaaattgtcaagaaaggggactcagcaattatgaaaagtgaagtgggatatggtaactgcaacaccaagtgtcaaactccaataggggcaataaactctagtatgccattccacaacatacaccctctcaccatcggggaatgccccaaatatgtgaaatcaaacaaattagtccttgcgactgggctcagaaatagtcctctaagagagagGGGaagaagaaaaaga

>H5N1_A/Guangdong-Shenzhen/1/2011(H5N1)

taccatgcaaacaactcgacagagcaggttgacacaataatggaaaagaacgttactgttacacatgctcaagacatactggagaagacgcacaatgggaagctctgcgatctaaatggagtgaagcctctgattttaaaagattgtagtgtagcaggatggctcctcgggaacccaatgtgtgacgaattcatcgatgtgccagaatggtcttacatagtagagaagaccaatccagccaatgacctctgttacccagggagtttcaacgattatgaagaattaaaacacctattgagcagaataaaccactttgagaaaatacgaatcattcccaaagattcttggccagatcatgaagcctcattgggggtgagcgcagcatgttcataccagggaaattcctcatttttcagaaatgtggtatggcttctcaaaaaggacgatgcatacccaataataacgaaaagctacaataataccaataaagaagatctcttggtactgtgggggatccatcatcctaatgatgaggcagagcagataaggctctataaaaactcaaccacctatgtttccattgggacatcaacactaaaccagagattggtaccaagaatagctactagatccaaagtaaacgggcaacgtggaaggatagatttcttctggacaattttaaaaccgaatgatgcaatcaacttcgagagtaatggaaatttcattgctccagaatatgcatacaaaattgtcaagaaaggagactcaacaattatgagaagtgaagtggaatatggtaactgcagcaccaggtgtcagactccgatgggggcgataaattctagtatgccattccacaacatacaccctctcaccatcggagaatgccccaaatatgtgaaatcaaacaaattagtccttgcgactgggctcagaaatagtcctcaaatagagagaagaagaagaaaaagg

>H5N1_A/Guangdong/1/2006

taccatgcaaacaactcgacagagcaggttgacacaataatggaaaagaacgttactgttacacatgcccaagacatactggaaaagacacacaacgggaagctctgcgatctagatggagtgaagcctctgattttaagagattgtagtgtagctggatggctcctcggaaacccaatgtgtgacgaattcatcaatgtgccggaatggtcttacatagtggagaaggccaacccagccaatgacctctgttacccagggaatttcaacgactatgaagaactgaaacacctattgagcagaataaaccattttgagaaaattcagatcatccccaaaagttcttggcccgatcatgaagcctcatcaggggtgagctcagcatgtccataccagggaacgccctcctttttcagaaatgtggtatggcttatcaaaaagaacaatacatacccaacaataaagagaagctacaataataccaaccaggaagatcttttgatactgtgggggattcatcattctaataatgcggcagagcagacaaagctctatcaaaacccaaccacctatatttccgttgggacatcaacactaaacctgagattggtaccaaaaatagctactagatccaaagtaaacgggcaaagtggaaggatggatttcttctggacaattttaaaaccgaatgatgcaatcaacttcgagagtaatggaaatttcattgctccagaatatgcatacaaaattgtcaagaaaggggactcagcaattatgaaaagtgaagtggaatatggtaactgcaacaccaagtgtcaaactccaataggggcgataaactctagtatgccattccacaacatacaccctctcaccatcggggaatgccccaaatatgtgaaatcaaacaaattagtccttgcgactgggctcagaaatagtcctctaagagagagGGGaagaagaaaaaga

>H5N1_A/Guangdong/1/2008

taccatgcaaacaactcgacagagcaggttgacacaataatggaaaagaacgttactgttacacatgcccaagacatactggagaagacacataacgggaaactctgcgatctagatggagtgaagcctctgattctacgagattgtagtgtagccggatggctcctcggaaacccaatgtgtgacgaattcatcaatgtgccggaatggtcttacatagtggagaaggccaacccagccaatgacctctgttacccagggaatttcaacgactatgaagaactgaaacacctattgagcagaataaaccattttgagaaaattcagatcatccccaaaagttcttggtccgatcatgaagcctcatcaggggtgagctcagcatgtccataccagggaacgccctcctttttcagaaatgtggtatggcttatcaaaaagaacaatacatacccaacaataaagagaagctacaataataccaaccaggaaaatcttttgatactgtgggggattcatcattctaatgatgcagcagagcagataaagctctatcaaaacccaaccacctatatttccgttgggacatcaacactaaaccagaggttggtaacaaaaatagccactagacccaaagtaaacgggcaaagtggaaggatggatttcttctggacaattttaaaaccgaatgatgcaatcaacttcgagagtaatggaaatttcattgctccagaatatgcatacaaaattgtcaaggaaggagactcagcaattatgaaaagtgaagtggaatatggtaactgcaacaccaagtgtcaaactccaataggggcgataaactctagtatgccattccacaacatacaccctctcaccatcggggaatgccccaaatatgtgaaatcaaacaaattagtccttgctactgggctcagaaatagtcctctaagagaaagGGGaagaagaaaaaga

>H5N1_A/Guangdong/2/2006

taccatgcaaacaactcgacagagcaggttgacacaataatggaaaagaacgttactgttacacatgcccaagatatactggaaaagacacacaacgggaagctctgcgatctagatggagtgaagcctctgattttaagagattgtagtgtagctggatggctcctcggaaacccaatgtgtgacgaattcatcaatgtgccggaatggtcttacatagtggagaaggccaacccagccaatgacctctgttacccagggaatttcaacgactatgaagaactgaaacacctattgagcagaataaaccattttgagaaaattcagatcatccccaaaagttcttggtccgatcatgaagcctcatcaggggtgagctcagcatgtccataccagggaacgccctcctttttcagaaatgtgatatggcttatcaaaaagaacaatacatacccaacaataaagagaagctacaataataccaaccaggaagatcttttgatactgtgggggattcatcattctaatgatgcggcagagcagacaaagctctatcaaaacccaaccacctatatttccgttgggacatcaacacttaaccagagattggtaccgaaaatagctactagatccaaagtaaacgggcaaagtggaaggatggatttcttctggacaattttaaaaccaaatgatgcaatcaacttcgagagtaatggaaatttcattgctccagaatatgcatacaaaattgtcaagaaaggggactcagcaattctgaaaagtgaagtggaatatggtaactgcaacaccaagtgtcaaactccaataggggcgataaactctagtatgccattccacaacatacaccctctcaccatcggggaatgccccaaatatgtgaaatcaaacaaattagtccttgcgactgggctcagaaatagtcctctaagagaaagGGGaagaagaaaaaga

>H5N1_A/Guangxi/1/2005

taccatgcaaacaactcgacagagcaggttgacacaataatggaaaagaacgttactgttacacatgcccaagatatactggaaaagacacacaacgggaagctctgcgatctagatggagtgaagcttctgattttaagagattgtagtgtagctggatggctcctcgggaacccaatgtgtgacgaattcatcaatgtgccggaatggtcttacatagtggagaaggccaacccagccaatgacctctgttacccagggaatttcaacgactatgaagaactgaaacacctattgagcagaataaaccattttgagaaaattcagatcatccccaaaagttcttggtccgatcatgaagcctcatcaggggggagctcagcatgtccataccagggaacgccctcctttttcagaaatgtggtatggcttatcaaaaagaacaatacatacccaacaataaagagaagctacaataataccaaccaggaagatcttttgatactgtgggggattcatcattctaatgatgcggcagagcagacaaagctctatcaaaacccaaccacctatatttccgttgggacatcaacactaaaccagagattggtaccaaaaatagctactagatccaaagtaaacgggcaaagtggaaggatggatttcttctggacaattttaaaaccgaatgatgcaatcaacttcgagagtaatggaaatttcattgctccagaatatgcatacaaaattgtcaagaaaggggactcagcaattatgaaaagtgaagtggaatatggtaactgcaacaccaagtgtcaaactccaataggggcgataaactctagtatgccattccacaacatacaccctctcaccatcggggaatgccccaaatatgtgaaatcaaacaaattagtccttgcgactgggctcagaaatagtcctctaagagaaagGGGaagaagaaaaaga

>H5N1_A/Guangxi/1/2008

taccatgcaaacaactcgacagagcaggttgacacaataatggagaagaacgttactgttacacatgctcaagatatactggaaaagacacacaacgggaaactctgtgatctagatggagtgaagcctctgattttaagagattgtagtgtagctggatggctcctcggaaacccaatgtgtgacgaattcatcaatgtgccggaatggtcatacatagtggagaaggccaacccagccaatgacctctgttacccagggaatttcaacgactatgaagaactgaaacacctattaagcaggataaaccattttgagaaaattcagatcatccccaaaaattcttggtccgatcatgaggcctcatcaggggtgagctcagcatgtccataccagggaacgccctcctttttcagaaatgtggtatggcttatcaaaaagaacaatacatacccaacaataaagagaagctacaataataccaaccaggaagatcttttgatactgtgggggattcatcattctaatgatgcagcagagcagacaaagctctatcaaaacccaaccacctatatttccgttgggacatcaacactgaaccagagattggtaccaaaaatagctactagatccaaagtaaacgggcaaagtggaaggatggatttcttctggacaattttaaaaccgaatgatgcaatcaacttcgagagtaatggaaatttcattgctccagaatatgcatacaaaattgtcaagaaaggggactcggcaattatgaaaagtgaggtggagtatggtaactgcaacaccaagtgtcaaacgccaataggggcgataaactctagtatgccattccacaacatacaccctctcaccatcggggaatgccccaaatatgtgaaatcaaacaaattagtccttgcgactgggctcagaaatagtcctctaagagaaagGGGaagaagaaaaaga

>H5N1_A/Guangxi/1/2009

taccatgcaaacaactcgacagagcaggttgacacaataatggaaaagaacgttactgttacacatgctcaagacatactggaaaagacacacaacgggaagctctgcgatctaaatggagtgaagcctctgattttaaaagattgtagtgtagcaggatggctcctcgggaacccaatgtgtgacgaattcatcgatgtgccagaatggtcttacatagtagagaaggccaatccagccaatgacctctgttacccagggaatttcaacgattatgaagaattgaaacacctattgagcagaataaaccattttgagaaaatacagatcatccccaaagattcttggccagatcatgaagcctcattgggggtgagctcarcatgtccataccagggaaattcctcctttttcagaaatgtggtatggcttatcaaaaagggcaatgcatacccaacaataaagaaaagctacaataataccaacaaagaagatctcttgatactgtgggggatccaccatcctaatgatgaggcagagcagacaaggctctaccaaaacccaaccacctatatttccattgggacatcaacactaaaccagagattggtaccaagaatagctactagatccaaagtaaacgggcaaagtggaaggatagatttcttctggacaattttaaaaccgaatgatgcaatcaacttcgagagtaatggaaatttcattgctccagaatatgcatacaaaattgtcaagaaaggagactcaacaattatgaaaagtgaagtggaatatggtaactgcaacaccaggtgtcagactccgatgggggcgattaactctagtatgccattccacaacatacaccctctcaccatcggagaatgtcccaaatatgtgaaatcaaacaaattagtccttgcgactgggctcagaaatagtcctcaaagagagagaagaagaagaaaaaga

>H5N1_A/Guangzhou/1/2006

taccatgcaaacaactcgacagagcaggttgacacaataatggaaaagaacgttactgttacacatgcccaagacatactggaaaagacacacaacgggaagctctgcgatctagatggagtgaagcctctgattttaagagattgtagtgtagctggatggctcctcggaaacccaatgtgtgacgaattcatcaatgtgccggaatggtcttacatagtggagaaggccaacccagccaatgacctctgttacccagggaatttcaacgactatgaagaactgaaacacctattgagcagaataaaccattttgagaaaattcagatcatctccaaaagttcttggtccgatcatgaagcctcatcaggggtgagctcagcatgtccataccagggaacgccctcctttttcagaaatgtggtatggcttatcaaaaagaacaatacatacccaacaataaagagaagctacaataataccaaccaggaagatcttttgatactgtggggaattcatcattctaataatgcggcagagcagacaaagctctatcaaaacccaaccacctatatttccgttgggacatcaacactaaacctgagattggtaccaaaaatagctactagatccaaagtaaacgggcaaagtggaaggatggatttcttctggacaattttaaaaccgaatgatgcaatcaacttcgagagtaatggaaatttcattgctccagaatatgcatacaaaattgtcaagaaaggggactcagcaattatgaaaagtgaagtggaatatggtaactgcaacaccaagtgtcaaactccaataggggcgataaactctagtatgccattccacaacatacaccctctcaccatcggggaatgccccaaatatgtgaaatcaaacaaattagtccttgcgactgggctcagaaatagtcctctaagagagagGGGaagaagaaaaaga

>H5N1_A/Guizhou/1/2009

taccatgcaaacaactcgacagagcaggttgacacaataatggaaaagaacgttactgttacacatgcccaagacatactggaaaagacacacaacgggaagctctgcgatctagatggagtgaagcctttgattttaagagattgtagtgtagctggatggctcctcggaaacccaatgtgtgacgaatttatcaatgtgccagaatggtcttacatagtggagaaggccaacccagccaatgacctctgttacccagggaatttcaacgactatgaagaactgaaacacctattgagcagaataaatcattttgagaaaattcagatcatccccaaaagttcttggtccgatcatgaagcctcatcaggggtgagctcagcatgtccgtatcagggaacgccctcttttttcaggaatgtagtatggcttatcaaaaagaacaatacatacccaacaataaagagaagctacaataacaccaaccaggaagatcttttggtactgtgggggattcaccattctaatgatgcggcagaacagataaagctctatcaaaacccaaccacctatgtttccgttggaacatcaacactaaaccagagattggtaccaaaaatagctactagatccaaagtaaacgggcaaagtggaaggatggatttcttctggacaattttaaaatcgaatgatgcaatcaacttcgagagtaatgggaatttcattgctccagaatatgcatacaaaattgtcaagaaaggggactcagcaattatgaaaagtgaagtggaatatggtaactgcagcaccaagtgtcaaactccaataggggcgataaactctagtatgccattccacaatatacaccctctcaccatcggggaatgccccaaatatgtgaaatcaaacaaattagtccttgcgactgggctcagaaatagtcctctaagagaaagGGGaagaagaaaaaga

>H5N1_A/Guizhou/1/2012

taccatgcaaacaactcaacagagcaggttgacacaataatggaaaagaacgtcactgttacacatgcccaagatatactggaaaggacacacaacgggaagctctgcgatctagatggagttaagcctctgattttaagagattgtagtgtagccggatggctcctcggaaacccaatgtgtgacgaattcatcaatgtgccggaatggtcttacatagtggagaaggccaacccggctaatgacctctgctacccagggaatctcaacgactatgaagaactgaaacacctattgagcagaataaaccattttgagaaaattcagatcatccccaaaagttcttggaccgatcatgaagcctcattgggagtgagcgcagcatgtccatacctggggacaccctcctttttcagaaatgtggtatggcttatcaagaagaacaatacatacccaacaataaagataagctacaataacaccaaccaggaagatcttttgatactgtgggggattcatcattctaatgatgagacagagcagataaagctctatcaaaacccaatcacctatgtttccgttgggacatcaacactaaatcagagattagtaccaaaaatagctaatagatccaaagtaaatgggcaaagtggaaggatggatttcttctggacaattctaaaaccggacgatgcaatcaacttcgagagtaatggaaatttcattgctccagaatatgcatacaaaattgtcaagaaaggagactcagcaattatgaaaagtgaagtggaatatggtcactgcaacaccaagtgtcaaactccaataggggcgataaactctagtatgccattccacaacatacaccctctcactatcggggaatgccccaaatatgtgaaatcaaacaaattagtccttgcgactgggctcagaaatagtcctctaagagaaaaGGGaagaagaaaaaga

>H5N1_A/Guizhou/1/2013

taccatgcaaacaactcaacagagcaggttgacacaataatggaaaagaacgtcactgttacacatgcccaagatatactggaaaggacacacaacgggaagctctgcgatctagatggagttaagcctctgattttaagagattgtagtgtagccggatggctcctcggaaacccaatgtgtgacgaattcatcaatgtgccggaatggtcttacatagtggagaaggccaacccggctaatgacctctgctacccagggaatctcaacgactatgaagaactgaaacacctattgagcagaataaaccattttgagaaaattcagatcatccccaaaagttcttggaccgatcatgaagcctcattgggagtgagcgcagcatgtccatacctggggacaccctcctttttcagaaatgtggtatggcttatcaagaagaacaatacatacccaacaataaagataagctacaataacaccaaccaggaagatcttttgatactgtgggggattcatcattctaatgatgagacagagcagataaagctctatcaaaacccaatcacctatgtttccgttgggacatcaacactaaatcagagattagtaccaaaaatagctaatagatccaaagtaaacgggcaaagtggaaggatggatttcttctggacaattctaaaaccggacgatgcaatcaacttcgagagtaatggaaatttcattgctccagaatatgcatacaaagttgtcaagaaaggagactcagcaattatgaaaagtgaagtggaatatggtcactgcaacaccaagtgtcaaactccaataggggcgataaactctagtatgccattccacaacatacaccctctcactatcggggaatgccccaaatatgtgaaatcaaacaaattattccttgcgactgggctcagaaatagtcctctaagagaaaaGGGaagaagaaaaaga

>H5N1_A/Guizhou/2/2013

taccatgcaaacaactcaacagagcaggttgacacaataatggaaaagaacgtcactgttacacatgcccaagatatactggaaaggacacacaacgggaagctctgcgatctagatggagttaagcctctgattttaagagattgtagtgtagccggatggctcctcggaaacccaatgtgtgacgaattcatcaatgtgccggaatggtcttacatagtggagaaggccaacccggctaatgacctctgctacccagggaatctcaacgactatgaagaactgaaacacctattgagcagaataaaccattttgagaaaattcagatcatacccaaaagttcttggaccgatcatgaagcctcattgggagtgagcgcagcatgtccatacctggggacaccctcctttttcagaaatgtggtatggcttatcaagaagaacaatacatacccaacaataaagataagctacaataacaccaaccaggaagatattttgatactgtgggggattcatcattctaatgatgagacagagcagataaagctctatcaaaacccaatcacctatgtttccgttgggacatcaacactaaatcagagattagtaccaaaaatagctaatagatccaaagtaaacgggcaaagtggaaggatggatttcttctggacaattctaaaaccggacgatgcaatcaacttcgagagtaatggaaatttcattgctccagaatatgcatacaaagttgtcaagaaaggagactcagcaattatgaaaagtgaagtggaatatggtcactgcaacaccaagtgtcaaactccaataggggcgataaactctagtatgccattccacaacatacaccctctcactatcggggaatgccccaaatatgtgaaatcaaacaaattagtccttgcgactgggctcagaaataatcctctaagagaaaaGGGaagaagaaaaaga

>H5N1_A/HK/212/03

taccatgcaaacaactcgacagagcaggttgacacaataatggaaaagaacgttactgttacacatgcccaagacatactggaaaagacacacaacgggaagctctgcgatctagatggagtgaagcctctaattttgagagattgtagtgtagctggatggctcctcggaaacccaatgtgtgacgaattcatcaatgtgccggaatggtcttacatagtggagaaggccaatccagccaatgacctctgttacccaggggatttcaacgactatgaagaattgaaacacctattgagcagaataaaccattttgagaaaattcagatcatccccaaaaattcttggtccagtcatgaagcctcattaggggtgagctcagcatgtccataccaaggaaagtcctcctttttcaggaatgtggtatggcttatcaaaaagaacaatgcatacccaacaataaagaggagctacaataataccaaccaagaagatcttttggtattgtgggggattcaccatcctaatgatgcggcagagcagactaggctctatcaaaacccaaccacctacatttccgttgggacatcaacactaaaccagagattggtaccaaaaatagctactagatccaaagtaaacgggcaaaatggaaggatggagttcttctggacaattttaaaaccgaatgatgcaatcaacttcgagagcaatggaaatttcattgctccagaatatgcatacaaaattgtcaagaaaggggactcagcaattatgaaaagtgaattggaatatggtaactgcaacaccaagtgtcaaactccaatgggggcgataaactctagtatgccattccacaatatacaccctctcaccatcggggaatgccccaaatatgtgaaatcaaacagattagtccttgcgactgggctcagaaatagccctcaaagagagagaagaagaaaaaagaga

>H5N1_A/Hanoi/30408/2005

taccatgcaaacaactcgacagagcaggttgacacaataatggaaaagaacgttactgttacacatgcccaagacatactggaaaagacacacaacggaaagctctgcgatctagatggagtgaagcctctaattttgagagattgtagtgtagctggatggctcctcggaaacccaatgtgtgacgaattcatcaatgtgccggaatggtcttacatagtggagaaggccaatccagtcaatgatctctgttacccaggggatttcaatgactatgaagaattgaaacacttattgagcagaataaaccattttgagaaaattcagatcatccccaaaagttcttggctcagtcatgaagcctcattaggggtgagctcagcatgtccataccagggaaagtcctcctttttcagaaatgtggtatggcttatcaaaaagaacagtacatacccaacaataaagaggagctacaataacaccaaccaagaagatctgttggtactgtgggggattcaccatcctaatgatgcggcagagcagacaaagctctatcaaaacccaaccacctatatttccgttgggacatcaacactaaaccagagattggtaccaagaatagctactagatccaaagtaaacgggcaaagtggaaggatggagttcttctggacaattttaaaaccgaatgatgcaatcaatttcgagagtaatggaaatttcattgccccagaatatgcatacaaaattgtcaagaaaggggactcaacaattatgaaaagtgaattggaatatggtaactgcaacaccaagtgtcaaacaccaatgggggcgataaattctagtatgccattccacaatatacaccctctcaccatcggggaatgccccaaatatgtgaaatcaaacagattagtccttgcgactgggctcagaaatagccctcaaagagagagGGGaagaaaaaagaga

>H5N1_A/Hong_Kong/156/97

taccatgcaaacaactcgacagagcaggttgacacaataatggaaaagaatgttactgttacacatgcccaagacatactggaaaggacacacaacgggaagctctgcgatctaaatggagtgaagcctctcattttgagggattgtagtgtagctggatggctcctcggaaaccctatgtgtgacgaattcatcaatgtgccggaatggtcttacatagtggagaaggccagtccagccaatgacctctgttatccagggaatttcaacgactatgaagaactgaaacacctattgagcagaataaaccattttgagaaaattcagatcatccccaaaagttcttggtccaatcatgatgcctcatcaggggtgagctcagcatgtccataccttgggaggtcctcctttttcagaaatgtggtatggcttatcaaaaagaacagtgcatacccaacaataaagaggagctacaataataccaaccaagaagatcttttggtactgtggggggttcaccatcctaatgatgcggcagagcagacaaagctctatcaaaatccaaccacctacatttccgttggaacatcaacactgaaccagagattggttccagaaatagctactagacccaaagtaaacgggcaaagtggaagaatggagttcttctggacaattttaaagccgaatgatgccatcaatttcgagagtaatggaaatttcattgctccagaatatgcatacaaaattgtcaagaaaggggactcaacaattatgaaaagtgaattggaatatggtaactgcaacaccaagtgtcaaactccaatgggggcgataaactctagtatgccattccacaacatacaccccctcaccatcggggaatgccccaaatatgtgaaatcaaacagattagtccttgcgactggactcagaaatacccctcaaagagagagaagaagaaaaaagaga

>H5N1_A/Hong_Kong/213/03

taccatgcaaacaactcgacagagcaggttgacacaataatggaaaagaacgttactgttacacatgcccaagacatactggaaaagacacacaacgggaagctctgcgatctagatggagtgaagcctctaattttgagagattgtagtgtagctggatggctcctcggaaacccaatgtgtgacgaattcatcaatgtgccggaatggtcttacatagtggagaaggccaatccagccaatgacctctgttacccaggggatttcaacgactatgaagaattgaaacacctattgagcagaataaaccattttgagaaaattcagatcatccccaaaaattcttggtccagtcatgaagcctcattaggggtgagctcagcatgtccataccaaggaaagtcctcctttttcaggaatgtggtatggcttatcaaaaagaacaatgcatacccaacaataaagaggagctacaataataccaaccaagaagatcttttggtattgtgggggattcaccatcctaatgatgcggcagagcagactaggctctatcaaaacccaaccacctacatttccgttgggacatcaacactaaaccagagattggtaccaaaaatagctactagatccaaagtaaacgggcaaaatggaaggatggagttcttctggacaattttaaaaccgaatgatgcaatcaacttcgagagcaatggaaatttcattgctccagaatatgcatacaaaattgtcaagaaaggggactcagcaattatgaaaagtgaattggaatatggtaactgcaacaccaagtgtcaaactccaatgggggcgataaactctagtatgccattccacaatatacaccctctcaccatcggggaatgccccaaatatgtgaaatcaaacagattagtccttgcgactgggctcagaaatagccctcaaagagagagaagaagaaaaaagaga

>H5N1_A/Hong_Kong/213/2003

taccatgcaaacaactcgacagagcaggttgacacaataatggaaaagaacgttactgttacacatgcccaagacatactggaaaagacacacaacgggaagctctgcgatctagatggagtgaagcctctaattttgagagattgtagtgtagctggatggctcctcggaaacccaatgtgtgacgaattcatcaatgtgccggaatggtcttacatagtggagaaggccaatccagccaatgacctctgttacccaggggatttcaacgactatgaagaattgaaacacctattgagcagaataaaccattttgagaaaattcagatcatccccaaaaattcttggtccagtcatgaagcctcattaggggtgagctcagcatgtccataccaaggaaagtcctcctttttcaggaatgtggtatggcttatcaaaaagaacaatgcatacccaacaataaagaggagctacaataataccaaccaagaagatcttttggtattgtgggggattcaccatcctaatgatgcggcagagcagactaggctctatcaaaacccaaccacctacatttccgttgggacatcaacactaaaccagagattggtaccaaaaatagctactagatccaaagtaaacgggcaaaatggaaggatggagttcttctggacaattttaaaaccgaatgatgcaatcaacttcgagagcaatggaaatttcattgctccagaatatgcatacaaaattgtcaagaaaggggactcagcaattatgaaaagtgaattggaatatggtaactgcaacaccaagtgtcaaactccaatgggggcgataaactctagtatgccattccacaatatacaccctctcaccatcggggaatgccccaaatatgtgaaatcaaacagattagtccttgcgactgggctcagaaatagccctcaaagagagagaagaagaaaaaagaga

>H5N1_A/Hong_Kong/378.1/2001

taccatgcaaacaactcgacagagctggttgacacaataatggaaaagaacgttactgttacacatgcccaagacatactggaaaagacacacaacgggaagctctgcgatctagatggagtgaagcctctaattttgagagattgtagtgtggctggatggctcctcggaaacccaatgtgtgacgaattcatcaatgtgccggaatggtcttacatagtggagaaggccaatccagccaatgacctctgttacccaggggatttcaacgactatgaagaactgaaacacctattgagcagaataaaccattttgagaaaattcagatcatccccaaaagttcttggcccaatcatgaagcctcatcaggggtgagctcagcatgtccataccaggggaagtcctcctttttcagaaatgtggtatggcttatcaaaaagaacagtgcatacccaacaataaagaggagctacaataataccaaccaagaagatcttttggtactgtgggggattcaccatcctaatgatgcggcagagcagacaaagctctatcaaaacccaaccacctatatttccgttggaacatcaacactaaaccagagattggtaccaaaaatagctactagatccaaagtaaacggacaaagtggaagaatggagttcttctggacaattttaaagccgaatgatgctatcaatttcgagagtaatggaaatttcattgctccagaatatgcatacaaaattgtcaagaaaggggactcagcaattatgaaaagtgaattggaatatggtaactgcaacaccaagtgtcaaactccactgggggcgataaactctagtatgccattccacaacatacaccctctcaccatcggggaatgccccaaatatgtgaagtcaaacagattagtccttgcgactggactcagaaatacccctcaaagagagagaagaagaaaaaagaga

>H5N1_A/Hong_Kong/481/97

taccatgcaaacaactcgacagagcaggttgacacaataatggaaaagaatgttactgttacacatgcccaagacatactggaaaggacacacaacgggaagctctgcgatctaaatggagtgaagcctctcattttgagggattgtagtgtagctggatggstcctcggaaaccctatgtgtgacgaattcctcaatgtgccggaatggtcttacatagtagagaagaccagtccagccaatgacctctgttatccagggcatttcaacgactatgaagaactgaaacacctattgagcagaataaaccattttgagaaaattcagatcatccccaaaagttcttggtccaatcatgatgcctcatcaggggtgagctcagcatgtccataccttgggaggtcctcctttttcagaaatgtggtatggcttatcaaaaagaacagtgcatacccaacaataaagaggagctacaataataccaaccaagaagatcttttggtactgtgggggattcaccatcctaatgatgcggcagagcagataaagctctatcaaaatccaacctcctacatttccgttggaacatcaacactgaaccagagattggttccagaaatagctactagacccaaagtaaacgggcaaagtggaagaatggagttcttctggacaattttaaagccgaatgatgccatcaatttcgagagtaatggaaatttcattgctccagaatatgcatacaaaattgtcaagaaaggggactcaacaattatgaaaagtgaattggaatatggtaactgcaacaccaagtgtcaaactccaatgggggcgataaactctagtatgccattccacaacatacaccccctcaccatcggggaatgccccaaatatgtgaaatcaaacagattagtccttgcgactggactcagaaatacccctcaaagagagagaaggagaaaaaagaga

>H5N1_A/Hong_Kong/482/97

taccatgcaaacaactcgacagagcaggttgacacaataatggaaaagaatgttactgttacacatgcccaagacatactggaaaggacacacaacgggaagctctgcgatctaaatggagtgaaacctctcattttgagggattgtagtgtagctggatggctcctcggaaaccctatgtgtgacgaattcatcaatgtgccggaatggtcttacatagtggagaaggccagtccagccaatgacctctgttatccagggaatttcaacgactatgaagaactgaaacacctattgagcagaataaaccattttgagaaaattcagatcatccccaaaagttcttggtccaatcatgatgcctcatcaggggtgagctcagcatgtccataccttgggaggtcctcctttttcagaaatgtggtatggcttatcaaaaagaacagtgcatacccaacaataaagaggagctacaataataccaaccaagaagatcttttggtactgtgggggattcaccatcctaatgatgcggcagagcagacaaagctctatcaaaatccaaccacctacatttccgttggaacatcaacactgaaccagagattggttccagaaatagctactagacccaaagtaaacgggcaaagtggaagaatggagttcttctggacaattttaaagccgaatgatgccatcaatttcgagagtaatggaaatttcattgccccagaatatgcatacaaaattgtcaagaaaggggactcaacaattatgaaaagtgaattggaatatggtaactgcaacaccaagtgtcaaactccaatgggggcgataaactctagtatgccattccacaacatacaccccctcaccatcggggaatgccccaaatatgtgaaatcaaacagattagttcttgcgactggactcagaaatacccctcaaagggagagaagaagaaaaaagaga

>H5N1_A/Hong_Kong/483/1997

taccatgcaaacaactcgacagagcaggttgacacaataatggaaaagaatgttactgttacacatgcccaagacatactggaaaggacacacaacgggaagctctgcgatctaaatggagtgaagcctctgattttgagggattgtagtgtagctggatggctcctcggaaaccctatgtgtgacgaattcatcaatgtgccggaatggtcttacatagtggagaaggccagtccagccaatgacctctgttatccagggaatttcaacgactatgaagaactgaaacacctattgagcagaataagccattttgagaaaattcagatcatccccaaaagttcttggtccaatcatgatgcctcatcaggggtaagctcagcatgtccataccttgggaagtcctcctttttcagaaatgtggtatggcttatcaaaaagaacagtacatacccaacaataaagaggagctacaataataccaaccaagaagatcttttggtactgtgggggattcaccatcctaatgatgcggcagagcagacaaagctctatcaaaacccaaccacctacatttccgttggaacatcaacactgaaccagagattggttccagaaatagctactagacccaaagtaaacgggcaaagtggaagaatagagttcttctggacaattttaaagccgaatgatgccatcaatttcgagagtaatggaaatttcattgctccagaatatgcatacaaaattgtcaagaaaggggactcaacaattatgaaaagtgaattggaatatggtaactgcaacaccaagtgtcaaactccaatgggggcgataaactctagtatgccattccacaacatacaccccctcaccatcggggaatgccccaaatatgtgaaatcaaacagattagtccttgcgactggactcagaaatgcccctcaaagagagagaagaagaaaaaagaga

>H5N1_A/Hong_Kong/485/1997

taccatgcaaacaactcgacagagcaagttgacacaataatggaaaagaatgttactgttacacatgcccaagacatactggaaaggacacacaacgggaagctctgcgatctaaatggagtgaagcctctgattttgagggattgtagtgtagctggatggctcctcggaaaccctatgtgtgacgaattcatcaatgtgcctgaatggtcttacatagtggagaaggccagtccagccaatgacctctgttatccagggaatttcaacgactatgaagaactgaaacacctattgagcagaataaaccattttgagaaaattcagataatccccaaaagttcttggtccaatcatgatgcctcatcaggggtgagctcagcatgtccataccttgggaggtcctcctttttcagaaatgtggtatggcttatcaaaaagaacagttcatacccaacaataaagaggagctacaataataccaaccaagaagatcttttggtactgtgggggattcaccatcctaatgatgcggcagagcagacaaggctctatcaaaacccaaccacctacatttccgttggaacatcaacactgaaccagagattggttccagaaatagctactagacccaaagtaaacgggcaaagtggaagaatggagttcttctggacaattttaaagccgaatgatgccatcaatttcgagagtaatggaaatttcattgctccagaatatgcatacaaaattgtcaagaaaggggactcaacaattatgaaaagtgaattggaatatggtaactgcaacaccaagtgtcaaactccaatgggggcaataaactctagtatgccattccacaacatacaccccctcaccatcggggaatgccccaaatatgtgaaatcaaacagattagtccttgcgactggactcagaaatacccctcaaagagagagaagaagaaaaaagaga

>H5N1_A/Hong_Kong/485/97

taccatgcaaacaactcgacagagcaagttgacacaataatggaaaagaatgttactgttacacatgcccaagacatactggaaaggacacacaacgggaagctctgcgatctaaatggagtgaagcctctgattttgagggattgtagtgtagctggatggctcctcggaaaccctatgtgtgacgaattcatcaatgtgcctgaatggtcttacatagtggagaaggccagtccagccaatgacctctgttatccagggaatttcaacgactatgaagaactgaaacacctattgagcagaataaaccattttgagaaaattcagataatccccaaaagttcttggtccaatcatgatgcctcatcaggggtgagctcagcatgtccataccttgggaggtcctcctttttcagaaatgtggtatggcttatcaaaaagaacagttcatacccaacaataaagaggagctacaataataccaaccaagaagatcttttggtactgtgggggattcaccatcctaatgatgcggcagagcagacaaggctctatcaaaacccaaccacctacatttccgttggaacatcaacactgaaccagagattggttccagaaatagctactagacccaaagtaaacgggcaaagtggaagaatggagttcttctggacaattttaaagccgaatgatgccatcaatttcgagagtaatggaaatttcattgctccagaatatgcatacaaaattgtcaagaaaggggactcaacaattatgaaaagtgaattggaatatggtaactgcaacaccaagtgtcaaactccaatgggggcaataaactctagtatgccattccacaacatacaccccctcaccatcggggaatgccccaaatatgtgaaatcaaacagattagtccttgcgactggactcagaaatacccctcaaagagagagaagaagaaaaaagaga

>H5N1_A/Hong_Kong/486/97

taccatgcaaacaactcgacagagcaggttgacacaataatggaaaagaatgttactgttacacatgcccaagacatactggaaaggacacacaacgggaagctctgcgatctaaatggagtgaaacctctcattttgagggattgtagtgtagctggatggctcctcggaaaccctatgtgtgacgaattcatcaatgtgccggaatggtcttacatagtggagaaggccagtccagccaatgacctctgttatccagggaatttcaacgactatgaagaactgaaacacctattgagcagaataaaccattttgagaaaattcagatcatccccaaaagttcttggtccaatcatgatgcctcatcaggggtgagctcagcatgtccataccttgggaggtcctcctttttcagaaatgtggtatggcttatcaaaaagaacagtgcatacccaacaataaagaggagctacaataataccaaccaagaagatcttttggtactgtgggggattcaccatcctaatgatgcggcagagcagacaaagctctatcaaaatccaaccacctacatttccgttggaacatcaacactgaaccagagattggttccagaaatagctactagacccaaagtaaacgggcaaagtggaagaatggagttcttctggacaattttaaagccgaatgatgccatcaatttcgagagtaatggaaatttcattgccccagaatatgcatacaaaattgtcaagaaaggggactcaacaattatgaaaagtgaattggaatatggtaactgcaacaccaagtgtcaaactccaatgggggcgataaactctagtatgccattccacaacatacaccccctcaccatcggggaatgccccaaatatgtgaaatcaaacagattagttcttgcgactggactcagaaatacccctcaaagggagagaagaagaaaaaagaga

>H5N1_A/Hong_Kong/488/97

taccatgcaaacaactcgacagagcaggttgacacaataatggaaaagaatgttactgttacacatgcccaagacatactggaaaggacacacaacgggaagctctgcgatctaaatggagtgaaacctctgattttgagggattgtagtgtagctggatggctcctcggaaaccctatgtgtgacgaattcatcaatgtgccggaatggtcttacatagtggagaaggccagtccagccaatgacctctgttatccagggaatttcaacgactatgaagaactgaaacacctattgagcagaataaaccattttgagaaaattcagatcatccccaaaagttcttggtccaatcatgatgcctcatcaggggtgagctcagcatgtccataccttgggaggtcctcctttttcagaaatgtggtatggcttatcaaaaagaacagtgcatacccaacaataaagaggagctacaataataccaaccaagaagatcttttggtactgtgggggattcaccatcctaatgatgcggcagagcagacaaagctctatcaaaatccaaccacctacatttccgttggaacatcaacactgaaccagagattggttccagaaatagctactagacccaaagtaaacgggcaaagtggaagaatggagttcttctggacaattttaaagccgaatgatgccatcaatttcgagagtaatggaaatttcattgccccagaatatgcatacaaaattgtcaagaaaggggactcaacaattatgaaaagtgaattggaatatggtaactgcaacaccaagtgtcaaactccaatgggggcgataaactctagtatgccattccacaacatacaccccctcaccatcggggaatgccccaaatatgtgaaatcaaacagattagttcttgcgactggactcagaaatacccctcaaagggagagaagaagaaaaaagaga

>H5N1_A/Hong_Kong/491/97

taccatgcaaacaactcgacagagcaggttgacacaataatggaaaagaatgttactgttacacatgcccaagacatactggaaaggacacacaacgggaagctctgcgatctaaatggagtgaagcctctcattttgagggattgtagtgtagctggatggctcctcggaaaccctatgtgtgacgaattcatcaatgtgccggaatggtcttacatagtggagaaggccagtccagccaatgacctctgttatccagggaatttcaacgactatgaagaactgaaacacctattgagcagaataaaccattttgagaaaattcagatcatccccaaaagttcttggtccaatcatgatgcctcatcaggggtgagctcagcatgtccataccttgggaggtcctcctttttcagaaacgtggtatggcttatcaaaaagaacagttcatacccaacaataaagaggagctacaataataccaaccaagaagatcttttggtactgtgggggattcaccatcctaatgatgcggcagagcagacaaagctctatcaaaacccaaccacctacatttccgttggaacatcaacactgaaccagagattggtcccagaaatagctactagacccaaagtaaacgggcaaagtggaagaatggagttcttctggacaattttaaagccgaatgatgccatcaatttcgagagtaatggaaatttcattgctccagaatatgcatacaaaattgtcaagaaaggggactcaacaattatgaaaagtgaattggaatatggtaactgcaacaccaagtgtcaaactccaatgggggcgataaactctagtatgccattccacaacatacaccccctcaccatcggggaatgccccaaatatgtgaaatcaaacagattagtccttgcgactggactcagaaatacccctcaaagagagagaagaagaaaaaagaga

>H5N1_A/Hong_Kong/503/97

taccatgcaaacaactcgacagagcaggttgacacaataatggaaaagaatgttactgttacacatgcccaagacatactggaaaggacacacaacgggaagctctgcgatctaaatggagtgaagcctctcattttgagggattgtagtgtagctggatggctcctcggaaaccccatgtgtgacgagttcatcaatgtgccggaatggtcttacatagtggaaaaggccagtccagccaatgacctctgttatccagggaatttcaacgactatgaagaactgaaacacctattgagcagaataaaccattttgagaaaattcagatcatccccaaaagttcttggtccaatcatgatgcctcatcaggggtgagctcagcatgtccataccttgggaggtcctcctttttcagaaacgtggtatggcttatcaaaaagaacagttcatacccaacaataaagaggagctacaataataccaaccaagaagatcttttggtactgtgggggattcaccatcctaatgatgcggcagagcagacaaagctctatcaaaacccaaccacctacatttccgttggaacatcaacactgaaccagagattggtcccagaaatagctactagacccaaagtaaacggccaaagtggaagaatggagttcttctggacaattttaaagccgaatgatgccatcaatttcgagagtaatggaaatttcattgctccagaatatgcatacaaaattgtcaagaaaggggactcaacaattatgaaaagtgaattggaatatggtaactgcaacaccaagtgtcaaactccaatgggggcgataaactctagtatgccattccacaacatacaccccctcaccatcggggaatgccccaaatatgtgaaatcaaacagattagtccttgcgactggactcagaaatacccctcaaagagagagaagaagaaaaaagaga

>H5N1_A/Hong_Kong/507/97

taccatgcaaacaactcgacagagcaggttgacacaataatggaaaagaatgttactgttacacatgcccaagacatactggaaaggacacacaacgggaagctctgcgatctaaatggagtgaaacctctcattttgagggattgtagtgtagctggatggctcctcggaaaccccatgtgtgacgaattcgtcaatgtgccggaatggtcttacatagtggagaaggccagtccagccaatgacctctgttatccagggaatttcaacgactatgaagaactgaaacacctattgagcagaataaaccattttgagaaaattcagatcatccccaaaagttcttggtccaatcatgatgcctcatcaggggtgagctcagcatgttcataccttgggaggtcctcctttttcagaaatgtggtatggcttatcaaaaagaacagtgcatacccgacaataaagaggagctacaataataccaaccaagaagatcttttggtactgtgggggattcaccatcctaatgatgcggcagagcagacaaagctctatcaaaatccaaccacctacatttccgttggaacatcaacactgaaccagagattggttccagaaatagctactagacccaaagtaaacgggcaaagtggaagaatggagttcttctggacaattttaaagccgaatgatgccatcaatttcgagagtaatggaaatttcattgccccagaatatgcatacaaaattgtcaagaaaggggactcaacaattatgaaaagtgaattggaatatggtaactgcaacaccaagtgtcaaactccaatgggggcgataaactctagtatgccattccacaacatacaccccctcaccatcggggaatgccccaaatatgtgaaatcaaacagattagttcttgcgactggactcagaaatacccctcaaagggagagaagaagaaaaaagaga

>H5N1_A/Hong_Kong/514/97

taccatgcaaacaactcaacagagcaagttgacacaataatggaaaagaatgttactgttacacatgctcaagacatactggaaaggacacacaacgggaagctctgcgatctaaatggagtgaagcctctgattttgagagattgtagtgtagctggatggctcctcggaaaccctatgtgtgacgaattcatcaatgtgccggaatggtcttacatagtggagaaggccagtccagccaatgacctctgttatccagggaacttcaacgactatgaagaactgaaacacctattgagcagaataaaccattttgagaaaattcagatgatccccaaaagttcttggtccaatcatgatgcctcatcagggttgagctcggcatgtccataccttgggaagtcctcctttttcagaaatgtggtatggcttatcaaaaagaacagttcatacccaacaataaagaggagctacaataataccaaccaagaagatcttttggtactgtgggggattcaccatcctaatgatgcggcagagcagacaaagctctatcaaaacccaaccacctacatttccgttggaacatcaacactgaaccagagattggttccagaaatagctactagacccaaagtaaacgggcaaagtggaagaatggagttcttctggacaattttaaagccgaatgatgccatcaatttcgagagtaatggaaatttcattgctccagaatatgcatacaaaattgtcaagaaaggggactcaacaattatgaaaagtgaattggaatatggtaactgtaacaccaagtgtcaaactccaatgggggcgataaactctagtatgccattccacaacatacaccccctcaccatcggggaatgccccaaatatgtgaaatcaaacagattagtccttgcgactggactcagaaatacccctcatagagagagaagaagaaaaaagaga

>H5N1_A/Hong_Kong/516/97

taccatgcaaacaactcgacagagcaggttgacacaataatggaaaagaatgttactgttacacatgcccaagacatactggaaaggacacacaacgggaagctctgcgatctaaatggagtgaaacctctcattttgagggattgtagtgtagctggatggctcctcggaaaccctatgtgtgacgaattcatcaatgtgccggaatggtcttacatagtggagaaggccagtccagccaatgacctctgttatccagggaatttcaacgactatgaagaactgaaacacctattgagcagaataaaccattttgagaaaattcagatcatccccaaaagttcttggtccaatcatgatgcctcatcaggggtgagctcagcatgtccataccttgggaggtcctcctttttcagaaatgtggtatggcttatcaaaaagaacagtgcatacccaacaataaagaggagctacaataataccaaccaagaagatcttttggtactgtgggggattcaccatcctaatgatgcggcagagcagacaaagctctatcaaaatccaaccacctacatttccgttggaacatcaacactgaaccagagattggttccagaaatagctactagacccaaagtaaacgggcaaagtggaagaatggagttcttctggacaattttaaagccgaatgatgccatcaatttcgagagtaatggaaatttcattgccccagaatatgcatacaaaattgtcaagaaaggggactcaacaattatgaaaagtgaattggaatatggtaactgcaacaccaagtgtcaaactccaatgggggcgataaactctagtatgccattccacaacatacaccccctcaccatcggggaatgccccaaatatgtgaaatcaaacagattagttcttgcgactggactcagaaatacccctcaaagggagagaagaagaaaaaagaga

>H5N1_A/Hong_Kong/532/1997

taccatgcaaacaactcgacagagcaggttgacacaataatggaaaagaatgttactgttacacatgcccaagacatactggaaaggacacacaacgggaagctatgcgatctaaatggagtgaagcctctcattttgagggattgtagtgtagctggatggctcctcggaaaccctatgtgtgacgaattcatcaatgtgccggaatggtcttacatagtggagaaggccagtccagccaatgacctctgttatccagggaatttcaacgactatgaagaactgaaacacctattgagcagaataaaccattttgagaaaattcagatcatccccaaaagttcttggtccaatcatgatgcctcatcaggggtgagctcagcatgtccataccttgggaggtcctcctttttcagaaatgtggtatggcttatcaaaaagaacagtacatacccaacaataaagaggagctacaataataccaaccaagaagatcttttggtactgtgggggattcaccatcctaatgatgcggcagagcagacaaagctctatcaaaacccaacaacctacatttccgttggaacatcaacactgaaccagagattggttccagaaatagctactagacccaaagtaaacgggcaaagtggaagaatggagttcttctggacaattttaaagccgaatgacgccatcaatttcgagagtaatggaaatttcattgctccagaatatgcatacaaaattgtcaagaaaggggactcaacaattatgaaaagtgaattggaatatggtaactgcaacaccaagtgtcaaactccaatgggggcgataaactctagtatgccattccacaacatacaccccctcaccatcggggaatgccccaaatatgtgaaatcaaacagattagtccttgcgactggactcagaaatacccctcaaagagagagaagaagaaaaaagaga

>H5N1_A/Hong_Kong/538/97

taccatgcaaacaactcgacagagcaggttgacacaataatggaaaagaatgttactgttacacatgcccaagacatactggaaaggacacacaacgggaagctctgcgatctaaatggagtgaaacctctcattttgagggattgtagtgtagctggatggctcctcggaaaccctatgtgtgacgaattcatcaatgtgccggaatggtcttacatagtggagaaggccagtccagccaatgacctctgttatccagggaatttcaacgactatgaagaactgaaacacctattgagcagaataaaccattttgagaaaattcagatcatccccaaaagttcttggtccaatcatgatgcctcatcaggggtgagctcagcatgtccataccttgggaggtcctcctttttcagaaatgtggtatggcttatcaaaaagaacagtgcatacccaacaataaagaggagctacaataataccaaccaagaagatcttttggtactgtgggggattcaccatcctaatgatgcggcagagcagacaaagctctatcaaaatccaaccacctacatttccgttggaacatcaacactgaaccagagattggttccagaaatagctactagacccaaagtaaacgggcaaagtggaagaatggagttcttctggacaattttaaagccgaatgatgccatcaatttcgagagtaatggaaatttcattgccccagaatatgcatacaaaattgtcaagaaaggggactcaacaattatgaaaagtgaattggaatatggtaactgcaacaccaagtgtcaaactccaatgggggcgataaactctagtatgccattccacaacatacaccccctcaccatcggggaatgccccaaatatgtgaaatcaaacagattagttcttgcgactggactcagaaatacccctcaaagggagagaagaagaaaaaagaga

>H5N1_A/Hong_Kong/542/97

taccatgcaaacaactcgacagagcaggttgacacaataatggaaaagaatgttactgttacacatgcccaagacatactggaaaggacacacaacgggaagctctgcgatctaaatggagtgaagcctctcattttgagggattgtagtgtagctggatggctcctcggaaaccctatgtgtgacgaattcatcaatgtgccggaatggtcttacatagtggagaaggccagtccagccaatgacctctgttatccagggaatttcaacgactatgaagaactgaaacacctattgagcagaataaaccattttgagaaaattcagatcatccccaaaagttcttggtccaatcatgatgcctcatcaggggtgagctcagcatgtccataccttgggaggtcctcctttttcagaaacgtggtatggcttatcaaaaagaacagttcatacccaacaataaagaggagctacaataataccaaccaagaagatcttttggtactgtgggggattcaccatcctaatgatgcggcagagcagacaaagctctatcaaaacccaaccacctacatttccgttggaacatcaacactgaaccagagattggtcccagaaatagctactagacccaaagtaaacggccaaagtggaagaatggagttcttctggacaattttaaagccgaatgatgccatcaatttcgagagtaatggaaatttcattgctccagaatatgcatacaaaattgtcaagaaaggggactcaacaattatgaaaagtgaattggaatatggtaactgcaacaccaagtgtcaaactccaatgggggcgataaactctagtatgccattccacaacatacaccccctcaccatcggggaatgccccaaatatgtgaaatcaaacagattagtccttgcgactggactcagaaatacccctcaaagagagagaagaagaaaaaagaga

>H5N1_A/Hong_Kong/5923/2012

taccatgcaaacaactcgacagagcaggttgacacaataatggaaaagaacgttactgttacacatgctcaagacatactggagaagacgcacaatgggaagctctgcgatctaaatggagtgaagcctctgattttaaaagattgtagtgtagcaggatggctcctcgggaacccaatgtgtgacgaattcatcgatgtgccagaatggtcttacatagtagagaagaccaatccagccaatgacctctgttacccagggagtttcaacgattatgaagaattaaaacacctattgagcagaataaaccactttgagaaaatacgaatcattcccaaagattcttggccagatcatgaagcctcattgggggtgagcgcagcatgttcataccagggaaattcctcatttttcagaaatgtggtatggcttctcaaaaaggacgatgcatacccaataataacgaaaagctacaataataccaataaagaagatctcttggtactgtgggggatccatcatcctaatgatgaagcagagcagataaggctctataaaaacccaaccacctatgtctccattgggacatcaacactaaaccagagattggtaccaagaatagctactagatccaaagtaaacgggcaacgtggaaggatagatttcttctggacaattttaaaaccgaatgatgcaatcaacttcgagagtaatggaaatttcattgctccagaatatgcatacaaaattgtcaagaaaggagactcaacaattatgagaagtgaagtggaatatggtaactgcagcaccaggtgtcagactccgatgggggcgataaattctagtatgccattccacaacatacaccctctcaccatcggagaatgccccaaatatgtgaaatcaaacaaattagtccttgcgactgggctcagaaatagtcctcaaatagagagaagaagaagaaaaagg

>H5N1_A/Hong_Kong/97/98

taccatgcaaacaactcgacagagcaggttgatacaataatggaaaagaatgttactgttacacatgcccaagacatactggaaaggacacacgacgggaagctctgcgatctaaatggagtgaaacctctcattttgagggattgtagtgtagctggatggctcctcggaaaccctatgtgtgacgaattcatcaatgtgccggaatggtcttacatagtggagaaggccagtccagccaatgacctctgttatccagggaatttcaacgactatgaagaactgaaacacctattgagcagaataaaccattttgagaaaattcagatcatccccaaaagttcttggtccaatcatgatgcctcatcaggggtgagctcagcatgtccataccttgggaggtcctcctttttcagaaatgtggtatggcttatcaaaaagaacagtgcatacccaacaataaagaggagctacaataataccaaccaagaagatcttttggtactgtgggggattcaccatcctaatgatgcggcagagcagacaaagctctatcaaaatccaaccacctacatttccgttggaacatcaacactgaatcagagattggttccagaaatagctactagacccaaagtaaacgggcaaagtggaagaatggagttcttctggacaattttaaagccgaatgatgccatcaatttcgagagtaatggaaatttcattgccccagaatatgcatacaaaattgtcaagaaaggggactcaacaattatgaaaagtgaattggaatatggtaactgcaacaccaagtgtcaaactccaatgggggcgataaactctagtatgccattccacaacatacaccccctcaccatcggggaatgccccaaatatgtgaaatcaaacagattagttcttgcgactggactcagaaatacccctcatagggagagaagaagaaaaaagaga

>H5N1_A/Hubei/1/2006

taccatgcaaacaactcgacagagcaggttgacacaataatggaaaagaacgttactgttacacatgcccaagacatactggaaaagacacacaacgggaagctctgcgatctagatggagtgaagcctctgattttaagagattgtagtgtagctggatggctccttggaaacccaatgtgtgacgaattcatcaatgtgccagaatggtcttacatagtggagaaggccaacccagccaatgacctctgttacccagggaatttcaacgactatgaagaactaaaacacttattgagcagaataaaccattttgagaaaattcagatcatccccaaaagttcttggtcagatcatgaagcctcatcaggggtgagctcagtatgtccataccagggaacgccctcctttttcagaaatgtggtatggcttatcaaaaagaacaatacatatccaacaataaagagaagctacaataataccaaccaggaagatcttttgatactgtggggaattcaccattctaatgatgcggcagagcagacaaagctctatcaaaacccaaccacctatatttccgttgggacatcaacactaaaccagagattggtaccaaaaatagctactagatccaaagtaaacgggcaaagtggaaggatggatttcttctggacagttttaaaaccgaatgatgcaatcaacttcgagagtaatggaaatttcattgctccagaatatgcatacaaaattgtcaagaaaggggactcagcaattatgaaaagtgaagtggaatatggtaactgcaacaccaagtgtcaaactccaataggggcgataaactctagtatgccattccacaacatacaccctctcaccatcggggaatgccccaaatatgtgaaatcaaacaaattagtccttgcgactgggctcagaaatagtcctctaagagaaagGGGaagaagaaaaaga

>H5N1_A/Hubei/1/2010

tatcatgcaaataactcgacagagcaggttgacacaataatggaaaagaacgtgactgttacacatgcccaagacatactggaaaagacacacaacggtaagctctgcgatctaaatggagtgaagcctctgattttaaaagattgtagtgtagcaggatggctcctcggaaatccaatgtgtgacgaattcatcaatgtgccagaatggtcttacatagtagagaaggccaatccagccaatgacctctgttacccagggaatttcaacgattatgaagaattgaaacacctattgagcaggataaaccattttgagaaaatacagatcatccccaaaaattcttggtcagatcatgaagcctcgttgggggtgagtgcagcatgtccataccagggaaaatcctccttcttcagaaatgtggtatggcttatcaaaaaggacaatgcatacccaacaataaagaaaggctacaataataccaaccaagaagatctcttggtactgtgggggattcaccatcctaatgatgaggcagagcagacaaggctctatcaaaacccaaccacctatatttccattgggacatcaacactaaaccagagattggtaccaaaaatagccactaggtccaaaataaacgggcaaagtggcaggatagatttcttctggacaattttaaaaccgaatgatgcaatccacttcgagagtaatggaaatttcattgctccagaatatgcatacaaaattgtcaagaaaggagactcaacaattatgaaaagtgaagtggaatatggtaactgcaacaccaggtgtcagactccaataggggcgataaactccagtatgccattccacaacatacaccctctcaccatcggagaatgtcccaaatatgtgaaatcaaacaaactagtccttgcgactgggctcagaaatagtcctcaaagagagagGGGaaggagaaaaaga

>H5N1_A/Hunan/1/2006

taccatgcaaacaactcgacagagcaggttgacacaataatggaaaagaacgttactgttacacatgcccaagatatactggaaaagacacacaacgggaagctctgcgatctagatggagtgaagcctctgattttaggagattgtagtgtagctggatggctcctcggaaacccgatgtgtgacgaattcatcaatgtgccggaatggtcttacatagtggagaaggccaacccagccaatgacctctgttacccagggaatttcaacgactatgaagaactgaaacacctattgagcagaataaaccattttgagaaaattcagatcatccccaaaagttcttggtccgatcatgaagcctcatcaggggtgagctcagcatgtccataccagggaacgccctcctttttcagaaatgtggtatggcttatcaaaaagaacaatacatacccaacaataaagagaagctacaataataccaaccaggaagatcttttgatactgtgggggattcatcattctaatgatgcggcagagcagacaaagctctatcaaaacccaaccacctatatttccgttgggacatcaacactaaaccagagattggtaccaaaaatagctactagatccaaagtaaacgggcaaagtggaaggatggatttcttctggacaattttaaaaccgaatgatgcaatcaacttcgagagtaatggaaatttcattgctccagaatatgcatacaaaattgtcaagaaaggggactcagcaattatgaaaagtgaagtggaatatggtaactgcaacaccaagtgtcaaactccaataggggcgataaactctagtatgccattccacaacatacaccctctcaccatcggggaatgccccaaatatgtgaaatcaaacaaattagtccttgcgactgggcttagaaatagtcccctaagagaaagGGGaagaagaaaaaga

>H5N1_A/Hunan/1/2008

taccatgcaaacaactcgacagagcaggttgacacaataatggaaaagaacgttactgttacacatgcccaaaacatactggaaaagacacataacgggaagctctgcgatatagatggagtgaagcctctgattttaaaagattgtagtgtagctggatggctcctcggaaatccaatgtgtgacgaattcatcaatgtgccggaatggtcttacatagtggagaaggccaacccagccaatgatctctgttacccggggaatttcaacgactatgaagaactgaaacacctattgagcagaataaatcattttgagaaaattcagatcatccccaaaagttcttggtccgatcatgaagcctcatcaggggtgagctcaacatgtccataccagggaacgccctcttttttcagaaatgtggtatggcttatcaaaaagaacaatacatacccaacaataaagagaagctacaataataccaaccaggaagatcttttgatactgtgggggattcatcattctaatgatgaggcagagcaggcaaagctctaccaaaacccaactacctatatttcagttgggacatcaacactaaaccagaggttggttccaaaaatagctactagatccaaagtaaacgggcaaagtggaaggatggatttcttctggacaattttaaaaccgaatgatgcaatcaacttcgagagtaatggaaatttcattgctccagaatatgcatacaaaattgtcaagaaaggggactcagcaattatgaaaagtgaagtggaatatggtaactgcaacaccaagtgtcaaactccaataggggcgataaactctagtatgccattccataacatacaccctctcaccatcggggaatgccccaaatatgtgaaatcaaacaaattagtccttgcgactgggctcagaaatagtcctctaagagaaagGGGaagaagaaaaaga

>H5N1_A/Hunan/1/2009

taccatgcaaacaactcgacagagcaggttgacacaataatggaaaagaacgttactgttacacatgcccaagacatactggaaaagacacacaacgggaagctctgcgatctagatggagtgaagcctctgattttaagagattgtagtgtagctggatggctcctcggaaacccaatgtgtgacgaatttatcaatgtgccagaatggtcttacatagtggagaaggccaacccagccaatgacctctgttacccagggaatttcaacgactatgaagaactgaaacacctattgagcagaataaatcattttgagaaaattcagatcatccccaaaagttcttggtccgatcatgaagcctcatcaggggtgagctcagyatgtccgtaccagggaacgccctcctttttcagaaatgtagtatggcttatcaaaaagaacaatacatacccaacaataaaaagaagctacaacaacaccaatcaggaagatctcttggtactgtgggggattcaccattctaatgatgcggcagaacagataaagctctatcaaaacccaaccacctatatttccgttggaacatcaacactaaaccagagattggtgccaaaaatagctactagatccaaagtaaacgggcaaagtggaaggatggatttcttctggacaattttaaaatcgaatgatgcaatcaacttcgagagtaatgggaatttcattgctccagaatatgcatacaaaattgtcaagaaaggggactcagcaattatgaaaagtgaagtggaatatggtaactgcaacaccaaatgtcaaactccaataggggcgataaactctagtatgccattccacaacatacaccctctcaccatcggggaatgccccaaatatgtgaaatcaaacaaattagtccttgcgactgggctcagaaatagtcctctaagagaaagGGGaagaaggaaaaga

>H5N1_A/Hunan/2/2009

taccatgcaaacaactcgacagagcaggttgacacaataatggaaaagaacgttactgttacacatgcccaaaacatactggaaaagacacacaacgggaagctctgcgatctagatggagtgaagcctttgattttaagagattgcagtgtagctggatggctcctcggaaacccaatgtgtgacgaatttatcaatgtgccagaatggtcttacatagtggagaaggccaacccagccaatgacctctgttacccagggaatttcaacgactatgaagaactgaaacacctattgagcagaataaatcattttgagaaaattcagatcatccccaaaagttcttggtccgatcatgaagcctcatcaggggtgagctcagcatgtccgtatcagggaacgccctcttttttcaggaatgtagtatggcttatcaaaaagaacaatacatacccaacaataaagagaagctacaataacaccaaccaggaagatcttttggtactgtgggggattcaccattctaatgatgcgacagaacagataaagctctatcaaaacccaaccacctatgtttccgttggaacatcaacactaaaccagagattggtaccaaaaatagctactagatccaaagtaaacgggcaaagtggaaggatggatttcttctggacaattttaaaatcgaatgatgcaatcaacttcgaaagtaatgggaatttcattgctccagaatatgcatacaaaattgtcaagaaaggggactcagcaattatgaaaagtgaagtggaatatggtaactgcagcaccaagtgtcaaactccgataggggcgataaactctagtatgccattccacaatatacaccctctcaccatcggggaatgccccaaatatgtgaaatcaaacaaattagtccttgcgactgggctcagaaatagtcctctaagagaaagGGGaagaagaaaaaga

>H5N1_A/Indonesia/160H/2005

taccatgcaaacaattcaacagagcaggttgacacaatcatggaaaagaacgttactgttacacatgcccaagacatattggaaaagacacacaacgggaagctctgcgatctggatggagtgaagcctctaattttaagagattgtagtgtagctggatggctcctcgggaacccgatgtgtgacgaattcatcaatgtaccggaatggtcttacatagtggagaaggccaatccaaccaatgacctctgttacccagggagtttcaacgactatgaagaactgaaacacctattgagcagaataaaccattttgagaaaattcagatcatccccaaaagttcttggtccgatcatgaagcctcatcaggagtgagttcagcatgtccatacctgggtagtccctccttttttagaaatgtggtatggcttatcaaaaagaacagtacatacccaacaataaagaaaagctacaataataccaaccaagaagatcttttggtactgtgggggattcaccatcctaatgatggggcagagcagacaaggctatatcaaaacccaaccacctatatttccattgggacatcaacactaaaccagagattggtaccaaaaatagctactagatccaaagtaaacgggcaaagtggaaggatggagttcttctggacaattttaaaacctaatgatgcaatcaacttcgagagtaatggaaatttcattgctccagaatatgcatacaaaattgtcaagaaaggggactcagcaattatgaaaagtgaattggaatatggtaactgcaacaccaagtgtcaaactccaatgggggcgataaactctagtatgccattccacaacatacaccctctcaccatcggggaatgccccaaatatgtgaaatcaaacagattagtccttgcaacagggctcagaaatagccctcaaagagaaagcagaagaaaaaagaga

>H5N1_A/Indonesia/175H/2005

taccatgcaaacaattcaacagagcaggttgacacaatcatggaaaagaacgttactgttacacatgcccaagacatattggaaaagacacacaacgggaagctctgcgatctagatggagtgaagcctctaattttaagagattgtagtgtagctggatggctcctcgggaacccaatgtgtgacgaattcatcaatgtaccggaatggtcttacatagtggagaaggccaatccaaccaatggcctctgttacccagggagtttcaacgactatgaagaactgaaacacctattgagcagaataaaccattttgagaaaattcagatcatccccaaaagttcttggtccgatcatgaagcctcatcaggagtgagctcagcatgtccatacctgggtagtccctccttttttagaaatgtggtatggcttatcaaaaagaacagtacatacccaacaataaagaaaagctacaataataccaaccaagaagatcttttggtactgtgggggattcaccatcctaatgatgcggcagagcagacaaggctatatcaaaacccaaccacctatatttccattgggacatcaacactaaaccagagattggtaccaaaaatagctactagatccaaagtaaacgggcaaagtggaaggatggagttcttctggacaattttaaaacctaatgatgcaatcaacttcgagagtaatgggaatttcattgctccagaatatgcatacaaaattgtcaagaaaggggactcagcaattatgaaaagtgaattggaatatggtaactgcaacaccaagtgtcaaactccaatgggggcgataaactctagtatgccattccacaacatacacccactcaccatcggggaatgccccaaatatgtgaaatcaaacagattagtccttgcaacagggctcagaaatagccctcaaagagaaagcagaagaaaaaagaga

>H5N1_A/Indonesia/195H/2005

taccatgcaaacaattcaacagagcaggttgacacaatcatggaaaagaacgttactgttacacatgcccaagacatactggaaaagacacacaacgggaagctctgcgatctagatggagtgaagcctctaattttaagagattgtagtgtagctggatggctcctcgggaacccaatgtgtgacgaattcatcaatgtaccggaatggtcttacatagtggagaaggccaatccaaccaatgacctctgttacccagggagtttcaacgactatgaagaactgaaacacctattgagcagaataaaccattttgagaaaattcaaatcatccccaaaagttcttggtccgatcatgaagcctcatcaggagtgagctcagcatgtccatacctgggaagtccctccttttttagaaatgtggtatggcttatcaaaaagaacagtacatacccaacaataaagaaaagctacaataataccaaccaagaagatcttttggtactgtggggaattcaccatcctaatgatgtggcagagcagacaaggctatatcaaaacccagccacctatatttccattgggacatcaacactaaaccagagattggtaccaaaaatagctactagatccaaagtaaacgggcaaagtggaaggatggagttcttctggacaattttaaaacctaatgatgcaatcaacttcgagagtaatggaaatttcattgctccagaatatgcatacaaaattgtcaagaaaggggactcagcaattatgaaaagtgaattggaatatggtaactgcaacaccaagtgtcaaactccaatgggggcgataaactctagtatgccattccacaacatacaccctctcactatcggggaatgccccaaatatgtgaaatcaaacagattagtccttgcaacagggctcagaaatagccctcaaagagagagcagaagaaaaaagaga

>H5N1_A/Indonesia/239H/2005

taccatgcaaacaattcaacagagcaggttgacacaatcatggaaaagaacgttactgttacacatgcccaagacatactggaaaagacacacaacgggaagctctgcgatctagatggagtgaagcctctaattttaagagattgtagtgtagctggatggctcctcgggaacccaatgtgtgacgaattcatcaatgtaccggaatggtcttacatagtggagaaggccaatccaaccaatgacctctgttacccaggaagtttcaacgactatgaagaactgaaacacctattgagcagaataaaccattttgagaaaattcaaatcatccccaaaagttcttggtccgatcatgaagcctcatcaggagtgagctcagcatgtccatacctgggaagtccctccttttttagaaatgtggtatggcttatcaaaaagaacagtacatacccaacaataaagaaaagctacaataataccaaccaagaagatcttttggtactgtggggaattcaccatcctaatgatgcggcagagcagacaaggctatatcaaaacccaaccacctatatttccattgggacatcaacactaaaccagagattggtaccaaaaatagctactagatccaaagtaaacgggcaaagtggaaggatggagttcttctggacaattttaaaccctaatgatgcaatcaacttcgagagtaatggaaatttcattgctccagaatatgcatacaaaattgtcaagaaaggggactcagcaattatgaaaagtgaattggaatatggtaactgcaacaccaagtgtcaaactccaatgggggcgataaactctagtatgccattccacaacatacaccctctcaccatcggggaatgccccaaatatgtgaaatcaaacagattagtccttgcaaaagggctcagaaatagccctcaaagagagagcagaagaaaaaagaga

>H5N1_A/Indonesia/245H/2005

taccatgcaaacaattcaacagagcaggttgacacaatcatggaaaagaacgttactgttacacatgcccaagacatactggaaaagacacacaacgggaagctctgcgatctagatggagtgaagcctctaattttaagagattgtagtgtagctggatggctcctcgggaacccaatgtgtgacgaattcatcaatgtaccggaatggtcttacatagtggagaaggccaatccaaccaatgacctctgttacccagggagtttcaacgactatgaagaactgaaacacctattgagcagaataaaccattttgagaaaattcaaatcatccccaaaagttcttggtccgatcatgaagcctcatcaggagtgagctcagcatgtccatacctgggaagtccctccttttttagaaatgtggtatggcttatcaaaaagaacagtacatacccaacaataaagaaaagctacaataataccaaccaagaagatcttttggtactgtggggaattcaccatcctaatgatgcggcagagcagacaaggctatatcaaaacccaaccacctatatttccattgggacatcaacactaaaccagagattggtaccaaaaatagctactagatccaaagtaaacgggcaaagtggaaggatggagttcttctggacaattttaaaccctaatgatgcaatcaacttcgagagtaatggaaatttcattgctccagaatatgcatacaaaattgtcaagaaaggggactcagcaattatgaaaagtgaattggaatatggtaactgcaacaccaagtgtcaaactccaatgggggcgataaactctagtatgccattccacaacatacaccctctcaccatcggggaatgccccaaatatgtgaaatcaaacagattagtccttgcaacagggctcagaaatagccctcaaagagagagcagaagaaaaaagaga

>H5N1_A/Indonesia/283H/2006

taccatgcaaacaattcaacagagcaggttgacacaatcatggaaaagaacgttactgttacacatgcccaagacatactggaaaagacacacaacgggaagctctgcgatctagatggagtgaagcctctaattttaagagattgtagtgtagctggatggctcctcgggaacccaatgtgtgacgaattcatcaatgtaccggaatggtcttacatagtggagaaggccaatccaaccaatgacctctgttacccaggaagtttcaacgactatgaagaactgaaacacctattgagcagaataaaccattttgagaaaattcaaatcatccccaaaagttcttggtccgatcatgaagcctcatcaggagtgagctcagcatgtccatacctgggaagtccctccttttttagaaatgtggtatggcttatcaaaaagaacagtacatacccaacaataaagaaaagctacaataataccaaccaagaagatcttttggtactgtggggaattcaccatcctaatgatgcggcagagcagacaaggctatatcaaaacccaaccacctatatttccattgggacatcaacactaaaccagagattggtaccaaaaatagctactagatccaaagtaaacgggcaaagtggaaggatggagttcttctggacaattttaaaccctaacgatgcaatcaacttcgagagtaatggaaatttcattgctccagaatatgcatacaaaattgtcaagaaaggggactcagcaattatgaaaagtgaattggaatatggtaactgcaacaccaagtgtcaaactccaatgggggcgataaactctagtatgccattccacaacatacaccctctcaccatcggggaatgccccaaatatgtgaaatcaaacagattagtccttgcaacagggctcagaaatagccctcaaagagagagcagaagaaaaaagaga

>H5N1_A/Indonesia/286H/2006

taccatgcaaacaattcaacagagcaggttgacacaatcatggaaaagaacgttactgttacacatgcccaagacatactggaaaagacacacaacgggaagctctgcgatctagatggagtgaagcctctaattttaagagattgtagtgtagctggatggctcctcgggaacccaatgtgtgacgaattcatcaatgtaccggaatggtcttacatagtggagaaggccaatccaaccaatgacctctgttacccaggaagtttcaacgactatgaagaactgaaacacctattgagcagaataaaccattttgagaaaattcaaatcatccccaaaagttcttggtccgatcatgaagcctcatcaggagtgagctcagcatgtccatacctgggaagtccctccttttttagaaatgtggtatggcttatcaaaaagaacagtacatacccaacaataaagaaaagctacaataataccaaccaagaagatcttttggtactgtggggaattcaccatcctaatgatgcggcagagcagacaaggctatatcaaaacccaaccacctatatttccattgggacatcaacactaaaccagagattggtaccaaaaatagctactagatccaaagtaaacgggcaaagtggaaggatggagttcttctggacaattttaaaccctaacgatgcaatcaacttcgagagtaatggaaatttcattgctccagaatatgcatacaaaattgtcaagaaaggggactcagcaattatgaaaagtgaattggaatatggtaactgcaacaccaagtgtcaaactccaatgggggcgataaactctagtatgccattccacaacatacaccctctcaccatcggggaatgccccaaatatgtgaaatcaaacagattagtccttgcaacagggctcagaaatagccctcaaagagaaagcagaagaaaaaagaga

>H5N1_A/Indonesia/292H/2006

taccatgcaaacaattcaacagagcaggttgacacaatcatggaaaagaacgttactgttacacatgcccaagacatactggaaaagacacacaacgggaagctctgcgatctagatggagtgaagcctctaattttaagagattgtagtgtagctggatggctcctcgggaacccaatgtgtgacgaattcatcaatgtaccggaatggtcttacatagtggagaaggccaatccaaccaatgacctctgttacccaggaagtttcaatgactatgaagaactgaaacacctattgagcagaataaaccattttgagaaaattcaaatcatccccaaaagttcttggtccgatcatgaagcctcatcaggagtgagctcagcatgtccatacctgggaagtccctccttttttagaaatgtggtatggcttatcaaaaagaacagtacatacccaacaataaagaaaagctacaataataccaaccaagaagatcttttggtactgtggggaattcaccatcctaatgatgcggcagagcagacaaggctatatcaaaacccaaccacctatatttccattgggacatcaacactaaaccagagattggtaccaaaaatagctactagatccaaagtaaacgggcaaagtggaaggatggagttcttctggacaattttaaaccctaacgatgcaatcaacttcgagagtaatggaaatttcattgctccagaatatgcatacaaaattgtcaagaaaggggactcagcaattatgaaaagtgaattggaatatggtaactgcaacaccaagtgtcaaactccaatgggggcgataaactctagtatgccattccacaacatacaccctctcaccatcggggaatgccccaaatatgtgaaatcaaacagattagtccttgcaacagggctcagaaatagccctcaaagagagagcagaagaaaaaagaga

>H5N1_A/Indonesia/298H/2006

taccatgcaaacaattcaacagagcaggttgacacaatcatggaaaagaacgttactgttacacatgctcaagacatactggaaaagacacacaacgggaagctctgcaatctagatggagtgaagcctctaattttaagagattgtagtgtagctggatggctcctcgggaacccaatgtgtgacgaattcatcaatgtaccggaatggtcttacatagtggagaaggccaatccaaccaatgacctctgttacccagggagtttcaacgactatgaagaactgaaacacctattgagcagaataaaccattttgagaaaattcaaatcatccccaaaagttcttggtccgatcatgaagcctcatcaggagtgagctcagcatgtccatacctgggaagtccctccttttttagaaatgtggtatggcttatcaaaaagaacagtacatacccaacaataaagaaaagctacaataataccaaccaagaagatcttttggtactgtggggaattcaccatcctaatgatgcggcagagcagacaaggctatatcaaaacccaaccacctatatttccattgggacatcaacactaaaccagagattggtaccaaaaatagctactagatccaaagtaaacgggcaaagtggaaggatggagttcttctggacaattttaaaacctaatgatgcaatcaacttcgagagtaatggaaatttcattgctccagaatatgcatacaaaattgtcaagaaaggggactcagcaattatgaaaagtgaattggaatatggtaactgcaacaccaagtgtcaaactccgatgggggcgataaactctagtatgccattccacaacatacaccctctcaccatcggggaatgccccaaatatgtgaaatcaaacagattagtccttgcaacagggctcagaaatagccctcaaagagagagcagaagaaaaaagaga

>H5N1_A/Indonesia/304H/2006

taccatgcaaacaattcaacagagcaggttgacacaatcatggaaaagaacgttactgttacacatgcccaagacatactggaaaagacacacaacgggaagctctgcgatctagatggagtgaagcctctaattttaagagattgtagtgtagctggatggctcctcgggaacccaatgtgtgacgaattcatcaatgtaccggaatggtcttacatagtggagaaggccaatccaaccaatggcctctgttacccaggaagtttcaacgactatgaagaactgaaacacctattgagcagaataaaccattttgagaaaattcaaatcatccccaaaagttcttggtccgatcatgaagcctcatcaggagtgagctcagcatgtccatacctgggaagtccctccttttttagaaatgtggtatggcttatcaaaaagaacagtacatacccaacaataaagaaaagctacaataataccaaccaagaagatcttttggtactgtggggaattcaccatcctaatgatgcggcagagcagacaaggctatatcaaaacccaaccacctatatttccattgggacatcaacactaaaccagagattggtaccaaaaatagctactagatccaaagtaaacgggcaaagtggaaggatggagtttttctggacaattttaaaccctaatgatgcaatcaacttcgagagtaatggaaatttcattgctccagaatatgcatacaaaattgtcaagaaaggggactcagcaattatgaaaagtgaattggaatatggtaactgcaacaccaagtgtcaaactccaatgggggcgataaactctagtatgccattccacaacatacaccctctcaccatcggggaatgccccaaatatgtgaaatcaaacagattagtccttgcaacagggctcagaaatagccctcaaagagagagcagaagaaaaaagaga

>H5N1_A/Indonesia/321H/2006

taccatgcaaacaattcaacagagcaggttgacacaatcatggaaaagaacgttactgttacacatgcccaagacatactggaaaagacacacaacgggaagctctgcgatctagatggagtgaagcctctaattttaaaagattgtagtgtagctggatggctcctcgggaacccaatgtgtgacgaattcatcaatgtaccggaatggtcttacatagtggagaaggccaatccaaccaatgacctctgttacccagggagtttcaacgactatgaagaactgaaacacctattgagcagaataaaccattttgagaaaattcaaatcatccccaaaagttcttggtccgatcatgaagcctcatcaggagtgagctcagcatgtccatacctgggaagtccctccttttttagaaatgtggtatggcttatcaaaaagaacagtacatacccaacaataaagaaaagctacaataataccaaccaagaagatcttttggtactgtggggaattcaccaccctaatgatgcggcagagcagacaaggctatatcaaaacccaaccacctatatttccattgggacatcaacactaaaccagagattggtaccaaaaatagctactagatccaaagtaaacgggcaaagtggaaggatggagttcttctggacaattttaaaacctaatgatgcaatcaacttcgagagtaatggaaatttcattgctccagaatatgcatacaaaattgtcaagaaaggggactcagcaattatgaaaagtgaattggaatatggtaactgtaacaccaagtgtcaaactccaatgggggcgataaactctagtatgccattccacaacatacaccctctcaccatcggggaatgccccaaatatgtgaaatcaaacagattagtccttgcaacagggctcagaaatagccctcaaagagagagcagaagaaaaaagaga

>H5N1_A/Indonesia/341H/2006

taccatgcaaacaattcaacagagcaggttgacacaatcatggaaaagaacgttactgttacacatgcccaagacatactggaaaagacacacaacgggaagctctgcgatctagatggagtgaagcctctaattttaagggattgtagtgtagctggatggcttctcgggaacccaatgtgtgacgaattcatcaatgtaccggaatggtcttacatagtggagaaggccaatccaaccaatgacctctgttacccagggagtttcaacgactatgaagaactgaaacacctattgagcagaataaaccattttgagaaaattcaaatcatccccaaaagttcttggtccgatcatgaagcctcatcaggagtgagctcagcatgtccatatctgggaagtccctccttttttagaaatgtggtatggcttatcaaaaagaacagtacatacccaacaataaaggaaagctacaataataccaaccaagaagatcttttggtactgtggggaattcaccatcctaatgatgcggcagagcagacaaggctatatcaaaacccaaccacctatatttccattgggacatcaacactcaaccagagattggtaccaaaaatagctactagatccaaagtaaacgggcaaagtggaaggatggagttcttctggacaattttaaaacctaatgatgcaatcaacttcgagagtaatggaaatttcattgctccagaatatgcatacaaaattgtcaagaaaggggactcagcaattatgaaaagtgaattggaatatggtaactgcaacaccaagtgtcaaactccaatgggggcgataaactctagtatgccattccacaacatacaccctctcaccatcggggaatgccccaaatatgtgaaatcaaacaaattagtccttgcaacagggctcagaaatagccctcaaagagagagcagaagaaaaaagaga

>H5N1_A/Indonesia/5/2005

taccatgcaaacaattcaacagagcaggttgacacaatcatggaaaagaacgttactgttacacatgcccaagacatactggaaaagacacacaacgggaagctctgcgatctagatggagtgaagcctctaattttaagagattgtagtgtagctggatggctcctcgggaacccaatgtgtgacgaattcatcaatgtaccggaatggtcttacatagtggagaaggccaatccaaccaatgacctctgttacccagggagtttcaacgactatgaagaactgaaacacctattgagcagaataaaccattttgagaaaattcaaatcatccccaaaagttcttggtccgatcatgaagcctcatcaggagtgagctcagcatgtccatacctgggaagtccctccttttttagaaatgtggtatggcttatcaaaaagaacagtacatacccaacaataaagaaaagctacaataataccaaccaagaagatcttttggtactgtggggaattcaccatcctaatgatgcggcagagcagacaaggctatatcaaaacccaaccacctatatttccattgggacatcaacactaaaccagagattggtaccaaaaatagctactagatccaaagtaaacgggcaaagtggaaggatggagttcttctggacaattttaaaacctaatgatgcaatcaacttcgagagtaatggaaatttcattgctccagaatatgcatacaaaattgtcaagaaaggggactcagcaattatgaaaagtgaattggaatatggtaactgcaacaccaagtgtcaaactccaatgggggcgataaactctagtatgccattccacaacatacaccctctcaccatcggggaatgccccaaatatgtgaaatcaaacagattagtccttgcaacagggctcagaaatagccctcaaagagagagcagaagaaaaaagaga

>H5N1_A/Indonesia/534H/2006

taccatgcaaacaattcaacagagcaggttgacacaataatggaaaagaacgtcactgtcacacatgcccaagacatactggaaaagacacacaacgggaagctctgcgatctagatggagtgaagcctctaattttaagagattgtagtgtagctggatggctcctcgggaacccaatgtgtgacgaattcatcaatgtaccggaatggtcttacatagtggagaaggccaatccagccaatggcctctgttacccagggaatttcaacgactatgaagaactgaaacacctattgagcagaataaatcattttgaaaaacttcagataatccccaaaagttcttggtccgatcatgaagcctcattaggggtgagctcagcatgtccatacctgggaaggtcctccttctttagaaatgtggtatggcttatcaaaaagaacaatacatacccaacaataaagagaagctacaataataccaaccaagaagatcttttggtactgtgggggattcaccatcctaatgatgcagcagagcagacaaggctatatcaaaacccaaccacttatatttccgttgggacatcaacactaaaccagagattggtaccaaaaatagctactagatccaaagtaaatgggcaaagtggaaggatggaattcttctggacaattttaaaaccgaatgatgcaatcaacttcgagagtaatggaaatttcattgctccagaatatgcatacaaaattgtaaagaaaggggactctgcaattatgaaaagtgaattggaatatggtgactgcaacaccaagtgtcaaactccaatgggggcgataaactctagtatgccattccacaacatacaccctctcaccatcggggaatgccccaaatatgtgaagtcaaacagattagtccttgcgactgggctcagaaatagccctcaaagagagagaagaagaaaaaagaga

>H5N1_A/Indonesia/535H/2006

taccatgcaaacaattcaacagagcaggttgacacaataatggaaaagaacgtcactgtcacacatgcccaagacatactggaaaagacacacaacgggaagctctgcgatctagatggagtgaagcctctaattttaagagattgtagtgtagctggatggctcctcgggaacccaatgtgtgacgaattcatcaatgtaccggaatggtcttacatagtggagaaggccaatccagccaatggcctctgttacccagggaatttcaacgactatgaagaactgaaacacctattgagcagaataaatcattttgaaaaacttcagataatccccaaaagttcttggtccgatcatgaagcctcattaggggtgagctcagcatgtccatacctgggaaggtcctccttctttagaaatgtggtatggcttatcaaaaagaacaatacatacccaacaataaagagaagctacaataataccaaccaagaagatcttttggtactgtgggggattcaccatcctaatgatgcagcagagcagacaaggctatatcaaaacccaaccacttatatttccgttgggacatcaacactaaaccagagattggtaccaaaaatagctactagatccaaagtaaatgggcaaagtggaaggatggaattcttctggacaattttaaaaccgaatgatgcaatcaacttcgagagtaatggaaatttcattgctccagaatatgcatacaaaattgtaaagaaaggggactctgcaattatgaaaagtgaattggaatatggtgactgcaacaccaagtgtcaaactccaatgggggcgataaactctagtatgccattccacaacatacaccctctcaccatcggggaatgccccaaatatgtgaagtcaaacagattagtccttgcgactgggctcagaaatagccctcaaagagagagaagaagaaaaaagaga

>H5N1_A/Indonesia/536H/2006

taccatgcaaacaattcaacagagcaggttgacacaataatggaaaagaacgtcactgtcacacatgcccaagacatactggaaaagacacacaacgggaagctctgcgatctagatggagtgaagcctctaattttaagagattgtagtgtagctggatggctcctcgggaacccaatgtgtgacgaattcatcaatgtaccggaatggtcttacatagtggagaaggccaatccagccaatggcctctgttacccagggaatttcaacgactatgaagaactgaaacacctattgagcagaataaatcattttgaaaaacttcagataatccccaaaagttcttggtccgatcatgaagcctcattaggggtgagctcagcatgtccatacctgggaaggtcctccttctttagaaatgtggtatggcttatcaaaaagaacaatacatacccaacaataaagagaagctacaataataccaaccaagaagatcttttggtactgtgggggattcaccatcctaatgatgcagcagagcagacaaggctatatcaaaacccaaccacttatatttccgttgggacatcaacactaaaccagagattggtaccaaaaatagctactagatccaaagtaaatgggcaaagtggaaggatggaattcttctggacaattttaaaaccgaatgatgcaatcaacttcgagagtaatggaaatttcattgctccagaatatgcatacaaaattgtaaagaaaggggactctgcaattatgaaaagtgaattggaatatggtgactgcaacaccaagtgtcaaactccaatgggggcgataaactctagtatgccattccacaacatacaccctctcaccatcggggaatgccccaaatatgtgaagtcaaacagattagtccttgcgactgggctcagaaatagccctcaaagagagagaagaagaaaaaagaga

>H5N1_A/Indonesia/538H/2006

taccatgcaaacaattcaacagagcaggttgacacaataatggaaaagaacgtcactgtcacacatgcccaagacatactggaaaagacacacaacgggaagctctgcgatctagatggagtgaagcctctaattttaagagattgtagtgtagctggatggctcctcgggaacccaatgtgtgacgaattcatcaatgtaccggaatggtcttacatagtggagaaggccaatccagccaatggcctctgttacccagggaatttcaacgactatgaagaactgaaacacctattgagcagaataaatcattttgaaaaacttcagataatccccaaaagttcttggtccgatcatgaagcctcattaggggtgagctcagcatgtccatacctgggaaggtcctccttctttagaaatgtggtatggcttatcaaaaagaacaatacatacccaacaataaagagaagctacaataataccaaccaagaagatcttttggtactgtgggggattcaccatcctaatgatgcagcagagcagacaaggctatatcaaaacccaaccacttatatttccgttgggacatcaacactaaaccagagattggtaccaaaaatagctactagatccaaagtaaatgggcaaagtggaaggatggaattcttctggacaattttaaaaccgaatgatgcaatcaacttcgagagtaatggaaatttcattgctccagaatatgcatacaaaattgtaaagaaaggggactctgcaattatgaaaagtgaattggaatatggtgactgcaacaccaagtgtcaaactccaatgggggcgataaactctagtatgccattccacaacatacaccctctcaccatcggggaatgccccaaatatgtgaagtcaaacagattagtccttgcgactgggctcagaaatagccctcaaagagagagaagaagaaaaaagaga

>H5N1_A/Indonesia/542H/2006

taccatgcaaacaattcaacagagcaggttgacacaatcatggaaaagaacgttactgttacacatgcccaagacatactggaaaagacacacaacgggaagctctgcgatctagatggagtgaagcctctaattttaagagattgtagtgtagctggatggctcctcgggaacccaatgtgtgacgaattcatcaatgtaccggaatggtcttacatagtggagaaggccaatccaaccaatgacctctgttacccagggagtttcaacgactatgaagaactgaaacatctattgagcagaataaaccattttgagaaaattcaaatcatccccaaaagttcttggtccgatcatgaagcctcatcaggagtgagctcagcatgtccatacctgggaagtccctccttttttagaaatgtggtatggcttatcaaaaagaacagtacatacccaacaataaagaaaagctacaataataccaaccaagaagatcttttggtactgtggggaattcaccatcctaatgatgcggcagagcagacaaggctatatcaaaacccaaccacctatatttccattgggacatcaacactaaaccagagattggtaccaaaaatagctactagatccaaagtaaacgggcaaagtggaaggatggagttcttctggacaattttaaaacctaatgatgcaatcaacttcgaaagtaatggaaatttcattgctccagaatatgcatacaaaattgtcaagaaaggggactcagcaattatgaaaagtgaattggaatatggtaactgcaacaccaagtgtcaaactccaatgggggcgataaactctagtatgccattccacaacatacaccctctcaccatcggggaatgccccaaatatgtgaaatcaaacagattagtccttgcaacagggctcagaaatagccctcaaagagagagcagaagaaaaaagaga

>H5N1_A/Indonesia/546H/2006

taccatgcaaacaattcaacagagcaggttgacacaataatggaaaagaacgtcactgtcacacatgcccaagacatactggaaaagacacacaacgggaagctctgcgatctagatggagtgaagcctctaattttaagagattgtagtgtagctggatggctcctcgggaacccaatgtgtgacgaattcatcaatgtaccggaatggtcttacatagtggagaaggccaatccagccaatggcctctgttacccagggaatttcaacgactatgaagaactgaaacacctattgagcagaataaatcattttgaaaaacttcagataatccccaaaagttcttggtccgatcatgaagcctcattaggggtgagctcagcatgtccatacctgggaaggtcctccttctttagaaatgtggtatggcttatcaaaaagaacaatacatacccaacaataaagagaagctacaataataccaaccaagaagatcttttggtactgtgggggattcaccatcctaatgatgcagcagagcagacaaggctatatcaaaacccaaccacttatatttccgttgggacatcaacactaaaccagagattggtaccaaaaatagctactagatccaaagtaaatgggcaaagtggaaggatggaattcttctggacaattttaaaaccgaatgatgcaatcaacttcgagagtaatggaaatttcattgctccagaatatgcatacaaaattgtaaagaaaggggactctgcaattatgaaaagtgaattggaatatggtgactgcaacaccaagtgtcaaactccaatgggggcgataaactctagtatgccattccacaacatacaccctctcaccatcggggaatgccccaaatatgtgaagtcaaacagattagtccttgcgactgggctcagaaatagccctcaaagagagagaagaagaaaaaagaga

>H5N1_A/Indonesia/546bH/2006

taccatgcaaacaattcaacagagcaggttgacacaataatggaaaagaacgtcactgtcacacatgcccaagacatactggaaaagacacacaacgggaagctctgcgatctagatggagtgaagcctctaattttaagagattgtagtgtagctggatggctcctcgggaacccaatgtgtgacgaattcatcaatgtaccggaatggtcttacatagtggagaaggccaatccagccaatggcctctgttacccagggaatttcaacgactatgaagaactgaaacacctattgagcagaataaatcattttgaaaaacttcagataatccccaaaagttcttggtccgatcatgaagcctcattaggggtgagctcagcatgtccatacctgggaaggtcctccttctttagaaatgtggtatggcttatcaaaaagaacaatacatacccaacaataaagagaagctacaataataccaaccaagaagatcttttggtactgtgggggattcaccatcctaatgatgcagcagagcagacaaggctatatcaaaacccaaccacttatatttccgttgggacatcaacactaaaccagagattggtaccaaaaatagctactagatccaaagtaaatgggcaaagtggaaggatggaattcttctggacaattttaaaaccgaatgatgcaatcaacttcgagagtaatggaaatttcattgctccagaatatgcatacaaaattgtaaagaaaggggactctgcaattatgaaaagtgaattggaatatggtgactgcaacaccaagtgtcaaactccaatgggggcgataaactctagtatgccattccacaacatacaccctctcaccatcggggaatgccccaaatatgtgaagtcaaacagattagtccttgcgactgggctcagaaatagccctcaaagagagagaagaagaaaaaagaga

>H5N1_A/Indonesia/560H/2006

taccatgcaaacaattcaacagagcaggttgacacaataatggaaaagaacgtcactgtcacacatgcccaagacatactggaaaagacacacaacgggaagctctgcgatctagatggagtgaagcctctaattttaagagattgtagtgtagctggatggctcctcgggaacccaatgtgtgacgaattcatcaatgtaccggaatggtcttacatagtggagaaggccaatccagccaatggcctctgttacccagggaatttcaacgactatgaagaactgaaacacctattgagcagaataaatcattttgaaaaacttcagataatccccaaaagttcttggtccgatcatgaagcctcattaggggtgagctcagcatgtccatacctgggaaggtcctccttctttagaaatgtggtatggcttatcaaaaagaacaatacatacccaacaataaagagaagctacaataataccaaccaagaagatcttttggtactgtgggggattcaccatcctaatgatgcagcagagcagacaaggctatatcaaaacccaaccacttatatttccgttgggacatcaacactaaaccagagattggtaccaaaaatagctactagatccaaagtaaatgggcaaagtggaaggatggaattcttctggacaattttaaaaccgaatgatgcaatcaacttcgagagtaatggaaatttcattgctccagaatatgcatacaaaattgtaaagaaaggggactctgcaattatgaaaagtgaattggaatatggtgactgcaacaccaagtgtcaaactccaatgggggcgataaactctagtatgccattccacaacatacaccctctcaccatcggggaatgccccaaatatgtgaagtcaaacagattagtccttgcgactgggctcagaaatagccctcaaagagagagaagaagaaaaaagaga

>H5N1_A/Indonesia/567H/2006

taccatgcaaacaattcaacagagcaggttgacacaatcatggaaaagaacgttactgttacacatgcccaagacatactggaaaagacacacaacgggaagctctgcgatctagatggagtgaagcctctaattttaagagattgtagtgtagctggatggctcctcgggaacccaatgtgtgacgaattcatcaatgtaccggaatggtcttacatagtggagaaggccaatccaaccaatgacctctgttacccagggagtttcaacgactatgaagaactgaaacacctattgagcagaataaaccattttgagaaaattcaaatcatccccaaaagttcttggtccgatcatgaagcctcatcaggagtgagctcagcatgtccatacctgggaagtccctccttttttagaaatgtggtatggcttatcaaaaagaacagtacatacccaacaataaagaaaagctacaataataccaaccaagaagatcttttggtactgtggggaattcaccatcctaatgatgcggcagagcagacaaggctatatcaaaacccaaccacctatatttccattgggacatcaacactaaaccagagattggtaccaaaaatagctactagatccaaagtaaacgggcaaagtggaaggatggagttcttctggacaattttaaaacctaatgatgcaatcaacttcgagagtaatggaaatttcattgctccagaatatgcatacaaagttgtcaagaaaggggactcagcaattatgaaaagtgaattggaatatggtaactgcaacaccaagtgtcaaactccaatgggggcgataaactctagtatgccattccacaacatacaccctctcaccatcggggaatgccccaaatatgtgaaatcaaacagattagtccttgcaacagggctcagaaatagccctcaaagagagagcagaagaaaaaagaga

>H5N1_A/Indonesia/569H/2006

taccatgcaaacaattcaacagagcaggttgacacaatcatggaaaagaacgttactgttacacatgcccaagacatactggaaaagacacacaatgggaagctctgcgatctagatggagtgaagcctctaattttaagagattgtagtgtagctggatggctcctcgggaacccaatgtgtgacgaattcatcaatgtaccggaatggtcttacatagtggagaaggccaatccaaccaatgacctctgttacccagggagtttcaacgactatgaagaactgaaacacctattgagcagaataaaccattttgagaaaattcaaatcatccccaaaagttcttggtccgaccatgaagcctcatcaggagtgagctcagcatgtccatacctgggaagtccctccttttttagaaatgtggtatggcttatcaaaaagaacagtacatacccaacaataaagaaaagctacaataataccaaccaagaagatcttttggtactgtggggaattcaccatcctaatgatgcggcagagcagacaaggctatatcaaaacccaaccacctatatttccattgggacatcaacactaaaccagagattggtaccaaaaatagctactagatccaaagtaaacgggcaaagtggaaggatggagttcttctggacaattttaaaacctaatgatgcaatcaacttcgagagtaatggaaatttcattgctccagaatatgcatacaaaattgtcaagaaaggggactcagcaattatgaaaagtgaattggaatatggtaactgcaacaccaagtgtcaaactccgatgggggcgataaactctagtatgccattccacaacatacaccctctcaccatcggggaatgccccaaatatgtgaaatcaaacagattagtccttgcaacagggctcagaaatagccctcaaagagagagcagaagaaaaaagaga

>H5N1_A/Indonesia/583H/2006

taccatgcaaacaattcaacagagcaggttgacacaatcatggaaaagaacgttactgttacacatgcccaagacatactggaaaagacacacaacgggaagctctgcgatctagatggagtgaagcctctaattttaagagattgtagtgtagctggatggctcctcgggaacccaatgtgtgacgaattcatcaatgtaccggaatggtcttacatagtggagaaggccaatccaaccaatgacctctgttacccagggagtttcaacgactatgaagaactgaaacatctattgagcagaataaaccattttgagaaaattcaaatcatccccaaaagttcttggtccgatcatgaagcctcatcaggagtgagctcagcatgtccatacctgggaagtccctccttttttagaaatgtggtatggcttatcaaaaagaacagtacatacccaacaataaagaaaagctacaataataccaaccaagaagatcttttggtactgtggggaattcaccatcctaatgatgcggcagagcagacaaggctatatcaaaacccaaccacctatatttccattgggacatcaacactaaaccagagattggtaccaaaaatagctactagatccaaagtaaacgggcaaagtggaaggatggagttcttctgggcaattttaaaacctaatgatgcaatcaacttcgagagtaatggaaatttcattgctccagaatatgcatacaaaattgtcaagaaaggggactcagcaattatgaaaagtgaattggaatatggtaactgcaacaccaagtgtcaaactccaatgggggcgataaactctagtatgccattccacaacatacaccctctcaccatcggggaatgccccaaatatgtgaaatcaaacagattagtccttgcaacagggctcagaaatagccctcaaagagagagcagaagaaaaaagaga

>H5N1_A/Indonesia/6/2005

taccatgcaaacaattcaacagagcaggttgacacaataatggaaaagaacgttactgttacacatgcccaagacatactggaaaagacacacaacgggaagctctgtgatctagatggagtgaagcctctaattttaagagattgtagtgtagctggatggctcctcgggaacccaatgtgtgacgaattcatcaatgtaccggaatggtcttacatagtggagaaggccaatccagccaatgacctctgctacccagggaatttcaatgactatgaagaactgaaacacctattgagcagaataaaccattttgagaaaattcagatcatccccaaaagttcttggtccgatcatgaagcctcatcaggggtgagctcagcatgtccatacctgggaacgccctccttttttagaaatgtggtatggcttatcaaaaagaacagtacatacccaacaataaaaagaagctacaataataccaaccaagaagatcttttggtactgtgggggattcaccatcctaatgatgcggcagagcaaacgaggctatatcaaaatccaaccacctatatttccgttgggacatcaacactgaaccagagattggtaccaaaaatagctaccagatccaaagtaaacggacaaagtggaaggatggagttcttctggacaattttaaaacctaatgatgcaatcaacttcgagagtaatggaaatttcattgctccagaatatgcctacaaaattgtcaagaaaggggactcagcaattatgaaaagtgaattggaatatggcaactgcaacaccaagtgtcaaactccaatgggggcgataaactctagtatgccattccacaacatacaccctctcaccatcggggaatgccccaaatatgtgaaatcaaacagattagtccttgcgactgggctcagaaatagccctcaaagagagagaagaagaaaaaagaga

>H5N1_A/Indonesia/604H/2006

taccatgcaaacaattcaacagagcaggttgacacaatcatggaaaagaacgttactgttacacatgcccaagacatactggaaaagacacacaacgggaagctctgcgatctagatggagtgaagcctctaattttaagagattgtagtgtagctggatggctcctcgggaacccaatgtgtgacgaattcatcaatgtaccggaatggtcttacatagtggagaaggccaatccaaccaatgacctctgttacccagggagtttcaacgactatgaagaactgaaacacctattgagcagaataaaccattttgagaaaattcaaatcatccccaaaagttcttggtccgatcatgaagcctcatcaggagtgagctcagcatgtccatacctgggaagtccttccttttttagaaatgtggtatggcttatcaaaaagaacagtacatacccaacaataaagacaagctacaataataccaaccaagaagatcttttggtactgtggggaattcaccattctaatgatgcggcagagcagacaaggctatatcaaaacccaaccacctatatttccattgggacatcaacactaaaccagagattggtaccaaaaatagctactagatccaaagtaaacgggcaaagtggaaggatggagttcttctggacaattttaaaacctaatgatgcaatcaacttcgagagtaatggaaatttcattgctccagaatatgcatacaaaattgtcaagaaaggggactcagcaattatgaaaagtgaattggaatatggtaactgcaacaccaagtgtcaaactccaatgggggcaataaactctagtatgccattccacaacatacaccctctcaccatcggggaatgccccaaatatgtgaaatcaaacagattagtccttgcaacagggctcagaaatagccctcaaagagagagcagaagaaaaaagaga

>H5N1_A/Indonesia/7/2005

taccatgcaaacaattcaacagagcaggttgacacaatcatggaaaagaacgttactgttacacatgcccaagacatattggaaaagacacacaacgggaagctctgcgatctagatggagtgaagcctctaattttaagagattgtagtgtagctggatggctcctcgggaacccaatgtgtgacgaattcatcaatgtaccggaatggtcttacatagtggagaaggccaatccaaccaatgacctctgttacccagggagtttcaacgactatgaagaactgaaacacctattgagcagaataaaccattttgagaaaattcagatcatccccaaaagttcttggtccgatcatgaagcctcatcaggagtgagttcagcatgtccatacctgggtagttcctccttttttagaaatgtggtatggcttatcaaaaagaacagtacatacccaacaataaagaaaagctacaataataccaaccaagaagatcttttggtactgtgggggattcaccatcctaatgatgcggcagagcagacaaggctatatcaaaacccaaccacctatatttccattgggacatcaacactaaaccagagattggtaccaaaaatagctactagatccaaagtaaatgggcaaagtggaaggatggagttcttctggacaattttaaaacctaatgatgcaatcaacttcgagagtaatggaaatttcattgctccagaatatgcatacaaaattgtcaagaaaggggactcagcaattatgaagagtgaattggaatatggtaactgcaacaccaagtgtcaaactccaatgggggcgataaactctagtatgccattccacaacatacaccctctcaccatcggggaatgccccaaatatgtgaaatcaaacagattagtccttgcaacagggctcagaaatagccctcaaagagaaagcagaagaaaaaagaga

>H5N1_A/Indonesia/7261/2008

taccatgcaaacaattcaacggagcaggttgacacaatcatggaaaagaacgttactgttacacatgcccaagacatactggaaaagacacacaacgggaagctctgcgatctagatggagtgaagcctctaattttaaaagattgtagtgtagctggatggctcctcgggaacccaatgtgtgacgaattcatcaacgtaccagaatggtcttacatagtggagaaggccaatccaaccaatgacctctgttacccagggagtttcaacgactatgaagaactgaaacacctattgagcagaataaaccattttgagaaaattcagatcatccccaaaagttcttggtccgatcatgaagcctcatcaggagtgagctcagcatgtccatacctgggaagtccctccttttttagaaatgtggtatggcttatcaaaaagaacagtacgtacccaacaataaagaaaacctacaataataccaaccaagaagatcttttgatactgtggggaattcaccatcctaataatgaggcagagcagacaatgctatatcaaaacccaaccacctatatttccattgggacatcaacactaaaccagagattggtaccaaaaatagctactagatccaaagtaaacgggcaaagtggaaggatggagtttttctggacaattttaaaaccaaatgatgcaatcaacttcgagagtaatggaaatttcattgctccagaatatgcatacaaaattgtcaagaaaggggactcagcaattatgaaaagtgaattggaatatggtaactgcaacaccaagtgtcaaactccaatgggggcgataaactctagtatgccattccacaacatacaccctctcaccatcggggaatgccccaaatatgtgaaatcaaacagattagtcctagcaacagggctcagaaatagccctcaaagagagagcagaaggaaaaagaga

>H5N1_A/Indonesia/7272/2008

taccatgcaaacaattcaacggagcaggttgacacaatcatggaaaagaacgttactgttacacatgcccaagacatactggaaaagacacacaacgggaagctctgcgatctagatggagtgaagcctctaattttaaaagattgtagtgtagctggatggctcctcgggaacccaatgtgtgacgaattcatcaacgtaccggaatggtcttacatagtggagaaggccaacccaaccaatgacctctgttacccagggagtttcaacgactatgaagaactgaaacacctattgagcagaataaaccattttgagaaaattcagatcatccccaaaagttcttggtccgatcatgaagcctcatcaggagtgagctcagcatgtccatacctgggaagtccctccttttttagaaatgtggtatggcttatcaaaaagaacagtacgtacccaacaataaagaaaacctacaataataccaaccaagaagatcttttgatactgtggggaattcaccatcctaataatggggcagagcagacaatgctatatcaaaatccaaccacctatatttccattgggacatcaacactaaaccagagattggtaccaaaaatagctactagatccaaagtaaacgggcaaagtggaaggatggagtttttctggacaattttaaaaccaaatgatgcaatcaacttcgagagtaatggaaatttcattgctccagaatatgcatacaaaattgtcaagaaaggggactcagcaattatgaaaagtgaattggaatatggtaactgcaacaccaagtgtcaaactccaatgggggcgataaactctagtatgccattccacaacatacaccctctcaccatcggggaatgccccaaatatgtgaaatcaaacagattagtcctagcaacagggctcagaaatagccctcaaagagagagcagaagaaaaaagaga

>H5N1_A/Indonesia/7379/2008

taccatgcaaacaattcaacagagcaggttgacacaatcatggaaaagaacgttactgttacacatgcccaagacatactggaaaagacacacaacgggaagctctgcgatctagatggagtgaagcctctaattttaagagattgtagtgtagctggatggctcctcgggaacccaatgtgtgacgaattcatcaatgtaccggaatggtcttacatagtggagaaggccaatccaaccaatgacctctgttacccagggagtttcaacgactatgaagaactgaaacatctattgagcagaataaaccattttgaaaaaattcaaatcatccccaaaagttattggtccgatcatgaagcctcatcaggagtgagcgcagcatgtccatacctgggaagtccctccttttttagaaatgtggtatggcttatcaaaaagaacagtacatacccaacaataaagaaaagctacaataataccaaccaagaagatcttttggtactgtggggaattcaccatcctaatgatgcggcagagcagacaaggctatatcaaaacccaaccacctatatttccattgggacatcaacactaaaccagagattggtaccaaaaatagctactagatccaaagtaaacgggcaaagtggaaggatggagttcttctggacaattttaaaacctaatgacgcaatcaatttcgagagtaatggaaatttcattgctccagaatatgcatacaaaattgtcaagaaaggggactcagcaattatgaaaagtgaattggaatatggtaattgcaacaccaagtgtcaaactccaatgggggcgataaactctagtatgccattccataacatacaccctctcaccatcggggaatgccccaaatatgtaaagtcaaacagattagtccttgcgacagggctcagaaatagccctcaaagagagagcagaagaaaaaagaga

>H5N1_A/Indonesia/8228/2008

taccatgcaaacaattcaacggagcaggttgacacaatcatggaaaagaacgttactgttacacatgcccaagacatactggaaaagacacacaacgggaagctctgcgatctagatggagtgaagcctctaattttaaaagattgtagtgtagctggatggctcctcgggaacccaatgtgtgacgaattcatcaacgtaccagaatggtcttacatagtggagaaggccaatccaaccaatgacctctgttacccagggagtttcaacgactatgaagaactgaaacacctattgagcagaataaaccattttgagaaaattcagatcatccccaaaagttcttggtccgatcatgaagcatcatcaggagtgagctcagcatgtccatacctgggaagtccctccttttttagaaatgtggtatggcttatcaaaaagaacagtacgtacccaacaataaagaaaacctacaataataccaaccaggaagatcttttgatactgtggggaattcaccatcctaataatgaggcagagcagacaatgctatatcaaaacccaaccacctatatttccattgggacatcaacactaaaccagagattggtaccaaaaatagctactagatccaaagtaaacgggcaaagtggaaggatggagtttttctggacaattttaaaaccaaatgatgcaatcaacttcgagagtaatggaaatttcattgctccagaatatgcatacaaaattgtcaagaaaggggactcagcaattatgaaaagtgaattggaatatggtaactgcaacaccaagtgtcaaactccaatgggggcgataaactctagtatgccattccacaacatacaccctctcaccatcggggaatgccccaaatatgtgaaatcaaacagattagtcctagcaacagggctcagaaatagccctcaaagagagagcagaaggaaaaagaga

>H5N1_A/Indonesia/CDC1031/2007

taccatgcaaacaattcaacagagcaggttgacacaatcatggaaaagaacgttactgttacacatgcccaagacatactggaaaagacacacaacgggaagctctgcgatctagatggagtgaagcctctaattttaagagattgtagtgtagctggatggctcctcgggaacccaatgtgtgacgaattcatcaacgtaccggaatggtcttacatagtggagaaggccaatccaaccaatgacctctgttacccagggagtttcaacgactatgaagaactgaaacacctattgagcagaataaaccattttgagaaaattcagatcatccccaaaagttcttggtccgatcatgaagcctcatcaggagtgagctcagcatgtccatacctgggaagtccctccttttttagaaatgtggtatggcttatcaaaaagaacagtacatacccaacaataaagaaaagctacaataataccaaccaagaagatcttttggtactgtggggaattcaccatcctaataatgaggcagagcagacaaggctatatcaaaacccaaccacctatatttccattgggacatcaacactaaaccagagattggtaccaaaaatagctactagatccaaagtaaacgggcaaagtggaaggatggagtttttctggacaattttaaaaccaaatgatgcaatcaacttcgagagtaatggaaatttcattgctccagaatatgcatacaaaattgtcaagaaaggggactcagcaattatgaaaagtgaattggaatatggtaactgcaacaccaagtgtcaaactccaatgggggcgataaactctagtatgccattccacaacatacaccctctcaccatcggggaatgccccaaatatgtgaaatcaaacagattagtccttgcaacagggctcagaaatagccctcaaagagagagcagaagaaaaaagaga

>H5N1_A/Indonesia/CDC1031RE2/2007

taccatgcaaacaattcaacagagcaggttgacacaatcatggaaaagaacgttactgttacacatgcccaagacatactggaaaagacacacaacgggaagctctgcgatctagatggagtgaagcctctaattttaagagattgtagtgtagctggatggctcctcgggaacccaatgtgtgacgaattcatcaacgtaccggaatggtcttacatagtggagaaggccaatccaaccaatgacctctgttacccagggagtttcaacgactatgaagaactgaaacacctattgagcagaataaaccattttgagaaaattcagatcatccccaaaagttcttggtccgatcatgaagcctcatcaggagtgagctcagcatgtccatacctgggaagtccctccttttttagaaatgtggtatggcttatcaaaaagaacagtacatacccaacaataaagaaaagctacaataataccaaccaagaagatcttttggtactgtggggaattcaccatcctaataatgaggcagagcagacaaggctatatcaaaacccaaccacctatatttccattgggacatcaacactaaaccagagattggtaccaaaaatagctactagatccaaagtaaacgggcaaagtggaaggatggagtttttctggacaattttaaaaccaaatgatgcaatcaacttcgagagtaatggaaatttcattgctccagaatatgcatacaaaattgtcaagaaaggggactcagcaattatgaaaagtgaattggaatatggtaactgcaacaccaagtgtcaaactccaatgggggcgataaactctagtatgccattccacaacatacaccctctcaccatcggggaatgccccaaatatgtgaaatcaaacagattagtccttgcaacagggctcagaaatagccctcaaagagagagcagaagaaaaaagaga

>H5N1_A/Indonesia/CDC1031T/2007

taccatgcaaacaattcaacagagcaggttgacacaatcatggaaaagaacgttactgttacacatgcccaagacatactggaaaagacacacaacgggaagctctgcgatctagatggagtgaagcctctaattttaagagattgtagtgtagctggatggctcctcgggaacccaatgtgtgacgaattcatcaacgtaccggaatggtcttacatagtggagaaggccaatccaaccaatgacctctgttacccagggagtttcaacgactatgaagaactgaaacacctattgagcagaataaaccattttgagaaaattcagatcatccccaaaagttcttggtccgatcatgaagcctcatcaggagtgagctcagcatgtccatacctgggaagtccctccttttttagaaatgtggtatggcttatcaaaaagaacagtacatacccaacaataaagaaaagctacaataataccaaccaagaagatcttttggtactgtggggaattcaccatcctaataatgaggcagagcagacaaggctatatcaaaacccaaccacctatatttccattgggacatcaacactaaaccagagattggtaccaaaaatagctactagatccaaagtaaacgggcaaagtggaaggatggagtttttctggacaattttaaaaccaaatgatgcaatcaacttcgagagtaatggaaatttcattgctccagaatatgcatacaaaattgtcaagaaaggggactcagcaattatgaaaagtgaattggaatatggtaactgcaacaccaagtgtcaaactccaatgggggcgataaactctagtatgccattccacaacatacaccctctcaccatcggggaatgccccaaatatgtgaaatcaaacagattagtccttgcaacagggctcagaaatagccctcaaagagagagcagaagaaaaaagaga

>H5N1_A/Indonesia/CDC1031T2/2007

taccatgcaaacaattcaacagagcaggttgacacaatcatggaaaagaacgttactgttacacatgcccaagacatactggaaaagacacacaacgggaagctctgcgatctagatggagtgaagcctctaattttaagagattgtagtgtagctggatggctcctcgggaacccaatgtgtgacgaattcatcaacgtaccggaatggtcttacatagtggagaaggccaatccaaccaatgacctctgttacccagggagtttcaacgactatgaagaactgaaacacctattgagcagaataaaccattttgagaaaattcagatcatccccaaaagttcttggtccgatcatgaagcctcatcaggagtgagctcagcatgtccatacctgggaagtccctccttttttagaaatgtggtatggcttatcaaaaagaacagtacatacccaacaataaagaaaagctacaataataccaaccaagaagatcttttggtactgtggggaattcaccatcctaataatgaggcagagcagacaaggctatatcaaaacccaaccacctatatttccattgggacatcaacactaaaccagagattggtaccaaaaatagctactagatccaaagtaaacgggcaaagtggaaggatggagtttttctggacaattttaaaaccaaatgatgcaatcaacttcgagagtaatggaaatttcattgctccagaatatgcatacaaaattgtcaagaaaggggactcagcaattatgaaaagtgaattggaatatggtaactgcaacaccaagtgtcaaactccaatgggggcgataaactctagtatgccattccacaacatacaccctctcaccatcggggaatgccccaaatatgtgaaatcaaacagattagtccttgcaacagggctcagaaatagccctcaaagagagagcagaagaaaaaagaga

>H5N1_A/Indonesia/CDC1032/2007

taccatgcaaacaattcaacagagcaggttgacacaatcatggaaaagaacgttactgttacacatgcccaagacatactggaaaagacacacaacgggaagctctgcgatctagatggagtgaagcctctaattttaagagattgtagtgtagctggatggctcctcgggaacccaatgtgtgacgaattcatcaacgtaccggaatggtcttacatagtggaaaaggccaatccaaccaatgacctctgttacccagggagtttcaacgactatgaagaactgaaacacctattgagcagaataaaccattttgagaaaattcagatcatccccaaaagttcttggtccgatcatgaagcctcatcaggagtgagctcagcatgtccatacctgggaagtccctccttttttagaaatgtggtatggcttatcaaaaagaacagtacatacccaacaataaagaaaagctacaataataccaaccaagaagatcttttggtactgtggggaattcaccatcctaataatgaggaagagcagacaaggctatatcaaaacccaaccacctatatttccattgggacatcaacactaaaccagagattggtaccaaaaatagctactagatccaaagtaaacgggcaaagtgggaggatggagttcttctggacaattttaaaaccaaatgatgcaatcaacttcgagagcaatggaaatttcattgctccagaatatgcatacaaaattgtcaagaaaggggactcagcaattatgaaaagtgaattggaatatagtaactgcaacaccaagtgtcaaactccaatgggggcgataaactctagtatgccattccacaacatacaccctctcaccatcggggaatgccccaaatatgtgaaatcaagcagattagtccttgcaacagggctcagaaatagccctcaaagagagagcagaagaaaaaagaga

>H5N1_A/Indonesia/CDC1032N/2007

taccatgcaaacaattcaacagagcaggttgacacaatcatggaaaagaacgttactgttacacatgcccaagacatactggaaaagacacacaacgggaagctctgcgatctagatggagtgaagcctctaattttaagagattgtagtgtagctggatggctcctcgggaacccaatgtgtgacgaattcatcaacgtaccggaatggtcttacatagtggaaaaggccaatccaaccaatgacctctgttacccagggagtttcaacgactatgaagaactgaaacacctattgagcagaataaaccattttgagaaaattcagatcatccccaaaagttcttggtccgatcatgaagcctcatcaggagtgagctcagcatgtccatacctgggaagtccctccttttttagaaatgtggtatggcttatcaaaaagaacagtacatacccaacaataaagaaaagctacaataataccaaccaagaagatcttttggtactgtggggaattcaccatcctaataatgaggaagagcagacaaggctatatcaaaacccaaccacctatatttccattgggacatcaacactaaaccagagattggtaccaaaaacagctactagatccaaagtaaacgggcaaagtgggaggatggagttcttctggacaattttaaaaccaaatgatgcaatcaacttcgagagcaatggaaatttcattgctccagaatatgcatacaaaattgtcaagaaaggggactcagcaattatgaaaagtgaattggaatatagtaactgcaacaccaagtgtcaaactccaatgggggcgataaactctagtatgccattccacaacatacaccctctcaccatcggggaatgccccaaatatgtgaaatcaagcagattagtccttgcaacagggctcagaaatagccctcaaagagagagcagaagaaaaaagaga

>H5N1_A/Indonesia/CDC1032T/2007

taccatgcaaacaattcaacagagcaggttgacacaatcatggaaaagaacgttactgttacacatgcccaagacatactggaaaagacacacaacgggaagctctgcgatctagatggagtgaagcctctaattttaagagattgtagtgtagctggatggctcctcgggaacccaatgtgtgacgaattcatcaacgtaccggaatggtcttacatagtggaaaaggccaatccaaccaatgacctctgttacccagggagtttcaacgactatgaagaactgaaacacctattgagcagaataaaccattttgagaaaattcagatcatccccaaaagttcttggtccgatcatgaagcctcatcaggagtgagctcagcatgtccatacctgggaagtccctccttttttagaaatgtggtatggcttatcaaaaagaacagtacatacccaacaataaagaaaagctacaataataccaaccaagaagatcttttggtactgtggggaattcaccatcctaataatgaggaagagcagacaaggctatatcaaaacccaaccacctatatttccattgggacatcaacactaaaccagagattggtaccaaaaatagctactagatccaaagtaaacgggcaaagtgggaggatggagttcttctggacaattttaaaaccaaatgatgcaatcaacttcgagagcaatggaaatttcattgctccagaatatgcatacaaaattgtcaagaaaggggactcagcaattatgaaaagtgaattggaatatagtaactgcaacaccaagtgtcaaactccaatgggggcgataaactctagtatgccattccacaacatacaccctctcaccatcggggaatgccccaaatatgtgaaatcaagcagattagtccttgcaacagggctcagaaatagccctcaaagagagagcagaagaaaaaagaga

>H5N1_A/Indonesia/CDC1046/2007

taccatgcaaacaattcaacagagcaggttgacacaatcatagaaaaaaacgttactgttacacatgcccaagacatactggaaaagacacacaacgggaagctctgcgatctagatggagtgaagcctctaattttaaaagattgtagtgtagctggatggctcctcgggaacccaatgtgtgacgaattcatcaacgtaccggaatggtcttacatagtggaaaaggccaatccaaccaatgacctctgttacccagggagtttcaacgactatgaagaactgaaacacctattgagcagaataaaccattttgagaaaattcagatcatccccaaaagttcttggtccgatcatgaagcctcatcaggagtgagctcagcatgtccatacctgggaagtccctccttttttagaaatgtggtatggcttatcaaaaagaacagtacatacccaacaataaagaaaagctacaataataccaaccaagaagatcttttggtactgtggggaattcaccatcctaataatgaggaagagcagacaaggctatatcaaaacccaaccacctatatttccattgggacatcaacactaaaccagagattggtaccaaaaatagctactagatccaaagtaaacgggcaaagtgggaggatggagttcttctggacaattttaaaaccaaatgatgcaatcaatttcgagagcaatggaaatttcattgctccagaatatgcatacaaaattgtcaagaaaggggactcagcaattatgaagagtgaattggaatatagtaactgcaacaccaagtgtcaaactccaatgggggcgataaactctagtatgccattccacaacatacaccctctcaccatcggggaatgccccaaatatgtgaaatcaagcagattagtccttgcaacagggctcagaaatagccctcaaagagagagcagaagaaaaaagaga

>H5N1_A/Indonesia/CDC1046T/2007

taccatgcaaacaattcaacagagcaggttgacacaatcatggaaaaaaacgttactgttacacatgcccaagacatactggaaaagacacacaacgggaagctctgcgatctagatggagtgaagcctctaattttaaaagattgtagtgtagctggatggctcctcgggaacccaatgtgtgacgaattcatcaacgtaccggaatggtcttacatagtggaaaaggccaatccaaccaatgacctctgttacccagggagtttcaacgactatgaagaactgaaacacctattgagcagaataaaccattttgagaaaattcagatcatccccaaaagttcttggtccgatcatgaagcctcatcaggagtgagctcagcatgtccatacctgggaagtccctccttttttagaaatgtggtatggcttatcaaaaagaacagtacatacccaacaataaagaaaagctacaataataccaaccaagaagatcttttggtactgtggggaattcaccatcctaataatgaggaagagcagacaaggctatatcaaaacccaaccacctatatttccattgggacatcaacactaaaccagagattggtaccaaaaatagctactagatccaaagtaaacgggcaaagtgggaggatggagttcttctggacaattttaaaaccaaatgatgcaatcaatttcgagagcaatggaaatttcattgctccagaatatgcatacaaaattgtcaagaaaggggactcagcaattatgaagagtgaattggaatatagtaactgcaacaccaagtgtcaaactccaatgggggcgataaactctagtatgccattccacaacatacaccctctcaccatcggggaatgccccaaatatgtgaaatcaagcagattagtccttgcaacagggctcagaaatagccctcaaagagagagcagaagaaaaaagaga

>H5N1_A/Indonesia/CDC1047/2007

taccatgcaaacaattcaacagagcaggttgacacaatcatggaaaaaaacgttactgttacacatgcccaagacatactggaaaagacacacaacgggaagctctgcgatctagatggagtgaagcctctaattttaaaagattgtagtgtagctggatggctcctcgggaacccaatgtgtgacgaattcatcaacgtaccggaatggtcttacatagtggaaaaggccaatccaaccaatgacctctgttacccagggagtttcaacgactatgaagaactgaaacacctattgagcagaataaaccattttgagaaaattcagatcatccccaaaagttcttggtccgatcatgaagcctcatcaggagtgagctcagcatgtccatacctgggaagtccctccttttttagaaatgtggtatggcttatcaaaaagaacagtacatacccaacaataaagaaaagctacaataataccaaccaagaagatcttttggtactgtggggaattcaccatcctaataatgaggaagagcagacaaggctatatcaaaacccaaccacctatatttccattgggacatcaacactaaaccagagattggtaccaaaaatagctactagatccaaagtaaacgggcaaagtgggaggatggagttcttctggacaattttaaaaccaaatgatgcaatcaatttcgagagcaatggaaatttcattgctccagaatatgcatacaaaattgtcaagaaaggggactcagcaattatgaagagtgaattggaatatagtaactgcaacaccaagtgtcaaactccaatgggggcgataaactctagtatgccattccacaacatacaccctctcaccatcggggaatgccccaaatatgtgaaatcaagcagattagtccttgcaacagggctcagaaatagccctcaaagagagagcagaagaaaaaagaga

>H5N1_A/Indonesia/CDC1047S/2007

taccatgcaaacaattcaacagagcaggttgacacaatcatggaaaaaaacgttactgttacacatgcccaagacatactggaaaagacacacaacgggaagctctgcgatctagatggagtgaagcctctaattttaaaagattgtagtgtagctggatggctcctcgggaacccaatgtgtgacgaattcatcaacgtaccggaatggtcttacatagtggaaaaggccaatccaaccaatgacctctgttacccagggagtttcaacgactatgaagaactgaaacacctattgagcagaataaaccattttgagaaaattcagatcatccccaaaagttcttggtccgatcatgaagcctcatcaggagtgagctcagcatgtccatacctgggaagtccctccttttttagaaatgtggtatggcttatcaaaaagaacagtacatacccaacaataaagaaaagctacaataataccaaccaagaagatcttttggtactgtggggaattcaccatcctaataatgaggaagagcagacaaggctatatcaaaacccaaccacctatatttccattgggacatcaacactaaaccagagattggtaccaaaaatagctactagatccaaagtaaacgggcaaagtgggaggatggagttcttctggacaattttaaaaccaaatgatgcaatcaatttcgagagcaatggaaatttcattgctccagaatatgcatacaaaattgtcaagaaaggggactcagcaattatgaagagtgaattggaatatagtaactgcaacaccaagtgtcaaactccaatgggggcgataaactctagtatgccattccacaacatacaccctctcaccatcggggaatgccccaaatatgtgaaatcaagcagattagtccttgcaacagggctcagaaatagccctcaaagagagagcagaagaaaaaagaga

>H5N1_A/Indonesia/CDC184/2005

taccatgcaaacaattcaacagagcaggttgacacaatcatggaaaagaacgttactgttacacatgcccaagacatattggaaaagacacacaacgggaagctctgcgatctggatggagtgaagcctctaattttaagagattgtagtgtagctggatggctcctcgggaacccgatgtgtgacgaattcatcaatgtaccggaatggtcttacatagtggagaaggccaatccaaccaatgacctctgttacccagggagtttcaacgactatgaagaactgaaacacctattgagcagaataaaccattttgagaaaattcagatcatccccaaaagttcttggtccgatcatgaagcctcatcaggagtgagttcagcatgtccatacctgggtagtccctccttttttagaaatgtggtatggcttatcaaaaagaacagtacatacccaacaataaagaaaagctacaataataccaaccaagaagatcttttggtactgtgggggattcaccatcctaatgatggggcagagcagacaaggctatatcaaaacccaaccacctatatttccattgggacatcaacactaaaccagagattggtaccaaaaatagctactagatccaaagtaaacgggcaaagtggaaggatggagttcttctggacaattttaaaacctaatgatgcaatcaacttcgagagtaatggaaatttcattgctccagaatatgcatacaaaattgtcaagaaaggggactcagcaattatgaaaagtgaattggaatatggtaactgcaacaccaagtgtcaaactccaatgggggcgataaactctagtatgccattccacaacatacaccctctcaccatcggggaatgccccaaatatgtgaaatcaaacagattagtccttgcaacagggctcagaaatagccctcaaagagaaagcagaagaaaaaagaga

>H5N1_A/Indonesia/CDC194P/2005

taccatgcaaacaattcaacagagcaggttgacacaatcatggaaaagaacgttactgttacacatgcccaagacatattggaaaagacacacaacgggaagctctgcgatctagatggagtgaagcctctaattttaagagattgtagtgtagctggatggctcctcgggaacccaatgtgtgacgaattcatcaatgtaccggaatggtcttacatagtggagaaggccaatccaaccaatggcctctgttacccagggagtttcaacgactatgaagaactgaaacacctattgagcagaataaaccattttgagaaaattcagatcatccccaaaagttcttggtccgatcatgaagcctcatcaggagtgagctcagcatgtccatacctgggtagtccctccttttttagaaatgtggtatggcttatcaaaaagaacagtacatacccaacaataaagaaaagctacaataataccaaccaagaagatcttttggtactgtgggggattcaccatcctaatgatgcggcagagcagacaaggctatatcaaaacccaaccacctatatttccattgggacatcaacactaaaccagagattggtaccaaaaatagctactagatccaaagtaaacgggcaaagtggaaggatggagttcttctggacaattttaaaacctaatgatgcaatcaacttcgagagtaatgggaatttcattgctccagaatatgcatacaaaattgtcaagaaaggggactcagcaattatgaaaagtgaattggaatatggtaactgcaacaccaagtgtcaaactccaatgggggcgataaactctagtatgccattccacaacatacacccactcaccatcggggaatgccccaaatatgtgaaatcaaacagattagtccttgcaacagggctcagaaatagccctcaaagagaaagcagaagaaaaaagaga

>H5N1_A/Indonesia/CDC287E/2005

taccatgcaaacaattcaacagagcaggttgacacaatcatggaaaagaacgttactgttacacatgcccaagacatactggaaaagacacacaacgggaagctctgcgatctagatggagtgaagcctctaattttaagagattgtagtgtagctggatggctcctcgggaacccaatgtgtgacgaattcatcaatgtaccggaatggtcttacatagtggagaaggccaatccaaccaatgacctctgttacccaggaagtttcaacgactatgaagaactgaaacacctattgagcagaataaaccattttgagaaaattcaaatcatccccaaaagttcttggtccgatcatgaagcctcatcaggagtgagctcagcatgtccatacctgggaagtccctccttttttagaaatgtggtatggcttatcaaaaagaacagtacatacccaacaataaagaaaagctacaataataccaaccaagaagatcttttggtactgtggggaattcaccatcctaatgatgcggcagagcagacaaggctatatcaaaacccaaccacctatatttccattgggacatcaacactaaaccagagattggtaccaaaaatagctactagatccaaagtaaacgggcaaagtggaaggatggagttcttctggacaattttaaaccctaatgatgcaatcaacttcgagagtaatggaaatttcattgctccagaatatgcatacaaaattgtcaagaaaggggactcagcaattatgaaaagtgaattggaatatggtaactgcaacaccaagtgtcaaactccaatgggggcgataaactctagtatgccattccacaacatacaccctctcaccatcggggaatgccccaaatatgtgaaatcaaacagattagtccttgcaaaagggctcagaaatagccctcaaagagagagcagaagaaaaaagaga

>H5N1_A/Indonesia/CDC287T/2005

taccatgcaaacaattcaacagagcaggttgacacaatcatggaaaagaacgttactgttacacatgcccaagacatactggaaaagacacacaacgggaagctctgcgatctagatggagtgaagcctctaattttaagagattgtagtgtagctggatggctcctcgggaacccaatgtgtgacgaattcatcaatgtaccggaatggtcttacatagtggagaaggccaatccaaccaatgacctctgttacccaggaagtttcaacgactatgaagaactgaaacacctattgagcagaataaaccattttgagaaaattcaaatcatccccaaaagttcttggtccgatcatgaagcctcatcaggagtgagctcagcatgtccatacctgggaagtccctccttttttagaaatgtggtatggcttatcaaaaagaacagtacatacccaacaataaagaaaagctacaataataccaaccaagaagatcttttggtactgtggggaattcaccatcctaatgatgcggcagagcagacaaggctatatcaaaacccaaccacctatatttccattgggacatcaacactaaaccagagattggtaccaaaaatagctactagatccaaagtaaacgggcaaagtggaaggatggagttcttctggacaattttaaaccctaatgatgcaatcaacttcgagagtaatggaaatttcattgctccagaatatgcatacaaaattgtcaagaaaggggactcagcaattatgaaaagtgaattggaatatggtaactgcaacaccaagtgtcaaactccaatgggggcgataaactctagtatgccattccacaacatacaccctctcaccatcggggaatgccccaaatatgtgaaatcaaacagattagtccttgcaaaagggctcagaaatagccctcaaagagagagcagaagaaaaaagaga

>H5N1_A/Indonesia/CDC292N/2005

taccatgcaaacaattcaacagagcaggttgacacaatcatggaaaagaacgttactgttacacatgcccaagacatactggaaaagacacacaacgggaagctctgcgatctagatggagtgaagcctctaattttaagagattgtagtgtagctggatggctcctcgggaacccaatgtgtgacgaattcatcaatgtaccggaatggtcttacatagtggagaaggccaatccaaccaatgacctctgttacccagggagtttcaacgactatgaagaactgaaacacctattgagcagaataaaccattttgagaaaattcaaatcatccccaaaagttcttggtccgatcatgaagcctcatcaggagtgagctcagcatgtccatacctgggaagtccctccttttttagaaatgtggtatggcttatcaaaaagaacagtacatacccaacaataaagaaaagctacaataataccaaccaagaagatcttttggtactgtggggaattcaccatcctaatgatgcggcagagcagacaaggctatatcaaaacccaaccacctatatttccattgggacatcaacactaaaccagagattggtaccaaaaatagctactagatccaaagtaaacgggcaaagtggaaggatggagttcttctggacaattttaaaccctaatgatgcaatcaacttcgagagtaatggaaatttcattgctccagaatatgcatacaaaattgtcaagaaaggggactcagcaattatgaaaagtgaattggaatatggtaactgcaacaccaagtgtcaaactccaatgggggcgataaactctagtatgccattccacaacatacaccctctcaccatcggggaatgccccaaatatgtgaaatcaaacagattagtccttgcaacagggctcagaaatagccctcaaagagagagcagaagaaaaaagaga

>H5N1_A/Indonesia/CDC292T/2005

taccatgcaaacaattcaacagagcaggttgacacaatcatggaaaagaacgttactgttacacatgcccaagacatactggaaaagacacacaacgggaagctctgcgatctagatggagtgaagcctctaattttaagagattgtagtgtagctggatggctcctcgggaacccaatgtgtgacgaattcatcaatgtaccggaatggtcttacatagtggagaaggccaatccaaccaatgacctctgttacccagggagtttcaacgactatgaagaactgaaacacctattgagcagaataaaccattttgagaaaattcaaatcatccccaaaagttcttggtccgatcatgaagcctcatcaggagtgagctcagcatgtccatacctgggaagtccctccttttttagaaatgtggtatggcttatcaaaaagaacagtacatacccaacaataaagaaaagctacaataataccaaccaagaagatcttttggtactgtggggaattcaccatcctaatgatgcggcagagcagacaaggctatatcaaaacccaaccacctatatttccattgggacatcaacactaaaccagagattggtaccaaaaatagctactagatccaaagtaaacgggcaaagtggaaggatggagttcttctggacaattttaaaccctaatgatgcaatcaacttcgagagtaatggaaatttcattgctccagaatatgcatacaaaattgtcaagaaaggggactcagcaattatgaaaagtgaattggaatatggtaactgcaacaccaagtgtcaaactccaatgggggcgataaactctagtatgccattccacaacatacaccctctcaccatcggggaatgccccaaatatgtgaaatcaaacagattagtccttgcaacagggctcagaaatagccctcaaagagagagcagaagaaaaaagaga

>H5N1_A/Indonesia/CDC326/2006

taccatgcaaacaattcaacagagcaggttgacacaatcatggaaaagaacgttactgttacacatgcccaagacatactggaaaagacacacaacgggaagctctgcgatctagatggagtgaagcctctaattttaagagattgtagtgtagctggatggctcctcgggaacccaatgtgtgacgaattcatcaatgtaccggaatggtcttacatagtggagaaggccaatccaaccaatgacctctgttacccaggaagtttcaacgactatgaagaactgaaacacctattgagcagaataaaccattttgagaaaattcaaatcatccccaaaagttcttggtccgatcatgaagcctcatcaggagtgagctcagcatgtccatacctgggaagtccctccttttttagaaatgtggtatggcttatcaaaaagaacagtacatacccaacaataaagaaaagctacaataataccaaccaagaagatcttttggtactgtggggaattcaccatcctaatgatgcggcagagcagacaaggctatatcaaaacccaaccacctatatttccattgggacatcaacactaaaccagagattggtaccaaaaatagctactagatccaaagtaaacgggcaaagtggaaggatggagttcttctggacaattttaaaccctaacgatgcaatcaacttcgagagtaatggaaatttcattgctccagaatatgcatacaaaattgtcaagaaaggggactcagcaattatgaaaagtgaattggaatatggtaactgcaacaccaagtgtcaaactccaatgggggcgataaactctagtatgccattccacaacatacaccctctcaccatcggggaatgccccaaatacgtgaaatcaaacagattagtccttgcaacagggctcagaaatagccctcaaagagagagcagaagaaaaaagaga

>H5N1_A/Indonesia/CDC326N/2006

taccatgcaaacaattcaacagagcaggttgacacaatcatggaaaagaacgttactgttacacatgcccaagacatactggaaaagacacacaacgggaagctctgcgatctagatggagtgaagcctctaattttaagagattgtagtgtagctggatggctcctcgggaacccaatgtgtgacgaattcatcaatgtaccggaatggtcttacatagtggagaaggccaatccaaccaatgacctctgttacccaggaagtttcaacgactatgaagaactgaaacacctattgagcagaataaaccattttgagaaaattcaaatcatccccaaaagttcttggtccgatcatgaagcctcatcaggagtgagctcagcatgtccatacctgggaagtccctccttttttagaaatgtggtatggcttatcaaaaagaacagtacatacccaacaataaagaaaagctacaataataccaaccaagaagatcttttggtactgtggggaattcaccatcctaatgatgcggcagagcagacaaggctatatcaaaacccaaccacctatatttccattgggacatcaacactaaaccagagattggtaccmaaaatagctactagatccaaagtaaacgggcaaagtggaaggatggagttcttctggacaattttaaaccctaacgatgcaatcaacttcgagagtaatggaaatttcattgctccagaatatgcatacaaaattgtcaagaaaggggactcagcaattatgaaaagtgaattggaatatggtaactgcaacaccaagtgtcaaactccaatgggggcgataaactctagtatgccattccacaacatacaccctctcaccatcggggaatgccccaaatatgtgaaatcaaacagattagtccttgcaacagggctcagaaatagccctcaaagagagagcagaagaaaaaagaga

>H5N1_A/Indonesia/CDC326N2/2006

taccatgcaaacaattcaacagagcaggttgacacaatcatggaaaagaacgttactgttacacatgcccaagacatactggaaaagacacacaacgggaagctctgcgatctagatggagtgaagcctctaattttaagagattgtagtgtagctggatggctcctcgggaacccaatgtgtgacgaattcatcaatgtaccggaatggtcttacatagtggagaaggccaatccaaccaatgacctctgttacccaggaagtttcaacgactatgaagaactgaaacacctattgagcagaataaaccattttgagaaaattcaaatcatccccaaaagttcttggtccgatcatgaagcctcatcaggagtgagctcagcatgtccatacctgggaagtccctccttttttagaaatgtggtatggcttatcaaaaagaacagtacatacccaacaataaagaaaagctacaataataccaaccaagaagatcttttggtactgtggggaattcaccatcctaatgatgcggcagagcagacaaggctatatcaaaacccaaccacctatatttccattgggacatcaacactaaaccagagattggtaccaaaaatagctactagatccaaagtaaacgggcaaagtggaaggatggagttcttctggacaattttaaaccctaacgatgcaatcaacttcgagagtaatggaaatttcattgctccagaatatgcatacaaaattgtcaagaaaggggactcagcaattatgaaaagtgaattggaatatggtaactgcaacaccaagtgtcaaactccaatgggggcgataaactctagtatgccattccacaacatacaccctctcaccatcggggaatgccccaaatatgtgaaatcaaacagattagtccttgcaacagggctcagaaatagccctcaaagagagagcagaagaaaaaagaga

>H5N1_A/Indonesia/CDC326T/2006

taccatgcaaacaattcaacagagcaggttgacacaatcatggaaaagaacgttactgttacacatgcccaagacatactggaaaagacacacaacgggaagctctgcgatctagatggagtgaagcctctaattttaagagattgtagtgtagctggatggctcctcgggaacccaatgtgtgacgaattcatcaatgtaccggaatggtcttacatagtggagaaggccaatccaaccaatgacctctgttacccaggaagtttcaacgactatgaagaactgaaacacctattgagcagaataaaccattttgagaaaattcaaatcatccccaaaagttcttggtccgatcatgaagcctcatcaggagtgagctcagcatgtccatacctgggaagtccctccttttttagaaatgtggtatggcttatcaaaaagaacagtacatacccaacaataaagaaaagctacaataataccaaccaagaagatcttttggtactgtggggaattcaccatcctaatgatgcggcagagcagacaaggctatatcaaaacccaaccacctatatttccattgggacatcaacactaaaccagagattggtaccaaaaatagctactagatccaaagtaaacgggcaaagtggaaggatggagttcttctggacaattttaaaccctaacgatgcaatcaacttcgagagtaatggaaatttcattgctccagaatatgcatacaaaattgtcaagaaaggggactcagcaattatgaaaagtgaattggaatatggtaactgcaacaccaagtgtcaaactccaatgggggcgataaactctagtatgccattccacaacatacaccctctcaccatcggggaatgccccaaatacgtgaaatcaaacagattagtccttgcaacagggctcagaaatagccctcaaagagagagcagaagaaaaaagaga

>H5N1_A/Indonesia/CDC329/2006

taccatgcaaacaattcaacagagcaggttgacacaatcatggaaaagaacgttactgttacacatgcccaagacatactggaaaagacacacaacgggaagctctgcgatctagatggagtgaagcctctaattttaagagattgtagtgtagctggatggctcctcgggaacccaatgtgtgacgaattcatcaatgtaccggaatggtcttacatagtggagaaggccaatccaaccaatgacctctgttacccaggaagtttcaacgactatgaagaactgaaacacctattgagcagaataaaccattttgagaaaattcaaatcatccccaaaagttcttggtccgatcatgaagcctcatcaggagtgagctcagcatgtccatacctgggaagtccctccttttttagaaatgtggtatggcttatcaaaaagaacagtacatacccaacaataaagaaaagctacaataataccaaccaagaagatcttttggtactgtggggaattcaccatcctaatgatgcggcagagcagacaaggctatatcaaaacccaaccacctatatttccattgggacatcaacactaaaccagagattggtaccaaaaatagctactagatccaaagtaaacgggcgaagtggaaggatggagttcttctggacaattttaaaccctaacgatgcaatcaacttcgagagtaatggaaatttcattgctccagaatatgcatacaaaattgtcaagaaaggggactcagcaattatgaaaagtgaattggaatatggtaactgcaacaccaagtgtcaaactccaatgggggcgataaactctagtatgccattccacaacatacaccctctcaccatcggggaatgccccaaatatgtgaaatcaaacagattagtccttgcaacagggctcagaaatagccctcaaagagagagcagaagaaaaaagaga

>H5N1_A/Indonesia/CDC357/2006

taccatgcaaacaattcaacagagcaggttgacacaatcatggaaaagaacgttactgttacacatgcccaagacatactggaaaagacacacaacgggaagctctgcgatctagatggagtgaagcctctaattttaagagattgtagtgtagctggatggctcctcgggaacccaatgtgtgacgaattcatcaatgtaccggaatggtcttacatagtggagaaggccaatccaaccaatggcctctgttacccaggaagtttcaacgactatgaagaactgaaacacctattgagcagaataaaccattttgagaaaattcaaatcatccccaaaagttcttggtccgatcatgaagcctcatcaggagtgagctcagcatgtccatacctgggaagtccctccttttttagaaatgtggtatggcttatcaaaaagaacagtacatacccaacaataaagaaaagctacaataataccaaccaagaagatcttttggtactgtggggaattcaccatcctaatgatgcggcagagcagacaaggctatatcaaaacccaaccacctatatttccattgggacatcaacactaaaccagagattggtaccaaaaatagctactagatccaaagtaaacgggcaaagtggaaggatggagtttttctggacaattttaaaccctaatgatgcaatcaacttcgagagtaatggaaatttcattgctccagaatatgcatacaaaattgtcaagaaaggggactcagcaattatgaaaagtgaattggaatatggtaactgcaacaccaagtgtcaaactccaatgggggcgataaactctagtatgccattccacaacatacaccctctcaccatcggggaatgccccaaatatgtgaaatcaaacagattagtccttgcaacagggctcagaaatagccctcaaagagagagcagaagaaaaaagaga

>H5N1_A/Indonesia/CDC370/2006

taccatgcaaacaattcaacagagcaggttgacacaatcatggaaaagaacgttactgttacacatgcccaagacatactggaaaagacacacaacgggaagctctgcgatctagatggagtgaagcctctaattttaaaagattgtagtgtagctggatggctcctcgggaacccaatgtgtgacgaattcatcaatgtaccggaatggtcttacatagtggagaaggccaatccaaccaatgacctctgttacccagggagtttcaacgactatgaagaactgaaacacctattgagcagaataaaccattttgagaaaattcaaatcatccccaaaagttcttggtccgatcatgaagcctcatcaggagtgagctcagcatgtccatacctgggaagtccctccttttttagaaatgtggtatggcttatcaaaaagaacagtacatacccaacaataaagaaaagctacaataataccaaccaagaagatcttttggtactgtggggaattcaccaccctaatgatgcggcagagcagacaaggctatatcaaaacccaaccacctatatttccattgggacatcaacactaaaccagagattggtaccaaaaatagctactagatccaaagtaaacgggcaaagtggaaggatggagttcttctggacaattttaaaacctaatgatgcaatcaacttcgagagtaatggaaatttcattgctccagaatatgcatacaaaattgtcaagaaaggggactcagcaattatgaaaagtgaattggaatatggtaactgtaacaccaagtgtcaaactccaatgggggcgataaactctagtatgccattccacaacatacaccctctcaccatcggggaatgccccaaatatgtgaaatcaaacagattagtccttgcaacagggctcagaaatagccctcaaagagagagcagaagaaaaaagaga

>H5N1_A/Indonesia/CDC370E/2006

taccatgcaaacaattcaacagagcaggttgacacaatcatggaaaagaacgttactgttacacatgcccaagacatactggaaaagacacacaacgggaagctctgcgatctagatggagtgaagcctctaattttaaaagattgtagtgtagctggatggctcctcgggaacccaatgtgtgacgaattcatcaatgtaccggaatggtcttacatagtggagaaggccaatccaaccaatgacctctgttacccagggagtttcaacgactatgaagaactgaaacacctattgagcagaataaaccattttgagaaaattcaaatcatccccaaaagttcttggtccgatcatgaagcctcatcaggagtgagctcagcatgtccatacctgggaagtccctccttttttagaaatgtggtatggcttatcaaaaagaacagtacatacccaacaataaagaaaagctacaataataccaaccaagaagatcttttggtactgtggggaattcaccaccctaatgatgcggcagagcagacaaggctatatcaaaacccaaccacctatatttccattgggacatcaacactaaaccagagattggtaccaaaaatagctactagatccaaagtaaacgggcaaagtggaaggatggagttcttctggacaattttaaaacctaatgatgcaatcaacttcgagagtaatggaaatttcattgctccagaatatgcatacaaaattgtcaagaaaggggactcagcaattatgaaaagtgaattggaatatggtaactgtaacaccaagtgtcaaactccaatgggggcgataaactctagtatgccattccacaacatacaccctctcaccatcggggaatgccccaaatatgtgaaatcaaacagattagtccttgcaacagggctcagaaatagccctcaaagagagagcagaagaaaaaagaga

>H5N1_A/Indonesia/CDC370P/2006

taccatgcaaacaattcaacagagcaggttgacacaatcatggaaaagaacgttactgttacacatgcccaagacatactggaaaagacacacaacgggaagctctgcgatctagatggagtgaagcctctaattttaaaagattgtagtgtagctggatggctcctcgggaacccaatgtgtgacgaattcatcaatgtaccggaatggtcttacatagtggagaaggccaatccaaccaatgacctctgttacccagggagtttcaacgactatgaagaactgaaacacctattgagcagaataaaccattttgagaaaattcaaatcatccccaaaagttcttggtccgatcatgaagcctcatcaggagtgagctcagcatgtccatacctgggaagtccctccttttttagaaatgtggtatggcttatcaaaaagaacagtacatacccaacaataaagaaaagctacaataataccaaccaagaagatcttttggtactgtggggaattcaccaccctaatgatgcggcagagcagacaaggctatatcaaaacccaaccacctatatttccattgggacatcaacactaaaccagagattggtaccaaaaatagctactagatccaaagtaaacgggcaaagtggaaggatggagttcttctggacaattttaaaacctaatgatgcaatcaacttcgagagtaatggaaatttcattgctccagaatatgcatacaaaattgtcaagaaaggggactcagcaattatgaaaagtgaattggaatatggtaactgtaacaccaagtgtcaaactccaatgggggcgataaactctagtatgccattccacaacatacaccctctcaccatcggggaatgccccaaatatgtgaaatcaaacagattagtccttgcaacagggctcagaaatagccctcaaagagagagcagaagaaaaaagaga

>H5N1_A/Indonesia/CDC370T/2006

taccatgcaaacaattcaacagagcaggttgacacaatcatggaaaagaacgttactgttacacatgcccaagacatactggaaaagacacacaacgggaagctctgcgatctagatggagtgaagcctctaattttaaaagattgtagtgtagctggatggctcctcgggaacccaatgtgtgacgaattcatcaatgtaccggaatggtcttacatagtggagaaggccaacccaaccaatgacctctgttacccagggagtttcaacgactatgaagaactgaaacacctattgagcagaataaaccattttgagaaaattcaaatcatccccaaaagttcttggtccgatcatgaagcctcatcaggagtgagctcagcatgtccatacctgggaagtccctccttttttagaaatgtggtatggcttatcaaaaagaacagtacatacccaacaataaagaaaagctacaataataccaaccaagaagatcttttggtactgtggggaattcaccaccctaatgatgcggcagagcagacaaggctatatcaaaacccaaccacctatatttccattgggacatcaacactaaaccagagattggtaccaaaaatagctactagatccaaagtaaacgggcaaagtggaaggatggagttcttctggacaattttaaaacctaatgatgcaatcaacttcgagagtaatggaaatttcattgctccagaatatgcatacaaaattgtcaagaaaggggactcagcaattatgaaaagtgaattggaatatggtaactgtaacaccaagtgtcaaactccaatgggggcgataaactctagtatgccattccacaacatacaccctctcaccatcggggaatgccccaaatatgtgaaatcaaacagattagtccttgcaacagggctcagaaatagccctcaaagagagagcagaagaaaaaagaga

>H5N1_A/Indonesia/CDC390/2006

taccatgcaaacaattcaacagagcaggttgacacaatcatggaaaagaacgttactgttacacatgcccaagacatactggaaaagacacacaacgggaagctctgcgatctagatggagtgaagcctctaattttaaaagattgtagtgtagctggatggcttctcgggaacccaatgtgtgacgaattcatcaatgtaccggaatggtcttacatagtggagaaggccaatccaaccaatgacctctgttacccagggagtttcaacgactatgaagaactgaaacacctattgagcagaataaaccattttgagaaaattcaaatcatccccaaaagttcttggtccgatcatgaagcctcatcaggagtgagctcagcatgtccatatctgggaagtccctccttttttagaaatgtggtatggcttatcaaaaagaacagtacatacccaacaataaaggaaagctacaataataccaaccaagaagatcttttggtactgtggggaattcaccatcctaatgatgcggcagagcagacaaggctatatcaaaacccaaccacctatatttccattgggacatcaacactcaaccagagattggtaccaaaaatagctactagatccaaagtaaacgggcaaagtggaaggatggagttcttctggacaattttaaaacctaatgatgcaatcaacttcgagagtaatggaaatttcattgctccagaatatgcatacaaaattgtcaagaaaggggactcagcaattatgaaaagtgaattggaatatggtaactgcaacaccaagtgtcaaactccaatgggggcgataaactctagtatgccattccacaacatacaccctctcaccatcggggaatgccccaaatatgtgaaatcaaacaaattagtccttgcaacagggctcagaaatagccctcaaagagagagcagaagaaaaaagaga

>H5N1_A/Indonesia/CDC523/2006

taccatgcaaacaattcaacagagcaggttgacacaatcatggaaaagaacgttactgttacacatgcccaagacatactggaaaagacacacaacgggaagctctgcgatctagatggagtgaagcctctaattttaagagattgtagtgtagctggatggctcctcgggaacccaatgtgtgacgaattcatcaatgtaccggaatggtcttacatagtggagaaggccaatccaaccaatgacctctgttacccagggagtttcaacgactatgaagaactgaaacacctattgagcagaataaaccattttgagaaaattcaaatcatccccaaaagttcttggtccgatcatgaagcctcatcaggagtgagctcagcatgtccatacctgggaagtccctccttttttagaaatgtggtatggcttatcaaaaagaacagtacatacccaacaataaagaaaagytacaataataccaaccaagaagatcttttggtactgtggggaattcaccatcctaataatgcggcagagcagacaaggctatatcaaaacccaaccacctatatttccattgggacatcaacactaaaccagagattggtaccaaaaatagctactagatccaaagtaaacgggcaaagtggaaggatggagttcttctggacaattttaaaacctaatgatgcaatcaacttcgagagtaatggaaatttcattgctccagaatatgcatacaaaattgtcaagaaaggggactcagcaattatgaaaagtgaattggaatatggtaactgcaacaccaagtgtcaaactccaatgggggcgataaactctagtatgccattccacaacatacaccctctcaccatcggggaatgccccaaatatgtgaaatcaaacagattagtccttgcaacagggctcagaaatagccctcaaagagagagcagaagaaaaaagaga

>H5N1_A/Indonesia/CDC523E/2006

taccatgcaaacaattcaacagagcaggttgacacaatcatggaaaagaacgttactgttacacatgcccaagacatactggaaaagacacacaacgggaagctctgcgatctagatggagtgaagcctctaattttaagagattgtagtgtagctggatggctcctcgggaacccaatgtgtgatgaattcatcaatgtaccggaatggtcttacatagtggagaaggccaatccaaccaatgacctctgttacccagggagtttcaacgactatgaagaactgaaacacctattgagcagaataaaccattttgagaaaattcaaatcatccccaaaagttcttggtccgatcatgaagcctcatcaggagtgagctcagcatgtccatacctgggaagtccctccttttttagaaatgtggtatggcttatcaaaaagaacagtacatacccaacaataaagaaaagctacaataataccaaccaagaagatcttttggtactgtggggaattcaccatcctaataatgcggcagagcagacaaggctatatcaaaacccaaccacctatatttccattgggacatcaacactaaaccagagattggtaccaaaaatagctactagatccaaagtaaacgggcaaagtggaaggatggagttcttctggacaattttaaaacctaatgatgcaatcaacttcgagagtaatggaaatttcattgctccagaatatgcatacaaaattgtcaagaaaggggactcagcaattatgaaaagtgaattggaatatggtaactgcaacaccaagtgtcaaactccaatgggggcgataaactctagtatgccattccacaacatacaccctctcaccatcggggaatgccccaaatatgtgaaatcaaacagattagtccttgcaacagggctcagaaatagccctcaaagagagagcagaagaaaaaagaga

>H5N1_A/Indonesia/CDC523T/2006

taccatgcaaacaattcaacagagcaggttgacacaatcatggaaaagaacgttactgttacacatgcccaagacatactggaaaagacacacaacgggaagctctgcgatctagatggagtgaagcctctaattttaagagattgtagtgtagctggatggctcctcgggaacccaatgtgtgatgaattcatcaatgtaccggaatggtcttacatagtggagaaggccaatccaaccaatgacctctgttacccagggagtttcaacgactatgaagaactgaaacacctattgagcagaataaaccattttgagaaaattcaaatcatccccaaaagttcttggtccgatcatgaagcctcatcaggagtgagctcagcatgtccatacctgggaagtccctccttttttagaaatgtggtatggcttatcaaaaagaacagtacatacccaacaataaagaaaagctacaataataccaaccaagaagatcttttggtactgtggggaattcaccatcctaataatgcggcagagcagacaaggctatatcaaaacccaaccacctatatttccattgggacatcaacactaaaccagagattggtaccaaaaatagctactagatccaaagtaaacgggcaaagtggaaggatggagttcttctggacaattttaaaacctaatgatgcaatcaacttcgagagtaatggaaatttcattgctccagaatatgcatacaaaattgtcaagaaaggggactcagcaattatgaaaagtgaattggaatatggtaactgcaacaccaagtgtcaaactccaatgggggcgataaactctagtatgccattccacaacatacaccctctcaccatcggggaatgccccaaatatgtgaaatcaaacagattagtccttgcaacagggctcagaaatagccctcaaagagagagcagaagaaaaaagaga

>H5N1_A/Indonesia/CDC582/2006

taccatgcaaacaattcaacagagcaggttgacacaatcatggaaaagaacgttactgttacacatgcccaagacatactggaaaagacacacaacgggaagctctgcgatctagatagagtgaagcctctaattttaagagattgtagtgtagctggatggctcctcgggaacccaatgtgtgacgaattcatcaatgtaccggaatggtcttacatagtggagaaggccaatccaaccaatgacctctgttacccagggagtttcaacgactatgaagaactgaaacacctattgagcagaataaaccattttgagaaaattcaaatcatccccaaaagttcttggtccgatcatgaagcctcatcaggagtgagctcagcatgtccatacctgggaagtccctccttttttagaaatgtggtatggcttatcaaaaagaacaggacatacccaacaataaagaaaagctacaataataccaaccaagaagatcttttggtactgtggggaattcaccatcctaatgatgcggcagagcagacaaggctatatcaaaacccaaccacctatatttccattgggacatcaacactaaaccagagattggtaccaaaaatagctactagatccaaagtaaacgggcaaagtggaaggatggagttcttctggacaattttaaagcctaatgatgcaatcaacttcgagagtaatggaaatttcattgctccagaatatgcatacaaaattgtcaagaaaggggactcagcaattatgaaaagtgaattagaatatggtaattgcaacaccaagtgtcaaactccaatgggggcgataaactctagtatgccattccacaacatacaccctctcaccatcggggaatgccccaaatatgtgaaatcaaacagattagtccttgcaacagggctcagaaatagccctcaaagagagagcagaagaaaaaagaga

>H5N1_A/Indonesia/CDC594/2006

taccatgcaaacaattcaacagagcaggttgacacaataatggaaaagaacgtcactgtcacacatgcccaagacatactggaaaagacacacaacgggaagctctgcgatctagatggagtgaagcctctaattttaagagattgtagtgtagctggatggctcctcgggaacccaatgtgtgacgaattcatcaatgtaccggaatggtcttacatagtggagaaggccaatccagccaatggcctctgttacccagggaatttcaacgactatgaagaactgaaacacctattgagcagaataaatcattttgaaaaacttcagataatccccaaaagttcttggtccgatcatgaagcctcattaggggtgagctcagcatgtccatacctgggaaggtcctccttctttagaaatgtggtatggcttatcaaaaagaacaatacatacccaacaataaagagaagctacaataataccaaccaagaagatcttttggtactgtgggggattcaccatcctaatgatgcagcagagcagacaaggctatatcaaaacccaaccacttatatttccgttgggacatcaacactaaaccagagattggtaccaaaaatagctactagatccaaagtaaatgggcaaagtggaaggatggaattcttctggacaattttaaaaccgaatgatgcaatcaacttcgagagtaatggaaatttcattgctccagaatatgcatacaaaattgtaaagaaaggggactctgcaattatgaaaagtgaattggaatatggtgactgcaacaccaagtgtcaaactccaatgggggcgataaactctagtatgccattccacaacatacaccctctcaccatcggggaatgccccaaatatgtgaagtcaaacagattagtccttgcgactgggctcagaaatagccctcaaagagagagaagaagaaaaaagaga

>H5N1_A/Indonesia/CDC595/2006

taccatgcaaacaattcaacagagcaggttgacacaataatggaaaagaacgtcactgtcacacatgcccaagacatactggaaaagacacacaacgggaagctctgcgatctagatggagtgaagcctctaattttaagagattgtagtgtagctggatggctcctcgggaacccaatgtgtgacgaattcatcaatgtaccggaatggtcttacatagtggagaaggccaatccagccaatggcctctgttacccagggaatttcaacgactatgaagaactgaaacacctattgagcagaataaatcattttgaaaaacttcagataatccccaaaagttcttggtccgatcatgaagcctcattaggggtgagctcagcatgtccatacctgggaaggtcctccttctttagaaatgtggtatggcttatcaaaaagaacaatacatacccaacaataaagagaagctacaataataccaaccaagaagatcttttggtactgtgggggattcaccatcctaatgatgcagcagagcagacaaggctatatcaaaacccaaccacttatatttccgttgggacatcaacactaaaccagagattggtaccaaaaatagctactagatccaaagtaaatgggcaaagtggaaggatggaattcttctggacaattttaaaaccgaatgatgcaatcaacttcgagagtaatggaaatttcattgctccagaatatgcatacaaaattgtaaagaaaggggactctgcaattatgaaaagtgaattggaatatggtgactgcaacaccaagtgtcaaactccaatgggggcgataaactctagtatgccactccacaacatacaccctctcaccatcggggaatgccccaaatatgtgaagtcaaacagattagtccttgcgactgggctcagaaatagccctcaaagagagagaagaagaaaaaagaga

>H5N1_A/Indonesia/CDC596/2006

taccatgcaaacaattcaacagagcaggttgacacaataatggaaaagaacgtcactgtcacacatgcccaagacatactggaaaagacacacaacgggaagctctgcgatctagatggagtgaagcctctaattttaagagattgtagtgtagctggatggctcctcgggaacccaatgtgtgacgaattcatcaatgtaccggaatggtcttacatagtggagaaggccaatccagccaatggcctctgttacccagggaatttcaacgactatgaagaactgaaacacctattgagcagaataaatcattttgaaaaacttcagataatccccaaaagttcttggtccgatcatgaagcctcattaggggtgagctcagcatgtccatacctgggaaggtcctccttctttagaaatgtggtatggcttatcaaaaagaacaatacatacccaacaataaagagaagctacaataataccaaccaagaagatcttttggtactgtgggggattcaccatcctaatgatgcagcagagcagacaaggctatatcaaaacccaaccacttatatttccgttgggacatcaacactaaaccagagattggtaccaaaaatagctactagatccaaagtaaatgggcaaagtggaaggatggaattcttctggacaattttaaaaccgaatgatgcaatcaacttcgagagtaatggaaatttcattgctccagaatatgcatacaaaattgtaaagaaaggggactctgcaattatgaaaagtgaattggaatatggtgactgcaacaccaagtgtcaaactccaatgggggcgataaactctagtatgccattccacaacatacaccctctcaccatcggggaatgccccaaatatgtgaagtcaaacagattagtccttgcgactgggctcagaaatagccctcaaagagagagaagaagaaaaaagaga

>H5N1_A/Indonesia/CDC597/2006

taccatgcaaacaattcaacagagcaggttgacacaataatggaaaagaacgtcactgtcacacatgcccaagacatactggaaaagacacacaacgggaagctctgcgatctagatggagtgaagcctctaattttaagagattgtagtgtagctggatggctcctcgggaacccaatgtgtgacgaattcatcaatgtaccggaatggtcttacatagtggagaaggccaatccagccaatggcctctgttacccagggaatttcaacgactatgaagaactgaaacacctattgagcagaataaatcattttgaaaaacttcagataatccccaaaagttcttggtccgatcatgaagcctcattaggggtgagctcagcatgtccatacctgggaaggtcctccttctttagaaatgtggtatggcttatcaaaaagaacaatacatacccaacaataaagagaagctacaataataccaaccaagaagatcttttggtactgtgggggattcaccatcctaatgatgcagcagagcagacaaggctatatcaaaacccaaccacttatatttccgttgggacatcaacactaaaccagagattggtaccaaaaatagctactagatccaaagtaaatgggcaaagtggaaggatggaattcttctggacaattttaaaaccgaatgatgcaatcaacttcgagagtaatggaaatttcattgctccagaatatgcatacaaaattgtaaagaaaggggactctgcaattatgaaaagtgaattggaatatggtgactgcaacaccaagtgtcaaactccaatgggggcgataaactctagtatgccattccacaacatacaccctctcaccatcggggaatgccccaaatatgtgaagtcaaacagattagtccttgcgactgggctcagaaatagccctcaaagagagagaagaagaaaaaagaga

>H5N1_A/Indonesia/CDC599/2006

taccatgcaaacaattcaacagagcaggttgacacaataatggaaaagaacgtcactgtcacacatgcccaagacatactggaaaagacacacaacgggaagctctgcgatctagatggagtgaagcctctaattttaagagattgtagtgtagctggatggctcctcgggaacccaatgtgtgacgaattcatcaatgtaccggaatggtcttacatagtggagaaggccaatccagccaatggcctctgttacccagggaatttcaacgactatgaagaactgaaacacctattgagcagaataaatcattttgaaaaacttcagataatccccaaaagttcttggtccgatcatgaagcctcattaggggtgagctcagcatgtccatacctgggaaggtcctccttctttagaaatgtggtatggcttatcaaaaagaacaatacatacccaacaataaagagaagctacaataataccaaccaagaagatcttttggtactgtgggggattcaccatcctaatgatgcagcagagcagacaaggctatatcaaaacccaaccacttatatttccgttgggacatcaacactaaaccagagattggtaccaaaaatagctactagatccaaagtaaatgggcaaagtggaaggatggaattcttctggacaattttaaaaccgaatgatgcaatcaacttcgagagtaatggaaatttcattgctccagaatatgcatacaaaattgtaaagaaaggggactctgcaattatgaaaagtgaattggaatatggtgactgcaacaccaagtgtcaaactccaatgggggcgataaactctagtatgccattccacaacatacaccctctcaccatcggggaatgccccaaatatgtgaagtcaaacagattagtccttgcgactgggctcagaaatagccctcaaagagagagaagaagaaaaaagaga

>H5N1_A/Indonesia/CDC599N/2006

taccatgcaaacaattcaacagagcaggttgacacaataatggaaaagaacgtcactgtcacacatgcccaagacatactggaaaagacacacaacgggaagctctgcgatctagatggagtgaagcctctaattttaagagattgtagtgtagctggatggctcctcgggaacccaatgtgtgacgaattcatcaatgtaccggaatggtcttacatagtggagaaggccaatccagccaatggcctctgttacccagggaatttcaacgactatgaagaactgaaacacctattgagcagaataaatcattttgaaaaacttcagataatccccaaaagttcttggtccgatcatgaagcctcattaggggtgagctcagcatgtccatacctgggaaggtcctccttctttagaaatgtggtatggcttatcaaaaagaacaatacatacccaacaataaagagaagctacaataataccaaccaagaagatcttttggtactgtgggggattcaccatcctaatgatgcagcagagcagacaaggctatatcaaaacccaaccacttatatttccgttgggacatcaacactaaaccagagattggtaccaaaaatagctactagatccaaagtaaatgggcaaagtggaaggatggaattcttctggacaattttaaaaccgaatgatgcaatcaacttcgagagtaatggaaatttcattgctccagaatatgcatacaaaattgtaaagaaaggggactctgcaattatgaaaagtgaattggaatatggtgactgcaacaccaagtgtcaaactccaatgggggcgataaactctagtatgccattccacaacatacaccctctcaccatcggggaatgccccaaatatgtgaagtcaaacagattagtccttgcgactgggctcagaaatagccctcaaagagagagaagaagaaaaaagaga

>H5N1_A/Indonesia/CDC610/2006

taccatgcaaacaattcaacagagcaggttgacacaatcatggaaaagaacgttactgttacacatgcccaagacatactggaaaagacacacaacgggaagctctgcgatctagatggagtgaagcctctaattttaagagattgtagtgtagctggatggctcctcgggaacccaatgtgtgacgaattcatcaatgtaccggaatggtcttacatagtggagaaggccaatccaaccaatgacctctgttacccagggagtttcaacgactatgaagaactgaaacatctattgagcagaataaaccattttgagaaaattcaaatcatccccaaaagttcttggtccgatcatgaagcctcatcaggagtgagctcagcatgtccatacctgggaagtccctccttttttagaaatgtggtatggcttatcaaaaagaacagtacatacccaacaataaagaaaagctacaataataccaaccaagaagatcttttggtactgtggggaattcaccatcctaatgatgcggcagagcagacaaggctatatcaaaacccaaccacctatatttccattgggacatcaacactaaaccagagattggtaccaaaaatagctactagatccaaagtaaacgggcaaagtggaaggatggagttcttctggacaattttaaaacctaatgatgcaatcaacttcgagagtaatggaaatttcattgctccagaatatgcatacaaaattgtcaagaaaggggactcagcaattatgaaaagtgaattggaatatggtaactgcaacaccaagtgtcaaactccaatgggggcgataaactctagtatgccattccacaacatacaccctctcaccatcggggaatgccccaaatatgtgaaatcaaacagattagtccttgcaacagggctcagaaatagccctcaaagagagagcagaagaaaaaagaga

>H5N1_A/Indonesia/CDC623/2006

taccatgcaaacaattcaacagagcaggttgacacaatcatggaaaagaacgttactgttacacatgcccaagacatactggaaaagacacacaacgggaagctctgcgacctagacggagtgaagcctctaattttaagagattgtagtgtagctggatggctcctcgggaacccaatgtgcgacgaattcatcaatgtaccggaatggtcttacatagtggagaaggccagtccaaccaaagacctctgttacccagggagtttcaacgactatgaagaactgaaacacctattgagcagaataaaccattttgagaaaattcaaatcatccccaaaagttcttggtctgaccatgaagcctcatcaggagtgagctcagcatgtccatacctgggaagtccctccttttttagaaatgtggtatggcttatcaaaaagaacagtacatacccaacaataaagaaaagctacaataataccaaccaagaagatcttttggtactgtggggaattcaccatcctaatgatgcggcagagcagacaaggctatatcaaaacccaaccacctatatttccattgggacatcaacactaaaccagagattggtaccaaaaatagctactagatccaaagtaaacgggcaaagtggaaggatggagttcttctggacaattttaaaacctaatgatgcaatcaacttcgagagtaatggaaatttcattgctccagaatatgcatacaaaattgtcaagaaaggggactcagcaattatgaaaagtgaattggaatatggtaactgcaacaccaagtgtcaaactccaatgggggcgataaactctagtatgccattccacaacatacaccctctcaccatcggggaatgccccaaatatgtgaaatcaaacagattagtccttgcaacagggctcagaaatagccctcaaagagagagcagaagaaaaaagaga

>H5N1_A/Indonesia/CDC623E/2006

taccatgcaaacaattcaacagagcaggttgacacaatcatggaaaagaacgttactgttacacatgcccaagacatactggaaaagacacacaacgggaagctctgcgacctagacggagtgaagcctctaattttaagagattgtagtgtagctggatggctcctcgggaacccaatgtgcgacgaattcatcaatgtaccggaatggtcttacatagtggagaaggccagtccaaccaaagacctctgttacccagggagtttcaacgactatgaagaactgaaacacctattgagcagaataaaccattttgagaaaattcaaatcatccccaaaagttcttggtctgaccatgaagcctcatcaggagtgagctcagcatgtccatacctgggaagtccctccttttttagaaatgtggtatggcttatcaaaaagaacagtacatacccaacaataaagaaaagctacaataataccaaccaagaagatcttttggtactgtggggaattcaccatcctaatgatgcggcagagcagacaaggctatatcaaaacccaaccacctatatttccattgggacatcaacactaaaccagagattggtaccaaaaatagctactagatccaaagtaaacgggcaaagtggaaggatggagttcttctggacaattttaaaacctaatgatgcaatcaacttcgagagtaatggaaatttcattgctccagaatatgcatacaaaattgtcaagaaaggggactcagcaattatgaaaagtgaattggaatatggtaactgcaacaccaagtgtcaaactccaatgggggcgataaactctagtatgccattccacaacatacaccctctcaccatcggggaatgccccaaatatgtgaaatcaaacagattagtccttgcaacagggctcagaaatagccctcaaagagagagcagaagaaaaaagaga

>H5N1_A/Indonesia/CDC624/2006

taccatgcaaacaattcaacagagcaggttgacacaatcatggaaaagaacgttactgttacacatgcccaagacatactggaaaagacacacaatgggaagctctgcgatctagatggagtgaagcctctaattttaagagattgtagtgtagctggatggctcctcgggaacccaatgtgtgacgaattcatcaatgtaccggaatggtcttacatagtggagaaggccaatccaaccaatgacctctgttacccagggagtttcaacgactatgaagaactgaaacacctattgagcagaataaaccattttgagaaaattcaaatcatccccaaaagttcttggtccgaccatgaagcctcatcaggagtgagctcagcatgtccatacctgggaagtccctccttttttagaaatgtggtatggcttatcaaaaagaacagtacatacccaacaataaagaaaagctacaataataccaaccaagaagatcttttggtactgtggggaattcaccatcctaatgatgcggcagagcagacaaggctatatcaaaacccaaccacctatatttccattgggacatcaacactaaaccagagattggtaccaaaaatagctactagatccaaagtaaacgggcaaagtggaaggatggagttcttctggacaattttaaaacctaatgatgcaatcaacttcgagagtaatggaaatttcattgctccagaatatgcatacaaaattgtcaagaaaggggactcagcaattatgaaaagtgaattggaatatggtaactgcaacaccaagtgtcaaactccgatgggggcgataaactctagtatgccattccacaacatacaccctctcaccatcggggaatgccccaaatatgtgaaatcaaacagattagtccttgcaacagggctcagaaatagccctcaaagagagagcagaagaaaaaagaga

>H5N1_A/Indonesia/CDC624E/2006

taccatgcaaacaattcaacagagcaggttgacacaatcatggaaaagaacgttactgttacacatgcccaagacatactggaaaagacacacaatgggaagctctgcgatctagatggagtgaagcctctaattttaagagattgtagtgtagctggatggctcctcgggaacccaatgtgtgacgaattcatcaatgtaccggaatggtcttacatagtggagaaggccaatccaaccaatgacctctgttacccagggagtttcaacgactatgaagaactgaaacacctattgagcagaataaaccattttgagaaaattcaaatcatccccaaaagttcttggtccgaccatgaagcctcatcaggagtgagctcagcatgtccatacctgggaagtccctccttttttagaaatgtggtatggcttatcaaaaagaacagtacatacccaacaataaagaaaagctacaataataccaaccaagaagatcttttggtactgtggggaattcaccatcctaatgatgcggcagagcagacaaggctatatcaaaacccaaccacctatatttccattgggacatcaacactaaaccagagattggtaccaaaaatagctactagatccaaagtaaacgggcaaagtggaaggatggagttcttctggacaattttaaaacctaatgatgcaatcaacttcgagagtaatggaaatttcattgctccagaatatgcatacaaaattgtcaagaaaggggactcagcaattatgaaaagtgaattggaatatggtaactgcaacaccaagtgtcaaactccgatgggggcgataaactctagtatgccattccacaacatacaccctctcaccatcggggaatgccccaaatatgtgaaatcaaacagattagtccttgcaacagggctcagaaatagccctcaaagagagagcagaagaaaaaagaga

>H5N1_A/Indonesia/CDC625/2006

taccatgcaaacaattcaacagagcaggttgacacaataatggaaaagaacgtcactgtcacacatgcccaagacatactggaaaagacacacaacgggaagctctgcgatctagatggagtgaagcctctaattttaagagattgtagtgtagctggatggctcctcgggaacccaatgtgtgacgaattcatcaatgtaccggaatggtcttacatagtggagaaggccaatccagccaatggcctctgttacccagggaatttcaacgactatgaagaactgaaacacctattgagcagaataaatcattttgaaaaacttcagataatccccaaaagttcttggtccgatcatgaagcctcattaggggtgagctcagcatgtccatacctgggaaggtcctccttctttagaaatgtggtatggcttatcaaaaagaacaatacatacccaacaataaagagaagctacaataataccaaccaagaagatcttttggtactgtgggggcttcaccatcctaatgatgcagcagagcagacaaggctatatcaaaacccaaccacttatatttccgttgggacatcaacactaaaccagagattggtaccaaaaatagctactagatccaaagtaaatgggcaaagtggaaggatggaattcttctggacaattttaaaaccgaatgatgcaatcaacttcgagagtaatggaaatttcattgctccagaatatgcatacaaaattgtaaagaaaggggactctgcaattatgaaaagtgaattggaatatggtgactgcaacaccaagtgtcaaactccaatgggggcgataaactctagtatgccattccacaacatacaccctctcaccatcggggaatgccccaaatatgtgaagtcaaacagattagtccttgcgactgggctcagaaatagccctcaaagagagagaagaagaaaaaagaga

>H5N1_A/Indonesia/CDC625L/2006

taccatgcaaacaattcaacagagcaggttgacacaataatggaaaagaacgtcactgtcacacatgcccaagacatactggaaaagacacacaacgggaagctctgcgatctagatggagtgaagcctctaattttaagagattgtagtgtagctggatggctcctcgggaacccaatgtgtgacgaattcatcaatgtaccggaatggtcttacatagtggagaaggccaatccagccaatggcctctgttacccagggaatttcaacgactatgaagaactgaaacacctattgagcagaataaatcattttgaaaaacttcagataatccccaaaagttcttggtccgatcatgaagcctcattaggggtgagctcagcatgtccatacctgggaaggtcctccttctttagaaatgtggtatggcttatcaaaaagaacaatacatacccaacaataaagagaagctacaataataccaaccaagaagatcttttggtactgtgggggcttcaccatcctaatgatgcagcagagcagacaaggctatatcaaaacccaaccacttatatttccgttgggacatcaacactaaaccagagattggtaccaaaaatagctactagatccaaagtaaatgggcaaagtggaaggatggaattcttctggacaattttaaaaccgaatgatgcaatcaacttcgagagtaatggaaatttcattgctccagaatatgcatacaaaattgtaaagaaaggggactctgcaattatgaaaagtgaattggaatatggtgactgcaacaccaagtgtcaaactccaatgggggcgataaactctagtatgccattccacaacatacaccctctcaccatcggggaatgccccaaatatgtgaagtcaaacagattagtccttgcgactgggctcagaaatagccctcaaagagagagaagaagaaaaaagaga

>H5N1_A/Indonesia/CDC634/2006

taccatgcaaacaattcaacagagcaggttgacacaatcatggaaaagaacgttactgttacacatgcccaagacatactggaaaagacacacaacgggaagctctgcgatctagatggagtgaagcctctaattttaagagattgtagtgtagctggatggctcctcgggaacccaatgtgtgacgaattcatcaatgtaccggaatggtcttacatagtggagaaggccaatccaaccaatgacctctgttacccagggagtttcaacgactatgaagaactgaaacacctattgagcagaataaaccattttgagaaaattcaaatcatccccaaaagttcttggtccgatcatgaagcctcatcaggagtgagctcagcatgtccatacctgggaagtccctccttttttagaaatgtggtatggcttatcaaaaagaacagtacatacccaacaataaagaaaagctacaataataccaaccaagaagatcttttggtactgtggggaattcaccatcctaatgatgcggcagagcagacaaggctatatcaaaacccaaccacctatatttccattgggacatcaacactaaaccagagattggtaccaaaaatggctactagatccaaagtaaacgggcaaagtggaaggatggagttcttctggacaattttaaaacctaatgatgcaatcaacttcgagagtaatggaaatttcattgctccagaatatgcatacaaaattgtcaagaaaggggactcagcaattatgaaaagtgaattggaatatggtaactgcaacaccaagtgtcaaactccaatgggggcgataaactctagtatgccattccacaacatacaccctctcaccatcggggaatgccccaaatatgtgaaatcaaacagattagtccttgcaacagggctcagaaatagccctcaaagagagagcagaagaaaaaagaga

>H5N1_A/Indonesia/CDC634P/2006

taccatgcaaacaattcaacagagcaggttgacacaatcatggaaaagaacgttactgttacacatgcccaagacatactggaaaagacacacaacgggaagctctgcgatctagatggagtgaagcctctaattttaagagattgtagtgtagctggatggctcctcgggaacccaatgtgtgacgaattcatcaatgtaccggaatggtcttacatagtggagaaggccaatccaaccaatgacctctgttacccagggagtttcaacgactatgaagaactgaaacacctattgagcagaataaaccattttgagaaaattcaaatcatccccaaaagttcttggtccgatcatgaagcctcatcaggagtgagctcagcatgtccatacctgggaagtccctccttttttagaaatgtggtatggcttatcaaaaagaacagtacatacccaacaataaagaaaagctacaataataccaaccaagaagatcttttggtactgtggggaattcaccatcctaatgatgcggcagagcagacaaggctatatcaaaacccaaccacctatatttccattgggacatcaacactaaaccagagattggtaccaaaaatggctactagatccaaagtaaacgggcaaagtggaaggatggagttcttctggacaattttaaaacctaatgatgcaatcaacttcgagagtaatggaaatttcattgctccagaatatgcatacaaagttgtcaagaaaggggactcagcaattatgaaaagtgaattggaatatggtaactgcaacaccaagtgtcaaactccaatgggggcgataaactctagtatgccattccacaacatacaccctctcaccatcggggaatgccccaaatatgtgaaatcaaacagattagtccttgcaacagggctcagaaatagccctcaaagagagagcagaagaaaaaagaga

>H5N1_A/Indonesia/CDC634T/2006

taccatgcaaacaattcaacagagcaggttgacacaatcatggaaaagaacgttactgttacacatgcccaagacatactggaaaagacacacaacgggaagctctgcgatctagatggagtgaagcctctaattttaagagattgtagtgtagctggatggctcctcgggaacccaatgtgtgacgaattcatcaatgtaccggaatggtcttacatagtggagaaggccaatccaaccaatgacctctgttacccagggagtttcaacgactatgaagaactgaaacacctattgagcagaataaaccattttgagaaaattcaaatcatccccaaaagttcttggtccgatcatgaagcctcatcaggagtgagctcagcatgtccatacctgggaagtccctccttttttagaaatgtggtatggcttatcaaaaagaacagtacatacccaacaataaagaaaagctacaataataccaaccaagaagatcttttggtactgtggggaattcaccatcctaatgatgcggcagagcagacaaggctatatcaaaacccaaccacctatatttccattgggacatcaacactaaaccagagattggtaccaaaaatggctactagatccaaagtaaacgggcaaagtggaaggatggagttcttctggacaattttaaaacctaatgatgcaatcaacttcgagagtaatggaaatttcattgctccagaatatgcatacaaaattgtcaagaaaggggactcagcaattatgaaaagtgaattggaatatggtaactgcaacaccaagtgtcaaactccaatgggggcgataaactctagtatgccattccacaacatacaccctctcaccatcggggaatgccccaaatatgtgaaatcaaacagattagtccttgcaacagggctcagaaatagccctcaaagagagagcagaagaaaaaagaga

>H5N1_A/Indonesia/CDC644/2006

taccatgcaaacaattcaacagagcaggttgacacaatcatggaaaagaacgttactgttacacatgcccaagacatactggaaaagacacacaacgggaagctctgcgatctagatggagtgaagcctctaattttaagagattgtagtgtagctggatggctcctcgggaacccaatgtgtgacgaattcatcaatgtaccggaatggtcttacatagtggagaaggccaatccaaccaatgacctctgttacccagggagtttcaacgactatgaagaactgaaacacctattgagcagaataaaccattttgagaaaattcaaatcatccccaaaagttcttggtccgatcatgaagcctcatcaggagtgagctcagcatgtccatacctgggaagtccctccttttttagaaatgtggtatggcttatcaaaaagaacagtacatacccaacaataaagaaaagctacaataataccaaccaagaagatcttttggtactgtggggaattcaccatcctaatgatgcggcagagcagacaaggctatatcaaaacccaaccacctatatttccattgggacatcaacactaaaccagagattggtaccaaaaatagctactagatccaaagtaaacgggcaaagtggaaggatggagttcttctggacaattttaaaacctaatgatgcaatcaacttcgagagtaatggaaatttcattgctccagaatatgcatacaaagttgtcaagaaaggggactcagcaattatgaaaagtgaattggaatatggtaactgcaacaccaagtgtcaaactccaatgggggcgataaactctagtatgccattccacaacatacaccctctcaccatcggggaatgccccaaatatgtgaaatcaaacagattagtccttgcaacagggctcagaaatagccctcaaagagagagcagaagaaaaaagaga

>H5N1_A/Indonesia/CDC644T/2006

taccatgcaaacaattcaacagagcaggttgacacaatcatggaaaagaacgttactgttacacatgcccaagacatactggaaaagacacacaacgggaagctctgcgatctagatggagtgaagcctctaattttaagagattgtagtgtagctggatggctcctcgggaacccaatgtgtgacgaattcatcaatgtaccggaatggtcttacatagtggagaaggccaatccaaccaatgacctctgttacccagggagtttcaacgactatgaagaactgaaacacctattgagcagaataaaccattttgagaaaattcaaatcatccccaaaagttcttggtccgatcatgaagcctcatcaggagtgagctcagcatgtccatacctgggaagtccctccttttttagaaatgtggtatggcttatcaaaaagaacagtacatacccaacaataaagaaaagctacaataataccaaccaagaagatcttttggtactgtggggaattcaccatcctaatgatgcggcagagcagacaaggctatatcaaaacccaaccacctatatttccattgggacatcaacactaaaccagagattggtaccaaaaatagctactagatccaaagtaaacgggcaaagtggaaggatggagttcttctggacaattttaaaacctaatgatgcaatcaacttcgagagtaatggaaatttcattgctccagaatatgcatacaaagttgtcaagaaaggggactcagcaattatgaaaagtgaattggaatatggtaactgcaacaccaagtgtcaaactccaatgggggcgataaactctagtatgccattccacaacatacaccctctcaccatcggggaatgccccaaatatgtgaaatcaaacagattagtccttgcaacagggctcagaaatagccctcaaagagagagcagaagaaaaaagaga

>H5N1_A/Indonesia/CDC669/2006

taccatgcaaacaattcaacagagcaggttgacacaatcatggaaaagaacgttactgttacacatgcccaagacatactggaaaagacacacaacgggaagctctgcgatctagatggagtgaagcctctaattttaagagattgtagtgtagctggatggctcctcgggaacccaatgtgtgacgaattcatcaatgtaccggaatggtcttacatagtggagaaggccaatccaaccaatgacctctgttacccagggagtttcaacgactatgaagaactgaaacatctattgagcagaataaaccattttgagaaaattcaaatcatccccaaaagttcttggtccgatcatgaagcctcatcaggagtgagctcagcatgtccatacctgggaagtccctccttttttagaaatgtggtatggcttatcaaaaagaacagtacatacccaacaataaagaaaagctacaataataccaaccaagaagatcttttggtactgtggggaattcaccatcctaatgatgcggcagagcagacaaggctatatcaaaacccaaccacctatatttccattgggacatcaacactaaaccagagattggtaccaaaaatagctactagatccaaagtaaacgggcaaagtggaaggatggagttcttctgggcaattttaaaacctaatgatgcaatcaacttcgagagtaatggaaatttcattgctccagaatatgcatacaaaattgtcaagaaaggggactcagcaattatgaaaagtgaattggaatatggtaactgcaacaccaagtgtcaaactccaatgggggcgataaactctagtatgccattccacaacatacaccctctcaccatcggggaatgccccaaatatgtgaaatcaaacagattagtccttgcaacagggctcagaaatagccctcaaagagagagcagaagaaaaaagaga

>H5N1_A/Indonesia/CDC669P/2006

taccatgcaaacaattcaacagagcaggttgacacaatcatggaaaagaacgttactgttacacatgcccaagacatactggaaaagacacacaacgggaagctctgcgatctagatggagtgaagcctctaattttaagagattgtagtgtagctggatggctcctcgggaacccaatgtgtgacgaattcatcaatgtaccggaatggtcttacatagtggagaaggccaatccaaccaatgacctctgttacccagggagtttcaacgactatgaagaactgaaacatctattgagcagaataaaccattttgagaaaattcaaatcatccccaaaagttcttggtccgatcatgaagcctcatcaggagtgagctcagcatgtccatacctgggaagtccctccttttttagaaatgtggtatggcttatcaaaaagaacagtacatacccaacaataaagaaaagctacaataataccaaccaagaagatcttttggtactgtggggaattcaccatcctaatgatgcggcagagcagacaaggctatatcaaaacccaaccacctatatttccattgggacatcaacactaaaccagagattggtaccaaaaatagctactagatccaaagtaaacgggcaaagtggaaggatggagttcttctgggcaattttaaaacctaatgatgcaatcaacttcgagagtaatggaaatttcattgctccagaatatgcatacaaaattgtcaagaaaggggactcagcaattatgaaaagtgaattggaatatggtaactgcaacaccaagtgtcaaactccaatgggggcgataaactctagtatgccattccacaacatacaccctctcaccatcggggaatgccccaaatatgtgaaatcaaacagattagtccttgcaacagggctcagaaatagccctcaaagagagagcagaagaaaaaagaga

>H5N1_A/Indonesia/CDC699/2006

taccatgcaaacaattcaacagagcaggttgacacaatcatggaaaagaacgttactgttacacatgcccaagacatactgggaaagacacacaacgggaagctctgcgatctagatggagtgaagcctctaattttaagagattgtagtgtagctggatggctcctcgggaacccaatgtgtgacgaattcatcaatgtaccggaatggtcttacatagtggagaaggccaatccaaccaatgacctctgttacccagggagtttcaacgactatgaagaactgaaacacctattgagcagaataaaccattttgagaaaattcaaatcatccccaaaagttcttggtccgatcatgaagcctcatcaggagtgagctcagcatgtccatacctgggaagtccttccttttttagaaatgtggtatggcttatcaaaaagaacagtacatacccaacaataaagacaagctacaataataccaaccaagaagatcttttggtactgtggggaattcaccattctaatgatgcggcagagcagacaaggctatatcaaaacccaaccacctatatttccattgggacatcaacactaaaccagagattggtaccaaaaatagctactagatccaaagtaaacgggcaaagtggaaggatggagttcttctggacaattttaaaacctaatgatgcaatcaacttcgagagtaatggaaatttcattgctccagaatatgcatacaaaattgtcaagaaaggggactcagcaattatgaaaagtgaattggaatatggtaactgcaacaccaagtgtcaaactccaatgggggcaataaactctagtatgccattccacaacatacaccctctcaccatcggggaatgccccaaatatgtgaaatcaaacagattagtccttgcaacagggctcagaaatagccctcaaagagagagcagaagaaaaaagaga

>H5N1_A/Indonesia/CDC7/2005

taccatgcaaacaattcaacagagcaggttgacacaatcatggaaaagaacgttactgttacacatgcccaagacatattggaaaagacacacaacgggaagctctgcgatctagatggagtgaagcctctaattttaagagattgtagtgtagctggatggctcctcgggaacccaatgtgtgacgaattcatcaatgtaccggaatggtcttacatagtggagaaggccaatccaaccaatgacctctgttacccagggagtttcaacgactatgaagaactgaaacacctattgagcagaataaaccattttgagaaaattcagatcatccccaaaagttcttggtccgatcatgaagcctcatcaggagtgagttcagcatgtccatacctgggtagttcctccttttttagaaatgtggtatggcttatcaaaaagaacagtacatacccaacaataaagaaaagctacaataataccaaccaagaagatcttttggtactgtgggggattcaccatcctaatgatgcggcagagcagacaaggctatatcaaaacccaaccacctatatttccattgggacatcaacactaaaccagagattggtaccaaaaatagctactagatccaaagtaaacgggcaaagtggaaggatggagttcttctggacaattttaaaacctaatgatgcaatcaacttcgagagtaatggaaatttcattgctccagaatatgcatacaaaattgtcaagaaaggggactcagcaattatgaagagtgaattggaatatggtaactgcaacaccaagtgtcaaactccaatgggggcgataaactctagtatgccattccacaacatacaccctctcaccatcggggaatgccccaaatatgtgaaatcaaacagattagtccttgcaacagggctcagaaatagccctcaaagagaaagcagaagaaaaaagaga

>H5N1_A/Indonesia/CDC739/2006

taccatgcaaacaattcaacagagcaggttgacacaatcatggaaaagaacgttactgttacacatgcccaagacatactgggaaaaacacacaacgggaagctctgcgatctagatggagtgaagcctctaattttaagagattgtagtgtagctggatggctcctcgggaacccaatgtgtgacgaattcatcaatgtaccggaatggtcttacatagtggagaaggccaatccaaccaatgacctctgttacccagggagtttcaacgactatgaagaactgaaacatctattgagcagaataaaccattttgagaaaattcaaatcatccccaaaagttcttggtccgatcatgaagcctcatcaggagtgagctcagcatgtccatacctgggaagtccctccttttttagaaatgtggtatggcttatcaaaaagaacagtacatacccaacaataaagaaaagctacaataataccaaccaagaagatcttttggtactgtggggaattcaccatcctaatgatgcggcagagcagacaaggctatatcaaaacccaaccacctatatttccattgggacatcaacactaaaccagagattggtaccaaaaatagctactagatccaaagtaaacgggcaaagtggaaggatggagttcttctgggcaattttaaaacctaatgatgcaatcaacttcgagagtaatggaaatttcattgctccagaatatgcatacaaaattgtcaagaaaggggactcagcaattatgaaaagtgaattggaatatggtaactgcaacaccaagtgtcaaactccaatgggggcgataaactctagtatgccattccacaacatacaccctctcaccatcggggaatgccccaaatatgtgaaatcaaacagattagtccttgcaacagggctcagaaatagcccacaaagagagagcagaagaaaaaagaga

>H5N1_A/Indonesia/CDC742/2006

taccatgcaaacaattcaacagagcaggttgacacaatcatggaaaagaacgttactgttacacatgcccaagacatactggaaaagacacacaacgggaagctctgcgatctagatggagtgaagcctctaattttaagagattgtagtgtagctggatggctcctcgggaacccaatgtgtgacgaattcatcaatgtaccggaatggtcttacatagtggagaaggccaatccaaccaatgacctctgttacccaggaagtttcaacgactatgaagaactgaaacacctattgagcagaataaaccattttgagaaaattcaaatcatccccaaaagttcttggtccgatcatgaagcctcatcaggagtgagctcagcatgtccatacctgggaagtccctccttttttagaaatgtggtatggcttatcaaaaagaacagtacatacccaacaataaagaaaagctacaataataccaaccgagaagatcttttggtactgtggggaattcaccatcctaatgatgcggcagagcagacaaggctatatcaaaacccaaccacctatatttccattgggacatcaacactaaaccagagattggtgccaaaaatagctactagatccaaagtaaatgggcaaagtggaaggatggagttcttctggacaattttaaaccctaatgatgcaatcaacttcgagagtaatggaaatttcattgctccagaatatgcatacaaaattgtcaagaaaggggactcagcaattatgaaaagtgaattggaatatggtaactgcaacaccaagtgtcaaactccaatgggggcgataaactctagtatgccattccacaacatacaccctctcaccatcggggaatgccccaaatatgtgaaatcaaacagattagtccttgcaacagggctcagaaatagccctcaaagagagagcagaagaaaaaagaga

>H5N1_A/Indonesia/CDC759/2006

taccatgcaaacaattcaacagagcaggttgacacaatcatggaaaagaacgttactgttacacatgcccaagacatactggaaaagacacacaacgggaagctctgcgatctagatggagtgaagcctctaattttaagagattgtagtgtagctggatggctcctcgggaacccaatgtgtgacgaattcatcaatgtaccggaatggtcttacatagtggagaaggccaatccaaccaatgacctctgttacccagggagtttcaacgactatgaagaactgaaacatctattgagcagaataaaccattttgagaaaattcaaatcatccccaaaagttcttggtccgatcatgaagcctcatcaggagtgagctcagcatgtccatacctgggaagtccctccttttttagaaatgtggtatggcttatcaaaaagaacagtacatacccaacaataaagaaaagctacaataataccaaccaagaagatcttttggtactgtggggaattcaccatcctaatgatgcggcagagcagacaaagctatatcaaaacccaaccacctatatttccattgggacatcaacactaaaccagagattggtaccaaaaatagctactagatccaaagtaaacgggcaaagtggaaggatggagttcttctgggcaattttaaaacctaatgatgcaatcaacttcgagagtaatggaaatttcattgctccagaatatgcatacaaaattgtcaagaaaggggactcagcaattatgaaaagtgaattggaatatggtaactgcaacaccaagtgtcaaactccaatgggggcgataaactctagtatgccattccacaacatacaccctctcaccatcggggaatgccccaaatatgtgaaatcaaacagattagtccttgcaacagggctcagaaatagccctcaaagagagagcagaagaaaaaagaga

>H5N1_A/Indonesia/CDC835/2006

taccatgcaaacaattcaacagagcaggttgacacaatcatggaaaagaacgttactgttacacatgcccaagacatactggaaaagacacacaacgggaagctctgcgatctagatggagtgaagcctctaattttaagagattgtagtgtagctggatggctcctcgggaacccaatgtgtgacgaattcatcaatgtaccggaatggtcttacatagtggagaaggccaatccaaccaatgacctctgttacccagggagtttcaacgactatgaagaactgaaacatctattgagcagaataaaccattttgagaaaattcaaatcatccccaaaagttcttggtccgatcatgaagcctcatcaggagtgagctcagcatgtccatacctgggaagtccctccttttttagaaatgtggtatggcttatcaaaaagaacagtacatacccaacaataaagaaaagctacaataataccaaccaagaggatcttttggtactgtggggaattcaccatcctaatgatgcggcagagcagacaaggctatatcaaaacccaaccacctatatttccattgggacatcaacactaaaccagagattggtaccaaaaatagctactagatccaaagtaaacgggcaaagtggaaggatggagttcttctggacaattttaaaacctaatgatgcaatcaacttcgagagtaatggaaatttcattgctccagaatatgcatacaaaattgtcaagaaaggggactcagcaattatgaaaagtgaattggaatatggtaactgcaacaccaagtgtcaaactccaatgggggcgataaactctagtatgccattccacaacatccaccctctcaccatcggggaatgccccaaatatgtgaaatcaaacagattagtccttgcaacagggctcagaaatagccctcaaagagagagcagaagaaaaaagaga

>H5N1_A/Indonesia/CDC836/2006

taccatgcaaacaattcaacagagcaggttgacacaatcatggaaaagaacgttactgttacacatgcccaagacatactggaaaagacacacaacgggaagctctgcgatctagatggagtgaagcctctaattttaagagattgtagtgtagctggatggctcctcgggaacccaatgtgtgacgaattcatcaatgtaccggaatggtcttacatagtggagaaggccaatccaaccaatgacctctgttacccagggagtttcaacgactatgaagaactgaaacatctattgagcagaataaaccattttgagaaaattcaaatcatccccaaaagttcctggtccgatcatgaagcctcatcaggagtgagctcagcatgtccatacctgggaagtccctccttttttagaaatgtggtatggcttatcaaaaagaacagtacatacccaacaataaagaaaagctacaataataccaaccaagaagatcttttggtactgtggggaattcaccatcctaatgatgcggcagagcagacaaggctatatcaaaacccaaccacctatatttccattgggacatcaacactaaaccagagattggtaccaaaaatagctactagatccaaagtaaacgggcaaagtggaaggatggagttcttctgggcaattttaaaacctaatgatgcaatcaacttcgagagtaatggaaatttcattgctccagaatatgcatacaaaattgtcaagaaaggggactcagcaattatgaaaagtgaattggaatatggtaactgcaacaccaagtgtcaaactccaatgggggcgataaactctagtatgccattccacaacatacaccctctcaccatcggggaatgccccaaatatgtgaaatcaaacagattagtccttgcaacagggctcagaaatagccctcaaagagagagcagaagaaaaaagaga

>H5N1_A/Indonesia/CDC836T/2006

taccatgcaaacaattcaacagagcaggttgacacaatcatggaaaagaacgttactgttacacatgcccaagacatactggaaaagacacacaacgggaagctctgcgatctagatggagtgaagcctctaattttaagagattgtagtgtagctggatggctcctcgggaacccaatgtgtgacgaattcatcaatgtaccggaatggtcttacatagtggagaaggccaatccaaccaatgacctctgttacccagggagtttcaacgactatgaagaactgaaacatctattgagcagaataaaccattttgagaaaattcaaatcatccccaaaagttcctggtccgatcatgaagcctcatcaggagtgagctcagcatgtccatacctgggaagtccctccttttttagaaatgtggtatggcttatcaaaaagaacagtacatacccaacaataaagaaaagctacaataataccaaccaagaagatcttttggtactgtggggaattcaccatcctaatgatgcggcagagcagacaaggctatatcaaaacccaaccacctatatttccattgggacatcaacactaaaccagagattggtaccaaaaatagctactagatccaaagtaaacgggcaaagtggaaggatggagttcttctgggcaattttaaaacctaatgatgcaatcaacttcgagagtaatggaaatttcattgctccagaatatgcatacaaaattgtcaagaaaggggactcagcaattatgaaaagtgaattggaatatggtaactgcaacaccaagtgtcaaactccaatgggggcgataaactctagtatgccattccacaacatacaccctctcaccatcggggaatgccccaaatatgtgaaatcaaacagattagtccttgcaacagggctcagaaatagccctcaaagagagagcagaagaaaaaagaga

>H5N1_A/Indonesia/CDC887/2006

taccatgcaaacaattcaacagagcaggttgacacaatcatggaaaagaacgttactgttacacatgcccaagacatactggaaaagacacacaacgggaagctctgcgatctagatggagtgaagcctctaattttaagagattgtagtgtagctggatggctcctcgggaacccaatgtgtgacgaattcatcaacgtaccggaatggtcttacatagtggagaaggccaatccaaccaatgacctctgttacccagggagtttcaacgactatgaagaactgaaacacctattgagcagaataaaccattttgagaaaattcagatcatccccaaaggttcttggtccgatcatgaagcctcatcaggagtgagctcagcatgtccatacctgagaagtccctccttttttagaaatgtggtatggcttatcaaaaagaacagtacatacccaacaataaagaaaagctacaataataccaaccaagaagatcttttggtactgtggggaattcaccatcctaataatgaggcagagcagacaaggctatatcaaaacccaaccacctatatttccattgggacatcaacactaaaccagagattggtaccaaaaatagctactagatccaaagtaaacgggcaaagtggaaggatggagttcttctggacaattttaaaaccaaatgatgcaatcaacttcgagagtaatggaaatttcattgctccagaatatgcatacaaaattgtcaagaaaggggactcagcaattatgaaaagtgaattggaatatggtaactgcaacaccaagtgtcaaactccaatgggggcgataaactctagtatgccattccacaacatacaccctctcaccatcggggaatgccccaaatatgtgaaatcaaacagattagtccttgcaacagggctcagaaatagccctcaaagagagagcagaagaaaaaagaga

>H5N1_A/Indonesia/CDC938/2006

taccatgcaaacaattcaacagagcaggttgacacaatcatggaaaagaacgttactgttacacatgcccaagacatactggaaaagacacacaacgggaagctctgcgatctagatggagtgaagcctctaattttaagagattgtagtgtagctggatggctcctcgggaacccaatgtgtgacgaattcatcaacgtaccggaatggtcttacatagtggaaaaggccaatccaaccaatgacctctgttacccagggagtttcaacgactatgaagaactgaaacacctattgagcagaataaaccattttgagaaaattcagatcatccccaaaagttcttggtccgatcatgaagcctcatcaggagtgagctcagcatgtccatacctgggaagtccctccttttttagaaatgtggtatggcttaccaaaaagaacagtacatacccaacaataaagaaaagctacaataataccaaccaagaagatcttttggtactgtggggaattcaccatcctaataatgaggcagagcagacaaggctatatcaaaacccaaccacctatatttccattgggacatcaacactaaaccagagattggtaccaaaaatagctactagatccaaagtaaacgggcaaagtggaaggatggagttcttctggacaattttaaaaccaaatgatgcaatcaacttcgagagtaatggaaatttcattgctccagaatatgcatacaaaattgtcaagaaaggggactcagcaattatgaaaagtgaattggaatatagtaactgcaacaccaagtgtcaaactccaatgggggcgataaactctagtatgccattccacaacatacaccctctcaccatcggggaatgccccaaatatgtgaaatcaaacagattagtccttgcaacagggctcagaaatagccctcaaagagagagcagaagaaaaaagaga

>H5N1_A/Indonesia/CDC938E/2006

taccatgcaaacaattcaacagagcaggttgacacaatcatggaaaagaacgttactgttacacatgcccaagacatactggaaaagacacacaacgggaagctctgcgatctagatggagtgaagcctctaattttaagagattgtagtgtagctggatggctcctcgggaacccaatgtgtgacgaattcatcaacgtaccggaatggtcttacatagtggaaaaggccaatccaaccaatgacctctgttacccagggagtttcaacgactatgaagaactgaaacacctattgagcagaataaaccattttgagaaaattcagatcatccccaaaagttcttggtccgatcatgaagcctcatcaggagtgagctcagcatgtccatacctgggaagtccctccttttttagaaatgtggtatggcttaccaaaaagaacagtacatacccaacaataaagaaaagctacaataataccaaccaagaagatcttttggtactgtggggaattcaccatcctaataatgaggcagagcagacaaggctatatcaaaacccaaccacctatatttccattgggacatcaacactaaaccagagattggtaccaaaaatagctactagatccaaagtaaacgggcaaagtggaaggatggagttcttctggacaattttaaaaccaaatgatgcaatcaacttcgagagtaatggaaatttcattgctccagaatatgcatacaaaattgtcaagaaaggggactcagcaattatgaaaagtgaattggaatatagtaactgcaacaccaagtgtcaaactccaatgggggcgataaactctagtatgccattccacaacatacaccctctcaccatcggggaatgccccaaatatgtgaaatcaaacagattagtccttgcaacagggctcagaaatagccctcaaagagagagcagaagaaaaaagaga

>H5N1_A/Indonesia/CDC940/2006

taccatgcaaacaattcaacagagcaggttgacacaatcatggaaaagaacgttactgttacacatgcccaagacatactggaaaagacacacaacgggaagctctgcgatctagatggagtgaagcctctaattttaagagattgtagtgtagctggatggctcctcgggaacccaatgtgtgacgaattcatcaatgtaccggaatggtcttacatagtggagaaggccaatccaaccaatgacctctgttacccagggagtttcaacgactatgaagaactgaaacatctattgagccgaataaaccattttgagaaaattcaaatcatccccaaaagttcttggtccgatcatgaagcctcatcaggagtgagctcagcatgtccatacctgggaagtccctccttttttagaaatgtggtatggcttatcaaaaagaacagtacatacccaacaataaagaaaagctacaataataccaaccaagaagatcttttggtactgtggggaattcaccatcccaatgatgcagcagagcagacaaggctatatcaaaacccaaccacctatatttccattgggacatcaacactaaaccagagattggtaccaaaaatagctactagatccaaagtaaacgggcaaagtggaaggatggagttcttctgggcaattttaaaacctaatgatgcaatcaacttcgagagtaatggaaatttcattgctccagaatatgcatacaaaattgtcaagaaaggggactcagcaattatgaaaagtgaattggaatatggtaactgcaacaccaagtgtcaaactccaatgggggcgataaactctagtatgccattccacaacatacaccctctcaccatcggggaatgccccaaatatgtgaaatcaaacagattagtccttgcaacagggctcagaaatagccctcaaagagagagcagaagaaaaaagaga

>H5N1_A/Indonesia/NIHRD/15023/2015

taccatgcaaacaattcaacagaacaggttgacacaatcatggaaaagaatgttacagttacacatgcccaagacatactggaaaggacacacaacgggaagctctgcgatctagatggagtgaagcctctaattttaaaagattgcagtgtagctggatggctcctcgggaacccaatgtgtgacgaatttatcaatgtcccagaatggtcttacatagtggagaaggccaatccgactaacgatctctgttacccagggagtttcaacgattatgaagaactgaaacacctattaagcagaataaaccatttcgagaaaattcagatcatccctaaaaattcttggtccgatcatgaagcatcatcaggagtgagctcagcatgcccatatctgggaagtccctccttttttagaaatgtggtatggcttatcaaaaagaacaatacgtacccaacaataaagaaaacctacaataataccaaccaagaagatcttttgatactgtggggaatccaccatcctaataatgaaatagagcagacaatgttatatcaaaacccaaccacctatatttccattgggacatcaacactaaaccagagactggtaccaaaaatagctactagatccaaagtgaacgggcaaagtggaaggatggagttcttctggacaattttaaaaccaaatgatgcaattaacttcgagagtaatggaaatttcattgccccagaatatgcatacaaaattgtcaagaaaggggattcggcaattatgaaaagtgaattggaatatggtaactgtaacaccaagtgtcaaactccaatgggggcgataaactctagtatgccattccacaacatacaccctctcaccatcggggaatgccccaaatatgtgaaatcaaacagattagtcctagcaacagggctcagaaatagccctcaaagggaaagcagaaggaaaaagaga

>H5N1_A/Indonesia/NIHRD10364/2010

taccatgcaaacaattcaacggagcaggttgacacaatcatggaaaagaacgttactgttacacatgcccaagacatactggaaaggacacacaacgggaagctctgcgatctagatggagtgaagcctctaattttaaaagattgcagtgtagctggatggctcctcgggaacccaatgtgtgacgaattcatcaacgtaccagaatggtcttacatagtggagaaggccaatccgaccaacgacctctgttacccaggaagtttcaacgactatgaagaactgaaacacctattaagcagaataaaccattttgagaaaattcagatcatccctaaaagttcttggtccgatcatgaagcatcatcaggagtgagctcagcatgcccatacctgggaagtccctccttttttagaaatgtggtatggcttatcaaaaagaacagtacgtacccaacaataaagaaaacctacaataataccaaccaagaagatcttttgatactgtggggaattcatcatcctaataatgaggcagagcagacaatgctatatcaaaacccaaccacctatatttccattgggacatcaacactaaaccagagattggtaccaaaaatagctactagatccaaagtaaacgggcaaagtggaaggatggagtttttctggacaattttaaaaccaaatgatgcaatcaacttcgagagtaatggaaatttcattgctccagaatatgcatacaaaattgtcaagaaaggggactcagcaattatgaaaagtgaattggaatatggtaactgcaacaccaagtgtcaaactccaatgggggcgataaactctagtatgccattccacaacatacaccctctcaccatcggggaatgccccaaatatgtgaaaacaaacagattagtccttgcaacagggctcagaaatagccctcaaagagagagcagaaggaaaaagaga

>H5N1_A/Indonesia/NIHRD10459/2010

taccatgcaaacaattcaacggaacaggttgacacaatcatggaaaagaacgttactgttacacatgcccaagacatactggaaaggacacacaacgggaagctctgcgatctagatggagtgaagcctctaattttaaaagattgcagtgtagctggatggctcctcgggaacccaatgtgtgacgaattcatcaacgtaccggaatggtcttacatagtggagaaggccaatccaacaaatgacctctgttacccagggagtttcaacgattatgaagaactgaaacacctattgagcagaataaaccatttcgaaaaaattcagatcatccctaaaagttcttggtccgatcatgaagcatcatcaggagtgagctcagcatgcccatacctgggaagtccctccttttttagaaatgtggtatggcttatcaaaaagaacagtacgtacccaacaataaagaaaacctacaataataccaaccaagaagatcttttgatactgtggggaattcatcatcctaataatgaggctgagcagacaatgctatatcaaaacccaaccacctatatttccattgggacatcaacactaaaccagagattggtaccaaaaatagctactagatccaaagtaaacgggcaaagtggaaggatggagtttttctggacaattttaaaaccaaatgatgcaatcaacttcgagagtaatggaaatttcattgctccagaatatgcatacaaaattgtcaagaaaggggactcagcaattatgaaaagtgaattggaatatggtaactgcaacaccaagtgtcaaactccaatgggggcgataaactctagtatgccattccacaacatacaccctctcaccatcggggaatgccccaaatatgtgaaatcaaacagattagtcctagcaacagggctcagaaatagccctcaaagagaaagcagaaagaaaaagaga

>H5N1_A/Indonesia/NIHRD10529/2010

taccatgcaaacaattcaacggagcaggttgacacaatcatggaaaagaacgttactgttacacatgcccaagacatactggagaagacacacaacgggaagctctgcgatctagatggagtgaagcctctaattttaaaagattgcagtgtagctggatggctcctcgggaacccaatgtgtgacgaattcatcaacgtaccagaatggtcttacatagtggagaaggccaatccaaccaatgacctctgttacccaggaagtttcaacgactatgaagaactgaaacacctattgagcagaataaaccattttgagaaaattcagatcatccccaaaagttcttggtccgatcatgaagcatcatcaggagtgagctcagcatgtccatacctgggaagtccctccttttttagaaatgtggtatggcttatcaaaaagaacagtacgtacccaacaataaagaaaacctacaataataccaaccaagaagatcttttgatactgtggggaattcaccatcctaataatgaggcagagcagacaatgctatatcaaaacccaaccacctatatttccattgggacatcaacactaaaccagagattggtaccaaaaatagctactagatccaaagtaaacgggcaaagtggaagggtggagtttttctggacaattttaaaaccaaatgatgcaatcaacttcgagagtaatggaaatttcattgctccagaatatgcatacaaaattgtcaagaaaggggactcagcaattatgagaagtgaattggaatatggtaactgcaacaccaagtgtcaaactccaatgggggcgataaactctagtatgccattccacaacatacaccctctcaccatcggggaatgccccaaatatgtgaaatcaaacagattagtcctagcgacagggctcagaaatagccctcaaagagaaggcagaaggaaaaagaga

>H5N1_A/Indonesia/NIHRD10612/2010

taccatgcaaacaattcaacggagcaggttgacacaatcatggaaaagaacgttactgttacacatgcccaagacatactggagaagacacacaacgggaagctctgcgatctagatggagtgaagcctctaattttaaaagattgcagtgtagctggatggctcctcgggaacccaatgtgtgacgaattcatcaacgtaccagaatggtcttacatagtggagaaggccaatccaaccaatgacctctgttacccaggaagtttcaacgactatgaagaactgaaacacctattgagcagaataaaccattttgagaaaattcagatcatccccaaaagttcttggtccgatcatgaagcatcatcaggagtgagctcagcatgtccatacctgggaagtccctccttttttagaaacgtggtatggcttatcaaaaagaacagtacgtacccaacaataaagaaaatctacaataataccaaccaagaagatcttttgatactgtggggaattcatcatcctaataatgaggctgagcagacaatgctatatcaaaacccaaccacctatatttccattgggacatcaacactaaaccagagattggtaccaaaaatagctactagatccaaagtaaacgggcaaagtggaaggatggagtttttctggacaattttaaaaccaaatgatgcaatcaacttcgagagtaatggaaatttcattgctccagaatatgcatacaaaattgtcaagaaaggggactcagcaattatgaaaagtgaattggaatatggtaactgcaacaccaagtgtcaaactccaatgggggcgataaactctagtatgccattccacaacatacaccctctcaccatcggggaatgccccaaatatgtgaaatcaaacagattagtcctagcaacagggctcagaaatagccctcaaagagaaagcagaaggaaaaagaga

>H5N1_A/Indonesia/NIHRD10623/2010

taccatgcaaacaattcaacggagcaggttgacacaatcatggaaaagaacgttactgttacacatgcccaagacatactggaaaggacacacaacgggaagctctgcgatctagatggagtgaagcctctaattttaaaagattgcagtgtagctggatggctcctcgggaacccaatgtgtgacgaattcatcaacgtaccagaatggtcttacatagtggagaaggccaatccgaccaatgacctctgttacccagggagtttcaacgactatgaagaactgaaacacctattaagcagaataaaccattttgagaaaattcagatcatccccaaaagttcttggtccgatcatgaagcatcatcaggagtgagctcagcatgcccatacctgggaagtccctccttttttagaaatgtggtatggcttatcaaaaagaacagtacgtacccaacaataaagaaaacctacaataataccaaccaagaagatcttttgatactgtggggaattcatcatcctaataatgaggcagagcagacaatgctatatcaaaacccaaccacctatatttccattgggacatcaacactaaaccagagattggtaccaaaaatagctactagatccaaagtaaacgggcaaagtggaaggatggagtttttctggacaattttaaaaccaaatgatgcaatcaacttcgagagtaatggaaatttcattgctccagaatatgcatacaaaattgtcaagaaaggggactcagcaattatgaaaagtgaattggaatatggtaactgcaacaccaagtgtcaaactccaatgggggcgataaactctagtatgccattccacaacatacaccctctcaccatcggggaatgccccaaatatgtgaaatcaaacagattagtcctagcaacagggctcagaaatagccctcaaagagaaagcagaaggaaaaagaga

>H5N1_A/Indonesia/NIHRD10728/2010

taccatgcaaacaattcaacggaacaggttgacacaatcatggaaaagaacgttactgttacacatgcccaagacatactggaaaggacacacaacgggaagctctgcgatctagatggagtgaagcctctaattttaaaagattgcagtgtagctggatggctcctcggaaacccaatgtgtgacgaattcatcaacgtaccagaatggtcttacatagtggagaaggccaatccgaccaatgacctctgttacccaggaggtttcaacgactatgaagaactgaaacacctattaagcagaataaaccatttcgagaaaattcagatcatccctaaaagttcttggtccgatcatgaagcatcatcaggagtgagctcagcatgcccatacctgggaagtccctccttttttagaaatgtggtatggcttatcaaaaagaacagtacgtacccaacaataaagaaaacctacaataataccaaccaagaagatcttttggtactgtggggaattcaccatccgaataatgaggcagagcagacaatgctatatcaaaacccaaccacctatatttccattgggacatcaacactaaaccagagattggtaccaaaaatagctactagatccaaagtaaacgggcaaagtggaaggatggagtttttctggacaattttaaaaccaaatgatgcaatcaacttcgagagtaaaggaaatttcattgctccagaatatgcatacaaaattgtcaagaaaggggactcagcaattatgaaaagtgaattggaatatggtaactgcaacaccaagtgtcaaactccaatgggggcgataaactctagtatgccattccacaacatacaccctctcaccatcggggaatgccccaaatatgtgaaatcaaacagattagtcctagcaacagggctcagaaatagccctcaaagagaaagcagaaggaaaaagaga

>H5N1_A/Indonesia/NIHRD11046/2011

taccatgcaaacaattcaacagaacaggttgacacaatcatggaaaagaacgttactgttacacatgcccaagacatactggaaaggacacacaacgggaagctctgcgatctagatggagtgaagcctctaattttaaaagattgcagtgtagctggatggctcctcgggaacccaatgtgtgacgaattcatcaacgtaccagaatggtcttacatagtggagaaggccaatccgaccaatgacctctgttacccaggaagtttcaacgactatgaagaactgaaacacctattaagcagaataaaccatttcgagaaaattcagatcatccctaaaagttcttggtccgatcatgaagcatcatcaggagtgagctcagcatgcccatacctgggaagtccctccttttttagaaatgtggtatggcttatcaaaaagaacagtacgtacccaacaataaagaaaacctacaataataccaaccaagaagatcttttggtactgtggggaattcaccatcctaatactgaggcagagcagacaatgctatatcaaaacccaaccacctatatttccattgggacatcaacactaaaccagagattggtaccaaaaatagctactagatccaaagtaaacgggcaaagtggaaggatggagtttttctggacaattttaaaaccaaatgatgcaatcaacttcgagagtaatggaaatttcattgctccagaatatgcatacaaaattgtcaagaaaggggactcagcaattatgaaaagtgaattggaatatggtaactgcaacaccaagtgtcaaactccaatgggggcgataaactctagtatgccattccacaacatacaccctctcaccatcggggaatgccccaaatatgtgaaatcaaacagattagtcctagcaacagggctcagaaatagccctcaaagagagagcagaaggaaaaagaga

>H5N1_A/Indonesia/NIHRD11073/2011

taccatgcaaacaattcaacggagcaggttgacacaatcatggaaaagaacgttactgttacacatgcccaagacatactggaaaagacacacaacgggaagctctgcgatctagatggagtgaagcctctaattttaaaagattgcagcgtagctggatggctcctcgggaacccaatgtgtgacgaattcatcaacgtaccagaatggtcttacatagtggagaaggccaatccgaccaatgacctctgttacccagggagtttcaacgactatgaagaactgaaacacctattgagcagaataaaccattttgagaaaattcagatcatccccaaaagttcttggtccgatcatgaagcatcatcaggagtgagctcagcatgcccatacctgggaagtccctccttttttagaaatgtggtatggcttatcaaaaagaacagtacgtacccaacaataaagaaaacctacaataataccaaccaagaagatcttttgatactgtggggaattcaccatcctaataatgaggcagagcagacaatgctatatcaaaacccaaccacctatatttccattgggacatcaacactaaaccagagattggtaccaaaaatagctactagatccaaagtaaacgggcaaagtggaaggatggagtttttctggacaattttaaaaccaaatgatgcaatcaacttcgagagtaatggaaatttcattgctccagaatatgcatacaaaattgtcaagaaaggggactcagcaattatgaaaagtgaattggaatatggtaactgcaacaccaagtgtcaaactccaatgggggcgataaactctagtatgccattccacaacatacaccctctcaccatcggggaatgccccaaatatgtgaaatcaaacagattagtcctagcaacagggctcagaaatagccctcaaagagaaagcagaaggaaaaagaga

>H5N1_A/Indonesia/NIHRD11073/2011(H5N1)

taccatgcaaacaattcaacggagcaggttgacacaatcatggaaaagaacgttactgttacacatgcccaagacatactggagaagacacacaacgggaagctctgcgatctagatggagtgaagcctctaattttaaaagattgcagcgtagctggatggctcctcgggaacccaatgtgtgacgaattcatcaacgtaccagaatggtcttacatagtggagaaggccaatccgaccaatgacctctgttacccagggagtttcaacgactatgaagaactgaaacacctattgagcagaataaaccattttgagaaaattcagatcatccccaaaagttcttggtccgatcatgaagcatcatcaggagtgagctcagcatgcccatacctgggaagtccctccttttttagaaacgtggtatggcttatcaaaaagaacagtacgtacccaacaataaagaaaacctacaataataccaaccaagaagatcttttgatactgtggggaattcaccatcctaataatgaggcagagcagacaatgctatatcaaaacccaaccacctatatttccattgggacatcaacactaaaccagagattggtaccaaaaatagctactagatccaaagtaaacgggaaaagtggaaggatggagtttttctggacaattttaaaaccaaatgatgcaatcaacttcgagagtaatggaaatttcattgctccagaatatgcatacaaaattgtcaagaaaggggactcagcaattatgaaaagtgaattggaatatggtaactgcaacaccaagtgtcaaactccaatgggggcgataaactctagtatgccattccacaacatacaccctctcaccatcggggaatgccccaaatatgtgaaatcaaacagattagtcctagcgacagggctcagaaatagccctcaaagagaaagcagaaggaaaaagaga

>H5N1_A/Indonesia/NIHRD11198/2011

taccatgcaaacaattcaacagaacaggttgacacaatcatggaaaagaacgttactgttacacatgcccaagacatactggaaaggacacacaacgggaagctctgcaatctagatggagtgaagcctctaattttaaaagattgcagtgtagctggatggctcctcgggaacccaatgtgtgacgaattcatcaacgtaccagaatggtcttacatagtggagaaggccaatccgaccaacgacctctgttacccagggagtttcaacgactatgaagaactgaaacacctattaagcagaataaaccatttcgagaaaattcagatcatccctaaaagttcttggtccgatcatgaagcatcatcaggagtgagctcagcatgcccatacctgggaagtccctccttttttagaaatgtggtatggcttatcaaaaagaacagtacgtacccaacaataaagaaaacctacaataataccaaccaagaagatcttttggtactgtggggaattcaccatcctaataatgaggcagagcagacaatgctatatcaaaacccaaccacctatatttccattgggacatcaacactaaaccagagattggtaccaaaaatagctactagatctaaagtaaacgggcaaagtggaaggatggagtttttctggacaattttaaaaccaaatgatgcaatcaacttcgagagtaatggaaatttcattgctccagaatatgcatacaaaattgtcaagaaaggggactcagcaattatgaaaagtgaattggaatatggtaactgcaacaccaagtgtcaaactccaatgggggcgataaactctagtatgccattccacaacatacaccctctcaccatcggggaatgccccaaatatgtgaaatcaaacagattagtcctagcgacagggctcagaaatagccctcaaagagaaagcagaaggaaaaagaga

>H5N1_A/Indonesia/NIHRD11454/2011

taccatgcaaacaattcaacagagcaggttgacacaatcatggaaaagaacgttactgttacacatgcccaagacatactggaaaagacacacaacgggaagctctgcgatctagatggagtgaagcctctaattttaagagattgtagtgtagctggatggctcctcgggaacccaatgtgtgacgaattcatcaatgtaccggaatggtcttacatagtggagaaggccaatccaaccaatgacctctgttacccaggaggtttcaacgactatgaagaactgaaacacctattgagcagaataaaccattttgagaaaattcaaatcatccccaaaagttcttggtccgatcatgaagcctcatcaggagtgagctcagcatgtccatatctgggaagtccctccttttttagaaatgtggtatggcttatcaaaaagaacagtacatacccaacaataaagaaaagctacaataacaccaaccaagaagatcttttggtactgtggggaattcaccatcctaatgatgcggcagagcagacaaggctatatcaaaacccaaccacctatatttccattgggacatcaacactaaaccagagattggtaccaaaaatagctactagatccaaagtaaacgggcaaagtggaaggatggagtttttctggacaattttaaaaccaaatgatgcaatcaacttcgagagtaatggaaatttcattgctccagaatatgcatacaaaattgtcaagaaaggggactcagcaattatgaaaagtgaattggaatatggtaactgcaacaccaagtgtcaaactccaatgggggcgataaactctagtatgccattccacaacatacaccctctcaccatcggggaatgccccaaatatgtgaaatcaaacagattagtcctagcaacagggctcagaaatagccctcaaagagagagcagaaggaaaaagaga

>H5N1_A/Indonesia/NIHRD11767/2011(H5N1)

taccatgcaaacaattcaacggagcaggttgacacaatcatggaaaagaacgttactgttacacatgcccaagacatactggagaagacacacaacgggaagctctgcgatctagatggagtgaagcctctaattttaaaagattgcagtgtagctggatggctcctcgggaatccaatgtgtgacgaattcatcaatgtaccagagtggtcttacatagtggagaaggccaatccaaccaatgacctctgttacccaggaagtttcaacgactatgaagaactgaaacatctattgagcagaataaaccattttgagaaaattcagatcatccccaaaagttcttggtccgatcatgaagcatcatcaggagtgagctcagcatgtccatacctgggaagtccctcctttttcagaaacgtggtatggcttatcaaaaagaacagtacatacccaacaataaagaaaacctacaacaataccaaccaagaagatcttttgatactgtggggaattcaccatcctaataatggggcagagcagacaatgctatatcaaaacccaaccacctatatttccattgggacatcaacactaaaccagagattggtaccaaaaatagctactagatccaaagtaaacgggcaaagtggaagaatggagtttttttggacaattttaaaaccaaatgatgcaatcaacttcgagagtaatggaaacttcattgctccagaatatgcatacaaaattgtcaagaaaggggactcagcaattatgaaaagtgaattggaatatggtaactgcaacaccaagtgtcaaaccccaatgggagcgataaactctagtatgccattccacaacatacaccctatcaccattggggaatgccccaaatatgtgaaatcaaacagactagtcttagcaacagggctcagaaacagccctcaaagagagagcagaaggaaaaagaga

>H5N1_A/Indonesia/NIHRD11771/2011

taccatgcaaacaattcaacggagcaggttgacacaatcatggaaaagaacgttactgttacacatgcccaagacatactggagaagacacacaacgggaagctctgcgatctagatggagtgaagcctctaattttaaaagattgcagtgtagctggatggctcctcgggaatccaatgtgtgacgaattcatcaatgtaccagagtggtcttacatagtggagaaggccaatccaaccaatgacctctgttacccaggaagtttcaacgactatgaagaactgaaacatctattgagcagaataaaccattttgagaaaattcagatcatccccaaaagttcttggtccgatcatgaagcatcatcaggagtgagctcagcatgtccatacctgggaagtccctcctttttcagaaacgtggtatggcttatcaaaaagaacagtacatacccaacaataaagaaaacctacaacaataccaaccaagaagatcttttgatactgtggggaattcaccatcctaataatggggcagagcagacaatgctatatcaaaacccaaccacctatatttccattgggacatcaacactaaaccagagattggtaccaaaaatagctactagatccaaagtaaacgggcaaagtggaagaatggagtttttttggacaattttaaaaccaaatgatgcaatcaacttcgagagtaatggaaacttcattgctccagaatatgcatacaaaattgtcaagaaaggggactcagcaattatgaaaagtgaattggaatatggtaactgcaacaccaagtgtcaaaccccaatgggagcgataaactctagtatgccattccacaacatacaccctatcaccattggggaatgccccaaatatgtgaaatcaaacagactagtcttagcaacagggctcagaaacagccctcaaagagagagcagaaggaaaaagaga

>H5N1_A/Indonesia/NIHRD11771/2011(H5N1)

taccatgcaaacaattcaacggagcaggttgacacaatcatggaaaagaacgttactgttacacatgcccaagacatactggagaagacacacaacgggaagctctgcgatctagatggagtgaagcctctaattttaaaagattgcagtgtagctggatggctcctcgggaatccaatgtgtgacgaattcatcaatgtaccagagtggtcttacatagtggagaaggccaatccaaccaatgacctctgttacccaggaagtttcaacgactatgaagaactgaaacatctattgagcagaataaaccattttgagaaaattcagatcatccccaaaagttcttggtccgatcatgaagcatcatcaggagtgagctcagcatgtccatacctgggaagtccctcctttttcagaaacgtggtatggcttatcaaaaagaacagtacatacccaacaataaagaaaacctacaacaataccaaccaagaagatcttttgatactgtggggaattcaccatcctaataatggggcagagcagacaatgctatatcaaaacccaaccacctatatttccattgggacatcaacactaaaccagagattggtaccaaaaatagctactagatccaaagtaaacgggcaaagtggaagaatggagtttttttggacaattttaaaaccaaatgatgcaatcaacttcgagagtaatggaaacttcattgctccagaatatgcatacaaaattgtcaagaaaggggactcagcaattatgaaaagtgaattggaatatggtaactgcaacaccaagtgtcaaaccccaatgggagcgataaactctagtatgccattccacaacatacaccctatcaccattggggaatgccccaaatatgtgaaatcaaacagactagtcttagcaacagggctcagaaacagccctcaaagagagagcagaaggaaaaagaga

>H5N1_A/Indonesia/NIHRD11797/2011(H5N1)

taccatgcaaacaattcaacggagcaggttgacacaatcatggagaagaacgttactgttacacatgcccaagacatactggagaagacacacaatgggaagctctgcgatctagatggcgtgaagcctctaattttaaaagattgcagtgtagctggatggctccttgggaacccaatgtgtgacgaattcatcaatgtaccagaatggtcttacatagtggagaaggccaatccaaccaatgacctctgttacccaggaagtttcaacgactatgaagaactgaaacacctattgagcagaataaaccattttgagaaaattcagatcatccccaaaagttcttggtccgatcatgaagcatcatcaggagtgagctcagcatgtccatacctgggaagtccctcctttttcagaaacgtggtatggcttatcaaaaagaacagtacatacccaacaataaagaaaacctacaacaataccaaccaagaagatcttttgatactgtggggaattcaccatcctaataatgttgcagagcagacaatgctatatcaaaacccaaccacctatatttccattgggacatcaacactaaaccagagattggtaccaaaaatagctactagatccaaagtaaacgggcaaagtggaaggatggaatttttttggacaattttaaaaccaaatgatgcaatcaacttcgagagtaatggaaatttcattgctccagaatatgcatacaaaattgtcaagaaaggggactcagcaattatgaaaagtgaattggagtatggtaactgcaacaccaagtgtcaaactccaatgggagcgataaactctagtatgccattccacaacatacaccctatcacgattggggaatgccccaaatatgtgaaatcaaacagattagtcttagcaacagggctcagaaacagccctcaaagagagagcagaaggaaaaagaga

>H5N1_A/Indonesia/NIHRD11931/2012

taccatgcaaacaattcaacggaacaggttgacacaatcatggaaaagaacgttactgttacacatgcccaagacatactggaaaggacacacaacgggaagctctgcgatctagatggagtgaagcctctaattttaaaagattgcagtgtagctggatggctcctcgggaacccaatgtgtgacgaattcatcaacgtaccagaatggtcttacatagtggagaaggccaatccgaccaacgacctctgttacccagggagtttcaacgactatgaagaactgaaacacctattaagcagaataaaccatttcgagaaaattcagatcatccctaaaaattcttggtccgatcatgaagcatcatcaggagtaagctcagcatgcccatatctgggaagtccctccttttttagaaatgtggtatggcttatcaaaaagaacagtacgtacccaacaataaagaaaacctacaataataccaaccaagaagatcttttgatactgtggggaattcaccatcctaataatgaggcagagcagacaatgctatatcaaaacccaaccacctatatttccattgggacatcaacactaaaccagagattggtacccaaaatagctactagatccaaagtaaacgggcaaagtggaaggatggagtttttctggacaattttaaaaccaaatgatgcaatcaacttcgagagtaatggaaatttcattgctccagaatatgcatacaaaattgttaagaaaggggactcagcaattatgaaaagtgaattggaatatggtaactgcaacaccaagtgtcaaactccaatgggggcgataaactctagtatgccattccacaacatacaccctctcaccatcggagaatgccccaaatatgtgaaatcaaacagattagtcctagcgacagggctcagaaatagccctcaaagagaaagcagaaggaaaaagaga

>H5N1_A/Indonesia/NIHRD11949/2012

taccatgcaaacaattcaacggaacaggttgacacaatcatggaaaagaacgttactgttacacatgcccaagacatactggaaaggacacacaacgggaagctctgcgatctagatggagtgaagcctctaattttaaaagattgcagtgtagctggatggctcctcgggaacccaatgtgtgacgaattcatcaacgtaccagaatggtcttacatagtggagaaggccaatccgaccaacgacctctgttacccagggagtttcaacgactatgaagaactgaaacacctattaagcagaataaaccatttcgagaaaattcagatcatccctaaaaattcttggtccgatcatgaagcatcatcaggagtaagctcagcatgcccatatctgggaagtccctccttttttagaaatgtggtatggcttatcaaaaagaacagtacgtacccaacaataaagaaaacctacaataataccaaccaagaagatcttttgatactgtggggaattcaccatcctaataatgaggcagagcagacaatgctatatcaaaacccaaccacctatatttccattgggacatcaacactaaaccagagattggtacccaaaatagctactagatccaaagtaaacgggcaaagtggaaggatggagtttttctggacaattttaaaaccaaatgatgcaatcaacttcgagagtaatggaaatttcattgctccagaatatgcatacaaaattgttaagaaaggggactcagcaattatgaaaagtgaattggaatatggtaactgcaacaccaagtgtcaaactccaatgggggcgataaactctagtatgccattccacaacatacaccctctcaccatcggagaatgccccaaatatgtgaaatcaaacagattagtcctagcgacagggctcagaaatagccctcaaagagaaagcagaaggaaaaagaga

>H5N1_A/Indonesia/NIHRD12078/2012

taccatgcaaacaattcaacggagcaggttgacacaatcatggaaaagaacgttactgttacacatgcccaagacatactggagaagacacacaatgggaagctctgcgatctagatggagtgaagcctctaattttaaaagattgcagtgtagctggatggctccttgggaacccaatgtgtgacgaattcatcaatgtaccagaatggtcttacatagtggagaaggccaatccaaccaatgacctctgttacccaggaagtttcaacgactatgaagaactgaaacacctattgagcagaataaaccagtttgagaaaattcagatcatccccaaaagttcttggtccgatcatgaagcatcatcaggagtgagctcagcatgtccatacctgggaagcccttcctttttcagaaacgtggtatggcttatcaaaaagaacagtacatacccaacaataaagaaaacctacaacaataccaaccaagaaaatcttttgatactgtggggaattcaccatcctaataatgaggcagagcagacaatgctatatcaaaacccaaccacctatatttccattgggacatcaacactaaaccagagattggtaccaaaaatagctactagatccaaagtaaacgggcaaagtggaaggatggagtttttttggacaattttaaaaccaaatgatgcaatcaacttcgagagtaatggaaatttcattgctccagaatatgcatacaaaattgtcaagaaaggggactcagcaattatgaaaagtgaattggaatatggtaattgcaacaccaagtgtcaaactccaatgggagcgataaactctagtatgccattccacaacatacaccctatcacgattggggaatgccccaaatatgtgaaatcaaacagattagtcttagcaacagggctcagaaacagccctcaaagagagagcagaaggaaaaagaga

>H5N1_A/Indonesia/NIHRD12130/2012

taccatgcaaacaattcaacggagcaggttgacacaatcatggaaaagaacgttactgttacacatgcccaagacatactggagaagacacacaatgggaagctctgcgatctagatggagtgaagcctctaattttaaaagattgcagtgtagctggatggctccttgggaacccaatgtgtgacgaattcatcaatgtaccagaatggtcttacatagtggagaaggccaatccaaccaatgacctctgttacccaggaagtttcaacgactatgaagaactgaaacacctattgagcagaataaaccagtttgagaaaattcagatcatccccaaaagttcttggtccgatcatgaagcatcatcaggagtgagctcagcatgtccatacctgggaagcccttcctttttcagaaacgtggtatggcttatcaaaaagaacagtacatacccaacaataaagaaaacctacaacaataccaaccaagaaaatcttttgatactgtggggaattcaccatcctaataatgaggcagagcagacaatgctatatcaaaacccaaccacctatatttccattgggacatcaacactaaaccagagattggtaccaaaaatagctactagatccaaagtaaacgggcaaagtggaaggatggagtttttttggacaattttaaaaccaaatgatgcaatcaacttcgagagtaatggaaatttcattgctccagaatatgcatacaaaattgtcaagaaaggggactcagcaattatgaaaagtgaattggaatatggtaattgcaacaccaagtgtcaaactccaatgggagcgataaactctagtatgccattccacaacatacaccctatcacgattggggaatgccccaaatatgtgaaatcaaacagattagtcttagcaacagggctcagaaacagccctcaaagagagagcagaaggaaaaagaga

>H5N1_A/Indonesia/NIHRD12162/2012

taccatgcaaacaattcaacggagcaggttgacacaatcatggaaaagaacgttactgttacacatgcccaagacatactggaaaagacacacaacgggaagctctgcgatctagatggagtgaagcctctaattttaaaagattgcagtgtagctggatggctcctcgggaacccaatgtgtgacgaattcatcaatgtaccagaatggtcttacatagtggagaaggccaatccaaccaatgacctctgttacccagggagtttcaacgactatgaagaactgaaacacctattgagcagaataaaccattttgagaaaattcagatcatccccaaaagttcttggtccgatcatgaagcatcatcaggagtgagctcagcatgtccatatctgggaagtccctccttttttagaaatgtggtatggcttatcaaaaagaacagtacgtacccaacaataaagaaaacctacaataataccaaccaagaagatcttttgatactgtggggaattcaccatcctaataatgaagcagagcagataatgctatatcaaaacccaaccacctatatttccattgggacatcaacactaaaccagaggttggtaccaaaaatagctactagatcaaaagtaaacgggcaaagtggaaggatggagtttttctggacaattttaaaaccaaatgatgcaatcaacttcgagagtaatggaaatttcattgctccagaatatgcatacaaaattgtcaagaaaggggactcggcaattatgaaaagtgaattggaatatggtaactgtaacaccaagtgtcaaactccaatgggggcgataaactctagtatgccattccacaacatacaccctctcaccatcggggaatgccccaaatatgtgaaatcaaatagattagtcctagcgacagggctcagaaacagccctcaaagagaaagcaaaaggaaaaagaga

>H5N1_A/Indonesia/NIHRD12377/2012

taccatgcaaacaattcaacggagcaggttgacacaatcatggagaagaacgttactgttacacatgcccaagacatactggagaagacacacaatgggaagctctgcgatctagatggcgtgaagcctctaattttaaaagattgcagtgtagctggatggctccttgggaacccaatgtgtgacgaattcatcaatgtaccagaatggtcttacatagtggagaaggccaatccaaccaatgacctctgttacccaggaagtttcaacgactatgaagaactgaaacacctattgagcagaataaaccattttgagaaaattcagatcatccccaaaagttcttggtccgatcatgaagcatcatcaggagtgagctcagcatgtccatacctgggaagtccctcctttttcagaaacgtggtatggcttatcaaaaagaacagtacatacccaacaataaagaaaacctacaacaataccaaccaagaagatcttttgatactgtggggaattcaccatcctaataatgatgcagagcagacaatgctatatcaaaacccaaccacctatatttccattgggacatcaacactaaaccagagattggtaccaaaaatagctactagatccaaagtaaacgggcaaagtggaaggatggaatttttttggacaattttaaaaccaaatgatgcaatcaacttcgagagtaatggaaatttcattgctccagaatatgcatacaaaattgtcaagaaaggggactcagcaattatgaaaagtgaattggagtatggtaactgcaacaccaagtgtcaaactccaatgggagcgataaactctagtatgccattccacaacatacaccctatcacgattggggaatgccccaaatatgtgaaatcaaacagattagtcttagcaacagggctcagaaacagccctcaaagagagagcagaaggaaaaagaga

>H5N1_A/Indonesia/NIHRD12452/2012

taccatgcaaacaattcaacggagcaggttgacacaatcatggaaaagaacgttactgttacacatgcccaagacatactggagaagacacacaatgggaagctctgcgatctagatggagtgaagcctctaattttaaaagattgcagtgtagctggatggctccttgggaacccaatgtgtgacgaattcatcaatgtaccagaatggtcttacatagtggagaaggccaatccaaccaatgacctctgttacccaggaagtttcaacgactatgaagaactgaaacacctattgagcagaataaaccattttgagaaaattcagatcatccccaaaagttcttggtccgatcatgaagcatcatcaggagtgagctcagcatgtccatatctgggaagtccctcctttttcagaaacgtggtatggcttatcaaaaagaacagcacatacccaacaataaagaaaacctacaacaataccaaccaagaagatcttttgatactgtggggaattcaccatcctaataatgaggcagagcagacaatactatatcaaaacccaaccacctatatttccattgggacatcaacactaaaccagagattggtaccaaaaatagctactagatccaaagtaaacgggcaaagtggaaggatggaatttttttggacaattttaaaaccaaatgatgcaatcaacttcgagagtaatggaaatttcattgctccagaatatgcatacaaaattgtcaagaaaggggactcagcaattatgaaaagtgaattggaatatggtaattgcaacaccaagtgtcaaactccaatgggagcgataaactctagtatgccattccacaacatacaccctatcacgattggggaatgccccaaatatgtgaaatcaaacagattagtcttagcaacagggctcagaaacagccctcaaagagagagcagaaggaaaaagaga

>H5N1_A/Indonesia/NIHRD12550/2012

taccatgcaaacaattcaacggagcaggttgacacaatcatggaaaagaacgttactgttacacatgcccaagacatactggagaagacacacaatgggaagctctgcgatctagatggagtgaagcctctaattttaaaagattgcagtgtagctggatggctccttgggaacccaatgtgtgacgaattcatcaatgtgccagaatggtcttacatagtggagaaggccaatccaaccaatgacctctgttacccaggaagtttcaacgactatgaagaactgaaacacctattgagcagaataaaccattttgagaaaattcagatcatccccaaaagttattggtccgatcatgaagcatcatcaggagtgagctcagcatgtccatacctgggaagcccctcctttttcagaaacgtggtatggcttatcaaaaagaacagtacatacccaacaataaagaaaacctacaacaataccaaccaagaagatcttttgatactgtggggaattcaccatcctaataatgaggcagagcagacaatgctatatcaaaacccaaccacctatatttccattgggacatcaacattaaaccagagattggtaccaaaaatagctactagatccaaagtaaacgggcaaagtggaaggatggaatttttttggacaattttaaaaccaaatgatgcaatcaacttcgagagtaatggaaatttcattgctccagaatatgcatacaaaattgtcaagaaaggggactcagcaattatgaaaagtgaattggaatatggcaattgcaacaccaagtgtcaaactccaatgggagcgataaattctagtatgccattccacaacatacaccctatcacgattggggaatgccccaaatatgtgaaatcaaacagattagtcttagcaacagggctcagaaacagccctcaaagagagagcagaaggaaaaagaga

>H5N1_A/Indonesia/NIHRD13157/2013

taccatgcaaacaattcaacggaacaggttgacacaatcatggaaaagaacgttagtgttacacatgcgcaagacatactggagaagacacacaacgggaagctctgtgatctagatggagtgaagcctctaattttaaaagattgcagtgtagctggatggctcctcgggaacccaatgtgtgacgaattcatcaatgtaccagaatggtcttacatagtagagaaggccaatccaaccaatgacctctgttacccaggaagtttcaccgactatgaagaactgaaacatctattgagcagaataaaccattttgagaaaattcagatcatccccaaaagttcttggtccgatcatgaagcatcatcaggagtgagctcagcatgtccatacctgggaagtccatcctttttcagaaacgtggtatggctcatcaaaaagaacagtacatacccaacaataaagaaaacctacaacaataccaaccaagaagatcttttgatactgtggggaattcaccatcctaataatgaggcagagcagacaatgctatatcaaaccccaaccacctatatttccattgggacatcaacactaaaccagagattggcaccaaaaatagctactagatccaaagtaaacgggcaaagtggaaggatggagtttttttggacaattttaaaaccaaatgatgcaatcaacttcgagagtaatggaaatttcattgctccagaatatgcatacaaaatcgtcaagaaaggggactcagcaattatgaaaagtgaattggaatatggtaactgcaacaccaagtgtcaaactccaatgggagcgataaactctagtatgccattccacaacatacaccctatcaccattggggaatgccccaaatatgtgaaatcaaacagattagtcttagcaacagggctcagaaacagccctcaaagggagagcagaaggaaaaagaga

>H5N1_A/Indonesia/NIHRD13233/2013

taccatgcaaacaattcaacggaacaggttgacacaatcatggaaaagaacgttactgttacacatgcgcaagacatactggagaagacacacaacgggaagctctgtgatctagatggagtgaagcctctaattttaaaagattgcagtgtagctggatggctcctcgggaacccaatgtgtgacgaattcatcaatgtaccagaatggtcttacatagtagagaaggccaatccaaccaatgacctctgttacccaggaagtttcaacgactatgaagaactgaaacacctattgagcagaataaaccattttgagaaaattcagatcatccccaaaagttcttggtccgatcatgaagcatcatcaggagtgagctcagcatgtccatatctgggaagtccctccttttttagaaatgtggtatggcttatcaaaaagaacagtacgtacccaacaataaagaaaacctacaataataccaaccaagaagatcttttgatactgtggggaattcaccatcctaataatgaagcagagcagacaatgctatatcaaaacccaaccacctatatttccattgggacatcaacactaaaccagagactggtaccaaaaatagctactagatccaaagtgaacggacaaagtggaaggatggagttcttctggacaattttaaaaccaaatgatgcaattaacttcgagagtaatggaaatttcattgctccagaatatgcatacaaaattgtcaagaaaggggattcggcaattatgaaaagtgaattggaatatggtaactgtaacaccaagtgtcaaactccaatgggggcgataaactctagtatgccattccacaacatacaccctctcaccatcggggaatgccccaaatatgtgaaatcaaacagattagtcctagcgacagggctcagaaatagccctcaaagggaaagcagaaggaaaaagaga

>H5N1_A/Indonesia/NIHRD13269/2013

taccatgcaaacaattcaacggaacaggttgacacaatcatggaaaagaatgttacagttacacatgcccaagacatactggaaaggacacacaacgggaagctctgcgatctagatggagtgaagcctctaattttaaaagattgcagtgtagctggatggctcctcgggaacccaatgtgtgacgaatttatcaatgtcccagaatggtcttacatagtggagaaggccaatccgactaacgatctctgttacccagggagtttcaacgactatgaagaactgaaacacctattaagcagaataaaccatttcgagaaaattcagatcatccctaaaagttcttggtccgatcatgaagcatcatcaggagtgagctcagcatgcccatatctgggaagtccctccttttttagaaatgtggtatggcttatcaaaaagaacagtacgtacccaacaataaagaaaacctacaataataccaaccaagaagatcttttgatactgtggggaattcaccatcctaataatgaagcagagcagacaatgctatatcaaaacccaaccacttatatttccattgggacatcaacactaaaccagagactggtaccaaaaatagctactagatccaaagtgaacggacaaagtggaaggatggagttcttctggacaattttaaaaccaaatgatgcaattaacttcgagagtaatggaaatttcattgctccagaatatgcatacaaaattgtcaagaaaggggattcggcaattatgaaaagtgaattggaatatggtaactgcaacaccaagtgtcaaactccaatgggggcgataaactctagtatgccattccacaacatacaccctctcaccattggggaatgccccaaatatgtgaaatcaaacagattagtcctagcgacagggctcagaaatagccctcaaagggaaagcagaaggaaaaagaga

>H5N1_A/Indonesia/NIHRD14122/2014

taccatgcaaacaattcaacggaacaggttgacacaatcatggaaaagaacgttagtgttacacatgcgcaagacatactggagaagacacacaacgggaagctctgtgatctagatggagtgaagcctctaattttaaaagattgcagtgtagctggatggctcctcgggaacccaatgtgtgacgaattcatcaatgtaccagaatggtcttacatagtagagaaggccaatccaaccaatgacctctgttacccaggaagtttcaacgactatgaggaactgaaacacctattgagcagaataaaccattttgagaaaattcagatcatccccaaaagttcttggtccgatcatgaagcatcatcaggagtgagctcagcatgtccatacctgggaagtccatcctttttcagaaacgtggtatggcttatcaaaaagaacagtacatacccaacaataaagaaaacctacaacaataccaaccaagaagatcttttgatactgtggggaattcaccatcctaataatgaggcagagcagacaatgctatatcaaaacccaaccacctatatttccattgggacatcaacactaaaccagagattggcaccaaaaatagctactagatccaaagtaaacgggcaaagtggaaggatggagtttttttggacaattttaaaaccaaatgatgcaatcaacttcgagagtaatggaaatttcattgctccagaatatgcatacaaaattgtcaagaaaggggactcagcaattatgaaaagtgaattggaatatggtaactgcaacaccaagtgtcaaactccaatgggagcgataaactctagtatgccattccacaacatacaccctatcaccattggggaatgccccaaatatgtgaaatcaaacagattagttttagcaacagggctcagaaacagccctcaaagagagagcagaagaaaaaagaga

>H5N1_A/Indonesia/NIHRD14157/2014

taccatgcaaacaattcaacggaacaggttgacacaatcatggaaaagaacgttacagttacacatgcccaagacatactggagaagacacacaacgggaagctctgtgatctagatggagtgaagcctctaattttaaaagattgcagtgtagctggatggctcctcgggaacccaatgtgtgacgaattcatcaatgtaccagaatggtcttacatagtggagaaggccaatccgaccaatgacctctgttacccaggaagtttcaccgactatgaagaactgaaacatctattgagcagaataaaccattttgagaaaattcagatcatccccaaaagttcttggtccgatcatgaagcatcatcaggagtgagctcagcatgtccatatctgggaagtccctccttttttagaaatgtggtatggcttatcaaaaagaacagtacgtacccaacaataaagaaaacctacaataataccaaccaagaagatcttttgatactgtggggaattcaccatcctaataatgaagcagagcagacaatgctatatcaaaacccaaccacctatatttccattgggacatcaacactaaaccagagactggtaccaaaaatagctactagatccaaagtgaacggacaaagtgggaggatggagttcttctggacaattttaaaaccaaatgatgcaattaacttcgagagtaatggaaatttcattgctccagaatatgcatacaaaattgtcaagaaaggggattcggcaattatgaaaagtgaattggaatatggtgactgtaacaccaagtgtcaaactccaatgggggcgataaactctagtatgccattccacaacatacaccctctcaccatcggggaatgccccaaatatgtgaaatcaaacagattagtcctagcgacagggctcagaaatagccctcaaagggaaagcagaaggaaaaagaga

>H5N1_A/Indonesia/NIHRD15023/2015

taccatgcaaacaattcaacagaacaggttgacacaatcatggaaaagaatgttacagttacacatgcccaagacatactggaaaggacacacaacgggaagctctgcgatctagatggagtgaagcctctaattttaaaagattgcagtgtagctggatggctcctcgggaacccaatgtgtgacgaatttatcaatgtcccagaatggtcttacatagtggagaaggccaatccgactaacgatctctgttacccagggagtttcaacgattatgaagaactgaaacacctattaagcagaataaaccatttcgagaaaattcagatcatccctaaaaattcttggtccgatcatgaagcatcatcaggagtgagctcagcatgcccatatctgggaagtccctccttttttagaaatgtggtatggcttatcaaaaagaacaatacgtacccaacaataaagaaaacctacaataataccaaccaagaagatcttttgatactgtggggaatccaccatcctaataatgaaatagagcagacaatgttatatcaaaacccaaccacctatatttccattgggacatcaacactaaaccagagactggtaccaaaaatagctactagatccaaagtgaacgggcaaagtggaaggatggagttcttctggacaattttaaaaccaaatgatgcaattaacttcgagagtaatggaaatttcattgccccagaatatgcatacaaaattgtcaagaaaggggattcggcaattatgaaaagtgaattggaatatggtaactgtaacaccaagtgtcaaactccaatgggggcgataaactctagtatgccattccacaacatacaccctctcaccatcggggaatgccccaaatatgtgaaatcaaacagattagtcctagcaacagggctcagaaatagccctcaaagggaaagcagaaggaaaaagaga

>H5N1_A/Indonesia/NIHRD15028/2015

taccatgcaaacaattcaacagaacaggttgacacaatcatggaaaagaatgttacagttacacatgcccaagacatactggaaaggacacacaacgggaagctctgcgatctagatggagtgaagcctctaattttaaaagattgcagtgtagctggatggctcctcgggaacccaatgtgtgacgaatttatcaatgtcccagaatggtcttacatagtggagaaggccaatccgactaacgatctctgttacccagggagtttcaacgattatgaagaactgaaacacctattaagcagaataaaccatttcgagaaaattcagatcatccctaaaaattcttggtccgatcatgaagcatcatcaggagtgagctcagcatgcccatatctgggaagtccctccttttttagaaatgtggtatggcttatcaaaaagaacaatacgtacccaacaataaagaaaacctacaataataccaaccaagaagatcttttgatactgtggggaatccaccatcctaataatgaaatagagcagacaatgttatatcaaaacccaaccacctatatttccattgggacatcaacactaaaccagagactggtaccaaaaatagctactagatccaaagtgaacgggcaaagtggaaggatggagttcttctggacaattttaaaaccaaatgatgcaattaacttcgagagtaatggaaatttcattgccccagaatatgcatacaaaattgtcaagaaaggggatccggcaattatgaaaagtgaattggaatatggtaactgtaacaccaagtgtcaaactccaatgggggcgataaactctagtatgccattccacaacatacaccctctcaccatcggggaatgccccaaatatgtgaaatcaaacagattagtcctagcaacagggctcagaaatagccctcaaagggaaagcagaaggaaaaagaga

>H5N1_A/Indonesia/NIHRD17109/2017

tatcatgcaaataactcgacagagcaggttgacacaataatggaaaagaacgttactgttacacatgcccaagacatactggaaaagacacacaacgggaagctctgcgatctaaatggagtgaagcctctgattttaaaggattgtagtgtagcgggatggctccttggaaatccattgtgtgacgaattcatcaatgtgccagaatggtcttacatagtagagaaggccaatccagccaatgacctctgttacccaggaaatttcaacgattatgaagaattgaaacacctattgagtaggataaaccattttgagaaaatacagatcatccccaaagattcttggtcagatcatgaagcctcattgggggtgagcgcagcgtgttcataccagggaaattcctccttcttcagaaatgtggtgtggcttatcaaaaagaacaatacatatccaacaataaagaaaagctacaataacaccaaccaagaagacctcttgatactgtgggggatccatcatcccaatgatgaggcagagcagataaagctttatcaaaacccaactacctatgtttccattgggacttcaacactaaaccagagattggtacccaaaatagccactagatccaaaataaacgggcaaaggggcaggatagatttcttttggataattttaaaaccgaatgatgcaatccactttgagagtaatggaaatttcattgctccagaatatgcatacaaaattgtcaagaaaggagactccacaatcatgagaagtgaagtggaatatggtaactgcaacaccaggtgccaaaccccaataggggcgataaactctagtatgccattccacaacatacaccctctcaccatcggagaatgtcccaaatatgtgaagtcaaacaaattagtccttgcaactgggctcagaaatagtcctcaaagagagagaagaagaaaaaaaaga

>H5N1_A/Indonesia/NIHRD7393/2008

taccatgcaaacaattcaacagagcaggttgacacaatcatggaaaagaacgttactgttacacatgcccaagacatactggaaaagacacacaacgggaagctctgcgatctagatggagtgaagcctttaattttaagagattgtagtgtagctggatggctcctcgggaacccaatgtgtgacgaattcatcaatgtaccggaatggtcttacatagtggaaaaggccagtccaaccaatgacctctgttacccagggagtttcaacgactatgaagaactgaaacacctattgagcagaataaaccattttgagaaaattcaaatcatccccaaaagttcttggtccgatcatgaagcctcatcaggagtgagctcagcatgtccatacctgggaagtccctccttttttagaaatgtgatatggcttatcaaaaagaacagtacatacccaacaataaagaaaagctacaataacaccaaccaagaagatcttttggtactgtggggaattcaccatcctaatgatgcggcagagcagacaaggctatatcaaaacccaaccacctacatttccattgggacatcaacactaaaccagagattggtaccaaaaatagctactagatctaaagtaaacgggcaaagtggaaggatggagttcttctggacaattttaaaacctaatgatgcaatcaacttcgagagtaatggaaatttcattgctccagaatatgcatacaaaattgtcaagaaaggggactcagcaattatgaaaagtgaattggaatatggtaactgcaacaccaaatgtcaaactccaatgggggcgataaactctagtatgccattccacaacatacaccctctcaccatcggggaatgccccaaatatgtaaaatcaaacagattagtccttgcaacagggctcagaaatagccctcaaagagagagcagaagaaaaaagaga

>H5N1_A/Indonesia/NIHRD7503/2008

taccatgcaaacaattcaacggagcaggttgacacaatcatggaaaagaacgttactgttacacatgcccaagacatactggaaaagacacacaacgggaagctctgcgatctagatggagtgaagcctctaattttaaaagattgtagtgtagctggatggctcctcgggaacccaatgtgtgacgaattcatcaacgtaccggaatggtcttacatagtggagaaggccaatccaactaatgacctctgttacccagggagtttcaacgactatgaagaactgaaacacctattgagcagaataaatcattttgagaaaattcagatcatccccaaaagttcttggtccgatcatgaagcctcatcaggagtgagctcagcatgtccatacctgggaagtccctccttttttagaaatgtggtatggcttatcaaaaagaacagtacgtacccaacaataaagaaaacctacaataataccaaccaagaagatcttttgatactgtggggaattcaccatcctaataatgaagcagagcagacaatgctatatcaaaacccaaccacctatatttccattgggacatcaacactaaaccagagattggtaccaaaaatagctactagatccaaagtaaacgggcaaagtggaaggatggagtttttctggacaattttaaaaccaaatgatgcaatcaacttcgagagtaatggaaatttcattgctccagaatatgcatacaaaattgtcaagaaaggggactcagcaattatgaaaagtgaattggaatatggtaactgcaacaccaagtgtcaaactccgatgggggcgataaactctagtatgccattccacaacatacaccctctcaccatcggggaatgccccaagtatgtgaaatcaaacagattagtcctagcaacagggctcagaaatagccctcaaagagagagcagaagaagaaagaga

>H5N1_A/Indonesia/NIHRD7781/2008

taccatgcaaacaactcaacagaacaggttgacacaataatggaaaagaacgttactgttacacatgcccaagacatactggaaaagacacacaacgggaagctctgcgatctagatggagtgaagcccctaatcttaagagattgtagtgtagctggatggctcctcgggaatccaatgtgtgacgaattcatcaatgtaccggaatggtcttacatagtggagaagtccaatccagccaatgacctctgttacccagggagtttcaacgactatgaagaactgaaacacctattgagcagaataaaccactttgagaaaattcagatcatccccaaaagttcttggtccgatcatgaagcctcatcaggagtgagctcagcatgcccatacctgggaagtccttccttttttagaaatgtggtatggcttatcaaaaagaacaatacatacccaacaataaagaaaagctacaataataccaaccaagaagatcttttggtactgtgggggattcaccatcctaatgatgcagcggagcagacaaggctatatcaaaatccaactacctatatttccgttgggacctcaacactaaatcagagatcggtaccaagaatagctactagatccaaagtaaacgggcaaagtggaagaatggagttcttctggacaattttaaaaccgaatgatgcaatcaatttcgaggttaatggaaatttcattgctccagaatatgcatacaaaattgtcaagaaaggggactcagcaattataaaaagtgaattggaatatggtaactgcaacaccaagtgtcaaactccaatgggggcgataaactctagtatgccattccacaacatacaccctctcaccatcggggaatgccccaaatatgtgaaatcaaacagattagtccttgcaacagggctcagaaatagccctcaaagggagggaagaagaaaaaagaga

>H5N1_A/Indonesia/NIHRD7802/2008

taccatgcaaacaattcaacggagcaggttgacacaatcatggaaaagaacgttactgttacacatgcccaagacatactggaaaagacacacaacgggaagctctgcgatctagatggagtgaagcctctaattttaaaagattgtagtgtagctggatggctcctcgggaacccaatgtgtgacgaattcatcaacgtaccggaatggtcttacatagtggagaaggccaacccaaccaatgacctctgttacccagggagtttcaacgactatgaagaactgaaacacctattgagcagaataaaccattttgagaaaattcagatcatccccaaaagttcttggtccgatcatgaagcctcatcaggagtgagctcagcatgtccatacctgggaagtccctccttttttagaaatgtggtatggcttatcaaaaagaacagtacgtacccaacaataaagaaaacctacaataataccaaccaagaagatcttttgatactgtggggaattcaccatcctaataatggggcagagcagacaatgctatatcaaaacccaaccacctatatttccattgggacatcaacactaaaccagagattggtaccaaaaatagctactagatccaaagtaaacgggcaaagtggaaggatggagtttttctggacaattttaaaaccaaatgatgcaatcaacttcgagagtaatggaaatttcattgctccagaatatgcatacaaaattgtcaagaaaggggactcagcaattatgaaaagtgaattggaatatggtaactgcaacaccaagtgtcaaactccaatgggggcgataaactctagtatgccattccacaacatacaccctctcaccatcggggaatgccccaaatatgtgaaatcaaacagattagtcctagcaacagggctcagaaatagccctcaaagagagagcagaagaaaaaagaga

>H5N1_A/Indonesia/NIHRD7988/2008

taccatgcaaacaattcaacagagcaggttgacacaatcatggaaaagaacgttactgttacacatgcccaagacatactggaaaagacacacaacgggaagctctgcgatctagatggagtgaagcctttaattttaagagattgtagtgtagctggatggctcctcgggaacccaatgtgtgacgaattcatcaatgtaccggaatggtcttacatagtggaaaaggccagtccaaccaatgacctctgttacccagggagtttcaacgactatgaagaactgaaacacctattgagcagaataaaccattttgagaaaattcaaatcatccccaaaagttcttggtccgatcatgaagcctcatcaggagtgagctcagcatgtccatacctgggaagtccctccttttttagaaatgtgatatggcttatcaaaaagaacagtacatacccaacaataaagaaaagctacaataacaccaaccaagaagatcttttggtactgtggggaattcaccatcctaatgatgcggcagagcagacaaggctatatcaaaacccaaccacctacatttccattgggacatcaacactaaaccagagattggtaccaaaaatagctactagatctaaagtaaacgggcaaagtggaaggatggagttcttctggacaattttaaaacctaatgatgcaatcaacttcgagagtaatggaaatttcattgctccagaatatgcatacaaaattgtcaagaaaggggactcagcaattatgaaaagtgaattggaatatggtaactgcaacaccaaatgtcaaactccaatgggggcgataaactctagtatgccattccacaacatacaccctctcaccatcggggaatgccccaaatatgtaaaatcaaacagattagtccttgcaacagggctcagaaatagccctcaaagagagagcagaagaaaaaagaga

>H5N1_A/Indonesia/NIHRD8987/2008

taccatgcaaacaattcaacggagcaggttgacacaatcatggaaaagaacgttactgttacacatgcccaagacatactggaaaagacacacaacgggaagctctgcgatctagatggagtgaagcctctaattttaaaagattgcagcgtagctggatggctcctcgggaacccaatgtgtgacgaattcatcaacgtaccagaatggtcttacatagtggagaaggccaatccgaccaatgacctctgttacccagggagtttcaacgactatgaagaactgaaacacctattgagcagaataaaccattttgagaaaattcagatcatccccaaaagttcttggtccgatcatgaagcatcatcaggagtgagctcagcatgcccatacctgggaagtccctccttttttagaaatgtggtatggcttatcaaaaagaacagtacgtacccaacaataaagaaaacctacaataataccaaccaagaagatcttttgatactgtggggaattcaccatcctaataatgaggcagagcagacaatgctatatcaaaacccaaccacctatatttccattgggacatcaacactaaaccagagattggtaccaaaaatagctactagatccaaagtaaacgggcaaagtggaaggatggagtttttctggacaattttaaaaccaaatgatgcaatcaacttcgagagtaatggaaatttcattgctccagaatatgcatacaaaaatgtccagaaaggggactcagcaattaggaaaagtgaattggaatatggtaactgcaacaccaagtgtaaaactccaatgggggcgataaactctagtatgccattccacaacatacaccctctcaccatcggggaatgccccaaatatgtgaaatcaaacagattagtcctagcaacagggctcagaaatagccctcaaagagaaagcagaaggaaaaagaga

>H5N1_A/Indonesia/NIHRD9152/2009

taccatgcaaacaattcaacagagcaagttgacacaatcatggaaaagaacgttactgttacacatgcccaagacatactggagaagacacacaacgggaagctctgcgacctagatggggtgaagcctctaattttaagagattgtagtgtagctggatggctcctcgggaatccaatgtgtgacgaattcatcaatgtaccagaatggtcttacatagtagagaaggccaatccaaccaatgacctctgttacccagggagtttcaacgactatgaagaactgaaacacctattgagcagaataaaccattttgagaaaattcaaatcatccccaaaagttcttggtccgatcatgaagcctcatcaggagtgagctcagcatgcccatatctgggaagtccctccttttttagaaatgtggtatggcttatcaaaaagaacggtacatacccaacaataaagaaaagctacaataacaccaaccaagaagatcttttggtactgtggggaattcaccatcctaataatgtggcagagcagacaaggctatatcaaaacccaaccacctatattttcattgggacatcaacactaaaccagagattggcaccaaaaatagctactagatccaaagtaaacgggcaaagtggaaggatggagttattctggacaattttaaaacctaatgatgcaatcaatttcgagagtaatggaaatttcattgctccagaatatgcatacaaaattgtcaagaaaggggactcagcaattatgagaagtgaattggaatatggtaactgcaacaccaagtgtcagactccaatgggggcgataaactctagtatgccattccacaacatacaccctctcaccatcggggaatgccccaaatatgtgaaatcaaacagattagtccttgcaacaggactcagaaatagccctcaaagagagagcagaagaaaaaagaga

>H5N1_A/Indonesia/NIHRD9158/2009

taccatgcaaacaattcaacggagcaggttgacacaatcatggaaaagaacgttactgttacacatgcccaagacatactggaaaaaacacacaacgggaagctctgcgatctagatggagtgaagcctctaattttaaaagattgtagtgtagctggatggctcctcgggaacccaatgtgtgacgaattcatcaacgtaccagaatggtcttacatagtggagaaggccaatccaaccaatgacctctgttacccagggagtttcaacgactatgaagaactgaaacacctattgagcagaataaaccattttgagaaaattcagatcatccccaaaagttcttggtccgatcatgaagcatcatcaggagtgagctcagcatgtccatacctgggaagtccctccttttttagaaatgtggtatggcttatcaaaaagaacagtacgtacccaacaataaagaaaacctacaataataccaaccaagaagatcttttgatactgtggggaattcaccatcctaataatgaggcagagcagacaatgctatatcaaaacccaaccacctatatttccattgggacatcaacactaaaccagagactggtaccaaaaatagctactagatccaaagtaaacgggcaaagtggaaggatggagtttttctggacaattttaaaaccaaatgatgcaatcaacttcgagagtaatggaaatttcattgctccagaatatgcatacaaaattgtcaagaaaggggactcagcaattatgaaaagtgaattggaatatggtaactgcaacaccaagtgtcaaactccaatgggggcgataaactctagtatgccattccacaacatacaccctctcactatcggggaatgccccaaatatgtgaaatcaaacagattagttctagcaacagggctcagaaatagccctcaaagagagagcagaaggaaaaagaga

>H5N1_A/Indonesia/NIHRD9160/2009

taccatgcaaacaattcaacggagcaggttgacacaatcatggaaaagaacgttactgttacacatgcccaagacatactggagaagacacacaacgggaagctctgcgatctagatggagtgaagcctctaattttaaaagattgcagtgtagctggatggctcctcgggaacccaatgtgtgacgaattcatcaacgtaccagaatggtcttacatagtggagaaggccaatccaaccaatgacctctgttacccaggaagtttcaacgactatgaagaactgaaacacctattgagcagaataaaccattttgagaaaattcagatcatccccaaaagttcttggtccgatcatgaagcatcatcaggagtgagctcagcatgtccatacctgggaagtccctccttttttagaaacgtggtatggcttatcaaaaagaacagtacgtacccaacaataaagaaaacctacaataataccaaccaagaagatcttttgatactgtggggaattcaccatcctaataatgaggcagagcagacaatgctatatcaaaacccaaccacctatatttccattgggacatcaacactaaaccagagattggtaccaaaaatagctactagatccaaagtaaacgggcaaagtggaaggatggagtttttttggacaattttaaaaccaaatgatgcaatcaacttcgaagttaatggaaatttcattgctccagaatatgcatacaaaattgtcaagaaaggggactcagcaattatgaaaagtgaattggaatatggtaactgcaacaccaagtgtcaaactccaatgggagcgataaactctagtatgccattccacaacatacaccctctcaccattggggaatgccccaaatatgtgaaatcaaacagattagtcctagcaacagggctcagaaatagccctcaaagagaaagcagaaggaaaaagaga

>H5N1_A/Indonesia/NIHRD9340/2009

taccatgcaaacaattcaacagagcaggttgacacaatcatggaaaagaacgttactgttacacatgcccaagacatactggaaaagacacacaacgggaagctctgcgatctagatggagtgaagcctctaattttaaaagattgcagtgtagctggatggctcctcgggaacccaatgtgtgatgaattcatcaacgtaccggaatggtcttacatagtggaaaaggccaatccaaccaatgacctctgttacccagggagtttcaacgactatgaagaactgaaacacctattgagcagaataaaccattttgagaaaattcagatcatccccaaaagttcttggtccgatcatgaagcatcatcaggagtgagctcagcatgtccatacctgggaagtccctccttttttagaaatgtggtatggcttatcaaaaagaacagtacgtacccaacaataaagaaaacctacaataataccaaccaagaagatcttttgatactgtggggaattcaccatcctaataatgaggcagagcagacaatgctatatcaaaacccaaccacctatatttccattgggacatcaacactaaaccagagatcggtaccaaaaatagctactagatccaaagtaaacgggcaaagtggaagaatggagtttttctggacaattttaaaaccaaatgatgcaatcaacttcgagagtaatggaaatttcattgctccagaatatgcatacaaaattgtcaagaaaggggactcagcaattatgaaaagtgaattggaatatggtaactgcaacaccaagtgtcaaactccaatgggggcgataaactctagtatgccattccacaacatacaccctctcactatcggggaatgccccaaatatgtgaaatcaaacagattagtccttgcaacagggctcagaaatagccctcaaagagagagcagaaggaaaaagaga

>H5N1_A/Indonesia/NIHRD9653/2009

taccatgcaaacaattcaacggagcaggttgacacaatcatggaaaagaacgttactgttacacatgcccaagacatactggaaaaaacacacaacgggaagctctgcgatctagatggagtgaagcctctaattttaaaagattgtagtgtagctggatggctcctcgggaacccaatgtgtgacgaattcatcaacgtaccagaatggtcttacatagtggagaaggccaatccaaccaatgacctctgttacccagggagtttcaacgactatgaagaactgaaacacctattgagcagaataaaccattttgagaaaattcagatcatccccaaaagttcttggtccgatcatgaagcatcatcaggagtgagctcagcatgtccatacctgggaagtccctccttttttagaaatgtggtatggcttatcaaaaagaacagtacgtacccaacaataaagaaaacctacaataataccaaccaagaagatcttttgatactgtggggaattcatcatcctaataatgaggcagagcagacaatgctatatcaaaacccaaccacctatatttccattgggacatcaacactaaaccagagattggtaccaaaaatagctactagatccaaagtaaacgggcaaagtggaaggatggagtttttctggacaattttaaaaccaaatgatgcaatcaacttcgagagtaatggaaatttcattgctccagaatatgcatacaaaattgtcaagaaaggggactcagcaattatgaaaagtgaattggaatatggtaactgcaacaccaagtgtcaaactccaatgggggcgataaactctagtatgccattccacaacatacaccctctcaccatcggggaatgccccaaatatgtgaaatcaaacagattagtcctagcaacagggctcagaaatagccctcaaagagaaagcagaaggaaaaagaga

>H5N1_A/Indonesia/NIHRD9665/2009

taccatgcaaacaattcaacagagcaggttgacacaatcatggaaaagaacgttactgttacacatgcccaagacatactggaaaagacacacaacgggaagctctgcgatctagatggagtgaagcctctaattttaaaagattgcagtgtagctggatggctcctcgggaacccaatgtgtgatgaattcatcaacgtaccagaatggtcttacatagtggagaaggccaatccgaccaatgacctctgttacccagggagtttcaacgactatgaagaactgaaacacctattgagcagaataaaccattttgagaaaattcagatcatccccaaaagttcttggtccgaccatgaagcatcatcaggagtgagctcagcatgtccatatctgggaagtccctccttttttagaaatgtggtatggcttatcaaaaaggacagtacgtacccaacaataaagaaaacctacaataataccaaccaagaagatcttttgatactgtggggaattcaccatcctaataatgaggcagagcagacaatgctatatcaaaacccaaccacctatatttccattgggacatcaacactaaaccagagattggtaccaaaaatagctactagatccaaagtaaacgggcaaagtggaaggatggagtttttctggacaattttaaaaccaaatgatgcaatcaacttcgagagtaatggaaatttcattgctccagaatatgcatacaaaattgtcaagaaaggagactcagcaattatgaaaagtgaattggaatatggtaactgcaacaccaagtgtcaaactccaatgggggcgataaactctagtatgccattccacaacatacaccctctcaccatcggggaatgccccaaatatgtgaaatcaaacagattagtcctagcaacagggctcagaaatagccctcaaagagaaagcagaaggaaaaggaga

>H5N1_A/Indonesia/TLL001/2006

taccatgcaaacaattcaacagagcaggttgacacaatcatggaaaagaacgttactgttacacatgcccaagacatattggaaaagacacacaacgggaagctctgcgatctagatggagtgaagcctctaattttaagagattgtagtgtagctggatggctcctcgggaacccaatgtgtgacgaattcatcaatgtaccggaatggtcttacatagtggagaaggccaatccaaccaatgacctctgttacccagggagtttcaacgactatgaagaactgaaacacctattgagcagaataaaccattttgagaaaattcagatcatccccaaaagttcttggtccgatcatgaagcctcatcaggagtgagttcagcatgtccatacctgggtagttcctccttttttagaaatgtggtatggcttatcaaaaagaacagtacatacccaacaataaagaaaagctacaataataccaaccaagaagatcttttggtactgtgggggattcaccatcctaatgatgcggcagagcagacaaggctatatcaaaacccaaccacctatatttccattgggacatcaacactaaaccagagattggtaccaaaaatagctactagatccaaagtaaatgggcaaagtggaaggatggagttcttctggacaattttaaaacctaatgatgcaatcaacttcgagagtaatggaaatttcattgctccagaatatgcatacaaaattgtcaagaaaggggactcagcaattatgaagagtgaattggaatatggtaactgcaacaccaagtgtcaaactccaatgggggcgataaactctagtatgccattccacaacatacaccctctcaccaccggggaatgccccaaatatgtgaaatcaaacagattagtccttgcaacagggctcagaaatagccctcaaagagaaagcagaagaaaaaagaga

>H5N1_A/Indonesia/TLL002/2006

taccatgcaaacaattcaacagagcaggttgacacaatcatggaaaagaacgttactgttacacatgcccaagacatattggaaaagacacacaacgggaagctctgcgatctagatggagtgaagcctctaattttaagagattgtagtgtagctggatggctcctcgggaacccaatgtgtgacgaattcatcaatgtaccggaatggtcttacatagtggagaaggccaatccaaccaatgacctctgttacccagggagtttcaacgactatgaagaactgaaacacctattgagcagaataaaccattttgagaaaattcagatcatccccaaaagttcttggtccgatcatgaagcctcatcaggagtgagttcagcatgtccatacctgggtagttcctccttttttagaaatgtggtatggcttatcaaaaagaacagtacatacccaacaataaagaaaagctacaataataccaaccaagaagatcttttggtactgtgggggattcaccatcctaatgatgcggcagagcagacaaggctatatcaaaacccaaccacctatatttccattgggacatcaacactaaaccagagattggtaccaaaaatagctactagatccaaagtaaatgggcaaagtggaaggatggagttcttctggacaattttaaaacctaatgatgcaatcaacttcgagagtaatggaaatttcattgctccagaatatgcatacaaaattgtcaagaaaggggactcagcaattatgaagagtgaattggaatatggtaactgcaacaccaagtgtcaaactccaatgggggcgataaactctagtatgccattccacaacatacaccctctcaccatcggggaatgccccaaatatgtgaaatcaaacagattagtccttgcaacagggctcagaaatagccctcaaagagaaagcagaagaaaaaagaga

>H5N1_A/Indonesia/TLL003/2006

taccatgcaaacaattcaacagagcaggttgacacaatcatggaaaagaacgttactgttacacatgcccaagacatactggaaaagacacacaacgggaagctctgcgatctagatggagtgaagcctctaattttaaaagattgtagtgtagctggatggctcctcgggaacccaatgtgtgacgaattcatcaatgtaccggaatggtcttacatagtggagaaggccaatccaaccaatgacctctgttacccagggagtttcaacgactatgaagaactgaaacacctattgagcagaataaaccattttgagaaaattcaaatcatccccaaaagttcttggtccgatcatgaagcctcatcaggagtgagctcagcatgtccatacctgggaagtccctccttttttagaaatgtggtatggcttatcaaaaagaacagtacatacccaacaataaagaaaagctacaataataccaaccaagaagatcttttggtactgtggggaattcaccaccctaatgatgcggcagagcagacaaggctatatcaaaacccaaccacctatatttccattgggacatcaacactaaaccagagattggtaccaaaaatagctactagatccaaagtaaacgggcaaagtggaaggatggagttcttctggacaattttaaaacctaatgatgcagtcaacttcgagagtaatggaaatttcattgctccagaatatgcatacaaaattgtcaagaaaggggactcagcaattatgaaaagtgaattggaatatggtaactgtaacaccaagtgtcaaactccaatgggggcgataaactctagtatgccattccacaacatacaccctctcaccatcggggaatgccccaaatatgtgaaatcaaacagattagtccttgcaacagggctcagaaatagccctcaaagagagagcagaagaaaaaagaga

>H5N1_A/Indonesia/TLL004/2006

taccatgcaaacaattcaacagagcaggttgacacaatcatggaaaagaacgttactgttacacatgcccaagacatactggaaaagacacacaacgggaagctctgcgatctagatggagtgaagcctctaattttaagagattgtagtgtagctggatggctcctcgggaacccaatgtgtgacgaattcaccaatgtaccggaatggtcttacatagtggagaaggccaatccaaccaatgacctctgttacccagggagtttcaacgactatgaagaactgaaacatctattgagcagaataaaccattttgagaaaattcaaatcatccccaaaagttcttggtccgatcatgaagcctcatcaggagtgagctcagcatgtccatacctgggaagtccctccttttttagaaatgcggtatggcttatcaaaaagaacagtacatacccaacaataaagaaaagctacaataataccaaccaagaagatcttttggtactgtggggaattcaccaycctaatgatgcggcagagcagacaaggctatatcaaaacccaaccacctatatttccattgggacatcaacactaaaccagagattggtaccaaaaatagctactagatccaaagtaaacgggcaaagtggaaggatggagttcttctggacaattttaaaacctaatgatgcaatcaacttcgaaagtaatggaaatttcattgctccagaatatgcatacaaaattgtcaagaaaggggactcagcaattatgaaaagtgaattggaatatggtaactgcaacaccaagtgtcaaactccaatgggggcgataaactctagtatgccattccacaacatacaccctctcaccatcggggaatgccccaaatatgtgaaatcaaacagattagtccttgcaacagggctcagaaatagccctcaaagagagagcagaagaaaaaagaga

>H5N1_A/Indonesia/TLL005/2006

taccatgcaaacaattcaacagagcaggttgacacaatcatggaaaagaacgttactgttacacatgcccaagacatactggaaaagacacacaacgggaagctctgcgatctagatggagtgaagcctctaattttaagagattgtagtgtagctggatggctcctcgggaacccaatgtgtgacgaattcatcaatgtaccggaatggtcttacatagtggagaaggccaatccaaccaatgacctctgttacccagggagtttcaacgactatgaagaactgaaacatctattgagcagaataaaccattttgagaaaattcaaatcatccccaaaagttcttggtccgatcatgaagcctcatcaggagtgagctcagcatgtccatacctgggaagtccctccttttttagaaatgtggtatggcttatcaaaaagaacagtacatacccaacaataaagaaaagctacaataataccaaccaagaagatcttttggtactgtggggaattcaccatcctaatgatgcggcagagcagacaaggctatatcaaaacccaaccacctatatttccattgggacatcaacactaaaccagagattggtaccaaaaatagctactagatccaaagtaaacgggcaaagtggaaggatggagttcttctggacaattttaaaacctaatgatgcaatcaacttcgaaagtaatggaaatttcattgctccagaatatgcatacaaaattgtcaagaaaggggactcagcaattatgaaaagtgaattggaatatggtaactgcaacaccaagtgtcaaactccaatgggggcgataaactctagtatgccattccacaacatacaccctctcaccatcggggaatgccccaaatatgtgaaatcaaacagattagtccttgcaacagggctcagaaatagccctcaaagagagagcagaagaaaaaagaga

>H5N1_A/Indonesia/TLL006/2006

taccatgcaaacaattcaacagagcaggttgacacaatcatggaaaagaacgttactgttacacatgcccaagacatactggaaaagacacacaatgggaagctctgcgatctagatggagtgaagcctctaattttaagagattgtagtgtagctggatggctcctcgggaacccaatgtgtgacgaattcatcaatgtaccggaatggtcttacatagtggagaaggccaatccaaccaatgacctctgttacccagggagtttcaacgactatgaagaactgaaacacctattgagcagaataaaccattttgagaaaattcaaatcatccccaaaagttcttggtccgaccatgaagcctcatcaggagtgagctcagcatgtccatacctgggaagtccctccttttttagaaatgtggtatggcttatcaaaaagaacagtacatacccaacaataaagaaaagctacaataataccaaccaagaagatcttttggtactgtggggaattcaccatcctaatgatgcggcagagcagacaaggctatatcaaaacccaaccacctatatttccattgggacatcaacactaaaccagagattggtaccaaaaatagctactagatccaaagtaaacgggcaaagtggaaggatggagttcttctggacaattttaaaacctaatgatgcaatcaacttcgagagtaatggaaatttcattgctccagaatatgcatacaaaattgtcaagaaaggggactcagcaattatgaaaagtgaattggaatatggtaactgcaacaccaagtgtcaaactccgatgggggcgataaactctagtatgccattccacaacatacaccctctcaccatcggggaatgccccaaatatgtgaaatcaaacagattagtccttgcaacagggctcagaaatagccctcaaagagagagcagaagaaaaaagaga

>H5N1_A/Indonesia/TLL007/2006

taccatgcaaacaattcaacagagcaggttgacacaatcatggaaaagaacgttactgttacacatgcccaagacatactggaaaagacacacaacgggaagctctgcgatctagatggagtgaagcctctaattttaagagattgtagtgtagctggatggctcctcgggaacccaatgtgtgacgaattcatcaatgtaccggaatggtcttacatagtggagaaggccaatccaaccaatgacctctgttacccagggagtttcaacgactatgaagaactgaaacacctattgagcagaataaaccattttgagaaaattcaaatcatccccaaaagttcttggtccgatcatgaagcctcatcaggagtgagctcagcatgtccatacctgggaagtccctccttttttagaaatgtggtatggcttatcaaaaagaacagtacatacccaacaataaagaaaagctacaataataccaaccaagaagatcttttggtactgtggggaattcaccatcctaatgatgcggcagagcagacaaggctatatcaaaacccaaccacctatatttccattgggacatcaacactaaaccagagattggtaccaaaaatggctactagatccaaagtaaacgggcaaagtggaaggatggagttcttctggacaattttaaaacctaatgatgcaatcaacttcgagagtaatggaaatttcattgctccagaatatgcatacaaaattgtcaagaaaggggactcagcaattatgaaaagtgaattggaatatggtaactgcaacaccaagtgtcaaactccaatgggggcgataaactctagtatgccattccacaacatacaccctctcaccatcggggaatgccccaaatatgtgaaatcaaacagattagtccttgcaacagggctcagaaatagccctcaaagagagagcagaagaaaaaagaga

>H5N1_A/Indonesia/TLL008/2006

taccatgcaaacaattcaacagagcaggttgacacaatcatggaaaagaacgttactgttacacatgcccaagacatactggaaaagacacacaatgggaagctctgcgatctagatggagtgaagcctctaattttaagagattgtagtgtagctggatggctcctcgggaacccaatgtgtgacgaattcatcaatgtaccggaatggtcttacatagtggagaaggccaatccaaccaatgacctctgttacccagggagtttcaacgactatgaagaactgaaacacctattgagcagaataaaccattttgagaaaattcaaatcatccccaaaagttcttggtccgaccatgaagcctcatcaggagtgagctcagcatgtccatacctgggaagtccctccttttttagaaatgtggtatggcttatcaaaaagaacagtacatacccaacaataaagaaaagctacaataataccaaccaagaagatcttttggtactgtggggaattcaccatcctaatgatgcggcagagcagacaaggctatatcaaaacccaaccacctatatttccattgggacatcaacactaaaccagagattggtaccaaaaatagctactagatccaaagtaaacgggcaaagtggaaggatggagttcttctggacaattttaaaacctaatgatgcaatcaacttcgagagtaatggaaatttcattgctccagaatatgcatacaaaattgtcaagaaaggggactcagcaattatgaaaagtgaattggaatatggtaactgcaacaccaagtgtcaaactccgatgggggcgataaactctagtatgccattccacaacatacaccctctcaccatcggggaatgccccaaatatgtgaaatcaaacagatcagtccttgcaacagggctcagaaatagccctcaaagagagagcagaagaaaaaagaga

>H5N1_A/Indonesia/TLL009/2006

taccatgcaaacaattcaacagagcaggttgacacaatcatggaaaagaacgttactgttacacatgcccaagacatactggaaaagacacacaacgggaagctctgcgatctagatggagtgaagcctctaattttaaaagattgtagtgtagctggatggctcctcgggaacccaatgtgtgacgaattcatcaatgtaccggaatggtcttacatagtggagaaggccaatccaaccaatgacctctgttacccagggagtttcaacgactatgaagaactgaaacacctattgagcagaataaaccattttgagaaaattcaaatcatccccaaaagttcttggtccgatcatgaagcctcatcaggagtgagctcagcatgtccatacctgggaagtccctccttttttagaaatgtggtatggcttatcaaaaagaacagtacatacccaacaataaagaaaagctacaataataccaaccaagaagatcttttggtactgtggggaattcaccaccctaatgatgcggcagagcagacaaggctatatcaaaacccaaccacctatatttccattgggacatcaacactaaaccagagattgktaccaaaaatagctactagatccaaagtaaacgggcaaagtggaaggatggagttcttctggacaattttaaaacctaatgatgcaatcaacttcgagagtaatggaaatttcattgctccagaatatgcatacaaaattgtcaagaaaggggactcagcaattatgaaaagtgaattggaatatggtaactgtaacaccaagtgtcaaactccaatgggggcgataaactctagtatgccattccacaacatacaccctctcaccatcggggaatgccccaaatatgtgaaatcaaacagattagtccttgcaacagggctcagaaatagccctcaaagagagagcagaagaaaaaagaga

>H5N1_A/Indonesia/TLL010/2006

taccatgcaaacaattcaacagagcaggttgacacaatcatggaaaagaacgttactgttacacatgcccaagacatactggaaaagacacacaacgggaagctctgcgatctagatggagtgaagcctctaattttaagagattgtagtgtagctggatggctcctcgggaacccaatgtgtgacgaattcatcaatgtaccggaatggtcttacatagtggagaaggccaatccaaccaatgacctctgttacccagggagtttcaacgactatgaagaactgaaacatctattgagcagaataaaccattttgagaaaattcaaatcatccccaaaagttcttggtccgatcatgaagcctcatcaggagtgagctcagcatgtccatacctgggaagtccctccttttttagaaatgtggtatggcttatcaaaaagaacagtacatacccaacaataaagaaaagctacaataataccaaccaagaagatcttttggtactgtggggaattcaccatcctaatgatgcggcagagcagacaaggctatatcaaaacccaaccacctatatttccattgggacatcaacactaaaccagagattggtaccaaaaatagctactagatccaaagtaaacgggcaaagtggaaggatggagttcttctggacaattttaaaacctaatgatgcaatcaacttcgaaagtaatggaaatttcattgctccagaatatgcatacaaaattgtcaagaaaggggactcagcaattatgaaaagtgaattggaatatggtaactgcaacaccaagtgtcaaactccaatgggggcgataaactctagtatgccattccacaacatacaccctctcaccatcggggaatgccccaaatatgtgaaatcaaacagattagtccttgcaacagggctcagaaatagccctcaaagagagagcagaagaaaaaagaga

>H5N1_A/Indonesia/TLL011/2006

taccatgcaaacaattcaacagagcaggttgacacaatcatggaaaagaacgttactgttacacatgcccaagacatactggaaaagacacacaacgggaagctctgcgatctagatggagtgaagcctctaattttaagagattgtagtgtagctggatggctcctcgggaacccaatgtgtgacgaattcatcaatgtaccggaatggtcttacatagtggagaaggccaatccaaccaatgacctctgttacccagggagtttcaacgactatgaagaactgaaacatctattgagcagaataaaccattttgagaaaattcaaatcatccccaaaagttcttggtccgatcatgaagcctcatcaggagtgagctcagcatgtccatacctgggaagtccctccttttttagaaatgtggtatggcttatcaaaaagaacagtacatacccaacaataaagaaaagctacaataataccaaccaagaagatcttttggtactgtggggaattcaccatcctaatgatgcggcagagcagacaaggctatatcaaaacccaaccacctatatttccattgggacatcaacactaaaccagagattggtaccaaaaatagctactagatccaaagtaaacgggcaaagtggaaggatggagttcttctggacaattttaaaacctaatgatgcaatcaacttcgaaagtaatggaaatttcattgctccagaatatgcatacaaaattgtcaagaaaggggactcagcaattatgaaaagtgaattggaatatggtaactgcaacaccaagtgtcaaactccaatgggggcgataaactctagtatgccattccacaacatacaccctctcaccatcggggaatgccccaaatatgtgaaatcaaacagattagtccttgcaacagggctcagaaatagccctcaaagagagagcagaagaaaaaagaga

>H5N1_A/Indonesia/TLL012/2006

taccatgcaaacaattcaacagagcaggttgacacaatcatggaaaagaacgttactgttacacatgcccaagacatactggaaaagacacacaacgggaagctctgcgatctagatggagtgaagcctctaattttaagagattgtagtgtagctggatggctcctcgggaacccaatgtgtgacgaattcatcaacgtaccggaatggtcttacatagtggaaaaggccaatccaaccaatgacctctgttacccagggagtttcaacgactatgaagaactgaaacacctattgagcagaataaaccattttgagaaaattcagatcatccccaaaagttcttggtccgatcatgaagcctcatcaggagtgagctcagcatgtccatacctgggaagtccctccttttttagaaatgtggtatggcttatcaaaaagaacagtacatacccaacaataaagaaaagctacaataataccaaccaagaagatcttttggtactgtggggaattcaccatcctaataatgaggaagagcagacaaggctatatcaaaacccaaccacctatatttccattgggacatcaacactaaaccagagattggtaccaaaaatagctactagatccaaagtaaacgggcaaagtgggaggatggagttcttctggacaattttaaaaccaaatgatgcaatcaacttcgagagcaatggaaatttcattgctccagaatatgcatacaaaattgtcaagaaaggggactcagcaattatgaaaagtgaattggaatatagtaactgcaacaccaagtgtcaaactccaatgggggcgataaactctagtatgccattccacaacatacaccctctcaccatcggggaatgccccaaatatgtgaaatcaagcagattagtccttgcaacagggctcagaaatagccctcaaagagagagcagaagaaaaaagaga

>H5N1_A/Indonesia/TLL014/2006

taccatgcaaacaattcaacagagcaggttgacacaatcatggaaaagaacgttactgttacacatgcccaagacatactggaaaagacacacaacgggaagctctgcgatctagatggagtgaagcctctaattttaagagattgtagtgtagctggatggctcctcgggaacccaatgtgtgacgaattcatcaacgtaccggaatggtcttacatagtggagaaggccaatccaaccaatgacctctgttacccagggagtttcaacgactatgaagaactgaaacacctattgagcagaataaaccattttgagaaaattcagatcatccccaaaagttcttggtccgatcatgaagcctcatcaggagtgagctcagcatgtccatacctgggaagtccctccttttttagaaatgtggtatggcttatcaaaaagaacagtacatacccaacaataaagaaaagctacaataataccaaccaagaagatcttttggtactgtggggaattcaccatcctaataatgaggcagagcagacaaggctatatcaaaacccaaccacctatatttccattgggacatcaacactaaaccagagattggtaccaaaaatagctactagatccaaagtaaacgggcaaagtggaaggatggagtttttctggacaattttaaaaccaaatgatgcaatcaacttcgagagtaatggaaatttcattgctccagaatatgcatacaaaattgtcaagaaaggggactcagcaattatgaaaagtgaattggaatatggtaactgcaacaccaagtgtcaaactccaatgggggcgataaactctagtatgccattccacaacatacaccctctcaccatcggggaatgccccaaatatgtgaaatcaaacagattagtccttgcaacagggctcagaaatagccctcaaagagagagcagaagaaaaaagaga

>H5N1_A/Indonesia/UT3006/2005

taccatgcaaacaattcaacagagcaggttgacacaatcatggaaaagaacgttactgttacacatgcccaagacatattggaaaagacacacaacgggaagctctgcgatctagatggagtgaagcctctaattttaagagattgtagtgtagctggatggctcctcgggaacccaatgtgtgacgaattcatcaatgtaccggaatggtcttacatagtggagaaggccaatccaaccaatgacctctgttacccagggagtttcaacgactatgaagaactgaaacacctattgagcagaataaaccattttgagaaaattcagatcatccccaaaagttcttggtccgatcatgaagcctcatcaggagtgagttcagcatgtccatacctgggtagttcctccttttttagaaatgtggtatggcttatcaaaaagaacagtacatacccaacaataaagaaaagctacaataataccaaccaagaagatcttttggtactgtgggggattcaccatcctaatgatgcggcagagcagacaaggctatatcaaaacccaaccacctatatttccattgggacatcaacactaaaccagagattggtaccaaaaatagctactagatccaaagtaaatgggcaaagtggaaggatggagttcttctggacaattttaaaacctaatgatgcaatcaacttcgagagtaatggaaatttcattgctccagaatatgcatacaaaattgtcaagaaaggggactcagcaattatgaagagtgaattggaatatggtaactgcaacaccaagtgtcaaactccaatgggggcgataaactctagtatgccattccacaacatacaccctctcaccatcggggaatgccccaaatatgtgaaatcaaacagattagtccttgcaacagggctcagaaatagccctcaaagagaaagcagaagaaaaaagaga

>H5N1_A/Iraq/1/2006

taccatgcaaacaactcgacagagcaggttgacacaataatggaaaagaacgtaactgttacacacgcccaagacatactggaaaagacacacaacgggaaactctgcgatctagatggagtgaagcctctaattttaagagattgtagtgtagctggatggctcctcgggaacccaatgtgtgacgaattcctcaatgtgccggaatggtcttacatagtggagaagatcaatccagccaatgacctctgttacccagggaatttcaacgactatgaagaactgaaacacctattgagcagaataaaccattttgagaaaattcagatcatacccaaaagttcttggtcagatcatgaagcctcatcaggggtgagctcagcatgtccataccagggaaggtcctccttttttagaaatgtggtatggcttatcaaaaaggacaatgcatacccaacaataaagagaagttacaataataccaatcaagaagatcttttggtactgtgggggattcaccatccaagtgatgcggcagagcagacaaggctctatcaaaacccaaccacctatatttccgttgggacatcaacactaaaccagagattggtaccaaaaatagctactagatccaaggtaaacgggcaaagtggaaggatggagttcttttggacaattttaaaaccgaatgatgcaataaactttgagagtaatggaaatttcattgctccagaaaatgcatacaaaattgtcaagaaaggggactcaacaattatgaaaagtgaattggaatatggtaactgcaacaccaagtgtcaaactccaataggggcgataaactctagtatgccattccacaacatccaccctctcaccataggggaatgccccaaatatgtgaaatcaaacagattagtccttgcgactgggctcagaaatagccctcaaggagagagaagaagaaaaaagaga

>H5N1_A/Iraq/659/2006

taccatgcaaacaactcgacagagcaggttgacacaataatggaaaagaacgtaactgttacacacgcccaagacatactggaaaagacacacaacgggaaactctgcgatctagatggagtgaagcctctaattttaagagattgtagtgtagctggatggctcctcgggaacccaatgtgtgacgaattcctcaatgtgccggaatggtcttacatagtggagaagatcaatccagccaatgacctctgttacccagggaatttcaacgactatgaagaactgaaacacctattgagcagaataaaccattttgagaaaattcagatcatacccaaaagttcttggtcagatcatgaagcctcatcaggggtgagctcagcatgtccataccagggaaggtcctccttttttagaaatgtggtatggcttatcaaaaaggacaatgcatacccaacaataaagagaagttacaataataccaatcaagaagatcttttggtactgtgggggattcaccatccaagtgatgcggcagagcagacaaggctctatcgaaacccaaccacctatatttccgttgggacatcaacactaaaccagagattggtgccaaaaatagctactagatccaaggtaaacgggcaaagtggaaggatggagttcttttggacaattttaaaaccgaatgatgcaataaactttgagagtaatggaaatttcattgctccagaaaatgcatacaaaattgtcaagaaaggggactcaacaattatgaaaagtgaattggaatatggtaactgcaacaccaagtgtcaaactccaataggggcgataaactctagtatgccattccacaacatccaccctctcaccataggggaatgccccaaatatgtgaaatcaaacagattagtccttgcgactgggctcagaaatagccctcaaggagagagaagaagaaaaaagaga

>H5N1_A/Iraq/754/2006

taccatgcaaacaactcgacagagcaggttgacacaataatggaaaagaacgtaactgttacacacgcccaagacatactggaaaagacacacaacgggaaactctgcgatctagatggagtgaagcctctaattttaagagattgtagtgtagctggatggctcctcgggaacccaatgtgtgacgaattcctcaatgtgccggaatggtcttacatagtggagaagatcaatccagccaatgacctctgttacccagggaatttcaacgactatgaagaactgaaacacctattgagcagaataaaccattttgagaaaattcagatcatacccaaaagttcttggtcagatcatgaagcctcatcaggggtgagctcagcatgtccataccagggaaggtcctccttttttagaaatgtggtatggcttatcaaaaaggacaatgcatacccaacaataaagagaagttacaataataccaatcaagaagatcttttggtactgtgggggattcaccatccaagtgatgcggcagagcagacaaggctctatcaaaacccaaccacctatatttccgttgggacatcaacactaaaccagagattggtaccaaaaatagctactagatccaaggtaaacgggcaaagtggaaggatggagttcttttggacaattttaaaaccgaatgatgcaataaactttgagagtaatggaaatttcattgctccagaaaatgcatacaaaattgtcaagaaaggggactcaacaattatgaaaagtgaattggaatatggtaactgcaacaccaagtgtcaaactccaataggggcgataaactctagtatgccattccacaacatccaccctctcaccataggggaatgccccaaatatgtgaaatcaaacagattagtccttgcgactgggctcagaaatagccctcaaggagagagaagaagaaaaaagaga

>H5N1_A/Iraq/755/2006

taccatgcaaacaactcgacagagcaggttgacacaataatggaaaagaacgtaactgttacacacgcccaagacatactggaaaagacacacaacgggaaactctgcgatctagatggagtgaagcctctaattttaagagattgtagtgtagctggatggctcctcgggaacccaatgtgtgacgaattcctcaatgtgccggaatggtcttacatagtggagaagatcaatccagccaatgacctctgttacccagggaatttcaacgactatgaagaactgaaacacctattgagcagaataaaccattttgagaaaattcagatcatacccaaaagttcttggtcagatcatgaagcctcatcaggggtgagctcagcatgtccataccagggaaggtcctccttttttagaaatgtggtatggcttatcaaaaaggacaatgcatacccaacaataaagagaagttacaataataccaatcaagaagatcttttggtactgtgggggattcaccatccaagtgatgcggcagagcagacaaggctctatcaaaacccaaccacctatatttccgttgggacatcaacactaaaccagagattggtaccaaaaatagctactagatccaaggtaaacgggcaaagtggaaggatggagttcttttggacaattttaaaaccgaatgatgcaataaactttgagagtaatggaaatttcattgctccagaaaatgcatacaaaattgtcaagaaaggggactcaacaattatgaaaagtgaattggaatatggtaactgcaacaccaagtgtcaaactccaataggggcgataaactctagtatgccattccacaacatccaccctctcaccataggggaatgccccaaatatgtgaaatcaaacagattagtccttgcgactgggctcagaaatagccctcaaggagagagaagaagaaaaaagaga

>H5N1_A/Iraq/756/2006

taccatgcaaacaactcgacagagcaggttgacacaataatggaaaagaacgtaactgttacacacgcccaagacatactggaaaagacacacaacgggaaactctgcgatctagatggagtgaagcctctaattttaagagattgtagtgtagctggatggctcctcgggaacccaatgtgtgacgaattcctcaatgtgccggaatggtcttacatagtggagaagatcaatccagccaatgacctctgttacccagggaatttcaacgactatgaagaactgaaacacctattgagcagaataaaccattttgagaaaattcagatcatacccaaaagttcttggtcagatcatgaagcctcatcaggggtgagctcagcatgtccataccagggaaggtcctccttttttagaaatgtggtatggcttatcaaaaaggacaatgcatacccaacaataaagagaagttacaataataccaatcaagaagatcttttggtactgtgggggattcaccatccaagtgatgcggcagagcagacaaggctctatcgaaacccaaccacctatatttccgttgggacatcaacactaaaccagagattggtaccaaaaatagctactagatccaaggtaaacgggcaaagtggaaggatggagttcttttggacaattttaaaaccgaatgatgcaataaactttgagagtaatggaaatttcattgctccagaaaatgcatacaaaattgtcaagaaaggggactcaacaattatgaaaagtgaattggaatatggtaactgcaacaccaagtgtcaaactccaataggggcgataaactctagtatgccattccacaacatccaccctctcaccataggggaatgccccaaatatgtgaaatcaaacagattagtccttgcgactgggctcagaaatagccctcaaggagagagaagaagaaaaaagaga

>H5N1_A/Jiangsu/1/2007

taccatgcaaacaactcgacagagcaggttgacacaataatggaaaagaacgttactgttacacatgcccaagacatactggagaagacacataacgggaaactctgcgatctagatggagtgaagcctctgattctacgagattgtagtgtagctggatggctcctcggaaacccaatgtgtgacgaattcatcaatgtgccggaatggtcttacatagtggagaaggccaacccagccaatgacctctgttacccagggaatttcaacgactatgaagaactgaaacacctattgagcagaataaaccattttgagaaaattcagatcatccccaaaagttcttggtccgatcatgaagcctcatcaggggtgagctcagcatgtccataccagggaacgccctcctttttcagaaatgtggtatggcttatcaaaaagaacaatacatacccaacaataaagagaagctacaataataccaaccaggaaaatcttttgatactgtgggggattcatcattctaatgatgcagcagagcagataaagctctatcaaaacccaaccacctatatttccgttgggacatcaacactaaaccagagattggtaccaaaaatagccactagatccaaagtaaacgggcaaagtggaaggatggatttcttctggacaattttaaaaccgaatgatgcaatcaacttcgagagtaatggaaatttcattgctccagaatatgcatacaaaattgtcaaggaaggagactcagcaattatgaaaagtgaagtggaatatggtaactgcaacaccaagtgtcaaactccaataggggcgataaactctagtatgccattccacaacatacaccctctcaccatcggggaatgccccaaatatgtgaaatcaaacaaattagtccttgctactgggctcagaaatagtcctctaagagaaagGGGaagaagaaaaaga

>H5N1_A/Jiangsu/2/2007
[truncated: 151,419 more chars]
