# Supplementary material for: Phylodynamics and Molecular Mutations of the Hemagglutinin Affecting Global Transmission and Host Adaptation of H5Nx Viruses
Source: Transbound Emerg Dis. 2023 Apr 14;2023:8855164. doi: 10.1155/2023/8855164 (PMC12017097; doi:10.1155/2023/8855164)
Supplement: Supplementary Materials — Supplemental Table 1: the number of the H5Nx HA sequences by subtype collected. Supplemental Table 2: the number of the H5Nx HA sequences by subtype and isolation year used for the phylogenetic analysis. Supplemental Table 3: amino acid mutations in the HA globular head region of reference human-isolated H5Nx viruses by clade. Supplemental Table 4: summary of natural selection pressure profiles of the H5Nx HAs by clade. Supplemental Data 1: initial sequence set of H5Nx HAs. Supplemental Data 2: selected sequence set of H5Nx HAs. Supplemental Data 3: sequence set of human-isolated H5Nx HAs. Supplemental Figure 1: the proportion of amino acid mutations around the HA globular head region of clade 2.3.4.4 H5Nx viruses by subtype and collection year. (a) The proportion of amino acid mutations around the HA globular head region of clade 2.3.4.4 H5Nx viruses is presented by each subtype (Supplemental Table 1). (b) The H5Nx HA sequences are divided into six periods; (a) 1997–2004 (n = 147), (b) 2005–2008 (n = 533), (c) 2009–2012 (n = 425), (d) 2013–2016 (n = 1082), (e) 2017–2020 (n = 842) and 2021-2022 (n = 860). Supplemental Figure 2: the proportion of the I155T and T160A mutations and molecular interactions of the HA globular head region of H5Nx viruses. (a) The proportion of the I155T (blue) and T160A (magenta) mutations in avian (dashed lines) and human-isolated H5Nx viruses (solid lines) is presented by each period (years). (b) Using the HA structure of VN1194, the receptor-binding pocket in H5 HA contains a conserved floor of residues Y95, W153, H183, Y195, and E190 (pale yellow), and Q226 and G228 (orange) that interact with α2,3 SA receptors. Residues 155 and 160 are colored blue and magenta. Supplemental Figure 3: structural analysis of the HA globular head region of human-isolated H5Nx viruses. Using the HA structure of VN1194, molecular interactions of the HA globular head region residues are estimated; (a) A/Nepal/19FL1997/2019 (H5N1) (NP19FL1197) in subclade 2. [file 8855164.f1.zip › SI_TBED_revision.pdf]

Supplemental Table 1. The number of the H5Nx HA sequences by subtype collected.

	Avian pecies										
Subtype	Chicken	Domestic duck	Turkey	Wild duck	Wild goose	Wild swan	Other wild birds	# of LPAI (%)	Avian sub-total	Human	Total
**H5N1**	675	386	91	155	119	61	245	68 (3.9%)	1732	516	2248
**H5N2**	112	80	89	277	33	14	53	416 (63.2%)	658		658
**H5N3**		27		42	5	1	15	77 (85.6%)	90		90
**H5N4**				1			4	4 (80.0%)	5		5
**H5N5**	2	7		25	3	7	9	25 (47.2%)	53		53
**H5N6**	62	107		62	37	48	49	5 (1.4%)	365	32	397
**H5N8**	202	135	95	116	160	124	114	10 (1.1%)	946	1	947
**H5N9**	1	3		13			7	19 (79.2%)	24		24
Total	1054	745	275	691	357	255	496	624 (16.2)	3873	549	4422

Supplemental Table 2. The number of the H5Nx HA sequences by subtype and isolation year used for the phylogenetic analysis.

Subtype	1996-2002	2003	2004	2005	2006	2007	2008	2009	2010	2011	2012	2013	2014	2015	2016	2017	2018	2019	2020	2021	2022	Total	HPAI	LPAI (%)
**H5N1**	12	12	37	44	56	40	27	18	23	17	24	23	21	22	9	5	0	2	4	27	11	434	428	6 (1.4)
**H5N2**		1	1		3	2	1	3	4	2	2	7	11	18	4	1			1	1	2	64	38	26 (68.4)
**H5N3**				1	2				1					1					2	5	1	13	9	4 (44.4)
**H5N4**																				2		2		2 (100)
**H5N5**							2	1	2	1									4	6		16	16	
**H5N6**												6	25	18	22	14	4	5	8	6		108	107	1 (0.9)
**H5N8**					1				1	1	2	4	17	5	4	12		2	24	25		98	96	2 (2.0)
**H5N9**							1					1		1					1			4	1	3 (75.0)
**Total**	12	13	38	45	62	42	31	22	31	21	28	41	74	65	39	32	4	9	44	72	14	739	695	44 (6.3)

Supplemental Table 3. Amino acid mutations in the HA globular head region of reference human-isolated H5Nx viruses by clade.

Virus name	Subtype	(Sub) clade	Amino acid position in the HAs of human-isolated H5Nx viruses (H3 numbering)																									
								130-loop								150-loop				190-helix								
			98	118	119	124	127	128	130	131	133	137	144	145	155	158	159	160	173	187	188	189	193	238	240	261	263	272
A/Hong Kong/483/1997	H5N1	0		N				N	D				K	S		N‡	S											
A/Hong Kong/213/2003	H5N1	1	D	S				S			L		K	S				A					R					
A/Cambodia/X0828324/2013	H5N1	1	V	S				S			L		Q	S		N‡												
A/Egypt/MOH-NRC-8434/2014	H5N1	2.1		D						S			R	S	T		D	A					R					
A/Indonesia/5/2005	H5N1	2.2	S	D									S			N‡	S						R					
A/Egypt/2546-NAMRU3/2008	H5N1	2.2		D									R	S	T	D		A					R					
A/Beijing/01/2003	H5N1	7	D	N				D					R			N‡					E							
A/Hubei/1/2010	H5N1	2.3.2.1a				N					L	A	K	S		D		A			E		R					
A/Nepal/19FL1997/2019	H5N1	2.3.2.1a			R	D					L	A	N‡	S		N		A			E		R					
A/Anhui/2/2005	H5N1	2.3.4.1	N	I	Q	S	S	D	E	A	S	S	T	P	I	N‡	N	T	Q	D	A	A	K	K	N	V	K	E
A/Sichuan/26221/2014	H5N6	2.3.4.4a			L		T	N		T	L	A	T				D	A		N			N					
A/Fujian-Sanyuan/21099/2017	H5N6	2.3.4.4b	S		L		P	N		T	L	A	T				D	A	R	N		E	N		D			G
A/Astrakhan/3212/ 2020	H5N8	2.3.4.4b	S		L		P	N		T	L	A	A				D	A	R	N		E	N		D			G
A/Chongqing/02/2021	H5N6	2.3.4.4b	S		L		P	N		T	L	A	A				D	A	R	N		E	N		D			G
A/Hangzhou/01/2021	H5N1	2.3.4.4b	S		L		P	N		T	L	A	A				D	A	R	N		E	N		D			G
A/England/215201407/2021	H5N6	2.3.4.4b	S		L		P	N		T	L	A	T				D	A	R	N		E	N		D			G
A/Hubei/29578/2016	H5N6	2.3.4.4d		T	L		P	N‡	Δ	T		A	K		T		D	A	R	N		E	N					
A/Guangzhou/39715/2014	H5N6	2.3.4.4e		T	L		P	N		T	L	A	M		T	N‡	D	T	R	N			N					
A/Hunan/55555/2016	H5N6	2.3.4.4g		T	L		P	N		T	L	A	V				D	A	S	N			D					
A/Changsha/1/2014	H5N6	2.3.4.4h		T	L		P	N‡	Δ	T		A	T		T		D	A	R	N			N					
A/Guangxi/13486/2017	H5N6	2.3.4.4h		T		R		N‡	Δ	T		A	M	A	T		D	A	K	S		E		R		I	T	
A/Guangdong/18SF020/2018	H5N6	2.3.4.4h		T		R		N‡	Δ	T		A	V	A	T		D	A	K	S		E	D	R		I	T	
A/Anhui/2021-00011/2020	H5N6	2.3.4.4h		T		E		N‡	Δ	T		A	V	A	T		D	A	K	S	V	E	D	R		I	T	
A/Chongging/00013/2021	H5N6	2.3.4.4h		T		E		N‡	Δ	T		A	V	A	T		D	A	K	S	V	E	V	R		I	T	

[a] Potential N-linked glycosylation site.

[b] Δ, amino acid deletion (ΔE130).

Supplemental Table 4. Summary of natural selection pressure profiles of the H5Nx HAs by clade.

			Positive selection			Negative selection		
**(Sub)clade**	**The number of HA sequences[a]**	**dN/dS**	**SLAC (%)**	**FUBAR (%)**	**MEME (%)**	**SLAC (%)**	**FUBAR (%)**	**MEME (%)**
the Gs/Gd lineage	697	0.21	9 (1.62)	2 (0.36)	13 (2.34)	393 (70.68)	430 (77.34)	n.d.[c]
2.3.2.1	129	0.18	2 (0.36)	3 (0.54)	8 (1.44)	174 (31.29)	329 (59.17)	n.d.
2.3.4.1-4	358	0.19	6 (1.08)	6 (1.08)	11 (1.98)	286 (51.44)	386 (69.42)	n.d.
2.3.4.4	306	0.17	2 (0.36)	3 (0.54)	9 (1.62)	258 (46.40)	385 (69.24)	n.d.

[a] The number of HA genes in the representative group for analysis within the Gs/Gd lineage.

[b] The proportion of amino acid sites = the number of amino acid sites under either positive or negative selection among total of 556 sites in the HA gene.

[c] n.d., not detected.

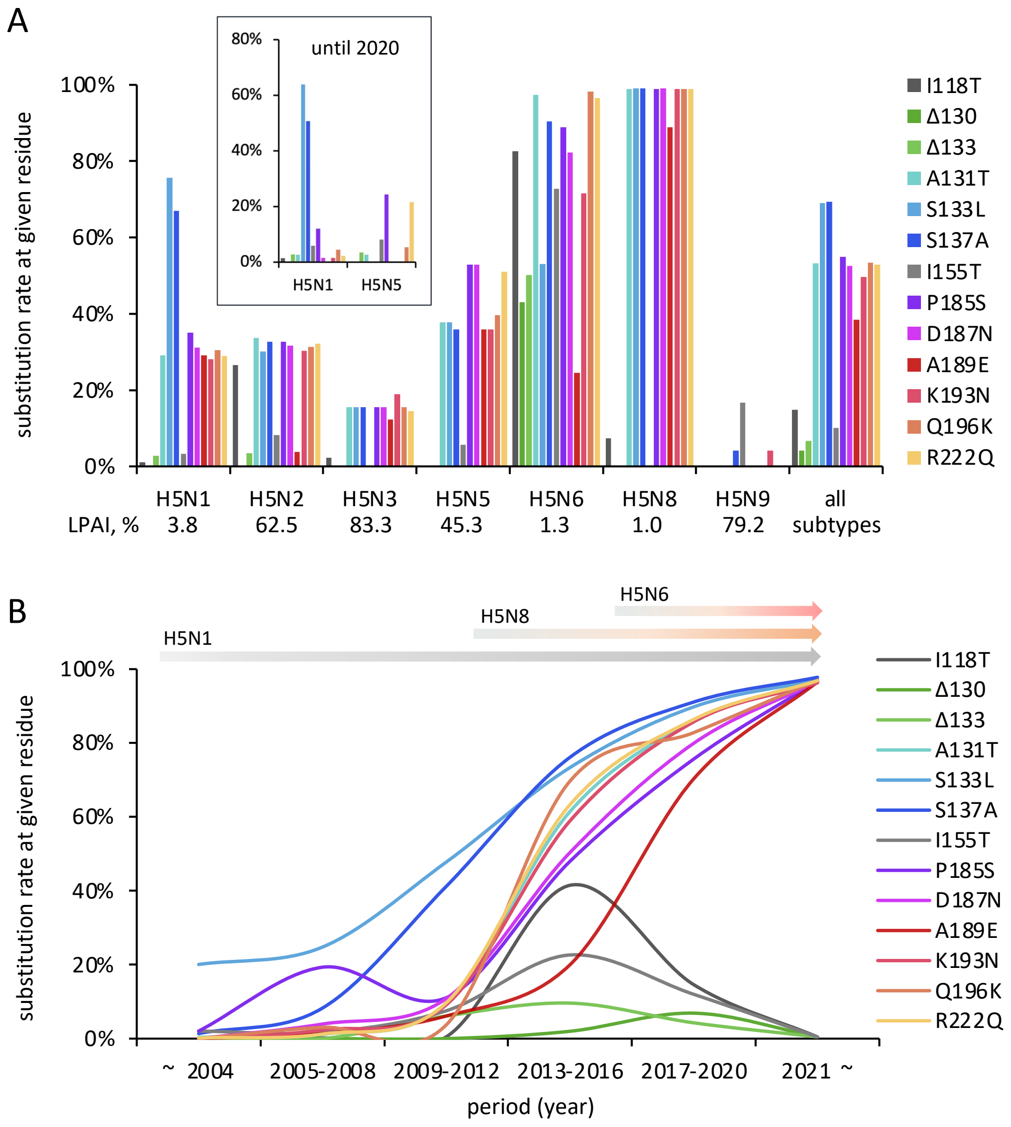

Supplemental Figure 1. The proportion of amino acid mutations around the HA globular head region of clade 2.3.4.4 H5Nx viruses by subtype and collection year. (A) The proportion of amino acid mutations around the HA globular head region of clade 2.3.4.4 H5Nx viruses is presented by each subtype. (B) The H5Nx HA sequences are divided into six periods; a. ~2004 (n = 147), b. 2005-2008 (n = 533), c. 2009-2012 (n = 425), d. 2013- 2016 (n = 1,083), e. 2017-2020 (n = 842) and 2021~ (n = 860).

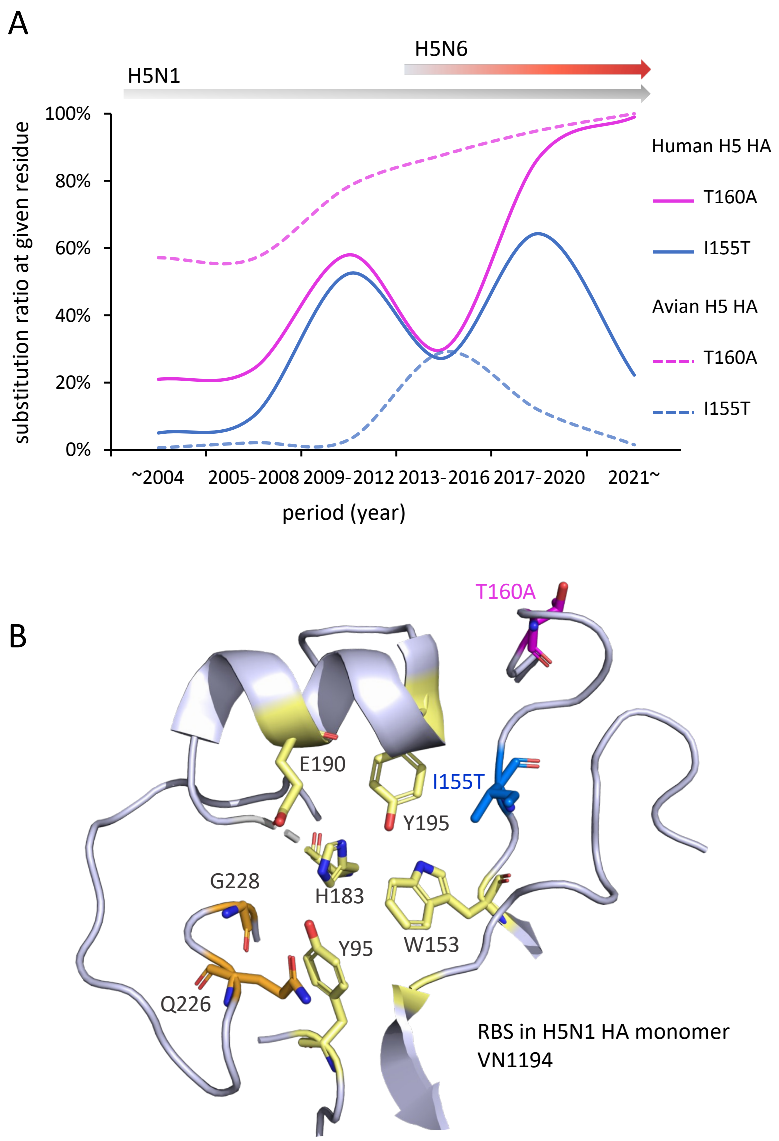

Supplemental Figure 2. The proportion of the I155T and T160A mutations and molecular interactions of the HA globular head region of H5Nx viruses. (A) The proportion of the I155T (blue) and T160A (magenta) mutations in avian (dashed lines) and human-isolated H5Nx viruses (solid lines) is presented into six periods. (B) Using the HA structure of VN1194, the receptor-binding pocket in H5 HA contains a conserved floor of residues Y95, W153, H183, Y195, and E190 (pale yellow), and Q226 and G228 (orange) that interact with α2,3 SA receptors. Residues 155 and 160 are colored blue and magenta.

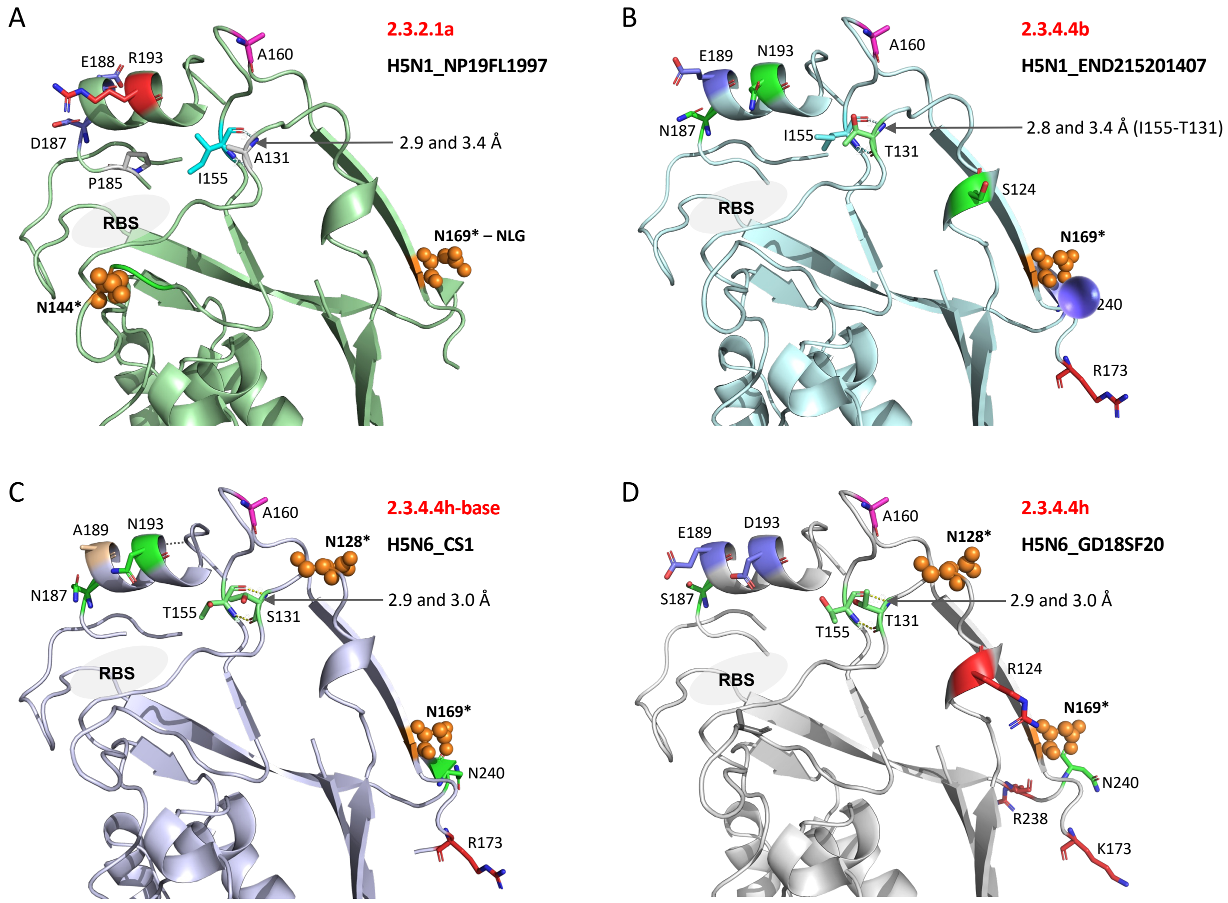

Supplemental Figure 3. Structural analysis of the HA globular head region of human-isolated H5Nx viruses. Using the HA structure of VN1194, the molecular changes and interactions region residues of the globular head in human-isolated H5Nx HAs were estimated in (A) A/Nepal/19FL1997/2019 (H5N1) (NP19FL1197) in subclade 2.3.2.1a; (B) A/England/215201407/2021 (H5N1) (END215201407) in subclade 2.3.4.4b; (C) A/Changsha/1/2014 (H5N6) (CS1) at the base of subclade 2.3.4.4h and (D) A/Guangdong/18SF020/2018 (H5N6) (GD18SF20) in subclade 2.3.4.4h.
